# Guide to the Vascular Flora of the Savannas and Flatwoods of Shaken Creek Preserve and Vicinity (Pender & Onslow Counties, North Carolina, U.S.A.)

**DOI:** 10.3897/BDJ.2.e1099

**Published:** 2014-05-16

**Authors:** Robert Thornhill, Alexander Krings, David Lindbo, Jon Stucky

**Affiliations:** † North Carolina State University, Raleigh, United States of America

**Keywords:** Floristic inventory, longleaf pine savannas, Atlantic Coastal Plain, Shaken Creek Preserve.

## Abstract

Shaken Creek Preserve (“SCP”) is a 2,448 ha (6,050 ac) natural area in Pender and Onslow Counties, North Carolina (U.S.A). Best known for its high-quality longleaf pine savanna habitat, the site contains seven savanna or savanna-like plant community types (i.e., flatwoods or sandhills), three of which are globally critically imperiled (G1): Sandy Pine Savanna (Rush Featherling subtype), Wet Loamy Pine Savanna, and Very Wet Loamy Pine Savanna. SCP hosts three Federally Endangered plant species and six Federal Species of Concern. Formerly a private hunting club, the site was virtually unknown to scientists until the 1990s; consequently, few biological inventories of SCP have been conducted. In particular, no systematic floristic inventories of the species-rich savannas have been undertaken, despite the fact that floristic data is critical to the effective management of any natural area. The goals of this study were to (1) inventory the vascular flora of the savannas, flatwoods, and sandhill community types on site through the collection of voucher specimens; (2) provide a comprehensive checklist of the flora based on collections and reports made from the site and from the same or similar habitats in the vicinity (i.e., within 2 miles of SCP); and (3) create an illustrated guide based on the checklist. In order to increase the usefulness of the guide, taxa not currently known from SCP but collected or reported from the same or similar habitats within two miles of SCP, are included in the guide. Eighty-three families containing 450 taxa, including thirty-two Significantly Rare and thirty-eight Watch List taxa, were collected or reported from SCP; an additional seven families containing a total of 102 taxa, including eighteen Significantly Rare and seven Watch List taxa, were collected or reported from the vicinity. In total, ninety families containing 552 taxa, including fifty Significantly Rare and forty-five Watch List taxa, are treated in the guide. Dichotomous keys are provided to all vouchered or reported families, genera, and species. The following features are provided for all species and infraspecific taxa: flowering and fruiting phenology; synonymy with *Manual of the Vascular Flora of the Carolinas*, the *Flora of North America*, and *Flora of the Southern and Mid-Atlantic States*; relevant voucher information; and, for most taxa, line drawings and/or photographs. For taxa collected from SCP, community types in which the taxa occur and estimates of abundance on site are also provided.

## Introduction

Shaken Creek Preserve (“SCP”) contains among the highest-quality savanna and flatwoods habitats known throughout the range of longleaf pine (*Pinus palustris* Mill.) ecosystems (LeBlond 2000). The 2,448 ha (6,050 ac) site is located in northeastern Pender County, North Carolina, with a small portion extending into adjacent Onslow County. Formerly a private hunting club, SCP was purchased by The Nature Conservancy in 2007. Previous botanical studies on site consisted of inventory work by LeBlond (2000) and plot studies by the Carolina Vegetation Survey (Peet et al. 2004). However, neither of these studies involved systematic surveying over multiple growing seasons, and neither included significant collecting efforts. Moreover, some savannas and flatwoods on site were not inventoried by either study. The goals of this study were to (1) inventory the vascular flora of the savanna, flatwoods, and sandhill community types on site through the collection of voucher specimens; (2) provide a comprehensive checklist of the flora based on collections and reports made from the site and from the same or similar habitats in the vicinity (i.e., within 2 miles of SCP, including all tracts comprising Sandy Run Savannas State Natural Area); and (3) create an illustrated guide based on the checklist.

### Setting

**Figure 1.**
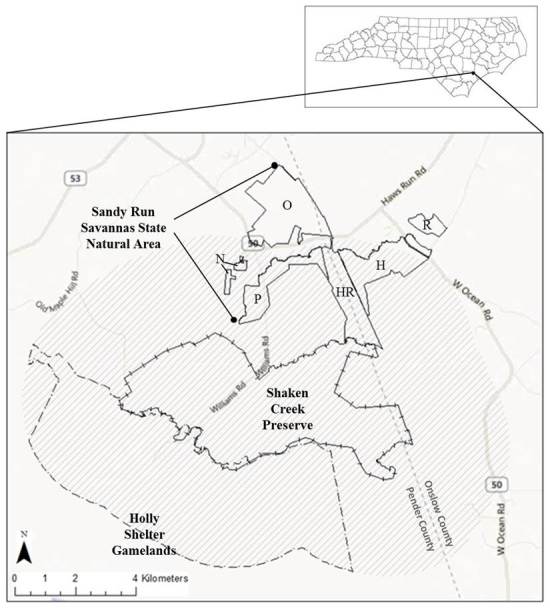
Location of areas included in this study. The striped portion designates areas within two miles of Shaken Creek Preserve. Three primary sites—Sandy Run Savannas State Natural Area, Shaken Creek Preserve, and the northern portion of Holly Shelter Gamelands—are labeled in bold. The individual tracts that comprise Sandy Run are labeled with the following abbreviations: **H** = Hancock, **HR** = Haw’s Run, **N** = The Neck Savanna, **O** = O’Berry, **P** = Patterson, **R** = RMK. Baseline imagery from Bing Maps Road, courtesy of Environmental Systems Research Institute (ESRI) (2011).

SCP is a 2,448 ha (6,050 ac) tract located between 34.566° and 34.611°N and 77. 614° and 77.720°W in northeastern Pender County, North Carolina, with a small portion extending approximately 0.3 km into Onslow County (Fig. 1). The geographic center of the site is approximately 8.3 km south-southeast of Maple Hill, NC and 21 km northwest of the nearest point in the Atlantic Ocean. The site lies in the outer Coastal Plain ecophysiographic province and within the Cape Fear Arch geological uplift, a region extending from southeastern North Carolina into northeastern South Carolina that is characterized by unusual outcroppings of Cretaceous deposits. This area supports a suite of at least forty-four endemic or near-endemic plant taxa, many of which are rare (LeBlond 2001).

**Figure 2.**
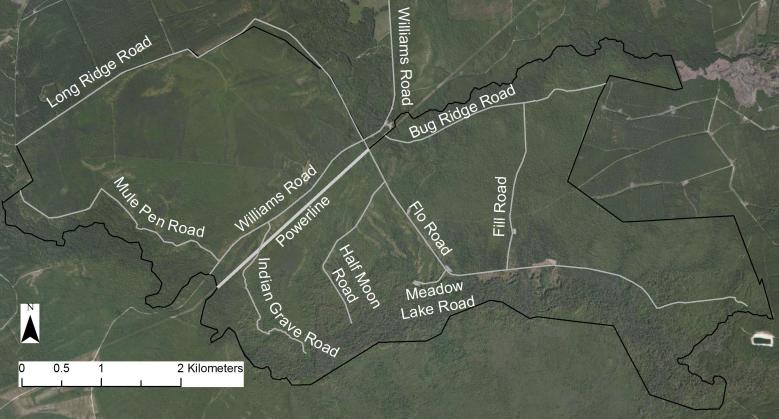
Aerial photograph of Shaken Creek Preserve with main roads labeled. Baseline imagery from Bing Maps Aerial, courtesy of Environmental Systems Research Institute (ESRI) (2011).

A small portion of SCP extends to the south of Shaken Creek, which otherwise forms the southern boundary of the property. The northeastern boundary follows Shelter Swamp Creek, while Flo Road west of its intersection with Williams Road forms the northwestern boundary (Fig. 2). With a small exception in the extreme northwest corner of the property, Long Ridge Road outlines the western property boundary. The irregular eastern boundary does not consistently follow any natural features or landmark. SCP is bordered to the southwest by Holly Shelter Gameland; to the northeast by Haw’s Run Mitigation Site, a future component of Sandy Run Savannas State Natural Area; and in other directions by private land. Primary access is south along Williams Road from NC Highway 50, though the site is not openly accessible to the public.

Sandy Run Savannas State Natural Area (“Sandy Run”) is a 1,214 ha (3,000 ac) site north of SCP (Fig. 1). It is comprised of seven tracts, six of which are currently owned by the NC Division of Parks and Recreation. One tract, Haw’s Run Mitigation Site, which abuts the northeastern portion of SCP, is currently owned by the NC Department of Transportation; however, transfer of this parcel to the NC Division of Parks and Recreation is impending. The various tracts comprising Sandy Run have experienced different management and land use histories, creating an array of habitats including several high-quality savannas and flatwoods. The flora of Sandy Run was recently inventoried by Taggart (2010). Though some portions of Sandy Run are slightly farther than two miles from SCP, vouchers or reports from all Sandy Run tracts were included in this work.

Holly Shelter Game Land is a 26,200 ha (64,742 ac) property south of SCP (Fig. 1). It is owned by the NC Wildlife Resources Commission, which manages the land for public outdoor recreation, particularly hunting and fishing. Only the northeast portion of the property lies within two miles of SCP; as such, plant vouchers or reports from other areas of Holly Shelter are not included in this work.

### History and Land Use

Prior to its purchase by The Nature Conservancy, the land comprising SCP was owned mostly by members of the Wallace Deer Club, a private hunting group established in the 1920s. The site was virtually unknown to scientists until 1997, when William Blanchard, a member of the Wallace Deer Club and part owner of the land, introduced it to Hervey McIver, a land manager and project coordinator with The Nature Conservancy. With permission from Blanchard, McIver and Richard LeBlond, then a botanist with the NC Natural Heritage Program, undertook the first preliminary surveys of the area and realized quickly that the site contained exceptionally high-quality savannas and numerous rare species. At the time, McIver was working with Blanchard to complete a deed to The Nature Conservancy for fifty acres Blanchard owned in the nearby Neck Savanna, now a tract within Sandy Run Savannas State Natural Area. Blanchard suggested that the land now comprising SCP should also be permanently conserved and eventually agreed to sell his shares of the property. However, purchasing SCP required not just the approval of Blanchard, but of all the approximately fifty landowners who inherited or purchased property rights to the site. After three years of negotiations, The Nature Conservancy closed on the property in 2007. Members of the Wallace Deer Club retained hunting rights to the property, but the site is now owned and managed by The Nature Conservancy.

The excellent quality of many of the savannas on site, as evidenced by the abundance of rare species, the high species richness, and the absence of invasive species, can be directly attributed to the Wallace Deer Club, whose members frequently burned particular areas in order to maintain both the hunting quality and the aesthetic of the land. Evidence of disturbances other than fire in the savannas and flatwoods on site is limited to a few ditches and occasional “borrow pits”, relatively small holes dug to “borrow” the soil in order to regrade and maintain the dirt roads on the property. Based on the size of the canopy trees, many savannas and flatwoods appear to have been logged as recently as the 1980s, though few or no effects on the ground layer are apparent today. Overall, the habitat quality of the site (especially those areas historically burned by members of the hunting club) remains excellent (LeBlond 2000).

### Climate

The climate at SCP is warm, temperate, and humid for much of the year. The nearest weather station is approximately 29 km away in Jacksonville (Onslow County: 34.7°N, 77.383°W) at 4.9 m above sea level. Over the thirty-year period from 1971 to 2000, the average annual temperature was 17.1 °C, with a mean annual precipitation of 1,397 mm. Average daily maximum temperature was 23.1 °C, and average daily minimum temperature was 11 °C (State Climate Office of NC, http://www.nc-climate.ncsu.edu/; Fig. 3a). The next closest weather station is approximately 35 km away in Willard (Pender County: 34.661°N, 78.046°W) at 16.7 m above sea level. Over the thirty-year period from 1971 to 2000, the average annual temperature was 17.7 °C, with a mean annual precipitation of 1,377 mm. Average daily maximum temperature was 24.2 °C, and average daily minimum temperature was 11.1 °C (State Climate Office of NC, http://www.nc-climate.ncsu.edu/; Fig. 3b). For both stations, monthly average temperatures were highest in July and August and lowest in January and February. Monthly precipitation amounts were highest in July and August for both stations and lowest in April and October in Jacksonville and April and November in Willard.

The annual growing season, defined as the number of days in five years out of ten during which the daily minimum temperatures exceed 28 °F (-2.2 °C), is 235 days in Pender County and 210 days in Onslow County (Barnhill 1990, Barnhill 1992).

Elevation at SCP ranges from 4 m (13 ft) to 12.9 m (42 ft) above sea level (NC Department of Transportation, http://www.ncdot.gov/it/gis/default.html).

### Soils

**Figure 4.**
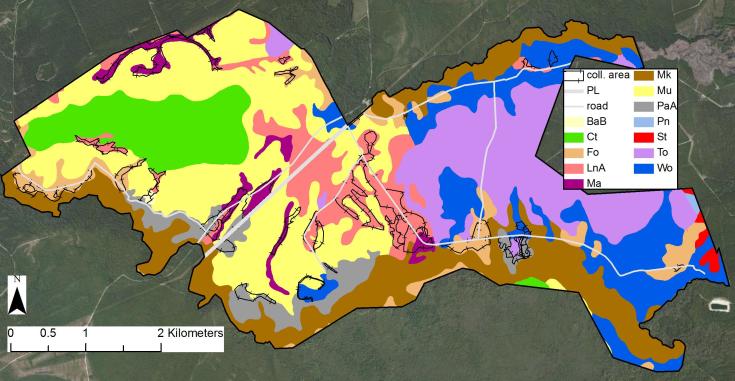
Soil mapping units at Shaken Creek Preserve. **Coll. area** = collection area; **PL** = powerline right-of-way; **BaB** = Baymeade fine sand; **Ct** = Croatan muck; **Fo** = Foreston loamy fine sand; **LnA** = Leon fine sand; **Ma** = Mandarin fine sand; **Mk** = Muckalee loam, frequently flooded; **Mu** = Murville muck; **PaA** = Pactolus fine sand; **Pn** = Pantego mucky fine sandy loam; **St** = Stallings loamy fine sand; **To** = Torhunta mucky fine sandy loam; **Wo** = Woodington fine sandy loam. Based on data from Barnhill (1990, 1992). Baseline imagery from Bing Maps Aerial, courtesy of Environmental Systems Research Institute (ESRI) (2011).

Twelve soil types representing five soil orders are mapped at SCP (Barnhill 1990, Barnhill 1992; Fig. 4). Ultisols are the most abundant in type, though Spodosols are the most abundant in area. Entisols comprise 21.4% of the total area and are the dominant soil order along Shaken Creek and Shelter Swamp Creek. While most areas mapped as Entisols support swamp communities, a few savannas do occur over Entisols, as along Alligator Lake Road and the eastern portion of Mule Pen Road. Histosols comprise 8.7% of the total area and, with one small exception, are restricted to the central western portion of the property, which is dominated by Pond Pine Woodland (Typic subtype) and High Pocosin (Typic subtype) communities (sensu Schafale 2012). No savanna or savanna-like community types are known to occur on Histosols at SCP. Inceptisols comprise 14.2% of the total area and are the dominant order in the central eastern portion of the property, an area that has not been managed with fire in many decades. Excepting one very small area on the west side of the north end of Alligator Lake Road, no savannas or savanna-like areas occur at SCP in areas mapped as Inceptisols. Spodosols comprise 36.6% of the total area and are the dominant soil order in the central, northwestern, and southestern portions of the property. Excellent examples of savannas and flatwoods occurring over Spodosols are apparent along Half Moon Road, Long Ridge Road, and the portion of Flo Road between the powerline right of way and Meadow Lake Road. Ultisols comprise 19.1% of the total area and are the dominant order in the northeastern and southeastern portions of the property. Many of the most species-rich savannas, most notably those along Flo Road east of its intersection with Fill Road, occur in areas mapped as Ultisols.

A brief synopsis of each of the twelve soil types, arranged by soil order, is provided below.

**Entisols**

*Muckalee (Mk) loam, frequently flooded* (Coarse-loamy, siliceous, superactive, non-acid, thermic Typic Fluvaquents)

Poorly-drained soils on floodplains. Slopes are 0–2%. Typical soil texture is loam in the upper 30 cm and sandy loam with thin strata of loamy sand or sand from 30 cm to 150 cm. These soils have a seasonal high water table between 15 cm and 46 cm below the soil surface and are frequently flooded for brief periods (Barnhill 1990). This is the primary mapping unit along Shaken Creek and Shelter Swamp Creek and is occupied predominantly by Blackwater Bottomland Hardwoods (High subtype) and other swamp communities.

*Pactolus (PaA) fine sand* (Thermic, coated Aquic Quartzipsamments)

Moderately well-drained or somewhat poorly-drained soils in slight depressions in uplands and on low ridges on terraces. Slopes are 0–2%. Typical soil texture is fine sand to 200 cm below the soil surface. These soils have a seasonal high water table between 46 cm and 76 cm below the soil surface and are not subject to flooding (Barnhill 1990). This mapping unit occurs primarily in narrow bands along the southern portion of the property. Three examples of the Very Wet Loamy Pine Savanna community occur on Pactolus soils.

**Histosols**

*Croatan (Ct) muck* (Loamy, siliceous, dysic, thermic Terric Haplosaprists)

Very poorly-drained soils on interstream divides. Slopes are 0–2%. Typical soil texture is muck in the upper 89 cm, fine sandy loam between 89 cm and 114 cm, sandy clay loam between 114 cm and 191 cm, and fine sandy loam between 191 cm and 200 cm. These soils have a seasonal high water table at or near the soil surface for about six months and are rarely flooded for brief periods (Barnhill 1990). This mapping unit is nearly restricted to the central-western portion of the property and supports Pond Pine Woodland (Typic subtype) and High Pocosin (Typic subtype) communities.

**Inceptisols**

*Torhunta (To) mucky fine sandy loam* (Coarse-loamy, siliceous, active, acid, thermic Typic Humaquepts)

Very poorly-drained soils on interstream areas and on stream terraces. Slopes are 0–2%. Typical soil texture is mucky fine sandy loam in the upper 8 cm, fine sandy loam between 8 cm and 152 cm, and sandy loam and sand to 200 cm. These soils have a seasonal high water table between 15 cm and 46 cm below the soil surface and are rarely flooded for brief periods (Barnhill 1990). This mapping unit occupies the central-eastern portion of the property, a fire-suppressed area that supports few or no savanna, flatwood, or sandhill communities.

**Spodosols**

*Leon (LnA) fine sand* (Sandy, siliceous, thermic Aeric Alaquods)

Poorly-drained soils on interstream areas. Slopes are 0–2%. Typical soil texture is fine sand to 200 cm below the soil surface. These soils have a seasonal high water table less than 30 cm below the soil surface and are not subject to flooding (Barnhill 1990). This is the primary mapping unit of the central portion of the property and is well-represented along Half Moon Road and the portion of Flo Road between the powerline cut and Meadow Lake Road. The best and most accessible examples of Wet Pine Flatwoods (Typic subtype), Sandy Pine Savanna (Typic subtype), and Sandy Pine Savanna (Rush featherling subtype) are found in areas mapped as Leon fine sand.

*Mandarin (Ma) fine sand* (Sandy, siliceous, thermic Oxyaquic Alorthods)

Somewhat poorly-drained soils on moderately elevated areas in interstream divides. Slopes are 0–2%. Typical soil texture is fine sand in the upper 101 cm and sand between 101 cm and 200 cm. These soils have a seasonal high water table between 46 cm and 107 cm below the soil surface and are not subject to flooding (Barnhill 1990). This mapping unit is restricted to narrow ridges in the central and western portions of the property. The largest example is in the northwest corner of the property south of Long Ridge Road, which supports extensive Wet Pine Flatwoods (Typic subtype) and Sandy Pine Savanna (Typic subtype) communities.

*Murville (Mu) muck* (Sandy, siliceous, thermic Umbric Endoaquods)

Very poorly-drained soils on interstream areas and in depressions. Slopes are 0–2%. Typical soil texture is muck in the upper 8 cm, mucky fine sand between 8 cm and 28 cm, fine sand between 28 cm and 124 cm, loamy fine sand between 124 cm and 140 cm, and fine sand between 140 cm and 200 cm. These soils have a seasonal high water table less than 30 cm below the soil surface and are not subject to flooding (Barnhill 1990). This mapping unit is the largest in area at SCP and dominates the west-central and western portions of the property. It is particularly abundant north of Mule Pen Road and along the portion of Indian Grave Road east of the powerline cut. Most areas mapped as Murville support Pond Pine Woodland (Typic subtype) communities.

**Ultisols**

*Baymeade (BaB) fine sand* (Loamy, siliceous, semiactive, thermic Arenic Hapludults)

Well-drained soils on low ridges and convex slopes in uplands. Slopes are 1–4%. Typical soil texture is fine sand in the upper 64 cm, fine sandy loam between 64 cm and 148 cm, and fine sand between 148 cm and 200 cm. These soils have a seasonal high water table between 122 cm and 152 cm below the soil surface and are not subject to flooding (Barnhill 1990). The smallest mapping unit on site, Baymeade fine sand is restricted to one area north of Mule Pen Road, approximately 1 km west of the intersection of Mule Pen Road and Williams Road. This small area is among the driest and “hilliest” at SCP and supports a Pine/Scrub Oak Sandhill (Mesic Transition subtype) community.

*Foreston (Fo) loamy fine sand* (Coarse-loamy, siliceous, semiactive, thermic Aquic Paleudults)

Moderately well-drained soils on slightly convex interstream divides near shallow drainageways. Slopes are 0–2%. Typical soil texture is loamy fine sand in the upper 33 cm, fine sandy loam between 33 cm and 102 cm, fine sandy loam with pockets of loamy fine sand between 102 cm and 140 cm, fine sandy loam with strata of loamy sand between 140 cm and 168 cm, and sandy clay loam with strata of sand and sandy loam between 168 cm and 200 cm. These soils have a seasonal high water table between 76 cm and 107 cm below the soil surface and are not subject to flooding (Barnhill 1990). Scattered throughout the property, this mapping unit is perhaps best represented along Flo Road near its intersection with Fill Road, an area that supports high-quality Wet Loamy Pine Savanna communities and, in depressional areas, Very Wet Loamy Pine Savanna communities.

*Pantego (Pn) mucky fine sandy loam* (Fine-loamy, siliceous, semiactive, thermic Umbric Paleaquults)

Very poorly-drained soils on interstream areas. Slopes are 0–2%. Typical soil texture is mucky fine sandy loam in the upper 25 cm, fine sandy loam between 25 cm and 61 cm, sandy clay loam between 61 cm and 150 cm, clay loam with strata of sandy clay loam between 150 cm and 183 cm, and sandy clay loam with thin strata of loamy sand between 183 cm and 200 cm. These soils have a seasonal high water table within 46 cm of the soil surface and are rarely flooded for brief periods (Barnhill 1990). This mapping unit is restricted to a small area in the extreme east-central portion of the property. No savannas, flatwoods, or sandhills are known from this area.

*Stallings (St) loamy fine sand* (Coarse-loamy, siliceous, semiactive, thermic Aeric Paleaquults)

Somewhat poorly-drained soils on interstream areas and in shallow depressions on convex divides. Slopes are 0–2%. Typical soil texture is loamy fine sand in the upper 30 cm, fine sandy loam between 30 cm and 114 cm, fine sandy loam with pockets of sandy clay loam between 114 cm and 168 cm, and sandy clay loam with thin layers of fine sandy loam between 168 cm and 200 cm. These soils have a seasonal high water table between 46 cm and 76 cm below the soil surface and are not subject to flooding (Barnhill 1992). As with the preceding map unit, Stallings loamy fine sand is restricted to a small area in the extreme east-central portion of the property. No savannas, flatwoods, or sandhills are known from this area.

*Woodington (Wo) fine sandy loam* (Coarse-loamy, siliceous, semiactive, thermic Typic Paleaquults)

Poorly-drained soils on interstream areas and in depressions near drainageways. Slopes are 0–2%. Typical soil texture is fine sandy loam in the upper 43 cm and fine sandy loam with pockets or strata of loamy fine sand between 43 cm and 200 cm. These soils have a seasonal high water table between 15 cm and 30 cm below the soil surface and are not generally subject to flooding, though low areas may be subject to ponding for brief periods (Barnhill 1990). This mapping unit occurs commonly in the northeastern and southeastern portions of the property, particularly along Bug Ridge Road, at the graded end of Half Moon Road, and along the far eastern portion of Flo Road. Scattered and usually small examples of savannas, flatwoods, and sandhills occur in areas mapped as Woodington fine sandy loam.

### Plant Community Types

**Figure 5.**
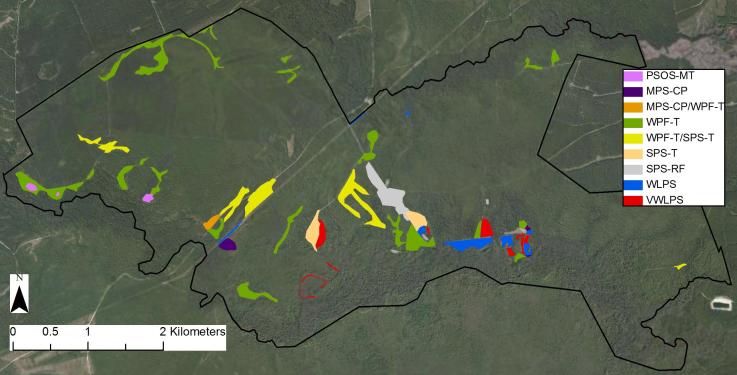
Approximate locations within Shaken Creek Preserve of the community types treated in this work. All known examples of the four Wet Pine savanna communities on site are mapped. The highest-quality examples of the other communities on site are also shown; however, fire-suppressed examples of some of these community types occur throughout the property and are not mapped. In the legend community types (sensu Schafale 2012) are arranged from driest to wettest (i.e., in order of increasing soil moisture). Areas in which two community types intergrade or co-occur in a mosaic are indicated by including the abbreviations of both community types, separated by a forward slash (e.g., MPS-CP/WPF-T). **PL** = powerline right-of-way; **PSOS-MT** = Pine/Scrub Oak Sandhill (Mesic Transition subtype); **MPS-CP** = Mesic Pine Savanna (Coastal Plain subtype); **WPF-T** = Wet Pine Flatwoods (Typic subtype); **SPS-T** = Sandy Pine Savannas (Typic subtype); **SPS-RF** = Sandy Pine Savanna (Rush Featherling subtype); **WLPS** = Wet Loamy Pine Savanna; **VWLPS** = Very Wet Loamy Pine Savanna. Baseline imagery from Bing Maps Aerial, courtesy of Environmental Systems Research Institute (ESRI) (2011).

Seven savanna, flatwoods, or sandhill plant community types were distinguished at SCP following Schafale (2012) (Table 1). Among these community types, three are globally critically imperiled (G1); the remainder are globally imperiled or vulnerable (G2G3). In general, the drier savanna or savanna-like community types (i.e., Pine/Scrub Oak Sandhill (Mesic Transition subtype) and Mesic Pine Savanna (Coastal Plain subtype)) are restricted to slightly-elevated ridges that occur mostly in the central and western portions of the property; other areas on site are typically dominated by wetter community types (i.e., Wet Pine Flatwoods (Typic subtype) or one of the four Wet Pine Savanna community types; Fig. 5).

As is true of most longleaf pine-dominated communities, all the community types treated herein are dependent on frequent, low-intensity fires to maintain their integrity (Figs 6, 7).

Though this work examines only savanna or savanna-like community types, it is worth noting that numerous other plant community types are present at SCP. Examples include Blackwater Bottomland Hardwoods (High subtype) along Shaken Creek and Shelter Swamp Creek, High Pocosin (Typic subtype) along the domed west-central portion of the property, and Pond Pine Woodland (Typic subtype and Canebrake subtype) along portions of Williams Road and Half Moon Road (Fig. 8).

In the following discussion community types are presented in order from driest to wettest (i.e., according to increasing soil moisture). For each community type the most similar NatureServe association (see http://www.natureserve.org/explorer) is provided in brackets.

**Pine/Scrub Oak Sandhill (Mesic Transition subtype; S2S3, G2G3)** [*Pinus palustris* / *Quercus incana* / *Aristida stricta* - *Sorghastrum nutans* - *Anthaenantia villosa* Woodland (CEGL003578)]. This community type is somewhat common in the Sandhills and outer Coastal Plain of North Carolina but is rare at SCP. Areas of this community type in which collection efforts have been made comprise approximately 4 ha (10 ac), all in the western portion of the property, particularly along Mule Pen Road. Associated soil series are Baymeade (Arenic Hapludult), Foreston (Aquic Paleudult), and Pactolus (Aquic Quartzipsamment; Barnhill 1990).

The canopy is dominated by *Pinus palustris* and several oak species, including *Quercus falcata* Michx., *Quercus incana* W. Bartram, *Quercus margarettae* (Ashe) Small, and Quercus marilandica Münchh. var. marilandica. Understory species include *Diospyros virginiana* L., *Gaylussacia dumosa* (Andrews) Torr. & A. Gray, *Gaylussacia frondosa* (L.) Torr. & A. Gray ex Torr., *Sassafras albidum* J. Presl, and *Vaccinium tenellum* Aiton. Vines are not abundant, but *Gelsemium sempervirens* J. St.-Hil. and *Smilax glauca* Walter are occasionally present. In the herb layer, *Aristida stricta* Michx. is abundant, and several dry-mesic species that are not found in the other communities (except sometimes in Mesic Pine Savanna (Coastal Plain subtype)) occur, including *Euphorbia ipecacuanhae* L., Lespedeza hirta (L.) Hornem. var. curtissii (Clewell) Isely, and *Tragia urens* L.

This community type is similar to and grades into Mesic Pine Savanna (Coastal Plain Subtype), from which it is distinguished by a substantial component of scrub oaks and by a less diverse herbaceous layer that generally contains fewer legume species. Most examples on site are fire-suppressed; in some cases, fire has not occurred in at least twenty years. To what extent the abundance of oaks in these cases is due simply to fire suppression rather than other environmental factors is unclear. Overall, this community type is not degraded as quickly in the absence of fire as other, wetter community types, which are subject to more rapid shrub invasion.

**Mesic Pine Savanna (Coastal Plain subtype; S2, G2G3)** [*Pinus palustris* / Amorpha herbacea var. herbacea / *Aristida stricta* - *Sorghastrum nutans* Woodland (CEGL003569)]. This community type is uncommon in North Carolina and rare at SCP. Areas of this community type in which collection efforts have been made comprise approximately 3.6 ha (9 ac), with the largest tract along Indian Grave Road and smaller tracts north of Alligator Lake Road. Sporadic examples of this community type occur in slightly elevated areas within Wet Loamy Pine Savannas and Very Wet Loamy Pine Savannas; however, due to their small size, such examples are not mapped. The soil series most commonly associated with this community type is Pactolus (Aquic Quartzipsamment), though small areas of this commuity type are mapped as Woodington (Typic Paleaquult; Barnhill 1990).

The canopy is dominated by *Pinus palustris*; oak species are generally absent or sparse. Understory species include *Gaylussacia dumosa*, *Gaylussacia frondosa*, and *Vaccinium tenellum*. *Amorpha georgiana* Wilbur and Amorpha herbacea Walter var. herbacea are excellent indicators when present, though these are not usually abundant. Vine species include *Apios americana* Medik. and *Centrosema virginianum* (L.) Benth., which are generally absent in other communities. The diverse ground layer includes several species not known from other community types, including *Danthonia sericea* Nutt., *Lechea minor* L., *Lespedeza angustifolia* (Pursh) Elliott, and *Stylosanthes biflora* (L.) Britton, Sterns & Poggenb.

This community type is similar to *Pine/Scrub Oak Sandhill* (Mesic Transition subtype), from which it is best distinguished by the absence of scrub oaks or their presence combined with wetland species, and a more diverse herbaceous layer that contains a relatively abundant and diverse component of legume species. It is distinguished from the Wet Pine Savanna community types by the lack of carnivorous plants species, the relative abundance and diversity of legume species, and the dominance of only one bunchgrass species, *Aristida stricta*, with little or no *Sporobolus pinetorum* Weakley & P.M. Peterson, no *Ctenium aromaticum*, and no *Muhlenbergia expansa* (Poir.) Trin.

**Wet Pine Flatwoods (Typic subtype; S3, G3)** [*Pinus palustris* / *Ilex glabra* / *Aristida stricta* Woodland (CEGL003648)] (Fig. 9)

The canopy consists of *Pinus palustris* and *Pinus serotina* Michx., with occasional *Pinus taeda* L. In addition to those species listed for the preceding community types, the sometimes-dense shrub layer also contains species characteristic of wetter soils, such as *Ilex glabra* (L.) A. Gray, *Kalmia carolina* Small, *Lyonia mariana* (L.) D. Don, *Morella pumila* (Michx.) Small, *Rhododendron atlanticum* (Ashe) Rehder, and *Rhododendron viscosum* (L.) Torr. Few vine taxa are present, though *Smilax laurifolia* L. is sometimes abundant. The dense herbaceous layer is dominated by *Aristida stricta*, usually with *Vaccinium crassifolium* Andrews codominant. *Pyxidanthera barbulata* Michx. and Pteridium aquilinum (L.) Kuhn var. pseudocaudatum (Clute) A. Heller are often subdominant and, when abundant, are good indicators of this community type.

The use of the terms “flatwoods” and “savannas” is notoriously variable, and sometimes contradictory, from person to person. In general “flatwoods” has been used to designate savanna-like areas that are shrubbier and/or less floristically diverse than true savannas. This work follows Schafale (2012) in distinguishing flatwoods by their floristic composition and lower small-scale species richness. While Wet Pine Flatwoods (Typic subtype) may have a naturally denser shrub layer than savannas, essentially all the community types treated in this work become shrubby in the absence of fire; relative shrub dominance is, therefore, a poor indicator of community type. Wet Pine Flatwoods (Typic subtype) often grades into Sandy Pine Savanna (Typic subtype), particularly on Spodosol soils. In these cases Wet Pine Flatwoods (Typic subtype) can be distinguished by the abundance of *Pyxidanthera barbulata*, Pteridium aquilinum var. pseudocaudatum, and *Vaccinium crassifolium*; the absence or near-absence of bunchgrass species characteristic of wetter sites, particularly *Ctenium aromaticum* and *Muhlenbergia expansa*; the absence of carnivorous species (with the exception of species of *Drosera* L.); and an overall lower small-scale species richness. In fire-suppressed areas, it is often difficult to determine whether the natural community type is Wet Pine Flatwoods (Typic subtype) or one of the Wet Pine Savannas, though some insight can be obtained by searching for remnant bunchgrasses and carnivorous plants, particularly species of *Sarracenia* L.

**Sandy Pine Savanna (Typic subtype; S3, G3)** [*Pinus palustris* - *Pinus serotina* / *Ctenium aromaticum* - *Muhlenbergia expansa* - *Carphephorus odoratissimus* Woodland (CEGL003658)]. Relatively common in North Carolina, this community type is the most common Wet Pine Savanna community at SCP. It often occurs in a mosaic with the closely related Wet Pine Flatwoods (Typic subtype). In areas where these two community types co-occur, Wet Pine Flatwoods (Typic subtype) generally occurs on slightly-elevated, drier sites and Sandy Pine Savanna (Typic subtype) on sites that are somewhat lower and wetter. The total area occupied by this community type at SCP is estimated at 13 ha (33 ac), with another 24 ha (60 ac) existing in a mosaic with Wet Pine Flatwoods (Typic subtype). Associated soil series are Leon (Aeric haplaquod) and Mandarin (Typic haplohumod; Barnhill 1990).

Canopy species are *Pinus palustris* and *Pinus serotina*. The composition of the shrub layer is geneally the same as Wet Pine Flatwoods (Typic subtype), though shrub density is often somewhat lower. As above, vines are sparse, but *Smilax laurifolia* is sometimes abundant, particularly in unburned areas. The species-rich herbaceous layer usually contains all the species present in Wet Pine Flatwoods (Typic subtype) plus many more, including several grasses (*Andropogon glaucopsis* Steud., *Andropogon glomeratus* (Walter) Britton, Sterns, & Poggenb., *Sporobolus pinetorum*, and, less commonly, *Ctenium aromaticum*), carnivorous plants (*Dionaea muscipula* J. Ellis and *Sarracenia flava* L.), and other herbs (*Osmundastrum cinnamomeum* (L.) C. Presl and *Polygala lutea* L.).

While *Pleea tenuifolia* Michx. is often found in Sandy Pine Savanna (Typic subtype), occurrences are scattered, and the species as a whole comprises only a minor component of the flora. In the closely-related Sandy Pine Savanna (Rush Featherling subtype), *Pleea tenuifolia* is a dominant species, generally as or more abundant than any single bunchgrass species. Sandy Pine Savanna (Typic subtype) can be distinguished from Wet Loamy Pine Savanna and Very Wet Loamy Pine Savanna by its coarser-textured soils and by the absence of a suite of species characteristic of wetter, richer sites, including *Chaptalia tomentosa* Vent., *Cirsium virginianum* (L.) Michx., *Eryngium* spp., *Lysimachia loomisii* Torr., *Polygala hookeri* Torr. & A. Gray, *Polygala ramosa* Elliott, and many species of *Rhynchospora* Vahl.

**Sandy Pine Savanna (Rush Featherling subtype; S1, G1)** [*Pinus palustris* - *Pinus serotina* / *Pleea tenuifolia* - *Aristida stricta* Woodland (CEGL003661)] (Fig. 10)

This community type is very similar to Sandy Pine Savanna (Typic subtype); both share the same canopy and vine species and most of the same herb species. However, the Rush Featherling subtype is distinguished by the dominance of *Pleea tenuifolia*, whose abundant white flowers in early autumn give rise to the colloquial community name “Snow in September.” The thick rhizomes of *Pleea tenuifolia* produce dense, broad clumps that create a somewhat hummocky topography. Species richness and diversity are sometimes lower in the Rush Featherling subtype than in the Typic subtype due to the sheer dominance of *Pleea tenuifolia*. The environmental factors responsible for this community type are unclear. At SCP both the Rush Featherling and Typic subtypes occur on Leon soils and in close proximity to one another. However, the author has noticed that *Pleea tenuifolia* is sometimes abundant in local depressions within the Typic subtype, an observation that suggests that *Pleea tenuifolia* possibly favors wetter soils. Perhaps, then, the Rush featherling subtype has a somewhat higher water table than the Typic subtype, though this hypothesis has not been tested.

**Wet Loamy Pine Savanna (S1, G1)** [*Pinus palustris* - *Pinus serotina* / *Ctenium aromaticum* - *Muhlenbergia expansa* - *Rhynchospora latifolia* Woodland (CEGL003660)] (Fig. 11a)

The canopy is dominated by *Pinus palustris* and *Pinus serotina*, with occasional *Pinus taeda*. The sparse to nearly absent understory consists of species similar to other Wet Pine Savanna community types. Vines are scarce, though several *Smilax* species treated in this work have been collected in thickets along the roadside edge of Wet Loamy Pine Savannas. The herbaceous layer is very diverse and generally includes all taxa present in the Sandy Pine Savanna communities plus many other taxa. Among bunchgrasses, *Ctenium aromaticum*, *Muhlenbergia expansa*, and *Sporobolus pinetorum* dominate or co-dominate with *Aristida stricta*. Herbs that are often present in Wet Loamy Pine Savannas but not in Sandy Pine Savannas include *Chaptalia tomentosa*, *Cirsium virginianum*, *Eryngium* L. spp., *Lysimachia loomisii*, *Polygala hookeri*, *Polygala ramosa*, and many *Rhynchospora* spp.

As their names imply, both Loamy Pine Savanna community types are distinguished from Sandy Pine Savanna community types by somewhat finer-textured soils. In general, finer-textured soils are more fertile than and have a higher water-holding capacity than coarser-textured soils—conditions that would seem to be favorable to the growth of most plant species. These environmental factors may explain, at least partially, the exceptionally high species richness of the Loamy Pine Savanna communities (Schafale 2012). Wet Loamy Pine Savannas are distinguished from Mesic Pine Savannas (Coastal Plain subtype) by the abundance of wetland plants and the near absence of legumes. Wet Loamy Pine Savannas are distinguished from Very Wet Loamy Pine Savannas by a lower abundance of boggy species (e.g., Eriocaulon decangulare L. var. decangulare, *Lachnocaulon anceps* (Walter) Morong, and *Taxodium ascendens* Brongn.) and by the absence of a suite of rare species (*Allium* species 1, *Carex lutea* LeBlond, and *Thalictrum cooleyi* H.E. Ahles).

**Very Wet Loamy Pine Savanna** (S1, G1) [*Pinus palustris* - *Pinus serotina* / *Magnolia virginiana* / *Sporobolus teretifolius* - *Carex striata* Woodland (CEGL004500)] (Fig. 11b)

Canopy species include those of other Wet Pine Savannas, though *Pinus palustris* is often less abundant. *Taxodium ascendens*, not usually found in the other communities, also frequently occurs. Shrub species that are more common in this community type than in others include *Morella cerifera* (L.) Small and *Ilex myrtifolia* Walter. Vines are generally uncommon, though *Mikania scandens* (L.) Willd. and Toxicodendron radicans (L.) Kuntze var. radicans are more likely to be found in this community type, particularly along swampy margins or in unburned sites, than in other community types. The herbaceous layer may include all taxa present in other Wet Pine Savannas plus an additional suite of rare species: *Allium* species 1, *Carex lutea*, and *Thalictrum cooleyi*, all of which are strong indicators for this community type. *Aristida stricta* is often scarce or even entirely absent, replaced by other bunchgrass species, particularly *Muhlenbergia expansa*. Many wetland herbs that are sometimes found in Wet Loamy Pine Savannas are often much more abundant in Very Wet Loamy Pine Savannas. Examples inlcude *Carex striata* Michx., *Chaptalia tomentosa*, and *Eryngium* spp. Boggy species, like Eriocaulon decangulare var. decangulare and *Lachnocaulon anceps*, which are restricted to borrow pits and depressions in other community types, are also more likely to occur in the savannas proper of this community type.

Globally, Very Wet Loamy Pine Savannas have a small, patchy distribution, and the environmental factors responsible for their occurrence are unclear. As noted by Schafale (2012), the abundance of wetland species would seem to indicate a wetter soil than that of Wet Loamy Pine Savannas. In most cases, however, Wet Loamy Pine Savannas grade directly into swamps or pocosins on their wet edges; why in rare cases Very Wet Loamy Pine Savannas form in these ecotonal positions is uncertain. The natural fire frequency of Very Wet Loamy Pine Savannas is also unclear but may be somewhat lower than that of the other savanna communities due to a higher water table and a suspected slightly higher natural shrub density. Some Very Wet Loamy Pine Savannas exhibit localized inclusions of calcium (in the form of marl) that increase soil pH in small areas (Schafale 2012). Rare in the Coastal Plain of NC, these inclusions were once thought to explain the curious distribution of this community type; however, many calcium deposits underlying Very Wet Loamy Pine Savannas appear to be several feet below the soil surface, where they would presumably exert little impact on the pH of the upper portion of the soil in which most plants root. Moreover, one study (Taggart 2012) suggested that even in Very Wet Loamy Pine Savannas in which inclusions of high pH have been reported, most of the soil throughout the savanna is still strongly acidic. Further research into the environmental factors associated with this community type is certainly warranted.

## Materials and methods

### Preliminary Species List

A preliminary list of plant taxa reported from SCP by LeBlond (2000) and by the Carolina Vegetation Survey (Peet et al. 2004) was compiled. Taxa collected or reported from various tracts comprising Sandy Run Savannas State Natural Area were also included; for these taxa, the following sources were referenced: LeBlond and Weakley (1991), LeBlond (1999), LeBlond (2000), Taggart (2010). Finally, taxon reports for Pender and Onslow Counties were obtained from the US Southeastern Flora Atlas (http://www.herbarium.unc.edu/seflora/firstviewer.htm), a resource that incorporates pertinent records from the USDA PLANTS Database (United States Department of Agriculture, Natural Resources Conservation Service 2012), Radford et al. (1968), and any specimens databased to-date by several herbaria, including those of North Carolina State University (NCSC) and the University of North Carolina at Chapel Hill (NCU). Culled from this initial checklist were taxa *either* reported by the various sources in habitats other than those studied in the present thesis *or*, in the case of the county records obtained through the Southeastern Flora Atlas, taxa whose habitat description in Weakley (2010) did not include savannas, flatwoods, or similar habitats. The resulting condensed list was subsequently used to search the herbarium collections of Duke University (DUKE), NCSC, NCU, and the University of North Carolina Wilmington (WNC) for any historic collections made from pertinent habitats in SCP, in tracts now comprising Sandy Run Savannas State Natural Area, or in other areas within two miles of SCP (Fig. 1).

### Field Work

Field work began in August 2010 and continued through October 2012. In order to capture the floristic diversity of SCP throughout the growing season, collecting trips (N=81) were made approximately weekly from mid-March 2011 through November 2011, biweekly from December 2011 to February 2012, weekly from early March 2012 through early September 2012, and biweekly from early September 2012 through mid-October 2012. Collecting efforts in 2011 centered on the extensive Wet Pine Savanna and Wet Pine Flatwoods community types along Flo Road and Half Moon Road (Fig. 2). In 2012 collection efforts extended to include all Wet Pine Savanna and Wet Pine Flatwoods community types throughout the property and the relatively few examples of Mesic Pine Savanna and Pine/Scrub Oak Sandhill community types on site (Fig. 5). Voucher specimens were collected in duplicate (or more) and deposited at NCSC. In addition, leaf samples were taken from most vouchers, desiccated in silica gel, and deposited in the NCSC DNA bank (see herbarium.ncsu.edu), where available for use by the scientific community. Specimen determinations were made by Robert Thornhill and were verified by the following: Richard LeBlond (*Dichanthelium*), Dr. Jon Stucky (Cyperaceae, Juncaceae, and Poaceae excluding *Dichanthelium*), and Dr. Alexander Krings (all other taxa). A list of all voucher specimens and associated data (except location data for rare or over-collected taxa) can be found in Suppl. material 6.

### Checklist

Following the completion of field work, herbarium research, and a digital querying of rare taxa reports within 2 miles of SCP (using the North Carolina Natural Heritage Program's MapViewer application http://www.ncnhp.org), a data-rich checklist of all the vascular flora collected or reported from savannas, flatwoods, or sandhill community types in SCP or the vicinity was prepared. (The checklist is available in spreadsheet format in Suppl. material 7.) Within the checklist, taxa are organized by major plant groups (i.e., Pteridophytes; Gymnosperms; Monocotyledons; Basal Angiosperms, Magnoliids, and Eudicotyledons), then alphabetically by family, genus, and species. Each taxon is accompanied by a brief entry that contains the following three sections:

- **Conservation Status**: For rare taxa (i.e., those listed by Gadd and Finnegan 2012), status and rank designations are provided in the following order: states status, federal status; state rank, global rank. The following abbreviations are used: STATE STATUS: **E** = Endangered; **T** = Threatened; **SC** = Special Concern: **-V** = Vulnerable, **-H** = Historical; **SR** = Significantly Rare: **-L** = Limited to North Carolina and adjacent states (endemic/near endemic), **-T** = Throughout, **-P** = Periphery of Range, **-O** = Other; **W** = Watch List: **W1** = rare but relatively secure, **W2** = rare but taxonomically questionable, **W5B** = exploited plants, **W7** = rare and poorly known. FEDERAL STATUS: **E** = Endangered; **FSC** = Federal Species of Concern. STATE RANK: **SH** = historical (known only from historical populations); **S1** = Critically imperiled, 1–5 populations in state; **S2** = Imperiled, 6–20 populations in state; **S3** = Vulnerable, 21–100 populations in state; **S4** = Apparently secure, 101–1000 populations in state; **S5** = Secure, 1001^+^ populations in state. GLOBAL RANK: **G1** = Critically imperiled, 1–5 populations in world; **G2** = Imperiled, 6–20 populations in world; **G3** = Vulnerable, 21–100 populations in world; **G4** = Apparently secure, 101–1000 populations in world; **G5** = Secure, 1001^+^ populations in world; **T**# = Global rank of a subspecies or variety; **Q** = Questionable taxonomy; **?** = Uncertain. (For a synopsis of all taxa of conservation concern treated in this work, see Tables 2, 3.)
- **Distribution**: The distribution of taxa is provided by listing the community types in which the taxa have been collected or reported within the study area. For taxa collected or reported from SCP, the community types (sensu Schafale 2012) in which the taxa occur on site are listed from driest to wettest (i.e., in order of increasing soil moisture) and are abbreviated as follows (see Table 1): **PSOS-MT** = Pine/Scrub Oak Sandhill (Mesic Transition subtype); **MPS-CP** = Mesic Pine Savanna (Coastal Plain subtype); **WPF-T** = Wet Pine Flatwoods (Typic subtype); **SPS-T** = Sandy Pine Savanna (Typic subtype); **SPS-RF** = Sandy Pine Savanna (Rush Featherling subtype); **WLPS** = Wet Loamy Pine Savanna; **VWLPS** = Very Wet Loamy Pine Savanna. For taxa not collected or reported from SCP but collected in the vicinity by Taggart (2010) or reported from the vicinity by LeBlond (1999) or LeBlond (2000), community types as provided by those authors are given. For all other taxa (i.e., those taxa collected or reported from the vicinity by sources other than the afoermentioned), habitat according to Weakley (2012) is provided in lieu of community types.
- **Notes**: Within each "notes" section, several bits of information are provided in the following order: 1) an estimate of abundance adadpted from Palmer et al. (1995) (see Table 4) for taxa collected by the senior author in SCP; 2) flowering and fruiting phenology from Weakley (2012) and supplemented, in some cases, with personal observation; 3) voucher information from specimens deposited in the following herbaria: DUKE, NCSC, NCU, and WNC. Within a list of vouchers, specimens collected in SCP are listed first, followed by specimens collected from the vicinity, which are arranged alphabetically by site name, then by tract name (if within Sandy Run), and finally by collector last name. For taxa of conservation concern that were collected in Sandy Run, the name of the tract in which the voucher was collected is purposefully omitted; and 4) in brackets, synonymy with Radford et al. (1968), the *Flora of North America Project*, and Weakley (2012).

Six taxa included in this guide bear numeric "placeholder" epithets, as currently listed in Weakley (2012). Of these six taxa, the following four are presumed to be new to science: *Allium* species 1, *Coreopsis* species 1, *Scleria* species 1, and *Xyris* species 1. The remaining two taxa–*Dichanthelium* species 3 and *Dichanthelium* species 12–were recognized by previous authors (see synonymy for those taxa in the checklist); however, the appropriate combination has yet to be made within *Dichanthelium*.Based on field observations by the senior author, instances of known hybridization appear to be rare in the flora. (Actual hybridization events may be more common but are beyond the scope of this research.) One notable exception, however, is Sarracenia × catesabaei Elliott (= *Sarracenia flava* L. × *Sarracenia purpurea* L.). (See the key to *Sarracenia* for a discussion of hybridization within that genus.) Hybrids are nottreated as separate taxa in this guide.

### Identification Keys

Dichotomous keys were created to all taxa collected or reported from savannas, flatwoods, or sandhill community types in SCP and the vicinity (i.e., in areas within two miles of SCP, including all tracts within Sandy Run Savannas State Natural Area). The order of the keys follows that of the checklist (i.e., a key is first provided to four main vascular plant groups, then within each of these groups, keys proceed alphabetically by family and then genus). In addition, three “auxiliary keys” are provided: a vegetative key to common savanna bunchgrasses (following the key to genera of Poaceae); a key to herbaceous eudicotyledonous taxa with simple, opposite, more-or-less ovate leaves (following the key to families of basal angiosperms, magnoliids, and eudicotyledons); and a vegetative key to frequently confused ericaceous subshrubs (following the key to genera of Ericaceae). Keys were adapted from Radford et al. (1968), the cited *Flora of North America* treatments, Weakley (2012), and personal notes. In the keys exceptional values for numeric character ranges are indicated in parentheses (e.g., leaf blade 1–2(–4) cm wide). Definitions, explanatory notes, and exceptional non-numeric character states are also placed in parentheses (e.g., corolla pink (rarely white)).

During herbarium searches, vouchers of taxa collected by others in SCP or in the vicinity but not collected by the senior author in SCP were carefully examined. In five cases the senior author disagreed with the determinations of such vouchers. Nevertheless, since the original determinations were always of taxa whose habitat and distribution make them plausible components of the flora, these taxa were included in the keys, where indicated by a plus (+) symbol. These taxa are not, however, formally treated in this work (i.e., they do not appear in the checklist) and are not included in summary statistics. Additionally, forty-four taxa that are not known from the habitats treated in this work but that often occur in roadsides or other disturbed areas immediately adjacent to such habitats, are also included in the keys, where indicated by a double-dagger (‡) symbol. These taxa, too, are neither formally treated in this work nor included in the summary statistics. Finally, though only one exotic taxon is reported in this work, several of the forty-four aforementioned taxa (those strictly of roadsides or disturbed areas) are exotic (i.e., not native to the Coastal Plain of North Carolina, sensu Weakley 2012). Exotic taxa are indicated in the keys with an asterisk (*).

## Data resources

Dichotomous keys were adapted from Radford et al. (1968), the cited *Flora of North America* treatments, Weakley (2012), and personal notes. Taxonomic concepts and nomenclature usually follow Weakley (2012) but in some cases follow the cited *Flora of North America* treatments. Status and ranks for taxa of conservation concern (i.e., those taxa listed by Gadd and Finnegan 2012) were adopted from Gadd and Finnegan (2012); see Tables 2, 3). Plant community types were identified using Schafale (2012). Line drawings were obtained from the following public domain works: Britton and Brown (1913), Hitchcock (1950), United States Department of Agriculture, Natural Resources Conservation Service (2012). All photographs, with the exception of those for *Carya tomentosa*, were taken by Robert Thornhill.

## Checklists

### PTERIDOPHYTES

#### Blechnaceae

##### Woodwardia areolata

(L.) Moore

###### Distribution

Wet pine savannas (SPS-RF, VWLPS), borrow pits, ditches.

###### Notes

Occasional. May-Sep. Thornhill 752, 876 (NCSU). Specimens seen in the vicinity: Sandy Run [Hancock]: Taggart SARU 78 (WNC!). [= RAB, FNA, Weakley].

##### Woodwardia virginica

(L.) Sm.

###### Distribution

Wet pine flatwoods (WPF-T), wet pine savannas (SPS-T, SPS-RF, WLPS, VWLPS), borrow pits, ditches, roadsides.

###### Notes

Frequent. Jun–Sep. Thornhill 570, 597, 616, 798 (NCSC). Specimens seen in the vicinity: Sandy Run [Hancock]: Taggart SARU 149 (WNC!). [= RAB, FNA, Weakley]

#### Dennstaedtiaceae

##### Pteridium aquilinum var. pseudocaudatum

(Clute) A. Heller

###### Distribution

Pine/scrub oak sandhills (PSOS-MT), mesic pine savannas (MPS-CP), wet pine flatwoods (WPF-T), wet pine savannas (SPS-T, SPS-RF, WLPS, VWLPS), roadsides.

###### Notes

Abundant. Jul–Sep. Thornhill 836, 1425 (NCSC). Specimens seen in the vicinity: Sandy Run [Hancock]: Taggart SARU 148 (WNC!). [= RAB, FNA; = Pteridium aquilinum (L.) Kuhn ssp. pseudocaudatum (Clute) Hultén sensu Weakley]

#### Lycopodiaceae

##### Lycopodiella alopecuroides

(L.) Cranfill

###### Distribution

Wet pine savannas (SPS-T, SPS-RF, WLPS, VWLPS).

###### Notes

Frequent. Jul–Sep. Thornhill 232, 785, 850 (NCSC). Specimens seen in the vicinity: Sandy Run [Hancock]: Taggart SARU 509 (WNC!). [< *Lycopodium alopecuroides* L. sensu RAB; = FNA, Weakley]

##### Lycopodiella appressa

(Chapm.) Cranfill

###### Distribution

Wet pine savannas (SPS-T, SPS-RF).

###### Notes

Occasional. Jul–Sep. Thornhill 810, 851 (NCSC). Specimens seen in the vicinity: Sandy Run [Hancock]: Taggart SARU 515 (WNC!). [= *Lycopodium appressum* (Chapm.) F.E. Lloyd & Underw. sensu RAB; = FNA, Weakley]

##### Pseudolycopodiella caroliniana

(L.) Holub

###### Distribution

Wet pine savannas (SPS-RF, WLPS).

###### Notes

Occasional. Jul–Sep. Thornhill 758, 958 (NCSC). Specimens seen in the vicinity: Sandy Run [Hancock]: Taggart SARU 508 (WNC!).

#### Osmundaceae

##### Osmunda spectabilis

Willd.

###### Distribution

Wet pine flatwoods (WPF-T), wet pine savannas (WLPS, VWLPS), ditches.

###### Notes

Occasional. Mar–Jun. Thornhill 202, 300 (NCSC). Specimens seen in the vicinity: Sandy Run [Hancock]: Taggart SARU 112 (WNC!). [= Osmunda regalis L. var. spectabilis (Willd.) A. Gray sensu RAB, FNA; = Weakley]

##### Osmundastrum cinnamomeum

(L.) C. Presl

###### Distribution

Wet pine flatwoods (WPF-T), wet pine savannas (SPS-T, SPS-RF, WLPS, VWLPS).

###### Notes

Frequent. Mar–May. Thornhill 201, 223, 255 (NCSC). Specimens seen in the vicinity: Sandy Run [Hancock]: Taggart SARU 75 (WNC!); Sandy Run [Neck]: Wilbur 67806 (DUKE!; as *Osmunda cinnamomea*). [= *Osmunda cinnamomea* L. sensu RAB, FNA; = Weakley]

#### Selaginellaceae

##### Selaginella apoda

(L.) C. Morren

###### Distribution

Wet pine savannas (WLPS, VWLPS).

###### Notes

Infrequent. Jun–Oct. Thornhill 1480 (NCSC). Specimens seen in the vicinity: Sandy Run [Hancock]: Taggart SARU 124 (WNC!); Sandy Run [Neck]: Sorrie 6385 (NCU!). [= RAB, FNA, Weakley]

### GYMNOSPERMS

#### Cuppressaceae

##### Chamaecyparis thyoides

(L.) Britton, Sterns & Poggenb.

###### Distribution

Depressions in pine savannas, ditches, borrow pits.

###### Notes

Rare. Mar–Apr; Oct–Nov. Thornhill 757 (NCSC). Specimens seen in the vicinity: Sandy Run [Hancock]: Sieren 3676 (WNC!), Taggart SARU 69 (WNC!); Sandy Run [O’Berry]: Weakley 7219 (NCU!). [= RAB, FNA, Weakley]

##### Juniperus virginiana var. virginiana

L.

###### Distribution

Thickets along roadside edges of wet pine savannas.

###### Notes

Rare. Jan–Feb; Oct–Nov. Thornhill 1381 (NCSC). [= Juniperus virginiana L. sensu RAB; = FNA, Weakley]

##### Taxodium ascendens

Brongn.

###### Distribution

Wet pine savannas (WLPS, VWLPS), ditches, borrow pits.

###### Notes

Frequent. Mar–Apr; Oct. Thornhill 474 (NCSC). Specimens seen in the vicinity: Sandy Run [Neck]: Wilbur 53703 (DUKE!); Sandy Run [O’Berry]: Taggart SARU 245 (WNC!). [= RAB; = Taxodium distichum L. var. imbricarium (Nutt.) Croom sensu FNA; = Weakley]

#### Pinaceae

##### Pinus elliottii var. elliottii

Engelm.

###### Ecological interactions

####### Native status

nonnative

###### Distribution

Wet pine flatwoods (WPF-T).

###### Notes

Infrequent. Jan–Feb.; Oct–Nov. Planted on site as a timber tree prior to site’s purchase by The Nature Conservancy. Thornhill 1554 (NCSC). [< *Pinus elliottii* Engelm. sensu RAB; = FNA, Weakley]

##### Pinus palustris

Mill.

###### Distribution

Pine/scrub oak sandhills (PSOS-MT), mesic pine savannas (MPS-CP), wet pine flatwoods (WPF-T), wet pine savannas (SPS-T, SPS-RF, WLPS, VWLPS).

###### Notes

Abundant. Mar–Apr; Sep–Oct. Thornhill 1066, 1067 (NCSC). Specimens seen in the vicinity: Sandy Run [O’Berry]: Taggart SARU 20 (WNC!). [= RAB, FNA, Weakley]

##### Pinus serotina

Michx.

###### Distribution

Wet pine flatwoods (WPF-T), wet pine savannas (SPS-T, SPS-RF, WLPS, VWLPS).

###### Notes

Abundant. Apr; Aug (or any time of the year in response to fire). Thornhill 472 (NCSC). Specimens seen in the vicinity: Sandy Run [Neck]: Wilbur 63779 (DUKE!); Sandy Run [O’Berry]: Taggart SARU 18 (WNC!). [= RAB, FNA, Weakley]

##### Pinus taeda

L.

###### Distribution

Wet pine flatwoods (WPF-T), wet pine savannas (WLPS, VWLPS).

###### Notes

Frequent. Mar–Apr; Oct–Nov. Thornhill 471, 1026 (NCSC). Specimens seen in the vicinity: Sandy Run [Hancock]: Taggart SARU 53 (WNC!). [= RAB, FNA, Weakley]

### MONOCOTYLEDONS

#### Agavaceae

##### Yucca filamentosa

L.

###### Distribution

Mesic pine savannas (MPS-CP).

###### Notes

Infrequent. Late Apr–early Jun; Sep–Oct. Thornhill 1011 (NCSC). Specimens seen in the vicinity: Sandy Run [Patterson]: Taggart SARU 157 (WNC!). [= Yucca filamentosa L. var. filamentosa sensu RAB; = FNA, Weakley]

#### Amaryllidaceae

##### Allium species 1

###### Ecological interactions

####### Conservation status

SR-L, FSC; S1S2, G1G2.

###### Distribution

Wet pine savannas (VWLPS).

###### Notes

Rare. Late Aug–early Oct; late Sep–Nov. Thornhill 839, 1009 (NCSC). Specimens seen in the vicinity: Highway 50: LeBlond 6362 (NCU!); Sandy Run: LeBlond 5541, 6361, 6363, 6370, 6377 (NCU!), Leonard 7581, 7582, 7584 (NCU!), Taggart SARU 452 (WNC!). [= Weakley]

#### Bromeliaceae

##### Tillandsia usneoides

(L.) L.

###### Distribution

Swampy margins of wet pine savannas (WLPS, VWLPS).

###### Notes

Infrequent. Apr–Jun. Thornhill 190 (NCSC). Specimens seen in the vicinity: Sandy Run [Hancock]: Taggart SARU 436 (WNC!). [= RAB, FNA, Weakley]

#### Burmanniaceae

##### Burmannia capitata

(Walter ex J.F. Gmel.) Mart.

###### Distribution

Depressions in wet pine flatwoods (WPF-T), wet pine savannas (WLPS), borrow pits, roadsides.

###### Notes

Infrequent. Jul–Nov. Thornhill 1472 (NCSC). Specimens seen in the vicinity: Sandy Run [Hancock]: Taggart SARU 651 (WNC!). [= RAB, FNA, Weakley]

#### Colchicaceae

##### Uvularia puberula

Michx.

###### Distribution

Pine savannas.

###### Notes

Early Apr–early May; Aug–Oct. Not seen in Shaken Creek Preserve by the senior author. Specimens seen in the vicinity: Sandy Run [Hancock]: Taggart SARU 46 (WNC!; as Uvularia puberula var. nitida). [= *Uvularia pudica* (Walter) Fernald sensu RAB; = FNA, Weakley]

#### Cyperaceae

##### Bulbostylis stenophylla

(Elliott) C.B. Clarke

###### Distribution

Pine savannas.

###### Notes

Jul–Oct. Not seen in Shaken Creek Preserve by the senior author. Specimens seen in the vicinity: Sandy Run [Neck]: Wilbur 53633 (DUKE!). [= RAB, FNA, Weakley]

##### Carex chapmanii

Steud.

###### Ecological interactions

####### Conservation status

W1; S3, G3.

###### Distribution

Wet pine savannas (VWLPS).

###### Notes

Apr–May. Reported from Sandy Run by LeBlond (2000), but no specimens have been seen in Shaken Creek Preserve by the senior author. [= RAB, FNA, Weakley]

##### Carex elliottii

Schwein. & Torr.

###### Distribution

Depressions in wet pine savannas (SPS-T), borrow pits, ditches.

###### Notes

Infrequent. May–Jun. Thornhill 532, 1271 (NCSC). Specimens seen in the vicinity: Sandy Run [Hancock]: Taggart SARU 275 (WNC!). [= RAB, FNA, Weakley]

##### Carex glaucescens

Elliott

###### Distribution

Wet pine flatwoods (WPF-T), wet pine savannas (SPS-T, SPS-RF, WLPS, VWLPS), borrow pits, ditches.

###### Notes

Frequent. Jul–Sep. Thornhill 12, 620, 692, 1100 (NCSC). Specimens seen in the vicinity: Sandy Run [Hancock]: Taggart SARU 486 (WNC!); Sandy Run [Neck]: Wilbur 53685 (DUKE!). [= RAB, FNA, Weakley]

##### Carex leptalea harperi

(Fernald) Calder & Roy L. Taylor

###### Distribution

Wet pine savannas (VWLPS).

###### Notes

May–Jun. Reported from Sandy Run [Watkins] by LeBlond (2000), but no specimens have been seen in Shaken Creek Preserve (in the pertinent habitats) by the senior author. [< *Carex leptalea* Wahlenb. sensu RAB; = FNA; = Carex leptalea Wahlenb. var. harperi (Fernald) sensu Weakley]

##### Carex lonchocarpa

Willd. ex Spreng.

###### Distribution

Wet pine savannas (WLPS, VWLPS).

###### Notes

Occasional. May–Jul. Thornhill 369, 456, 462, 463, 1395 (NCSC). Specimens seen in the vicinity: Sandy Run [Neck]: Taggart SARU 606 (WNC!). [= Carex folliculata L. var. australis L.H. Bailey sensu RAB; = FNA, Weakley]

##### Carex lutea

LeBlond

###### Ecological interactions

####### Conservation status

State E, Fed E; S2, G2.

###### Distribution

Wet pine savannas (VWLPS).

###### Notes

Infrequent. May–early Jun. Sorrie 10149 (NCU!), Thornhill 1277 (NCSC). Specimens seen in the vicinity: Sandy Run: Taggart SARU 88, SARU 701, SARU 702, SARU 703 (WNC!). [= FNA, Weakley]

##### Carex physorhyncha

Liebm. ex Steud.

###### Ecological interactions

####### Conservation status

W1; S2S3, G5T5.

###### Distribution

Dry woodlands.

###### Notes

Late Mar–May. Reported from Sandy Run by LeBlond and Weakley (1991), but no specimens have been seen in Shaken Creek Preserve by the senior author. [= RAB; = Carex albicans Willd. ex Spreng. var. australis (L.H. Bailey) Rettig sensu FNA; = Weakley]

##### Carex striata

Michx.

###### Distribution

Wet pine savannas (SPS-RF, WLPS, VWLPS), borrow pits, ditches.

###### Notes

Occasional. May–Jun. Thornhill 1272, 1280, 1290 (NCSC). Specimens seen in the vicinity: Sandy Run [Neck]: Taggart SARU 607 (WNC!; as Carex striata var. brevis). [= *Carex walteriana* L.H. Bailey sensu RAB; = FNA; > Carex striata var. brevis L.H. Bailey, Carex striata var. striata sensu Weakley]

##### Carex venusta

Dewey

###### Distribution

Boggy depressions in pine savannas.

###### Notes

May–Jun. Not seen in Shaken Creek Preserve (in pertinent habitats) by the senior author. Specimens seen in the vicinity: Sandy Run [Hancock]: Sorrie 6395 (NCU!; as *Carex oblita*). [= RAB, FNA; > *Carex venusta* Dewey, *Carex oblita* Steud. sensu Weakley]

##### Cladium jamaicense

Crantz

###### Distribution

Wet pine savannas (VWLPS).

###### Notes

Infrequent. Jul–Oct. Thornhill 1361 (NCSC). Specimens seen in the vicinity: Sandy Run [Neck]: Taggart SARU 575 (WNC!). [= RAB, FNA, Weakley]

##### Cladium mariscoides

(Muhl.) Torr.

###### Ecological interactions

####### Conservation status

SR-O; S3, G5.

###### Distribution

Wet pine savannas (WLPS).

###### Notes

Rare. Jul–Sep. Reported from Shaken Creek Preserve by LeBlond (2000), but no specimens have been seen by the senior author. [= RAB, FNA, Weakley]

##### Cyperus haspan

L.

###### Distribution

Pine savannas.

###### Notes

Jul–Sep. Not seen in Shaken Creek Preserve (in perintent habitats) by the senior author. Specimens seen in the vicinity: Sandy Run [Haw’s Run]: Taggart SARU 659 (WNC!); Sandy Run [Neck]: Wilbur 53635 (DUKE!). [= RAB, FNA, Weakley]

##### Dulichium arundinaceum var. arundinaceum

(L.) Britton

###### Distribution

Depressions in wet pine savannas (WLPS), ditches.

###### Notes

Infrequent. Jul–Oct. Thornhill 1387, 1532 (NCSC). Specimens seen in the vicinity: Sandy Run [RMK]: Taggart SARU 232 (WNC!). [< *Dulichium arundinaceum* (L.) Britton sensu RAB; = FNA, Weakley]

##### Eleocharis baldwinii

(Torr.) Chapm.

###### Distribution

Depressions in wet pine flatwoods (WPF-T), ditches, and borrow pits.

###### Notes

Infrequent. Jun–Oct. Thornhill 1462, 1512, 1523 (NCSC). [= RAB, FNA, Weakley]

##### Eleocharis equisetoides

(Elliott) Torr.

###### Ecological interactions

####### Conservation status

W1; S3, G4.

###### Distribution

Borrow pits.

###### Notes

Rare. Jun–Sep. LeBlond 4988 (NCU!), Thornhill 279 (NCSC). [= RAB, FNA, Weakley]

##### Eleocharis microcarpa

Torr.

###### Distribution

Depressions in wet pine flatwoods (WPF-T) and wet pine savannas (SPS-T, SPS-RF, WLPS, VWLPS), borrow pits, ditches.

###### Notes

Occasional. Jun–Sep. Thornhill 18, 505, 723, 1432 (NCSC). Specimens seen in the vicinity: Sandy Run [Hancock]: Taggart SARU 621 (WNC!). [< *Eleocharis microcarpa* Torr. sensu RAB; = FNA, Weakley]

##### Eleocharis obtusa

(Willd.) Schult.

###### Distribution

Depressions in pine savannas, ditches, other wet, disturbed areas.

###### Notes

Jun–Oct. Not seen in Shaken Creek Preserve by the senior author. Specimens seen in the vicinity: Sandy Run [Neck]: Wilbur 55285 (DUKE!). [< *Eleocharis ovata* R. Br. sensu RAB; = FNA, Weakley]

##### Eleocharis tuberculosa

(Michx.) Roem. & Schult.

###### Distribution

Wet pine savannas (SPS-T, SPS-RF, VWLPS), ditches, roadsides.

###### Notes

Occasional. Jun–Sep. Thornhill 1305, 1493 (NCSC). Specimens seen in the vicinity: Sandy Run [Haw’s Run]: Taggart SARU 555 (WNC!). [= RAB, FNA, Weakley]

##### Fimbristylis puberula var. puberula

(Michx.) Vahl

###### Distribution

Wet pine savannas (SPS-T, SPS-RF, WLPS, VWLPS).

###### Notes

Infrequent. Jul–Sep. Thornhill 326, 373, 518 (NCSC). Specimens seen in the vicinity: Sandy Run [Hancock]: Taggart SARU 194 (WNC!); Sandy Run [Neck]: Levy s.n. (DUKE!). [< *Fimbristylis spadicea* (L.) Vahl sensu RAB; = FNA, Weakley]

##### Fuirena breviseta

(Coville) Coville

###### Distribution

Wet pine savannas (WLPS, VWLPS).

###### Notes

Infrequent. Jul–Oct. Thornhill 736, 737, 852 (NCSC). Specimens seen in the vicinity: Sandy Run [Haw’s Run]: Taggart SARU 381 (WNC!); Sandy Run [Neck]: Wilbur 53687 (DUKE!; as *Fuirena squarrosa*). [< *Fuirena squarrosa* Michx. sensu RAB; = FNA, Weakley]

##### Fuirena pumila

(Torr.) Spreng.

###### Distribution

Wet pine savannas (WLPS).

###### Notes

Rare. Jul–Oct. Thornhill 1533 (NCSC). [= RAB, FNA, Weakley]

##### Isolepis carinata

Hook. & Arn. ex Torr.

###### Ecological interactions

####### Conservation status

SR-P; S1, G5.

###### Distribution

Wet pine savannas (SPS-T), adjacent roadsides.

###### Notes

Rare. May–Jun. Thornhill 1263 (NCSC). [= *Scirpus koilolepis* (Steud.) Gleason sensu RAB; = FNA, Weakley]

##### Kyllinga odorata

Vahl

###### Distribution

Disturbed areas in wet pine flatwoods (WPF-T), roadsides.

###### Notes

Infrequent. Jul–Sep. Thornhill 1363 (NCSC). Specimens seen in the vicinity: Sandy Run [Hancock]: Taggart SARU 442 (WNC!). [= *Cyperus sesquiflorus* (Torr.) Mattf. & Kük. sensu RAB; = FNA, Weakley]

##### Rhynchospora baldwinii

A. Gray

###### Distribution

Wet pine flatwoods (WPF-T), wet pine savannas (SPS-T, WLPS, VWLPS)

###### Notes

Infrequent. Jul–Aug. Thornhill 629 (NCSC). [= RAB, FNA, Weakley]

##### Rhynchospora caduca

Elliott

###### Distribution

Wet pine flatwoods (WPF-T), wet pine savannas (SPS-RF, WLPS, VWLPS).

###### Notes

Frequent. Jul–Sep. Thornhill 729, 733, 868, 959, 1345 (NCSC). Specimens seen in the vicinity: Sandy Run [Hancock]: Taggart SARU 369 (WNC!); Sandy Run [Neck]: Wilbur 53656, 53657, 53683 (DUKE!). [= RAB, FNA, Weakley]

##### Rhynchospora cephalantha var. cephalantha

A. Gray

###### Distribution

Wet pine flatwoods (WPF-T), wet pine savannas (SPS-T, SPS-RF, WLPS, VWLPS), ditches.

###### Notes

Frequent. Jul–Oct. Thornhill 9, 661, 721, 735, 783, 796, 822 (NCSC). Specimens seen in the vicinity: Sandy Run [Hancock]: Taggart SARU 385 (WNC!; as Rhynchospora cephalantha var. pleiocephala); Sandy Run [Neck]: Taggart 81 (NCU; as *Rhynchospora cephalantha*); Sandy Run [Patterson]: Taggart SARU 635 (WNC!). [< *Rhynchospora cephalantha* sensu RAB, FNA; = Weakley]

##### Rhynchospora chalarocephala

Fernald & Gale

###### Distribution

Wet pine savannas (SPS-T), adjacent roadsides.

###### Notes

Frequent. Jul–Sep. Thornhill 15, 814, 901 (NCSC). Specimens seen in the vicinity: Sandy Run [Hancock]: Taggart SARU 505 (WNC!); Sandy Run [Neck]: Wilbur 57612, 57615 (DUKE!). [= RAB, FNA, Weakley]

##### Rhynchospora chapmanii

M.A. Curtis

###### Distribution

Wet pine savannas (SPS-T, SPS-RF, WLPS, VWLPS).

###### Notes

Occasional. Jul–Sep. Thornhill 777, 809, 1505 (NCSC). Specimens seen in the vicinity: Sandy Run [Hancock]: Taggart SARU 518 (WNC!); Sandy Run [Neck]: Wilbur 57616, 57622 (DUKE!). [= RAB, FNA, Weakley]

##### Rhynchospora ciliaris

(Michx.) C. Mohr

###### Distribution

Wet pine savannas (SPS-T, SPS-RF, WLPS, VWLPS), wet pine flatwoods (WPF-T).

###### Notes

Frequent. Jul–Sep. Thornhill 397, 506, 511, 654 (NCSC). Specimens seen in the vicinity: Sandy Run [Hancock]: Taggart SARU 386 (WNC!); Sandy Run [Neck]: Wilbur 57609 (DUKE!). [= RAB, FNA, Weakley]

##### Rhynchospora colorata

(L.) H. Pfeiff.

###### Distribution

Wet pine savannas (WLPS, VWLPS).

###### Notes

Infrequent. May–Sep. Thornhill 319, 328, 441, 484, 684 (NCSC). Specimens seen in the vicinity: Sandy Run [Hancock]: Taggart SARU 173 (WNC!). [= *Dichromena colorata* (L.) H. Pfeiff. sensu RAB; = FNA, Weakley]

##### Rhynchospora corniculata

(Lam.) A. Gray

###### Distribution

Cypress savannas, other wetlands.

###### Notes

Jul–Sep. Reported from Sandy Run [Neck] by LeBlond and Weakley (1991), but no specimens have been seen in Shaken Creek Preserve by the senior author. [= RAB, FNA; < Rhynchospora corniculata (Lam.) A. Gray var. corniculata sensu Weakley]

##### Rhynchospora debilis

Gale

###### Distribution

Pine savannas.

###### Notes

Jul–Sep. Not seen in Shaken Creek Preserve by the senior author. Specimens seen in the vicinity: Sandy Run [Neck]: LeBlond 2260 (NCU!). [= RAB, FNA, Weakley]

##### Rhynchospora decurrens

Chapm.

###### Ecological interactions

####### Conservation status

State T, FSC; S1S2, G3G4.

###### Distribution

Wet pine savannas (VWLPS).

###### Notes

Rare. Jul–Aug. Thornhill 1390 (NCSC). [= RAB, FNA, Weakley]

##### Rhynchospora distans

(Michx.) Vahl

###### Distribution

Wet pine flatwoods (WPF-T), wet pine savannas (SPS-RF).

###### Notes

Infrequent. Jun–Sep. Thornhill 659 (NCSC). Specimens seen in the vicinity: Sandy Run [O’Berry]: Taggart SARU 516 (WNC!; as Rhynchospora fascicularis var. distans). [< *Rhynchospora fascicularis* (Michx.) Vahl sensu RAB, FNA; = Weakley]

##### Rhynchospora divergens

Chapm. ex M.A. Curtis

###### Ecological interactions

####### Conservation status

SR-P; S2, G4.

###### Distribution

Pine savannas.

###### Notes

May–Sep. Not seen in Shaken Creek Preserve by the senior author. Specimens seen in the vicinity: Sandy Run: LeBlond 4586 (NCU!), Taggart SARU 612 (WNC!). [= RAB, FNA, Weakley]

##### Rhynchospora fascicularis

(Michx.) Vahl

###### Distribution

Wet pine flatwoods (WPF-T), wet pine savannas (SPS-T, SPS-RF).

###### Notes

Jun–Sep. Reported from Shaken Creek Preserve by LeBlond (2000), but no specimens have been seen by the senior author. [< RAB, FNA; = Weakley]

##### Rhynchospora filifolia

A. Gray

###### Distribution

Wet pine savannas (SPS-T, WLPS, VWLPS).

###### Notes

Frequent. Jul–Sep. Thornhill 396, 635, 697, 727, 811 (NCSC). [= RAB, FNA, Weakley]

##### Rhynchospora galeana

Naczi, W.M. Knapp & G. Moor

###### Ecological interactions

####### Conservation status

SR-P; S2S3, G3G4.

###### Distribution

Wet pine savannas (SPS-RF).

###### Notes

Infrequent. Jul–Sep. LeBlond 6111 (NCU); Thornhill 784 (NCSC). Specimens seen in the vicinity: Sandy Run: Taggart SARU 666 (WNC!; as *Rhynchospora breviseta*). [= *Rhynchospora breviseta* (Gale) Channell sensu RAB, FNA; = Weakley]

##### Rhynchospora globularis

(Chapm.) Small

###### Distribution

Wet pine savannas (VWLPS), adjacent roadsides.

###### Notes

Infrequent. Jun–Sep. Thornhill 252 (NCSC). [< RAB; = Rhynchospora globularis (Chapm.) Small var. globularis sensu FNA; = Weakley]

##### Rhynchospora glomerata var. glomerata

(L.) Vahl

###### Distribution

Pine savannas.

###### Notes

Jul–Sep. Not seen in Shaken Creek Preserve by the senior author. Specimens seen in the vicinity: Sandy Run [Neck]: Taggart SARU 533 (WNC!). [< *Rhynchospora glomerata* (L.) Vahl sensu RAB, FNA; = Weakley]

##### Rhynchospora gracilenta

A. Gray

###### Distribution

Wet pine savannas (SPS-RF, WLPS, VWLPS).

###### Notes

Occasional. Jul–Sep. Thornhill 626, 1096, 1103 (NCSC). Specimens seen in the vicinity: Sandy Run [Hancock]: Taggart SARU 473 (WNC!). [= RAB, FNA, Weakley]

##### Rhynchospora inexpansa

(Michx.) Vahl

###### Distribution

Wet pine savannas (SPS-T, SPS-RF, WLPS), adjacent roadsides.

###### Notes

Frequent. Jul–Sep. Thornhill 633, 645, 652, 655, 695 (NCSC). Specimens seen in the vicinity: Sandy Run [Neck]: Wilbur 53640, 53670 (DUKE!); Sandy Run [O’Berry]: Taggart SARU 519 (WNC!). [= RAB, FNA, Weakley]

##### Rhynchospora latifolia

(Baldwin) W.W. Thomas

###### Distribution

Wet pine savannas (WLPS, VWLPS), ditches, borrow pits.

###### Notes

Occasional. May–Sep. Thornhill 11, 356, 451, 529 (NCSC). Specimens seen in the vicinity: Sandy Run [Haw’s Run]: Taggart SARU 185 (WNC!); Sandy Run [Neck]: Levy s.n. (DUKE!; as *Dichromena latifolia*), Wilbur 53697 (DUKE!; as *Dichromena latifolia*). [= *Dichromena latifolia* Baldwin ex Elliott sensu RAB; = FNA, Weakley]

##### Rhynchospora macrostachya

Torr. ex A. Gray

###### Distribution

Wet pine savannas (WLPS), borrow pits.

###### Notes

Infrequent. Jul–Sep. Thornhill 918 (NCSC). Specimens seen in the vicinity: Sandy Run [Haw’s Run]: Taggart SARU 256 (WNC!; as Rhynchospora macrostachya var. macrostachya); Sandy Run [Neck]: Wilbur 53684 (DUKE!). [= RAB, FNA, Weakley]

##### Rhynchospora microcarpa

Baldwin ex A. Gray

###### Ecological interactions

####### Conservation status

SR-P; S2, G5.

###### Distribution

Wet pine savannas (VWLPS).

###### Notes

Infrequent. Jul–Aug. Thornhill 517, 731 (NCSC). [= RAB, FNA, Weakley]

##### Rhynchospora microcephala

(Britton) Britton ex Small

###### Distribution

Pine savannas.

###### Notes

Jul–Oct. Not seen in Shaken Creek Preserve by the senior author. Specimens seen in the vicinity: Sandy Run [Neck]: LeBlond 2387 (NCU!). [= RAB, FNA, Weakley]

##### Rhynchospora mixta

Britton

###### Distribution

Depressions in wet pine savannas (VWLPS), ditches.

###### Notes

Rare. Jun–Aug. More commonly a species of swamps and marshes, *Rhynchospora mixta* was reported from savannas in Sandy Run by LeBlond (1999). A specimen (Thornhill 1407, NCSC) was collected in a swamp in Shaken Creek Preserve by the senior author, but no specimens have been seen in savannas. [= RAB, FNA, Weakley]

##### Rhynchospora nitens

(Vahl) A. Gray

###### Ecological interactions

####### Conservation status

W1; S3, G4?.

###### Distribution

Wet pine savannas (WLPS), ditches, borrow pits.

###### Notes

Occasional. Jul–Aug. Thornhill 17, 931 (NCSC). Specimens seen in the vicinity: Sandy Run: Taggart SARU 415 (WNC!). [= *Psilocarya nitens* (Vahl) Alph. Wood sensu RAB; = FNA, Weakley]

##### Rhynchospora oligantha

A. Gray

###### Ecological interactions

####### Conservation status

W1; S3, G4.

###### Distribution

Pine savannas.

###### Notes

Jul–Aug. Not seen in Shaken Creek Preserve by the senior author. Specimens seen in the vicinity: Sandy Run: Taggart SARU 554 (WNC!). [= RAB, FNA, Weakley]

##### Rhynchospora pallida

M.A. Curti

###### Ecological interactions

####### Conservation status

W1; S3, G3.

###### Distribution

Wet pine flatwoods (WPF-T), wet pine savannas (SPS-T, SPS-RF, WLPS).

###### Notes

Infrequent. Jul–Sep. Thornhill 14, 663 (NCSC). Specimens seen in the vicinity: Sandy Run: Taggart SARU 215 (WNC!). [= RAB, FNA, Weakley]

##### Rhynchospora pinetorum

Britton & Small

###### Ecological interactions

####### Conservation status

SR-T; S2, G5?T3?.

###### Distribution

Wet pine savannas (VWLPS).

###### Notes

Infrequent. Jul–Sep. Thornhill 515 (NCSC). Specimens seen in the vicinity: Sandy Run: Taggart SARU 473 (WNC!). [< *Rhynchospora globularis* (Chapm.) Small sensu RAB; = Rhynchospora globularis (Chapm.) Small var. pinetorum (Britton & Small) Gale sensu FNA; = Weakley]

##### Rhynchospora plumosa

Elliott

###### Distribution

Wet pine flatwoods (WPF-T), wet pine savannas (SPS-T, SPS-RF, WLPS, VWLPS).

###### Notes

Frequent. Jul–Aug. Thornhill 313, 357, 443, 467, 468, 726, 772, 778, 929 (NCSC). Specimens seen in the vicinity: Sandy Run [Hancock]: Taggart SARU 239 (WNC!); Sandy Run [Neck]: Taggart 27 (NCU!); Wilbur 57623 (DUKE!). [= RAB, FNA, Weakley]

##### Rhynchospora pusilla

Chapm. ex M.A. Curtis

###### Distribution

Wet pine savannas (SPS-T, VWLPS), adjacent roadsides.

###### Notes

Occasional. Jun–Sep. Thornhill 24, 504, 507, 566, 860 (NCSC). Specimens seen in the vicinity: Sandy Run [Hancock]: Taggart SARU 372 (WNC!). [= *Rhynchospora intermixta* C. Wright sensu RAB; = FNA, Weakley]

##### Rhynchospora rariflora

(Michx.) Elliott

###### Distribution

Wet pine savannas (WLPS, VWLPS).

###### Notes

Occasional. Jul–Sep. Thornhill 516, 579, 1102, 1355 (NCSC). [= RAB, FNA, Weakley]

##### Rhynchospora scirpoides

(Torr.) Griseb.

###### Ecological interactions

####### Conservation status

W1; S3, G4.

###### Distribution

Pine savannas.

###### Notes

Jul–Sep. Not seen in Shaken Creek Preserve by the senior author. Specimens seen in the vicinity: Sandy Run: Wilbur 57613 (DUKE!), Wilbur 57619 (DUKE!). [= *Psilocarya scirpoides* Torr. sensu RAB; = FNA, Weakley]

##### Rhynchospora thornei

Kral

###### Ecological interactions

####### Conservation status

SC-V, FSC; S2, G3.

###### Distribution

Wet pine savannas (VWLPS), adjacent roadsides.

###### Notes

Infrequent. Jul–Sep. LeBlond 6127 (NCU!). Specimens seen in the vicinity: Sandy Run: LeBlond 2851 (DUKE!), LeBlond 5514 (NCSC!), Taggart SARU 634 (WNC!). [= FNA, Weakley]

##### Rhynchospora torreyana

A. Gray

###### Distribution

Wet pine savannas (WLPS, VWLPS).

###### Notes

Occasional. Jul–Sep. Thornhill 8, 775, 779 (NCSC). Specimens seen in the vicinity: Sandy Run [Haw’s Run]: Taggart SARU 317 (WNC!); Sandy Run [Neck]: Wilbur 53638, 53646, 53647 (DUKE!). [= RAB, FNA, Weakley]

##### Rhynchospora wrightiana

Boeck.

###### Ecological interactions

####### Conservation status

W1; S3, G5.

###### Distribution

Wet pine savannas (SPS-T).

###### Notes

Infrequent. Jul–Sep. Thornhill 16 (NCSC). [= RAB, FNA, Weakley]

##### Schoenoplectus pungens var. pungens

(Vahl) Palla

###### Distribution

Wet pine savannas (WLPS), adjacent roadsides.

###### Notes

Infrequent. Mid-May–Jun; Jun–Sep. Thornhill 767 (NCSC). [< *Scirpus americanus* (Pers.) sensu RAB; = FNA, Weakley]

##### Scirpus cyperinus

(L.) Kunth

###### Distribution

Depressions in wet pine flatwoods (WPF-T), borrow pits, ditches.

###### Notes

Rare. (Jul–)Aug–Sep. Thornhill 543, 1061 (NCSC). Specimens seen in the vicinity: Sandy Run [Haw’s Run]: Taggart SARU 313 (WNC!); Sandy Run [Neck]: Wilbur 53662 (DUKE!). [= RAB, FNA, Weakley]

##### Scirpus lineatus

Michx.

###### Ecological interactions

####### Conservation status

State T; S2, G4.

###### Distribution

Pine savannas.

###### Notes

May–Jul. Not seen in Shaken Creek Preserve by the senior author. Specimens seen in the vicinity: Sandy Run: Taggart SARU 670 (WNC!). [= *Scirpus fontinalis* R.M. Harper sensu RAB; = FNA, Weakley]

##### Scleria baldwinii

(Torr.) Steud.

###### Ecological interactions

####### Conservation status

State T; S2, G4.

###### Distribution

Wet pine savannas (VWLPS).

###### Notes

Infrequent. Jun–Jul. Thornhill 433, 576, 1091 (NCSC). [= RAB, FNA, Weakley]

##### Scleria ciliata var. ciliata

Michx.

###### Distribution

Wet pine savannas (VWLPS), wet pine flatwoods (WPF-T).

###### Notes

Occasional. May–Aug. Thornhill 1138, 1318, 1514 (NCSC). [< *Scleria ciliata* Michx. sensu RAB; = FNA, Weakley]

##### Scleria ciliata var. glabra

(Chapm.) Fairey

###### Distribution

Wet pine flatwoods (WPF-T).

###### Notes

May–Aug. Reported from Shaken Creek Preserve by LeBlond (2000), but no specimens have been seen by the senior author. [< *Scleria ciliata* Michx. sensu RAB; = FNA, Weakley]

##### Scleria georgiana

Core

###### Ecological interactions

####### Conservation status

W1; S3, G4.

###### Distribution

Wet pine savannas (VWLPS).

###### Notes

Jun–Aug. Reported from Shaken Creek Preserve by LeBlond (2000), but no specimens have been seen by the senior author. Specimens seen in the vicinity: Sandy Run: Taggart SARU 315 (WNC!). [= RAB, FNA, Weakley]

##### Scleria minor

(Britton) W. Stone

###### Distribution

Wet pine savannas (SPS-T, SPS-RF, WLPS).

###### Notes

Infrequent. Jun–Aug. Thornhill 786, 1406, 1582 (NCSC). Specimens seen in the vicinity: Sandy Run [Hancock]: Taggart SARU 253 (WNC!); Sandy Run [Neck]: LeBlond 2058 (NCU!). [= RAB, FNA, Weakley]

##### Scleria muehlenbergii

Steud.

###### Distribution

Wet pine savannas (SPS-RF, WLPS, VWLPS).

###### Notes

Occasional. Jun–Sep. Thornhill 927, 939, 1107 (NCSC). Specimens seen in the vicinity: Sandy Run [Haw’s Run]: Taggart SARU 667 (WNC!). [< *Scleria reticularis* Michx. sensu RAB; = FNA, Weakley]

##### Scleria pauciflora var. caroliniana

(Willd.) Alph. Wood

###### Distribution

Wet pine flatwoods (WPF-T), wet pine savannas (WLPS).

###### Notes

Occasional. Jun–Sep. Thornhill 1413 (NCSC). Specimens seen in the vicinity: Sandy Run [Hancock]: Taggart SARU 370 (WNC!). [< *Scleria pauciflora* Muhl. ex Willd. sensu RAB; = FNA, Weakley]

##### Scleria species 1

###### Ecological interactions

####### Conservation status

SR-L, FSC; S1, G1.

###### Distribution

Wet pine savannas (VWLPS).

###### Notes

May–Sep. Not seen in Shaken Creek Preserve by the senior author. Specimens seen in the vicinity: Sandy Run: LeBlond 5722B (NCU!), Taggart SARU 316 (WNC!). [= Weakley]

##### Scleria triglomerata

Michx.

###### Distribution

Wet pine flatwoods (WPF-T).

###### Notes

Occasional. May–Sep. Thornhill 1321, 1322, 1323, 1360 (NCSC). Specimens seen in the vicinity: Sandy Run [Neck]: Taggart SARU 329 (WNC!). [< *Scleria triglomerata* Michx. sensu RAB, FNA; = Weakley]

##### Scleria verticillata

Muhl. ex Willd.

###### Ecological interactions

####### Conservation status

SR-P; S2, G5.

###### Distribution

Pine savannas.

###### Notes

Jul–Sep. Not seen in Shaken Creek Preserve by the senior author. Specimens seen in the vicinity: Sandy Run: LeBlond 2373 (NCU!). [= RAB, FNA, Weakley]

#### Dioscoreaceae

##### Dioscorea villosa

L.

###### Distribution

Swampy margins of wet pine savannas (VWLPS).

###### Notes

Rare. Apr–Jun; Sep–Nov. Thornhill 975 (NCSC). Specimens seen in the vicinity: Sandy Run [Patterson]: Taggart SARU 501 (WNC!). [> Dioscorea villosa L. var. villosa, Dioscorea villosa L. var. hirticaulis (Bartlett) H.E. Ahles sensu RAB; = FNA, Weakley]

#### Eriocaulaceae

##### Eriocaulon compressum

Lam.

###### Distribution

Pine savannas (SPS-RF).

###### Notes

Apr–Oct. Reported from Shaken Creek Preserve by LeBlond (2000), but no specimens have been seen by the senior author. [= RAB, FNA, Weakley]

##### Eriocaulon decangulare var. decangulare

L.

###### Distribution

Depressions in wet pine flatwoods (WPF-T) and pine savannas (SPS-T, SPS-RF, WLPS, VWLPS).

###### Notes

Frequent. Jun–Oct. Thornhill 437, 466, 499, 512, 634, 660, 662, 717, 802 (NCSC). Specimens seen in the vicinity: Sandy Run [Neck]: Wilbur 55306 (DUKE!; as *Eriocaulon decangulare*); Sandy Run [O’Berry]: Taggart SARU 165 (WNC!). [< *Eriocaulon decangulare* L. sensu RAB; = FNA, Weakley]

##### Lachnocaulon anceps

(Walter) Morong

###### Distribution

Depressions in wet pine flatwoods (WPF-T) and pine savannas (SPS-T, SPS-RF, WLPS, VWLPS).

###### Notes

Frequent. May–Oct. Thornhill 338, 438, 446, 452, 464, 498, 586 (NCSC). Specimens seen in the vicinity: Sandy Run [Hancock]: Taggart SARU 120 (WNC!); Sandy Run [Neck]: Wilbur 55298 (DUKE!); Sandy Run [Patterson]: Taggart SARU 217 (WNC!; as *Lachnocaulon beyrichianum*). [= RAB, FNA, Weakley]

##### Syngonanthus flavidulus

(Michx.) Ruhland

###### Ecological interactions

####### Conservation status

W1; S3, G5.

###### Distribution

Pine savannas, flatwoods, and adjacent ditches.

###### Notes

May–Oct. Not seen in Shaken Creek Preserve by the senior author. Specimens seen in the vicinity: Holly Shelter: LeGrand s.n. (NCU!). [= RAB, FNA, Weakley]

#### Haemodoraceae

##### Lachnanthes caroliniana

(Lam.) Dandy

###### Distribution

Depressions in wet pine flatwoods (WPF-T) and pine savannas (SPS-T, SPS-RF, WLPS, VWLPS), ditches.

###### Notes

Frequent. Jun–early Sep; Sep–Nov. Thornhill 370, 447, 493, 494, 495, 496 (NCSC). Specimens seen in the vicinity: Sandy Run [Hancock]: Wyland s.n. (NCSC!); Sandy Run [Neck]: Wilbur 53676 (DUKE!); Sandy Run [RMK]: Taggart SARU 221 (WNC!). [= RAB, FNA, Weakley]

#### Heloniadaceae

##### Chamaelirium luteum

(L.) A. Gray

###### Ecological interactions

####### Conservation status

W5B; S5, G5.

###### Distribution

Ecotone between mesic pine savanna (MPS-CP) and pond pine woodland.

###### Notes

Rare. Mar–May; Sep–Nov. Thornhill 1274 (NCSC). [= RAB, FNA, Weakley]

#### Hypoxidaceae

##### Hypoxis curtissii

Rose

###### Distribution

Wet pine savannas (SPS-RF, VWLPS).

###### Notes

Mar–Jun; May–Jul. Reported from Shaken Creek Preserve by LeBlond (2000), but no specimens have been seen by the senior author. [= Hypoxis hirsuta (L.) Coville var. leptocarpa (Engelm. & A. Gray) Brackett sensu RAB; = FNA, Weakley]

##### Hypoxis hirsuta

(L.) Coville

###### Distribution

Wet pine flatwoods (WPF-T), wet pine savannas (WLPS, VWLPS), adjacent roadsides.

###### Notes

Frequent. Mar–Jun; May–Jul. Thornhill 105, 140, 254 (NCSC). Specimens seen in the vicinity: Sandy Run [Patterson]: Taggart SARU 34 (WNC!). [= Hypoxis hirsuta (L.) Coville var. hirsuta sensu RAB; = FNA, Weakley]

##### Hypoxis sessilis

L.

###### Ecological interactions

####### Conservation status

SR-P; SH, G4.

###### Distribution

Wet pine savannas (SPS-T, WLPS, VWLPS).

###### Notes

Occasional. Apr(–later, especially in response to fire); May(–later, especially in response to fire). The specimens collected by the author lack seeds, which are the most accurate means of distinguishing this species from *Hypoxis wrightii*. However, the floral and vegetative features (see key above) of the specimens match the descriptions of *Hypoxis sessilis*, and the specimens themselves appear similar to *Hypoxis sessils* specimens examined at NCU (Britt 195, Leonard and Davis SWL–748, Radford 147, and Sorrie 12618). Thornhill 124, 176, 218, 295 (NCSC). [= RAB, FNA, Weakley]

##### Hypoxis wrightii

(Baker) Brackett

###### Distribution

Wet pine savannas (SPS-T, WLPS), adjacent roadsides.

###### Notes

Occasional. Mar–Apr(–later, especially in response tofire); Apr–May(–later, especially in response to fire). Thornhill 36, 157, 244 (NCSC). Specimens seen in the vicinity: Sandy Run [Haw’s Run]: Taggart SARU 255 (WNC!). [= *Hypoxis micrantha* Pollard sensu RAB; = FNA, Weakley]

#### Iridaceae

##### Iris tridentata

Pursh

###### Distribution

Wet pine flatwoods (WPF-T), wet pine savannas (SPS-T, SPS-RF, WLPS, VWLPS).

###### Notes

Occasional. Late May–Jun; Aug–Oct. Thornhill 317, 351, 380, 384, 1300 (NCSC). Specimens seen in the vicinity: Sandy Run [Hancock]: Taggart SARU 175 (WNC!); Sandy Run [Neck]: Levy s.n. (DUKE!), Wilbur 55316 (DUKE!). [= RAB, FNA, Weakley]

##### Iris verna var. verna

L.

###### Distribution

Wet pine flatwoods (WPF-T), wet pine savannas (SPS-T, SPS-RF, WLPS, VWLPS).

###### Notes

Frequent. Mar–May; May–Jun. Thornhill 76, 77, 89, 99, 100 (NCSC). Specimens seen in the vicinity: Sandy Run [Hancock]: Taggart SARU 45 (WNC!); Sandy Run [Neck]: Wilbur 60085 (DUKE!). [= RAB, FNA, Weakley]

##### Iris virginica var. virginica

L.

###### Distribution

Margins of wet pine savannas (WLPS, VWLPS) and adjacent swamps, borrow pits, ditches.

###### Notes

Occasional. Apr–May; Jul–Sep. Thornhill 241 (NCSC). Specimens seen in the vicinity: Sandy Run [Haw’s Run]: Taggart SARU 110 (WNC!). [< *Iris virginica* L. sensu RAB, FNA, Weakley]

##### Sisyrinchium albidum

Raf.

###### Distribution

Pine savannas.

###### Notes

Mar–Jun; May–Jun. Reported from Sandy Run [Neck] by LeBlond and Weakley (1991), but no specimens have been seen in Shaken Creek Preserve by the senior author. [< RAB; = FNA, Weakley]

##### Sisyrinchium angustifolium

Mill.

###### Distribution

Wet pine flatwoods (WPF-T), wet pine savannas (WLPS, VWLPS).

###### Notes

Occasional. Mar–Jun; May–Jul. Thornhill 195, 1401 (NCSC). [= RAB, FNA, Weakley]

##### Sisyrinchium arenicola

E.P. Bicknell

###### Distribution

Pine savannas and adjacent roadsides.

###### Notes

Mar–Jun; Jun–Aug.). Not seen in Shaken Creek Preserve by the senior author. Specimens seen in the vicinity: Sandy Run [Neck]: Wilbur 55244, 55281, 55323, 55324 (DUKE!). [< *Sisyrinchium fuscatum* E.P. Bicknell sensu FNA; = Weakley]

##### Sisyrinchium atlanticum

E.P. Bicknell

###### Distribution

Pine/scrub oak sandhills (PSOS-MT), wet pine flatwoods (WPF-T), wet pine savannas (WLPS, VWLPS).

###### Notes

Occasional. Mar–Jun; Jun–Aug. Thornhill 187, 196, 221, 372, 394 (NCSC). Specimens seen in the vicinity: Sandy Run [Patterson]: Taggart SARU 74 (WNC!). [= Sisyrinchium mucronatum Michx. var. atlanticum (E.P. Bicknell) H.E. Ahles sensu RAB; = FNA, Weakley]

##### Sisyrinchium capillare

E.P. Bicknell

###### Distribution

Wet pine flatwoods (WPF-T), wet pine savannas (WLPS, VWLPS).

###### Notes

Occasional. Mar–Jun; May–Jun. Thornhill 111, 127, 138, 191, 192, 194, 208 (NCSC). Specimens seen in the vicinity: Sandy Run [Hancock]: Taggart SARU 178 (WNC!). [< *Sassafras albidum* Raf. sensu RAB; = FNA, Weakley]

#### Juncaceae

##### Juncus acuminatus

Michx.

###### Distribution

Wet pine savannas (VWLPS).

###### Notes

May–Aug. Not seen in Shaken Creek Preserve by the senior author. Specimens seen in the vicinity: Sandy Run [Neck]: Wilbur 55304 (DUKE!). [= RAB, FNA, Weakley]

##### Juncus biflorus

Elliott

###### Distribution

Wet pine flatwoods (WPF-T), wet pine savannas (SPS-T, SPS-RF, WLPS, VWLPS), adjacent roadsides.

###### Notes

Frequent. Jun–Oct. Thornhill 455, 461, 627, 853, 1372 (NCSC). Specimens seen in the vicinity: Sandy Run [Haw’s Run]: Taggart SARU 318 (WNC!); Sandy Run [Patterson]: Taggart SARU 632 (WNC!; as *Juncus marginatus*). [= RAB; < *Juncus marginatus* Rostk. sensu FNA; = Weakley]

##### Juncus bufonius

L.

###### Distribution

Margins of wet pine savannas (SPS-T) and adjacent roadsides.

###### Notes

Occasional. Jun–Nov. Thornhill 297 (NCSC). Specimens seen in the vicinity: Sandy Run [Hancock]: Taggart SARU 300 (WNC!; as Juncus bufonius var. bufonius); Sandy Run [Neck]: Wilbur 55313 (DUKE!). [= RAB, FNA, Weakley]

##### Juncus canadensis

J. Gay ex Laharpe

###### Distribution

Depressions and borrow pits in wet pine savannas (SPS-T).

###### Notes

Rare. Jul–Oct. Thornhill 19 (NCSC). Specimens seen in the vicinity: Sandy Run [Hancock]: Taggart SARU 618 (WNC!). [= RAB, FNA, Weakley]

##### Juncus coriaceus

Mack.

###### Distribution

Wet pine savannas (SPS-RF), adjacent roadsides.

###### Notes

Infrequent. Jun–Sep. Thornhill 957 (NCSC). Specimens seen in the vicinity: Sandy Run [Haw’s Run]: Taggart SARU 610 (WNC!). [= RAB, FNA, Weakley]

##### Juncus dichotomus

Elliott

###### Distribution

Wet pine savannas (WLPS, VWLPS), adjacent roadsides.

###### Notes

Frequent. Jun–Oct. Thornhill 286, 339, 340, 453, 575 (NCSC). Specimens seen in the vicinity: Sandy Run [Hancock]: Taggart SARU 259 (WNC!); Sandy Run [Neck]: Wilbur 53663 (DUKE!). [> *Juncus dichotomus* Elliott, *Juncus platyphyllus* (Wiegand) Fernald sensu RAB; = FNA, Weakley]

##### Juncus diffusissimus

Buckley

###### Distribution

Wet pine savannas (VWLPS).

###### Notes

May–Sep. Not seen in Shaken Creek Preserve by the senior author. Specimens seen in the vicinity: Sandy Run [Neck]: Wilbur 55286 (DUKE!). [= RAB, FNA, Weakley]

##### Juncus effusus solutus

(Fernald & Wiegand) Hämet-Ahti

###### Distribution

Disturbed areas in wet pine flatwoods (WPF-T), adjacent roadsides.

###### Notes

Rare. Jun–Sep. Thornhill 1339 (NCSC). Specimens seen in the vicinity: Sandy Run [Hancock]: Taggart SARU 257 (WNC!). [< *Juncus effusus* L. sensu RAB, FNA; = Weakley]

##### Juncus elliottii

Chapm.

###### Distribution

Margins of wet pine savannas (WLPS) and adjacent roadsides.

###### Notes

Occasional. May–Sep. Thornhill 333, 336 (NCSC). [= RAB, FNA, Weakley]

##### Juncus marginatus

Rostk.

###### Distribution

Depressions in wet pine savannas (WLPS).

###### Notes

Infrequent. Jun–Sep. Thornhill 1374 (NCSC). Specimens seen in the vicinity: Sandy Run [Neck]: Wilbur 53693, 57628 (DUKE!). [= RAB; < *Juncus marginatus* Rostk. sensu FNA; = Weakley]

##### Juncus megacephalus

M.A. Curtis

###### Distribution

Wet pine savannas (VWLPS), adjacent roadsides.

###### Notes

Occasional. Jun–Aug. Thornhill 405, 519, 574, 732 (NCSC). Specimens seen in the vicinity: Sandy Run [Hancock]: Taggart SARU 298 (WNC!); Sandy Run [Neck]: Wilbur 53666, 55284 (DUKE!). [= RAB, FNA, Weakley]

##### Juncus pelocarpus

E. Mey.

###### Distribution

Depressions in wet pine savannas (VWLPS), borrow pits.

###### Notes

Infrequent. Jul–Oct. Thornhill 854, 933, 1125, 1191 (NCSC). Specimens seen in the vicinity: Sandy Run [Neck]: Wilbur 57693 (DUKE!; as *Juncus abortivus*). [> *Juncus abortivus* Chapm. sensu RAB; = FNA, Weakley]

##### Juncus polycephalos

Michx.

###### Distribution

Wet pine savannas (WLPS, VWLPS).

###### Notes

Frequent. Jul–Sep. Thornhill 247, 344, 780 (NCSC). Specimens seen in the vicinity: Sandy Run [Haw’s Run]: Taggart SARU 167 (WNC!); Sandy Run [Neck]: Wilbur 53714 (DUKE!). [= *Juncus polycephalus* Michx. sensu RAB, FNA; = Weakley]

##### Juncus repens

Michx.

###### Distribution

Depressions in wet pine savannas (SPS-RF), borrow pits.

###### Notes

Infrequent. Jun–Oct. Thornhill 410, 713 (NCSC). Specimens seen in the vicinity: Sandy Run [Hancock]: Taggart SARU 229 (WNC!). [= RAB, FNA, Weakley]

##### Juncus scirpoides var. compositus

R.M. Harper

###### Distribution

Wet pine savannas (SPS-T, SPS-RF), adjacent roadsides.

###### Notes

Infrequent. Jun–Oct. Thornhill 934 (NCSC). [< *Juncus scirpoides* Lam. sensu RAB, FNA; = Weakley]

##### Juncus scirpoides var. scirpoides

Lam.

###### Distribution

Wet pine flatwoods (WPF-T), wet pine savannas (SPS-T, WLPS), adjacent roadsides.

###### Notes

Occasional. Jun–Oct. Thornhill 781 (NCSC). Specimens seen in the vicinity: Sandy Run [Hancock]: Taggart SARU 633 (WNC!). [< *Juncus scirpoides* Lam. sensu RAB, FNA; = Weakley]

##### Juncus trigonocarpus

Steud.

###### Distribution

Borrow pits in pine savannas, ditches.

###### Notes

Infrequent. Jul–Oct. Thornhill 968 (NCSC). [= RAB, FNA, Weakley]

##### Juncus validus var. validus

Coville

###### Distribution

Disturbed, wet areas in wet pine flatwoods (WPF-T), roadsides, ditches.

###### Notes

Occasional. Jul–Sep. Thornhill 465, 856 (NCSC). Specimens seen in the vicinity: Sandy Run [Neck]: Wilbur 57630 (DUKE!). [< *Juncus validus* Covile sensu RAB; = FNA, Weakley]

#### Liliaceae

##### Lilium catesbaei

Walter

###### Distribution

Wet pine flatwoods (WPF-T), wet pine savannas (SPS-T, SPS-RF, WLPS, VWLPS).

###### Notes

Occasional. Mid Jun–mid Sep; Sep–Nov. Thornhill 664, 673 (NCSC). Specimens seen in the vicinity: Sandy Run [Neck]: Taggart SARU 463 (WNC!). [> Lilium catesbaei L. var. catesbaei, Lilium catesbaei var. longii Fernald sensu RAB; = FNA, Weakley]

#### Melanthiaceae

##### Amianthium muscitoxicum

(Walter) A. Gray

###### Distribution

Wet pine savannas (WLPS).

###### Notes

Rare. May–Jul; Jul–Sep. Thornhill 1400 (NCSC). [= *Amianthium muscaetoxicum* (Walter) A. Gray sensu RAB; = FNA, Weakley]

##### Stenanthium densum

(Desr.) Zomlefer & Judd

###### Distribution

Wet pine savannas (SPS-T, SPS-RF, WLPS, VWLPS).

###### Notes

Frequent. Apr–early Jun; late May–Jul. Thornhill 275, 354, 487, 492 (NCSC). Specimens seen in the vicinity: Sandy Run [Hancock]: Taggart SARU 174 (WNC!); Sandy Run [Neck]: Levy s.n. (DUKE!; as *Amianthium muscitoxicum*), Wilbur 55317 (DUKE!; as *Zigadenus densus*). [= *Zigadenus densus* (Desr.) Fernald sensu RAB, FNA; = Weakley]

##### Veratrum virginicum

(L.) W.T. Aiton

###### Distribution

Wet pine savannas (WLPS, VWLPS).

###### Notes

Infrequent. Jun–Aug; Aug–Oct. Thornhill 1010 (NCSC). Specimens seen in the vicinity: Sandy Run [Neck]: Taggart SARU 646 (WNC!); Sandy Run [Neck]: Wilbur 57658 (DUKE!). [= *Melanthium virginicum* L. sensu RAB, FNA; = Weakley]

##### Zigadenus glaberrimus

Michx.

###### Distribution

Wet pine flatwoods (WPF-T), wet pine savannas (SPS-T, SPS-RF, WLPS).

###### Notes

Frequent. Late Jun–early Sep; Aug–Nov. Thornhill 678, 714, 797, 915 (NCSC). Specimens seen in the vicinity: Highway 50: Wilbur 9426 (DUKE!); Sandy Run [Neck]: Taggart SARU 365 (WNC!), Wilbur 53706, 57661 (DUKE!). [= RAB, FNA, Weakley]

#### Nartheciaceae

##### Aletris aurea

Walter

###### Distribution

Wet pine savannas (WLPS, VWLPS).

###### Notes

Occasional. Mid May–Jul; Aug. Thornhill 426, 535, 536 (NCSC). Specimens seen in the vicinity: Sandy Run [Haw’s Run]: Taggart SARU 309 (WNC!); Sandy Run [Neck] Wilbur 53674 (DUKE!). [= RAB, FNA, Weakley]

##### Aletris farinosa

L.

###### Ecological interactions

####### Conservation status

W5B; S5, G5.

###### Distribution

Wet pine flatwoods (WPF-T), wet pine savannas (SPS-T, SPS-RF, WLPS, VWLPS).

###### Notes

Frequent. Late Apr–early Jun; Jul–Aug. Thornhill 248, 274, 291, 324, 382, 385 (NCSC). Specimens seen in the vicinity: Sandy Run: Taggart SARU 121 (WNC!), Wilbur 55294 (DUKE!). [= RAB, FNA, Weakley]

##### Aletris lutea

Small

###### Distribution

Wet pine savannas (VWLPS).

###### Notes

Rare. Apr–mid Jun; May–Jul. Thornhill 556 (NCSC). [= FNA, Weakley]

#### Orchidaceae

##### Calopogon barbatus

(Walter) Ames

###### Distribution

Wet pine savannas (SPS-T, SPS-RF, WLPS, VWLPS).

###### Notes

Occasional. Apr–early May. Thornhill 162, 188, 189, 193, 206 (NCSC). Specimens seen in the vicinity: Sandy Run [Patterson]: Taggart SARU 105 (WNC!). [= RAB, FNA, Weakley]

##### Calopogon pallidus

Chapm.

###### Distribution

Wet pine savannas (SPS-T, SPS-RF, WLPS, VWLPS).

###### Notes

Occasional. May–Jul. Thornhill 322, 399, 408 (NCSC). Specimens seen in the vicinity: Sandy Run [Hancock]: Taggart SARU 179 (WNC!). [= RAB, FNA, Weakley]

##### Calopogon tuberosus var. tuberosus

(L.) Britton, Sterns & Poggenb.

###### Distribution

Wet pine savannas (SPS-RF, WLPS, VWLPS).

###### Notes

Infrequent. Apr–Jul. Thornhill 421 (NCSC). Specimens seen in the vicinity: Sandy Run [Hancock]: Taggart SARU 187 (WNC!). [= *Calopogon pulchellus* R. Br. sensu RAB; = FNA, Weakley]

##### Cleistesiopsis divaricata

(L.) Pansarin & F. Barros

###### Ecological interactions

####### Conservation status

W1; S3, G4.

###### Distribution

Wet pine flatwoods (WPF-T), wet pine savannas (SPS-T, SPS-RF, WLPS, VWLPS).

###### Notes

Infrequent. May–mid Jun. Thornhill 386 (NCSC). Specimens seen in the vicinity: Sandy Run: Taggart SARU 587 (WNC!; as *Cleistes divaricata*). [< *Cleistes divaricata* (L.) Ames sensu RAB, FNA; = Weakley]

##### Cleistesiopsis oricamporum

P.M. Br.

###### Ecological interactions

####### Conservation status

W7; S2, G3?.

###### Distribution

Wet pine flatwoods (WPF-T).

###### Notes

May–Jul. Reported from Shaken Creek Preserve by LeBlond (2000; as *Cleistes bifaria* (Fernald) Catling & Gregg), but no specimens have been seen by the senior author. [< *Cleistes divaricata* (L.) Ames sensu RAB; < *Cleistes bifaria* (Fernald) Catling & Gregg sensu FNA; = Weakley]

##### Platanthera blephariglottis

(Willd.) Lindl.

###### Ecological interactions

####### Conservation status

W1; S3?, G4G5.

###### Distribution

Wet pine savannas (SPS-RF).

###### Notes

Rare. Jul–Sep. Thornhill 1521 (NCSC). Specimens seen in the vicinity: Highway 50: Wilbur 9425 (DUKE!); Sandy Run: Taggart SARU 424 (WNC!). [< Habenaria blephariglottis (Willd.) Hook. var. blephariglottis sensu RAB; = Platanthera blephariglottis (Willd.) Lindl. var. blephariglottis sensu FNA; = Weakley]

##### Platanthera ciliaris

(L.) Lindl.

###### Distribution

Wet pine savannas (SPS-RF).

###### Notes

Jul–Sep. Reported from Shaken Creek Preserve by LeBlond (2000), but no specimens have been seen by the senior author. Specimens seen in the vicinity: Sandy Run [Neck]: Taggart SARU 441 (WNC!). [= *Habenaria ciliaris* (L.) R. Br. sensu RAB; = FNA, Weakley]

##### Platanthera cristata

(Michx.) Lindl.

###### Distribution

Wet pine savannas (SPS-RF).

###### Notes

Rare. Jul–Sep. Thornhill 1448 (NCSC). Specimens seen in the vicinity: Highway 50: Wilbur 9438 (DUKE!; as *Habenaria cristata*); Sandy Run [Hancock]: Taggart SARU 360 (WNC!). [= *Habenaria cristata* (Michx.) R. Br. sensu RAB; = FNA, Weakley]

##### Platanthera integra

(Nutt.) A. Gray ex L.C. Beck

###### Ecological interactions

####### Conservation status

SC-V; S2, G3G4.

###### Distribution

Wet pine savannas (SPS-RF).

###### Notes

Jul–Sep. Reported from Shaken Creek Preserve by LeBlond (2000), but no specimens have been seen by the senior author. Specimens seen in the vicinity: Highway 50: Wilbur 9428 (DUKE!; as *Habenaria integra*). [= *Habenaria integra* (Nutt.) Spreng. sensu RAB; = FNA, Weakley]

##### Platanthera nivea

(Nutt.) Luer

###### Ecological interactions

####### Conservation status

State T; S1, G5.

###### Distribution

Pine savannas.

###### Notes

May–Sep. Reported from Sandy Run by LeBlond and Weakley (1991), but no specimens have been seen in Shaken Creek Preserve by the senior author. [= *Habenaria nivea* (Nutt.) Spreng. sensu RAB; = FNA, Weakley]

##### Pogonia ophioglossoides

(L.) Ker Gawl.

###### Distribution

Wet pine savannas (SPS-RF, VWLPS).

###### Notes

Rare. Mar–Jun. Thornhill 1286 (NCSC). Specimens seen in the vicinity: Sandy Run [Neck]: Taggart SARU 190 (WNC!). [= RAB, FNA, Weakley]

##### Spiranthes cernua

(L.) Rich.

###### Distribution

Wet pine flatwoods (WPF-T), wet pine savannas (VWLPS).

###### Notes

Occasional. Jul–Nov. Thornhill 489, 1173, 1356 (NCSC). Specimens seen in the vicinity: Sandy Run [Haw’s Run]: Taggart SARU 543 (WNC!). [= Spiranthes cernua (L.) Rich. var. cernua sensu RAB; = FNA, Weakley]

##### Spiranthes eatonii

Ames ex P.M. Br.

###### Ecological interactions

####### Conservation status

State E; S2, G2G4.

###### Distribution

Pine savannas (WLPS, VWLPS).

###### Notes

Infrequent. Feb–May. Thornhill 483, 1303, 1377 (NCSC). [= FNA, Weakley]

##### Spiranthes lacera var. gracilis

(Bigelow) Luer

###### Distribution

Pine savannas.

###### Notes

Aug–Sep. Not seen in Shaken Creek Preserve by the senior author. Specimens seen in the vicinity: Sandy Run [Hancock]: Taggart SARU 285 (WNC!). [= Spiranthes gracilis (Bigelow) Beck var. gracilis sensu RAB; = FNA, Weakley]

##### Spiranthes laciniata

(Small) Ames

###### Ecological interactions

####### Conservation status

SC-V; S2, G4G5.

###### Distribution

Pine savannas (WLPS).

###### Notes

Infrequent. May–Aug. Thornhill 390, 477 (NCSC). Specimens seen in the vicinity: Sandy Run [Hancock]: Taggart SARU 177 (WNC!). [= RAB, FNA, Weakley]

##### Spiranthes longilabris

Lindl.

###### Ecological interactions

####### Conservation status

State E; S1, G3.

###### Distribution

Pine savannas.

###### Notes

Late Oct–Dec. Reported within a two-mile radius of Shaken Creek Preserve by the North Carolina Natural Heritage Program (http://www.ncnhp.org) (EO status “current”, accuracy “medium”), but no specimens have been seen in Shaken Creek Preserve by the senior author. [= RAB, FNA, Weakley]

##### Spiranthes praecox

(Walter) S. Watson

###### Distribution

Pine savannas (SPS-T, VWLPS).

###### Notes

Rare. Mar–Jul. Thornhill 1301 (NCSC). Specimens seen in the vicinity: Sandy Run [Neck]: Taggart SARU 589 (WNC!). [< *Spiranthes praecox* (Walter) S. Watson sensu RAB, FNA; = Weakley]

##### Spiranthes vernalis

Englem. & A. Gray

###### Distribution

Pine savannas (WLPS) and adjacent roadsides.

###### Notes

Infrequent. Mar–Jul. Thornhill 1556 (NCSC). [= RAB, FNA, Weakley]

#### Poaceae

##### Agrostis altissima

(Walter) Tuck.

###### Ecological interactions

####### Conservation status

SR-T; S2, G4.

###### Distribution

Wet pine savannas (WLPS, VWLPS).

###### Notes

Occasional. Oct–Nov. Thornhill 1060, 1076, 1132, 1164, 1192 (NCSC). Specimens seen in the vicinity: Sandy Run: LeBlond 2595, 4655, 4672 (NCU!), Taggart SARU 550 (WNC!). [< *Agrostis perennans* (Walter) Tuck. sensu RAB, FNA; = Weakley]

##### Agrostis hyemalis

(Walter) Britton, Sterns, & Poggenb.

###### Distribution

Wet pine savannas (SPS-T, WLPS), adjacent roadsides.

###### Notes

Occasional. Mar–Jul. Thornhill 226, 287, 299, 332, 406, 413 (NCSC). Specimens seen in the vicinity: Old Maple Hilll Road: Wilbur 55268 (DUKE!). [< *Agrostis hyemalis* (Walter) Britton, Stern, & Poggenb. sensu RAB; = FNA, Weakley]

##### Agrostis perennans

(Walter) Tuck.

###### Distribution

Wet pine savannas (WLPS), adjacent roadsides.

###### Notes

Infrequent. Aug–Oct. Thornhill 1021 (NCSC). [< *Agrostis perennans* (Walter) Tuck. sensu RAB, FNA; = Weakley]

##### Amphicarpum amphicarpon

(Pursh) Nash

###### Ecological interactions

####### Conservation status

W1; S3, G4.

###### Distribution

Wet pine savannas (SPS-T, SPS-RF), adjacent roadsides.

###### Notes

Occasional. Aug–Oct. Thornhill 23, 821 (NCSC). Specimens seen in the vicinity: Sandy Run: Taggart SARU 538 (NCU!). [= *Amphicarpum purshii* Kunth sensu RAB; = FNA, Weakley]

##### Andropogon dealbatus

(C. Mohr ex Hack.) Weakley & LeBlond

###### Distribution

Wet pine savannas (SPS-T, VWLPS).

###### Notes

Occasional. Sep–Oct. Thornhill 1113, 1118, 1196 (NCSC). Specimens seen in the vicinity: Sandy Run [Haw’s Run]: Taggart SARU 647 (as *Andropogon capillipes* var. 2), Taggart SARU 662 (WNC!). [< *Andropogon virginicus* L. sensu RAB; < Andropogon virginicus L. var. glaucus Hack. sensu FNA; = Weakley]

##### Andropogon gerardii

Vitman

###### Distribution

Wet pine savannas (WLPS, VWLPS).

###### Notes

Occasional. Jul–Oct. Thornhill 690, 848, 1040, 1098 (NCSC). Specimens seen in the vicinity: Sandy Run [Hancock]: Taggart SARU 408 (WNC!), Wilbur 8391 (DUKE!). [= RAB, FNA, Weakley]

##### Andropogon glaucopsis

Steud.

###### Distribution

Wet pine flatwoods (WPF-T), wet pine savannas (SPS-T, SPS-RF).

###### Notes

Occasional. Sep–Oct. Thornhill 21, 1119, 1160 (NCSC). Specimens seen in the vicinity: Sandy Run [Hancock]: Taggart SARU 489 (WNC!). [< *Andropogon virginicus* L. sensu RAB; = Andropogon glomeratus var. glaucopsis (Elliott) C. Mohr sensu FNA; = Weakley]

##### Andropogon glomeratus

(Walter) Britton, Sterns, & Poggenb.

###### Distribution

Wet pine flatwoods (WPF-T), wet pine savannas (SPS-T, SPS-RF, WLPS, VWLPS).

###### Notes

Frequent. Sep–Oct. Thornhill 1064, 1151, 1157, 1162, 1218, 1219, 1241, 1243, 1244 (NCSC). Specimens seen in the vicinity: Sandy Run [Hancock]: Taggart SARU 541 (WNC!). [< *Andropogon virginicus* L. sensu RAB; = Andropogon glomeratus (Walter) Britton, Sterns, & Poggenb. var. glomeratus sensu FNA; = Weakley]

##### Andropogon hirsutior

(Hack.) Weakley & LeBlond

###### Distribution

Wet pine flatwoods (WPF-T), wet pine savannas (SPS-RF, WLPS, VWLPS).

###### Notes

Occasional. Sep–Oct. Thornhill 1065, 1154, 1165, 1200 (NCSC). [< *Andropogon virginicus* L. sensu RAB; = Andropogon hirsutior (Walter) Britton, Sterns, & Poggenb. var. hirsutior (Hack.) C. Mohr sensu FNA; = Weakley]

##### Andropogon mohrii

(Hack.) Hack. ex Vasey

###### Ecological interactions

####### Conservation status

State T; S2, G4?.

###### Distribution

Pine savannas.

###### Notes

Sep–Oct. Not seen in Shaken Creek Preserve by the senior author. Specimens seen in the vicinity: Sandy Run: Taggart SARU 539 (WNC!). [= RAB; = Andropogon liebmannii Hack. var. pungensis (Ashe) C.S. Campb. sensu FNA; = Weakley]

##### Andropogon perangustatus

Nash

###### Ecological interactions

####### Conservation status

W1; S2S3, G4.

###### Distribution

Wet pine savannas (WLPS).

###### Notes

Infrequent. Sep–Oct. Thornhill 1051, 1246 (NCSC). [= Andropogon gyrans Ashe var. stenophyllus (Hack.) C.S. Campb. sensu FNA; = Weakley]

##### Andropogon tenuispatheus

(Nash) Nash

###### Distribution

Wet pine savannas (WLPS).

###### Notes

Infrequent. Sep–Oct. Thornhill 1193, 1247 (NCSC). Specimens seen in the vicinity: Sandy Run [Neck]: Wilbur 57638 (DUKE!; as Andropogon glomeratus var. pumilus). [< *Andropogon virginicus* L. sensu RAB; = Andropogon glomeratus (Walter) Britton, Sterns, & Poggenb. var. pumilus (Vasey) Vasey ex L.H. Dewey sensu FNA; = Weakley]

##### Andropogon virginicus var. decipiens

C.S. Campb.

###### Ecological interactions

####### Conservation status

W7; S1S2, G5T4.

###### Distribution

Wet pine savannas (SPS-T, VWLPS), adjacent roadsides.

###### Notes

Occasional. Sep–Oct. Thornhill 20, 1112, 1194, 1195, 1217 (NCSC). [< *Andropogon virginicus* L. sensu RAB; = FNA, Weakley]

##### Andropogon virginicus var. virginicus

L.

###### Distribution

Wet pine savannas (SPS-T, WLPS), adjacent roadsides.

###### Notes

Occasional. Sep–Oct. Thornhill 808, 1216, 1245 (NCSC). Specimens seen in the vicinity: Sandy Run [Hancock]: Taggart SARU 3 (WNC!). [< *Andropogon virginicus* L. sensu RAB; = FNA, Weakley]

##### Anthenantia rufa

(Elliott) Schult.

###### Ecological interactions

####### Conservation status

W1; S2, G5.

###### Distribution

Wet pine flatwoods (WPF-T), wet pine savannas (SPS-T, SPS-RF, WLPS, VWLPS), and adjacent roadsides.

###### Notes

Frequent. Sep–Oct. Thornhill 53, 687, 812, 857, 878, 981 (NCSC). Specimens seen in the vicinity: Sandy Run: Taggart SARU 433 (WNC!). [= *Anthaenantia rufa* (Elliott) Schult. sensu RAB; = FNA, Weakley]

##### Aristida palustris

(Chapm.) Vasey

###### Distribution

Wet pine savannas (WLPS, VWLPS).

###### Notes

Occasional. Aug–Oct. Thornhill 631, 776, 788, 1059 (NCSC). Specimens seen in the vicinity: Sandy Run [Neck]: LeBlond 2586 (NCU!). [= *Aristida affinis* (Schult.) Kunth sensu RAB; = FNA, Weakley]

##### Aristida simpliciflora

Chapm.

###### Ecological interactions

####### Conservation status

State E; S1S2, G3G4.

###### Distribution

Wet pine savannas (WLPS).

###### Notes

Rare. Aug–Oct. Reported from Shaken Creek Preserve by LeBlond (2000), but no specimens have been seen on site by the senior author. Specimens seen in the vicinity: Sandy Run: LeBlond 5277 (NCU!). [= FNA, Weakley]

##### Aristida stricta

Michx.

###### Distribution

Pine/scrub oak sandhills (PSOS-MT), mesic pine savannas (MPS-CP), wet pine flatwoods (WPF-T), wet pine savannas (SPS-T, SPS-RF, WLPS, VWLPS).

###### Notes

Abundant. Sep–Nov. Thornhill 653, 820, 1020, 1072 (NCSC). Specimens seen in the vicinity: Highway 50: Wilbur 9429 (DUKE!); Sandy Run [Hancock]: Taggart SARU 1 (WNC!). [< *Aristida stricta* Michx. sensu RAB, FNA; = Weakley]

##### Aristida virgata

Trin.

###### Distribution

Wet pine flatwoods (WPF-T), wet pine savannas (SPS-T, SPS-RF, WLPS, VWLPS).

###### Notes

Frequent. Aug–Oct. Thornhill 641, 787, 858, 912, 913, 964 (NCSC). Specimens seen in the vicinity: Sandy Run [Hancock]: Taggart SARU 451 (WNC!); Sandy Run [Neck]: Taggart SARU 551 (WNC!; as *Aristida simpliciflora*). [= RAB; = Aristida purpurascens Poir. var. virgata (Trin.) Allred sensu FNA; = Weakley]

##### Arundinaria gigantea

(Walter) Muhl.

###### Distribution

Wet pine flatwoods.

###### Notes

Apr–Jul. AI199Not seen in Shaken Creek Preserve (in the relevant habitats) by the senior author. Specimens seen in the vicinity: Sandy Run [Patterson]: Taggart SARU 564 (WNC!). [< RAB; = FNA, Weakley]

##### Arundinaria tecta

(Walter) Muhl.

###### Distribution

Wet pine flatwoods (WPF-T), wet pine savannas (SPS-T, SPS-RF, WLPS, VWLPS).

###### Notes

Frequent. Apr–Jul. Thornhill 916, 917, 1281 (NCSC). Specimens seen in the vicinity: Sandy Run [O’Berry]: Taggart SARU 161 (WNC!). [< *Arundinaria gigantea* (Walter) Muhl. sensu RAB; = FNA, Weakley]

##### Calamagrostis coarctata

Eaton

###### Distribution

Wet pine savannas (VWLPS), adjacent roadsides.

###### Notes

Occasional. Jul–Oct. Thornhill 865, 973, 1033, 1094, 1242 (NCSC). Specimens seen in the vicinity: Sandy Run [Hancock]: Taggart SARU 432 (WNC!; as *Calamagrostis cinnoides*); Sandy Run [Neck]: LeBlond 1937 (NCU!; as *Calamagrostis cinnoides*). [= *Calamagrostis cinnoides* (Muhl.) W.P.C. Barton sensu RAB, FNA; = Weakley]

##### Calamovilfa brevipilis

(Torr.) Hack. ex Scribn. & Southw.

###### Ecological interactions

####### Conservation status

W1; S3, G4.

###### Distribution

Wet pine savannas (SPS-RF).

###### Notes

Infrequent. Jun–Oct. Thornhill 640, 648, 963, 1063 (NCSC). Specimens seen in the vicinity: Highway 50: Wilbur 9424 (DUKE!). [= RAB, FNA, Weakley]

##### Chasmanthium laxum

(L.) Yates

###### Distribution

Wet pine savannas (SPS-RF, WLPS, VWLPS), adjacent roadsides.

###### Notes

Occasional. Jun–Oct. Thornhill 647, 738, 774, 1198 (NCSC). Specimens seen in the vicinity: Sandy Run [Hancock]: Taggart SARU 273 (WNC!). [= *Uniola laxa* (L.) Britton, Sterns, & Poggenb. sensu RAB; = FNA, Weakley]

##### Coelorachis rugosa

(Nutt.) Nash

###### Ecological interactions

####### Conservation status

W1; S3, G5.

###### Distribution

Wet pine savannas (SPS-T, WLPS, VWLPS), adjacent roadsides.

###### Notes

Occasional. Jun–Oct. Thornhill 813, 877 (NCSC). Specimens seen in the vicinity: Sandy Run: Taggart SARU 490 (WNC!), Wilbur 57655 (DUKE!; as *Manisuris rugosa*). [= *Manisuris rugosa* (Nutt.) Kuntze sensu RAB; = FNA, Weakley]

##### Coleataenia anceps anceps

(Michx.) Soreng

###### Distribution

Wet pine savannas (SPS-RF, VWLPS), adjacent roadsides.

###### Notes

Occasional. Jun–Oct. Thornhill 962, 1108, 1474 (NCSC). Specimens seen in the vicinity: Sandy Run [Hancock]: Taggart SARU 435 (WNC!; as Panicum anceps Michx. var. anceps). [= Panicum anceps Michx. var. anceps sensu RAB; = Panicum anceps Michx. ssp. anceps sensu FNA; = Weakley]

##### Coleataenia anceps rhizomata

(Hitchc. & Chase) Soreng

###### Distribution

Wet pine savannas (WLPS, VWLPS), adjacent roadsides.

###### Notes

Occasional. Jun–Oct. Thornhill 782, 1054, 1110 (NCSC). [= Panicum anceps Michx. var. rhizomatum (Hitchc. & Chase) Fernald sensu RAB; = Panicum anceps Michx. ssp. rhizomatum (Hitchc. & Chase) Freckmann & Lelong sensu FNA; = Weakley]

##### Coleataenia longifolia combsii

(Scribn. & C.R. Ball) Soreng

###### Distribution

Wet pine savannas (WLPS, VWLPS), adjacent roadsides.

###### Notes

Occasional. Jul–Oct. Thornhill 980, 1107, 1127, 1152 (NCSC). [= Panicum longifolium Torr. var. combsii (Scribn. & C.R. Ball) Fernald sensu RAB; = Panicum rigidulum Bosc ex Nees ssp. combsii (Scribn. & C.R. Ball) Freckmann & Lelong sensu FNA; = Weakley]

##### Coleataenia longifolia longifolia

(Torr.) Soreng

###### Distribution

Wet pine flatwoods (WPF-T), wet pine savannas (SPS-T, SPS-RF, VWLPS), adjacent roadsides.

###### Notes

Frequent. Jul–Oct. Thornhill 13, 26, 936, 1126, 1159, 1221 (NCSC). Specimens seen in the vicinity: Sandy Run [Hancock]: Taggart SARU 540 (WNC!; as Panicum longifolium var. longifolium). [= Panicum longifolium Torr. var. longifolium sensu RAB; = Panicum rigidulum Bosc ex Nees ssp. pubescens (Vasey) Freckmann & Lelong sensu FNA; = Weakley]

##### Ctenium aromaticum

(Walter) Alph. Wood

###### Distribution

Wet pine savannas (SPS-T, SPS-RF, WLPS, VWLPS).

###### Notes

Abundant. Jun–Aug(–later in response to fire). Thornhill 318, 449, 539, 649, 877 (NCSC). Specimens seen in the vicinity: Highway 50: Wilbur 9430 (DUKE!); Sandy Run [Hancock]: Ahles 58375 (NCU!), Taggart SARU 242 (WNC!); Sandy Run [Neck]: Levy s.n. (DUKE!), Wilbur 53694 (DUKE!). [= RAB, FNA, Weakley]

##### Danthonia sericea

Nutt.

###### Distribution

Mesic pine savannas (MPS-CP).

###### Notes

Infrequent. Apr–Jun. Thornhill 1288 (NCSC). Specimens seen in the vicinity: Old Maple Hill Road: Wilbur 67108 (DUKE!). [=Danthonia sericea Nutt. var. sericea sensu RAB; < FNA; = Weakley]

##### Dichanthelium acuminatum var. acuminatum

(Sw.) Gould & C.A. Clark

###### Distribution

Pine savannas.

###### Notes

May–Oct. Not seen in Shaken Creek Preserve by the senior author. Specimens seen in the vicinity: Sandy Run [Hancock]: Taggart SARU 653 (WNC!). [< *Panicum lanuginosum* Elliott sensu RAB; = Dichanthelium acuminatum (Sw.) Gould & C. A. Clark var. acuminatum sensu FNA; = Weakley]

##### Dichanthelium acuminatum var. fasciculatum

(Torr.) Freckmann

###### Distribution

Wet pine savannas (SPS-T, WLPS, VWLPS).

###### Notes

Infrequent. May–Aug. Thornhill 272 (NCSC). Specimens seen in the vicinity: Sandy Run [Neck]: Wilbur 55307 (DUKE!; as *Panicum lanuginosum*). [< *Panicum lanuginosum* Elliott sensu RAB; = Dichanthelium acuminatum (Sw.) Gould & C. A. Clark ssp. fasciculatum (Torr.) Freckmann & Lelong sensu FNA; = Weakley]

##### Dichanthelium acuminatum var. lindheimeri

(Nash) Gould & C.A. Clark

###### Distribution

Borrow pits within and roadsides adjacent to wet pine savannas.

###### Notes

May–Sep. Reported from Shaken Creek Preserve by LeBlond (2000), but no specimens have been seen on site by the senior author. [< *Panicum lanuginosum* Elliott sensu RAB; = Dichanthelium acuminatum (Sw.) Gould & C. A. Clark ssp. lindheimeri (Nash) Freckmann & Lelong sensu FNA; = Weakley]

##### Dichanthelium caerulescens

(Hack. ex Hitchc.) Correll

###### Ecological interactions

####### Conservation status

State E; S1S2, G2G3.

###### Distribution

Wet pine savannas (WLPS, VWLPS).

###### Notes

Rare. Jun–Oct. LeBlond 4851 (NCU); Thornhill 1308 (NCSC). [< *Panicum dichotomum* L. sensu RAB; < Dichanthelium dichotomum (L.) Gould ssp. roanokense (Ashe) Freckmann & Lelong sensu FNA; = Weakley]

##### Dichanthelium chamaelonche chamaelonche

(Trin.) Freckmann & Lelong

###### Distribution

Wet pine flatwoods (WPF-T).

###### Notes

Infrequent. Apr–Sep. Thornhill 1297 (NCSC). [< *Panicum chamaelonche* Trin. sensu RAB; < Dichanthelium chamaelonche (Trin.) Freckmann & Lelong ssp. chamaelonche sensu FNA; = Weakley]

##### Dichanthelium commutatum commutatum

(Schult.) Gould

###### Distribution

Margins of wet pine savannas (VWLPS) and adjacent swamps.

###### Notes

Rare. May–Oct. Thornhill 1494 (NCSC). [< *Panicum commutatum* Schult. sensu RAB; > Dichanthelium commutatum (Schult.) Gould ssp. commutatum, Dichanthelium commutatum (Schult.) Gould ssp. equilaterale (Scribn.) Freckmann & Lelong sensu FNA; = Weakley]

##### Dichanthelium consanguineum

(Kunth) Gould & C.A. Clark

###### Distribution

Wet pine savannas (SPS-RF, WLPS, VWLPS).

###### Notes

Occasional. Apr–Sep. Thornhill 246, 285, 296, 1295 (NCSC). [< *Panicum consanguineum* Kunth sensu RAB; = FNA, Weakley]

##### Dichanthelium dichotomum var. nitidum

(Lam.) LeBlond

###### Distribution

Wet pine savannas (WLPS).

###### Notes

Infrequent. May–Oct. Thornhill 312 (NCSC). [< *Panicum dichotomum* L. sensu RAB; = Dichanthelium dichotomum (L.) Gould ssp. nitidum (Lam.) Freckmann & Lelong sensu FNA; = Weakley]

##### Dichanthelium dichotomum var. roanokense

(Ashe) LeBlond

###### Ecological interactions

####### Conservation status

W1; S2, G5T4?.

###### Distribution

Wet pine savannas (WLPS, VWLPS).

###### Notes

Occasional. May–Oct. Thornhill 273, 347, 960 (NCSC). Specimens seen in the vicinity: Sandy Run: Sorrie 6381 (NCU!), Taggart SARU 609 (WNC!). [< *Panicum dichotomum* L. sensu RAB; < Dichanthelium dichotomum (L.) Gould ssp. roanokense (Ashe) Freckmann & Lelong sensu FNA; = Weakley]

##### Dichanthelium dichotomum

(L.) Gould

###### Distribution

Wet pine savannas (VWLPS).

###### Notes

May–Oct. Reported from near Sandy Run by LeBlond (1999), but no specimens have been seen in Shaken Creek Preserve by the senior author. [< *Panicum dichotomum* L. sensu RAB; = Dichanthelium dichotomum (L.) Gould ssp. dichotomum sensu FNA; = Weakley]

##### Dichanthelium ensifolium

(Baldwin ex Elliott) Gould

###### Distribution

Pine/scrub oak sandhills (PSOS-MT), mesic pine savannas (MPS-CP), wet pine flatwoods (WPF-T), wet pine savannas (SPS-T, SPS-RF, WLPS, VWLPS), roadsides.

###### Notes

Frequent. May–Oct. Thornhill 276, 288, 361, 400, 1161, 1294 (NCSC). [< *Panicum ensifolium* Baldwin ex Elliott sensu RAB; = Dichanthelium ensifolium (Baldwin ex Elliott) Gould ssp. ensifolium sensu FNA; = Weakley]

##### Dichanthelium leucothrix

(Nash) Freckmann

###### Distribution

Wet pine savannas (SPS-T, SPS-RF, WLPS, VWLPS), adjacent roadsides.

###### Notes

Frequent. May–Oct. Thornhill 268, 402, 928, 1293, 1309 (NCSC). [= *Panicum leucothrix* Nash sensu RAB; = Dichanthelium acuminatum (Sw.) Gould & C. A. Clark ssp. leucothrix (Nash) Freckmann & Lelong sensu FNA; = Weakley]

##### Dichanthelium longiligulatum

(Nash) Freckmann

###### Distribution

Wet pine savannas (SPS-T, WLPS, VWLPS), adjacent ditches.

###### Notes

Occasional. May–Sep. Thornhill 250, 401, 440, 1220, 1306, 1310, 1347 (NCSC). Specimens seen in the vicinity: Sandy Run [Hancock]: Taggart SARU 654 (WNC!). [= *Panicum longiligulatum* Nash sensu RAB; = Dichanthelium acuminatum (Sw.) Gould & C. A. Clark ssp. longiligulatum (Nash) Freckmann & Lelong sensu FNA; = Weakley]

##### Dichanthelium mattamuskeetense

(Ashe) Mohlenbr.

###### Distribution

Wet pine flatwoods (WPF-T), wet pine savannas (SPS-T, SPS-RF, WLPS).

###### Notes

Occasional. May–Oct. Thornhill 257, 279, 289, 1313, 1506 (NCSC). [< *Panicum dichotomum* L. sensu RAB; < Dichanthelium dichotomum (L.) Gould ssp. mattamuskeetense (Nash) Freckmann & Lelong sensu FNA; = Weakley]

##### Dichanthelium ovale var. addisonii

(Nash) Gould & C.A. Clark

###### Distribution

Dry to damp, sandy woods and fields.

###### Notes

May–Oct. Reported from Sandy Run [Neck] by LeBlond and Weakley (1991), but no specimens have been seen in Shaken Creek Preserve by the senior author. [= *Panicum commonsianum* Ashe sensu RAB; = Dichanthelium ovale (Elliott) Gould & C. A. Clark ssp. pseudopubescens (Nash) Freckmann & Lelong sensu FNA; = Weakley]

##### Dichanthelium ovale var. ovale

(Elliott) Gould & C.A. Clark

###### Ecological interactions

####### Conservation status

W1; S2S3, G5T5.

###### Distribution

Wet pine savannas (WLPS).

###### Notes

Rare. May–Oct. Thornhill 1401 (NCSC). Specimens seen in the vicinity: Sandy Run: Taggart SARU 537 (WNC!). [= *Panicum ovale* Elliott sensu RAB; = Dichanthelium ovale (Elliott) Gould & C. A. Clark ssp. ovale sensu FNA; = Weakley]

##### Dichanthelium scabriusculum

(Elliott) Gould & C.A. Clark

###### Distribution

Wet pine savannas (SPS-RF, WLPS, VWLPS), adjacent roadsides, ditches.

###### Notes

Frequent. May–Oct. Thornhill 407, 618, 688, 691, 1166, 1167, 1168, 1169 (NCSC). Specimens seen in the vicinity: Sandy Run [Haw’s Run]: Taggart SARU 544 (WNC!). [< *Panicum scabriusculum* Elliott sensu RAB; < *Dichanthelium scabriusculum* (Elliott) Gould & C.A. Clark sensu FNA; = Weakley]

##### Dichanthelium scoparium

(Lam.) Gould

###### Distribution

Wet pine savannas (WLPS), adjacent roadsides, ditches.

###### Notes

Occasional. May–Oct. Thornhill 571, 643, 791 (NCSC). Specimens seen in the vicinity: Sandy Run [Neck]: Wilbur 53642 (DUKE!). [= *Panicum scoparium* Lam. sensu RAB; = FNA, Weakley]

##### Dichanthelium species 12 (=chrysopsidifolium)

###### Distribution

Pine savannas.

###### Notes

May–Oct. Not seen in Shaken Creek Preserve by the senior author. Specimens seen in the vicinity: Sandy Run [Haw’s Run]: Taggart SARU 609 (WNC!; as *Dichanthelium aciculare* (Desv. ex Poir.) Gould & C.A. Clark). [< *Panicum consanguineum* Kunth sensu RAB; < *Dichanthelium consanguineum* (Kunth) Gould & C.A. Clark sensu FNA; = Weakley]

##### Dichanthelium species 3 (=lancearium)

###### Distribution

Wet pine flatwoods (WPF-T).

###### Notes

Occasional. May–Sep. Thornhill 1298, 1315, 1325 (NCSC). Specimens seen in the vicinity: Sandy Run [Neck]: Wilbur 55275 (DUKE!; as *Panicum lancearium*). [= *Panicum lancearium* Trin. sensu RAB; < Dichanthelium portoricense (Desv. ex Ham.) B.F. Hansen & Wunderlin spp. patulum (Scribn. & Merr.) Freckmann & Lelong sensu FNA; = Weakley]

##### Dichanthelium sphaerocarpon

(Elliott) Gould

###### Distribution

Wet pine savannas (VWLPS), adjacent roadsides.

###### Notes

Infrequent. May–Oct. Thornhill 773, 1416 (NCSC). Specimens seen in the vicinity: Sandy Run [Haw’s Run]: Taggart SARU 542 (WNC!); Sandy Run [Neck]: LeBlond 2262 (NCU!), Wilbur 55280 (DUKE!; as *Panicum sphaerocarpon*). [= *Panicum sphaerocarpon* Elliott sensu RAB; = FNA, Weakley]

##### Dichanthelium strigosum var. leucoblepharis

(Trin.) Freckmann

###### Distribution

Wet pine savannas (WLPS).

###### Notes

May–Oct. Reported from Shaken Creek Preserve by LeBlond (2000), but no specimens have been seen on site by the senior author. [= *Panicum ciliatum* Elliott sensu RAB; = Dichanthelium strigosum (Muhl. ex Elliott) Freckmann ssp. leucoblepharis (Trin.) Freckmann & Lelong sensu FNA; = Weakley]

##### Dichanthelium strigosum var. strigosum

(Muhl. ex Elliott) Freckmann

###### Distribution

Wet pine savannas (WLPS).

###### Notes

Infrequent. May–Oct. Thornhill 1414 (NCSC). [< *Panicum strigosum* Muhl. ex Elliott sensu RAB; = Dichanthelium strigosum (Muhl. ex Elliott) Freckmann ssp. strigosum sensu FNA; = Weakley]

##### Dichanthelium tenue

(Muhl.) Freckmann & Lelong

###### Distribution

Wet pine flatwoods (WPF-T), wet pine savannas (SPS-RF, WLPS).

###### Notes

Occasional. May–Oct. Thornhill 301, 1307, 1326, 1411, 1415 (NCSC). [= *Panicum tenue* Muhl. sensu RAB; = FNA, Weakley]

##### Dichanthelium villosissimum var. villosissimum

(Nash) Freckmann

###### Distribution

Wet pine flatwoods (WPF-T), wet pine savannas (VWLPS).

###### Notes

Infrequent. Apr–Sep. Thornhill 1324 (NCSC). [= *Panicum villosissimum* Nash sensu RAB; = Dichanthelium ovale (Elliott) Gould & C. A. Clark subsp. villosissimum (Nash) Freckmann & Lelong sensu FNA; = Weakley]

##### Dichanthelium webberianum

(Nash) LeBlond

###### Ecological interactions

####### Conservation status

W1; S3, GNR.

###### Distribution

Wet pine flatwoods (WPF-T), wet pine savannas (SPS-RF)

###### Notes

Occasional. May–Aug. Thornhill 961, 1314, 1316 (NCSC). [= *Panicum webberianum* Nash sensu RAB; < *Dichanthelium portoricense (Desv. ex Ham.) B.F. Hansen & Wunderlin spp. patulum* (Scribn. & Merr.) Freckmann & Lelong sensu FNA; = Weakley]

##### Eragrostis elliottii

S. Watson

###### Distribution

Wet pine savannas (VWLPS).

###### Notes

Infrequent. Sep–Oct. Thornhill 1093, 1190 (NCSC). Specimens seen in the vicinity: Sandy Run [Hancock]: Taggart SARU 524 (WNC!). [= RAB, FNA, Weakley]

##### Eragrostis refracta

(Muhl. ex Elliott) Scribn.

###### Distribution

Wet pine savannas (SPS-T, SPS-RF, VWLPS), adjacent roadsides.

###### Notes

Frequent. Jul–Oct. Thornhill 22, 815, 816, 862 (NCSC). Specimens seen in the vicinity: Sandy Run [Hancock]: Taggart SARU 416 (WNC!). [= RAB, FNA, Weakley]

##### Gymnopogon brevifolius

Trin.

###### Distribution

Wet pine savannas (WLPS, VWLPS).

###### Notes

Infrequent. Aug–Oct. Thornhill 1056, 1071, 1153, 1156 (NCSC). [= RAB, FNA, Weakley]

##### Muhlenbergia capillaris

(Lam.) Trin.

###### Distribution

Roadside immediately adjacent and scraped area within wet pine savanna (VWLPS).

###### Notes

Rare. Late Aug–Oct. Thornhill 1114, 1199 (NCSC). [< RAB; = FNA, Weakley]

##### Muhlenbergia expansa

(Poir.) Trin.

###### Distribution

Wet pine savannas (WLPS, VWLPS).

###### Notes

Abundant. Late Aug–Oct. Thornhill 771, 864, 1012, 1013, 1014, 1015, 1016 (NCSC). Specimens seen in the vicinity: Sandy Run [Hancock]: Taggart SARU 434 (WNC!); Sandy Run [Neck]: Wilbur 57656, 57666 (DUKE!). [= RAB, FNA, Weakley]

##### Muhlenbergia torreyana

(Schult.) Hitchc.

###### Ecological interactions

####### Conservation status

SC-V; S2, G3.

###### Distribution

Wet pine savannas (WLPS).

###### Notes

Rare. Aug–Nov. LeBlond 4859 (NCU!), Sorrie 9501 (NCU!), Thornhill 1053 (NCSC). [= FNA, Weakley]

##### Panicum dichotomiflorum var. puritanorum

Svenson

###### Ecological interactions

####### Conservation status

SR-P; S1, G5T4.

###### Distribution

Wet pine savannas (SPS-T).

###### Notes

Rare. Jul–Oct. Thornhill 935 (NCSC). [= Panicum dichotomiflorum Michx. subsp. puritanorum (Svenson) Freckmann & Lelong sensu FNA; = Weakley]

##### Panicum hemitomon

Schult.

###### Distribution

Wet pine savannas (VWLPS).

###### Notes

Occasional. Jun–Jul. Not seen in Shaken Creek Preserve by the senior author. Specimens seen in the vicinity: Sandy Run [Hancock]: Taggart SARU 241 (WNC!). Though reported by Taggart (2010) as occurring in anthopogenic wetlands and swamps, specimen label data for Taggart’s voucher indicates that the specimen occurred “as a single colony within [a] wet savanna”, where growing with numerous species of savanna affinity, including *Ctenium aromaticum*, *Panicum virgatum*, *Polygala ramosa*, and *Rhynchospora* spp. [= RAB, FNA, Weakley]

##### Panicum verrucosum

Muhl.

###### Distribution

Wet pine savannas (VWLPS).

###### Notes

Occasional. Aug–Oct. Thornhill 1062, 1109, 1124 (NCSC). Specimens seen in the vicinity: Sandy Run [Hancock]: Taggart SARU 611 (WNC!). [= RAB, FNA, Weakley]

##### Panicum virgatum

L.

###### Distribution

Wet pine savannas (WLPS, VWLPS), ditches.

###### Notes

Occasional. Jun–Oct. Thornhill 789, 790, 866, 869 (NCSC). Specimens seen in the vicinity: Sandy Run [Hancock]: Taggart SARU 248 (WNC!; as Panicum virgatum var. virgatum); Sandy Run [Neck]: Wilbur 53711 (DUKE!). [= RAB, FNA; > *Panicum virgatum* L. various varieties sensu Weakley]

##### Paspalum floridanum

Michx.

###### Distribution

Pine savannas.

###### Notes

Aug–Oct. Not seen in Shaken Creek Preserve by the senior author. Specimens seen in the vicinity: Sandy Run [Neck]: Wilbur 53652, 57639 (DUKE!). [= RAB, FNA, Weakley]

##### Paspalum praecox var. curtisianum

(Steud.) Vasey

###### Ecological interactions

####### Conservation status

W1; S2S3, G4 (as P. praecox)

###### Distribution

Wet pine savannas (WLPS, VWLPS).

###### Notes

Occasional. Jun–Oct. Thornhill 403, 434, 450, 572, 577, 1055, 1088, 1099, 1163 (NCSC). [= RAB; < *Paspalum praecox* Walter sensu FNA; = Weakley]

##### Paspalum praecox var. praecox

Walter

###### Ecological interactions

####### Conservation status

W1; S2S3, G4 (as P. praecox).

###### Distribution

Wet pine savannas (VWLPS).

###### Notes

Rare. May–Jul. Thornhill 734 (NCSC). Specimens seen in the vicinity: Sandy Run: Taggart SARU 195 (WNC!). [= RAB; < *Paspalum praecox* Walter sensu FNA; = Weakley]

##### Paspalum setaceum var. ciliatifolium

(Michx.) Vasey

###### Distribution

Pine savannas.

###### Notes

Jun–Sep. Not seen in Shaken Creek Preserve by the senior author. Specimens seen in the vicinity: Sandy Run [Haw’s Run]: Taggart SARU 545 (WNC!). [< *Paspalum setaceum* Michx. sensu RAB; = FNA, Weakley]

##### Paspalum setaceum var. muhlenbergii

(Nash) D.J. Banks

###### Distribution

Wet pine savannas (SPS-RF, VWLPS), adjacent roadsides.

###### Notes

Infrequent. Jun–Sep. Thornhill 646, 1077 (NCSC). [< *Paspalum setaceum* Michx. sensu RAB; = FNA, Weakley]

##### Paspalum setaceum var. setaceum

Michx.

###### Distribution

Wet pine flatwoods (WPF-T), wet pine savannas (VWLPS), adjacent roadsides.

###### Notes

Occasional. Jun–Sep. Thornhill 549, 1531 (NCSC). [< *Paspalum setaceum* Michx. sensu RAB; = FNA, Weakley]

##### Saccharum baldwinii

Spreng.

###### Distribution

Wet pine savannas (WLPS, VWLPS).

###### Notes

Infrequent. Jul–Oct. Thornhill 1111, 1189 (NCSC). [= *Erianthus strictus* Baldwin sensu RAB; = FNA, Weakley]

##### Saccharum brevibarbe var. contortum

(Elliott) R.D. Webster

###### Distribution

Wet pine flatwoods.

###### Notes

Late Jul–Oct. Not seen in Shaken Creek Preserve by the senior author. Specimens seen in the vicinity: Sandy Run [RMK]: Taggart SARU 477 (WNC!). [= *Erianthus contortus* Elliott sensu RAB; = FNA, Weakley]

##### Saccharum coarctatum

(Fernald) R.D. Webster

###### Distribution

Wet pine savannas (WLPS), ditches.

###### Notes

Occasional. Sep–Oct. Thornhill 1019, 1249 (NCSC). [< *Erianthus brevibarbis* Michx. sensu RAB; = FNA, Weakley]

##### Saccharum giganteum

(Walter) Pers.

###### Distribution

Wet pine savannas (WLPS), ditches.

###### Notes

Occasional. Sep–Oct. Thornhill 1023, 1187, 1188, 1215 (NCSC). Specimens seen in the vicinity: Sandy Run [Hancock]: Taggart SARU 4 (WNC!); Sandy Run [Neck]: Wilbur 57647, 57670 (DUKE!; as *Erianthus giganteus*). [= *Erianthus giganteus* (Walter) P. Beauv. sensu RAB; = FNA, Weakley]

##### Schizachyrium scoparium var. scoparium

(Michx.) Nash

###### Distribution

Pine/scrub oak sandhills (PSOS-MT), mesic pine savannas (MPS-CP), wet pine flatwoods (WPF-T), wet pine savannas (SPS-T, SPS-RF, WLPS, VWLPS).

###### Notes

Frequent. (Jun–)Aug–Oct. Thornhill 769, 849, 863, 867, 914, 926, 1248 (NCSC). Specimens seen in the vicinity: Highway 50: Wilbur 9431 (DUKE!); Sandy Run [Hancock]: Taggart SARU 536 (WNC!). [< *Andropogon scoparius* Michx. sensu RAB; = FNA, Weakley]

##### Setaria parviflora

(Poir.) Kerguélen

###### Distribution

Wet pine savannas (VWLPS), adjacent roadsides.

###### Notes

Infrequent. May–Oct. Thornhill 711, 768, 855, 1373 (NCSC). Specimens seen in the vicinity: Sandy Run [Hancock]: Taggart SARU 240 (WNC!). [= *Setaria geniculata* P. Beauv. sensu RAB; = FNA, Weakley]

##### Sorghastrum nutans

(L.) Nash

###### Distribution

Pine/scrub oak sandhills (PSOS-MT), mesic pine savannas (MPS-CP), wet pine flatwoods (WPF-T).

###### Notes

Infrequent. Sep–Oct. Thornhill 1095, 1158 (NCSC). Specimens seen in the vicinity: Sandy Run [Hancock]: Taggart SARU 465 (WNC!), Wilbur 57654 (DUKE!). [= RAB, FNA, Weakley]

##### Sporobolus pinetorum

Weakley & P.M. Peterson

###### Ecological interactions

####### Conservation status

W1; S3, G3.

###### Distribution

Wet pine flatwoods (WPF-T), wet pine savannas (SPS-T, SPS-RF, WLPS, VWLPS).

###### Notes

Abundant. Jun–Sep(–later in response to fire). Thornhill 651, 656, 694, 699, 724, 770, 818, 1018 (NCSC). Specimens seen in the vicinity: Highway 50: Wilbur 9430 (DUKE!; as *Sporobolus teretifolius*); Sandy Run: Sorrie 5889 (NCU!), Taggart SARU 560 (WNC!). [>< *Sporobolus teretifolius* R.M. Harper sensu RAB; = FNA, Weakley]

#### Smilacaceae

##### Smilax bona-nox

L.

###### Distribution

Wet pine savannas (VWLPS), wet pine flatwoods (WPF-T), adjacent roadsides.

###### Notes

Rare (in pertinent habitats). Late Apr–May; Sep–Nov. Thornhill 1409, 1485 (NCSC). Specimens seen in the vicinity: Sandy Run [Patterson]: Taggart SARU 414 (WNC!). [= RAB, FNA, Weakley]

##### Smilax glauca

Walter

###### Distribution

Pine/scrub oak sandhills (PSOS-MT), wet pine savannas (SPS-T, WLPS, VWLPS).

###### Notes

Occasional. Late Apr–early Jun; Sep–Nov. Thornhill 294, 395, 837, 1052, 1235 (NCSC). Specimens seen in the vicinity: Sandy Run [RMK]: Taggart SARU 282 (WNC!). [= RAB, FNA, Weakley]

##### Smilax laurifolia

L.

###### Distribution

Wet pine flatwoods (WPF-T), wet pine savannas (SPS-T, SPS-RF, WLPS, VWLPS).

###### Notes

Frequent. Jul–Aug; Sep–Oct. (of 2nd year). Thornhill 168, 181, 262 (NCSC). Specimens seen in the vicinity: Sandy Run [O’Berry]: Taggart SARU 159 (WNC!). [= RAB, FNA, Weakley]

##### Smilax rotundifolia

L.

###### Distribution

Wet pine flatwoods (WPF-T).

###### Notes

Infrequent. Apr–May; Sep–Nov. Thornhill 1359 (NCSC). Specimens seen in the vicinity: Sandy Run [Hancock]: Taggart SARU 170 (WNC!). [= RAB, FNA, Weakley]

##### Smilax smallii

Morong

###### Distribution

Margins of wet pine savannas (VWLPS) and swamp forests.

###### Notes

Occasional. Jun–Jul; Apr–Jun (of 2nd year). Thornhill 1182, 1492 (NCSC). [= RAB, FNA, Weakley]

#### Tofieldiaceae

##### Pleea tenuifolia

Michx.

###### Distribution

Wet pine flatwoods (WPF-T), wet pine savannas (SPS-T, SPS-RF, WLPS, VWLPS).

###### Notes

Frequent (abundant in SPS-RF). Sep–Oct. Thornhill 27 (NCSC). Specimens seen in the vicinity: Sandy Run [Hancock]: Taggart SARU 469 (WNC!); Sandy Run [Neck]: Wilbur 57623A (DUKE!). [= RAB, FNA, Weakley]

##### Tofieldia glabra

Nutt.

###### Distribution

Wet pine savannas (SPS-T, SPS-RF, VWLPS).

###### Notes

Infrequent. (Late Aug–)late Sep–Oct; Oct–Nov. Thornhill 1121, 1202, 1208 (NCSC). Specimens seen in the vicinity: Sandy Run [Hancock]: Taggart SARU 507 (WNC!); Sandy Run [Neck]: Wilbur 57649 (DUKE!). [= RAB, FNA, Weakley]

##### Triantha racemosa

(Walter) Small

###### Distribution

Wet pine savannas (SPS-T, WLPS, VWLPS).

###### Notes

Occasional. Jun–early Aug; late Sep–Oct. Thornhill 551, 604, 667, 682 (NCSC). Specimens seen in the vicinity: Sandy Run [Hancock]: Taggart SARU 348 (WNC!), Wyland s.n. (NCSC!; as Tofieldia racemosa var. racemosa); Sandy Run [Neck]: Wilbur 53692 (DUKE!; as *Tofieldia racemosa*). [= Tofieldia racemosa (Walter) Britton, Sterns & Poggenb. var. racemosa RAB; = FNA, Weakley]

#### Trilliaceae

##### Trillium pusillum var. pusillum

Michx.

###### Ecological interactions

####### Conservation status

State E, FSC; S2, G3T2.

###### Distribution

Margins of pine savannas and adjacent swamps.

###### Notes

Late Mar–May; Jun–Jul. Reported from Sandy Run by LeBlond and Weakley (1991), but no specimens have been seen in Shaken Creek Preserve by the senior author. [< *Trillium pusillum* Michx. sensu RAB; < FNA; = Weakley]

#### Xyridaceae

##### Xyris ambigua

Beyr. ex Kunth

###### Distribution

Wet pine savannas (SPS-T, SPS-RF, WLPS, VWLPS) and borrow pits.

###### Notes

Frequent. Jun–Aug. Thornhill 573, 588, 628, 669, 685, 708, 719, 801 (NCSC). Specimens seen in the vicinity: Sandy Run [Hancock]: Taggart SARU 384 (WNC!). [= RAB, FNA, Weakley]

##### Xyris baldwiniana

Schult.

###### Distribution

Wet pine savannas (SPS-T).

###### Notes

Rare. Jun–Jul. Thornhill 528 (NCSC). Specimens seen in the vicinity: Sandy Run [RMK]: Taggart SARU 236 (WNC!). [= RAB, FNA, Weakley]

##### Xyris brevifolia

Michx.

###### Ecological interactions

####### Conservation status

W1; S3, G4G5.

###### Distribution

Wet pine savannas (SPS-T, SPS-RF).

###### Notes

Infrequent. Jun–Aug. Thornhill 37, 800 (NCSC). Specimens seen in the vicinity: Sandy Run: Taggart SARU 350 (WNC!). [= RAB, FNA, Weakley]

##### Xyris caroliniana

Walter

###### Distribution

Pine/scrub oak sandhills (PSOS-MT), mesic pine savannas (MPS-CP), wet pine flatwoods (WPF-T), wet pine savannas (SPS-T, SPS-RF, WLPS, VWLPS), roadsides.

###### Notes

Frequent. Jun–Jul. Thornhill 578, 587, 677 (NCSC). Specimens seen in the vicinity: Sandy Run [Patterson]: Taggart SARU 303 (WNC!). [= RAB, FNA, Weakley]

##### Xyris curtissii

Malme

###### Distribution

Wet pine savannas (SPS-RF, WLPS).

###### Notes

Occasional. Jul–Aug. Thornhill 748, 793, 1507 (NCSC). Specimens seen in the vicinity: Sandy Run [Haw’s Run]: Taggart SARU 649 (WNC!). [= RAB; = Xyris difformis Malme var. curtissii (Malme) Kral sensu FNA; = Weakley]

##### Xyris fimbriata

Elliott

###### Distribution

Wet pine savannas (SPS-T), borrow pits.

###### Notes

Infrequent. Sep–Oct. Thornhill 31, 919 (NCSC). [= RAB, FNA, Weakley]

##### Xyris flabelliformis

Chapm.

###### Ecological interactions

####### Conservation status

W1; S1, G4.

###### Distribution

Wet pine savannas (VWLPS).

###### Notes

May–Jun. Reported from Sandy Run by LeBlond (2000), but no specimens have been seen in Shaken Creek Preserve by the senior author. [= RAB, FNA, Weakley]

##### Xyris floridana

(Kral) E.L. Bridges & Orzell

###### Ecological interactions

####### Conservation status

SR-P; S1, G5T4T5.

###### Distribution

Pine savannas, flatwoods, and adjacent ditches.

###### Notes

Aug. Reported from Sandy Run by LeBlond (1999), but no specimens have been seen in Shaken Creek Preserve by the senior author. [= Xyris difformis Chapm. var. floridana Kral sensu FNA; = Weakley]

##### Xyris iridifolia

Chapm.

###### Ecological interactions

####### Conservation status

W7; S2, G4G5T4T5.

###### Distribution

Borrow pits and local depressions within pine savannas, ditches.

###### Notes

Jul–Sep. Not seen in Shaken Creek Preserve by the senior author. Specimens seen in the vicinity: Sandy Run: Taggart SARU 658 (WNC!; as *Xyris difformis* Chapm.). [= RAB; = Xyris laxifolia Mart. var. iridifolia (Chapm.) Kral sensu FNA; = Weakley]

##### Xyris jupicai

Rich.

###### Distribution

Borrow pits within pine savannas.

###### Notes

Jul–Sep. Reported from Shaken Creek Preserve by LeBlond (2000), but no specimens have been seen by the senior author. [= RAB, FNA, Weakley]

##### Xyris scabrifolia

R.M. Harper

###### Ecological interactions

####### Conservation status

SC-V, FSC; S2, G3.

###### Distribution

Pine savannas.

###### Notes

Jul–Sep. Not seen in Shaken Creek Preserve by the senior author. Specimens seen in the vicinity: Sandy Run: McMillan 1788 (NCU!). [< FNA; = Weakley]

##### Xyris species 1

###### Ecological interactions

####### Conservation status

W2; S2, G2.

###### Distribution

Wet pine savannas (SPS-T).

###### Notes

Rare. Jul–Sep. Thornhill 902 (NCSC). Specimens seen in the vicinity: Sandy Run: Taggart SARU 650 (WNC!). [< *Xyris curtissii* Malme sensu RAB; < Xyris difformis Malme var. curtissii (Malme) Kral sensu FNA; = Weakley]

### BASAL ANGIOSPERMS, MAGNOLIIDS, and EUDICOTYLEDONS

#### Adoxaceae

##### Viburnum nudum

L.

###### Distribution

Ditches within and adjacent to wet pine flatwoods (WPF-T).

###### Notes

Infrequent. Apr–May; Aug–Oct. Thornhill 239, 1330 (NCSC). Specimens seen in the vicinity: Sandy Run [O’Berry]: Taggart SARU 91 (WNC!). [= RAB, Weakley]

#### Altingiaceae

##### Liquidambar styraciflua

L.

###### Distribution

Mesic pine savannas (MPS-CP), wet pine flatwoods (WPF-T), wet pine savannas (SPS-T, SPS-RF, WLPS, VWLPS).

###### Notes

Occasional (frequent only in areas not recently burned). Apr–May; Aug–Sep. Thornhill 346, 432 (NCSC). Specimens seen in the vicinity: Old Maple Hill Road: Wilbur 55264 (DUKE!); Sandy Run [O’Berry]: Taggart SARU 164 (WNC!). [= RAB, FNA, Weakley]

#### Anacardiaceae

##### Rhus copallinum var. copallinum

L.

###### Distribution

Pine/scrub oak sandhills (PSOS-MT), mesic pine savannas (MPS-CP), wet pine flatwoods (WPF-T).

###### Notes

Infrequent. Jul–Sep; Aug–Oct. Thornhill 329, 954 (NCSC). Specimens seen in the vicinity: Sandy Run [Neck]: Levy s.n. (DUKE!; as *Rhus copallina*); Sandy Run [Patterson]: Taggart SARU 412 (WNC!). [< *Rhus copallina* L. sensu RAB; = Weakley]

##### Toxicodendron radicans var. radicans

(L.) Kuntze

###### Distribution

Swampy margins of wet pine flatwoods (WPF-T) and wet pine savannas (VWLPS).

###### Notes

Infrequent. Late Apr–May; Aug–Oct. Thornhill 136 (NCSC). Specimens seen in the vicinity: Sandy Run [Hancock]: Taggart SARU 199 (WNC!). [< *Rhus radicans* L. sensu RAB; = Weakley]

#### Apiaceae

##### Centella erecta

(L. f.) Fernald

###### Distribution

Wet pine savannas (WLPS, VWLPS), margins of borrow pits, roadsides.

###### Notes

Frequent. Jun–Aug; Jul–Sep. Thornhill 832, 879 (NCSC). Specimens seen in the vicinity: Sandy Run [Hancock]: Taggart SARU 450 (WNC!). [= *Centella asiatica* (L.) Urb. sensu RAB, Weakley]

##### Eryngium aquaticum var. aquaticum

L.

###### Distribution

Wet pine savannas (VWLPS).

###### Notes

Rare. Jul–Sep. Thornhill 842, 1537, 1548 (NCSC). Specimens seen in the vicinity: Sandy Run [Haw’s Run]: Taggart SARU 661 (WNC!). [= RAB, Weakley]

##### Eryngium aquaticum var. ravenelii

(A. Gray) Mathias & Constance

###### Ecological interactions

####### Conservation status

SR-P; S1, G4T2T4Q.

###### Distribution

Pine savannas.

###### Notes

Jul–Sep. Not seen in Shaken Creek Preserve by the senior author. Specimens seen in the vicinity: Sandy Run: LeBlond 5425 (NCU!), Taggart SARU 411 (WNC!), Wilbur 57680 (DUKE!; as *Eryngium aquaticum*). [= RAB, Weakley]

##### Eryngium integrifolium

Walter

###### Distribution

Wet pine savannas (WLPS, VWLPS).

###### Notes

Occasional. Aug–Oct. Thornhill 7, 951 (NCSC). Specimens seen in the vicinity: Sandy Run [Hancock]: Taggart SARU 421 (WNC!); Sandy Run[Neck]: Wilbur 57676 (DUKE!). [= RAB, Weakley]

##### Eryngium yuccifolium var. synchaetum

A. Gray ex J.M. Coult. & Rose

###### Ecological interactions

####### Conservation status

W2; S2, G5T5.

###### Distribution

Wet pine savannas (WLPS, VWLPS).

###### Notes

Infrequent. Jun–Aug. Thornhill 689, 703 (NCSC). Specimens seen in the vicinity: Sandy Run: Levy s.n. (DUKE!; as *Eryngium yuccifolium*), Taggart SARU 280 (WNC!), Wilbur 53709 (DUKE!; as *Eryngium yuccifolium*). [= RAB, Weakley]

##### Eryngium yuccifolium var. yuccifolium

Michx.

###### Distribution

Wet pine savannas (WLPS, VWLPS).

###### Notes

Infrequent. Jun–Aug. Thornhill 830 (NCSC). Specimens seen in the vicinity: Sandy Run [Neck]: Taggart SARU 340 (WNC!). [= RAB, Weakley]

##### Oxypolis rigidior

(L.) Raf.

###### Distribution

Wet pine savannas (VWLPS).

###### Notes

Rare. Aug–Oct; Oct–Nov. Thornhill 1043 (NCSC). Specimens seen in the vicinity: Sandy Run [Neck]: Taggart SARU 484 (WNC!). [= RAB, Weakley]

##### Oxypolis ternata

(Nutt.) A. Heller

###### Distribution

Wet pine savannas (VWLPS).

###### Notes

Rare. Sep–Oct; Oct–Nov. Thornhill 1070, 1128 (NCSC). Specimens seen in the vicinity: Sandy Run [Hancock]: Taggart SARU 494 (WNC!; as *Oxypolis denticulata*). [= RAB, Weakley]

##### Ptilimnium capillaceum

(Michx.) Raf.

###### Distribution

Wet pine savannas (VWLPS), adjacent ditches.

###### Notes

Rare. Jun–Aug; Jul–Sep. Thornhill 622 (NCSC). Specimens seen in the vicinity: Sandy Run [Hancock]: Taggart SARU 346 (WNC!). [= RAB, Weakley]

##### Tiedemannia filiformis var. filiformis

(Walter) Feist & S.R. Downie

###### Distribution

Wet pine savannas (WLPS, VWLPS), ditches, borrow pits.

###### Notes

Occasional. Jul–Aug; Aug–Sep. Thornhill 742, 833, 983, 1039 (NCSC). Specimens seen in the vicinity: Sandy Run [Neck]: Taggart SARU 347 (WNC!; as *Oxypolis filiformis*). [= *Oxypolis filiformis* (Walter) Britton sensu RAB; = Weakley]

#### Apocynaceae

##### Asclepias lanceolata

Walter

###### Distribution

Wet pine savannas (SPS-RF, WLPS, VWLPS).

###### Notes

Infrequent. Jun–Aug; Aug–Sep. Thornhill 1, 378, 423 (NCSC). Specimens seen in the vicinity: Sandy Run [Hancock]: Taggart SARU 150 (WNC!); Sandy Run [Neck]: Wilbur 55303 (DUKE!). [= RAB, Weakley]

##### Asclepias longifolia

Michx.

###### Ecological interactions

####### Conservation status

W1; S2S3, G4G5.

###### Distribution

Wet pine savannas (WLPS, VWLPS).

###### Notes

Infrequent. May–Jun; Jun–Jul. Thornhill 249, 278, 355 (NCSC). Specimens seen in the vicinity: Sandy Run: Taggart SARU 137 (WNC!). [= RAB, Weakley]

##### Asclepias pedicellata

Walter

###### Ecological interactions

####### Conservation status

SC-V; S3, G4.

###### Distribution

Wet pine flatwoods (WPF-T).

###### Notes

Rare. Jul–Aug. [= RAB, Weakley]

##### Asclepias rubra

L.

###### Distribution

Wet pine flatwoods.

###### Notes

Jun–Jul; Jul–Sep. Not seen in Shaken Creek Preserve by the senior author. Specimens seen in the vicinity: Sandy Run [Neck]: Taggart SARU 277 (WNC!), Wilbur 55302, 55326 (DUKE!). [= RAB, Weakley]

#### Aquifoliaceae

##### Ilex coriacea

(Pursh) Chapm.

###### Distribution

Wet pine flatwoods (WPF-T), wet pine savannas (SPS-T, SPS-RF).

###### Notes

Frequent. Apr–May; Sep–Oct. Thornhill 261, 309 (NCSC). Specimens seen in the vicinity: Sandy Run [Neck]: Wilbur 53668, 63782 (DUKE!); Sandy Run [O’Berry]: Taggart SARU 104 (WNC!). [= RAB, Weakley]

##### Ilex glabra

(L.) A. Gray

###### Distribution

Mesic pine savannas (MPS-CP), wet pine flatwoods (WPF-T), wet pine savannas (SPS-T, SPS-RF, WLPS, VWLPS).

###### Notes

Abundant. May–Jun; Sep–Nov. Thornhill 43, 135, 171, 186, 237 (NCSC). Specimens seen in the vicinity: Sandy Run [Neck]: Wilbur 53701 (DUKE!); Sandy Run [Patterson]: Taggart SARU 50 (WNC!). [= RAB, Weakley]

##### Ilex myrtifolia

Walter

###### Distribution

Wet pine savannas (WLPS, VWLPS).

###### Notes

Occasional. May–Jun; Oct–Nov. Thornhill 271, 284, 435, 1185 (NCSC). Specimens seen in the vicinity: Sandy Run [RMK]: Taggart SARU 131 (WNC!). [= Ilex cassine L. var. myrtifolia (Walter) Sarg. sensu RAB; = Weakley]

##### Ilex opaca var. opaca

Aiton

###### Distribution

Wet pine flatwoods (WPF-T), wet pine savannas (SPS-RF).

###### Notes

Rare. Apr–Jun; Sep–Oct. Thornhill 97 (NCSC). Specimens seen in the vicinity: Sandy Run [Hancock]: Taggart SARU 102 (WNC!). [< *Ilex opaca* Aiton sensu RAB; = Weakley]

#### Araliaceae

##### Aralia spinosa

L.

###### Distribution

Mesic pine savannas.

###### Notes

Jun–Sep. Not seen in Shaken Creek Preserve by the senior author. Specimens seen in the vicinity: Sandy Run [RMK]: Taggart SARU 428 (WNC!). [= RAB, Weakley]

#### Asteraceae

##### Arnica acaulis

(Walter) Britton, Sterns & Poggenb.

###### Distribution

Pine savannas, sandhills, sandy woodlands, and disturbed areas.

###### Notes

Late Mar–early Jun. Reported from Sandy Run [Neck] by LeBlond and Weakley (1991), but no specimens have been seen in Shaken Creek Preserve by the senior author. [= RAB, FNA, Weakley]

##### Arnoglossum ovatum var. lanceolatum

(Nutt.) D.B. Ward

###### Ecological interactions

####### Conservation status

SR-P; S2, G4G5.

###### Distribution

Wet pine savannas (VWLPS).

###### Notes

Rare. Late Jul–Oct. Thornhill 943 (NCSC). Specimens seen in the vicinity: Sandy Run: Taggart SARU 376 (WNC!). [= *Cacalia lanceolata* Nutt. sensu RAB; < *Arnoglossum ovatum* (Walter) H. Rob. sensu FNA; Weakley]

##### Baccharis glomeruliflora

Pers.

###### Ecological interactions

####### Conservation status

SC-H; S1, G4.

###### Distribution

Ecotone of pine savannas and swamp forests.

###### Notes

Rare. Oct–Nov. Not seen in Shaken Creek Preserve by the senior author. Specimens seen in the vicinity: Sandy Run: Taggart SARU 668, SARU 669 (WNC!). [= RAB, FNA, Weakley]

##### Balduina uniflora

Nutt.

###### Distribution

Wet pine flatwoods (WPF-T), wet pine savannas (SPS-RF, WLPS, VWLPS), roadsides.

###### Notes

Occasional. Late Jul–Sep. Thornhill 870 (NCSC). Specimens seen in the vicinity: Sandy Run [Hancock]: Taggart SARU 409 (WNC!); Sandy Run [Neck]: Wilbur 53637 (DUKE!; as *Helenium pinnatifidum*). [= RAB, FNA, Weakley]

##### Bigelowia nudata var. nudata

(Michx.) DC.

###### Distribution

Wet pine flatwoods (WPF-T), wet pine savannas (WLPS, VWLPS).

###### Notes

Occasional. Aug–Oct. Thornhill 750, 967, 1081, 1082 (NCSC). Specimens seen in the vicinity: Sandy Run [Hancock]: Taggart SARU 418 (WNC!); Sandy Run [Neck]: Wilbur 57614, 57667 (DUKE!). [< *Chondrophora nudata* (Michx.) Britton sensu RAB; = FNA, Weakley]

##### Carphephorus bellidifolius

(Michx.) Torr. & A. Gray

###### Distribution

Mesic pine savannas (MPS-CP).

###### Notes

Rare. Aug–Oct. Thornhill 1542 (NCSC). Specimens seen in the vicinity: Sandy Run [Neck]: Taggart SARU 491 (WNC!). [= RAB, FNA, Weakley]

##### Carphephorus tomentosus

(Michx.) Torr. & A. Gray

###### Distribution

Mesic pine savannas (MPS-CP), wet pine flatwoods (WPF-T), wet pine savannas (SPS-T).

###### Notes

Occasional. Aug–Oct. Thornhill 1000, 1120, 1519 (NCSC). Specimens seen in the vicinity: Sandy Run [Hancock]: Taggart SARU 461 (WNC!); Sandy Run [Neck]: Wilbur 57620 (DUKE!). [= RAB, FNA, Weakley]

##### Chaptalia tomentosa

Vent.

###### Distribution

Pine savannas (WLPS, VWLPS).

###### Notes

Infrequent. Feb–May. Thornhill 87, 94 (NCSC). Specimens seen in the vicinity: Sandy Run [Hancock]: Taggart SARU 9 (WNC!). [= RAB, FNA, Weakley]

##### Chrysopsis mariana

(L.) Elliott

###### Distribution

Pine savannas.

###### Notes

Late Jun–Oct. Not seen in Shaken Creek Preserve by the senior author. Specimens seen in the vicinity: Sandy Run [Hancock]: Taggart SARU 495 (WNC!). [= *Heterotheca mariana* (L.) Shinners sensu RAB; = FNA, Weakley]

##### Cirsium horridulum var. horridulum

Michx.

###### Distribution

Wet pine savannas (VWLPS), adjacent roadsides.

###### Notes

Rare. Late Mar–early Jun. Thornhill 245 (NCSC). Specimens seen in the vicinity: Sandy Run [Hancock]: Taggart SARU 24 (WNC!). [=*Carduus spinosissimus* Walter sensu RAB; = FNA, Weakley]

##### Cirsium horridulum var. vittatum

(Small) R.W. Long

###### Distribution

Wet pine savannas (VWLPS), adjacent roadsides.

###### Notes

Infrequent. May–Jul. Thornhill 377 (NCSC). Specimens seen in the vicinity: Highway 50: LeBlond 4252 (NCU!); Sandy Run [Hancock]: Taggart SARU 592 (WNC!). [= *Carduus smallii* (Britton) H.E. Ahles sensu RAB; = FNA, Weakley]

##### Cirsium lecontei

Torr. & A. Gray

###### Ecological interactions

####### Conservation status

SC-V; S2, G2G3.

###### Distribution

Wet pine savannas (SPS-T).

###### Notes

Rare. Jun–Aug. Thornhill 1454 (NCSC). Specimens seen in the vicinity: Sandy Run: Taggart SARU 250 (WNC!). [= *Carduus lecontei* (Torr. & A. Gray) Pollard sensu RAB; = FNA, Weakley]

##### Cirsium repandum

Michx.

###### Distribution

Pine savannas.

###### Notes

Feb–May. Not seen in Shaken Creek Preserve by the senior author. Specimens seen in the vicinity: Sandy Run [Hancock]: Taggart SARU 342 (WNC!). [= *Carduus repandus* (Michx.) Pers. sensu RAB; = FNA, Weakley]

##### Cirsium virginianum

(L.) Michx.

###### Distribution

Wet pine savannas (WLPS, VWLPS).

###### Notes

Occasional. Aug–Oct. Thornhill 923, 945 (NCSC). Specimens seen in the vicinity: Sandy Run [Hancock]: Taggart SARU 455 (WNC!), Wilbur 57657 (DUKE!). [= *Carduus virginianus* L. sensu RAB; = FNA, Weakley]

##### Coreopsis falcata

F.E. Boynton

###### Distribution

Wet pine flatwoods (WPF-T), wet pine savannas (SPS-T, SPS-RF, WLPS, VWLPS), borrow pits, adjacent roadsides.

###### Notes

Occasional. Early May–early Jul(–later). Thornhill 219, 352, 367, 368, 392 (NCSC). Specimens seen in the vicinity: Sandy Run [Haw’s Run]: Taggart SARU 145 (WNC!); Sandy Run [Neck]: Wilbur 55321 (DUKE!). [= RAB; < *Coreopsis gladiata* Walter sensu FNA; = Weakley]

##### Coreopsis linifolia

Nutt.

###### Distribution

Wet pine flatwoods (WPF-T), wet pine savannas (SPS-T, SPS-RF, WLPS, VWLPS), borrow pits, adjacent roadsides.

###### Notes

Frequent. Early Jul–late Oct. Thornhill 6, 38, 841, 875, 976, 978, 979, 1044 (NCSC). Specimens seen in the vicinity: Sandy Run [Hancock]: Taggart SARU 456 (WNC!); Sandy Run [Neck]: Wilbur 57677 (DUKE!; as *Coreopsis gladiata*). [= *Coreopsis angustifolia* Aiton sensu RAB; < *Coreopsis gladiata* Walter sensu FNA; = Weakley]

##### Coreopsis palustris

Sorrie

###### Ecological interactions

####### Conservation status

SR-P; S1S2, G3G4Q.

###### Distribution

Pine savannas.

###### Notes

Sep–Oct. Not seen in Shaken Creek Preserve by the senior author. Specimens seen in the vicinity: Sandy Run: Taggart SARU 422 (WNC!; as *Coreopsis helianthoides*). [= *Coreopsis helianthoides* Beadle sensu RAB; < *Coreopsis gladiata* Walter sensu FNA; = Weakley]

##### Coreopsis species 1

###### Ecological interactions

####### Conservation status

SR-L; S1, G1?.

###### Distribution

Wet pine savannas (VWLPS), adjacent roadsides.

###### Notes

Rare. Sep–Oct. Thornhill 1171 (NCSC). Specimens seen in the vicinity: Sandy Run: LeBlond 4600, 4654, 5424 (NCU!), Taggart SARU 504 (WNC!). [= Weakley]

##### Elephantopus nudatus

A. Gray

###### Distribution

Mesic pine savannas (MPS-CP), wet pine flatwoods (WPF-T), adjacent roadsdies.

###### Notes

Occasional. Late Jul–Sep. Thornhill 1045, 1086 (NCSC). Specimens seen in the vicinity: Sandy Run [O’Berry]: Taggart SARU 398 (WNC!). [= RAB, FNA, Weakley]

##### Erechtites hieraciifolius

(L.) Raf. ex DC.

###### Distribution

Disturbed areas in pine savannas, dry edges of borrow pits, roadsides.

###### Notes

Rare. Late Jul–Nov. Thornhill 938, 1370 (NCSC). Specimens seen in the vicinity: Sandy Run [Hancock]: Taggart SARU 407 (WNC!). [< RAB; = Erechtites hieraciifolius (L.) Raf. ex DC. var. hieraciifolius sensu FNA; = Weakley]

##### Erigeron vernus

(L.) Torr. & A. Gray

###### Distribution

Wet pine savannas (SPS-T, SPS-RF, WLPS, VWLPS), adjacent roadsides.

###### Notes

Frequent. Late Mar–Jun. Thornhill 112, 177, 213, 217, 766 (NCSC). Specimens seen in the vicinity: Sandy Run [Neck]: Taggart 185 (NCU!), Wilbur 55318, 67091 (DUKE!). [= RAB, FNA, Weakley]

##### Eupatorium capillifolium

(Lam.) Small

###### Distribution

Disturbed (sometimes only slightly so) areas in wet pine flatwoods (WPF-T) and wet pine savannas (SPS-RF, VWLPS), adjacent roadsides.

###### Notes

Rare (frequent in more disturbed areas). Sep–Nov. Thornhill 952, 1143 (NCSC). Specimens seen in the vicinity: Sandy Run [Neck]: Taggart 503 (NCU!). [= Eupatorium capillifolium (Lam.) Small var. capillifolium sensu RAB; = FNA, Weakley]

##### Eupatorium hyssopifolium

L.

###### Distribution

Pine savannas.

###### Notes

Late Jul–Oct. Not seen in Shaken Creek Preserve by the senior author. Specimens seen in the vicinity: Sandy Run [Haw’s Run]: Taggart SARU 639 (WNC!); Sandy Run [Neck]: Wilbur 57618 (DUKE!). [< RAB; = Eupatorium hyssopifolium L. var. hyssopifolium sensu FNA; = Weakley]

##### Eupatorium leucolepis

(DC.) Torr. & A. Gray

###### Distribution

Wet pine savannas (WLPS, VWLPS).

###### Notes

Frequent. Aug–Oct. Thornhill 828, 925, 982, 1213 (NCSC). Sandy Run [Hancock]: Taggart SARU 357 (WNC!). [< RAB; < Eupatorium leucolepis (DC.) Torr. & A. Gray var. leucolepis sensu FNA; = Weakley]

##### Eupatorium mohrii

Greene

###### Distribution

Pine savannas.

###### Notes

Aug–Oct. Not seen in Shaken Creek Preserve by the senior author. Specimens seen in the vicinity: Sandy Run [Hancock]: Taggart SARU 403 (WNC!). [< *Eupatorium recurvans* Small sensu RAB; < FNA; = Weakley]

##### Eupatorium perfoliatum

L.

###### Distribution

Pine savannas.

###### Notes

Aug–Oct. Not seen in Shaken Creek Preserve by the senior author. Specimens seen in the vicinity: Sandy Run [Hancock]: Taggart SARU 466 (WNC!); Sandy Run [Neck]: Wilbur 57642, 57657 (DUKE!). [= RAB, FNA, Weakley]

##### Eupatorium pilosum

Walter

###### Distribution

Wet pine flatwoods (WPF-T), wet pine savannas (SPS-T, SPS-RF).

###### Notes

Occasional. Aug–Oct. Thornhill 947, 1520 (NCSC). Specimens seen in the vicinity: Sandy Run [Hancock]: Taggart SARU 356 (WNC!); Sandy Run [Neck]: Wilbur 53712 (DUKE!). [= RAB, FNA, Weakley]

##### Eupatorium recurvans

Small

###### Ecological interactions

####### Conservation status

W7; S1?, G3G4Q.

###### Distribution

Wet pine savannas (SPS-RF, WLPS).

###### Notes

Infrequent. Aug–Oct. Thornhill 1122, 1146, 1206, 1237 (NCSC). [< RAB; < *Eupatorium mohrii* Greene sensu FNA; = Weakley]

##### Eupatorium rotundifolium

L.

###### Distribution

Wet pine savannas (WLPS), adjacent roadsides.

###### Notes

Rare. Aug–Oct. Thornhill 759 (NCSC). Specimens seen in the vicinity: Sandy Run [Hancock]: Taggart SARU 349 (WNC!). [= Eupatorium rotundifolium L. var. rotundifolium sensu RAB, FNA; = Weakley]

##### Eurybia compacta

G.L. Nesom

###### Distribution

Wet pine savannas (WLPS, VWLPS).

###### Notes

Infrequent. Late Jul–Oct. Thornhill 50, 969, 1522 (NCSC). Specimens seen in the vicinity: Sandy Run [Hancock]: Taggart SARU 448 (WNC!). [= *Aster gracilis* Nutt. sensu RAB; = FNA, Weakley]

##### Eurybia paludosa

(Aiton) G.L. Nesom

###### Distribution

Wet pine savannas (SPS-T, WLPS, VWLPS).

###### Notes

Frequent. Jul–Oct. Thornhill 102, 924, 969, 1041, 1209, 1522 (NCSC). Specimens seen in the vicinity: Sandy Run [Hancock]: Taggart SARU 347 (WNC!); Sandy Run [Neck]: Wilbur 57664 (DUKE!; as *Aster paludosus*). [= *Aster paludosus* Aiton sensu RAB; = FNA, Weakley]

##### Euthamia caroliniana

(L.) Greene ex Porter & Britton

###### Distribution

Wet pine flatwoods (WPF-T), wet pine savannas (SPS-RF, WLPS, VWLPS).

###### Notes

Occasional. Sep–Dec. Thornhill 1123, 1129, 1144 (NCSC). Specimens seen in the vicinity: Sandy Run [Hancock]: Taggart SARU 480 (WNC!); Sandy Run [Neck]: Wilbur 57634 (DUKE!; as *Euthamia minor*). [> *Solidago microcephala* (Nutt.) Bush, >< *Solidago tenuifolia* Pursh sensu RAB; = FNA, Weakley]

##### Helenium autumnale

L.

###### Distribution

Wet pine savannas (WLPS, VWLPS), adjacent roadsides.

###### Notes

Infrequent. Sep–Oct. Thornhill 1029, 1087, 1550 (NCSC). Specimens seen in the vicinity: Sandy Run [Hancock]: Taggart SARU 460 (WNC!); Sandy Run [Neck]: Wilbur 57631 (as *Helenium pinnatifidum*), 57674 (DUKE!). [= RAB, FNA, Weakley]

##### Helenium pinnatifidum

(Schwein. ex Nutt.) Rydb.

###### Ecological interactions

####### Conservation status

SR-P; S2, G4.

###### Distribution

Pine savannas and adjacent ditches.

###### Notes

Apr–May. Reported from Sandy Run by LeBlond and Weakley (1991), but no specimens have been seen in Shaken Creek Preserve by the senior author. [= RAB, FNA, Weakley]

##### Helianthus angustifolius

L.

###### Distribution

Wet pine savannas (WLPS, VWLPS).

###### Notes

Occasional. (Jul–)Sep–Oct(–frost). Thornhill 51, 1140, 1141 (NCSC). Specimens seen in the vicinity: Sandy Run [Hancock]: Taggart SARU 405 (WNC!); Sandy Run [Neck]: Wilbur 57668 (DUKE!). [= RAB, FNA, Weakley]

##### Helianthus heterophyllus

Nutt.

###### Distribution

Depressions in wet pine flatwoods (WPF-T), wet pine savannas (WLPS, VWLPS).

###### Notes

Infrequent. Aug–Oct. Thornhill 989, 1090 (NCSC). Specimens seen in the vicinity: Sandy Run [Hancock]: Taggart SARU 459 (WNC!). [= RAB, FNA, Weakley]

##### Hieracium gronovii

L.

###### Distribution

Mesic pine savannas (MPS-CP).

###### Notes

Rare. Jul–Nov. Thornhill 1549 (NCSC). Specimens seen in the vicinity: Sandy Run [O’Berry]: Taggart SARU 397 (WNC!). [= RAB, FNA, Weakley]

##### Ionactis linariifolia

(L.) Greene

###### Distribution

Mesic pine savannas (MPS-CP), wet pine flatwoods (WPF-T).

###### Notes

Infrequent. Aug–Nov. Thornhill 1186 (NCSC). Specimens seen in the vicinity: Sandy Run [Hancock]: Taggart SARU 492 (WNC!). [= *Aster linariifolius* L. sensu RAB; = FNA, Weakley]

##### Liatris pilosa

(Aiton) Willd.

###### Distribution

Mesic pine savannas (MPS-CP), wet pine flatwoods (WPF-T).

###### Notes

Infrequent. (Aug–)Sep–Oct(–Nov). Thornhill 52, 1176, 1545 (NCSC). [< *Liatris graminifolia* Willd. sensu RAB; = FNA, Weakley]

##### Liatris spicata var. resinosa

(Nutt.) Gaiser

###### Distribution

Wet pine savannas (SPS-RF, WLPS, VWLPS).

###### Notes

Occasional. (Jul–)Aug–Oct(–Nov). Thornhill 829, 874, 956 (NCSC). Specimens seen in the vicinity: Sandy Run [Hancock]: Taggart SARU 410 (WNC!). [= RAB, FNA, Weakley]

##### Marshallia graminifolia

(Walter) Small

###### Distribution

Wet pine savannas (SPS-T, SPS-RF, WLPS, VWLPS).

###### Notes

Occasional. Late Jul–mid Oct. Thornhill 5, 701, 741, 807 (NCSC). Specimens seen in the vicinity: Sandy Run [Patterson]: Taggart SARU 362 (WNC!). [= RAB; < *Marshallia graminifolia* (Walter) Small sensu FNA; = Weakley]

##### Mikania scandens

(L.) Willd.

###### Distribution

Wet pine savannas (VWLPS), adjacent swamps.

###### Notes

Infrequent. Jul–Oct. Thornhill 824, 834 (NCSC). Specimens seen in the vicinity: Sandy Run [Haw’s Run]: Taggart SARU 312 (WNC!). [= RAB, FNA, Weakley]

##### Packera paupercula

(Michx.) Á. Löve & D. Löve

###### Ecological interactions

####### Conservation status

SR-T; S1, G2G3 (as *Packera crawfordii* (Britton) A.M. Mahoney & R.R. Kowal).

###### Distribution

Pine savannas.

###### Notes

Apr–May. Not seen in Shaken Creek Preserve by the senior author. Specimens seen in the vicinity: Sandy Run: LeBlond 6409 (NCU!; as *Packera crawfordii*), Taggart SARU 10 (WNC!; as *Packera crawfordii*/*Packera paupercula*), and Weakley 7216 (NCU!; as *Packera crawfordii*). Whether the entity treated as *Packera crawfordii* deserves recognition as distinct (either varietally or specifically) from *Packera paupercula* s.l. is still unclear. [= *Senecio pauperculus* Michx. sensu RAB; = FNA; > *Packera crawfordii* (Britton) A.M. Mahoney & R.R. Kowal, Packera paupercula (Michx.) Á. Löve & D. Löve var. paupercula sensu Weakley]

##### Pityopsis graminifolia var. latifolia

(Fernald) Semple & F.D. Bowers

###### Distribution

Mesic pine savannas (MPS-CP), wet pine flatwoods (WPF-T), wet pine savannas (SPS-RF, WLPS).

###### Notes

Occasional. Jun–Oct. Thornhill 761, 922, 985, 1145 (NCSC). Specimens seen in the vicinity: Sandy Run [Patterson]: Taggart SARU 363 (WNC!). [> Heterotheca nervosa (Willd.) Shinners var. nervosa (Small) Shinners ex Ahles, *Heterotheca correllii* (Fernald) H.E. Ahles sensu RAB; = FNA, Weakley]

##### Pluchea baccharis

(Mill.) Pruski

###### Distribution

Wet pine savannas (VWLPS).

###### Notes

Jun–Jul. Reported from Sandy Run [O’Berry Tract] by LeBlond (2000), but no specimens have been seen in Shaken Creek Preserve by the senior author. [= *Pluchea rosea* R.K. Godfrey sensu RAB; = FNA, Weakley]

##### Pluchea foetida

(L.) DC.

###### Distribution

Wet pine savannas (VWLPS), borrow pits.

###### Notes

Infrequent. Aug–Oct. Thornhill 672, 686, 955 (NCSC). Specimens seen in the vicinity: Sandy Run [Hancock]: Taggart SARU 354 (WNC!), Wilbur 53659 (DUKE!). [= RAB, FNA, Weakley]

##### Prenanthes autumnalis

Walter

###### Distribution

Wet pine savannas (WLPS, VWLPS).

###### Notes

Rare. Sep–Nov. Thornhill 1174, 1232 (NCSC). Specimens seen in the vicinity: Sandy Run [Hancock]: Taggart SARU 549 (WNC!). [= RAB, FNA, Weakley]

##### Pseudognaphalium obtusifolium

(L.) Hilliard & Burtt

###### Distribution

Mesic pine savannas (MPS-CP), adjacent roadsides and disturbed areas.

###### Notes

Infrequent. Aug–Oct. Thornhill 1530 (NCSC). Specimens seen in the vicinity: Sandy Run [RMK]: Taggart SARU 566 (WNC!). [= *Gnaphalium obtusifolium* L. sensu RAB; = FNA, Weakley]

##### Pterocaulon pycnostachyum

(Michx.) Elliott

###### Distribution

Pine/scrub oak sandhills (PSOS-MT), mesic pine savannas (MPS-CP).

###### Notes

Occasional. May–Jun. Thornhill 387, 1296 (NCSC). Specimens seen in the vicinity: Sandy Run [Neck]: Levy s.n. (DUKE!), Taggart SARU 278 (WNC!), Wilbur 53637, 55274 (DUKE!). [= RAB, FNA, Weakley]

##### Pyrrhopappus carolinianus

(Walter) DC.

###### Distribution

Roadside margins of wet pine savannas (VWLPS) and roadsides.

###### Notes

Occasional. Mar–Jun. Thornhill 481, 657 (NCSC). Specimens seen in the vicinity: Sandy Run [Hancock]: Taggart SARU 63 (WNC!). [> Pyrrhopappus carolinianus (Walter) DC. var. carolinianus sensu RAB; = FNA, Weakley]

##### Sericocarpus linifolius

(L.) Britton, Sterns & Poggenb.

###### Distribution

Mesic pine savannas (MPS-CP), wet pine savannas (SPS-RF).

###### Notes

Rare. Jun–Jul. Thornhill 1003 (NCSC). Specimens seen in the vicinity: Sandy Run [Hancock]: Taggart SARU 264 (WNC!; as S. tortifolius). [= *Aster solidagineus* Michx. sensu RAB; = FNA, Weakley]

##### Silphium compositum var. compositum

Michx.

###### Distribution

Pine savannas.

###### Notes

May–Sep. Not seen in Shaken Creek Preserve by the senior author. Specimens seen in the vicinity: Sandy Run [Hancock]: Taggart SARU 470 (WNC!). [< Silphium compositum Michx. var. compositum sensu RAB; < *Silphium compositum* Michx. sensu FNA; = Weakley]

##### Solidago fistulosa

Mill.

###### Distribution

Wet pine savannas (VWLPS).

###### Notes

Aug–Nov. Reported from Sandy Run [O’Berry Tract] by LeBlond (2000), but no specimens have been seen in Shaken Creek Preserve by the senior author. [= RAB, FNA, Weakley]

##### Solidago gracillima

Torr. & A. Gray

###### Ecological interactions

####### Conservation status

W1; S3, G4?.

###### Distribution

Wet pine savannas (WLPS, VWLPS).

###### Notes

Occasional. Aug–Oct. Thornhill 942, 1078, 1130, 1236 (NCSC). [= RAB; = Solidago stricta Aiton ssp. gracillima (Torr. & A. Gray) Semple sensu FNA; = Weakley]

##### Solidago odora

Aiton

###### Distribution

Pine savannas.

###### Notes

Jul–Oct. Not seen in Shaken Creek Preserve by the senior author. Specimens seen in the vicinity: Sandy Run [RMK]: Taggart SARU 476 (WNC!). [= RAB; = Solidago odora Aiton ssp. odora sensu FNA; = Weakley]

##### Solidago pinetorum

Small

###### Distribution

Pine savannas.

###### Notes

Jul–Sep. Not seen in Shaken Creek Preserve by the senior author. Specimens seen in the vicinity: Sandy Run [Hancock]: Taggart SARU 496 (WNC!). [= RAB, FNA, Weakley]

##### Solidago puberula var. pulverulenta

(Nutt.) Chapm.

###### Distribution

Wet pine flatwoods (WPF-T), wet pine savannas (WLPS).

###### Notes

Infrequent. Sep–Oct. Thornhill 1038 (NCSC). Specimens seen in the vicinity: Sandy Run [Hancock]: Taggart SARU 354 (WNC!). [= RAB; = Solidago puberula Nutt. var. pulverulenta (Nutt.) Chapm. sensu FNA; = Weakley]

##### Solidago pulchra

Small

###### Ecological interactions

####### Conservation status

W1; S3, G3.

###### Distribution

Wet pine savannas (SPS-T, SPS-RF, WLPS, VWLPS).

###### Notes

Frequent. Jul–Sep. Thornhill 39, 40, 909, 1036, 1495 (NCSC). Specimens seen in the vicinity: Sandy Run: Taggart SARU 401 (WNC!), Wilbur 57672 (DUKE!). [< *Solidago stricta* Aiton sensu RAB; = FNA, Weakley]

##### Solidago stricta

Aiton

###### Distribution

Wet pine savannas (SPS-RF, WLPS).

###### Notes

Infrequent. Late Aug–Oct. Thornhill 1136 (NCSC). Specimens seen in the vicinity: Sandy Run [O’Berry]: Taggart SARU 512 (WNC!). [< RAB; = Solidago stricta Aiton spp. stricta sensu FNA; = Weakley]

##### Symphyotrichum dumosum

(L.) G.L. Nesom

###### Distribution

Wet pine savannas (SPS-RF, WLPS, VWLPS).

###### Notes

Occasional. Late Aug–Oct. Thornhill 46, 1131, 1170, 1184, 1210, 1211, 1212 (NCSC). Specimens seen in the vicinity: Sandy Run [Hancock]: Taggart SARU 391 (WNC!); Sandy Run [Neck]: Wilbur 57673 (DUKE!; as *Aster dumosus*). [= *Aster dumosus* L. sensu RAB; = FNA; > *Symphyotrichum dumosum* (L.) G.L. Nesom various varieties sensu Weakley]

##### Symphyotrichum lateriflorum

(L.) Á. Löve & D. Löve

###### Distribution

In a wide variety of dry to moist habitats.

###### Notes

Sep–Nov. Not seen in Shaken Creek Preserve by the senior author. Specimens seen in the vicinity: Sandy Run [Neck]: LeBlond 2590 (NCU!; as Symphyotrichum lateriflorum var. lateriflorum). [< *Aster lateriflorus* (L.) Britton sensu RAB; = FNA; < *Symphyotrichum lateriflorum* (L.) Á. Löve & D. Löve various varieties sensu Weakley]

##### Symphyotrichum novi-belgii var. elodes

(Torr. & A. Gray) G.L. Nesom

###### Distribution

Pine savannas and marshes.

###### Notes

Late Sep–Nov. Reported from Sandy Run [Haw’s Run] by LeBlond (1999), but no specimens have been seen in Shaken Creek Preserve by the senior author. [< *Aster novi-belgii* L. sensu RAB; = FNA, Weakley]

##### Symphyotrichum pilosum var. pilosum

(Willd.) G.L. Nesom

###### Distribution

Woodland borders, old fields, disturbed areas.

###### Notes

Sep–Nov. Not seen in Shaken Creek Preserve by the senior author. Specimens seen in the vicinity: Sandy Run [Neck]: Wilbur 57641, 57643 (DUKE!; as *Aster pilosus* Willd.). [< *Aster pilosus* Willd. sensu RAB; = FNA, Weakley]

##### Symphyotrichum walteri

(Alexander) G.L. Nesom

###### Distribution

Mesic pine savannas (MPS-CP), wet pine flatwoods (WPF-T).

###### Notes

Infrequent. Oct–Dec. Thornhill 971 (NCSC). Specimens seen in the vicinity: Sandy Run [Hancock]: Taggart SARU 552 (WNC!). [= *Aster squarrosus* Walter sensu RAB; = FNA, Weakley]

##### Trilisa odoratissima

(J.F. Gmel.) Cass.

###### Distribution

Pine/scrub oak sandhills (PSOS-MT), wet pine flatwoods (WPF-T), wet pine savannas (SPS-T, SPS-RF).

###### Notes

Frequent. Late Jul–Oct; Sep–Nov. Thornhill 1518 (NCSC). Specimens seen in the vicinity: Sandy Run [Hancock]: Taggart SARU 497 (WNC!; as *Carphephorus odoratissimus*). [< *Trilisa odoratissima* (J.F. Gmel.) Cass. sensu RAB; = Carphephorus odoratissimus (J. F. Gmel.) H. J.-C. Hebert var. odoratissimus sensu FNA; = Weakley]

##### Trilisa paniculata

(J.F. Gmel.) Cass.

###### Distribution

Wet pine flatwoods (WPF-T), wet pine savannas (SPS-RF).

###### Notes

Infrequent. Aug–Oct; Sep–Nov. Thornhill 1540 (NCSC). Specimens seen in the vicinity: Sandy Run [Neck]: Taggart SARU 530 (WNC!; as *Carphephorus paniculatus*). [= RAB; = *Carphephorus paniculatus* (J.F. Gmel.) H.J.-C. Hebert sensu FNA; = Weakley]

##### Vernonia angustifolia

Michx.

###### Distribution

Sandhills.

###### Notes

Late Jun–early Sep; Sep–Oct. Reported from Sandy Run [Neck] by LeBlond and Weakley (1991), but no specimens have been seen in Shaken Creek Preserve by the senior author. [> Vernonia angustifolia Michx. var. angustifolia, Vernonia angustifolia Michx. var. scabberima (Nutt.) S.B. Jones & W.Z. Faust sensu RAB; = FNA; > *Vernonia angustifolia* Michx. various varieties sensu Weakley]

##### Vernonia noveboracensis

(L.) Michx.

###### Distribution

Pine savannas.

###### Notes

Jul–Sep; Aug–Oct. Not seen in Shaken Creek Preserve by the senior author. Specimens seen in the vicinity: Sandy Run [Hancock]: Taggart SARU 355 (WNC!). [= RAB, FNA, Weakley]

#### Bignoniaceae

##### Bignonia capreolata

L.

###### Distribution

Margins of wet pine savannas (WLPS) and adjacent swamps.

###### Notes

Infrequent. Apr–May; Jul–Aug. Thornhill 1148 (NCSC). Specimens seen in the vicinity: Sandy Run [Patterson]: Taggart SARU 323 (WNC!). [= *Anisostichus capreolata* (L.) Bureau sensu RAB; = Weakley]

##### Campsis radicans

(L.) Seem. ex Bureau

###### Distribution

Margins of wet pine flatwoods (WPF-T) and adjacent swamps.

###### Notes

Infrequent. Jun–Jul; Sep–Oct. Thornhill 1482 (NCSC). Specimens seen in the vicinity: Sandy Run [Hancock]: Taggart SARU 198 (WNC!). [= RAB, Weakley]

#### Campanulaceae

##### Lobelia canbyi

A. Gray

###### Distribution

Wet pine flatwoods (WPF-T), wet pine savannas (SPS-T, SPS-RF, WLPS, VWLPS).

###### Notes

Frequent. Jul–Nov. Thornhill 34, 49, 740, 804, 840, 873 (NCSC). Specimens seen in the vicinity: Sandy Run [Hancock]: Taggart SARU 446 (WNC!). [= RAB, Weakley]

##### Lobelia glandulosa

Walter

###### Distribution

Wet pine savannas (SPS-T, WLPS, VWLPS).

###### Notes

Occasional. Sep–Oct. Thornhill 35, 921 (NCSC). Specimens seen in the vicinity: Sandy Run [Hancock]: Taggart SARU 478 (WNC!). [= RAB, Weakley]

##### Lobelia nuttallii

Schult.

###### Distribution

Wet pine flatwoods (WPF-T), wet pine savannas (SPS-T, SPS-RF, WLPS, VWLPS).

###### Notes

Frequent. May–Nov. Thornhill 3, 337, 427, 479, 488, 526 (NCSC). Specimens seen in the vicinity: Sandy Run [Hancock]: Taggart SARU 152 (WNC!); Sandy Run [Neck]: Wilbur 53644, 55299 (DUKE!). [= RAB, Weakley]

#### Caprifoliaceae

##### Lonicera sempervirens

L.

###### Distribution

Dry forests and woodlands.

###### Notes

Mar–Jul(–Nov); Jul–Sep. Not seen in Shaken Creek Preserve by the senior author. Specimens seen in the vicinity: Sandy Run [Neck]: Wilbur 53654 (DUKE!). [= RAB, Weakley]

#### Cistaceae

##### Lechea minor

L.

###### Distribution

Mesic pine savannas (MPS-CP).

###### Notes

Rare. Jul–Aug; Aug–Oct. Thornhill 1551 (NCSC). [= RAB, Weakley]

##### Lechea pulchella var. ramosissima

(Hodgdon) Sorrie & Weakley

###### Distribution

Wet pine savannas (SPS-RF, WLPS), adjacent roadsides.

###### Notes

Infrequent. Jun–Aug; Aug–Oct. Thornhill 623, 751 (NCSC). Specimens seen in the vicinity: Sandy Run [Patterson]: Taggart SARU 364 (WNC!). [< *Lechea leggettii* Britton & Hollick sensu RAB; = Weakley]

#### Clethraceae

##### Clethra alnifolia

L.

###### Distribution

Wet pine flatwoods (WPF-T), wet pine savannas (SPS-T, SPS-RF, WLPS, VWLPS).

###### Notes

Frequent. Jun–Jul; Sep–Oct. Thornhill 585, 613, 670, 715 (NCSC). Specimens seen in the vicinity: Sandy Run [Hancock]: Taggart SARU 258 (WNC!); Sandy Run [Neck]: Wilbur 53648, 53702 (DUKE!). [= Clethra alnifolia L. var. alnifolia sensu RAB; = FNA, Weakley]

#### Convolvulaceae

##### Cuscuta gronovii

Willd. ex Roem. & Schult.

###### Distribution

Margins of wet pine savannas (VWLPS) and adjacent swamps.

###### Notes

Rare. Grows on a wide variety of hosts, both herbaceous and woody (Weakley 2012). Aug–Oct. Thornhill 1042, 1085 (NCSC). [= RAB, Weakley]

##### Cuscuta pentagona

Engelm.

###### Distribution

Pine savannas.

###### Notes

Usually found on low-growing herbaceous hosts (Radford et al. 1968). May–Nov. Not seen in Shaken Creek Preserve by the senior author. Specimens seen in the vicinity: Sandy Run [Haw’s Run]: Taggart SARU 637 (WNC!). [= RAB, Weakley]

#### Cornaceae

##### Cornus stricta

Lam.

###### Distribution

Wet pine flatwoods (WPF-T).

###### Notes

Rare. Apr–May; Jul–Aug. Thornhill 1491 (NCSC). Specimens seen in the vicinity: Sandy Run [Neck]: Wilbur 53653, 53655, 67087 (DUKE!); Sandy Run [Patterson]: Taggart SARU 211 (WNC!). [= RAB, Weakley]

#### Cyrillaceae

##### Cyrilla racemiflora

L.

###### Distribution

Wet pine flatwoods (WPF-T), wet pine savannas (SPS-T, SPS-RF, WLPS, VWLPS).

###### Notes

Frequent. May–Jul; Sep–Oct. Thornhill 430, 448, 540, 614 (NCSC). Specimens seen in the vicinity: Sandy Run [Neck]: Wilbur 53681 (DUKE!); Sandy Run [RMK]: Taggart SARU 229 (WNC!). [= RAB, FNA, Weakley]

#### Diapensiaceae

##### Pyxidanthera barbulata

Michx.

###### Distribution

Wet pine flatwoods (WPF-T), wet pine savannas (SPS-T, SPS-RF).

###### Notes

Frequent. Mar–Apr; May–Jun. Thornhill 81, 83 (NCSC). Specimens seen in the vicinity: Sandy Run [Patterson]: Taggart SARU 19 (WNC!). [= Pyxidanthera barbulata Michx. var. barbulata sensu RAB; = FNA, Weakley]

#### Droseraceae

##### Dionaea muscipula

J. Ellis

###### Ecological interactions

####### Conservation status

SC-V, FSC; S3, G3.

###### Distribution

Depressions in wet pine flatwoods (WPF-T), wet pine savannas (SPS-T, SPS-RF, WLPS, VWLPS).

###### Notes

Occasional. May–Jun; Jun–Jul. Thornhill 314, 381, 383 (NCSC). Specimens seen in the vicinity: Sandy Run: Bell 17110 (NCU!), Taggart SARU 114 (WNC!), Wilbur 55289 (DUKE!). [= RAB, Weakley]

##### Drosera brevifolia

Pursh

###### Distribution

Wet pine flatwoods (WPF-T), wet pine savannas (SPS-T, SPS-RF, WLPS, VWLPS).

###### Notes

Abundant. Apr–May. Thornhill 104, 159 (NCSC). Specimens seen in the vicinity: Sandy Run [Hancock]: Taggart SARU 82 (WNC!). [=*Drosera leucantha* Shinners sensu RAB; = Weakley]

##### Drosera capillaris

Poir.

###### Distribution

Wet pine flatwoods (WPF-T), wet pine savannas (SPS-T, SPS-RF, WLPS, VWLPS).

###### Notes

Frequent. May–Aug. Thornhill 292, 371, 411 (NCSC). Specimens seen in the vicinity: Sandy Run [Hancock]: Taggart SARU 172 (WNC!). [= RAB, Weakley]

##### Drosera intermedia

Hayne

###### Distribution

Wet pine savannas (SPS-T, SPS-RF, WLPS), borrow pits, ditches.

###### Notes

Frequent. Jul–Sep. Thornhill 29, 161, 666 (NCSC). Specimens seen in the vicinity: Sandy Run [Hancock]: Taggart SARU 144 (WNC!); Sandy Run [Neck]: Wilbur 55301 (DUKE!). [= RAB, Weakley]

#### Ebenaceae

##### Diospyros virginiana

L.

###### Distribution

Pine/scrub oak sandhills (PSOS-MT), mesic pine savannas (MPS-CP), wet pine flatwoods (WPF-T), wet pine savannas (SPS-T, SPS-RF, WLPS, VWLPS).

###### Notes

Frequent. May–Jun; Sep–Dec. Thornhill 283, 709 (NCSC). Specimens seen in the vicinity: Sandy Run [Patterson]: Taggart SARU 213 (WNC!). [= RAB, FNA, Weakley]

#### Ericaceae

##### Chamaedaphne calyculata

(L.) Moench

###### Distribution

Pine savannas.

###### Notes

Mar–Apr; Jun–Oct. Not seen in Shaken Creek Preserve by the senior author. Specimens seen in the vicinity: Holly Shelter: Fox 158 (NCSC!); Sandy Run [Hancock]: Taggart SARU 15 (WNC!). [= *Cassandra calyculata* (L.) D. Don sensu RAB; = FNA, Weakley]

##### Eubotrys racemosa

(L.) Nutt.

###### Distribution

Wet pine flatwoods (WPF-T).

###### Notes

Occasional. Late Mar–early Jun; Sep–Oct. Thornhill 1291, 1471, 1511, 1546 (NCSC). Specimens seen in the vicinity: Sandy Run [Neck]: Wilbur 55259 (DUKE!; as *Leucothoe racemosa*); Sandy Run [O’Berry]:Taggart SARU 93 (WNC!). [= *Leucothoe racemosa* (L.) A. Gray sensu RAB; = FNA, Weakley]

##### Gaylussacia dumosa

(Andrews) Torr. & A. Gray

###### Distribution

Mesic pine savannas (MPS-CP), wet pine flatwoods (WPF-T), wet pine savannas (SPS-T, SPS-RF, WLPS, VWLPS).

###### Notes

Abundant. Mar–Jun; Jun–Oct. Thornhill 165, 197, 210, 214, 228, 233, 258, 592, 806 (NCSC). Specimens seen in the vicinity: Sandy Run [Hancock]: Taggart SARU 113 (WNC!). [< RAB; = FNA, Weakley]

##### Gaylussacia frondosa

(L.) Torr. & A. Gray ex Torr.

###### Distribution

Pine/scrub oak sandhills (PSOS-MT), mesic pine savannas (MPS-CP), wet pine flatwoods (WPF-T), wet pine savannas (SPS-T, SPS-RF, WLPS, VWLPS).

###### Notes

Abundant. Late Mar–May; Jun–Aug. Thornhill 120, 146, 204, 212, 215, 229, 234, 290 (NCSC). Specimens seen in the vicinity: Sandy Run [Neck]: Wilbur 53650, 55288, 55291, 63768, 63781, 67097 (DUKE!); Sandy Run [Patterson]: Taggart SARU 106 (WNC!). [= Gaylussacia frondosa var. frondosa sensu RAB; = FNA, Weakley]

##### Kalmia carolina

Small

###### Distribution

Wet pine flatwoods (WPF-T), wet pine savannas (SPS-T, SPS-RF).

###### Notes

Frequent. Apr–May(–Sep); Sep–Oct. Thornhill 164, 182 (NCSC). Specimens seen in the vicinity: Sandy Run [O’Berry]: Taggart SARU 68 (WNC!), Weakley 7218 (NCU!). [= Kalmia angustifolia L. var. caroliniana (Small) Fernald sensu RAB, FNA; = Weakley]

##### Leucothoe axillaris

(Lam.) D. Don

###### Distribution

Pine savannas.

###### Notes

Late Mar–May; Sep–Oct. Not seen in Shaken Creek Preserve by the senior author. Specimens seen in the vicinity: Sandy Run [O’Berry]: Taggart SARU 669 (WNC!). [= Leucothoe axillaris (Lam.) D. Don var. axillaris sensu RAB; = FNA, Weakley]

##### Lyonia ligustrina

(L.) DC.

###### Distribution

Wet pine flatwoods (WPF-T), wet pine savannas (SPS-T, SPS-RF, WLPS, VWLPS).

###### Notes

Frequent. Late Apr–Jul; Sep–Oct. If one chooses to recognize varieties, the material collected by the author would generally be referable to var. *Lyonialigustrinafoliosiflora*, the more common variety on the North Carolina Coastal Plain. Thornhill 417, 591, 911, 1526 (NCSC). Specimens seen in the vicinity: Sandy Run [Neck]: Wilbur 53649, 53704, 55305, 55320 (DUKE!); Sandy Run [O’Berry]: Taggart SARU 92 (WNC!; as var. *Lyonialigustrinafoliosiflora*), Taggart SARU 226 (WNC!; as var. *Lyonialigustrinaligustrina*). [= RAB; > Lyonia ligustrina var. foliosiflora (Michx.) Fernald, Lyonia ligustrina var. ligustrina sensu FNA, Weakley]

##### Lyonia lucida

(Lam.) K. Koch

###### Distribution

Wet pine flatwoods (WPF-T), wet pine savannas (SPS-T, SPS-RF, WLPS, VWLPS).

###### Notes

Frequent. Apr–early Jun; Sep–Oct. Thornhill 116, 133, 167, 180 (NCSC). Specimens seen in the vicinity: Holly Shelter: Fox 160 (NCSC!); Sandy Run [Neck]: Wilbur 53667, 63770, 63783, 63784, 63785, 67088 (DUKE!); Sandy Run [O’Berry]: Taggart SARU 71 (WNC!), Weakley 7221 (NCU!). [= RAB, FNA, Weakley]

##### Lyonia mariana

(L.) D. Don

###### Distribution

Wet pine flatwoods (WPF-T), wet pine savannas (SPS-T, SPS-RF, WLPS, VWLPS).

###### Notes

Frequent. Apr–May; Sep–Oct. Thornhill 118, 178, 236 (NCSC). Specimens seen in the vicinity: Sandy Run [Hancock]: Taggart SARU 96 (WNC!); Sandy Run [Neck]: Wilbur 55308, 63766, 67096 (DUKE!). [= RAB, FNA, Weakley]

##### Rhododendron atlanticum

(Ashe) Rehder

###### Distribution

Wet pine flatwoods (WPF-T), wet pine savannas (SPS-T, SPS-RF).

###### Notes

Occasional. Apr–May(–later). Thornhill 113, 179 (NCSC). Specimens seen in the vicinity: Sandy Run [Hancock]: Taggart SARU 62 (WNC!); Sandy Run [Neck]: Levy s.n. (DUKE!), Wilbur 63767, 67098 (DUKE!). [= RAB, FNA, Weakley]

##### Rhododendron viscosum

(L.) Torr.

###### Distribution

Wet pine flatwoods (WPF-T), wet pine savannas (SPS-T, SPS-RF).

###### Notes

Occasional. Late May–Jul; Jul–Oct. Thornhill 225, 266, 308 (NCSC). Specimens seen in the vicinity: Sandy Run [Neck]: LeBlond 4972 (NCU; as Rhododendron viscosum var. serrulatum); Sandy Run [Patterson]: Taggart SARU 596 (WNC!). [> Rhododendron viscosum (L.) Torr. var. serrulatum (Small) Ahles, Rhododendron viscosum (L.) Torr. var. viscosum sensu RAB, Weakley; = FNA]

##### Vaccinium arboreum

Marshall

###### Distribution

Mesic pine savannas.

###### Notes

Late Apr–Jun; Sep–Oct. Not seen in Shaken Creek Preserve by the senior author. Specimens seen in the vicinity: Sandy Run [RMK]: Taggart SARU 219 (WNC!). [= RAB, FNA, Weakley]

##### Vaccinium crassifolium

Andrews

###### Distribution

Wet pine flatwoods (WPF-T), wet pine savannas (SPS-T, SPS-RF, WLPS, VWLPS).

###### Notes

Abundant. Apr–May; Jun–Jul. Thornhill 117, 153, 160, 185 (NCSC). Specimens seen in the vicinity: Sandy Run [Hancock]: Taggart SARU 76 (WNC!). [= RAB; < FNA; = Weakley]

##### Vaccinium formosum

Andrews

###### Distribution

Wet pine flatwoods (WPF-T), wet pine savannas (SPS-T, SPS-RF, WLPS).

###### Notes

Occasional. Late Feb–May; Jun–Aug. Thornhill 147, 150, 166, 173, 183, 264, 303, 305 (NCSC). Specimens seen in the vicinity: Sandy Run [Hancock]: Taggart SARU 23 (WNC!). [< *Vaccinium corymbosum* L. sensu RAB, FNA; = Weakley]

##### Vaccinium fuscatum

Aiton

###### Distribution

Wet pine flatwoods (WPF-T), wet pine savannas (SPS-T, SPS-RF, WLPS, VWLPS).

###### Notes

Frequent. Late Feb–May; Jun–Aug. Thornhill 78, 79, 82, 92, 101, 267, 277, 302, 763 (NCSC). Specimens seen in the vicinity: Sandy Run [Patterson]: Taggart SARU 216 (WNC!). [= *Vaccinium atrococcum* (Gray) A. Heller sensu RAB; < *Vaccinium corymbosum* L. sensu FNA; = Weakley]

##### Vaccinium stamineum

L.

###### Distribution

Wet pine savannas (SPS-RF).

###### Notes

Apr–Jun; Aug–Oct. Reported from Shaken Creek Preserve by LeBlond (2000), but no specimens have been seen by the senior author. [> Vaccinium stamineum L. var. stamineum sensu RAB; = FNA; > *Vaccinium stamineum* L. various varieties sensu Weakley]

##### Vaccinium tenellum

Aiton

###### Distribution

Pine/scrub oak sandhills (PSOS-MT), mesic pine savannas (MPS-CP), wet pine flatwoods (WPF-T), wet pine savannas (SPS-T, SPS-RF, WLPS, VWLPS).

###### Notes

Abundant. Late Mar–early May; Jun–Jul. Thornhill 145, 184, 755, 1222, 1423, 1424 (NCSC). Specimens seen in the vicinity: Holly Shelter: Fox 161 (NCSC!); Sandy Run [Hancock]: Ahles 28232 (NCU!); Sandy Run [Neck]: Wilbur 63769, 63775 (DUKE!); Sandy Run [Patterson]: Taggart SARU 73 (WNC!). [= RAB, FNA, Weakley]

##### Zenobia pulverulenta

(W. Bartram ex Willd.) Pollard

###### Distribution

Wet pine flatwoods (WPF-T), wet pine savannas (SPS-T, SPS-RF).

###### Notes

Frequent. Apr–Jun; Sep–Oct. Thornhill 231, 259, 307, 508 (NCSC). Specimens seen in the vicinity: Sandy Run [Hancock]: Taggart SARU 181 (WNC!). [= RAB, FNA, Weakley]

#### Euphorbiaceae

##### Cnidoscolus stimulosus

(Michx.) Engelm. & A. Gray

###### Distribution

Pine/scrub oak sandhills (PSOS-MT), mesic pine savannas (MPS-CP), wet pine flatwoods (WPF-T).

###### Notes

Occasional. Late Mar–Aug; May–Sep. Thornhill 1275 (NCSC). Specimens seen in the vicinity: Sandy Run [RMK]: Taggart SARU 220 (WNC!). [= RAB, Weakley]

##### Euphorbia ipecacuanhae

L.

###### Distribution

Pine/scrub oak sandhills (PSOS-MT), mesic pine savannas (MPS-CP).

###### Notes

Infrequent. Feb–May. Thornhill 1418 (NCSC). Specimens seen in the vicinity: Sandy Run [Neck]: Wilbur 55277 (DUKE!). [= RAB, Weakley]

##### Tragia urens

L.

###### Distribution

Pine/scrub oak sandhills (PSOS-MT).

###### Notes

Rare. May–Oct. Thornhill 1419 (NCSC). Specimens seen in the vicinity: Sandy Run [Hancock]: Ahles 28231A (NCU!; one duplicate specimen labeled as *Tragia linearifolia* Elliott). [= RAB, Weakley]

#### Fabaceae

##### Amorpha georgiana

Wilbur

###### Ecological interactions

####### Conservation status

State E, FSC; S2, G3.

###### Distribution

Mesic pine savannas (MPS-CP).

###### Notes

Rare. Apr–Jun; Jul–Oct. Thornhill 1073, 1239 (NCSC); Thornhill 1177 (NCU). [< RAB; = Weakley]

##### Amorpha herbacea var. herbacea

Walter

###### Distribution

Mesic pine savannas (MPS-CP).

###### Notes

Rare. May–Jul; Jul–Oct. Thornhill 1541 (NCSC). Specimens seen in the vicinity: Sandy Run [Neck]: Wilbur 55279 (DUKE!). [< *Amorpha herbacea* Walter sensu RAB; = Weakley]

##### Apios americana

Medik.

###### Distribution

Wet pine flatwoods (WPF-T).

###### Notes

Rare. Jun–Aug; Jul–Sep. Thornhill 1179 (NCSC). [= RAB, Weakley]

##### Baptisia cinerea

(Raf.) Fernald & B.G. Schub.

###### Distribution

Pine savannas.

###### Notes

Late Apr–Jun; Jun–Jul. Not seen in Shaken Creek Preserve by the senior author. Specimens seen in the vicinity: Sandy Run [Hancock]: Taggart SARU 129 (WNC!). [= RAB, Weakley]

##### Baptisia tinctoria

(L.) Vent.

###### Distribution

Pine savannas and wet pine flatwoods.

###### Notes

Apr–Aug; Jul–Nov. Not seen in Shaken Creek Preserve by the senior author. Specimens seen in the vicinity: Sandy Run [Hancock]: Taggart SARU 176 (WNC!). [= RAB, Weakley]

##### Centrosema virginianum

(L.) Benth.

###### Distribution

Mesic pine savannas (MPS-CP).

###### Notes

Occasional. Jun–Aug; Jul–Oct. Thornhill 972 (NCSC). Specimens seen in the vicinity: Sandy Run [Hancock]: Taggart SARU 423 (WNC!). [= RAB, Weakley]

##### Chamaecrista fasciculata var. fasciculata

(Michx.) Greene

###### Distribution

Pine savannas, wet pine flatwoods.

###### Notes

Jun–Sep; Jul–Nov. Not seen in Shaken Creek Preserve by the senior author. Specimens seen in the vicinity: Sandy Run [Hancock]: Taggart SARU 406 (WNC!). [< *Cassia fasciculata* Michx. sensu RAB; = Weakley]

##### Chamaecrista nictitans var. nictitans

(L.) Moench

###### Distribution

Wet pine savannas (VWLPS), adjacent roadsides.

###### Notes

Infrequent. Jun–Oct; Jul–Nov. Thornhill 1172 (NCSC). Specimens seen in the vicinity: Sandy Run [Patterson]: Taggart SARU 643 (WNC!). [< *Cassia nictitans* L. sensu RAB; = Weakley]

##### Crotalaria purshii

DC.

###### Distribution

Mesic to dry pinelands, sandy openings, roadsides.

###### Notes

Reported from Sandy Run [Neck] by LeBlond and Weakley (1991) (and seen there by the senior author), but no specimens have been seen in Shaken Creek Preserve. [= RAB, Weakley]

##### Desmodium ciliare

(Muhl. ex Willd.) DC.

###### Distribution

Fields, woodland borders, disturbed areas.

###### Notes

Jun–Sep; Aug–Oct. Reported from Sandy Run [Neck] by LeBlond and Weakley (1991), but no specimens have been seen in Shaken Creek Preserve by the senior author. [= RAB, Weakley]

##### Desmodium lineatum

(Michx.) DC.

###### Distribution

Sandhills and other dry forests and woodlands.

###### Notes

Jun–Sep; Aug–Oct. Reported from Sandy Run [Neck] by LeBlond and Weakley (1991), but no specimens have been seen in Shaken Creek Preserve by the senior author. [= RAB, Weakley]

##### Desmodium paniculatum

(L.) DC.

###### Distribution

Pine savannas and flatwoods, fields, woodland borders, disturbed areas.

###### Notes

Jun–Sep; Aug–Oct. Reported from Sandy Run [Neck] by LeBlond and Weakley (1991), but no specimens have been seen in Shaken Creek Preserve by the senior author. [= RAB; > Desmodium paniculatum var. epepetiolatum B.G. Schub, Desmodium paniculatum var. paniculatum sensu Weakley]

##### Desmodium tenuifolium

Torr. & A. Gray

###### Distribution

Wet pine flatwoods (WPF-T), wet pine savannas (WLPS, VWLPS).

###### Notes

Occasional. Jul–Aug; Aug–Oct. Thornhill 827, 1046, 1084 (NCSC). Specimens seen in the vicinity: Sandy Run [Hancock]: Ahles 32702 (NCU!), Taggart SARU 447 (WNC!). [= RAB, Weakley]

##### Galactia regularis

(L.) Britton, Sterns, & Poggenb.

###### Distribution

Wet pine flatwoods (WPF-T).

###### Notes

Jul–Sep; Aug–Oct. Reported from Shaken Creek Preserve by LeBlond (2000), but no specimens have been seen on site by the senior author. Specimens seen in the vicinity: Sandy Run [Hancock]: Taggart SARU 368 (WNC!; reported for roadsides and disturbed areas). [> *Galactia macreei* M.A. Curtis, *Galactia volubilis* (L.) Britton sensu RAB; = Weakley]

##### Indigofera caroliniana

Mill.

###### Distribution

Mesic pine savannas.

###### Notes

Jun–Aug; Jul–Oct. Not seen in Shaken Creek Preserve by the senior author. Specimens seen in the vicinity: Sandy Run [RMK]: Taggart SARU 389 (WNC!). [= RAB, Weakley]

##### Lespedeza angustifolia

(Pursh) Elliott

###### Distribution

Mesic pine savannas (MPS-CP).

###### Notes

Rare. Aug–Oct; Sep–Nov. Thornhill 1553 (NCSC). [= RAB, Weakley]

##### Lespedeza capitata

Michx.

###### Distribution

Pine/scrub oak sandhills (PSOS-MT), mesic pine savannas (MPS-CP), wet pine savannas (WLPS), roadsides.

###### Notes

Infrequent. Aug–Oct; Sep–Nov. Thornhill 1031, 1075 (NCSC). Specimens seen in the vicinity: Sandy Run [Hancock]: Taggart SARU 467 (WNC!); Sandy Run [Neck]: Wilbur 57632 (DUKE!). [= RAB, Weakley]

##### Lespedeza hirta var. curtissii

(Clewell) Isely

###### Distribution

Pine/scrub oak sandhills (PSOS-MT).

###### Notes

Rare. Aug–Oct; Sep–Nov. Thornhill 1539 (NCSC). Specimens seen in the vicinity: Sandy Run [RMK]: Taggart SARU 475 (WNC!). [< *Lespedeza hirta* (L.) Hornem. sensu RAB; = Weakley]

##### Strophostyles umbellata

(Muhl. ex Willd.) Britton

###### Distribution

Dry, sandy woodlands.

###### Notes

Jun–Sep; Aug–Oct. Reported from Sandy Run [Neck] by LeBlond and Weakley (1991), but no specimens have been seen in Shaken Creek Preserve by the senior author. [= RAB, Weakley]

##### Stylosanthes biflora

(L.) Britton, Sterns & Poggenb.

###### Distribution

Mesic pine savannas (MPS-CP).

###### Notes

Rare. Jun–Aug; Jul–Oct. Thornhill 1473 (NCSC). [= RAB, Weakley]

##### Tephrosia florida

(F. Dietr.) C.E. Wood

###### Distribution

Wet pine flatwoods (WPF-T).

###### Notes

May–Jul; Jun–Sep. Reported from Shaken Creek Preserve by LeBlond (2000), but no specimens have been seen on site by the senior author. [= RAB, Weakley]

##### Tephrosia hispidula

(Michx.) Pers.

###### Distribution

Wet pine flatwoods (WPF-T), wet pine savannas (WLPS).

###### Notes

Occasional. May–Aug; Jul–Oct. Thornhill 416, 553, 602, 702 (NCSC). Specimens seen in the vicinity: Sandy Run [Hancock]: Taggart SARU 321 (WNC!). [= RAB, Weakley]

##### Tephrosia spicata

(Walter) Torr. & A. Gray

###### Distribution

Woodlands and roadsides.

###### Notes

Jun–Aug; Jul–Oct. Reported from Sandy Run [Neck] by LeBlond and Weakley (1991), but no specimens have been seen in Shaken Creek Preserve by the senior author. [= RAB, Weakley]

##### Zornia bracteata

J.F. Gmel.

###### Distribution

Flatwoods, sandhills, sandy roadsides.

###### Notes

Jun–Aug; Jul–Oct. Reported from Sandy Run [Neck] by LeBlond and Weakley (1991), but no specimens have been seen in Shaken Creek Preserve by the senior author. [= RAB, Weakley]

#### Fagaceae

##### Quercus coccinea

Münchh.

###### Distribution

Pine/scrub oak sandhills (PSOS-MT).

###### Notes

Infrequent. Apr; Sep–Nov (of second year). Thornhill 1543 (NCSC). [= RAB, FNA, Weakley]

##### Quercus falcata

Michx.

###### Distribution

Pine/scrub oak sandhills (PSOS-MT).

###### Notes

Infrequent. Apr; Sep–Nov (of second year). Thornhill 1329 (NCSC). Specimens seen in the vicinity: Sandy Run [Neck]: Taggart SARU 297, (WNC!), Wilbur 55278 (DUKE!). [= Quercus falcata var. falcata sensu RAB; = FNA, Weakley]

##### Quercus incana

W. Bartram

###### Distribution

Pine/scrub oak sandhills (PSOS-MT).

###### Notes

Infrequent. Apr; Sep–Nov (of second year). Thornhill 1327 (NCSC). Specimens seen in the vicinity: Sandy Run [Neck]: Taggart SARU 295 (WNC!). [= RAB, FNA, Weakley]

##### Quercus laurifolia

Michx.

###### Distribution

Wet pine flatwoods (WPF-T), wet pine savannas (WLPS, VWLPS).

###### Notes

Frequent. Mar–Apr; Sep–Nov (of second year). Thornhill 429, 1022 (NCSC). Specimens seen in the vicinity: Sandy Run [Hancock]: Taggart SARU 206 (WNC!). [< RAB; = FNA, Weakley]

##### Quercus margarettae

(Ashe) Small

###### Distribution

Pine/scrub oak sandhills (PSOS-MT).

###### Notes

Infrequent. Apr; Sep–Nov. Thornhill 1328 (NCSC). Specimens seen in the vicinity: Sandy Run [Neck]: Taggart SARU 296 (WNC!). [= *Quercus margaretta* Ahles ex Small sensu RAB; = FNA, Weakley]

##### Quercus marilandica var. marilandica

Münchh.

###### Distribution

Pine/scrub oak sandhills (PSOS-MT).

###### Notes

Infrequent. Apr; Sep–Nov (of second year). Thornhill 1317 (NCSC). [< *Quercus marilandica* Münchh. sensu RAB; = FNA, Weakley]

##### Quercus nigra

L.

###### Distribution

Wet pine savannas (SPS-RF, WLPS, VWLPS).

###### Notes

Occasional. Apr; Sep–Nov (of second year). Thornhill 203, 207, 282, 362 (NCSC). Specimens seen in the vicinity: Sandy Run [Neck]: Wilbur 55257 (DUKE!); Sandy Run [Patterson]: Taggart SARU 212 (WNC!). [= RAB, FNA, Weakley]

##### Quercus stellata

Wangenh.

###### Distribution

Upland forests and woodlands.

###### Notes

Apr; Sep–Nov (of same year). Not seen in Shaken Creek Preserve by the senior author. Specimens seen in the vicinity: Sandy Run [Neck]: Wilbur 55283 (DUKE!). [= RAB, FNA, Weakley]

##### Quercus velutina

Lam.

###### Distribution

Wet pine flatwoods.

###### Notes

Apr; Sep–Oct (of second year). Not seen in Shaken Creek Preserve by the senior author. Specimens seen in the vicinity: Sandy Run [Neck]: Taggart SARU 584 (WNC!). [= RAB, FNA, Weakley]

#### Gelsemiaceae

##### Gelsemium sempervirens

J. St.-Hil.

###### Distribution

Pine/scrub oak sandhills (PSOS-MT), mesic pine savannas (MPS-CP), wet pine flatwoods (WPF-T).

###### Notes

Occasional. Mar–early May; Sep–Nov. Thornhill 84 (NCSC). Specimens seen in the vicinity: Sandy Run [Hancock]: Taggart SARU 11 (WNC!). [= RAB, Weakley]

#### Gentianaceae

##### Bartonia verna

Raf. ex Barton

###### Ecological interactions

####### Conservation status

W1; S2, G5?.

###### Distribution

Wet pine savannas (SPS-T).

###### Notes

Infrequent. (Nov–)Feb–Apr(–Jun); Apr–Jun. Thornhill 1250 (NCSC). Specimens seen in the vicinity: Sandy Run: Taggart SARU 572 (WNC!). [= RAB, Weakley]

##### Bartonia virginica

(L.) Britton, Sterns & Poggenb.

###### Distribution

Wet pine savannas (SPS-T, SPS-PF, WLPS, VWLPS).

###### Notes

Infrequent. Jul–Oct; Sep–Oct. Thornhill 749, 907 (NCSC). Specimens seen in the vicinity: Sandy Run [Hancock]: Taggart SARU 493 (WNC!). [= RAB, Weakley]

##### Gentiana autumnalis

L.

###### Distribution

Wet pine flatwoods (WPF-T), wet pine savannas (WLPS, VWLPS).

###### Notes

Occasional. Late Sep–mid Jan. Thornhill 47, 1234 (NCSC). Specimens seen in the vicinity: Sandy Run [Patterson]: Taggart SARU 510 (WNC!). [= RAB, Weakley]

##### Gentiana catesbaei

Walter

###### Distribution

Wet pine savannas (SPS-T, WLPS, VWLPS).

###### Notes

Infrequent. Late Sep–Nov. Thornhill 1204, 1230, 1233 (NCSC). Specimens seen in the vicinity: Sandy Run [Hancock]: Taggart SARU 548 (WNC!). [= RAB, Weakley]

##### Gentiana saponaria

L.

###### Distribution

Wet pine savannas (WLPS, VWLPS).

###### Notes

Rare. Sep–Nov. Thornhill 1183, 1231 (NCSC). [= RAB, Weakley]

##### Sabatia angularis

(L.) Pursh

###### Distribution

Forests, woodlands, marshes, fields.

###### Notes

Jul–Aug; Sep–Oct. Not seen in Shaken Creek Preserve by the senior author. Specimens seen in the vicinity: Sandy Run [Neck]: Wilbur 53669, 53672 (DUKE!). [= RAB, Weakley]

##### Sabatia brachiata

Elliott

###### Distribution

Pine savannas and flatwoods.

###### Notes

Late May–Jul; Aug–Sep. Not seen in Shaken Creek Preserve by the senior author. Specimens seen in the vicinity: Sandy Run [Hancock]: Ahles 28234 (NCU!), Taggart SARU 260 (WNC!). [= RAB, Weakley]

##### Sabatia campanulata

(L.) Torr.

###### Distribution

Wet pine savannas (WLPS, VWLPS).

###### Notes

Occasional. Jun–Aug; Sep–Oct. Thornhill 550, 560, 609, 619 (NCSC). Specimens seen in the vicinity: Sandy Run [Hancock]: Taggart SARU 314 (WNC!). [= RAB, Weakley]

##### Sabatia difformis

(L.) Druce

###### Distribution

Wet pine flatwoods (WPF-T), wet pine savannas (SPS-T, SPS-RF, WLPS, VWLPS).

###### Notes

Frequent. May–Sep; Sep–Dec. Thornhill 420, 485, 521, 525, 555, 582, 583, 584, 706 (NCSC). Specimens seen in the vicinity: Sandy Run [Hancock]: Taggart SARU 247 (WNC!), Wilbur 53689 (DUKE!). [= RAB, Weakley]

##### Sabatia gentianoides

Elliott

###### Distribution

Wet pine savannas (VWLPS).

###### Notes

Rare. Jul–Aug; Sep–Oct. Thornhill 1450 (NCSC). Specimens seen in the vicinity: Sandy Run [Hancock]: Taggart SARU 375 (WNC!). [= RAB, Weakley]

#### Haloragaceae

##### Proserpinaca pectinata

Lam.

###### Distribution

Depressions in pine savannas (WLPS), borrow pits, ditches.

###### Notes

Occasional. Jun–Oct. Thornhill 358, 509, 621 (NCSC). Specimens seen in the vicinity: Sandy Run [Hancock]: Taggart SARU 271 (WNC!). [= RAB, Weakley]

#### Hamamelidaceae

##### Fothergilla gardenii

L.

###### Distribution

Margins of wet pine flatwoods (WPF-T) and pocosins.

###### Notes

Infrequent. Mar–May; Sep–Oct. Thornhill 1273 (NCSC). Specimens seen in the vicinity: Sandy Run [Patterson]: Taggart SARU 51 (WNC!). [= RAB, FNA, Weakley]

#### Hypericaceae

##### Hypericum brachyphyllum

(Spach) Steud.

###### Ecological interactions

####### Conservation status

SC-V; S1S2, G5.

###### Distribution

Wet pine savannas (SPS-T, WLPS, VWLPS).

###### Notes

Frequent. Jul–Sep. LeBlond 4989, 5736A (NCSC!); Thornhill 415, 478, 527, 606, 615, 716 (NCSC). Specimens seen in the vicinity: Sandy Run: LeBlond 5771 (NCSC!), Taggart SARU 247 (WNC!). [= Weakley]

##### Hypericum canadense

L.

###### Distribution

Wet pine savannas (SPS-T, SPS-RF), adjacent roadsides.

###### Notes

Infrequent. Jul–Sep. Thornhill 707, 720, 795, 803, 904, 940 (NCSC). [= RAB, Weakley]

##### Hypericum cistifolium

Lam.

###### Distribution

Wet pine flatwoods (WPF-T), wet pine savannas (SPS-T, SPS-RF, WLPS, VWLPS).

###### Notes

Frequent. Jun–Aug. Thornhill 459, 607, 683, 718, 799, 805, 843 (NCSC). [= RAB, Weakley]

##### Hypericum crux-andreae

(L.) Crantz

###### Distribution

Wet pine flatwoods (WPF-T), wet pine savannas (SPS-RF, WLPS, VWLPS).

###### Notes

Occasional. Jun–Oct. Thornhill 700, 746 (NCSC). Specimens seen in the vicinity: Sandy Run [Hancock]: Taggart SARU 308 (WNC!). [=*Hypericum stans* (Michx. ex Willd.) W.P. Adams & N. Robson sensu RAB; = Weakley]

##### Hypericum densiflorum var. densiflorum

Pursh

###### Distribution

Wet pine flatwoods (WPF-T), wet pine savannas (SPS-T, SPS-RF, VWLPS).

###### Notes

Occasional. Jun–Sep. Thornhill 910, 953, 1205 (NCSC). Specimens seen in the vicinity: Sandy Run [Neck]: Taggart SARU 325 (WNC!; as *Hypericum densiflorum*). [< *Hypericum densiflorum* Pursh sensu RAB; = Weakley]

##### Hypericum denticulatum

Walter

###### Distribution

Wet pine flatwoods (WPF-T), wet pine savannas (WLPS).

###### Notes

Infrequent. Jul–Sep. Thornhill 762, 9665 (NCSC). [= Hypericum denticulatum var. denticulatum sensu RAB; = Weakley]

##### Hypericum galioides

Lam.

###### Distribution

Wet pine flatwoods (WPF-T), wet pine savannas (SPS-RF, WLPS, VWLPS).

###### Notes

Occasional. Jun–Aug. Thornhill 436, 563, 632, 747, 831, 944 (NCSC). Specimens seen in the vicinity: Sandy Run [Haw’s Run]: Taggart SARU 628 (WNC!); Sandy Run [Neck]: LeBlond 2252 (NCU!), Sorrie 5884 (NCU!). [= RAB, Weakley]

##### Hypericum gentianoides

(L.) Britton, Sterns, & Poggenb.

###### Distribution

Wet pine flatwoods (WPF-T), wet pine savannas (SPS-RF, VWLPS), adjacent roadsides.

###### Notes

Occasional. Jul–Oct. Thornhill 513, 599, 636 (NCSC). Specimens seen in the vicinity: Sandy Run [O’Berry]: Taggart SARU 246 (WNC!). [= RAB, Weakley]

##### Hypericum gymnanthum

Engelm. & A. Gray

###### Distribution

Wet pine flatwoods (WPF-T), wet pine savannas (VWLPS), adjacent roadsides.

###### Notes

Occasional. Jun–Sep. Thornhill 1278, 1453 (NCSC). Specimens seen in the vicinity: Sandy Run [O’Berry]: Taggart SARU 292 (WNC!; as Hypericum mutilum var. mutilum). [= RAB, Weakley]

##### Hypericum hypericoides

(L.) Crantz

###### Distribution

Wet pine savannas (WLPS, VWLPS).

###### Notes

Infrequent. May–Aug. Thornhill 460, 846, 1147 (NCSC). Specimens seen in the vicinity: Sandy Run [Hancock]: Taggart SARU 103 (WNC!). [= RAB, Weakley]

##### Hypericum lloydii

(Svenson) W.P. Adams

###### Distribution

Pine savannas.

###### Notes

Jun–Sep. Not seen in Shaken Creek Preserve by the senior author. Specimens seen in the vicinity: Sandy Run [Hancock]: Taggart SARU 378 (WNC!; determination by the senior author of this specimen is tentative; the voucher may represent merely a branch of *Hypericum galioides*). [= RAB, Weakley]

##### Hypericum mutilum var. mutilum

L.

###### Distribution

Roadsides adjacent to and scrapes within pine savannas (SPS-RF, VWLPS).

###### Notes

Occasional. Jun–Oct. Thornhill 1340, 1461, 1464, 1487 (NCSC). [< *Hypericum mutilum* L. sensu RAB; = Weakley]

##### Hypericum setosum

L.

###### Distribution

Wet pine flatwoods (WPF-T), adjacent roadsides.

###### Notes

Rare. May–Sep. Thornhill 1479 (NCSC). Specimens seen in the vicinity: Sandy Run [Hancock]: Taggart SARU 420 (WNC!). [= RAB; Weakley]

##### Hypericum tenuifolium

Pursh

###### Distribution

Wet pine flatwoods (WPF-T).

###### Notes

Occasional. Jun–Sep. Thornhill 1312, 1319 (NCSC). Specimens seen in the vicinity: Sandy Run [Hancock]: Taggart SARU 261 (WNC!); Sandy Run [Neck]: Wilbur 53686 (DUKE!). [= *Hypericum reductum* (Svenson) W.P. Adams sensu RAB; = Weakley]

##### Hypericum virginicum

L.

###### Distribution

Boggy depressions within or near flatwoods or savannas.

###### Notes

Jul–Sep. No specimens have been seen on site by the senior author; however, one specimen (Thornhill 1479, NCSC) was collected in a boggy depression adjacent to a flatwoods just north (< 1 mile) of Shaken Creek Preserve. The presence of this species may be expected in boggy areas on site. [= RAB; Weakley]

#### Juglandaceae

##### Carya tomentosa

(Poir.) Nutt.

###### Distribution

Mesic pine savannas.

###### Notes

Apr–May; Oct. Not seen in Shaken Creek Preserve by the senior author. Specimens seen in the vicinity: Sandy Run [RMK]: Taggart SARU 223 (WNC!; as *Carya alba*). [= RAB, FNA, Weakley]

#### Lamiaceae

##### Hyptis alata

Shinners

###### Distribution

Wet pine savannas (WLPS, VWLPS).

###### Notes

Occasional. Late Jun–Sep. Thornhill 705, 826, 946 (NCSC). Specimens seen in the vicinity: Sandy Run [Hancock]: Taggart SARU 404 (WNC!); Sandy Run [Neck]: Wilbur 57669 (DUKE!). [= RAB, Weakley]

##### Lycopus amplectens

Raf.

###### Ecological interactions

####### Conservation status

W1; S3, G5.

###### Distribution

Wet pine savannas (VWLPS).

###### Notes

Rare. Jun–Nov. Thornhill 1089 (NCSC). Specimens seen in the vicinity: Sandy Run: LeBlond 4848, 5069 (NCU!), Taggart SARU 468 (WNC!). [= RAB, Weakley]

##### Lycopus rubellus

Moench

###### Distribution

Wet pine savannas (WLPS).

###### Notes

Rare. Jun–Nov. Thornhill 1150 (NCSC). Specimens seen in the vicinity: Sandy Run [Hancock]: Taggart SARU 498 (WNC; see note preceding genus key). [= Lycopus rubellus Moench var. rubellus sensu RAB; = Weakley]

##### Physostegia purpurea

(Walter) S.F. Blake

###### Distribution

Wet pine savannas (WLPS, VWLPS).

###### Notes

Infrequent. Late May–early Aug; Jun–Sep. Thornhill 419, 422, 425, 564 (NCSC). Specimens seen in the vicinity: Sandy Run [Hancock]: Taggart SARU 238 (WNC!; as Physostegia virginiana ssp. praemorsa); Sandy Run [Neck]: Levy s.n. (DUKE!); Wilbur 53695 (DUKE!). [< *Dracocephalum purpureum* (Walter) E.M. McClint. ex Gleason sensu RAB; = Weakley]

##### Pycnanthemum flexuosum

(Walter) Britton, Sterns, & Poggenb.

###### Distribution

Wet pine savannas (WLPS, VWLPS).

###### Notes

Frequent. Jun–Sep; Sep–Oct. Thornhill 603, 679, 704, 948, 1547 (NCSC). Specimens seen in the vicinity: Sandy Run [Neck]: Taggart SARU 191 (WNC!), Wilbur 53641, 53699 (DUKE!). [= RAB, Weakley]

##### Pycnanthemum setosum

Nutt.

###### Ecological interactions

####### Conservation status

SR-T; S2, G4.

###### Distribution

Dry pinelands.

###### Notes

Mid Jun–Aug; Aug–Oct. The specimen for this report (Thornhill 1547, NCSC), which was collected by the author at the edge of a dirt road and powerline savanna in Shaken Creek Preserve, has calyx lobes somewhat shorter and leaves somewhat narrower than is typical for *Pycnanthemum setosum*. However, based on comparisons to specimens at NCSC and NCU and following the advice of better botanists (in particular, Derrick Poindexter (NCU)), the specimen seems at least to align most closely with *Pycnanthemum setosum*. More study is needed to clarify the taxonomy of this genus. *Pycnanthemum setosum* is also reported within a 2-mile radius of Shaken Creek Preserve by the North Carolina Natural Heritage Program (http://www.ncnhp.org) (EO status “current”, accuracy “medium”), though no vouchers for this report have been seen by the senior author. [= RAB, Weakley]

##### Scutellaria integrifolia

L.

###### Distribution

Wet pine savannas (WLPS, VWLPS).

###### Notes

Frequent. May–Jul; Jul–Aug. Thornhill 316, 363 (NCSC). Specimens seen in the vicinity: Sandy Run [Hancock]: Taggart SARU 117 (WNC!), Wilbur 55319 (DUKE!). [> Scutellaria integrifolia L. var. integrifolia, Scutellaria integrifolia L. var. hispida Benth. sensu RAB; = Weakley]

#### Lauraceae

##### Persea palustris

(Raf.) Sarg.

###### Distribution

Wet pine flatwoods (WPF-T), wet pine savannas (SPS-T, SPS-RF).

###### Notes

Frequent. May–Jun; Sep–Oct. Thornhill 497, 542 (NCSC). Specimens seen in the vicinity: Sandy Run [Patterson]: Taggart SARU 154 (WNC!). [< *Persea borbonia* (L.) Spreng. sensu RAB; = FNA, Weakley]

##### Sassafras albidum

J. Presl

###### Distribution

Pine/scrub oak sandhills (PSOS-MT), mesic pine savannas (MPS-CP).

###### Notes

Infrequent. Mar–Apr; Jun–Jul. Thornhill 1534 (NCSC). Specimens seen in the vicinity: Sandy Run [RMK]: Taggart SARU 222 (WNC!). [= RAB, FNA, Weakley]

#### Lentibulariaceae

##### Pinguicula caerulea

Walter

###### Distribution

Wet pine savannas (SPS-T, SPS-RF, WLPS, VWLPS).

###### Notes

Occasional. Apr–May. Thornhill 103, 126, 141 (NCSC). Specimens seen in the vicinity: Sandy Run [Hancock]: Taggart SARU 55 (WNC!); Sandy Run [Neck]: Wilbur 63790, 67099 (DUKE!). [= RAB, Weakley]

##### Pinguicula pumila

Michx.

###### Ecological interactions

####### Conservation status

State E; S2, G4.

###### Distribution

Wet pine savannas (SPS-RF, WLPS, VWLPS).

###### Notes

Infrequent. Apr–May. Thornhill 108, 125, 143, 163 (NCSC). Specimens seen in the vicinity: Sandy Run: Taggart SARU 574 (WNC!). [= RAB, Weakley]

##### Utricularia juncea

Vahl

###### Distribution

Wet pine savannas (SPS-T), borrow pits.

###### Notes

Rare. Jul–Sep. Thornhill 32 (NCSC). Specimens seen in the vicinity: Sandy Run [Patterson]: Taggart SARU 645 (WNC!). [= RAB, Weakley]

##### Utricularia subulata

L.

###### Distribution

Wet pine flatwoods (WPF-T), wet pine savannas (SPS-T, WLPS, VWLPS).

###### Notes

Frequent. Mar–Jul(–later). Thornhill 107, 158, 216 (NCSC). Specimens seen in the vicinity: Sandy Run [Hancock]: Taggart SARU 143 (WNC!); Sandy Run [Neck]: Wilbur 63791 (DUKE!). [= RAB, Weakley]

#### Linaceae

##### Linum floridanum var. chrysocarpum

C.M. Rogers

###### Ecological interactions

####### Conservation status

State T; S1S2, G5?T3?.

###### Distribution

Pine savannas.

###### Notes

Jun–Oct. Not seen in Shaken Creek Preserve by the senior author. Specimens seen in the vicinity: Sandy Run: LeBlond 2536 (NCU!), Taggart SARU 565 (WNC!). [< Linum virginianum L. var. floridanum Planch. sensu RAB; = Weakley]

##### Linum floridanum var. floridanum

(Planch.) Trel.

###### Distribution

Wet pine savannas (WLPS, VWLPS).

###### Notes

Occasional. Jun–Oct. Thornhill 374, 569, 605, 753, 823 (NCSC). Specimens seen in the vicinity: Sandy Run [Haw’s Run]: Taggart SARU 525 (WNC!). [< Linum virginianum L. var. floridanum Planch. sensu RAB; = Weakley]

##### Linum intercursum

E.P. Bicknell

###### Distribution

Wet pine savannas (SPS-RF).

###### Notes

Infrequent. Jun–Oct. Thornhill 486 (NCSC). [< Linum virginianum L. var. floridanum Planch. sensu RAB; = Weakley]

##### Linum medium var. texanum

(Planch.) Fernald

###### Distribution

Wet pine savannas (VWLPS).

###### Notes

Infrequent. Jun–Oct. Thornhill 568 (NCSC). Specimens seen in the vicinity: Highway 50: Wilbur 8390 (DUKE!; as *Linum medium*); Sandy Run [Neck]: Taggart SARU 47 (WNC!; as *Linum medium*), Wilbur 55267 (DUKE!; as *Linum medium*). [< Linum virginianum L. var. medium Planch. sensu RAB; = Weakley]

##### Linum striatum

Walter

###### Distribution

Wet pine savannas (WLPS, VWLPS).

###### Notes

Infrequent. Jun–Oct. Thornhill 970 (NCSC). [= RAB, Weakley]

#### Linderniaceae

##### Lindernia dubia var. anagallidea

(Michx.) Cooperr.

###### Distribution

Wet pine flatwoods (WPF-T).

###### Notes

Rare. Jun–Sep. Thornhill 1510 (NCSC). [= *Lindernia anagallidea* (Michx.) Pennell sensu RAB; = Weakley]

#### Loganiaceae

##### Mitreola petiolata

(J.F. Gmel.) Torr. & A. Gray

###### Distribution

Wet pine savannas (VWLPS).

###### Notes

Rare. Jul–Sep; Sep–Nov. Thornhill 844 (NCSC). Specimens seen in the vicinity: Sandy Run [Hancock]: Taggart SARU 374 (WNC!); Sandy Run [Neck]: Wilbur 53688 (DUKE!). [= Cynoctonum mitreola (L.) Britton sensu RAB; = Weakley]

##### Mitreola sessilifolia

(J.F. Gmel.) G. Don

###### Distribution

Wet pine savannas (VWLPS), ditches.

###### Notes

Infrequent. Late Jun–Aug; Sep–Oct. Thornhill 558, 681 (NCSC). Specimens seen in the vicinity: Sandy Run [Hancock]: Taggart SARU 334 (WNC!); Sandy Run [Neck]: Wilbur 53700 (DUKE!). [= *Cynoctonum sessilifolium* J.F. Gmel. sensu RAB; = Weakley]

#### Lythraceae

##### Ammannia coccinea

Rottb.

###### Distribution

Wet pine flatwoods, ditches, other wet places.

###### Notes

Jul–Oct. Not seen in Shaken Creek Preserve by the senior author. Specimens seen in the vicinity: Sandy Run [Neck]: LeBlond 2831 (NCU!), Wilbur 57640 (DUKE!). [= RAB, Weakley]

#### Magnoliaceae

##### Liriodendron tulipifera

L.

###### Distribution

Pine savannas, wet pine flatwoods.

###### Notes

Apr–Jun; Sep–Oct. Not seen in Shaken Creek Preserve by the senior author. Specimens seen in the vicinity: Sandy Run [O’Berry]: Taggart SARU 162 (WNC!; as *Liriodendron tulipifera* var. 1), Weakley 7217 (NCU!; as Liriodendron tulipifera var. variabilis). [= RAB, FNA; > *Liriodendron tulipifera* var. 1, Liriodendron tulipifera var. tulipifera sensu Weakley]

##### Magnolia virginiana

L.

###### Distribution

Wet pine flatwoods (WPF-T), wet pine savannas (SPS-T, SPS-RF).

###### Notes

Frequent. Apr–Jul; Jul–Oct. Thornhill 235, 263 (NCSC). Specimens seen in the vicinity: Sandy Run [O’Berry]: Taggart SARU 160 (WNC!; as Magnolia virginiana var. australis), Taggart SARU 553 (WNC!; as Magnolia virginiana var. virginiana). [= RAB, FNA; > Magnolia virginiana L. var. australis Sarg., Magnolia virginiana L. var. virginiana sensu Weakley]

#### Melastomataceae

##### Rhexia alifanus

Walter

###### Distribution

Wet pine flatwoods (WPF-T), wet pine savannas (SPS-T, SPS-RF, WLPS, VWLPS).

###### Notes

Frequent. May–Sep. Thornhill 524, 557, 580 (NCSC). Specimens seen in the vicinity: Sandy Run [Hancock]: Taggart SARU 263 (WNC!); Sandy Run [Neck]: Wilbur 53691 (DUKE!). [= RAB, Weakley]

##### Rhexia lutea

Walter

###### Distribution

Wet pine savannas (SPS-T, SPS-RF, WLPS, VWLPS).

###### Notes

Occasional. Apr–Jul(–later in response to fire). Thornhill 311, 320, 353, 393 (NCSC). Specimens seen in the vicinity: Sandy Run [Hancock]: Taggart SARU 188 (WNC!); Sandy Run [Neck]: Levy s.n. (DUKE!), Wilbur 53679, 55314 (DUKE!). [= RAB, Weakley]

##### Rhexia mariana var. exalbida

Michx.

###### Distribution

Wet pine savannas (SPS-T, SPS-RF, WLPS, VWLPS).

###### Notes

Occasional. Jun–Sep. Thornhill 501, 520, 567, 593, 668 (NCSC). [= RAB, Weakley]

##### Rhexia mariana var. mariana

L.

###### Distribution

Wet pine savannas (WLPS).

###### Notes

Rare. May–Oct. Thornhill 617 (NCSC). Specimens seen in the vicinity: Sandy Run [RMK]: Taggart SARU 224 (WNC!). [= RAB, Weakley]

##### Rhexia nashii

Small

###### Distribution

Wet pine flatwoods (WPF-T), wet pine savannas (SPS-T, SPS-RF, WLPS).

###### Notes

Frequent. May–Oct. Thornhill 595, 739, 794, 906 (NCSC). Specimens seen in the vicinity: Sandy Run [Hancock]: Taggart SARU 429 (WNC!); Sandy Run [Neck]: Wilbur 53643 (DUKE!). [= Rhexia mariana var. purpurea Michx. sensu RAB; = Weakley]

##### Rhexia petiolata

Walter

###### Distribution

Wet pine flatwoods (WPF-T), wet pine savannas (SPS-T, SPS-RF, WLPS, VWLPS).

###### Notes

Frequent. Jun–Sep. Thornhill 665, 675, 792 (NCSC). Specimens seen in the vicinity: Sandy Run [Hancock]: Taggart SARU 414 (WNC!); Sandy Run [Neck]: Wilbur 53675 (DUKE!). [= RAB, Weakley]

#### Myricaceae

##### Morella caroliniensis

(Mill.) Small

###### Distribution

Wet pine flatwoods (WPF-T), wet pine savannas (SPS-T, SPS-RF, WLPS, VWLPS).

###### Notes

Frequent. Apr; Aug–Oct. Thornhill 121, 134, 149, 154, 169 (NCSC). Specimens seen in the vicinity: Highway 50: Wilbur 8388 (DUKE!; as *Myrica heterophylla*); Sandy Run [Hancock]: Taggart SARU 180 (WNC!); Sandy Run [Neck]: Wilbur 67095 (DUKE!). [=*Myrica heterophylla* Raf. sensu RAB, FNA; = Weakley]

##### Morella cerifera

(L.) Small

###### Distribution

Wet pine flatwoods (WPF-T), wet pine savannas (SPS-T, SPS-RF, WLPS, VWLPS).

###### Notes

Frequent. Apr; Aug–Oct. Thornhill 131, 132, 148, 170 (NCSC). Specimens seen in the vicinity: Sandy Run [Haw’s Run]: Taggart SARU 16 (WNC!); Sandy Run [Neck]: Wilbur 53682 (DUKE!). [= Myrica cerifera L. var. cerifera sensu RAB; < *Myrica cerifera* L. sensu FNA; = Weakley]

##### Morella pumila

(Michx.) Small

###### Distribution

Wet pine flatwoods (WPF-T), wet pine savannas (SPS-T, SPS-RF, WLPS).

###### Notes

Frequent. Apr; Aug–Oct. Thornhill 119, 152, 172 (NCSC). Specimens seen in the vicinity: Sandy Run [Neck]: Wilbur 60088 (DUKE!; as *Myrica pusilla*), Wilbur 63780 (DUKE!; as Morella pumila var. cerifera); Sandy Run [Patterson]: Taggart SARU 302 (WNC!). [= Myrica cerifera L. var. pumila Michx. sensu RAB; < *Myrica cerifera* L. sensu FNA; = Weakley]

#### Nyssaceae

##### Nyssa biflora

Walter

###### Distribution

Wet pine flatwoods (WPF-T), wet pine savannas (SPS-T, SPS-RF, WLPS).

###### Notes

Occasional. Apr–Jun; Aug–Oct. Thornhill 230, 256 (NCSC). Specimens seen in the vicinity: Sandy Run [RMK]: Taggart SARU 235 (WNC!). [= Nyssa sylvatica Marshall var. biflora (Walter) Sarg. sensu RAB; = Weakley]

##### Nyssa sylvatica

Marshall

###### Distribution

Wet pine flatwoods (WPF-T).

###### Notes

Apr–Jun; Aug–Oct. Reported from Shaken Creek Preserve by LeBlond (2000), but no specimens have been seen on site by the senior author. Specimens seen in the vicinity: Sandy Run [RMK]: Taggart SARU 582 (WNC!). [= Nyssa sylvatica Marshall var. sylvatica sensu RAB; = Weakley]

#### Oleaceae

##### Fraxinus caroliniana

Mill.

###### Distribution

Swampy margins of wet pine savannas (VWLPS), borrow pits, ditches.

###### Notes

Infrequent. May; Jul–Oct. Thornhill 242 (NCSC). Specimens seen in the vicinity: Sandy Run [Patterson]: Taggart SARU 155 (WNC!). [= RAB, Weakley]

#### Onagraceae

##### Ludwigia hirtella

Raf.

###### Distribution

Wet pine savannas (WLPS, VWLPS).

###### Notes

Infrequent. Jun–Sep. Thornhill 1446 (NCSC). Specimens seen in the vicinity: Sandy Run [Hancock]: Taggart SARU 306 (WNC!). [= RAB, Weakley]

##### Ludwigia linearis

Walter

###### Distribution

Wet pine savannas (SPS-T, SPS-RF), borrow pits.

###### Notes

Infrequent. Jun–Sep. Two varieties are recognized by Weakley (2012): var. *Ludwigialinearislinearis*, with the cells of the seed surface oriented parallel to the long axis of the seed, and var. *Ludwigialinearispuberula* Engelm. & A. Gray, with the cells of the seed surface oriented irregularly or elongated perpendicularly to the long axis of the seed. This character, best seen at ≥ 20× magnification, is the only non-overlapping morphological character that distinguishes the two varieties. If varieties are recognized, the specimens collected by the senior author would be referable to var. *Ludwigialinearislinearis*. A specimen collected from Sandy Run [Hancock] (Taggart SARU 379, WNC) has been reported as var. *Ludwigialinearispuberula* (Taggart 2010). Thornhill 30, 941, 1117, 1203 (NCSC). [= RAB; > Ludwigia linearis Walter var. linearis, Ludwigia linearis Walter var. puberula Engelm. & A. Gray sensu Weakley]

##### Ludwigia maritima

R.M. Harper

###### Ecological interactions

####### Conservation status

W7; S2S3, G5.

###### Distribution

Wet pine savannas (SPS-RF), adjacent roadsides.

###### Notes

Rare. Jun–Sep. Thornhill 1207 (NCSC). Specimens seen in the vicinity: Sandy Run: Taggart SARU 652 (WNC!). [= RAB, Weakley]

##### Ludwigia microcarpa

Michx.

###### Distribution

Wet pine savannas (VWLPS), adjacent roadsides.

###### Notes

Occasional. Jul–Oct. Thornhill 559, 845, 1440, 1524 (NCSC). Specimens seen in the vicinity: Sandy Run [Neck]: Wilbur 53660, 53690, 57653, 57665 (DUKE!); Sandy Run [RMK]: Taggart SARU 417 (WNC!). [= RAB, Weakley]

##### Ludwigia virgata

Michx.

###### Distribution

Wet pine savannas (WLPS, VWLPS).

###### Notes

Occasional. Jun–Sep. Thornhill 546, 554, 608, 624, 674 (NCSC). Specimens seen in the vicinity: Sandy Run [Neck]: Wilbur 53677 (DUKE!); Sandy Run [Patterson]: Taggart SARU 304 (WNC!). [= RAB, Weakley]

##### Oenothera fruticosa var. unguiculata

Fernald

###### Ecological interactions

####### Conservation status

W7; S2S3, G5T2T3.

###### Distribution

Wet pine savannas (WLPS, VWLPS).

###### Notes

Occasional. Apr–Aug. LeBlond 4976 (NCU!); Thornhill 364, 376, 424, 480, 565, 1050 (NCSC). Specimens seen in the vicinity: Sandy Run: LeBlond 4575, 4978 (NCU!); Taggart SARU 95 (WNC!). [< *Oenothera fruticosa* L. sensu RAB; = Weakley]

#### Orobanchaceae

##### Agalinis aphylla

(Nutt.) Raf.

###### Ecological interactions

####### Conservation status

W1; S3, G3G4.

###### Distribution

Wet pine savannas (WLPS, VWLPS).

###### Notes

Occasional. Sep–Oct; Oct–Nov. Thornhill 44, 1008, 1047 (NCSC). Specimens seen in the vicinity: Holly Shelter: Sorrie 8623 (NCU!); Sandy Run: Leonard 7601 (NCU!), Taggart SARU 458 (WNC!). [= RAB, Weakley]

##### Agalinis fasciculata

(Elliott) Raf.

###### Distribution

Wet pine savannas (SPS-RF).

###### Notes

Rare. Aug–Oct; Oct. Thornhill 1544 (NCSC). Specimens seen in the vicinity: Sandy Run [Hancock]: Taggart SARU 521 (WNC!); Sandy Run [Neck]: LeBlond 2587 (NCU!), Wilbur 57627 (DUKE!). [= RAB, Weakley]

##### Agalinis linifolia

(Nutt.) Britton

###### Ecological interactions

####### Conservation status

W1; S3, G4?.

###### Distribution

Wet pine savannas (WLPS, VWLPS).

###### Notes

Occasional. Aug–Sep; Sep–Oct. Thornhill 965, 977, 1037 (NCSC). Specimens seen in the vicinity: Sandy Run: Taggart SARU 454 (WNC!). [= RAB, Weakley]

##### Agalinis obtusifolia

Raf.

###### Ecological interactions

####### Conservation status

W1; S2S3, G4G5Q.

###### Distribution

Wet pine savannas (WLPS, VWLPS).

###### Notes

Infrequent. Sep–Oct; Oct–Nov. Thornhill 1083, 1264 (NCSC). Specimens seen in the vicinity: Sandy Run: Taggart SARU 471 (WNC!). [= RAB, Weakley]

##### Agalinis purpurea

(L.) Pennell

###### Distribution

Wet pine savannas (SPS-T, SPS-RF, WLPS, VWLPS).

###### Notes

Frequent. Aug–Oct; Sep–Nov. Thornhill 45, 1116, 1134, 1139, 1201 (NCSC). Specimens seen in the vicinity: Sandy Run [Hancock]: Taggart SARU 522 (WNC!); Sandy Run [Neck]: Wilbur 57644, 57662 (DUKE!). [= RAB, Weakley]

##### Agalinis setacea

(J.F. Gmel.) Raf.

###### Distribution

Wet pine flatwoods (WPF-T).

###### Notes

Sep–Oct; Oct–Nov. Reported from Shaken Creek by LeBlond (2000), but no specimens have been seen by the senior author. [= RAB, Weakley]

##### Agalinis virgata

Raf.

###### Ecological interactions

####### Conservation status

SR-P; S2, G3G4Q.

###### Distribution

Pine savannas.

###### Notes

Sep–Oct; Oct–Nov. Reported from Sandy Run by LeBlond and Weakley (1991), but no specimens have been seen in Shaken Creek Preserve by the senior author. [= RAB, Weakley]

##### Pedicularis canadensis

L.

###### Distribution

Pine savannas.

###### Notes

Apr–May; May–Jul. Not seen in Shaken Creek Preserve by the senior author. Specimens seen in the vicinity: Sandy Run [Neck]: Taggart SARU 577 (WNC!). [= RAB, Weakley]

##### Seymeria cassioides

(J.F. Gmel.) S.F. Blake

###### Distribution

Wet pine flatwoods (WPF-T), wet pine savannas (SPS-T, SPS-RF, WLPS, VWLPS).

###### Notes

Frequent. Aug–Oct. Thornhill 28, 33, 1048 (NCSC). Specimens seen in the vicinity: Sandy Run [Neck]: Taggart SARU 502 (WNC!); Wilbur 57650 (DUKE!). [= RAB, Weakley]

#### Parnassiaceae

##### Parnassia caroliniana

Michx.

###### Ecological interactions

####### Conservation status

State T, FSC; S2, G3.

###### Distribution

Wet pine savannas (WLPS, VWLPS), particularly along margins of adjacent swamps.

###### Notes

Frequent. Sep–Nov(–Dec). Thornhill 48, 1175 (NCSC). Specimens seen in the vicinity: Sandy Run: Taggart SARU 529 (WNC!). [= RAB, Weakley]

#### Phrymaceae

##### Mimulus ringens var. ringens

L.

###### Distribution

Pine savannas.

###### Notes

Jun–Sep. Not seen in Shaken Creek Preserve by the senior author. Specimens seen in the vicinity: Sandy Run [Haw’s Run]: Taggart SARU 453 (WNC!). [< *Mimulus ringens* L. sensu RAB; = Weakley]

#### Plantaginaceae

##### Nuttallanthus canadensis

(L.) D.A. Sutton

###### Distribution

Roadside margins of wet pine savannas (SPS-T).

###### Notes

Infrequent. Mar–May. Thornhill 224 (NCSC). Specimens seen in the vicinity: Sandy Run [Hancock]: Taggart SARU 42 (WNC!). [< *Linaria canadensis* (L.) Dum. Cours. sensu RAB; = Weakley]

##### Penstemon australis

Small

###### Distribution

Pine savannas, wet pine flatwoods.

###### Notes

May–Jul; Jul–Aug. Not seen in Shaken Creek Preserve by the senior author. Specimens seen in the vicinity: Highway 50: Wilbur 55337 (DUKE!). [< RAB; = Weakley]

##### Penstemon laevigatus

Aiton

###### Distribution

Wet pine savannas (VWLPS).

###### Notes

May–Jun; Jul–Aug. Not seen in Shaken Creek Preserve by the senior author. Specimens seen in the vicinity: Sandy Run [Hancock]: Taggart SARU 118 (WNC!; as *Penstemon australis*); Sandy Run [Neck]: Wilbur 55254, 55292 (DUKE!). [= RAB, Weakley]

##### Plantago sparsiflora

Michx.

###### Ecological interactions

####### Conservation status

State T, FSC; S1S2, G3.

###### Distribution

Pine savannas, adjacent roadsides.

###### Notes

Apr–Oct. Not seen in Shaken Creek Preserve by the senior author. Specimens seen in the vicinity: Sandy Run: LeBlond 4564 (NCU!); Leonard 8428 (NCU!); Leonard 8515 (DUKE!, NCSC!); Levy s.n. (DUKE!); Taggart SARU 108 (WNC!); Wilbur 53777, 55315 (DUKE!). [= RAB, Weakley]

##### Sophronanthe pilosa

(Michx.) Small

###### Distribution

Wet pine savannas (SPS-T, VWLPS), adjacent roadsides.

###### Notes

Occasional. Jun–Sep. Thornhill 538, 1304 (NCSC). Specimens seen in the vicinity: Sandy Run [Hancock]: Taggart SARU 648 (WNC!); Sandy Run [Neck]: Wilbur 53645, 53671, 53680, 57636 (DUKE!; as *Gratiola pilosa*). [= *Gratiola pilosa* Michx. sensu RAB; = Weakley]

#### Polygalaceae

##### Polygala brevifolia

Nutt.

###### Distribution

Wet pine savannas (SPS-T, SPS-RF, WLPS).

###### Notes

Infrequent. Jun–Oct. Thornhill 712, 908 (NCSC). Specimens seen in the vicinity: Sandy Run [Hancock]: Taggart SARU 400 (WNC!). [= RAB, Weakley]

##### Polygala cruciata

L.

###### Distribution

Wet pine savannas (SPS-RF, WLPS, VWLPS).

###### Notes

Frequent. Jun–Oct. Thornhill 414, 552, 598, 680, 744 (NCSC). Specimens seen in the vicinity: Highway 50: Wilbur 9427 (DUKE!); Sandy Run [Hancock]: Taggart SARU 266 (WNC!; as Polygala cruciata var. cruciata); Sandy Run [Neck]: Wilbur 53678 (DUKE!). [= RAB, Weakley]

##### Polygala hookeri

Torr. & A. Gray

###### Ecological interactions

####### Conservation status

SC-V; S2S3, G3.

###### Distribution

Wet pine savannas (WLPS, VWLPS).

###### Notes

Occasional. Jun–Aug. Thornhill 4, 476, 612 (NCSC). Specimens seen in the vicinity: Sandy Run: Taggart SARU 322 (WNC!). [= RAB, Weakley]

##### Polygala incarnata

L.

###### Distribution

Wet pine flatwoods (WPF-T), wet pine savannas (WLPS, VWLPS).

###### Notes

Occasional. Jun–Jul. Thornhill 544, 710, 760 (NCSC). Specimens seen in the vicinity: Sandy Run [Haw’s Run]: Taggart SARU 184 (WNC!), Wilbur 53705 (DUKE!). [= RAB, Weakley]

##### Polygala lutea

L.

###### Distribution

Wet pine flatwoods (WPF-T), wet pine savannas (SPS-T, SPS-RF, WLPS, VWLPS), adjacent roadsides.

###### Notes

Abundant. Apr–Oct. Thornhill 2, 199, 260, 315, 321, 412 (NCSC). Specimens seen in the vicinity: Sandy Run [Hancock]: Taggart SARU 80 (WNC!); Sandy Run [Neck]: Wilbur 53707 (DUKE!), Wyland s.n. (NCSC!). [= RAB, Weakley]

##### Polygala ramosa

Elliott

###### Distribution

Wet pine savannas (WLPS, VWLPS), adjacent roadsides.

###### Notes

Occasional. Jun–Sep. Thornhill 365, 404, 610, 671 (NCSC). Specimens seen in the vicinity: Sandy Run [Hancock]: Taggart SARU 135 (WNC!); Sandy Run [Neck]: Wilbur 53715 (DUKE!). [= RAB, Weakley]

##### Polygala verticillata

L.

###### Distribution

Dry woodlands, woodland borders, and openings.

###### Notes

Jun–Sep. Not seen in Shaken Creek Preserve by the senior author. Specimens seen in the vicinity: Sandy Run [Hancock]: Bradley 3388 (NCU!). [= Polygala verticillata var. verticillata sensu RAB; > Polygala verticillata var. isocycla Fernald, Polygala verticillata var. verticillata sensu Weakley]

#### Primulaceae

##### Lysimachia asperulifolia

Poir.

###### Ecological interactions

####### Conservation status

State E, Fed E; S3, G3.

###### Distribution

Wet pine flatwoods (WPF-T) and wet pine savannas (SPS-T), usually along margins of adjacent pond pine woodlands or pocosins.

###### Notes

Rare. May–Jun; Aug–Oct. Since only sterile individuals were seen on site by the senior author, no vouchers specimens were taken. Specimens seen in the vicinity: Holly Shelter: Sorrie 8452 (NCU!). [= *Lysimachia asperulaefolia* Poir. sensu RAB; = FNA, Weakley]

##### Lysimachia loomisii

Torr.

###### Ecological interactions

####### Conservation status

W1; S3, G3.

###### Distribution

Wet pine savannas (WLPS).

###### Notes

Rare. May–Jun; Aug–Oct. Thornhill 310, 345 (NCSC). Specimens seen in the vicinity: Sandy Run: Cooper 425 (WNC!), Taggart SARU 168 (WNC!). [= RAB, FNA, Weakley]

#### Ranunculaceae

##### Clematis crispa

L.

###### Distribution

Wet pine savannas (VWLPS), adjacent swamp margins.

###### Notes

Infrequent. Apr–Aug. Thornhill 561, 838 (NCSC). Specimens seen in the vicinity: Sandy Run [Hancock]: Taggart SARU 66 (WNC!). [= RAB, FNA, Weakley]

##### Thalictrum cooleyi

H.E. Ahles

###### Ecological interactions

####### Conservation status

State E, Fed E; S2, G2.

###### Distribution

Wet pine savannas (VWLPS).

###### Notes

Infrequent. Late Jun–early Jul; Aug–Oct. LeBlond 474 (stored in personal collection of the collector), Sorrie 9502 (NCU!). Specimens seen in the vicinity: Sandy Run: Ahles 58369 (NCU!), Gardner s.n. (NCU!), Taggart SARU 193 (WNC!). [= RAB, FNA, Weakley]

#### Rhamnaceae

##### Berchemia scandens

(Hill) K. Koch

###### Distribution

Wet pine savannas (VWLPS), adjacent swamp margins.

###### Notes

Infrequent. Apr–May; Aug–Oct. Thornhill 950, 1137 (NCSC). Specimens seen in the vicinity: Sandy Run [Hancock]: Taggart SARU 171 (WNC!); Sandy Run [Neck]: Wilbur 67085 (DUKE!). [= RAB, Weakley]

#### Rosaceae

##### Amelanchier canadensis

(L.) Medik.

###### Distribution

Wet pine flatwoods (WPF-T) and adjacent roadsides.

###### Notes

Rare. Mar–Apr; May–Jun. Thornhill 1258 (NCSC). Specimens seen in the vicinity: Sandy Run [Hancock]: Taggart SARU 111 (WNC!). [= RAB, Weakley]

##### Amelanchier spicata

(Lam.) K. Koch

###### Distribution

Dry, acidic, rocky sites.

###### Notes

Mar–Apr; May–Jun. Not seen in Shaken Creek Preserve by the senior author. Specimens seen in the vicinity: Sandy Run [Neck]: Wilbur 60087 (DUKE!). [= RAB, Weakley]

##### Aronia arbutifolia

(L.) Pers.

###### Distribution

Wet pine savannas (SPS-T, SPS-RF, WLPS, VWLPS).

###### Notes

Frequent. Mar–May; Sep–Nov. Thornhill 93, 96, 155 (NCSC). Specimens seen in the vicinity: Sandy Run [O’Berry]: Taggart SARU 17 (WNC!). [= Sorbus arbutifolia (L.) Heynh. var. arbutifolia sensu RAB; = Weakley]

##### Potentilla simplex

Michx.

###### Distribution

Pine savannas.

###### Notes

Apr–Jun; Apr–Jul. Not seen in Shaken Creek Preserve by the senior author. Specimens seen in the vicinity: Sandy Run [Hancock]: Taggart SARU 83 (WNC!). [= RAB, Weakley]

##### Prunus serotina var. serotina

Ehrh.

###### Distribution

Mesic pine savannas.

###### Notes

Apr–May; Jul–Aug. Not seen in Shaken Creek Preserve by the senior author. Specimens seen in the vicinity: Sandy Run [Haw’s Run]: Taggart SARU 558 (WNC!); Sandy Run [Neck]: Wilbur 63776 (DUKE!). [< RAB; = Weakley]

##### Rosa palustris

Marshall

###### Distribution

Wet pine savannas (VWLPS), particularly along margins of adjacent swamps.

###### Notes

Rare. May–Jul; Sep–Oct. Thornhill 1251 (NCSC). Specimens seen in the vicinity: Sandy Run [Haw’s Run]: Taggart SARU 330 (WNC!); Sandy Run [Neck]: Wilbur 55296 (DUKE!). [= RAB, Weakley]

##### Rubus cuneifolius

Pursh

###### Distribution

Wet pine flatwoods, mesic pine savannas.

###### Notes

Late Apr–early Jun; Jun–Jul. Not seen in Shaken Creek Preserve by the senior author. Specimens seen in the vicinity: Sandy Run [RMK]: Taggart SARU 227 (WNC!). [= RAB, Weakley]

##### Rubus pensilvanicus

Poir.

###### Distribution

Pine savannas (WLPS, VWLPS), particularly along roadsides or disturbed areas.

###### Notes

Infrequent. Apr–May; late May–Jul. Thornhill 200, 220, 1285, 123, 198, 444 (NCSC). Specimens seen in the vicinity: Sandy Run [RMK]: Taggart SARU 335 (WNC!; as R. argutus). [> *Rubus argutus* Link, *Rubus betulifolius* Small sensu RAB; = Weakley]

#### Rubiaceae

##### Diodia teres

Walter

###### Distribution

Scrapes in wet pine flatwoods (WPF-T), roadsides, and other dryish, disturbed areas.

###### Notes

Infrequent. Jun–Dec. Thornhill 900 (NCSC). Specimens seen in the vicinity: Sandy Run [Hancock]: Taggart SARU 351 (WNC!). [= RAB, Weakley]

##### Diodia virginiana

L.

###### Distribution

Wet pine savannas (WLPS, VWLPS), particularly along or near adjacent roadsides.

###### Notes

Frequent. Jun–Dec. Thornhill 280, 596, 984 (NCSC). Specimens seen in the vicinity: Sandy Run [Hancock]: Taggart SARU 136 (WNC!), Wilbur 53698 (DUKE!). [= RAB, Weakley]

##### Mitchella repens

L.

###### Distribution

Margins of wet pine savannas (VWLPS) and adjacent swamps.

###### Notes

Infrequent. May–Jun; Jun–Jul. Thornhill 835, 1262 (NCSC). Specimens seen in the vicinity: Sandy Run [RMK]: Taggart SARU 133 (WNC!). [= RAB, Weakley]

##### Oldenlandia uniflora

L.

###### Distribution

Wet pine savannas (SPS-RF), adjacent roadsides, margins of borrow pits.

###### Notes

Infrequent. Aug–Oct. Thornhill 903, 1115 (NCSC). [= RAB, Weakley]

#### Salicaceae

##### Salix caroliniana

Michx.

###### Distribution

Borrow pits within and roadside thickets adjacent to wet pine savannas (VWLPS).

###### Notes

Rare. Mar–Apr. Thornhill 243 (NCSC). Specimens seen in the vicinity: Sandy Run [Hancock]: Taggart SARU 254 (WNC!); Sandy Run [Neck]: Wilbur 63778, 63786, 63789, 67090 (DUKE!). [= RAB, FNA, Weakley]

#### Santalacaceae

##### Phoradendron leucarpum var. leucarpum

(Raf.) Reveal & M.C. Johnst.

###### Distribution

Parasitic on various trees (frequently on *Acer rubrum*) along margins of wet pine savannas (WLPS) and swamps.

###### Notes

Infrequent. Oct–Nov(–Mar); Nov–Jan(–May). Thornhill 90 (NCSC). Specimens seen in the vicinity: Sandy Run [Patterson]: Taggart SARU 562 (WNC!; as Phoradendron serotinum (Raf.) M. C. Johnst. ssp. serotinum). [< *Phoradendron serotinum* (Raf.) M.C. Johnst. sensu RAB; = Weakley]

#### Sapindaceae

##### Acer rubrum

L.

###### Distribution

Mesic pine savannas (MPS-CP), wet pine flatwoods (WPF-T), wet pine savannas (SPS-T, SPS-RF, WLPS, VWLPS), roadsides.

###### Notes

Abundant. Jan–Mar; Apr–Jul. If one chooses to recognize varieties within Acer rubrum, the specimens collected by the senior author are referable to var. Acerrubrumtrilobum Torr. & A. Gray ex K. Koch. Thornhill 80, 265, 281 (NCSC). Specimens seen in the vicinity: Sandy Run [Hancock]: Taggart SARU 8 (WNC!; as Acer rubrum var. trilobum); Sandy Run [Neck]: Wilbur 67089 (DUKE!). [= RAB; > *Acer rubrum* L. various varieties sensu Weakley]

#### Sarraceniaceae

##### Sarracenia flava

L.

###### Ecological interactions

####### Conservation status

W5B; S3S4, G5?.

###### Distribution

Wet pine savannas (SPS-T, SPS-RF, WLPS, VWLPS).

###### Notes

Frequent Mar–Apr; May–Jun. Thornhill 115, 137, 342, 343, 359, 391 (NCSC). Specimens seen in the vicinity: Sandy Run: Taggart SARU 56 (WNC!), Wyland s.n. (NCSC!). [= RAB, FNA, Weakley]

##### Sarracenia purpurea venosa

(Raf.) Fernald

###### Distribution

Wet pine savannas (SPS-T, SPS-RF, WLPS, VWLPS).

###### Notes

Frequent. Apr–May; Jun–Jul. Thornhill 114, 130, 174 (NCSC). Specimens seen in the vicinity: Sandy Run [Hancock]: Taggart SARU 81 (WNC!), Wyland s.n. (NCSC!). [< *Sarracenia purpurea* L. sensu RAB; = FNA, Weakley]

##### Sarracenia rubra rubra

Walter

###### Ecological interactions

####### Conservation status

W5B; S3, G4T3T4.

###### Distribution

Wet pine flatwoods (WPF-T).

###### Notes

Apr–May; Jun–Jul. Reported from Shaken Creek Preserve by LeBlond (2000), but no specimens have been seen on site by the senior author. Specimens seen in the vicinity: Sandy Run: Taggart SARU 568 (WNC!). [< *Sarracenia rubra* Walter sensu RAB; = FNA, Weakley]

#### Symplocaceae

##### Symplocos tinctoria

(L.) L’Hér.

###### Distribution

Wet pine flatwoods (WPF-T), wet pine savannas (WLPS, VWLPS).

###### Notes

Occasional. Mar–May; Aug–Sep. Thornhill 144, 765 (NCSC). Specimens seen in the vicinity: Sandy Run [Neck]: Wilbur 55258 (DUKE!); Sandy Run [Patterson]: Taggart SARU 52 (WNC!). [= RAB, FNA, Weakley]

#### Tetrachondraceae

##### Polypremum procumbens

L.

###### Distribution

Wet pine savannas (SPS-RF, VWLPS), adjacent roadsides.

###### Notes

Frequent. Late May–Sep; Aug–Oct. Thornhill 482, 547, 594, 949 (NCSC). Specimens seen in the vicinity: Sandy Run [Hancock]: Taggart SARU 373 (WNC!); Sandy Run [Neck]: Wilbur 53634, 57633 (DUKE!). [= RAB, Weakley]

#### Theaceae

##### Gordonia lasianthus

(L.) J. Ellis

###### Distribution

Wet pine flatwoods (WPF-T), wet pine savannas (SPS-T, SPS-RF).

###### Notes

Occasional. Jul–Sep; Sep–Oct. Thornhill 306 (NCSC). Specimens seen in the vicinity: Sandy Run [Hancock]: Taggart SARU 214 (WNC!). [= RAB, FNA, Weakley]

#### Violaceae

##### Viola brittoniana

Pollard

###### Ecological interactions

####### Conservation status

W7; S2?, G4G5.

###### Distribution

Margin of pine savanna (VWLPS) and adjacent swamp.

###### Notes

Rare. Apr–May. Thornhill 1261 (NCSC). [> Viola brittoniana var. brittoniana, Viola brittoniana var. pectinata (E.P. Bicknell) Alexander sensu RAB; = Weakley]

##### Viola lanceolata var. lanceolata

L.

###### Distribution

Wet pine savannas (WLPS, VWLPS).

###### Notes

Occasional. Mar–May. Thornhill 109, 1254 (NCSC). Specimens seen in the vicinity: Sandy Run [Haw’s Run]: Taggart SARU 109 (WNC!). [< *Viola lanceolata* L. sensu RAB; = Weakley]

##### Viola lanceolata var. vittata

(Greene) Weath. & Griscom

###### Distribution

Wet pine savannas (WLPS, VWLPS).

###### Notes

Infrequent. Feb–May. Thornhill 85 (NCSC). [< *Viola lanceolata* L. sensu RAB; = Weakley]

##### Viola primulifolia

L.

###### Distribution

Wet pine savannas (SPS-T, SPS-RF, WLPS, VWLPS).

###### Notes

Frequent. Mar–May. Thornhill 75, 91, 175 (NCSC). Specimens seen in the vicinity: Sandy Run [Hancock]: Taggart SARU 5 (WNC!). [= RAB, Weakley]

##### Viola sagittata var. sagittata

Aiton

###### Distribution

Wet pine flatwoods (WPF-T), wet pine savannas (VWLPS).

###### Notes

Infrequent. Apr. Thornhill 86, 88, 106, 110 (NCSC). Specimens seen in the vicinity: Sandy Run [Haw’s Run]: Taggart SARU 54 (WNC!; as *Viola sagittata*). [> *Viola sagittata*, *Viola emarginata* (Nutt.) Leconte sensu RAB; = Weakley]

##### Viola septemloba

Leconte

###### Distribution

Wet pine savannas (WLPS, VWLPS).

###### Notes

Infrequent. Late Mar–early May. Thornhill 95, 139 (NCSC). [< RAB; = Weakley]

##### Viola sororia var. missouriensis

(Greene) L.E. McKinney

###### Distribution

Wet pine flatwoods (WPF-T), wet pine savannas (WLPS, VWLPS).

###### Notes

Occasional. Mar–May. Thornhill 122, 1257, 1260 (NCSC). Specimens seen in the vicinity: Sandy Run [Neck]: LeBlond 1938 (NCU!; as *Viola affinis*); Sandy Run [Patterson]: Taggart SARU 573 (WNC!; as *Viola affinis*). [= *Viola affinis* Leconte sensu RAB; = Weakley]

#### Vitaceae

##### Parthenocissus quinquefolia

(L.) Planch.

###### Distribution

Wet pine flatwoods (WPF-T), margins of wet pine savannas (VWLPS) and adjacent swamps.

###### Notes

Infrequent. May–Jul; Jul–Aug. Thornhill 974 (NCSC). Specimens seen in the vicinity: Sandy Run [Hancock]: Taggart SARU 200 (WNC!). [= RAB, Weakley]

##### Vitis rotundifolia var. rotundifolia

Michx.

###### Distribution

Wet pine savannas (WLPS, VWLPS), particularly along swamp margins or near roadsides.

###### Notes

Infrequent. May–Jun; Aug–Oct. Thornhill 331, 1092, 1240 (NCSC). Specimens seen in the vicinity: Sandy Run [Hancock]: Taggart SARU 203 (WNC!). [< *Vitis rotundifolia* sensu RAB; = Muscadinia rotundifolia (Michx.) Small var. rotundifolia sensu Weakley]

## Identification Keys

### KEYS TO THE MAJOR VASCULAR PLANT GROUPS

**Table:** 

1	Plant reproducing by spores	**Pteridophytes**
–	Plant reproducing by seeds	2
2	(1’.) Seeds borne on cones (fleshy and berry-like in Juniperus virginiana var. virginiana); leaves needle-like or scale-like, < 3 mm wide	**Gymnosperms**
–	Seeds borne in fruits; leaves various	3
3	(2’.) Plant exhibiting ≥ 2 of the following characters: cotyledon 1; stem vascular bundles scattered; leaves parallel veined; floral parts in 3s	**Monocotyledons**
–	Plant exhibiting ≥ 2 of the following characters: cotyledons 2; stem vascular bundles in a ring; leaves not parallel veined; floral parts in 4s or 5s	**Basal angiosperms, magnoliids, eudicotyledons**

Key adapted from Radford et al. (1968).

### PTERIDOPHYTES

**Table:** 

1	Leaves simple, scale-like, < 2 cm long, each leaf with 1, unbranched vein; sporangia borne in strobili at the tips of shoots	2
–	Leaves pinnatifid to 2-pinnate, “ferny”, > 2 cm long, each leaf bearing numerous pinnately-branched veins; sporangia borne in sori on the undersides of modified or unmodified pinnae	3
2	(1.) Strobili cylindrical, 3–20 mm wide; spores of one size; sporophylls of similar size	Lycopodiaceae
–	Strobili quadrangular or flattened, 1–2.5(–3.5) mm wide; spores dimorphic, megaspores larger and borne in larger sporangia than microspores; sporophylls somewhat dimorphic, the basal (megasporophylls) usually larger than proximal (microsporophylls)	Selaginellaceae [*Selaginella apoda*] Fig. 13
3	(1’.) Stipules present, wing-like; sori and indusia lacking	Osmundaceae
–	Stipules absent; sori and indusia present (only false indusia apparent in Pteridium aquilinum var. pseudocaudatum)	4
4	Sori borne along midribs of pinnae and pinnules, discrete, in chainlike rows, with true indusia; leaves lanceolate in outline, pinnatifid to pinnate-pinnatifid, pinnae lacking distinct caudate tips	Blechnaceae [*Woodwardia*]
–	Sori marginal, essentially continuous, covered by false indusia; leaves triangular to ovate in outline, 2-pinnate, pinnae with distinct caudate tips	Dennstaedtiaceae [Pteridium aquilinum var. pseudocaudatum] Fig. 14

Key adapted from Radford et al. (1968), Smith (1993), and Weakley (2012).

Note: Understanding some basic terminology is critical to the successful use of the following keys to Pteridophytes families, genera, and species. Pinnate indicates lobing (usually of leaves or leaf segments) entirely to the midrib, whereas pinnatifid indicates lobing to *near* the midrib. Pinnate-pinnatifid refers to a leaf that is once-pinnate and whose segments (pinnae) are themselves pinnatifid. The spore-producing structures of many ferns are borne in masses called sori, which may be either exposed or covered by the margin of the leaves (a false indusium) or a separate structure (a true indusium). Leaf-like structures that bear sporangia are called sporophylls; these may be similar to the sterile leaves or may be highly modified (e.g., the compacted, cone-like structures, or strobili, of Lycopodiaceae).

### [Blechnaceae] *Woodwardia* Sm.

**Table:** 

1	Sterile and fertile leaves dissimilar, fertile taller and with much narrower pinnae; sterile leaves pinnatifid, pinnae therefore appearing connected at the bases; sterile pinnae finely serrate, unlobed, with veins forming two or more rows of areoles (interconnecting loops) between the midribs and the margins	*Woodwardia areolata*
–	Sterile and fertile leaves similar; sterile leaves pinnate, pinnae therefore separate; sterile pinnae entire, pinnatifid, with veins forming a single row of areoles between the midribs and the margins	*Woodwardia virginica*

Key adapted from Radford et al. (1968) and Cranfill (1993).

Fig. 15

### Lycopodiaceae

**Table:** 

1	Erect stems not strongly differentiated, peduncle bearing leaves of similar size to (or only slightly smaller than) those of the strobilus; leaves of the erect stem spirally arranged, not reduced to scales, spreading, overlapping; leaves of the horizontal stem 0.5–1.2 mm wide, ciliate-denticulate or entire	* Lycopodiella *
–	Erect stems strongly differentiated into a nearly bare peduncle and a leafy strobilus; leaves of the erect stem whorled, reduced, scale-like, usually appressed, not overlapping; leaves of the horizontal stem 1.3–2.1 mm wide, entire	*Pseudolycopodiella caroliniana* Fig. 16

Key adapted from Radford et al. (1968), Wagner and Beitel (1993), and Weakley (2012).

### [Lycopodiaceae] *Lycopodiella* Holub

**Table:** 

1	Fertile leaves of strobilus spreading at maturity, toothed, some or all teeth ≥ 0.3 mm long; strobili 12–20 mm wide, 3–6 mm wider than subtending stem; horizontal stems strongly arching	*Lycopodium alopecuroides*
–	Fertile leaves of strobilus appressed at maturity, entire or with teeth < 0.3 mm long; strobili 3–6 mm wide, 0–2 mm wider than subtending stem; horizontal stems appressed to ground throughout their length	*Lycopodiella appressa*

Key adapted from Wagner and Beitel (1993), Weakley (2012).

Fig. 17

### Osmundaceae

**Table:** 

1	Fertile leaves dimorphic, lower pinnae sterile, relatively broad, upper pinnae fertile, reduced; sterile leaves 2-pinnate, lacking tufts of orangish hairs near base of pinnae	*Osmunda spectabilis* Fig. 18
–	Fertile leaves monomorphic, all pinnae fertile, reduced; sterile leaves pinnate-pinnatifid, with persistent tufts of orangish hairs near base of pinnae	*Osmundastrum cinnamomeum* Fig. 19

Key adapted from Whetstone and Atkinson (1993), Weakley (2012).

### GYMNOSPERMS

**Table:** 

1	Leaves scale-like or needle-like, < 1.5 cm long, not in fascicles; cones berry-like or woody, scales valvate or imbricate, if imbricate then leaves opposite and scale-like; seeds 1–3 per scale	Cupressaceae
–	Leaves needle-like, (10–)12–45 cm long, in fascicles of 2–3 leaves; cones woody, scales imbricate; seeds 2 per scale	Pinaceae [*Pinus*]

Key adapted from Eckenwalder and Thieret (1993).

### Cupressaceae

**Table:** 

1	Leaves linear, 3–17 mm long, alternate, deciduous; cones woody; seeds (1–)2 per scale	* Taxodium *
–	Leaves scale-like, 1–3 mm long, opposite or whorled, evergreen; cones berry-like or woody; seeds 1–2(–3) per scale	2
2	Plants monoecious; mature female cones woody, 4–9 mm broad; branchlets generally arrayed in one plane, creating a “fan-like” appearance	*Chamaecyparis thyoides* Fig. 20
–	Plants dioecious; mature female cones fleshy and berry-like, 3–6(–7) mm broad; branchlets arrayed in numerous planes, creating a “bushy” appearance	Juniperus virginiana var. virginiana Fig. 21

Key adapted from Watson and Eckenwalder (1993), Weakley (2012).

### [Cupressaceae] *Taxodium* Rich.

**Table:** 

1	Leaves mostly vertically ascending, appressed and overlapping, spirally arranged; branchlets ascending from twigs, secundly erect; bark 1–2.5 cm thick, furrowed, dark-brown, not exfoliating; larger knees short, rarely > 4 dm tall, with thick, compact bark on top; trees of isolated depressions, wet savannas, pocosins, other wet peaty habitats, and, less commonly, blackwater swamps	*Taxodium ascendens*
–	Leaves pendent to horizontally spreading to laterally divergent, spirally arranged but generally appearing distichous (“featherlike”); bark < 1 cm thick, exfoliating in shreddy, orange-brown strips; larger knees often tall, frequently > 4 dm tall, with thin, shreddy bark on top; trees of blackwater swamps (and other habitats outside of SCP, including brownwater swamps, natural lakes, and millponds)	*Taxodium distichum*‡

Key adapted from Watson (1993), Weakley (2012).

Note: *Taxodium distichum* (L.) Rich. has not been found in savannas or flatwoods on site, though it has been seen in swamps. Nevertheless, it is included in the key below (where indicated by a double-dagger symbol, ‡) to facilitate the distinguishing of it from *Taxodium ascendens* Brongn., an occasional component of the wettest savannas. In the following key leaf and branchlet characters of *Taxodium ascendens* refer to mature trees; foliage of juvenile trees often mimics that of *Taxodium distichum*. Leaf and branchlet characters of *Taxodium distichum* refer to both mature and juvenile trees; however, in the crowns of mature *Taxodium distichum*, leaf and branchlet characters sometimes mimic those of *Taxodium ascendens*. For these reasons, accurate identification of the two species often requires observation of other, non-foliage features, including the stature of the “knees”, the thickness and texture of the bark, and the habitat in which the plant grows.

Fig. 22

### [Pinaceae] *Pinus* L.

**Table:** 

1	Cones about as broad as long, top-shaped, 3–6 cm long, serotinous; trunks typically producing adventitious sprouts (“epicormic branches”), especially in reponse to fire	*Pinus serotina*
–	Cones distinctly longer than broad, not top-shaped, collectively (5–)6–25 cm long, not serotinous; trunks not producing adventitious sprouts	2
2	(1’.) Leaves 20–45 cm long; mature seed cones 15–25 cm long; terminal buds ovoid, 3–4 cm long, scales silvery white, margins fringed	*Persea palustris*
–	Leaves (10–)12–20(–23) cm long; mature seed cones 6–18(–20) cm long; terminal buds cylindric, 1–2 cm long, silvery brown or reddish brown, margins fringed or entire	3
3	(2’.) Seed cones glossy, on stalks 1.5–3 cm long; leaves dark green, glossy, in fascicles of 2 and 3	*Pinus elliottii var. elliottii
–	Seed cones dull, sessile; leaves yellowish-green, dull, in fascicles of (2)3	*Pinus taeda*

Key adapted from Radford et al. (1968), Kral (1993), and Weakley (2012).

Fig. 23

### MONOCOTYLEDONS

**Table:** 

1	Plant an epiphyte; roots absent	Bromeliaceae [*Tillandsia usneoides*] Fig. 24
–	Plant terrestrial or aquatic, not an epiphyte; roots present	2
2	(1’.) Plant a vine or liana, climbing by twining or by stipular tendrils	3
–	Plant an herb or shrub, erect or prostrate, but not climbing	4
3	(2.) Plant climbing by twining; leaves with 9–11 palmate veins; ovary inferior; fruit a capsule	Dioscoreaceae [*Dioscorea villosa*] Fig. 25
–	Plant climbing by stipular tendrils; leaves with 3–5 palmate veins; ovary superior; fruit a berry	Smilacaceae [*Smilax*]
4	(2’.) Primary inflorescences of (1–)2–many-flowered spikelets (consisting of reduced flowers, each subtended by 1–many scales, scales spirally or distichously arranged), spikelets variously arranged in dense to diffuse spikes, racemes, or panicles; perianth absent or reduced to chaff, scales, bristles, or paddle-like structures; fruit 1-seeded; [grasses and sedges]	5
–	Primary inflorescences not of spikelets; perianth present, large or small but not reduced to chaff, scales, bristles, or paddle-like structures (reduced to scales in Eriocaulaceae, with flowers borne in dense, white, gray, or yellowish-tan heads terminating stems); fruit ≥ 1-seeded	6
5	(4.) Margins of leaf sheaths fused from base to apex, not split apically (rarely and irregularly split in age); culms solid, usually triangular in cross-section; leaves 3-ranked (reduced to sheaths and not evidently 3-ranked in *Eleocharis*); fruit subtended by 1 scale	Cyperaceae
–	Margins of leaf sheaths not fused from base to apex, split (and generally overlapping) apically; culms usually solid, terete in cross-section; leaves usually 2-ranked; fruit usually subtended by 2 scales (lemma and palea)	Poaceae
6	(4’.) Leaves terminating in a stiff, spinose apex, margins fraying into twisted, filamentous threads; plants woody or suffrutescent	Agavacaeae [*Yucca filamentosa*] Fig. 26
–	Leaves not terminating in a stiff, spinose apex, margins not fraying into filamentous threads; plants herbaceous	7
7	(6’.) Leaves 3, whorled at apex of stem, closely subtending flower; flowers solitary	Trilliaceae [Trillium pusillum var. pusillum] Fig. 27
–	Leaves various but not as above; flowers usually numerous, rarely solitary	8
8	(7’.) Inflorescences of dense, white, gray, or yellowish-tan heads; flowers small, individually indistinct	Eriocaulaceae
–	Inflorescences various, not of dense, white or grayish heads; flowers relatively large, or small but individually distinct	9
9	(8’.) Inflorescence of variously shaped, compact spikes terminating a scape; flowers and fruits subtended by imbricate scales; petals yellow, strongly clawed, blade flat, opening and withering within 1 day	Xyridaceae [*Xyris*]
–	Inflorescence not as above; flowers and fruits not subtended by imbricate scales; petals not yellow, or if yellow then campanulate and remaining open > 1 day	10
10	(9’.) Ovary superior or partly inferior	11
–	Ovary inferior	18
11	(10.) Sepals and petals bract-like, chartaceous (with a dry, papery texture), persistent and not withering even after fruiting	Juncaceae [*Juncus*]
–	Sepals and petals not bract-like, membranous to subcoriaceous, not persistent, or persistent but withering before fruiting, variously colored but not brown or green (green or greenish-yellow in *Uvularia puberula*)	12
12	(11’.) Sepals and petals conspicuously clawed, predominantly crimson (rarely pinkish), bases yellow and often with purplish spots	Liliaceae [*Lilium catesbaei*] Fig. 28
–	Sepals and petals not clawed, white, yellow, green, blue, purple, or pinkish, bases not differentiated in color, lacking purplish spots	13
13	(12’.) Flowers axillary, 1–3 per stem; perianth green or greenish-yellow	Colchicaceae [*Uvularia puberula*] Fig. 29
–	Flowers borne in spikes, racemes, panicles, thyrses, or umbels, many per stem; perianth white, yellow, blue, purple, or pinkish	14
14	(13’.) Inflorescence an umbel; perianth purple to pinkish	Amaryllidaceae [*Allium* species 1] Fig. 30
–	Inflorescence a spike, raceme, panicle, or thyrse; perianth white, yellow, or orange	15
15	(14’.) Flowers imperfect, plants dioecious; staminate inflorescence a raceme (rarely a spike), pistillate inflorescence a raceme or spike; leaves oblanceolate, 1.5–6 cm wide	Heloniadaceae [*Chamaelirium luteum*] Fig. 31
–	Flowers perfect, plants hermaphroditic; inflorescence a raceme, thyrse, or panicle; leaves linear or lanceolate, < 2.8 cm wide	16
16	(15’.) Perianth campanulate, outer surface farinose (with a mealy texture)	Nartheciaceae [*Aletris*]
–	Perianth broadly spreading, outer surface not farinose	17
17	(16’.) Leaves radial, to 32 mm wide; inflorescences panicles or racemes; tepals with greenish or yellow glands at base, or glands absent	Melanthiaceae
–	Leaves equitant (sometimes somewhat radial in *Pleea tenuifolia*, with flowers subtended by conspicuous, spathelike bracts and plants often forming dense cushions), to 6 mm wide; inflorescences racemes or thyrses; tepals lacking greenish or yellow glands at base	Tofieldiaceae
18	(10’.) Roots and rhizomes red; perianth densely wooly	Haemodoraceae [*Lachnanthes caroliniana*] Fig. 32
–	Roots and rhizomes brown, black, white, or yellowish; perianth glabrous or pilose basally, not densely wooly	19
19	(18’.) Perianth zygomorphic	Orchidaceae
–	Perianth actinomorphic	20
20	(19’.) Perianth yellow	Hypoxidaceae [*Hypoxis*]
–	Perianth blue, purple, or white	21
21	(20’.) Plants very slender, inconspicuous even when flowering; perianth 2–6 mm long, connate from base to near apex, lobes 0.3–0.5 mm long; flowers in capitate clusters or solitary	Burmanniaceae [*Burmannia capitata*]
–	Plants somewhat slender to robust, conspicuous, at least when flowering; perianth 5–100+ mm long, distinct, or connate only basally with perianth lobes ≥ 5 mm long; flowers in spikes, in fascicles within spathes, or solitary	Iridaceae

Key adapted from Radford et al. (1968), Weakley (2012).

### Cyperaceae

**Table:** 

1	Achenes enclosed in a perigynium; flowers unisexual	* Carex *
–	Achenes not enclosed in a perigynium; flowers unisexual or bisexual	2
2	(1’.) Achene white or grayish, subtended by a hypogynium (a distinctive collar or ridge of a different texture or color than the achene body) or not	* Scleria *
–	Achene brown, red, or tannish, not subtended by a hypogynium	3
3	(2’.) Leaves absent; spikes 1 per culm, terminal	* Eleocharis *
–	Leaves present; spikes ≥ 1 per culm, terminal or axillary	4
4	(3’.) Spikelet scales distichous	5
–	Spikelet scales spirally arranged, imbricate	7
5	(4.) Inflorescence axillary; leaves prominently 3-ranked, cauline; perianth bristles 6–9	*Dulichium arundinaceum* Fig. 33
–	Inflorescence terminal; leaves not noticeably 3-ranked, predominantly basal; perianth bristles absent	6
6	(5’.) Inflorescence unbranched; spikes 1–4, sessile; spikelet scales 1–3, conspicuously keeled	*Kyllinga odorata* Fig. 34
–	Inflorescence branched; spikes usually numerous, pedunculate; spikelet scales 5–many, rounded	* Cyperus *
7	(4’.) Style base hardened, differentiated from achene body, persistent as a tubercle at apex of achene	8
–	Style base not as above; tubercle absent	9
8	(7.) Apex of leaf sheaths fimbriate-ciliate; perianth bristles absent; leaves capillary, 0.5 mm wide	* Bulbostylis *
–	Apex of leaf sheaths glabrous; perianth bristles usually present; leaves capillary or broad, 0.5–15 mm wide	* Rhynchospora *
9	(7’.) Perianth bristles present	10
–	Perianth bristles absent	12
10	(9.) Perianth scales 3, stalked, paddle-shaped; perianth bristles 3	* Fuirena *
–	Perianth scales absent; perianth bristles usually 4–8	11
11	11. (10’.) Culm strongly 3-sided, edges sharp; cauline leaves absent; inflorescence congested; inflorescence bract 1, appearing as a continuation of the culm	Schoenoplectus pungens var. pungens Fig. 35
–	Culms terete or with rounded edges; cauline leaves 5–10; inflorescence diffuse, nodding; inflorescence bracts numerous, the largest appearing similar to cauline leaves	*Scirpus cyperinus*
12	(9’.) Style fringed along margins	* Fimbristylis *
–	Style entire along margins	13
13	(12’.) Culms > 50 cm tall; inflorescence branched; spikes numerous, some pedunculate; plant perennial	* Cladium *
–	Culm 1–25 cm tall; inflorescence unbranched; spikes 1–3, sessile; plant annual	*Isolepis carinata*

Key adapted from Radford et al. (1968), Ball et al. (2002), and Weakley (2012).

### [Cyperaceae] *Bulbostylis* Kunth

**Table:** 

1	Inflorescence diffuse, umbelliform, not exceeded by bracts; spikelets pedicellate, fertile scales 1–1.3 mm long	*Bulbostylis ciliatifolia*+
–	Inflorescence compact, more-or-less capitate, exceeded by at least some bracts; spikelets typically sessile (rarely a few short-pedicellate), fertile scales 3–4 mm long	*Bulbostylis stenophylla*

Key adapted from Kral (2002c), Weakley (2012).

Note: *Bulbostylis ciliatifolia* (Elliott) Fernald, though not seen in or reported from the study area, is likely to occur in wet savannas and adjacent roadsides and is therefore included in the key below, where indicated with a plus (+) symbol.

### [Cyperaceae] Carex L.

**Table:** 

1	Perigynia and leaf sheaths glaucous; plants fruiting Jul–Aug	*Carex glaucescens*
–	Perigynia and leaf sheaths not glaucous; plants collectively fruiting late Mar–Jul	2
2	(1’.) Style deciduous, jointed at base	3
–	Style persistent, not jointed at base	8
3	(2.) Spikes solitary, similar, androgynous (staminate flowers above the pistillate flowers in the same inflorescence); perigynia 0.8–1.3 mm wide; pistillate scales whitish	Carex leptalea ssp. harperi
–	Spikes numerous, dissimilar, terminal spikes staminate or rarely gynecandrous (staminate flowers below the pistillate flowers in the same inflorescence), narrow, lateral spikes pistillate, broader; perigynia 1.2–3.3 mm wide; pistillate scales reddish, yellow, brown, or green (margins white in *Carex physorhyncha*)	4
4	(3’.) Leaf blades 12–14 mm wide; perigynia 25–32-veined	*Carex chapmanii*
–	Leaf blades 0.8–7.2 mm wide; perigynia 0–22-veined	5
5	(4’.) Perigynia veinless, 2.7–3.1 mm long, 0.8–1.1 mm wide; pistillate scale margins white, differentiated from scale body; culms 10–45 cm long	*Carex physoryncha*
–	Perigynia 8–22-veined, 3.5–9 mm long, 1.2–3.3 mm wide; pistillate scale margins yellowish, brown or green, not distinctly whitened and differentiated from scale body; culms 30–110(–125) cm long	6
6	(5’.) Perigynia conspicuously red-dotted, well-spaced along rachis, 1–3 mm apart, rachis clearly visible between perigynia; spikes nodding or erect	*Carex venusta*
–	Perigynia not red-dotted, congested on rachis, rachis not visible between perigynia; spikes erect	7
7	(6’.) All perigynia ascending; pistillate spikes cylindrical, 2.5–3.5 cm long	*Carex striata*
–	Basal perigynia strongly reflexed; pistillate spikes subgloblose to short-cylindric, 0.8–1.1 cm long	*Carex lutea*
8	(2’.) Perigynia narrowly ovate, tapering somewhat abruptly and forming a distinct, narrowed beak, 7–11-veined, 5.6–8.8 mm long, 2.5–3.5 times as long as wide	*Carex elliottii*
–	Perigynia lanceolate, gradually tapering to the apex and not forming a distinct, narrowed beak, 20–26 veined, (8.3–)10.5–13.5(–15) mm long, 4–7 times as long as wide	*Carex lonchocarpa*

Key adapted from Radford et al. (1968), Ball et al. (2002).

References: Cochrane (2002), Crins (2002), Crins and J.H. (2002), Reznicek (2002), Reznicek and Catling (2002), Reznicek and Ford (2002), Standley (2002)

Fig. 36

### [Cyperaceae] Cladium P. Browne

**Table:** 

1	Culms 1–3 m tall, 5–10 mm wide; leaf blades 5–11 mm wide, margins harshly scabrous, the teeth apparent without magnification; rhizomes short, upright stems therefore forming dense clumps	*Cladium jamaicense*
–	Culms 0.3–1 m tall, 1–2 mm wide; leaf blades 2–3 mm wide, margins and lower surface of midvein smooth to slightly scabrous, the teeth (when present) visible only with magnification; rhizomes creeping, the upright stems therefore scattered in loose colonies	*Cladium mariscoides*

Key adapted from Tucker (2002), Weakley (2012).

Fig. 37

### [Cyperaceae] Cyperus L.

**Table:** 

1	Spikelets borne in open, digitate clusters; leaves usually reduced to sheaths	*Cyperus haspan*
–	Spikelets borne in dense, headlike clusters or spicately along a conspicuous rachis (rachis only 2–5 mm long and therefore somewhat inconspicuous in *Cyperus compressus*); leaves not reduced to sheaths, bearing conspicuous blades	2
2	(1’.) Culms obtusely trigonous to terete, scaberulous; adaxial leaf blade surface densely hirtellous (bearing small, stiff hairs); spikes turbinate (top-shaped: broadest at apex, narrowing to base), most spikelets reflexed (distalmost may be spreading), proximal nearly parallel to inflorescence ray (peduncle), inflorescence rays scaberulous	*Cyperus plukenetii*‡
–	Culms sharply trigonous, glabrous; adaxial leaf blade surface glabrous; spikes cylindric or globose, spikelets spreading or ascending (proximal spikelets may be somewhat reflexed but never nearly parallel to inflorescence ray), inflorescence rays glabrous	3
3	(2’.) Spikelets with (8–)16–36(–42) scales, scales strongly distichous, spreading, deciduous	*Cyperus compressus*‡
–	Spikelets with (1–)2–5(–6) scales, scales not strongly distichous, appressed, persistent	4
4	(3’.) Leaf blades flat in cross-section; spikelets (10–)30–50, 4–8 mm long; achene 2–2.4 mm long	*Cyperus croceus*‡
–	Leaf blades V- or M-shaped in cross section; spikelets 40–120, 2.2–4(–4.5) mm long; achene 1.2–1.7 mm long	*Cyperus retrorsus*‡

Key adapted from Radford et al. (1968), Tucker et al. (2002).

Note: *Cyperus* appears to be at most only a minor component of the savanna and flatwood flora in Shaken Creek Preserve and in the vicinity. At Shaken Creek Preserve, no *Cyperus* species have been outside of disturbed areas; of the nine *Cyperus* species reported from Sandy Run by Taggart (2010), only one, *Cyperus haspan* L., was reported from pine savannas or flatwoods. Nevertheless, a few *Cyperus* species could be found in savannas or flatwoods, and the following key attempts to accommodate such discovery. Included in the key below are those species seen by the senior author growing in disturbed areas near savannas or flatwoods in Shaken Creek Preserve and also those species reported by Taggart (2010) as growing in disturbed areas but whose habitat description in Weakley (2012) includes savannas and/or flatwoods. Such taxa are indicated by a double-dagger symbol (‡).

Fig. 38

### [Cyperaceae] Eleocharis R. Br.

**Table:** 

1	Culm as broad or broader than spike, nodose-septate	*Eleocharis equisetoides*
–	Culm narrower than spike, not nodose-septate	2
2	(1’.) Achenes coarsely reticulate, tubercle at least as long and wide as achene, 0.9–1.7(–2.4) mm long, 0.7–2(–2.2) mm wide; plants not producing vegetative proliferations at the tips of arching culms	*Eleocharis tuberculosa*
–	Achenes smooth to finely reticulate, tubercle not nearly as long and wide as achene, 0.1–0.5 mm long, 0.1–0.8 mm wide; plants producing vegetative proliferations at the tips of arching culms or not	3
3	(2’.) Spike (2–)5–13 mm long, (2–)3–4 mm wide; styles 2- or 3-fid; perianth bristles exceeding tubercle; achenes lenticular, 0.9–1.2(–1.3) mm long, 0.7–0.9 mm wide	*Eleocharis obtusa*
–	Spike 1–2 mm long, 0.5–2 mm wide; styles 3-fid; perianth bristles shorter than or equaling achene; achenes trigonous or subterete, 0.5–0.9 mm long, 0.1–0.5 mm wide	4
4	(3’.) Floral scales distichous, (1.5–)2–5 mm long	*Eleocharis baldwinii*
–	Floral scales spirally imbricate, 0.8–1.5 mm long	Eleocharis microcarpa var. filiculmis

Key adapted from Smith et al. (2002), Weakley (2012).

Fig. 39

### [Cyperaceae] Fimbristylis Vahl

**Table:** 

1	Plants perennial, rhizomatous; culms to 100 cm tall, bases swollen; fertile scales 2.5–3.5 mm long, puberulent at least distally	Fimbristylis puberula var. puberula
–	Plants annual, not rhizomatous; culms 5–50 cm tall, bases not swollen; fertile scales 1.5–2 mm long, glabrous	2
2	(1’.) Styles 2-fid; achene lenticular; culms 5–50 cm tall; leaf blades 1–1.5(–2) mm wide	*Fimbristylis annua*‡
–	Styles 3-fid; achene trigonous; culms 5–20(–30) cm tall; leaf blades 1–3 mm wide	*Fimbristylis autumnalis*‡

Key adapted from Kral (2002a), Weakley (2012).

Note: *Fimbristylis annua* (All.) Roem. & Schult., collected along Old Maple Hill Road (*Ahles 32497*, NCU!), and *Fimbristylis autumnalis* (L.) Roem. & Schult., collected in a roadside in Sandy Run [Neck] (*Taggart SARU 555* (WNC!), have not been collected in or reported from savannas or flatwoods in Shaken Creek Preserve or in the vicinity. Nevertheless, since Fimbristylis puberula (Michx.) Vahl var. puberula, the common *Fimbristylis* of savannas and flatwoods, can also occasionally occur in roadsides, all three species are included in the key below.

Fig. 40

### [Cyperaceae] Fuirena Rottb.

**Table:** 

1	Plants perennial, rhizomatous; perianth bristles < ½ length of achene body, usually smooth	*Fuirena breviseta*
–	Plants annual, cespitose; perianth bristles > ½ length of achene body, retrorsely-barbellate	*Fuirena pumila*

Key adapted from Kral and Persoon (2002), Weakley (2012).

Fig. 41

### [Cyperaceae] Rhynchospora Vahl

**Table:** 

1	Tubercle 10–20(–21) mm long; styles simple or bifid apically; plants robust, 0.8–2 m tall	1
–	Tubercle < 3 mm long; styles 2; plants slender, usually ≤ 1 m tall	3
2	(1.) Bristles shorter than achene body	*Rhynchospora corniculata*
–	Bristles greatly exceeding (ca. 2× longer than) achene body	*Rhynchospora macrostachya*
3	(1’.) Inflorescence bracts basally white, apically green, broad, numerous	4
–	Inflorescence bracts green throughout (golden or brown in age), slender to broad, 1–numerous	5
4	(3.) Basal inflorescence bract (1.4–)2–5 mm wide, the white portion (2.5–)9–25 mm long, forming a diffuse, irregular boundary at junction with green portion; achene 1.0–1.2 mm wide, tubercle decurrent onto margins of achene; plants to 0.7 m tall	*Rhynchospora colorata*
–	Basal inflorescence bract 5–12 mm wide, the white portion 22–55 mm long, forming an abrupt boundary at junction with green portion; achene 1.2–1.5 mm wide, tubercle truncate at base; plants to 1 m tall	*Rhynchospora latifolia*
5	(3’.) Bristles plumose at least basally; leaf blades ≤ 1.5 mm wide	6
–	Bristles not plumose, or bristles absent; leaf blades of various widths, 0.2–7 mm wide	8
6	(5.) Spikelets 2–4 mm long, sessile, usually densely clustered; achene 1.4–1.8 mm long, 0.9–1.4 mm wide; leaves filiform to linear, to 1.5 mm wide	*Rhynchospora plumosa*
–	Spikelets (4–)5–8 mm long, at least some stalked, solitary or loosely clustered; achene 1.7–2.6 mm long, 1.2–2.0 mm wide; leaves filiform, 0.2–0.3 mm wide	7
7	(6’.) Achene 1.7–2.0 mm long, 1.2–1.5 mm wide, apex broadly rounded at tubercle base, bristles < ½ length of achene body	*Rhynchospora galeana*
–	Achene 1.9–2.6 mm long, 1.5–2.0 mm wide, apex constricted at tubercle base, bristles ≥ ¾ length of achene body	*Rhynchospora oligantha*
8	(5’.) Bristles retrorsely barbed	9
–	Bristles antrorsely barbed or entire, or bristles absent	12
9	(8.) Achene 1.1–1.8 mm wide	10
–	Achene 0.65–0.95 mm wide	11
10	(9.) Achenes 1(2) per spikelet, 2.1–2.6 mm long; leaf blades 1.5–3 mm wide	Rhynchospora cephalantha var. cephalantha
–	Achenes (1)2(3) per spikelet, 1.5–2.0 mm long; leaf blades 2.5–5 mm wide	Rhynchospora glomerata var. glomerata
11	(9’.) Spikelets mostly pale reddish-brown, spreading to erect, in turbinate to hemispheric clusters; achene 1.6–1.8 mm; tubercle < 0.5 mm wide at base	*Rhynchospora chalarocephala*
–	Spikelets dark reddish-brown, lowest usually reflexed, in mostly globose clusters; achene 1.3–1.6 mm long; tubercle ≥ 0.5 mm wide at base	*Rhynchospora microcephala*
12	(8’.) Achene surface smooth, faintly striate, or remotely pitted	13
–	Achene surface horizontally ridged or wrinkled, or faintly to strongly reticulate	23
13	(12.) Bristles 12	*Rhynchospora baldwinii*
–	Bristles 0–6	14
14	(13’.) Leaf margin long-ciliate, apex bluntly acute	*Rhynchospora ciliaris*
–	Leak margin entire or short-pubescent on the margins, apex long-acuminate	15
15	(14’.) Spikelet scales white or whitish; bristles absent, or bristles 1–3 and reduced	16
–	Spikelet scales tan, reddish, or brown; bristles present, if reduced then 4–6	17
16	(15.) Achene 1.0–1.2 mm long, 0.8–1.0 mm wide, smooth; base of plant not bulbous, not enveloped in bladeless sheaths	*Rhynchospora chapmanii*
–	Achene 1.4–1.8 mm long, 1.2–1.5 mm wide, finely vertically striate; base of plant bulbous, enveloped in bladeless sheaths	*Rhynchospora pallida*
17	(15’.) Achene 0.6–0.9 mm wide, tubercle margin setose	*Rhynchospora filifolia*
–	Achene > 1 mm wide, tubercle margins entire or roughened but not setose	18
18	(17’.) Tubercle 1.0–2.6 mm long	*Rhynchospora gracilenta*
–	Tubercle 0.2–0.8 mm long	19
19	(18’.) Bristles ≤ ½ length of achene body	20
–	Bristles > ½ length of achene body	21
20	(19.) Leaf blades ≤ 1 mm wide; culms 20–45 cm tall	*Rhynchospora debilis*
–	Leaf blades 2–4 mm wide; culms 100–150 cm tall	*Rhynchospora fascicularis*, in part
21	(19’.) Basal leaf blades (at least some) nearly as long as the culm, ≤ 1.3 mm wide	*Rhynchospora wrightiana*
–	Basal leaf blades conspicuously shorter than culm, 1.3–4.0 mm wide	22
22	(21’.) Achene suborbicular, 1.2–1.5 mm wide, longer bristles less than half (rarely exceeding) length of achene body; larger basal leaves 1.3–2.5 mm wide	*Rhynchospora distans*
–	Achene elliptic, 1.1–1.3 mm wide, longer bristles equaling or exceeding length of achene body; larger basal leaf blades 2–4 mm wide	*Rhynchospora fascicularis*, in part
23	(12’.) Culm and leaf blades filiform, 0.2–0.5 mm wide	24
–	Culm and leaf blades broader, not filiform, > 0.5 mm wide	27
24	(23.) Achene 1.3–1.6 mm long, 0.9–1.4 mm wide, tubercle triangular, 0.3–0.6 mm long, bristles present (readily apparent at 10× magnification)	*Rhynchospora rariflora*
–	Achene 0.5–0.9 mm long, 0.5–0.7 mm wide; tubercle button-like or short conic, 0.1–0.2 mm long, bristles absent (or present in *Rhynchospora thornei*, but not or only barely apparent at 10× magnification)	25
25	(24’.) Achene elliptical, including tubercle 1.0–1.2 mm long, tubercle short conic, rounded apically, bristles present (though not readily apparent at 10× magnification)	*Rhynchospora thornei*
–	Achene obovate, including tubercle 0.6–0.9 mm long, tubercle button-like, flat or nearly so apically, bristles absent	26
26	(25’.) Achene smooth or weakly reticulate, not prominently transversely ridged	*Rhynchospora divergens*
–	Achene prominently horizontally ridged	*Rhynchospora pusilla*
27	(23’.) One or both achene faces flat to concave	28
–	Both achene faces convex	31
28	(27.) Achene narrowly elliptical, ≥ 2× as long as broad, tubercle subulate, 0.8–1.2 mm long	*Rhynchospora inexpansa*
–	Achene broadly elliptical to obovate, ≤ 2× as long as broad, tubercle triangular, 0.2–0.4 mm long	29
29	(28’.) Achene 1.3–1.8 mm long, 0.9–1.2 mm wide, faces flat, 10–12-ridged	*Rhynchospora torreyana*
–	Achene 0.8–1.4 mm long, 0.7–1.2 mm wide, faces slightly biconvex, 6–12-ridged	30
30	(29’.) Achene faces averaging 8–12 ridges, tubercle base usually convex upon achene, decurrent along achene margins; spikelet clusters elongate	*Rhynchospora decurrens*, in part
–	Achene faces averaging 6–7 ridges, tubercle base usually flat across achene, not decurrent along achene margins; spikelet clusters usually congested	*Rhynchospora microcarpa*, in part
31	(27’.) Achene 1.4–2.5 mm wide, apex (*not* tubercle base) thickened, rim-like, forming a distinct buttress immediately below tubercle, not constricted	32
–	Achene 0.7–1.5 mm wide, apex neither thickened nor rim-like (*tubercle base* may be thickened and rim-like, but then distinguished from achene by a constriction at the achene apex), not forming a buttress immediately below tubercle, constricted or not	33
32	(31.) Achene 2.0–2.7 mm long, 2.0–2.5 mm wide	*Rhynchospora grayi*+
–	Achene 1.5–1.8 mm long, 1.4–1.7 mm wide	*Rhynchospora harveyi*+
33	(31’.) Bristles absent; achenes 0.7–1.0 mm long	34
–	Bristles present (sometimes deciduous in *Rhynchospora decurrens*, with achenes 1.0–1.4 mm long); achenes 0.7–1.8 mm long	35
34	(33.) Tubercle crescent-shaped, broader than long, 0.1–0.3 mm long; achene strongly transversely rugose	*Rhynchospora nitens*
–	Tubercle conical to subulate, as long as broad or longer, ≥ 0.5 mm long; achene weakly transversely rugose	*Rhynchospora scirpoides*
35	(33’.) Bristles longer than or equaling tubercle	36
–	Bristles shorter than or equaling achene body	38
36	(35.) Tubercle 0.2–0.3 mm long, margins smooth	*Rhynchospora microcarpa*, in part
–	Tubercle 0.4–0.8 mm long, margins edges setose or bearing irregular, waxy protuberances	37
37	(36’.) Achene broadly obovoid to suborbicular, 1.2–1.6 mm wide, with prominent horizontal ridges; leaf blades 4–7 mm wide	*Rhynchospora caduca*
–	Achene narrowly obovoid, 0.8–1.1(–1.2) mm wide, lacking horizontal ridges or with horizontal ridges poorly developed; leaf blades 3–5 mm wide	*Rhynchospora mixta*
38	(35’.) Achene lenticular in cross-section, faces slightly convex, bristles ≥ ½ as long as achene body (sometimes exceeding achene body in *Rhynchospora microcarpa*); inflorescence branches capillary	39
–	Achene narrowly to broadly elliptical in cross-section, faces broadly convex or round, bristles ≤ ¾ length of achene body	40
39	(38.) Achene faces averaging 8–12 ridges, tubercle base usually convex upon achene, decurrent along achene margins; spikelet clusters elongate	*Rhynchospora decurrens*, in part
–	Achene faces averaging 6–7 ridges, tubercle base usually flat across achene, not decurrent along achene margins; spikelet clusters usually congested	*Rhynchospora microcarpa*, in part
40	(38’.) Alveoli (surface cells of achene body) narrow, longer than wide, horizontal walls raised, forming horizontal ridges across achene body, tubercle 0.2–0.4 mm long, 0.5–0.7 mm wide at base	*Rhynchospora globularis*
–	Alveoli nearly as wide as long, horizontal walls not or only slightly raised, not forming ridges horizontal across achene body, tubercle 0.35–0.7 mm long, 0.7–0.9 mm wide at base	*Rhynchospora pinetorum*

Key adapted from Kral (2002b), Weakley (2012).

Note: *Rhynchospora grayi* Kunth, of dry, sandy sites, and *Rhynchospora harveyi* W. Boott, of wet savannas, were reported from Sandy Run by Taggart (2010). However, the vouchers for these taxa (*Taggart SARU 600*, WNC and *Taggart SARU 636*, WNC, respectively) appear to the senior author to be *Rhynchospora pinetorum* Britton & Small and *Rhynchospora mixta* Britton, respectively. Though not otherwise reported or collected in Shaken Creek Preserve or the vicinity, these taxa may occur in the area and are therefore maintained in the key below, where indicated by a plus (+) symbol.

Figs 42, 43, 44

### [Cyperaceae] Scirpus L.

**Table:** 

1	Fertile culms erect; sheaths of proximal leaves red-brown to green; inflorescence terminal; perianth bristles conspicuous, exceeding spikelet scales at maturity	*Scirpus cyperinus*
–	Fertile culms reclining; sheaths of proximal leaves whitish; inflorescence terminal and axillary in 2–3 distal leaves; perianth bristles inconspicuous, not or only slightly exceeding spikelet scales at maturity	*Scirpus lineatus*

Key adapted from Whittemore and Schuyler (2002).

Fig. 45

### [Cyperaceae] Scleria P.J. Bergius

**Table:** 

1	Achene base tapering, essentially continuous with achene body, indented or with small pits, hypogynium (a distinctive collar or ridge at achene base that differs in texture or color from achene body) absent	2
–	Achene base rounded or truncate, abruptly differentiated from achene body by gap or zone of different color or texture, hypogynium present	4
2	(1.) Spikelet clusters 2–9, well-spaced along rachis; spikelets 2–3(–4) mm long	*Scleria verticillata*
–	Spikelet clusters 1, terminal; spikelets 4–10 mm long	3
3	(2’.) Achene 3–4 mm long, base lacking granular pits in concave sides	*Scleria baldwinii*
–	Achene 2–3 mm long, base with granular pits in concave sides	*Scleria georgiana*
4	(1’.) Hypogynium minutely papillose and forming a continuous band, not divided into distinct tubercles or lobes	5
–	Hypogynium with 3 or 6 distinct tubercles or with 3 lanceolate lobes	6
5	(4.) Leaf blades 1–3 mm wide; achene 1.5–2 mm long	*Scleria minor*
–	Leaf blades (3–)5–9 mm wide; achene 2.0–3.3 mm long	*Scleria triglomerata*
6	(4’.) Hypogynium with 3 lanceolate lobes appressed to achene base	*Scleria muehlenbergii*
–	Hypogynium with 3 or 6 distinct, papillose tubercles	7
7	(6’.) Achenes 1.5–2.0 mm long, tubercles 6, paired but distinctly separate	Scleria pauciflora var. caroliniana
–	Achenes 2.0–3.6 mm long, tubercles 3, often 2-lobed but fused, or 6 and achene > 2.5 mm long (in *Scleria* species 1)	8
8	(7’.) Achene 2.6–3.3(–3.6) mm long, 2.0–2.6 mm wide; larger leaf blades 3–7 mm wide; tubercles 3, 2-lobed, or 6, separate	*Scleria* species 1
–	Achene 2.0–2.5(–3.0) mm long, 1.5–2.0(–2.3) mm wide; larger leaf blades 1–3.5 mm wide, sheaths pubescent between and on nerves; tubercles 3, 2-lobed	9
9	(8’.) Culms and/or sheaths pubescent; leaf blades ciliate, ca. 2 mm wide; bracts ciliate; plants usually of loamy sands (e.g., ultisols)	Scleria ciliata var. ciliata
–	Culms, sheaths, blades, and bracts glabrous; leaf blades 1–3.5 mm wide; plants usually of sandy soils (e.g., spodosols)	Scleria ciliata var. glabra

Key adapted from Reznicek et al. (2002), Weakley (2012).

Note: Measurements of achene length in the key below are taken from the base of the achene and include the hypogynium when present.

Fig. 46a, b, c, d, e

### Eriocaulaceae

**Table:** 

1	Scapes glabrous, 20–110 cm tall; base of leaf blades with evident lacunae (air spaces); roots septate	* Eriocaulon *
–	Scapes pilose at least proximally, 15–40 cm tall; base of leaf blades lacking evident lacunae; roots not septate	2
2	(1’.) Scapes pubescent with eglandular hairs; roots dark, branched; heads pale gray to white; leaf blades gradually tapering through most of their lengths, bases not abruptly flared	*Lachnocaulon anceps*
–	Scapes pubescent with at least some glandular hairs, especially distally; roots pale, unbranched; heads yellowish-tan to gray; leaf blades narrowly linear, abruptly flared at base	*Syngonanthus flavidulus* Fig. 47

Key adapted from Kral (2000a), Weakley (2012).

### [Eriocaulaceae] Eriocaulon L.

**Table:** 

1	Heads soft, compressed and nearly flattened when squeezed; leaves pale green, seldom exceeding scape sheath, apex attenuate to subulate	*Eriocaulon compressum*
–	Heads hard, little compressed when squeezed; leaves dark green, mostly exceeding scape sheath, apex acute to obtuse	Eriocaulon decangulare var. decangulare

Key adapted from Kral (2000a), Weakley (2012).

Fig. 48a, b, c

### [Eriocaulaceae] Lachnocaulon L.

**Table:** 

1	Mature inflorescence 4–7(–9) mm wide; scapes pilose throughout; seeds dull, longitudinal striations prominent at 10× magnification; leaf blades linear, 2.5–6(–12) cm wide at widest point, tapering gradually to the tip	*Lachnocaulon anceps*
–	Mature inflorescence 3.5–4(–5) mm wide; scapes glabrous or glabrate distally; seeds lustrous, longitudinal striations obscure at 10× magnification; leaf blades narrowly linear, 1.5–4 cm wide at widest point, tapering abruptly to the tip	*Lachnocaulon beyrichianum*+

Key adapted from Kral (2000a), Weakley (2012).

Note: *Lachnocaulon beyrichianum* Sporl. ex Körn. was reported from Sandy Run by Taggart (2010); however, the voucher for this report (*Taggart SARU 217*, WNC) appears, based on the pubescent scapes and gradually tapering leaf tips, to be *Lachnocaulon anceps* (Walter) Morong. Though not otherwise reported for or collected in Shaken Creek Preserve or the vicinity, *Lachnocaulon beyrichianum* may occur in the area and is therefore maintained in the key below, where indicated by a plus (+) symbol.

Fig. 49

### [Hypoxidaceae] Hypoxis L.

**Table:** 

1	Leaves glabrous or glabrate with a few scattered hairs near base	2
–	Leaves sparsely to densely pubescent, at least near base	3
2	(1.) Pedicel usually shorter than bracts; tepals usually ≤ 2× as long as ovary; ovary cylindric, glabrate or sparsely pubescent	*Hypoxis curtissii*
–	Pedicel usually > 2× as long as bracts; tepals much longer than ovary; ovary obconic, densely pubescent	*Hypoxis hirsuta*
3	(1’.) Tepals 1.5–2× as long as ovaries; seeds black, covered by a loose, iridescent membrane, surface pebbled (with rounded projections), (1.3–)1.4–2 mm in diam.; leaf blades 0.9–5 mm wide	*Hypoxis sessilis*
–	Tepals < 1.5× as long as ovaries; seeds brown, not covered by a membrane, surface minutely muricate (with pointed projections), 0.9–1.1(–1.3) mm in diam.; leaf blades (0.5–)0.7–2.1(–2.6) mm wide	*Hypoxis wrightii*

Key adapted from Herndon (2002), Weakley (2012).

Fig. 50

### Iridaceae

**Table:** 

1	Sepals and petals dissimilar, sepals larger, conspicuously marked with a patch of contrasting color (“signal”), petals smaller, of uniform color; style branches broad, petaloid, arching over basal portion of sepals; stems not winged	* Iris *
–	Sepals and petals similar; styles filiform, not broad and petaloid, not arching over basal portion of sepals; stems frequently winged (inconspicuously so or uwinged in *Sisyrinchium capillare*), appearing somewhat similar to leaves	* Sisyrinchium *

Key adapted from and Radford et al. (1968) and Goldblatt (2002).

### [Iridaceae] Iris L.

**Table:** 

1	Stems 5–15 cm tall; basal leaves 3–15 cm long, 0.3–1.3 cm wide, glaucous; rhizomes branches cord-like, narrower than parent rhizome	Iris verna var. verna
–	Stems 30–100 cm tall; basal leaves 30–80 cm long, 1.5–3 cm wide, glaucous or not; rhizomes branches thick, similar in size to parent rhizome	2
2	(1’.) Basal leaves 30–50 cm long, 1.5–2.3 cm wide, glaucous; petals inconspicuous, hidden among sepal bases, 1–1.5(–2) cm long, 0.3–0.5 cm wide	*Iris tridentata*
–	Basal leaves 60–80 cm long, 2.5–3 cm wide, not glaucous; petals conspicuous, 3–7 cm long, 1–3 cm wide	Iris virginica var. virginica

Key adapted from Henderson (2002), Weakley (2012).

Fig. 51a, b, c, d, e

### [Iridaceae] Sisyrinchium L.

**Table:** 

1	Tepals distinctly campanulate basally, flaring apically, maroon, pink, lavender, or yellow, with a maroon patch near the base; plants annual, usually < 20 cm tall (rarely to 36 cm tall); capsules tan with purplish sutures (and sometimes apex)	*Sisyrinchium rosulatum*‡
–	Tepals rotate to subrotate, flaring basally, blue to violet, lacking a maroon patch near the base; plants perennial, usually > 20 cm tall (to 45 cm tall); capsules tan or brown, lacking purplish streaking on sutures	2
2	(1’.) Inflorescences paired at stem apex (rarely actually solitary but often appearing so by the concealing of the inner inflorescence by a large, leaf-like bract), sessile (rarely outer borne on branch-like peduncle to 7 mm long), closely subtended and often enveloped by large leaf-like bract (often appearing nearly as a continuation of the stem) that frequently conceals the inner inflorescence; outer spathe bract connate 0–1.5 mm at base; stems 0.5-3.4 mm wide	3
–	Inflorescences solitary, terminating stem or branch-like peduncles, not closely subtended and enveloped by a leaf-like bract; outer spathe bract connate 2–6 mm at base; stems 0.8–5 mm wide	4
3	Stems 1.3–3.4 mm wide, obviously winged; leaf bases not persistent in fibrous tufts	*Sassafras albidum*
–	Stems 0.5–1.0 mm wide, not or only obscurely winged; leaf bases persistent in fibrous tufts	*Sisyrinchium capillare*
4	(2’.) Leaf bases persistent in fibrous tufts	*Sisyrinchium arenicola*
–	Leaf bases not persistent in fibrous tufts	5
5	(4’.) Stems 2.3–5 mm wide; capsules 4–7 mm in diam.	*Sisyrinchium angustifolium*
–	Stems 0.8–1.9 mm wide; capsules 2–4.1 mm in diam.	*Sisyrinchium atlanticum*

Key adapted from Cholewa and Henderson (2002), Weakley (2012).

NOTE: The inflorescence of *Sisyrinchium* is comprised of 1–11(–15), usually pedicellate flowers (and their tiny, hyaline bracteoles) that emerge from within two green or purplish-tinged “spathes.” Inflorescences and associated spathes occur singly at the tips of branch-like peduncles in most species but are characteristically paired at the stem apex in *Sassafras albidum* and *Sisyrinchium capillare*. In these latter two species, one of the two inflorescences is often concealed by a large, erect, leaf-bract, which may give the false impression that only one inflorescence is present.

Several unusual *Sisyrinchium* specimens were collected in Sandy Run [Neck] by Wilbur, who determined the specimens to be *Sisyrinchium arenicola*, a taxon listed by Weakley (2012) as occurring from MD northward. The specimens have the following features, all of which agree with *Sisyrinchium arenicola*: stems 2.7–3.4 mm wide, leaf bases persistent in fibrous tufts, outer spathe bracts 12–21 mm long, and capsules 3.2–4.1 mm long. Pending further review, these specimens are here treated as *Sisyrinchium arenicola*, despite the geographic anomaly. *Sisyrinchium rosulatum* E.P. Bicknell has not been found in pertinent habitats in Shaken Creek Preserve or the vicinity; however, the species has been collected along roadsides very near savannas and flatwoods and is, for convenience, included in the key below, where indicated by a double dagger symbol (‡).

Fig. 52a, b, c, d, e

### [Juncaceae] Juncus L.

**Table:** 

1	Inflorescence appearing lateral, inflorescence bract terete, erect, appearing as a continuation of the culm	2
–	Inflorescences appearing terminal, inflorescence bract flat, involute, or terete, erect or ascending, not appearing as a continuation of the culm	3
2	(1.) At least some sheaths at base of plant with well-developed blades; inflorescence bract channeled (with a narrow groove marking point at which blade edges have rolled together); capsules 1-locular, widely ovoid to nearly globose, 3.5–5 mm long	*Juncus coriaceus*
–	Sheaths at base of plant lacking blades; inflorescence bract not channeled; capsules 3-locular, broadly ellipsoid to oblate, 1.5–3.2 mm long	Juncus effusus ssp. solutus
3	(1’.) Leaf blades not septate	4
–	Leaf blades septate (often difficult to detect in *Juncus pelocarpus*, keyed here and above)	8
4	(3.) Flowers borne in glomerules of 2 or more, not subtended by 2 bracteoles (though with bracteole at base of pedicel)	5
–	Flowers borne singly, subtended by 2 bracteoles (in addition to bracteole at base of pedicel)	7
5	(4.) Plant creeping or ascending, aquatic, submersed portions sterile, emersed portions fertile; perianth 6–10 mm long	*Juncus repens*
–	Plant erect, terrestrial in wet to moist habitats; perianth < 6 mm long	6
6	(5’.) Widest leaf blade (2.6–)3.1–4.5(–5.4) mm wide, sheath of lowest leaf (3.2–)4.3–7.8(–9.7) cm long; tallest culm (27.2–)50.8–81.2(–100.7) cm tall; anthers exserted beyond tepals, (0.5–)0.6–1.0(–1.3) mm long; stem base (3.4–)5.8–9.6(–12.0) mm wide	*Juncus biflorus*
–	Widest leaf blade (1.3–)1.6–2.6(–3.5) mm wide, sheath of lowest leaf (1.7–)2.2–3.8(–4.7) cm long; tallest culm (19.2–)26.0–44.0(–56.8) cm tall; anthers hidden by tepals, (0.2–)0.3–0.5(–0.7) mm long; stem base (0.4–)2.0–4.4(–6.0) mm wide	*Juncus marginatus*
7	(4’.) Plant annual, 0.5–4 dm tall; rhizomes absent; leaves flat; cauline leaves present	*Juncus bufonius*
–	Plant perennial, to 0.7–10 (usually >4) dm tall; rhizomes present; leaves terete, slightly channeled, or flat; cauline leaves present or absent	8
8	(7’.) Rhizomes short; leaves terete, slightly channeled or flat; cauline leaves absent; flowers not replaced by bulbils	*Juncus dichotomus*
–	Rhizomes elongate; cauline leaves present; leaves terete; cauline leaves present; flowers often replaced by bulbils	*Juncus pelocarpus*, in part
9	(3’.) Seeds 1.1–2.6 mm long, conspicuously tailed at both ends	10
–	Seeds < 0.7 mm long, not tailed at both ends	11
10	(9.) Mature capsules brown to reddish brown, 3.0–4.0(–4.5) mm long, < 1.5 mm longer than perianth; seeds (including tails) 1.1–1.9 mm long; heads 5–50-flowered	*Juncus canadensis*
–	Mature capsules dark reddish-purple, 4.0–5.0 mm long, 2 mm longer than perianth; seeds (including tails) 1.8–2.6 mm long; heads 3–7-flowered	*Juncus trigonocarpus*
11	(9’.) Flowers solitary, often aborted and replaced by bulbils; inflorescence diffuse	*Juncus pelocarpus*, in part
–	Flowers borne in glomerules of 3 or more, rarely aborted; inflorescence diffuse to congested	12
12	(11’.) Heads spherical or nearly so, 20–60-flowered, 6–15 mm in diam.	13
–	Heads turbinate to hemispherical, (1)2–20-flowered (rarely to 50-flowered in *Juncus diffusissimus*), 3–10 mm in diam.	18
13	(12.) Leaves flat or nearly so, linear to narrowly elliptical in cross section	14
–	Leaves terete, round in cross section	15
14	(13.) Leaf septa incomplete (individual septa not spanning the width of the blade); apex of capsule united at maturity	*Juncus polycephalos*
–	Leaf septa complete (individual septa spanning the width of the blade); apex of capsule splitting at maturity	Juncus validus var. validus
15	(13’.) Rhizomes absent; tepals lanceolate	*Juncus acuminatus*, in part
–	Rhizomes present (sometimes inconspicuous, short, hard, knotty); tepals lanceolate-subulate	16
16	(15’.) Uppermost cauline leaf blade (below inflorescence bract) lacking septa, conspicuously shorter than its sheath; inner tepals shorter than outer tepals	*Juncus megacephalus*
–	Uppermost cauline leaf blade (below inflorescence bract) septate, as long as or longer than its sheath; tepals of similar length	17
17	(16’.) Heads lobed; mature capsules 2.0–3.0 mm long	Juncus scirpoides var. compositus
–	Heads globose, not lobed; mature capsule 3.0–4.5 mm long	Juncus scirpoides var. scirpoides
18	(12’.) Mature capsules ≥ 2 mm longer than perianth, 4.0–5.2 mm long	*Juncus diffusissimus*
–	Mature capsules < 1.5 mm longer than perianth, 2.4–3.5(–4.0) mm long	19
19	(18’.) Heads 5–50; capsules stramineous, 2.8–3.5(–4) mm long; inner and outer tepals of similar length, 2.6–3.5(–3.9) mm long	*Juncus acuminatus*, in part
–	Heads 40–100(–200); capsules chestnut-brown, 2.4–2.9 mm long; inner tepals (1.8–)2.4–2.8 mm long, shorter than outer tepals, outer tepals (2.2–)2.6–2.9 mm long	*Juncus elliottii*

Key adapted from Brooks and Clemants (2000), Knapp and Naczi (2008), and Weakley (2012).

Figs 53, 54, 55a, b, c

### Melanthiaceae

**Table:** 

1	Inflorescence racemose	2
–	Inflorescence paniculate	3
2	(1.) Basal leaves usually ≥ 4, (5–)7–10(–28) mm wide, not enclosed basally by a sheath; capsule ca. as long as broad, 5–7 mm long, 5–7 mm wide	*Amianthium muscitoxicum* Fig. 56
–	Basal leaves 1–3, 2–7 mm wide, enclosed at base by a purple sheath 3–8 cm long; capsule ≥ 2× as long as broad, 7–9 mm long, 3–4 mm wide	*Stenanthium densum* Fig. 57
3	(1’.) Inflorescence axes scurfy-pubescent, rough to the touch	*Veratrum virginicum* Fig. 58
–	Inflorescence axes glabrous, smooth to the touch	*Zigadenus glaberrimus* Fig. 59

Key adapted from Weakley (2012).

### [Nartheciaceae] Aletris L.

**Table:** 

1	Perianth white to creamy white	*Aletris farinosa*
–	Perianth yellow to golden yellow	2
2	(1’.) Perianth campanulate or short-cylindric, 6–7 mm long, ≤ 2× as long as broad, lobes erect	*Aletris aurea*
–	Perianth long-cylindric, 8–12 mm long, > 2.5× as long as broad, lobes slightly to strongly spreading	*Aletris lutea*

Key adapted from Sullivan (2002), Weakley (2012).

Fig. 60a, b, c, d, e

### Orchidaceae

**Table:** 

1	Lip spurred; flowers white, yellow, or orange, numerous	* Platanthera *
–	Lip not spurred; flowers white, pink, magenta, or purple, solitary to few, or numerous	2
2	(1’.) Flowers arranged in distinct spirals (often appearing 3–4 ranked if spiral is “tight”), white, relatively small, 3–5 mm wide	* Spiranthes *
–	Flowers not in distinct spirals, pink, magenta, or purple (rarely white, then most commonly in *Calopogon*), larger, typically ≥ 1 cm wide	3
3	(2’.) Flowers non-resupinate, lip oriented upwards, bearing numerous orange or yellow clavellate trichomes reminiscent of stamens	* Calopogon *
–	Flowers resupinate, lip oriented downwards, not bearing numerous stamen-like trichomes	4
4	(3’.) Sepals brown to purple, (2.4–)3.0–6.5 cm long; leaf coriaceous	* Cleistesiopsis *
–	Sepals pink (rarely white), 1.4–2.3 cm long; leaf herbaceous	*Pogonia ophioglossoides* Fig. 61

Key adapted from Romero-González et al. (2002), Weakley (2012).

### [Orchidaceae] *Calopogon* R. Br.

**Table:** 

1	Flowers spaced < 1 cm apart, not fragrant, opening nearly simultaneously; leaf appressed to inflorescence at flowering	*Calopogon barbatus*
–	Flowers spaced > 1 cm apart, faintly fragrant, opening sequentially; leaf not appressed to inflorescence at flowering	2
2	(1’.) Lateral sepals strongly falcate, 10–15 mm long, 5–9 mm wide; petals lanceolate to weakly pandurate (fiddle-shaped), falcate, 9–18 mm long, 3–5.5 mm wide, lip 9–13 mm long	*Calopogon pallidus*
–	Lateral sepals straight to slightly falcate, 13–26 mm long, 5–16 mm wide; petals obpandurate (inversely fiddle-shaped), straight, 15–28 mm long, 4–14 mm wide, lip 11–23 mm long	Calopogon tuberosus var. tuberosus

Key adapted from Goldman et al. (2002).

Fig. 62a, b, c

### [Orchidaceae] *Cleistesiopsis* Pansarin & F. Barros

**Table:** 

1	Column 21–25(–29) mm long, lip (26–)34–55 mm long, central keel with 1–3 continuous basal ridges; fresh flower with daffodil-like odor	*Cleistes divaricata*
–	Column 13–19 mm long, lip 21–33(–38.5) mm long, central keel with 5–7 discontinuous basal ridges; fresh flower with vanilla odor	*Cleistesiopsis oricamporum*

Key adapted from Gregg and Catling (2002), Weakley (2012).

Fig. 63

### [Orchidaceae] Platanthera Rich.

**Table:** 

1	Lip entire or minutely crenulate	2
–	Lip prominently fringed	3
2	(1.) Flowers golden-yellow, spur 4–8 mm long, lip minutely crenulate	*Platanthera integra*
–	Flowers white, spur 11–23 mm long, lip entire	*Platanthera nivea*
3	(1’.) Flowers white	*Platanthera blephariglottis*
–	Flowers orange	4
4	(3’.) Spur 20–35 mm long, longer than ovary, undivided segment of lip 8–12 mm long; ovary 12–27 mm long	*Platanthera ciliaris*
–	Spur 4–10 mm long, shorter than ovary, undivided segment of lip 4–6 mm long; ovary 7–13 mm long	*Platanthera cristata*

Key adapted from Sheviak (2002), Weakley (2012).

Fig. 64

### [Orchidaceae] Spiranthes Rich.

**Table:** 

1	Adaxial surface of lip glabrous, prominently veined, veins greenish (rarely cream-colored or yellowish), divergent, terminally widened, extending almost to lip apex; plants flowering Mar–Jul	*Spiranthes praecox*
–	Adaxial surface of lip glabrous or pubescent, veins absent or present, if present then neither prominent nor terminally widened, green or white, divergent or straight, extending to lip apex or not; plants flowering collectively Feb–Dec	2
2	(1’.) Lateral sepals widely diverging from base, 8–10 mm long; lip distinctly dilated basally, yellow centrally; inflorescence secund to twisted usually no more than 180° from bottom to top; plants flowering late Oct–Dec	*Spiranthes longilabris*
–	Lateral sepals spreading to appressed, not widely diverging from base, 3.8–10 mm long; lip not distinctly dilated basally, white or creamy centrally; inflorescence usually with several spiral cycles (rarely nearly secund); plants flowering Feb–Nov	3
3	(2’.) Lip apex laciniate-dentate; leaves usually linear with length/width ratio > 30 (rarely broader on stout, leafy plants > 1 m tall), present at flowering; plants 20–95(–100+) cm tall	*Spiranthes laciniata*
–	Lip apex undulate to crisped, occasionally ragged, not laciniate-dentate; leaves lanceolate to ovate to obovate, with length/width ratio < 30, present or absent at flowering; plants 10–65 cm tall	4
4	(3’.) Flowers relatively large, perianth 5–12 mm long, lip often darker centrally but not green or greenish yellow	*Spiranthes cernua*
–	Flowers relatively small, perianth 3–5 mm long, lip green centrally	5
5	(4’.) Lateral sepals spatulate, green basally, white apically; leaves oblanceolate, usually present (but withering) at anthesis; flowering Feb–May	*Spiranthes eatonii*
–	Lateral sepals acuminate, white throughout; leaves obovate, absent at anthesis; flowering Jul–Oct(–Nov)	Spiranthes lacera var. gracilis

Key adapted from Sheviak and Brown (2002), Weakley (2012).

Figs 65, 66

### Poaceae

**Table:** 

1	Culm perennial, woody, often developing complex branching systems from the upper nodes; [tribe *Bambuseae*]	* Arundinaria *
–	Culm annual or facultatively perennial, herbaceous, not developing complex branching systems from the upper nodes	2
2	(1’.) Spikelets almost always with 2 florets, lower floret in spikelet always sterile or staminate, frequently reduced to lemma or absent, upper floret bisexual, staminate, or sterile, unawned or awned from the lemma apices or, if lemmas bilobed, from the sinuses; glumes membranous and upper lemma stiffer than lower lemma, or both florets reduced and concealed by firm to leathery glumes; rachilla not prolonged beyond the second floret; [tribes *Andropogoneae* and *Paniceae*]	3
–	Spikelets **either** not with 2 florets **or** with 2 and lower floret bisexual or upper floret awned from the back or base of the lemma; glumes usually membranous; lemmas scarious to indurate; rachilla sometimes prolonged beyond the distal floret; [various tribes]	17
3	(2.) Spikelets in sessile-pedicellate pairs, not arranged in obvious rows on 1 side of rachis; glumes stiff, coriaceous to indurate, usually subequal in length, one or usually both exceeding the upper floret (excluding awn); lemmas hyaline; paleas hyaline or absent; [tribe *Andropogoneae*]	4
–	Spikelets solitary or if paired, then forming 2–4 obvious rows on 1 side of rachis; glumes flexible, membranous, lower glumes usually shorter than upper glumes or absent, upper glumes usually shorter than or nearly equaling upper floret; lower lemmas membranous; upper lemmas typically coriaceous to indurate, occasionally membranous; upper paleas similar in texture to upper lemmas; [tribe *Paniceae*]	8
4	(3.) Spikelets embedded in a thickened rachis	*Coelorachis rugosa* Fig. 67
–	Spikelets not embedded in a thickened rachis	5
5	(4’.) Pedicellate spikelet perfect	* Saccharum *
–	Pedicellate spikelet staminate, vestigial, or absent	6
6	(5’.) Leaf sheath auriculate at apex; inflorescences terminal, with elongate rachises and branches with numerous racemes; peduncles and branches not subtended by a modified leaf	*Sorghastrum nutans* Fig. 68
–	Leaf sheath not auriculate at apex; inflorescences terminal and axillary, composed of clusters of 1–7(–13) racemes on a common peduncle; peduncles subtended by and often partially included in a modified leaf	7
7	(6’.) Racemes 2–7(–13) per peduncle; summit of raceme internodes generally flat, not strongly cup-shaped; lower glumes of sessile spikelets flat or concave	* Andropogon *
–	Racemes 1 per peduncle; summit of raceme internodes strongly cup-shaped; lower glumes of sessile spikelets convex	Schizachyrium scoparium var. scoparium Fig. 69
8	(3’.) Subterranean spikelets fertile; aerial spikelets sterile; leaf sheaths and blade surfaces hirsute, margins ciliate	*Amphicarpum amphicarpon* Fig. 70
–	Subterranean spikelets absent, aerial spikelets fertile; leaf sheaths, blades, and margins glabrous or variously pubescent	9
9	(8’.) Spikelets subtended by an involucre of 4–12 bristles	*Setaria parviflora* Fig. 71
–	Spikelets not subtended by an involucre of bristles	10
10	(9’.) Upper glumes and lower lemmas conspicuously villous, hairs 0.6–1.5 mm long, purplish at maturity; upper lemmas and upper paleas cartilaginous, flexible at maturity, dark brown; upper lemma margins flat, hyaline	*Anthenantia rufa* Fig. 72
–	Upper glumes and lower lemmas glabrous or pubescent, if pubescent then not conspicuously villous with purplish hairs 0.6–1.5 mm long; upper lemmas and upper paleas chartaceous or indurate, rigid at maturity, white, stramineous, or golden brown; upper lemma margins typically involute, not hyaline	11
11	(10’.) Spikelets in racemes, arranged in 2–4 conspicuous rows on one side of rachis	* Paspalum *
–	Spikelets in panicles, not arranged in conspicuous rows on one side of rachis	12
12	(11’.) Basal leaves distinctly shorter and broader than cauline leaves, ovate to lanceolate, forming an over-wintering rosette	*Dichanthelium*, in part
–	Basal leaves similar to cauline leaves, usually linear to lanceolate but varying from filiform to ovate not forming an over-wintering rosette	13
13	(12’.) Plants producing terminal panicles in spring; culms branching usually from middle and lower cauline nodes in summer, these branches further branching once or more by fall; upper florets not disarticulating at maturity	*Dichanthelium*, in part
–	Plants producing terminal terminal panicles in late summer or fall; culms usually not branching from middle and lower cauline nodes or, if so, branches rarely further branched; upper florets disarticulating or not at maturity	14
14	(13’.) Plants annual, lacking rhizomes or hard, knotty crowns; spikelets tuberculate or not	*Panicum*, in part
–	Plants perennial, with either rhizomes or hard, knotty crowns; spikelets smooth, not tuberculate	15
15	(14’.) Plants with hard, knotty crowns, lacking rhizomes; upper lemmas 1.2–1.6 mm long	*Coleataenia*, in part
–	Plants with rhizomes; upper lemmas 1.6–4 mm long	16
16	(15’.) Culms slightly compressed below; ligules ≤ 0.5 mm long; spikelets subsecund, usually some obliquely bent above first glume, pedicels appressed; apex of upper lemma lacking papillae, with minute tuft hairs	*Coleataenia*, in part
–	Culms terete; ligules 2–6 mm long; spikelets not secund, essentially straight (not obliquely bent above first glume), pedicels (at least some) spreading;apex of upper lemma with simple or compound papillae, glabrous	*Panicum virgatum*
17	(2’.) Lemma awn 3-branched; spikelets with 1 floret; [tribe *Aristideae*]	* Aristida *
–	Lemma awn unbranched or absent; spikelets with ≥ 1 floret	18
18	(17’.) Spike solitary; awn of upper glume horizontal at maturity; spikelets in 2 rows on 1 side of rachis; [tribe *Cynodonteae*, in part]	*Ctenium aromaticum* Fig. 73
–	Spikes numerous; awn of upper glume erect at maturity or absent; spikelets in 2 rows on 1 side of rachis (in *Gymnopogon*) or not (in remaining genera)	19
19	(18’.) Spikelets with (2–)3–5(–7) florets, basalmost floret sterile; [tribe *Centotheceae*]	*Chasmanthium laxum* Fig. 74
–	Spikelets with either 1(–2) or (4–)6–30 florets, basalmost floret not sterile	20
20	(19’.) Spikelets with (4–)6–30 florets	21
–	Spikelets with 1(–2) florets	22
21	(20.) Lemmas awned, awns geniculate, twisted; glumes longer than florets; plants flowering Apr–Jun; [tribe *Danthonieae*]	*Danthonia sericea* Fig. 75
–	Lemmas unawned; glumes shorter than florets; plants flowering Jul–Oct; [tribe *Cynodonteae*, in part]	* Eragrostis *
22	(20’.) Glumes exceeding lemmas; [tribe *Poeae*]	23
–	Glumes shorter than or nearly equal to lemmas; [tribe *Cynodonteae*]	24
23	(22.) Callus beard absent	* Agrostis *
–	Callus beard > ½ length of lemmas	*Calamagrostis coarctata* Fig. 76
24	(22’.) Callus beard present	25
–	Callus beard absent	26
25	(24.) Spikelets not strongly appressed to rachis; lemma unawned	*Calamovilfa brevipilis* Fig. 77
–	Spikelets strongly appressed to rachis; lemma awned	*Gymnopogon brevifolius* Fig. 78
26	(24’.) Ligules membranous, 1.8–5(–10) mm long; lemmas awned or not	*Muhlenbergia*, in part
–	Ligules ciliate, 0.2–1 mm long; lemmas never awned	27
27	(26’.) Plants forming clonal patches of small, evenly-spaced tufts; rhizomes elongate, scaly; summit of leaf sheaths hardened into a cartilaginous rim	*Muhlenbergia torreyana*
–	Plants forming broad, dense tussocks; rhizomes absent; summit of leaf sheaths not hardened into a cartilaginous rim	*Sporobolus pinetorum*

Key adapted from Radford et al. (1968), Barkworth (2003a), Barkworth (2003b), Barkworth (2007), Weakley (2012).

### Vegetative Key To Common Savanna Bunchgrasses

**Table:** 

1	Leaf blades distinctly bi-colored, bluish on adaxial surface, bright green on abaxial surface, flat, (1–)3–5 mm wide; ligule 1–3 mm long	*Ctenium aromaticum*
–	Leaf blades concolored, either yellowish, bluish green, or dark green on both surfaces, involute to flat, 0.75–3 mm long (to 5 mm long in *Calamovilfa brevipilis*); ligule 0.2–0.5 mm long (1–3 mm long in *Muhlenbergia expansa*)	2
2	(1’.) Ligule 1–3 mm long, membranous; old leaf bases fibrous and curly, not at all hardened	*Muhlenbergia expansa*
–	Ligule 0.2–0.5 mm long, ciliate; old leaf bases not fibrous and curly, more or less hardened	3
3	(2’.) Leaf blades flat, 2–5 mm wide, apex long-acuminate, base tapered, outer junction of leaf sheath and blade with a yellow annulum (band), leaf bases strongly hardened, shiny; plants forming large, clonal patches; culms coarse, to 1.5 m tall	*Calamovilfa brevipilis*
–	Leaf blades involute or flat, 0.75–2 mm wide, apex abruptly acute or gradually tapering, base not tapered, outer junction of leaf sheath and blade lacking a yellow annulum, leaf bases slightly to moderately hardened, shiny or dull; plants forming non-clonal clumps; culms relatively delicate, typically 0.6–1.2 m tall	4
4	(3’.) Leaf blades almost always involute, sparsely pilose basally (and often throughout length), margins entire, leaf bases slightly hardened, dull	*Aristida stricta*
–	Leaf blades flat, becoming involute upon drying, glabrous, margins scaberulous (especially basally), best observed by running finger from apex to base of leaf, leaf bases moderately hardened, somewhat shiny	*Sporobolus pinetorum*

Key adapted from notes provided by Richard LeBlond and from Weakley (2012).

### [Poaceae] Agrostis L.

**Table:** 

1	Spikelets ovate to narrowly ovate, 1.2–2 mm long, greenish or purplish; glumes 1–2 mm long; lemmas 0.8–1.2 mm long, never awned; flowering Mar–Jul	*Agrostis hyemalis*
–	Spikelets lanceolate to narrowly ovate, (1.8–)2.2–3.5(–3.7) mm long, green to tawny; glumes 1.8–3.2 mm long; lemmas 1.3–2.2 mm long, awned or awnless; flowering Jun–Nov	2
2	(1’.) Lemma 1.8–3 mm long, minutely but abundantly scabrous (as seen at ≥ 20× magnification); anthers 0.7–1.2 mm long; spikelets (2.3–)2.7–3.5(–3.7) mm long, usually clustered near the tips of the branchlets; panicle branches scabrous; culms to 15 dm tall	*Agrostis altissima*
–	Lemma 1.4–2 mm long, glabrous; anthers 0.3–0.6 mm long; spikelets (1.8–)2.2–2.7(–3.2) mm long, usually not clustered near the tips of the branchlets; panicle branches glabrous to scabrous; culms to 10 dm tall	*Agrostis perennans*

Key adapted from Harvey (2007), Weakley (2012).

Fig. 79

### [Poaceae] Andropogon L.

**Table:** 

1	Pedicellate spikelets staminate, 3.5–12 mm long; sessile spikelets 5–11 mm long	*Andropogon gerardii*
–	Pedicellate spikelets sterile, vestigial, or absent; sessile spikelets 2.6–8.4 mm long	2
2	(1’.) Leaves strongly glaucous (often appearing powdery-white and leaving a white residue on fingers when rubbed), glabrous	3
–	Leaves not or only slightly glaucous (never appearing powdery-white, not leaving a white residue on fingers when rubbed), glabrous or pubescent	5
3	(2.) Ligules (0.9–)1.5(–2.0) mm long, apex entire or with ciliations to 0.2 mm long; leaf blades 33–75 cm long, averaging 40 cm; pubescence below raceme sheaths moderate to dense; raceme sheaths (1.3–)2.0–2.5(–3.0) mm wide	*Andropogon glaucopsis*
–	Ligules (0.2–)0.4(–0.5) mm long, apex with ciliations 0.3–1.2 mm long; leaf blades 12–38 cm long, averaging 19 cm; pubescence below raceme sheaths absent to dense; raceme sheaths (2.7–)3.1–3.8(–5.5) mm wide	4
4	(3’.) Branchlet below attachment of raceme sheath glabrous at summit; raceme sheaths (2.1–)2.6–3.8(–4.9) cm long; spikelets (2.6–)3.2–3.5(–3.9) mm long; leaf blades 2–5 mm wide, averaging 3.5 mm	*Andropogon capillipes*+
–	Branchlet below attachment of raceme sheath pubescent at summit, hairs 2–4 mm long; raceme sheaths (2.4–)3.2–4.8(–6.0) cm long; spikelets (3.0–)3.5–3.9(–4.4) mm long; leaf blades 2.5–6.5 mm wide, averaging 5 mm	*Andropogon dealbatus*
5	(2’.) Inflorescence units with (2–)4–7(–13) racemes; raceme sheaths (4.1–)5.3–8.0(–10.1) mm wide; hairs of the rachis internodes and pedicels yellowish when dry	*Andropogon mohrii*
–	Inflorescence units with 2–5(–7) racemes; raceme sheaths (1.5–)2.0–4.8(–6.3) mm wide; hairs of the rachis internode and pedicel gray to white when dry	6
6	(5’.) Culm sheaths antrorsely scabrous (and often hirsute); leaf blades usually > 35 cm long	7
–	Culm sheaths not scabrous (though often hirsute); leaf blades < 35 cm long (except in *Andropogon tenuispatheus*)	9
7	(6.) Keels of lower glume often scabrous below the middle; ligules (0.6–)0.8(–1.3) mm long (usually < 1 mm long), apex with ciliations 0.2–0.9 mm long; raceme sheaths (1.5–)2.0–2.5(–3.0) mm wide (usually < 2.5 mm wide)	*Andropogon tenuispatheus*, in part
–	Keels of lower glume scabrous only above the middle, smooth below; ligules (0.7–)1.2(–2.2) mm long (usually > 1 mm long), apex entire or with ciliations to 0.3 mm long; raceme sheaths (2.0–)2.4–3.4(–4.7) mm wide (usually > 2.5 mm wide)	8
8	(7’.) Inflorescences oblong to obpyramidal; spikelets (3.8–)4.1–4.4(–5.0) mm long; anthers usually not withering and persistent within spikelet; mature peduncles (4–)11–35(–60) mm long (usually some > 10 mm long)	*Andropogon glomeratus*
–	Inflorescences (linear to) oblong; spikelets (3.4–)3.6–3.8(–4.6) mm log; anthers usually withering and persistent within spikelet; peduncles (2–)3–5(–8) mm long	*Andropogon hirsutior*
9	(6’.) Ligules (0.8–)1.1(–1.5) mm long, apex entire or with ciliations to 0.1 mm long; basal leaves often filiform, < 1.5 mm wide, strongly erect	*Andropogon perangustatus*
–	Ligules (0.2–)0.5(–0.9) mm long, apex with ciliations 0.2–1.3 mm long; basal leaves flat, usually > 2 mm wide, soon arching	10
10	(9’.) Keels of lower glume often scabrous below the middle; leaves usually > 44 cm long	*Andropogon tenuispatheus*, in part
–	Keels of lower glume scabrous only above middle; leaves usually < 31 cm long	11
11	(10’.) Raceme sheaths (2.2–)2.5–3.8(–4.5) cm long, (1.7–)2.4–3.1(–4.0) mm wide; racemes 2(3) per inflorescence unit; spikelets (3.0–)3.3–3.6(–4.0) mm long	Andropogon virginicus var. decipiens
–	Raceme sheaths (2.3–)3.4–5.2(–6.7) cm long, (2.7–)3.3–4.0(–5.5) mm wide; racemes 2–5(–7) per inflorescence unit; spikelets (2.9–)3.7–3.9(–4.7) mm long	Andropogon virginicus var. virginicus

Key adapted primarily from Weakley (2012), with supplemental information from Campbell (2003).

Note: The complex inflorescences of *Andropogon* necessitate the use of specialized terminology in botanical keys. In *Andropogon* (and *Schizachyrium*) paired spikelets create V-shaped dispersal units consisting of the following parts: the sessile spikelet at base; the pedicellate spikelet (usually vestigial or absent) at the summit of the pedicel, which forms one arm of the “V”; and the rachis internode (lacking a spikelet at summit), which forms the other arm of the “V.” (In the key below, spikelet measurements do not include lengths of awns, when present.) Several V-shaped dispersal units are aggregated one on top of the other to create a raceme (or “rame”). Several racemes are digitately aggregated at the apex of a peduncle and enclosed at least partially by a leaf-like raceme sheath. The racemes, peduncle, and subtending raceme sheath collectively form the inflorescence unit.

*Andropogon capillipes* Nash (sensu Weakley 2012), of dry to mesic pine flatwoods and sandhills, was reported from Sandy Run by Taggart (2010) (as “*Andropogon capillipes* Nash var. 2”). However, the voucher for this taxon (*Taggart SARU 647*, WNC) appears to the senior author to be *Andropogon dealbatus* (C. Mohr ex Hack.) Weakley & LeBlond (sensu Weakley 2012) based on the pubescence of the inflorescence branchlets subtending the raceme sheaths and the lengths of the raceme sheaths. Though not otherwise reported or collected in Shaken Creek Preserve or the vicinity, *Andropogon capillipes* may occur in the area and is therefore maintained in the key below, where indicated by a plus (+) symbol.

Fig. 80a, b, c, d, e

### [Poaceae] Aristida L.

**Table:** 

1	Plant forming dense, broad clumps, flowering only in the growing season following fire; leaves entirely or predominantly basal, mostly > 3 dm long, 0.5–1.5 mm wide, almost always tightly involute	*Aristida stricta*
–	Plant solitary or forming small clumps, flowering not strongly influenced by fire; leaves entirely or predominantly cauline, < 3 dm long (to 3.5 dm long in *Aristida palustris* with leaves 2–4 mm wide), flat to slightly folded but not tightly involute	2
2	(1’.) Lower glume prominently 2-keeled, (7.5–)9–13 mm long; central awns 15–40 mm long	*Aristida palustris*
–	Lower glume 1-keeled, or if 2-keeled, 6–9 mm long; central awns 10–20 mm long	3
3	(2’.) Awns spreading, central awn twisted basally; lower inflorescence nodes with (1–)2(–3) spikelets, 1 spikelet pedicellate, 1 spikelet sessile; basal culm internode 0.3–0.6 mm wide; callus beard (ring of hairs immediately subtending floret) 0.6–1.0 mm long	*Aristida simpliciflora*
–	Lateral awns usually erect to ascending, central awn not twisted basally; lower inflorescence nodes with (2–)3 or more spikelets, spikelets pedicellate to subsessile; basal culm internode 0.7–1.2 mm wide; callus beard 0.2–0.6 mm long	*Aristida virgata*

Key adapted from Allred (2003), Weakley (2012).

Fig. 81

### [Poaceae] Arundinaria Michx.

**Table:** 

1	Rhizomes lacking air canals; primary branches with 0–1 compressed basal internodes; culm internodes usually grooved above node; culm leaves deciduous, blades 0.8–1.3 cm wide	*Arundinaria gigantea*
–	Rhizomes with air canals; primary branches with 2–5 compressed basal internodes; culm internodes usually grooved above node; culm leaves persistent to tardily deciduous, blades 0.8–2 cm wide	*Arundinaria tecta*

Key adapted from Clark and Triplett (2007).

Fig. 82

### [Poaceae] *Coleataenia* Griseb.

**Table:** 

1	Rhizomes present; upper lemmas 1.6–4 mm long	2
–	Rhizomes absent, plants with hard, knotty crowns; upper lemmas 1.2–1.6 mm long	3
2	(1.) Rhizomes usually < 3 cm long; leaves 20–50 cm long, 4–18 mm wide; spikelets 2.5–3.9 mm long, acuminate; first glume with 3–5 green nerves	Coleataenia anceps ssp. anceps
–	Rhizomes usually > 4 cm long; leaves 10–30(–40) cm long, 2–10 mm wide; spikelets 2.2–2.8 mm long, acute to short-acuminate; first glume with 1–3 green nerves	Coleataenia anceps ssp. rhizomata
3	(1’.) Ligules 0.5–1.5 mm long; spikelets 2.4–4.0 mm long, 3.5–5× as long as wide, erect on pedicels	Coleataenia longifolia ssp. combsii
–	Ligules 1–3 mm long; spikelets 2.0–2.7 mm long, 2.5–4× as long as wide, often obliquely positioned on pedicels	Coleataenia longifolia ssp. longifolia

Key adapted from Weakley (2012).

References: Zuloaga et al. (2010).

Fig. 83

### [Poaceae] *Dichanthelium* (Hitch. & Chase) Gould

**Table:** 

1	Leaves basally disposed, basal leaf blades similar in size and shape to those of lower culm, usually erect to ascending, not forming a distinct rosette, culm leaves 2–4; culms with only upper 2–4 internodes elongated, branching from near base in fall	2
–	Leaves well-distributed along culm, basal leaf blades usually shorter and broader than those of culm, typically spreading, forming a distinct rosette, or basal blades absent, culm leaves 3–14; culms with usually all internodes elongated, typically branching from midculm nodes in fall	4
2	(1.) Leaf blades 1–3 mm wide, glabrous, margins eciliate or ciliate basally; spikelets 0.9–1.2 mm long, glabrous	Dichanthelium chamaelonche ssp. chamaelonche, in part
–	Leaf blades 3–8 mm wide; spikelets 1.1–2.1 mm long (if < 1.5 mm, then leaf blades *either* pubescent *or* margins ciliate to apex), glabrous or pubescent; [sect. *Strigosa*]	3
3	(2’.) Leaf blade surfaces glabrous; spikelets pubescent, 1.5–2.1 mm long	Dichanthelium strigosum var. leucoblepharis
–	Leaf blade surfaces pilose; spikelets glabrous, 1.1–1.6 mm long	Dichanthelium strigosum var. strigosum
4	(1’.) Leaf blades thick, bases cordate, margins white, cartilaginous; spikelets usually spherical to broadly obovoid or broadly ellipsoid, 1.2–1.8 mm long; [sect. *Sphaerocarpa*]	*Dichanthelium sphaerocarpon*
–	Leaf blades thick or thin, bases various but not cordate, margins usually not white and cartilaginous; spikelets not both spherical and < 1.9 mm long	5
5	(4’.) Lower glumes thinner and more weakly veined than upper glumes, attached ca. 0.2 mm below upper glumes, bases clasping pedicels; spikelets attenuate basally	6
–	Lower glumes similar in texture and vein prominence to upper glumes, attached immediately below upper glumes, bases not clasping pedicels; spikelets usually not attenuate basally	9
6	(5.) Leaf blades 2–7 cm long, ca. 10× as long as wide, not or only slightly involute, spreading, lacking raised veins, not longitudinally wrinkled; spikelets obovoid-obpyriform when viewed dorsally, strongly planoconvex when viewed laterally; [sect. *Lancearia*]	7
–	Leaf blades 4–16 cm long, > 14× as long as wide, often involute, stiffly erect or ascending, with prominently raised veins, lower blades usually longitudinally wrinkled; spikelets ellipsoid to obovoid when viewed dorsally, biconvex when viewed laterally; [sect. *Angustifolia*]	8
7	(6.) Fertile lemma and palea papillose; spikelets 2.2–2.6 mm long; lower culm leaf blades 6–12 mm wide, glabrous	*Dichanthelium webberianum*
–	Fertile lemma and palea minutely reticulate but not papillose; spikelets (1.8–)1.9–2.2(–2.3) mm long; lower culm leaf blades 4–8 mm wide, glabrous, glabrate, or puberulent (especially abaxially)	*Dichanthelium* species 3 (= *Dichantheliumlancearium*)
8	(6’.) Leaf blades 10–15× as long wide; spikelets 2.3–3.0 mm long	*Dichanthelium consanguineum*
–	Leaf blades 15–20× as long wide; spikelets 1.9–2.2 mm long	*Dichanthelium* species 12 (= *Dichantheliumchrysopsidifolium*)
9	(5’.) Rhizomes 3–5 mm thick; culm leaves (5–)7–14, sheaths strongly hispid or viscid, mottled with pale spots, constricted apically; plants to 1.5 m tall; [sect. *Clandestina*]	10
–	Rhzomes ≤ 2 mm thick, or absent; culm leaves 3–7(–9), sheaths not viscid, rarely hispid, not mottled with pale spots or constricted apically; plants typically < 1 m tall	11
10	(9.) Nodes glabrous or puberulent, neither swollen nor subtended by a glabrous, viscid band; leaf blades glabrous or sparsely pubescent; ligule membranous	*Dichanthelium scabriusculum*
–	Nodes densely bearded, often swollen, immediately subtended by a glabrous, viscid band; leaf blades (and sheaths and culm internodes) densely velvety-pubescent; ligule ciliate	*Dichanthelium scoparium*
11	(9’.) Ligule membranous basally, sometimes ciliate apically; leaf blades 5–25 mm wide, bases cordate-clasping; spikelets 2.2–3.7 mm long; [sect. *Macrocarpa*]	Dichanthelium commutatum var. commutatum, in part
–	Ligule absent or entirely ciliate, lacking membranous portion; leaf blades 1–12(–18) mm wide, bases tapered, round, or truncate, sometimes subcordate; spikelets 1.1–3 mm long	12
12	(11’.) Ligule ≤ 1.8 mm long, pseudoligule (a ring of longer, usually less dense hairs behind the shorter, denser hairs of the ligule proper) absent; culms and at least upper sheaths glabrous or sparsely pubescent with hairs of 1 length only; spikelets glabrous or pubescent	13
–	Ligule (including adjacent pseudoligule, when present) 1–5 mm long, *or* culms and sheaths puberulent (with very short hairs, often ca. 0.1 mm long) and with longer hairs; spikelets subglabrous to pubescent; [sect. *Lanuginosa*]	21
13	(12.) Leaf blades 5–25 mm wide, bases cordate-clasping; spikelets 2.2–3.7 mm long; [sect. *Macrocarpa*]	Dichanthelium commutatum var. commutatum, in part
–	Leaf blades 5–14 mm wide, bases tapered, round, or subcordate; spikelets 1.4–2.5 mm long	14
14	(13’.) Culms (20–)40–100 cm tall, not delicate, usually > 1 mm thick; spikelets 1.4–2.8 mm long; leaf blades 3.5–14 cm long, 5–14 mm wide; [sect. *Dichanthelium*]	15
–	Culms 5–40(–55) cm tall, delicate, usually < 1 mm thick; spikelets 1.1–1.7 mm long; leaf blades 1.5–6 cm long, 1.5–6 mm wide; [sect. *Ensifolia*]	19
15	(14.) Lower culm nodes bearded, beard hairs usually retrorse	16
–	Lower culm nodes glabrous or puberulent, not bearded	17
16	(15.) Spikelets 1.4–2.2 mm long; first glume 0.3–0.9 mm long; fertile lemma 1.4–1.7 mm long; lowest vernal culm blades glabrous	Dichanthelium dichotomum var. nitidum
–	Spikelets (2.0–)2.2–2.8 mm long; first glume 0.5–1.3 mm long; fertile lemma 1.8–2.3 mm long; lowest vernal culm blades pubescent at least abaxially	*Dichanthelium mattamuskeetense*
17	(15’.) Spikelets 1.4–1.8 mm long; first glume 0.3–0.8 mm long; fertile lemma 1.3–1.5 mm long; mature vernal panicles usually short-exserted with ascending branches; fresh foliage bluish-glaucous	*Dichanthelium caerulescens*
–	Spikelets 1.7–2.3 mm long; first glume 0.6–1.1 mm long; fertile lemma 1.6–1.9 mm long; mature vernal panicles usually long-exserted with spreading branches; fresh foliage not bluish-glaucous	18
18	(17’.) Vernal culm blades spreading to deflexed, flexuous; spikelets 1.8–2.3 mm long, base green, rarely purplish	Dichanthelium dichotomum var. dichotomum
–	Vernal culm blades stiffly erect; spikelets 1.5–1.8 mm long, base often purplish	Dichanthelium dichotomum var. roanokense
19	(14’.) Culms reclining or weakly erect; culm leaves 4–9, blades generally lacking prominent white, cartilaginous margins; ligules 0.2–1(–1.8) mm long	*Dichanthelium ensifolium*
–	Culms erect, sometimes geniculate basally; culm leaves 3–5, blades with prominent white, cartilaginous margins; ligules 0.2–0.7 mm long	20
20	(19’.) Spikelets 0.9–1.2 mm long, glabrous; leaf blades 1.5–4(–5) cm long, 1–2.5(–3) mm wide, mostly 15–20× as long as wide	Dichanthelium chamaelonche ssp. chamaelonche, in part
–	Spikelets (1.2–)1.4–1.7 mm long, pubescent; leaf blades 2–7 cm long, 3–6 mm wide, ca. 10× as long as wide	*Dichanthelium tenue*
21	(12’.) Spikelets 2.1–3 mm long; pseudoligule present; sheaths often with hairs to 4 mm long	22
–	Spikelets 1.1–2.1 mm long; pseudoligule absent; sheaths glabrous or pubescent with hairs ≤ 3 mm long	24
22	(21.) Node beard hairs retrorse; lower culm internodes and lower leaf sheaths with hairs spreading or retrorse, papillose-based, often > 4 mm long; spikelets 1.8–2.5 mm long	Dichanthelium villosissimum var. villosissimum
–	Node beard hairs spreading to ascending; lower culm internodes and lower leaf sheaths with hairs ascending or appressed, not papillose-based, < 4 mm long; spikelets 2.1–3.1 mm long	23
23	(22’.) Spikelets 2.1–2.6 mm long; lower culm blades usually sparsely appressed-pubescent adaxially, eciliate or ciliate at base only	Dichanthelium ovale var. addisonii
–	Spikelets 2.5–3.1 mm long; lower culm blades usually glabrous adaxially except for long hairs at or near margin, appearing ciliate	Dichanthelium ovale var. ovale
24	(21’.) Internodes glabrous	25
–	Internodes variously pubescent	26
25	(24.) Internodes glabrous to pubescent; larger vernal blades usually > 6 cm long, basal margins prominently long-ciliate; spikelets (1.3–)1.4–1.7 mm long; longer hairs of ligule usually > 3 mm long; plants often yellowish-green	Dichanthelium acuminatum var. lindheimeri, in part
–	Internodes glabrous (rarely the lowest slightly pubescent); larger vernal blades usually < 7 cm long, basal margins slightly ciliate or glabrous; spikelets 1.1–1.5 mm long; longer hairs of ligule usually < 3 mm long; plants often purplish-green	*Dichanthelium longiligulatum*
26	(24’.) Sheaths and internodes of vernal culms gray-villous, hairs 2–4 mm long, dense, tangled, or matted; leaf blades velvety-pubescent on abaxial surfaces, margins ciliate ≥ ½ length of leaf blade (from base to middle of leaf blade or further)	Dichanthelium acuminatum var. acuminatum
–	Sheaths and internodes of vernal culms glabrous or variously pubescent but not grayish-villous; leaf blades glabrous or pilose but not velvety-pubescent on abaxial surfaces, margins eciliate or ciliate < ½ length of leaf blade (only basally)	27
27	(26’.) Peduncle, panicle axis, and often middle and upper internodes glabrous; sheaths lacking hairs or papillae, at least near mid-length; nodes glabrous; spikelets (1.3–)1.4–1.7 mm long	Dichanthelium acuminatum var. lindheimeri, in part
–	Peduncle, panicle axis, and internodes puberulent (with hairs 0.1 mm long), pubescent, or pilose; sheaths papillose-pilose to hispid; nodes usually pubescent; spikelets 1.1–2 mm long	28
28	(27’.) Spikelets 1.5–2.0 mm long; leaf blades 5–12 cm long, 6–12 mm wide; peduncle, panicle axis, and sheaths variously pilose, not puberulent	Dichanthelium acuminatum var. fasciculatum
–	Spikelets 1.1–1.5 mm long; leaf blades 4–7 cm long, 4–7 mm wide; peduncle, panicle axis, and sheaths often puberulent, with or without longer hairs	*Dichanthelium leucothrix*

Key adapted from Freckmann and Lelong (2003a), Weakley (2012).

Note/Disclaimer: Keying specimens of *Dichanthelium* can be a painstaking, revision-filled process involving the interpretation of challenging characters and the repeated measurements of minute structures; positive identification is sometimes elusive. Nonetheless, with careful effort and practice, most specimens can be successfully identified. Several words of clarification, largely borrowed from Weakley (2012), are warranted regarding the key below. First, spikelet measurements refer only to mature spikelets, recognizable by their firm, plump, usually whitish fertile lemmas; immature spikelets are typically longer than mature spikelets and are not accounted for in the key. When measuring spikelet length, measure from the point of attachment of the lower glume to the apex of the highest spikelet structure (usually either the upper glume or the fertile lemma). Pubescence, an oft-used character in distinguishing *Dichanthelium* taxa, must also be evaluated carefully. When assessing internode pubescence, ignore the basalmost internode, which is generally shortened and uncharacteristic, and examine the first few elongated internodes. Nodes that are referred to as “bearded” have hairs that are longer, often denser, and of either a different structure or orientation than the hairs of the internodes and sheaths. Ligules are also an important diagnostic feature in *Dichanthelium*. When attempting to identify a specimen, examine several ligules, as the length and structure of ligules can vary within individuals. (For a more comprehensive overview of best practices in identifying *Dichanthelium* taxa, see the introduction to *Dichanthelium* in Weakley 2012.)

Figs 84, 85, 86

### [Poaceae] Eragrostis Wolf

**Table:** 

1	Pedicels divergent, lower pedicels of each branch longer than spikelets; lemmas 1.8–4.4 mm long; disarticulation of lemmas only, paleas and glumes persistent	*Eragrostis elliottii*
–	Pedicels appressed, lower pedicels of each branch shorter than spikelets; lemmas 1.4–2.8 mm long; disarticulation of lemmas and paleas, glumes persistent	*Eragrostis refracta*

Key adapted from Peterson (2003), Weakley (2012).

Fig. 87

### [Poaceae] Muhlenbergia Schreb.

**Table:** 

1	Rhizomes creeping, densely covered with imbricate scales; culms and sheaths flattened at base; leaves distichous; spikelets 1.5–2 mm long, unawned; plants forming clonal patches of evenly-spacted tufts	*Muhlenbergia torreyana*
–	Rhizomes absent; culm and sheaths terete; leaves not distichous; spikelets 2.5–5 mm long (excluding awns, if present), awned or not; plants forming non-clonal, broad tussocks	2
2	(1’.) Glumes < ½ as long as lemmas; lemmas with awns to 18 mm long	*Muhlenbergia capillaris*
–	Glumes > ½ as long as lemmas; lemmas unawned or with awns < 3 mm long	*Muhlenbergia expansa*

Key adapted from Weakley (2012).

Fig. 88a, b, c

### [Poaceae] Panicum L.

**Table:** 

1	Glumes and lower lemmas tuberculate; ligule 0.2–0.5 mm long	*Panicum verrucosum*
–	Glumes and lower lemmas smooth; ligule 0.5–6 mm long	2
2	(1’.) Panicle < 1 cm wide at maturity; upper glume and lower lemma 3–5-vJeined; ligule 0.5–1 mm long	*Panicum hemitomon*
–	Panicle 4–20 cm wide at maturity; upper glume and lower lemma 7–11-veined; ligule 0.5–6 mm long	3
3	(2’.) Plant lacking rhizomes or hard knotty crowns, annual; culms 30–60 cm tall, ≤ 2 mm wide; spikelets 1.8–2.2 mm long	Panicum dichotomiflorum var. puritanorum
–	Rhizomes or hard knotty crowns present; culms 40–300 cm tall, 3–5 mm wide; spikelets 2.5–8 mm long	*Panicum virgatum*

Key adapted from Freckmann and Lelong (2003b), Weakley (2012).

Fig. 89

### [Poaceae] Paspalum L.

**Table:** 

1	Spikelets solitary, not paired with rudimentary spikelets or naked pedicels	2
–	Spikelets paired, second spikelet functional, rudimentary, or at least represented by a naked pedicel	3
2	(1.) Panicles comprised of 1–6 racemosely-aranged branches; ligule 1.5–3.8 mm long	Paspalum laeve var. laeve‡
–	Panicles usually comprised of a pair of terminal branches, occasionally with 1(–5) branches below the terminal pair; ligule 0.2–0.5 mm long	*Paspalum notatum‡
3	(1’.) Spikelet margins silky-ciliate	4
–	Spikelet margins glabrous	5
4	(3.) Panicle branches 2–7; spikelets 2.3–4.0 mm long	*Paspalum dilatatum ssp. dilatatum‡
–	Panicle branches (4–)10–30; spikelets 1.8–2.8 mm long	*Paspalum urvillei‡
5	(3’.) Upper glumes 5-veined; spikelets 2.9–4.1 mm long	*Paspalum floridanum*
–	Upper glumes 3-veined; spikelets 1.1–3.1 mm long	6
6	(5’.) Panicle terminal; spikelets 2.1–3.1 mm long, 2–2.8 mm wide; [vars. of *Paspalum praecox*]	7
–	Panicles both terminal and axillary, axillary panicles often enclosed within subtending leaf sheath; spikelets 1.4–2.5 mm long, 1–2 mm wide; [vars. of *Paspalum setaceum*]	8
7	(6.) Lower leaf sheaths villous or hirsute	Paspalum praecox var. curtisianum
–	Lower leaf sheaths glabrous or sparsely papillose-pubescent	Paspalum praecox var. praecox
8	(6’.) Leaf blade surfaces glabrous or glabrate with a few hairs on the midvein, margins ciliate; leaves dark green to purple	Paspalum setaceum var. ciliatifolium
–	Leaf blade surfaces hiruste, margins hirsute; leaves light green to dark green	9
9	(8’.) Spikelets 1.8–2.5 mm long, 1.5–2.0 mm wide, light green to green; lower lemmas usually with evident midveins	Paspalum setaceum var. muhlenbergii
–	Spikelets 1.4–1.9 mm long, 1.1–1.6 mm wide, pale yellow to light green; lower lemmas usually lacking evident midveins	Paspalum setaceum var. setaceum

Key adapted from Radford et al. (1968), Allen and Hall (2003), and Weakley (2012).

Note: In disturbed areas adjacent to savannas (e.g., roadsides, powerline cuts, mowed areas), several weedy, generally exotic *Paspalum* taxa often co-occur with native *Paspalum* taxa. In order to facilitate accurate identification in such areas, these weedy taxa are included in the key below, where distinguished by a double-dagger symbol (‡).

Fig. 90

### [Poaceae] Saccharum L.

**Table:** 

1	Mature lemma awn spirally coiled at base, spirals usually 2–4	Saccharum brevibarbe var. contortum
–	Mature lemma awn straight to curved at base, not spirally coiled	2
2	(1’.) Callus beard (ring of hairs immediately subtending floret) longer than spikelets, (7–)15–20(–25) mm long; lowest inflorescence node densely pilose	*Saccharum giganteum*
–	Callus beard absent, or at most as long as spikelets, 0–5 mm long; lowest inflorescence node glabrous or sparsely pilose	3
3	(2’.) Callus beard (ring of hairs immediately subtending floret) absent or to 2 mm long, shorter than spikelets; panicles 1–2.5 cm wide	*Scleria baldwinii*
–	Callus beard 3–5 mm long, frequently as long as spikelets; panicles 3–7 cm wide	*Saccharum coarctatum*

Key adapted from Webster (2003) and Weakley (2012).

Fig. 91

### [Smilacaceae] Smilax L.

**Table:** 

1	Abaxial leaf surface (and often stem) glaucous	*Smilax glauca*
–	Abaxial leaf surface (and stem) green	2
2	(1’.) Stem prickles abundant, thin, needle-like, shiny, brown or black	*Smilax hispida*‡
–	Stem prickles fewer, broad-based and awl-like, green, brown, or black	3
3	(2’.) Leaf margins thickened with a prominent vein, occasionally spinose; peduncle ≥ 1.5 times as long as petiole of subtending leaf; prickles paired at most or all nodes	*Smilax bona-nox*
–	Leaf margins not thickened with a prominent vein, never spinose; peduncle < 1.5 times as long as petiole of subtending leaf; prickles not paired at most nodes	4
4	(3’.) Leaves deciduous or semi-evergreen, blades ovate, ovate-oblong, narrowly ovate, suborbicular, or reniform, base rounded to cordate	5
–	Leaves evergreen, blades oblong, oblong-lanceolate, oblong-linear, lance-ovate, or narrowly ovate, base cuneate to attenuate (sometimes rounded in *Smilax laurifolia*, with thick, evergreen leaves)	6
5	(4.) Berries dark blue to black; leaves semi-evergreen, blades ovate to broadly ovate, 4–17 cm long, 4–16 cm wide, margins usually with minute, whitish, flattened enations (small projections); plant of dry and wet habitats	*Smilax rotundifolia*
–	Berries red; leaves deciduous, blades ovate-lanceolate to ovate-oblong, 6–10 cm long, 3–7 cm wide, margins entire; plant of wet habitats	*Smilax walteri*‡
6	(4’.) Leaves thick, coriaceous, not variegated, blades linear, oblong, lance-oblong, or narrowly elliptic, not conspicuously reticulate, apex abruptly narrowed, acute or rounded, base 3-veined, midvein significantly more prominent than lateral veins as seen on abaxial surface	*Smilax laurifolia*
–	Leaves thin, subcoriaceous, often variegated, lanceolate-ovate to narrowly ovate, conspicuously reticulate, apex gradually narrowed, acute or acuminate, base 5-veined, midvein not more prominent than lateral veins as seen on abaxial surface	*Smilax smallii*

Key adapted from Holmes (2002), Weakley (2012).

Note: Two species keyed below—*Smilax hispida* Raf. and *Smilax walteri* Pursh — have been seen on site only in swamps. Though their discovery in other habitats is unlikely, both species could be found along the swampy margins of the wettest savannas. They are therefore included below, where indicated by a doube dagger symbol (‡).

Fig. 92a, b, c, d, e

### Tofieldiaceae

**Table:** 

1	Inflorescence bracts large, spathelike; tepals 9–17 mm long; stamens (6–)9(–12); plants usually forming dense, broad tussocks	*Pleea tenuifolia* Fig. 93
–	Inflorescence bracts minute, not spathelike; tepals 2.5–5 mm long; stamens 6; plants not forming dense, broad tussocks	2
2	(1’.) Flowers attached to the scape singly (inflorescence therefore a raceme); scape glabrous; flowering (late Aug–)late Sep–Oct	*Tofieldia glabra* Fig. 94
–	Flowers attached to the scape in groups of 3–7 (inflorescence therefore a thyrse); scape scurfy-scabrous; flowering Jun–Aug	*Triantha racemosa* Fig. 95

Key adapted from Weakley (2012).

### [Xyridaceae] Xyris L.

**Table:** 

1	Leaf blades ≤ 1 mm wide, terete or elliptical in cross-section	*Xyris baldwiniana*
–	Leaf blades (1–)2–25 mm wide, flat in cross-section	2
2	(1’.) Most leaf blades < 10 cm long (rarely some to 15 cm long in *Xyris brevifolia*, with spikes 5–7(–10) mm long)	3
–	Most leaf blades > 10 cm long (rarely some only 5 cm long in *Xyris ambigua*, with spikes 10–20(–30) mm long)	6
3	(2.) Keel of lateral sepals firm, entire to papillate or ciliolate	4
–	Keel of lateral sepals scarious, lacerate or fimbriate	5
4	(3.) Spikes mostly as broad as long; margins of fertile bracts scarious, lacerate, often reflexed, with red inner band; keel of lateral sepal straight to slightly curved, entire to remotely ciliate, apex distinctly reddish	*Xyris brevifolia*
–	Spikes mostly longer than broad; margins of fertile bracts firm, entire or erose, not reflexed, lacking red inner band; keel of lateral sepals strongly curved, densely ciliate, apex not distinctly reddish	*Xyris flabelliformis*
5	(3’.) Leaf blades spreading-recurved to ascending, 2–4.5 mm wide, bases pinkish or purplish; spikes 3–5(–7) mm long, often abruptly acute; seeds 0.4–0.5 mm long, yellowish-amber	*Xyris curtissii*
–	Leaf blades ascending to erect, 1–2 mm wide, bases tan to brown; spikes 3–7(–12) mm long, blunt; seeds 0.3–0.4 mm long, reddish-brown to brown	*Xyris* species 1
6	(2’.) Keel of lateral sepals long-fimbriate apically, fimbriate tip conspicuously protruding beyond subtending bract (sometimes degraded and less evident on older spikes)	7
–	Keel of lateral sepals short-ciliate or lacerate, tip included within subtending bract	8
7	(6.) Leaf blades strongly spirally twisted, (1.5–)2–5 mm wide, leaf bases indurated, bulbous, deeply set in substrate, dark brown	*Xyris caroliniana*
–	Leaf blades not or only slightly twisted, 5–25 mm wide, leaf bases soft, not bulbous, shallowly set in substrate, pale green	*Xyris fimbriata*
8	(6’.) Scapes flexuous, usually spirally twisted; upper portion of leaf blades conspicuously twisted; plant bases bulbous, deeply set in substrate	*Xyris scabrifolia*
–	Scapes usually not flexuous, usually not spirally twisted; upper portion of leaf blades not conspicuously twisted; plant bases neither bulbous nor deeply set in substrate	9
9	(8’.) Keel of lateral sepals firm, short-ciliate, strongly curved; petal blades 10 mm long	*Xyris ambigua*
–	Keel of lateral sepals scarious, lacerate, slightly curved; petal blades 3–4 mm long	10
10	(9’.) Scapes distinctly widened distally, 3–4 mm wide below spike; leaf blades 10–25 mm wide, sheaths red or purple; spikes 20–35 mm long	*Xyris iridifolia*
–	Scapes not distinctly widened distally, 0.5–3 mm wide below spike; leaf blades 1.5–5(–15) mm wide, sheaths red, tan, light green, brown, or purple; spikes 6–15(–25) mm long	11
11	(10’.) Leaf sheaths red to purple, surfaces papillate; seeds farinose (with a mealy surface), not translucent	*Xyris floridana*
–	Leaf sheaths tan, light green, or brown, surfaces smooth; seeds not farinose, translucent	*Xyris jupicai*

Key adapted from Kral (2000b), Weakley (2012).

Fig. 96a, b, c, d, e

### BASAL ANGIOSPERMS, MAGNOLIIDS, AND EUDICOTYLEDONS (KEY TO KEYS)

**Table:** 

1	Plant epiphytic	Santalacaceae [Phoradendron leucarpum ssp. leucarpum] Fig. 97
–	Plant terrestrial or aquatic, not epiphytic	2
2	(1’.) Plants woody; [trees, shrubs, and lianas]	Key 1
–	Plants herbaceous; [herbs and vines]	Key 2

### [BASAL ANGIOSPERMS, MAGNOLIIDS, AND EUDICOTYLEDONS] KEY 1: WOODY PLANTS (TREES, SHRUBS, AND LIANAS)

**Table:** 

1	Plant a liana, climbing by means of adventitious roots, tendrils, or twining stems	2
–	Plant a tree or shrub, not climbing	8
2	(1.) Leaves compound	3
–	Leaves simple	5
3	(2.) Leaves opposite, leaflets either 2 or 7–15	Bignoniaceae
–	Leaves alternate, leaflets 3–5(–7)	4
4	(3’.) Tendrils absent; leaves pinnately trifoliate, leaflets 3; terminal and axillary buds naked; fruit a white to gray drupe	Anacardiaceae [Toxicodendron radicans var. radicans] Fig. 98
–	Tendrils bearing terminal discs; leaves palmately compound, leaflets (3–)5(–7); terminal and axillary buds imbricate; fruit a black or dark blue berry	Vitaceae [*Parthenocissus quinquefolia*] Fig. 99
5	(2’.) Plants climbing by tendrils; leaves serrate, often shallowly 3–5(–7)-lobed	Vitaceae [Vitis rotundifolia var. rotundifolia] Fig. 100
–	Plants climbing by twining; leaves entire, unlobed	6
6	(5’.) Leaves alternate, deciduous; flowers inconspicuous, greenish-white	Rhamnaceae [*Berchemia scandens*] Fig. 101
–	Leaves opposite, evergreen to partially evergreen, flowers showy, yellow or red	7
7	(6’.) Leaves glaucous abaxially, distalmost 1 or 2 pairs (those immediately below inflorescence) usually connate; corollas red; fruit a berry	Caprifoliaceae [*Lonicera semepervirens*] Fig. 102
–	Leaves neither glaucous nor connate; corollas yellow; fruit a capsule	Gelsemiaceae [*Gelsemium sempervirens*] Fig. 103
8	(1’.) Leaves opposite or whorled	9
–	Leaves alternate	14
9	(8.) Leaves whorled (rarely some leaves alternate or opposite on fast-growing branches)	Ericaceae [*Kalmia carolina*] Fig. 104
–	Leaves opposite	10
10	(9’.) Leaves pinnately-compound	Oleaceae [*Fraxinus caroliniana*] Fig. 105
–	Leaves simple	11
11	(10’.) Leaves 3–5-lobed, palmately-veined	Sapindacae [*Acer rubrum*] Fig. 106
–	Leaves unlobed, pinnately-veined	12
12	(11’.) Leaf blades ≤ 2 cm wide, surfaces often glandular-punctate, petioles ≤ 3 mm long, bases with an articulation (narrow line, groove, or abrupt change of color and texture) at junction with stem; corolla yellow; stamens > 10; fruit a capsule	Hypericaceae [*Hypericum*, in part]
–	Leaf blades 1.5–12 cm wide, surfaces not glandular-punctate, bases lacking an articulation, petioles (at least some) ≥ 10 mm long; corollas white or creamy-white; stamens 4–5; fruit a drupe	13
13	(12’.) Hairs of abaxial leaf surface white, 2-branched (“Y-shaped”); petals 4	Cornaceae [*Cornus stricta*] Fig. 107
–	Hairs of abaxial leaf surface reddish, unbranched; petals 5	Adoxaceae [*Viburnum nudum*] Fig. 108
14	(8’.) Leaves compound	15
–	Leaves simple	19
15	(14.) Stems armed with numerous prickles and/or spines	16
–	Stems unarmed, lacking prickles and spines	17
16	(15.) Stems erect, to 8 m tall; leaves 2(–3)-pinnately compound, leaflets numerous (>10); fruit a drupe	Araliaceae [*Aralia spinosa*] Fig. 109
–	Stems arching, trailing, or erect, to 2 m tall; leaves 1-pinnately or 1-palmately compound, leaflets 3–9; fruit *either* an aggregate of drupes *or* an aggregate of achenes enclosed within a fleshy hypanthium (“hip”)	Rosaceae, in part
17	(15’.) Rachis winged between leaflets; fruit a drupe	Anacardiaceae [Rhus copallinum var. copallinum] Fig. 110
–	Rachis not winged; fruit a nut or legume	18
18	(17’.) Plant a small shrub, to 1.5 m tall; leaflets > 10, ≤ 3.2 cm long, not aromatic when crushed; fruit a legume	Fabaceae [*Amorpha*]
–	Plant a large tree, to 36 m tall; leaflets (5–)7–9, 4–19 cm long, strongly aromatic when crushed; fruit a nut	Juglandaceae [*Carya tomentosa*] Fig. 111
19	(14’.) Flowers borne in heads subtended by an involucre of bracts	Asteraceae [*Baccharis glomeruliflora*]
–	Flowers borne variously but not as above	20
20	(19’.) Leaves palmately 5–7-lobed, margins glandular-serrate; fruit a multiple of sharp-tipped capsules	Altingiaceae [*Liquidambar styraciflua*] Fig. 112
–	Leaves either unlobed or pinnately-lobed, margins various; fruit various but not a multiple of capsules	21
21	(20’.) Fruit a nut (acorn) bearing a basal cupule (“cap”); axillary buds clustered at twig tips, scales imbricate	Fagaceae [*Quercus*]
–	Fruit various but not a nut; axillary buds not *both* clustered at twig tips *and* with scales imbricate	22
22	(21’.) Leaves pinnately-lobed	23
–	Leaves unlobed	24
23	(22.) Leaf lobes (2)4 or 6(8), blade symmetrical, apex broadly-notched or truncate; fruit an aggregate of samaras	Magnoliaceae [*Liriodendron tulipifera*] Fig. 113
–	Leaf lobes 0–3, if 2 then blade asymmetrical (with central lobe larger than lateral lobe, leaf therefore distinctly “mitten-shaped”), apex obtuse to acute; fruit a drupe	Lauraceae [*Sassafras albidum*] Fig. 114
24	(22’.) Fruits dry (capsules, aggregates of follicles, or dry drupes)	25
–	Fruits fleshy (berries, pomes, or fleshy drupes)	33
25	(24.) Fruit a cone-like aggregate of follicles; seeds red, fleshy, pendent by thin threads (funiculi); stipular scars encircling twig	Magnoliaceae [*Magnolia virginiana*] Fig. 115
–	Fruit a capsule or dry drupe; seeds not red, fleshy, and pendent by thin threads; stipular scars not encircling twigs	26
26	(25’.) Flowers unisexual and arranged in catkins; leaves *either* aromatic when crushed and densely glandular-punctate (at least abaxially) *or* serrate and glaucous abaxially	27
–	Flower bisexual, or unisexual and not arranged in catkins; leaves various, but *neither* aromiatc and densely glandular-punctate abaxially *nor* serrate and glaucous abaxially	28
27	(26.) Leaves densely glandular-punctate (at least abaxially), margins coarsely toothed in distal half, not glaucous abaxially; fruit a dry drupe	Myricaceae [*Morella*]
–	Leaves not glandular-punctate, margins serrate throughout, glaucous abaxially; fruit a capsule	Salicaceae [*Salix caroliniana*] Fig. 116
28	(26’.) Plant prostrate; leaves 1–2.5 mm wide, evergreen	Diapensiaceae [*Pyxidanthera barbulata*] Fig. 117
–	Plant erect; leaves >3 mm wide, deciduous or evergreen	29
29	(28’.) Fruit a dry drupe, indehiscent; stems slightly ridged immediately below point of attachment of most petioles	Cyrillaceae [*Cyrilla racemiflora*] Fig. 118
–	Fruit a capsule, dehiscent; stems not ridged immediately below point of attachment of petioles	30
30	(29’.) Plant a tree, to 26 m tall; flowers solitary, axillary; stamens > 50	Theaceae [*Gordonia lasianthus*] Fig. 119
–	Plant a shrub, < 6 m tall; flowers numerous, borne in racemes or spikes; stamens ≤ 10	31
31	(30’.) Abaxial surface of leaf blade densely stellate-pubescent, hairs persistent; flowers mostly imperfect, borne in spikes; petals absent	Hamamelidaceae [*Fothergilla gardenii*] Fig. 120
–	Abaxial surface of leaf blade glabrous or variously pubescent, if stellate-pubescent then very sparsely so and hairs deciduous in age; flowers perfect, borne in racemes; petals present	32
32	(31’.) Young twigs, inflorescence rachises, pedicels, and calyces stellate-pubescent; leaves oblanceolate to elliptic-oblanceolate, widest above middle; corolla rotate, petals connate ≤ ½ length, lobes 5–8 mm long	Clethraceae [*Clethra alnifolia*] Fig. 121
–	Young twigs, inflorescence rachises, pedicels, and calyces glabrous or variously pubescent but not stellate-pubescent; leaves lanceolate, ovate, or elliptic, widest at or below middle (sometimes wider above middle in *Chamaedaphne calyculata*, with leaves and twigs distinctly scurfy-lepidote); corolla urceolate, campanulate, globose, or rotate, petals connate ≥ ½ length, lobes *either*< 4 mm long *or* 7–24 mm long	Ericaceae, in part
33	(24’.) Twigs and young bark with numerous horizontal lenticels appearing as distinct striations; twigs and crushed foliage with “bitter almond” smell; petioles bearing 2 glands near junction with blade	Rosaceae [Prunus serotina var. serotina] Fig. 122
–	Twigs and young bark lacking horizontal lenticels appearing as distinct striations; twigs and crushed foliage not aromatic, or aromatic but not with “bitter almond” smell; petioles not bearing 2 glands near junction with blade	34
34	(33’.) Fruit a red pome; leaves *either* serrate *or* crenate and with reddish trichomes on midrib of adaxial surface	Rosaceae, in part
–	Fruit a berry or drupe, seldom red at maturity; leaves various but *neither* serrate (except minutely so in Ericaceae [*Vaccinium tenellum*]) *nor* crenate and with reddish trichomes in midrib of adaxial surface	35
35	(34’.) Leaves evergreen	36
–	Leaves deciduous (sometimes tardily so)	38
36	(35.) Stems creeping, mat-forming; leaves (0.2–)0.3–1.8(–2.5) cm long; corolla urceolate, petals united to near apex; fruit a berry containing numerous (> 10) seeds	Ericaceae [*Vaccinium tenellum*]
–	Stems erect; leaves 2–14 cm long; corolla rotate, petals separate or united only at base; fruit a drupe containing either 1 or 4–8 seeds	37
37	(36’.) Leaves not aromatic when crushed, margins spinose, crenate, or occasionally entire, generally lacking deforming galls; drupes containing 4–8 seeds	Aquifoliaceae [*Ilex*]
–	Leaves spicy-aromatic when crushed, margins entire, often with numerous deforming galls; drupes containing 1 seed	Lauraceae [*Persea palustris*] Fig. 123
38	(35’.) Plant a shrub, generally multi-trunked; flowers perfect; fruit a berry, blue, purple, or black, < 2 cm in diam.; seeds ≥ 10, minute	Ericaceae, in part
–	Plant a small to large tree, single-trunked; flowers imperfect or perfect; fruit a drupe or berry, if berry then orange to yellow, (2–)3–5(–7.5) cm in diam., with 3–8 large seeds	39
39	(38’.) Leaves coriaceous, thick, usually sweet-tasting, tardily deciduous (some leaves persistent through mid-winter or early spring), petioles prominently yellow; flowers perfect; stamens 30–50, in 5 fascicles; fruit a green drupe, 3–6 mm in diam.	Symplocaceae [*Symplocos tinctoria*] Fig. 124
–	Leaves membranous, thin, not sweet-tasting, promptly deciduous (falling by mid- to late fall), petioles brown; flowers imperfect (or at least functionally so); stamens 5–16, distinct; fruit a berry or drupe, if drupe then blue-black, 7–12 mm in diam.	40
40	(39’.) Vascular bundle scars 1 per leaf scar; leaves generally widest at or below middle, not toothed; fruit a berry, orange at maturity, (2–)3–5(–7.5) cm in diam., subtended by accrescent, leathery calyx	Ebenaceae [*Diospyros virginiana*] Fig. 125
–	Vascular bundle scars 3 per leaf scar; leaves generally widest at or above middle, occasionally toothed; fruit a drupe, blue-black at maturity, 0.7–1.2 cm in diam., not subtended by accrescent, leathery calyx	Nyssaceae [*Nyssa*]

Key adapted from Radford et al. (1968), Weakley (2012).

### [BASAL ANGIOSPERMS, MAGNOLIIDS, AND EUDICOTYLEDONS] KEY 2: HERBACEOUS PLANTS (HERBS AND VINES)

**Table:** 

1	Flowers borne in heads subtended by an involucre of bracts	2
–	Flowers various, but not as above	3
2	(1.) Calyx present, not modified into scales, awns, or bristles; petals separate; fruit 2-seeded	Apiaceae [*Eryngium*]
–	Calyx absent or present and modified into scales, awns, or bristles; petals connate; fruit 1-seeded	Asteraceae
3	(1’.) Leaves compound (unifoliate and appearing simple in *Crotalaria purshii*, with mid- and upper cauline leaves bearing conspicuous, decurrent, inversely-sagittate stipules), pulvini (thickenings at base of petioles and petiolules) evenly cylindrical; corollas zygomorphic; fruit a legume	Fabaceae
–	Leaves compound or simple (if simple, then mid- and upper cauline leaves lacking conspicuous, decurrent, inversely-sagittate stipules), pulvini absent or not evenly cylindrical; corollas zygomorphic, actinomorphic, or absent; fruit various, not a legume	4
4	(3’.) Plant with stinging trichomes	Euphorbiaceae, in part
–	Plants lacking stinging trichomes	5
5	(4’.) Plants carnivorous	6
–	Plants not carnivorous	7
6	(5.) Leaf blades (at least some) modified into “snap-traps” consisting of 2 lobes, lobes subreniform, hinged, margins bristly; inflorescence an umbelliform cyme; [Venus flytrap]	Droseraceae [*Dionaea muscipula*] Fig. 126
–	Leaf blades not as above; inforescence racemose, or flower solitary	7
7	(6’.) Leaves (at least some) modified into prominent, water-storing, tubular pitchers	Sarraceniaceae [*Sarracenia*]
–	Leaves not modified into pitchers	8
8	(7’.) Plants terrestrial or aquatic, producing subterranean or aquatic bladders; leaves filiform; corolla purple or yellow	Lentibulariaceae [*Utricularia*]
–	Plants terrestrial, not producing bladders; leaves ovate, lanceolate or spatulate; corolla white, pink, blue, or purple	9
9	(8’.) Leaves red, long-petiolate, with prominent stipitate-glands, margins flat, or at least not involute; corolla actinomorphic	Droseraceae [*Drosera*]
–	Leaves yellow-green, sessile, lacking prominent stipitate-glands, margins involute; corolla zygomorphic	Lentibulariaceae [*Pinguicula*]
10	(5’.) Plants with milky sap	11
–	Plants with clear sap	13
11	(10.) Corona (appendages between petals and stamens) present; fruit a follicle	Apocynaceae
–	Corona absent; fruit a capsule	12
12	(11’.) Leaves alternate, serrate; flowers perfect, not borne in a cyathium (flower-like involucre often with petaloid appendages; staminate flowers consisting of a single stamen, pistillate flowers of a single pistil); corolla present, blue or purple (rarely all white)	Campanulaceae [*Lobelia*, in part]
–	Leaves opposite, entire; flowers imperfect, borne in a cyathium; corolla absent (though petaloid appendages of cyathia present, these greenish)	Euphorbiaceae [*Euphorbia ipecacuanhae*] Fig. 127
13	(10’.) Plants holoparasitic; stems bright orange, twining; leaves and roots absent	Convolvulaceae [*Cuscuta*]
–	Plants autotrophic or hemiparasitic; stems generally green or brown, not orange, erect; leaves and roots present (sometimes reduced)	14
14	(13’.) Cauline leaves absent, leaves all basal or appearing so	15
–	Leaves cauline or basal and cauline	18
15	(14.) Flower solitary	16
–	Inflorescence a spike or head-like umbel	17
16	(15.) Leaves entire, unlobed; flowers actinomorphic; petals white with conspicuous green venation; staminodia 5, 3-parted basally	Parnassiaceae [*Parnassia caroliniana*] Fig. 128
–	Leaves crenate, lobed, or dissected; flowers zygomorphic; petals white, blue, or purple, lacking conspicuous green venation; staminodia absent	Violaceae [*Viola*]
17	(15’.) Leaves ovate, 1.5–4(–10) cm long; inflorescence an umbel	Apiaceae [*Centella erecta*] Fig. 129
–	Leaves lanceolate to elliptic-lanceolate, 10–20 cm long; inflorescence a spike	Plantaginaceae [*Plantago sparsiflora*] Fig. 130
18	(14’.) Sepals dimorphic, outer 2 linear, inner 3 ovate to elliptic; petals 3, reddish; plants producing overwintering, prostrate shoots in late fall	Cistaceae [Lechea pulchella var. ramosissima]
–	Sepals similar, or dissimilar but not as above; petals various; plants not producing overwintering, prostrate shoots	19
19	(18’.) Calyx connate, lobes 5, 2 lateral lobes (“wings”) relatively large, petaloid; corolla usually smaller than calyx, connate, lobes 3, lower lobe usually lacerate, fringed, or lobed apically	Polygalaceae [*Polygala*]
–	Calyx and corolla free or connate, if connate then not as above	20
20	(19’.) Lower cauline leaves opposite, upper cauline leaves alternate (excluding those with 1 pair of opposite leaves subtending inflorescence)	21
–	All cauline leaves either alternate or opposite (rarely whorled	23
21	(20.) Flower zygomorphic; corolloa blue or purple	Plantaginaceae [*Nuttallanthus canadensis*] Fig. 131
–	Flower actinomorphic; corolla white or yellow	22
22	(21’.) Leaves scale-like, 1–3 mm long; corolla white	Gentianaceae [*Bartonia*]
–	Leaves not scale-like, 8–35 mm long; corolla yellow	Linaceae [*Linum*]
23	(20’.) Leaves in whorls of 3 or 4	Primulaceae [*Lysimachia*, in part]
–	Leaves alternate or opposite	24
24	(23’.) Leaves opposite	25
–	Leaves alternate	45
25	(24.) Leaves pinnately decompound, segments filiform	Orobanchaceae [*Seymeria cassioides*]
–	Leaves simple	26
26	(25’.) Ovary wholly or partly inferior	27
–	Ovary superior	29
27	(26.) Leaves auriculate-clasping, to 15 cm long	Lythraceae [*Ammannia coccinea*, in part] Fig. 132
–	Leaves not auriculate-clasping, 0.8–7 cm long; petals present	28
28	(27’.) Stems erect; leaf venation distinctive (secondary veins subparallel to primary vein, rejoining apically; tertiary veins perpendicular to secondary veins); interpetiolar stipules absent; hypanthium conspicuous, urceolate at maturity; petals usually pink, rarely white	Melastomataceae [*Rhexia*]
–	Stems prostrate, trailing, or erect; leaf venation pinnate, not as above; interpetiolar stipules present; hypanthium not conspicuous, not urceolate; petals usually white, rarely pink	Rubiaceae
29	(26’.) Plant weakly climbing (by means of twisted petioles); calyx petaloid, bluish; petals absent	Ranunculaceae [*Clematis crispa*] Fig. 133
–	Plants erect or trailing, not climbing; calyx not petaloid, usually green; petals present (sometimes absent in Lythraceae [*Ammannia coccinea*])	30
30	(29’.) Plants hemiparasitic; leaves linear, sometimes reduced to scales, bases not auriculate-clasping; corolla weakly zygomorphic, purple or pinkish	Orobanchaceae [*Agalinis*]
–	Plants not parasitic; leaf various, bases various; corolla actinomorphic or zygomorphic, color various (if purple or pinkish, then *either* corolla strongly zygomorphic and leaves not linear or reduced to scales *or* corolla actinomorphic and leaf bases distinctly auriculate-clasping)	31
31	(30’.) Corollas zygomorphic (weakly zygomorphic *Sophronanthe pilosa* keyed here and below)	32
–	Corollas actinomorphic	36
32	(31.) Ovary appearing 4-lobed; fruit a schizocarp of 4 nutlets (or 1–3 by abortion)	Lamiaceae
–	Ovary appearing unlobed or 2-lobed; fruit a a capsule, seeds numerous	33
33	(32’.) Inflorescence a distinct thyrse or panicle	Plantaginaceae [*Penstemon*]
–	Flowers axillary	34
34	(33’.) Corolla 2.5–4 cm long; functional stamens 4	Phrymaceae [Mimulus ringens var. ringens] Fig. 134
–	Corolla 0.6–1.1 cm long; functional stamens 2 (sometimes 2 staminodes present)	35
35	(34’.) Stems glabrous, usually diffusely branched from base; leaves glabrous; pair of bractlets immediately subtending calyx absent; staminodes conspicuous	Linderniaceae [Lindernia dubia var. anagallidea] Fig. 135
–	Stems pilose, usually unbranched; leaves pilose; pair of bractlets immediately subtending calyx present; staminodes reduced or absent	Plantaginaceae [*Sophronanthe pilosa*, in part] Fig. 136
36	(31’.) Corolla yellow	37
–	Corolla white, pink, blue, or lavender	38
37	(36.) Leaves opposite; flowers solitary at tips of branches or borne in cymes (flowers racemose in *Hypericum gentianoides*, with leaves reduced to scales); petals separate; stamens generally > 10	Hypericaceae [*Hypericum*, in part]
–	Leaves opposite or whorled, never reduced to scales; flowers borne in terminal racemes; petals connate basally; stamens 5	Primulaceae [*Lysimachia*, in part]
38	(36’.) Stems wiry, usually purplish; leaves scale-like, 1–3 mm long; corolla white	Gentianaceae [*Bartonia*]
–	Stems thicker, not wiry, usually green; leaves not scale-like, > 5 mm long; corolla white, pink, blue, or purple	39
39	(38’.) Plants somewhat succulent; leaves (at least the lower) auriculate-clasping; corollas absent, or to 2 mm long	Lythraceae [*Ammannia coccinea*, in part] Fig. 132
–	Plants not succulent; leaves not auriculate-clasping; corollas present, ≥ 1 mm long	40
40	(39’.) Corolla white, 1–3 mm long	41
–	Corolla white, pink, or blue, > 3 mm long	42
41	(40.) Leaves > 5 mm wide; inflorescence helicoid (with flowers borne on one side of a spiral), not leafy	Loganiaceae [*Mitreola*]
–	Leaves < 3 mm wide; inflorescence not helicoid, leafy	Tetrachondraceae [*Polypremum procumbens*] Fig. 137
42	(40’.) Corolla blue to violet (rarely whitish), campanulate, tubular, or funnelform, 3–6 cm long, bearing pleat-like appendages between corolla lobes	Gentianaceae [*Gentiana*]
–	Corolla white or pink, rotate or petals free, < 3 cm long, lacking pleat-like appendages	43
43	(42’.) Stems pilose; leaves serrate to entire, pilose; corolla white, obscurely zygomorphic, petals 4	Plantaginaceae [*Sophronanthe pilosa*, in part] Fig. 136
–	Stems glabrous; leaves entire, glabrous; corolla white or pink, actinomorphic, petals 5–12	44
44	(43’.) Petals 5–12, connate basally, white or pink; stamens not fascicled	Gentianaceae [*Sabatia*]
–	Petals 5, free, pink; stamens fascicled in 3 groups of 3	Hypericaceae [*Hypericum virginicum*]
45	(24’.) Inflorescence of umbels; leaves pinnately- or ternately-compound, or reduced to septate phyllodes lacking blades	Apiaceae, in part
–	Inflorescence various, not of umbels; leaves simple or variously-compound, not reduced to septate phyllodes lacking blades	46
46	(45’.) Leaves pinnately lobed, divided, or dissected	47
–	Leaves not lobed, divided, or dissected, or leaves ternately-compound	48
47	(46.) Leaves pinnately dissected, leaf segments filiform; flowers 3-merous, ca. 1 mm long; petals absent	Haloragaceae [*Proserpinaca palustris*]
–	Leaves pinnately lobed or divided, leaf segments broader, not filiform; flowers 4-merous, 18–22 mm long; petals present	Orobanchaceae [*Pedicularis canadensis*] Fig. 138
48	(46’.) Leaves compound	49
–	Leaves simple	50
49	(48.) Leaves ternately-compound or -decompound, leaflets 3–9, margins entire; flowers imperfect, petals absent	Ranunculaceae [*Thalictrum cooleyi*] Fig. 139
–	Leaves palmately compound, leaflets 5, margins serrate; flowers perfect, petals present	Rosaceae [*Potentilla simplex*] Fig. 140
50	(48’.) Corolla blue, zygomorphic; ovary superior	Campanulaceae [*Lobelia*, in part]
–	Corolla yellow or white, actinomorphic, or corolla absent; ovary inferior	Onagraceae

Key adapted in part from Radford et al. (1968).

### Auxiliary Key To Common Herbs With Opposite, More-Or-Less Ovate Leaves

**Table:** 

1	Leaves with translucent glandular dots when backlit, margins entire; corollas yellow	Hypericaceae [*Hypericum*]
–	Leaves lacking translucent glandular dots when backlit, margins various; corollas white, sometimes shaded with lavender or pink	2
2	(1’.) Interpetiolar stipules present, lacerate or fimbriate	Rubiaceae
–	Interpetiolar stipules absent	3
3	(2’.) Stems conspicuously pubescent or stipitate-glandular, hairs or glands 1(–1.5) mm long; flowers axillary, pedicels absent or present and much shorter than subtending leaf	4
–	Stems glabrous or glabrate, hairs (if present) scattered, short (< 0.5 mm long); flowers *either* terminal in helicoid cymes *or* axillay and with pedicels to 25 mm long, much longer than subtending leaf	5
4	(3.) Stems stipitate-glandular, sticky to the touch; leaves petiolate; corolla purple	*Cuphea carthagenensis‡
–	Stems pilose, not sticky to the touch; leaves sessile; corolla white	Plantaginaceae [*Sophronanthe pilosa*] Fig. 136
5	(3’.) Leaves strongly ascending to appressed, margins entire; flowers borne in a terminal helicoid cyme	Loganiaeae [*Mitreola sessilifolia*] Fig. 141b
–	Leaves spreading, margins serrulate or entire; flowers axillary or borne in a terminal helicoid cyme	6
6	(5’.) Stems usually strongly quadrangular; leaves usually strongly aromatic, margins serrate, crenate, or shallowly lobed, rarely entire; fruit a schizocarp of 4 nutlets (or 1–3 by abortion)	Lamiaceae
–	Stems terete or only slightly quadrangular; leaves not aromatic, margins serrulate or entire; fruit a capsule	7
7	(6’.) Leaves 3–8 cm long, 1–3.5 cm wide, margins entire, petioles 3–15 mm long; flowers borne in a terminal helicoid cyme	Loganiaeae [*Mitreola petiolata*] Fig. 141a
–	Leaves 0.5–4.5 cm long, 0.5–1.2 cm wide, margins serrulate or entire, petioles 0–2(–3) mm long; flowers axillary	8
8	(7’.) Leaves ovate to ovate-lanceolate, 5–17 mm long, 2–8 mm wide, bases broadly rounded	Linderniaceae [Lindernia dubia var. anagallidea] Fig. 135
–	Leaves elliptic to oblanceolate, 10–45 mm long, 5–12 mm wide, bases narrowly cuneate	Mecardonia acuminata var. acuminata‡

Note: The following key provides a means of distinguishing some of the common savanna and roadside herbs with opposite, more or less ovate leaves. Though occasional in ditches and roadsides on site, *Cuphea carthagenensis* (Jacq.) J.F. Macbr. (Lythraceae) and Mecardonia acuminata (Walter) Small var. acuminata (Plantaginaceae) have not been found in savannas or flatwoods at Shaken Creek Preserve or in the vicinity. These taxa are not formally treated in this work but are included in the key below, where indicated by a double-dagger symbol (‡).

### Anacardiaceae

**Table:** 

1	Plant a shrub or small tree; leaves imparipinnate, leaflets 15–31, rachis winged; inflorescences dense, terminal; fruits red, glandular-pubescent	Rhus copallinum var. copallinum Fig. 110
–	Plant a shrub or vine climbing by means of adventitious roots; leaves trifoliolate, rachis unwinged; inflorescences openly branched, axillary; fruits white or yellow, glabrous or puberulent	Toxicodendron radicans var. radicans Fig. 98

Key adapted from Weakley (2012).

### Apiaceae

**Table:** 

1	Plants acaulescent; leaves simple, ovate to oblong, erect, membranous to subcoriaceous, conspicuously palmately-veined from base; involucre of 2 conspicuous, ovate bracts	*Centella erecta* Fig. 129
–	Plants caulescent; leaves deeply divided (appearing compound) or if simple, then spreading, coriaceous, and not distinctly palmately-veined from base; involucre various, but not of 2 conspicuous, ovate bracts	2
2	(1’.) Flowers borne in compact, globose to subglobose heads, blue, green, or white, subtended individually by a tricuspidate or ovate to lanceolate bractlet	* Eryngium *
–	Flowers borne in open umbels, white, not subtended individually by a bractlet (though entire umbellets subtended by an involucel of inconspicuous, linear bractlets)	3
3	(2’.) Leaf blades absent, leaves reduced to septate, terete, hollow phyllodes	* Tiedemannia *
–	Leaf blades present, leaves compound or decompound, not reduced to phyllodes	4
4	(3’.) Leaves pinnately or ternately compound, leaflets 1–13, linear, lanceolate, or narrowly elliptic, 5–40 mm wide; fruit strongly flattened dorsally, prominently winged; plants 6–15 dm tall; roots tuberous-thickened	* Oxypolis *
–	Leaves pinnately decompound, ultimate leaf segments numerous (> 13), filiform, < 1 mm wide; fruit subterete, not winged; plants 1–8 dm tall; roots fibrous	*Ptilimnium capillaceum* Fig. 142

Key adapted from Radford et al. (1968), Feist et al. (2012), and Weakley (2012).

### [Apiaceae] Eryngium L

**Table:** 

1	Basal and lower cauline leaf blades lanceolate, ovate, ellipitic, or oblong, 3–7(–10) cm long, apex acute to obtuse, base cordate to truncate, with a length/width ratio of 1.5–3(–6)	*Eryngium integrifolium*
–	Basal and lower caluine leaf blades linear to oblanceolate, 10–100 cm long, apex acuminate to acute, base clasping, with a length/width ratio of 5–50	2
2	(1’.) Leaves with major veins parallel, margins bristly; flowers green or greenish white	3
–	Leaves with major veins pinnate-reticulate, margins bristly or entire; flowers bluish	4
3	(2.) Larger leaves < 1.5 cm wide, marginal bristles on basal portion of leaf usually in fascicles of 2–3 (often requiring careful examination to see)	Eryngium yuccifolium var. synchaetum
–	Larger leaves > 1.5 cm wide, marginal bristles of leaves solitary	Eryngium yuccifolium var. yuccifolium
4	(2’.) Mature styles 3.0–3.5 mm long, slightly exceeding bractlets; middle cusp of bractlets elongate, distinctly longer than lateral cusps; heads subglobose to hemispherical, 6–12 mm in diam.	Eryngium aquaticum var. aquaticum
–	Mature styles 4.0–6.0 mm long, much exceeding bractlets; middle cusp of bractlets subequal in length to lateral cusps; heads globose, 9–15 mm in diam.	Eryngium aquaticum var. ravenelii

Key adapted from Weakley (2012).

Fig. 143

### [Apiaceae] Oxypolis Raf.

**Table:** 

1	Leaf blades absent, leaves reduced to septate, terete, hollow phyllodes	[*Tiedemannia*]
–	Leaf blades present, leaves compound or decompound, not reduced to phyllodes	2
2	(1’.) Leaves pinnately-compound; leaflets (5–)7–11(–13), usually toothed (rarely entire), venation reticulate	*Oxypolis rigidior*
–	Leaves ternately-compound; leaflets 1–3, entire, venation parallel	*Oxypolis ternata*

Key adapted from Feist et al. (2012), Weakley (2012).

Fig. 144

### [Apiaceae] Tiedemannia Dc.

**Table:** 

1	Mature fruits with corky-thickened peripheral ribs, narrowly rectangular in cross-section, edges 0.8–2 mm wide, nearly as thick as at center of fruit; plants with stoloniferous rhizomes 1–3(–10) dm long; lower nodes often losing their leaves by flowering	*Tiedemannia canbyi*‡
–	Mature fruits with peripheral ribs progressively thinning away from seed cavity, lenticular in cross-section, edges 0.2 mm thick, distinctly thinner than at center of fruit; plants with stout rhizomes or caudices, not long-stoloniferous; lower nodes usually retaining their leaves until flowering	Tiedemannia filiformis ssp. filiformis

Key adapted from Feist et al. (2012), Weakley (2012).

Note: *Tiedemannia canbyi* (J.M. Coult. & Rose) Feist & S.R. Downie, a federally listed endangered species of clay-based Carolina bays and other depressional wetlands, has not been seen at Shaken Creek Preserve or in the vicinity. Nevertheless, since suitable habitat exists on site, the presence of *Tiedemannia canbyi* at Shaken Creek Preserve or in the vicinity should perhaps not be completely dismissed. (See Feist et al. (2012) for a discussion of the phylogeny of *Oxypolis* *s.l.* and the circumscription of *Tiedemannia* and *Oxypolis* *s.s.*)

Fig. 145

### [Apocynaceae] Asclepias L.

**Table:** 

1	Leaf blades 2.5–4.5 cm long, puberulent below; corolla lobes erect, creamy yellow to greenish white	*Asclepias pedicellata*
–	Leaf blades 7–20 cm long, glabrous below or pubescent along veins; corolla lobes reflexed, orange, red, lavender, or greenish white and apically tinged with rose-purple	2
2	(1’.) Corolla lobes greenish white with rose-purple tips, 3.5–5 mm long; leaves opposite, subopposite, or whorled	*Asclepias longifolia*
–	Corolla lobes orange, red, or lavender, 7–11 mm long; leaves opposite	3
3	(2’.) Leaf blades linear to narrowly lanceolate, 7–20 cm long, 0.5–1.5 cm wide; corolla lobes orange or bright red, 8–11 mm long	*Asclepias lanceolata*
–	Leaf blades lanceolate, 9–12 cm long, 1.5–3 cm wide; corolla lobes dull red to lavender, 7–9 mm long	*Asclepias rubra*

Key adapted from Radford et al. (1968), Weakley (2012).

Fig. 146

### [Aquifoliaceae] Ilex L.

**Table:** 

1	Leaves with spinose prickles (at least one at apex, and usually several along margins), prickles 2–6 mm long; adaxial leaf surface dull	Ilex opaca var. opaca
–	Leaves *either* lacking spinose prickles *or* with prickles < 1 mm long; adaxial leaf surface lustrous	2
2	(1’.) Drupes red (rarely orange or yellow); calyx and corolla 4-lobed; leaf blades lanceolate to narrowly oblong, 3–7 times as long as wide, usually entire, lacking dark punctate dots below	*Ilex myrtifolia*
–	Drupes black; calyx and corolla 5–9-lobed; leaf blades obovate to elliptic, 1.5–4 times as long as wide, entire, crenate, or with spinose prickles, with dark punctate dots below	3
3	(2’.) Leaf blades 1.5–3× as long as wide, usually 2–3 cm wide, margins entire or spinose, prickles (when present) projecting outward from leaf margin; drupe 7–10 mm in diam., lustrous	*Ilex coriacea*
–	Leaf blades 3–4× as long as wide, rarely as wide as 2 cm, margins entire basally, crenate apically (rarely entire throughout), prickles curving forward along leaf margin; drupe 5–7 mm in diam., dull or slightly lustrous	*Ilex glabra*

Key adapted from Radford et al. (1968), Weakley (2012).

Fig. 147

### Asteraceae

**Table:** 

1	Plant a shrub	*Baccharis glomeruliflora*
–	Plant an herb or twining vine	2
2	(1’.) Plant a twining vine; leaves opposite, bases cordate, margins coarsely toothed	*Mikania scandens* Fig. 148
–	Plant an herb; leaves alternate, opposite, or whorled, bases various, margins various	3
3	(2’.) Plants with milky sap; heads liguliflorous (with only ray flowers)	4
–	Plants with clear sap; heads discoid (with only disc flowers) or radiate (with both ray and disc flowers)	6
4	(3.) Heads nodding, spicate or racemose; ray flowers pinkish or purple	*Prenanthes autumnalis* Fig. 149
–	Heads erect, paniculiform or thyrsiform; ray flowers yellow	5
5	(4’.) Leaf margins entire or denticulate; involucres 7–10 mm long; phyllaries lanceolate or linear, neither distinctly widest nor bilobed at apices; cypselae not beaked	*Hieracium gronovii* Fig. 150
–	Leaf margins dentate to pinnately lobed; involucres 17–24 mm long; phyllaries widest at apices, often bilobed apically; cypselae with beak 7–10+ mm long	*Pyrrhopappus carolinianus* Fig. 151
6	(3’.) Heads discoid	7
–	Heads radiate	20
7	(6.) Leaves and phyllaries prominently prickly-spiny; [thistles]	* Cirsium *
–	Leaves and phyllaries not prickly-spiny	8
8	(7’.) Most or all leaves opposite or whorled (distal cauline leaves sometimes alternate, but majority of leaves still opposite or whorled); flowers white	* Eupatorium *
–	Leaves *either* alternate *or* predominantly basal with cauline leaves few, reduced; flower color various	9
9	(8’.) Leaves densely white-tomentose beneath, hairs entirely obscuring green leaf surface	10
–	Leaves glabrous or pubescent beneath, if pubescent then hairs not entirely obscuring green leaf surface	11
10	(9.) Leaf bases not decurrent, stems not appearing winged	*Pseudognaphalium obtusifolium* Fig. 152
–	Leaf bases decurrent, stems therefore appearing conspicuously winged	*Pterocaulon pycnostachyum* Fig. 153
11	(9’.) Heads enclosed by 3 ovate to deltate, leaf-like bracts	*Elephantopus nudatus* Fig. 154
–	Heads not enclosed by 3 leaf-like bracts	12
12	(11’.) Heads spicate (rarely racemose)	* Liatris *
–	Heads paniculate, corymbose, or thysiform	13
13	(12’.) Phyllaries of similar length, in 1 series	14
–	Phyllaries of differing lengths, in ≥ 2 series	15
14	(13.) Leaves glaucous beneath, margins entire; involucres 8–10 mm long; cypselae 4–5 mm long	Arnoglossum ovatum var. lanceolatum Fig. 155
–	Leaves not glaucous beneath, margins subentire to serrate or weakly pinnately-lobed; involucres 10–17 mm long; cypselae 2.3–3 mm long	*Erechtites hieraciifolius* Fig. 156
15	(13’.) Basal rosette absent	16
–	Basal rosette present, apparent at anthesis	17
16	(15.) Outer phyllaries obtuse to acute; flowers tiny, individually indistinct, numerous (ca. 100+ per head), corollas pink, purple, or white; leaf surfaces not scabrous	* Pluchea *
–	Outer phyllaries acuminate; flowers relatively large, individually distinct, less numerous (ca. 161–20 per head), corollas purple; leaf surfaces (at least adaxial) somewhat scabrous	* Vernonia *
17	(15’.) Flowers yellow; heads in flat-topped corymbs	Bigelowia nudata var. nudata Fig. 157
–	Flowers purple or whitish; heads corymbose (flat-topped or rounded), paniculate, or thyrsiform	18
18	(17’.) Peduncles 5–50 cm long; flowers purple to pale-lavender, rarely whitish; pappus of 5 scales	*Marshallia graminifolia* Fig. 158
–	Peduncles < 5 cm long; flowers purple; pappus of numerous capillary bristles	19
19	(18’.) Involucres mostly 7–12(–15) mm; phyllaries 15–40+ in 3–5+ series; leaves with resin dots	* Carphephorus *
–	Involucres 3.5–6 mm; phyllaries 5–12 in 1–2(–3) series; leaves lacking resin dots	* Trilisa *
20	(6’.) Abaxial leaf surface densely white-tomentose, appearing solidly white, adaxial leaf surface glabrous to glabrate, green, margins denticulate; heads terminal, solitary; plants flowering Feb–May	*Chaptalia tomentosa* Fig. 159
–	Leaf surfaces glabrous or variously pubescent but not densely white-tomentose and appearing solidly white abaxially, margins various; heads various; flowering May–Nov (except in *Erigeron vernus*, which flowers as early as late Mar)	21
21	(20’.) Ray flowers yellow	22
–	Ray flowers white, pink, blue, or purple	33
22	(21.) Phyllaries in 2 distinct series, outer phyllaries green, spreading, narrower than inner, inner phyllaries stramineous to brownish, erect	* Coreopsis *
–	Phyllaries in 1–several series, if in 2 series then not strongly dimorphic (in contrast to above)	23
23	(22’.) Leaves opposite, or predominantly basal but with 1–few pairs of opposite cauline leaves	24
–	Leaves alternate or entirely basal, or predominantly basal but with 1–few alternate cauline leaves	25
24	(23.) Leaf venation parallel; stem pubescence glandular; phyllaries in 1 series	*Arnica acaulis* Fig. 160
–	Leaf venation pinnate; stem pubescence eglandular; phyllaries in 2–3 series	* Helianthus *
25	(23’.) Stems and leaves sparsely to densely silky-sericeous	26
–	Stems and leaves glabrous or variously pubescent but not silky-sericeous	27
26	(25.) Leaf blades oblanceolate, spatulate, elliptic, or ovate, not grass-like, glabrate to densely silky-sericeous, not appearing silvery; plants fibrous-rooted or short-rhizomatous	*Chrysopsis mariana* Fig. 161
–	Leaf blades linear to lanceolate, grass-like, usually densely silky-sericeous and appearing silvery; plants long-rhizomatous	Pityopsis graminifolia var. latifolia Fig. 162
27	(25’.) Basal leaves to 52 cm wide, usually deeply lobed, bases cordate, cauline leaves absent or few, reduced	Silphium compositum var. compositum
–	Basal leaves absent or present, < 10 cm wide, entire to deeply lobed, bases various but not cordate, cauline leaves numerous, or few and reduced	28
28	(27’.) Leaf blades decurrent onto stem, stem therefore appearing winged	*Helenium*, in part
–	Leaf blades not decurrent onto stem, stem not appearing winged	29
29	(28’.) Phyllaries in 1–2 series; leaves (usually at least some) pinnately-lobed to pinnatifid	30
–	Phyllaries in 3–5 series; leaves entire or serrate, not pinnately-lobed or pinnatifid	31
30	(29.) Heads 1(–3) per stem; pappus of scales, 1.2–1.5 mm long	*Helenium pinnatifidum*
–	Heads (2–)5–20 per stem; pappus of capillary bristles, 3.5–4.5 mm long	*Packera paupercula*
31	(29’.) Heads 1–4 per stem, broad (generally > 3 cm wide); involucres 15–25 mm wide; pappus of scales only, scales 1.3–2.2 mm long; basal leaves spatulate, thick, succulent	*Balduina uniflora* Fig. 163
–	Heads many (> 4) per stem, small (generally < 3 cm wide); involucres 1.7–10 mm wide; pappus of capillary bristles, with or without scales; basal leaves absent or present, if present not spatulate, thick, and succulent	32
32	(31’.) Heads corymbose; leaves densely glandular; basal leaves absent at anthesis	*Euthamia caroliniana* Fig. 164
–	Heads paniculate, racemose, or in axillary fascilcles; leaves not densely glandular; basal leaves present or absent at anthesis	* Solidago *
33	(21’.) Basal leaves rosette-forming, persistent, thick, somewhat succulent, cauline leaves few, conspicuously reduced	*Erigeron vernus* Fig. 165
–	Basal leaves absent or present, if rosette-forming then withering by anthesis, not thick, not succulent, cauline leaves typically numerous, not conspicuously reduced	34
34	(33’.) Phyllaries keeled; leaves stiff, most perpendicular to stem, 12–40 mm long, 1–3 mm wide	*Ionactis linariifolia* Fig. 166
–	Phyllaries flat or rounded, not keeled; leaves not stiff, 5–120+ mm long, 1–20 mm wide	35
35	(34’.) Heads in panicles	* Symphyotrichum *
–	Heads in flat-topped corymbs	36
36	(35’.) Involucres campanulate to cylindro-campanulate, 6.5–11 mm long; ray florets (5–)8–35, lavender or bluish, often pale, but not white	* Eurybia *
–	Involucres cylindric, 4–6 mm long; ray florets 1–6, white	*Sericocarpus linifolius* Fig. 167

Key adapted from Barkley et al. (2006), Radford et al. (1968), Weakley (2012).

### [Asteraceae] Baccharis L.

**Table:** 

1	Heads in axillary glomerules scattered along branches, most heads sessile (a few pedunculate); pistillate involucres 5–6 mm long, staminate involucres 4–5 mm long	*Baccharis glomeruliflora*
–	Heads in loose pedunculate clusters in broad paniculiform arrays, most heads pedunculate (a few sessile); pistillate involucres 3–5 mm long, staminate involucres 3–5 mm long	*Baccharis halimifolia*‡

Key adapted from Sundberg and Bogler (2006), Weakley (2012).

Note: *Baccharis halimifolia* L., the common *Baccharis* of disturbed areas throughout the North Carolina Coastal Plain, has not been seen by the senior author in savannas or flatwoods within Shaken Creek Preserve, though it has been seen commonly in disturbed areas within the property. The key below distinguishes *Baccharis halimifolia* from the much rarer *Baccharis glomeruliflora*, which was recently re-discovered in NC at Sandy Run by Taggart and Wichmann (2011).

### [Asteraceae] Carphephorus Cass.

**Table:** 

1	Involucres 3.5–6 mm; phyllaries 5–12 in 1–2(–3) series; leaves lacking resin dots	[*Trilisa*]
–	Involucres mostly 7–12(–15) mm; phyllaries 15–40+ in 3–5+ series; leaves with resin dots	2
2	(1’.) Stems, peduncles, phyllaries, and corollas lacking glands; stems (except for peduncles) glabrous or glabrate, pubescence short and appressed; phyllaries broadly elliptic to elliptic-obovate, glabrous (except ciliate margins), lacking glands, apices rounded	*Carphephorus bellidifolius*
–	Stems, peduncles, phyllaries, and corollas gland-dotted; stems (at least lower portion) conspicuously spreading-hirsute; phyllaries ovate-lanceolate to broadly ovate, villous and gland-dotted, apices acute to obtuse	*Carphephorus tomentosus*

Key adapted from Nesom (2006b), Weakley (2012).

Fig. 168

### [Asteraceae] Cirsium Mill.

**Table:** 

1	Heads immediately subtended by several involucre-like, spiny-toothed leaves nearly as long as the involucre proper; flowers white, yellow, or purple	2
–	Heads pedunculate (rarely with 1 or 2 reduced leaves below), not immediately subtended by several involucre-like, spiny-toothed leaves nearly as long as the involucre proper; flowers white, pink, or purple	3
2	(1.) Stems densely tomentose; involucres more-or-less tomentose	Cirsium horridulum var. horridulum
–	Stems glabrous or sparsely tomentose; involucres glabrous	Cirsium horridulum var. vittatum
3	(1’.) Leaves densely white-tomentose below, hairs persistent, obscuring green leaf surface	*Cirsium virginianum*
–	Lower leaf surface densely tomentose only on young leaves, becoming sparsely tomentose to glabrate in age, hairs not persistent, obscuring green leaf surface only on young leaves	4
4	(3’.) Peduncles 5–30 cm long; stems usually unbranched, distal half nearly leafless or only sparsely leafy	*Cirsium lecontei*
–	Peduncles 0–2 cm long; stems usually branched, distal half usually leafy	*Cirsium repandum*

Key adapted from Keil (2006), Weakley (2012).

Fig. 169

### [Asteraceae] Coreopsis L.

**Table:** 

1	At least one leaf per plant with 1–few slender lobes near base (very rarely no leaves with basal lobes); stems with 3–7 nodes below inflorescence; cypsela body oblong; plants flowering early May–early Jul(–later)	*Coreopsis falcata*
–	Leaves lacking basal lobes; stems with 6–30 nodes below inflorescence; cypsela body oblanceolate; plants flowering early Jul–Oct	2
2	(1’.) Basal leaves present at anthesis; cauline leaves abruptly reduced upward; cypselae brown or purple, 2–3 mm long, 0.7–1 mm wide, awns 1.3–1.5 mm long	*Coreopsis linifolia*
–	Basal and lower cauline leaves (at least lower 4 nodes) absent at anthesis; cauline leaves uniformly reduced upward; cypselae black, 3–4 mm long, 1–1.2 mm wide, awns 0.2–1.0 mm long	3
3	(2’.) Leaf blades broadly to narrowly elliptical, 10–45 mm wide, 5–15× as long as wide; cypsela awns 0.7–1.0 mm long	*Coreopsis palustris*
–	Leaf blades linear to linear-oblanceolate, 2–7 mm wide, 20–50× as long as wide; cypsela awns 0.2–0.4 mm long	*Coreopsis* species 1

Key adapted from Radford et al. (1968), Weakley (2012).

References: Strother (2006b).

Fig. 170a, b, c

### [Asteraceae] Erigeron L.

**Table:** 

1	Cauline leaves clasping; ray florets 100–150	*Erigeron quercifolius*‡
–	Cauline leaves sessile, not clasping; ray florets 25–40 or 50–100	2
2	(1’.) Ray florets 50–100, with pappus of short, slender scales < 1 mm long	Erigeron strigosus var. strigosus‡
–	Ray florets 25–40, with pappus of capillary bristles 2.5–3.3 mm long	*Erigeron vernus*

Key adapted from Radford et al. (1968), Nesom (2006c), and Weakley (2012).

Note: *Erigeron vernus* (L.) Torr. & A. Gray is the only species of *Erigeron* seen by the author in savannas or flatwoods at Shaken Creek Preserve. However, *Erigeron quercifolius* Lam. is common on roadsides and in disturbed areas near savannas, and Taggart (2010) reported Erigeron strigosus Muhl. ex Willd. var. strigosus from similar, disturbed areas in Sandy Run. These latter two taxa are included in the key below, where indicated by a double-dagger symbol (‡).

Fig. 165

### [Asteraceae] Eupatorium L.

**Table:** 

1	Leaves pinnatifid or pinnate, leaf segments capillary or linear, 0.2–5 mm wide	2
–	Leaves simple, ≥ 5 mm wide (except *Eupatorium hyssopifolium*, with leaves 2–5 mm wide)	3
2	(1.) Leaves glabrous, sparsely glandular-punctate, basal leaf segments 1–1.5 mm wide, upper leaf segments 0.2–0.5 mm wide; phyllaries glabrate or glabrous, rarely gland-dotted	*Eupatorium capillifolium*
–	Leaves pubescent, densely glandular-punctate, basal leaf segments 2–5 mm wide, upper leaf segments 1–2.5 mm wide; phyllaries usually puberulent, gland-dotted	*Eupatorium compositifolium*
3	(1’.) Leaf bases connate-perfoliate	*Eupatorium perfoliatum*
–	Leaf bases various but not connate-perfoliate	4
4	(3’.) Leaf blades 13–45 mm wide, generally broadest near base, bases broadly cuneate, truncate, or subcordate	5
–	Leaf blades 2–10(–20) mm wide, generally broadest near middle or tip, bases narrowly cuneate	6
5	(4.) Leaf blades elliptic, lanceolate, or lance-ovate, (1.5–)2–2.5× as long as wide, margins often purple; distal leaves and main inflorescence branches often alternate cypselae 3–4 mm long	*Eupatorium pilosum*
–	Leaf blades deltate to suborbiculate, rarely ovate, 1–2× as long as wide, margnis not purple; distal leaves and main inflorescence branches opposite; cypselae 2–3 mm long	*Eupatorium rotundifolium*
6	(4’.) Phyllaries acuminate to attenuate; leaves immediately subtending inflorescence opposite or subopposite	*Eupatorium leucolepis*
–	Phyllaries obtuse to acute; leaves immediately subtending inflorescence alternate (sometimes opposite or whorled in *Eupatorium hyssopifolium*)	7
7	(6’.) Stems arising from crowns or caudices; leaves often whorled, occasionally opposite (sometimes alternate distally), spreading or ascending (not deflexed)	*Eupatorium hyssopifolium*
–	Stems arising from thickened (ca. 1 cm in diam.) horizontal rhizomes; leaves alternate or opposite (not whorled), deflexed, spreading, or ascending	8
8	(7’.) Involucres 5–7 mm long, inner phyllaries (at least some) acute; stems (6–)10–15 dm tall, not usually branching near the base	*Eupatorium mohrii*
–	Involucres 3–4 mm long, all phyllaries rounded apically; stems 3–6(–7) dm tall, often erectly branching from near base	*Eupatorium recurvans*

Key adapted from Siripun and Schilling (2006), Weakley (2012).

Note: *Eupatorium compositifolium* Walter, though not seen at Shaken Creek Preserve or reported from Sandy Run, is a common species of sandy disturbed areas in the Coastal Plain. It is included in the key below, as it may be occur along disturbed margins of savannas and flatwoods.

Fig. 171a, b, c, d, e

### [Asteraceae] Eurybia (Cass.) Cass.

**Table:** 

1	Ray florets (5–)8–14, corollas 5–8(–10) mm long; disc florets 10–20; involucres 6.5–9 mm long, slightly shorter than pappi; phyllaries 24–35; peduncles with 1–2 bracts; cauline leaves 2–12 mm wide; basal leaf blades 8–20 mm wide, petioles > 20 mm long	*Eurybia compacta*
–	Ray florets 15–35, corollas (10–)15–20 mm long; disc florets 25–60; involucres 9–11 mm long, much shorter than pappi; phyllaries 40–65+; peduncles with 2–5 bracts; cauline leaves 2–6 mm wide; basal leaf blades 5–9 mm wide, petioles < 20 mm long	*Eurybia paludosa*

Key adapted from Brouillet (2006).

Fig. 172a, b, c

### [Asteraceae] Euthamia (Nutt.) Cass.

**Table:** 

1	Heads in panicles, racemes, or axillary fascicles; leaves not densely glandular, basal leaves present or absent at anthesis	[*Solidago*]
–	Heads in corymbs; leaves densely glandular, basal leaves absent at anthesis	*Euthamia caroliniana*

Key adapted from Radford et al. (1968), Weakley (2012).

Fig. 164

### [Asteraceae] Helenium L.

**Table:** 

1	Heads 5–70 per plant, borne in paniculiform arrays; basal leaves withered at anthesis, entire or weakly lobed, cauline leaves not reduced upward, strongly decurrent onto stem, decurrency extending the length of the internode, stems therefore appearing conspicuously winged; plants flowering Sep–Oct	*Helenium autumnale*
–	Heads 1(–3) per plant, usually borne singly; basal leaves present at anthesis, usually pinnatifid (rarely dentate, repand, or entire), cauline leaves reduced upward, not or only weakly decurrent onto stem, decurrency extending <0.5 cm below node, not spanning the length of the internode, stems therefore appearing unwinged or only weakly winged; plants flowering Apr–May	*Helenium pinnatifidum*

Key adapted from Bierner (2006).

Fig. 173

### [Asteraceae] Helianthus L.

**Table:** 

1	Leaves cauline, blades narrowly lanceolate to linear, 0.15–0.5(–1) cm wide	*Helianthus angustifolius*
–	Leaves basally disposed (cauline leaves few and abruptly reduced), blades ovate or lanceolate to spatulate, 1.2–4.3 cm wide	*Helianthus heterophyllus*

Key adapted from Schilling (2006).

Fig. 174

### [Asteraceae] Liatris Schreb.

**Table:** 

1	Basal and lower cauline leaves 1-veined; corolla tubes pilose within; stems glabrous or pilose	*Ludwigia pilosa*
–	Basal and lower cauline leaves 3–5-veined; corolla tubes glabrous within; stems glabrous	Liatris spicata var. resinosa

Key adapted from Nesom (2006d), Weakley (2012).

Fig. 175

### [Asteraceae] *Packera* A. & D. Löve

**Table:** 

1	Heads 20–70(–100+); disc corolla tubes 1.5–2 mm long, limbs 1.5–2 mm long; pappus 2.5–3 mm long; cypselae 0.75–1 mm long	*Packera anonyma*‡
–	Heads 2–10+; disc corolla tubes 2–3 mm long, limbs 2–3 mm long; pappus 3.5–4.5 mm long; cypselae 1–2 mm long	*Packera paupercula*

Key adapted from Trock (2006), Weakley (2012).

Note: *Packera anonyma* (Alph. Wood) W.A. Weber & Á. Löve was collected along a roadside in Sandy Run [Haw’s Run] (*Taggart SARU 89*, WNC!) but has not been seen in savannas or flatwoods within Sandy Run or Shaken Creek Preserve. Nonetheless, in order to facilitate distinguishing this taxon and *Packera paupercula* (Michx.) Á. Löve & D. Löve, *Packera anonyma* is included in the key below, where indicated by a doube dagger symbol (‡).

Fig. 176

### [Asteraceae] Pluchea Cass.

**Table:** 

1	Leaves petiolate	*Pluchea camphorata*‡
–	Leaves sessile	2
2	(1’.) Phyllaries and corollas rose-pink to purplish; phyllaries usually arachnose (bearing long, soft, entangled hairs), sometimes also with viscid hairs; involucres 4–6 mm long, 5–9 mm wide	*Pluchea baccharis*
–	Phyllaries and corollas usually creamy white or yellowish, rarely greenish, pink, or purple; phyllaries involucres sparsely arachnose and with sessile glands; involucres 5–10 mm long, 6–9(–12) mm wide	*Pluchea foetida*

Key adapted from Nesom (2006a), Weakley (2012).

Note: *Pluchea camphorata* (L.) DC. has not been seen at Shaken Creek Preserve or reported by Taggart from Sandy Run; however, LeBlond and Weakley (1991) reported the species from Sandy Run [Neck], though the habitat association of the plant was not reported. According to Weakley (2012), *Pluchea camphorata* occurs in “bottomland sloughs, clay flatwoods, [and] other freshwater wetlands”; its presence at Shaken Creek Preserve is possible. As such, it is included in the key below and, because not definitely known from the savannas or flatwoods, indicated by a double-dagger symbol (‡).

Fig. 177

### [Asteraceae] *Solidago* L.

**Table:** 

1	Leaves predominantly cauline, basal and lower cauline leaves as large as or smaller than middle and upper cauline leaves, or basal and lower cauline leaves withering by anthesis	2
–	Leaves basally disposed, basal and lower cauline leaves larger and longer-petiolate than middle and upper cauline leaves, usually persistent	3
2	(1.) Aerial stems arising from elongated, creeping rhizomes, conspicuously spreading-hirsute (at least distally); crushed leaves not anise-scented	*Solidago fistulosa*
–	Aerial stems arising from short, stout caudices, puberulent in lines decurrent from leaf bases (at least distally); crushed leaves typically anise-scented	*Solidago odora*
3	(1’.) Stems puberulent	Solidago puberula var. pulverulenta
–	Stems glabrous	4
4	(3’.) Petiole bases of basal and lower cauline leaves not sheathing stem; disc florets 5–9; involucres 3–4 mm long; pappus 2–3 mm long; cypselae 1 mm long	*Sporobolus pinetorum*
–	Petiole bases of basal and lower cauline leaves sheathing stem; disc florets 8–30; involucres 3.5–5 mm long; pappus 3–4 mm long; cypselae 1.5–2.5 mm long	5
5	(4’.) Leaf margins smooth, entire; ray flowers 8–13 per head; disk flowers 14–25 per head; pappus (2.5–)3.0–3.5 mm long, bristles basally fused and flattened; plants to 1 m tall	*Solidago pulchra*
–	Basal leaf margins scabrous, often toothed; ray flowers 2–7 per head; disk flowers 6–16 per head; pappus 2.2–4.5(–5.0) mm long, bristles not basally fused, filiform; plants to 2 m tall	6
6	(5’.) Margins of upper cauline leaves scabrous (or at least tuberculate); proximal inflorescence branches often elongate; pappus 2.2–4.0 mm long	*Solidago gracillima*
–	Margins of upper cauline leaves generally entire; proximal inflorescence branches not elongate; pappus 4.0–4.5(–5.0) mm long	*Solidago stricta*

Key adapted from Semple and Cook (2006), Weakley (2012).

Fig. 178a, b, c, d, e

### [Asteraceae] Symphyotrichum Nees

**Table:** 

1	Distal cauline leaves reflexed, 5–30 mm long, bases cordate-clasping	*Symphyotrichum walteri*
–	Distal cauline leaves reflexed, spreading, or ascending, (25–)30–120 mm long, bases cordate-clasping or not (if leaves <30 mm long, then bases not cordate-clasping)	2
2	(1’.) Leaves cordate-clasping; pappus 4–6 mm long	Symphyotrichum novi-belgii var. elodes
–	Leaves not cordate-clasping; pappus 3–4 mm long	3
3	(2’.) Apices of phyllaries involute; adaxial leaf surface usually pilose throughout	Symphyotrichum pilosum var. pilosum
–	Apices of phyllaries flat; adaxial leaf surface short-strigose or glabrous (if pilose, then only along the major veins)	4
4	(3’.) Leaves firm, abaxial surface glabrous or short-strigose along midvein; peduncles (0.5–)1–5 cm long, bracts 5–16+; ray flowers 15–33, corollas pale blue, pink, lavender, or white	*Symphyotrichum dumosum*
–	Leaves pliable, abaxial surface usually pilose along midvein; peduncles 0–1 cm long (rarely longer), bracts 1–7; ray flowers 8–15(–23), corollas white (rarely pinkish or purplish)	*Symphyotrichum lateriflorum*

Key adapted from Brouillet et al. (2006).

Fig. 179

### [Asteraceae] Trilisa Cass.

**Table:** 

1	Stems glabrous; heads in corymbiform, flat-topped arrays; peduncles glabrous	*Trilisa odoratissima*
–	Stems densely villoso-hirsute; heads in thyrsiform, often ± columnar arrays; peduncles stipitate-glandular	*Trilisa paniculata*

Key adapted from Nesom (2006b), Weakley (2012).

Fig. 180

### [Asteraceae] Vernonia Schreb.

**Table:** 

1	Mid-cauline leaf blades lance-linear to filiform, 2–4(–8+) mm wide, (8–)12–30(–60+)× as long as wide, margins entire or serrulate; florets 12–20(–30) per head; cypselae 2.5–3 mm long	*Vernonia angustifolia*
–	Mid-cauline leaf blades lanceolate, 15–45(–60+) mm wide, (3.3–)4–6× as long as wide, margins prominently serrate; florets 30–45(–65) per head; cypselae 3.5–4+ mm long	*Vernonia noveboracensis*

Key adapted from Strother (2006a), Weakley (2012).

Fig. 181

### Bignoniaceae

**Table:** 

1	Leaflets 2, margins entire; leaves bearing a terminal, 3-branched tendril	*Bignonia capreolata* Fig. 182
–	Leaflets 7–15, margins serrate; tendrils absent	*Campsis radicans* Fig. 183

Key adapted from Radford et al. (1968), Weakley (2012).

### [Campanulaceae] Lobelia L.

**Table:** 

1	Flowers relatively large, corolla tube 8–14 mm long, fenestrate (with a pair of narrow openings) at base, longest corolla lobe 9–12 mm long	*Lobelia glandulosa*
–	Flowers relatively small, corolla tube 3–4 mm long, not fenestrate at base, longest corolla lobe 4–7 mm long	2
2	(1’.) Lower lip of corolla pubescent inside at base; calyx lobes 3–5 mm long; pedicels and usually ovary pubescent, bracts longer than pedicels; plants flowering Jul–Nov	*Lobelia canbyi*
–	Lower lip of corolla glabrous; calyx lobes 1.5–3 mm long; pedicels and ovary glabrous or pedicels sparsely pubescent, bracts shorter than or rarely equaling pedicels; plants flowering May–Nov	*Lobelia nuttallii*

Key adapted from Radford et al. (1968), Weakley (2012).

Fig. 184

### [Cistaceae] Lechea L.

**Table:** 

1	Outer (slender) sepals equaling or exceeding inner (broad) sepals; stem leaves 6–12 mm long	*Lechea minor*
–	Outer (slender) sepals shorter than inner (broad) sepals; stem leaves 10–25 mm long	Lechea pulchella var. ramosissima

Key adapted from Weakley (2012).

Fig. 185

### [Convolvulaceae] Cuscuta L.

**Table:** 

1	Stylopodium (thickened ridge at base of style) present; corolla lobes obtuse, shorter than corolla tube; capsule 2.5–4 mm broad	*Cuscuta gronovii*
–	Stylopodium absent; corolla lobes acute, nearly equaling to slightly exceeding corolla tube; capsule 1.5–2.5 mm broad	*Cuscuta pentagona*

Key adapted from Weakley (2012).

Fig. 186

### Droseraceae

**Table:** 

1	Carnivory occurring actively via “snap-trap” leaves; leaves bearing numerous, stiff, marginal bristles that interlock when trap closes; inflorescence an umbelliform cyme; stamens 10–20	*Dionaea muscipula* Fig. 126
–	Carnivory occurring passively via “fly-paper” leaves; leaves lacking marginal bristles, beset with copious, red, stipitate-glandular hairs along blade and sometimes petiole; inflorescence a raceme; stamens 5	* Drosera *

Key adapted from Weakley (2012).

### [Droseraceae] Drosera L.

**Table:** 

1	Scape stipitate-glandular, 2–6 cm long; basal rosettes 0.8–3.5 cm wide; stipules absent or obsolete (consisting of a few hair-like segments); petals white, occasionally tinged with pink; seeds black, crateriform (bowl-shaped), minutely reticulate	*Drosera brevifolia*
–	Scape glabrous, 5–15 cm long; basal rosettes (2–)3–12 cm wide; stipules fimbriate; petals white or pink; seeds *either* brown and coarsely 14–16-ridged (*Drosera brevifolia*) *or* reddish brown to black and densely papillose (*Drosera intermedia*)	2
2	(1’.) Petioles with few to numerous long trichomes; plants acaulescent; scape straight at base; petals pink (sometimes fading to white); seeds brown, coarsely 14–16-ridged, not papillose	*Drosera capillaris*
–	Petioles glabrous; plants typically with a leafy stem 1–10 cm long; scape arching at base; petals white; reddish brown to black, not ridged, densely papillose	*Drosera intermedia*

Key adapted from Weakley (2012).

Fig. 187a, b, c

### Ericaceae

**Table:** 

1	Ovary inferior; fruit a berry	2
–	Ovary superior; fruit a capsule	3
2	(1.) Ovary 10-locular; seeds 10, relatively large (slightly crunchy when chewing fruit); abaxial leaf surface glandular, glands yellow or orangish, sessile or stipitate	* Gaylussacia *
–	Ovary 4–5-locular; seeds > 10, tiny (unnoticeable when chewing fruit); abaxial leaf surface eglandular, or glandular and glands red, stipitate	* Vaccinium *
3	(1’.) Leaves coriaceous, evergreen, adaxial surface *either* dark green and shiny *or* dull olive green and lepidote (covered with small, white or yellowish scurfy scales)	4
–	Leaves membraneous or subcoriaceous, deciduous, adaxial surface light to dark green, dull, not lepidote	7
4	(3.) Twigs and leaf surfaces prominently lepidote	*Chamaedaphne calyculata* Fig. 188
–	Twigs and abaxial leaf surfaces glabrous or variously pubescent but not lepidote	5
5	(4’.) Most leaves whorled (some leaves occasionally opposite or alternate)	*Kalmia carolina* Fig. 104
–	Leaves alternate	6
6	(5’.) Leaf margins sharply serrate, blades lacking prominent perimarginal vein	*Leucothoe axillaris* Fig. 189
–	Leaf margins entire, blades with prominent perimarginal vein ca. 1 mm from blade margin	*Lyonia lucida* Fig. 190c, d
7	(3’.) Corolla funnelform, lobes 7–24 mm long; capsule elongate, > 2× as long as broad, 7–24 mm long	* Rhododendron *
–	Corolla urceolate, campanulate, or globose, lobes < 5 mm long; capsule oblate (globose but depressed apically and basally), ovoid, globose or subglobose, nearly as broad as long as broader, 2–6.5 mm long	8
8	(7’.) Leaf margins crenate; corolla campanulate; capsule oblate (round with apical and basal depressions)	*Zenobia pulverulenta* Fig. 191
–	Leaf margins spinulose-serrate, serrulate, or entire; corolla urceolate or globose; capsule ovoid, globose or subglobose	9
9	(8’.) Leaf margins spinulose-serrate; inflorescence of racemes produced along stems of previous year; filaments lacking spurs; capsules not thickened and whitish along sutures; seeds 5–10 per capsule	*Eubotrys racemosa* Fig. 192
–	Leaf margins entire or minutely serrulate; inflorescence of umbellate-racemes produced in fascicles along stems of previous year (*Lyonia mariana*) *or* terminal panicles produced on stems of current year (*Lyonia ligustrina*); filaments bearing 2 short spurs; capsules thickened and whitish along sutures; seeds 100–300+ per capsule	*Lyonia*, in part

Key adapted from Tucker (2009), Weakley (2012).

### Vegetative Key To Common, Erect Ericaceous Subshrubs (i.e., shrubs generally < 0.5 m tall)

**Table:** 

1	Stems green to base; twigs of the season verrucose (with numerous, small, whitish bumps distinct from lenticels), eglandular; abaxial leaf surface stipitate-glandular, glands red	*Vaccinium tenellum*
–	Stems usually brown or reddish at base; twigs of the season not verrucose, stipitate-glandular; abaxial leaf surface *either* stipitate-glandular with glands yellow or orange *or* strigillose with hairs appressed, red basally, usually pale apically	2
2	(1’.) Leaf blades 1.5–4 cm long, 0.6–2.2 cm wide, abaxial surface stipitate-glandular, glands not appressed, yellow or orange	*Gaylussacia dumosa*
–	Leaf blades 3–7 cm long, 1–3.5 cm wide, abaxial surface strigillose, hairs appressed, red basally, usually pale apically	*Lyonia ligustrina*

### [Ericaceae] Gaylussacia Kunth

**Table:** 

1	Plants 1–3(–4) dm tall; leaf blades, pedicels, and sepals stipitate-glandular; petioles 0.5–1.5 mm long, leaf blades 0.3–1 cm wide; inflorescence bracts equaling or longer than pedicels, persistent	*Gaylussacia dumosa*
–	Plants 7.5–20 dm tall; leaf blades, pedicels, and sepals glandular-punctate; petioles 2–3 mm long, leaf blades 2–3 cm wide; inflorescence bracts shorter than pedicels, caducous	*Gaylussacia frondosa*

Key adapted from Sorrie et al. (2009b).

Fig. 193

### [Ericaceae] Lyonia Nutt.

**Table:** 

1	Leaves evergreen, coriaceous, shiny, with a prominent perimarginal vein	*Lyonia lucida*
–	Leaves deciduous, subcoriaceous, dull, lacking a prominent perimarginal vein	2
2	(1’.) Leaf margins serrulate; inflorescence a terminal panicle developing on stems of current year; corolla 3–5 mm long; capsule 2.5–3 mm long	*Lyonia ligustrina*
–	Leaf margins entire; inflorescence of umbellate-racemes developing in fascicles along stems of previous year; corolla 7–14 mm long; capsule 4–6 mm long	*Lyonia mariana*

Key adapted from Judd (2009), Weakley (2012).

Fig. 190

### [Ericaceae] Rhododendron L.

**Table:** 

1	Shrub 0.5–1 m tall; pedicels 4–15(–20) mm long; flowers opening before or during emergence and expansion of leaves; sepals 1.5–5 mm long	*Rhododendron atlanticum*
–	Shrub or small tree to 7 m tall; pedicels 5–27 mm long; flowers opening after emergence and expansion of leaves; sepals 0.1–1 mm long	*Rhododendron viscosum*

Key adapted from Judd and Kron (2009).

Fig. 194

### [Ericaceae] Vaccinium L.

**Table:** 

1	Stems trailing; leaves evergreen; [sect. *Herptothamnus*]	*Vaccinium crassifolium*
–	Stems erect; leaves deciduous (evergreen to tardily deciduous in *Vaccinium arboreum*)	2
2	(1’.) Twigs of the season verrucose (with numerous, small, whitish bumps distinct from lenticels); inflorescences lacking leaf-like bracts; [sect. *Cyanococcus*]	3
–	Twigs of the season not verrucose; inflorescences with leaf-like bracts	6
3	(2.) Plants colonial, 1–7.5 dm tall; abaxial leaf surface stipitate-glandular	*Vaccinium tenellum*
–	Plants not colonial, 10–50 dm tall; abaxial leaf surface eglandular	4
4	(3’.) Leaf blades 0.7–3.5 cm long, 0.3–1.5 cm wide, margins serrulate; twigs slender, numerous; berry black	*Vaccinium elliottii*‡
–	Leaf blades 3–10 cm long, 1.5–4.5 cm wide, margins entire, ciliate, or serrulate margins; twigs stouter, fewer; berries blue or black	5
5	(4’.) Young twigs glabrous; leaf surfaces glabrous, margins eciliate; corollas 8–12 mm long; berries blue	*Vaccinium formosum*
–	Young twigs puberulent; leaf surfaces pubescent, margins ciliate; corollas 5–8 mm long; berries black	*Vaccinium fuscatum*
6	(2’.) Leaves usually lustrous, blades obovate to oblong, 2.2–4 cm long, 1.2–2 cm wide, abaxial surface stipitate-glandular; corolla broadly urceolate to narrowly campanulate, stamens included; berry black, lustrous, 7–9 mm in diam.; [sect. *Batodendron*]	*Vaccinium arboreum*
–	Leaves dull, blades elliptic, 2–8 cm long, 0.9–3.2 cm wide, abaxial surface eglandular; pedicels continuous with calyx tubes; corolla campanulate, stamens long-exserted; berry variously colored, often glaucous, 7–18 mm in diam.; [sect. *Polycodium*]	*Vaccinium stamineum*

Key adapted from Vander Kloet (2009), Weakley (2012).

Note: Not seen in Shaken Creek Preserve or reported from Sandy Run, *Vaccinium elliottii* Chapm. was extensively collected on roadsides and “woodland edges” on Old Maple Hill Road (*Wilbur 55249*, *55251*, *63754*, *63758*, *63763*, *63765*; DUKE!). Though generally a species of bottomlands, sandy slopes, and terraces, its presence along sandy stream margins of savannas and flatwoods cannot be ruled out. It is therefore included in the key below, where indicated by a double-dagger symbol (‡).

Figs 195, 196

### Euphorbiaceae

**Table:** 

1	Plant with stinging trichomes; leaves palmately or ternately lobed or divided, margins serrate (very rarely entire); calyx petaloid, white	*Cnidoscolus stimulosus* Fig. 197
–	Plant with or without stinging trichomes; leaves not lobed or divided, margins serrate, undulate, or entire; calyx petaloid, greenish or purplish, or absent and flowers borne in cyathia (flower-like involucre often with petaloid appendages; staminate flowers consisting of a single stamen, pistillate flowers of a single pistil)	2
2	(1’.) Plant lacking stinging trichomes; stems with copious white latex, spreading or erect; leaves usually opposite, rarely alternate, margins entire; flowers borne in cyathia; capsule glabrous	*Euphorbia ipecacuanhae* Fig. 127
–	Plants with stinging trichomes; stems without white latex, erect; leaves alternate, margins irregularly serrate, undulate, or entire; flowers not borne in cyathia; capsule strigillose	*Tragia urens* Fig. 198

Key adapted from Weakley (2012).

### Fabaceae

**Table:** 

1	Plants woody, < 1.5 m tall; leaves glandular-punctate	* Amorpha *
–	Plants herbaceous or suffruticose, heights various; leaves not glandular-punctate	2
2	(1’.) Leaves unifoliolate, appearing simple; stipules of mid- and upper cauline leaves conspicuous, decurrent, inversely-sagittate	*Crotalaria purshii* Fig. 199
–	Leaves (at least most on each plant) obviously compound, with ≥ 3 leaflets; stipules not as above	3
3	(2’.) Leaflets ≤ 4	4
–	Leaflets ≥ 5	12
4	(3.) Leaves palmately compound, leaflets (1–3)4	*Zornia bracteata* Fig. 200
–	Leaves pinnately compound, leaflets 3	5
5	(4’.) Stamens distinct; petals yellow; legume inflated; plants often drying black	* Baptisia *
–	Stamens monadelphous or diadelphous; petals yellow, white, red, pink, blue, or purple; legume flattened or somewhat inflated; plants drying green to brown, not distinctly black	6
6	(5’.) Standard petal ≥ 2 cm long, ca. 2× as long as other petals; petals light blue to lavender; plant twining; legume linear, 7–14 cm long	*Centrosema virginianum* Fig. 201
–	Standard petal ≤ 2 cm long, ≤ 1.5× as long as other petals; petals white, yellow, pink, red, purple, or blue; plant twining or not; legume various, 0.3–5 cm long	7
7	(6’.) Plant trailing or climbing by twining	8
–	Plant erect, not climbing	10
8	(7.) Corollas 4–6 mm long; fruit indehiscent, 2–3-seeded, transversely partitioned into 1-seeded segments, densely uncinulate (with minute hairs hooked at tips), attaching readily to clothes, hair, etc.	*Desmodium lineatum*
–	Corolas 10–20 mm long; fruit dehiscent, 5–many-seeded, appressed-pubescent (hairs not hooked at tip), not attaching readily to clothes, hair, etc.	9
9	(8’.) Flowers borne racemosely, 1–3 per node, pedicels 1–5 mm long; keel petals neither beaked nor strongly curved; style not bearded along upper surface	*Galactia regularis* Fig. 202
–	Flowers borne in capitate clusters, usually > 3 per cluster, pedicels 0–1 mm long; keel petals beaked and strongly curved; style bearded along upper surface	*Strophostyles umbellata* Fig. 203
10	(7’.) Stipules connate and sheathing stem, partially adnate to petiole; petals bright yellow	*Stylosanthes biflora* Fig. 204
–	Stipules free, neither sheathing stem not adnate to petiole; petals white, pink, red, purple, or blue	11
11	(10’.) Stipels present, persistent; pair of bractlets subtending calyx absent; fruit 2–several-seeded, transversely partitioned into 1-seeded segments, densely uncinulate (with minute hairs hooked at tip)	*Desmodium*, in part
–	Stipels absent; leaflets orbicular to linear, (0.75–)1–12× as long as wide; pairs of bractlest subtending calyx present; fruit 1-seeded, unsegmented, short-puberulent (hairs not hooked at tip)	* Lespedeza *
12	(3’.) Leaves paripinnate (with even number of leaflets); petals bright yellow	* Chamaecrista *
–	Leaves imparipinnate (with odd number of leaflets); petals white, pink, red, purplish, or yellow-brown, not bright yellow	13
13	(12’.) Plants climbing by twining; leaflets (3)5 or 7	*Apios americana* Fig. 205
–	Plants erect or prostrate, not climbing; leaflets 7–23	14
14	(13’.) Leaflets minutely strigillose, trichomes 2-branched and attached at middle (Y-shaped); corollas pink to yellowish-brown, ≤ 6 mm long; legume 5–10 mm long	*Indigofera caroliniana*
–	Leaflets short-pubescent or pilose, trichomes simple, unbranched, attached at base; petals initially white, turning pink, drying purple, 10–17 mm long; legume 30–50 mm long	* Tephrosia *

Key adapted from Radford et al. (1968), Weakley (2012).

References: Wilbur (1963).

### [Fabaceae] Amorpha L.

**Table:** 

1	Plant glabrous or sparsely pubescent; leaflet mucros tapered apically; legume glabrous	*Amorpha georgiana*
–	Plant densely pubescent; leaflet mucros swollen apically; legume short-pubescent (rarely glabrate)	*Amorpha herbacea*

Key adapted from Radford et al. (1968), Weakley (2012).

Fig. 206

### [Fabaceae] Baptisia Vent.

**Table:** 

1	Plant appressed-pubescent; stipules of mid- and lower-cauline leaves 1–2(–4) cm long, persistent or tardily deciduous, leaflets 3–7(–9) cm long, petiolules ≥ 2 mm long; corolla 20–25 mm long; racemes 1(–3)	*Baptisia cinerea*
–	Plant glabrous or sparsely pubescent; stipules minute, caducous; leaflets 0.6–2(–4) cm long, petiolules 0–1 mm long; corolla 9–16 mm long; racemes numerous	*Baptisia tinctoria*

Key adapted from Radford et al. (1968), Weakley (2012).

Fig. 207

### [Fabaceae] Chamaecrista Moench

**Table:** 

1	Petiolar glands borne near middle of petiole, sessile; inflorescence a 1–6-flowered fascicle; pedicels 10–20 mm long; corolla 2.5–3.5 cm in diam., larger petals 15–20 mm long; functional stamens 10	Cassia fasciculata var. fasciculata
–	Petiolar glands borne near apex of petiole (immediately below lowest pair of leaflets), short-stipitate; inflorescence solitary, or 2–3-flowered and borne in short raceme; pedicels 1–4 mm long; corolla 0.8–1.0 cm in diam., larger petals 4–7(–8) mm long; functional stamens 5	Cassia nictitans var. nictitans

Key adapted from Radford et al. (1968), Weakley (2012).

Fig. 208

### [Fabaceae] Desmodium Desv.

**Table:** 

1	Stems trailing; terminal leaflets 0.9–1.5× as long as wide	*Desmodium lineatum*
–	Stems erect; terminal leaflets (2.5–)3–12(–15)× as long as wide	2
2	(1’.) Leaflets narrowly linear, terminal leaflets < 10 mm wide, (4–)8–12(–15)× as long as wide; petioles of mid-cauline leaves 1–10(–15) mm long	*Desmodium tenuifolium*
–	Leaflets broader, terminal leaflets *either* > 15 mm wide *or* < 4× as long as wide; petioles of mid-cauline leaves usually > 15 mm long	3
3	(2’.) Petals 3–5 mm long; fruits with 1–2(–3) segments; stipes 1–2 mm long, usually shorter than calyx tube; stems uncinulate-puberulent (with short, hooked hairs) and also usually pilose (with long, straight hairs)	*Desmodium ciliare*
–	Petals 6–8 mm long; fruits with 3–6 segments; stipes 2–3.5 mm long, longer than calyx tube; stems glabrous or uncinulate-puberulent, very rarely sparsely pilose	*Desmodium paniculatum*

Key adapted from Radford et al. (1968), Weakley (2012).

Fig. 209

### [Fabaceae] Lespedeza Michx.

**Table:** 

1	Leaf blades oblanceolate, distinctly widest at apex, base and apex dissimilar (base cuneate, apex rounded, truncate, or retuse); racemes much shorter than subtending leaf; calyx lobes < 3.5 mm long	*Lespedeza cuneata‡
–	Leaf blades various but not oblanceolate, widest at middle, base and apex similar (e.g., both rounded, both cuneate, etc.); racemes nearly equaling to much exceeding subtending leaf; calyx lobes > 3.5 mm long	2
2	(1’.) Peduncles shorter than subtending leaf, inflorescence therefore hardly exceeding subtending leaf; leaflets (2–)2.5–5(–8)× as long as wide; calyx lobes 6–10 mm long	*Lespedeza capitata*
–	Peduncles longer than subtending leaf, inflorescence therefore greatly exceeding subtending leaf; leaflets *either* 1.3–1.8× as long as wide *or* 4–8(–10)× as long as wide; calyx lobes 3–7 mm long	3
3	(2’.) Leaflets narrowly oblong-elliptic to linear, 4–8(–10)× as long as wide	*Lespedeza angustifolia*
–	Leaflets widely-oblong to orbicular, 1.3–1.8× as long as wide	Lespedeza hirta var. curtissii

Key adapted from Radford et al. (1968), Weakley (2012).

Note: The invasive *Lespedeza cuneata* (Dum. Cours.) G. Don. has not been found in any well-managed savannas or flatwoods on site; however, it sometimes occurs along roadsides adjacent to such areas and is a frequent component of food plots and other disturbed areas throughout the property. To facilitate the distinguishing of *Lespedeza cuneata* from its congeners on site, *Lespedeza cuneata* is included in the key below, where indicated by a double-dagger symbol (‡).

Fig. 210

### [Fabaceae] Tephrosia Pers.

**Table:** 

1	Petiole 1–4× as long as basalmost leaflet of each leaf, leaflets 10–50 mm long; peduncle and inflorescence rachis strongly flattened, prominently 2(–3)-angled	*Tephrosia florida*
–	Petiole 1/3–1× as long as basalmost leaflet of each leaf, leaflets 7–27(–37) mm long; peduncle and inflorescence rachis terete or inconpicuously 2–4-angled	2
2	(1’.) Plants inconpicuously pubescent, hairs gray, appressed or spreading, relatively short; leaflets 2–7 mm wide (5–6 mm wide avg.), apices mostly acute; inflorescence with 1–3(–5) nodes	*Tephrosia hispidula*
–	Plants conspicuously pilose, hairs rusty-brown, spreading, relatively long; leaflets 6–14 mm wide (8 mm wide avg.), apices mostly obtuse; inflorescence with 2–20 nodes	*Tephrosia spicata*

Key adapted from Radford et al. (1968), Weakley (2012).

Fig. 211

### [Fagaceae] Quercus L.

**Table:** 

1	Leaf blades broadest at apex	2
–	Leaf blades broadest at middle or base	5
2	(1.) Leaf blades 5–9-lobed, lobes lateral, awns 15–50 per leaf	*Quercus velutina*, in part
–	Leaf blades unlobed or 1–3-lobed, lobes apical, awns 1–20 per leaf	3
3	(2’.) Twigs glabrous; leaf blades 1.5–6(–7) cm wide, abaxial surface glabrous (excluding tufts of tomentum in vein axils); trees of wet habitats	*Quercus nigra*
–	Twigs pubescent; leaf blades (4–)7–20 cm wide, abaxial surface pubescent; trees of dry habitats	4
4	(2’.) Petiole (14–)20–50 mm long, glabrous to sparsely pubescent; abaxial leaf surface densely pubescent, hairs stellate (though stellate structure is difficult to detect at 10× magnification); trees to 30 m tall; bark narrowly fissured	*Quercus falcata*, in part
–	Petioles 5–15(–20) mm long, sparsely to densely pubescent; abaxial leaf surface sparsely to densely pubescent, pubescence consisting of a mixture of glandlike and stellate hairs (whose structure is easily visible at 10× magnification); trees to 15 m tall; bark blocky	Quercus marilandica var. marilandica
5	(1’.) Leaf blades unlobed	6
–	Leaf blades lobed	7
6	(5.) Abaxial leaf surface densely tomentose, appearing nearly white; trees to 10 m tall, of dry habitats	*Quercus incana*
–	Abaxial leaf surface glabrous or glabrescent, appearing light green; trees to 40 m tall, of wet habitats	*Quercus laurifolia*
7	(5’.) Leaf lobes lacking bristle tips, often divergent at right angles and creating a cruciform shape; acorns developing in one growing season, germinating in fall	8
–	Leaf lobes with bristle tips (these sometimes deciduous), not divergent at right angles and forming a cruciform pattern; acorns developing in two growing seasons, germinating in spring	9
8	(7.) Woody twigs of the season glabrous or glabrescent, hairs scattered, deciduous, 2-forked; petioles of mature leaves 3–10(–15) mm long; leaf blades (2.5–)4–8(–13.5) cm long, 3–5(–7)-lobed, overall form only occasionally cruciform	*Quercus margaretta*
–	Woody twigs of the season pubescent (especially apically), hairs dense, persistent, stellate; petioles of mature leaves 15–20 mm long; leaf blades (5–)7.5–15(–20) cm long, usually 5-lobed, overall form typically cruciform	*Quercus stellata*
9	(7’.) Twigs densely pubescent; leaves with abaxial surfaces densely and persistently tomentose, primary lobes 3–7, awns 6–20, bases rounded; sun leaves with terminal lobe typically elongated, often falcate, shade leaves shallowly 3-lobed near broad apex (resembling leaves of Quercus marilandica var. marilandica); acorn cups 3–7 mm long	*Quercus falcata*, in part
–	Twigs glabrous of sparsely pubescent; leaves with abaxial surfaces glabrous or densely tomentose and hairs deciduous, primary lobes 5–9, awns 15–50, bases obtuse to truncate, not rounded; sun and shade leaves not as above; acorn cups 7–14 mm long	10
10	(9’.) Terminal buds reddish-brown basally, usually silvery apically, not or only weakly angled, 3–5(–7) mm long; leaf blades glabrous; acorn cups reddish, glossy, often thickened; nut typically with 1–many concentric grooves apically	*Quercus coccinea*
–	Terminal buds grayish throughout, strongly 4-angled, 7–10 mm long; leaf blades densely tomentose abaxially when young, becoming glabrous with age; acorn cups yellowish or brownish, dull, not thickened; nut lacking concentric grooves apically	*Quercus velutina*, in part

Key adapted from Jensen (1997), Weakley (2012).

Figs 212, 213a, b, c

### Gentianaceae

**Table:** 

1	Leaves reduced, scale-like, 1–3 mm long, appressed to stem	* Bartonia *
–	Leaves not reduced, not scale-like, ≥ 15 mm long, spreading to ascending	2
2	(1’.) Corolla blue to violet or whitish, lobes 4–5, shorter than corolla tube	* Gentiana *
–	Corolla pink or white, lobes 5–14, much longer than corolla tube	* Sabatia *

Key adapted from Radford et al. (1968), Weakley (2012).

### [Gentianaceae] Bartonia Muhl. Ex Willd.

**Table:** 

1	Corolla lobes white, spatulate to obovate, spreading, 4–9 mm long; flowering (Nov–)Feb–Apr(–Jun)	*Bartonia verna*
–	Corolla lobes green to creamy white, oblong to ovate or lance-ovate, ascending or erect, 2–3(–5.2) mm long; flowering Jul–Oct	*Bartonia virginica*

Key adapted from Radford et al. (1968), Weakley (2012).

Fig. 214a, b, c

### [Gentianaceae] Gentiana L.

**Table:** 

1	Flowers solitary (rarely 2 or 3); corolla spotted within; leaves twisted, oblanceolate to oblinear	*Gentiana autumnalis*
–	Flowers clustered; corolla not spotted within; leaves planar, ovate, elliptic, or lanceolate (rarely linear)	2
2	(1’.) Leaves bright green, ovate, widest near base; calyx lobes longer than tube; corolla campanulate, lobes spreading, usually 2–4 mm longer than corolla appendages (pleat-like tissue between corolla lobes)	*Gentiana catesbaei*
–	Leaves dark green, linear to elliptic, widest near middle; calyx lobes shorter than or nearly equal to calyx tube; corolla cylindric-oblanceolate, lobes usually incurved, rarely exceeding appendages by > 2 mm	*Gentiana saponaria*

Key adapted from Radford et al. (1968), Weakley (2012).

Fig. 215a, b, c, d, e

### [Gentianaceae] Sabatia Adans.

**Table:** 

1	Calyx lobes 10; corolla lobes (7–)8–12(–14), pink; pedicels < 5 mm long	*Sabatia gentianoides*
–	Calyx lobes 5; corolla lobes 5–6(–7), pink or white; longer pedicels > 5 mm long or if shorter, then corolla white	2
2	(1’.) Upper branches of main stem alternate; plant perennial with short rhizomes; basal leaves absent at anthesis, cauline leaves 1–5 mm wide; calyx lobes 7–20 mm long; corolla pink	*Sabatia campanulata*
–	Upper branches of main stem opposite; plant annual and lacking rhizomes (*Sabatia brachiata*, *Sabatia angularis*) or perennial with short rhizomes (*Sabatia difformis*); basal leaves present or absent at anthesis, cauline leaves 2–40 mm wide; calyx lobes 2–15 mm long; corolla pink or white	3
3	(2’.). Plants perennial, rhizomatous; corolla white; pedicels 1–2(–5) mm long	*Sabatia difformis*
–	Plants annual, not rhizomatous; corolla pink (rarely white); longer pedicels > 5 mm long	4
4	(3’.) Stems winged (at least basally); leaves ovate, clasping, < 2× as long as wide	*Sabatia angularis*
–	Stems not winged; leaves elliptic to lanceolate, more or less tapered to the base, mostly > 3× as long as wide	*Sabatia brachiata*

Key adapted from Radford et al. (1968), Weakley (2012).

Figs 216, 217a, b, c

### [Haloragaceae] Proserpinaca L.

**Table:** 

1	Emersed (exposed) leaves serrate, submersed leaves pectinate with 8–14 pairs of divisions, divisions 5–30 mm long; fruits 2.3–6.0 mm wide	Persea palustris var. palustris‡
–	Emersed leaves pinnatifid to pectinate, submersed leaves pectinate with 4–12 pairs of divisions 2–7.5 mm long; fruits 2.0–3.6 mm wide	*Proserpinaca pectinata*

Key adapted from Radford et al. (1968), Weakley (2012).

Note: Proserpinaca palustris L. var. palustris was reported from swamps and anthopogenic wetlands in Sandy Run (Taggart 2010; Sandy Run [Patterson]: *Taggart SARU 588* (WNC!)). Though not usually occuring in savannas or flatwoods, this taxon is included in the key below to distinguish it from *Proserpinaca pectinata* Lam., with which *Persea palustris* may occur in wet, disturbed areas (e.g., borrow pits and ditches) near or within savannas and flatwoods.

Fig. 218

### [Hypericaceae] Hypericum L.

**Table:** 

1	Petals pink; stamens in 3 fascicles of 3 stamens each; hypogynous glands present between fascicles of stamens	*Hypericum virginicum*
–	Petals yellow; stamens distinct or fascicled, if fascicled then not in 3 fascicles of 3 stamens each; hypogynous glands absent	2
2	(1’.) Plant a shrub; leaves with an articulation (a narrow line, groove, or abrupt change of color and texture) at petiole base, petiole therefore appearing jointed at junction with stem	3
–	Plant an herb (suffrutescent in *Hypericum cistifolium* and infrequently in *Hypericum gentianoides*); leaves lacking an articulation at base, petiole therefore not appearing jointed at junction with stem but merging gradually into stem with no break, groove, or abrupt change in color or texture	10
3	(2.) Leaves acicular to narrowly linear, 0.5–1.5(–2) mm wide, margins generally parallel	4
–	Leaves lanceolate, elliptic, or oblanceolate, largest leaves > 2 mm wide, margins not parallel	7
4	(3.) Longest leaves 5–16 mm long; flowers 13–15 mm in diam.	5
–	Longest leaves 13–30 mm long; flowers 10–26 mm in diam.	6
5	(4.) Primary branch internodes with 2 ridged or winged angles extending basally from midribs (but not margins) of paired leaves; leaf surfaces glossy; capsules 3–6 mm long; seeds reddish-amber or brown, alveoli not in distinct longitudinal rows	*Hypericum brachyphyllum*
–	Primary branch internodes with 6 ridged or winged angles extending basally from midribs and margins of paired leaves; leaf surfaces dull; capsules 6–9 mm long; seeds dark red to black, alveoli in distinct longitudinal rows	*Hypericum tenuifolium*
6	(4’.) Plant erect, 5–20 dm tall, not forming dense clumps; flowers 13–26 mm in diam.	*Hypericum galioides*, in part
–	Plant more-or-less decumbent, < 5 dm tall, forming dense clumps; flowers 10–12 mm in diam.	*Hypericum lloydii*
7	(3’.) Petals 4; sepals 4 (rarely 2 in *Hypericum crux-andreae*); plant 3–10 dm tall; leaves 8–40 mm long	8
–	Petals 5; sepals 5; plant 2–20 dm tall; leaves (10–)20–70 mm long	9
8	(7.) Leaves (5–)7–20 mm wide, base rounded or subcordate; styles and carpels 3 (rarely 4)	*Hypericum crux-andreae*
–	Leaves 1–7 mm wide, base cuneate; styles and carpels 2	*Hypericum hypericoides*
9	(7’.) Leaves (1.8–)2.8–8.3(–11) mm wide, mostly 2.5–5× as long as wide, largest leaves always > 4 mm wide, usually > 30 mm long; seeds 0.9–1.6 mm long	Hypericum densiflorum var. densiflorum
–	Leaves 1.5–5(–7) mm wide, mostly 5–10× as long as wide, largest leaves usually < 5 mm wide, usually < 30 mm long; seeds 0.6–0.8 mm long	*Hypericum galioides*, in part
10	(2’.) Leaves appressed, reduced, scale-like, < 1 mm wide; inflorescence racemose	*Hypericum gentianoides*
–	Leaves spreading or ascending, not reduced, not scale-like, > 1 mm wide; inflorescence cymose	11
11	(10’.) Plants suffruticose (somewhat woody basally); axillary fascicles of leaves present	*Hypericum cistifolium*
–	Plants herbaceous throughout; axillary fascicles of leaves absent	12
12	(11’.) Stems and leaves densely pubescent; leaves strongly ascending to nearly appressed	*Hypericum setosum*
–	Stems and leaves glabrous; leaves spreading (strongly ascending or sometimes nearly appressed in *Hypericum gymnanthum*)	13
13	(12’.) Stamens 50–80; styles 2–4 mm long	*Hypericum denticulatum*
–	Stamens 5–22; styles 0.5–1.5 mm long	14
14	(13’.) Leaves lanceolate to linear, 6–30 mm long, 0.5–3 mm wide, base attenuate to cuneate, 1–3-nerved	*Hypericum canadense*
–	Leaves ovate to elliptic, 3–35 mm long, 2–15 mm wide, base rounded to cordate-clasping, 3–7-nerved	15
15	(14’.) Plants strict or sparingly branched; inflorescence few-flowered, with few or no leaf-like bracts; sepals lanceolate, 1.5–4.5 mm long	*Hypericum gymnanthum*
–	Plants usually diffusely branched; inflorescence many-flowered, with numerous leaf-like bracts; sepals linear or narrowly elliptic, 1.5–3 mm long	Hypericum mutilum var. mutilum

Key adapted from Radford et al. (1968), Weakley (2012).

Figs 219, 220, 221a, b, c

### Lamiaceae

**Table:** 

1	Flowers in sessile, cymose clusters in leaf axils; anther-bearing stamens 2	* Lycopus *
–	Flowers in racemes or if in cymose clusters, then not sessile in leaf axils; anther-bearing stamens 4	2
2	(1’.) Inflorescence an open raceme	3
–	Inflorescence a compact, cymose, often head-like cluster	4
3	(2.) Stems glabrous; lower cauline leaves elliptic, lanceolate, oblanceolate; calyx lacking distinctive crest on upper surface	*Physostegia purpurea* Fig. 222
–	Stems canescent or pilose; lower cauline leaves deltate-ovate; calyx with distinctive crest on upper surface	*Scutellaria integrifolia* Fig. 223
4	(2’.) Inflorescences axillary, heads borne on leafless peduncles; leaf blades lanceolate to lanceolate-rhombic, 5–15 cm long, 1.6–6 cm wide	*Hyptis alata*
–	Inflorescence corymbose, heads terminating leafy branches; leaf blades elliptic to elliptic-lanceolate, 1.5–5 cm long, 0.3–3 cm wide	* Pycnanthemum *

Key adapted from Radford et al. (1968).

### [Lamiaceae] Lycopus L.

**Table:** 

1	Leaves evidently petiolate, petioles narrowly winged, bases narrowly cuneate, not clasping	*Lycopus rubellus*
–	Leaves sessile, bases narrowly or broadly cuneate to round, often clasping	2
2	(1’.) Leaf blades ovate to lanceolate, bases usually rounded, upper leaves scarcely narrower than lower leaves; calyx lobes 1–2× as long as tube	*Lycopus amplectens*
–	Leaf blades lanceolate to linear, bases cuneate, upper leaves conspicuously narrower (and often also shorter) than lower leaves; calyx lobes ≥ 2× as long as tube	*Lycopus angustifolius*+

Key adapted from Radford et al. (1968), Weakley (2012).

Note: *Lycopus angustifolius* Elliott (SR-P; S1, G4?Q) has not been reported from or collected in Shaken Creek Preserve or the vicinity; however, the species could be found in open, wet areas on site, as around the margins of borrow ponds or ditches. Moreover, one specimen collected from Sandy Run and reported as *Lycopus rubellus* (*Taggart SARU 498*, WNC) appears to the senior author, based on the nearly sessile leaves, to be close to (and may actually represent) *Lycopus angustifolius*. For these reasons (and as a safety precaution!), *Lycopus angustifolius* is included in the key below, where indicated by a plus (+) symbol.

Fig. 224

### [Lamiaceae] Physostegia Benth.

**Table:** 

1	Most or all larger leaves with margins bluntly serrate or entire, bases clasping or not	*Physostegia purpurea*
–	Most or all larger leaves with margins sharply serrate, bases not clasping	Physostegia virginiana ssp. praemorsa+

Key adapted from Weakley (2012).

Note: Physostegia virginiana (L.) Benth. ssp. praemorsa (Shinners) P.D. Cantino was reported from Sandy Run by Taggart (2012). However, the voucher for this taxon (*Taggart SARU 238*, WNC!) appears to the senior author, based on the bluntly serrate leaf margins, to be *Physostegia purpurea* (Walter) S.F. Blake. Though not otherwise reported or collected in Shaken Creek Preserve or the vicinity, Physostegia virginiana ssp. praemorsa may occur in the area and, in order to facilitate its distinction from *Physostegia purpurea*, is maintained in the key below, where indicated by a plus (+) symbol.

Fig. 222

### [Lamiaceae] Pycnanthemum Michx.

**Table:** 

1	Calyx lobes 2.3–3.3(–5) mm long; leaves 3–15 mm wide	*Pycnanthemum flexuosum*
–	Calyx lobes 1.7–2 mm long; leaves 10–30 mm wide	*Pycnanthemum setosum*

Key adapted from Radford et al. (1968), Weakley (2012).

Fig. 225

### Lauraceae

**Table:** 

1	Leaves evergreen, unlobed, often with numerous deforming galls along margins; inflorescence cymose; flowers bisexual; drupe dark blue to black, ca. 8 mm in diam.	*Persea palustris* Fig. 123
–	Leaves deciduous, 0–2(–5) lobed, generally lacking deforming galls along margins; inflorescence racemose or paniculate; flowers unisexual; drupe blue, ca. 1 cm in diam.	*Sassafras albidum* Fig. 114

Key adapted from van der Werff (1993).

### Lentibulariaceae

**Table:** 

1	Leaves borne in basal rosettes, ovate or elliptic; carnivory occurring passively via viscid adaxial leaf surfaces; flowers purple, blue, or white, solitary on bractless peduncles	* Pinguicula *
–	Leaves or leaf segments borne along subterranean or submersed stems, linear; carnivory occurring actively via bladder-like traps; flowers yellow or purple, in (1–)many-flowered racemes, each pedicel subtended by a minute bract	* Utricularia *

Key adapted from Weakley (2012).

### [Lentibulariaceae] Pinguicula L.

**Table:** 

1	Corolla purple, > 2 cm long (including spur), > 1.8 cm wide; palate exserted from throat of corolla; rosettes usually 5–10(–15) cm in diam.; seeds (0.4–)0.5–0.8 mm long	*Pinguicula caerulea*
–	Corolla white to pale lavender, < 2 cm long (including spur), < 1.5 cm wide, palate included within throat of corolla; rosettes usually 2–4 cm in diam.; seeds 0.4 mm long	*Pinguicula pumila*

Key adapted from Radford et al. (1968), Weakley (2012).

Fig. 226

### [Lentibulariaceae] Utricularia L.

**Table:** 

1	Plant terrestrial, principal branch system within soil; bladders 0.2–1.1 mm long, mostly < 1.0 mm long; corolla yellow; seeds 0.2–0.25 mm long	2
–	Plants aquatic, floating unattached in water (sometimes deposited on land, but then principal branch system lying on, not within, soil); bladders 0.7–5.0 mm long, mostly > 1.0 mm long; corolla purple or yellow; seeds 0.5–2.0 mm long	3
2	(1.) Bract subtending pedicel not peltate, associated with 2 bracteoles, bracteoles 1–2.5 mm long; corolla spur oriented downward or backward, at approximately a right angle to the lower lip	*Utricularia juncea*
–	Bract subtending pedicel peltate (attached near middle), not associated with 2 bracteoles; corolla spur oriented forward, essentially appressed to lower lip	*Utricularia subulata*
3	(1’.) Flowers yellow; leaves divided into alternate segments with lateral traps	*Utricularia biflora*‡
–	Flowers purple; leaves divided into verticillate segments with terminal traps	*Utricularia purpurea*‡

Key adapted from Weakley (2012).

Note: The following, strictly aquatic species of *Utricularia* have been collected in Shaken Creek Preserve or the vicinity: *Utricularia biflora* Lam. (Sandy Run [Haw’s Run]: *Taggart SARU 326*, WNC!) and *Utricularia purpurea* Walter (Shaken Creek Preserve: *Thornhill 418*, NCSC). Though not expected in savannas or flatwoods, these interesting species have been found in borrow pits within or ditches adjacent to savannas or flatwoods. They are included in the key below, where indicated by a double-dagger symbol (‡).

Fig. 227

### [Linaceae] Linum L.

**Table:** 

1	Fruit as long as broad or longer, (2–)2.2–3.2(–3.3) mm long, apex acute, apiculate, or obtuse; leaves mostly 1.3–4.3 mm wide	2
–	Fruit broader than long, (1.3–)1.5–2.1(–2.3) mm long, apex depressed, flattened, or broadly rounded; leaves mostly 1.9–9.3 mm wide	4
2	(1.) Septa of fruit ciliate, false septa incomplete, exposed portions of fruit purple; leaves (1.2–)2.3–4.3(–5.6) mm wide, usually 25–50 below inflorescence	*Linum intercursum*
–	Septa of fruit glabrous, false septa essentially complete, exposed portions of fruit purple or yellow; leaves (1.0–)1.3–2.0(–3.2) mm wide, usually 50–120 below inflorescence	3
3	(2’.) Fruit ovate, (2.8–)3.0–3.2(–3.3) mm long, 2.5–3.1 mm in diam., apex minutely apiculate (with a small, slender point), exposed portions yellow; seeds 2.1-2.4 mm long; anthers averaging 1.2 mm long	Linum floridanum var. chrysocarpum
–	Fruit pyriform (pear-shaped), (2.0–)2.3–2.8(–3.0) mm long, 1.7–2.6 mm in diam., apex rounded, exposed portions purple; seeds (1.6–)1.7–2.0(–2.1) mm long; anthers averaging 0.8 mm long	Linum floridanum var. floridanum
4	(1’.) Margins of inner sepals with conspicuous stipitate glands	Linum medium var. texanum
–	Margins of the inner sepals eglandular, or with a few inconspicuous, sessile glands	*Linum striatum*

Key adapted from Weakley (2012).

Fig. 228a, b, c

### [Loganiaceae] Mitreola L.

**Table:** 

1	Leaf blades 2–8 cm long, bases cuneate to attenuate, sessile or with petioles to 15 mm long	*Mitreola petiolata*
–	Leaf blades 1–4 cm long, bases rounded, sessile or with petioles to 1 mm long	*Mitreola sessilifolia*

Key adapted from Radford et al. (1968), Weakley (2012).

Note: For assistance in distinguishing the following taxa from some similar herbs with opposite, more-or-less ovate leaves, see the auxilliary key immediately following the key to dicot families.

### Magnoliaceae

**Table:** 

1	Leaves lobed, abaxial surface not glaucous, apex emarginate to truncate; stipules free from petiole; tepals greenish-yellow, bases with orange blaze	*Liriodendron tulipifera* Fig. 113
–	Leaves not lobed, abaxial surface glaucous, apex acute to obtuse; stipules adnate to petiole; tepals creamy white (rarely greenish or yellow to orange-yellow), bases lacking orange blaze	*Magnolia virginiana* Fig. 115

Key adapted from Meyer (1997).

### [Melastomataceae] Rhexia L.

**Table:** 

1	Anthers straight, 1–2.5 mm long	2
–	Anthers curved, 5–11 mm long	3
2	(1.) Petals yellow; stem internodes moderately to sparsely glandular-hirsute; leaf blades oblong, linear, or spatulate	*Rhexia lutea*
–	Petals lavender to pink; stem internodes glabrous; leaf blades ovate or widely elliptic	*Rhexia petiolata*
3	(1’.) Stem nodes and internodes glabrous; stem and leaves blue-green, leaves very strongly ascending to nearly appressed	*Rhexia alifanus*
–	Stem nodes and usually also the internodes hirsute; stem and leaves green, leaves spreading to somewhat ascending	4
4	(3’.) Leaf blades linear or narrowly elliptic, 1–5(–7) mm wide	Rhexia mariana var. exalbida
–	Leaf blades lanceolate, elliptic, or ovate, (5–)7–20(–35) mm wide	5
5	(4’.) Petals 12–15(–18) mm long, abaxial surface glabrous; anthers 5–8 mm long; mature hypanthium 6–10(–11) mm long, glandular-setose	Rhexia mariana var. mariana
–	Petals (18–)20–25 mm long, abaxial surface glandular-hirsute; anthers 8–11 mm long; mature hypanthium (9–)10–15(–20) mm long, glabrous or glabrate	*Rhexia nashii*

Key adapted from Radford et al. (1968), Weakley (2012).

Fig. 229

### [Myricaceae] Morella Lour.

**Table:** 

1	Leaf blades elliptic to obovate, mostly 1.5–4 cm wide, 2–4× as long as wide, not or only sparsely glandular-punctate on adaxial surface; mature fruits 3.0–4.5 mm in diam.	*Morella caroliniensis*
–	Leaves oblanceolate, mostly 0.5–1.5 cm wide, 4–6× as long as wide, densely glandular-punctate on both surfaces; mature fruits 2.0–3.5 mm in diam.	2
2	(1’.) Plants medium shrubs to small trees, typically 2–10 m tall, not stoloniferous; leaves of fertile branches 4–9 cm long, 8–20 mm wide	*Morella cerifera*
–	Plants small shrubs, typically < 1 m tall, strongly stoloniferous; leaves of fertile branches 1.5–4 cm long, 3–8 mm wide	*Morella pumila*

Key adapted from Radford et al. (1968), Weakley (2012).

References: Bornstein (1997).

Fig. 230a, b, c, d, e

### [Nyssaceae] Nyssa L.

**Table:** 

1	Leaf bades thick, somewhat stiff, generally widest beyond middle; fruits (1–)2(–3) per peduncle; trunk typically swollen or buttressed at base; trees of swamps, pocosins, and depressions in pine savannas and flatwoods	*Nyssa biflora*
–	Leaf blades thin, pliable, generally widest near middle; fruits (2–)3–5(–8) per peduncle; trunk neither swollen nor buttressed at base; trees of dry to mesic upland forests, less commonly in bottomlands or other wetlands such as pine savannas and flatwoods	*Nyssa sylvatica*

Key adapted from Weakley (2012).

Fig. 231

### Onagraceae

**Table:** 

1	Petals present or absent, if present then often caducous; calyx tube not extended beyond summit of ovary, sepals persistent on capsule; stamens 4, 8, or 10–14	* Ludwigia *
–	Petals present, not caducous; calyx tube extending beyond summit of ovary, sepals deciduous; stamens 8	Oenothera fruticosa var. unguiculata Fig. 232

Key adapted from Weakley (2012).

### [Onagraceae] Ludwigia L.

**Table:** 

1	Leaves decurrent; sepals 4–7; petals 4–7; stamens 8–14; capsule obpyramidal	*Ludwigia decurrens*‡
–	Leaves not decurrent; sepals 4; petals 0–4; stamens 4; capsule various	2
2	(1’.) Pedicels 2–15 mm long; petals present, 4–15 mm long, persistent or caducous; capsules subglobose to spheric or cubic, dehiscence by an apical pore	3
–	Pedicels 0–1(–5) mm long; petals absent or present, if present then 0–6 mm long and caducous; capsules cylindrical, narrowly obconical, or narrowly obpyramidal, dehiscence irregularly loculicidal	6
3	(2.) Leaf bases cuneate; pedicels 2–5 mm long; nectary discs at style base more or less flat, inconspicuous	*Ludwigia alternifolia*‡
–	Leaf bases rounded or truncate; pedicels 4–15 mm long; nectary discs at style base domed, conspicuous	4
4	(3’.) Styles 6–10 mm long; plants glabrous, glabrescent, or pubescent with very short hairs	*Ludwigia virgata*
–	Styles 1.5–3 mm long; plants pubescent with short to long, spreading to shaggy hairs	5
5	(4’.) Sepals narrowly deltoid, broadest at or near base, 3–4× as long as wide, ascending or spreading in fruit; plants nearly glabrous or pubescent with long spreading hairs; bracteoles 5–10 mm long	*Ludwigia hirtella*
–	Sepals ovate, broadest near middle, ca. 2× as long as wide, conspicuously reflexed in fruit; plants pubescent with short, appressed or spreading hairs; bracteoles 2–4 mm long	*Ludwigia maritima*
6	(2’.) Capsules cylindrical to narrowly obpyramidal, at least 2.5–5× as long as broad; petals present	*Ludwigia linearis*
–	Capsules subglobose, obovoid, or broadly obpyramidal, 1–1.5× as long as broad; petals absent	7
7	(6’.) Plants glabrous	*Ludwigia microcarpa*
–	Plants densely pilose throughout	*Ludwigia pilosa*‡

Key adapted from Radford et al. (1968), Weakley (2012).

Note: The following species of *Ludwigia* have been collected in ditches, borrow pits, and/or roadsides in Shaken Creek Preserve or the vicinity: *Ludwigia alternifolia* L. (Sandy Run [Patterson]: *Taggart SARU 439*, WNC!), *Ludwigia decurrens* Walter (Shaken Creek Preserve: *Thornhill 1439*, NCSC), and *Ludwigia pilosa* Walter (Shaken Creek Preserve: *Thornhill 589*, *600*, *611*, NCSC). These taxa often co-occur in such areas with taxa of savanna affinities. They are, therefore, included in the key below, where indicated by a double-dagger symbol (‡).

Fig. 233a, b, c

### Orobanchaceae

**Table:** 

1	Leaves alternate, pinnately-lobed or -parted, 5–15 cm long; inflorescence spicate, compact; corolla strongly zygomorphic	*Pedicularis canadensis* Fig. 138
–	Leaves opposite, *either* unlobed (in *Agalinis*) *or* pinnately decompound (in *Seymeria*), ≤ 5 cm long; inflorescence racemose, diffuse; corolla nearly actinomorphic	2
2	(1’.) Leaves simple (reduced and inconspicuous in *Agalinis aphylla*); calyx lobes shorter than tube; corolla lavender to pink	* Agalinis *
–	Leaves pinnately decompound; calyx lobes longer than tube; corolla yellow	*Seymeria cassioides*

The following taxa are all hemiparasitic on the roots of a variety of species. Key adapted from Radford et al. (1968).

### [Orobanchaceae] Agalinis Raf.

**Table:** 

1	Plant perennial, rhizomatous; corolla 3–4 cm long	*Agalinis linifolia*
–	Plant annual, roots fibrous; corollas < 3 cm long (to 3.8 cm long in *Agalinis fasciculata* and *Agalinis purpurea*)	2
2	(1’.) Leaves scale-like, < 2.5 mm long	*Agalinis aphylla*
–	Leaves not scale-like, > 8 mm long	3
3	(2’.) Pedicels < 1.5× as long as calyx, mostly 1–5 mm long at anthesis, mostly < 8 mm long in fruit	4
–	Pedicels > 2.5× as long as calyx, mostly 5–20 mm long at anthesis, mostly > 10 mm long in fruit	6
4	(3.) Axillary fascicles of leaves numerous, well-developed; stems scabrous	*Agalinis fasciculata*
–	Axillary fascicles of leaves absent or few and poorly-developed; stems glabrous or weakly scaberulous	5
5	(4’.) Branches spreading or ascending; stems more-or-less scaberulous; corollas 18–38 mm long	*Agalinis purpurea*
–	Branches erect; stems glabrous; corollas 20–25 mm long	*Aristida virgata*
6	(3’.) Calyx tube conspicuously reticulate-veined; corolla 1–1.5 cm long, lacking 2 yellow lines within; capsule 2–3 mm in diam.; living plants yellowish-green, lacking purple pigment	*Agalinis obtusifolia*
–	Calyx tube lacking conspicuous venation; corolla 1.5–2.5 cm long, throat with 2 yellow lines within; capsule 3–4 mm in diam.; living plants dark green, usually somewhat purplish	*Agalinis setacea*

Key adapted from Radford et al. (1968), Weakley (2012).

Fig. 234a, b, c, d, e

### Plantaginaceae

**Table:** 

1	Plant scapose (leaf-bearing stems absent, leaves restricted to a basal rosette)	*Plantago sparsiflora* Fig. 130
–	Plants cauline (leaf-bearing stems present, basal rosettes of leaves present or absent)	2
2	(1’.) Cauline leaves alternate; inflorescence a raceme	*Nuttallanthus canadensis* Fig. 131
–	Cauline leaves opposite; inflorescence a thyrse or panicle, or flowers solitary in leaf axils	3
3	(2’.) Inflorescence a thyrse or panicle; corolla reddish to purple, 15–25 mm long	* Penstemon *
–	Flowers solitary in leaf axils; corolla white, sometimes shaded with purple, 6–8 mm long	*Sophronanthe pilosa* Fig. 136

Key adapted from Radford et al. (1968), Weakley (2012).

### [Plantaginaceae] Penstemon Schmidel

**Table:** 

1	Lower corolla lobes projecting beyond upper lobes, corolla throat strongly pleated ventrally; sterile filament densely bearded most of its length; plants 2–7 dm tall	*Penstemon australis*
–	Lower corolla lobes essentially equaling upper lobes, corolla throat not or only faintly pleated ventrally; sterile filament bearded only along distal 1/3 of its length; plants 4–10 dm tall	*Penstemon laevigatus*

Key adapted from Radford et al. (1968), Weakley (2012).

Fig. 235

### [Plantaginaceae] Plantago L.

**Table:** 

1	Bracts subtending basal flowers in inflorescence conspicuously exserted, ≥ 2× as long as subtended flower	*Plantago aristata‡
–	Bracts subtending basal flowers in inflorescence not conspicuously exserted, ≤ 1× as long as subtended flower	2
2	(1’.) Leaf blades broadly ovate to elliptic, 1–3× as long as wide, bases cuneate or rounded, petioles conspicuous; scapes solid and terete	3
–	Leaf blades mostly oblanceolate or lanceolate, (3–)4–10× as long as wide, bases attenuate, petioles inconspicuous or absent; scapes *either* hollow and terete *or* solid and 5-angled	4
3	(2.) Capsule 2.5–4 mm long, dehiscent near middle; sepals broadly ovate, ca. 1.5× as long as wide, mostly obtuse; petioles usually green and pubescent at base	*Plantago major‡
–	Capsule 4–6 mm long, dehiscent below middle; sepals narrowly elliptic, 2–4× as long as wide, mostly acute; petioles usually purple and glabrous at base	*Plantago rugelii*‡
4	(2’.) Bracts and calyx pubescent, at least on keels; plants annual, flowering late Mar–Jun, then soon withering	*Plantago virginica*‡
–	Bracts and calyx glabrous; plants perennial, flowering Apr–Nov, not soon withering	5
5	(4’.) Spikes very densely flowered (≥ 8 flowers/fruits per cm), rachis hidden; scape solid, 5-angled	*Plantago lanceolata‡
–	Spikes loosely flowered (3–6 flowers/fruits per cm), rachis visible throughout length; scape hollow, terete	*Plantago sparsiflora*

Key adapted from Radford et al. (1968), Weakley (2012).

Note: Of the several species of *Plantago* reported for various habitats in Sandy Run, only *Plantago sparsiflora* Michx. was reported for pine savannas or flatwoods (Taggart 2010). However, *Plantago sparsiflora* also occurs in disturbed areas near pine savannas (e.g., in scrapes and roadside ditches), where several weedy congeners are often found. In order to facilitate the distinction of *Plantago sparsiflora* from related species, the key below includes all species of *Plantago* reported from disturbed areas in Sandy Run by Taggart (2010) or collected from disturbed areas by the senior author in Shaken Creek Preserve. Species restricted to disturbed areas are indicated by a double-dagger symbol (‡).

Fig. 130

### [Polygalaceae] Polygala L.

**Table:** 

1	Fresh flowers orange or yellow	2
–	Fresh flowers pink or purple, often with white or green portions	4
2	(1.) Inflorescence a dense raceme; flowers orange (drying yellow)	*Polygala lutea*
–	Inflorescence a dense to open, many-branched cyme; flowers yellow	3
3	(2’.) Plants 4.5–12 dm tall; stems solitary; basal leaves linear-lanceolate, 3.5–14 cm long, ca. 15–20× as long as wide, persistent as a basal rosette, cauline leaves linear-subulate, sharp-tipped, strongly reduced, becoming bractlike distally; seeds glabrous, 0.7–0.9 mm long	*Polygala cymosa*‡
–	Plants 1–4 dm tall; stems 1–several from base of plant; basal leaves spatulate, 3–7 cm long, ca. 10× as long as wide, usually not persistent after flowering, cauline leaves narrowly spatulate to linear, blunt-tipped, only slightly reduced, not bractlike distally; seeds pubescent, 0.5–0.7 mm long	*Polygala ramosa*
4	(1’.) Leaves alternate, glaucous; corolla ≥ 2× as long as “wings” (lateral, petaloid sepals)	*Polygala incarnata*
–	Leaves whorled (at least at lower nodes), not glaucous; corolla ≤ 1× as long as “wings”	5
5	(4’.) Racemes 3–6 mm in diam., pointed in outline	*Polygala verticillata*
–	Racemes 7–15 mm in diam., rounded in outline (pointed in *Polygala hookeri*)	6
6	(5’.) Racemes sparsely flowered (ca. 10 flowers per cm), to 6 cm long (including portion with dropped fruits), 0.7–1.2 cm in diam., apex pointed	*Polygala hookeri*
–	Racemes densely flowered (ca. 20 flowers per cm), to 4.5 cm long (including portion with dropped fruits), 0.7–2 cm in diam., apex rounded to truncate	7
7	(6’.) Bracts ca. 1 mm long; “wings” 1.5–2.5 mm wide, acute	*Polygala brevifolia*
–	Bracts 1.5–3 mm long; “wings” 3–4 mm wide, acuminate	*Polygala cruciata*

Key adapted from Weakley (2012).

Note: *Polygala ramosa* Elliott was collected along a roadside in Sandy Run ([Watkins]: *Taggart SARU 399* (WNC!)). Though not reported or seen in savannas or flatwoods in Shaken Creek Preserve or the vicinity, it is often a component of wet pine savannas and is included in the key below, where indicated by a double-dagger symbol (‡).

Figs 236, 237a, b, c, d, e

### [Primulaceae] Lysimachia L.

**Table:** 

1	Leaves 2–4 per node, blades 8–20 mm wide, with 3–5 prominent veins	*Lysimachia asperulifolia*
–	Leaves 2 per node, blades 1–8 mm wide, with 1 prominent vein	*Lysimachia loomisii*

Key adapted from Cholewa (2009), Weakley (2012).

Fig. 238

### Ranunculaceae

**Table:** 

1	Plant a weakly climbing vine, hermaphroditic; leaves opposite; flowers perfect; sepals petaloid, 2.5–5 cm long, persistent in fruit; stamens included within sepals, not conspicuous	*Clematis crispa* Fig. 133
–	Plant an erect herb, dioecious; leaves alternate; flowers usually imperfect, occasionally perfect; sepals not petaloid, 0.1–1.8 cm long, not persisten in fruit; stamens exserted, conspicuous (on staminate flowers)	*Thalictrum cooleyi* Fig. 139

Key adapted from Weakley (2012).

References: Whittemore and Parfitt (1997).

### Rosaceae

**Table:** 

1	Stems with numerous prickles and/or spines; fruit an aggregate of drupelets or of achenes enclosed within fleshy hypanthium (“hip”)	2
–	Stems lacking prickles and spines; fruit a pome, drupe, or aggregate of achenes not enclosed within a fleshy hypanthium	3
2	(1.) Leaves pinnate, leaflets 5–9; petals pink; fruit an aggregate of achenes enclosed within fleshy, red hypanthium	*Rosa palustris* Fig. 239
–	Leaves trifoliolate or palmate, leaflets 3–5; petals white (rarely pink); fruit an aggregate of purple to black drupelets not enclosed within hypanthium	* Rubus *
3	(1’.) Plant an herb; petals yellow; fruit an aggregate of achenes	*Potentilla simplex* Fig. 140
–	Plant a shrub or tree; petals white (rarely pinkish in *Amelanchier*); fruit a pome or drupe	4
4	(3’.) Petiole with 2 glands present near junction with blade; ovary superior; fruit a drupe; plant a medium to tall tree	Prunus serotina var. serotina Fig. 122
–	Petiole eglandular; ovary inferior; fruit a pome; plant a shrub to small tree	5
5	(4’.) Adaxial leaf surface lacking reddish-brown trichomes along midrib; flowers and fruits in racemes	* Amelanchier *
–	Adaxial leaf surface with reddish-brown trichomes along midrib; flowers and fruits in corymbs	*Aronia arbutifolia* Fig. 240

Key adapted from Radford et al. (1968), Weakley (2012).

### [Rosaceae] Amelanchier Medik.

**Table:** 

1	Plant not rhizomatous	*Amelanchier canadensis*
–	Plant rhizomatous	2
2	(1’.) Summit of ovary glabrous or sparsely pubescent; pome purple; expanding leaves glabrous to densely tomentose below	*Amelanchier obovalis*‡
–	Summit of ovary densely wooly; pome red; expanding leaves densely tomentose below	*Amelanchier spicata*

Key adapted from Radford et al. (1968), Weakley (2012).

Note: Though not seen in or reported from Shaken Creek Preserve or the vicinity, *Amelanchier obovalis* (Michx.) Ashe, a common species of pine savannas and pocosins of the North Carolina Coastal Plain, is included in the key below, where indicated by a double-dagger symbol (‡).

Fig. 241

### [Rosaceae] Rubus L.

**Table:** 

1	Primocanes (non-flower-bearing stems) prostrate, creeping, or low-arching, rooting at tips or nodes; abaxial leaflet surface sparsely pubescent to glabrous; sepals 6–8 mm long	2
–	Primocanes erect, ascending, or high-arching, not rooting; abaxial leaflet surface densely tomentose or pubescent; sepals 4–6 mm long	3
2	(1.) Stems bearing stout-based, usually recurved prickles, lacking narrow-based bristles; leaves deciduous; flowers usually ≥ 2 per branch	*Rubus flagellaris*‡
–	Stems with or without stout-based, recurved prickles, bearing narrow-based bristles; leaves tardily deciduous, turning red and persistent in winter; flowers usually 1 per branch	*Rubus trivialis*‡
3	(1’.) Leaflets oblanceolate to obovate, conspicuously widest beyond middle, apex usually obtuse or rounded; abaxial leaflet surface densely white- or gray-tomentose	*Rubus cuneifolius*
–	Leaflets lanceolate to ovate, widest below or near middle, apex usually acute or acuminate; abaxial leaflet surface softly pubescent but not white- or gray-tomentose	*Rubus pensilvanicus*

Key adapted from Radford et al. (1968), Weakley (2012).

Note: *Rubus flagellaris* Willd. and *Rubus trivialis* Michx. were reported from roadsides and other disturbed areas in Sandy Run by Taggart (2010). While neither of these species has been seen in savannas or flatwoods within Shaken Creek Preserve, both could be found in such habitats, particularly where disturbed or not burned recently. Both species are included in the key below, where indicated by a double-dagger symbol (‡).

Fig. 242

### Rubiaceae

**Table:** 

1	Plant prostrate, rooting at nodes; leaves ovate, about as wide as long; flowers paired; ovaries connate and developing into a single red (rarely whitish) berry	*Mitchella repens* Fig. 243
–	Plant erect, not rooting at nodes; leaves lanceolate, elliptic, or oblanceolate, distinctly longer than wide; flowers solitary or in few-flowered cymes, not paired; ovaries and fruit not as above	2
2	(1’.) Fruit comprised of 2 indehiscent, 1-seeded carpels; leaves 2–7 cm long	* Diodia *
–	Fruit comprised of 1 apically dehiscent, many-seeded carpel; leaves 1–2 cm long	*Oldenlandia uniflora* Fig. 244

Key adapted from Radford et al. (1968), Weakley (2012).

Note: For assistance in distinguishing the following taxa from some similar herbs with opposite, more or less ovate leaves, see the auxilliary key immediately following the key to dicot families.

### [Rubiaceae] Diodia L.

**Table:** 

1	Sepals 4, similar in size, 2–4 mm long; style entire; leaves mostly 2–4 cm long, 2–6 mm wide; plants of dry sites	*Diodia teres*
–	Sepals 2 or if 4, then 2 markedly reduced, 4–6 mm long; style bifid; leaves mostly 2–7 cm long, 4–12 mm wide; plants of mesic or wet sites	*Diospyros virginiana*

Key adapted from Radford et al. (1968), Weakley (2012).

Fig. 245

### [Sarraceniaceae] Sarracenia L.

**Table:** 

1	Pitchers decumbent, urceolate (urn-shaped: broader basally, contracted apically); hoods erect or with lobes arched together (but not covering orifices horizontally); petals red to maroon	Sarracenia purpurea var. venosa
–	Pitchers erect, tubiform (trumpet-shaped: widening from base to apex); hoods more-or-less horizontal, at least partially covering orifices; petals either yellow or red to maroon	2
2	(1’.) Petals yellow; pitcher hood 3–10 cm long, (3–)5–14 cm wide, margins reflexed	*Sarracenia flava*
–	Petals maroon; pitcher hood 0.7–4.5 cm long, 0.7–4 cm wide, margins not reflexed	Sarracenia rubra ssp. rubra

Key adapted from Mellichamp and Case (2009), Weakley (2012).

Note: Hybridization is common among many taxa of *Sarracenia*. Though not included in the key below, hybrids are generally recognizable by their intermediate morphology. At Shaken Creek Preserve, Sarracenia × catesbaei Elliott (= *Sarracenia flava* L. × *Sarracenia purpurea* L.), with its erect but relatively dwarfed (compared to *Sarracenia flava*) stature, can usually be found in savannas where both parents co-occur (as in the savannas along Flo Road, east of Meadow Lake Road). Hybrids involving Sarracenia rubra Walter ssp. rubra, a species reported for the site by LeBlond (2000) but not seen by the senior author, are also possible, though the presumably small population size of *Sarracenia rubra* would likely limit extensive occurrences of such hybrids.

Fig. 246

### [Violaceae] Viola L.

**Table:** 

1	Plant producing stolons; corolla white	2
–	Plant not producing stolons; corolla blue-violet	4
2	(1.) Leaf blades lance-ovate, base broadly cuneate to subtruncate	*Viola primulifolia*
–	Leaf blades linear to lanceolate, base narrowly cuneate	3
3	(2’.) Leaf blades lanceolate, < 8× as long as wide; plant glabrous	Viola lanceolata var. lanceolata
–	Leaf blades linear or narrowly lanceolate, > 10× as long as wide; plant glabrous to pubescent	Viola lanceolata var. vittata
4	(1’.) Most or all leaf blades longer than wide, narrowly ovate to long-triangular, apices acute	5
–	Leaf blades as wide as long or wider, ovate to suborbicular, apices obtuse (rarely acute)	7
5	(4.) Leaf margins with all teeth uniform, leaf bases cordate	Viola sororia var. missouriensis, in part
–	Leaf margins with basal teeth distinctly longer than middle and upper, leaf bases truncate to subcordate	6
6	(5’.) Leaves broadly triangular in outline, not much longer than wide, margins with basal teeth numerous, fine	*Viola brittoniana*
–	Leaves narrowly ovate-triangular in outline, much longer than wide, margins with basal teeth few, coarse	Viola sagittata var. sagittata
7	(4’.) Leaf blades deeply lobed (at least basally), margins crenate to entire	*Viola septemloba*
–	Leaf blades unlobed, margins toothed	Viola sororia var. missouriensis, in part

Key adapted from Weakley (2012).

Note: The leaf characters in the following key refer to mature leaves; the earliest one or two leaves in many species of *Viola* display atypical shapes and/or margins, which are not accounted for in the key.

Fig. 247a, b, c, d, e

### Vitaceae

**Table:** 

1	Leaves palmately compound, leaflets (3–)5(–7); tendrils several-branched, terminating in disks	*Parthenocissus quinquefolia* Fig. 99
–	Leaves simple, often shallowly 3–5(–7)-lobed; tendrils unbranched, lacking disks	Vitis rotundifolia var. rotundifolia Fig. 100

Key adapted from Weakley (2012).

## Analysis

### Floristic Summary

The flora of the savannas, flatwoods, and sandhills of SCP proper, based on vouchered specimens and reports (i.e., LeBlond 2000), consists of 450 taxa (i.e., species, subspecies, or varieties) in 204 genera and eighty-three families (Table 5). Of these 450 taxa, 432 (96%) are vouchered; eighteen (4%) are known only from reports. Thirty-two taxa (7.1%) are listed as Significantly Rare (Table 2), and thirty-eight (8.4%) are on the NCNHP Watch List (Table 3). Three species are federally endangered (*Carex lutea*, *Lysimachia asperulifolia*, and *Thalictrum cooleyi*), and six are Federal Species of Concern (*Allium* species 1, *Amorpha georgiana*, *Dionaea muscipula*, *Parnassia caroliniana* Michx., *Rhynchospora decurrens* Chapm., and *Rhynchospora thornei* Kral).

An additional 102 taxa in twenty-seven genera and seven families were collected or reported from savannas or flatwoods in the vicinity of SCP (i.e., within two miles of SCP, including Sandy Run Savannas State Natural Area; Table 5). Of these 102 additional taxa, seventy-seven (75.5%) are vouchered; twenty-five (24.5%) are known only from reports. Eighteen taxa (17.6%) are listed as Significantly Rare (Table 2), and seven (6.9%) are on the NCNHP Watch List (Table 3). Four taxa are Federal Species of Concern (*Plantago sparsiflora* Michx., *Scleria* species 1, Trillium pusillum Michx. var. pusillum, and *Xyris scabrifolia* R.M. Harper).

**Figure 248.**
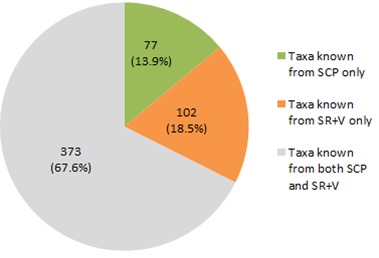
Number and relative percentage of taxa treated in this work that were collected or reported from either Shaken Creek Preserve (“SCP”), the vicinity (i.e., within two mile of Shaken Creek Preserve, including Sandy Run Savannas State Natural Area; “SR+V”), or in both Shaken Creek Preserve and the vicinity (“SCP and SR+V”). “Taxa” includes species, subspecies, and varieties. Suppl. material 2

In total, 552 taxa in 231 genera and ninety families are treated in this guide (Table 5). Seventy-seven taxa (13.9%) were collected or reported only from SCP; 102 taxa (18.5%) were collected or reported only from the vicinity of SCP; and 373 taxa (67.6%) were collected or reported from both SCP and the vicinity (Fig. 248). Of the 552 total taxa, 514 (93.1%) are vouchered; thirty-eight (6.9%) are known only from reports. Fifty taxa (9.1%) are listed as Significantly Rare (Table 2), and forty-five (8.2%) are on the NCNHP Watch List (Table 3). Three taxa are federally endangered, and ten are Federal Species of Concern.

**Figure 249.**
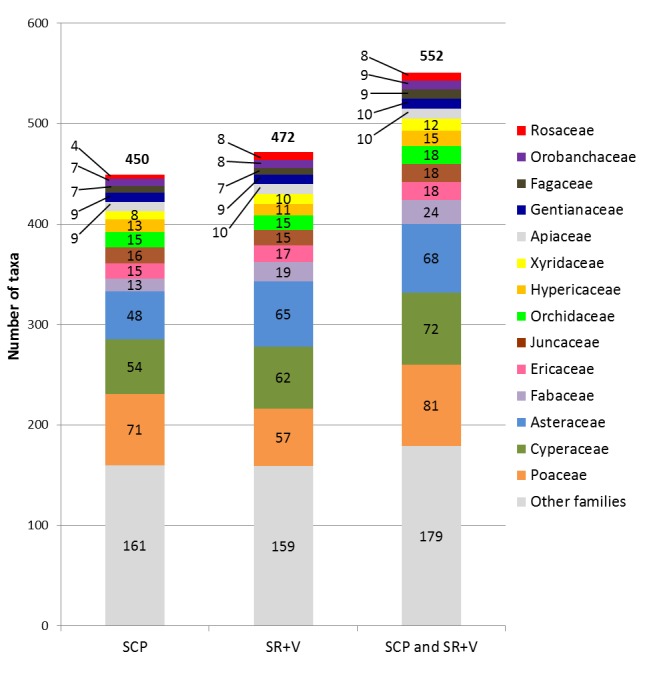
Comparison of the richest plant families present in the savannas, flatwoods, and sandhills in Shaken Creek Preserve (“SCP”), in the vicinity (i.e., within two mile of Shaken Creek Preserve, including Sandy Run Savannas State Natural Area; “SR+V”), and in both Shaken Creek Preserve and the vicinity (“SCP and SR+V”). “Taxa” includes species, subspecies, and varieties. Families represented by ≥ 8 total taxa are represented individually; families represented by < 8 total taxa are subsumed in the “Other families” category. Values appearing within or beside the columns indicate the total number of taxa from each indicated family; values appearing above each column indicate the total number of taxa across all familles. Values include taxa vouchered or known only from reports. Suppl. material 3

**Figure 250.**
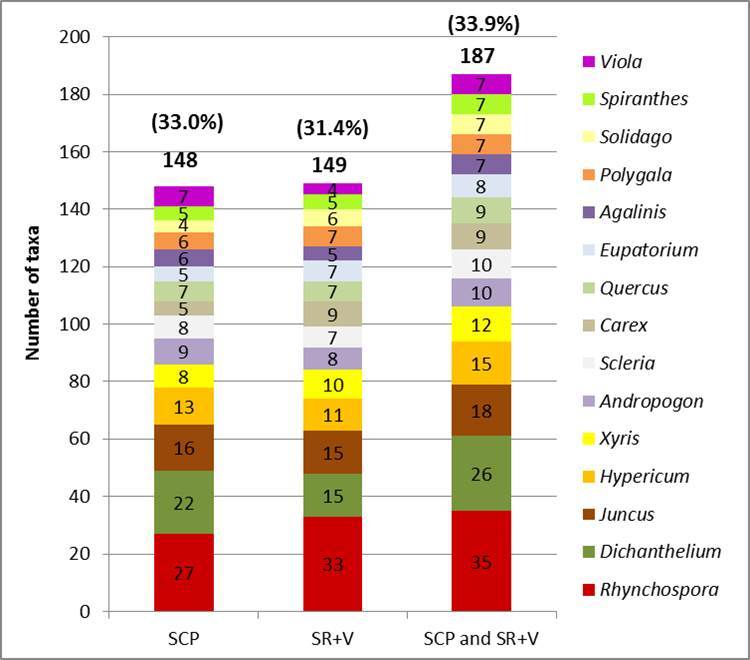
Comparison of the richest genera in the savannas, flatwoods, and sandhills in Shaken Creek Preserve (“SCP”), in the vicinity (i.e., within two mile of Shaken Creek Preserve, including Sandy Run Savannas State Natural Area; “SR+V”), and in both Shaken Creek Preserve and the vicinity (“SCP and SR+V”). “Taxa” includes species, subspecies, and varieties. Genera represented by ≥ 7 total taxa are represented individually; genera represented by < 7 total taxa are not included. Values appearing within the columns indicate the total number of taxa from each included genus. Values appearing above each column indicate the total number of taxa across all included genera; percentages appearing above each column indicate the percentage of the flora of the particular area that is represented by the included genera. Values include taxa vouchered or known only from reports. Suppl. material 4

Among all taxa treated in this guide, the eudicotyledons are the most species-rich group, containing 290 taxa; the monocotyledons are a close second, containing 242 taxa. The richest families among eudicotyledons are Asteraceae (68 taxa), Fabaceae (24 taxa), Ericaceae (18 taxa), Hypericaceae (15 taxa), Apiaceae (10 taxa), and Gentianaceae (10 taxa; Fig. 249). The richest genera among eudicotyledons are *Hypericum* L. (15 taxa), *Quercus* L. (9 taxa), *Eupatorium* L. (8 taxa), *Agalinis* Raf. (7 taxa), *Polygala* L. (7 taxa), *Solidago* L. (7 taxa), and *Viola* L. (7 taxa; Fig. 250). The richest families among monocotyledons are Poaceae (81 taxa), Cyperaceae (72 taxa), Juncaceae (18 taxa), Orchidaceae (18 taxa), and Xyridaceae (12 taxa; Fig. 249). The richest genera among monocotyledons are *Rhynchospora* Vahl (35 taxa), *Dichanthelium* (Hitchc. & Chase) Gould (26 taxa), *Juncus* L. (18 taxa), *Xyris* L. (12 taxa), *Andropogon* L. (10 taxa), and *Scleria* P.J. Bergius (10 taxa; Fig. 250).

Among all taxa treated in this guide, the most species-rich habit is herbs (447 taxa), followed by trees and shrubs (83 taxa), and vines (22 taxa). Among the herbs, Poaceae (81 taxa), Cyperaceae (72 taxa), and Asteraceae (66 taxa) are the richest families, followed by Fabaceae (18 taxa), Juncaceae (18 taxa), and Orchidaceae (18 taxa). The richest family of trees and shrubs is Ericaceae (18 taxa), followed by Fagaceae (9 taxa), Hypericaceae (8 taxa), Rosaceae (7 taxa), Aquifoliaceae (4 taxa), and Pinaceae (4 taxa). The richest families of vines are Smilacaceae (5 taxa) and Fabaceae (4 taxa), followed by Bignoniaceae (2 taxa), Convolvulaceae (2 taxa), and Vitaceae (2 taxa; Fig. 12a, b, c).

Among the community types included in this work, the most species-rich is Very Wet Loamy Pine Savanna; the least species-rich is Pine/Scrub Oak Sandhill (Mesic Transition subtype; Fig. 12d).

Only one exotic taxon, Pinus elliottii Engelm. var. elliottii, which was planted by a timber company in a flatwoods in SCP prior to the site’s purchase by The Nature Conservancy, was collected or reported from pertinent habitats in the study area.

One species, *Aletris lutea* Small, is here reported as a state record; another taxon, Panicum dichotomiflorum Michx. var. puritanorum Svenson, is reported as a Pender County record.

## Supplementary Material

Supplementary material 1 Climate diagram data Data type: Climate normals
File: oo_5756.xlsx Robert Thornhill

Supplementary material 2 Pie chart data Data type: Occurence
File: oo_5757.xlsx Robert Thornhill

Supplementary material 3 Richest families data Data type: Occurence
File: oo_5758.xlsx Robert Thornhill

Supplementary material 4 Richest genera data Data type: Occurence
File: oo_5759.xlsx Robert Thornhill

Supplementary material 5 Taxa by habit and community type data Data type: Occurence
File: oo_5760.xlsx Robert Thornhill

Supplementary material 6 List of Voucher Specimens Collected by the Senior Author Data type: Occurences
Brief description: This spreadsheet lists all specimecns (and associated data) collected by the senior author from throughout Shaken Creek Preserve, including specimens collected from the community types treated in this manuscript and from several other community types not treated here (example: swamps, roadsides, etc.). Location data for rare taxa (i.e., those listed in table 2) and for *Chamaelirium luteum* and all *Sarracenia* spp. (which face some degree of collection pressure) has been removed. The list is currently sorted to match the order of the checklist in the manuscript but can easily be resorted any number of ways, including alphabetically by taxon.
File: oo_7003.xls Robert Thornhill

Supplementary material 7 Checklist of Taxa Data type: occurence
Brief description: This file is simply a spreadsheet of the data presented in the checklist portion of the manuscript.
File: oo_7004.xls Robert Thornhill

## Figures and Tables

**Figure 3a.**
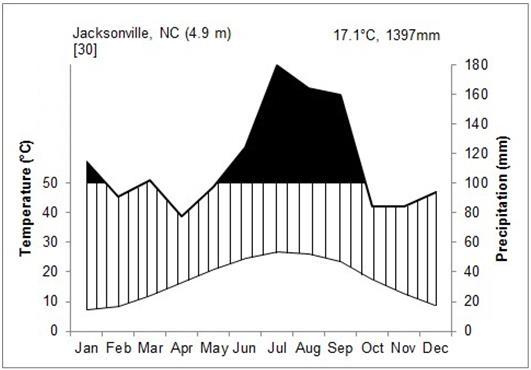
Walter climate diagram for the weather station in Jacksonville, NC (Onslow Co.).

**Figure 3b.**
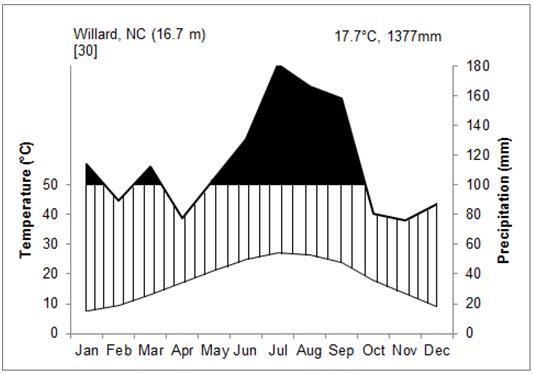
Walter climate diagram for the weather station in Willard, NC (Pender Co.).

**Figure 6a.**
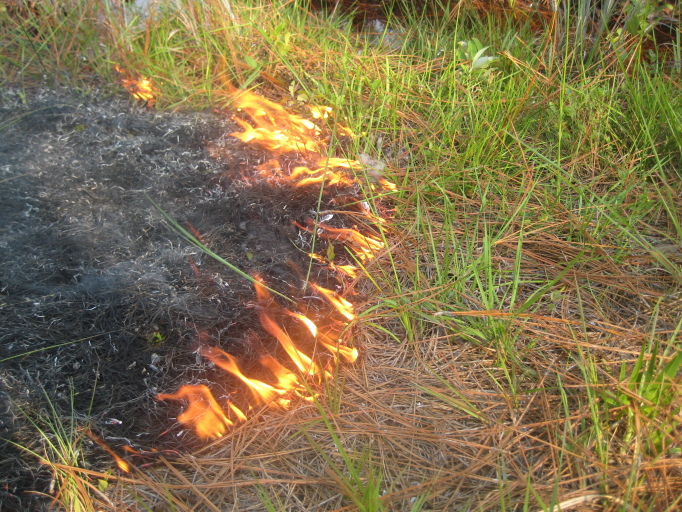
In savannas that are frequently burned and lack significant woody vegetation, fires are small enough to be stepped over harmlessly (photo by R. Thornhill).

**Figure 6b.**
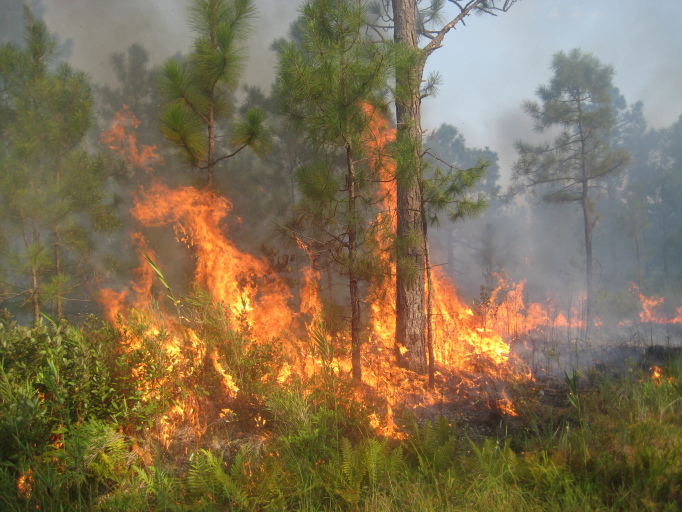
Fire intensity increases along the woody margins of savannas and flatwoods (photo by R. Thornhill).

**Figure 7a.**
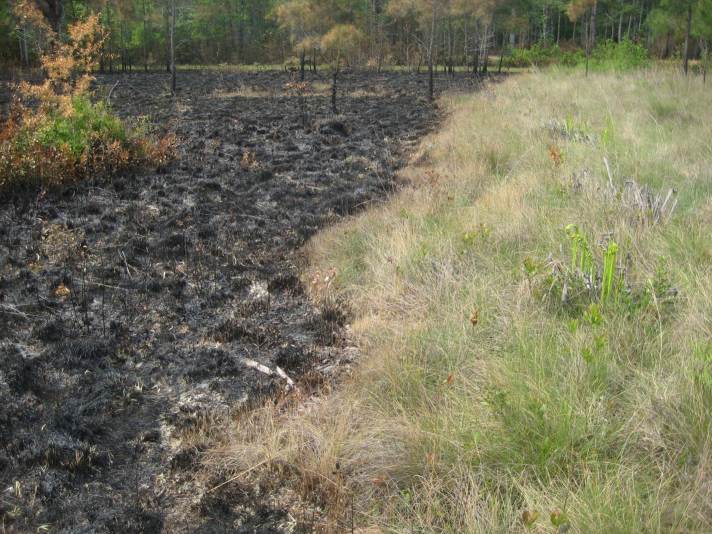
April 30, two days after a burn (photo by R. Thornhill).

**Figure 7b.**
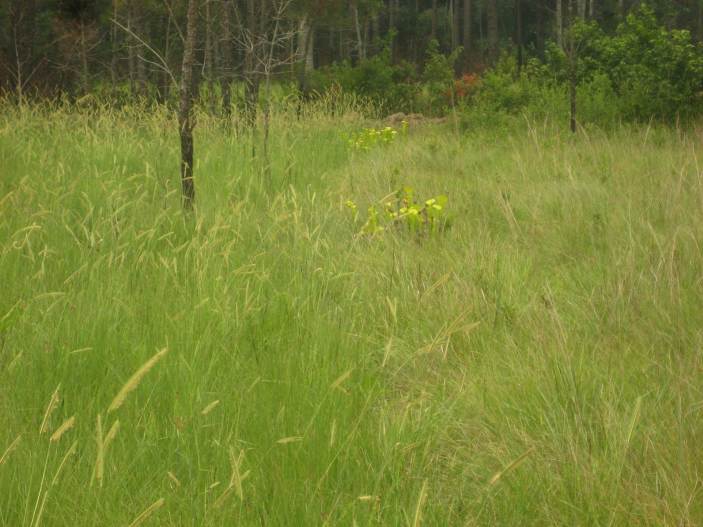
June 27, just less than two months after a burn. Notice the profusion of flowering stalks of *Ctenium aromaticum* in the burned area (left) versus their near-absence in the unburned (right) area (photo by R. Thornhill).

**Figure 8a.**
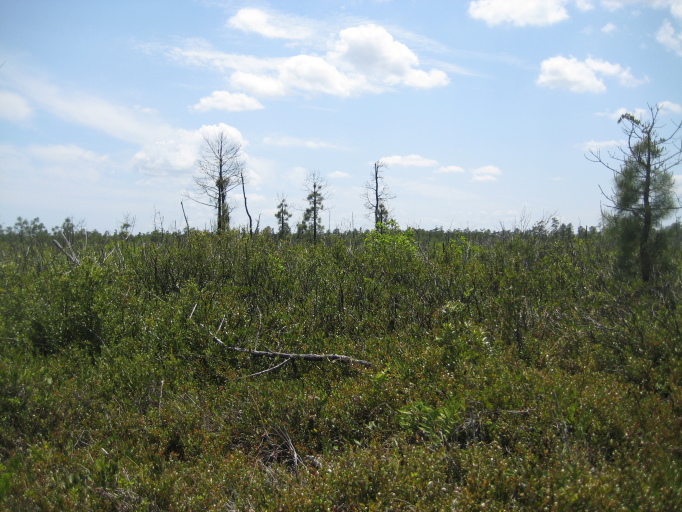
Pocosin: note the dense growth of low (mostly evergreen) shrubs and the absence of tall trees (photo by R. Thornhill).

**Figure 8b.**
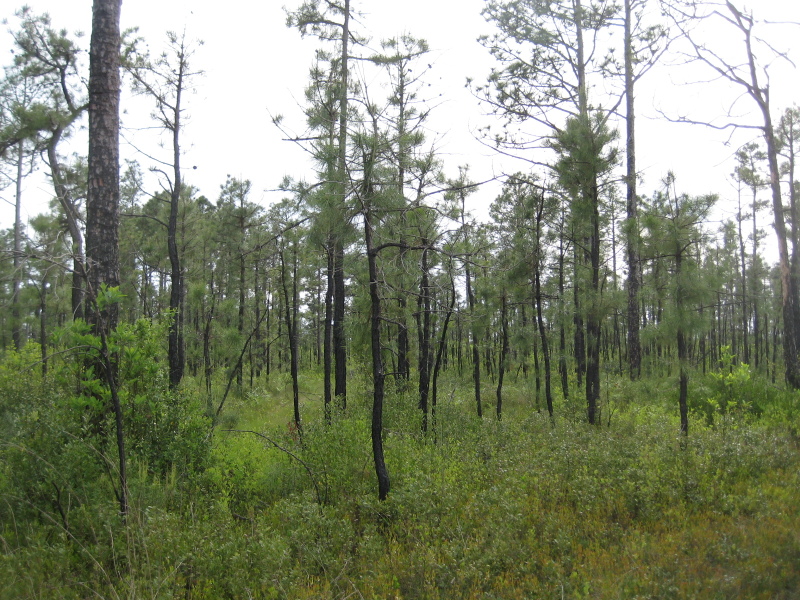
Pone Pine Woodland: note the dense stand of *Pinus serotina* (Pond pine) that characterizes this community (photo by R. Thornhill).

**Figure 9a.**
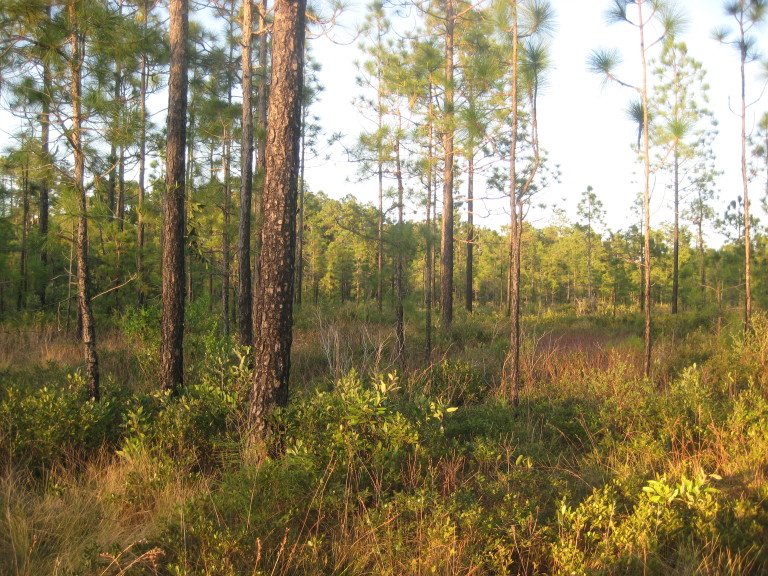
A typical wet pine flatwoods (photo by R. Thornhill).

**Figure 9b.**
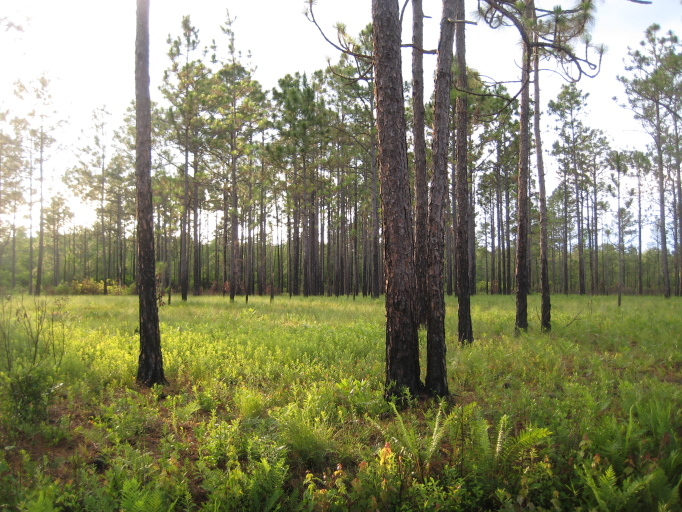
When frequently burned, the physiognomy of wet pine flatwoods resembles that of Pine Savannas; however, species diversity and overall richness, especially at a small scale, is lower in in this community type than in true savannas (photo by R. Thornhill).

**Figure 10a.**
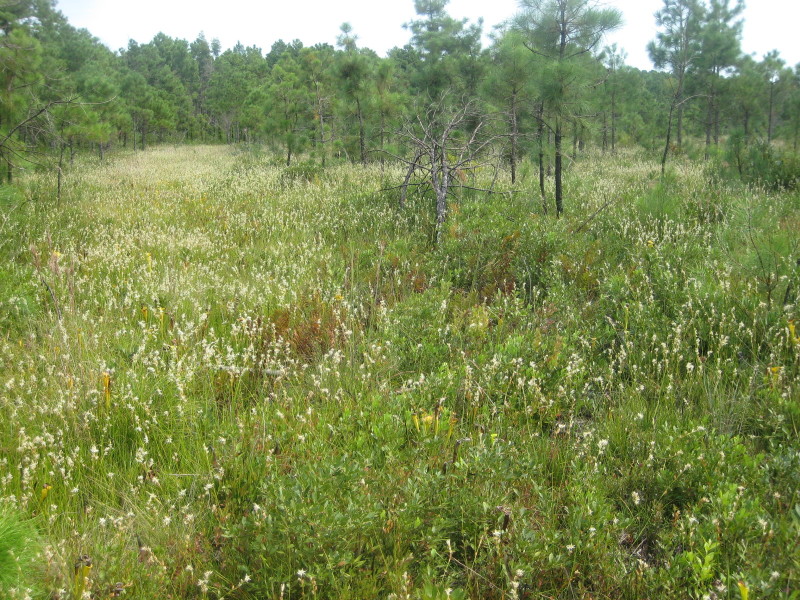
A super-abundance of the white-flowered *Pleea tenuifolia* (Rush featherling) characterizes this community type (photos by R. Thornhill).

**Figure 10b.**
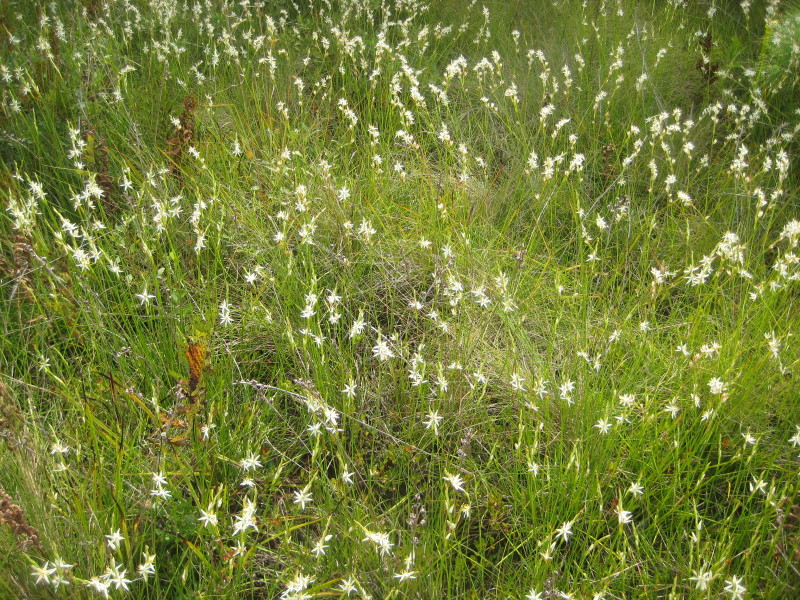


**Figure 11a.**
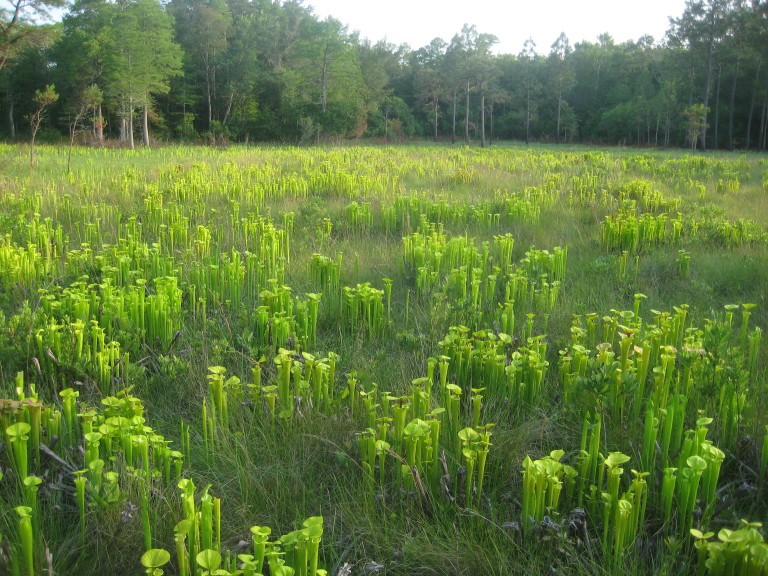
This Wet Loamy Pine Savanna, treeless and dominated by the charismatic Sarracenia flava (yellow pitcher plant), is one of the most stunning vistas in Shaken Creek Preserve – or perhaps anywhere (photo by R. Thornhill)!

**Figure 11b.**
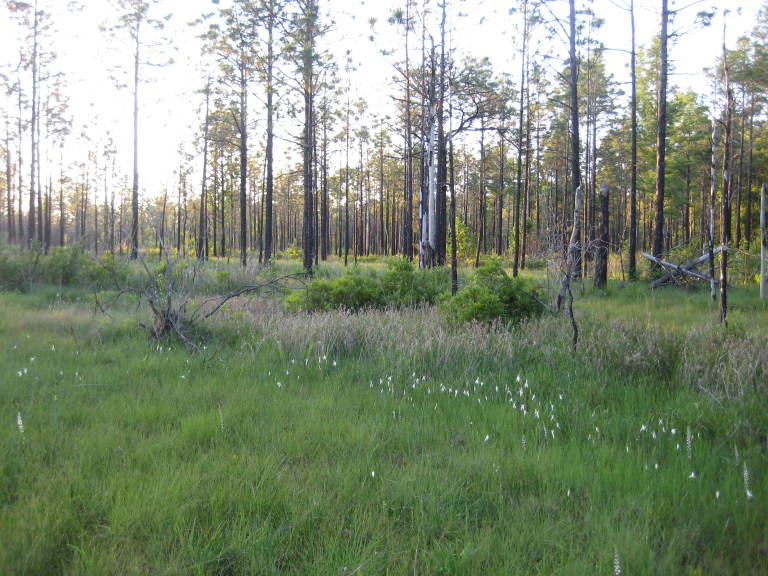
Very Wet Loamy Pine Savannas harbor a suite of exceptionally rare species, often found in slight depressions, like the narrow one running from left to right in this photograph (photo by R. Thornhill).

**Figure 12a.**
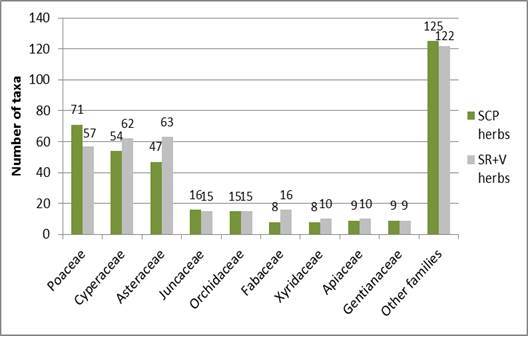
Taxonomic distribution of herbaceous taxa. Families represented by ≥ 8 herbaceous taxa in either Shaken Creek Preserve or in the vicinity are represented individually; families represented by < 8 herbaceous taxa in both Shaken Creek Preserve and in the vicinity are subsumed in the “Other families” category.

**Figure 12b.**
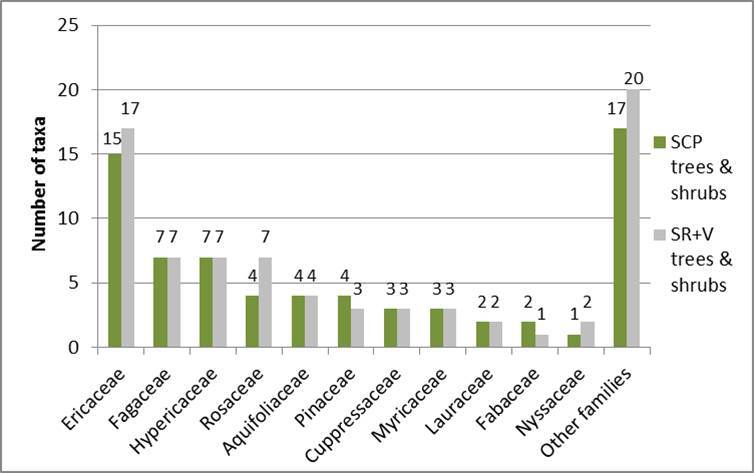
Taxonomic distribution of tree and shrub taxa. Families represented by ≥ 2 tree or shrub taxa in either Shaken Creek Preserve or in the vicinity are represented individually; families represented by only 1 tree or shrub taxon in both Shaken Creek Preserve and in the vicinity comprise the “Other families” category.

**Figure 12c.**
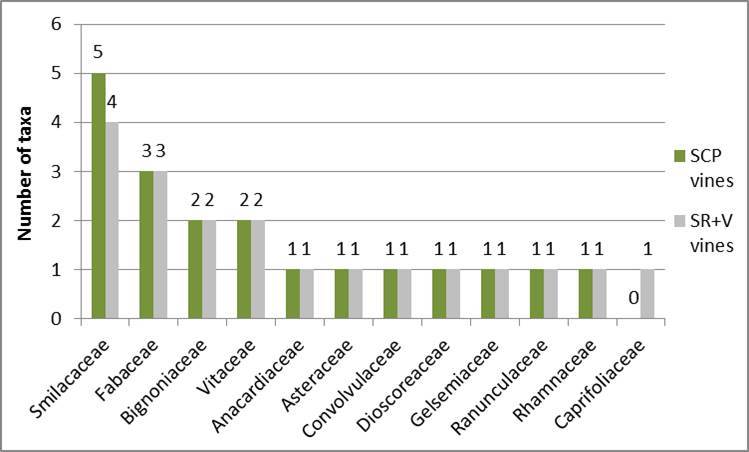
Taxonomic distribution of vines (herbaceous or woody plants that climb by means of holdfasts or by twining). All families containing vine taxa are included.

**Figure 12d.**
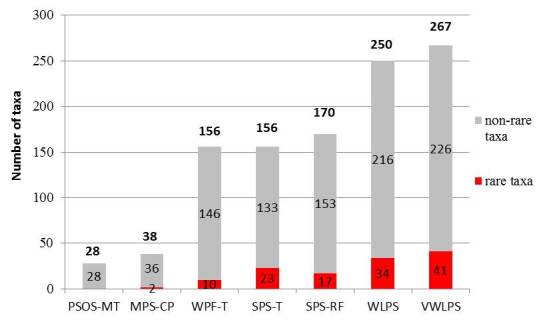
Distribution by community type of taxa collected or reported from Shaken Creek Preserve. Values above columns indicate the total number of taxa (i.e., rare and non-rare) collected or reported from each community type. "Rare taxa” are those taxa listed by the NC Natural Heritage Program as Significantly Rare or Watch List (Gadd and Finnegan 2012). Community types follow Schafale (2012) and are arranged according to increasing soil moisture (i.e., from the driest to wettest community type). Abbreviations are as follows: PSOS-MT = Pine/Scrub Oak Sandhill (Mesic Transition subtype); MPS-CP = Mesic Pine Savanna (Coastal Plain subtype); WPF-T = Wet Pine Flatwoods (Typic subtype); SPS-T = Sandy Pine Savanna (Typic subtype); SPS-RF = Sandy Pine Savanna (Rush Featherling subtype); WLPS = Wet Loamy Pine Savanna; VWLPS = Very Wet Loamy Pine Savanna.

**Figure 13.**
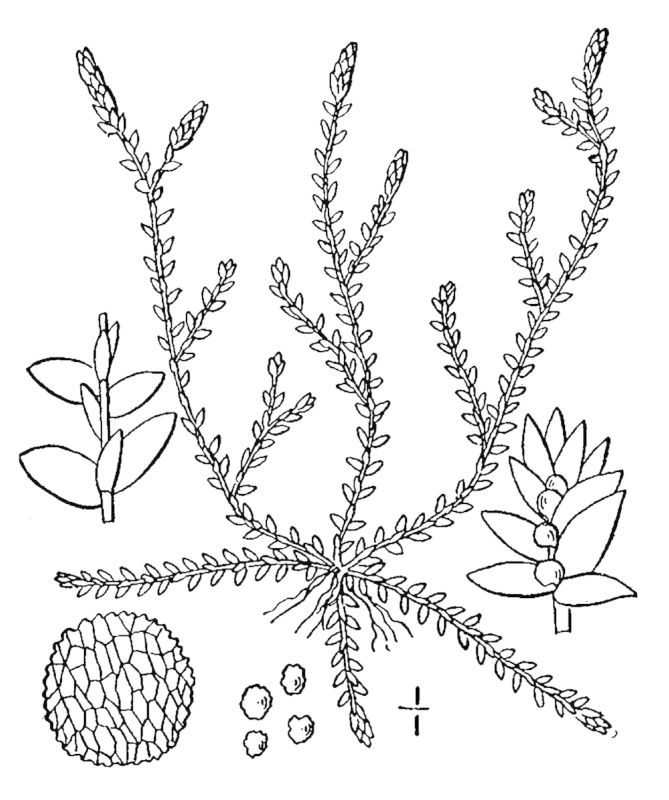
*Selaginella apoda* (from Britton and Brown 1913).

**Figure 14a.**
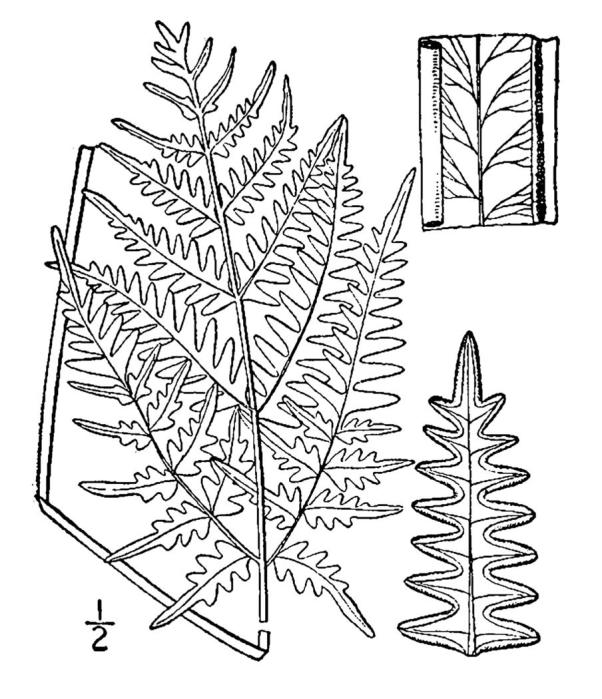
From Britton and Brown (1913).

**Figure 14b.**
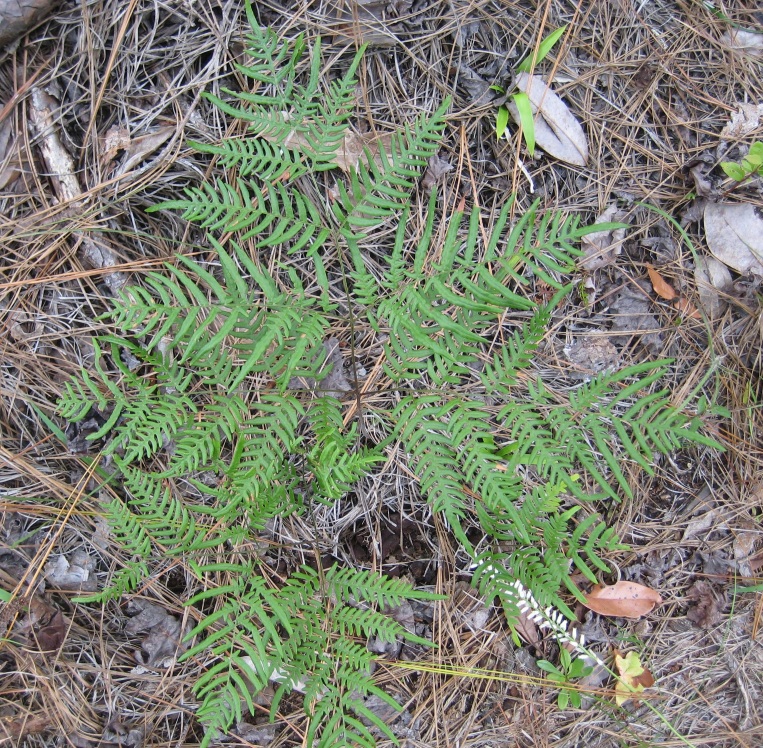
Pteridium aquilinum var. pseudocaudatum (photo by R. Thornhill).

**Figure 15a.**
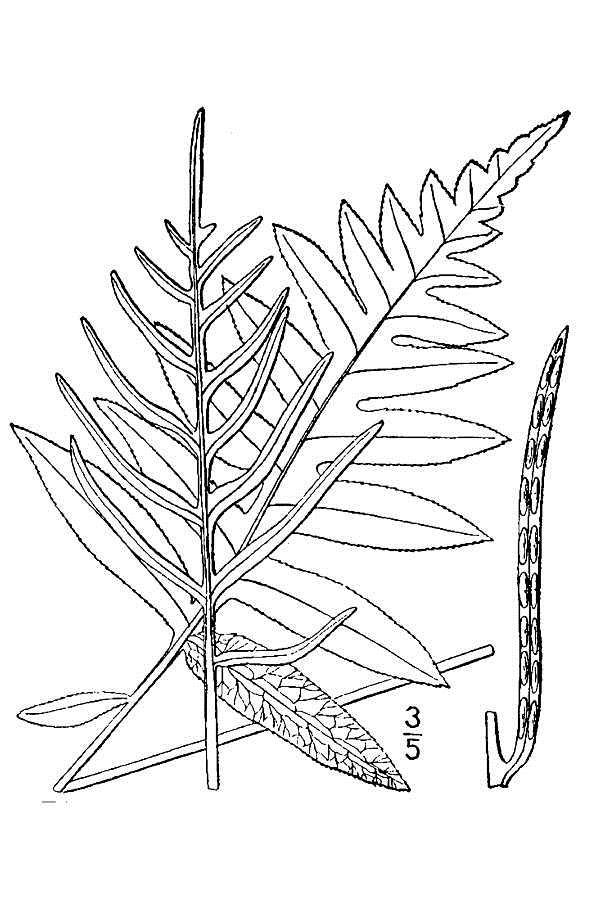
*Woodwardia areolata* (from USDA-NRCS 2012).

**Figure 15b.**
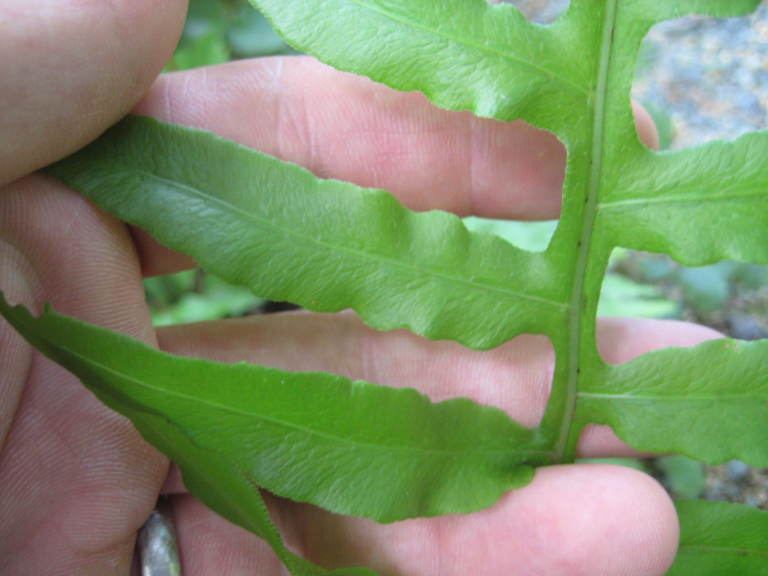
*Woodwardia areolata*: showing winged rachis (photo by R. Thornhill).

**Figure 15c.**
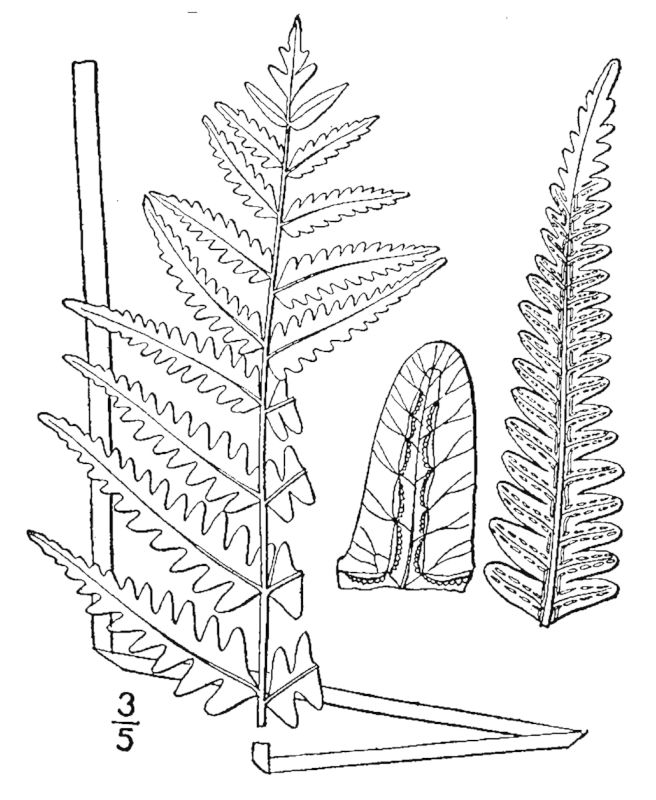
*Woodwardia virginica* (from Britton and Brown 1913).

**Figure 15d.**
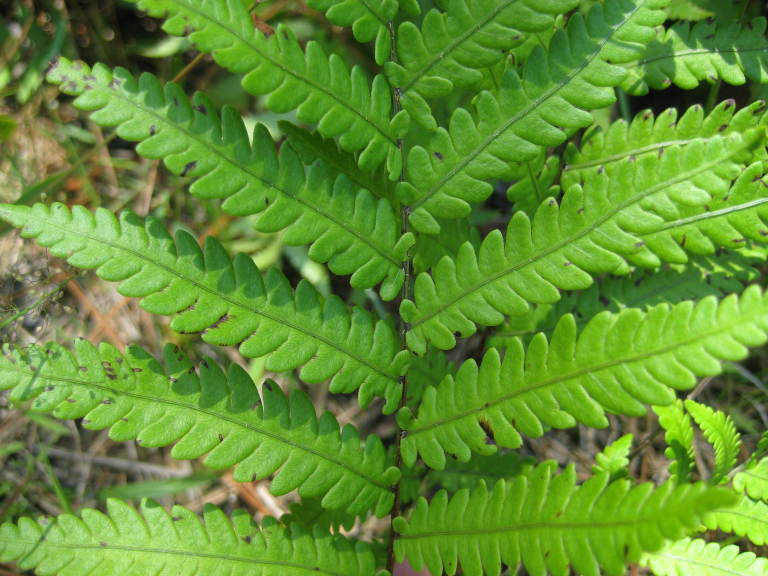
*Woodwardia virginica* (photo by R. Thornhill).

**Figure 16.**
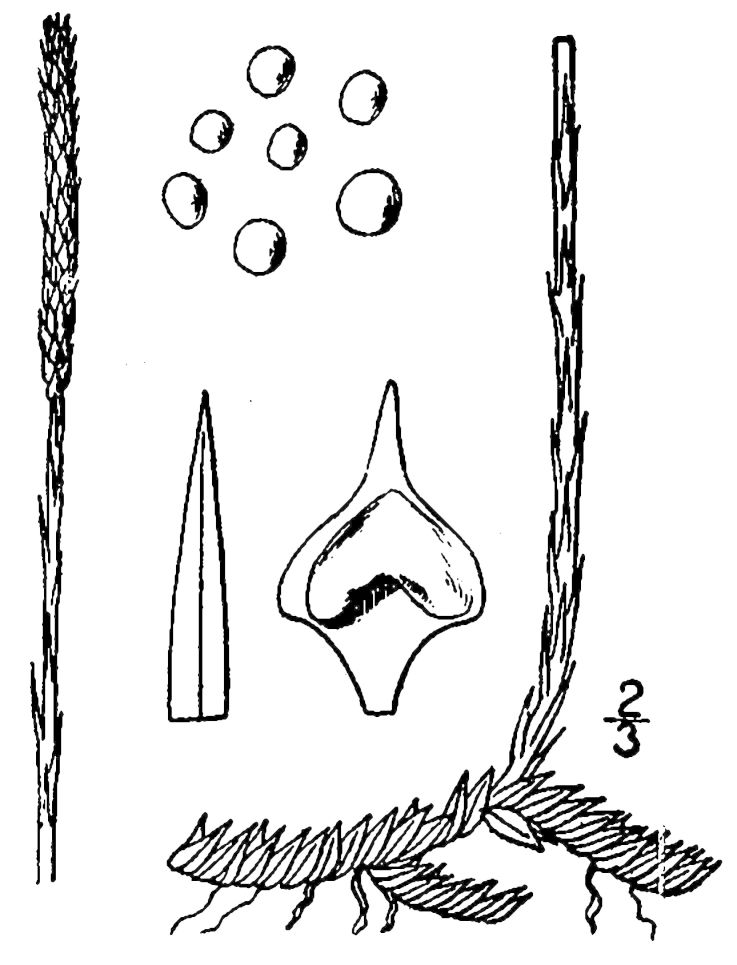
*Pseudolycopodiella caroliniana* (from Britton and Brown 1913).

**Figure 17a.**
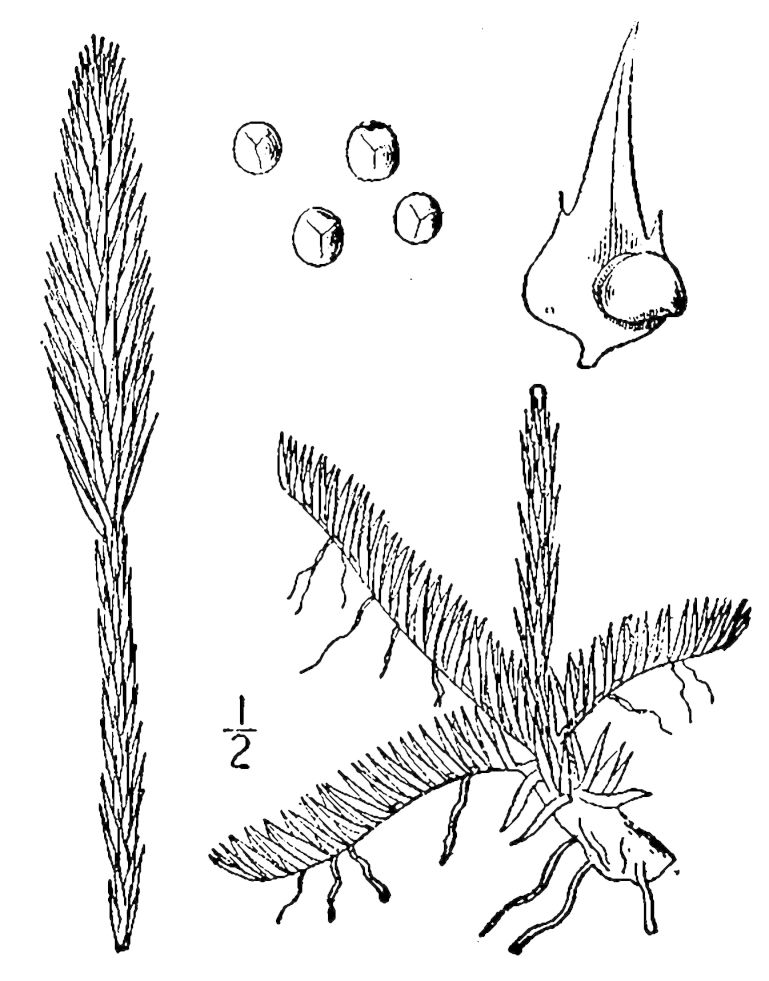
*Lycopodium alopecuroides* (from Britton and Brown 1913).

**Figure 17b.**
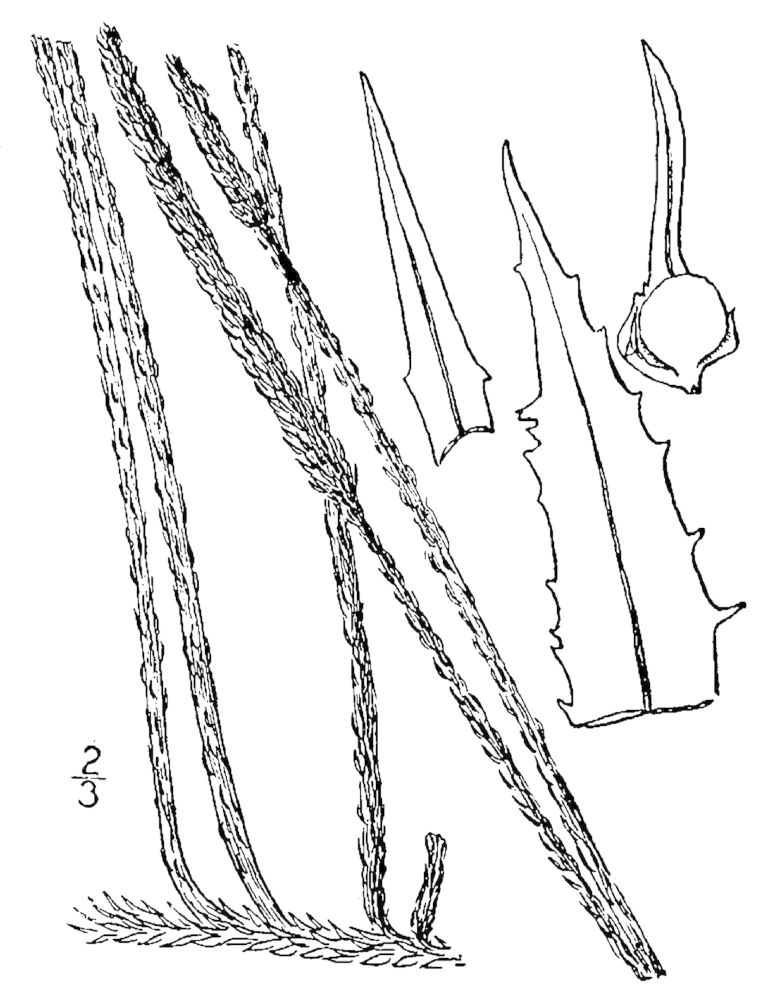
*Lycopodiella appressa* (from Britton and Brown 1913).

**Figure 18.**
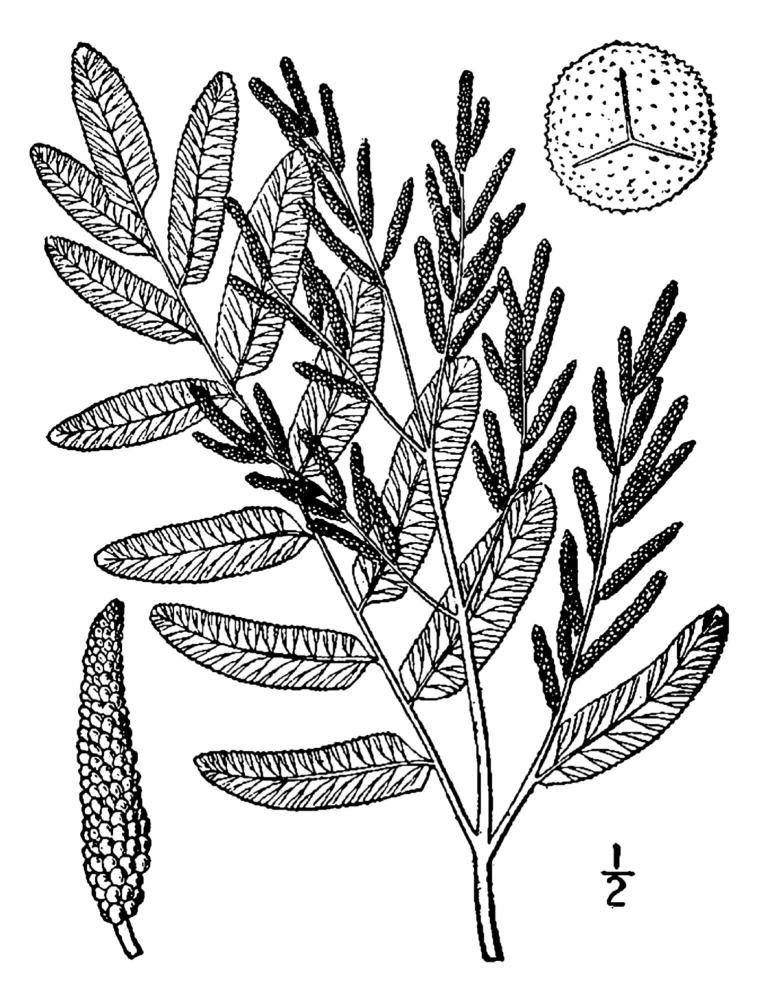
*Osmunda spectabilis* (from Britton and Brown 1913).

**Figure 19a.**
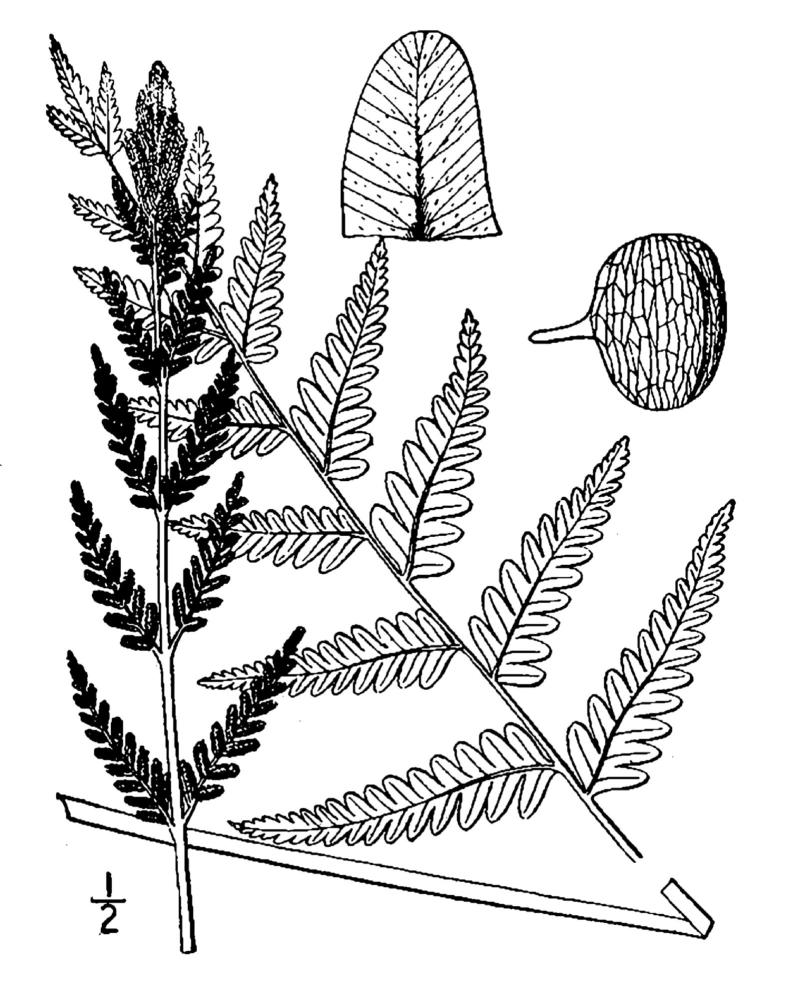
From Britton and Brown 1913.

**Figure 19b.**
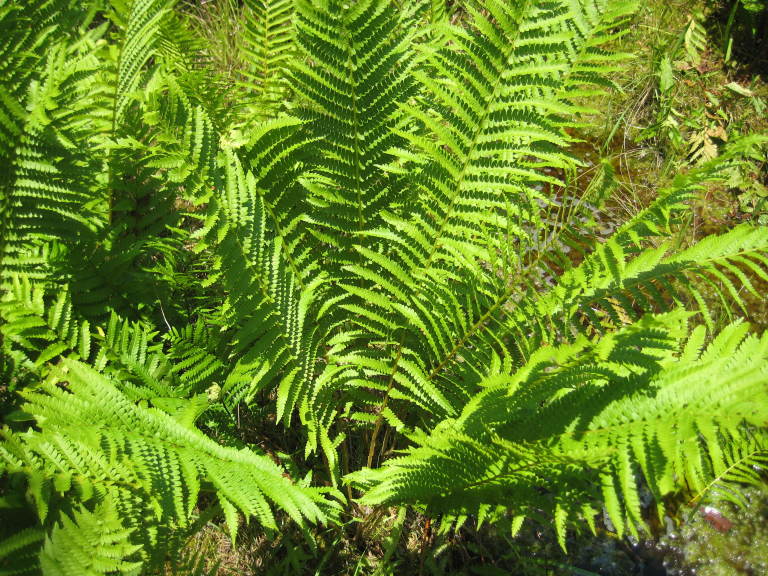
Photo by R. Thornhill.

**Figure 20.**
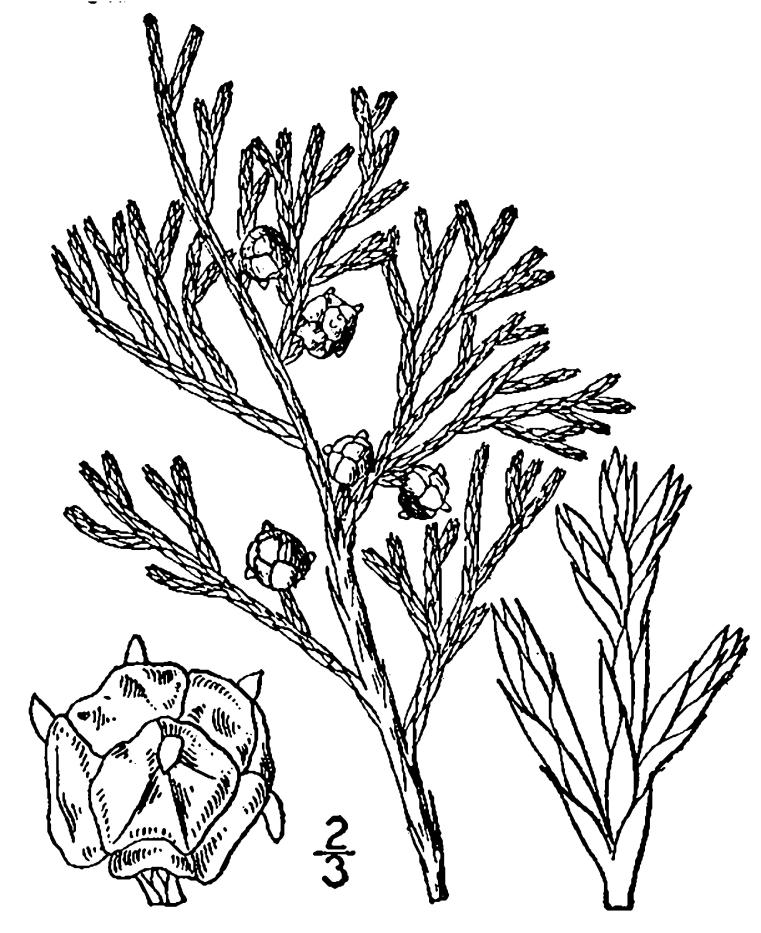
*Chamaecyparis thyoides* (from Britton and Brown 1913).

**Figure 21.**
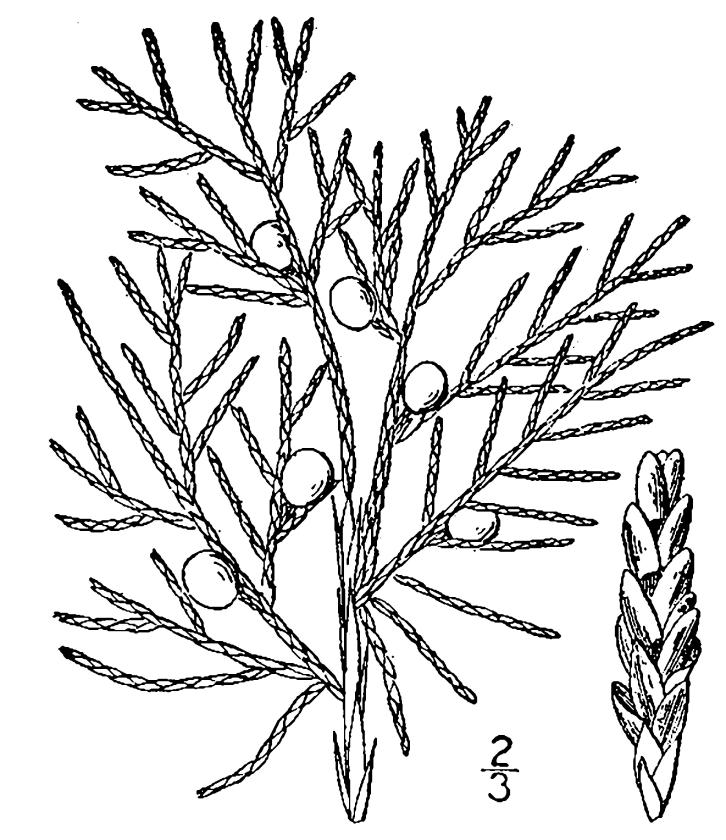
*Juniperus virginiana* (from Britton and Brown 1913).

**Figure 22a.**
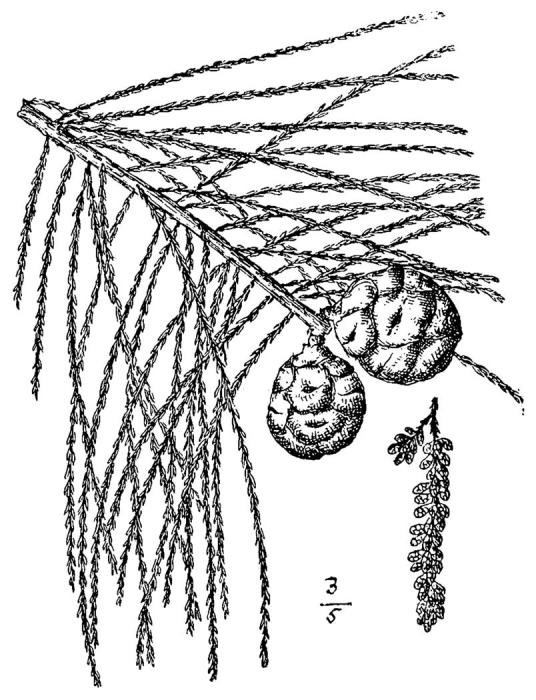
From Britton and Brown (1913).

**Figure 22b.**
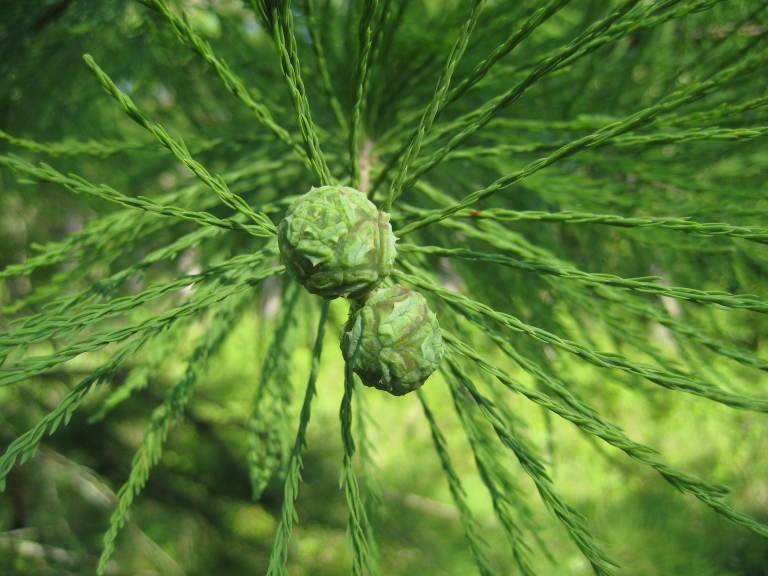
Photo by R. Thornhill

**Figure 23a.**
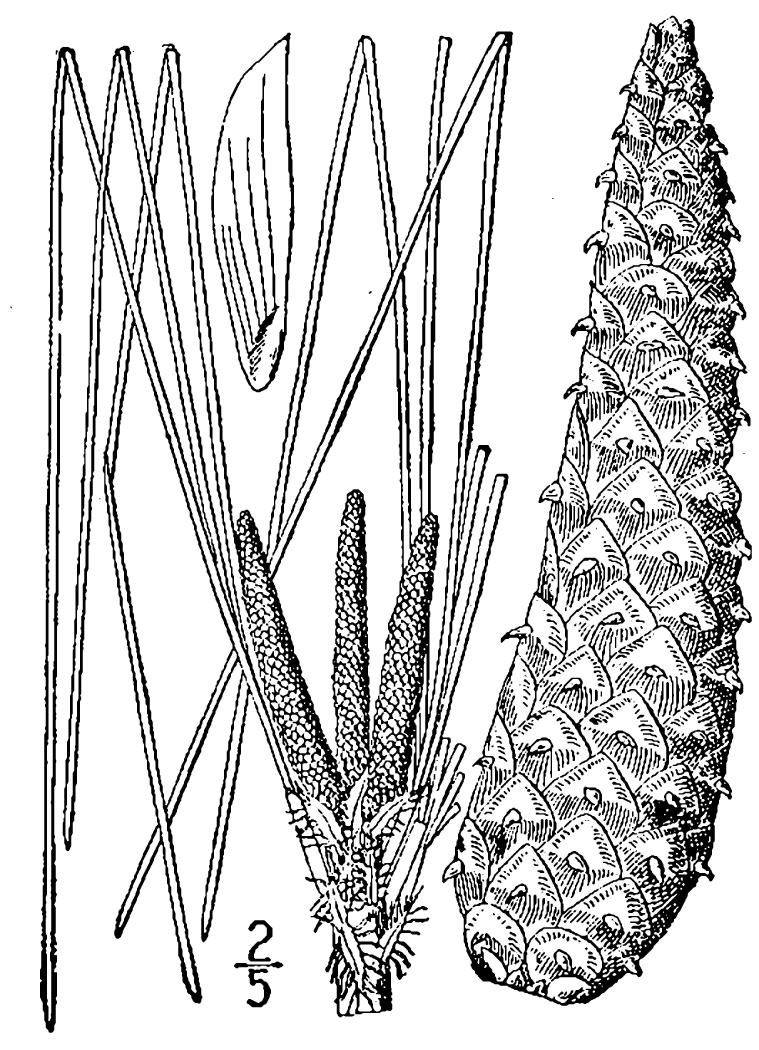
*Persea palustris* (from Britton and Brown 1913).

**Figure 23b.**
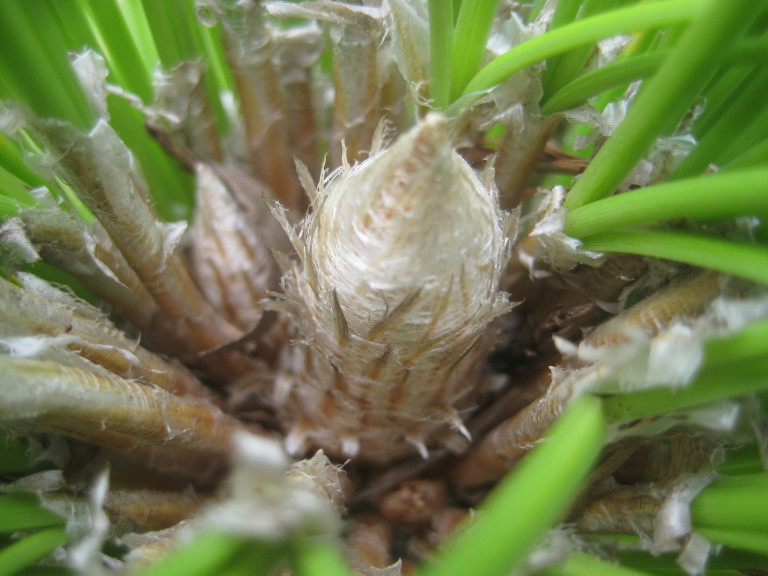
*Persea palustris*: terminal bud. Note the white, fimbriate scales. (Photo by R. Thornhill.)

**Figure 23c.**
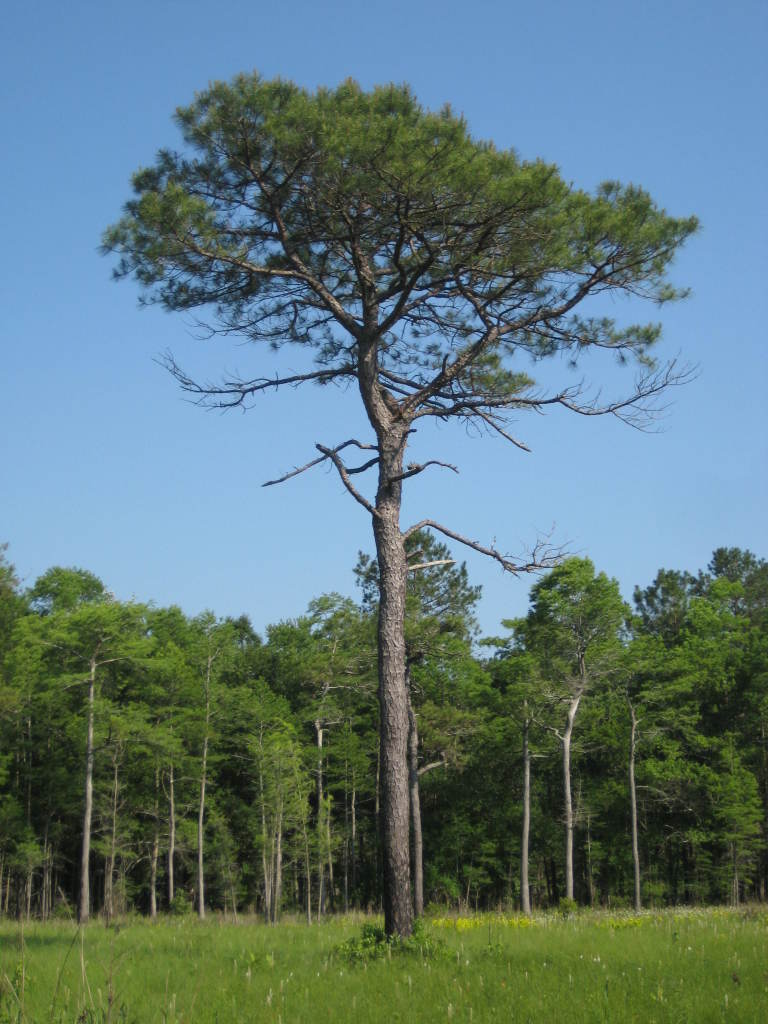
*Pinus serotina* (photo by R. Thornhill).

**Figure 23d.**
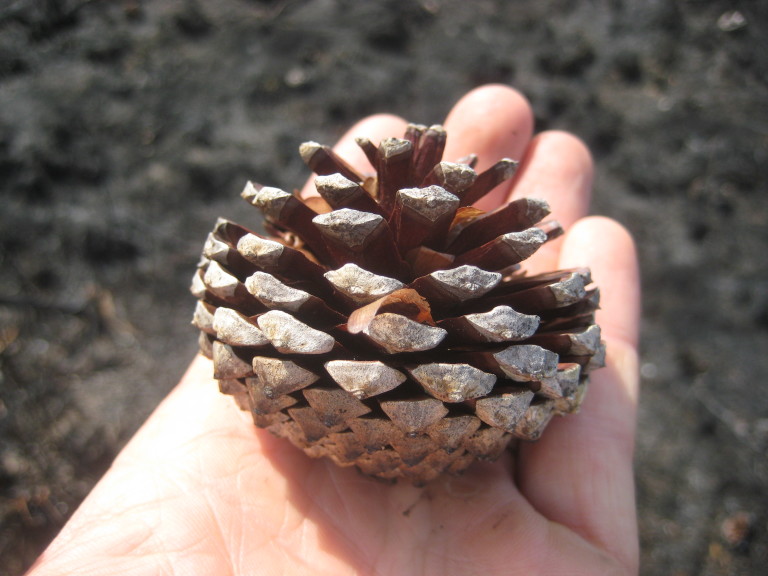
*Pinus serotina*: female cone (photo by R. Thornhill).

**Figure 23e.**
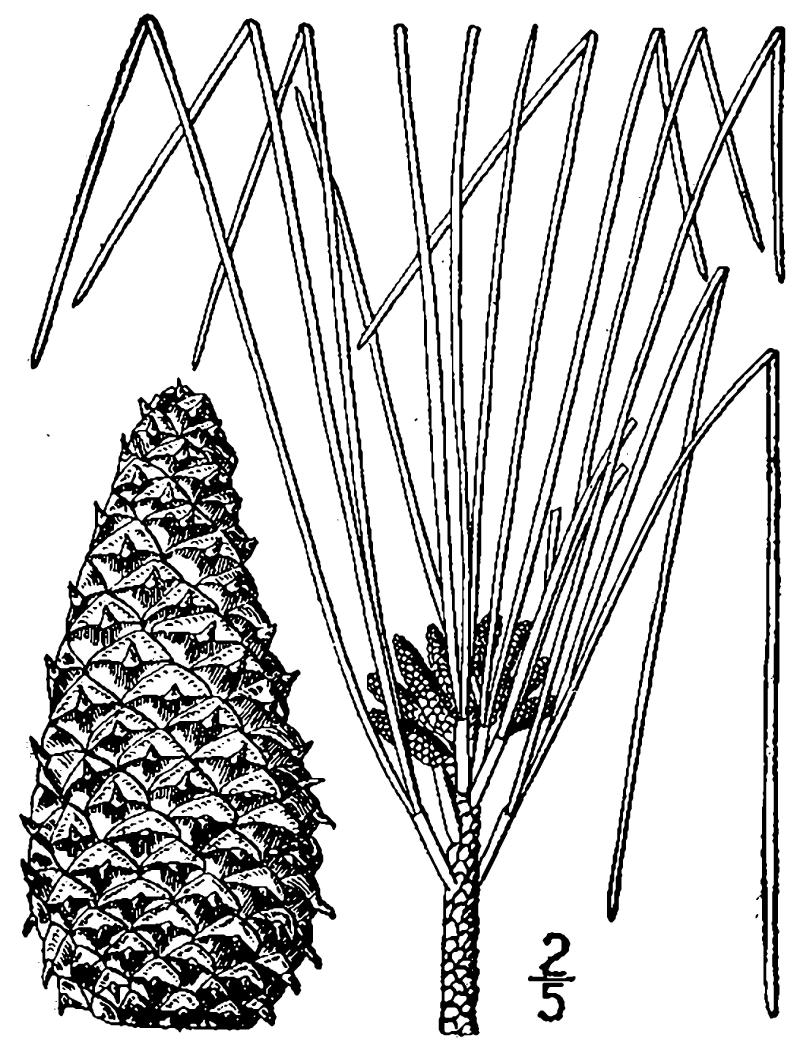
*Pinus taeda* (from Britton and Brown 1913).

**Figure 24.**
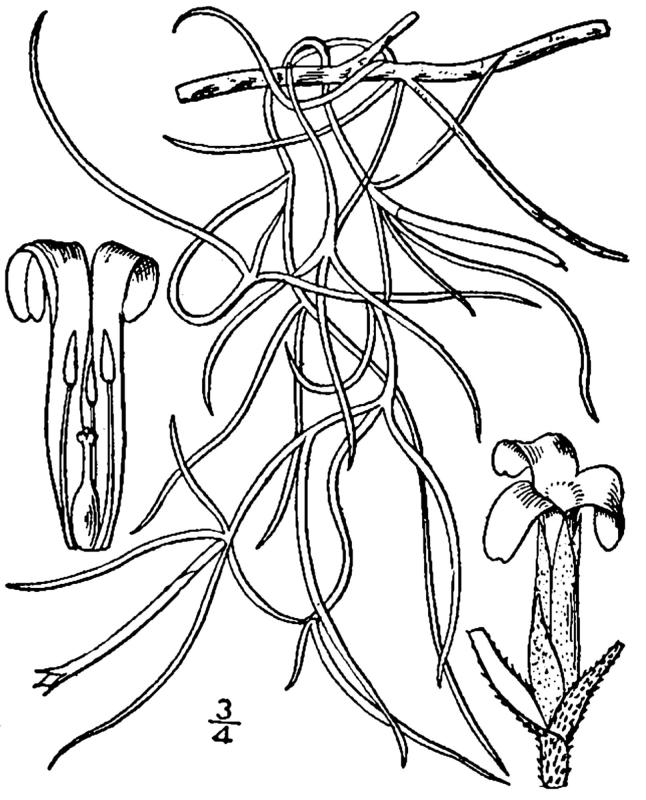
*Tillandsia usneoides* (from Britton and Brown 1913).

**Figure 25a.**
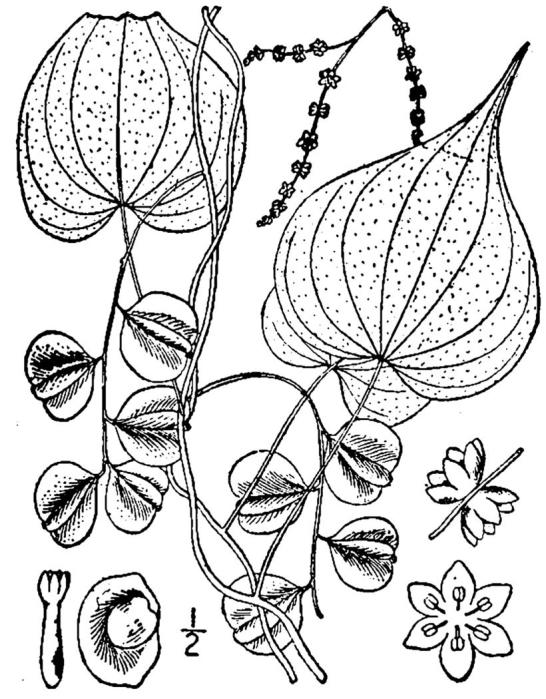
From Britton and Brown 1913.

**Figure 25b.**
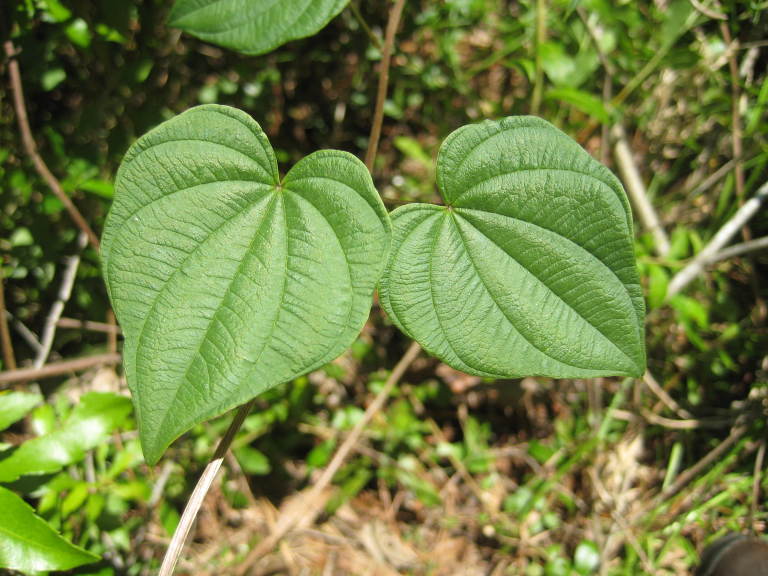
Photo by R. Thornhill.

**Figure 26a.**
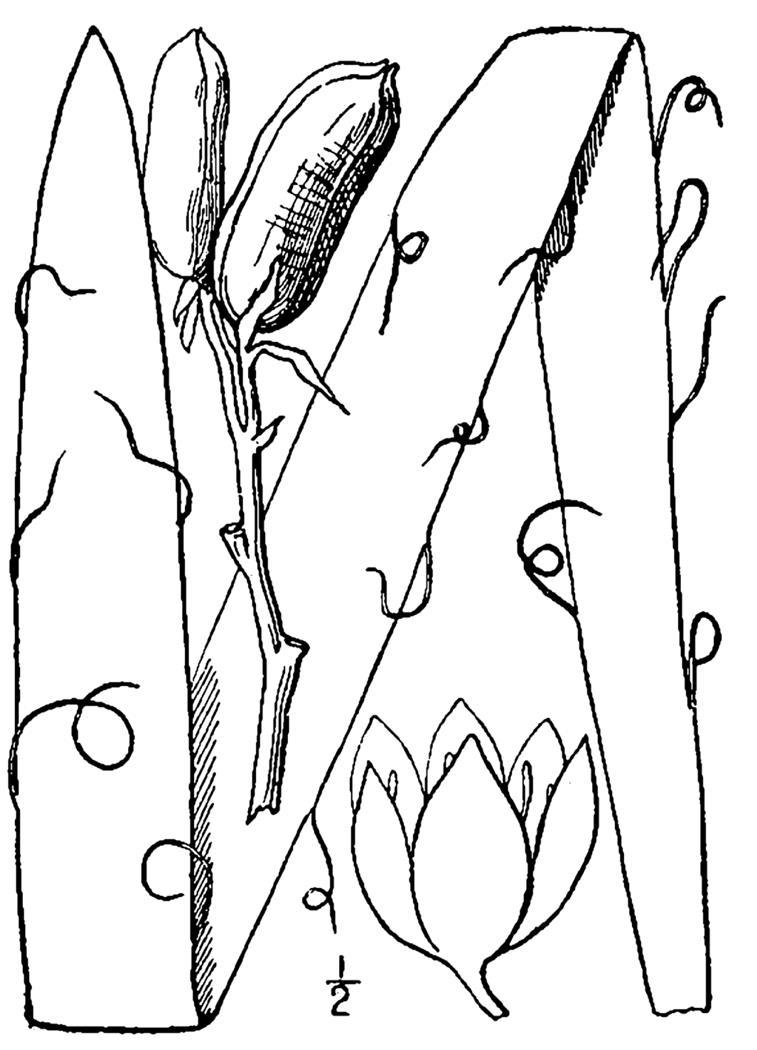
From Britton and Brown (1913).

**Figure 26b.**
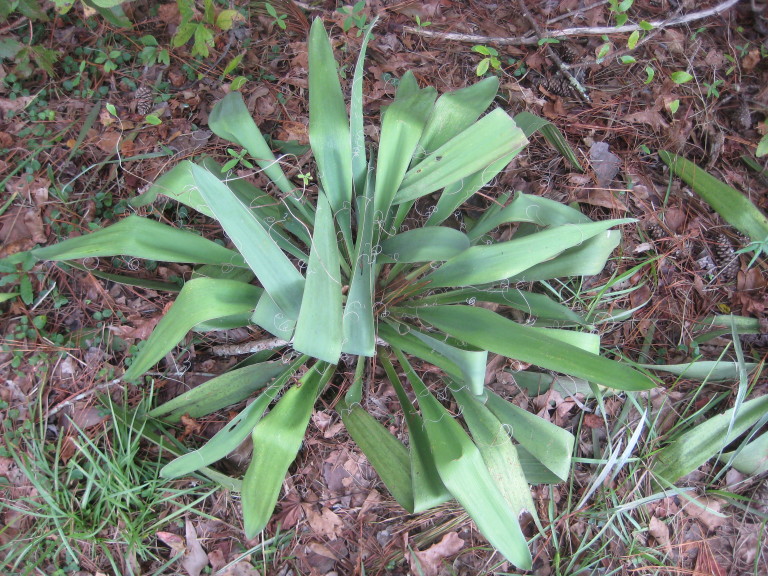
Photo by R. Thornhill.

**Figure 27.**
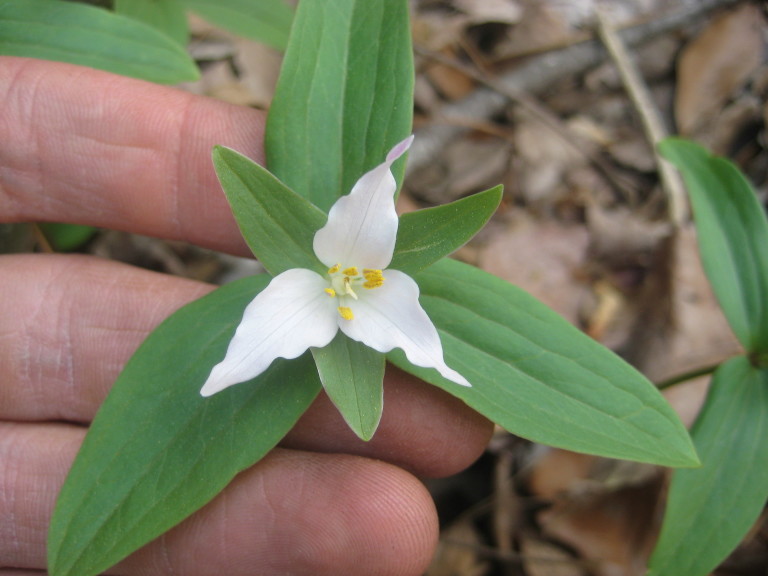
Trillium pusillum var. pusillum (photo by R. Thornhill).

**Figure 28a.**
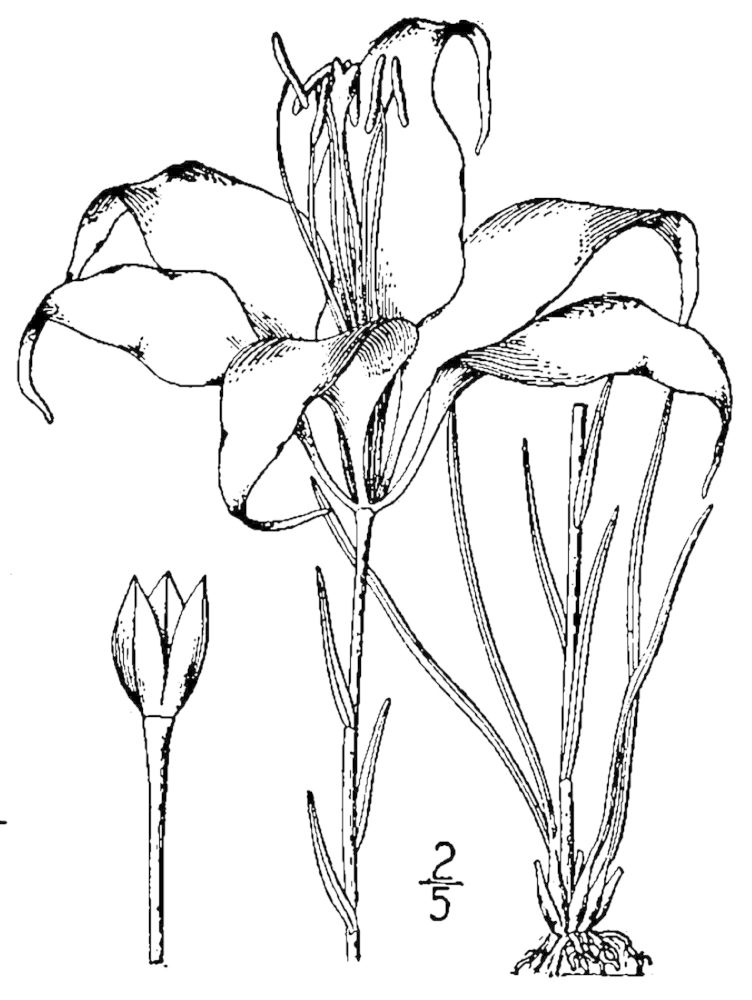
From Britton and Brown (1913).

**Figure 28b.**
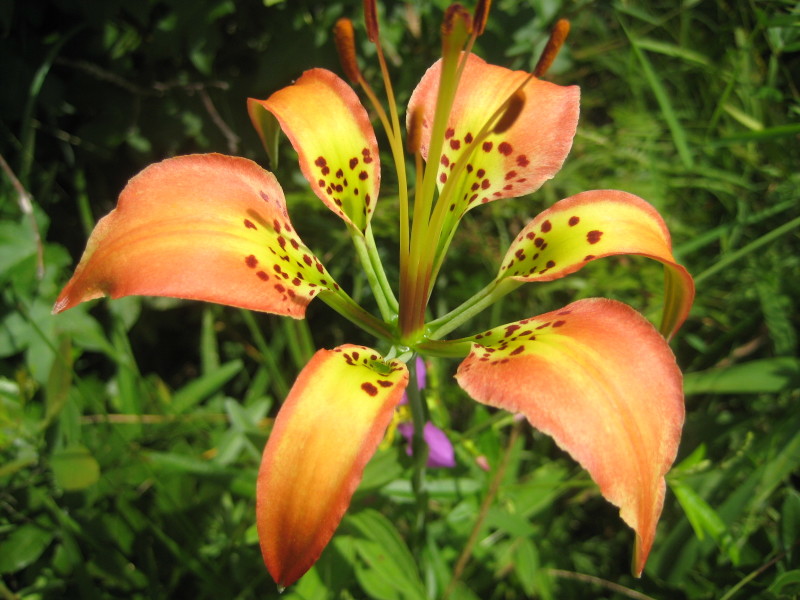
Photo by R. Thornhill.

**Figure 29.**
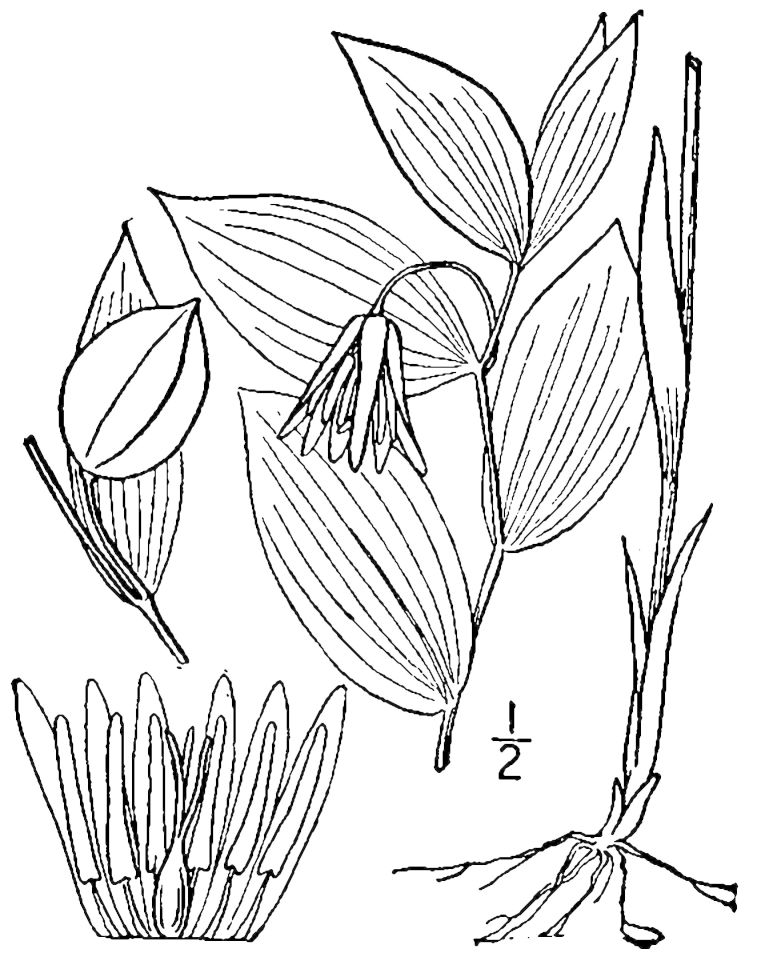
*Uvularia puberula* (from Britton and Brown 1913).

**Figure 30.**
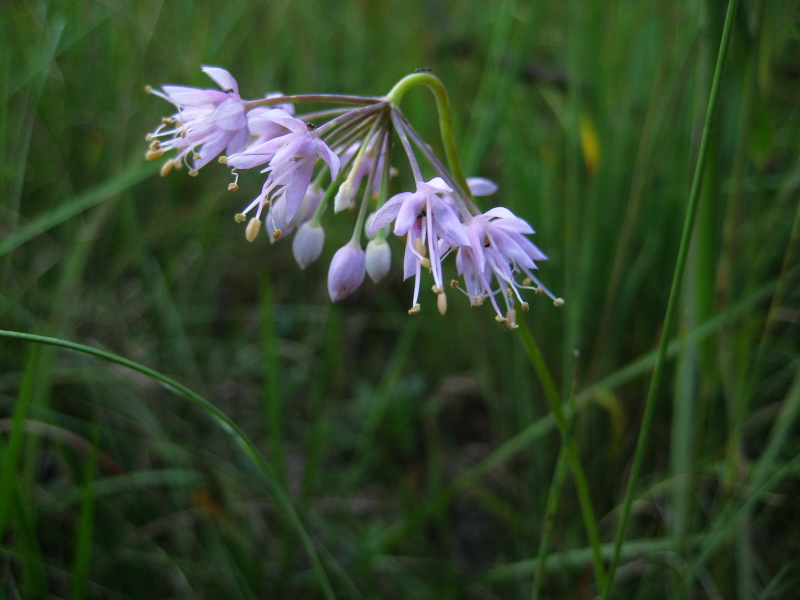
*Allium* species 1

**Figure 31.**
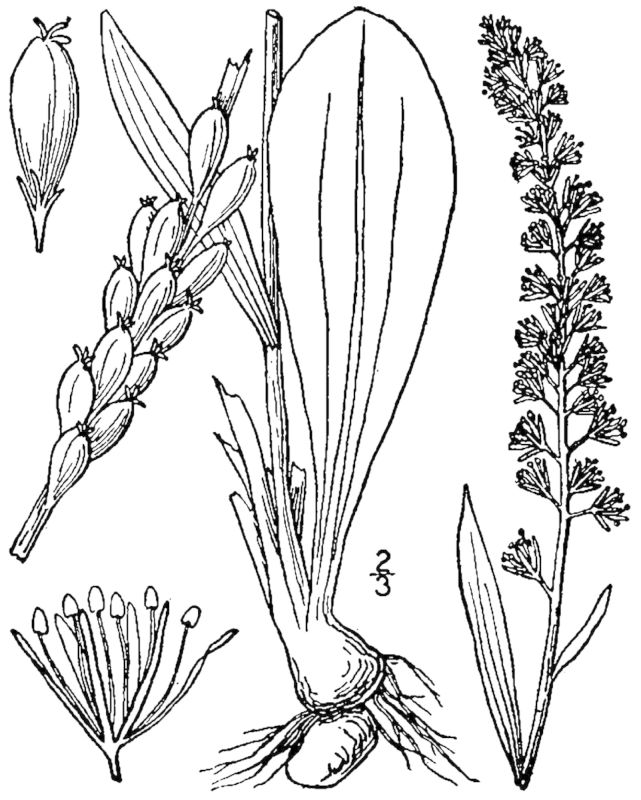
*Chamaelirium luteum* (from Britton and Brown 1913).

**Figure 32a.**
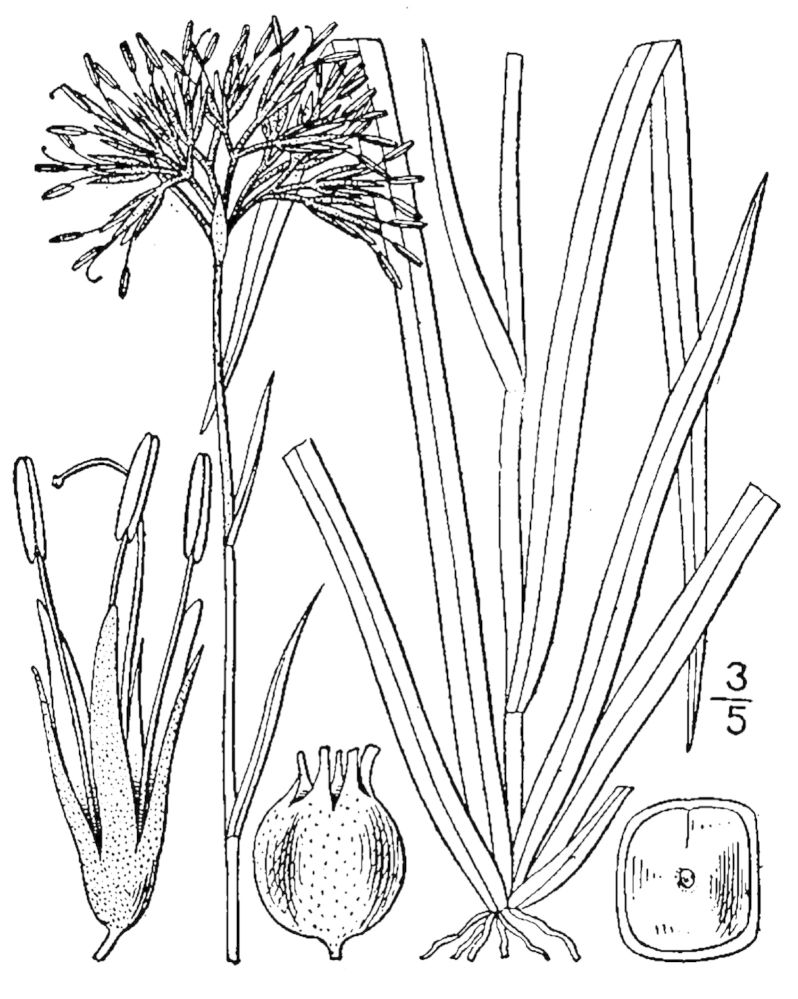
From Britton and Brown 1913.

**Figure 32b.**
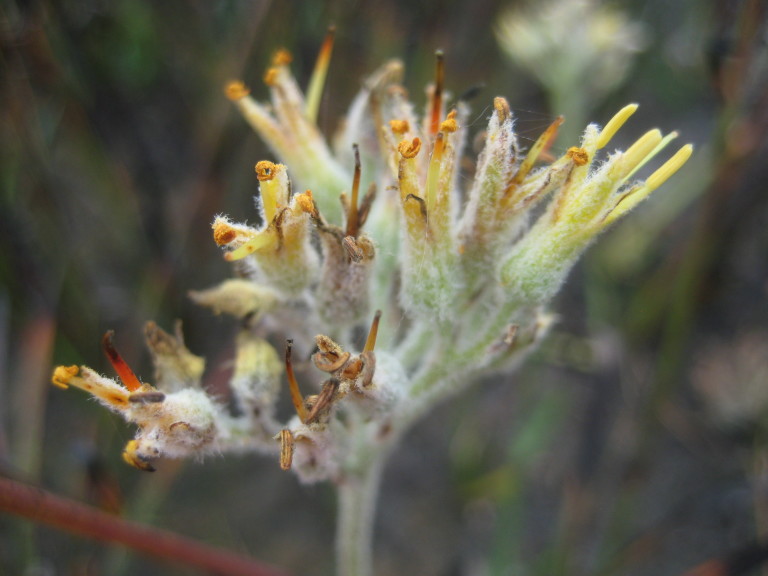
Photo by R. Thornhill.

**Figure 33.**
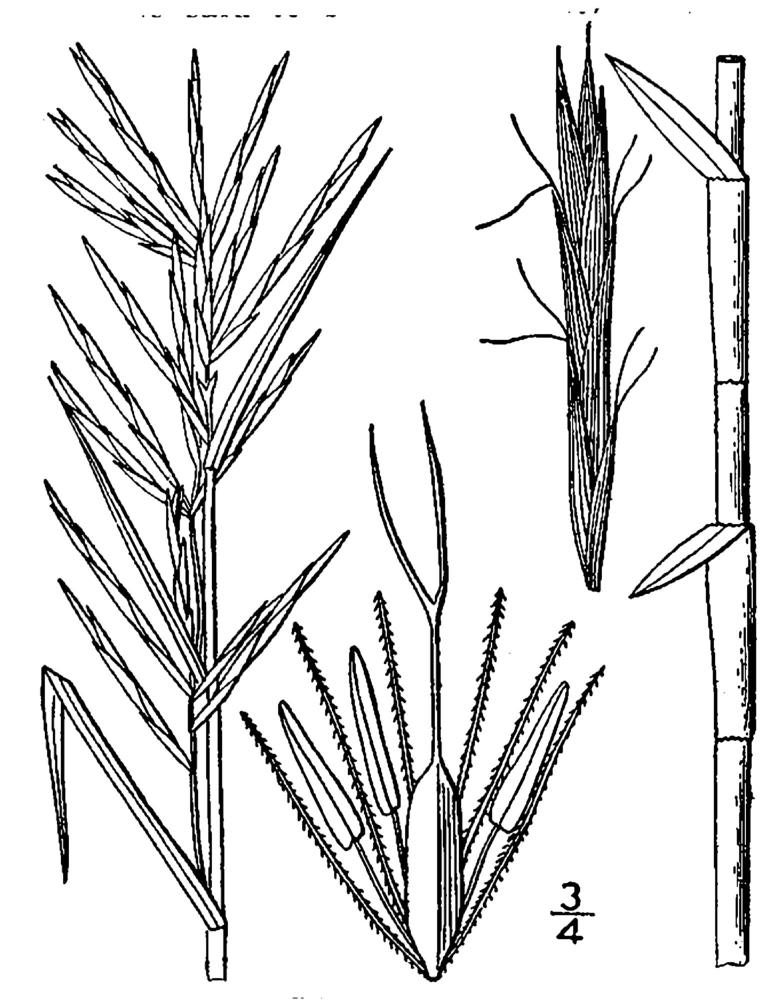
*Dulichium arundinaceum* (from Britton and Brown 1913).

**Figure 34.**
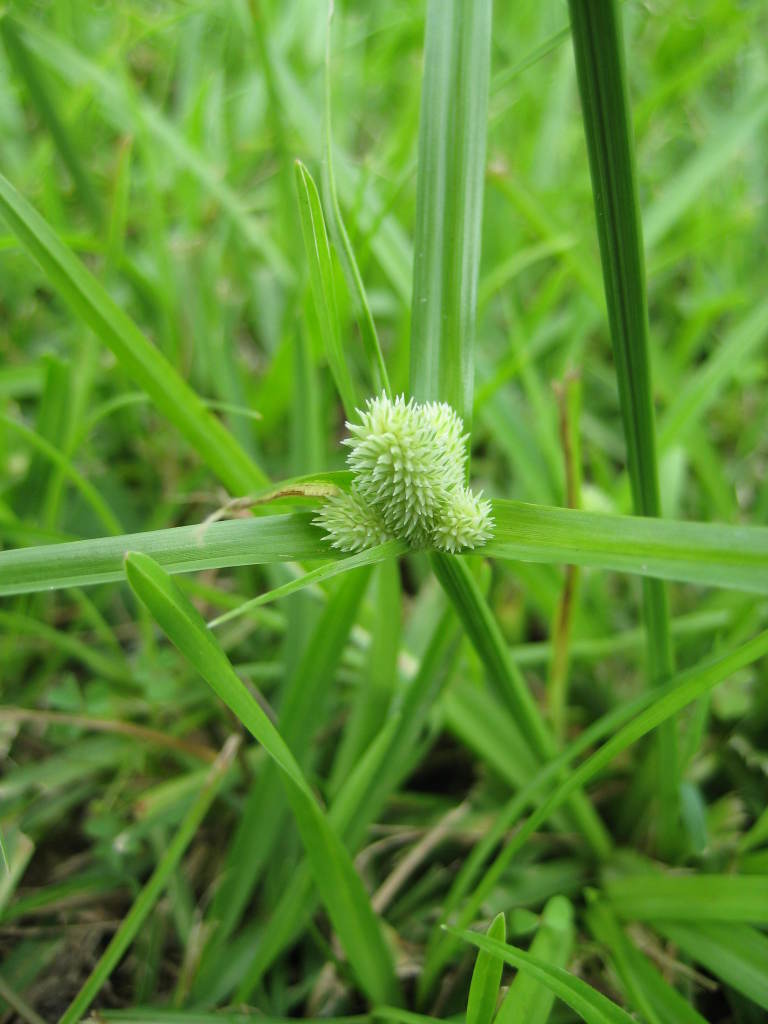
*Kyllinga odorata* (photo by R. Thornhill).

**Figure 35.**
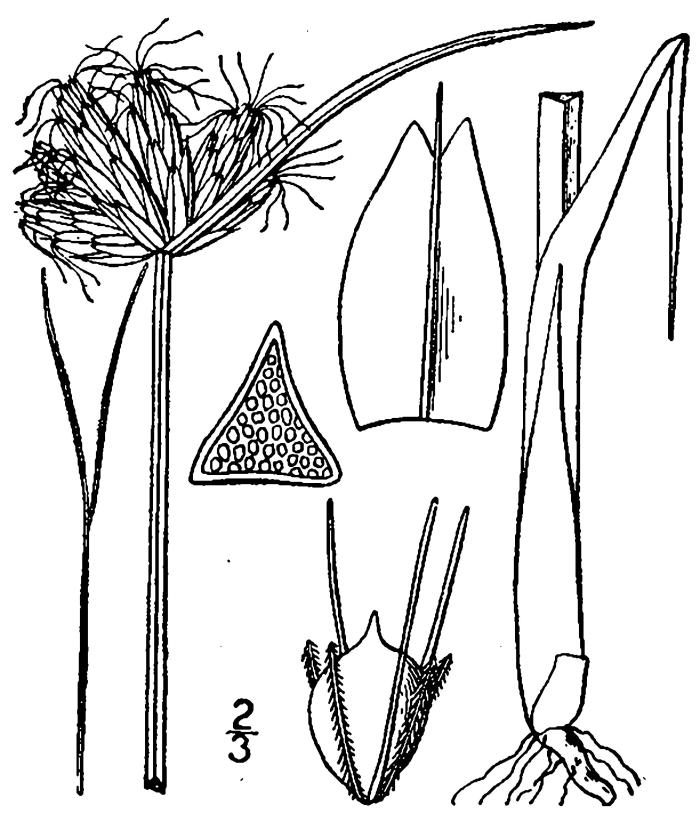
*Schoenoplectus pungens* (from Britton and Brown 1913).

**Figure 36a.**
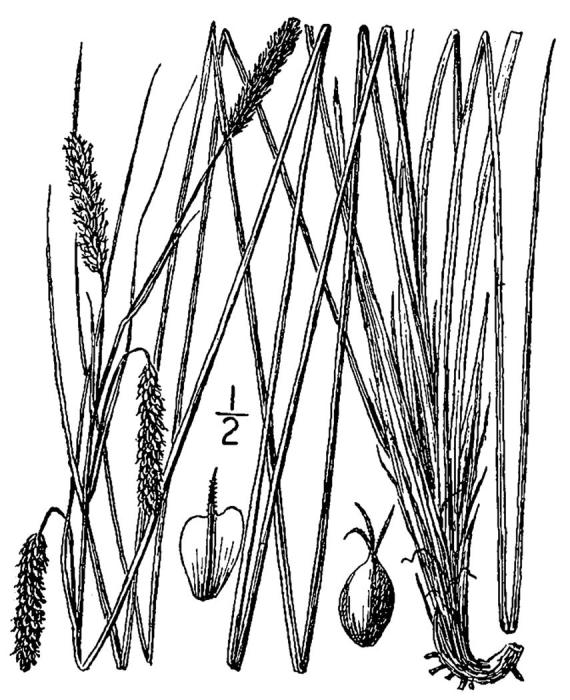
*Carex glaucescens* (from Britton and Brown 1913).

**Figure 36b.**
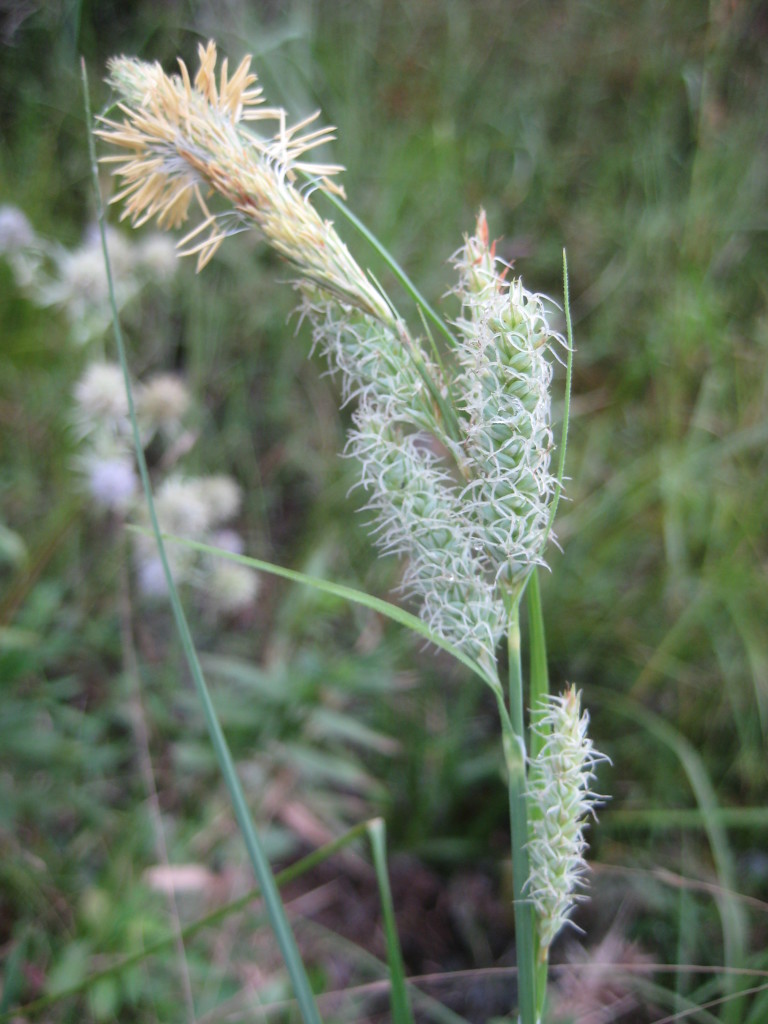
*Carex glaucescens*: flowering spikes (photo by R. Thornhill).

**Figure 36c.**
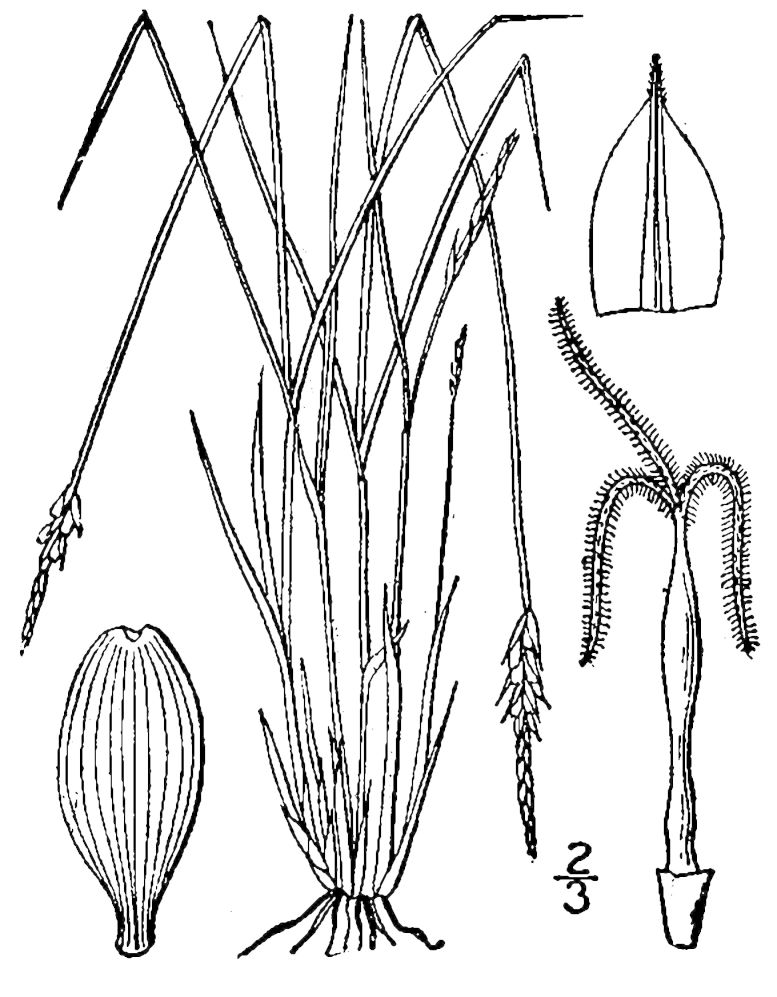
*Carex leptalea* (from Britton and Brown 1913).

**Figure 36d.**
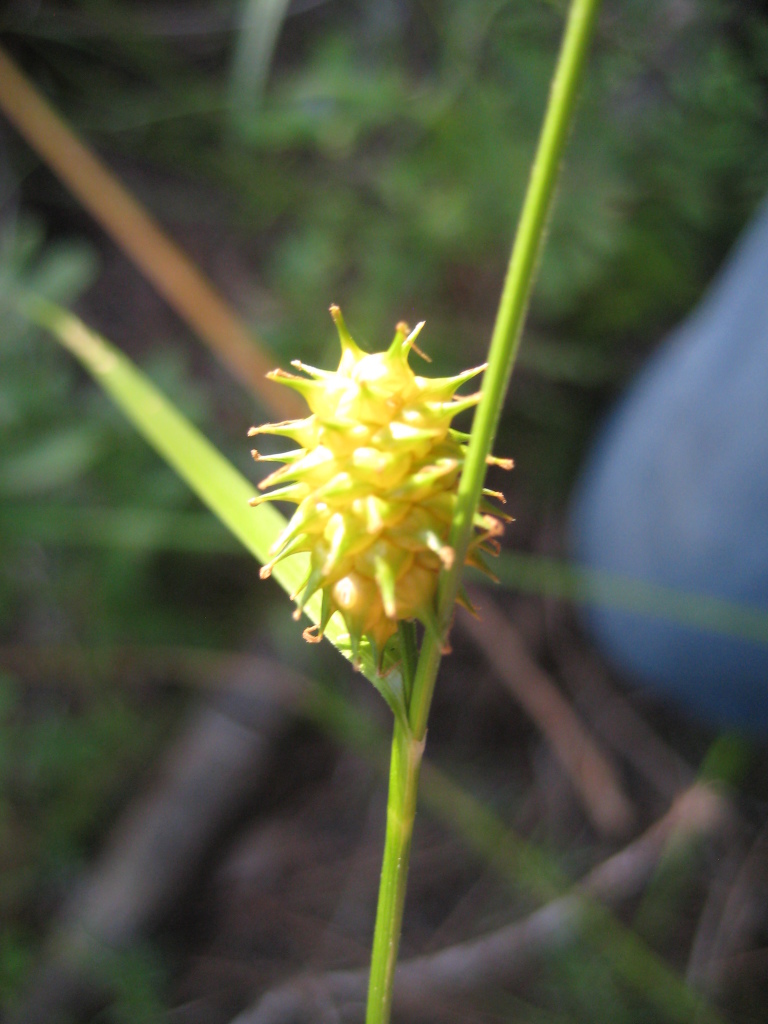
*Carex lutea*: mature female spike. Note distinctive deflexed basal perigynia. (Photo by R. Thornhill.)

**Figure 36e.**
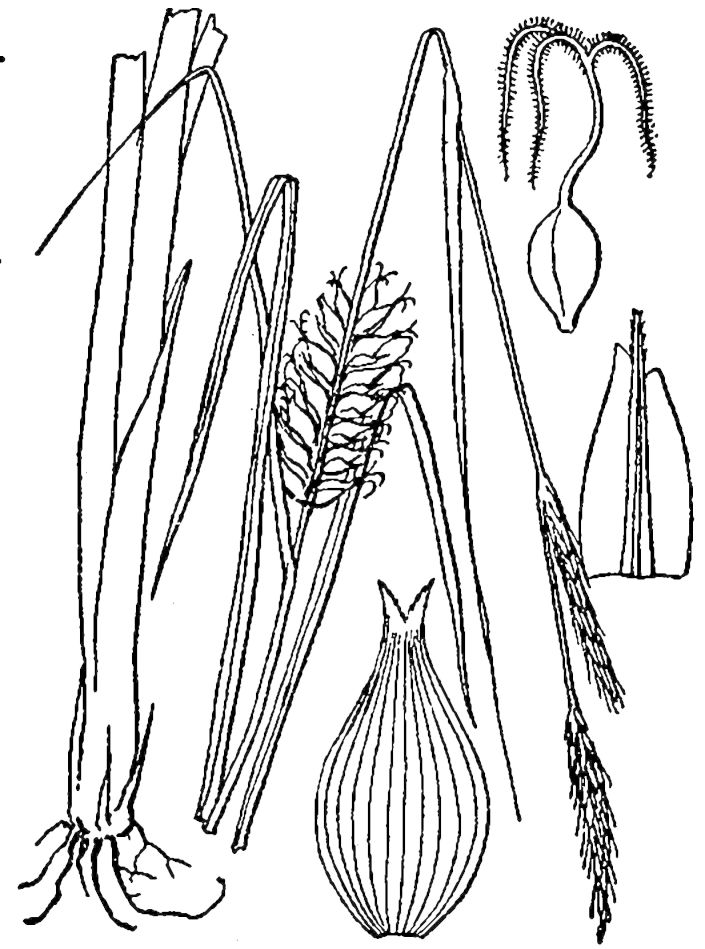
*Carex striata* (from Britton and Brown 1913).

**Figure 36f.**
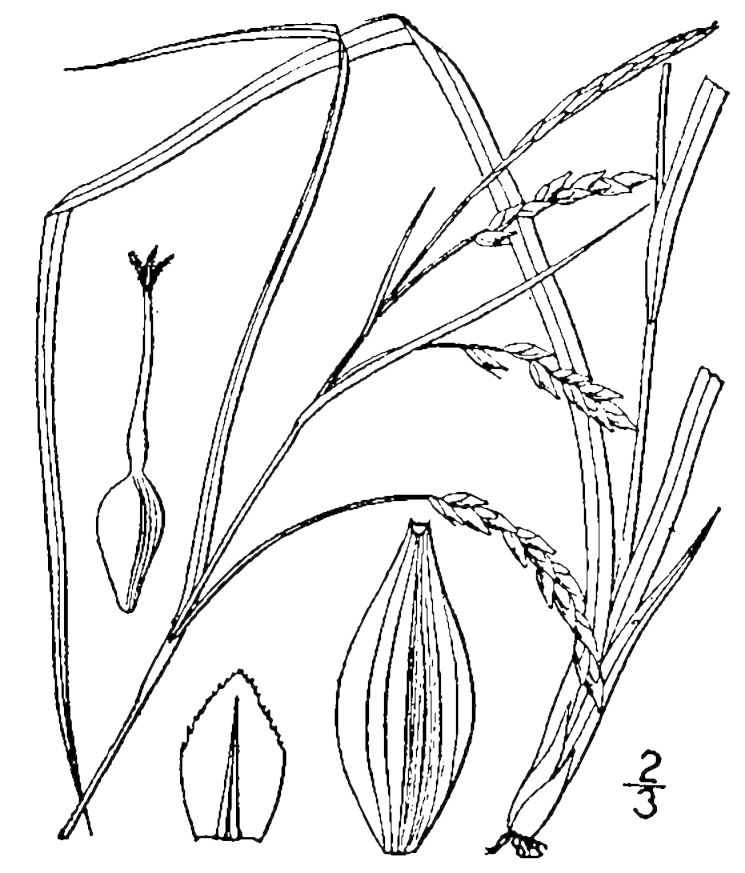
*Carex venusta* (from Britton and Brown 1913).

**Figure 37a.**
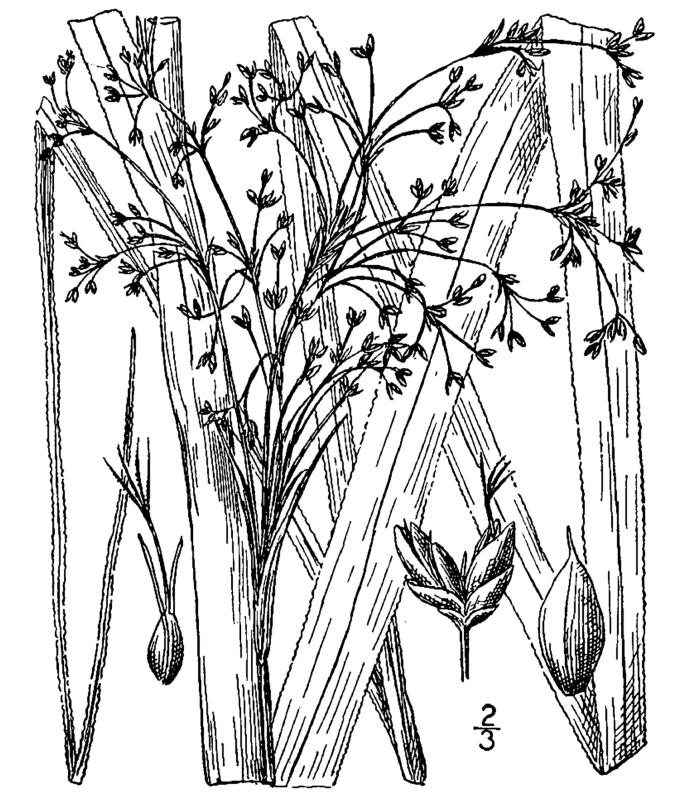
*Cladium jamaicense* (from Britton and Brown 1913).

**Figure 37b.**
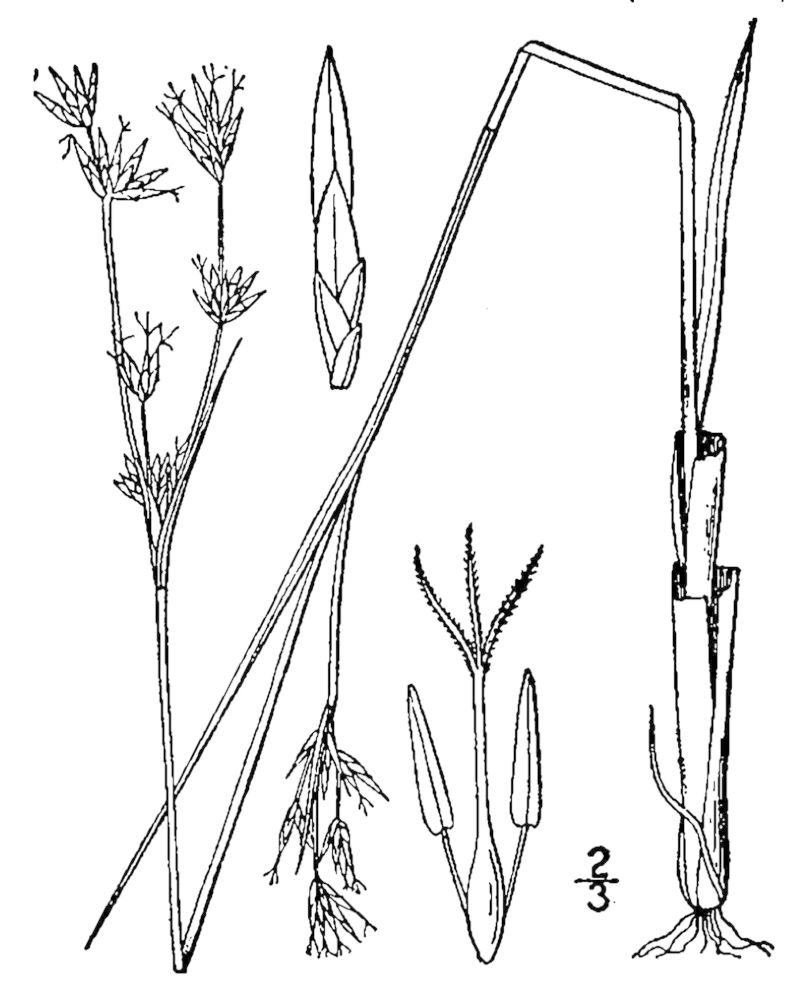
*Cladium mariscoides* (from Britton and Brown 1913).

**Figure 38a.**
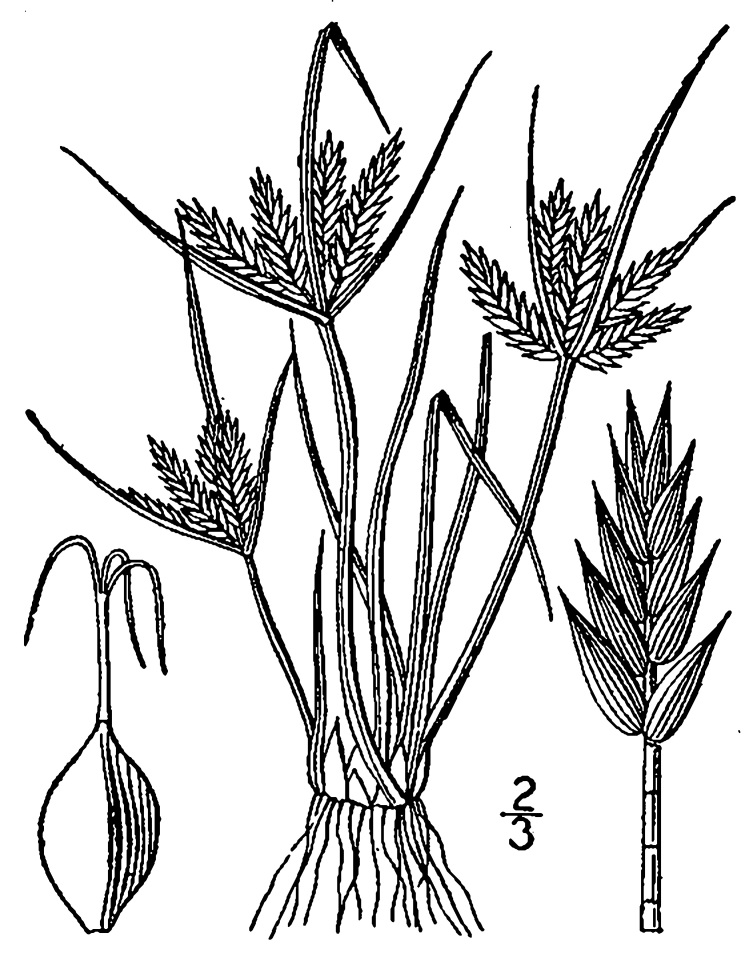
*Cyperus compressus* (from Britton and Brown 1913)

**Figure 38b.**
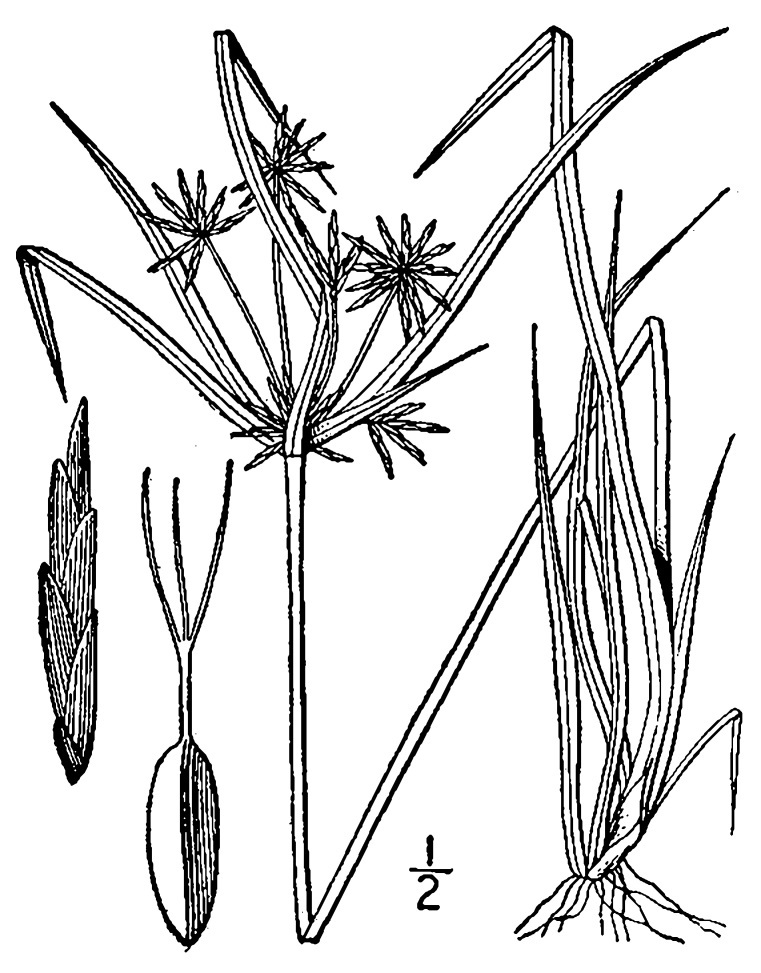
*Cyperus croceus* (from Britton and Brown 1913)

**Figure 38c.**
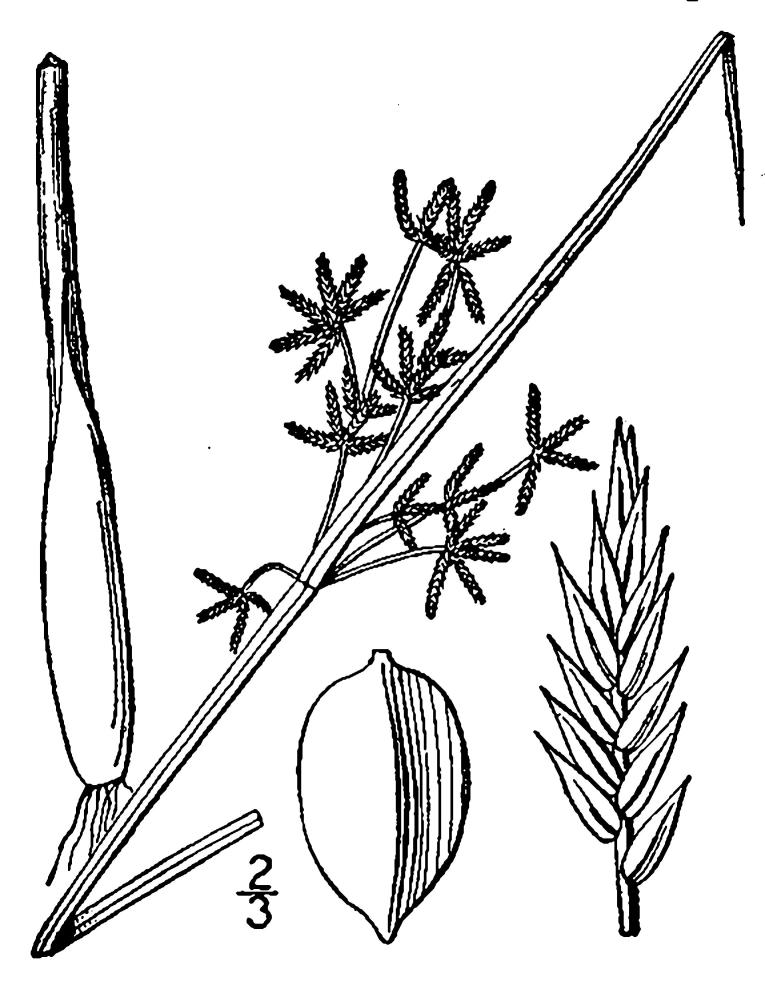
*Cyperus haspan* (from Britton and Brown 1913)

**Figure 38d.**
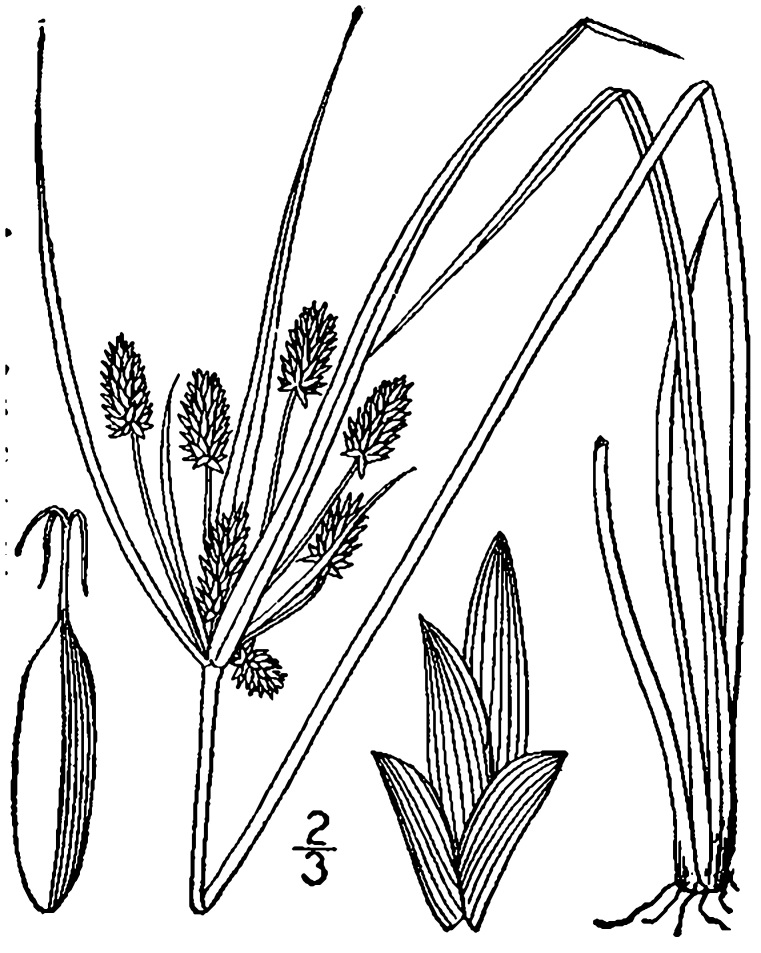
*Cyperus retrorsus* (from Britton and Brown 1913)

**Figure 39a.**
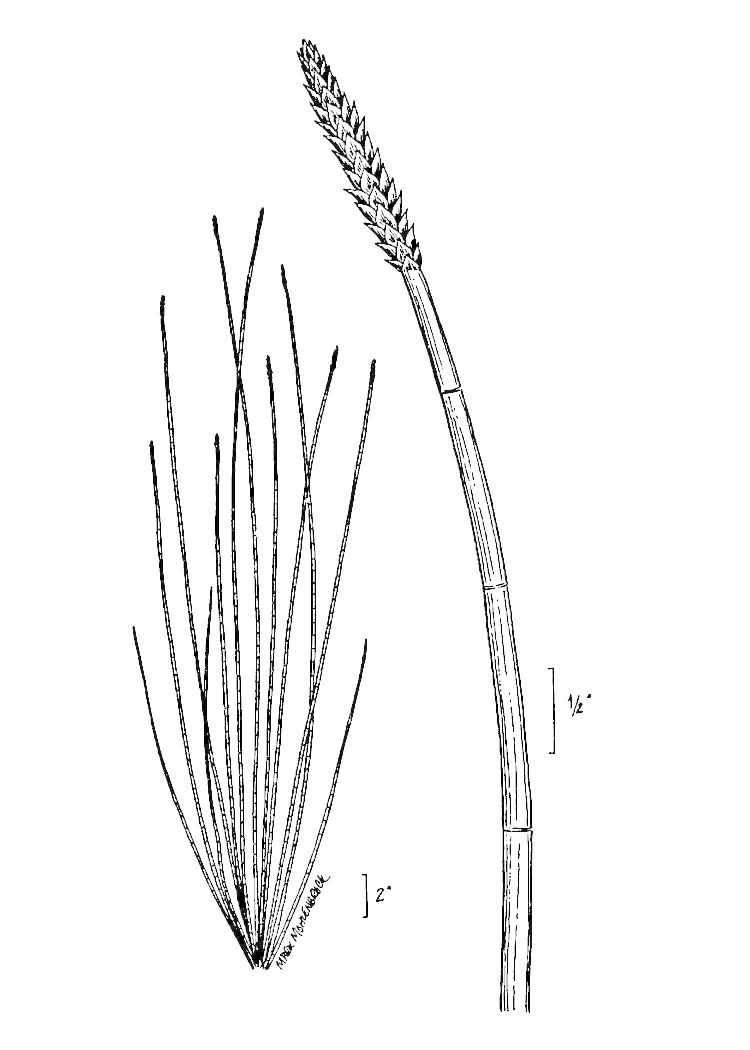
*Eleocharis equisetoides* (from USDA-NRCS 2012).

**Figure 39b.**
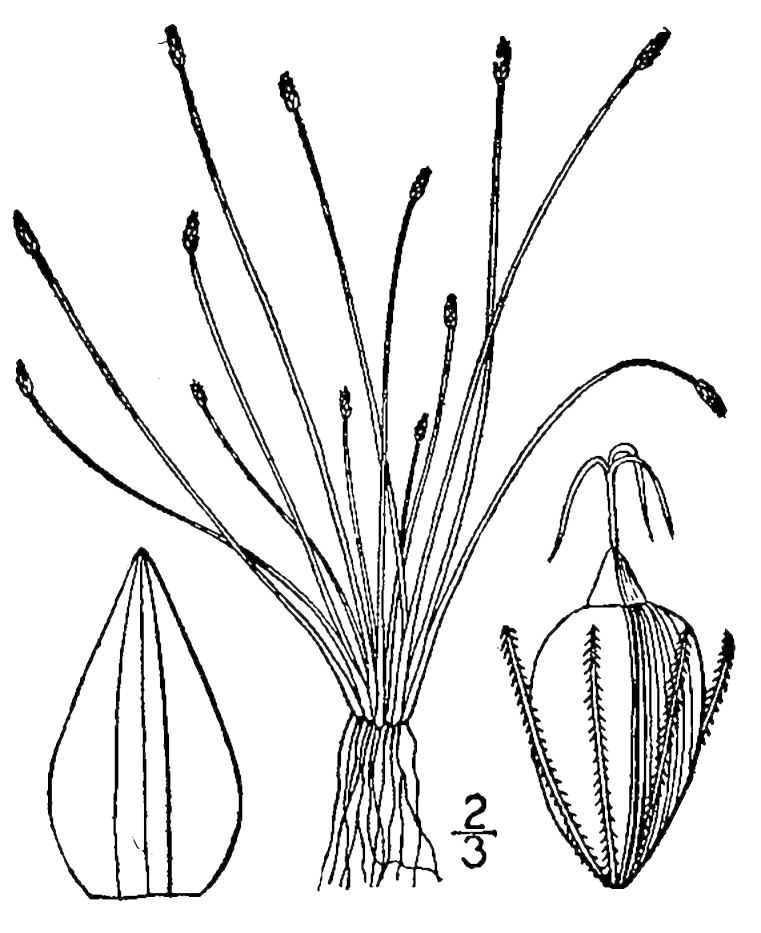
*Eleocharis microcarpa* (from Britton and Brown 1913).

**Figure 39c.**
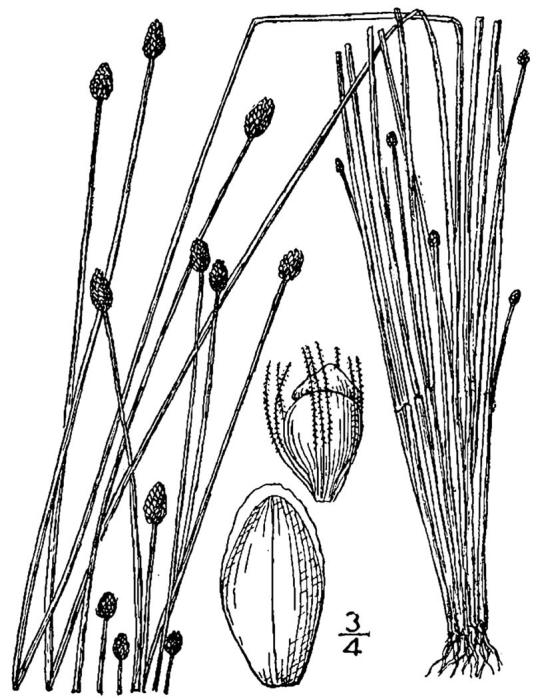
*Eleocharis obtusa* (from Britton and Brown 1913).

**Figure 39d.**
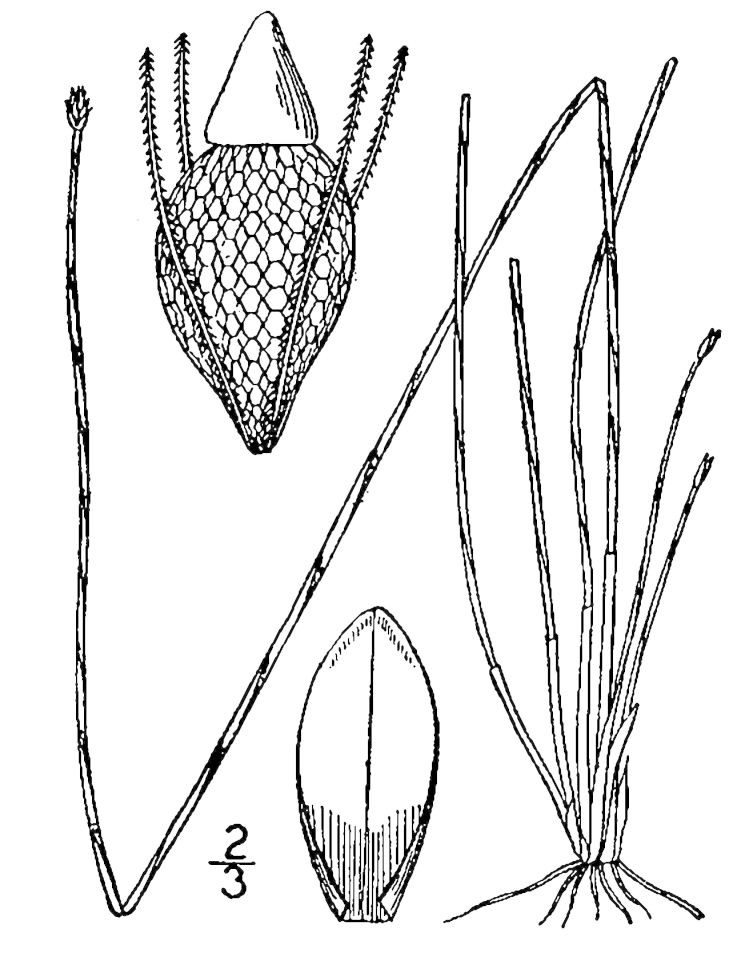
*Eleocharis tuberculosa* (from Britton and Brown 1913).

**Figure 40.**
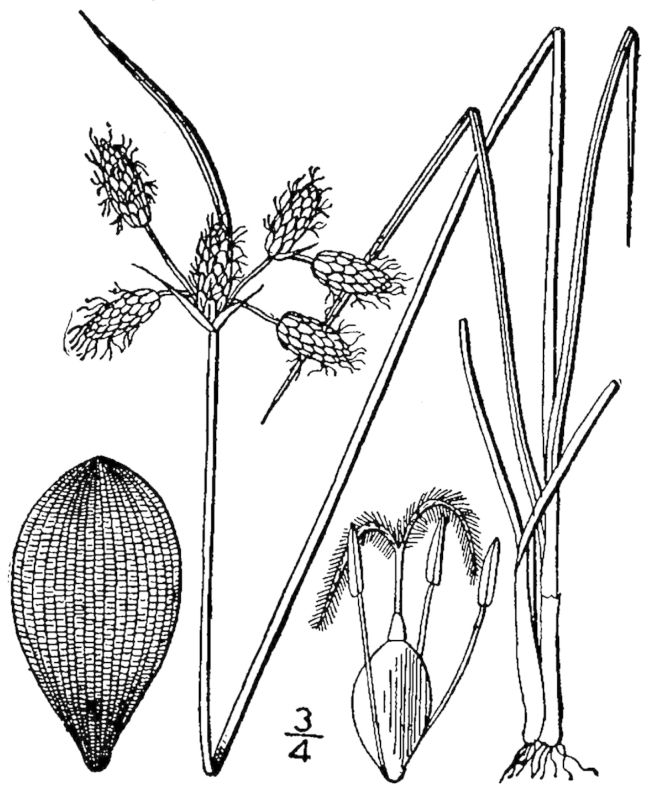
*Fimbristylis puberula* (from Britton and Brown 1913).

**Figure 41a.**
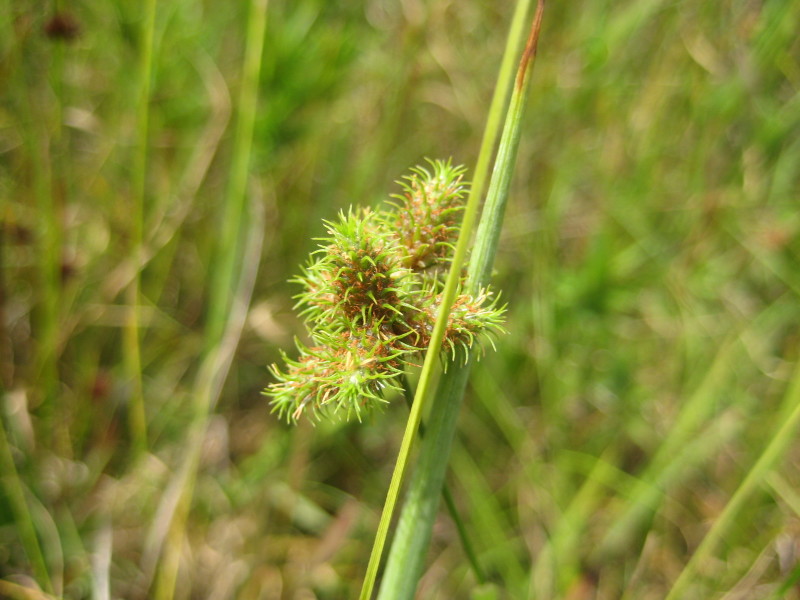
*Fuirena breviseta* (photo by R. Thornhill).

**Figure 41b.**
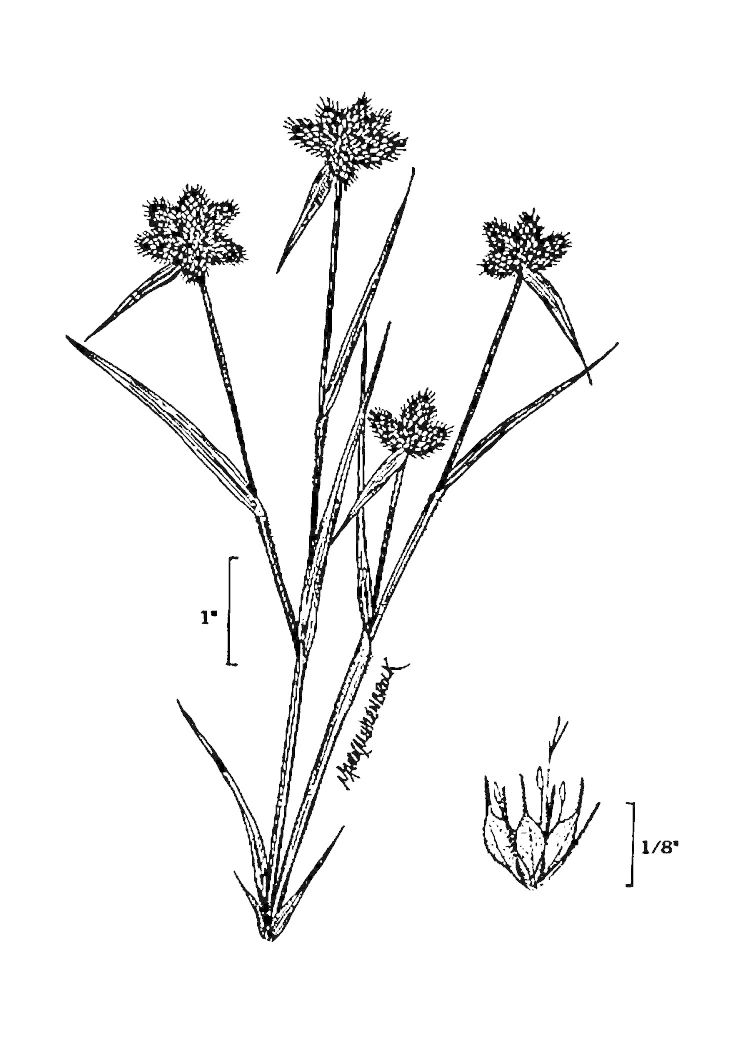
*Fuirena pumila* (from USDA-NRCS 2012).

**Figure 42a.**
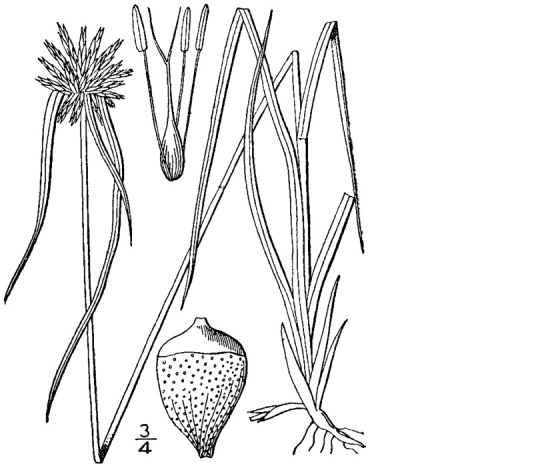
*Rhynchospora colorata* (from Britton and Brown 1913).

**Figure 42b.**
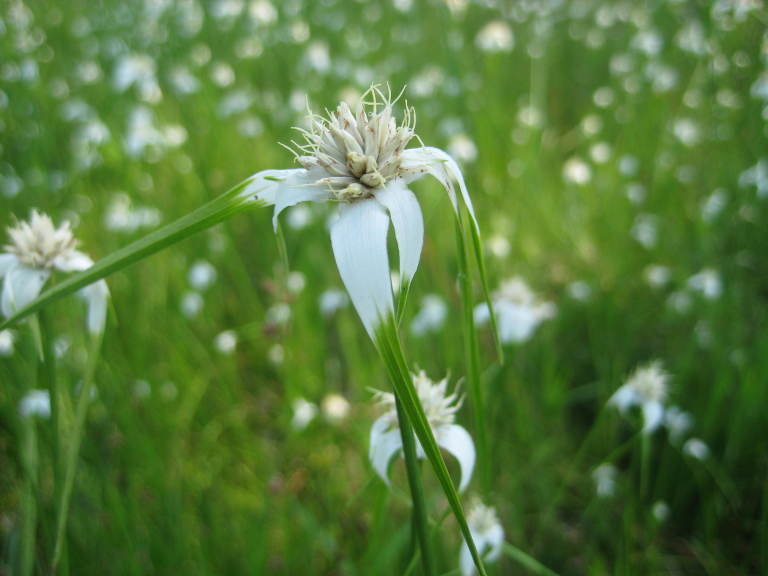
*Rhynchospora colorata* (photo by R. Thornhill).

**Figure 42c.**
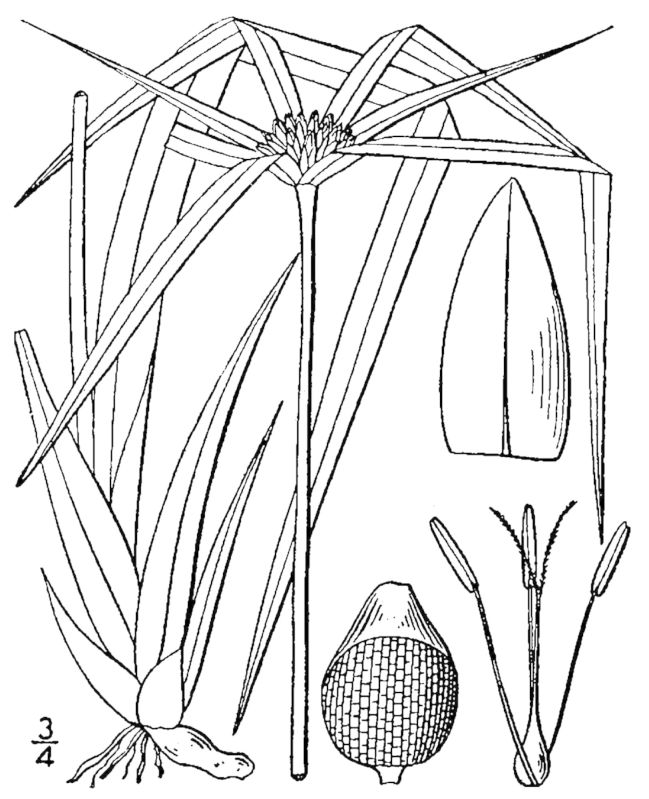
*Rhynchospora latifolia* (from Britton and Brown 1913).

**Figure 42d.**
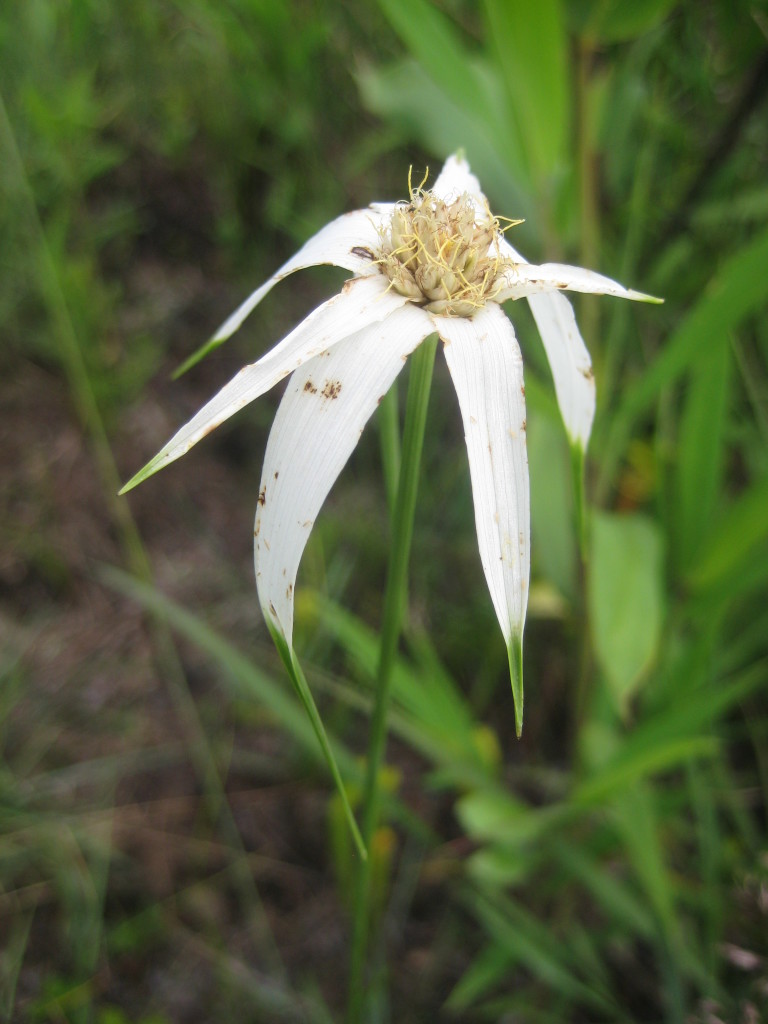
*Rhynchospora latifolia* (photo by R. Thornhill).

**Figure 43a.**
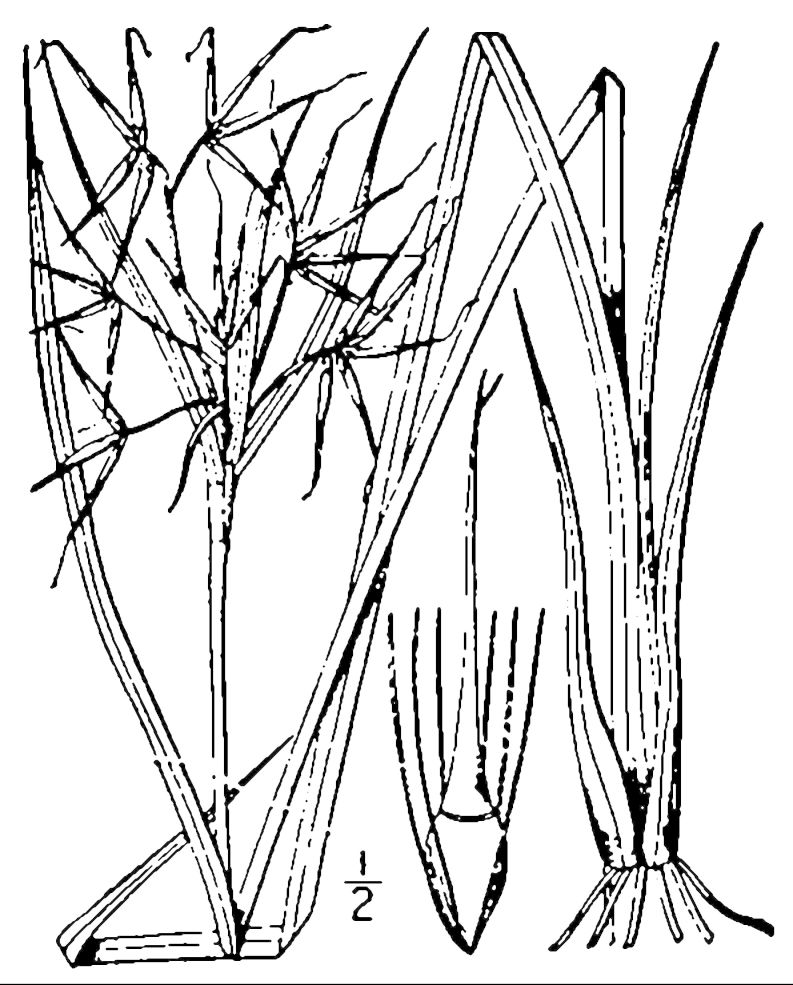
*Rhynchospora corniculata* (from Britton and Brown 1913).

**Figure 43b.**
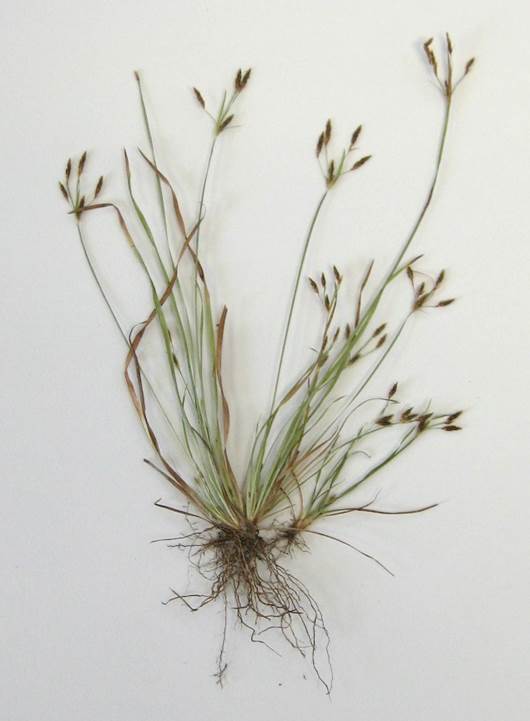
*Rhynchospora divergens* (photo by R. Thornhill).

**Figure 43c.**
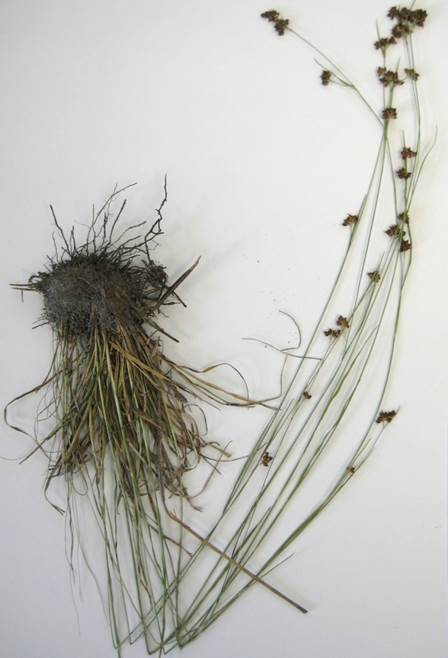
*Rhynchospora globularis* (photo by R. Thornhill).

**Figure 43d.**
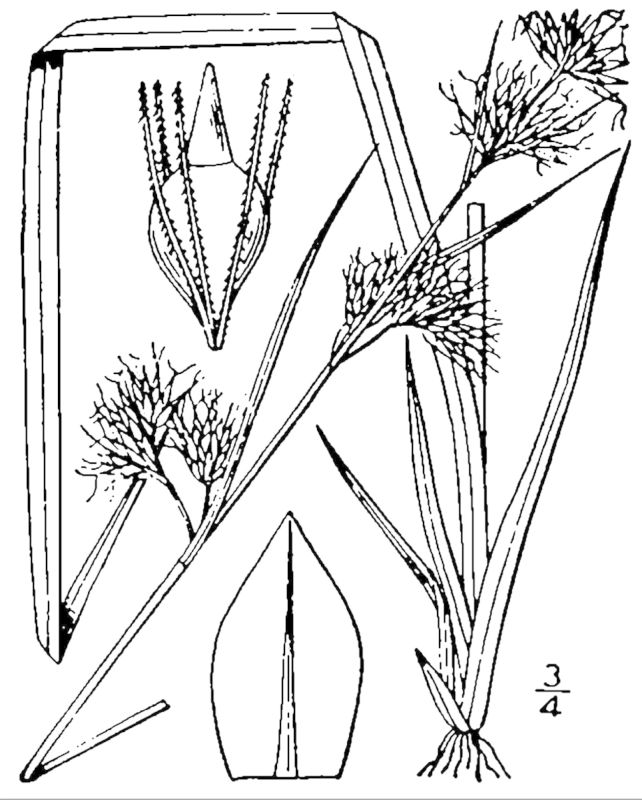
*Rhynchospora glomerata* (from Britton and Brown 1913).

**Figure 43e.**
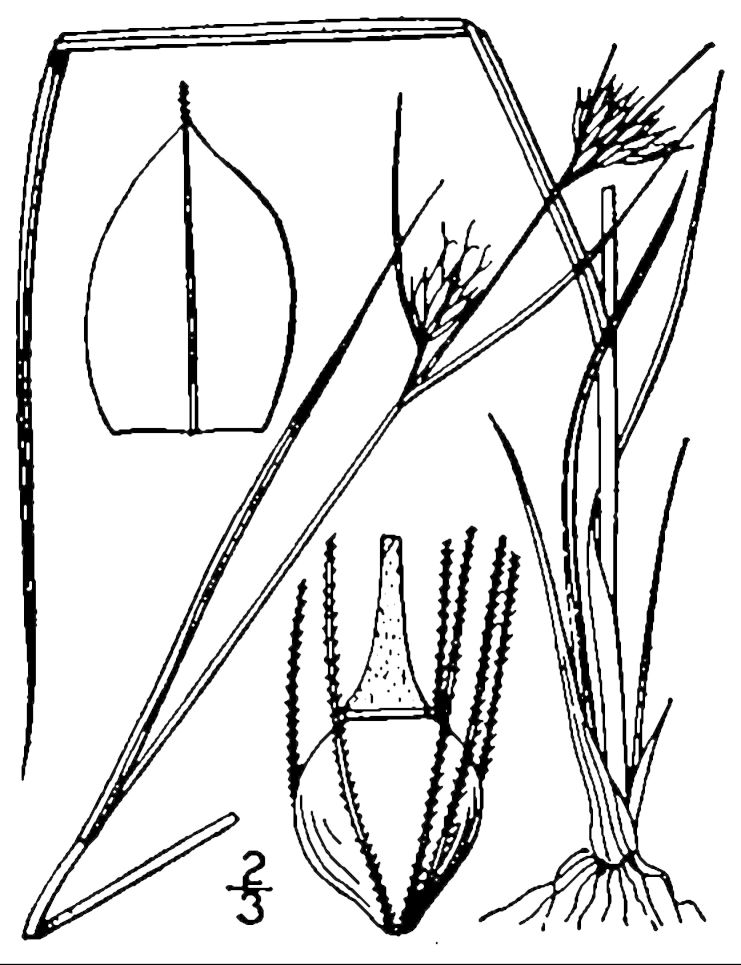
*Rhynchospora gracilenta* (from Britton and Brown 1913).

**Figure 43f.**
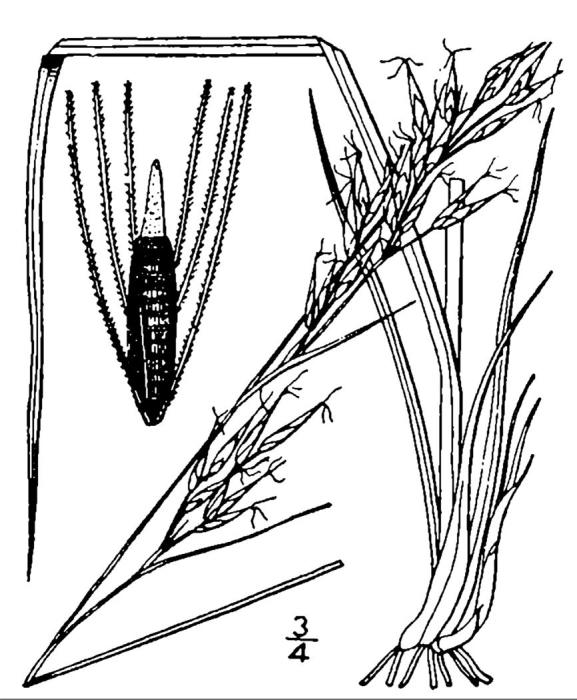
*Rhynchospora inexpansa* (from Britton and Brown 1913).

**Figure 44a.**
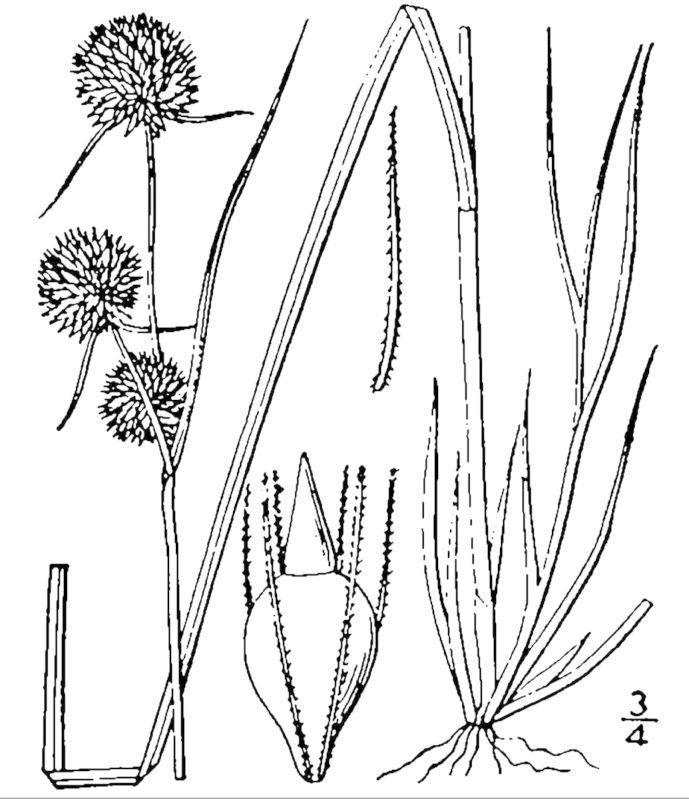
*Rhynchospora microcephala* (from Britton and Brown 1913).

**Figure 44b.**
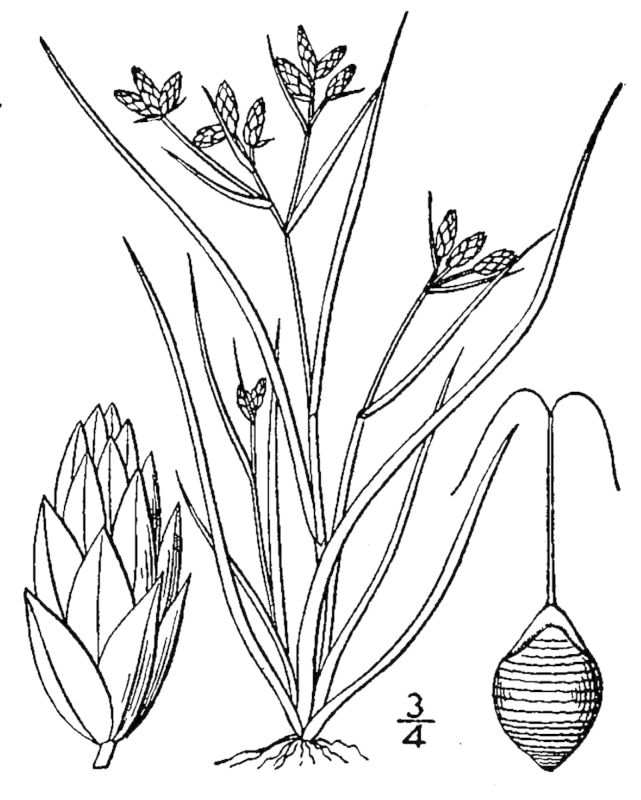
*Rhynchospora nitens* (from Britton and Brown 1913).

**Figure 44c.**
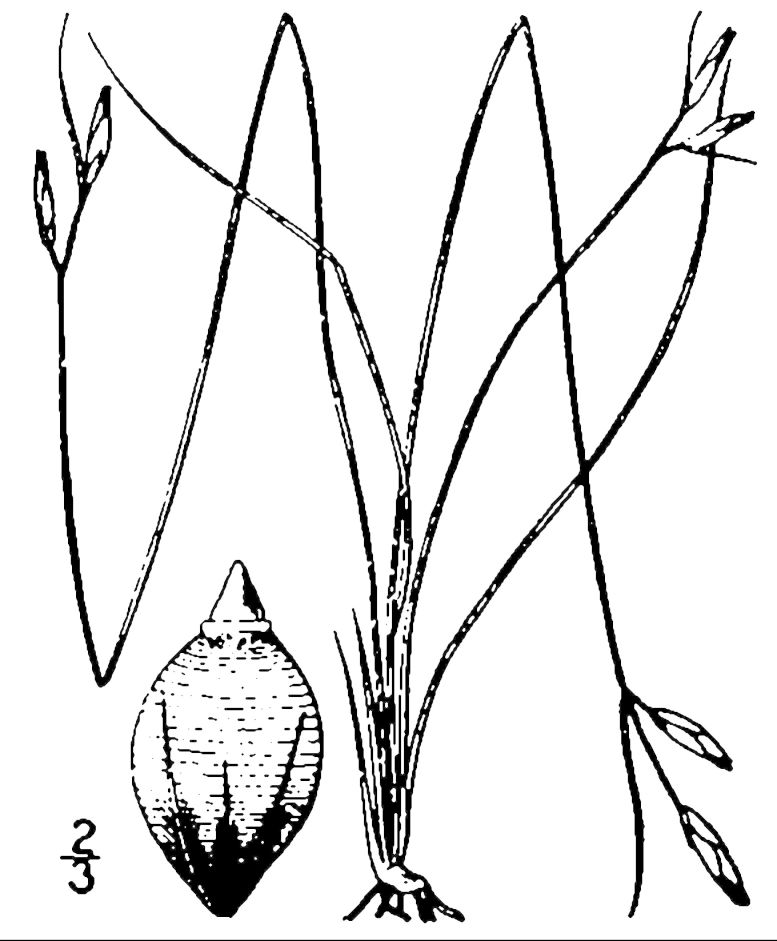
*Rhynchospora oligantha* (from Britton and Brown 1913).

**Figure 44d.**
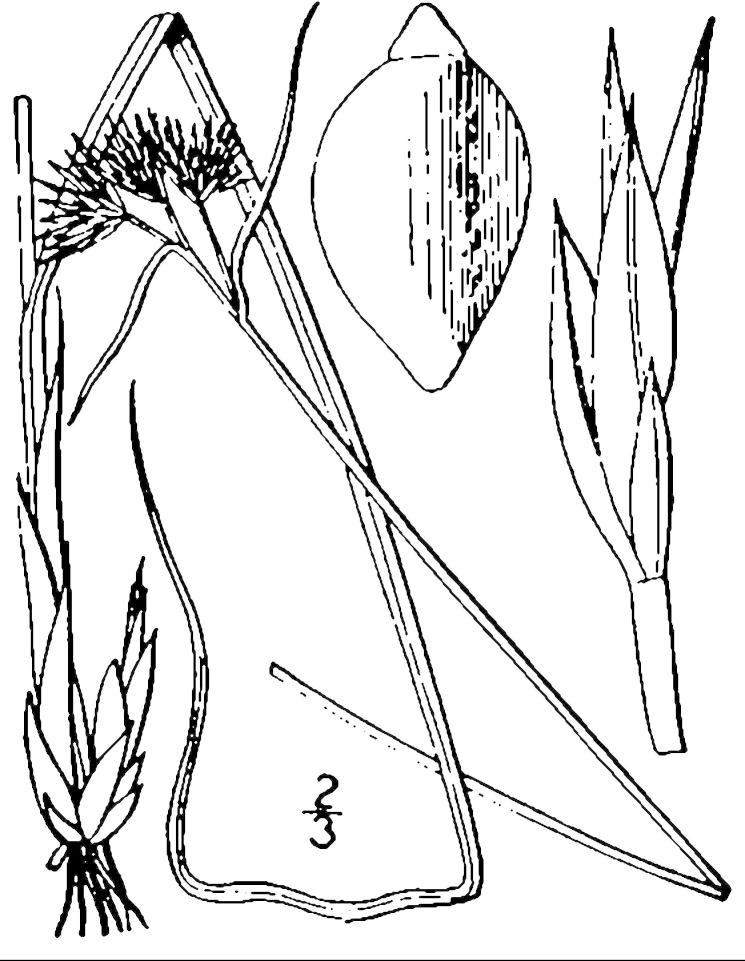
*Rhynchospora pallida* (from Britton and Brown 1913).

**Figure 44e.**
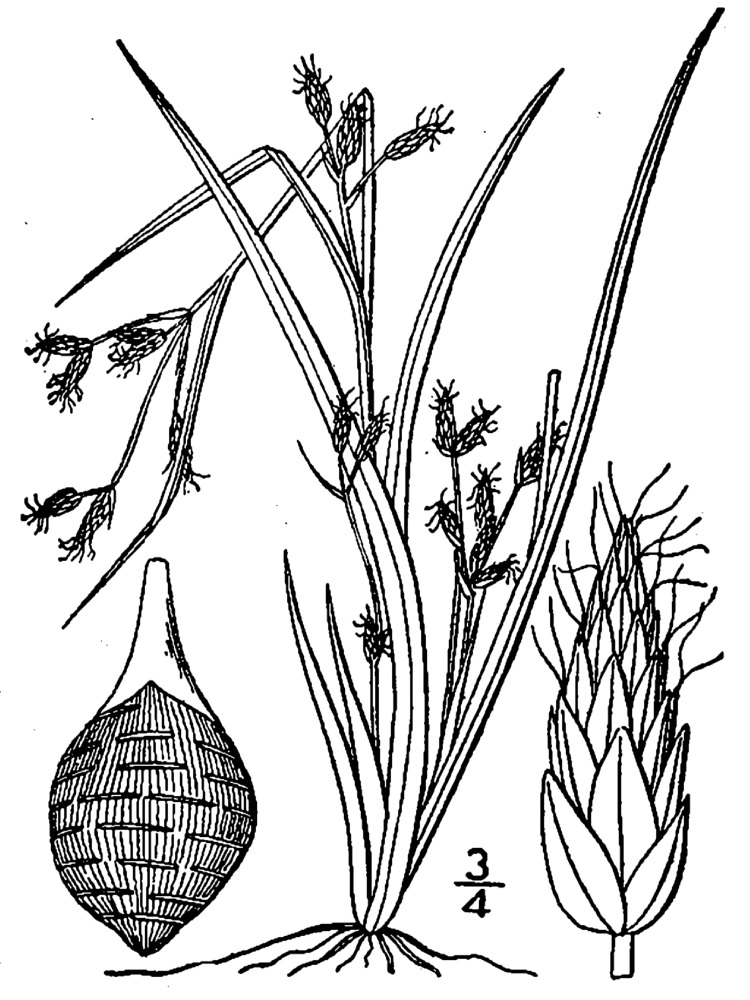
*Rhynchospora scirpoides* (from Britton and Brown 1913).

**Figure 44f.**
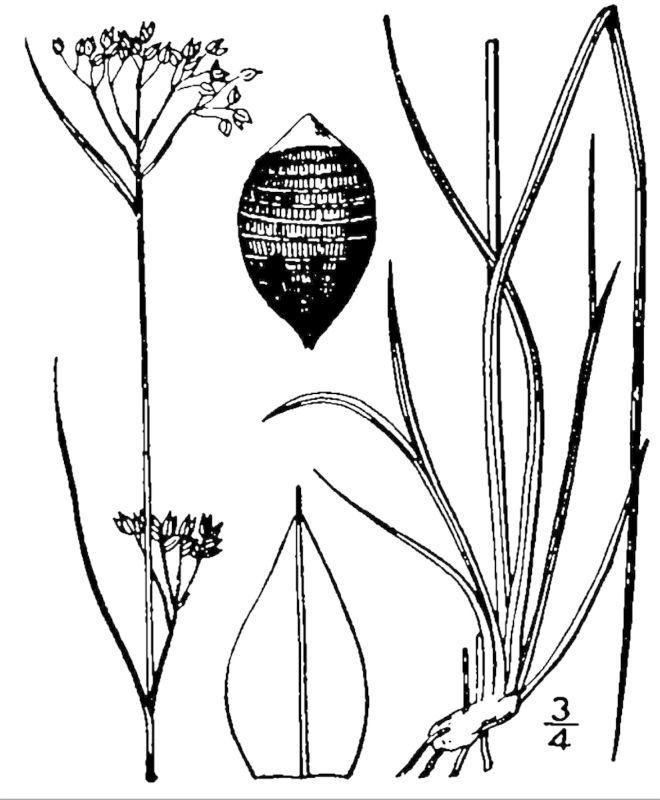
*Rhynchospora torreyana* (from Britton and Brown 1913).

**Figure 45a.**
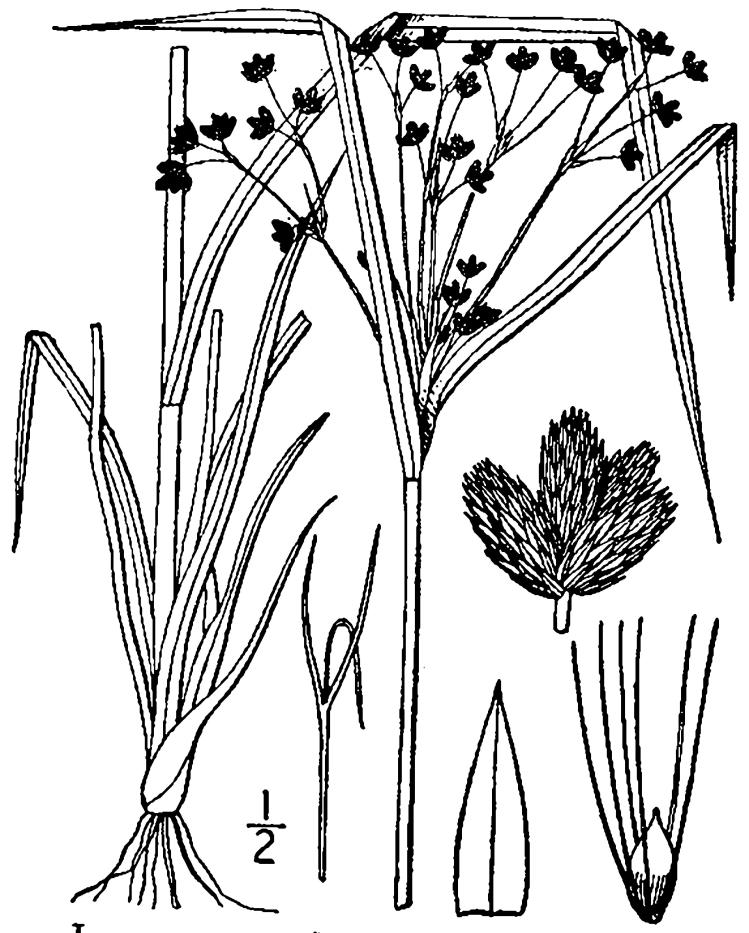
*Scirpus cyperinus* (from Britton and Brown 1913).

**Figure 45b.**
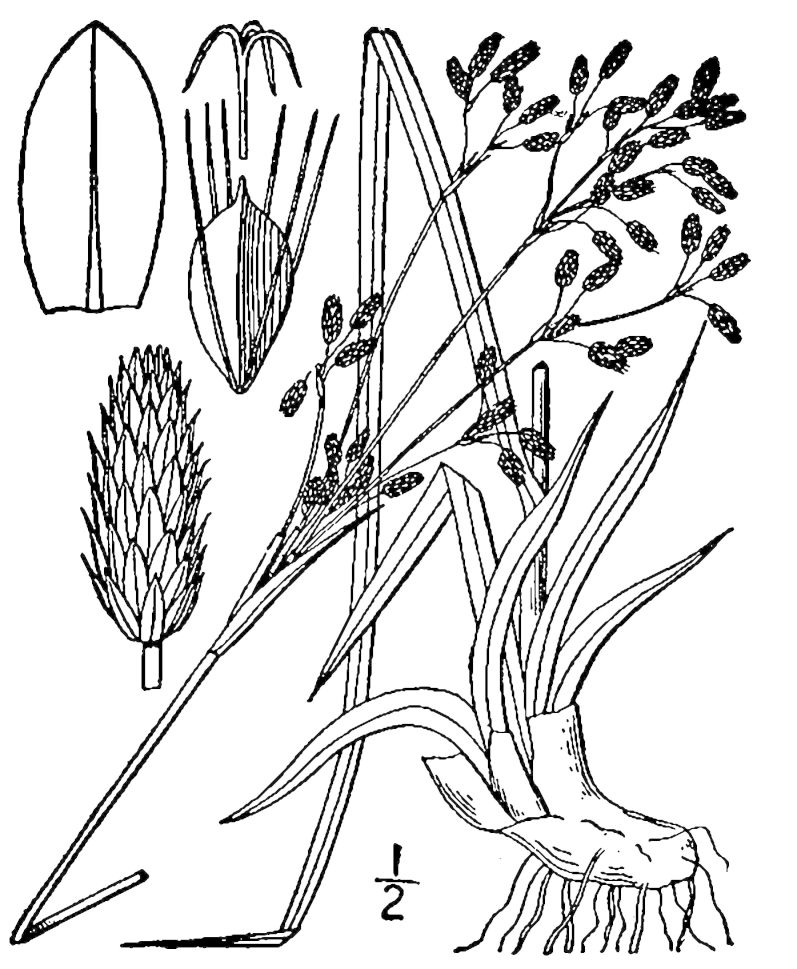
*Scirpus lineatus* (from Britton and Brown 1913).

**Figure 46a.**
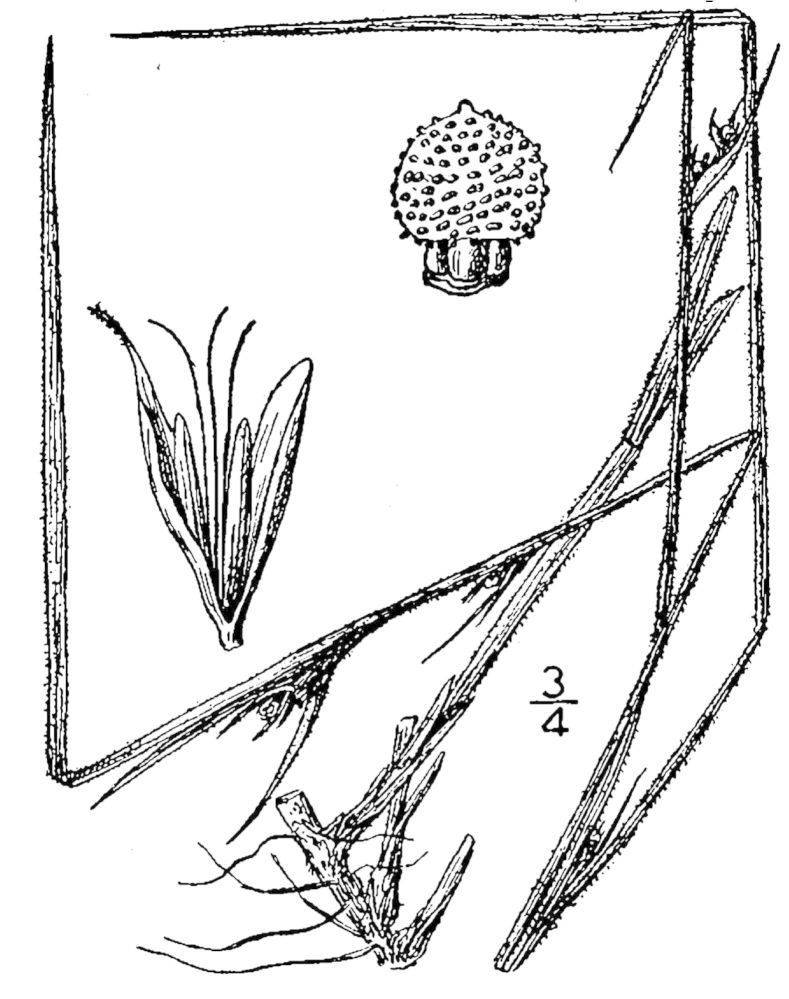
*Scleria ciliata* (from Britton and Brown 1913).

**Figure 46b.**
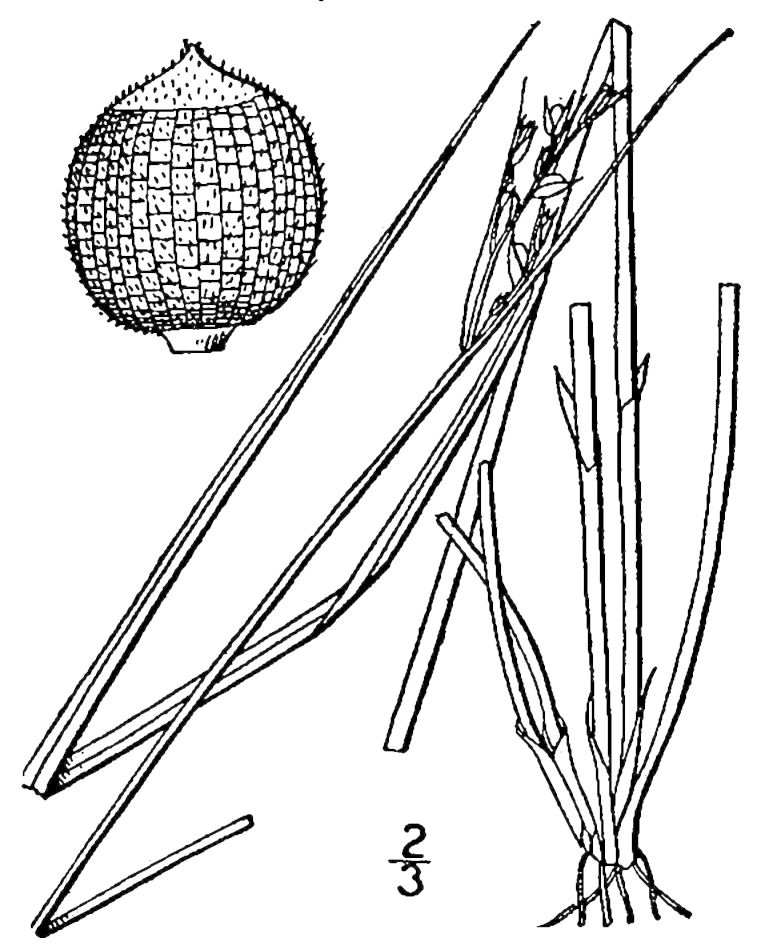
*Scleria muehlenbergii* (from Britton and Brown 1913).

**Figure 46c.**
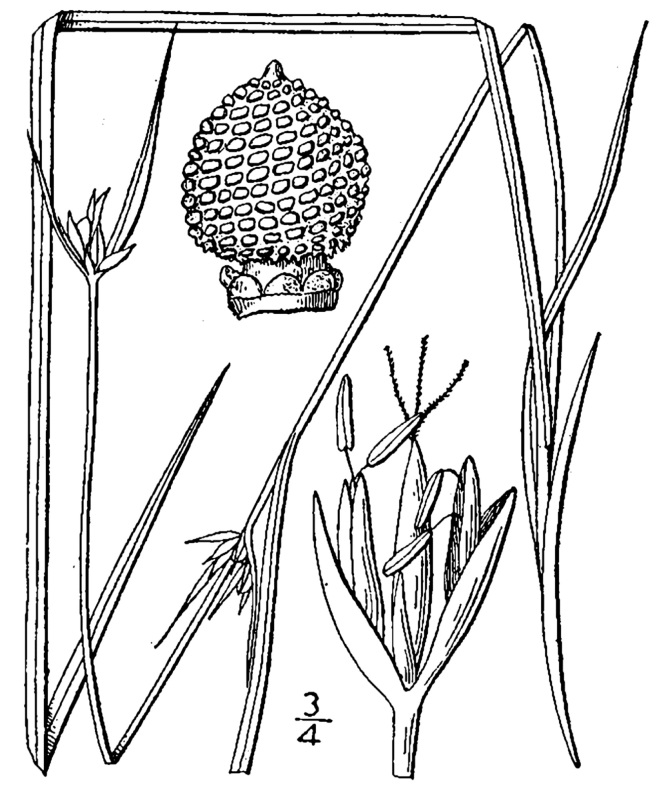
*Scleria pauciflora* (from Britton and Brown 1913).

**Figure 46d.**
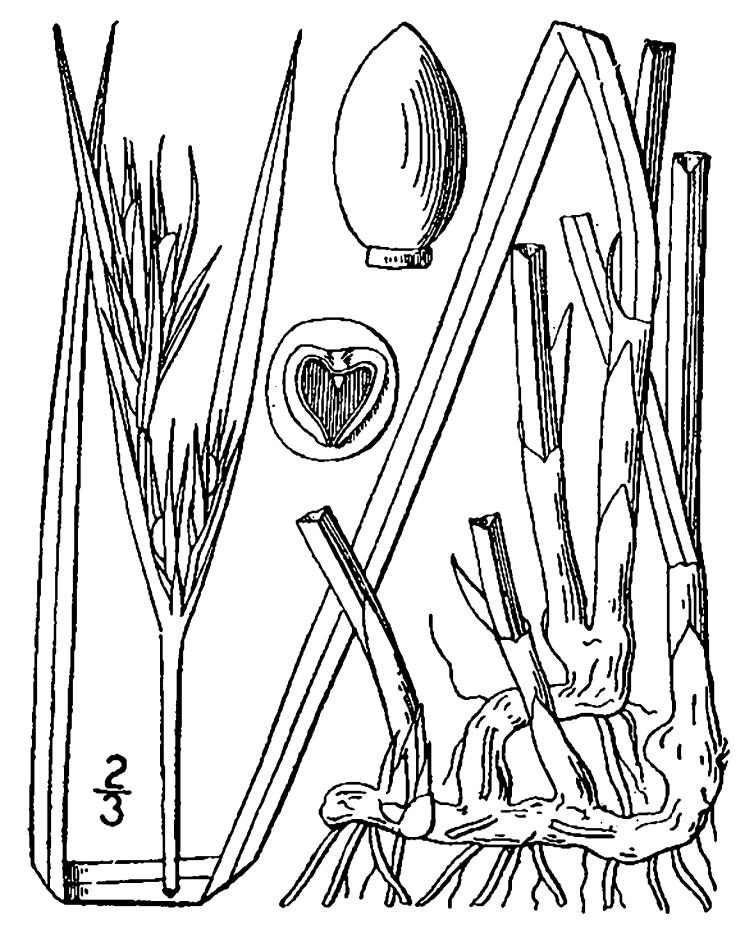
*Scleria triglomerata* (from Britton and Brown 1913).

**Figure 46e.**
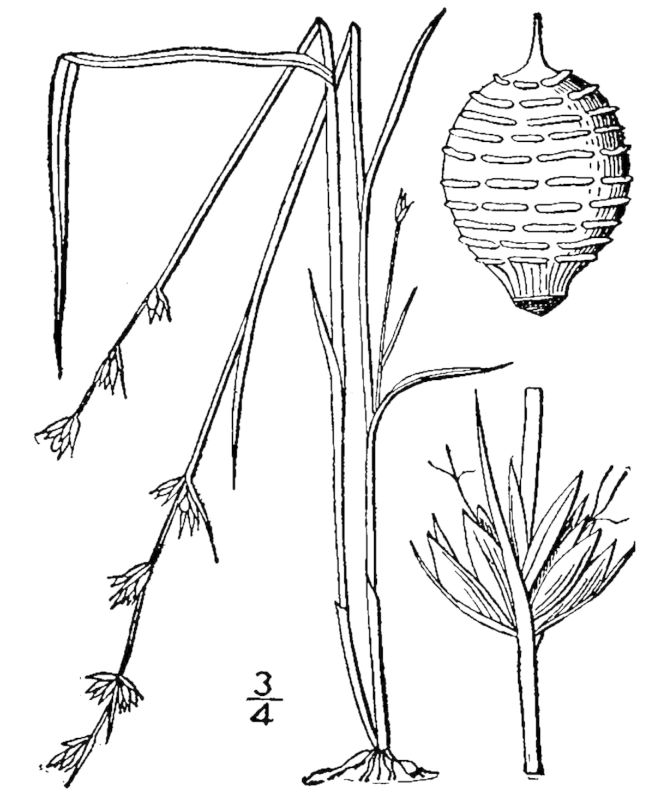
*Scleria verticillata* (from Britton and Brown 1913).

**Figure 47.**
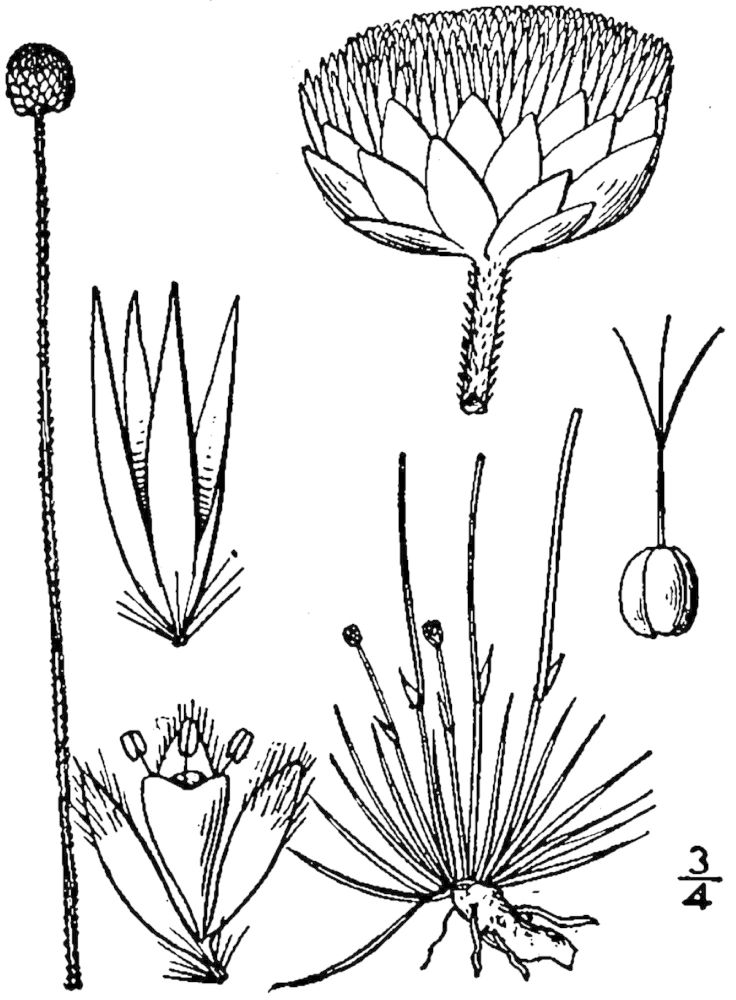
*Syngonanthus flavidulus* (from Britton and Brown 1913).

**Figure 48a.**
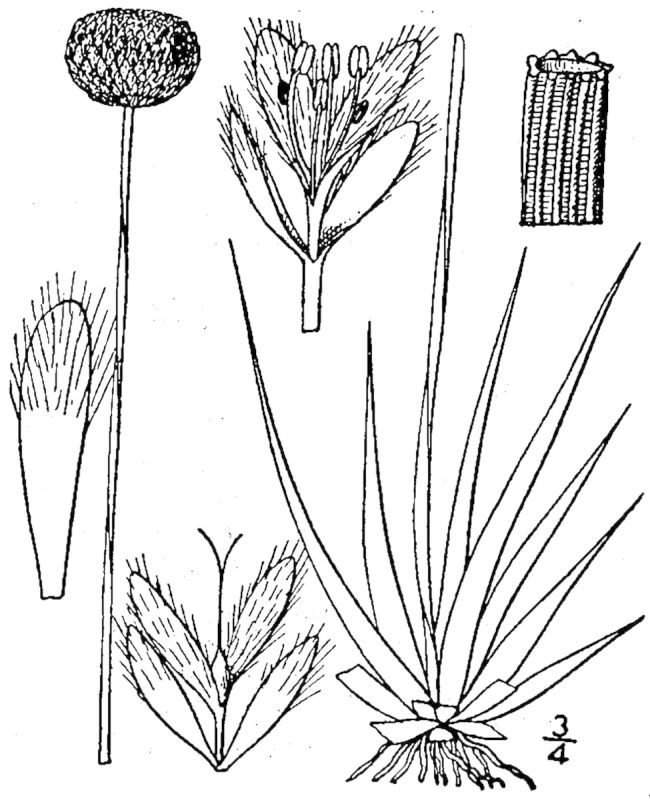
*Eriocaulon compressum* (from Britton and Brown 1913).

**Figure 48b.**
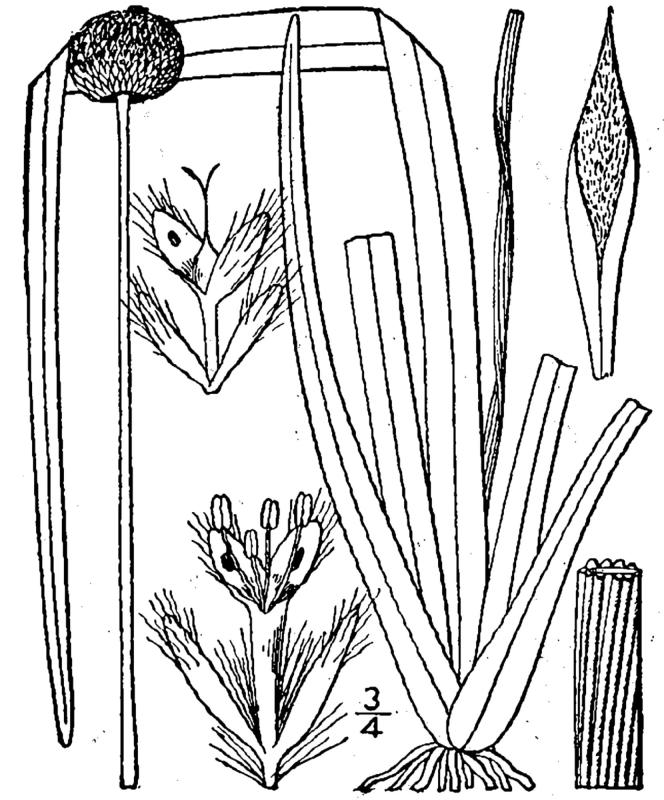
*Eriocaulon decangulare* (from Britton and Brown 1913).

**Figure 48c.**
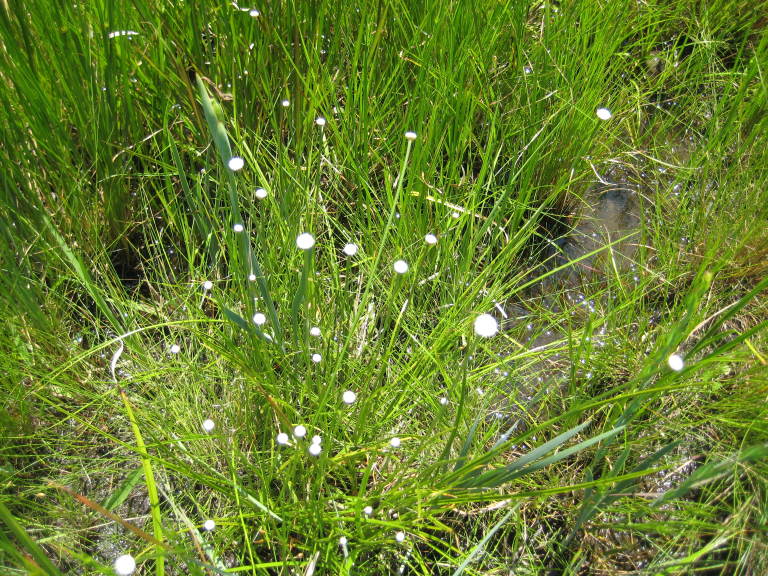
Eriocaulon decangulare var. decangulare (photo by R. Thornhill).

**Figure 49a.**
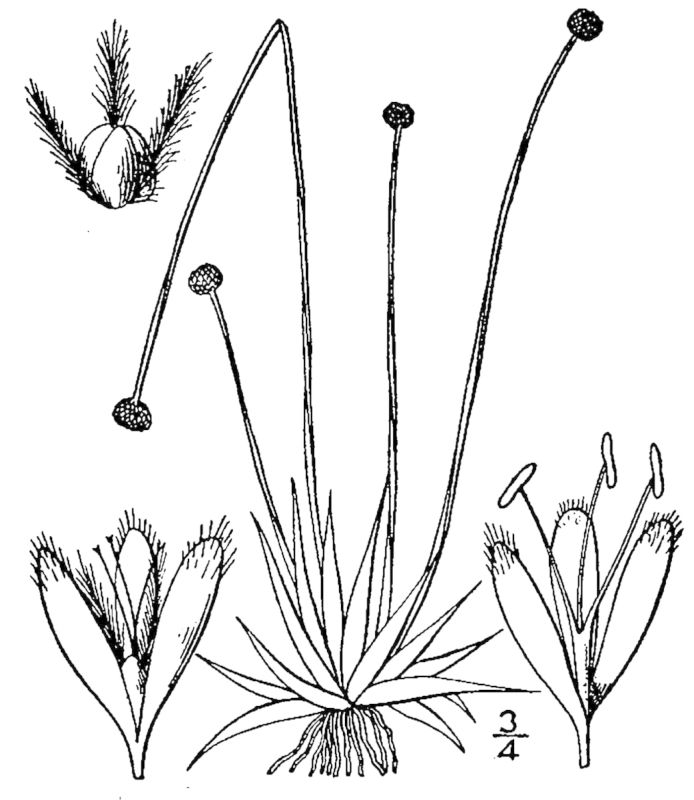
From Britton and Brown 1913.

**Figure 49b.**
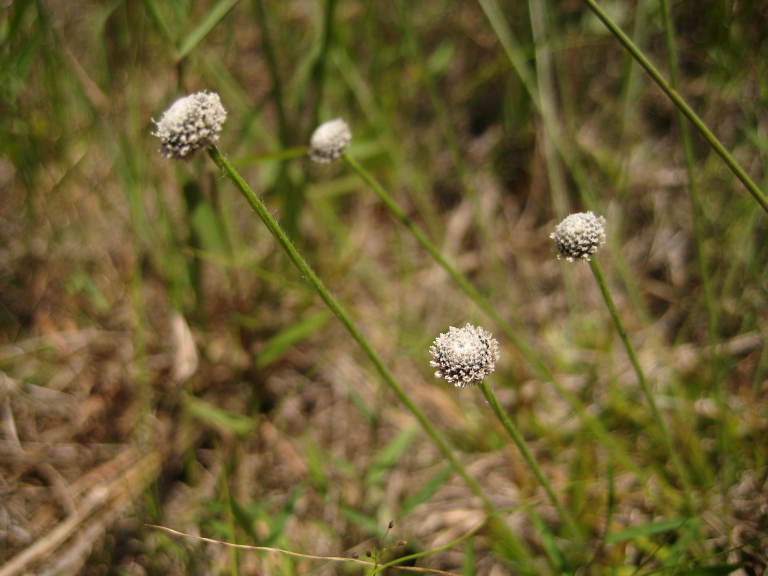
Photo by R. Thornhill.

**Figure 50.**
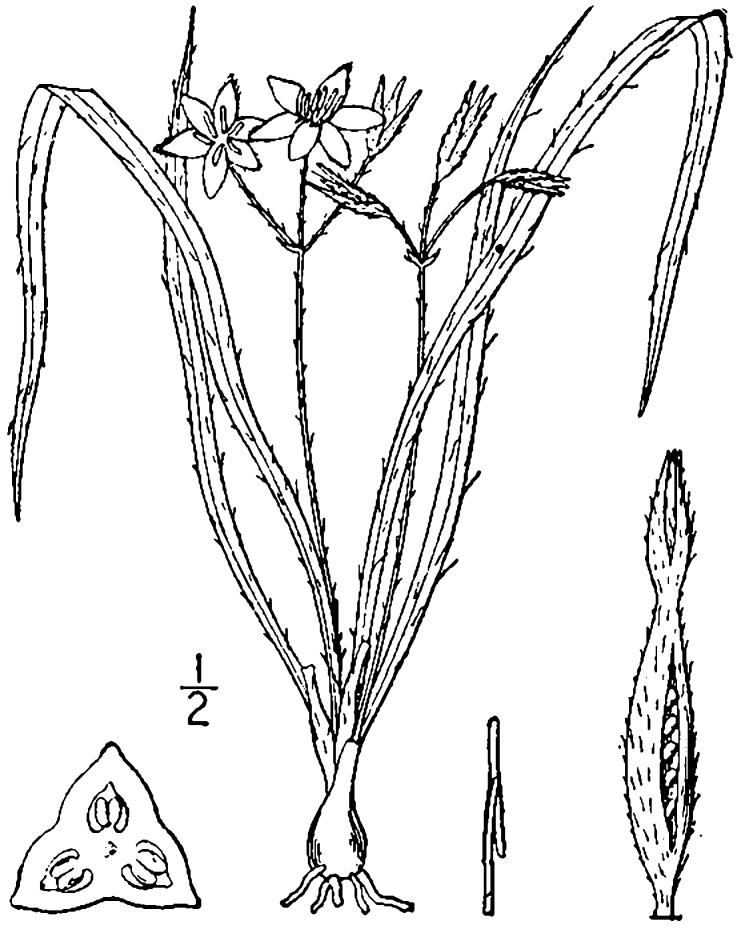
*Hypoxis hirsuta* (from Britton and Brown 1913).

**Figure 51a.**
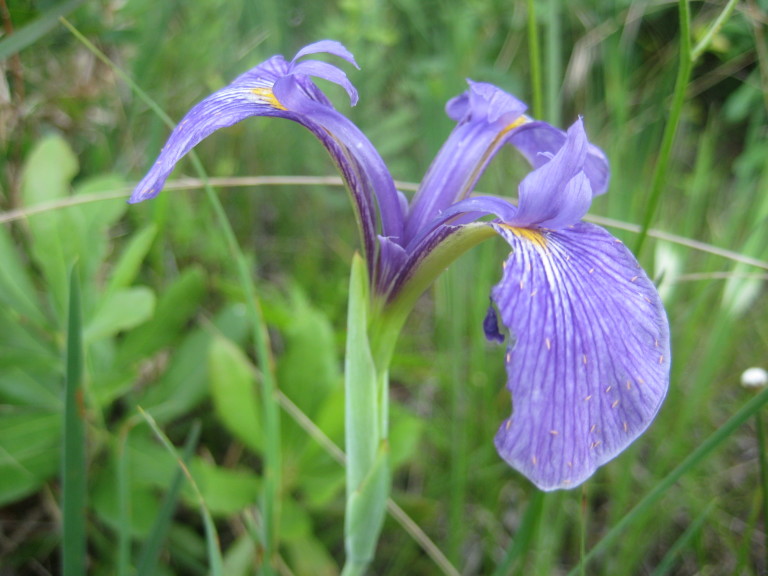
*Iris tridentata* (photo by R. Thornhill).

**Figure 51b.**
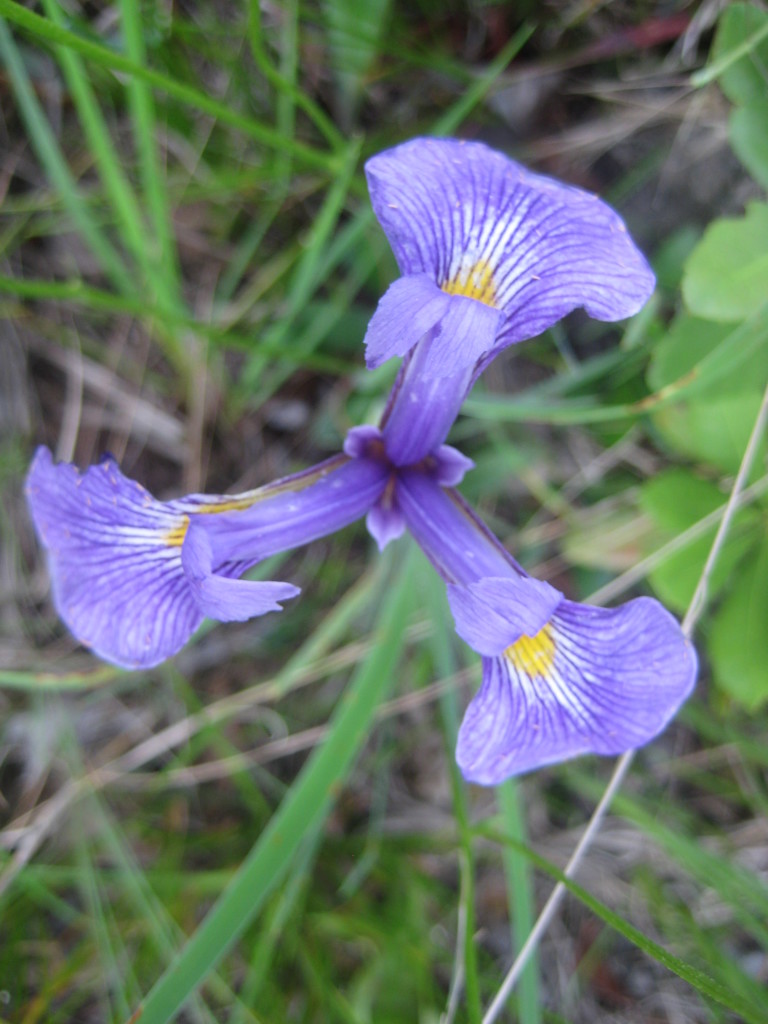
*Iris tridentata*. Note the reduced petals between the broad petaloid sepals (photo by R. Thornhill).

**Figure 51c.**
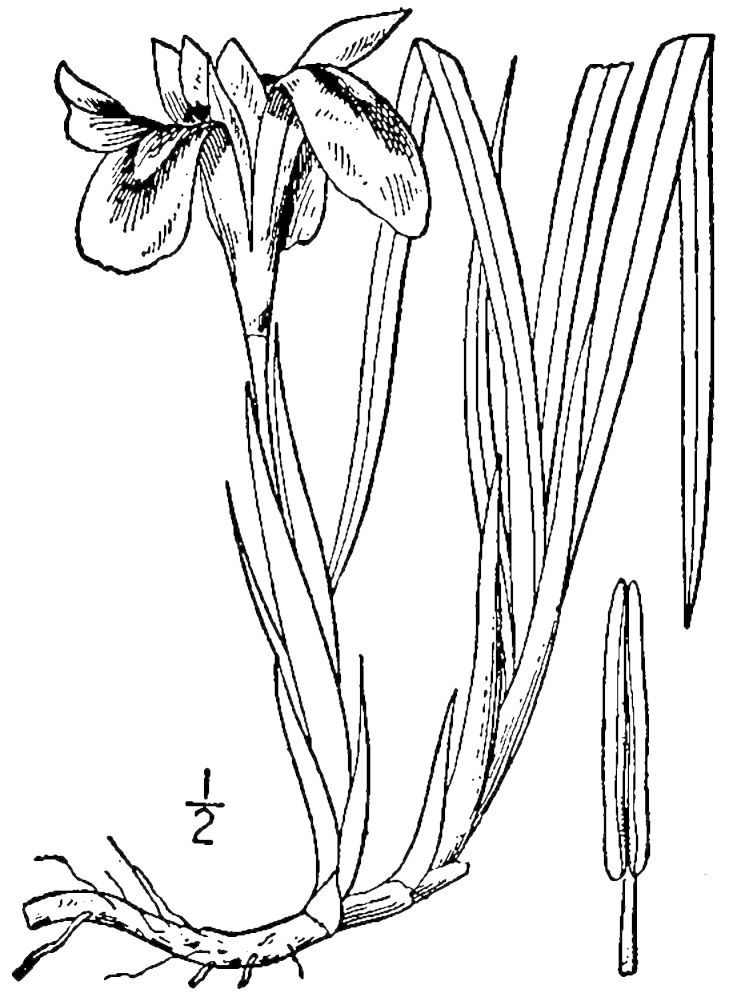
*Iris verna* (from Britton and Brown 1913).

**Figure 51d.**
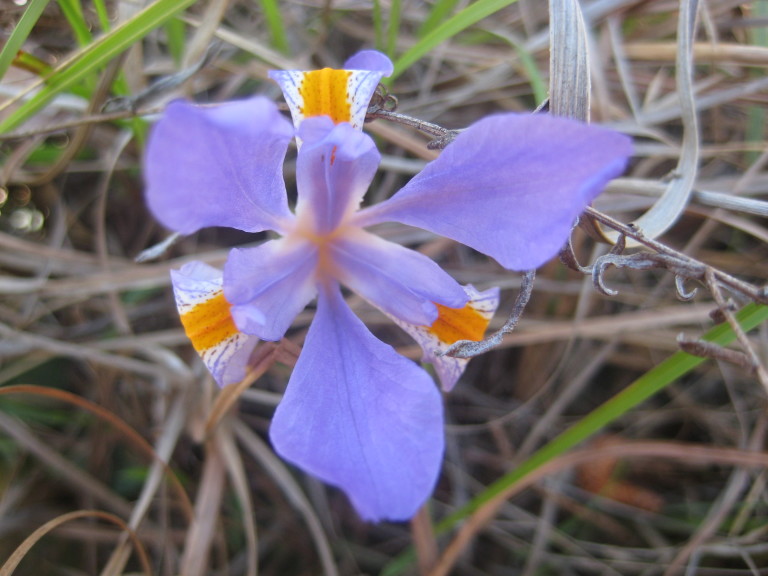
Iris verna var. verna (photo by R. Thornhill).

**Figure 51e.**
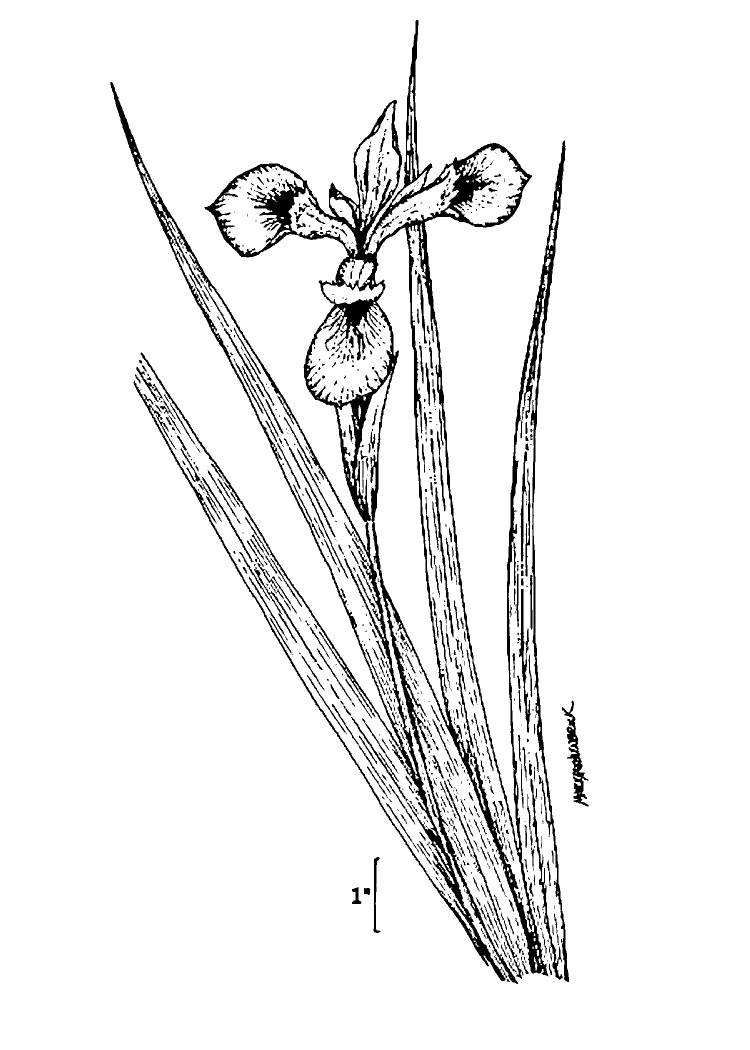
*Iris virginica* (from USDA-NRCS 2012).

**Figure 52a.**
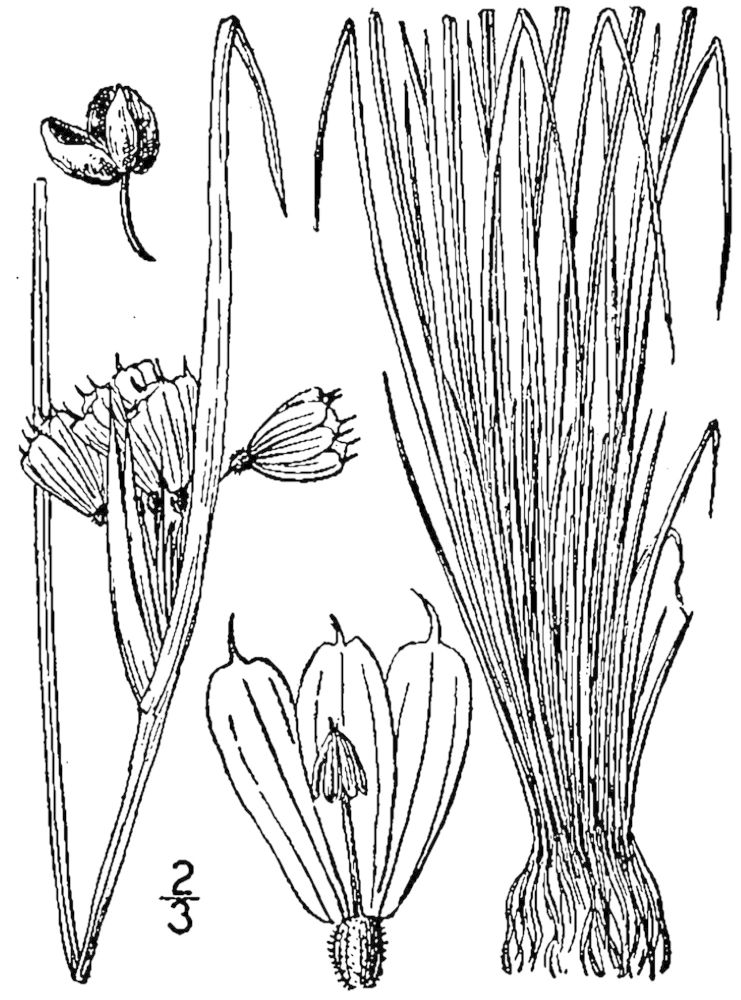
*Sassafras albidum* (from Britton and Brown 1913).

**Figure 52b.**
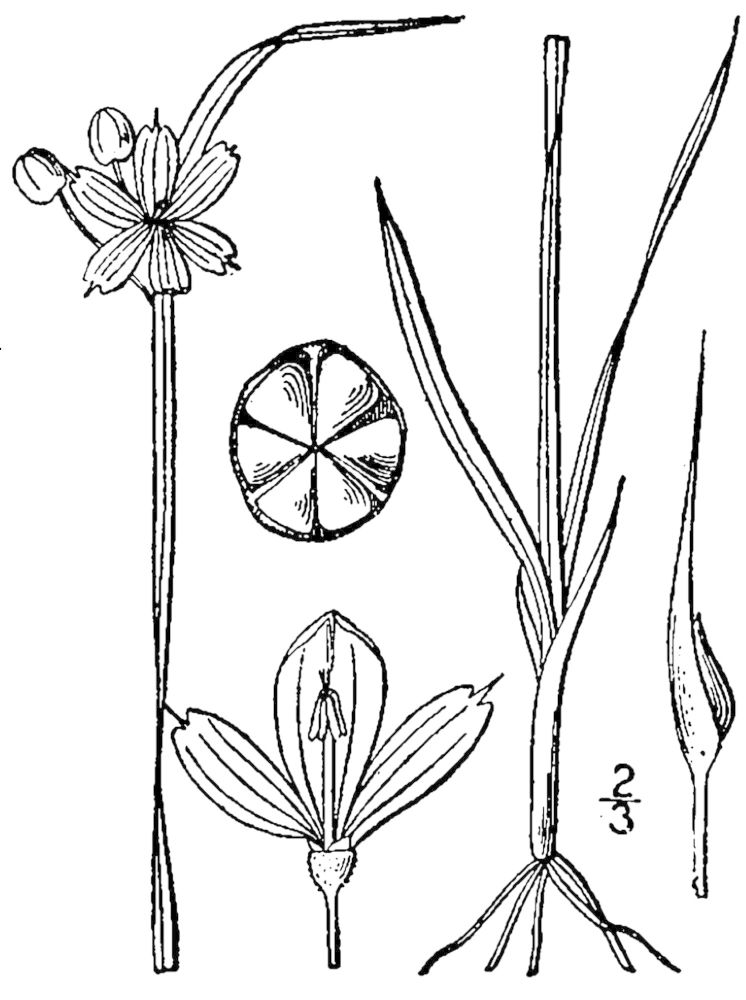
*Sisyrinchium angustifolium* (from Britton and Brown 1913).

**Figure 52c.**
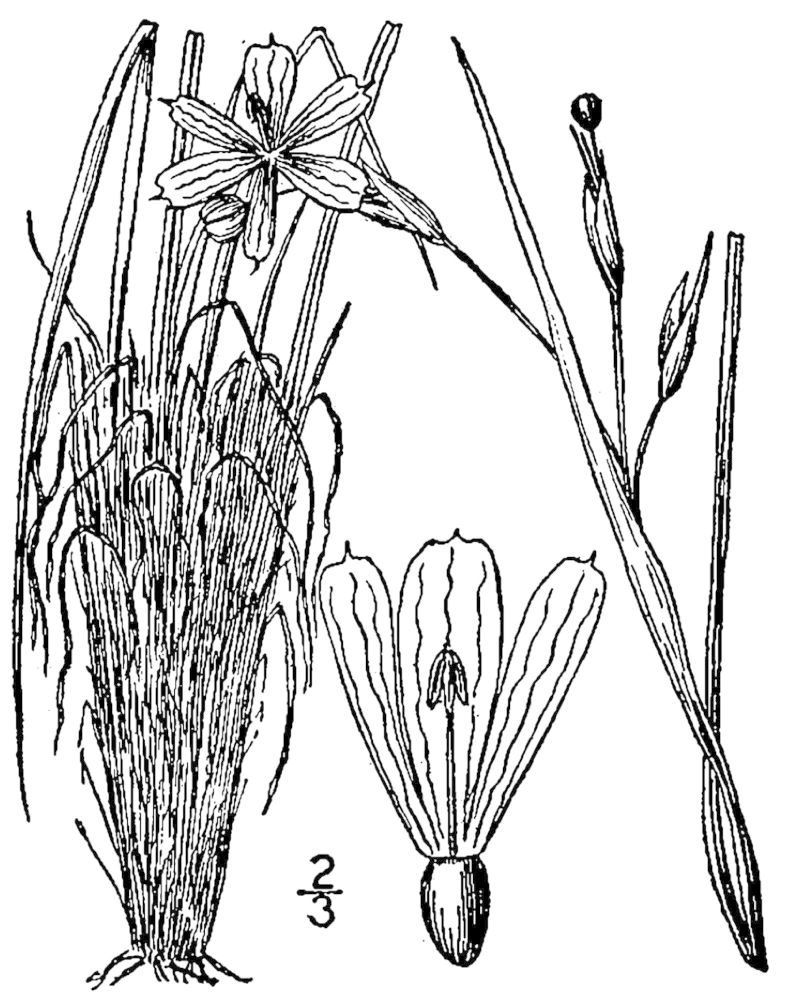
*Sisyrinchium arenicola* (from Britton and Brown 1913).

**Figure 52d.**
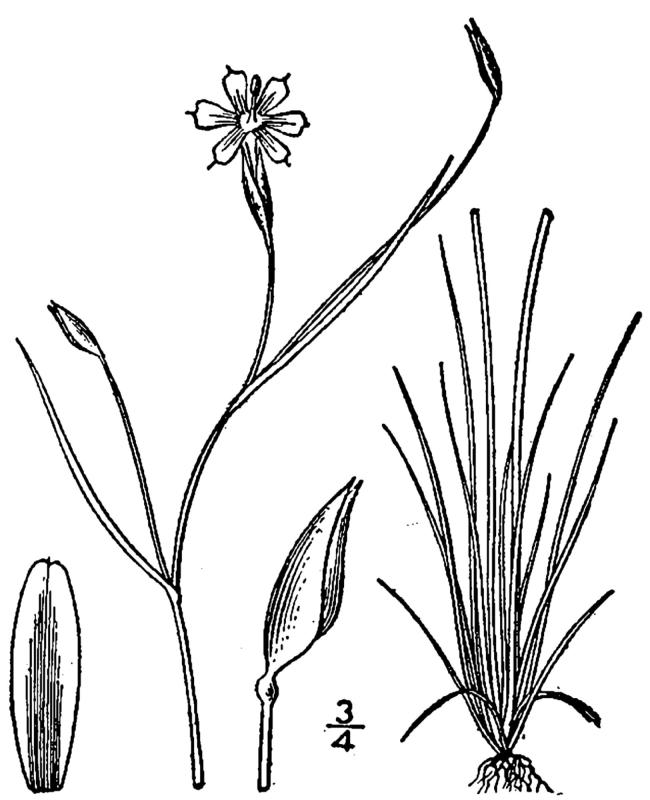
*Sisyrinchium atlanticum* (from Britton and Brown 1913).

**Figure 52e.**
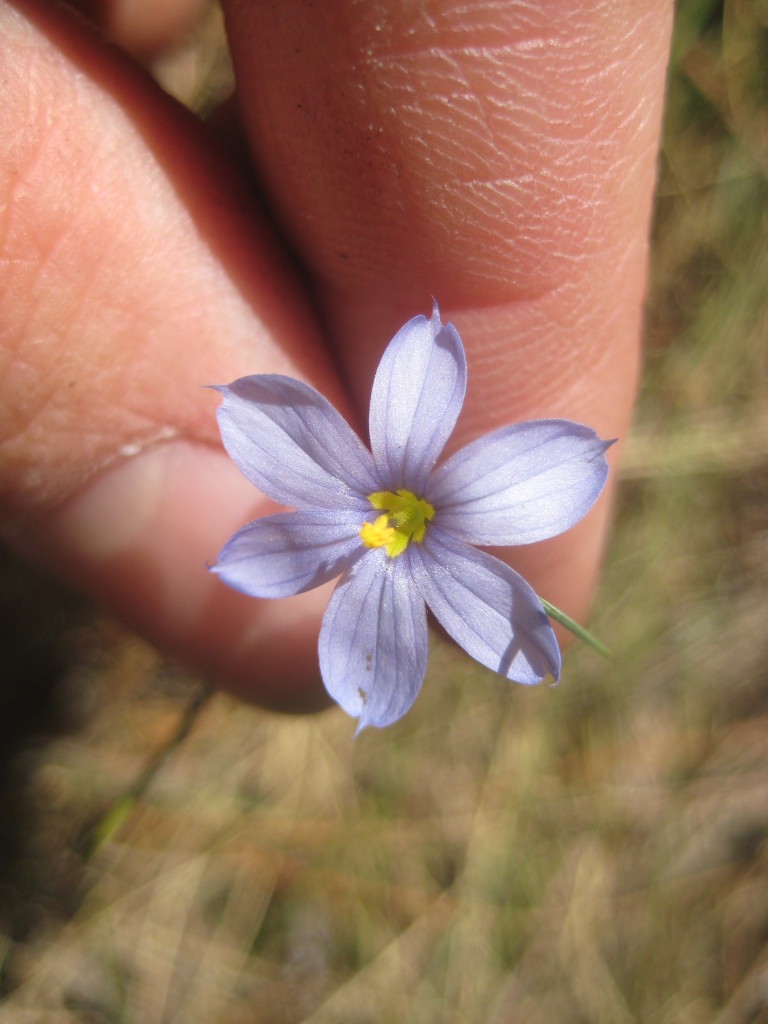
*Sisyrinchium capillare* (photo by R. Thornhill).

**Figure 53a.**
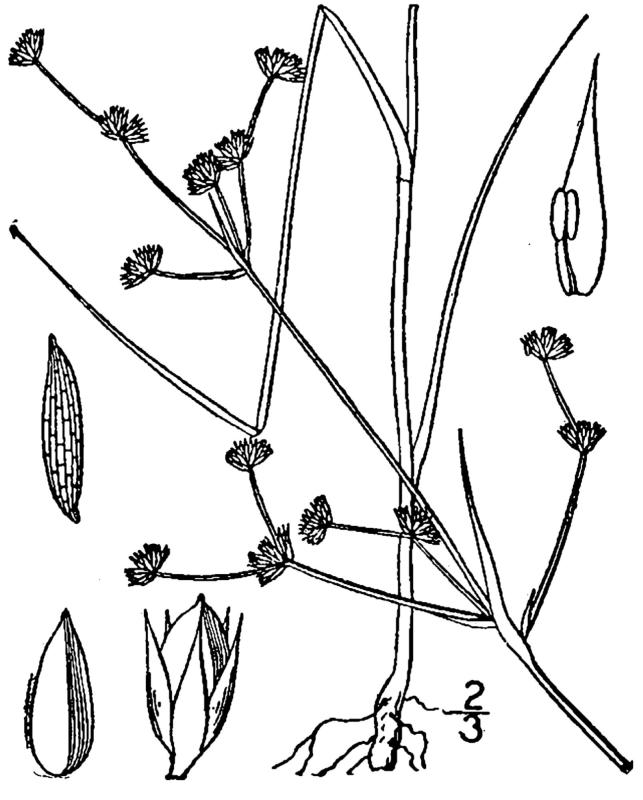
*Juncus acuminatus* (from Britton and Brown 1913).

**Figure 53b.**
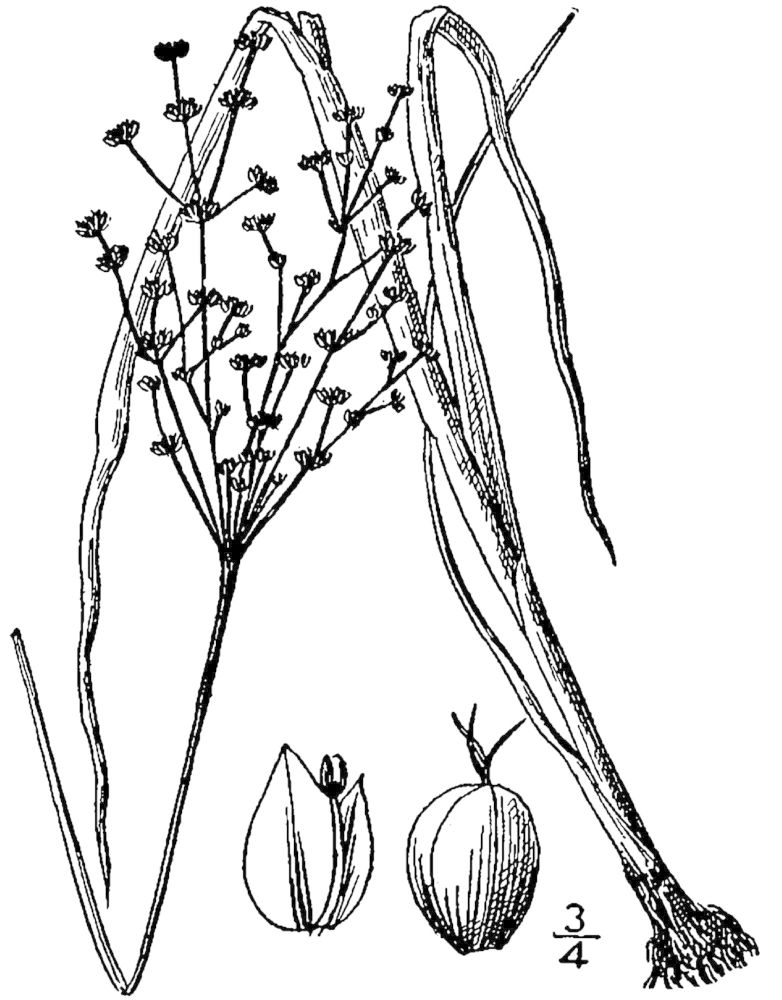
*Juncus biflorus* (from Britton and Brown 1913).

**Figure 53c.**
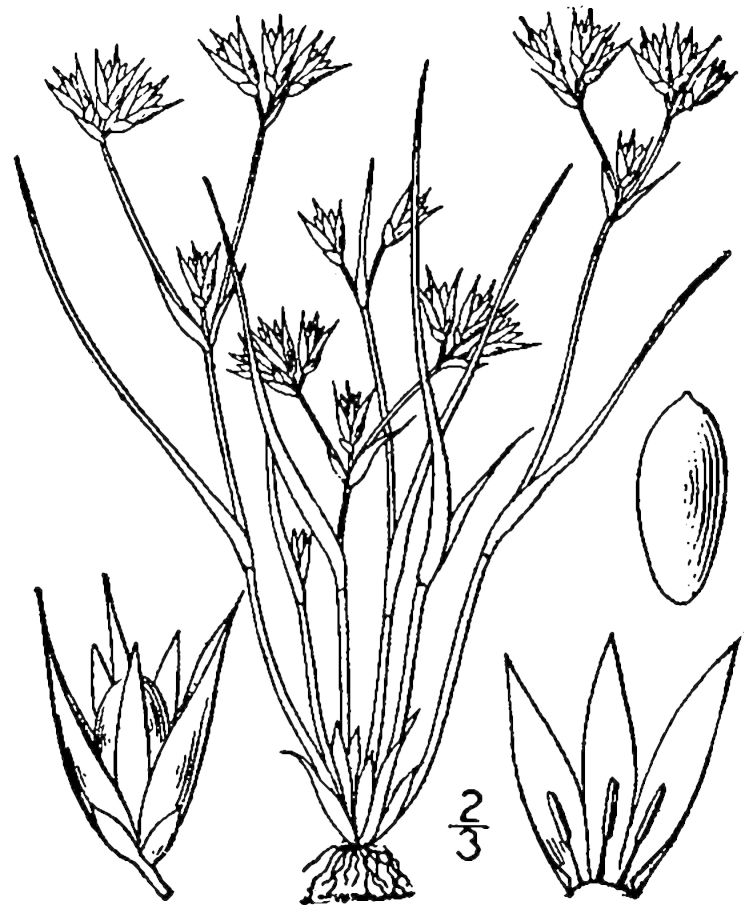
*Juncus bufonius* (from Britton and Brown 1913).

**Figure 53d.**
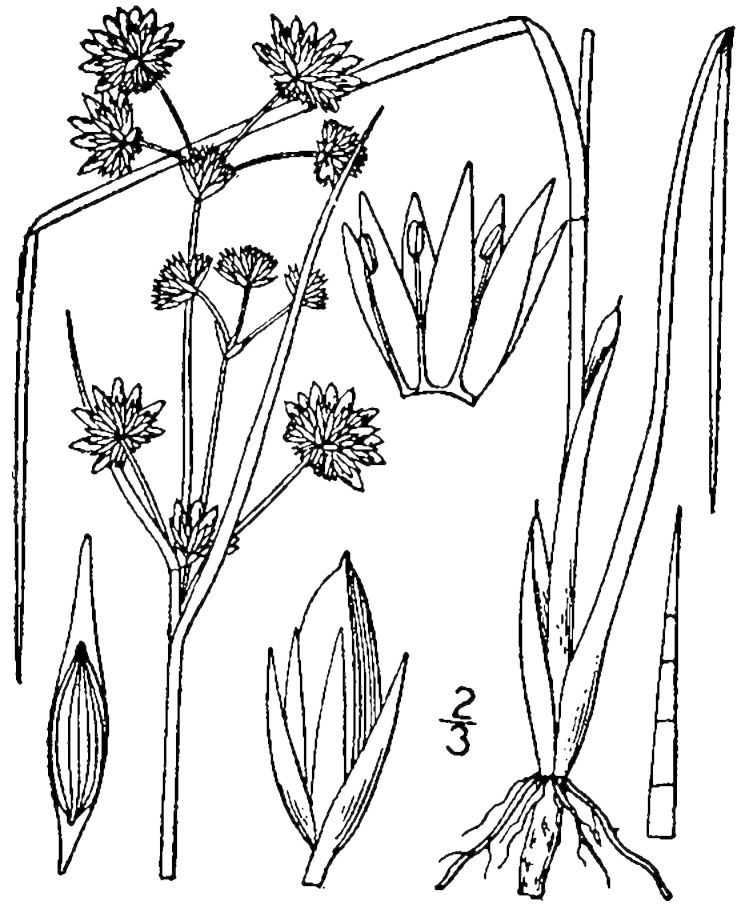
*Juncus canadensis* (from Britton and Brown 1913).

**Figure 53e.**
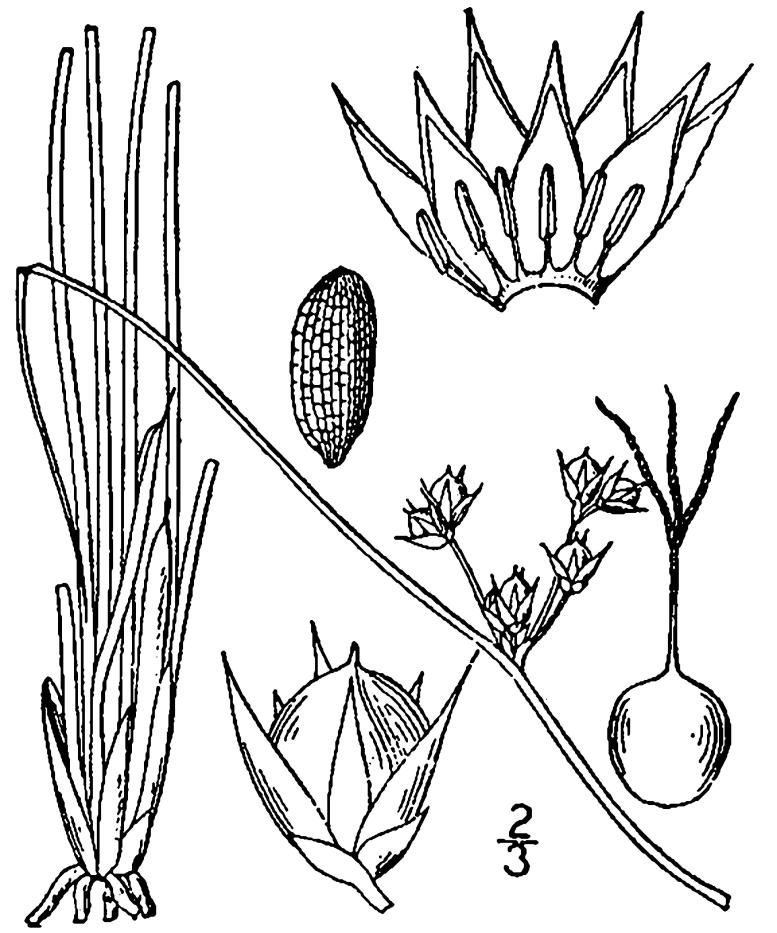
*Juncus coriaceus* (from Britton and Brown 1913).

**Figure 53f.**
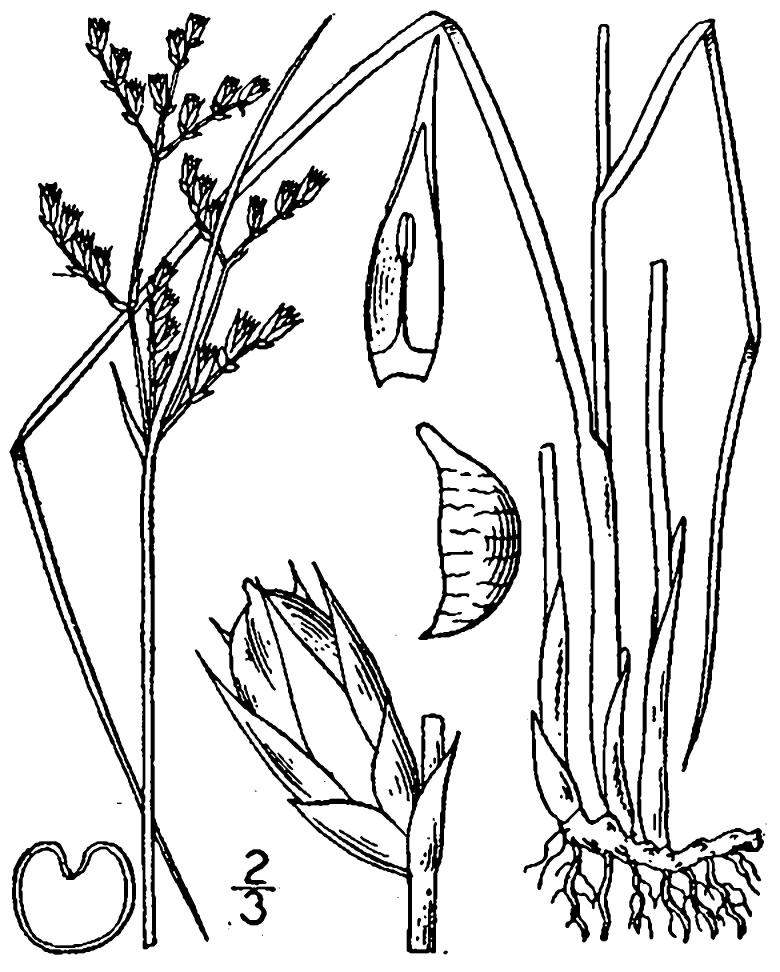
*Juncus dichotomus* (from Britton and Brown 1913).

**Figure 54a.**
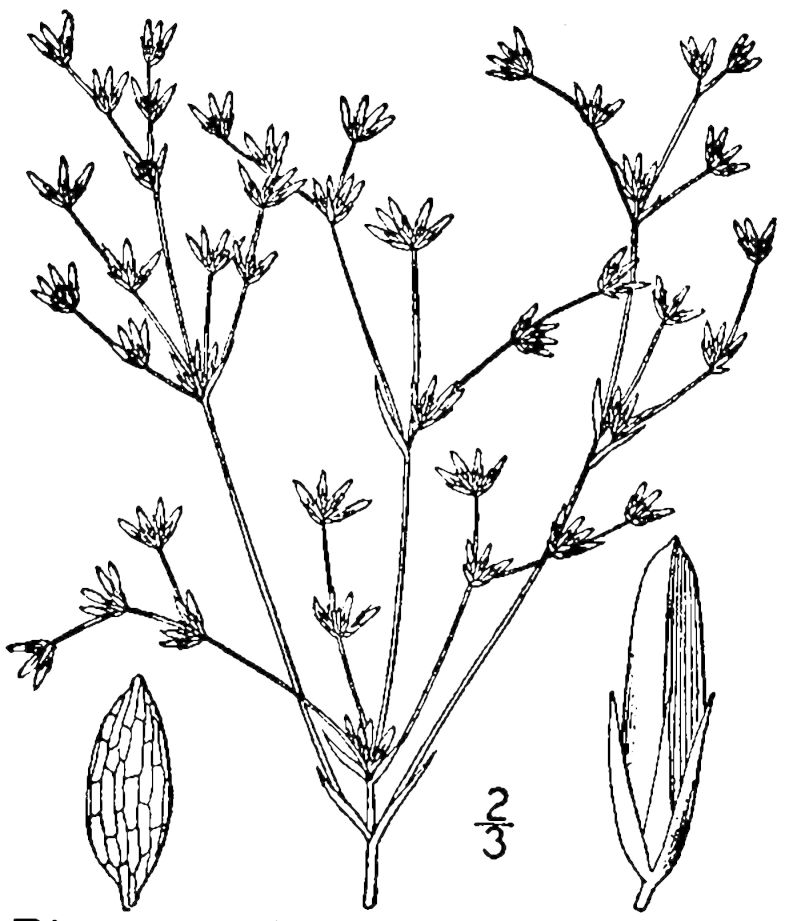
*Juncus diffusissimus* (from Britton and Brown 1913).

**Figure 54b.**
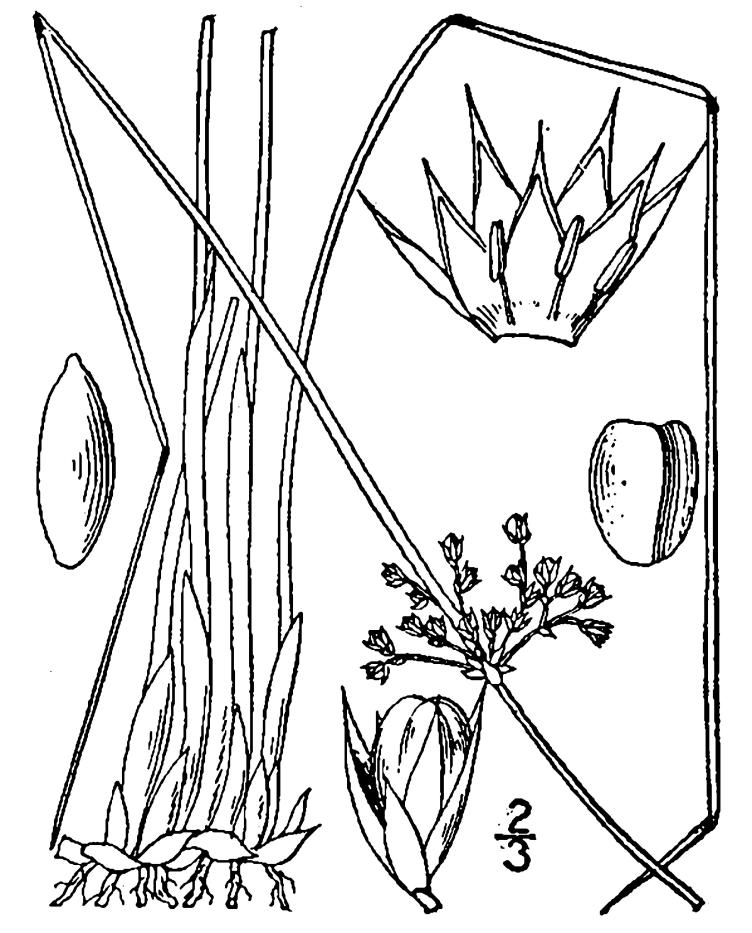
*Juncus effusus* (from Britton and Brown 1913).

**Figure 54c.**
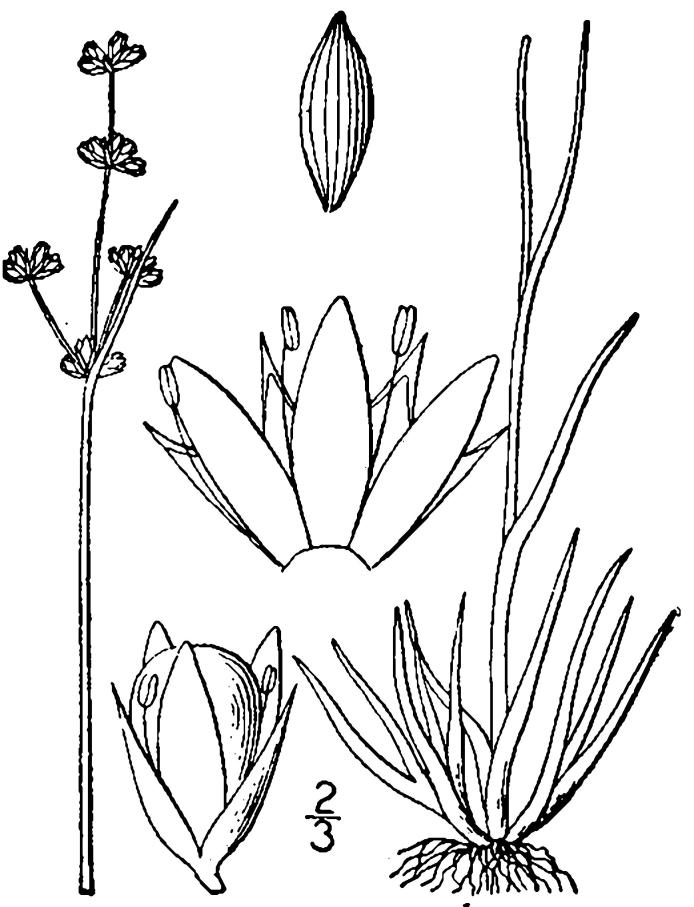
*Juncus marginatus* (from Britton and Brown 1913).

**Figure 54d.**
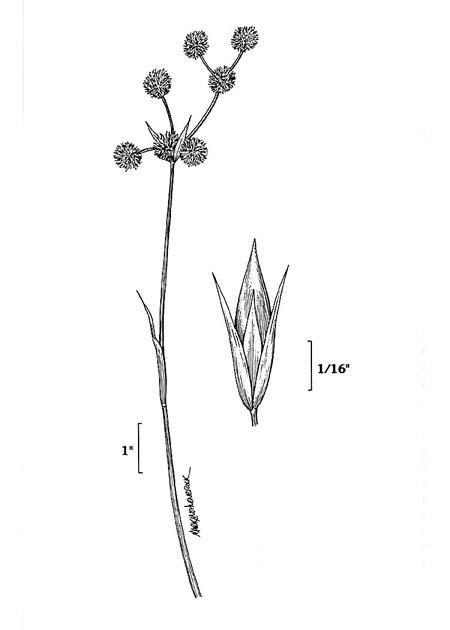
*Juncus megacephalus* (from USDA-NRCS 2012).

**Figure 54e.**
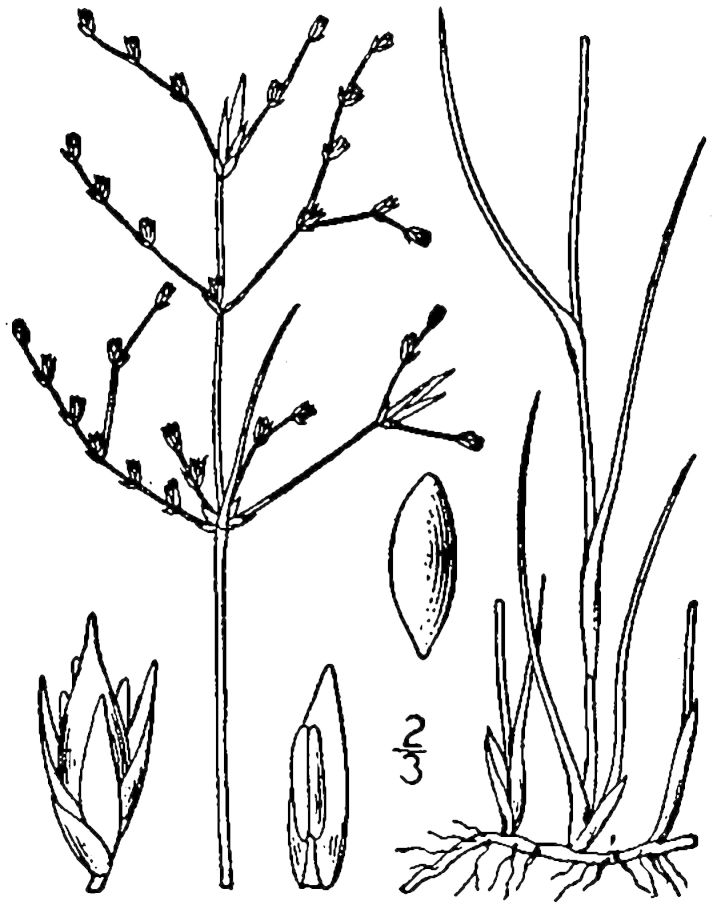
*Juncus pelocarpus* (from Britton and Brown 1913).

**Figure 54f.**
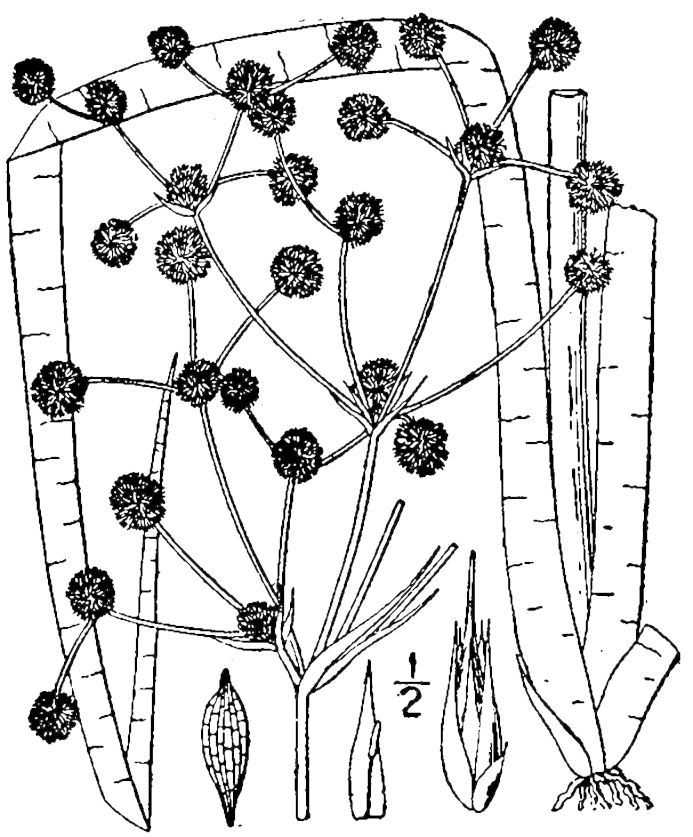
*Juncus polycephalos* (from Britton and Brown 1913).

**Figure 55a.**
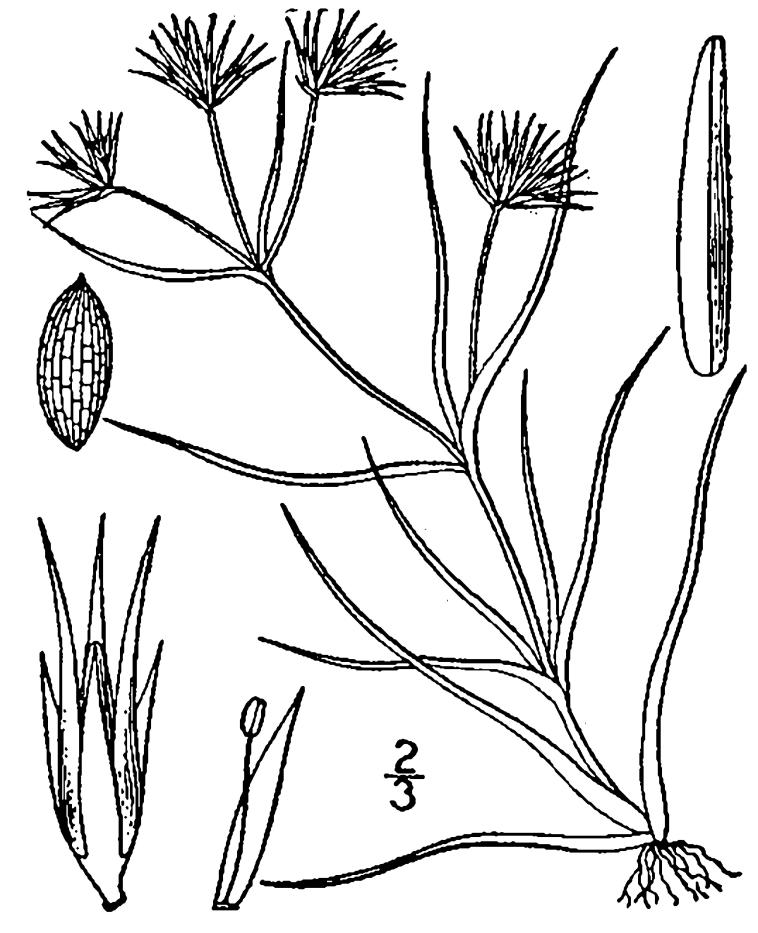
*Juncus repens* (from Britton and Brown 1913).

**Figure 55b.**
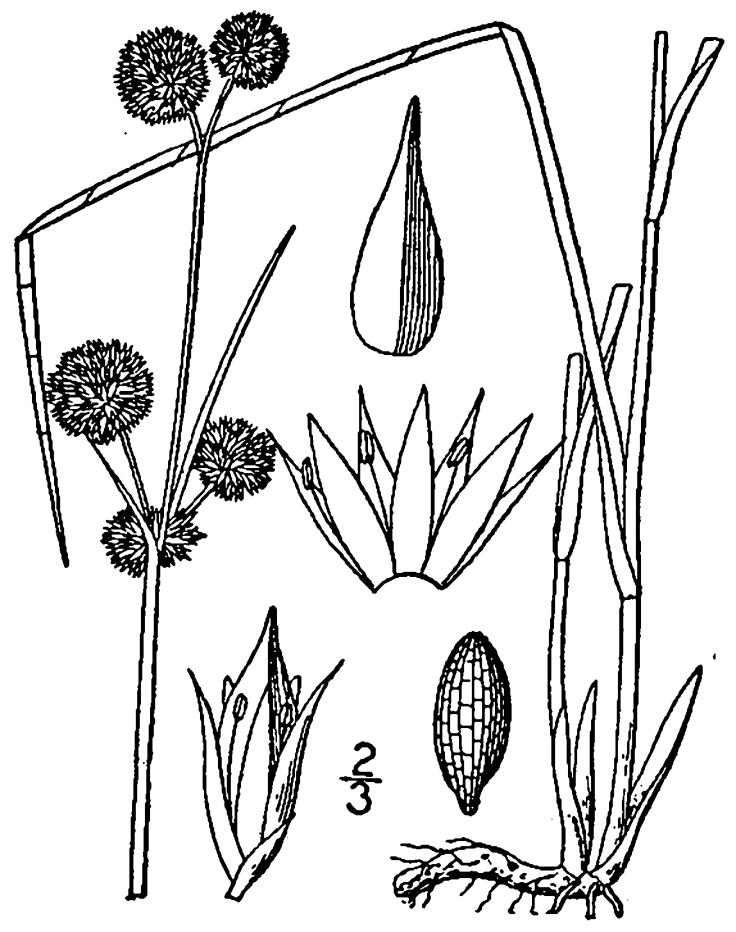
*Juncus scirpoides* (from Britton and Brown 1913).

**Figure 55c.**
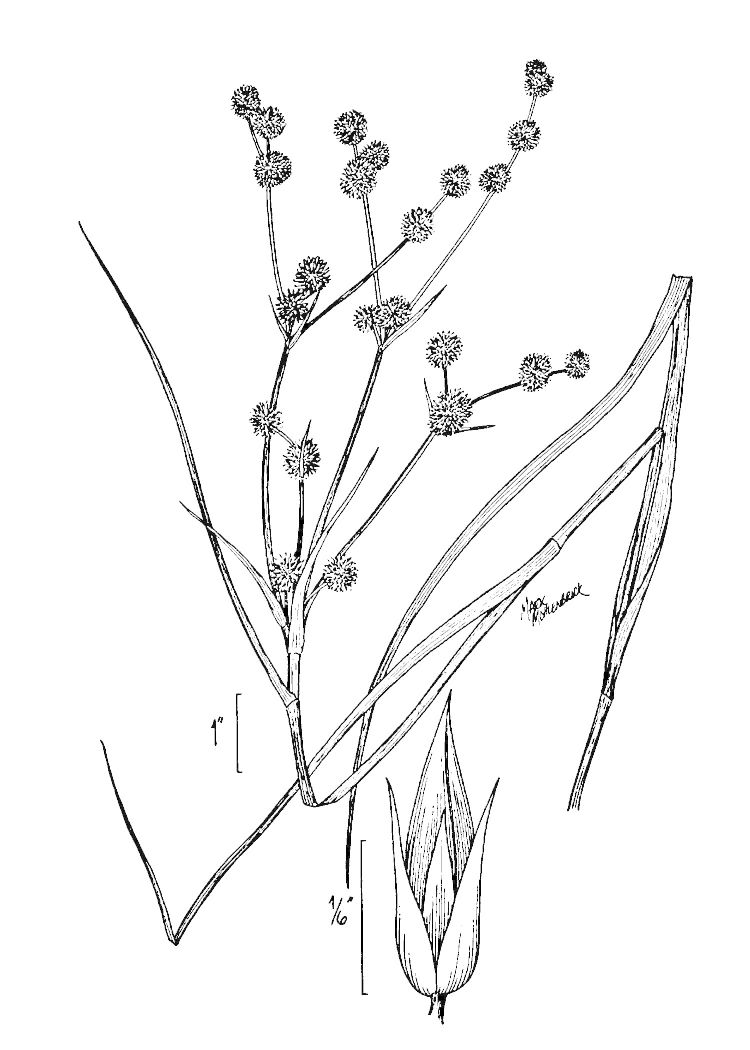
*Juncus validus* (from USDA-NRCS 2012).

**Figure 56.**
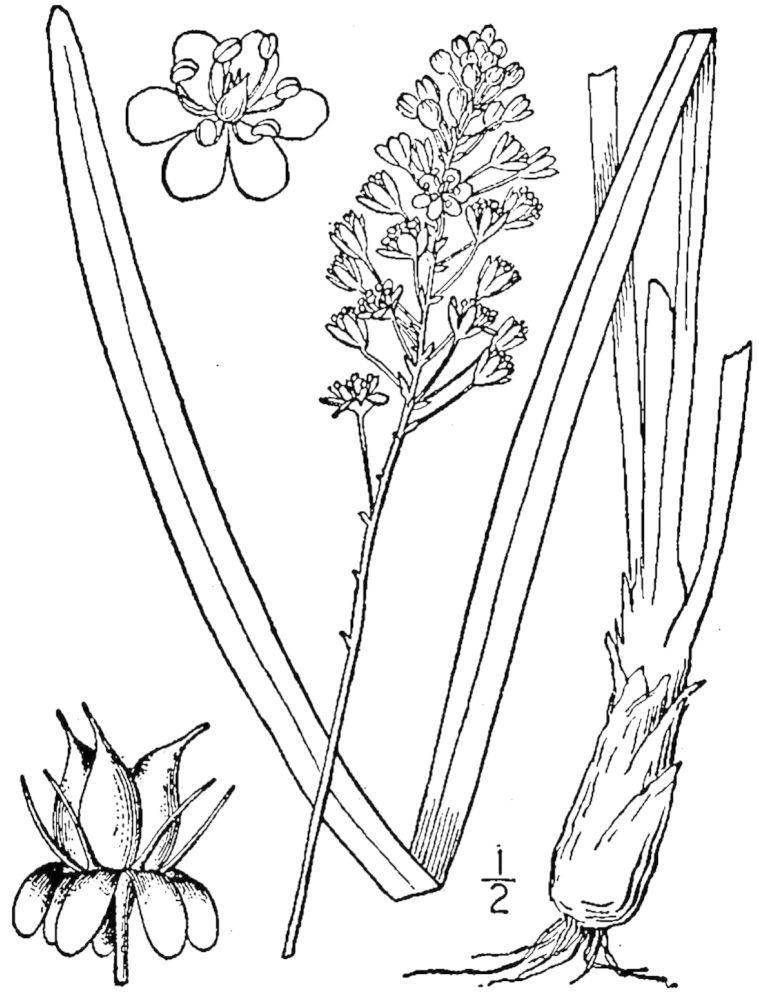
*Amianthium muscitoxicum* (from Britton and Brown 1913).

**Figure 57.**
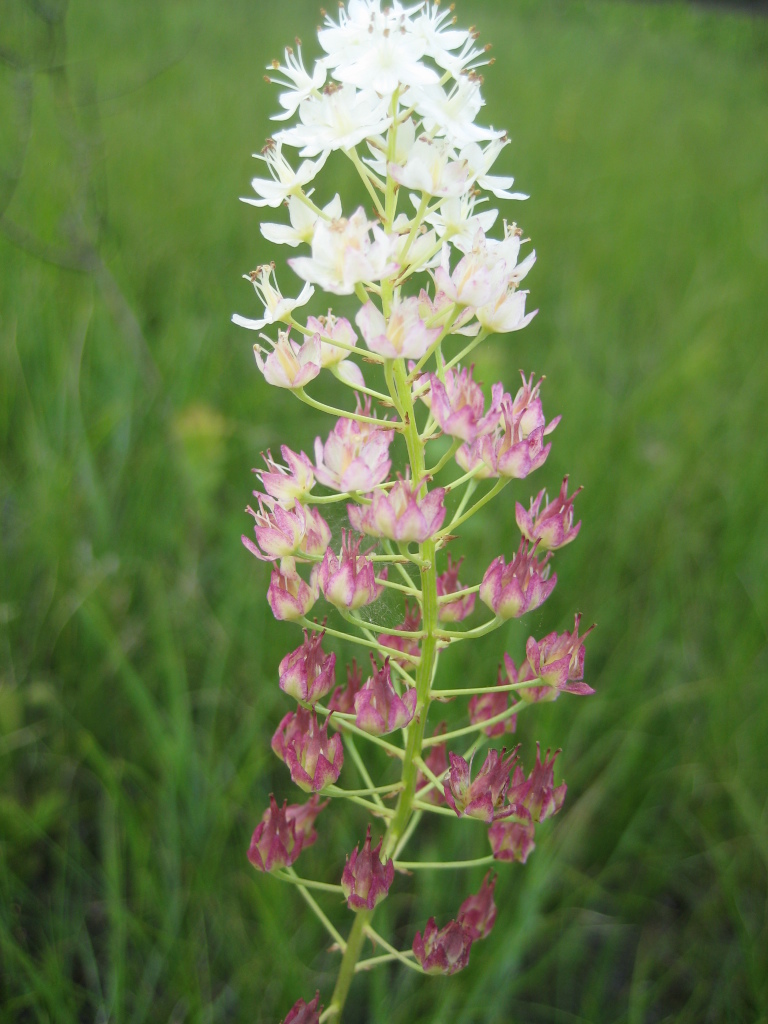
*Stenanthium densum* (photo by R. Thornhill).

**Figure 58a.**
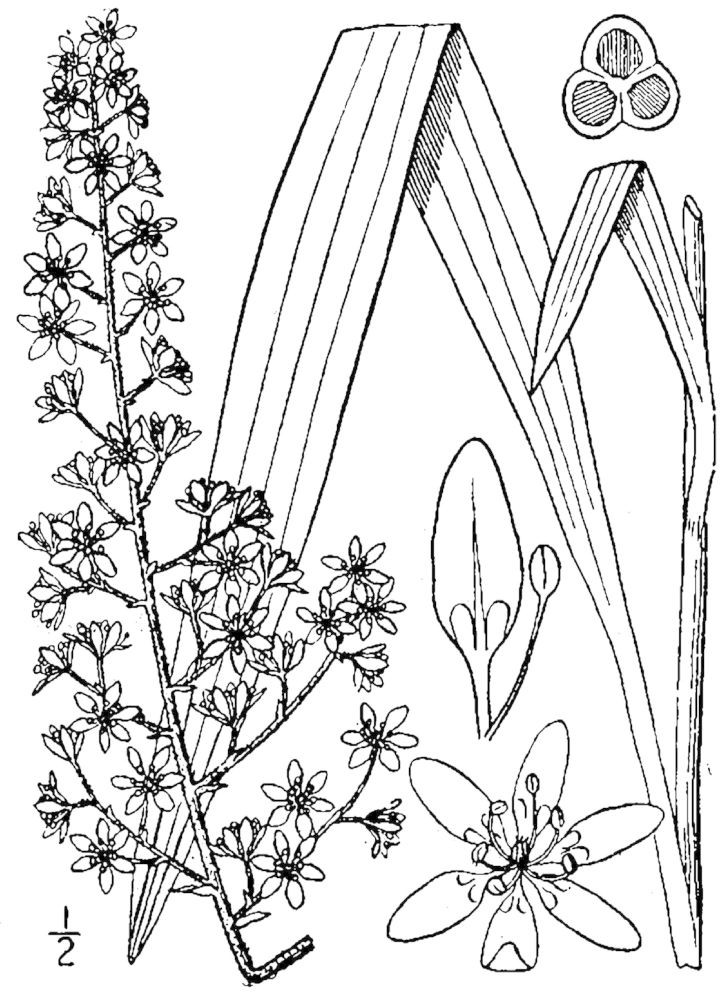
From Britton and Brown (1913).

**Figure 58b.**
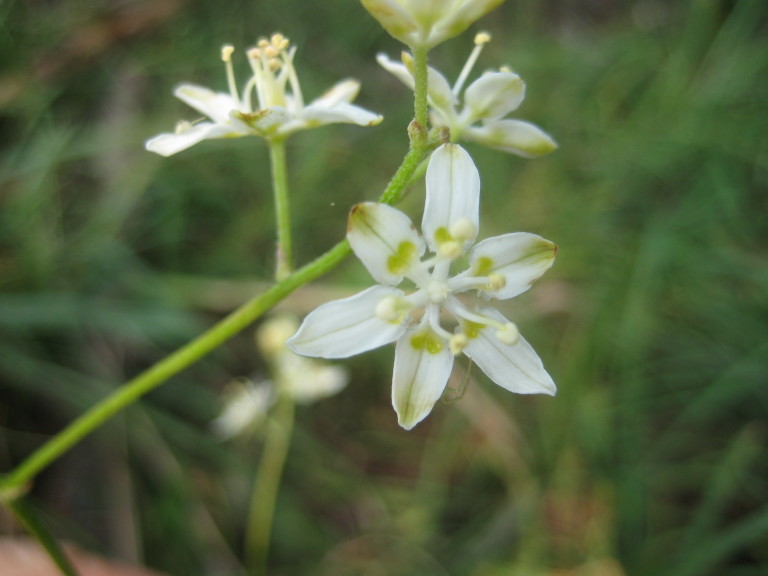
Photo by R. Thornhill.

**Figure 59a.**
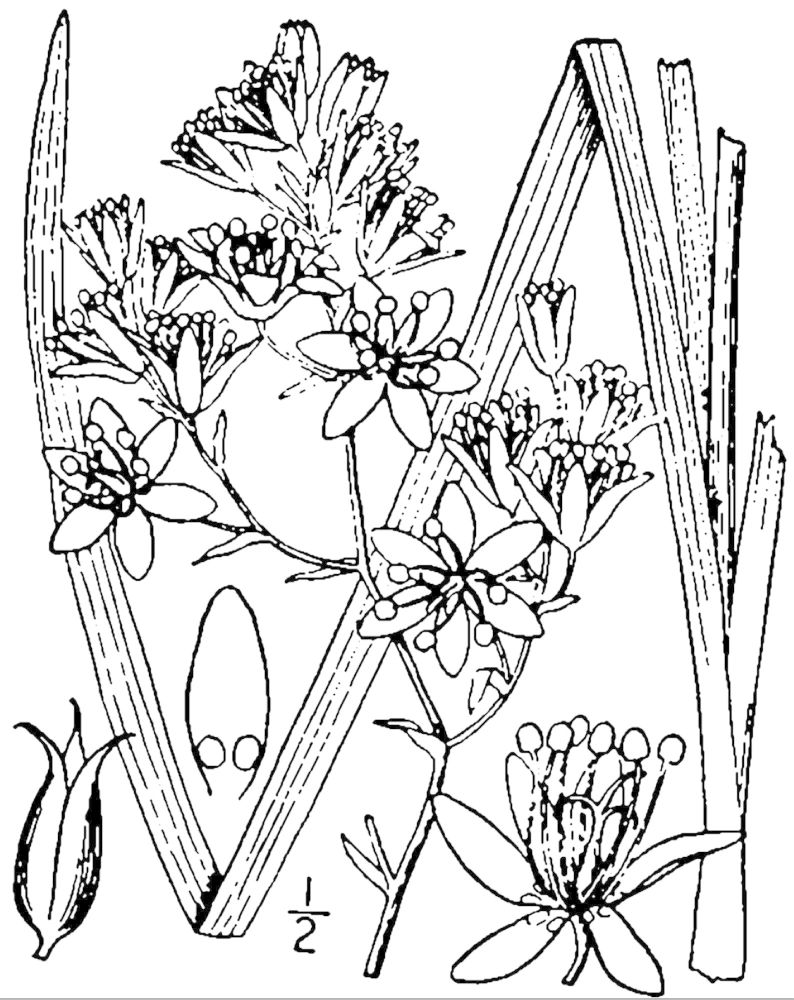
from Britton and Brown (1913)

**Figure 59b.**
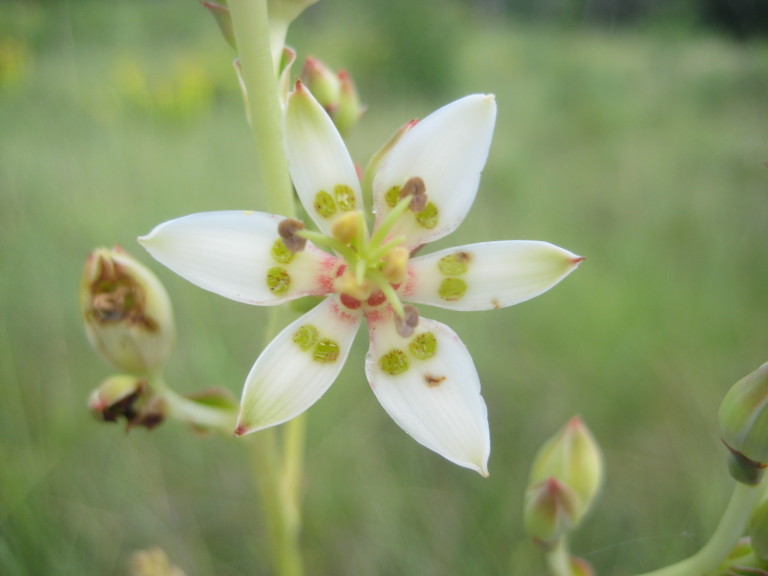


**Figure 60a.**
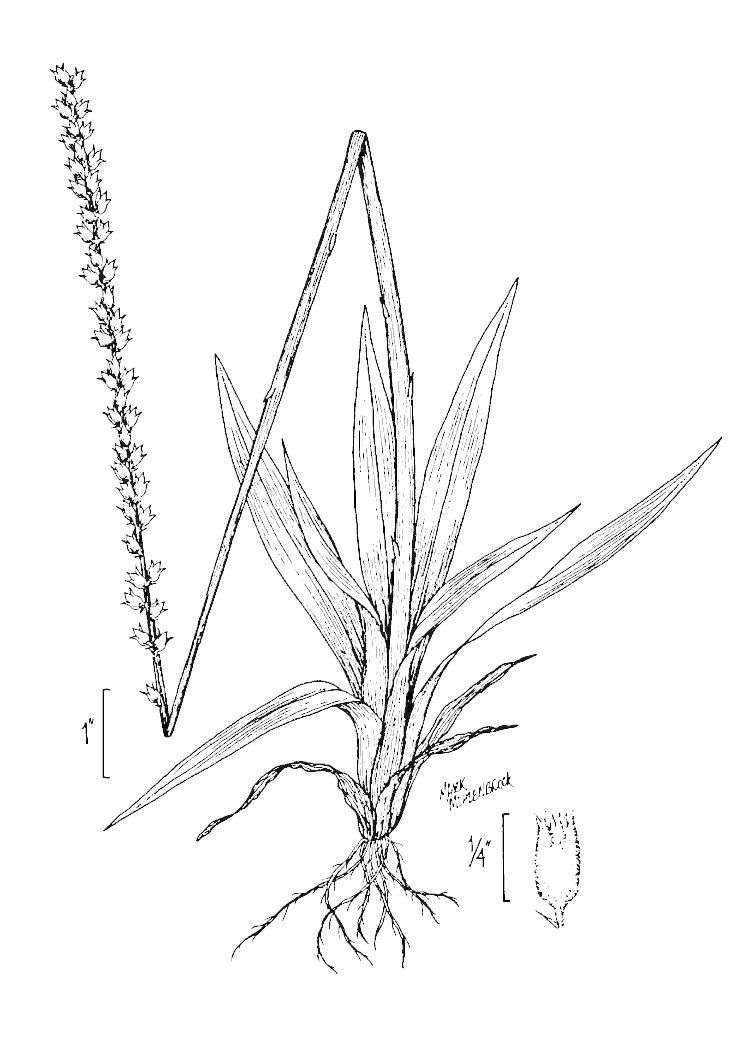
*Aletris aurea* (from USDA-NRCS 2012).

**Figure 60b.**
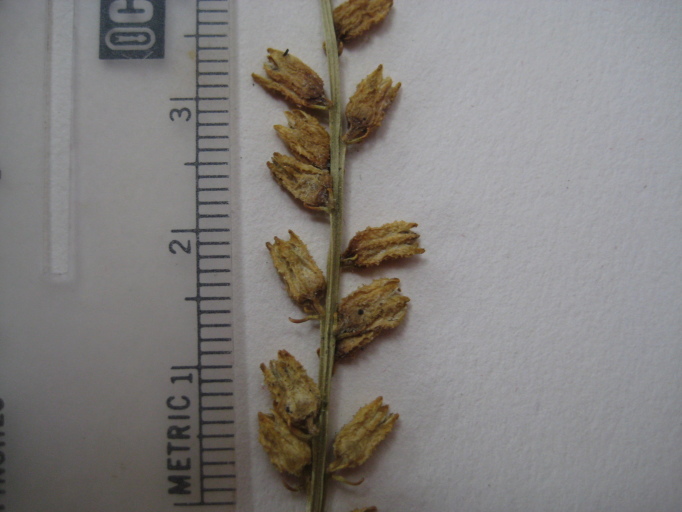
*Aletris aurea* (photo of dried specimen by R. Thornhill).

**Figure 60c.**
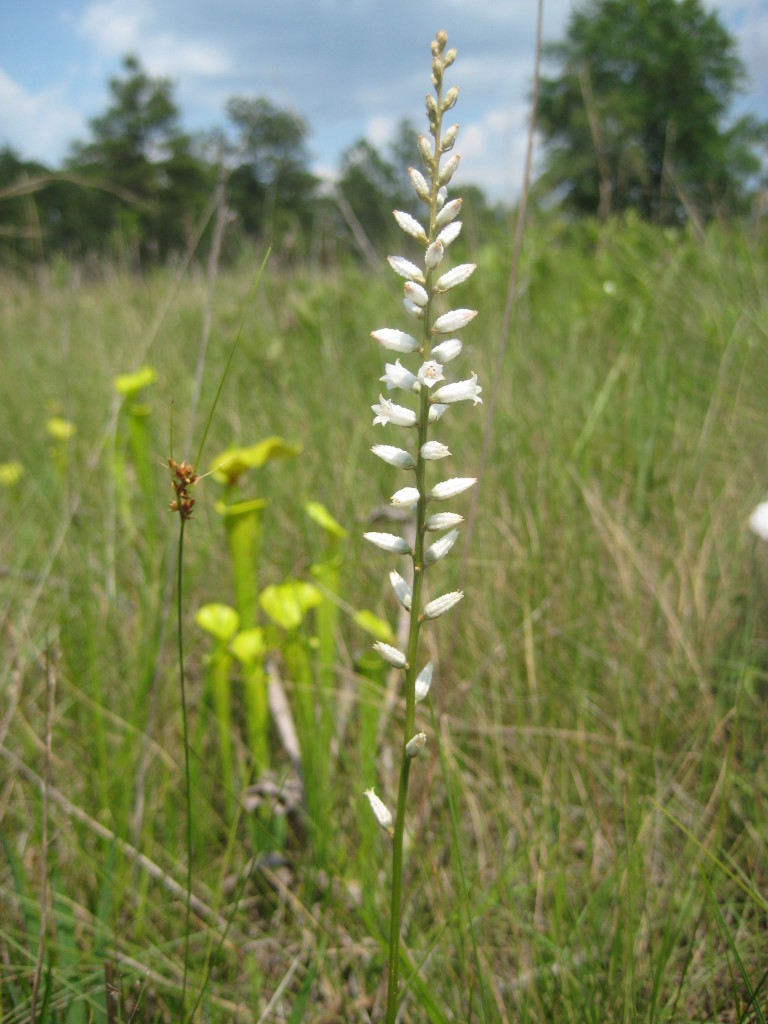
*Aletris farinosa* (photo by R. Thornhill).

**Figure 60d.**
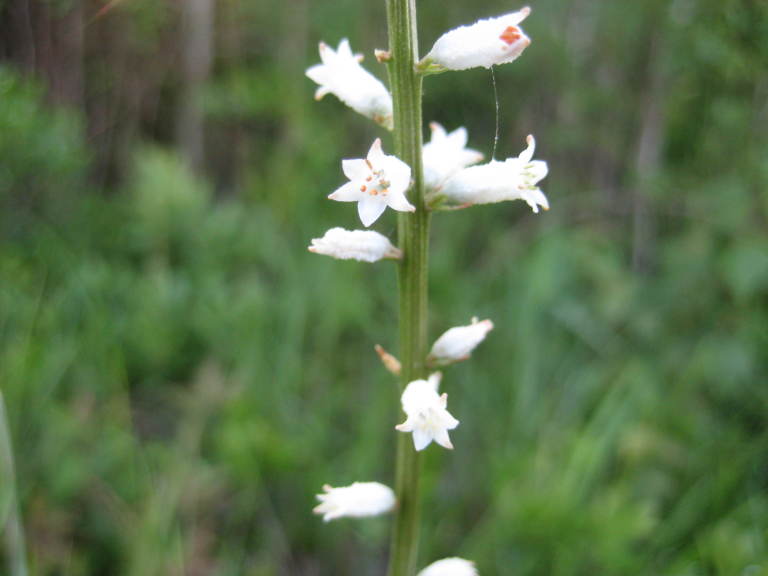
*Aletris farinosa* (photo by R. Thornhill).

**Figure 60e.**
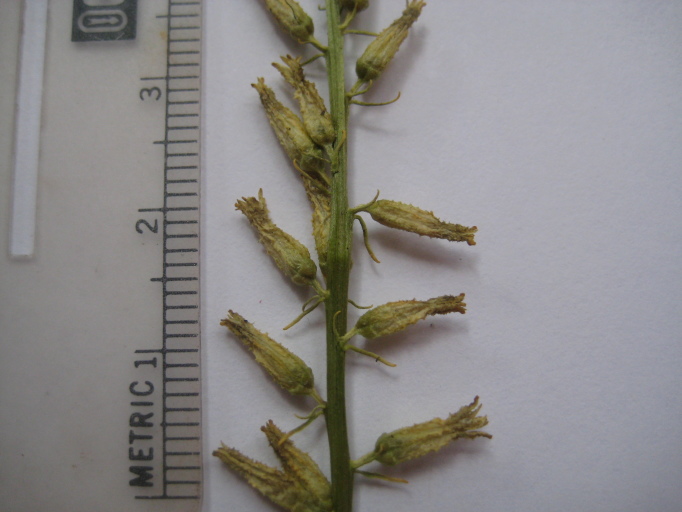
*Aletris lutea* (photo of Thornhill 556 specimen by R. Thornhill).

**Figure 61.**
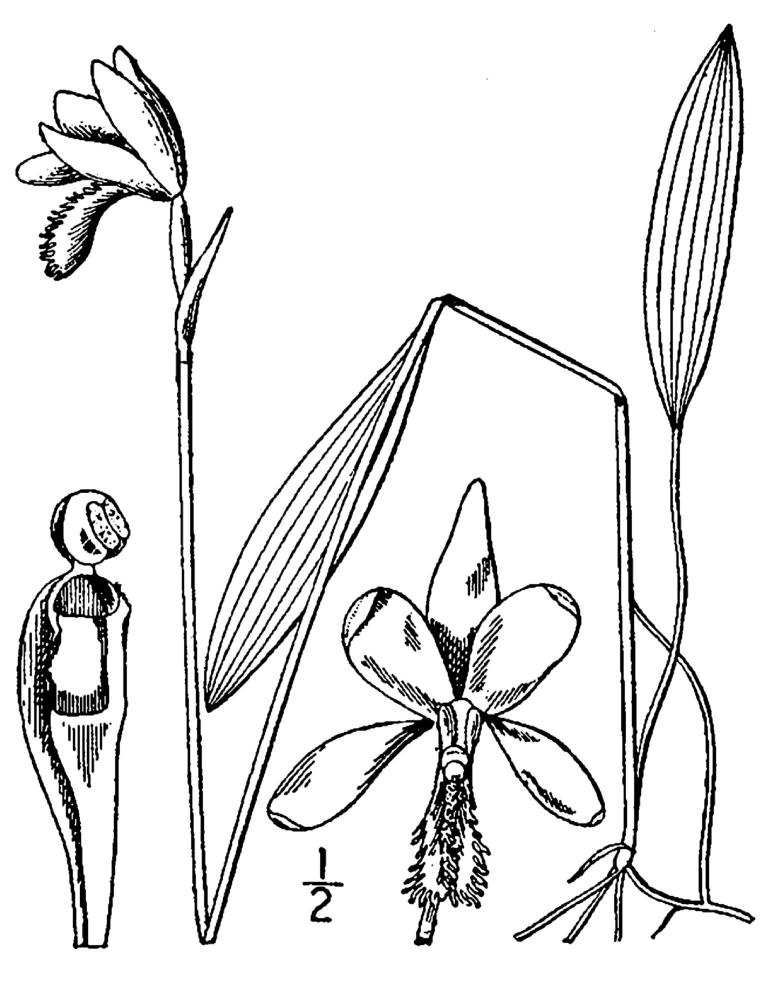
*Pogonia ophioglossoides* (from Britton and Brown 1913).

**Figure 62a.**
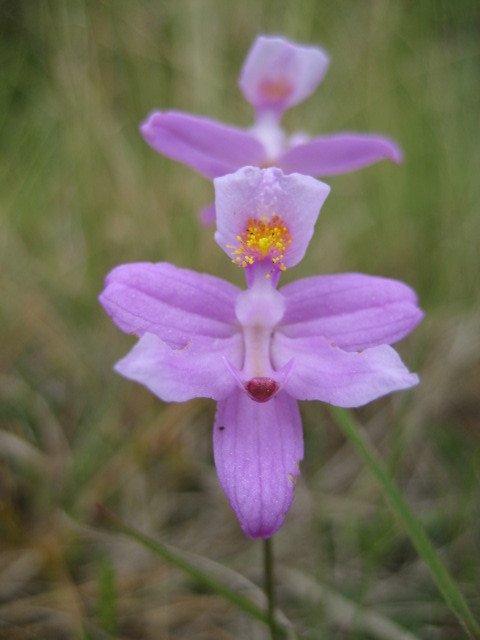
*Calopogon barbatus* (photo by R. Thornhill).

**Figure 62b.**
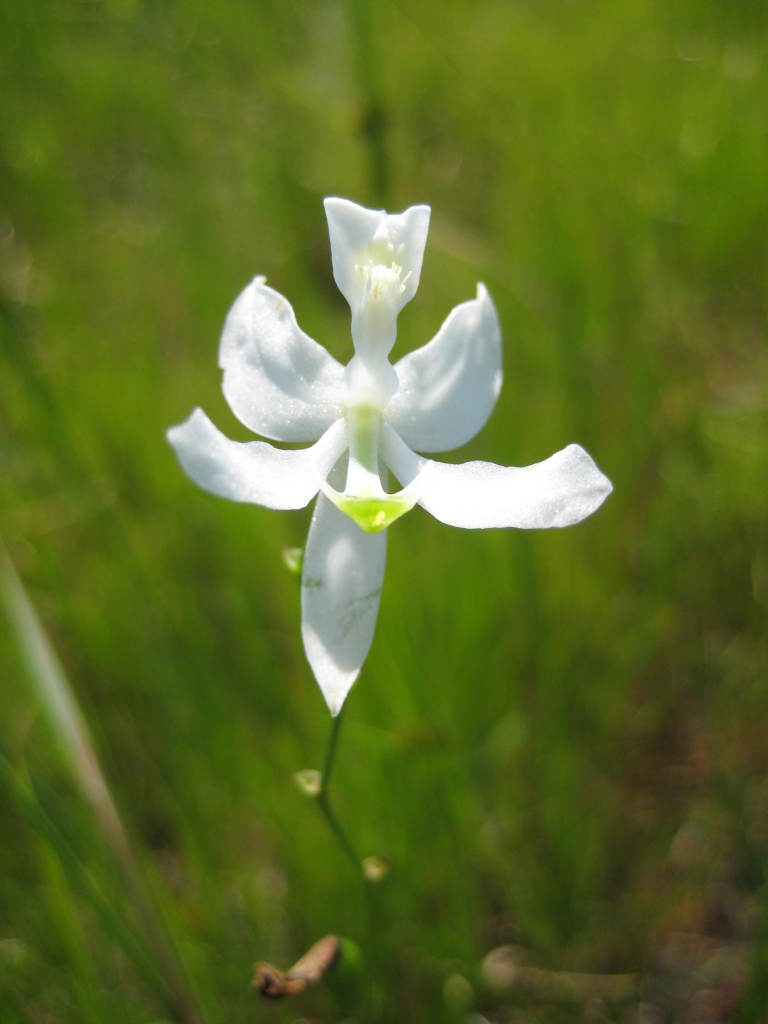
*Calopogon pallidus* (white form; photo by R. Thornhill).

**Figure 62c.**
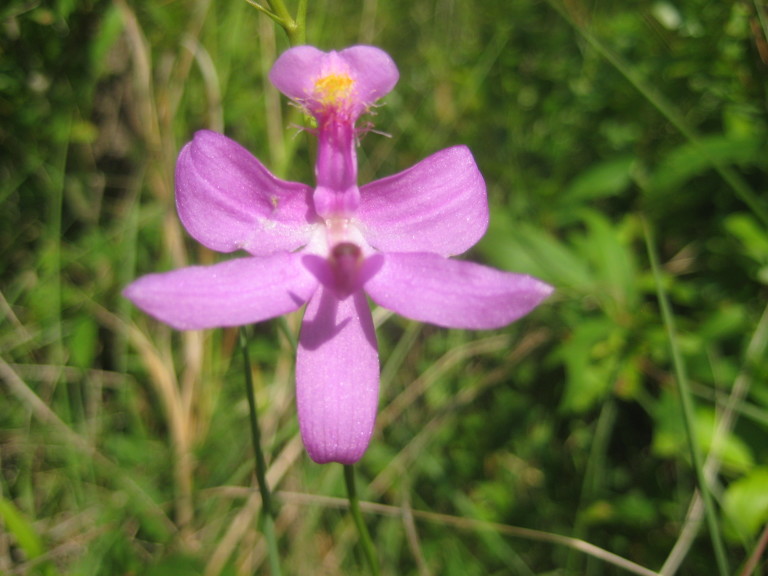
Calopogon tuberosus var. tuberosus (photo by R. Thornhill).

**Figure 63a.**
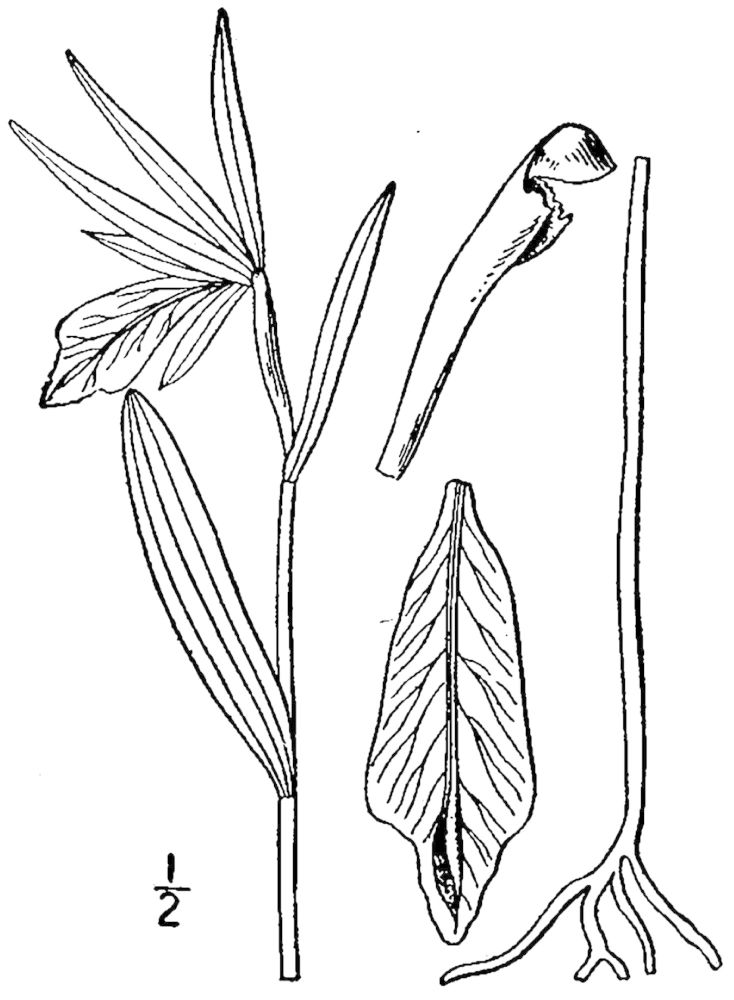
from Britton and Brown (1913).

**Figure 63b.**
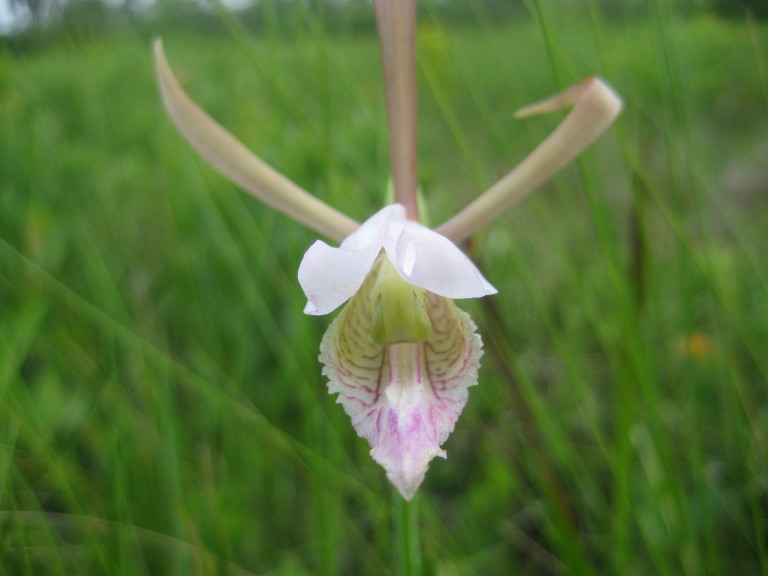


**Figure 64a.**
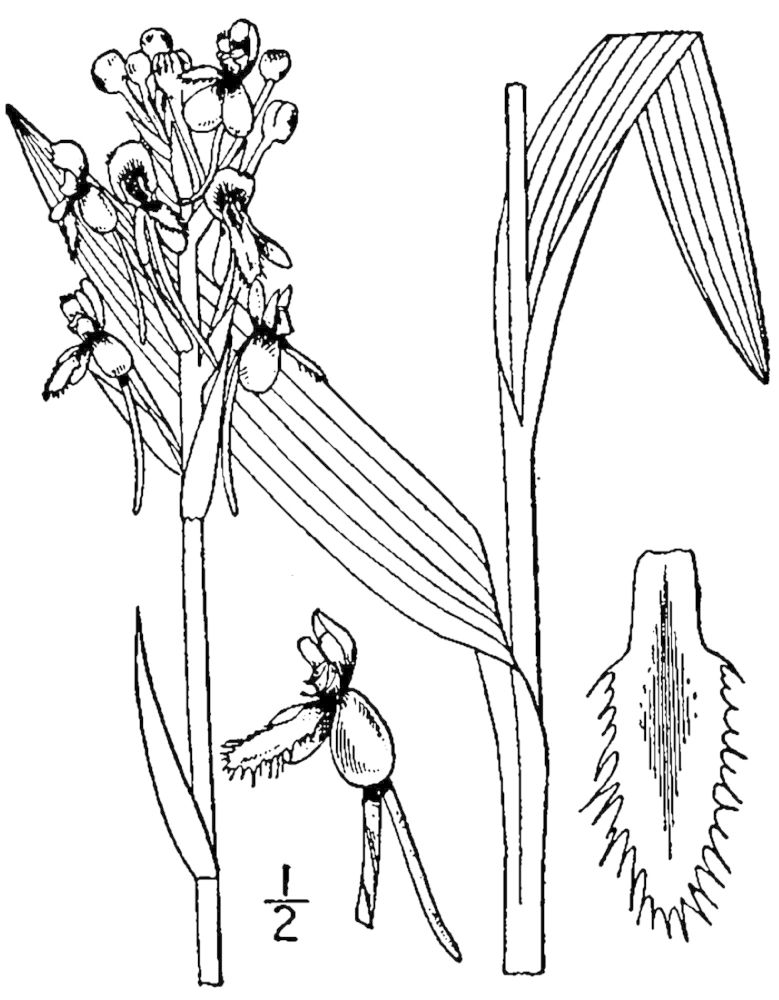
*Platanthera blephariglottis* (from Britton and Brown 1913).

**Figure 64b.**
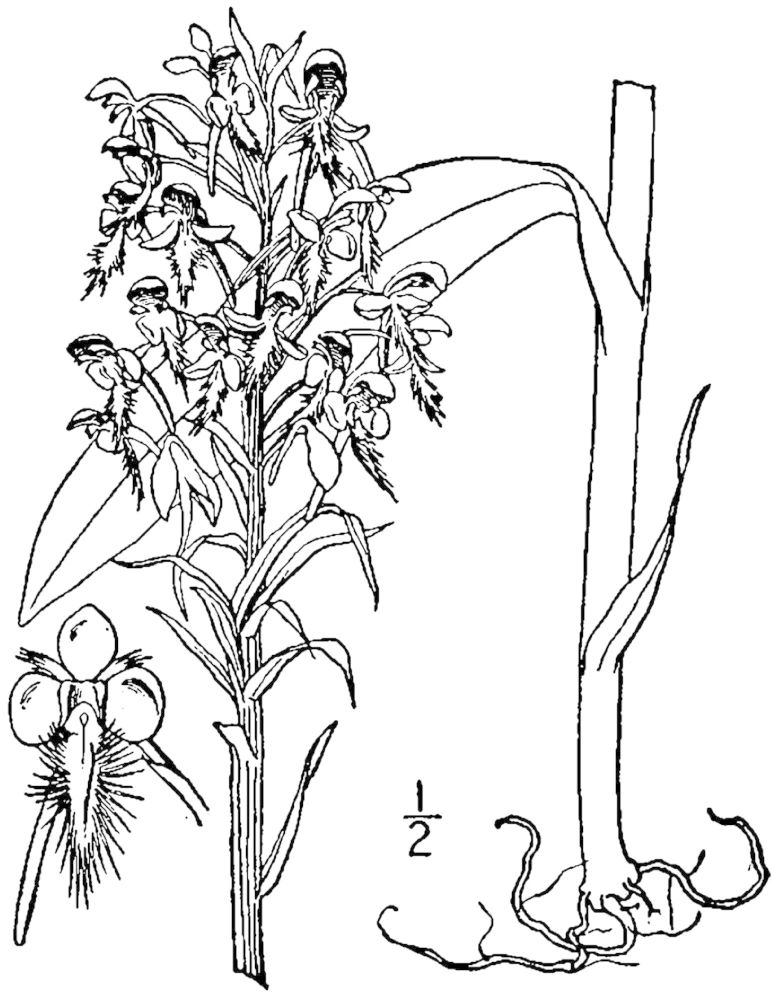
*Platanthera ciliaris* (from Britton and Brown 1913).

**Figure 64c.**
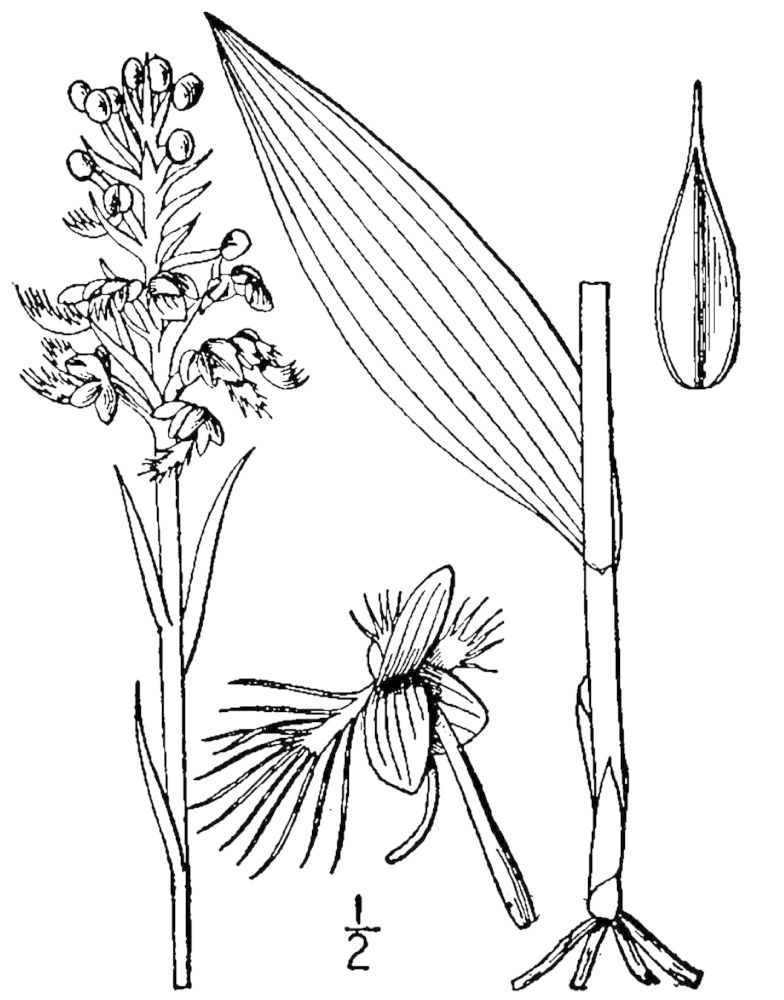
*Platanthera cristata* (from Britton and Brown 1913).

**Figure 64d.**
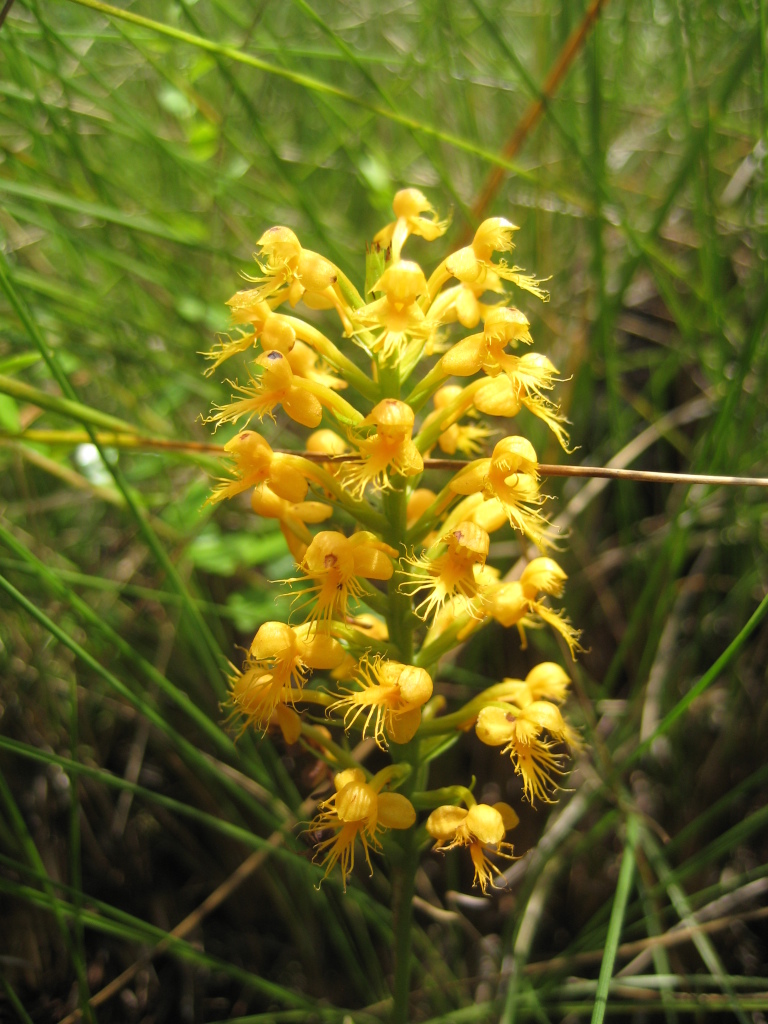
*Platanthera cristata* (photo by R. Thornhill).

**Figure 64e.**
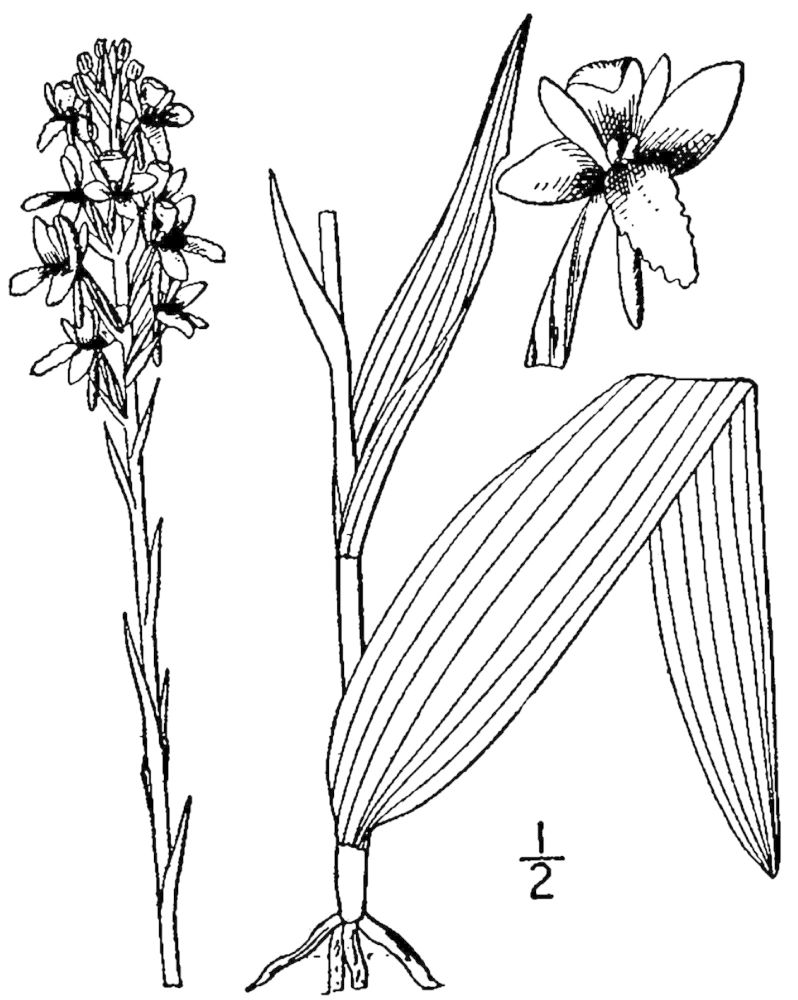
*Platanthera integra* (from Britton and Brown 1913).

**Figure 64f.**
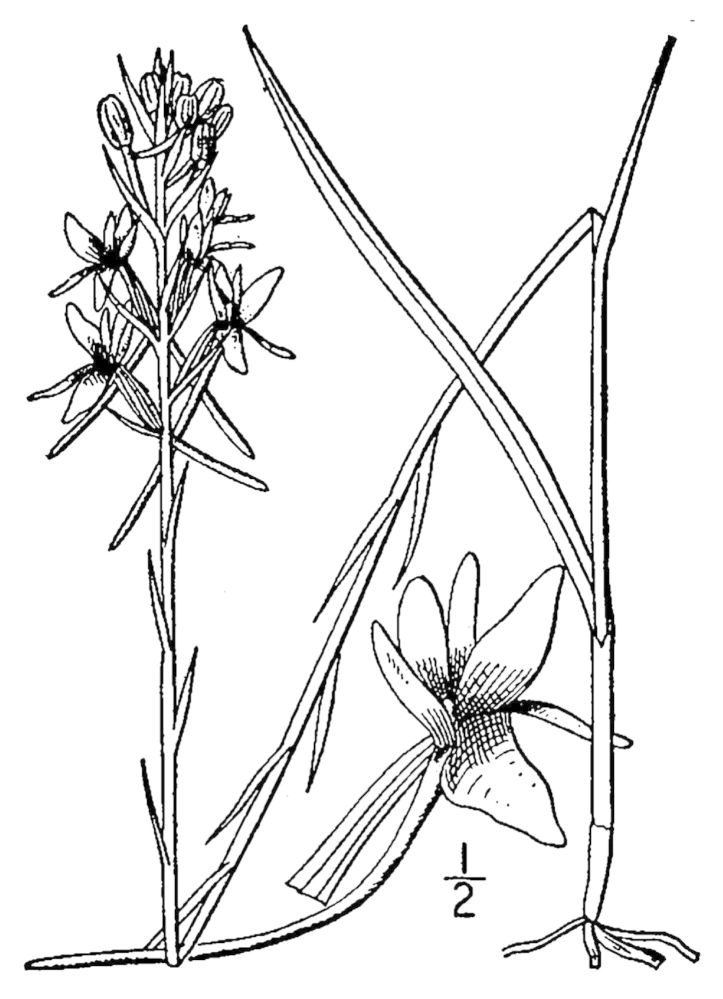
*Platanthera nivea* (from Britton and Brown 1913).

**Figure 65a.**
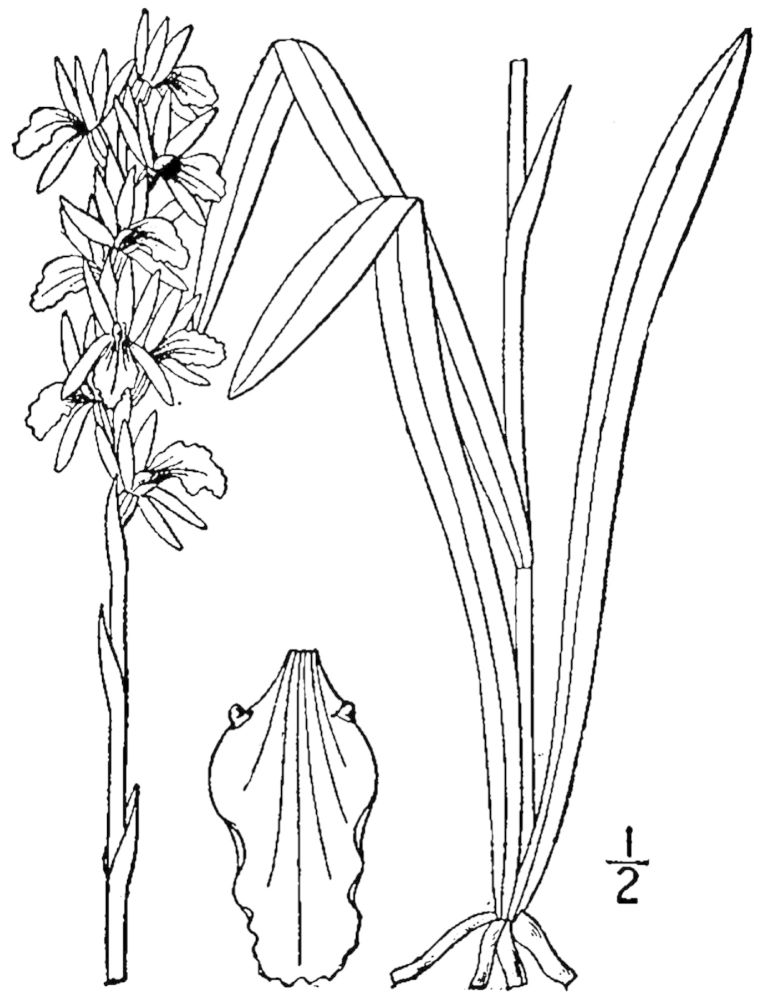
*Spiranthes cernua* (from Britton and Brown 1913).

**Figure 65b.**
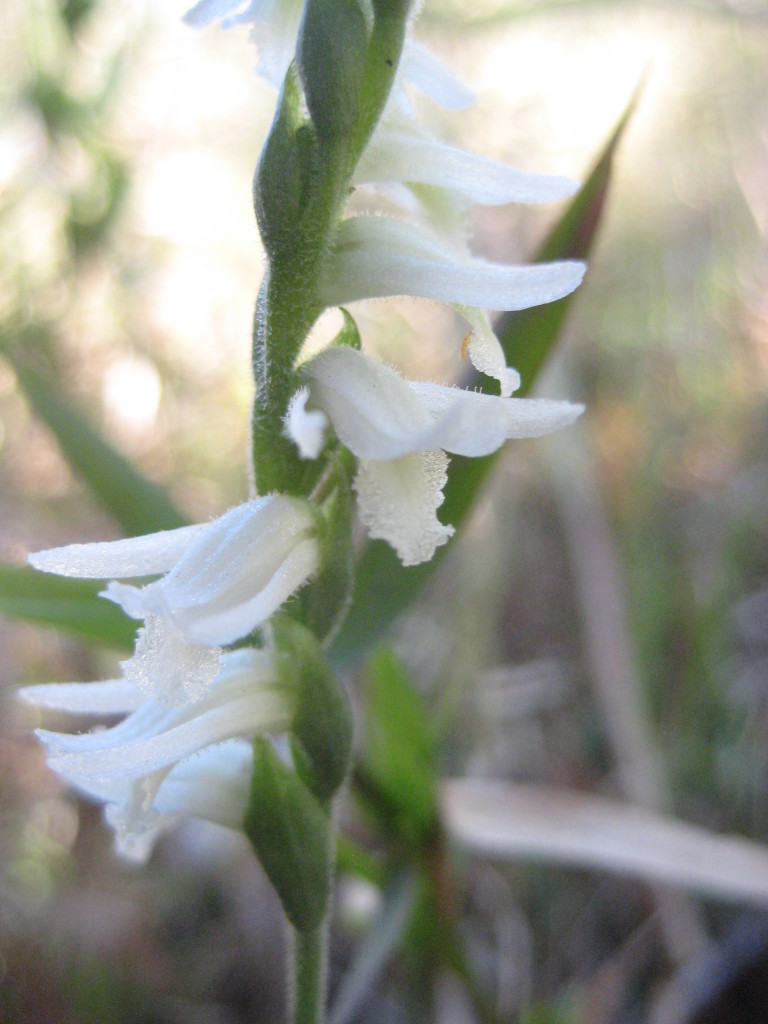
*Spiranthes cernua* (photo by R. Thornhill).

**Figure 65c.**
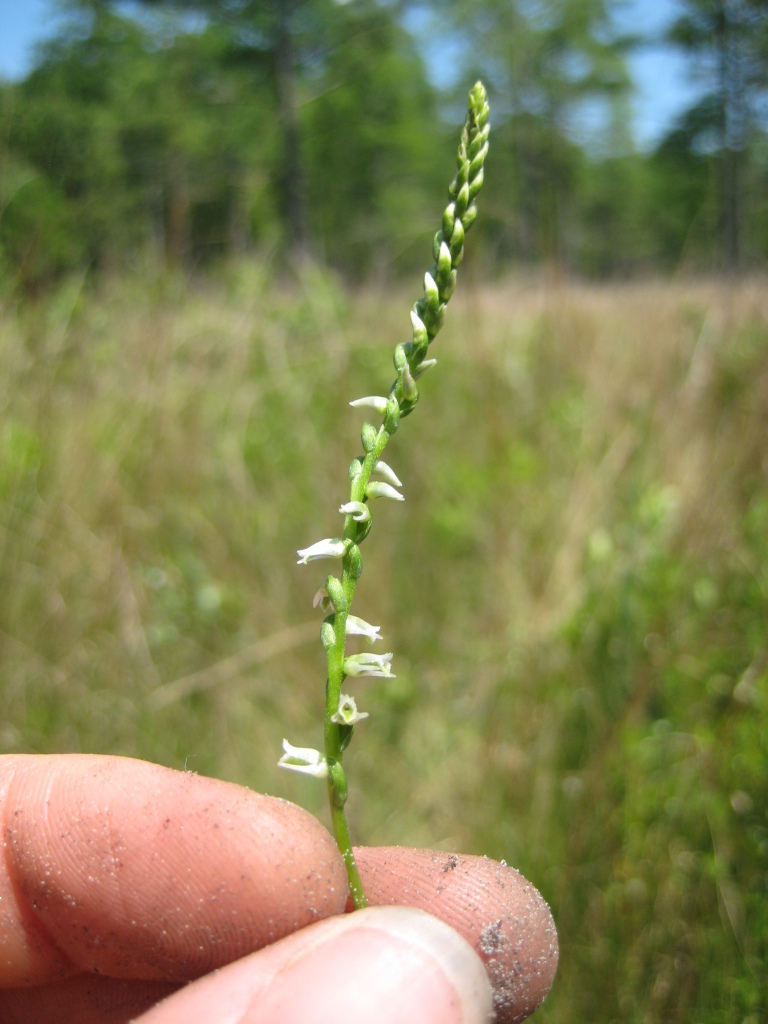
*Spiranthes eatonii* (photo by R. Thornhill).

**Figure 65d.**
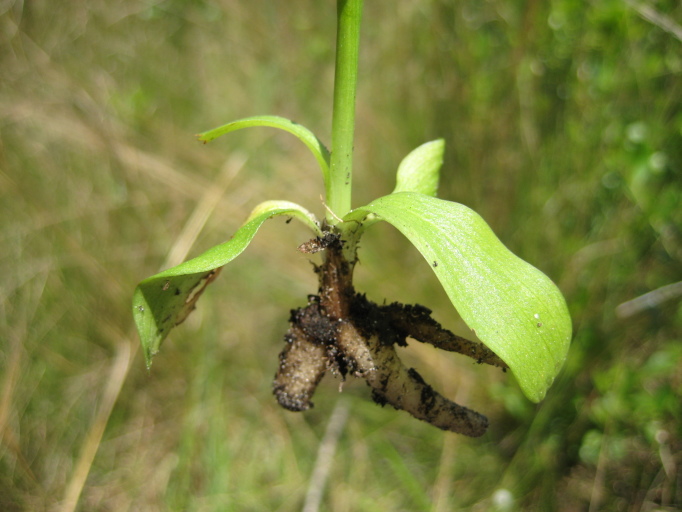
*Spiranthes eatonii* (photo by R. Thornhill).

**Figure 66a.**
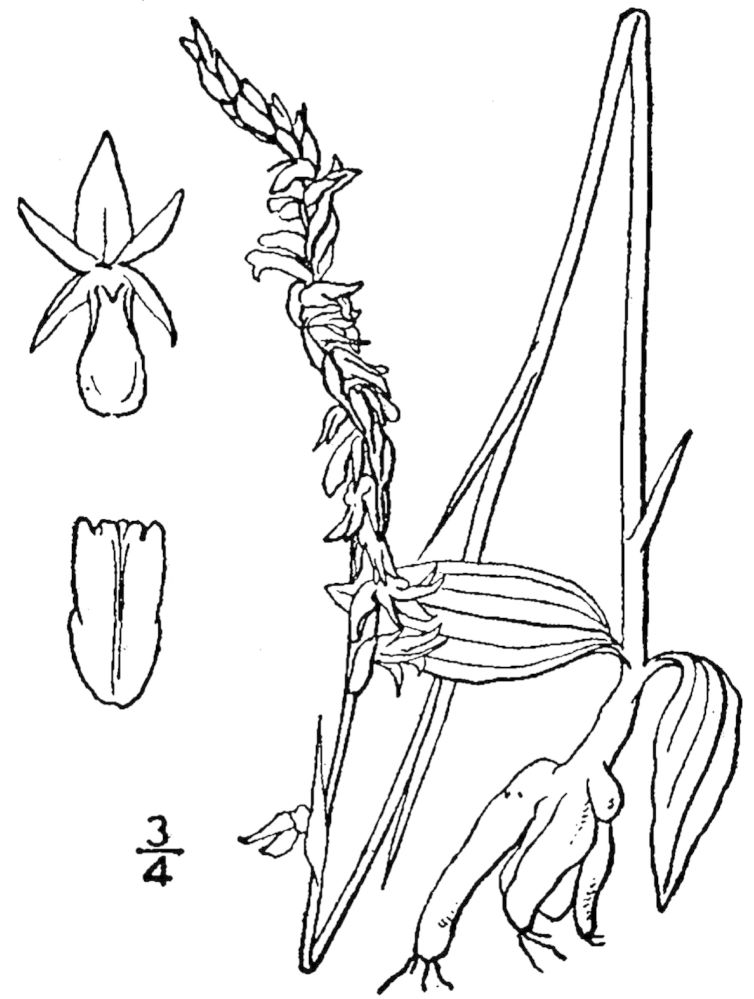
Spiranthes lacera var. gracilis (from Britton and Brown 1913).

**Figure 66b.**
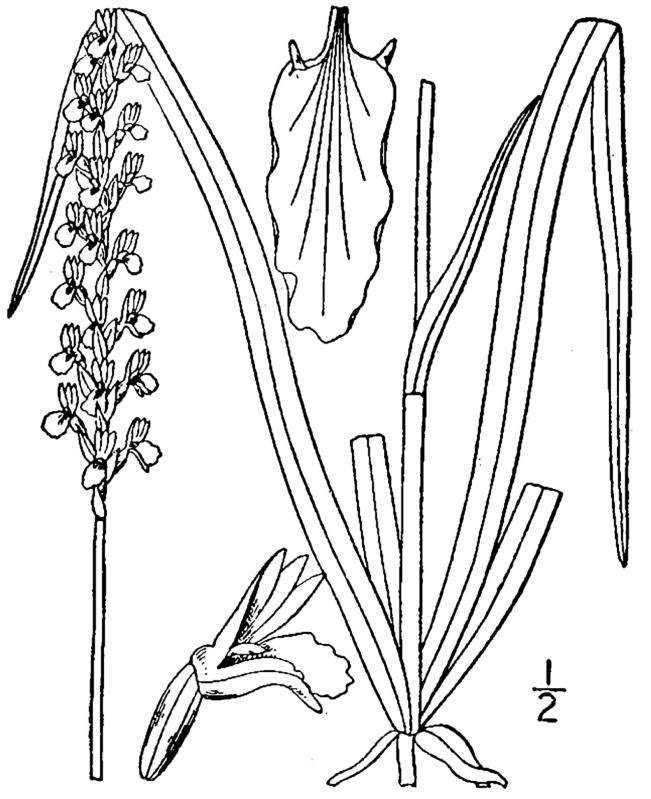
*Spiranthes praecox* (from Britton and Brown 1913).

**Figure 66c.**
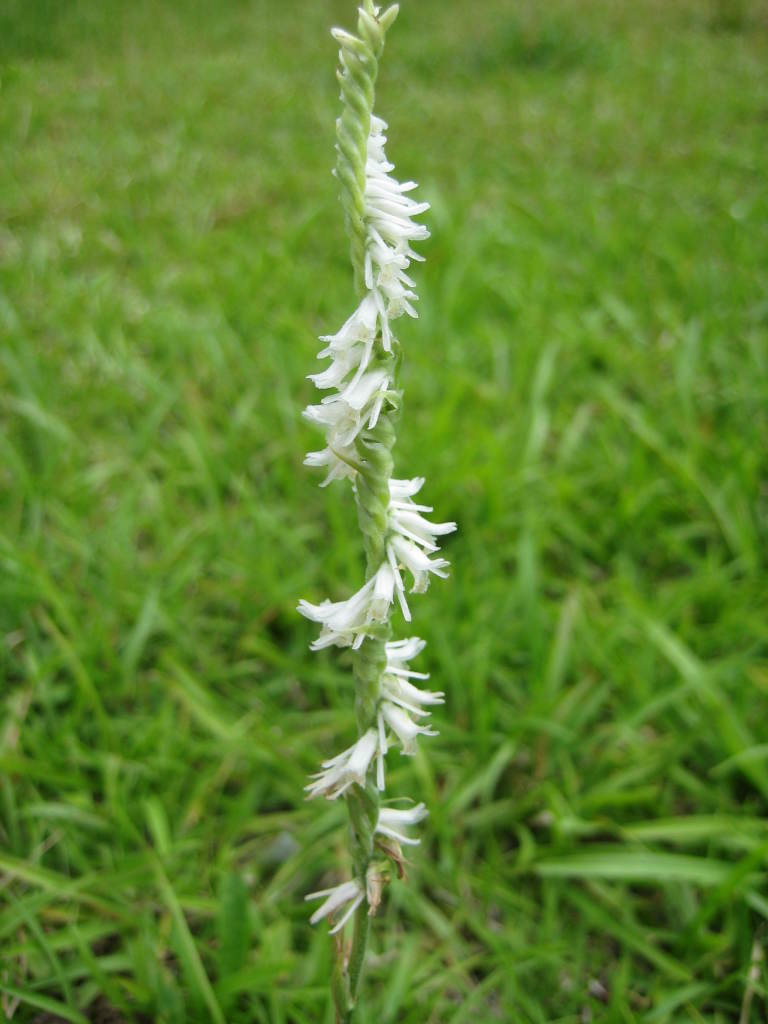
*Spiranthes vernalis* (photo by R. Thornhill).

**Figure 66d.**
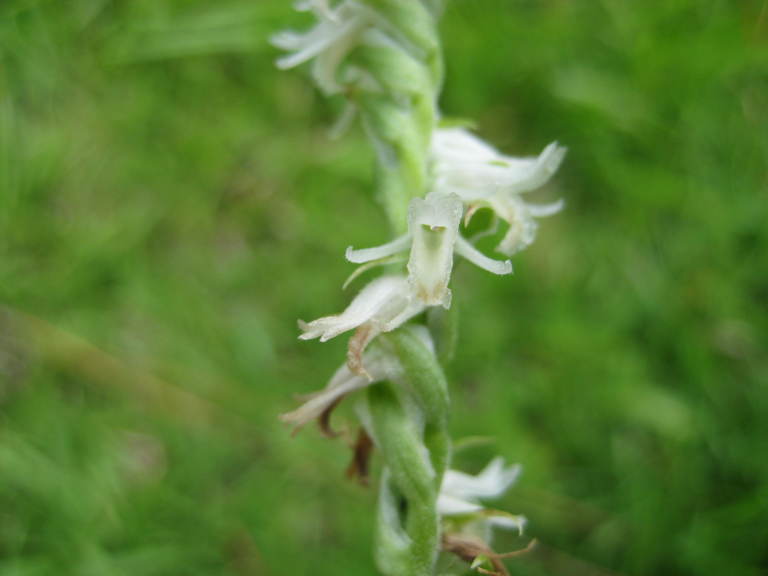
*Spiranthes vernalis* (photo by R. Thornhill).

**Figure 67.**
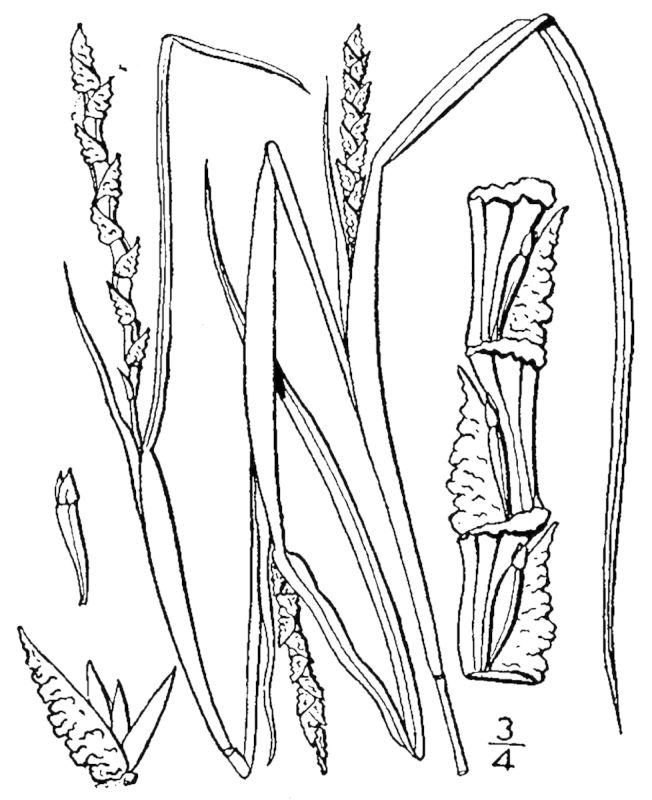
*Coelorachis rugosa* (from Britton and Brown 1913).

**Figure 68.**
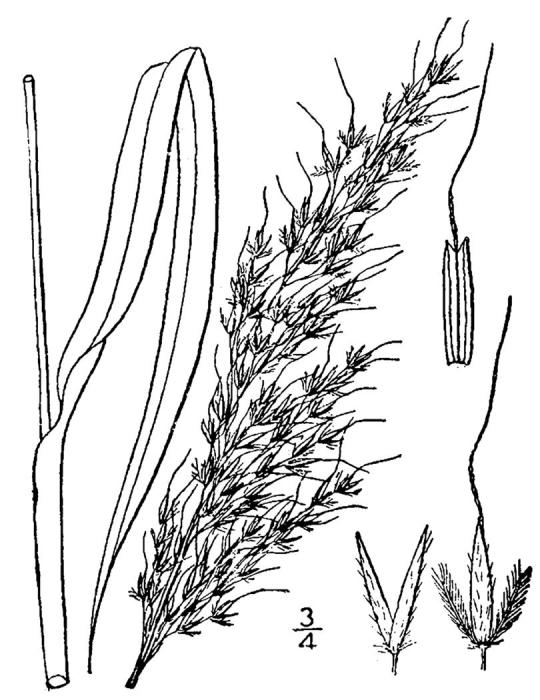
*Sorghastrum nutans* (from Britton and Brown 1913).

**Figure 69.**
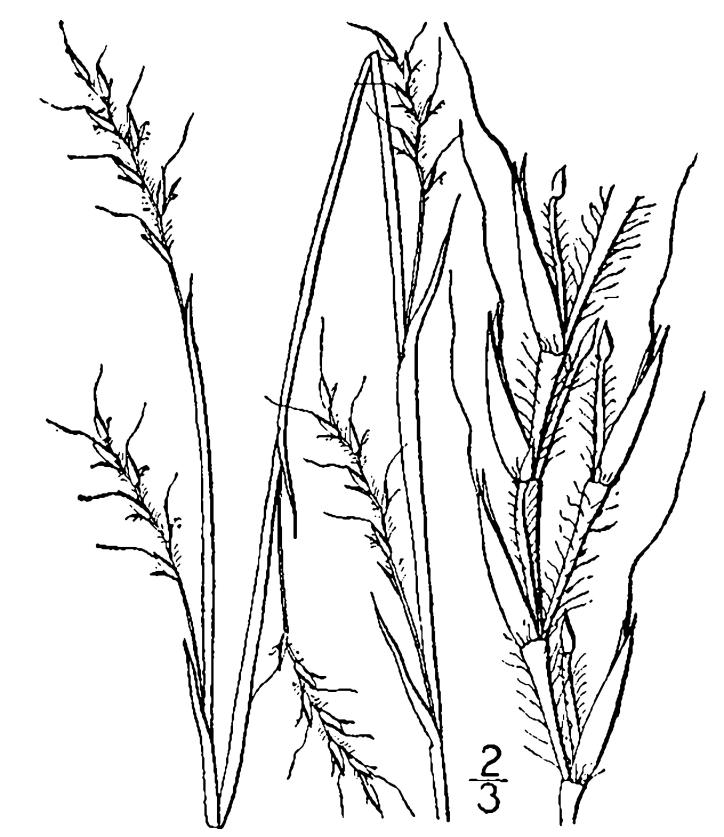
*Schizachyrium scoparium* (from Britton and Brown 1913).

**Figure 70.**
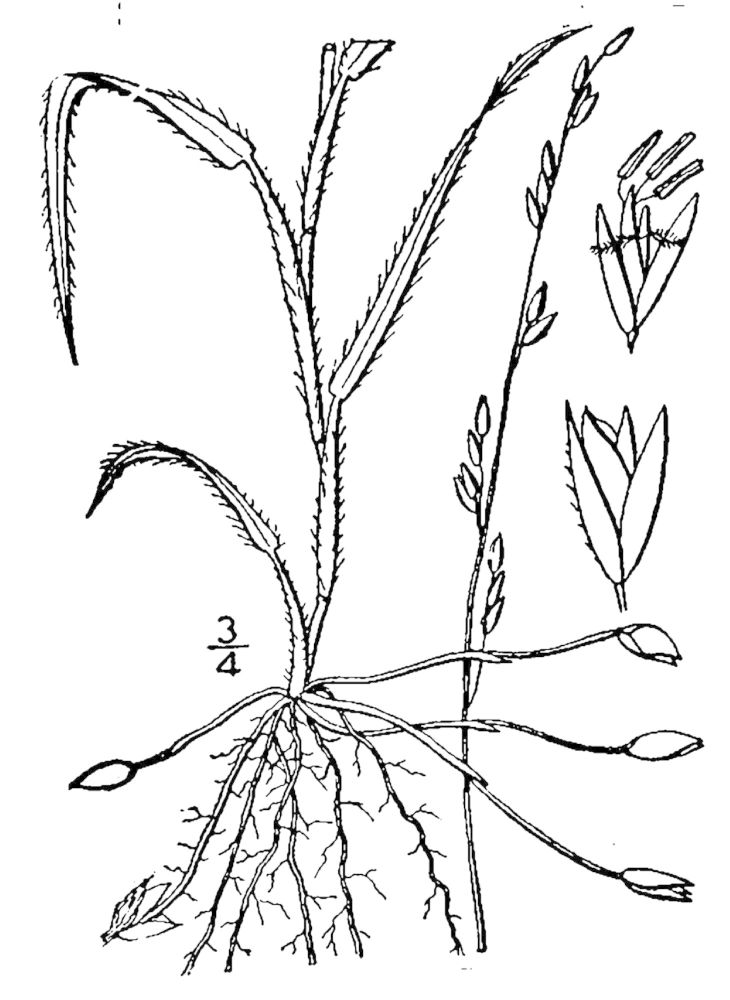
*Amphicarpum amphicarpon* (from Britton and Brown 1913).

**Figure 71.**
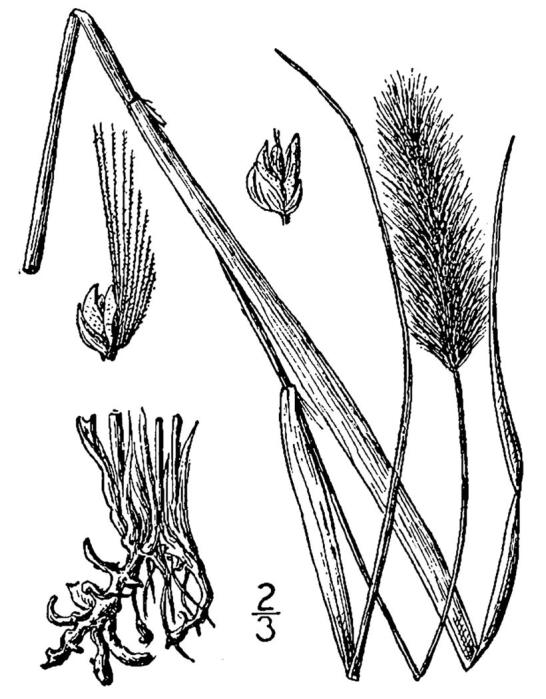
*Setaria parviflora* (from Britton and Brown 1913).

**Figure 72.**
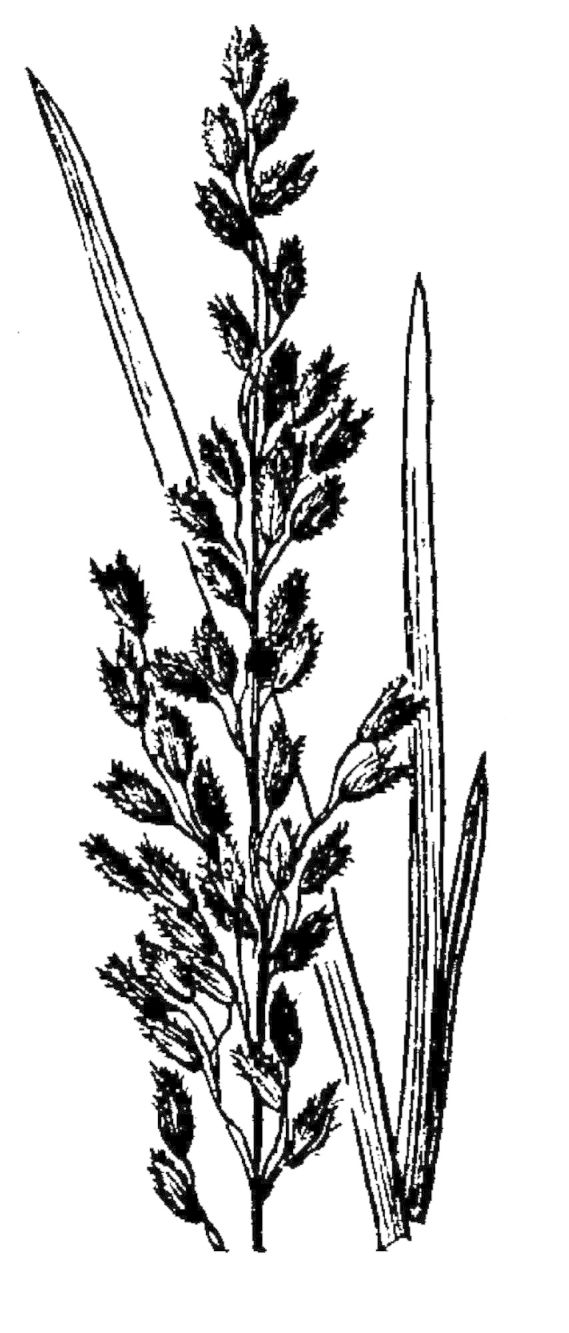
*Anthenantia rufa* (from Hitchcock 1950).

**Figure 73a.**
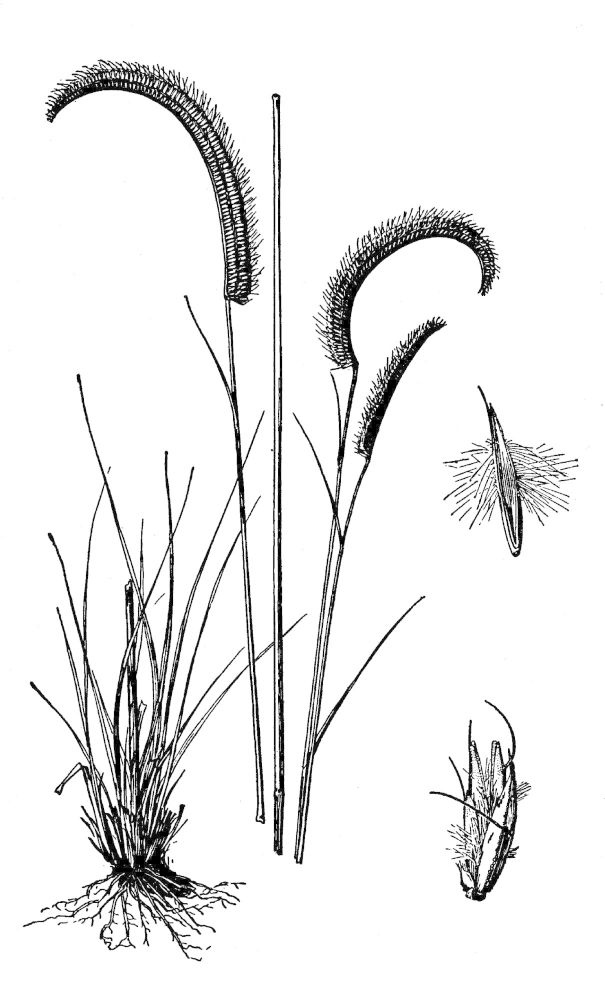
From Britton and Brown (1913).

**Figure 73b.**
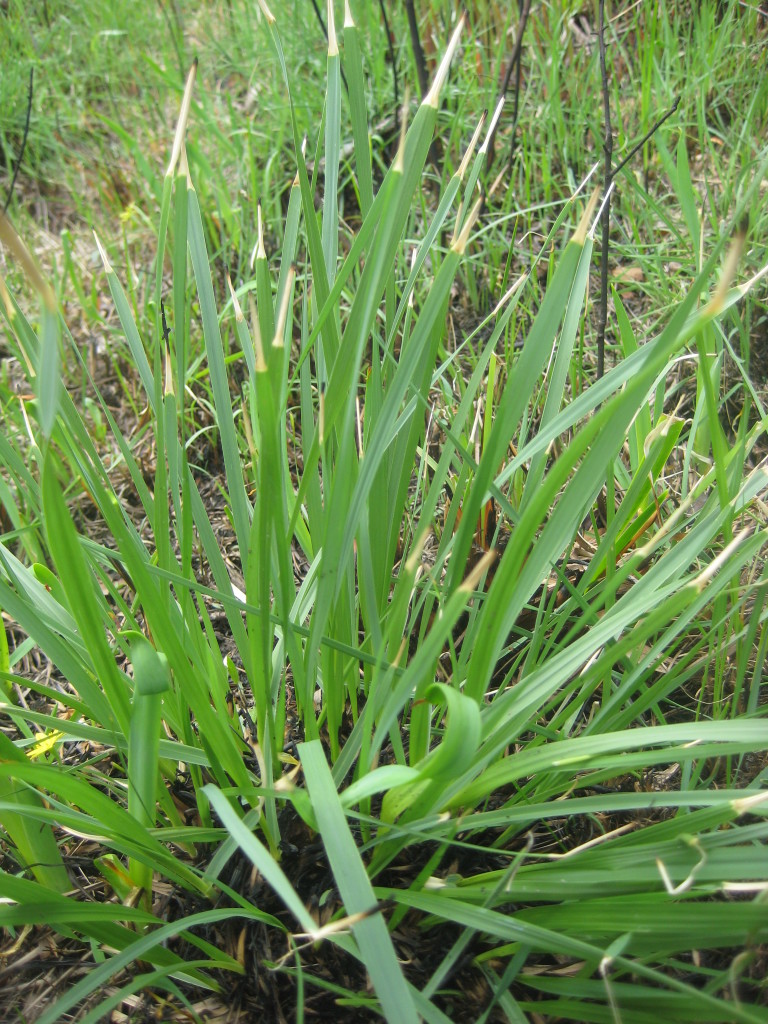
Note the bicolored, fairly broad leaves (photo by R. Thornhill).

**Figure 73c.**
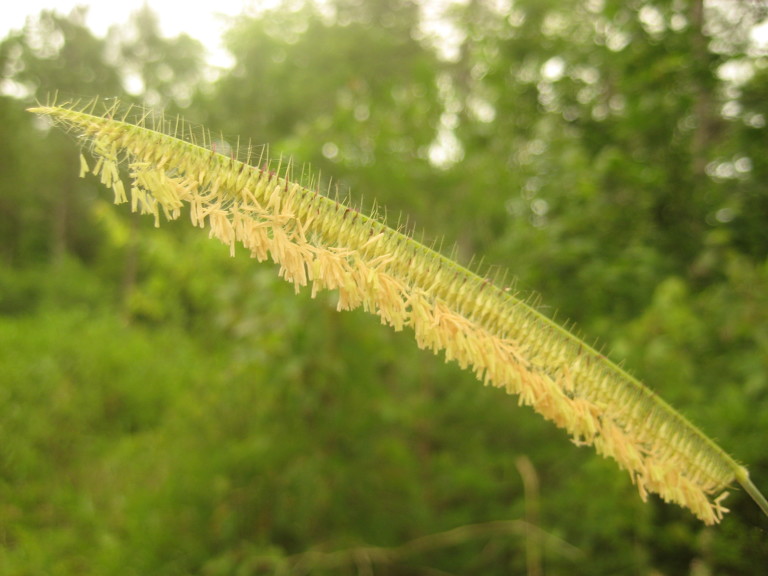
Spike at anthesis (photo by R. Thornhill).

**Figure 73d.**
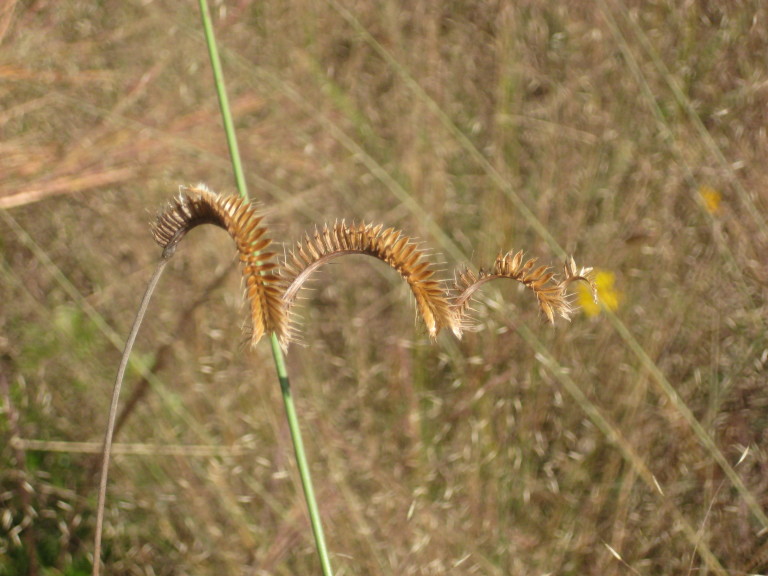
Most spikes coil as they age (photo by R. Thornhill).

**Figure 74.**
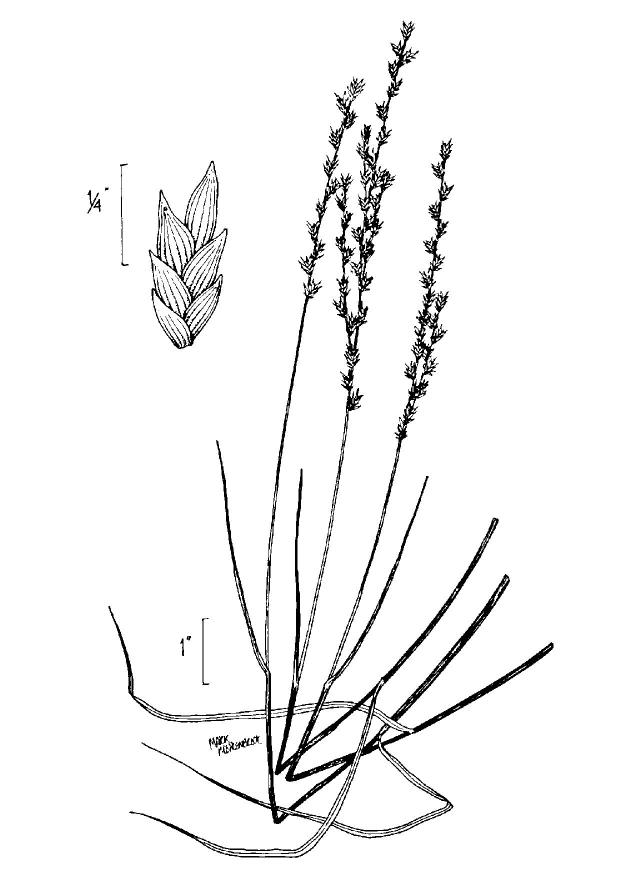
*Chasmanthium laxum* (from USDA-NRCS 2012).

**Figure 75.**
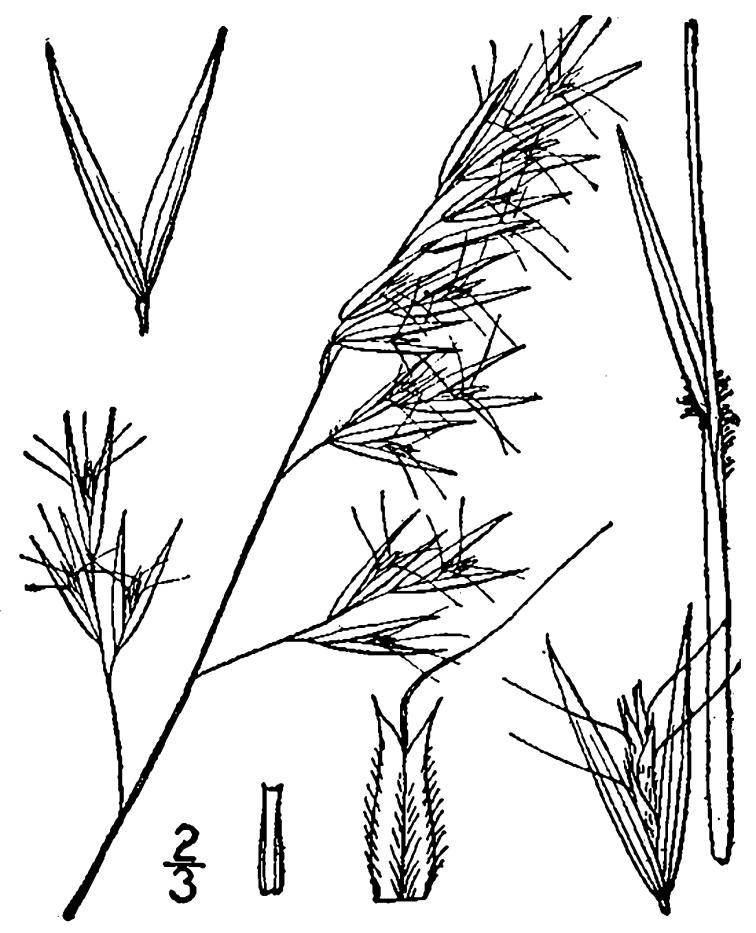
*Danthonia sericea* (from Britton and Brown 1913).

**Figure 76.**
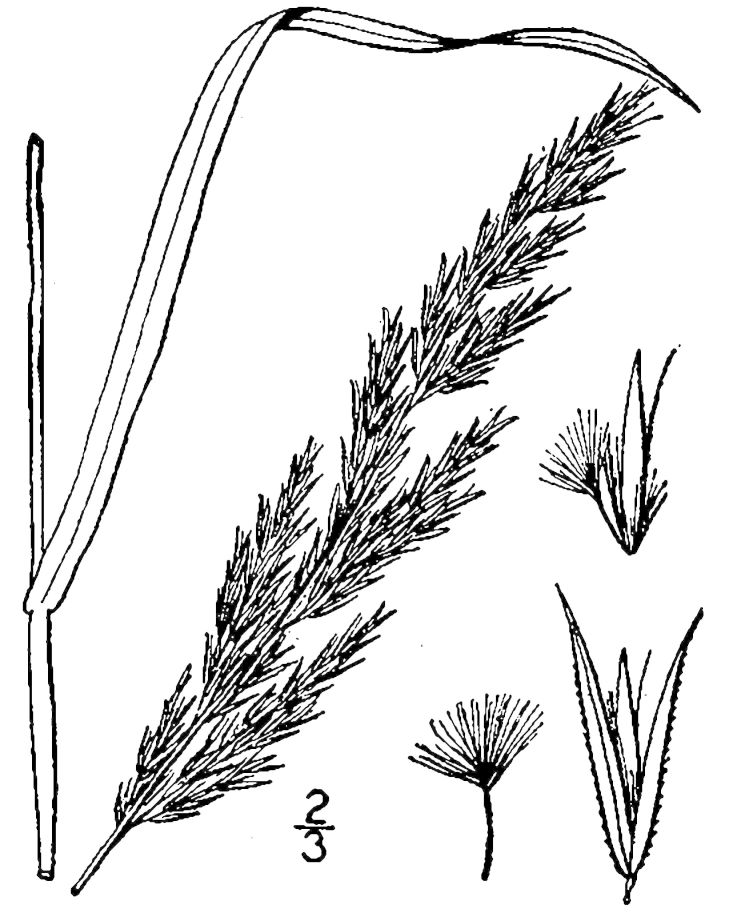
*Calamagrostis coarctata* (from Britton and Brown 1913).

**Figure 77.**
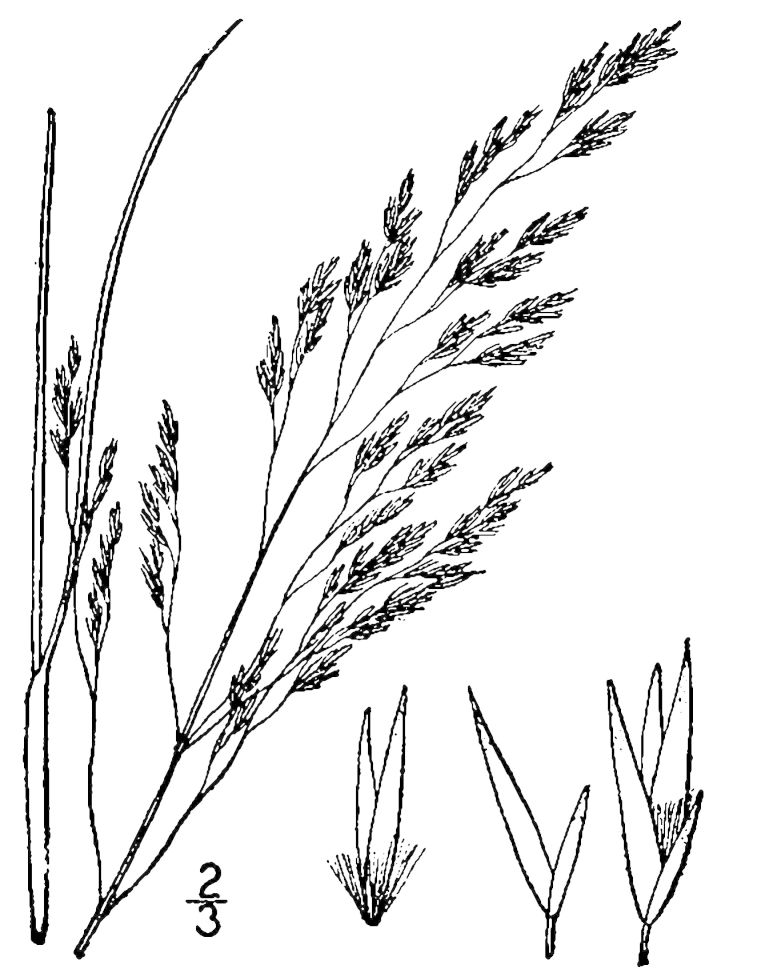
*Calamovilfa brevipilis* (from Britton and Brown 1913).

**Figure 78.**
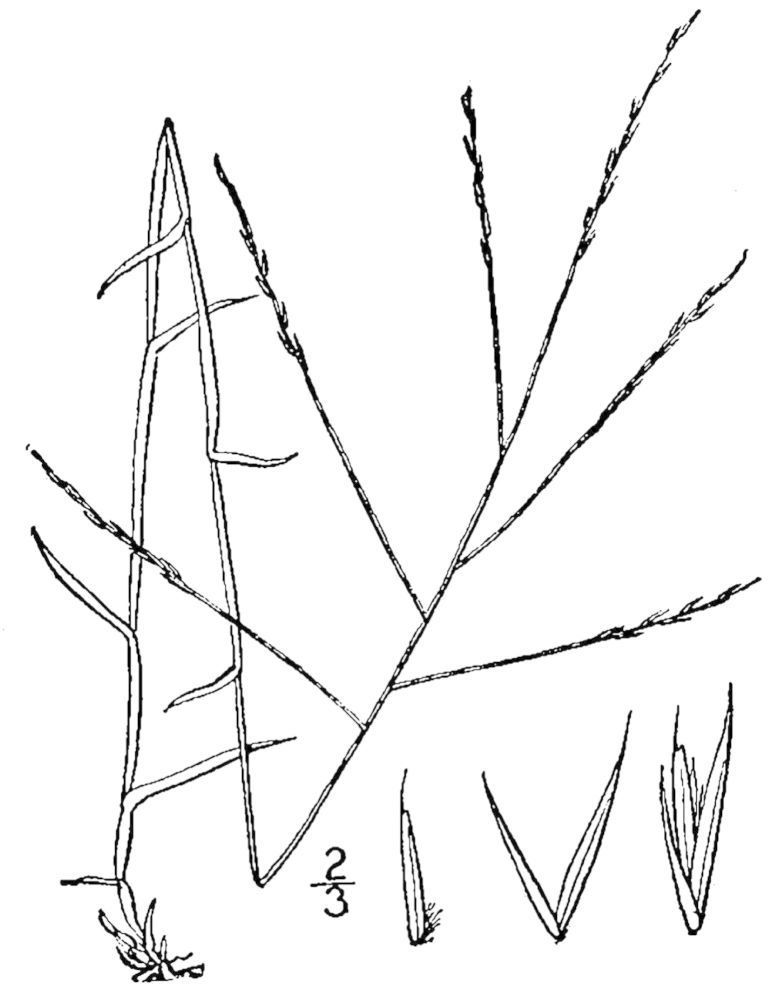
*Gymnopogon brevifolius* (from Britton and Brown 1913).

**Figure 79a.**
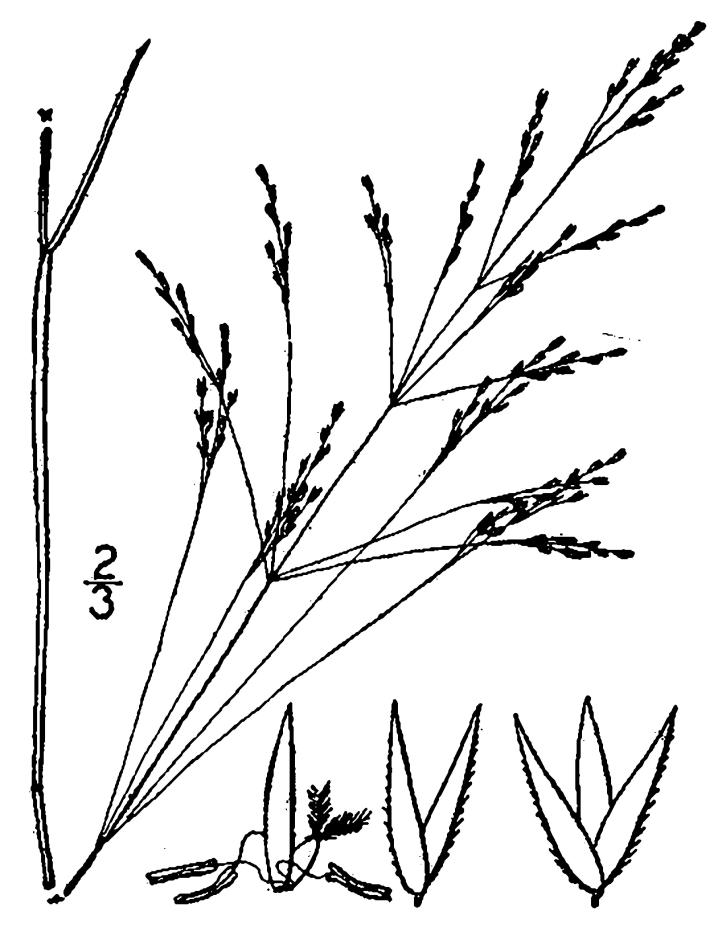
*Agrostis hyemalis* (from Britton and Brown 1913).

**Figure 79b.**
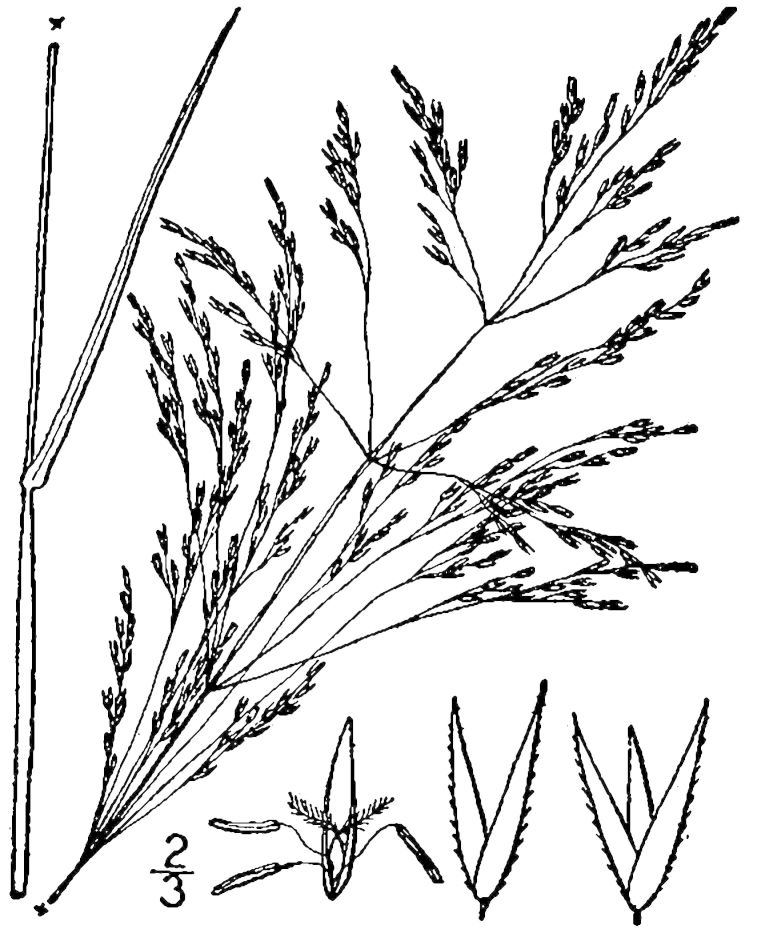
*Agrostis perennans* (from Britton and Brown 1913).

**Figure 80a.**
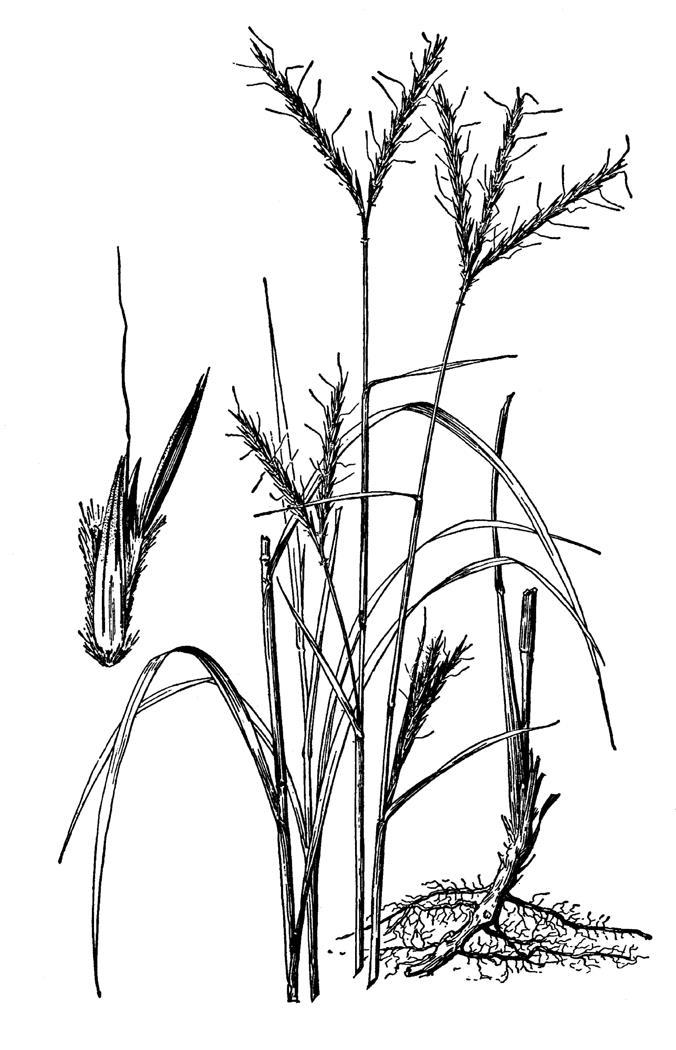
*Andropogon gerardii* (from Hitchcock 1950).

**Figure 80b.**
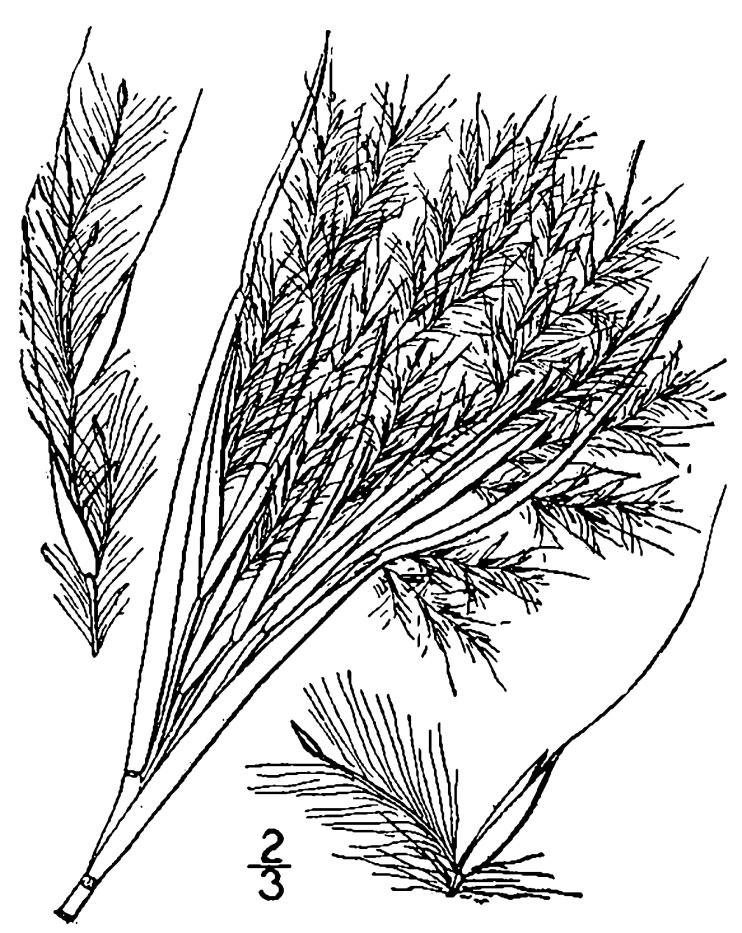
*Andropogon glomeratus* (from Britton and Brown 1913).

**Figure 80c.**
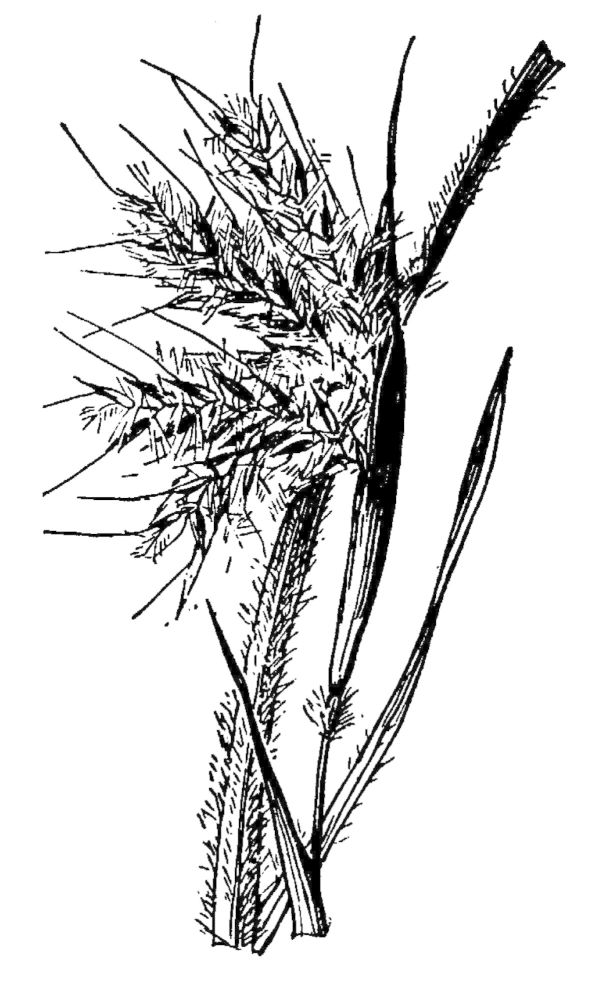
*Andropogon mohrii* (from Hitchcock 1950).

**Figure 80d.**
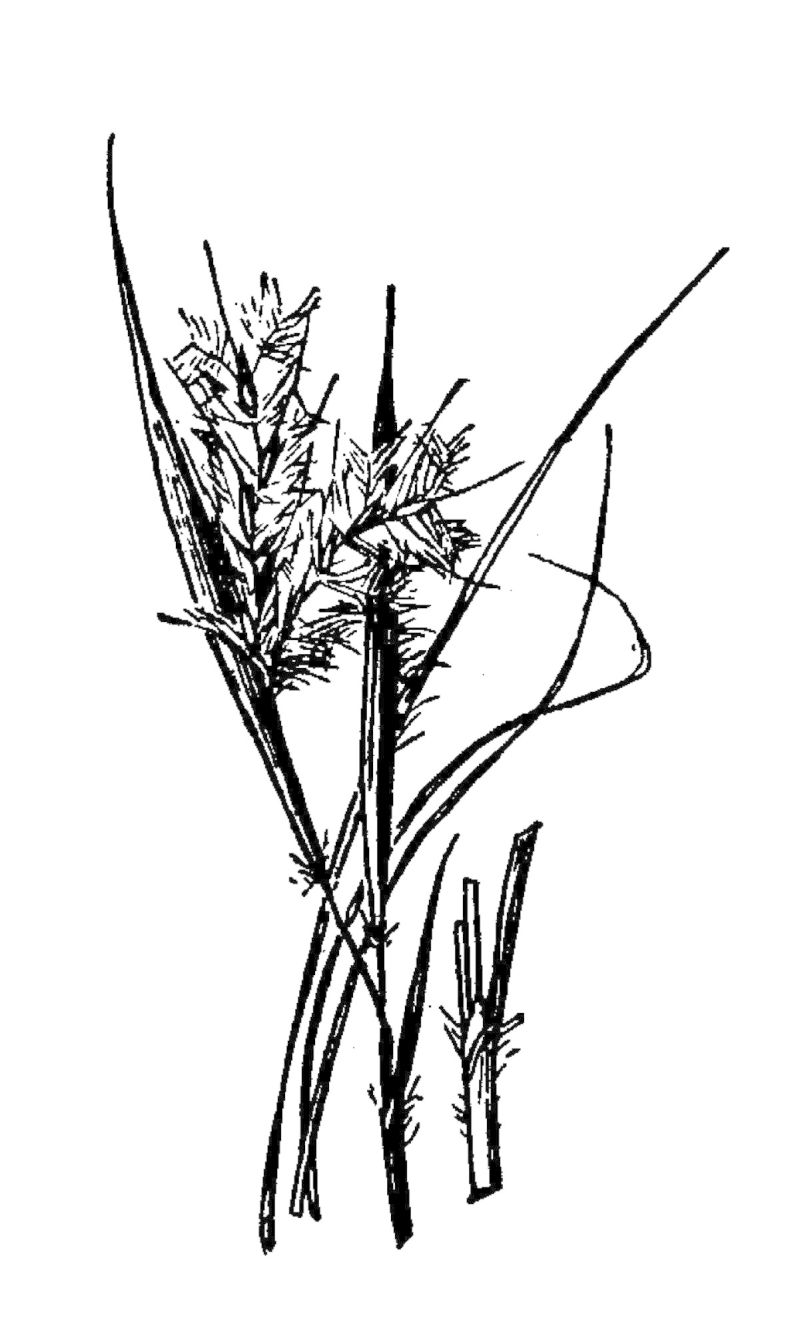
*Andropogon perangustatus* (from Hitchcock 1950).

**Figure 80e.**
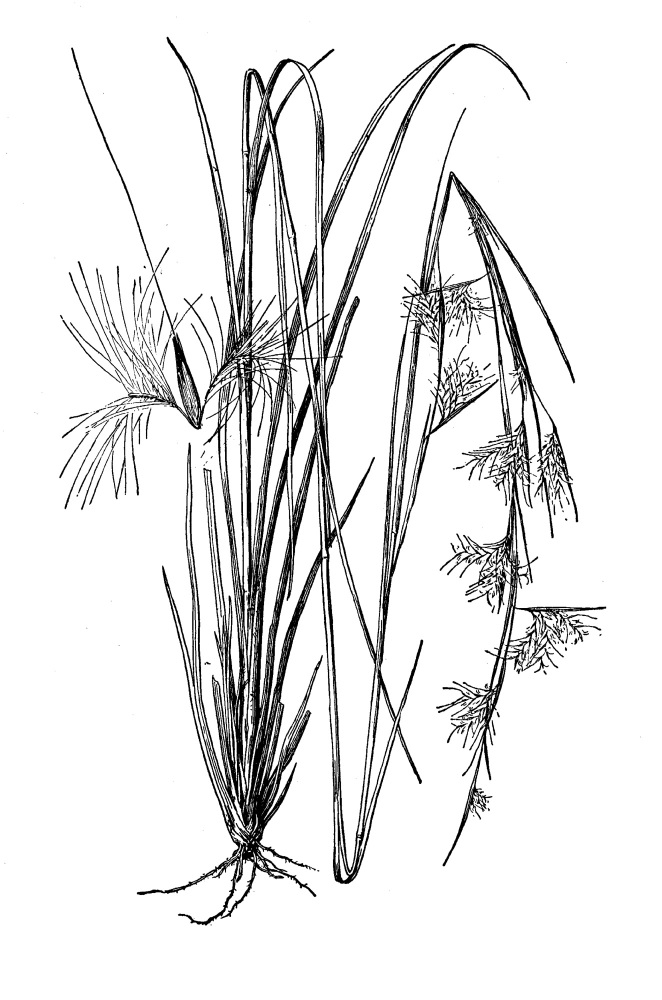
*Andropogon virginicus* (from Britton and Brown 1913).

**Figure 81a.**
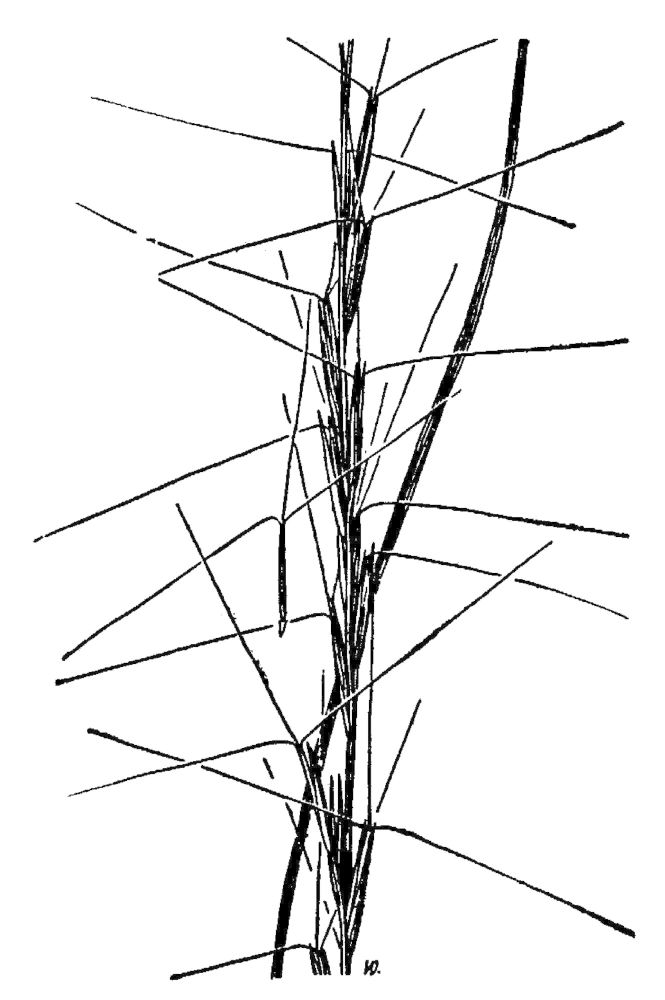
*Aristida palustris* (from Hitchcock 1950).

**Figure 81b.**
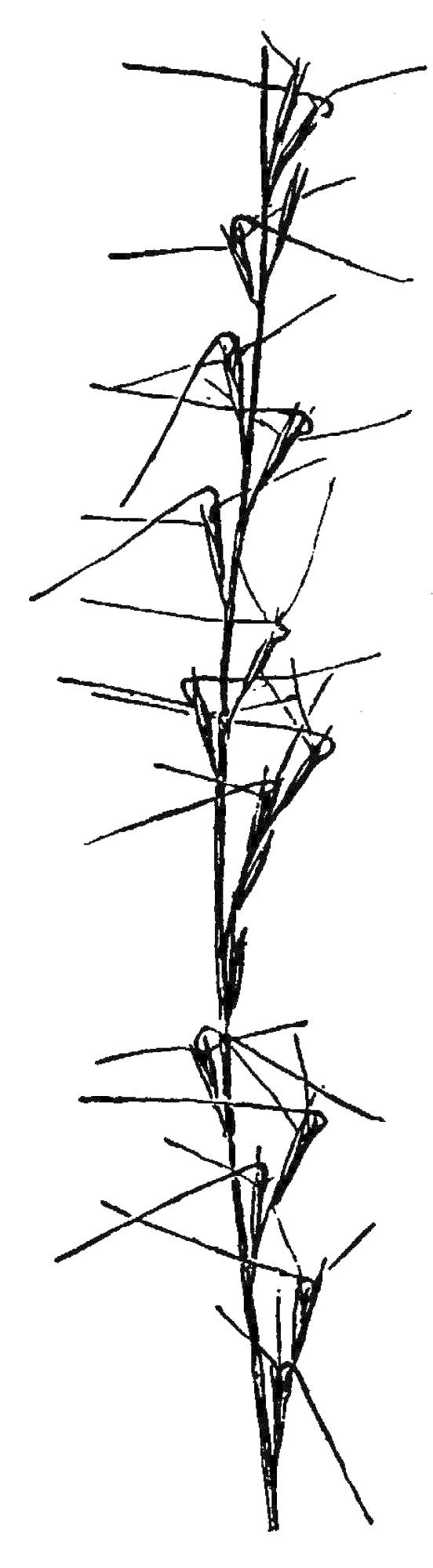
*Aristida simpliciflora* (from Hitchcock 1950).

**Figure 81c.**
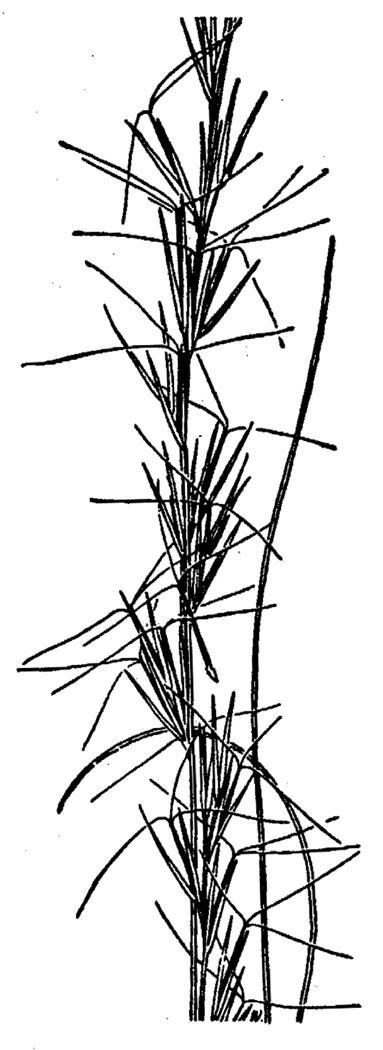
*Aristida stricta* (from Hitchcock 1950).

**Figure 81d.**
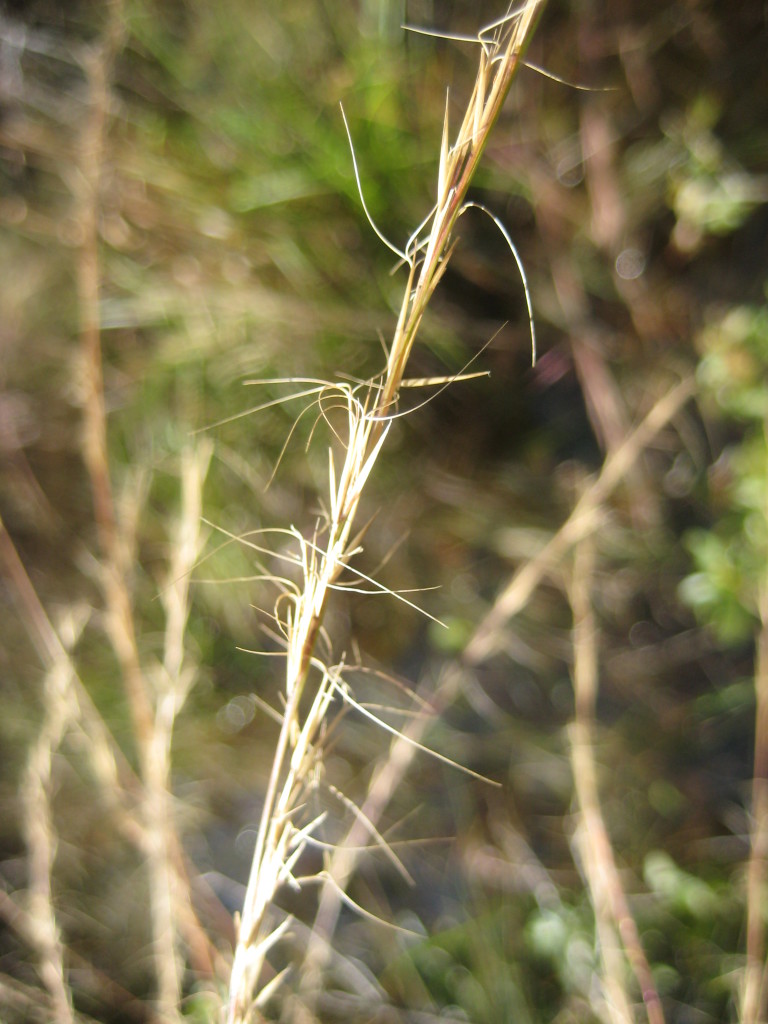
*Aristida stricta*: close-up of spikelets (Photo by R. Thornhill).

**Figure 81e.**
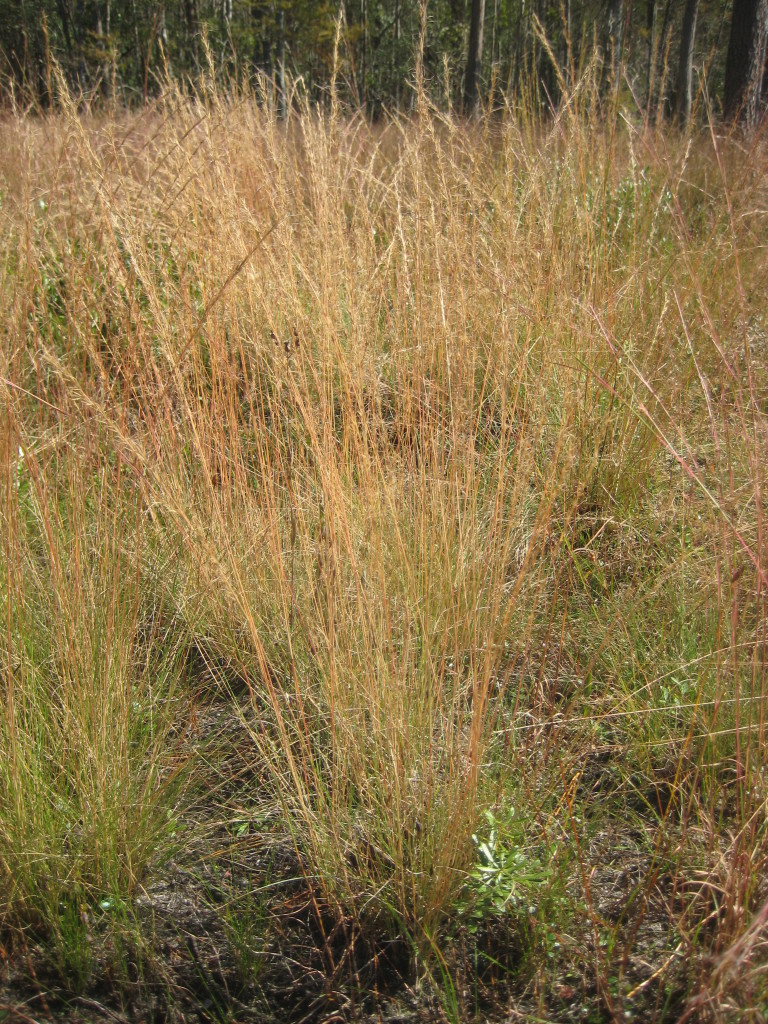
*Aristida stricta*: habit (photo by R. Thornhill).

**Figure 81f.**
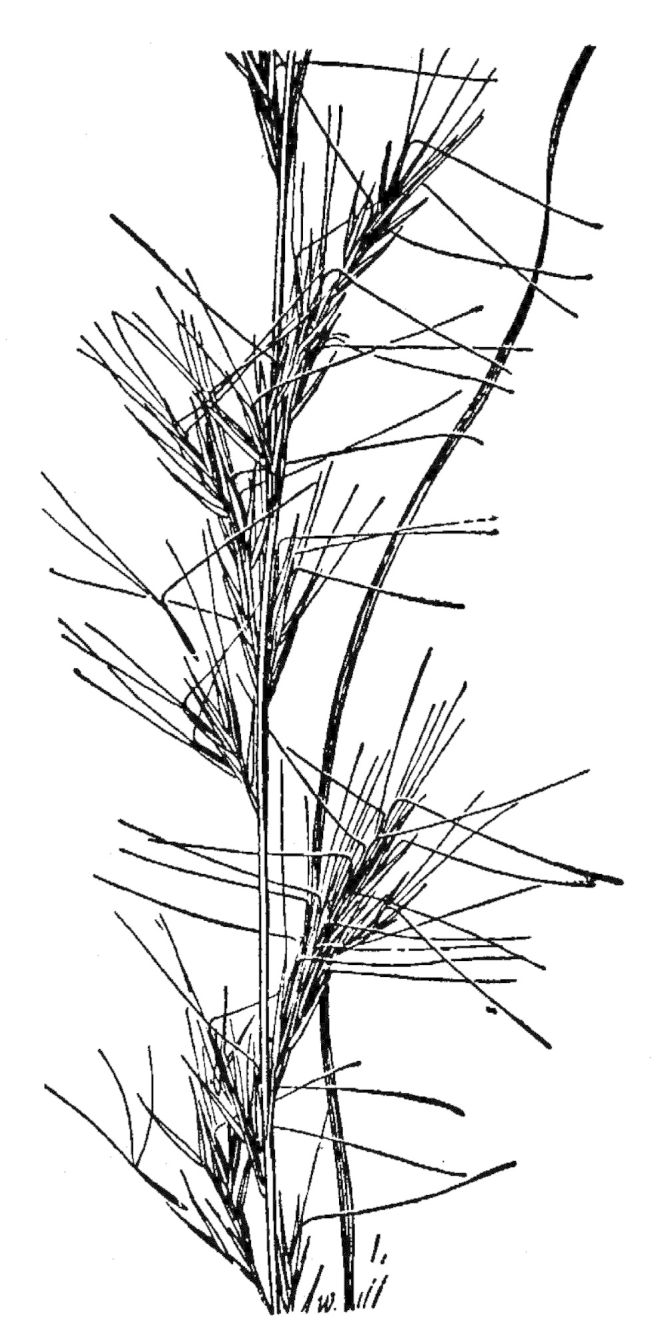
*Aristida virgata* (from Hitchcock 1950).

**Figure 82a.**
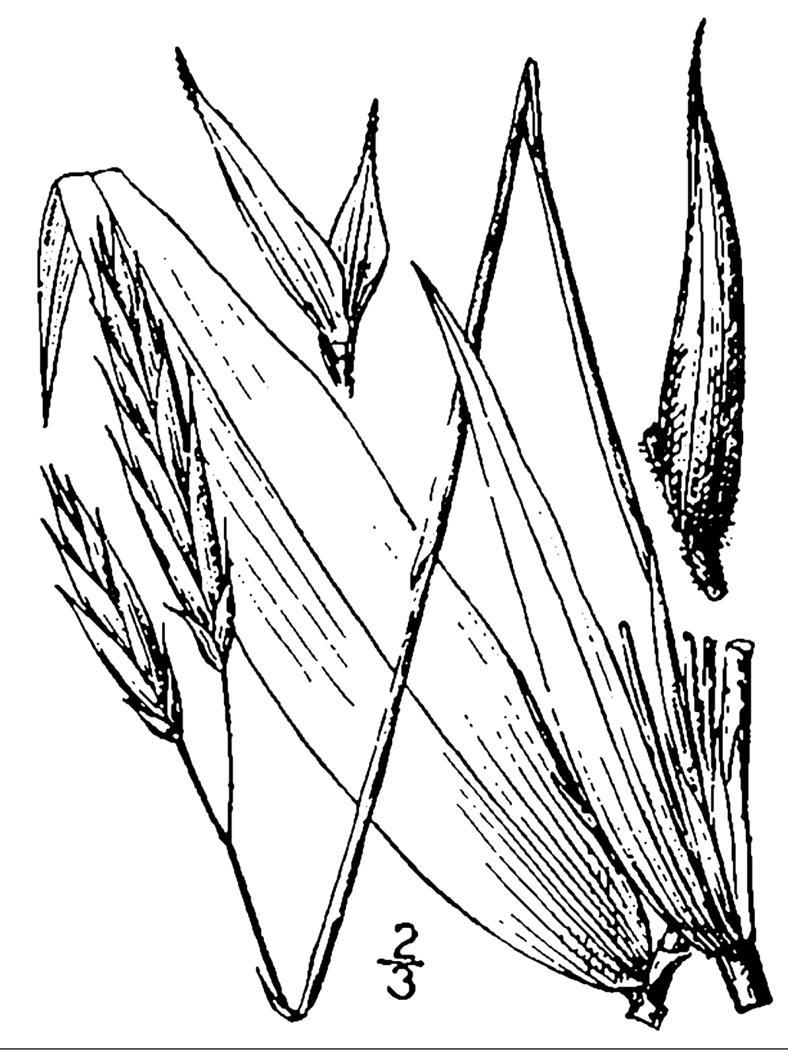
*Arundinaria gigantea* (from Britton and Brown 1913).

**Figure 82b.**
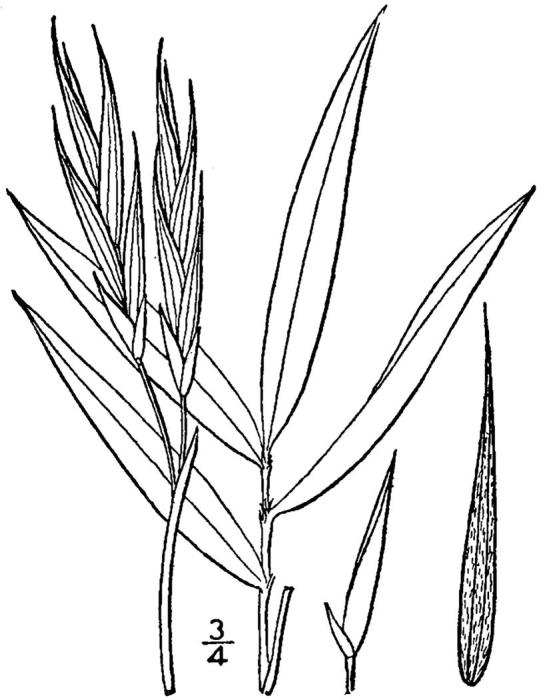
*Arundinaria tecta* (from Britton and Brown 1913).

**Figure 83a.**
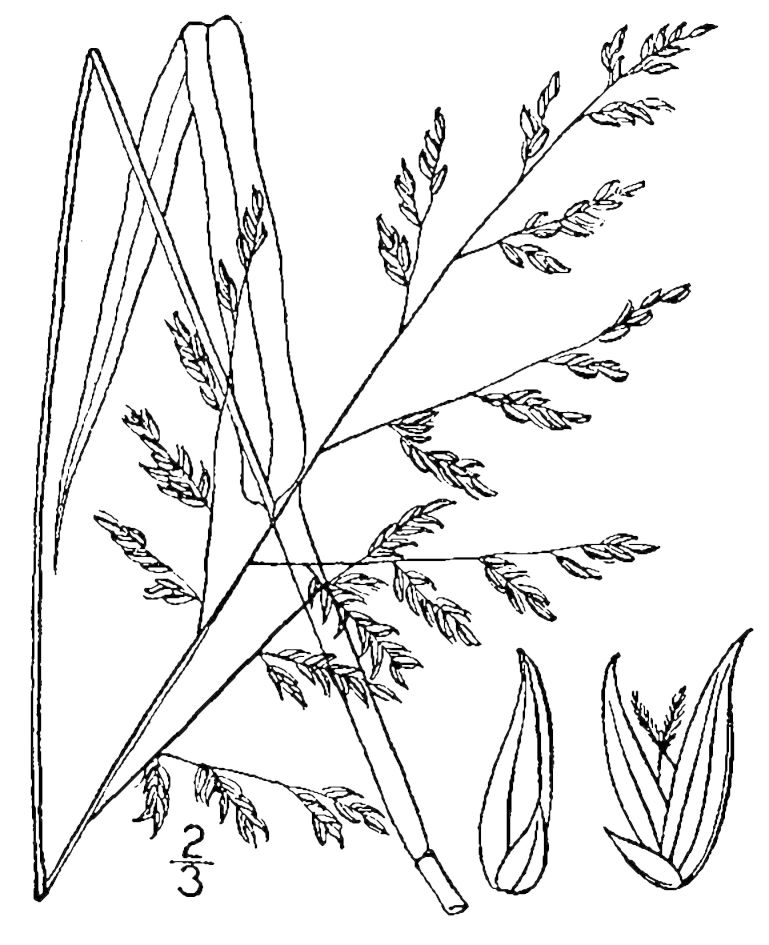
*Coleataenia anceps* (from Britton and Brown 1913).

**Figure 83b.**
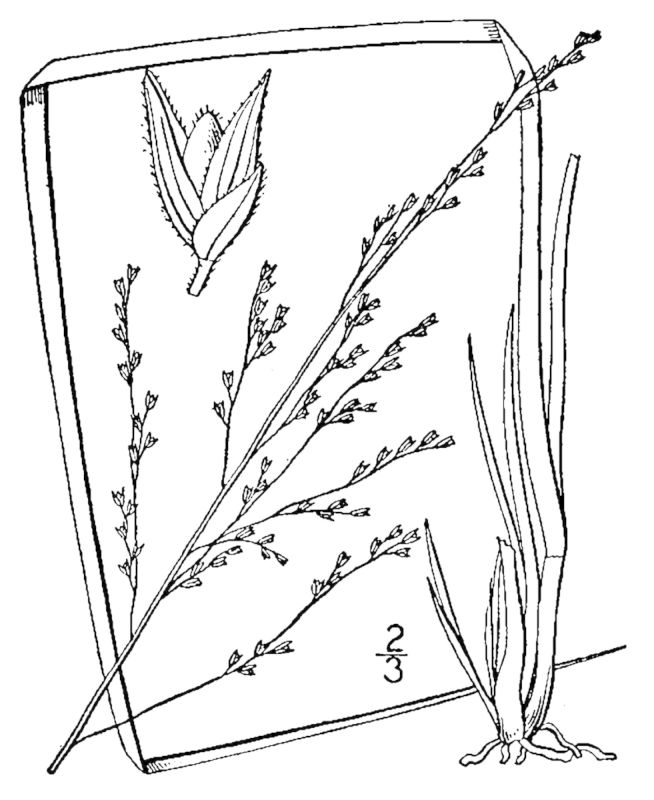
Coleataenia longifolia ssp. longifolia (from Britton and Brown 1913).

**Figure 84a.**
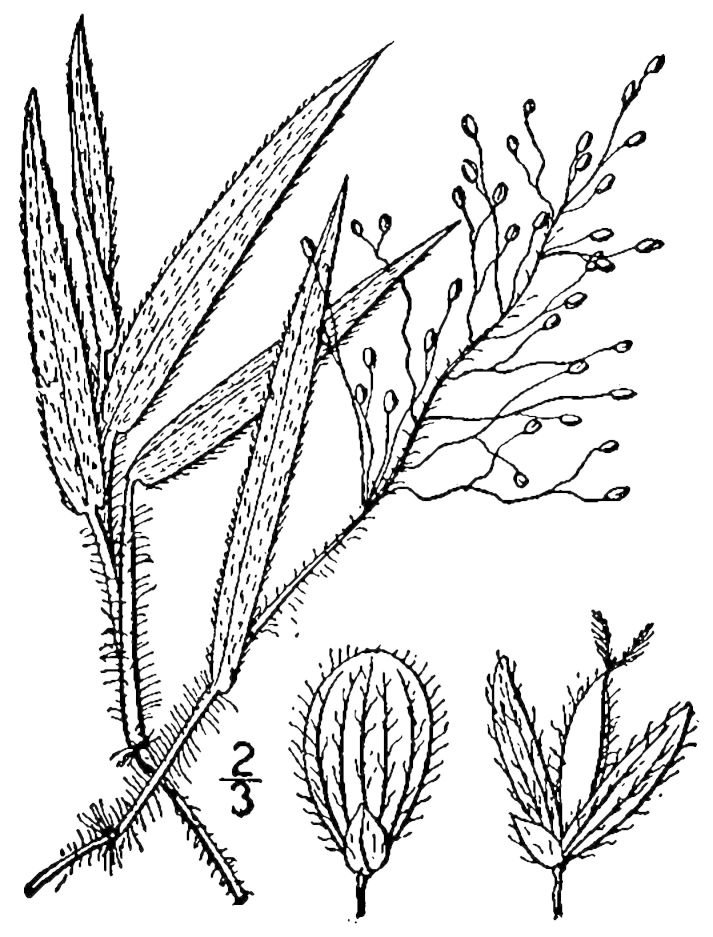
Dichanthelium acuminatum var. fasciculatum (from Britton and Brown 1913).

**Figure 84b.**
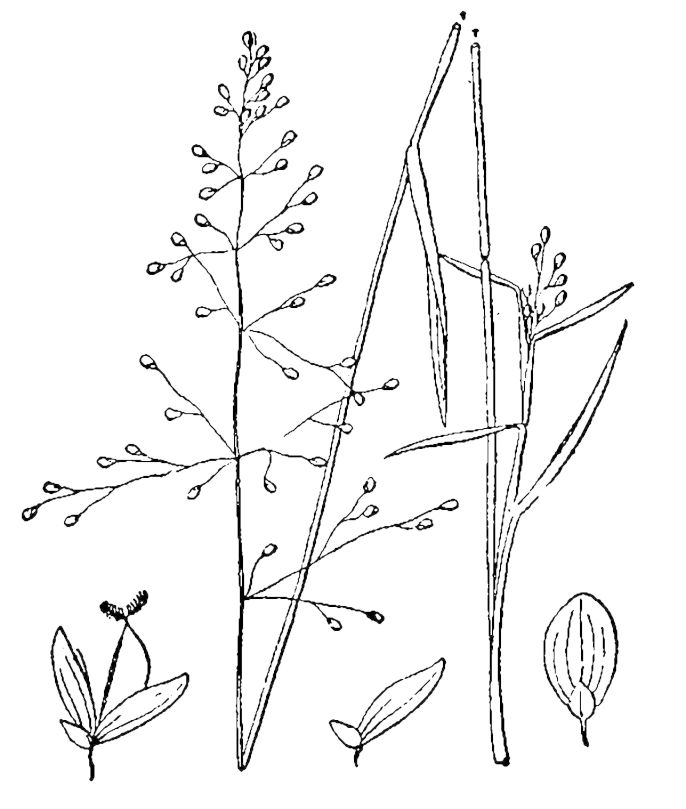
Dichanthelium acuminatum var. lindheimeri (from Britton and Brown 1913).

**Figure 84c.**
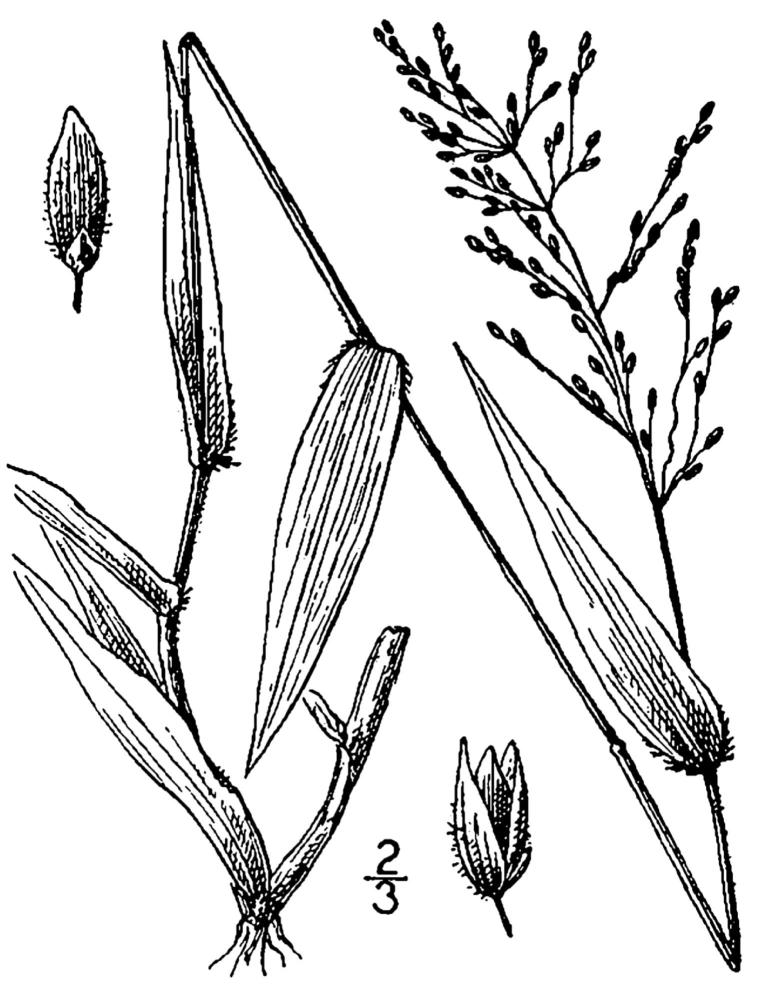
*Dichanthelium commutatum* (from Britton and Brown 1913).

**Figure 84d.**
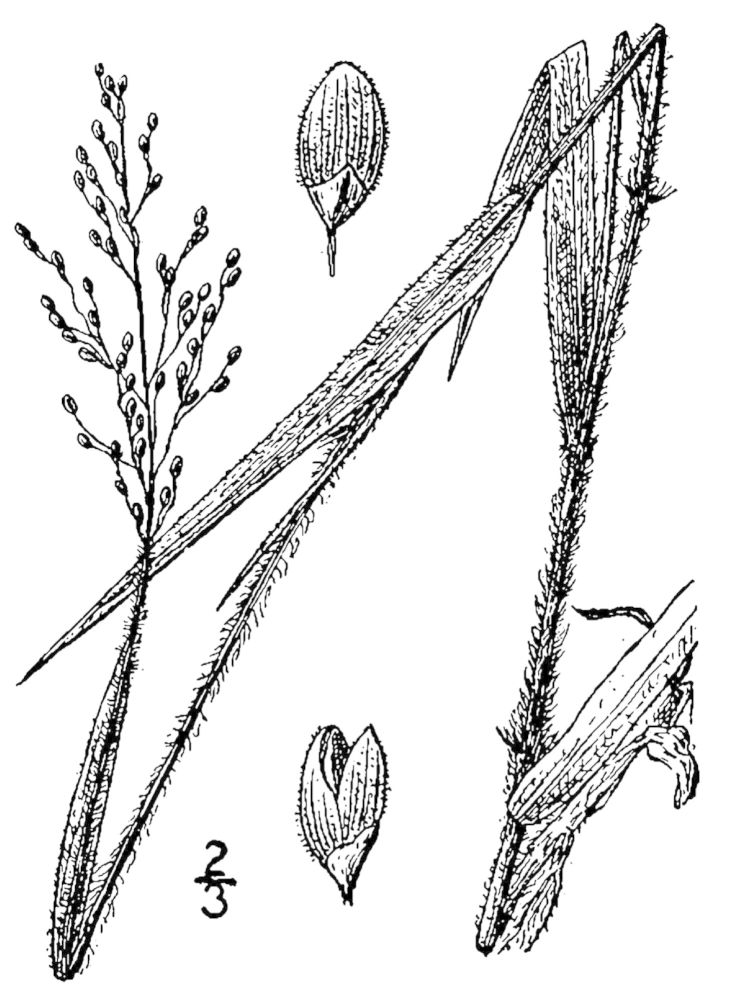
*Dichanthelium consanguineum* (from Britton and Brown 1913).

**Figure 84e.**
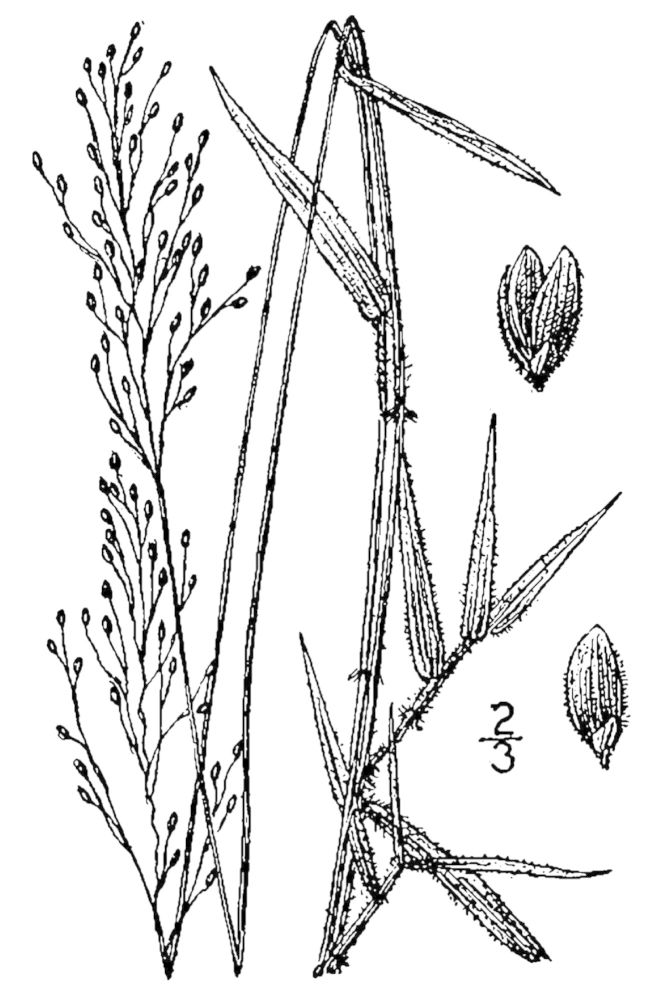
*Dichanthelium dichotomum* (from Britton and Brown 1913).

**Figure 84f.**
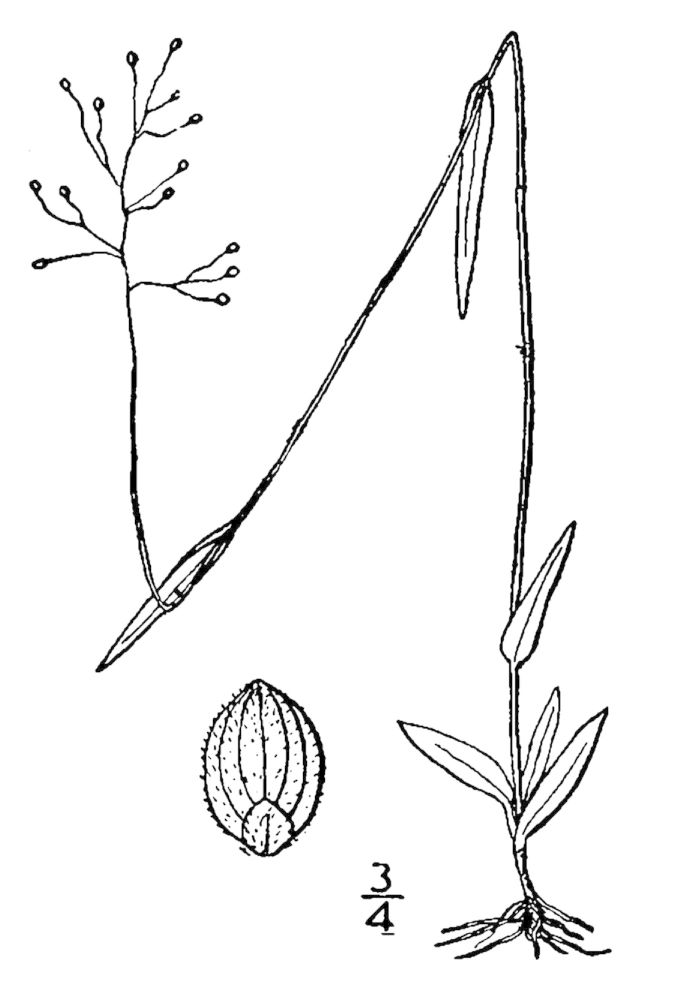
*Dichanthelium ensifolium* (from Britton and Brown 1913).

**Figure 85a.**
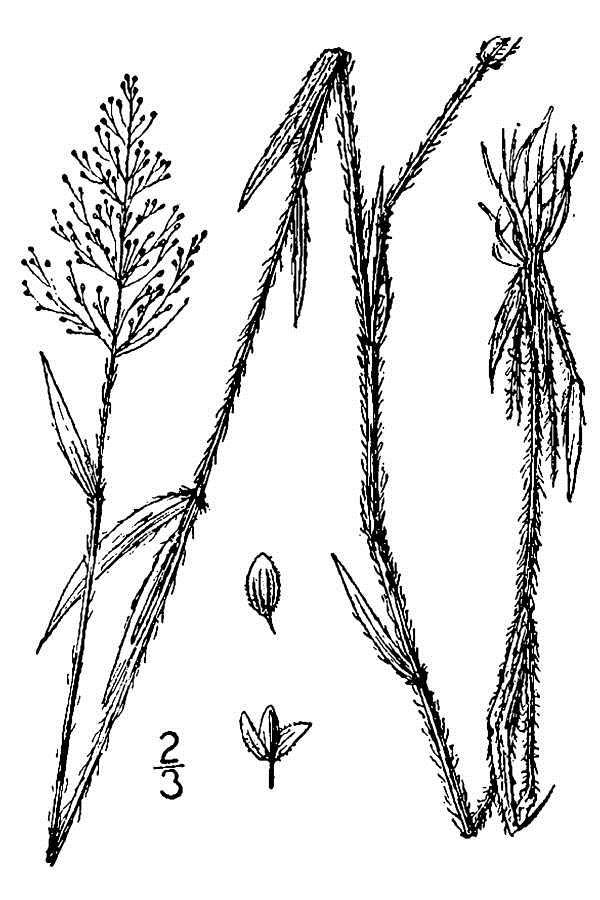
*Dichanthelium leucothrix* (from Britton and Brown 1913).

**Figure 85b.**
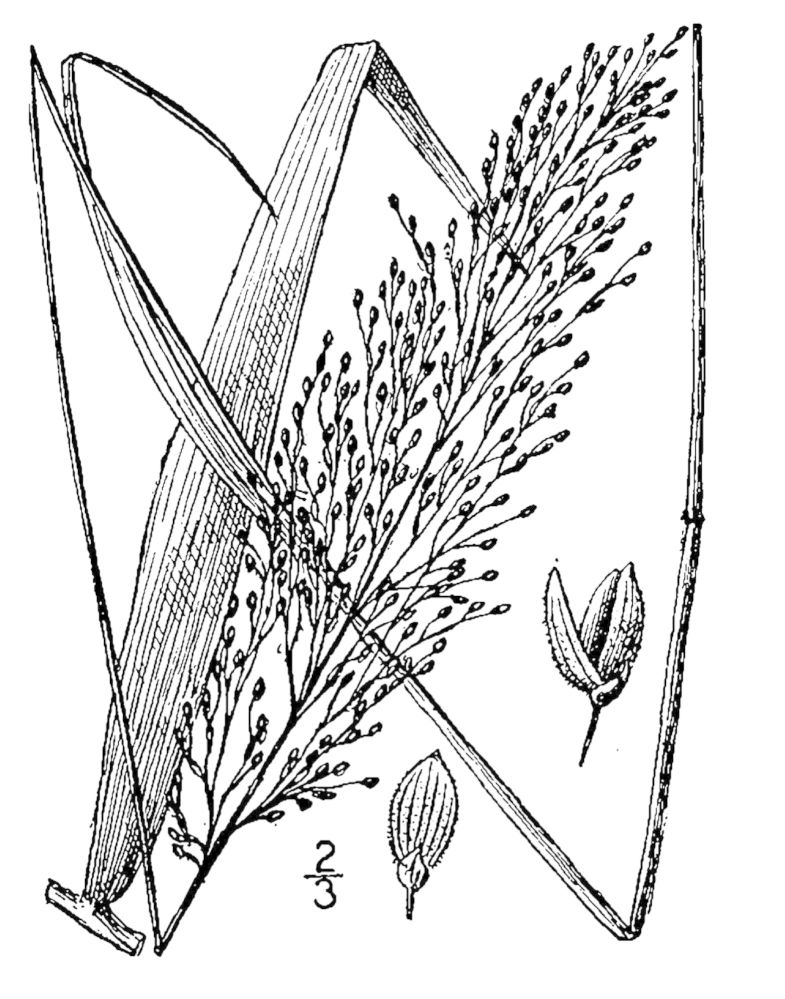
*Dichanthelium mattamuskeetense* (from Britton and Brown 1913).

**Figure 85c.**
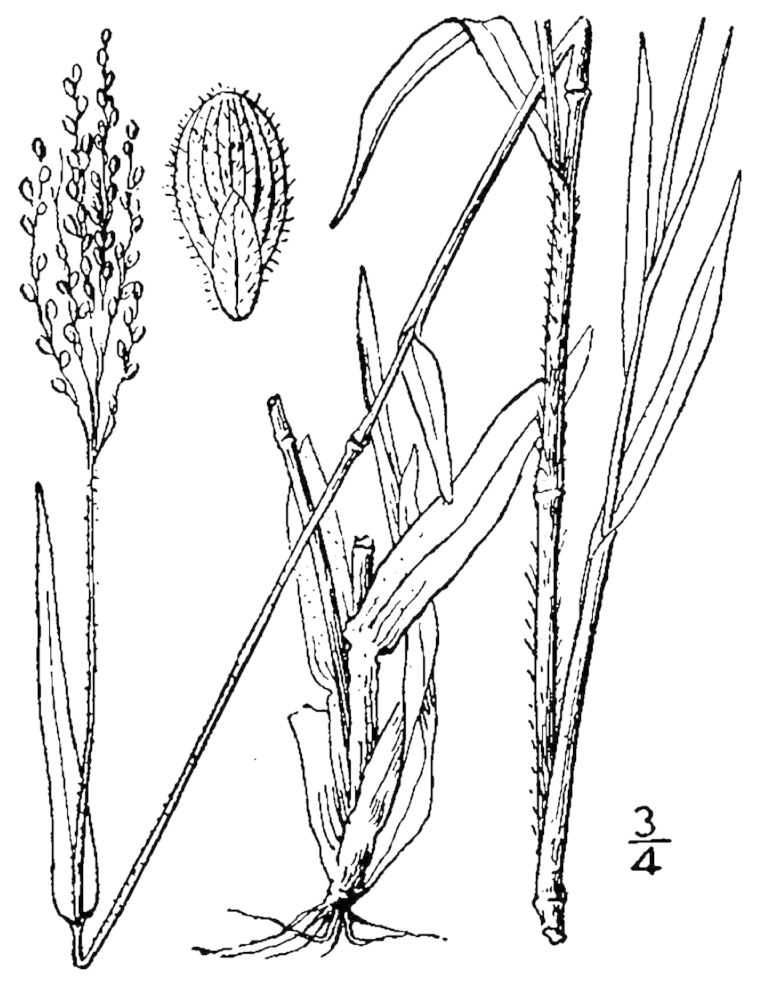
Dichanthelium ovale var. addisonii (from Britton and Brown 1913).

**Figure 85d.**
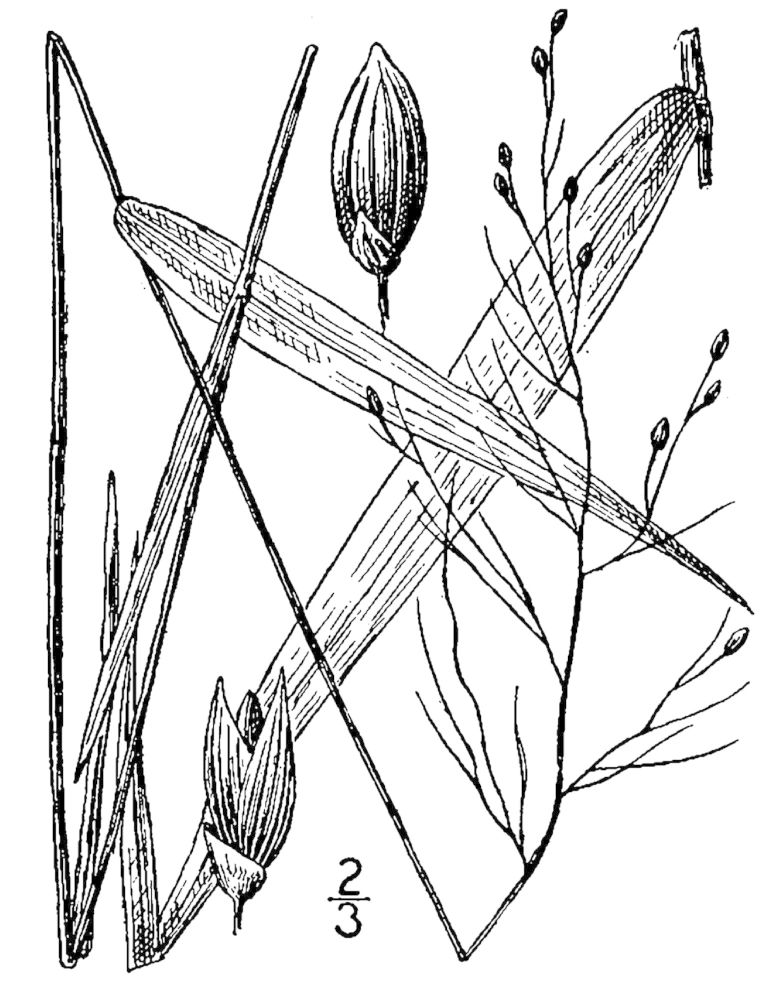
*Dichanthelium scabriusculum* (from Britton and Brown 1913).

**Figure 85e.**
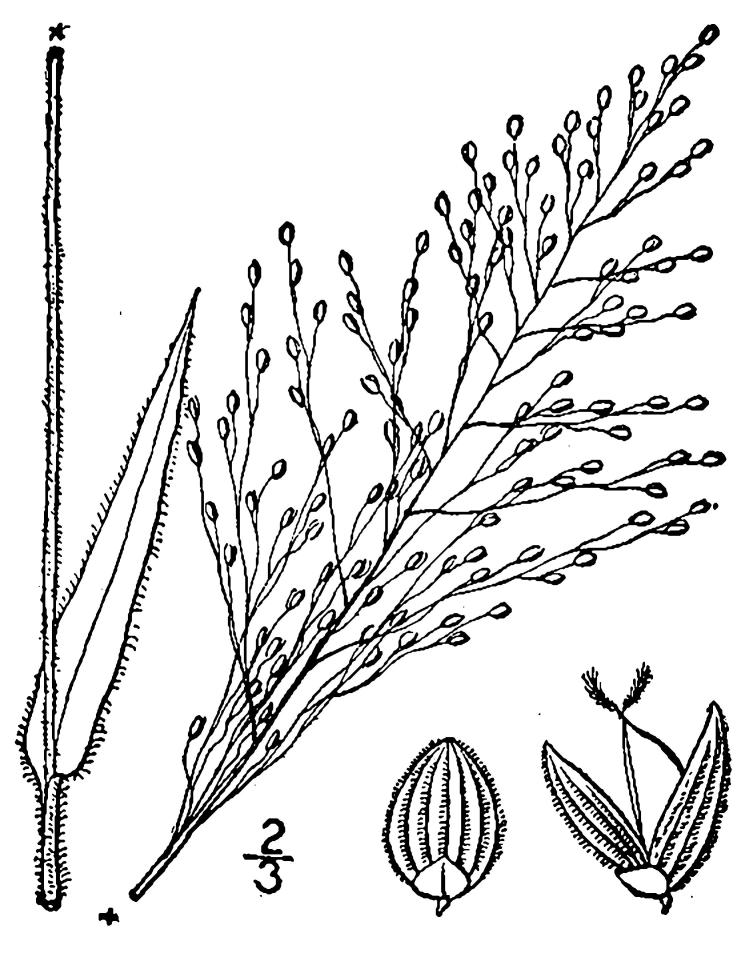
*Dichanthelium scoparium* (from Britton and Brown 1913).

**Figure 85f.**
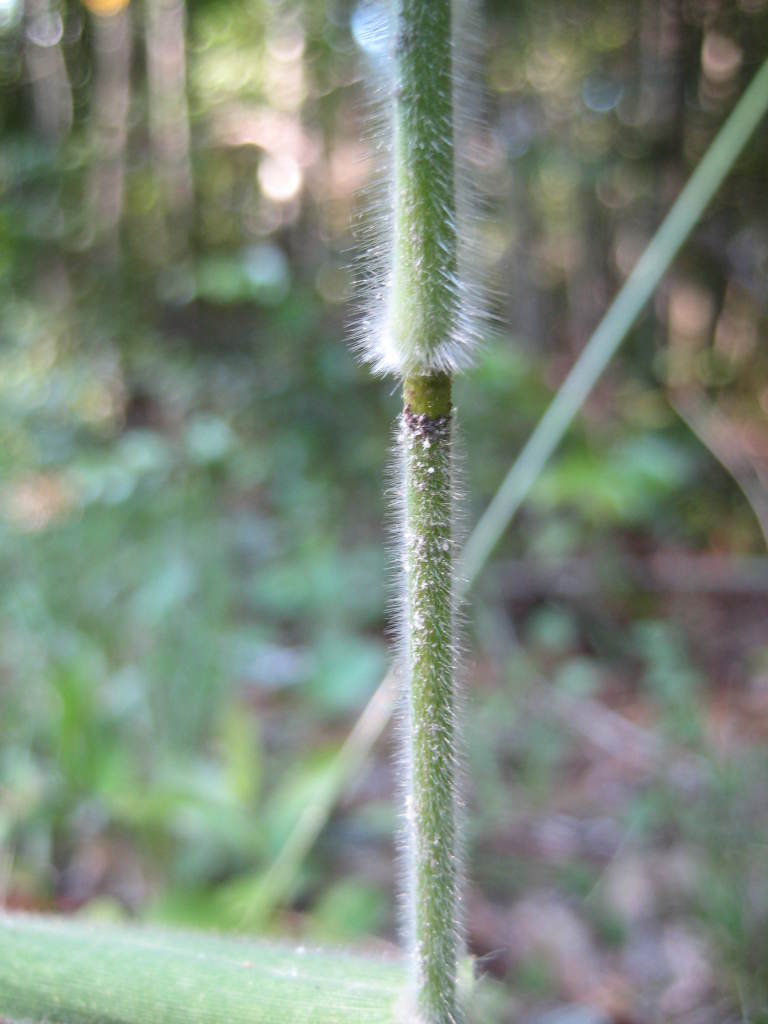
*Dichanthelium scoparium*: note the bearded node subtended by a glabrous band (photo by R. Thornhill).

**Figure 86a.**
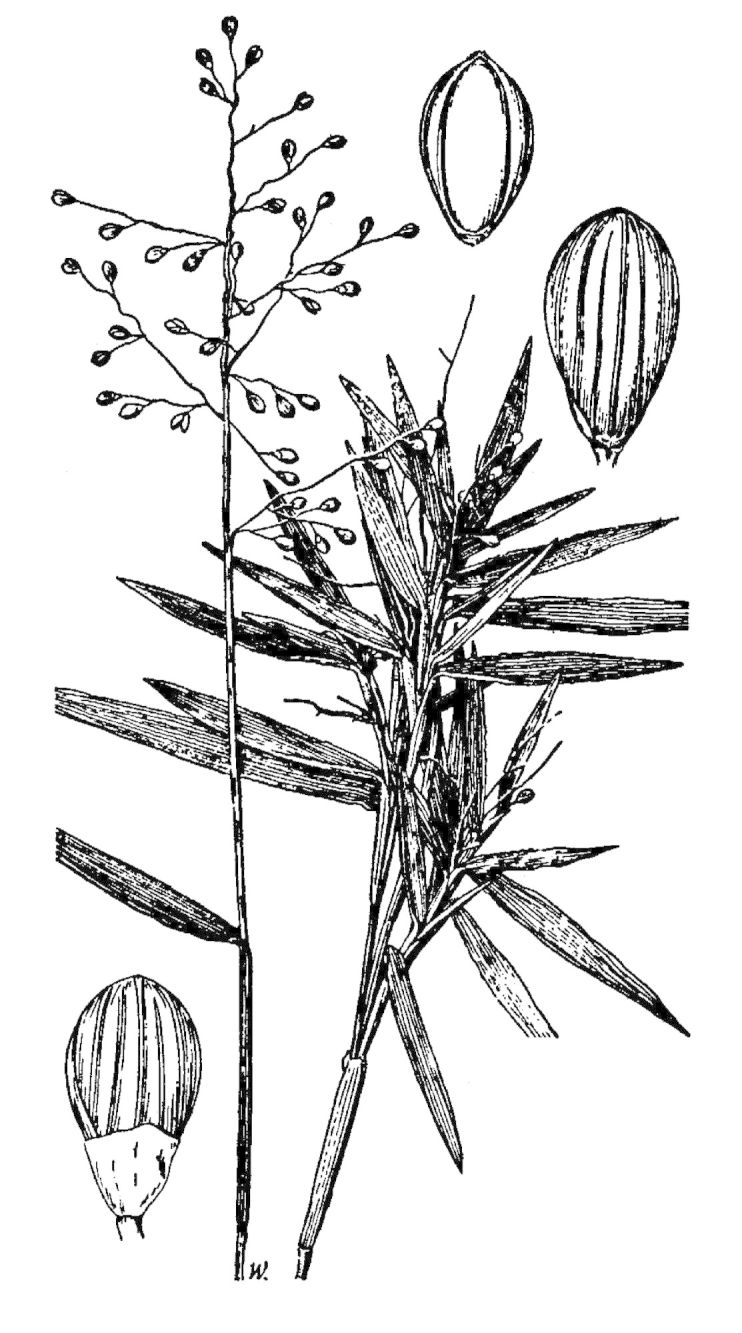
*Dichanthelium* species 3 (=*Dichantheliumlancearium*) (from Britton and Brown 1913).

**Figure 86b.**
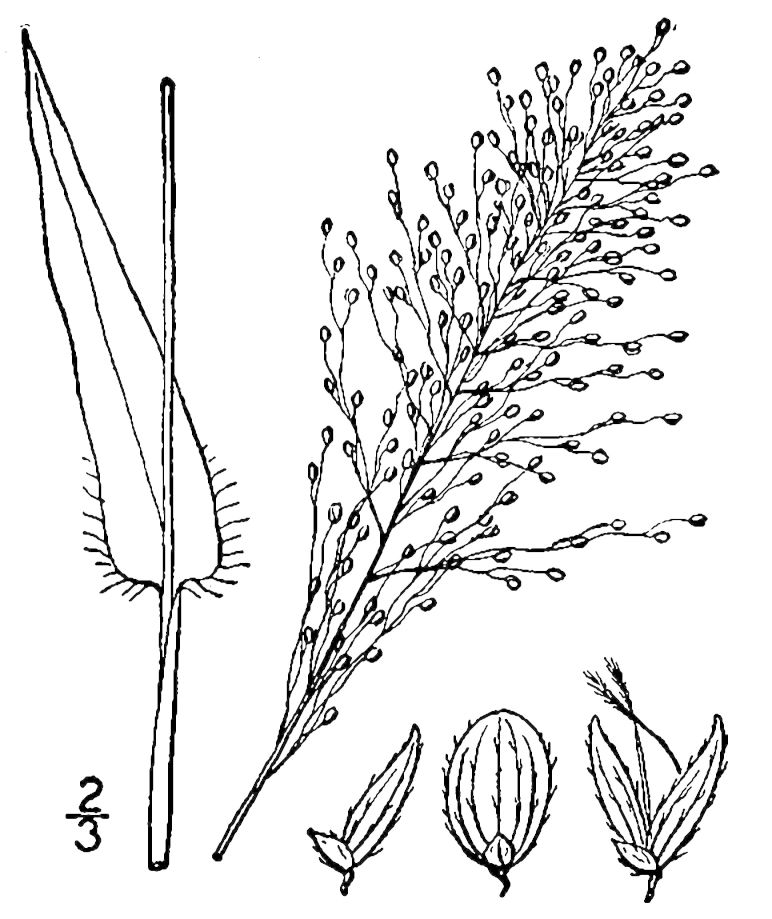
*Dichanthelium sphaerocarpon* (from Hitchcock 1950).

**Figure 86c.**
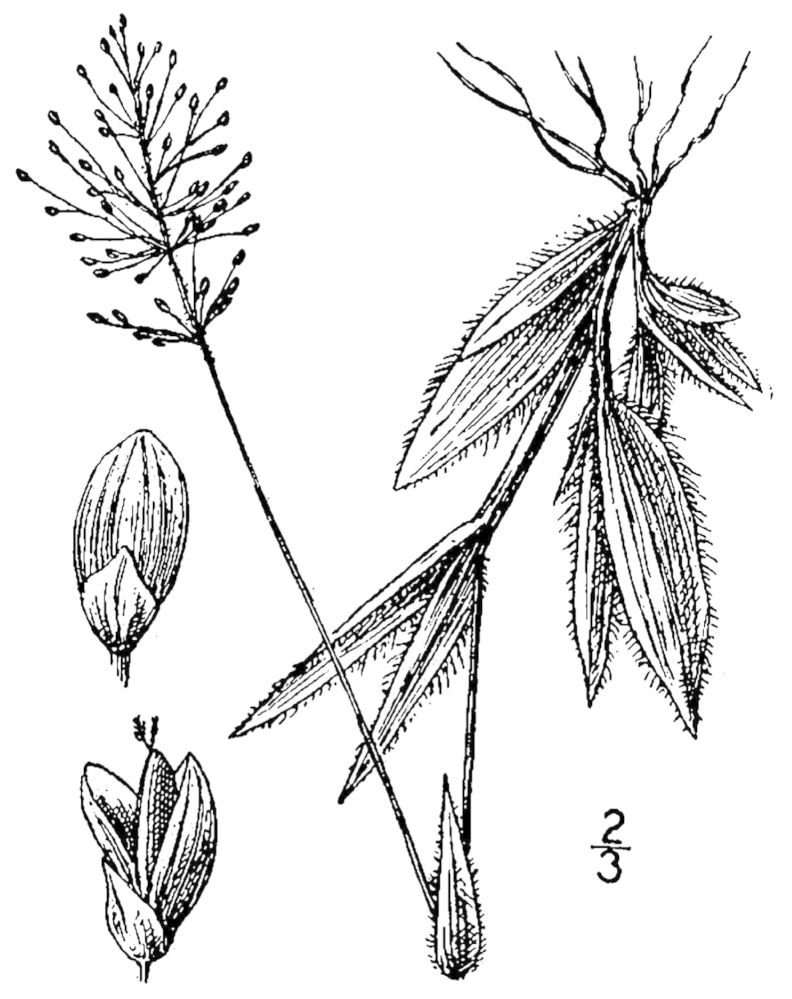
Dichanthelium strigosum var. leucoblepharis (from Britton and Brown 1913).

**Figure 86d.**
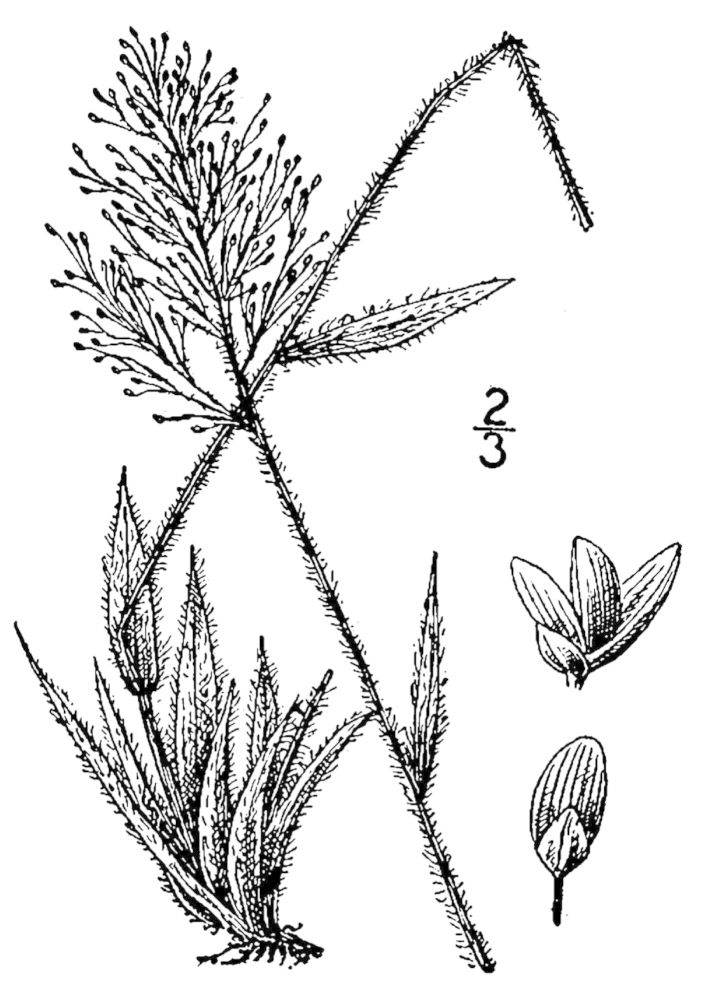
Dichanthelium strigosum var. strigosum (from Britton and Brown 1913).

**Figure 86e.**
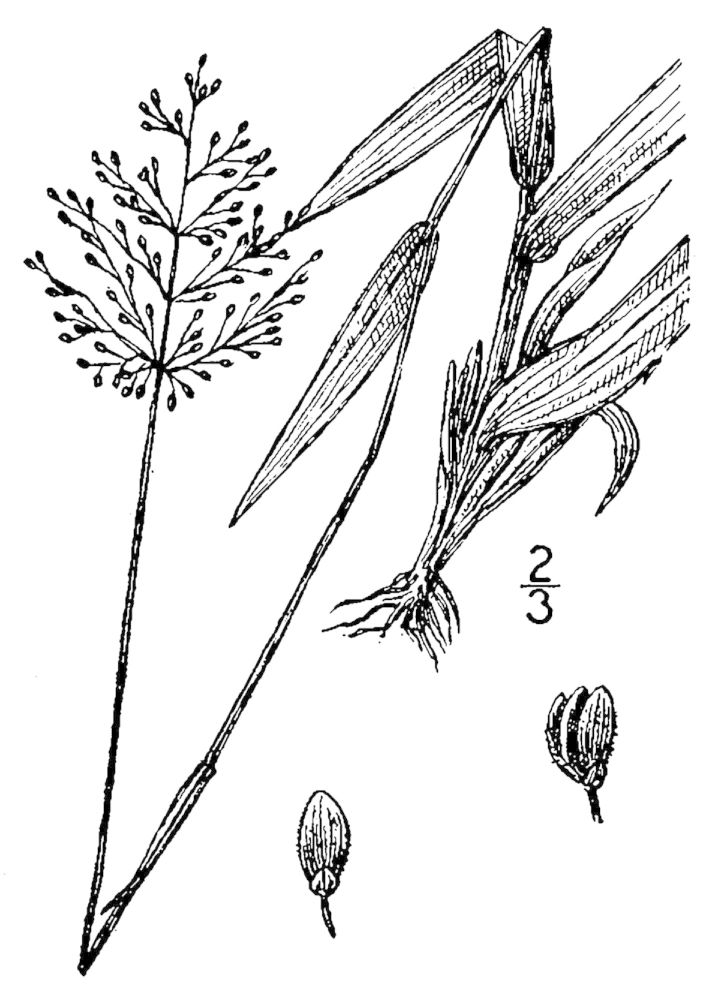
*Dichanthelium tenue* (from Britton and Brown 1913).

**Figure 86f.**
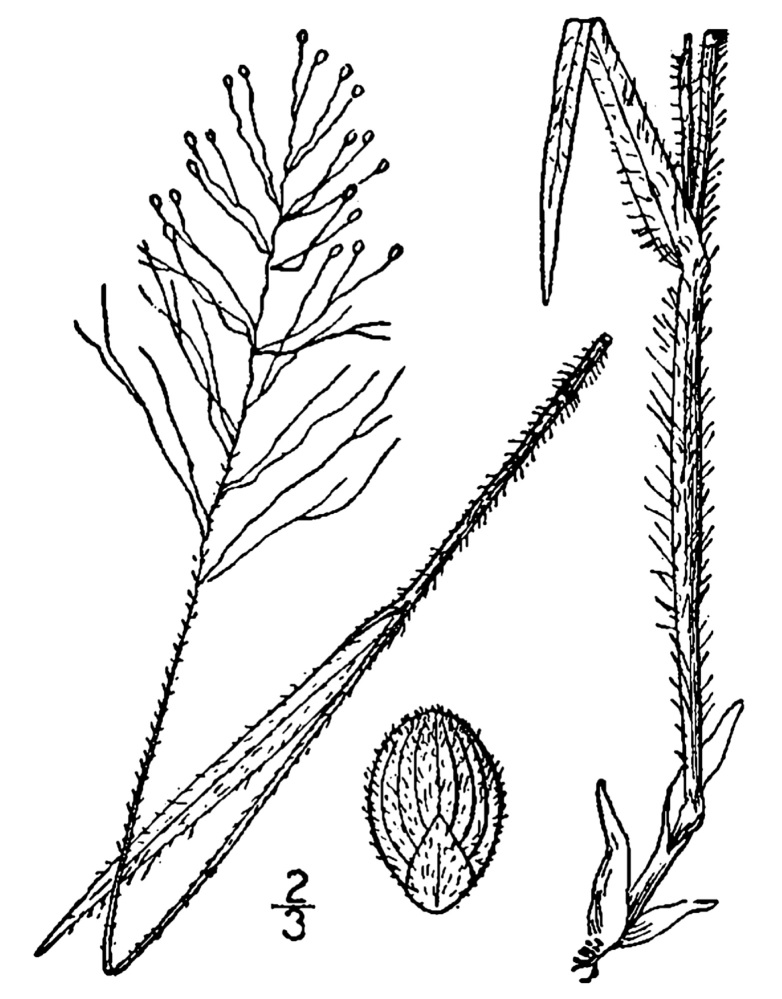
Dichanthelium villosissimum var. villosissimum (from Britton and Brown 1913).

**Figure 87a.**
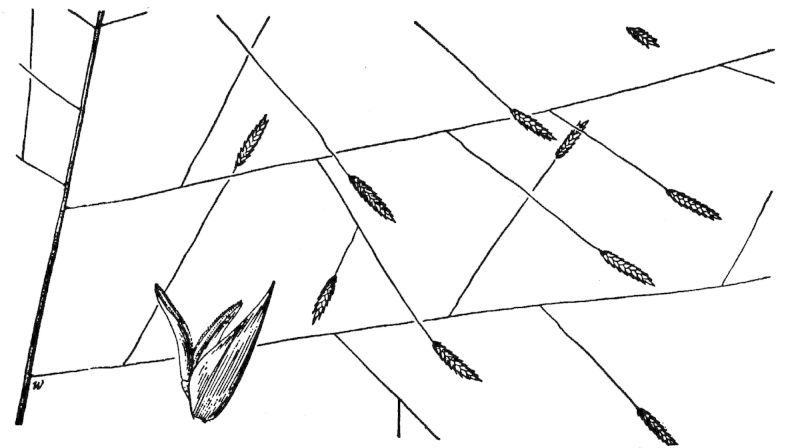
*Eragrostis elliottii* (from Hitchcock 1950).

**Figure 87b.**
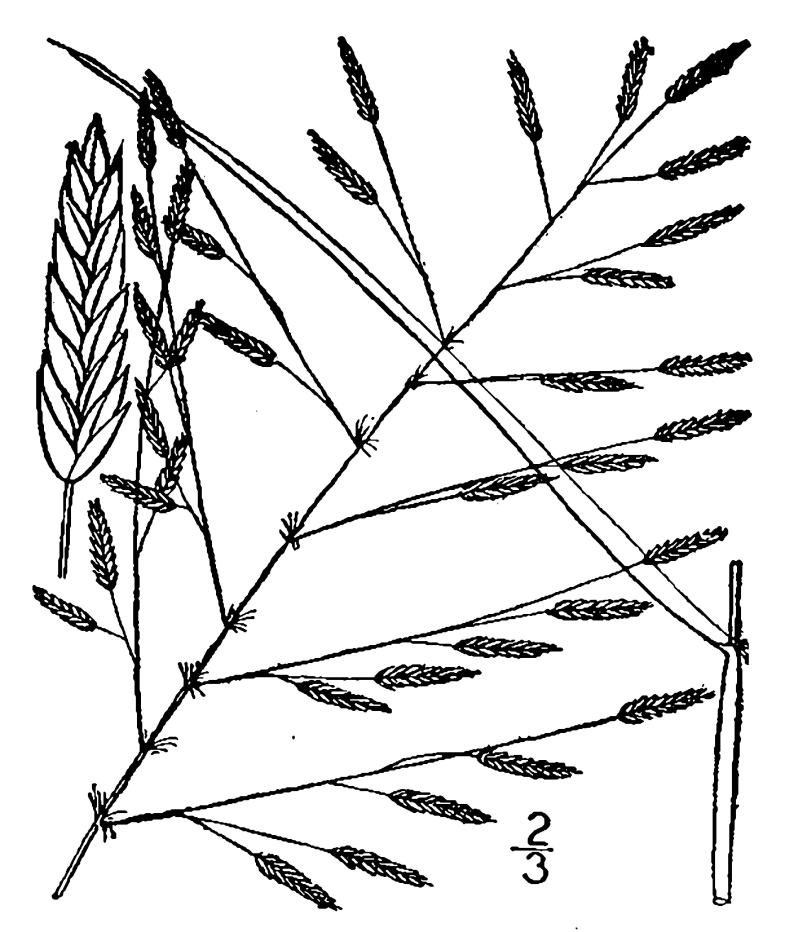
*Eragrostis refracta* (from Britton and Brown 1913).

**Figure 88a.**
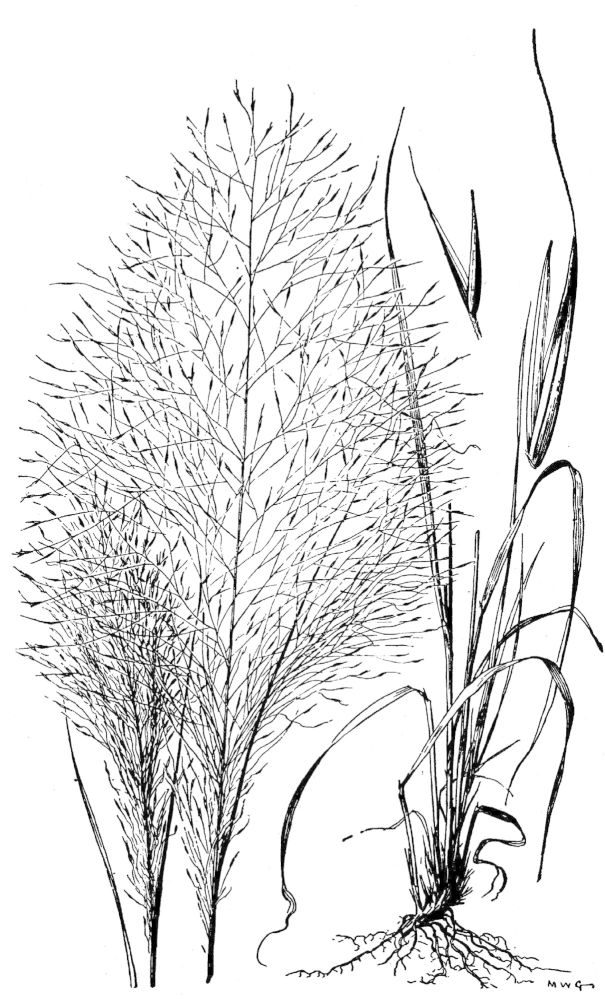
*Muhlenbergia capillaris* (from Hitchcock 1950).

**Figure 88b.**
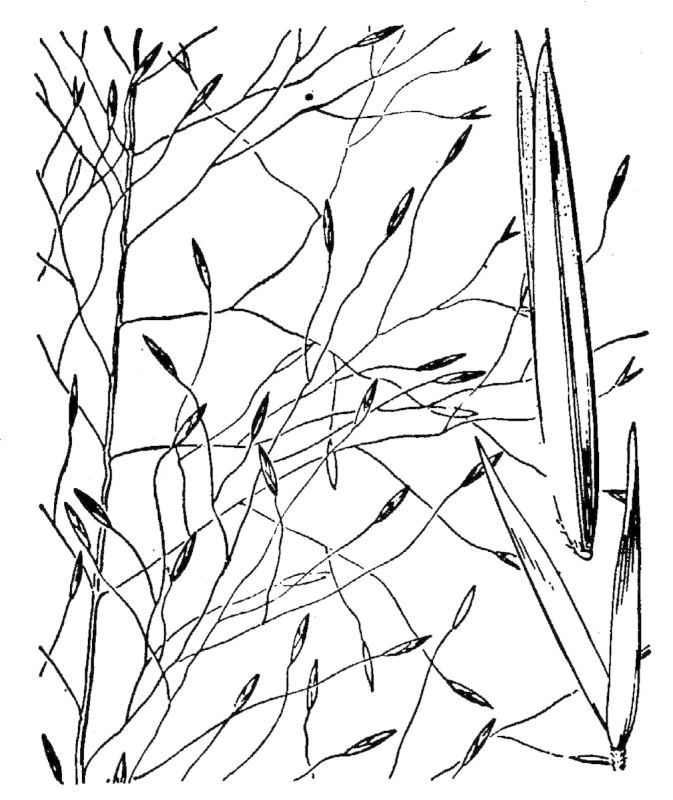
*Muhlenbergia expansa* (from Hitchcock 1950).

**Figure 88c.**
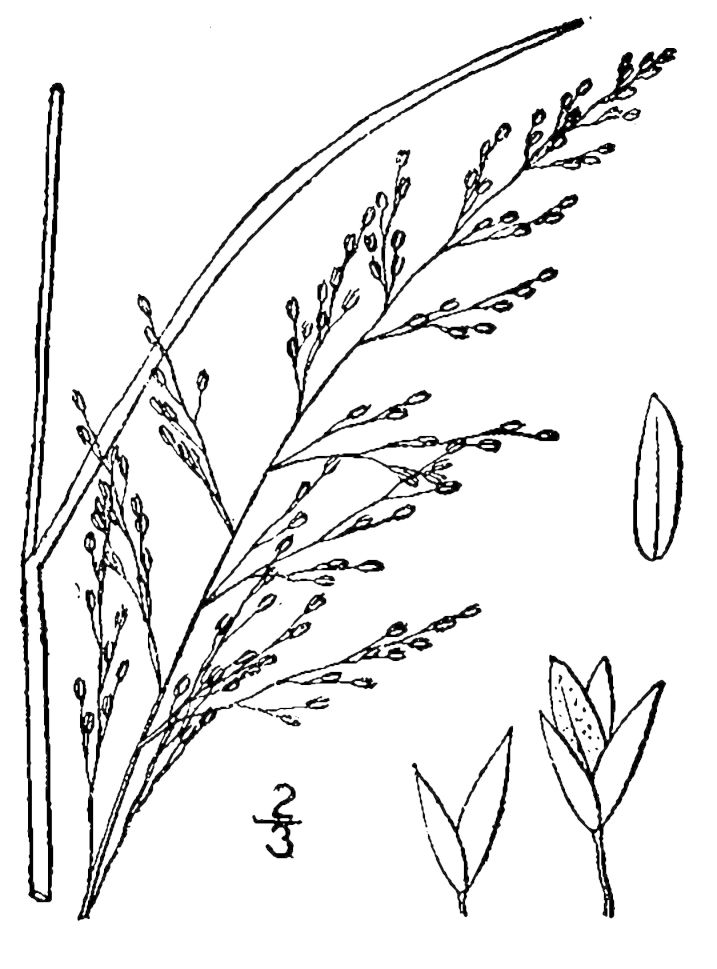
*Muhlenbergia torreyana* (from Britton and Brown 1913).

**Figure 89a.**
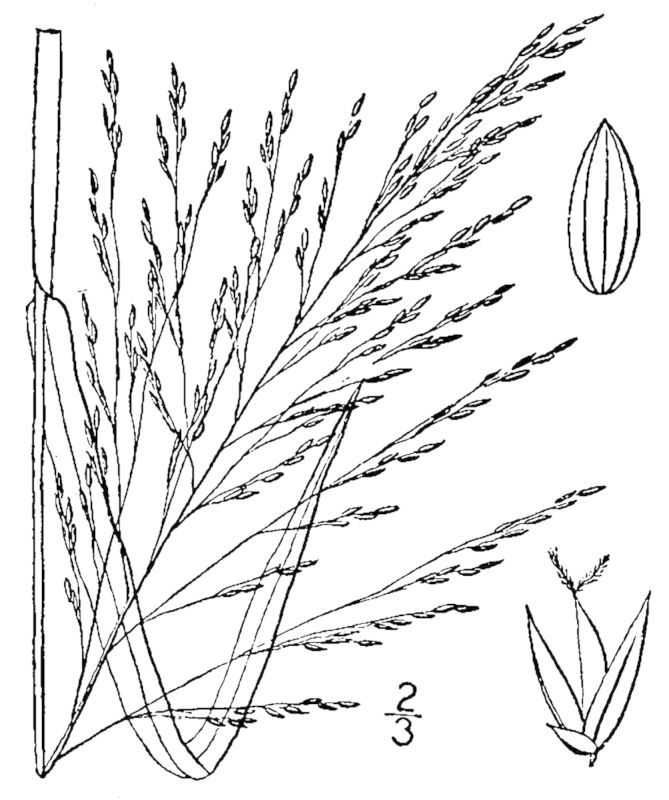
*Panicum dichotomiflorum* (from Britton and Brown 1913).

**Figure 89b.**
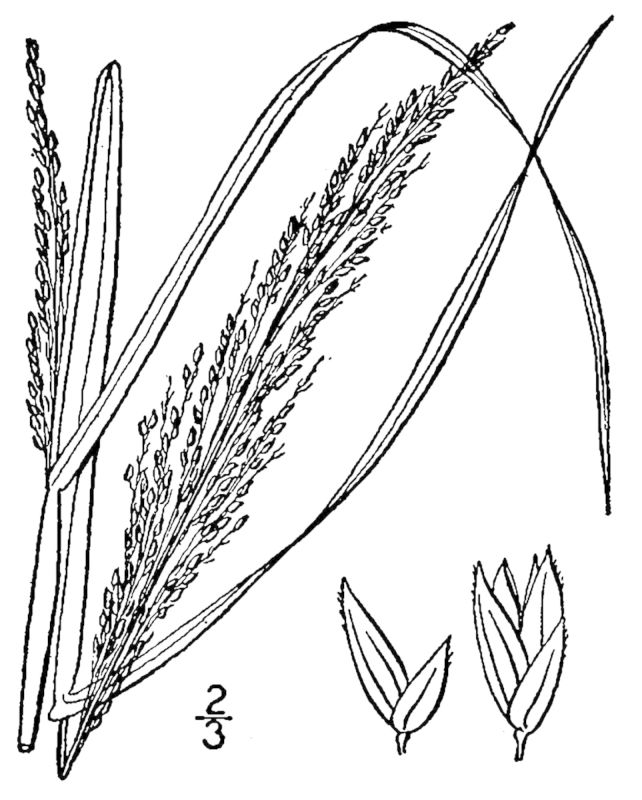
*Panicum hemitomon* (from Britton and Brown 1913).

**Figure 89c.**
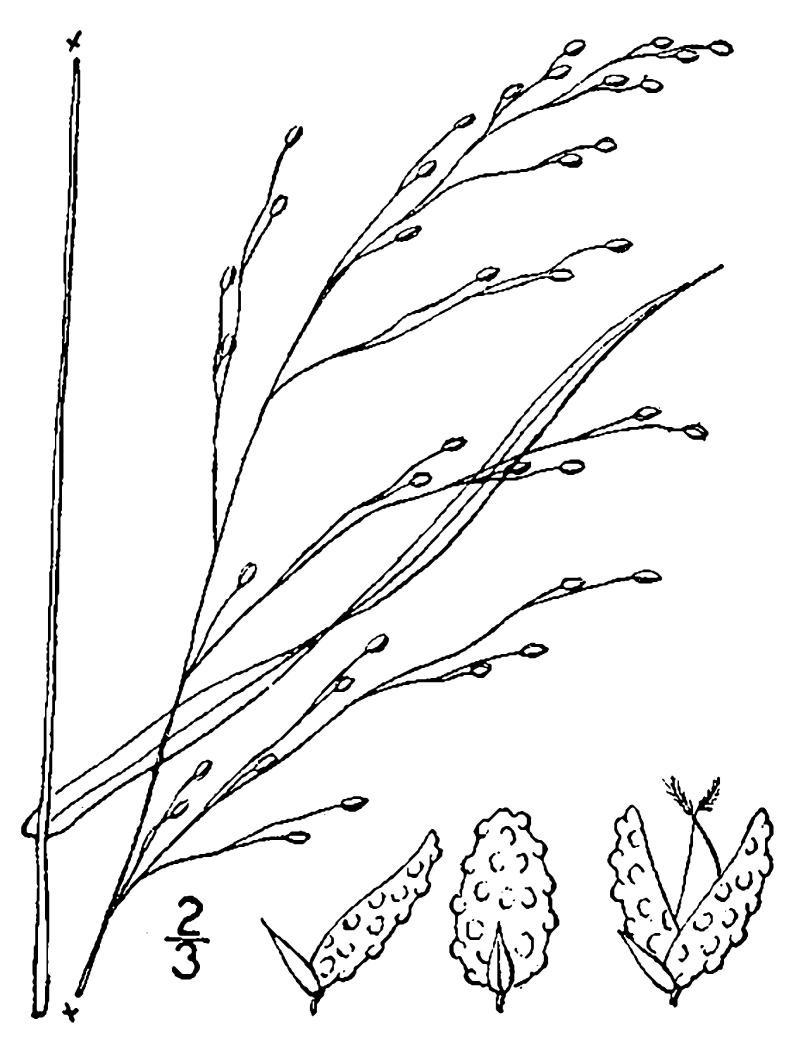
*Panicum verrucosum* (from Britton and Brown 1913).

**Figure 89d.**
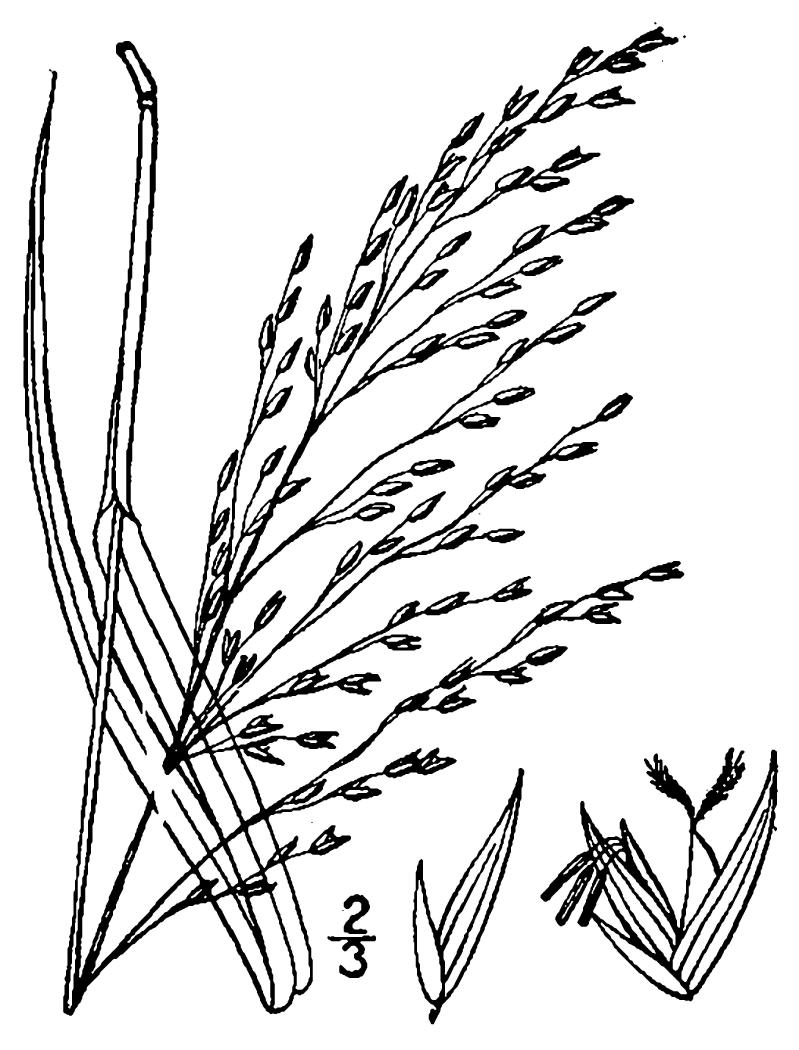
*Panicum virgatum* (from Britton and Brown 1913).

**Figure 90a.**
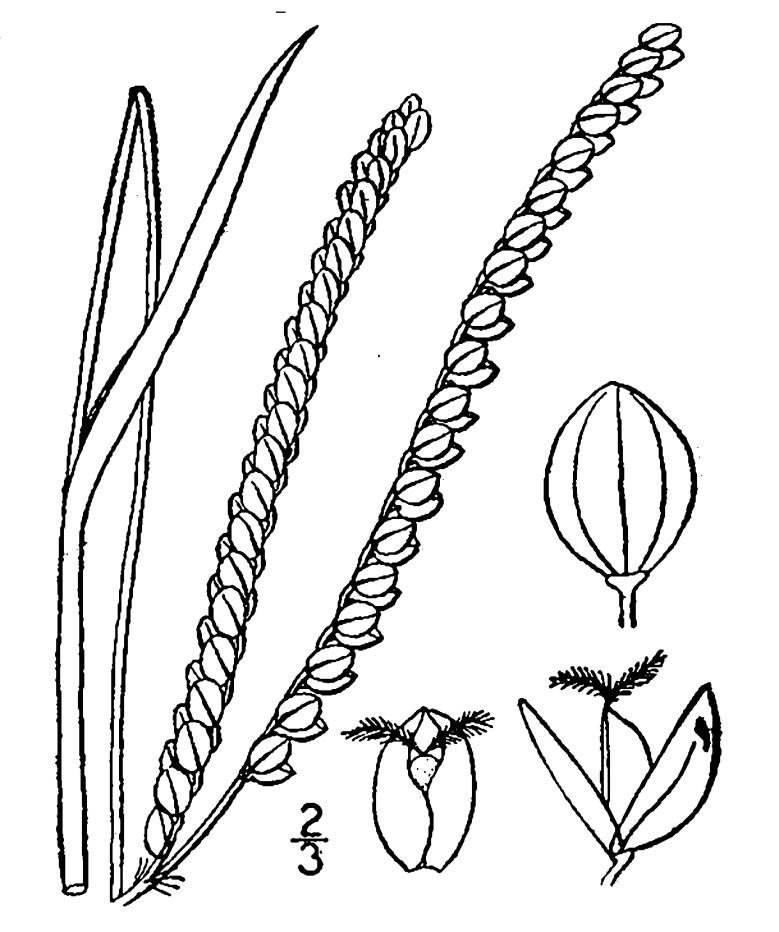
*Paspalum floridanum* (from Hitchcock 1950).

**Figure 90b.**
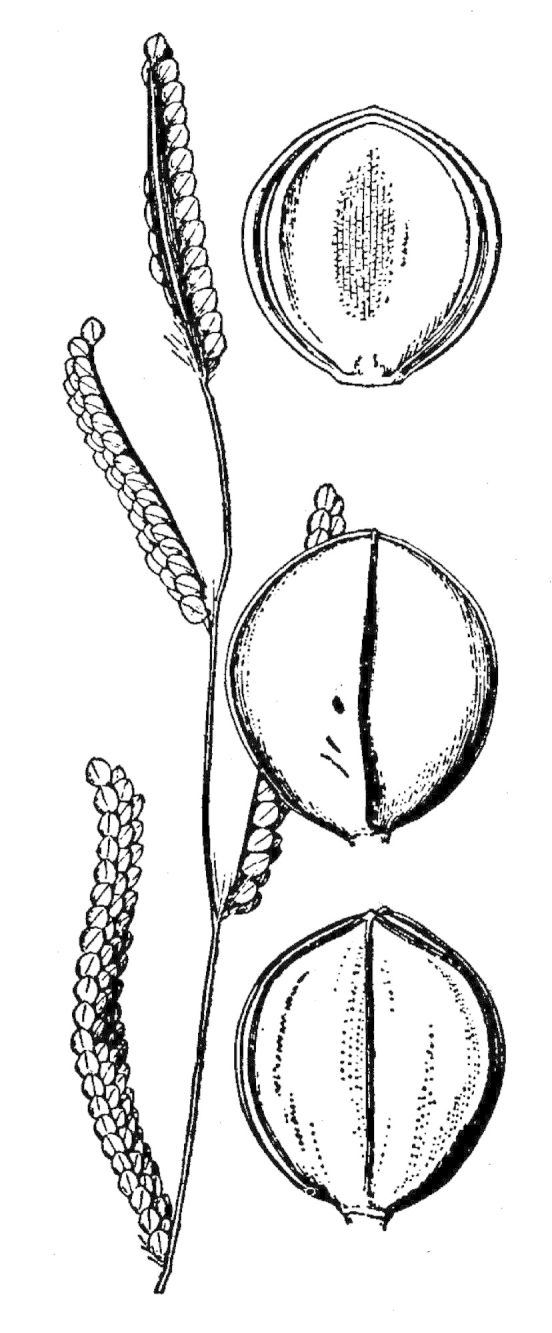
*Paspalum praecox* (from Hitchcock 1950).

**Figure 90c.**
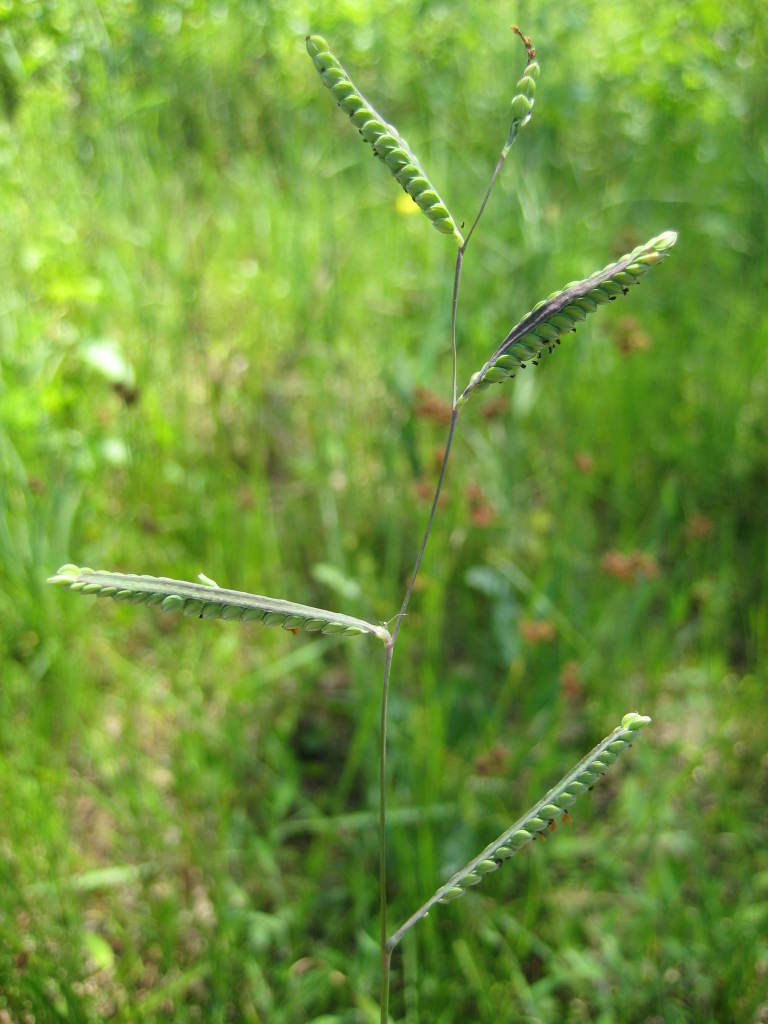
Paspalum praecox var. praecox (photo by R. Thornhill).

**Figure 90d.**
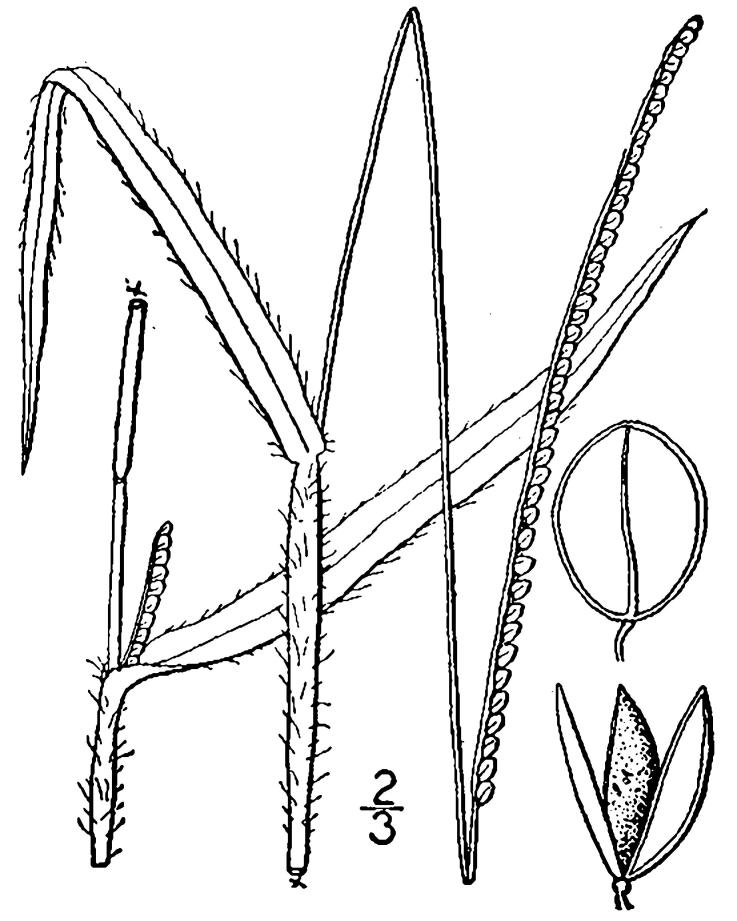
*Paspalum setaceum* (from Britton and Brown 1913).

**Figure 91a.**
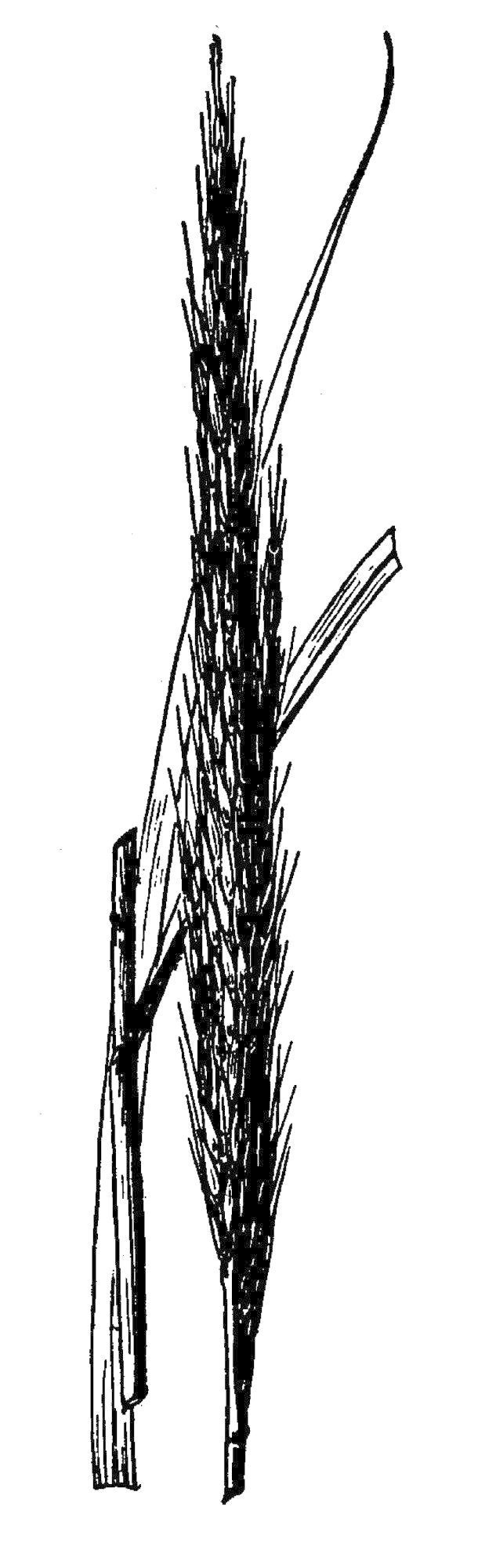
*Scleria baldwinii* (from Hitchcock 1950).

**Figure 91b.**
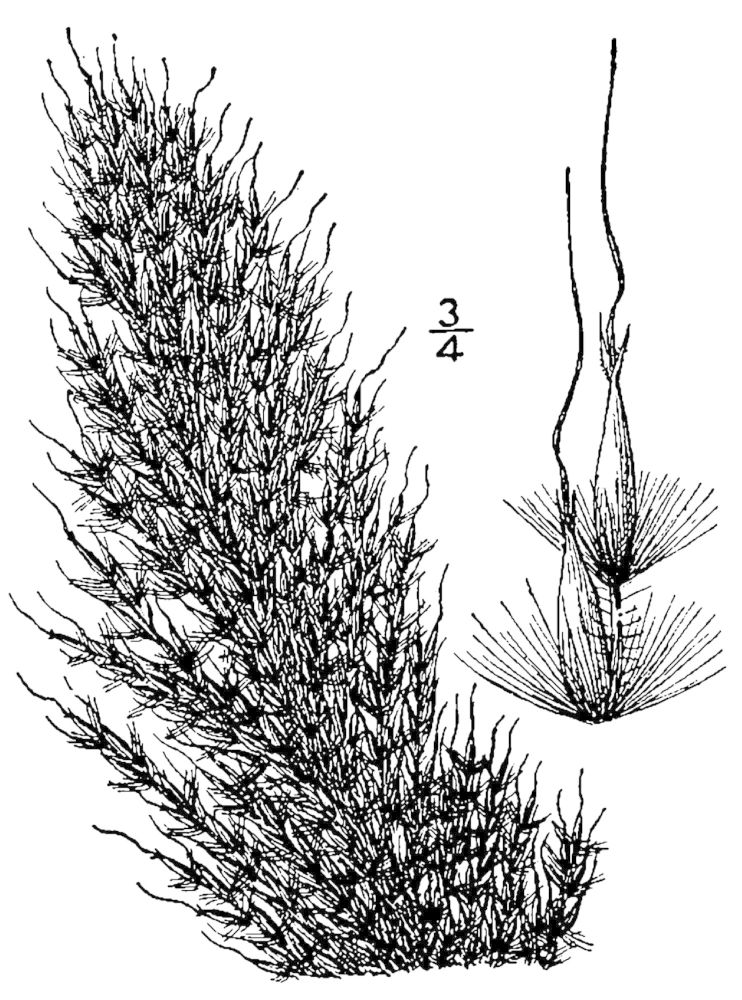
Saccharum brevibarbe var. contortum (from Hitchcock 1950).

**Figure 91c.**
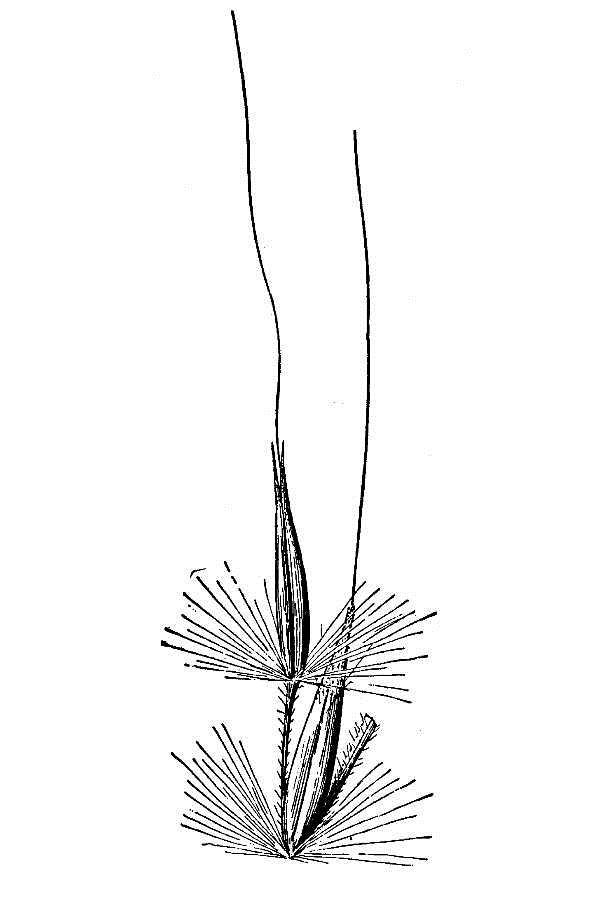
*Saccharum coarctatum* (from Hitchcock 1950).

**Figure 91d.**
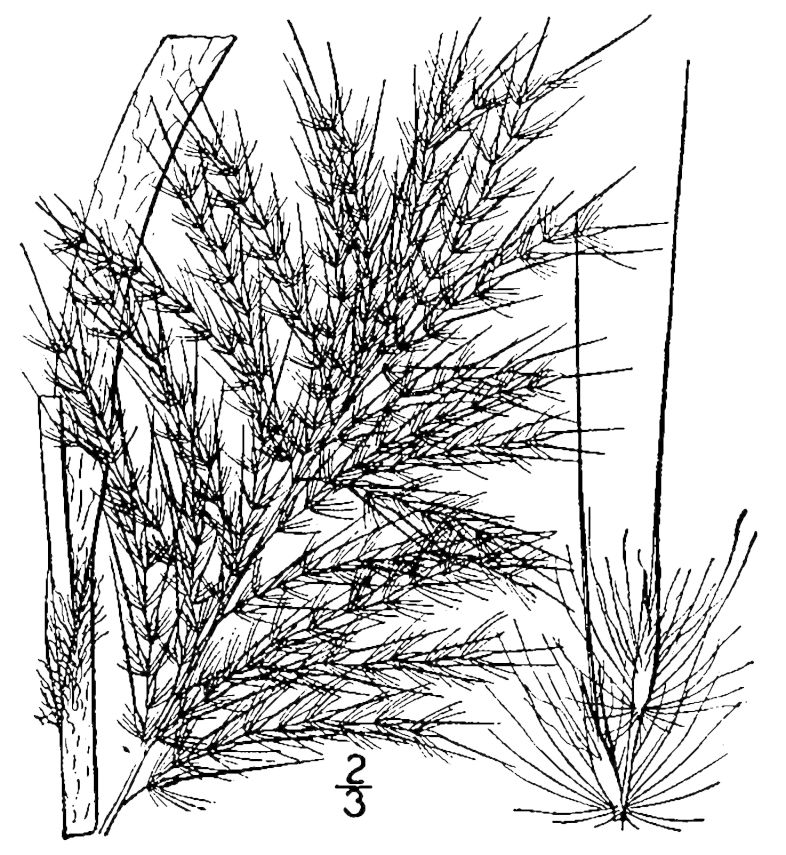
*Saccharum giganteum* (from Britton and Brown 1913).

**Figure 92a.**
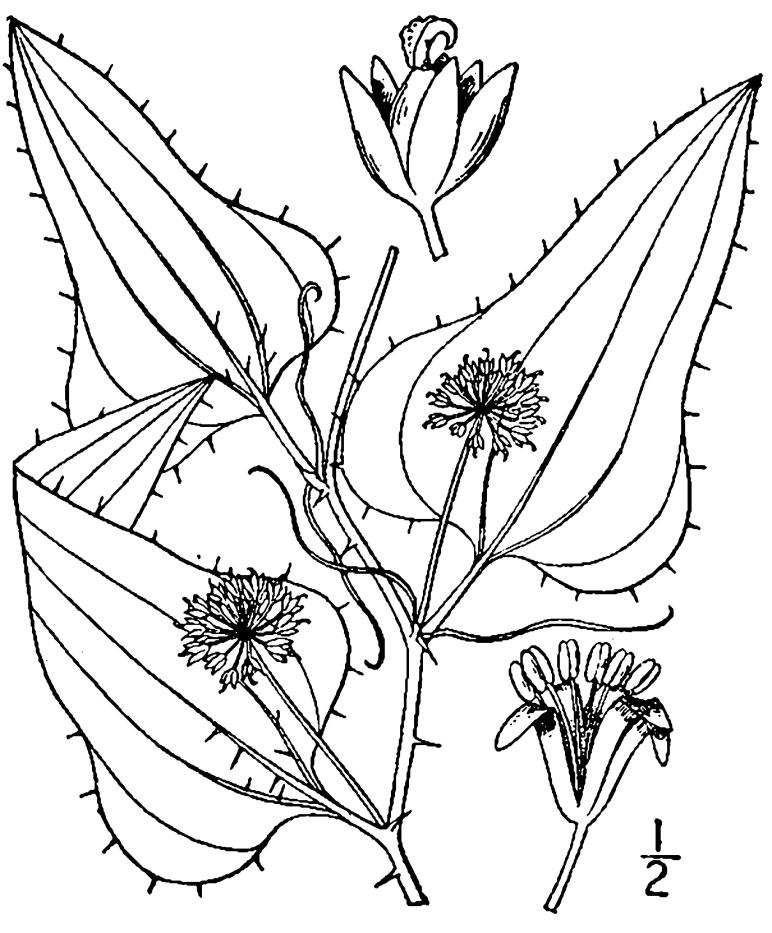
*Smilax bona-nox*: note that the marginal prickles shown in this illustration, though not always present in this taxon, are diagnostic when present (from Britton and Brown 1913).

**Figure 92b.**
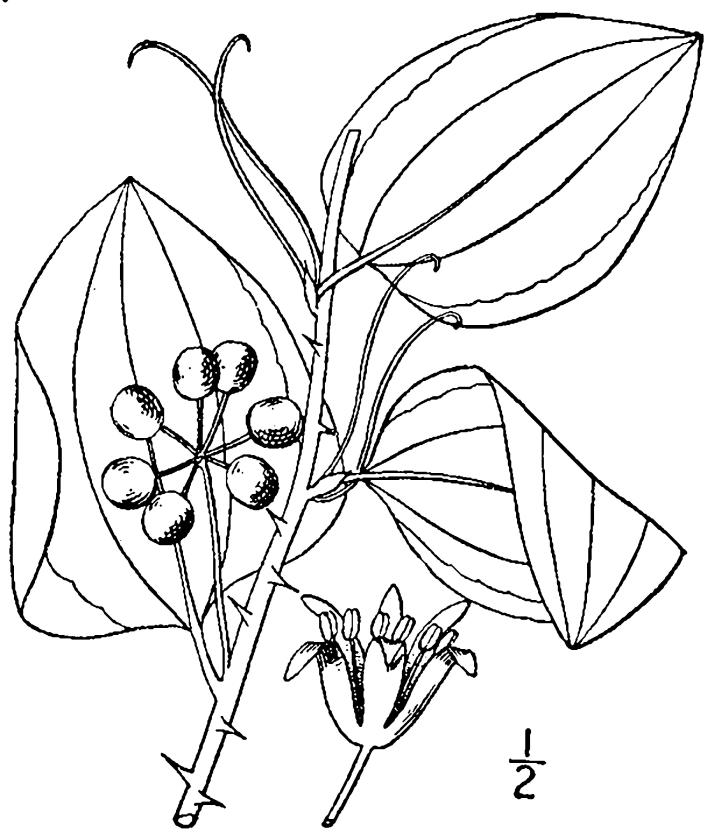
*Smilax glauca* (from Britton and Brown 1913).

**Figure 92c.**
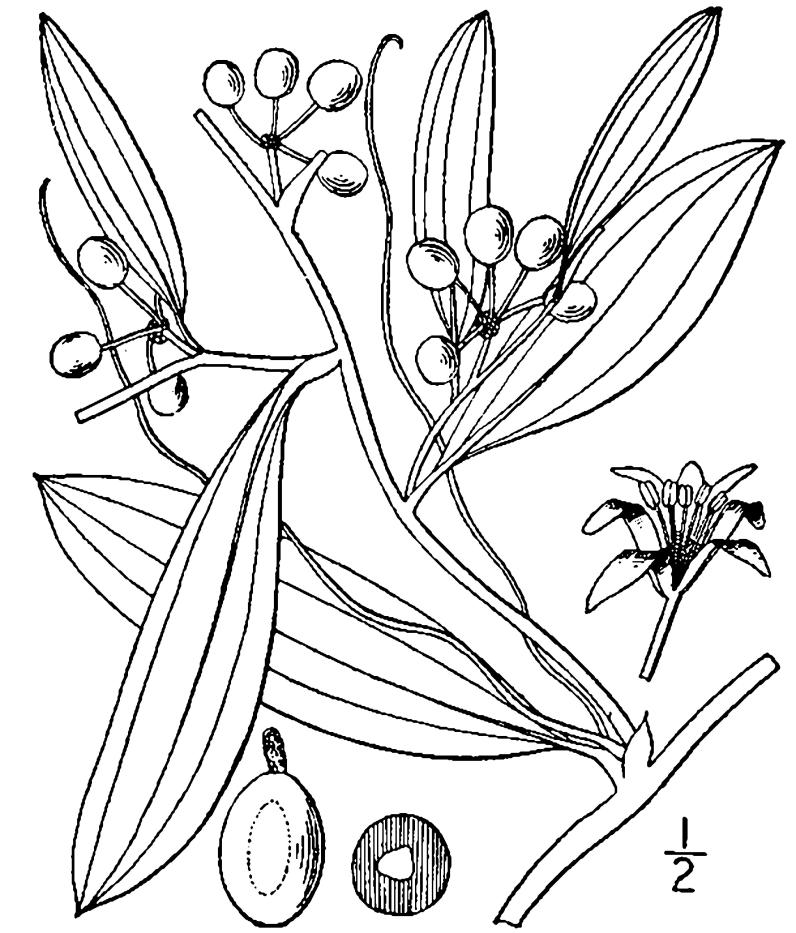
*Smilax laurifolia* (from Britton and Brown 1913).

**Figure 92d.**
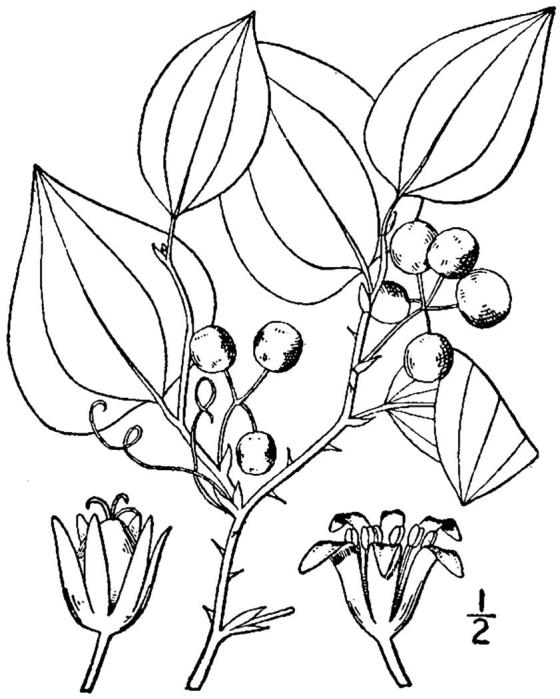
*Smilax rotundifolia* (from Britton and Brown 1913).

**Figure 92e.**
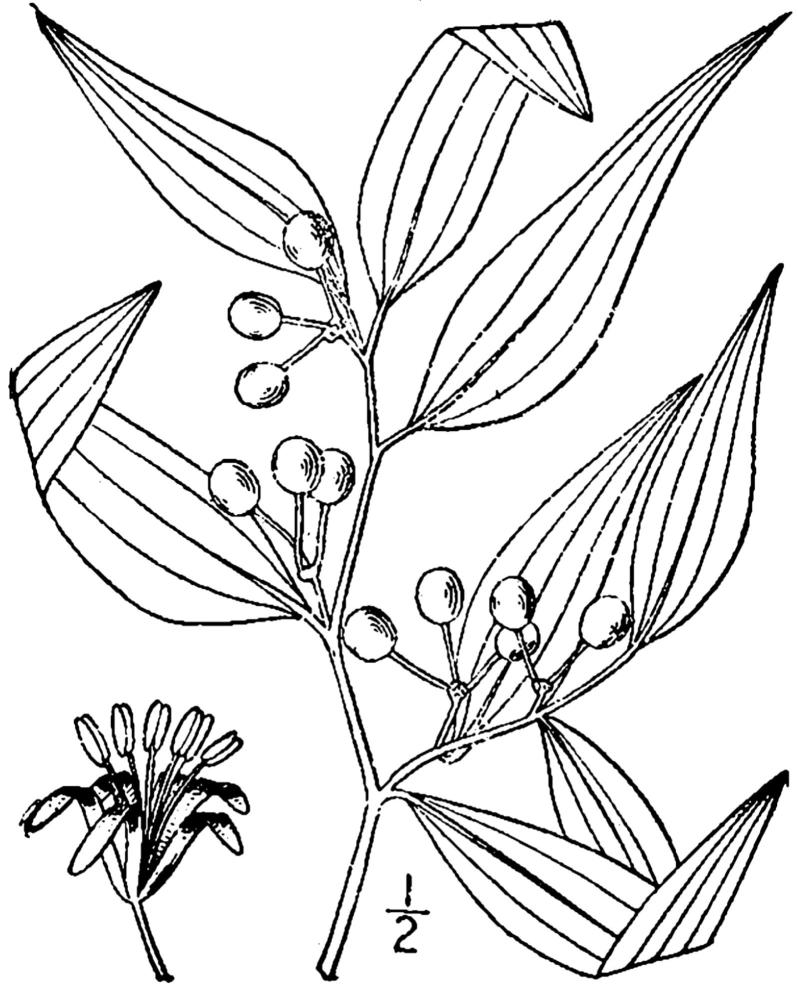
*Smilax smallii* (from Britton and Brown 1913).

**Figure 93a.**
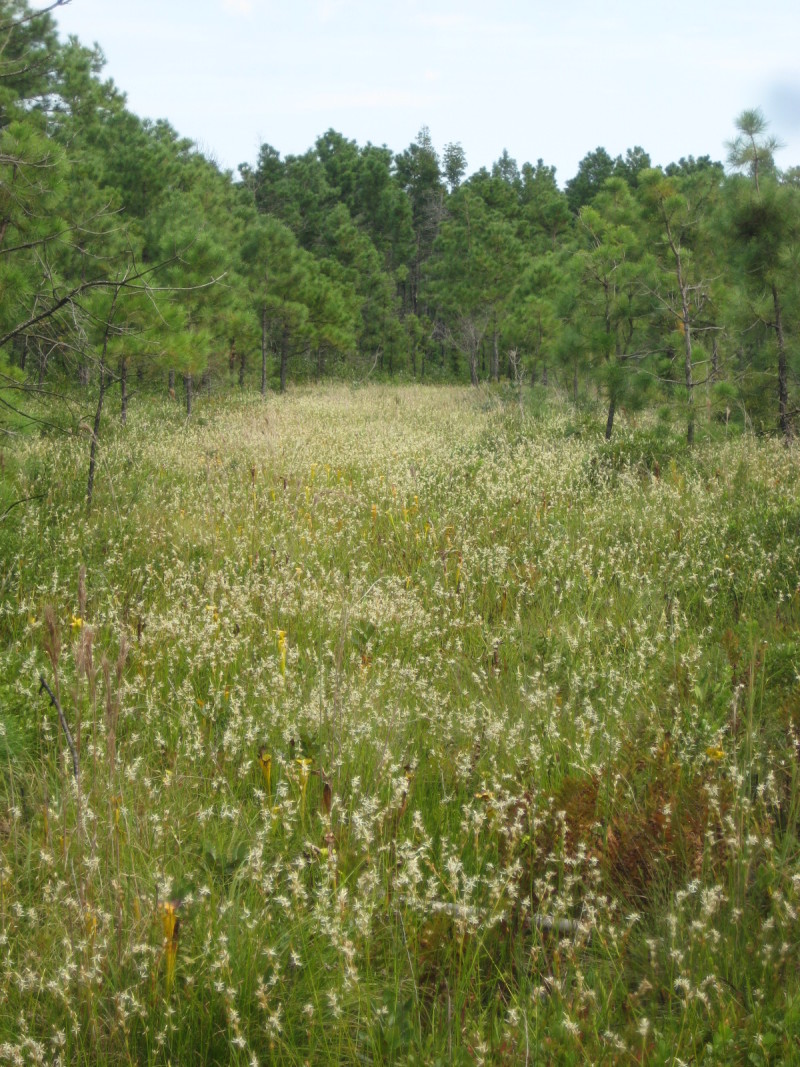
"Snow in September": abundance of flowering Pleea tenuifolia in mid-September (photo by R. Thornhill).

**Figure 93b.**
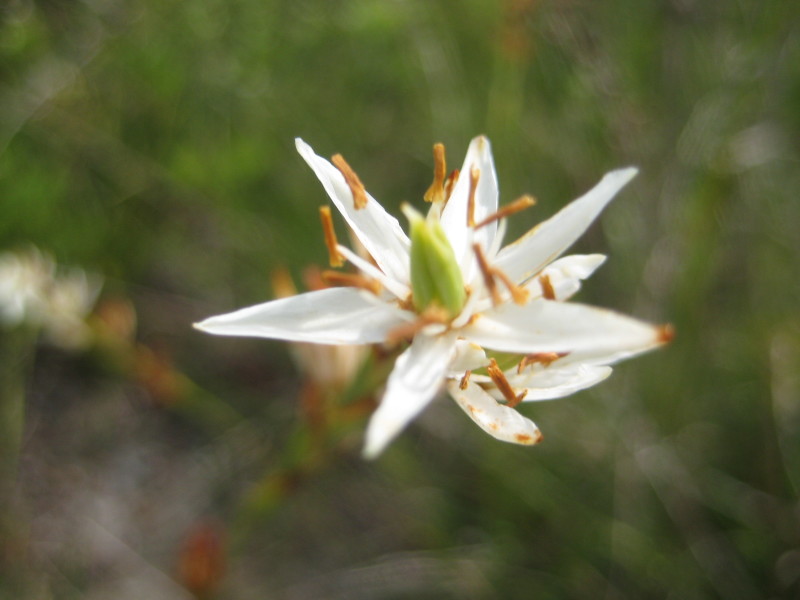
Close-up of flower (photo by R. Thornhill).

**Figure 94.**
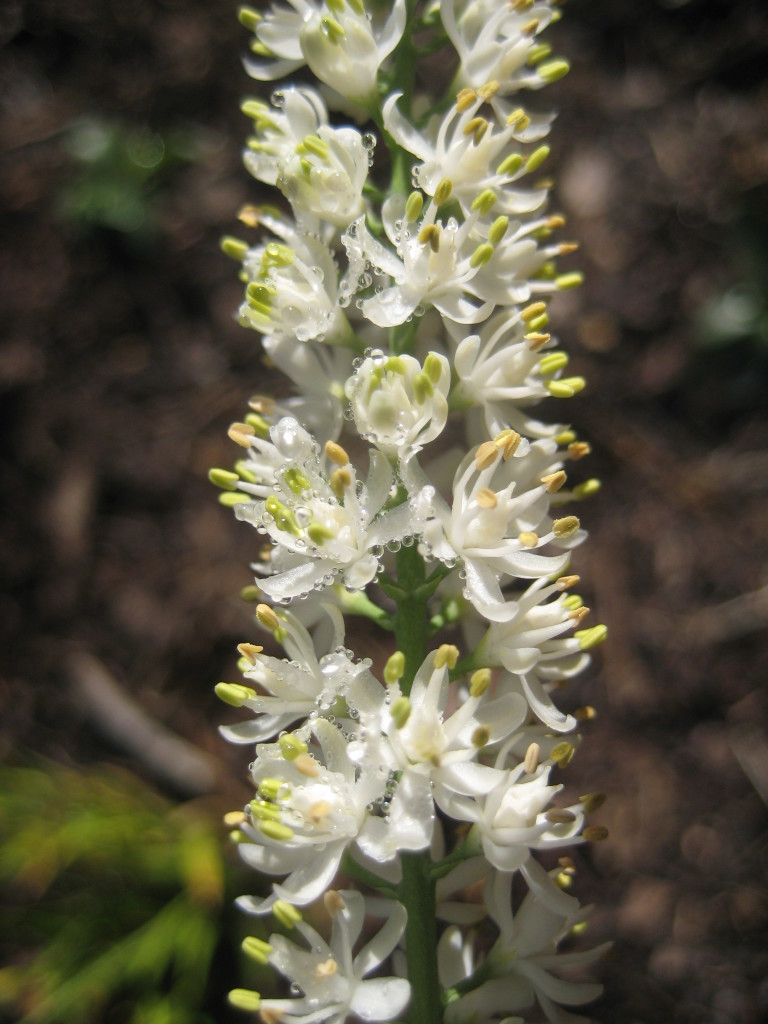
*Tofieldia glabra* (photo by R. Thornhill).

**Figure 95a.**
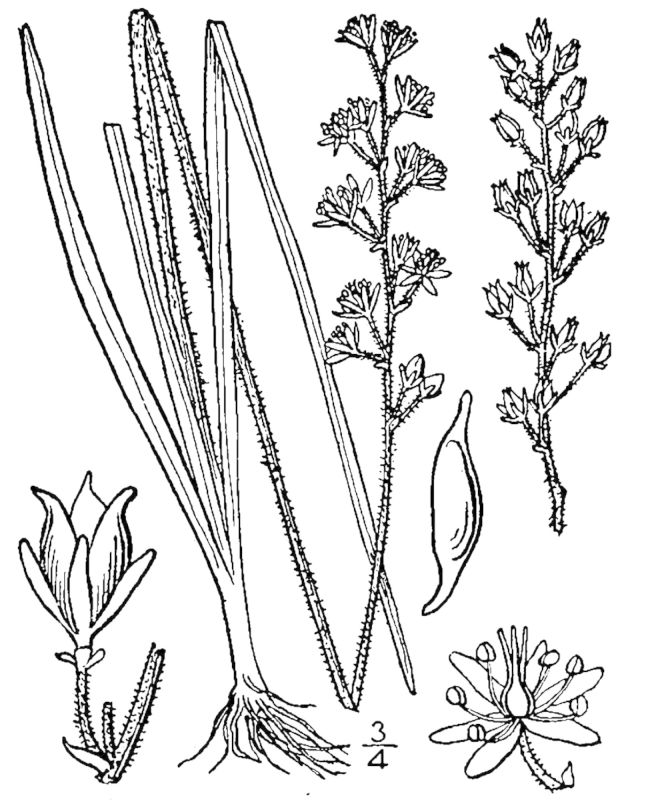
From Britton and Brown (1913).

**Figure 95b.**
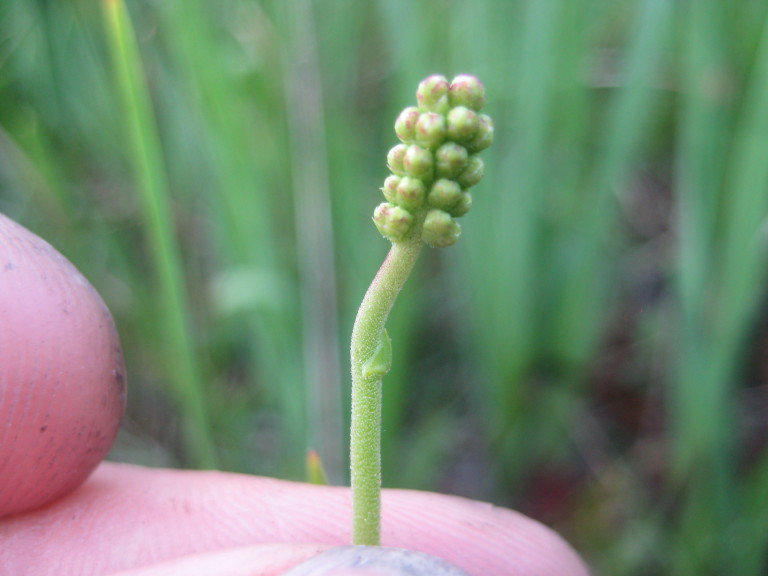
Close-up of flower buds (photo by R. Thornhill).

**Figure 96a.**
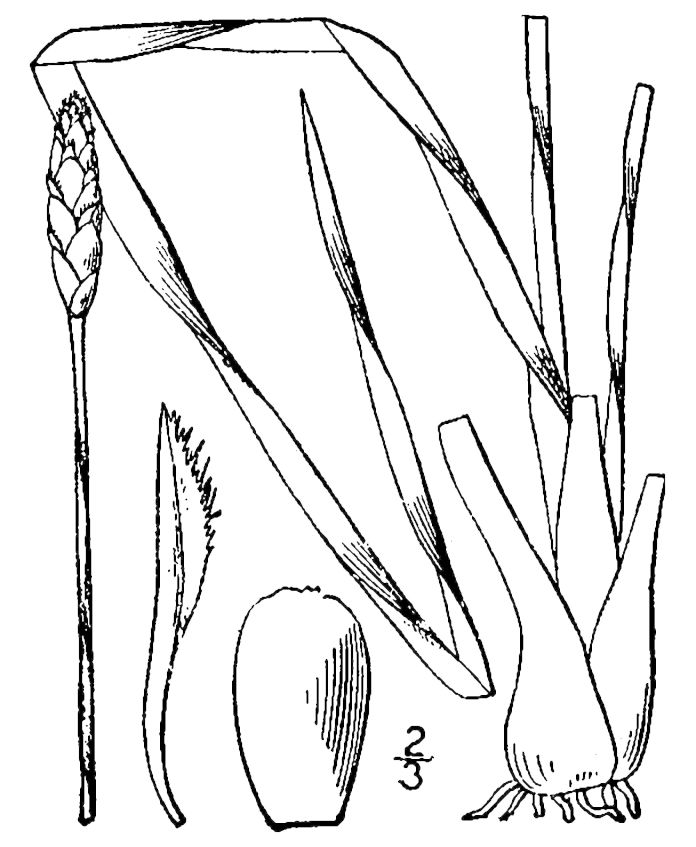
From Britton and Brown (1913).

**Figure 96b.**
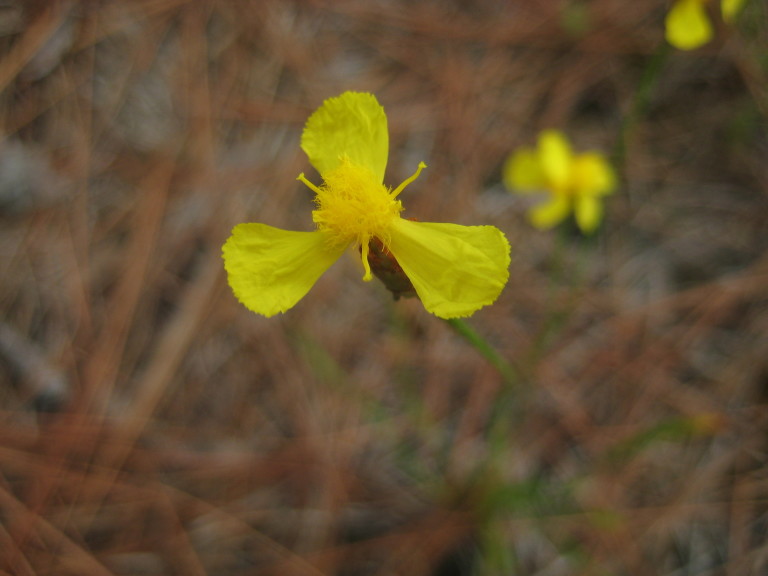
Close-up of flower (photo by R. Thornhill).

**Figure 96c.**
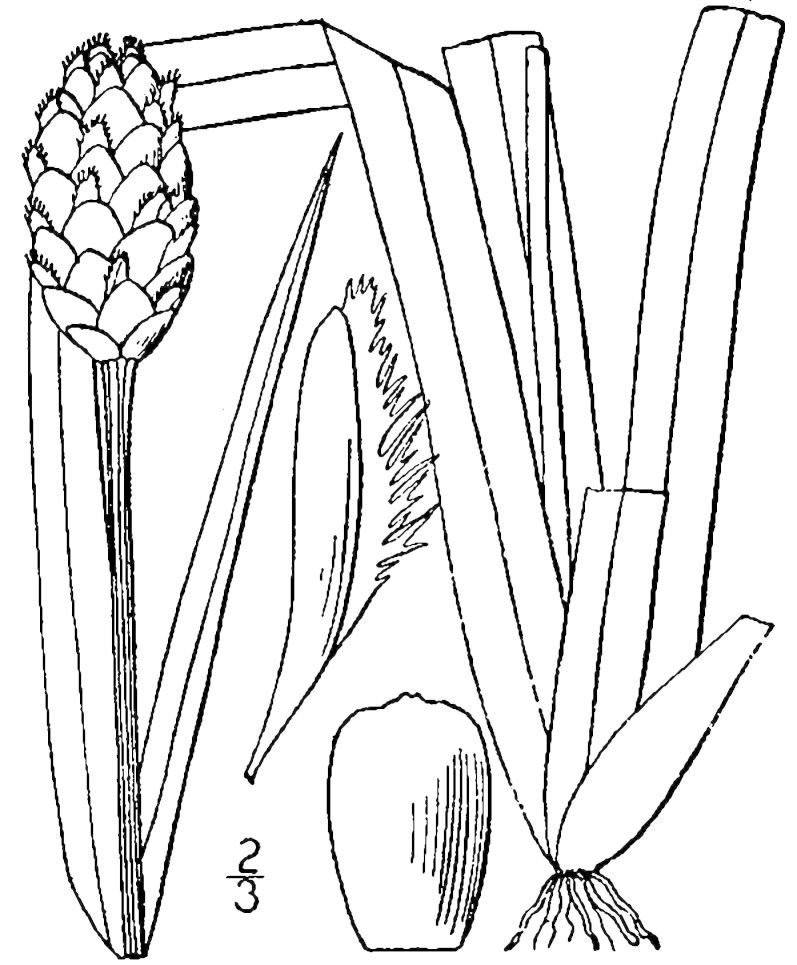
*Xyris fimbriata* (from Britton and Brown 1913).

**Figure 96d.**
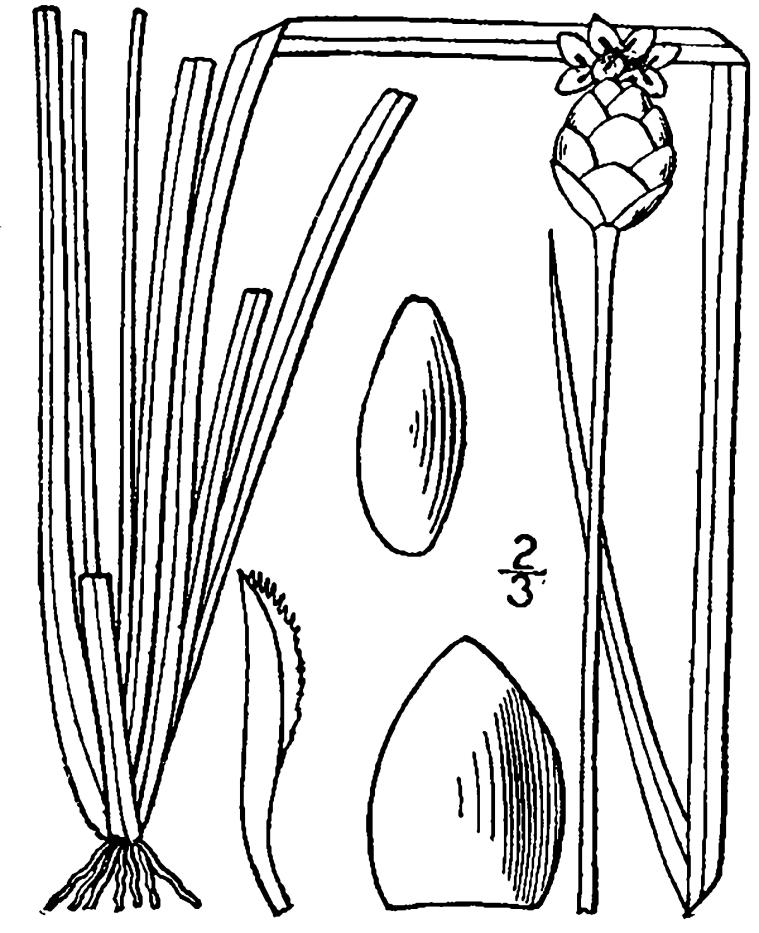
*Xyris jupicai* (from Britton and Brown 1913).

**Figure 96e.**
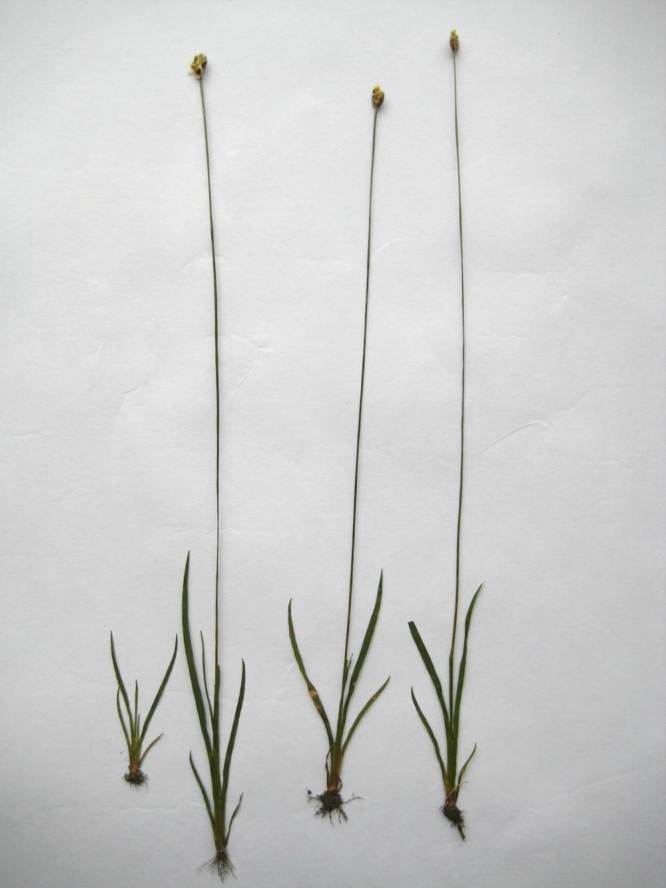
*Xyris* species 1 (photo of Thornhill 902 specimen by R. Thornhill).

**Figure 97.**
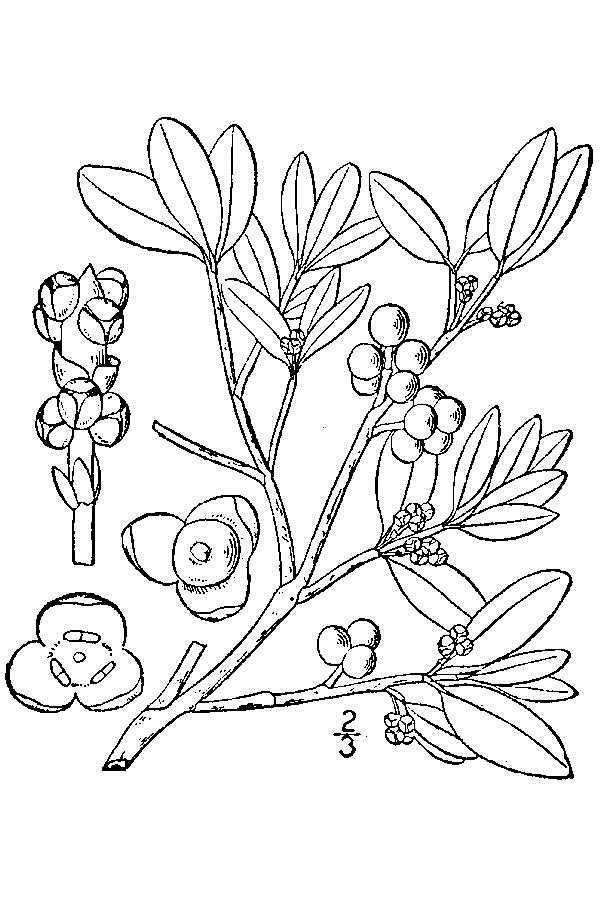
*Phoradendron leucarpum* (from Britton and Brown 1913).

**Figure 98.**
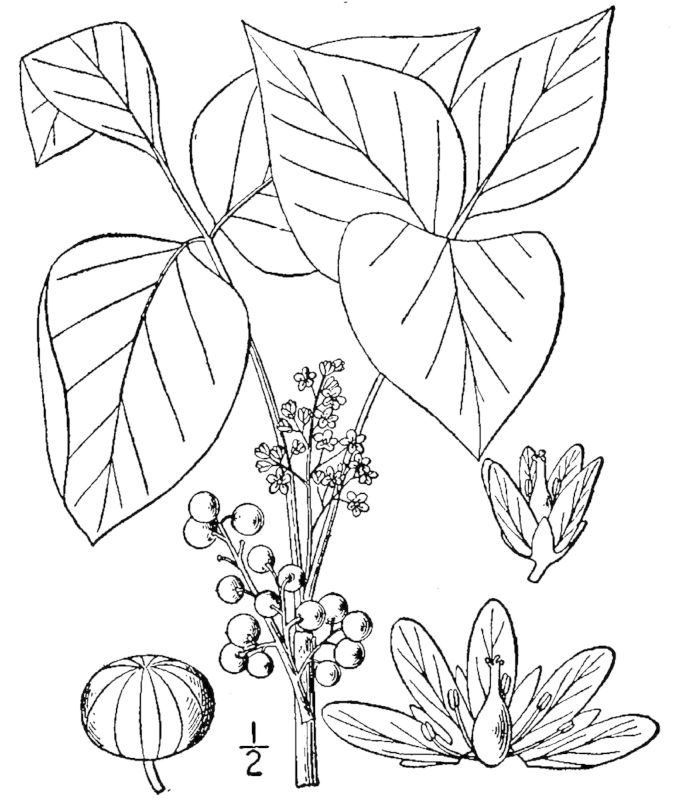
*Toxicodendron radicans* (from Britton and Brown 1913).

**Figure 99.**
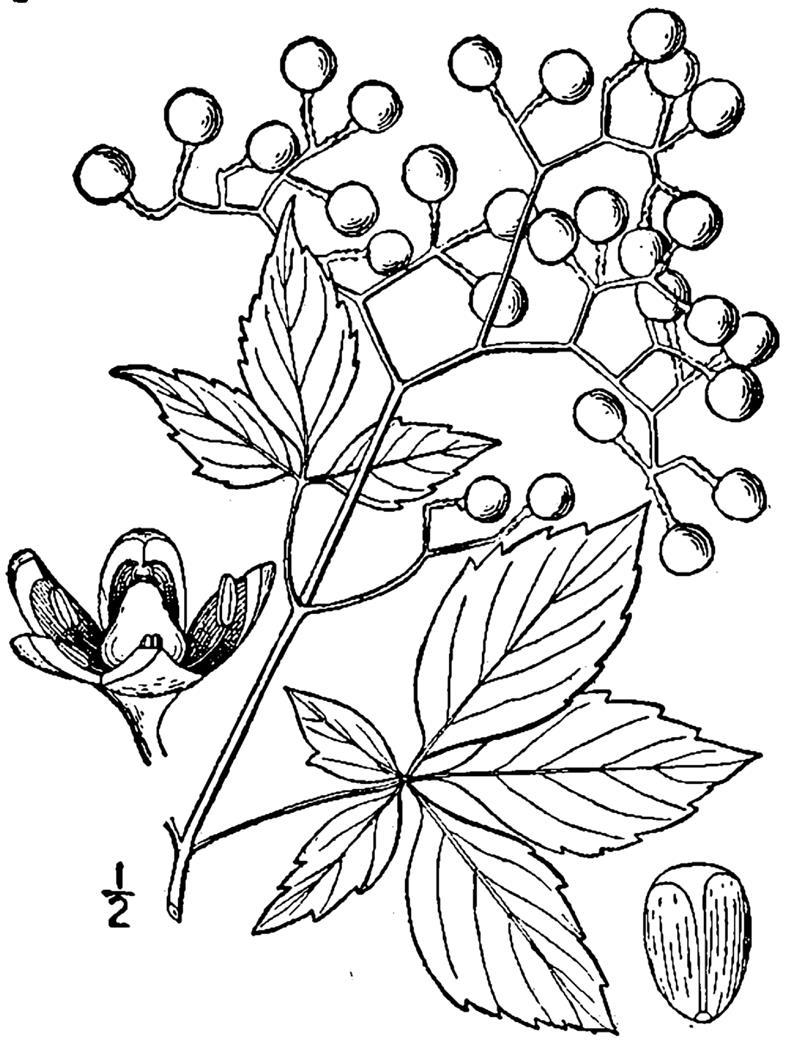
*Parthenocissus quinquefolia* (from Britton and Brown 1913).

**Figure 100.**
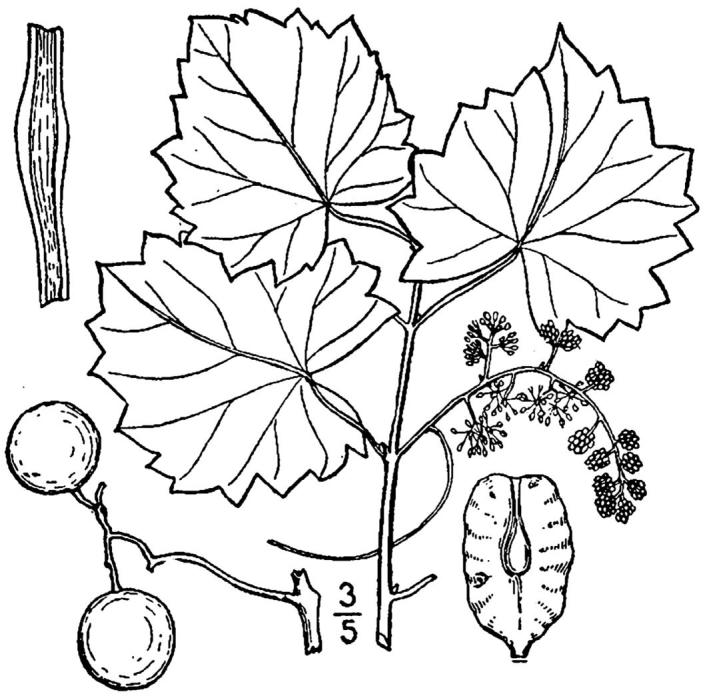
*Vitis rotundifolia* (from Britton and Brown 1913).

**Figure 101.**
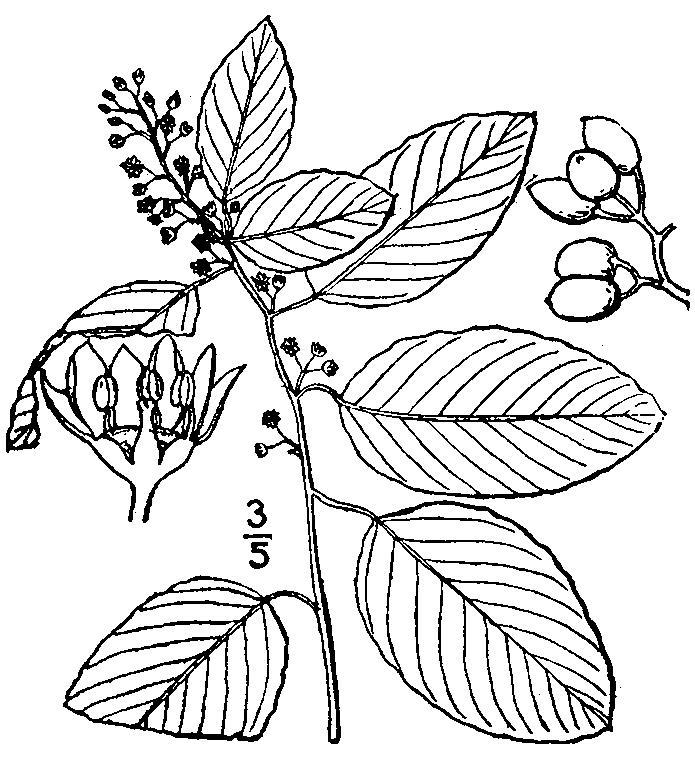
*Berchemia scandens* (from Britton and Brown 1913).

**Figure 102.**
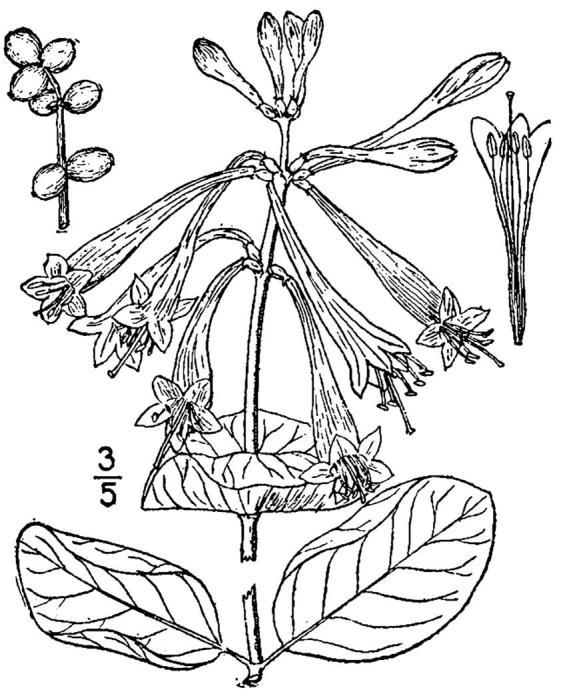
*Lonicera sempervirens* (from Britton and Brown 1913).

**Figure 103.**
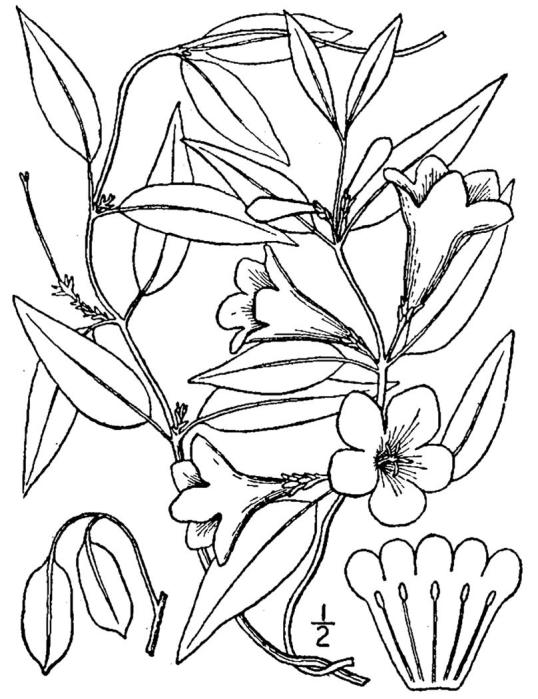
*Gelsemium sempervirens* (from Britton and Brown 1913).

**Figure 104.**
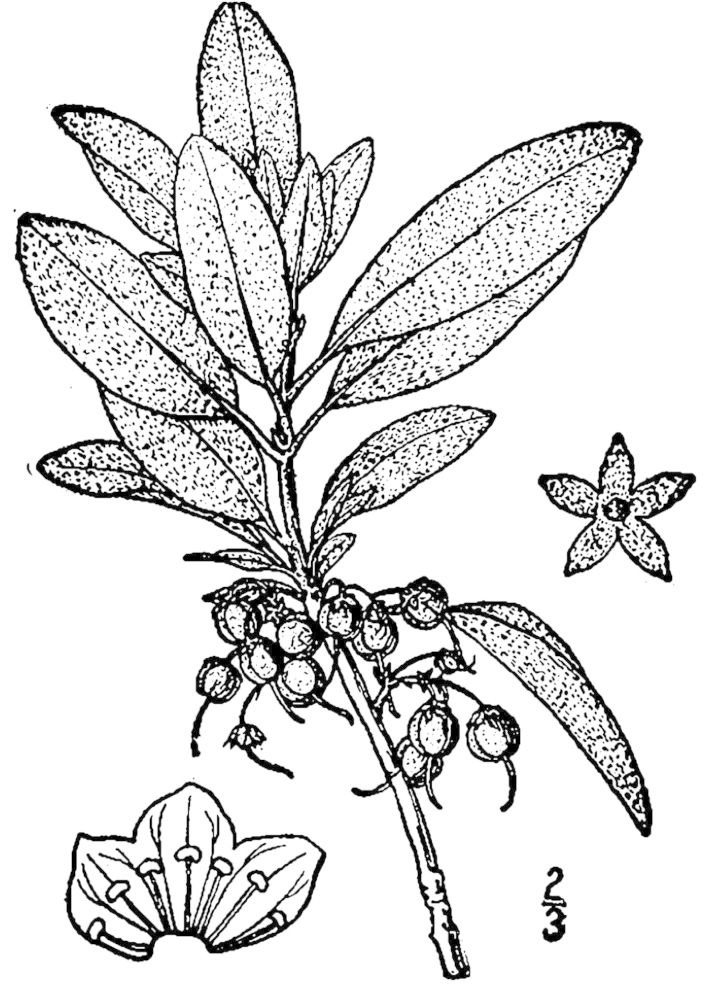
*Kalmia carolina* (from Britton and Brown 1913).

**Figure 105.**
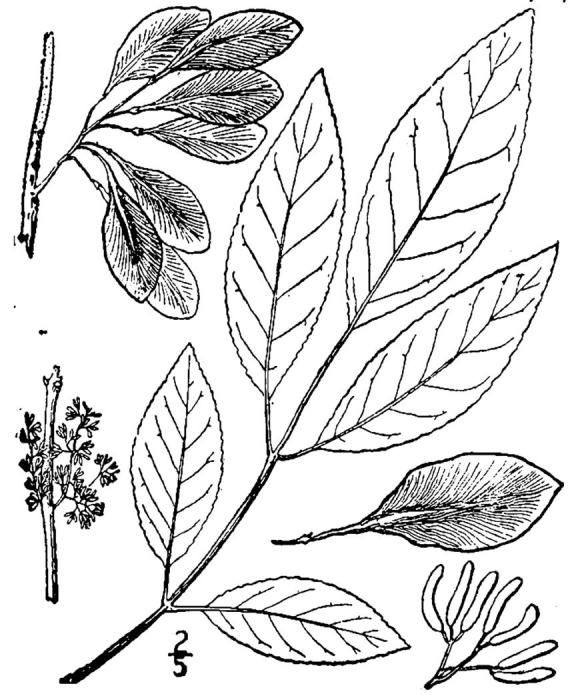
*Fraxinus caroliniana* (from Britton and Brown 1913).

**Figure 106a.**
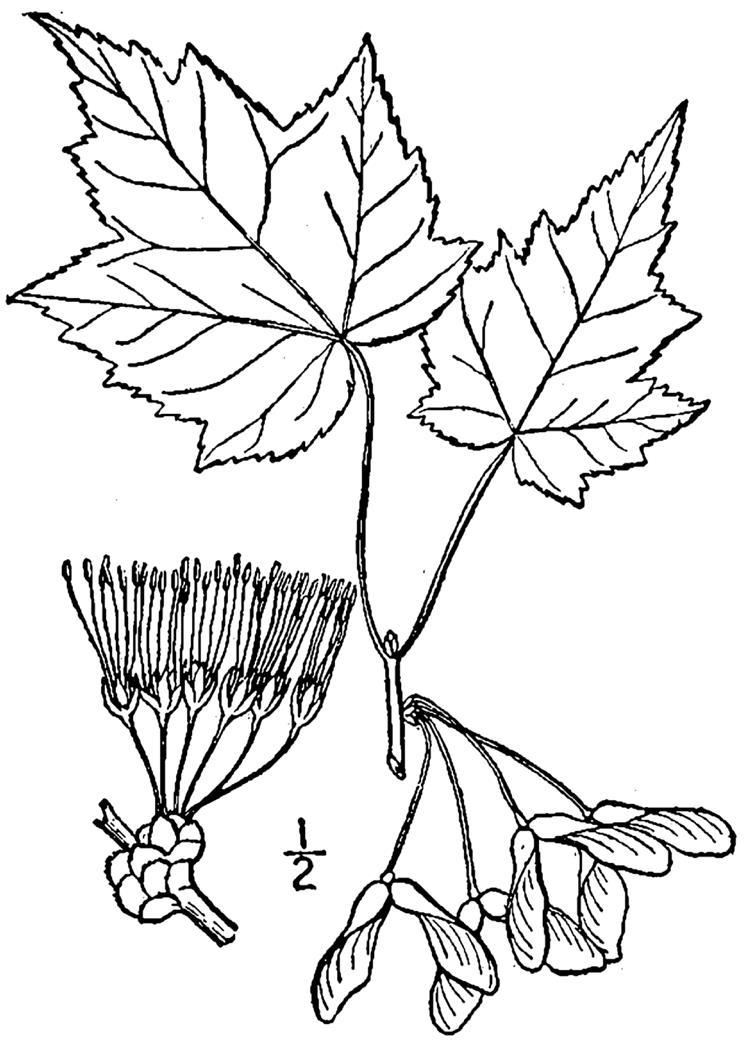
From Britton and Brown (1913).

**Figure 106b.**
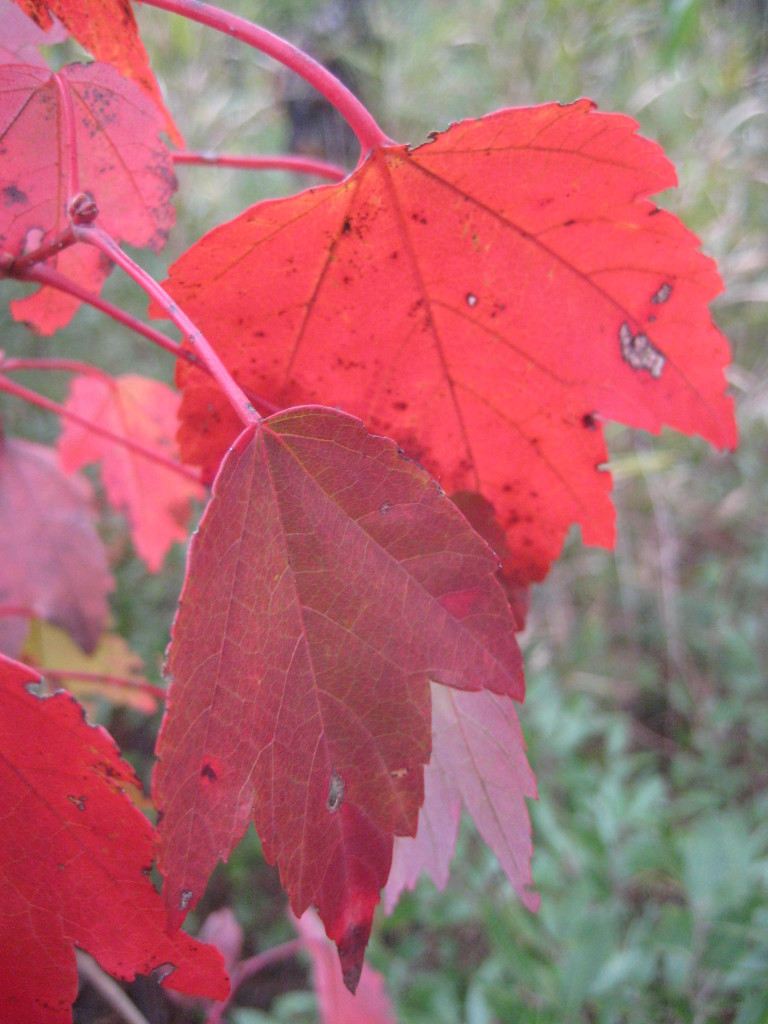
Fall foliage (photo by R. Thornhill).

**Figure 107.**
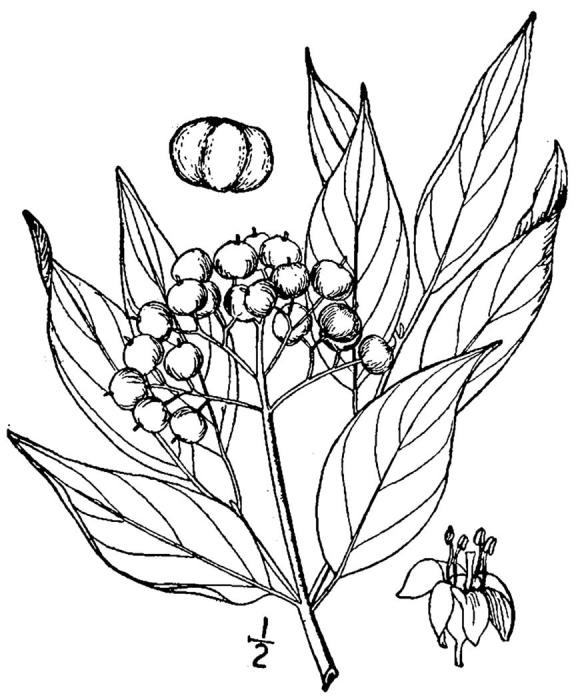
*Cornus stricta* (from Britton and Brown 1913).

**Figure 108.**
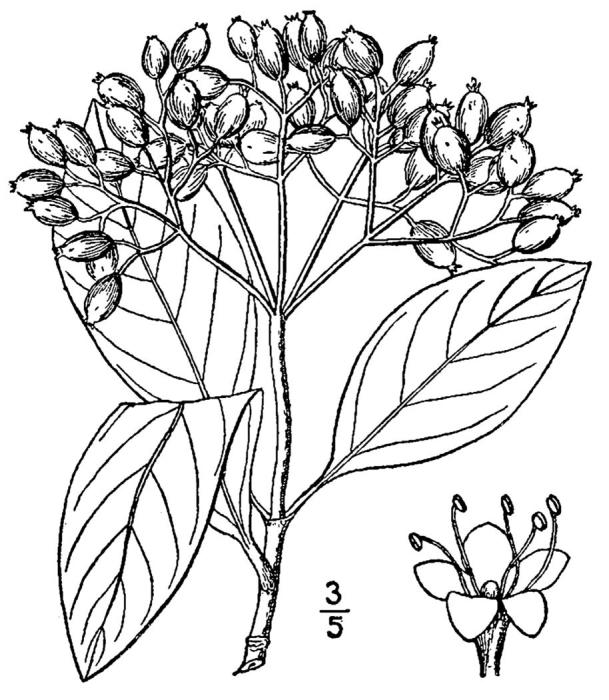
*Viburnum nudum* (from Britton and Brown 1913).

**Figure 109.**
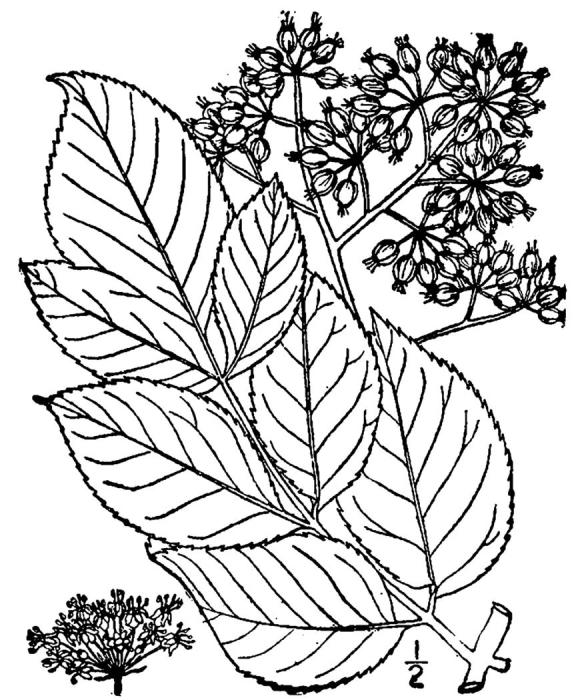
*Aralia spinosa* (from Britton and Brown 1913).

**Figure 110.**
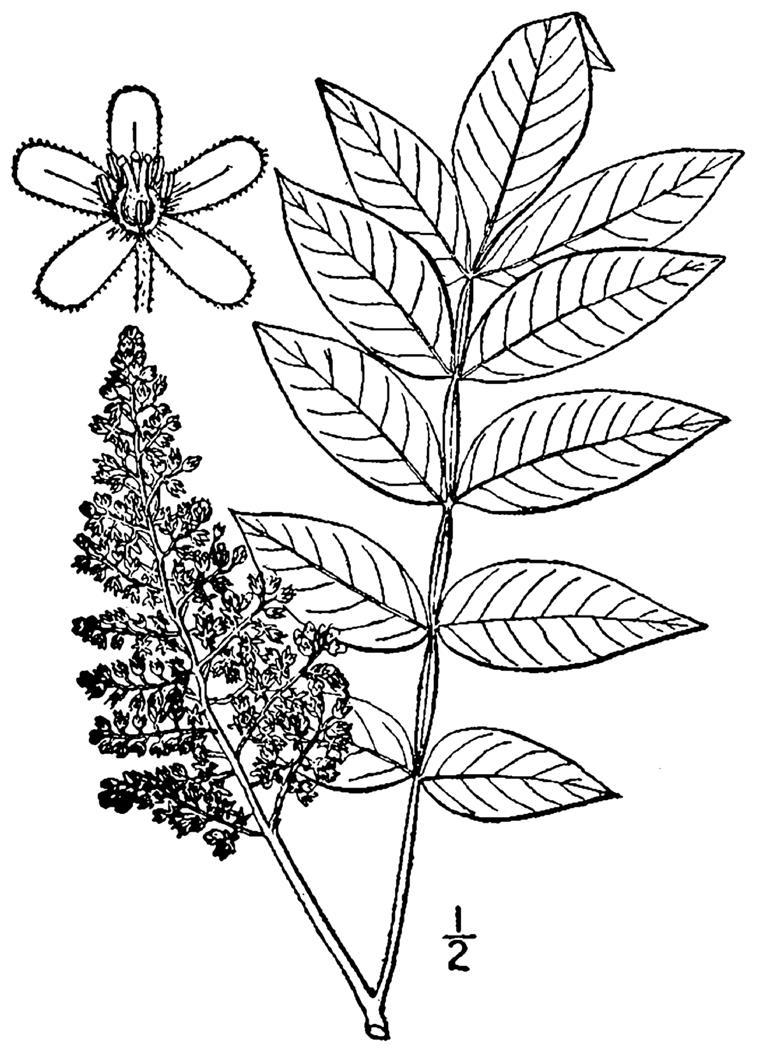
*Rhus copallinum* (from Britton and Brown 1913).

**Figure 111a.**
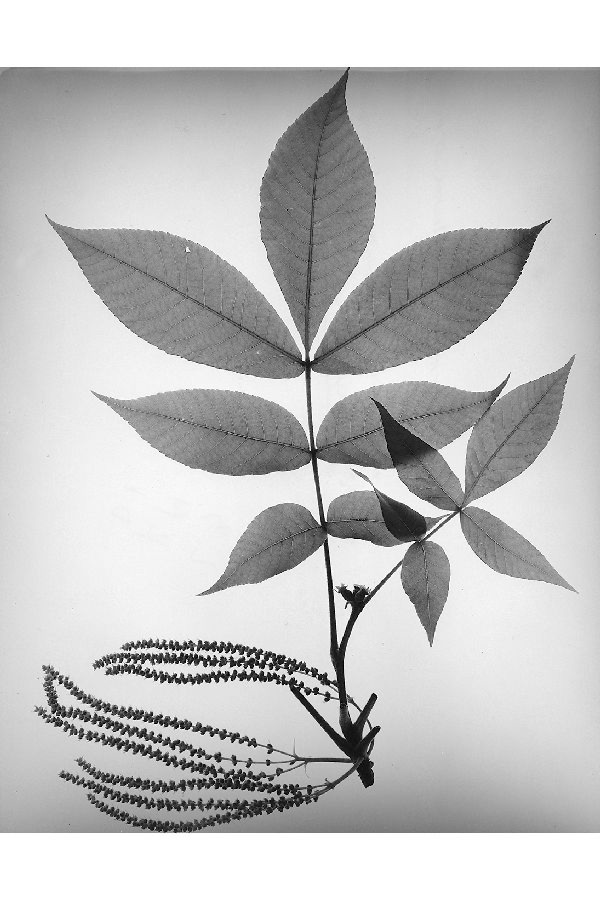
Photo by W.D. Brush (from USDA-NRCS 2012).

**Figure 111b.**
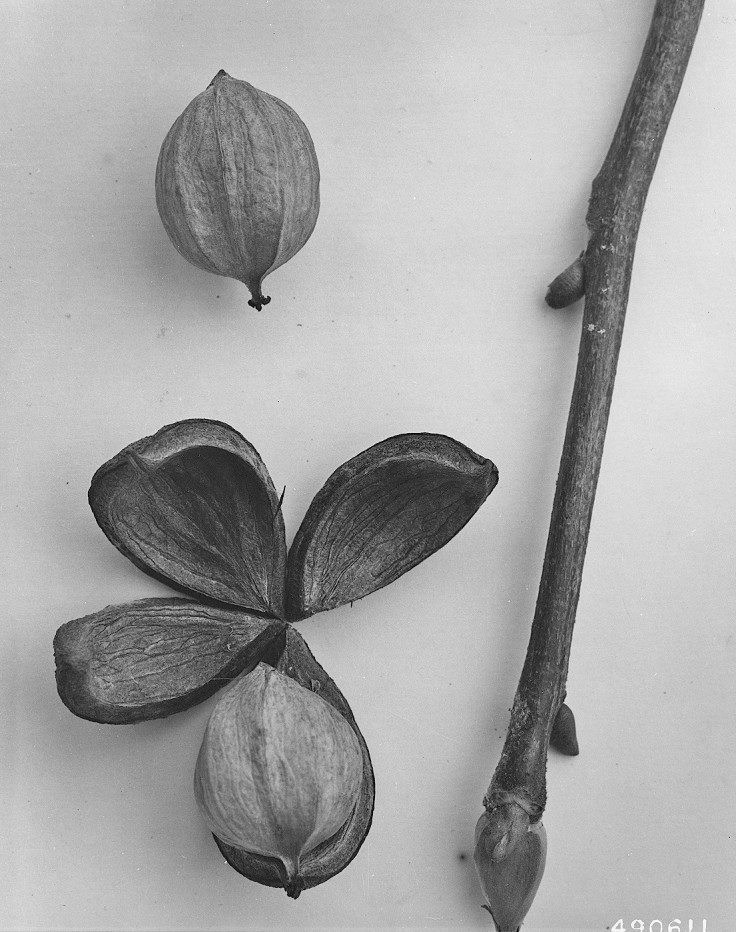
Photo by W.D. Brush (from USDA-NRCS 2012).

**Figure 112.**
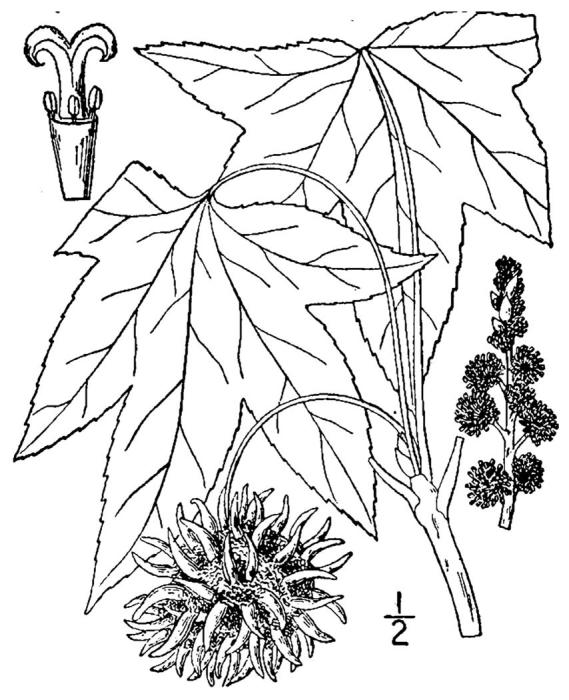
*Liquidambar styraciflua* (from Britton and Brown 1913).

**Figure 113.**
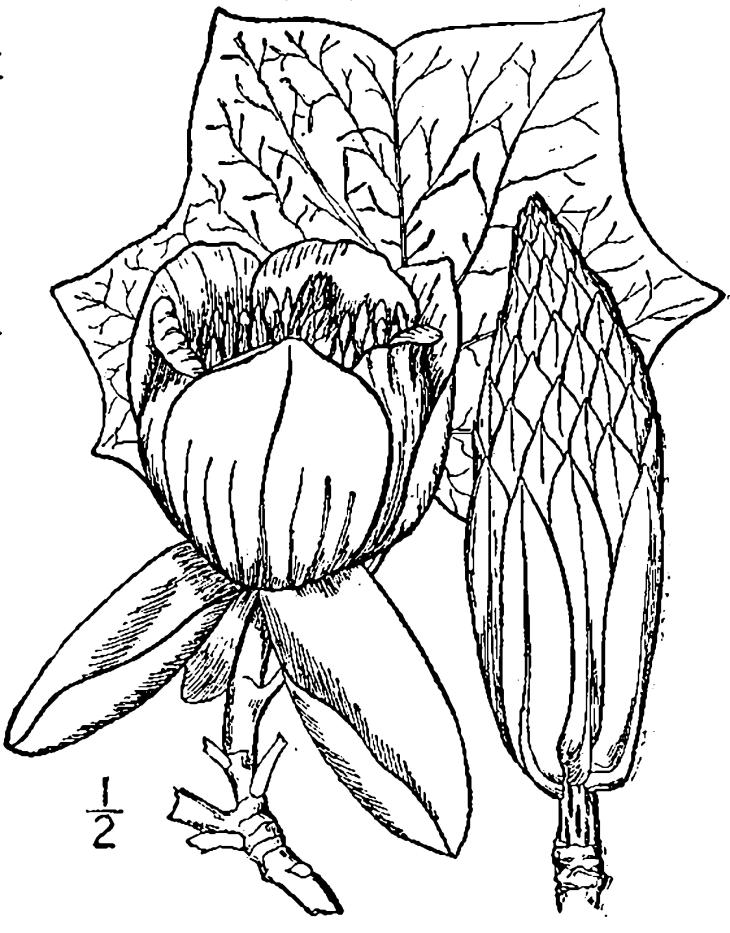
*Liriodendron tulipifera* (from Britton and Brown 1913).

**Figure 114.**
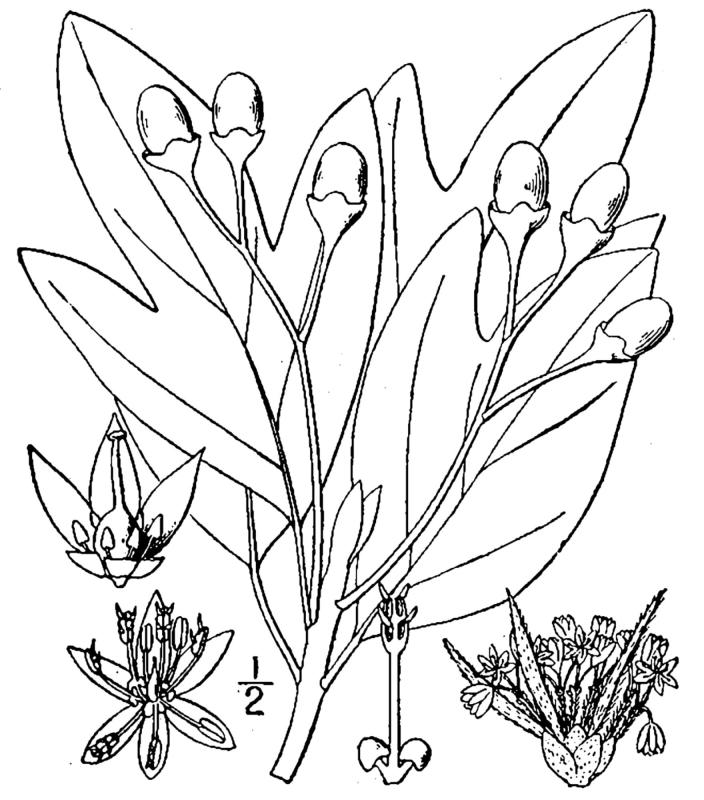
*Sassafras albidum* (from Britton and Brown 1913).

**Figure 115a.**
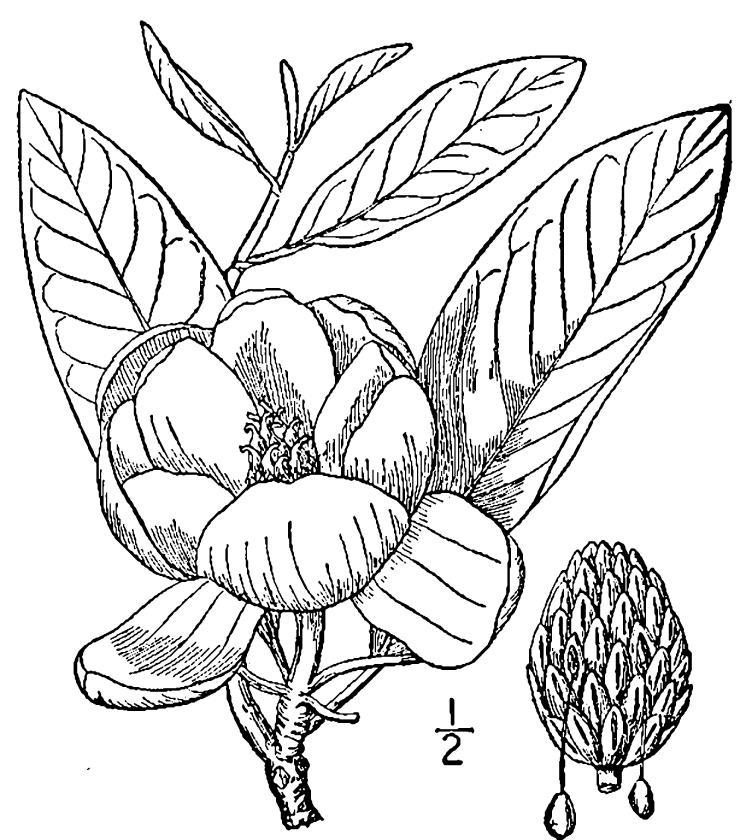
From Britton and Brown (1913).

**Figure 115b.**
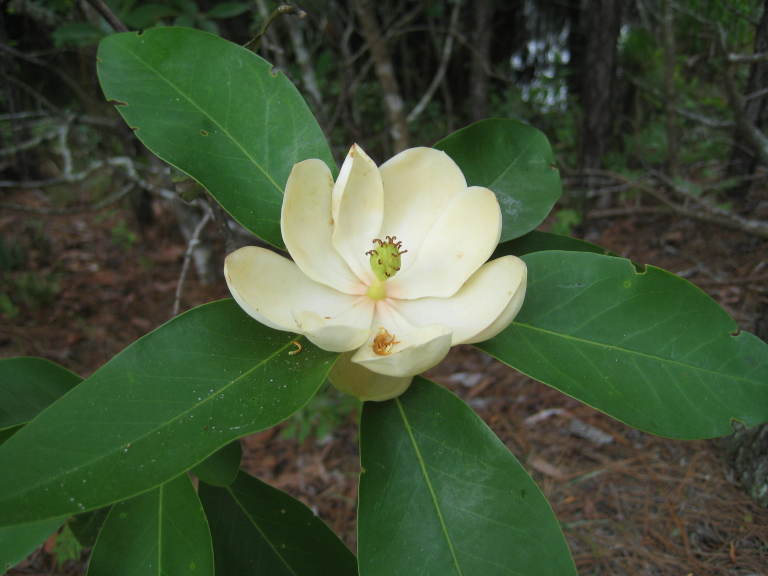
Photo by R. Thornhill.

**Figure 116.**
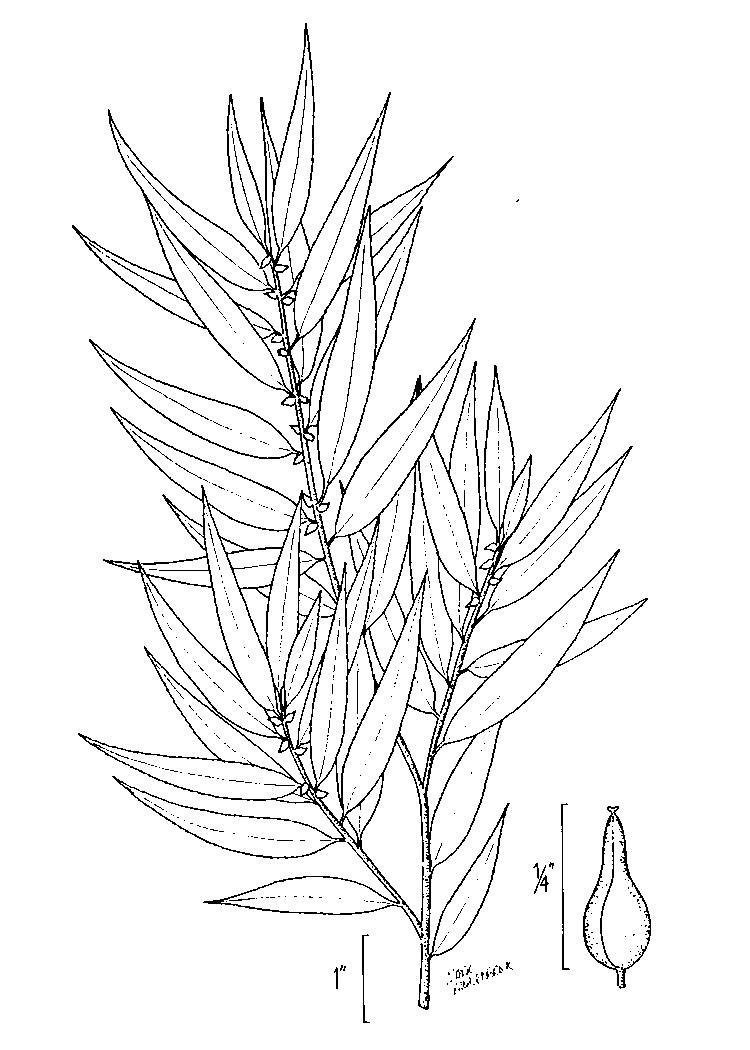
*Salix caroliniana* (from USDA-NRCS 2012).

**Figure 117a.**
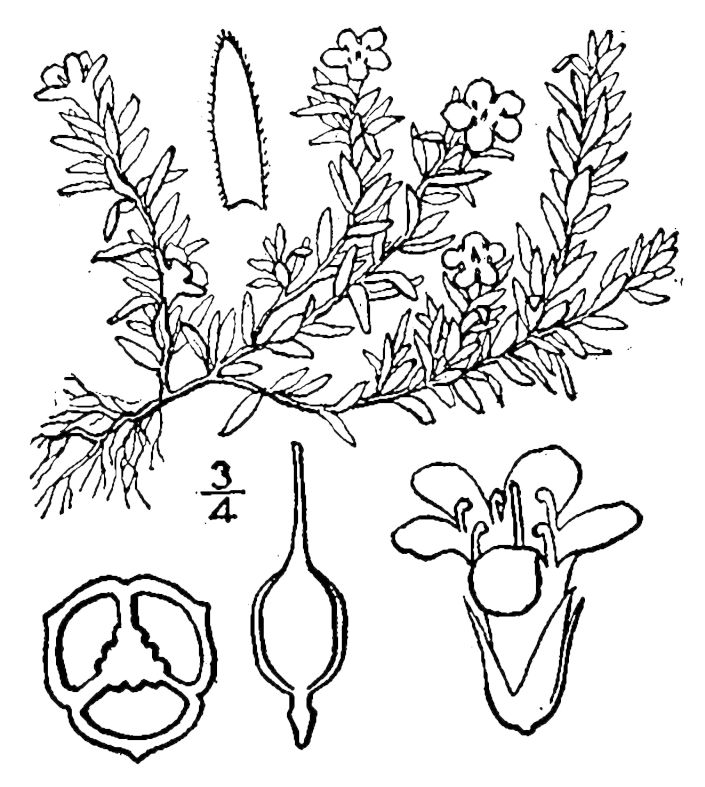
From Britton and Brown (1913).

**Figure 117b.**
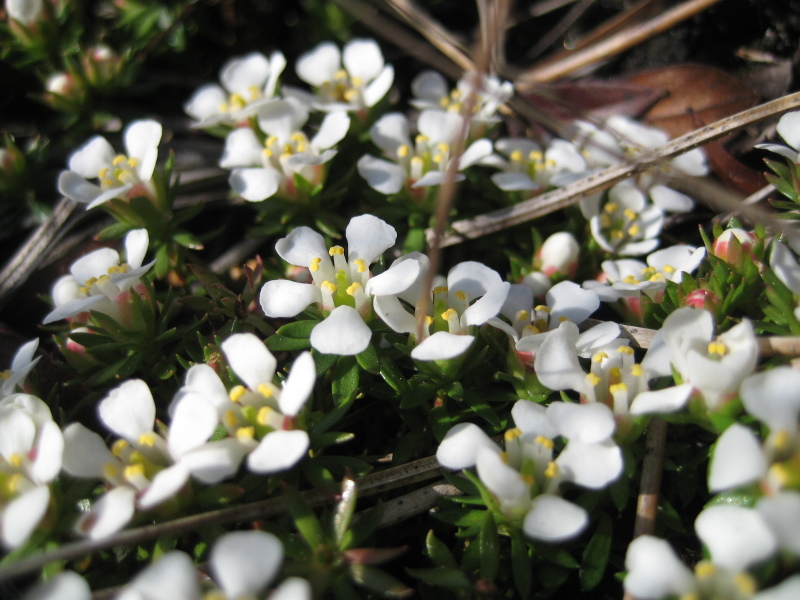
Photo by R. Thornhill.

**Figure 118a.**
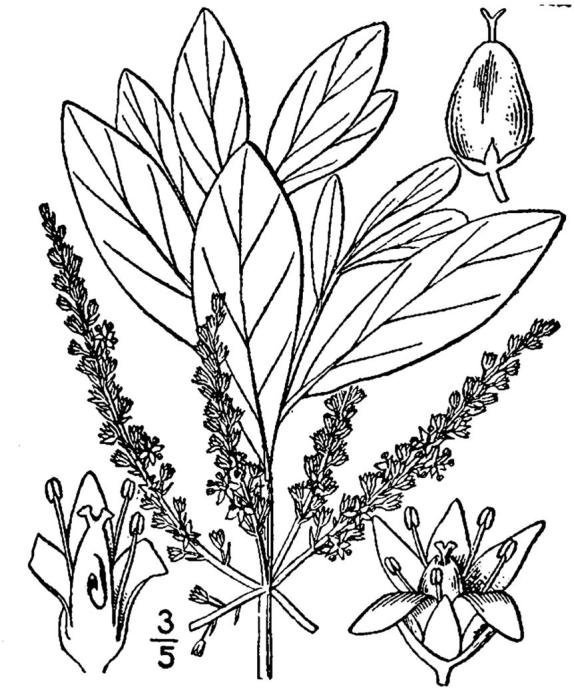
From Britton and Brown (1913).

**Figure 118b.**
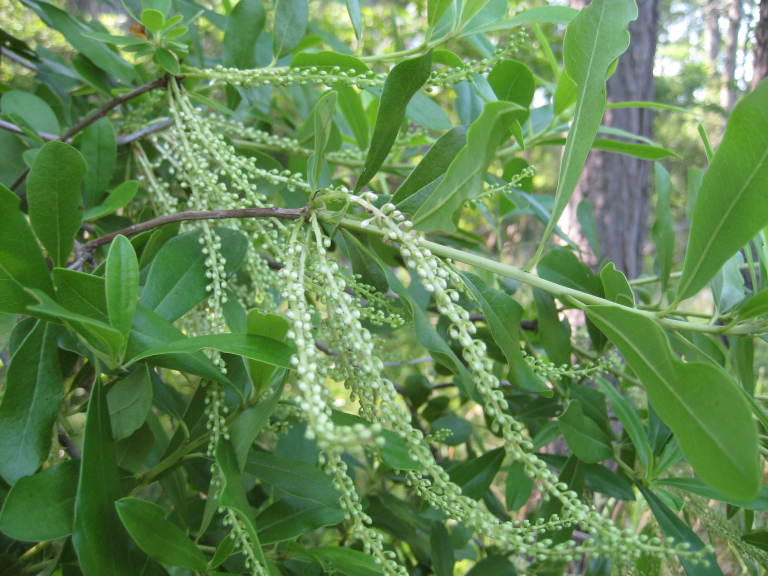
Photo by R. Thornhill.

**Figure 119a.**
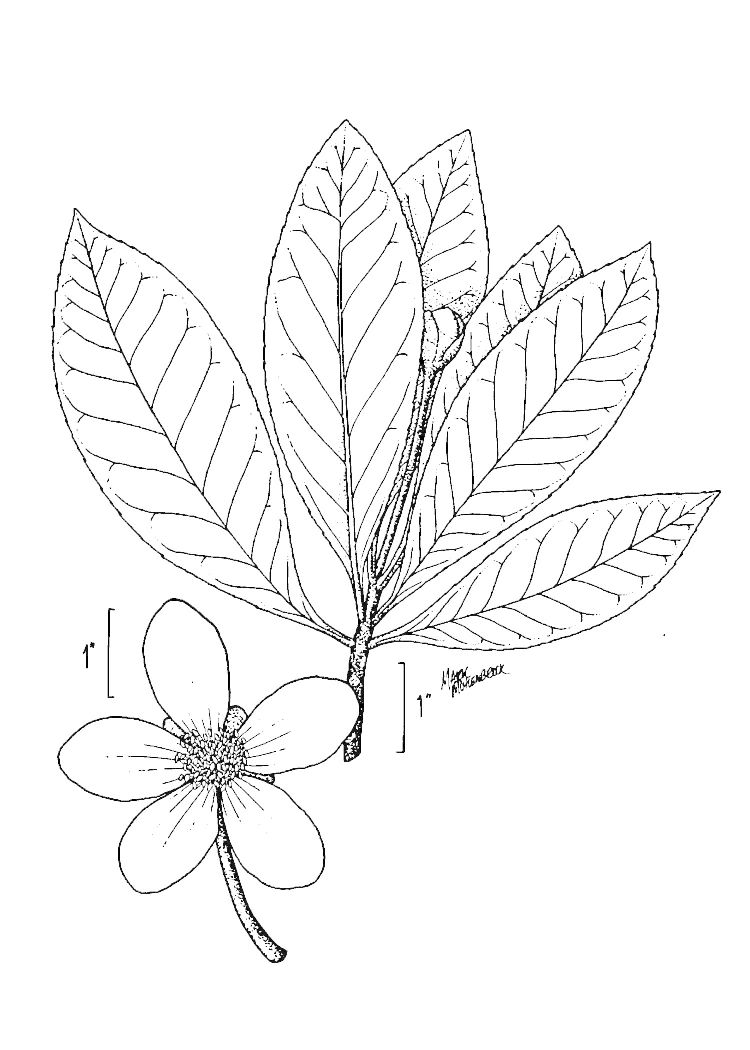
From USDA-NRCS (2012).

**Figure 119b.**
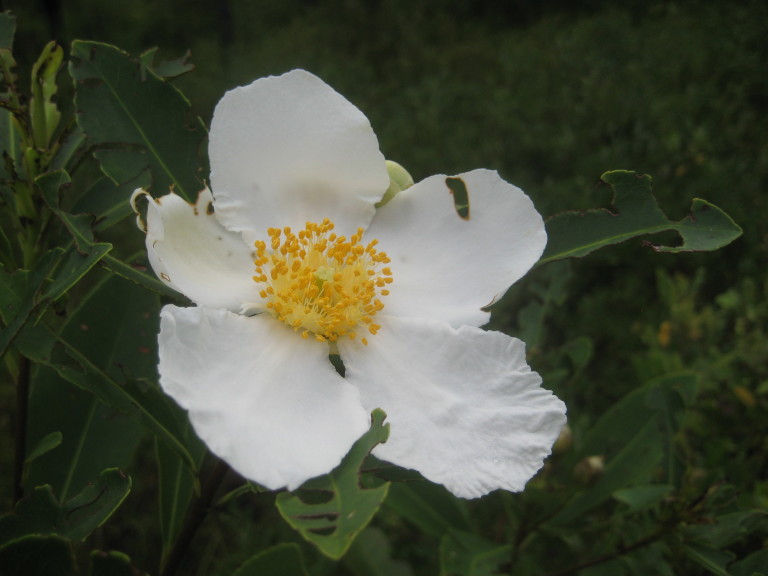
Photo by R. Thornhill.

**Figure 120.**
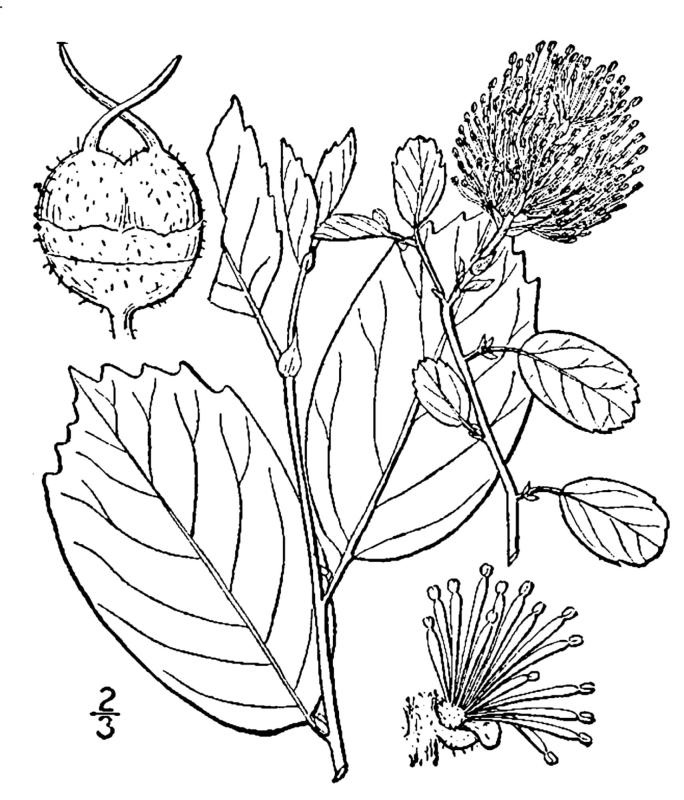
*Fothergilla gardenii* (from Britton and Brown 1913).

**Figure 121a.**
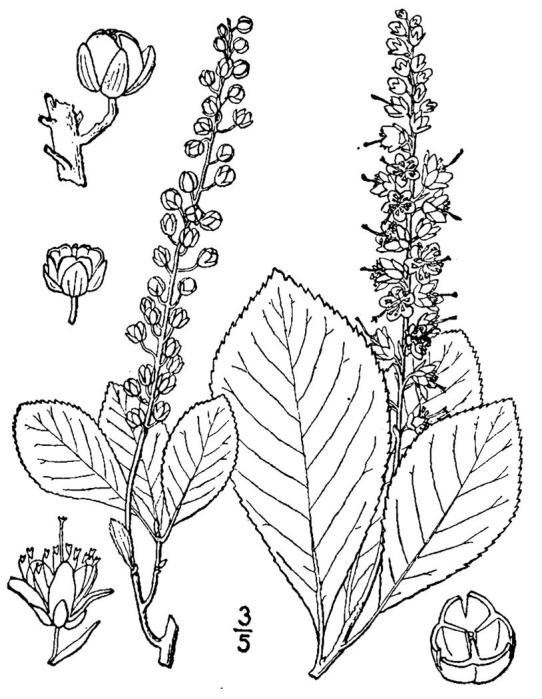
From Britton and Brown (1913).

**Figure 121b.**
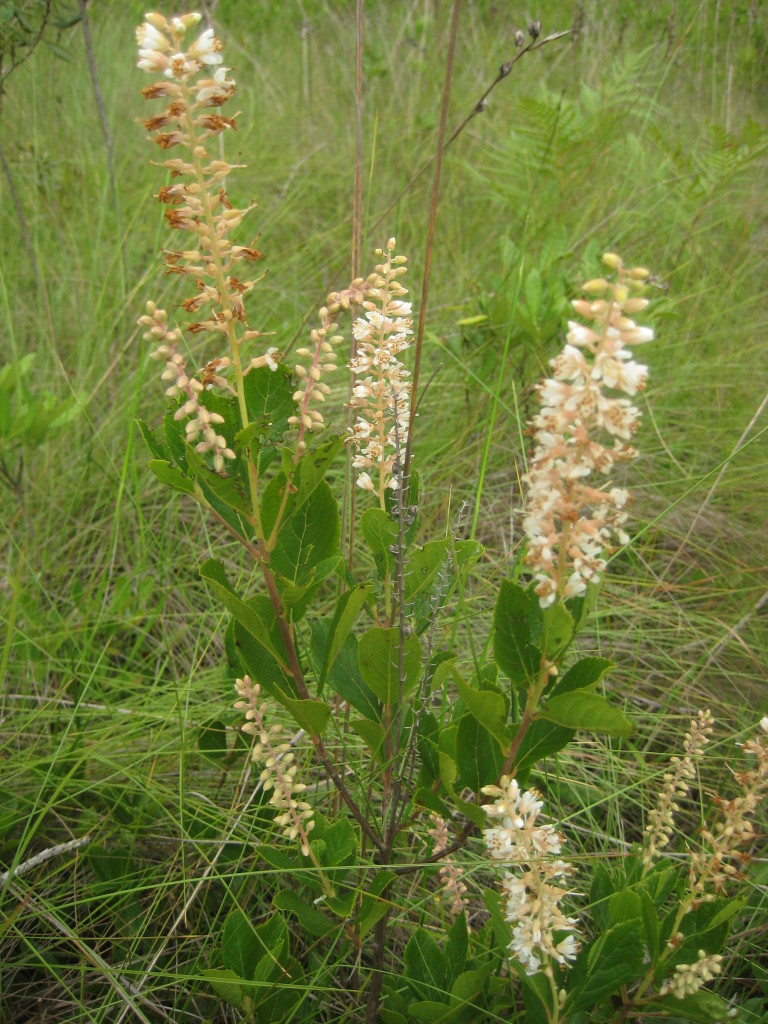
Photo by R. Thornhill

**Figure 122a.**
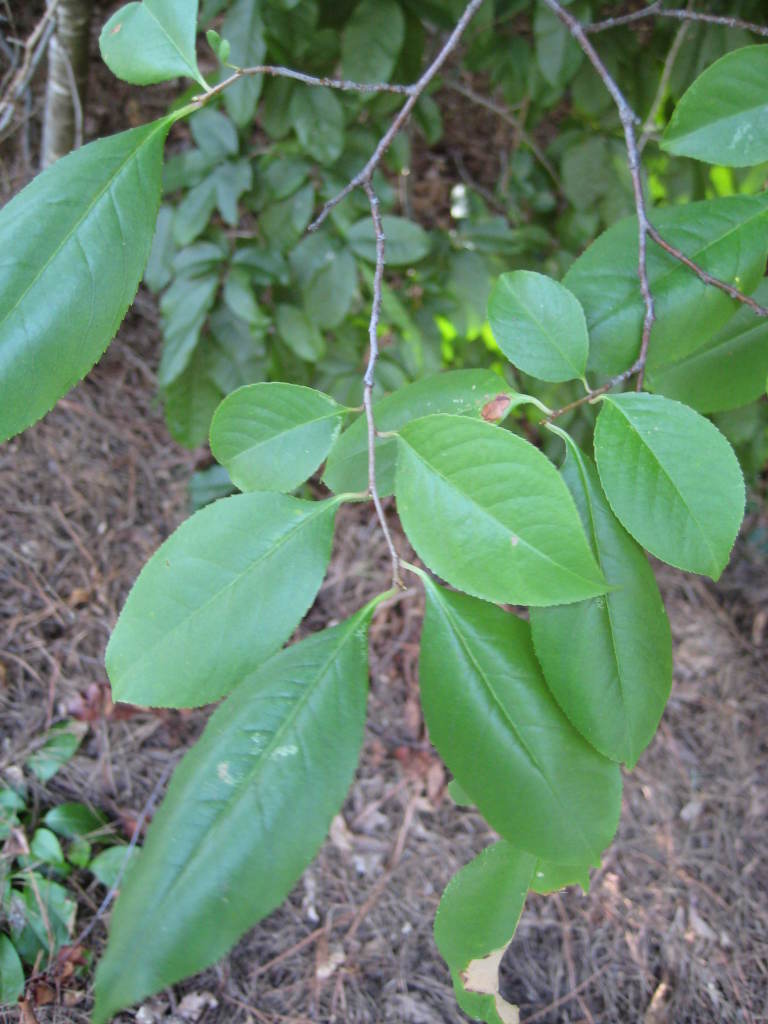
Photo by R. Thornhill.

**Figure 122b.**
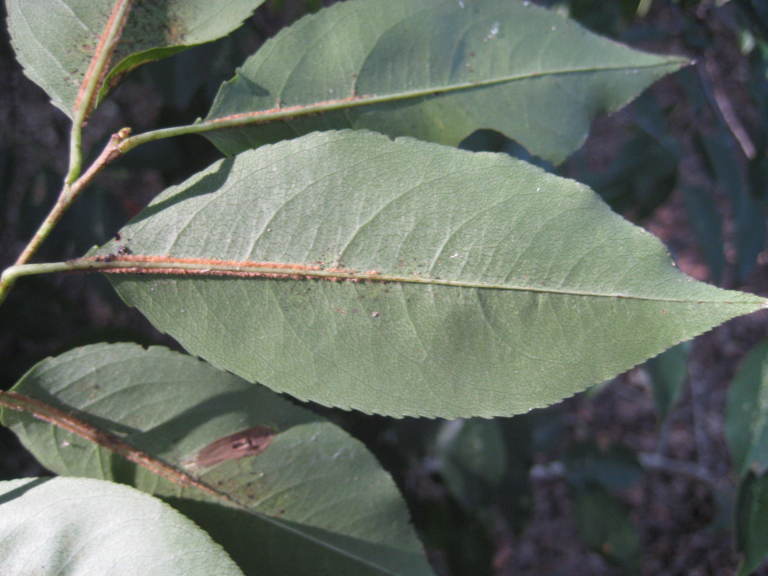
Midvein of lower leaf surfaces is sometimes covered with rusty-colored, felt-like hairs (photo by R. Thornhill).

**Figure 122c.**
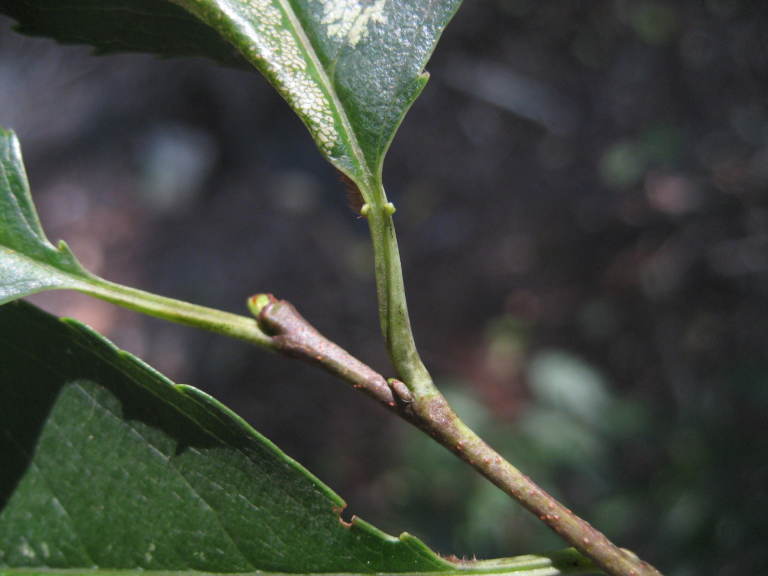
A pair of petiolar glands is usually present near junction with leaf blade (photo by R. Thornhill).

**Figure 122d.**
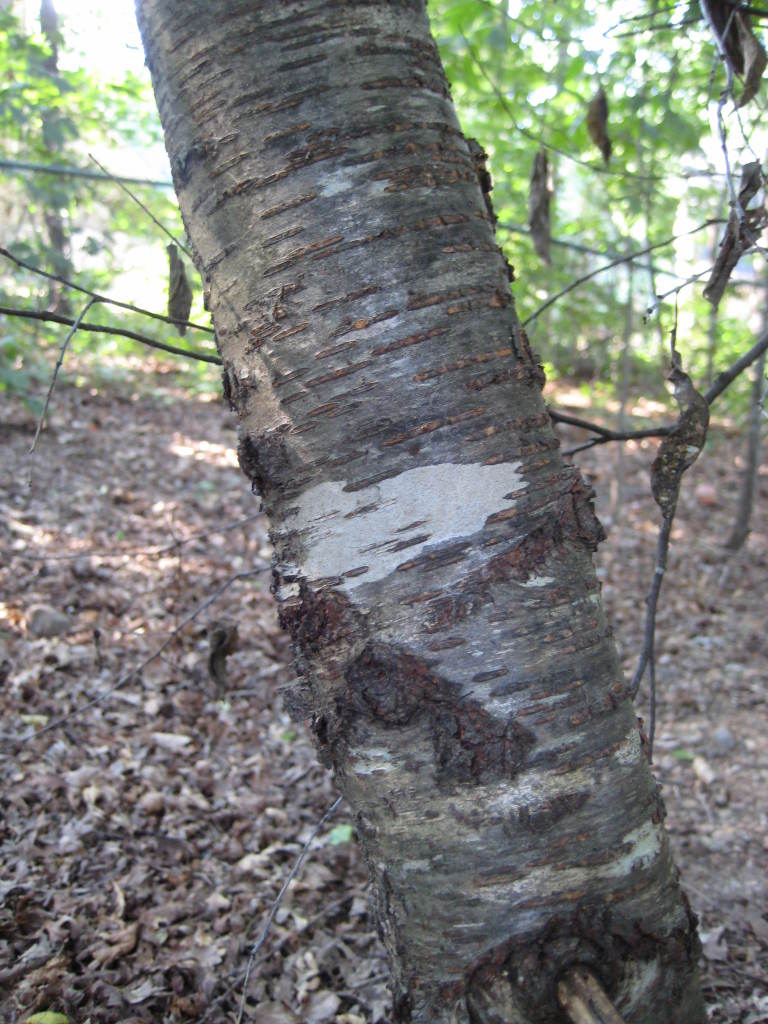
Bark has distinctive horizontal banding of lenticels (photo by R. Thornhill).

**Figure 123a.**
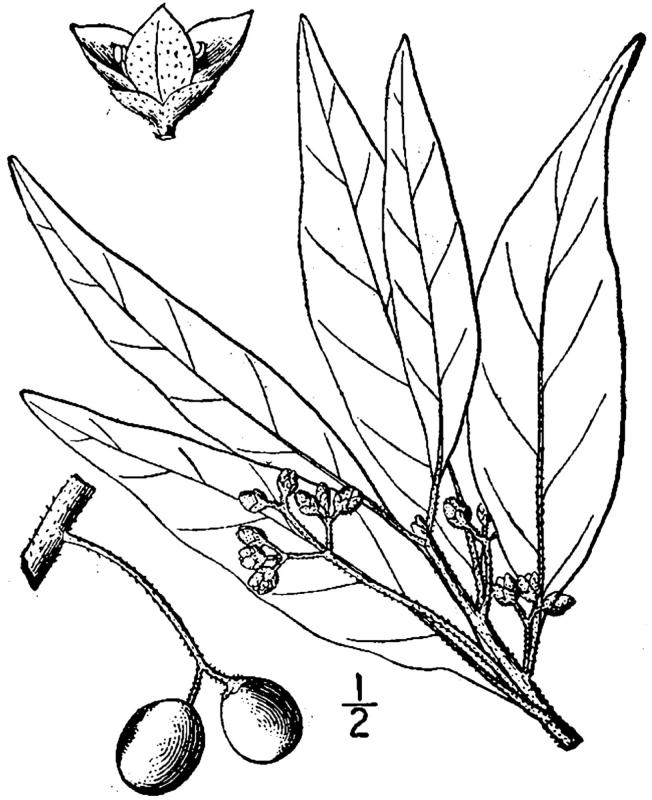
From Britton and Brown (1913).

**Figure 123b.**
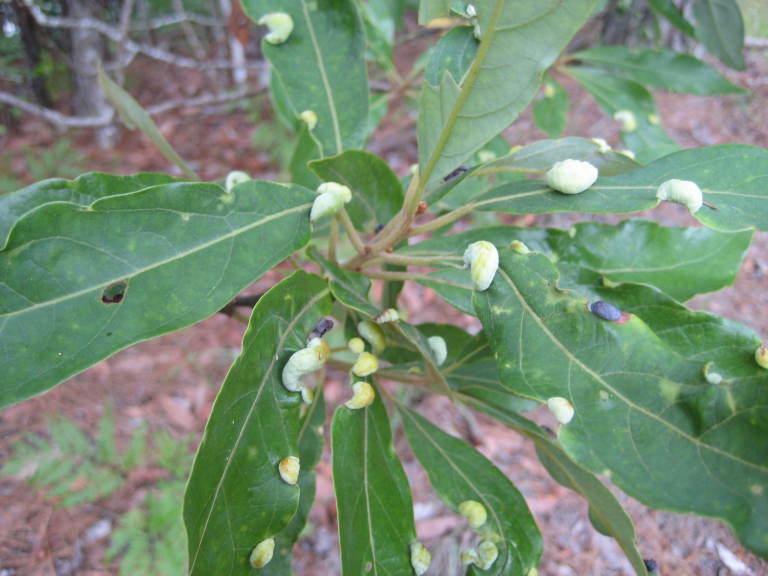
Note the characteristic galls along the leaf margins (photo by R. Thornhill).

**Figure 124.**
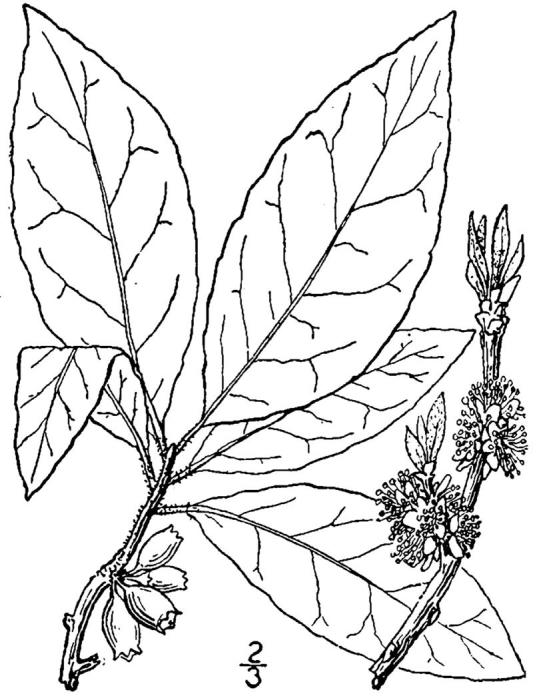
*Symplocos tinctoria* (from Britton and Brown 1913).

**Figure 125.**
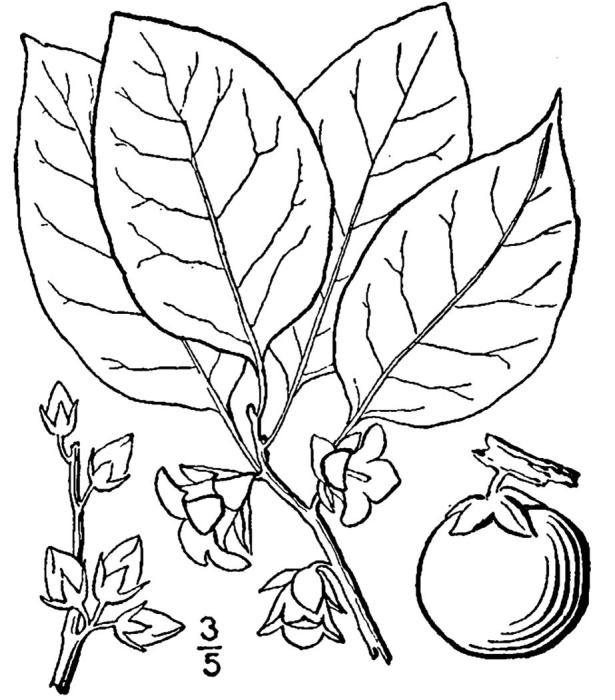
*Diospyros virginiana* (from Britton and Brown 1913).

**Figure 126a.**
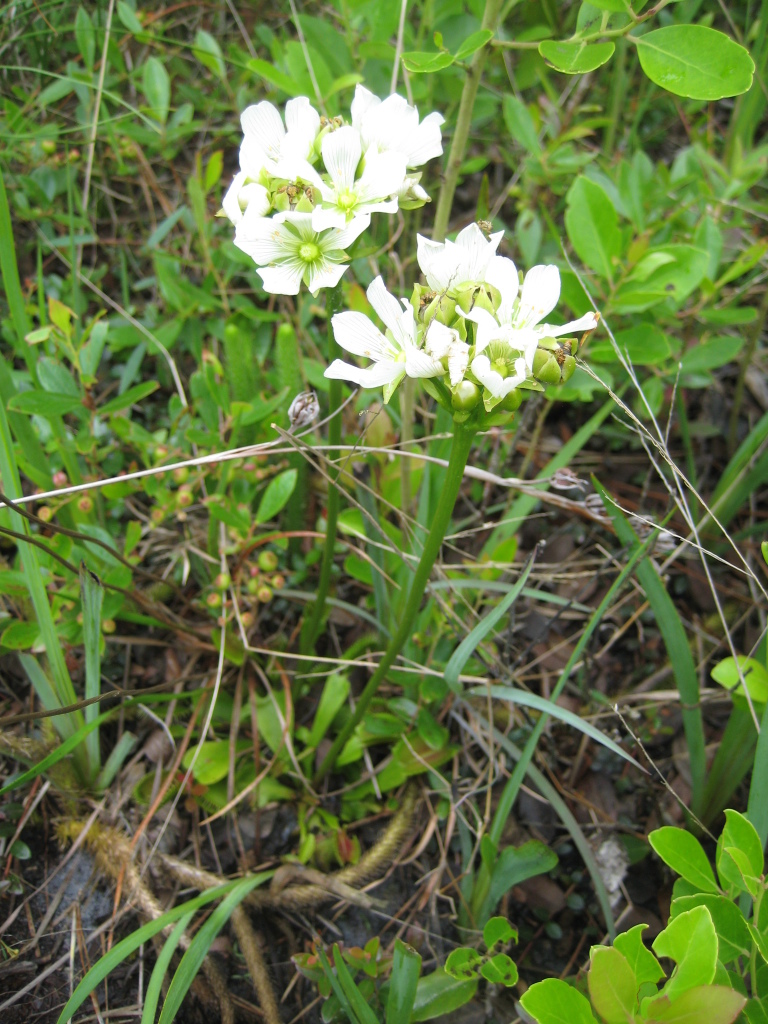
Photo by R. Thornhill.

**Figure 126b.**
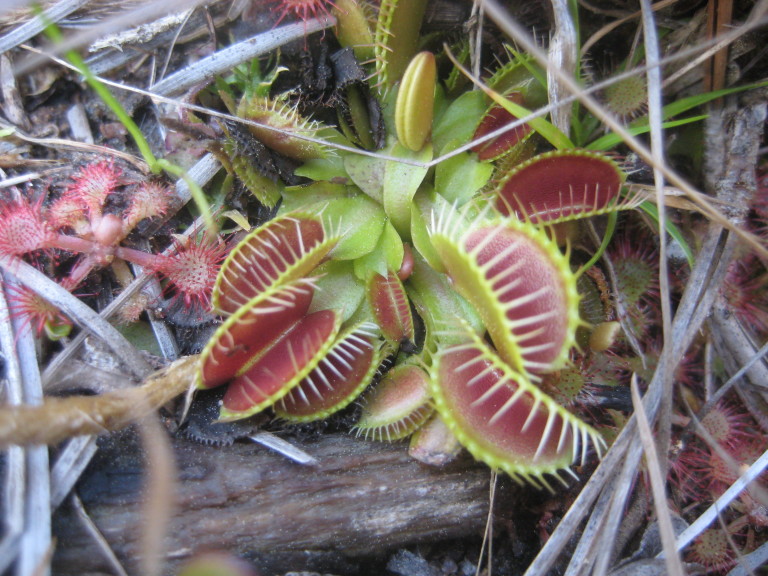
Photo by R. Thornhill.

**Figure 127a.**
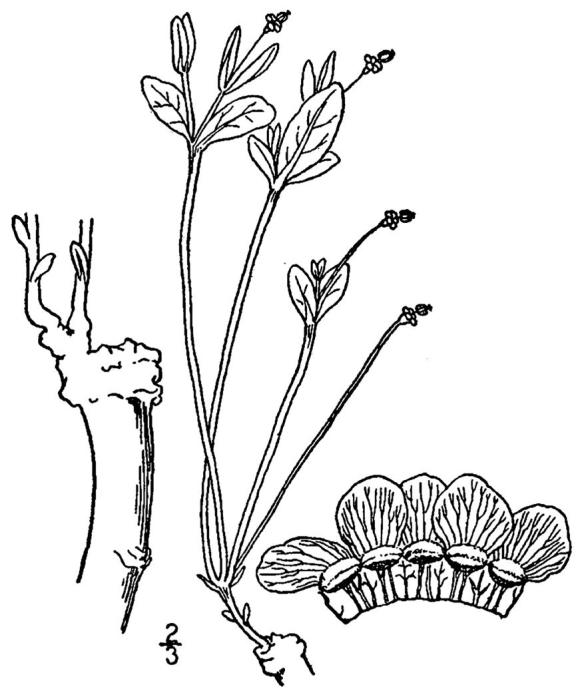
From Britton and Brown (1913).

**Figure 127b.**
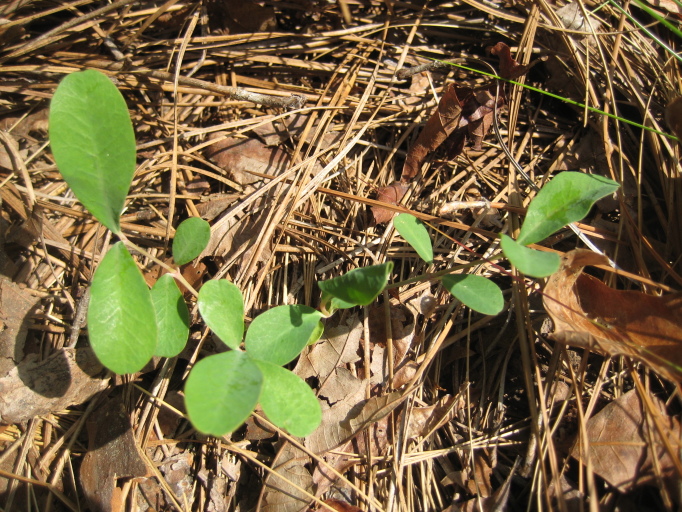
Note that leaf shape in this species varies from elliptic (as in the above photo) to linear (photo by R. Thornhill).

**Figure 128.**
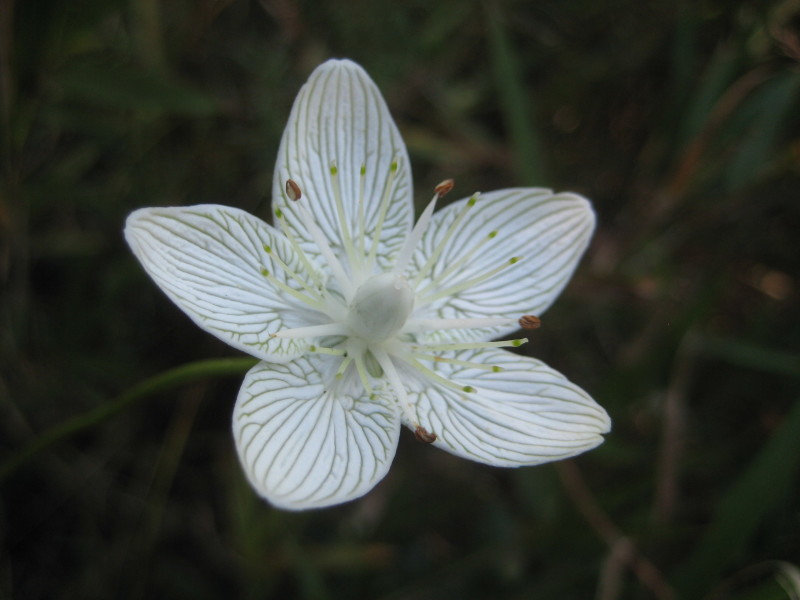
*Parnassia caroliniana* (photo by R. Thornhill).

**Figure 129a.**
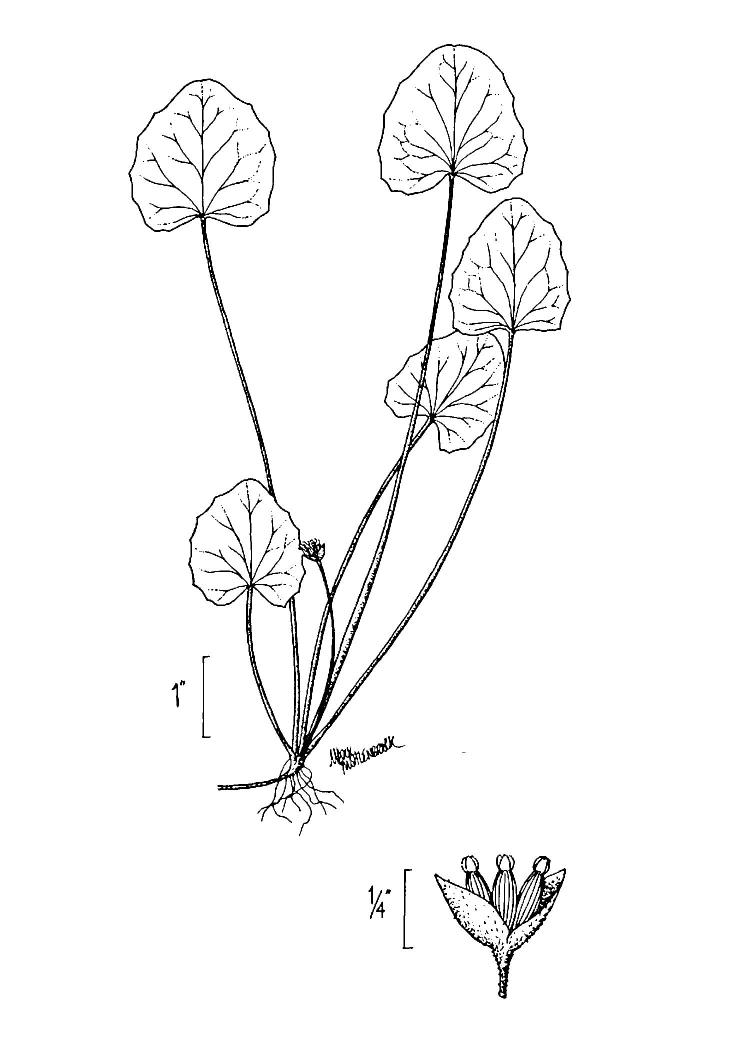
From USDA-NRCS (2012).

**Figure 129b.**
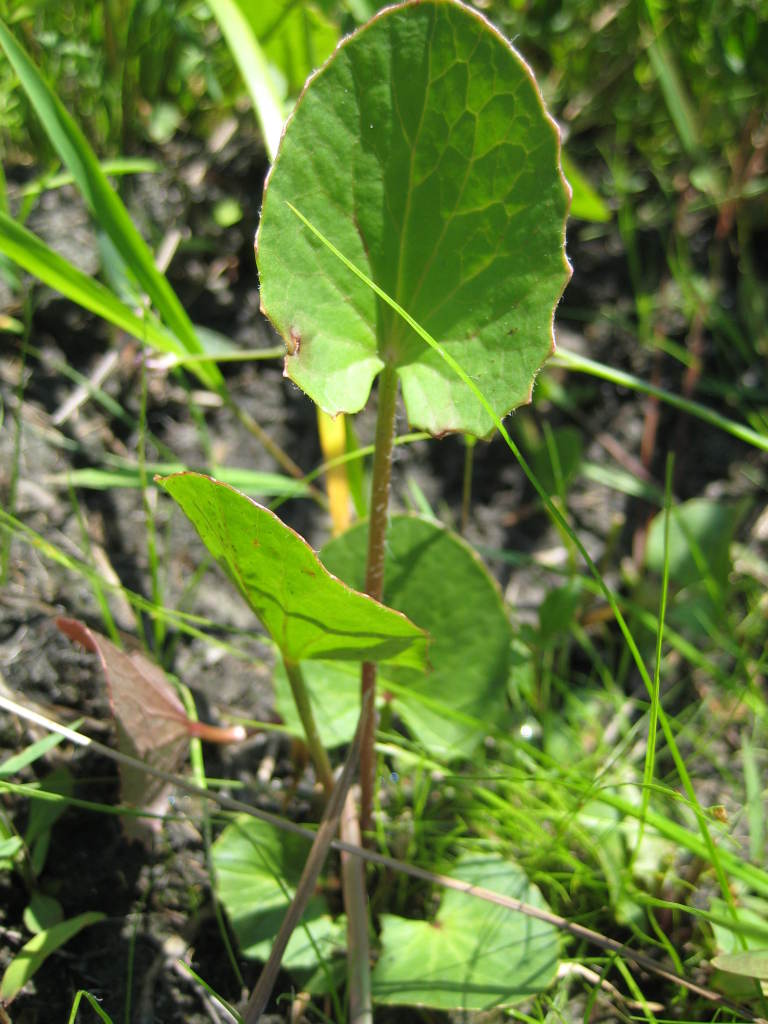
Photo by R. Thornhill.

**Figure 130a.**
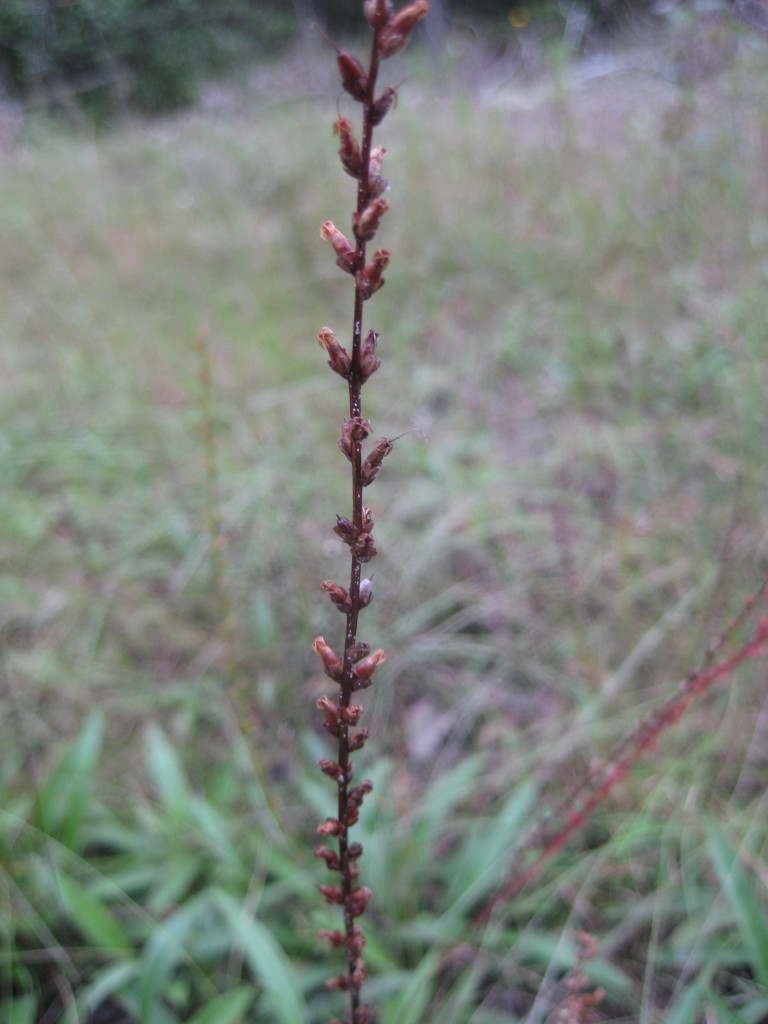
Inflorescence (photo by R. Thornhill).

**Figure 130b.**
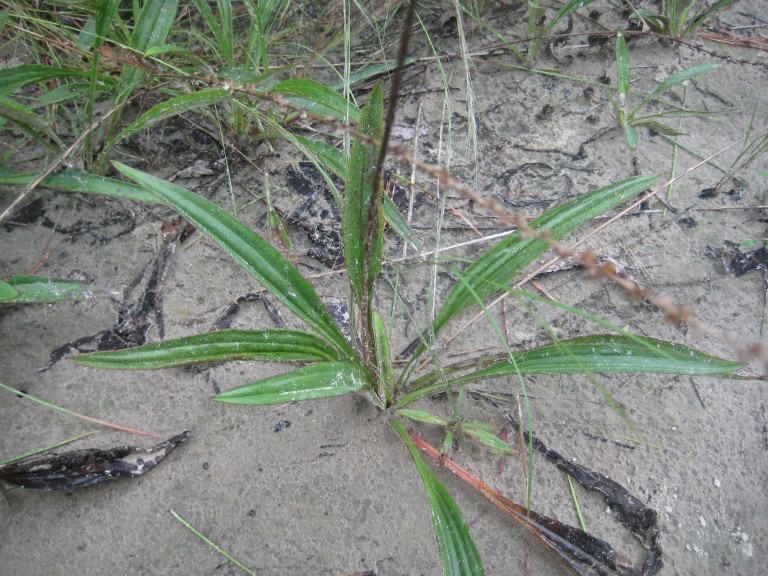
Basal leaves (photo by R. Thornhill).

**Figure 131.**
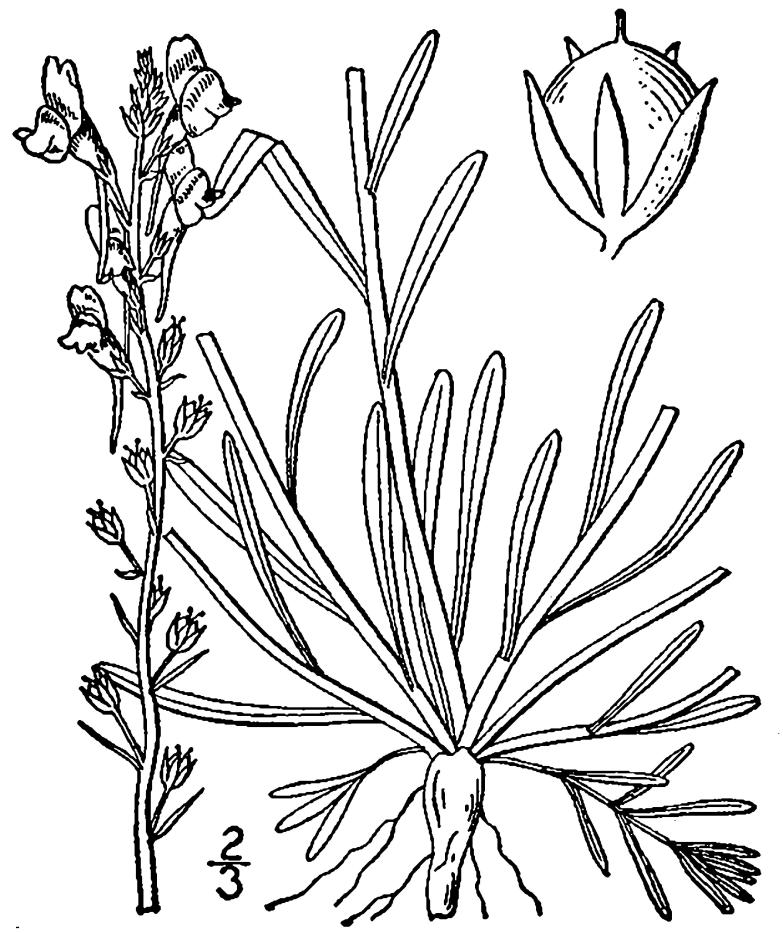
*Nuttallanthus canadensis* (from Britton and Brown 1913).

**Figure 132.**
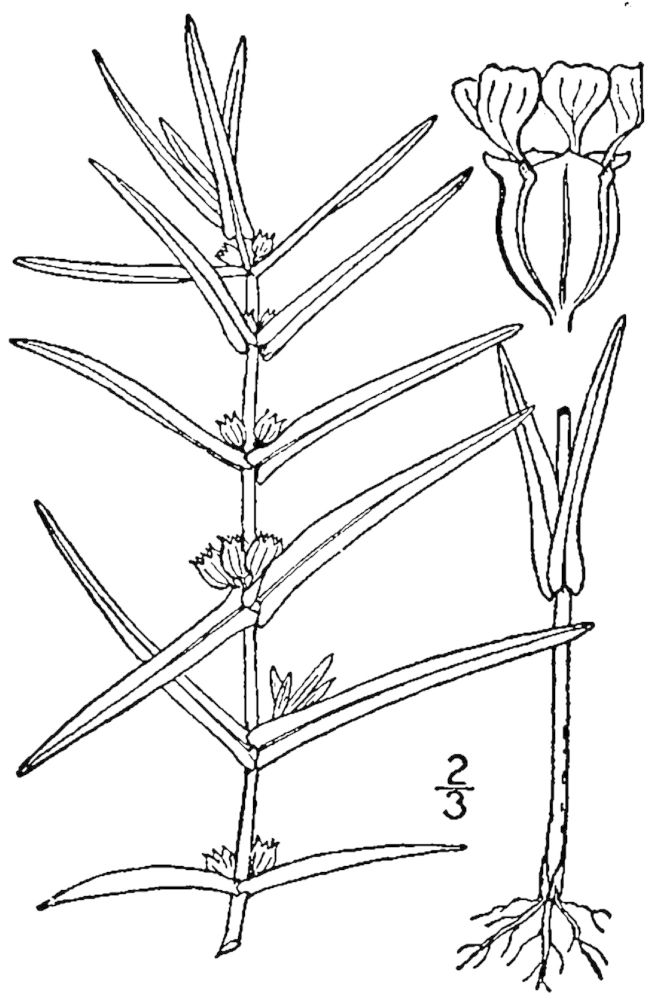
*Ammannia coccinea* (from Britton and Brown 1913).

**Figure 133a.**
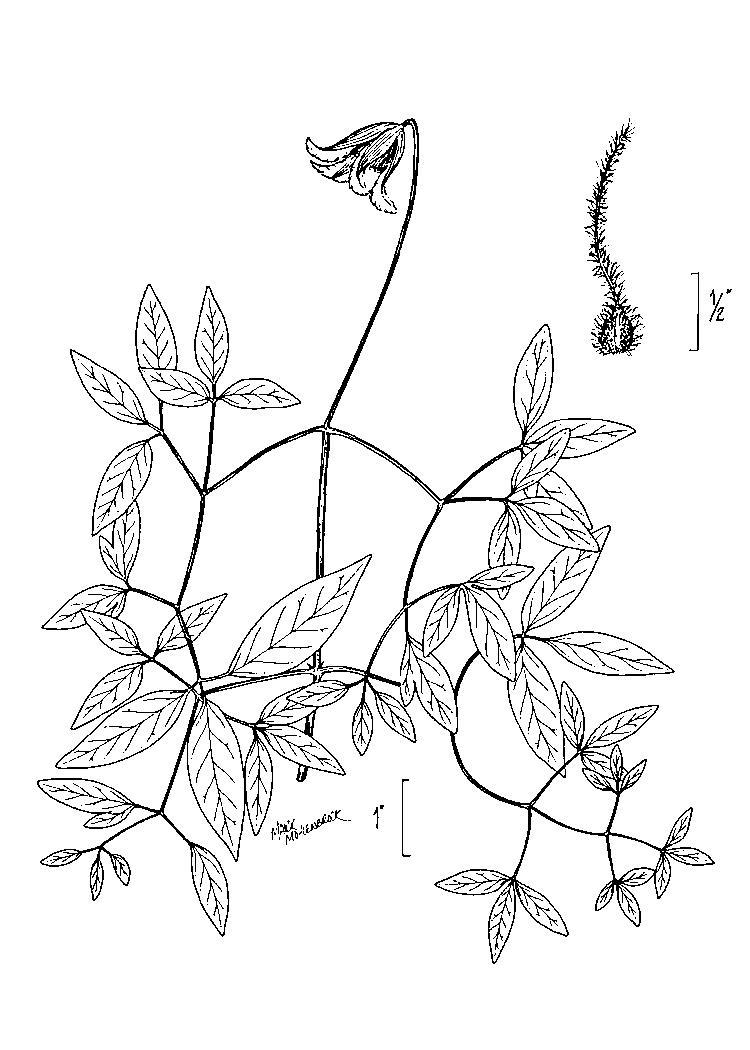
From USDA-NRCS (2012).

**Figure 133b.**
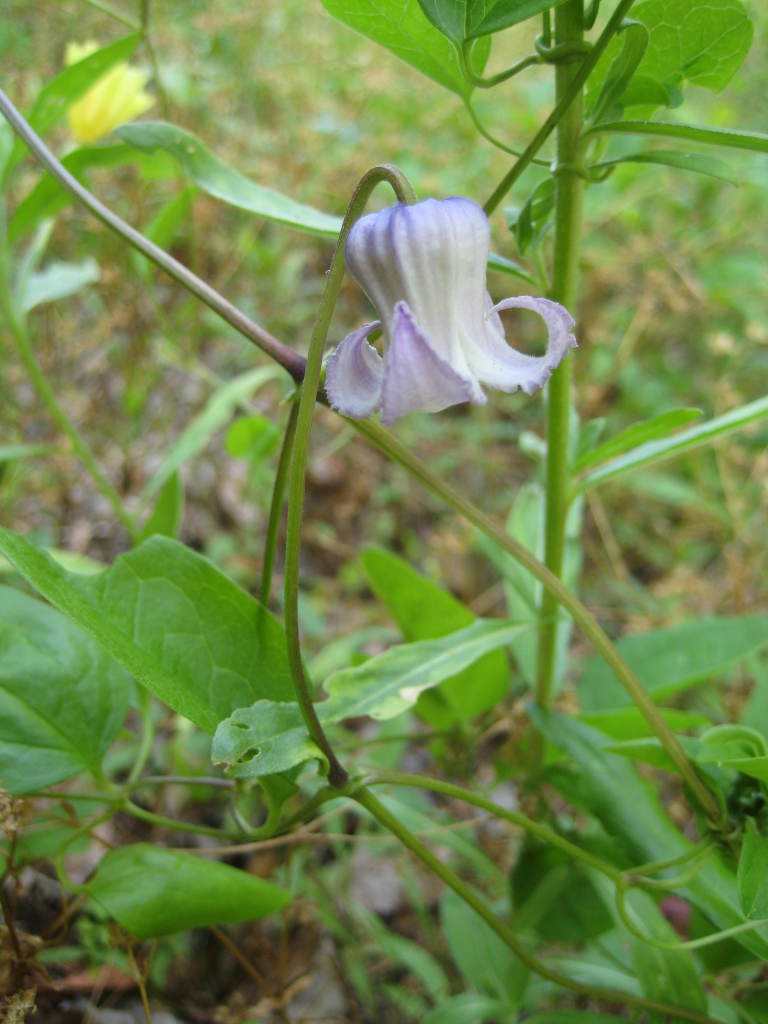
Photo by R. Thornhill.

**Figure 134.**
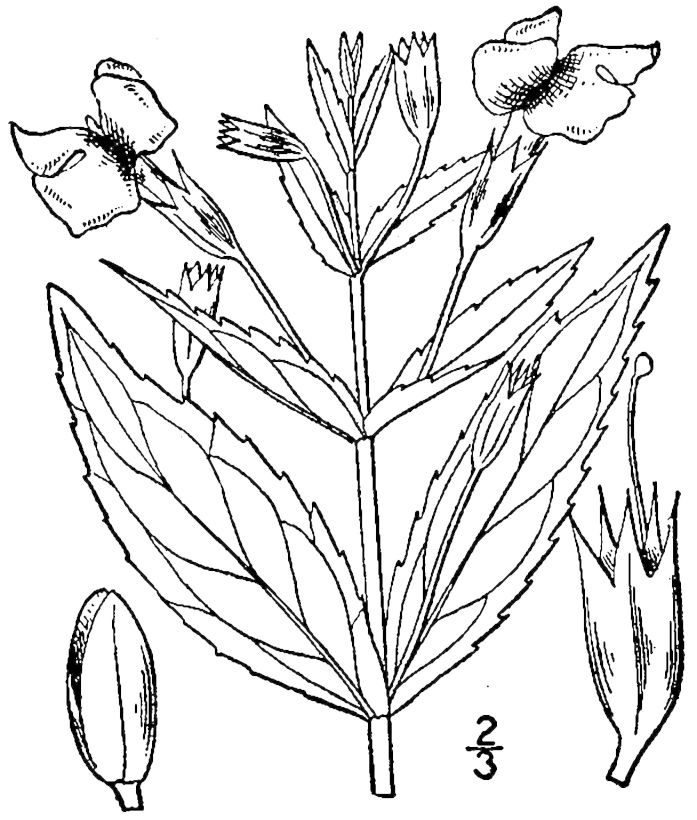
*Mimulus ringens* (from Britton and Brown 1913).

**Figure 135.**
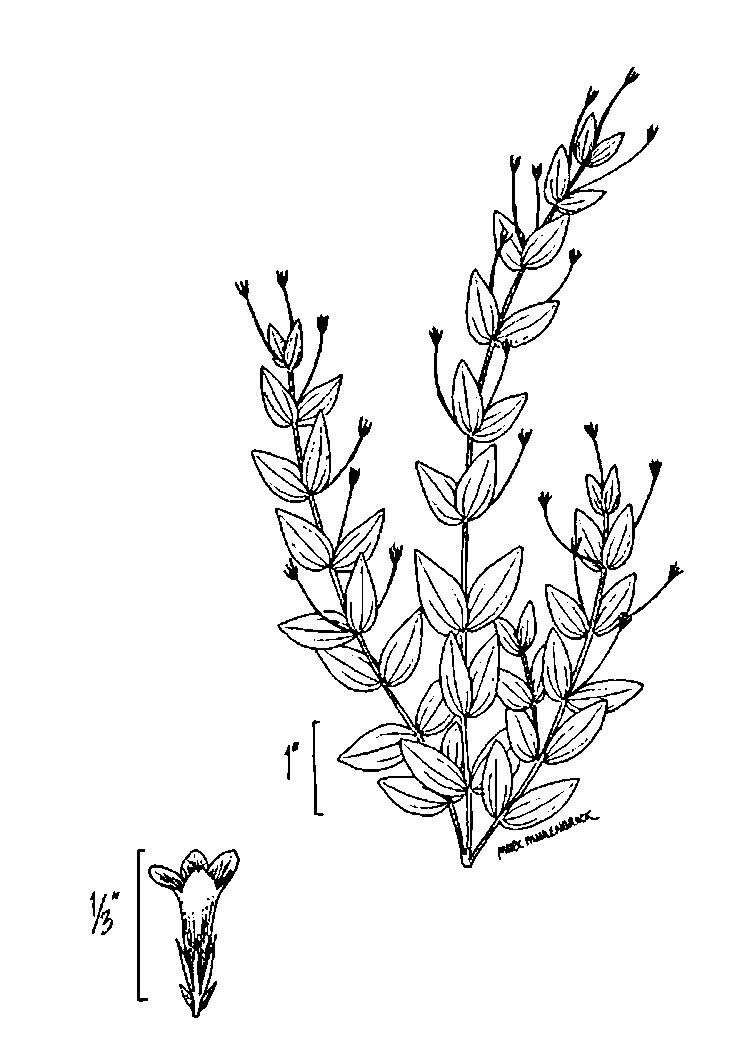
*Lindernia dubia* (from USDA-NRCS 2012).

**Figure 136.**
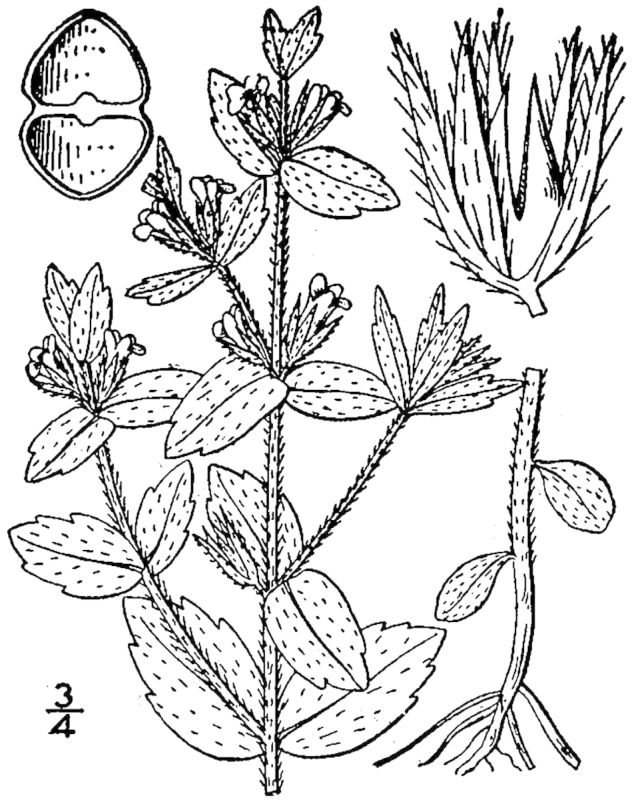
*Sophronanthe pilosa* (from Britton and Brown 1913).

**Figure 137a.**
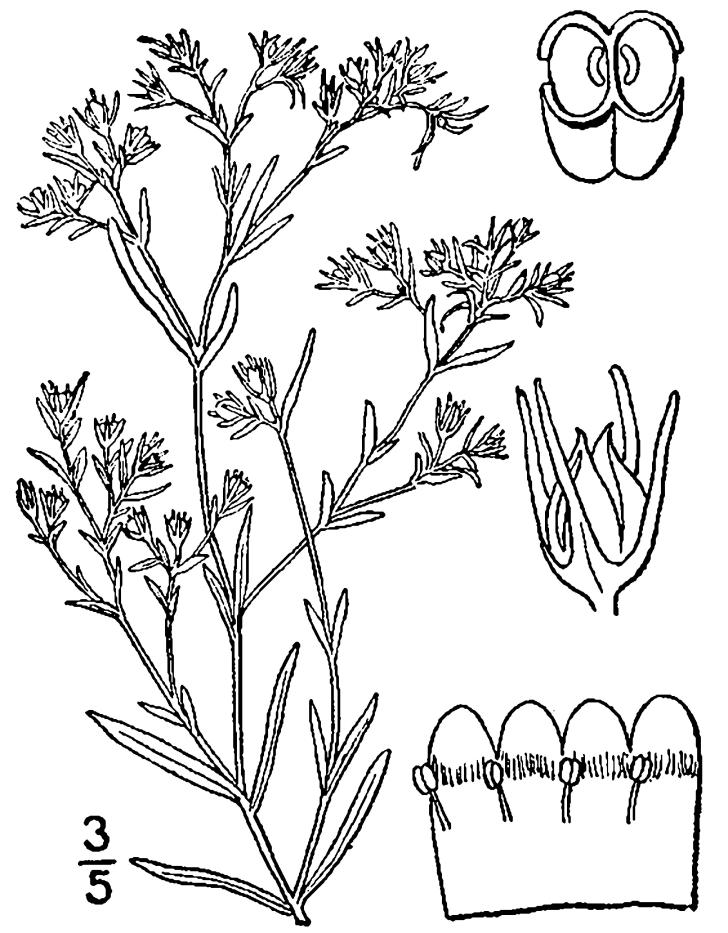
From Britton and Brown (1913).

**Figure 137b.**
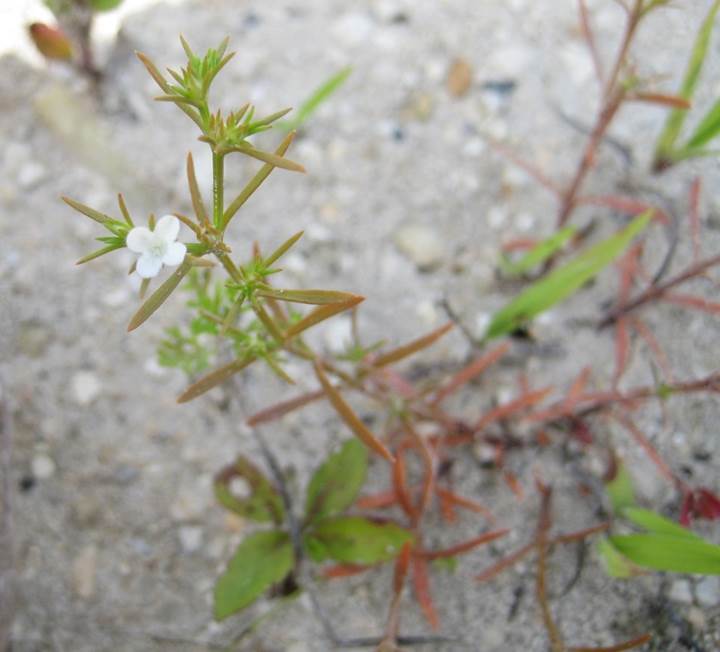
Photo by R. Thornhill.

**Figure 138.**
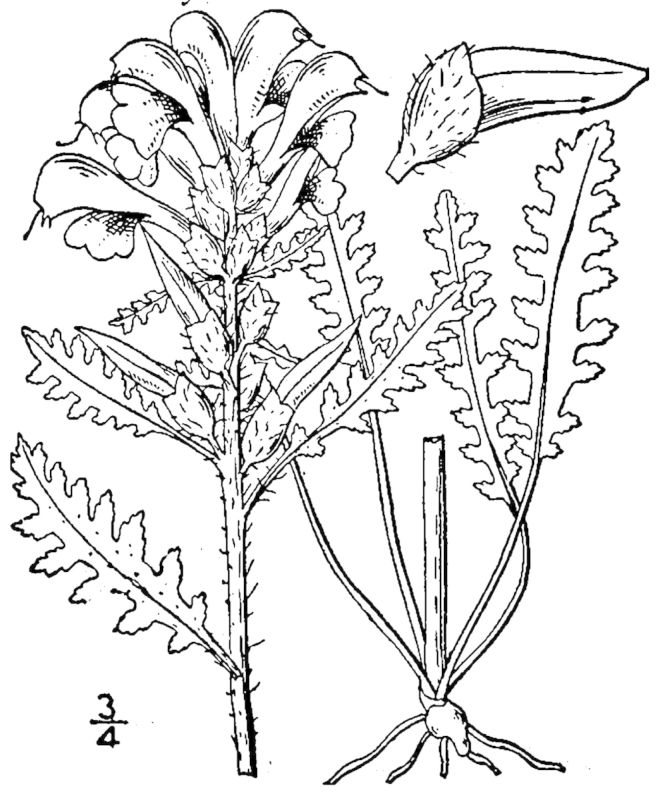
*Pedicularis canadensis* (from Britton and Brown 1913).

**Figure 139a.**
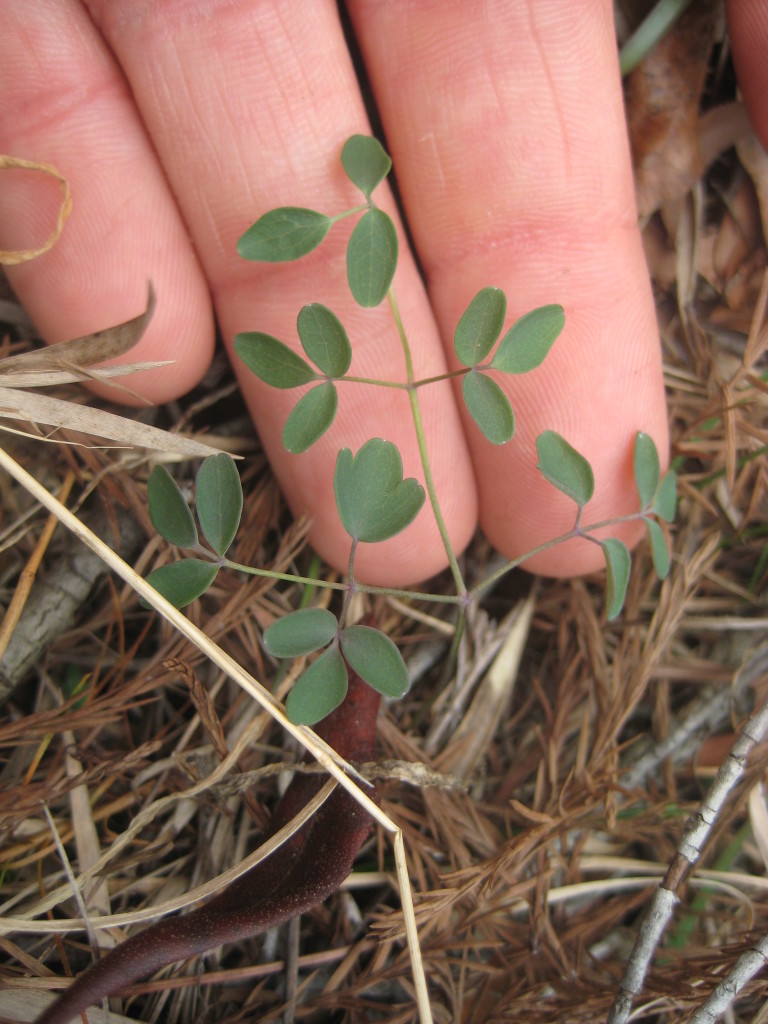
Basal leaflets are more broadly elliptic than cauline leaflets (photo by R. Thornhill).

**Figure 139b.**
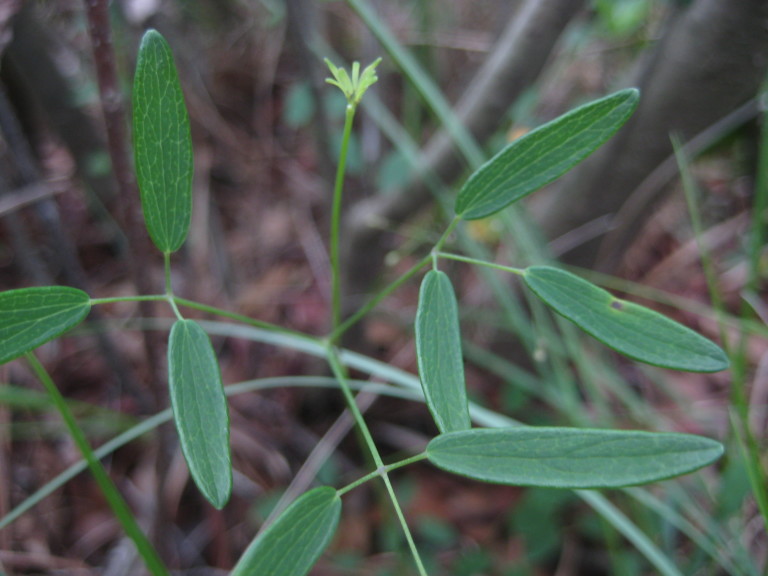
Distinctly narrow cauline leaflets (photo by R. Thornhill).

**Figure 139c.**
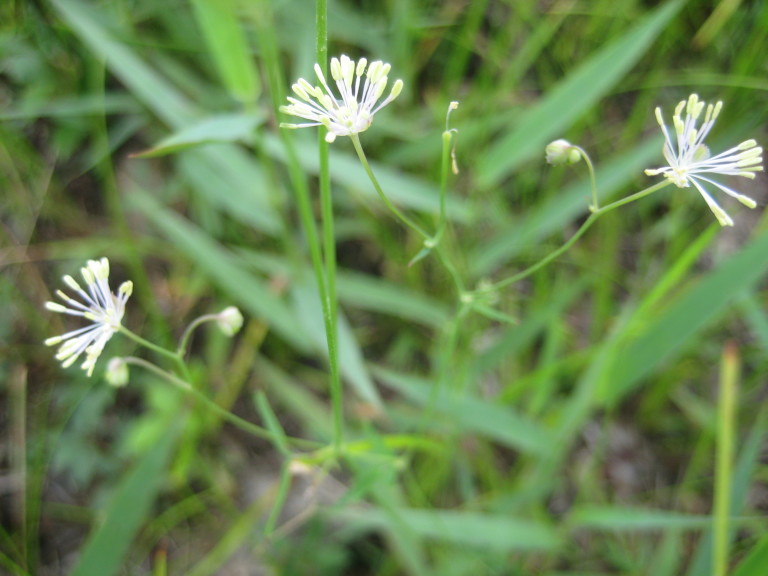
Inflorescence (male flowers) (photo by R. Thornhill).

**Figure 139d.**
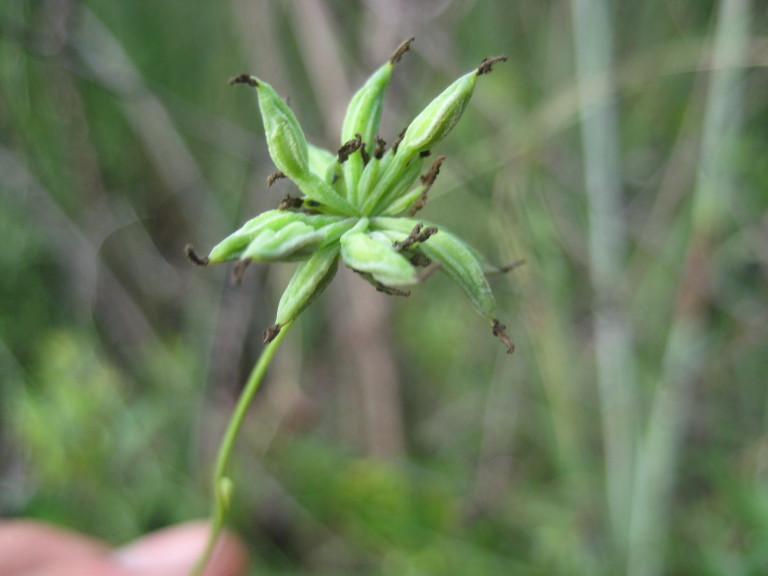
Fruits (photo by R. Thornhill).

**Figure 140.**
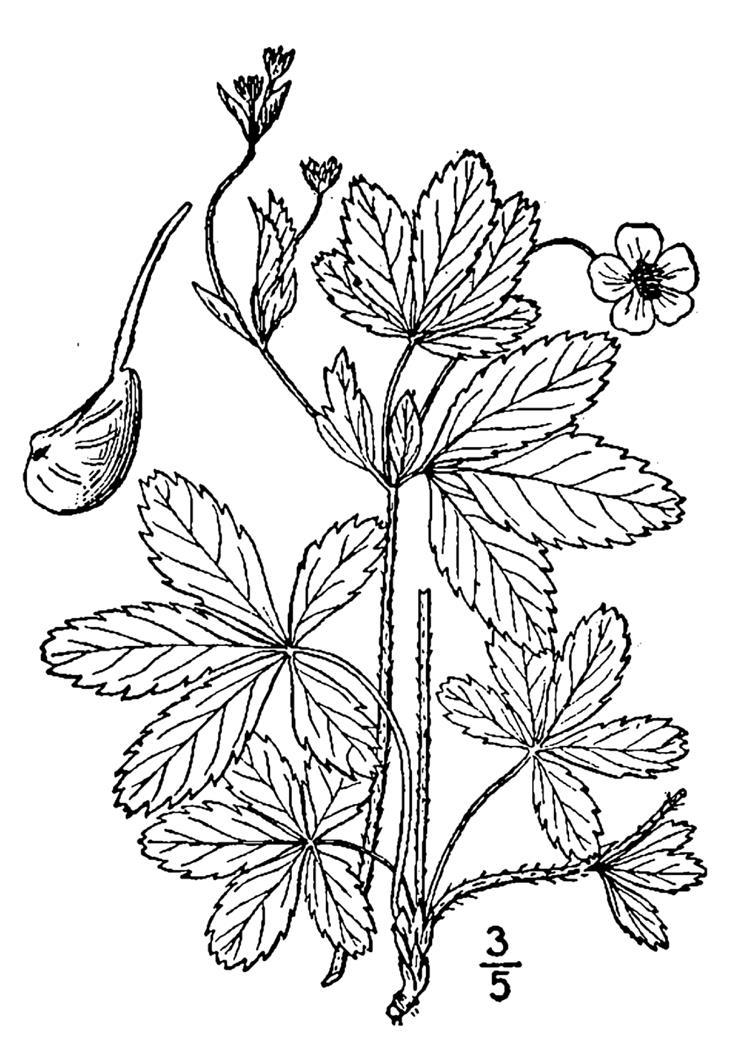
*Potentilla simplex* (from Britton and Brown 1913).

**Figure 141a.**
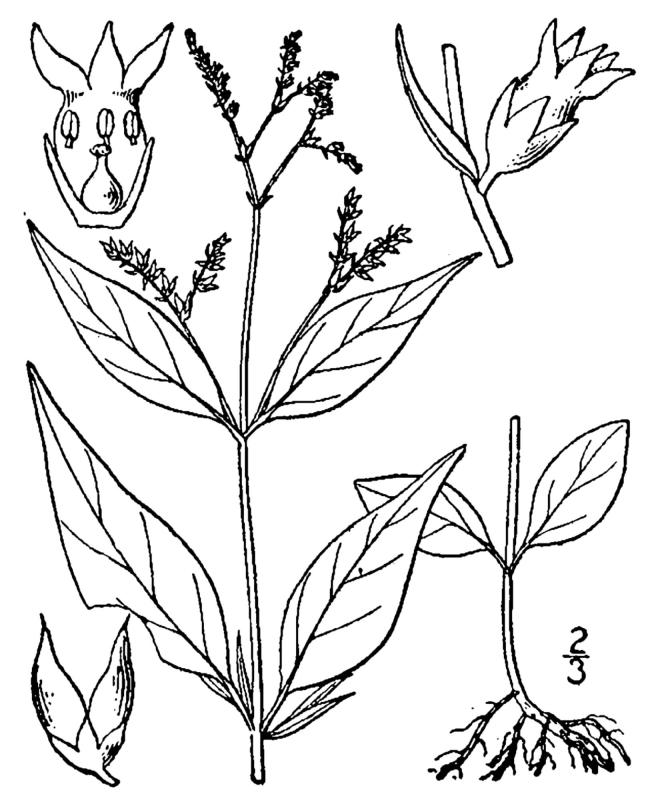
*Mitreola petiolata* (from Britton and Brown 1913).

**Figure 141b.**
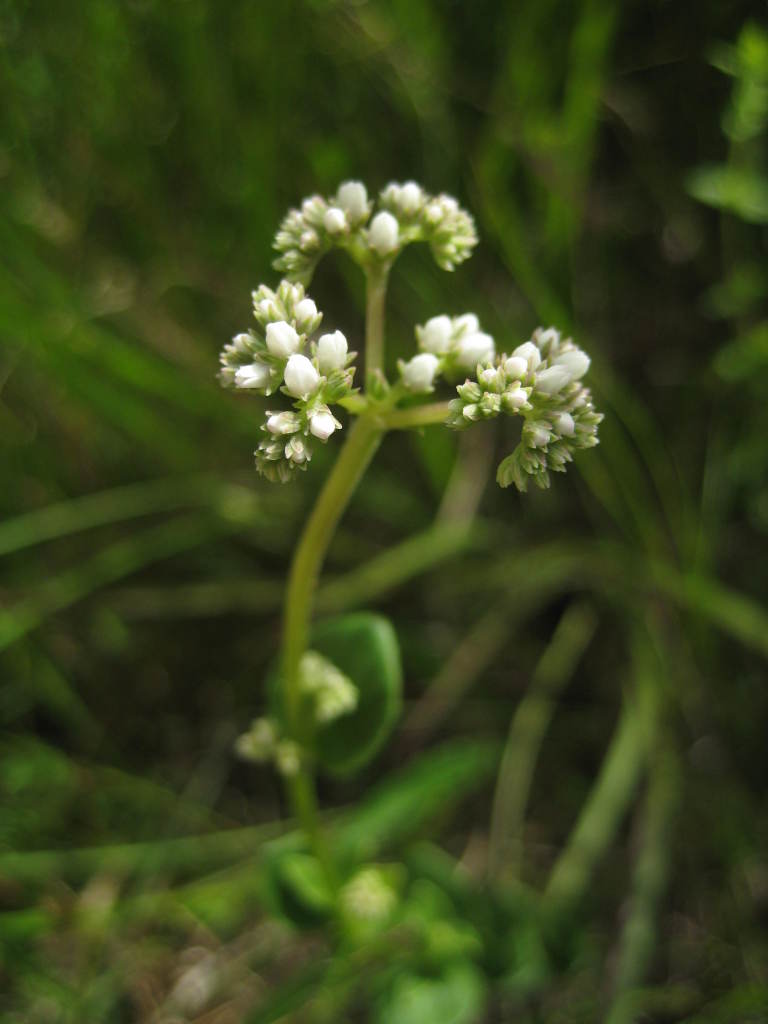
*Mitreola sessilifolia* (photo by R. Thornhill).

**Figure 142a.**
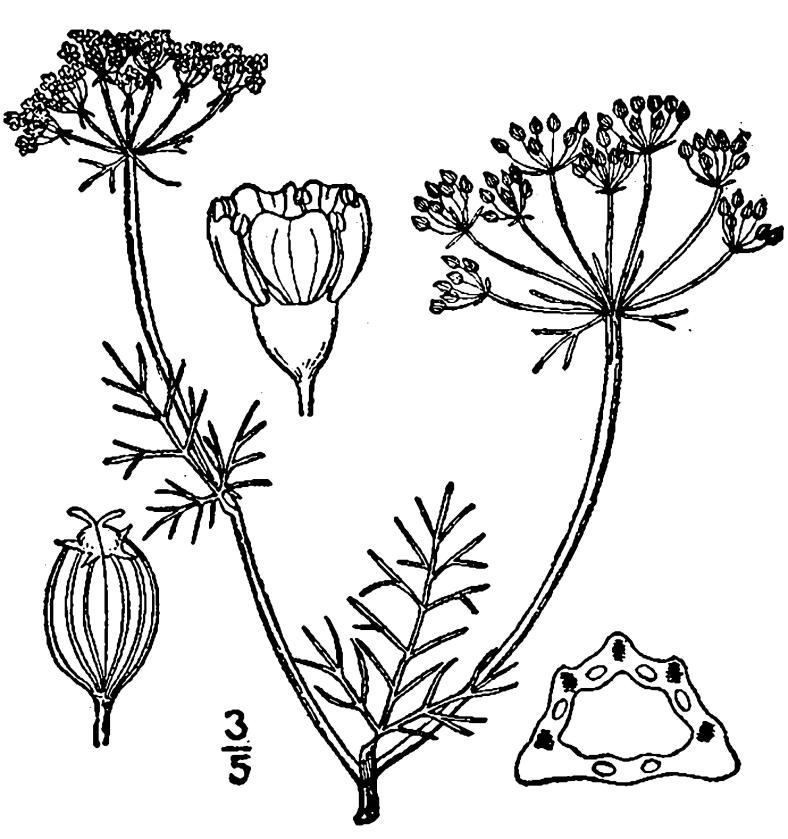
From Britton and Brown (1913).

**Figure 142b.**
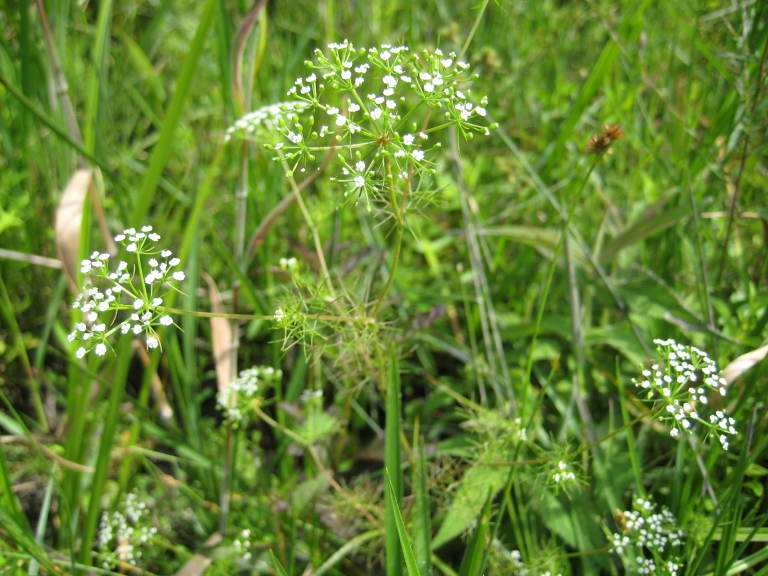
Photo by R. Thornhill.

**Figure 143a.**
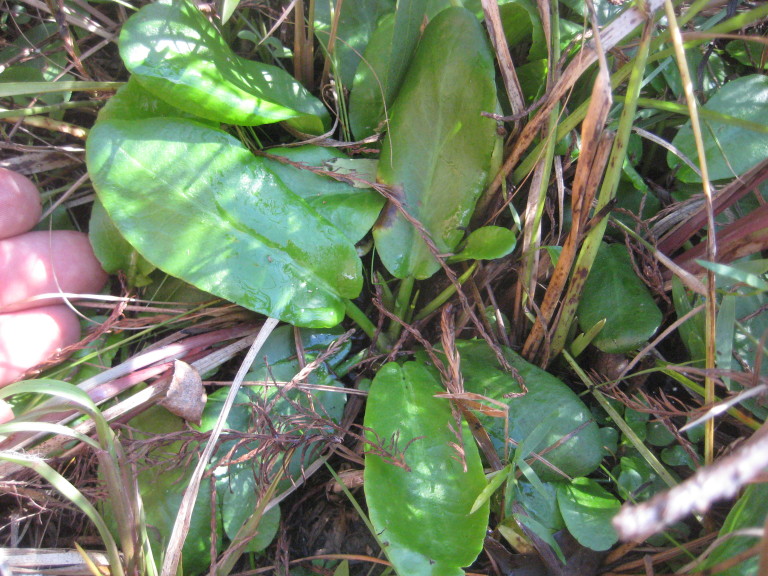
*Eryngium integrifolium*: basal leaves (photo by R. Thornhill).

**Figure 143b.**
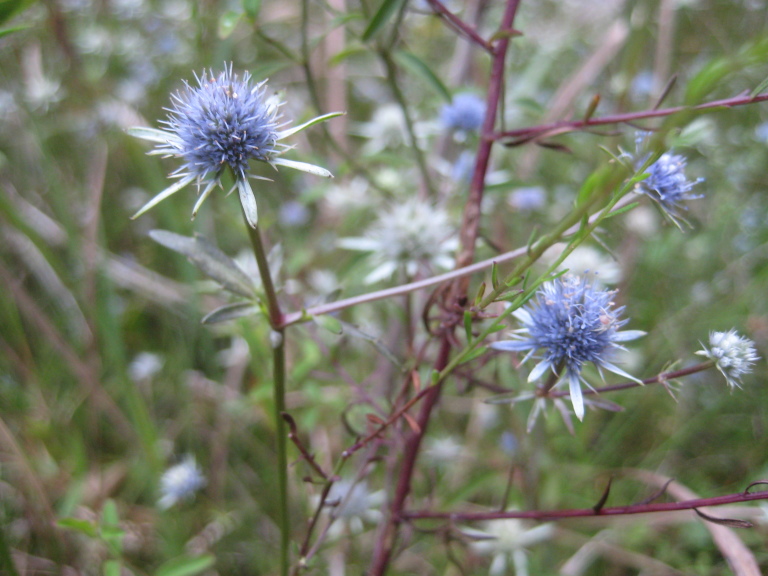
*Eryngium integrifolium*: inflorescence (photo by R. Thornhill).

**Figure 143c.**
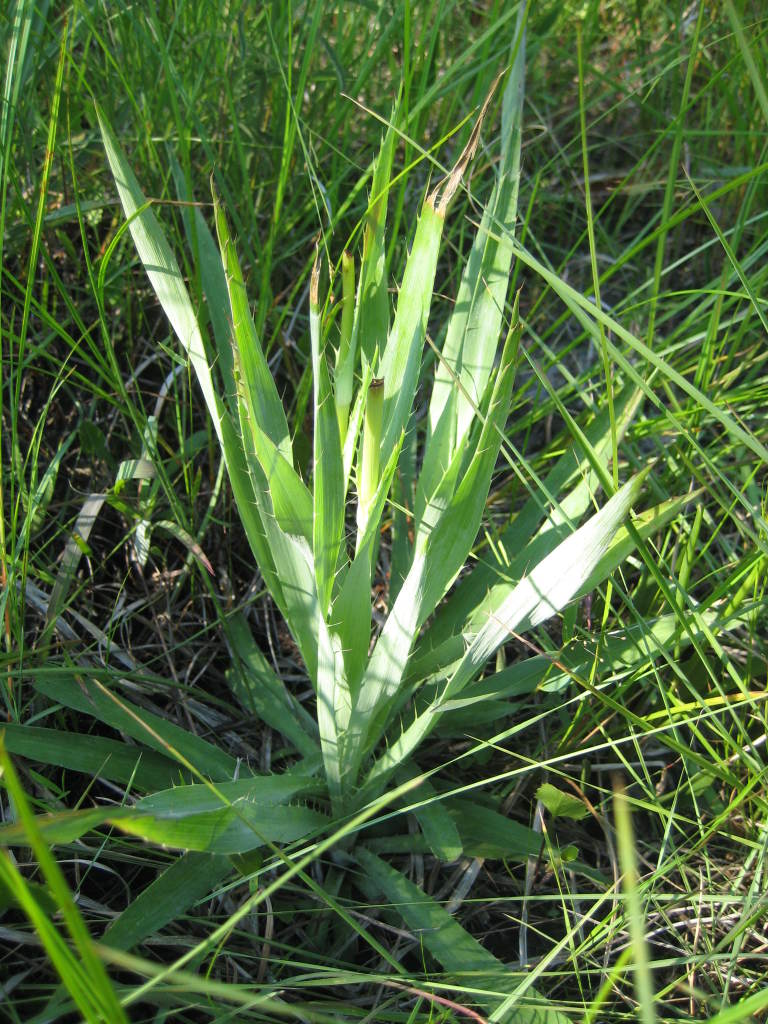
Eryngium yuccifolium var. yuccifolium: basal leaves (photo by R. Thornhill).

**Figure 143d.**
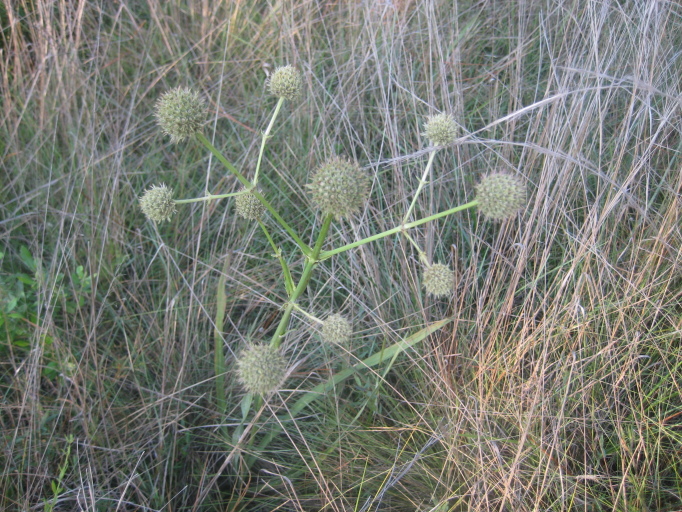
Eryngium yuccifolium var. yuccifolium: inflorescence (photo by R. Thornhill).

**Figure 144a.**
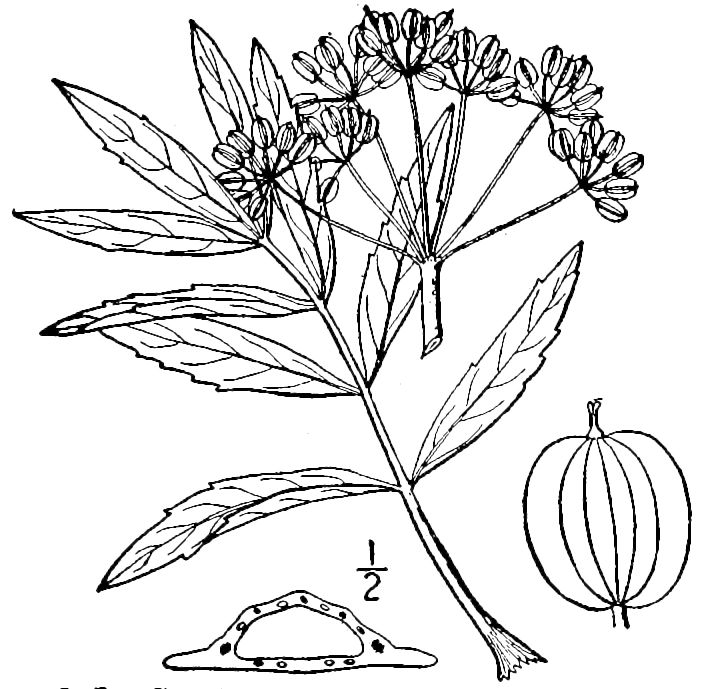
From Britton and Brown (1913).

**Figure 144b.**
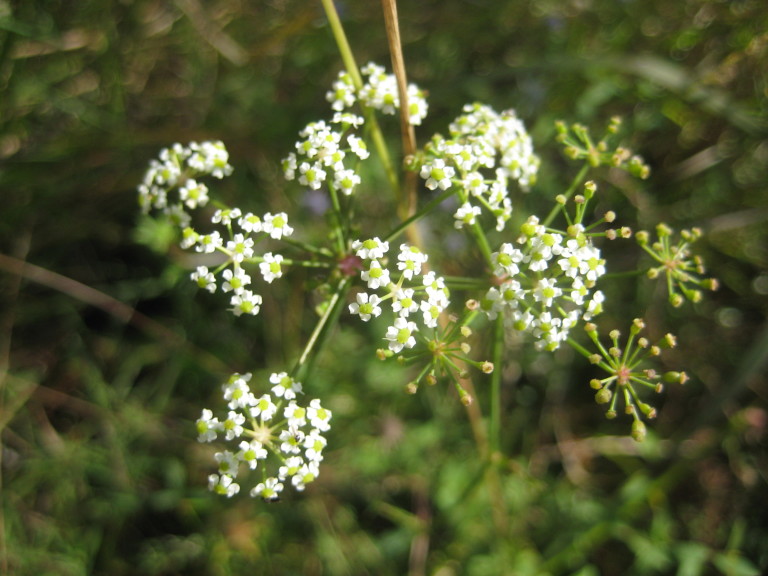
Photo by R. Thornhill.

**Figure 145.**
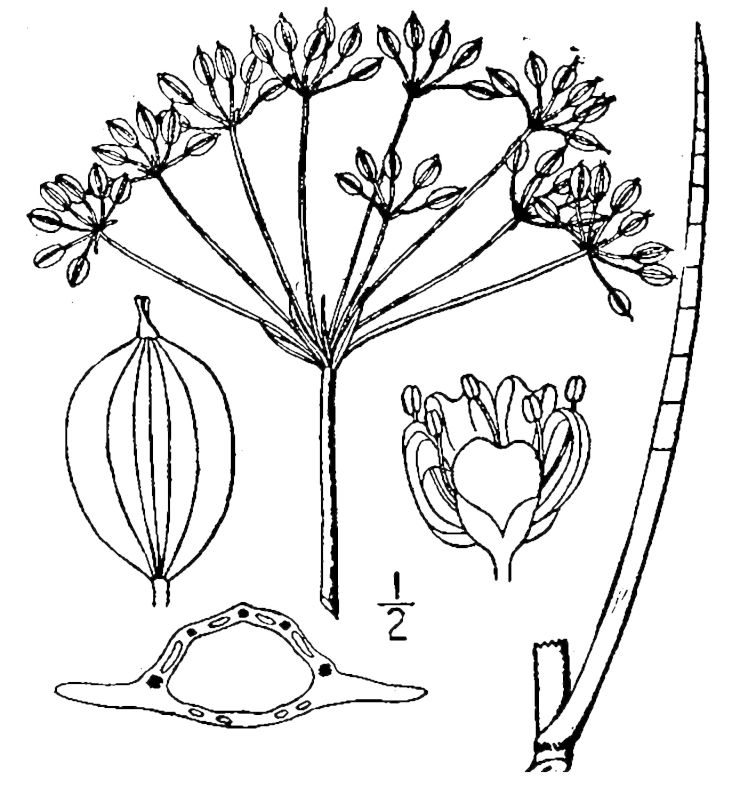
*Tiedemannia filiformis* (from Britton and Brown 1913).

**Figure 146a.**
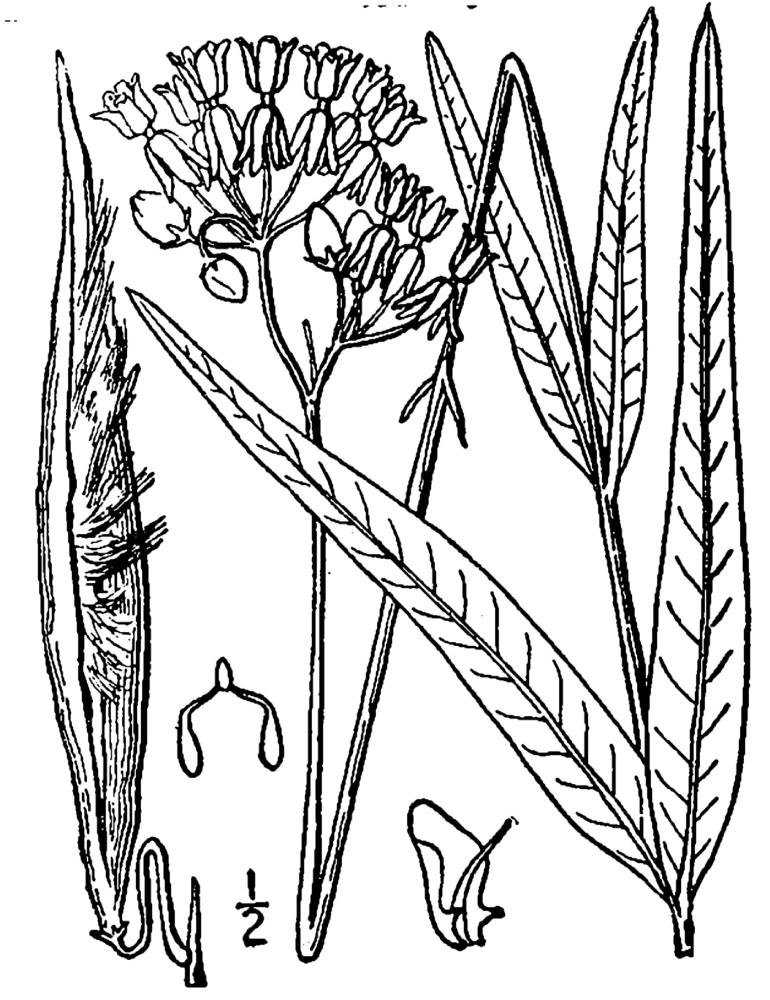
*Asclepias lanceolata* (from Britton and Brown 1913).

**Figure 146b.**
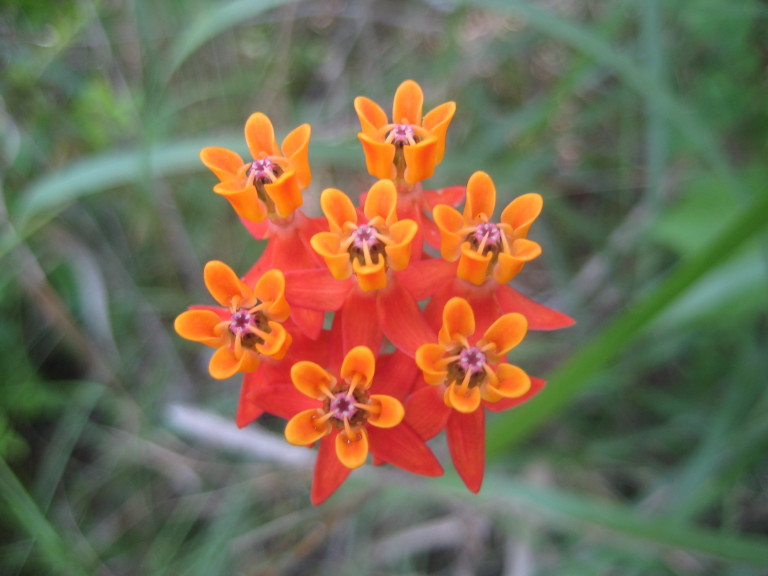
*Asclepias lanceolata* (photo by R. Thornhill).

**Figure 146c.**
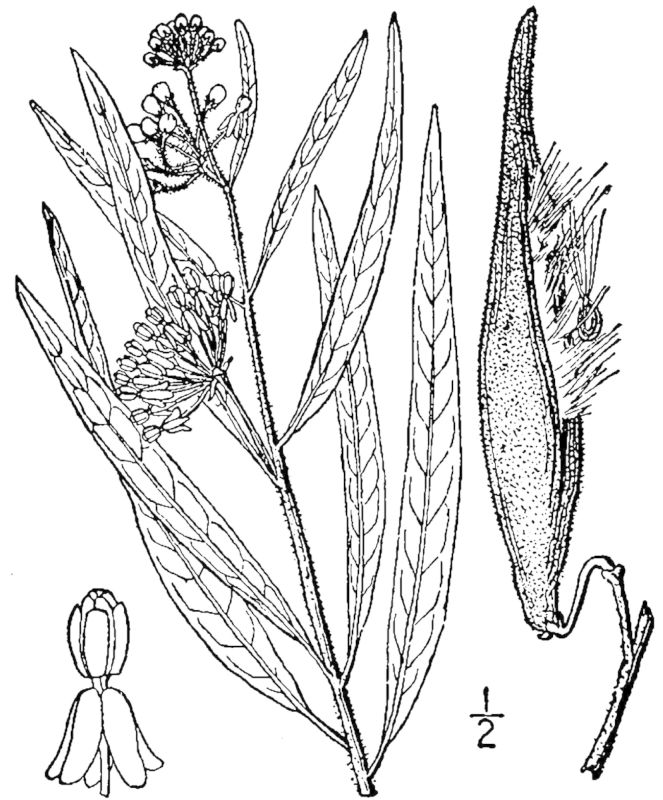
*Asclepias longifolia* (from Britton and Brown 1913).

**Figure 146d.**
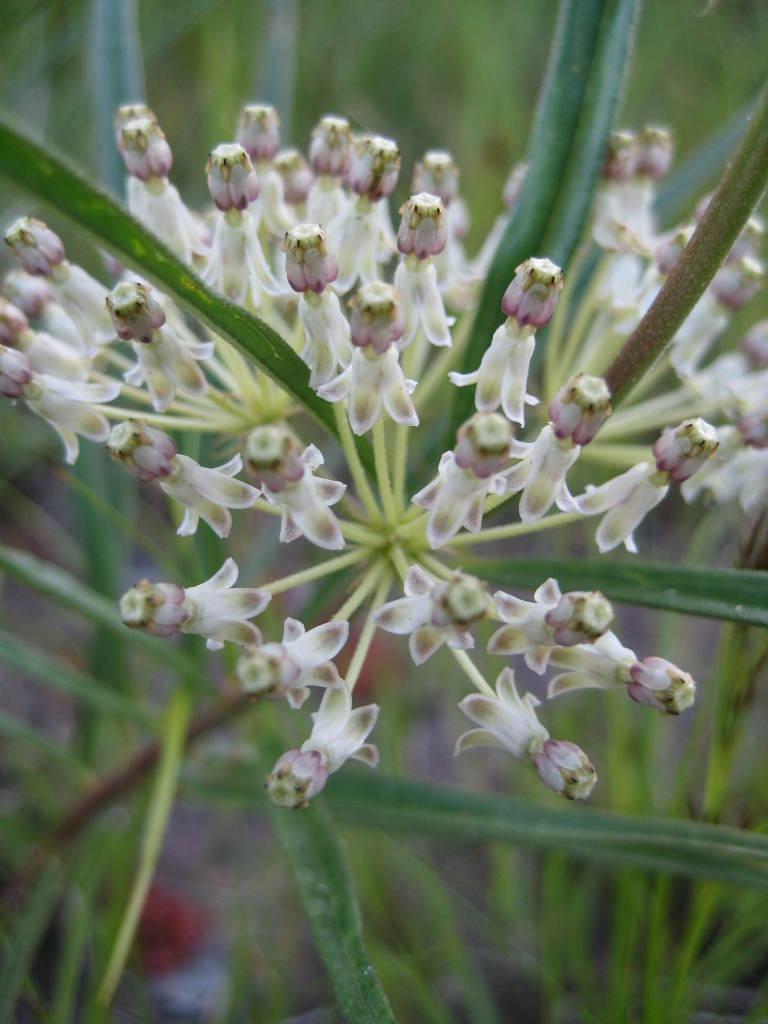
*Asclepias longifolia* (photo by R. Thornhill).

**Figure 146e.**
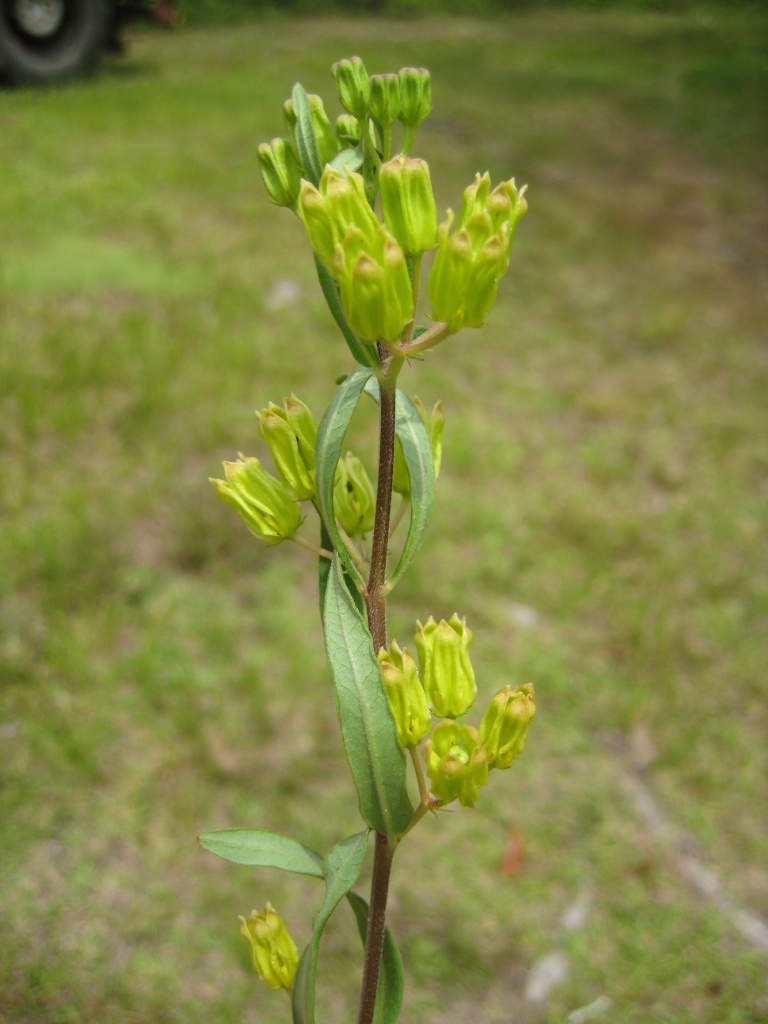
*Asclepias pedicellata* (photo by R. Thornhill).

**Figure 146f.**
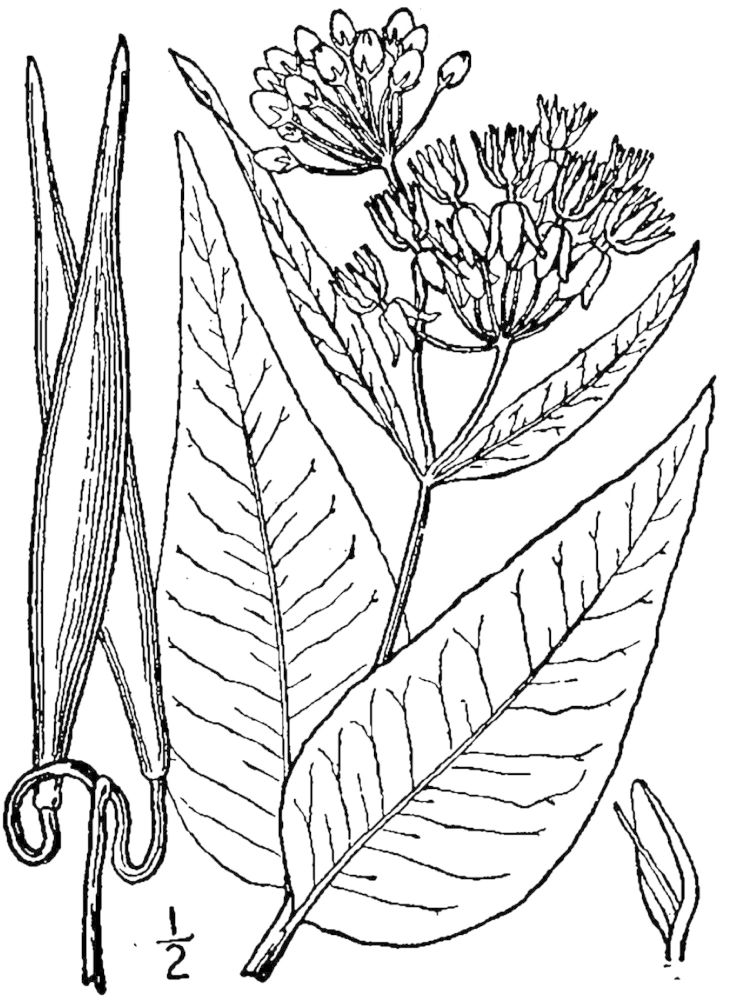
*Asclepias rubra* (from Britton and Brown 1913).

**Figure 147a.**
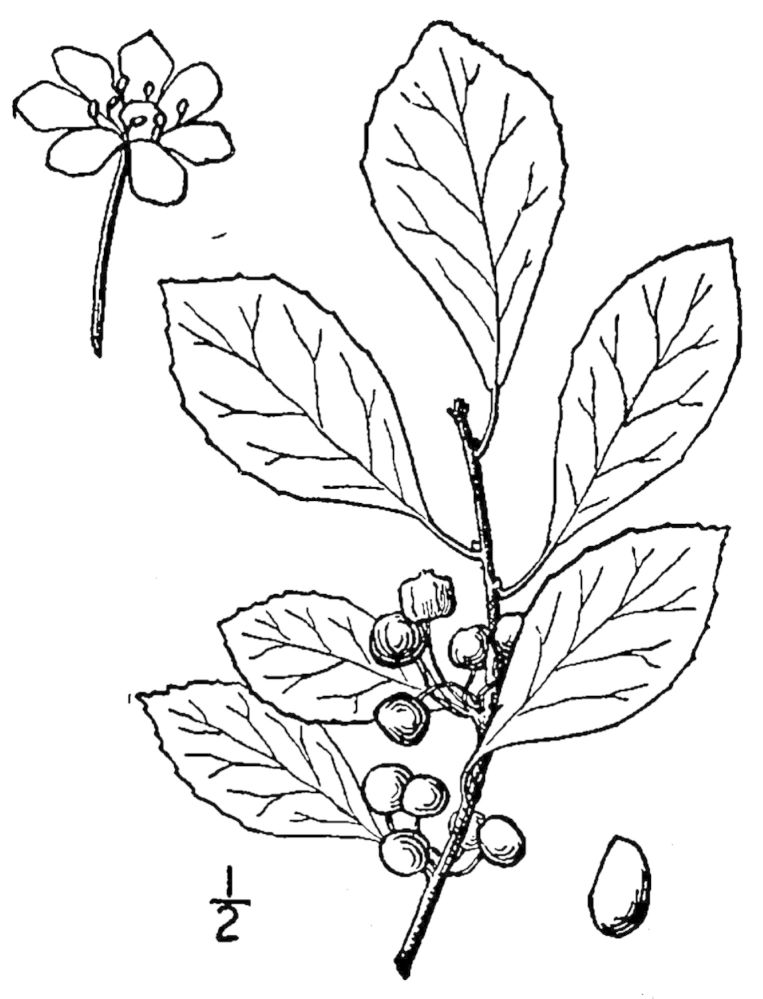
*Ilex coriacea* (from Britton and Brown 1913).

**Figure 147b.**
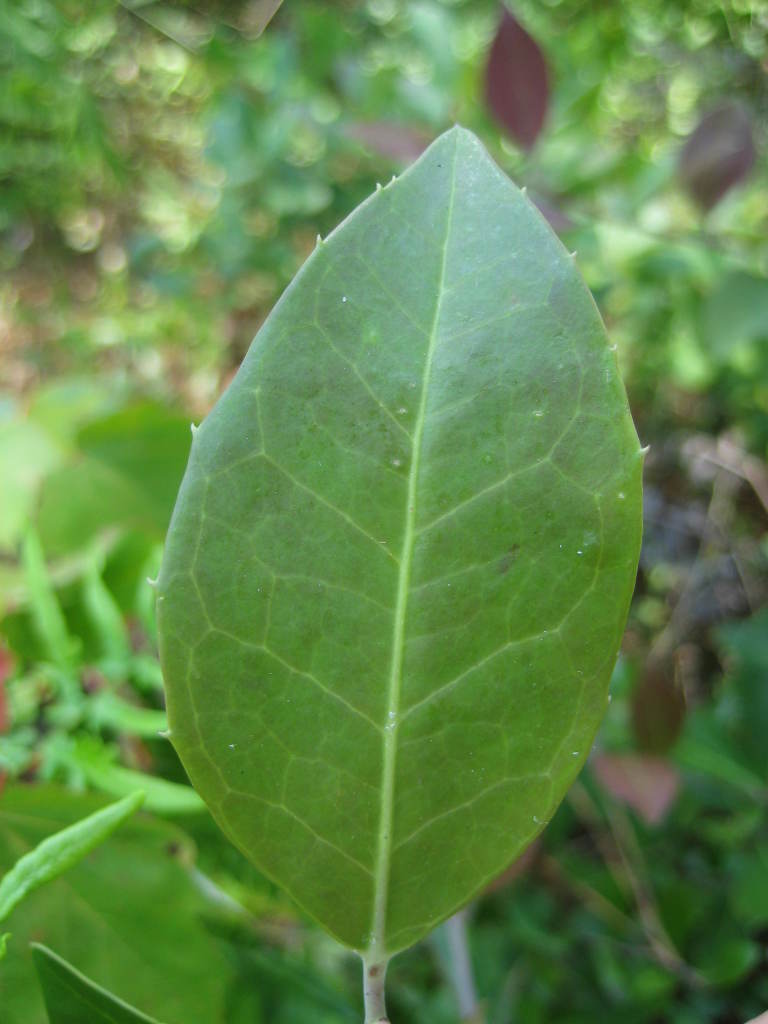
*Ilex coriacea*: note the outwardly-pointed prickles on the leaf margins (photo by R. Thornhill).

**Figure 147c.**
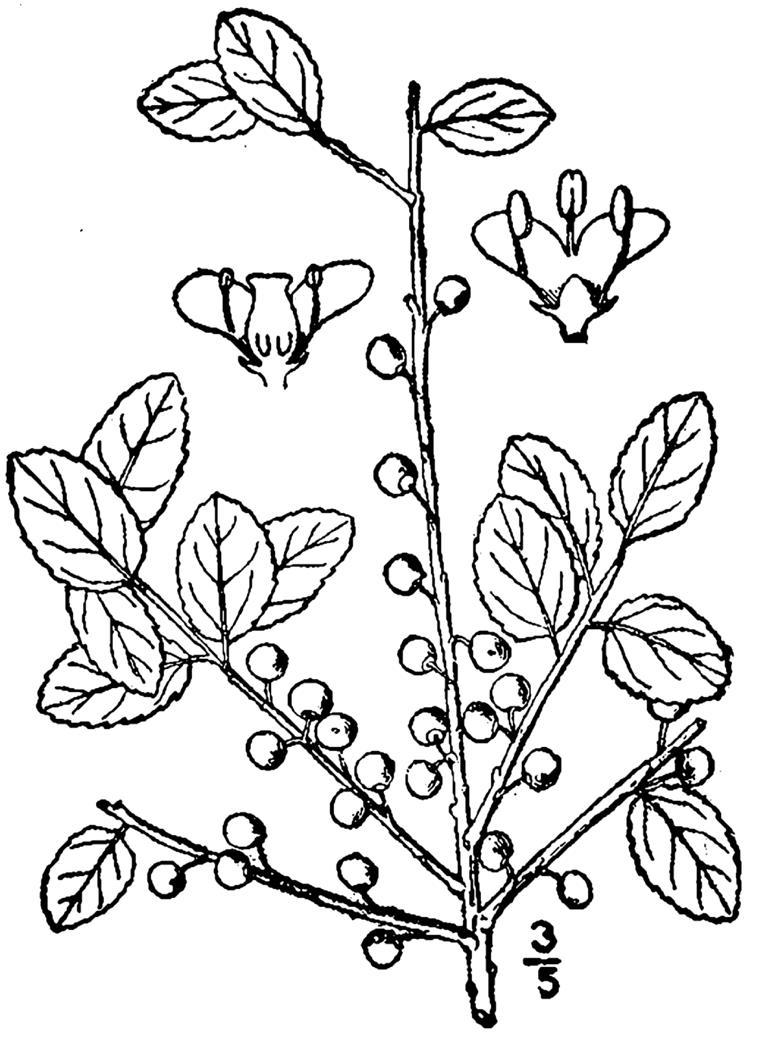
*Ilex glabra* (from Britton and Brown 1913).

**Figure 147d.**
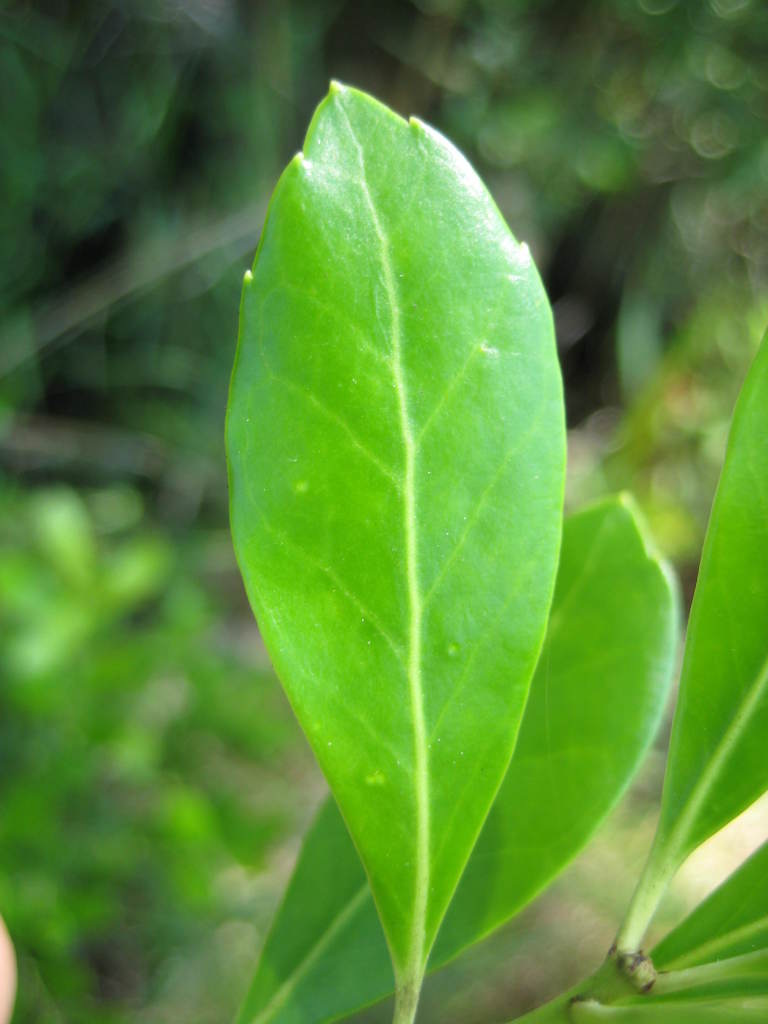
*Ilex glabra*: note the inwardly-curved teeth on the leaf margins (photo by R. Thornhill).

**Figure 147e.**
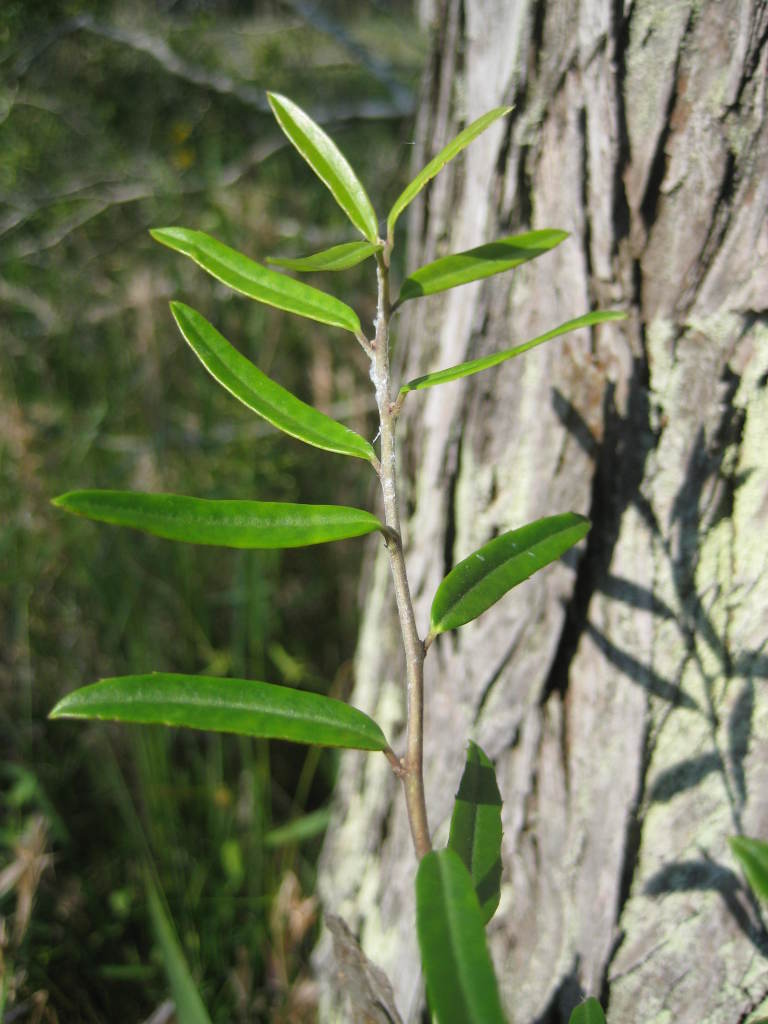
*Ilex myrtifolia* (photo by R. Thornhill).

**Figure 147f.**
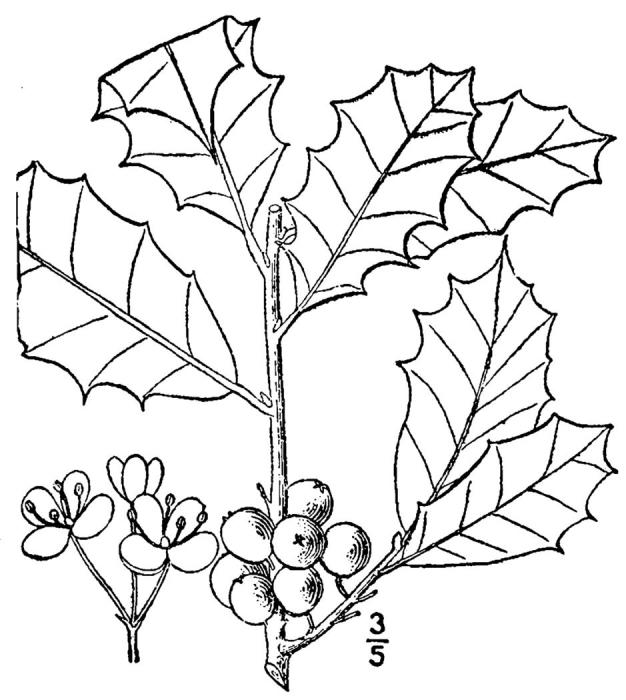
*Ilex opaca* (from Britton and Brown 1913).

**Figure 148.**
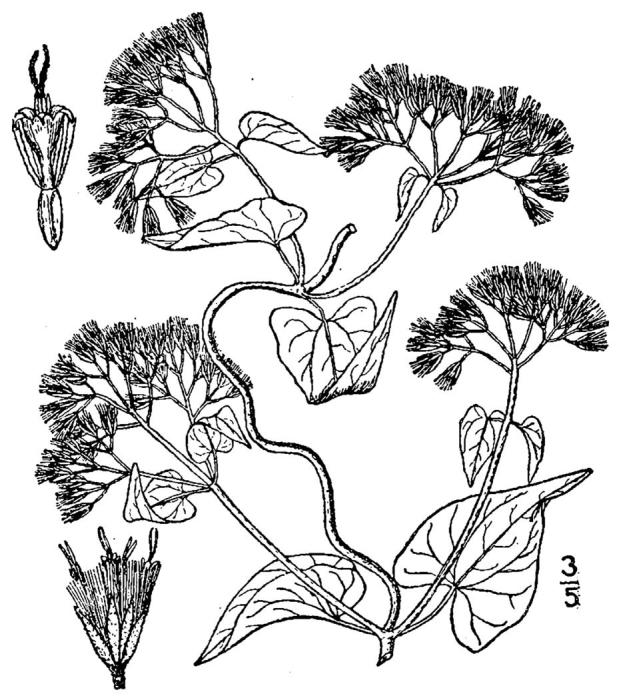
*Mikania scandens* (from Britton and Brown 1913).

**Figure 149a.**
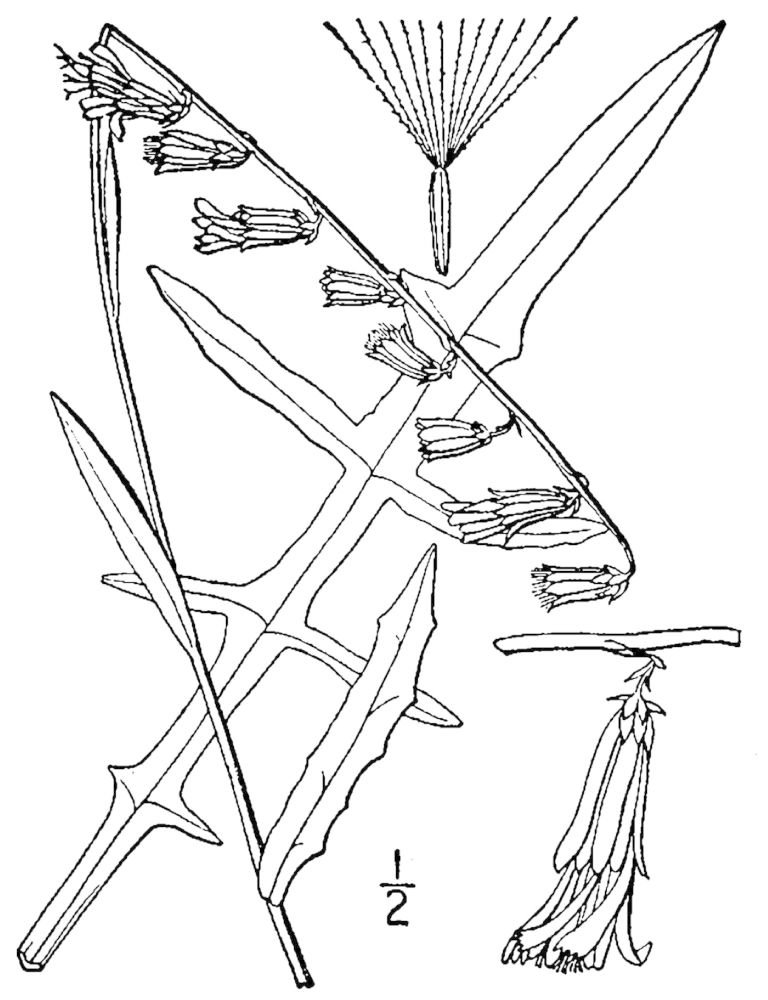
From Britton and Brown (1913).

**Figure 149b.**
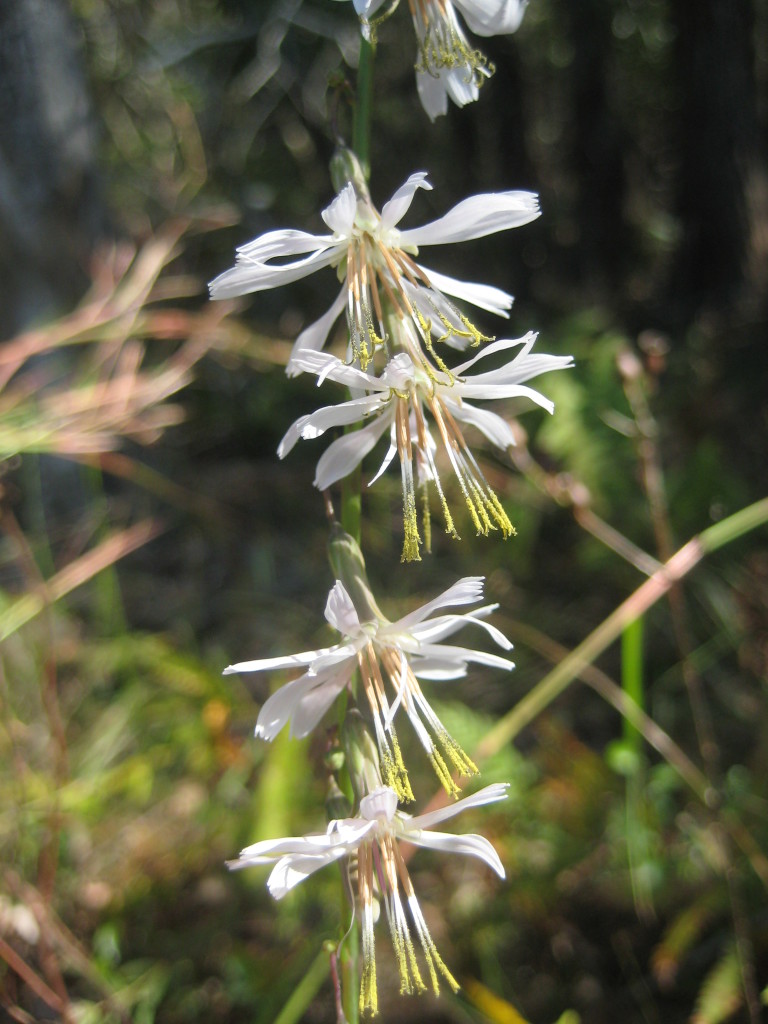
Photo by R. Thornhill.

**Figure 150a.**
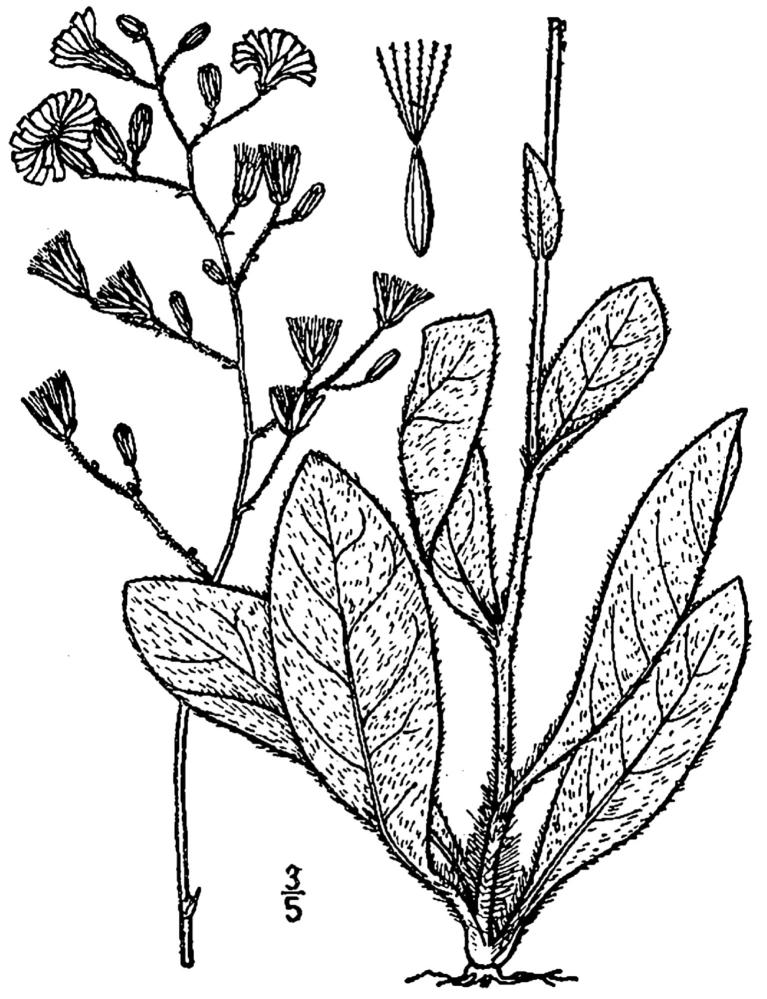
From Britton and Brown (1913).

**Figure 150b.**
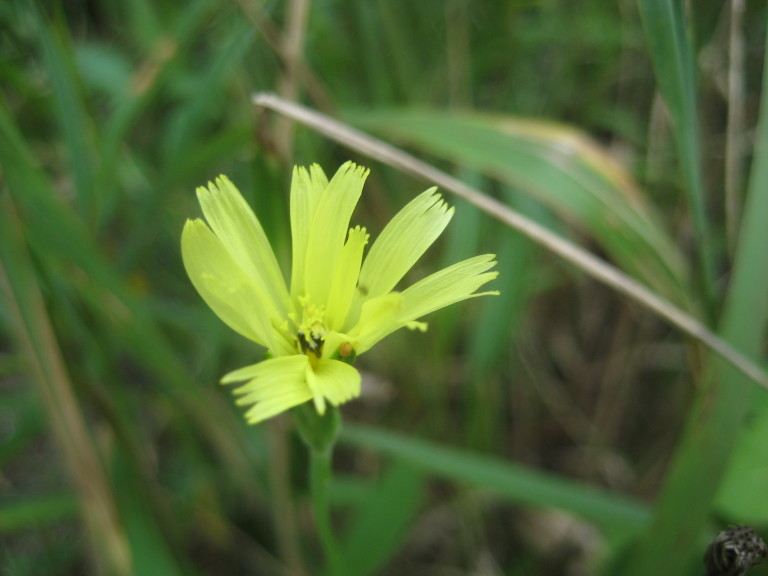
Photo by R. Thornhill.

**Figure 151.**
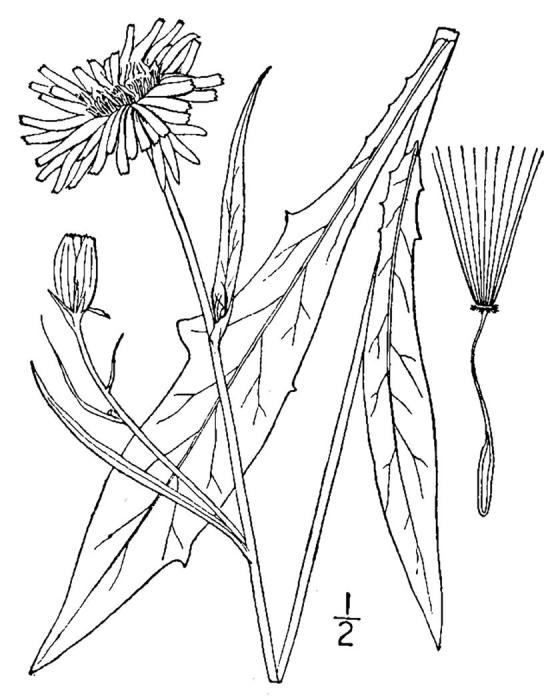
*Pyrrhopappus carolinianus* (from Britton and Brown 1913).

**Figure 152.**
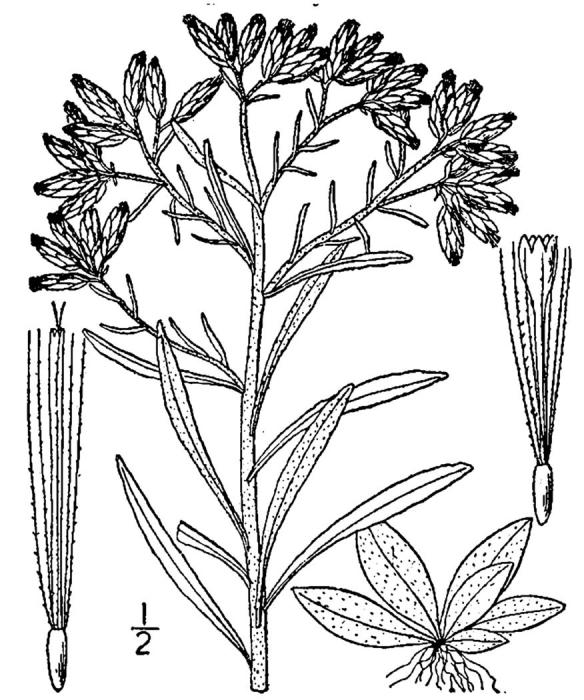
*Pseudognaphalium obtusifolium* (from Britton and Brown 1913).

**Figure 153a.**
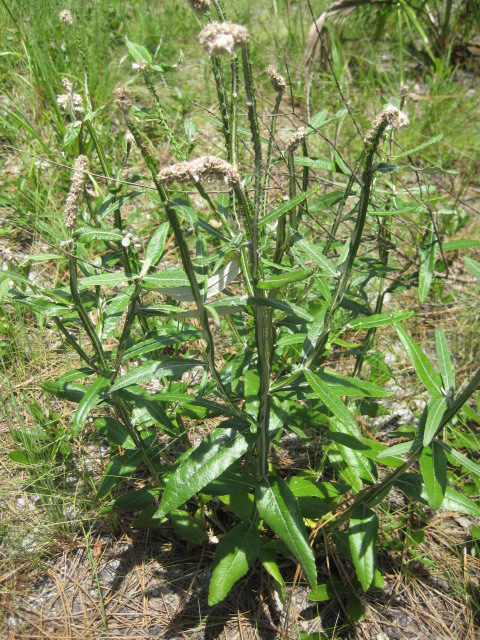
Photo by R. Thornhill.

**Figure 153b.**
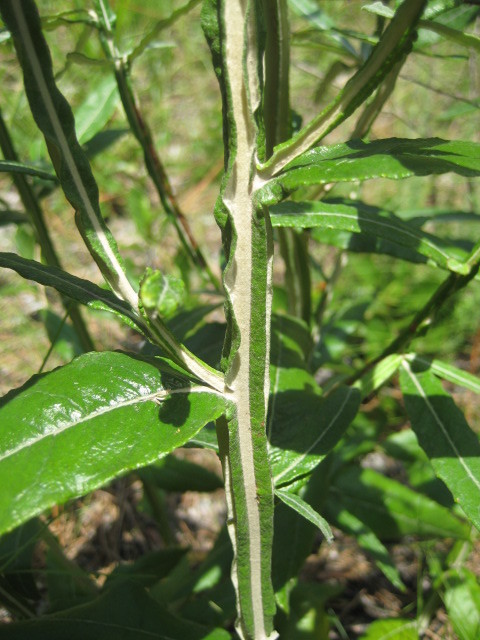
Photo by R. Thornhill.

**Figure 154a.**
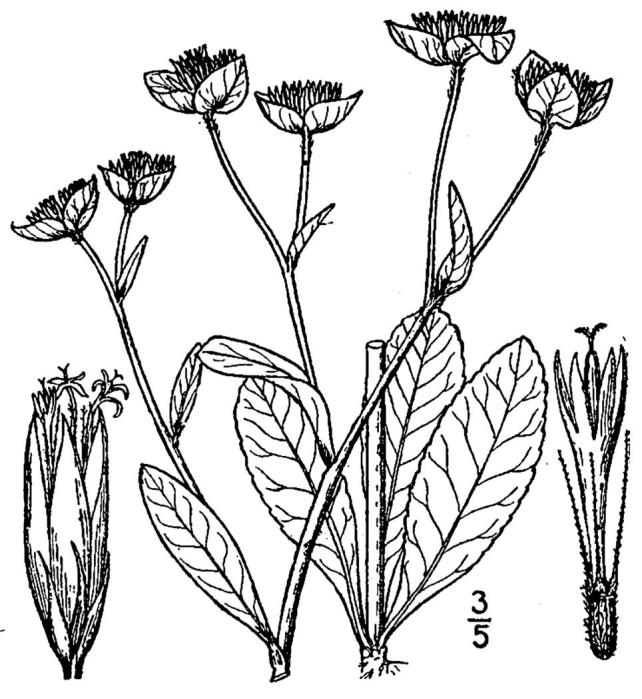
From Britton and Brown (1913).

**Figure 154b.**
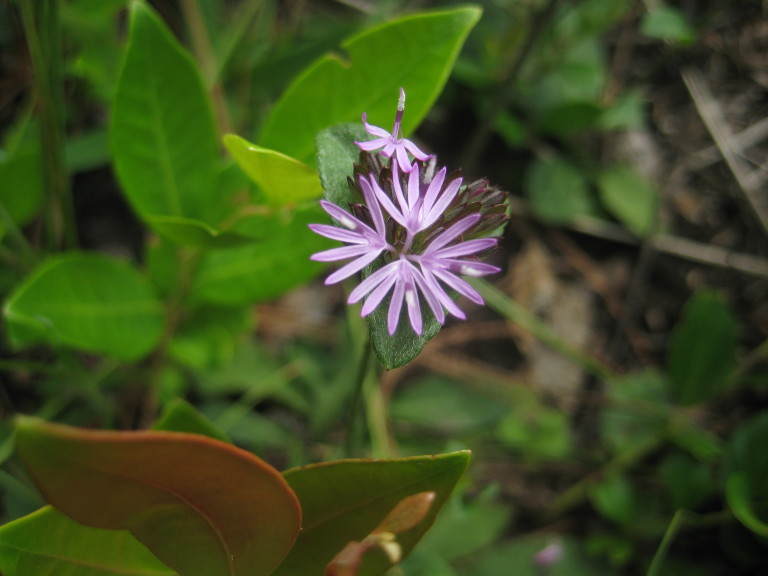
Photo by R. Thornhill.

**Figure 155.**
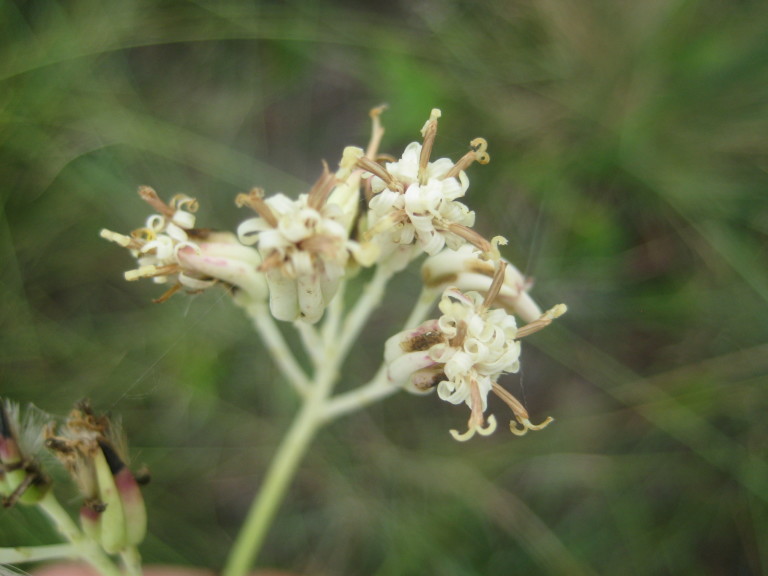
Arnoglossum ovatum var. lanceolatum (photo by R. Thornhill).

**Figure 156.**
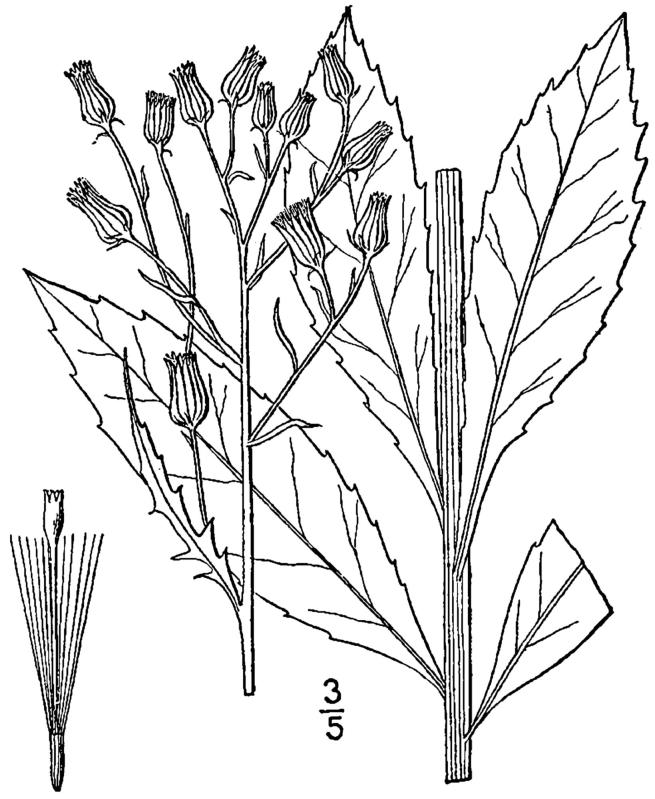
*Erechtites hieraciifolius* (from Britton and Brown 1913).

**Figure 157.**
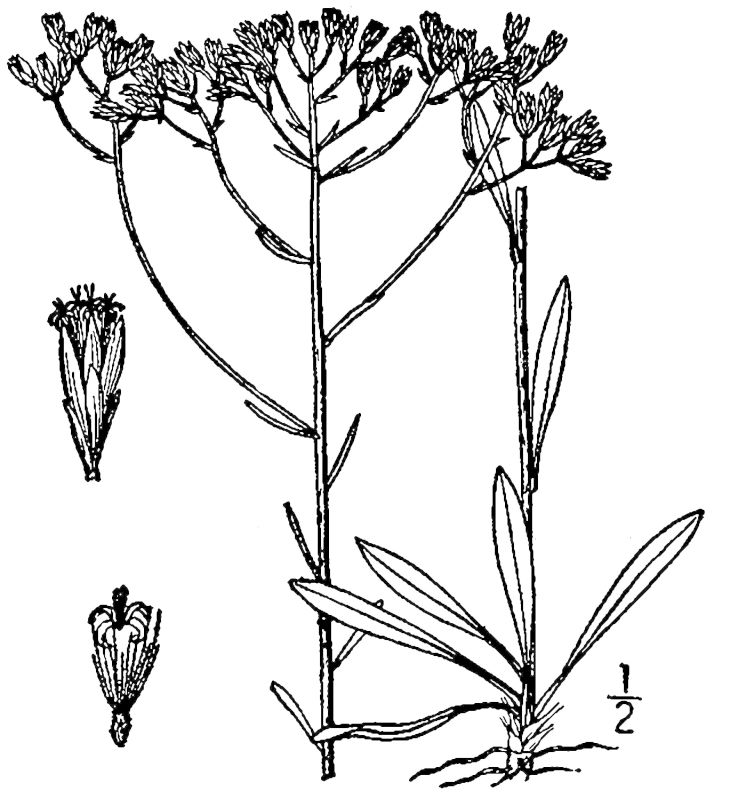
Bigelowia nudata var. nudata (from Britton and Brown 1913).

**Figure 158.**
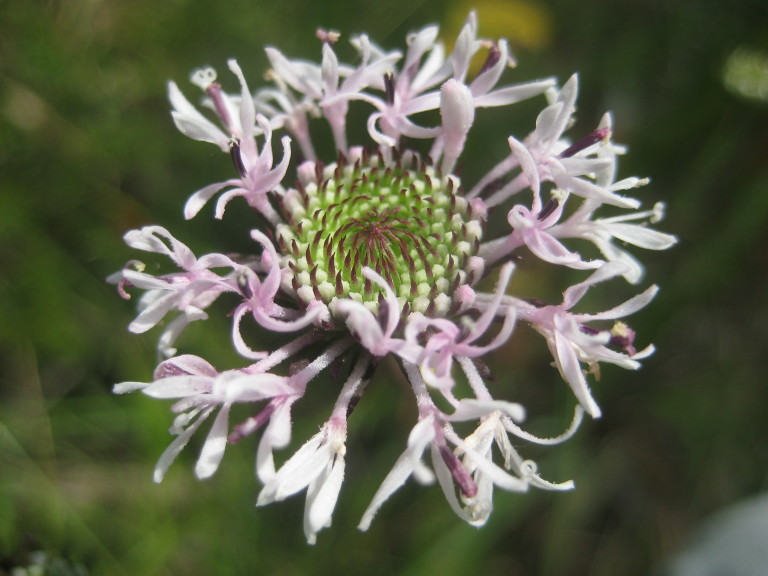
*Marshallia graminifolia* (photo by R. Thornhill).

**Figure 159a.**
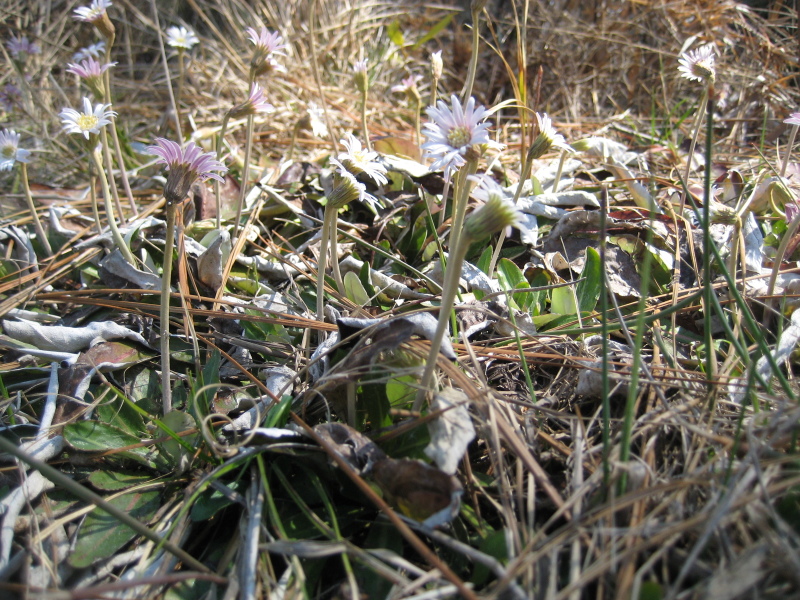
Note the white undersurface of the curled leaves (photo by R. Thornhill).

**Figure 159b.**
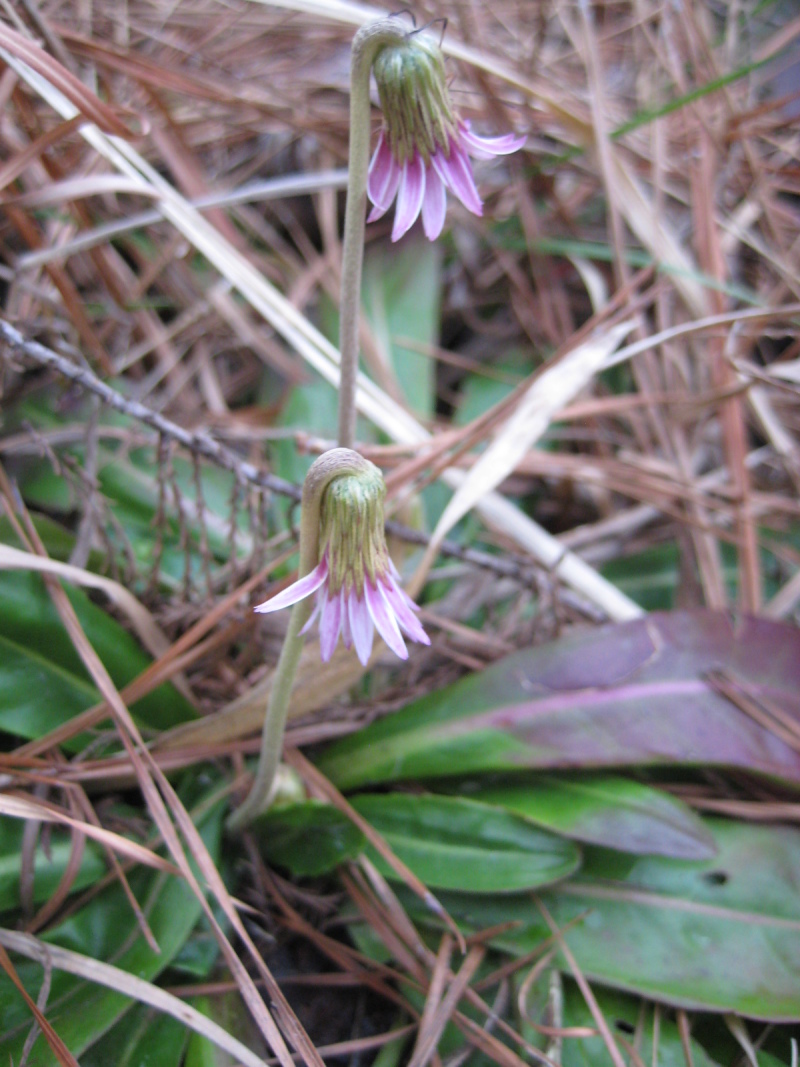
Photo by R. Thornhill

**Figure 160.**
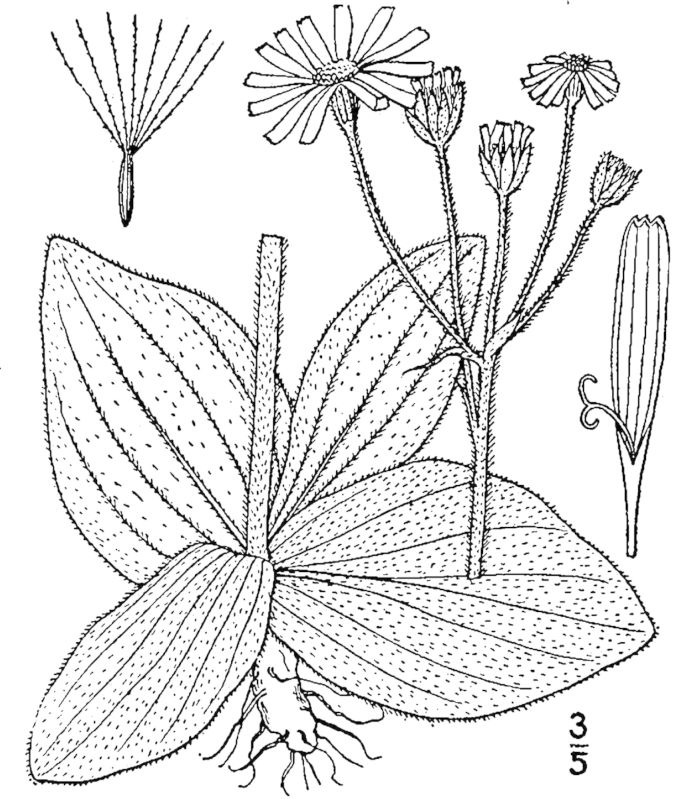
*Arnica acaulis* (from Britton and Brown 1913).

**Figure 161.**
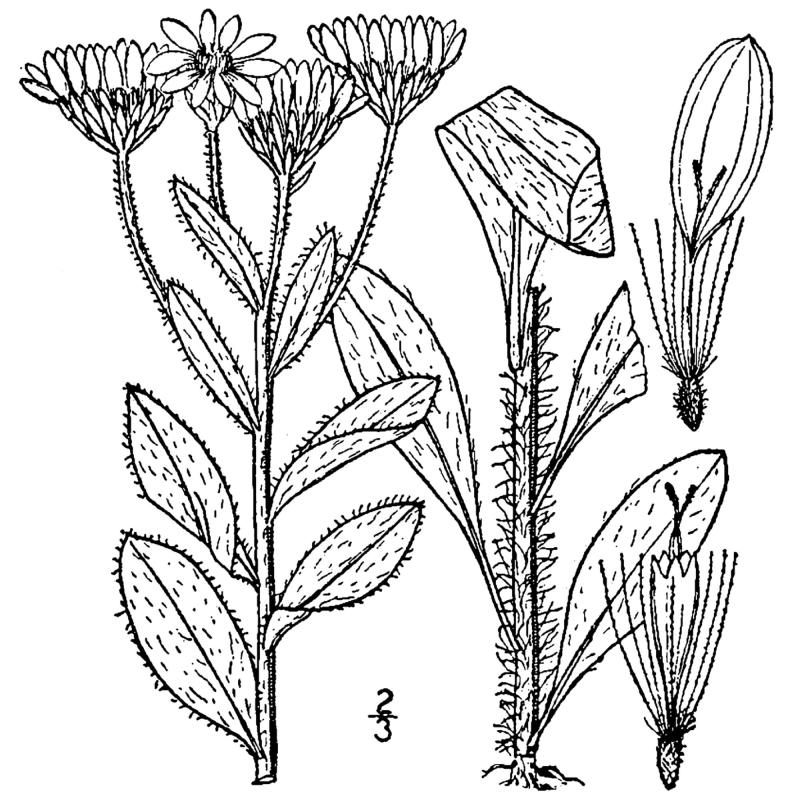
*Chrysopsis mariana* (from Britton and Brown 1913).

**Figure 162a.**
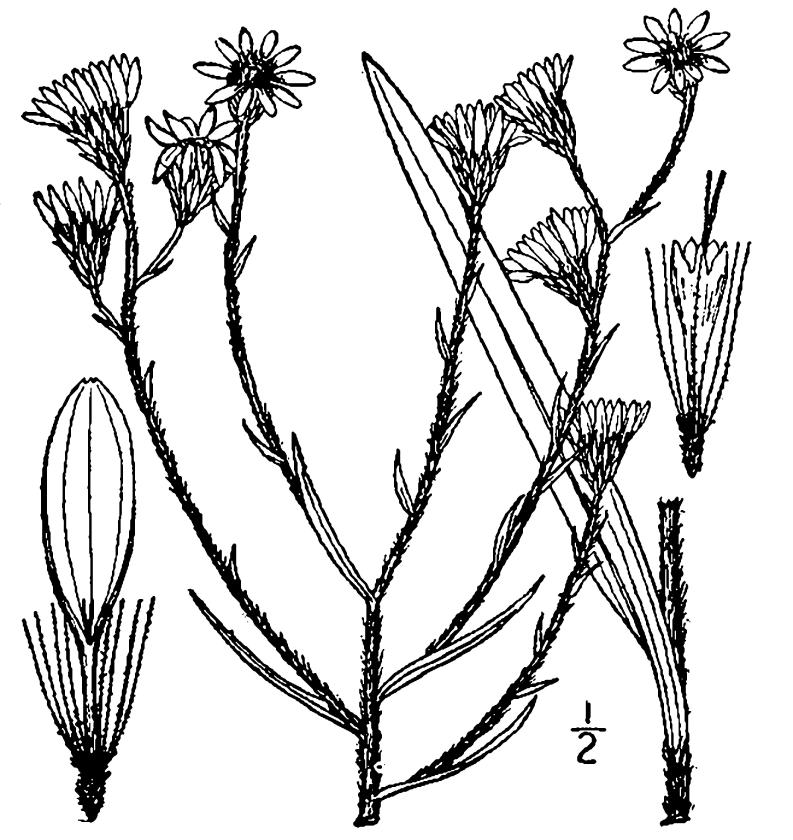
From Britton and Brown (1913).

**Figure 162b.**
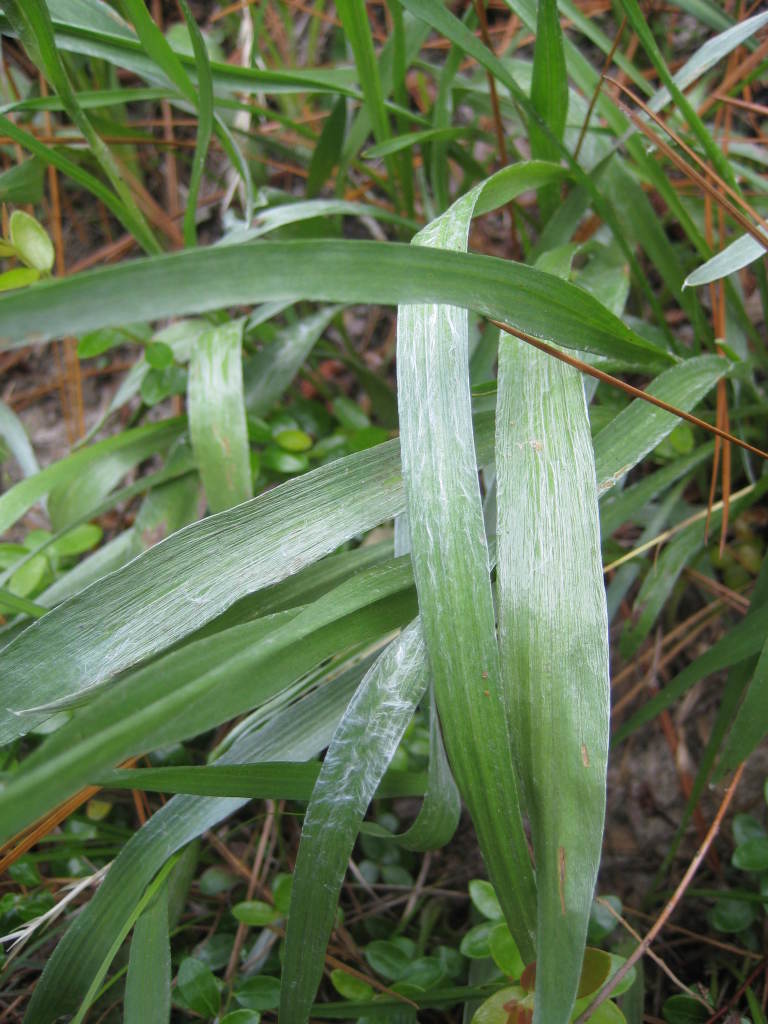
Photo by R. Thornhill.

**Figure 163a.**
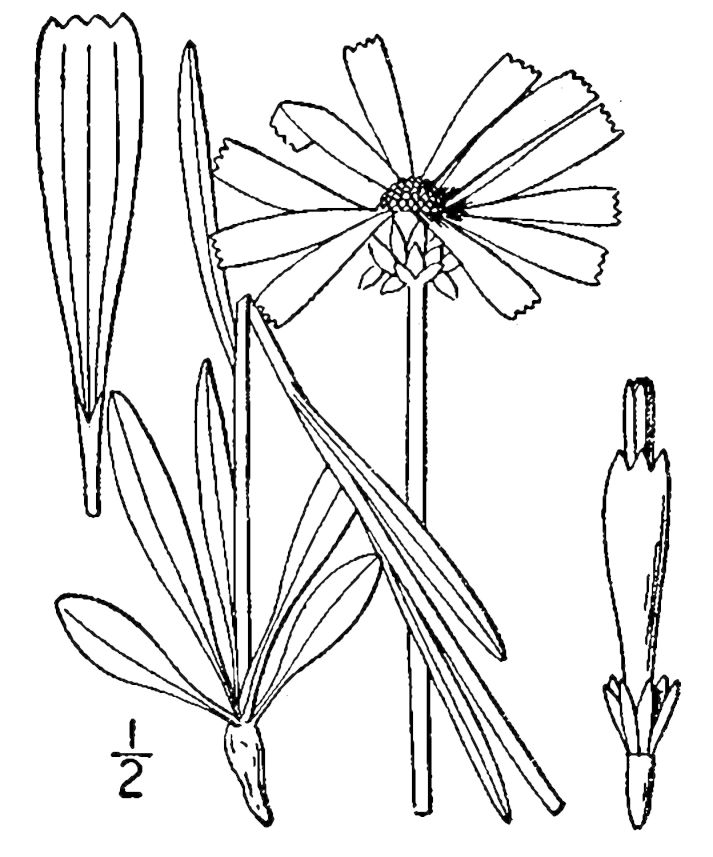
From Britton and Brown (1913).

**Figure 163b.**
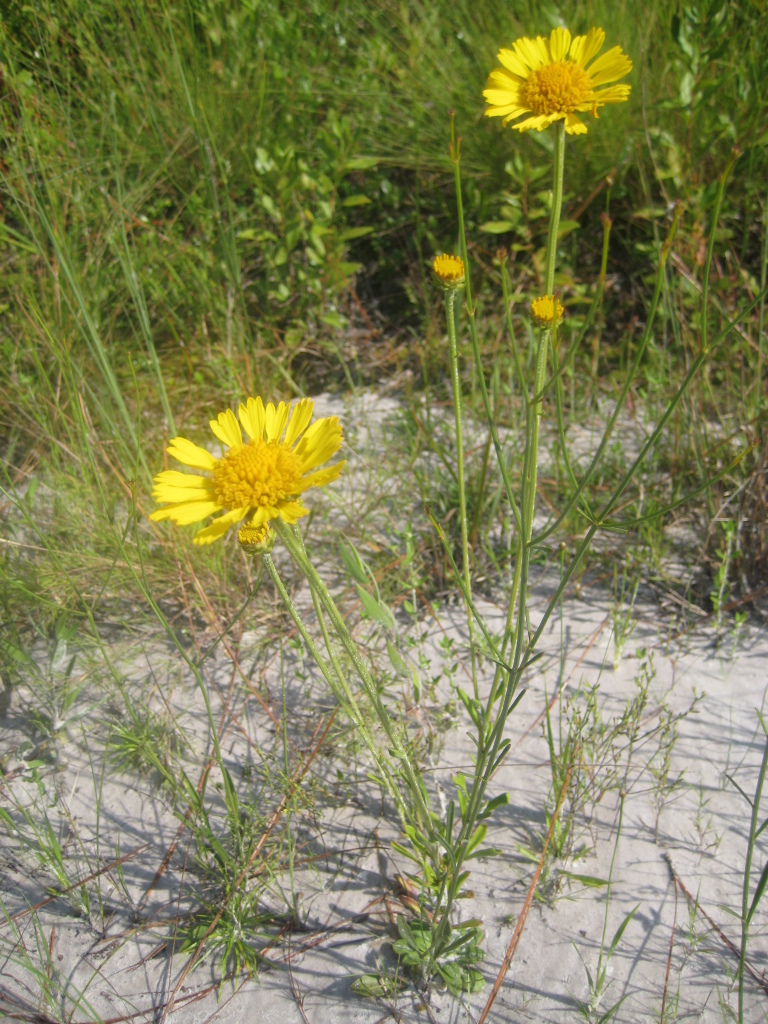
Photo by R. Thornhill.

**Figure 164a.**
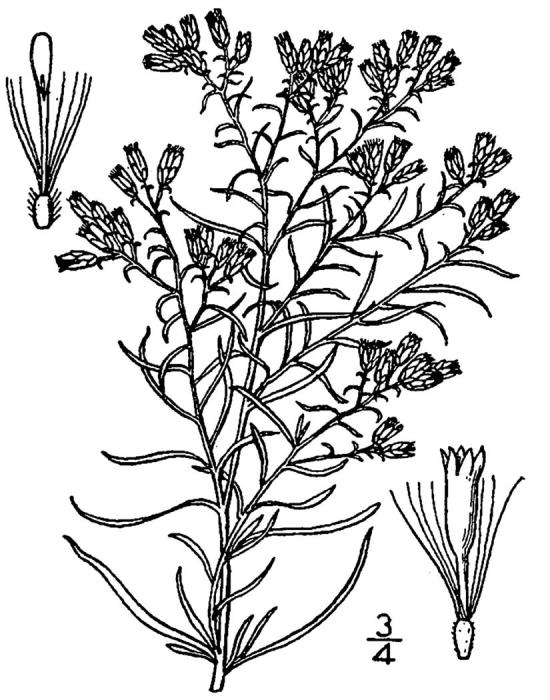
From Britton and Brown (1913).

**Figure 164b.**
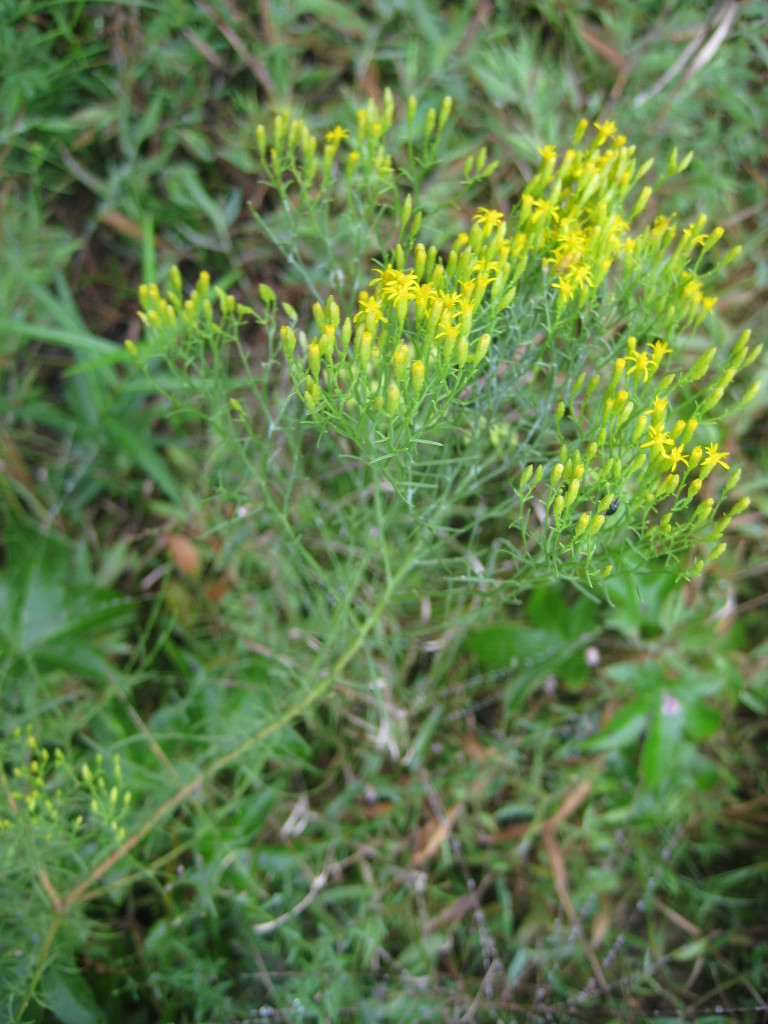
Photo by R. Thornhill.

**Figure 165a.**
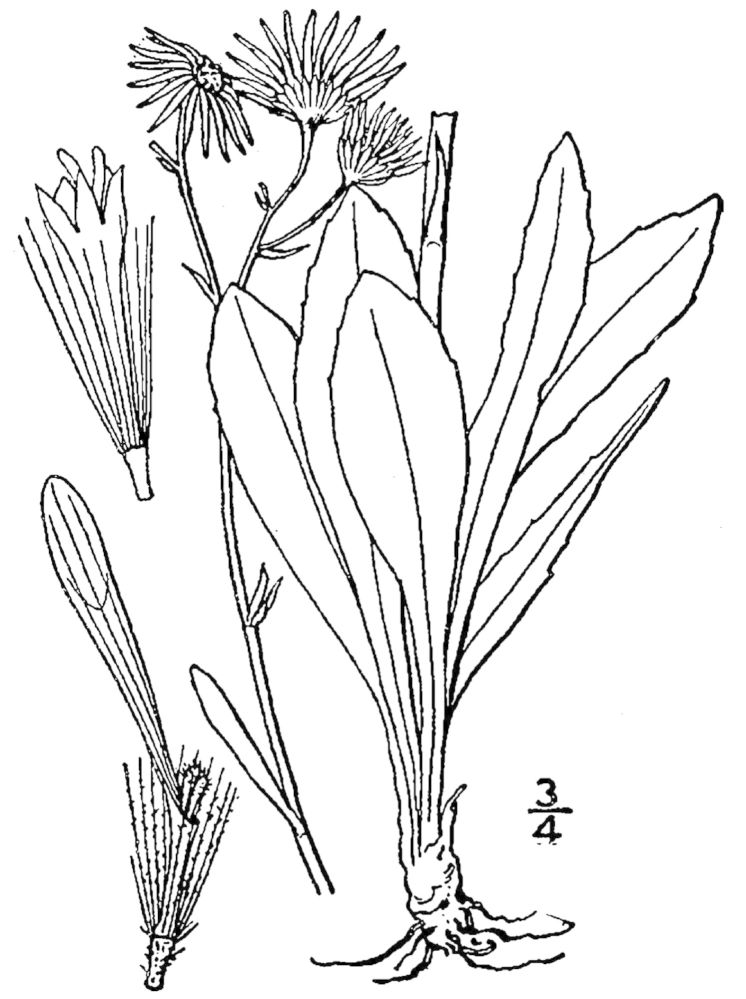
From Britton and Brown (1913).

**Figure 165b.**
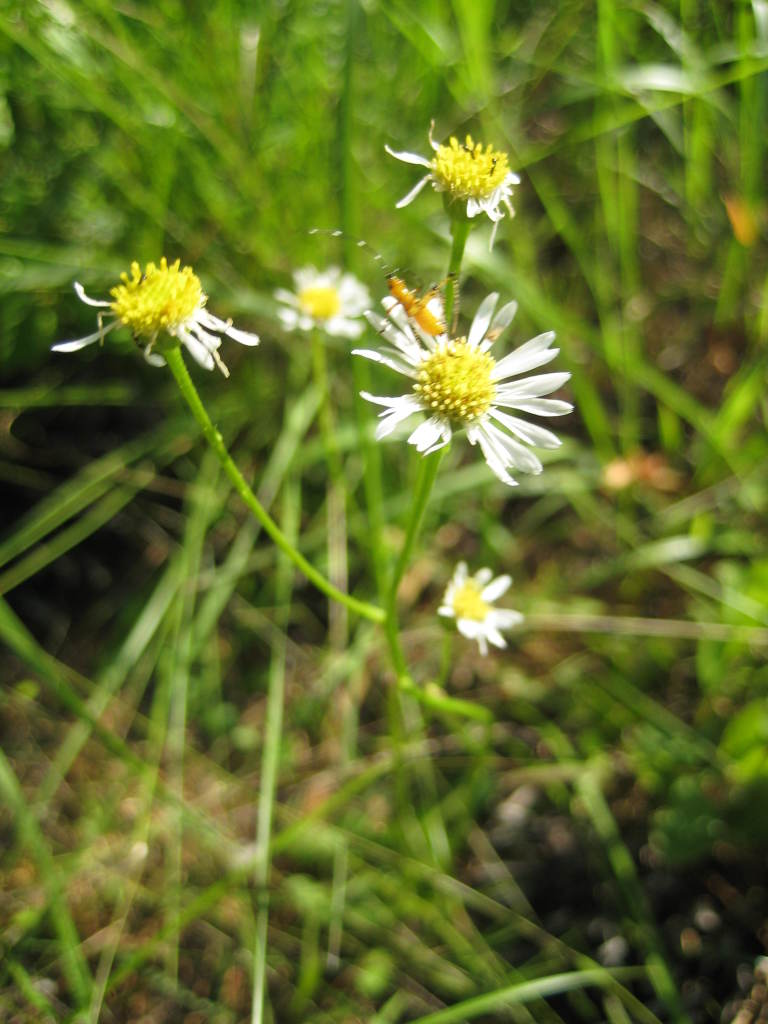
Photo by R. Thornhill.

**Figure 166.**
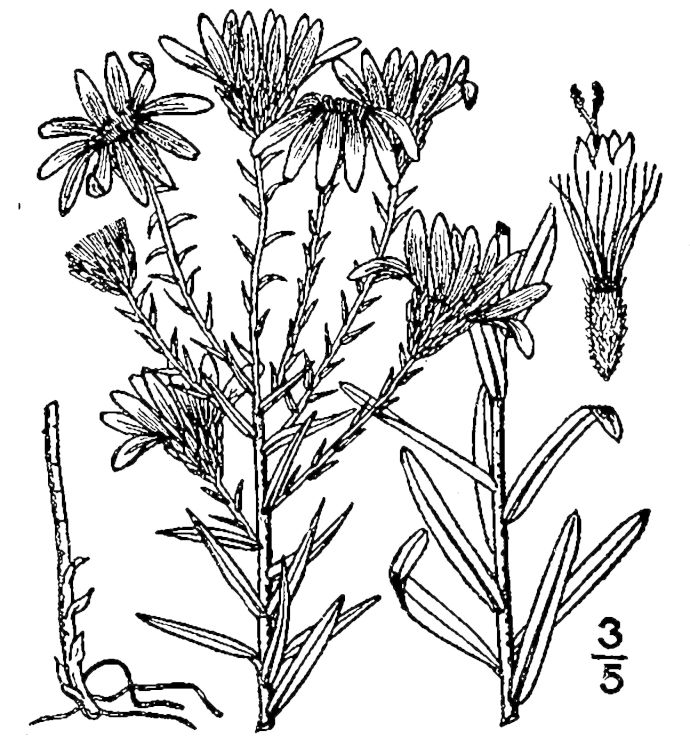
*Ionactis linariifolia* (from Britton and Brown 1913).

**Figure 167a.**
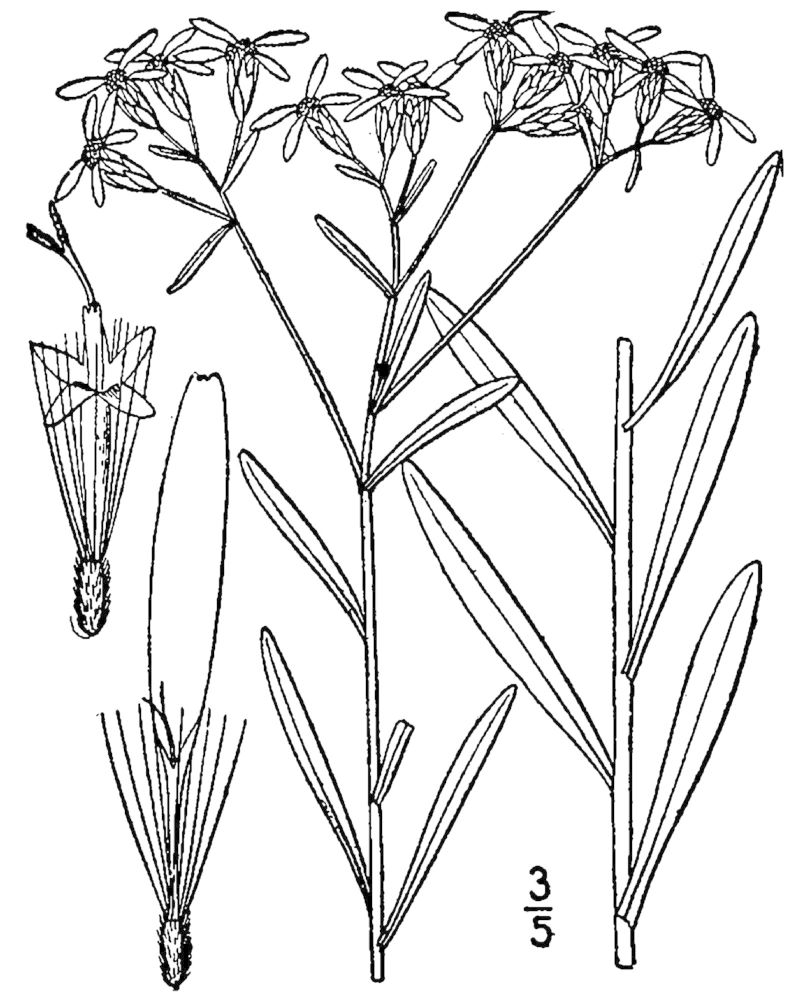
From Britton and Brown (1913).

**Figure 167b.**
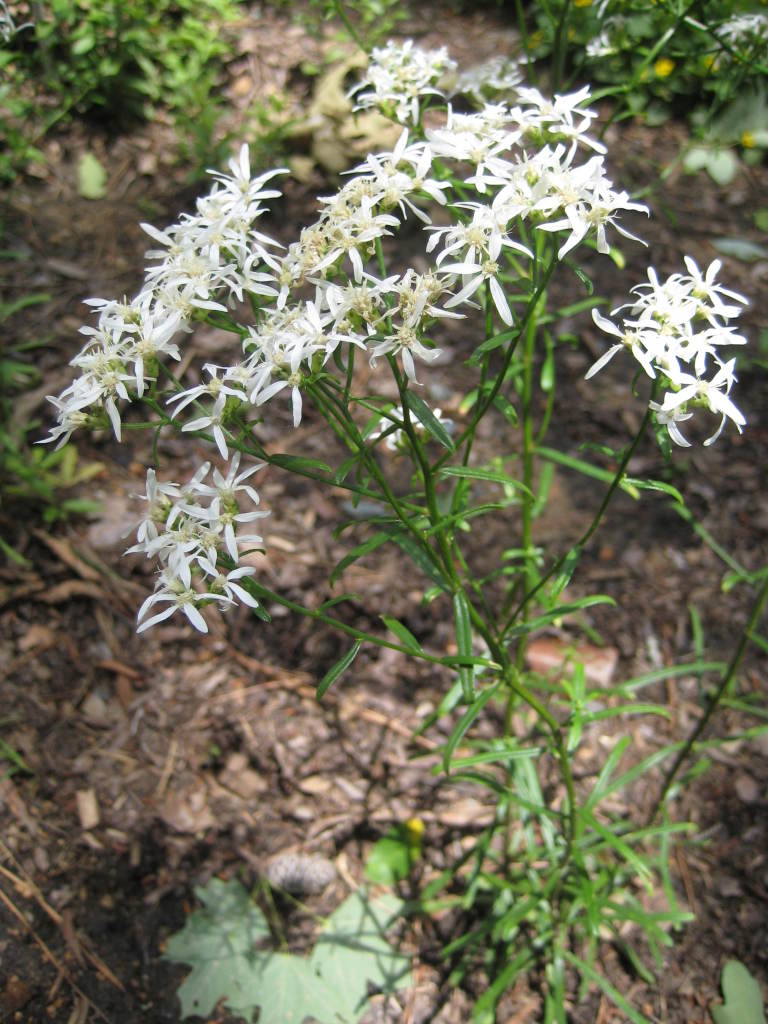
Photo by R. Thornhill.

**Figure 168a.**
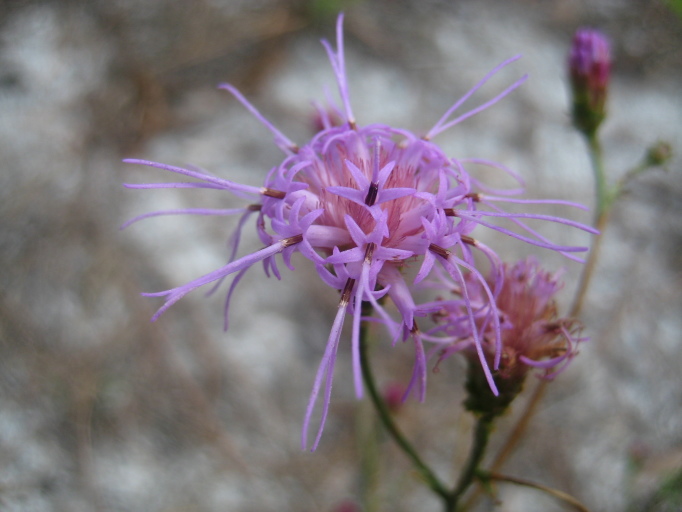
Photo by R. Thornhill

**Figure 168b.**
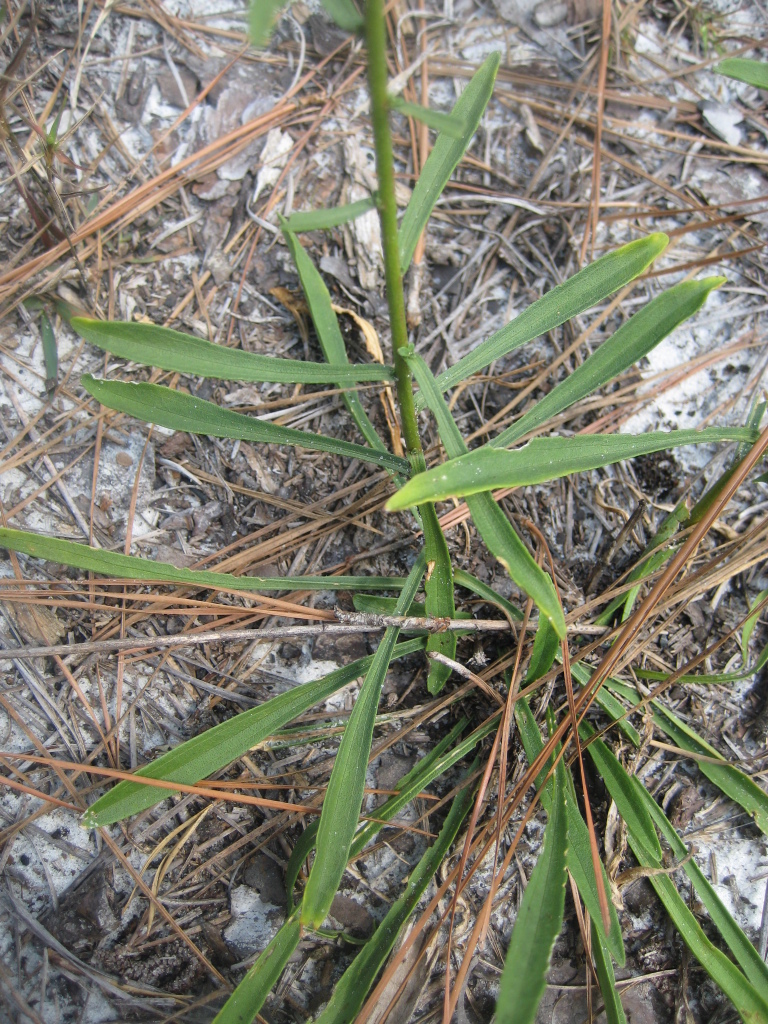
Photo by R. Thornhill

**Figure 169a.**
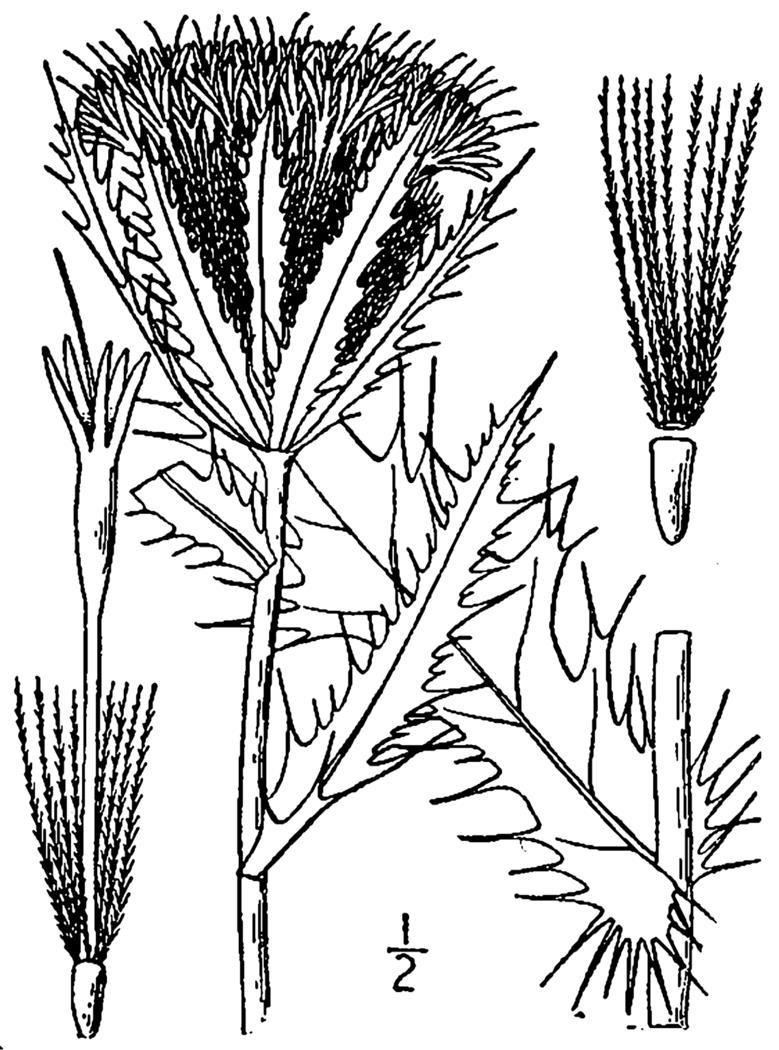
*Cirsium horridulum* (from Britton and Brown 1913).

**Figure 169b.**
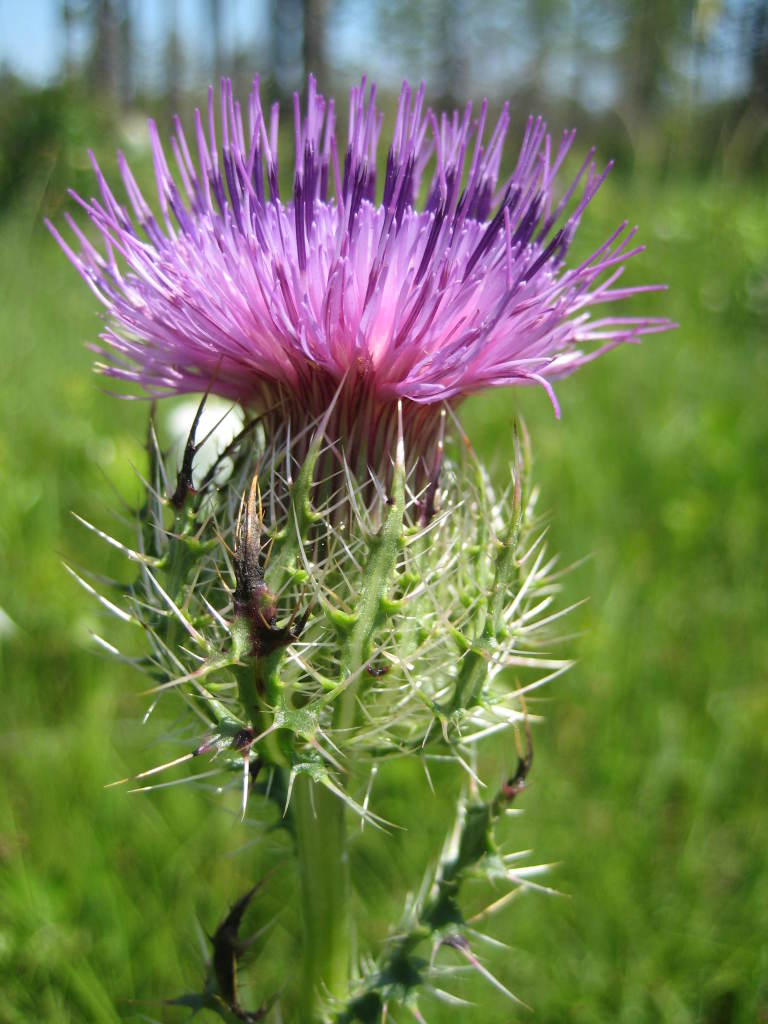
Cirsium horridulum var. vittatum (photo by R. Thornhill).

**Figure 169c.**
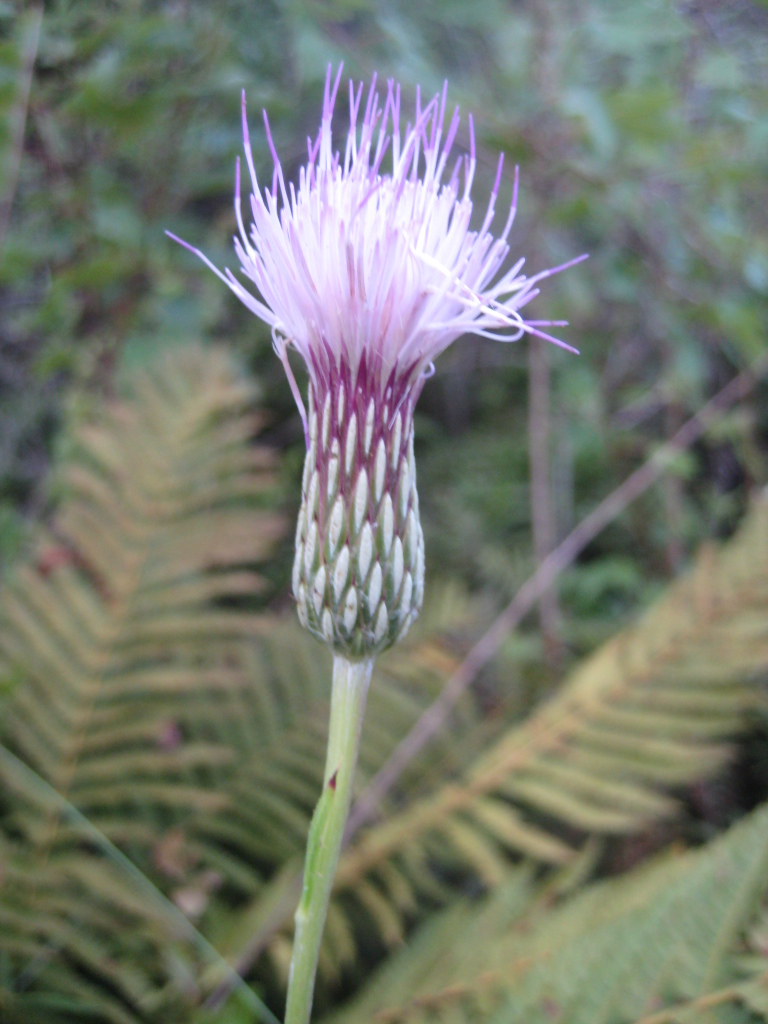
*Cirsium lecontei* (photo by R. Thornhill).

**Figure 169d.**
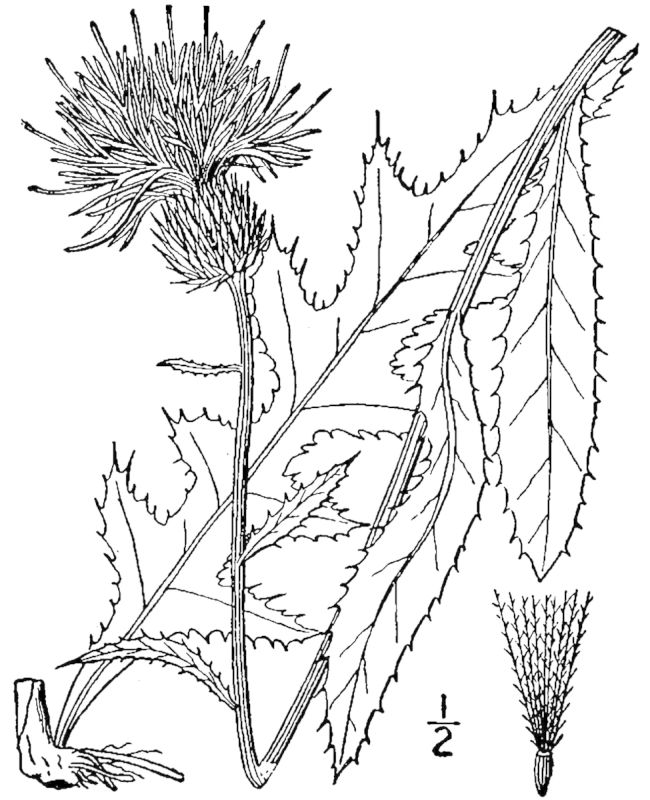
*Cirsium virginianum* (from Britton and Brown 1913).

**Figure 170a.**
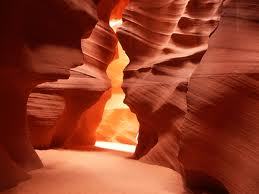
*Coreopsis falcata* (photo by R. Thornhill).

**Figure 170b.**
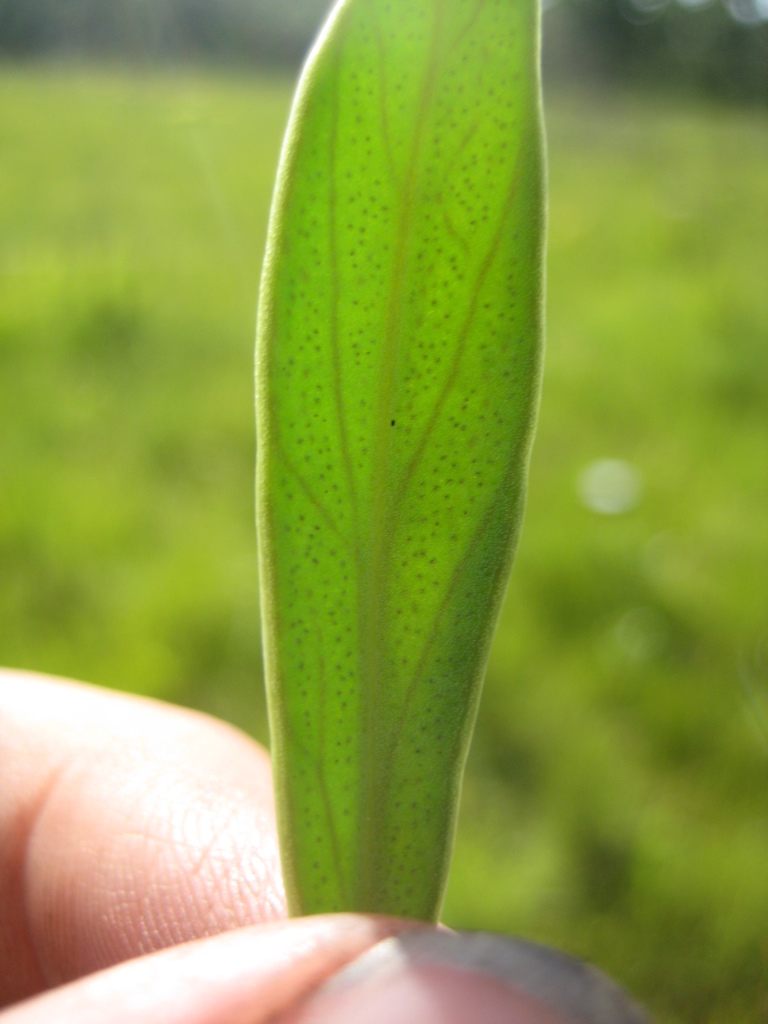
*Coreopsis linifolia*: basal leaf. When backlit, the distinctive combination of pinnate venation and black dots are evident. (Photo by R. Thornhill).

**Figure 170c.**
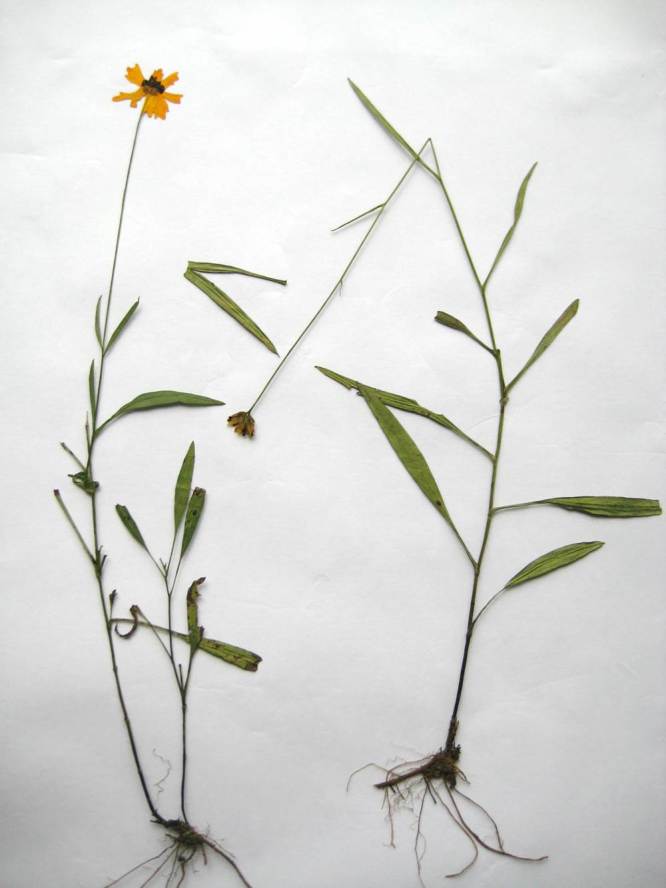
*Coreopsis* species 1 (photo of Thornhill 1171 specimen by R. Thornhill).

**Figure 171a.**
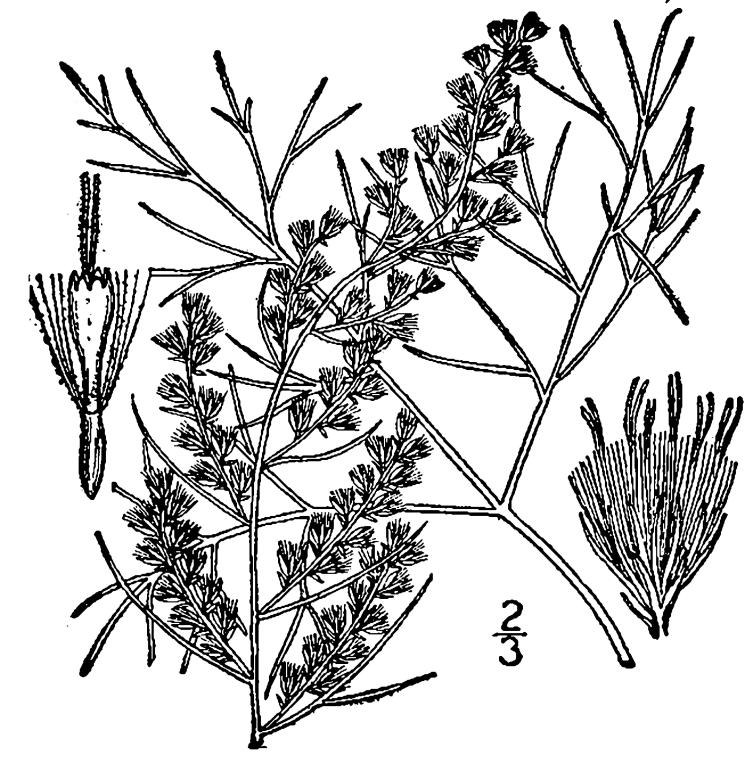
*Eupatorium capillifolium* (from Britton and Brown 1913).

**Figure 171b.**
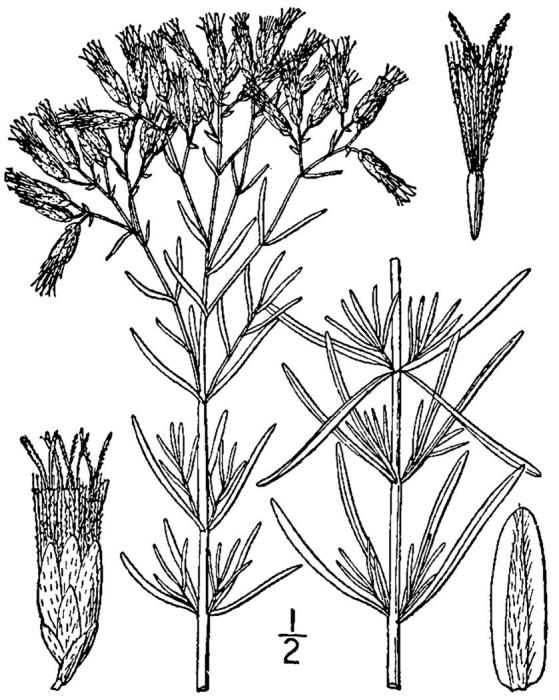
*Eupatorium hyssopifolium* (from Britton and Brown 1913).

**Figure 171c.**
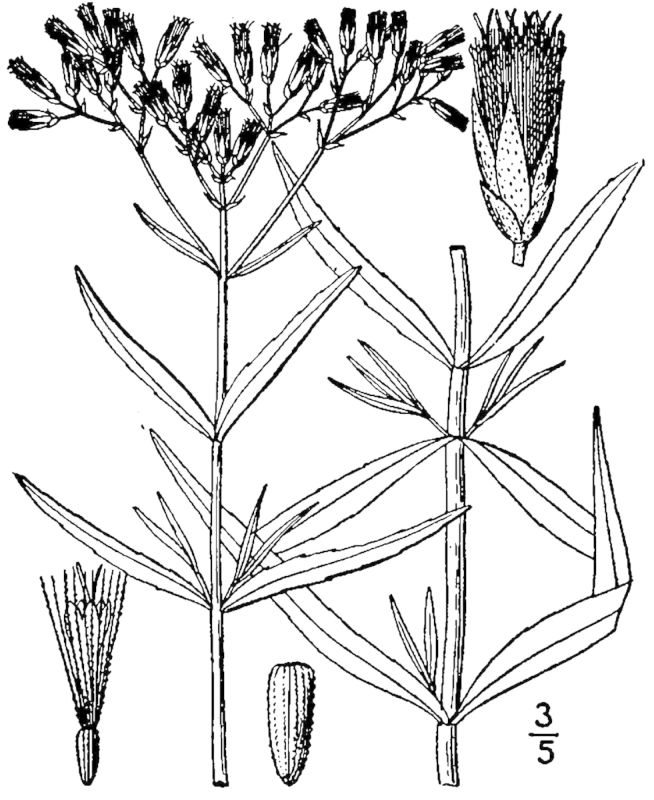
*Eupatorium leucolepis* (from Britton and Brown 1913).

**Figure 171d.**
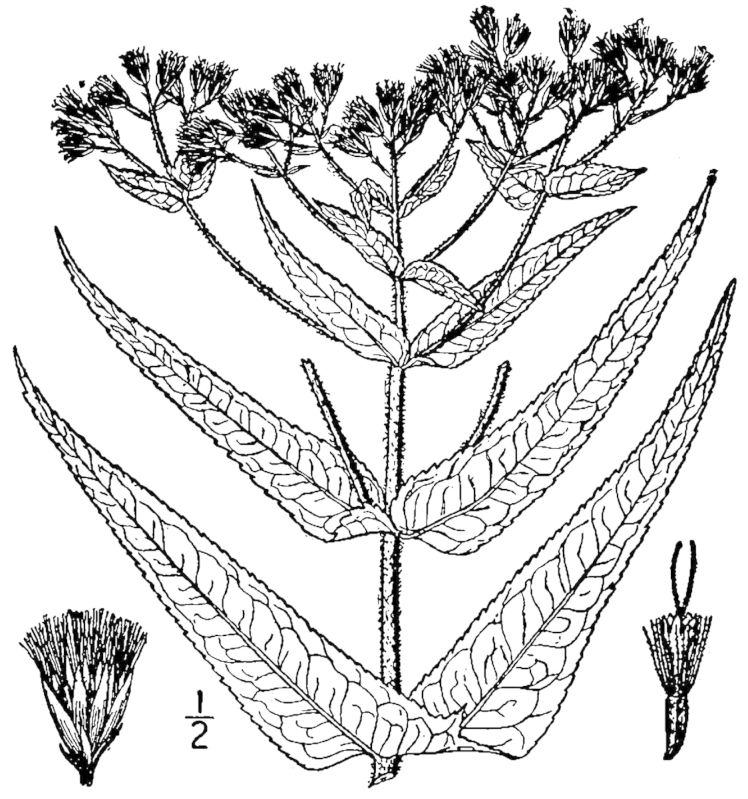
*Eupatorium perfoliatum* (from Britton and Brown 1913).

**Figure 171e.**
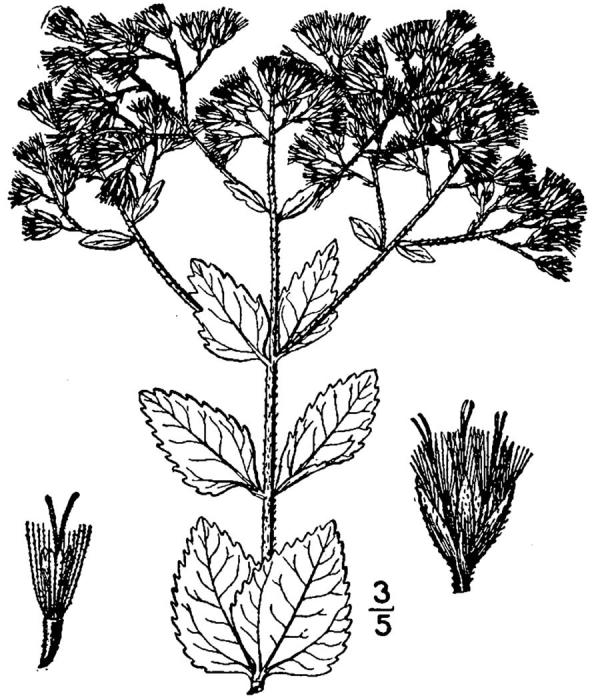
*Eupatorium rotundifolium* (from Britton and Brown 1913).

**Figure 172a.**
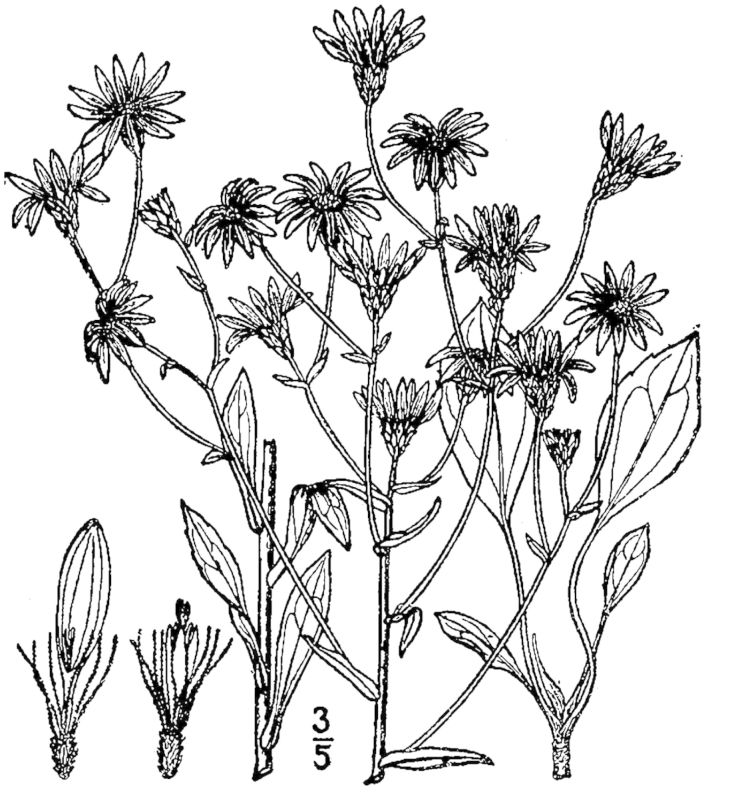
*Eurybia compacta* (from Britton and Brown 1913).

**Figure 172b.**
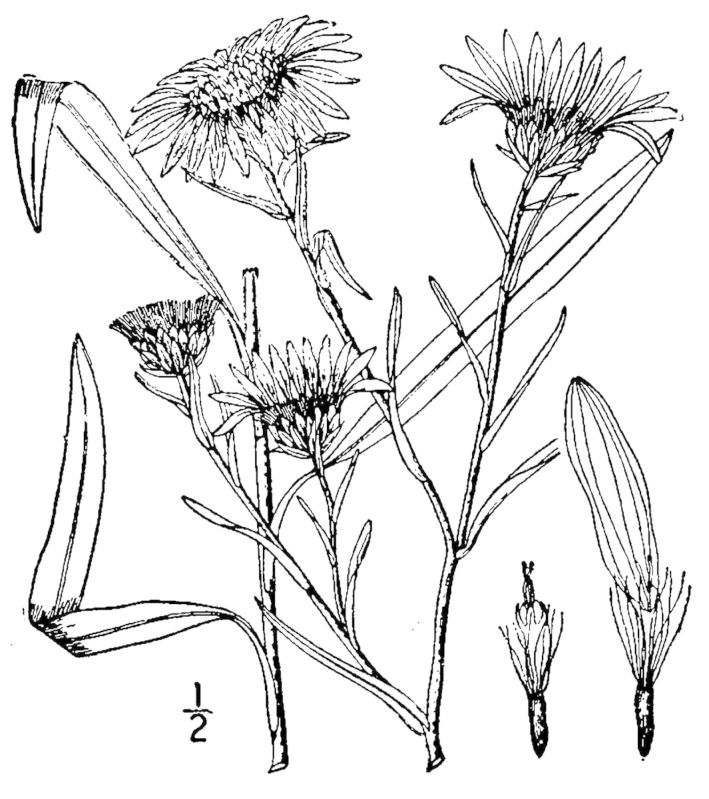
*Eurybia paludosa* (from Britton and Brown 1913).

**Figure 172c.**
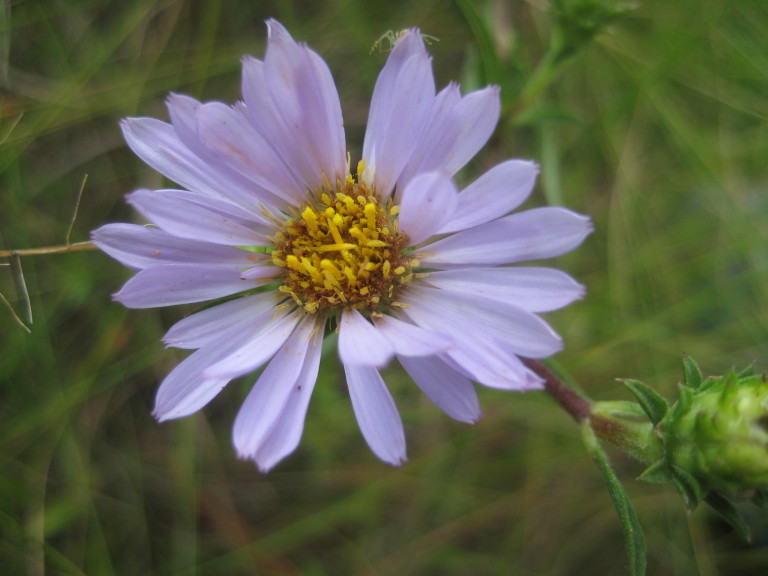
*Eurybia paludosa* (photo by R. Thornhill).

**Figure 173.**
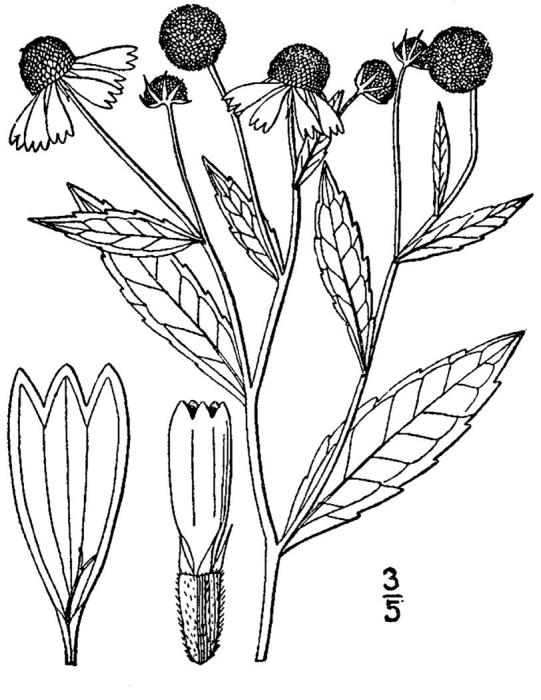
*Helenium autumnale* (from Britton and Brown 1913).

**Figure 174a.**
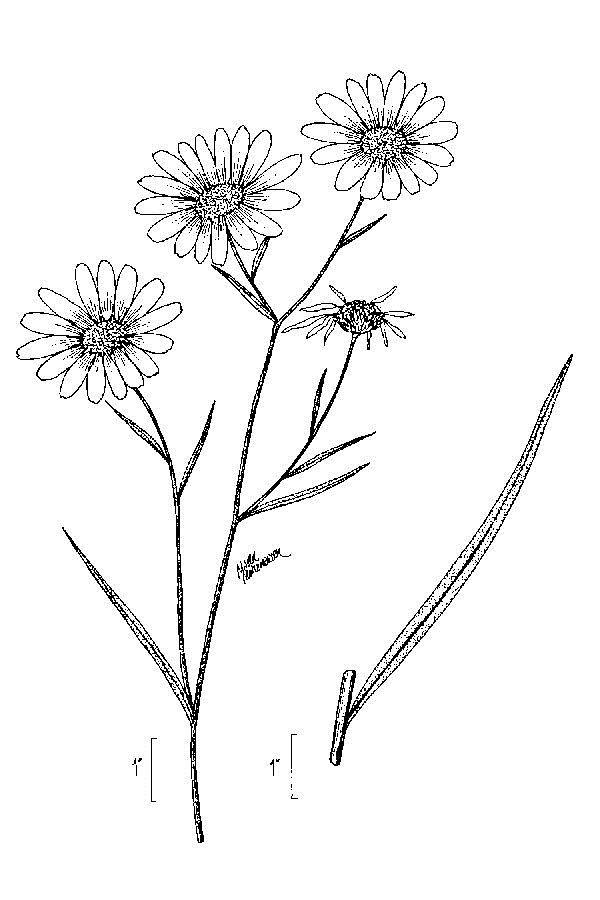
*Helianthus angustifolius* (from USDA-NRCS 2012).

**Figure 174b.**
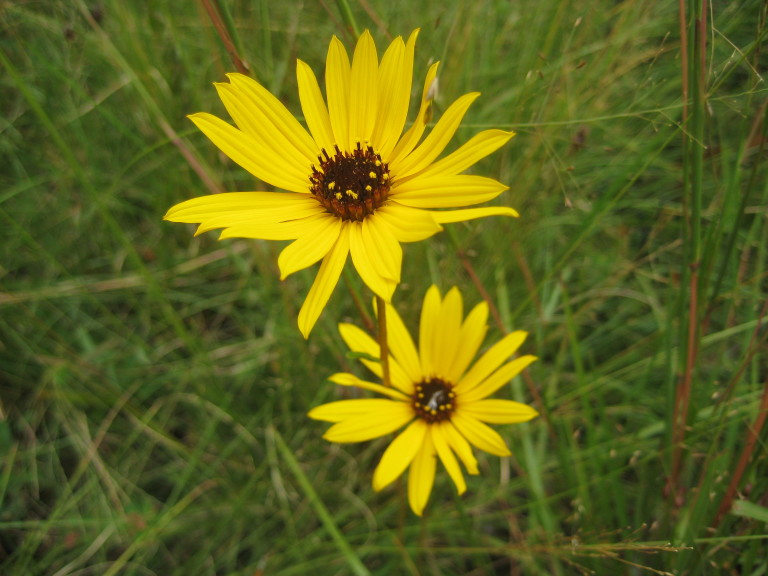
*Helianthus heterophyllus* (photo by R. Thornhill).

**Figure 175a.**
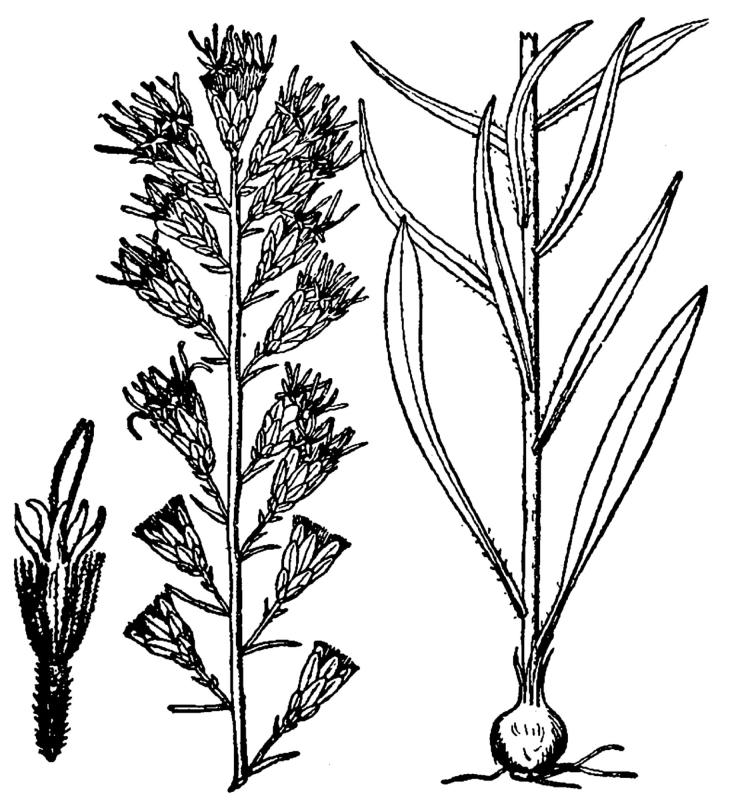
*Ludwigia pilosa* (from Britton and Brown 1913).

**Figure 175b.**
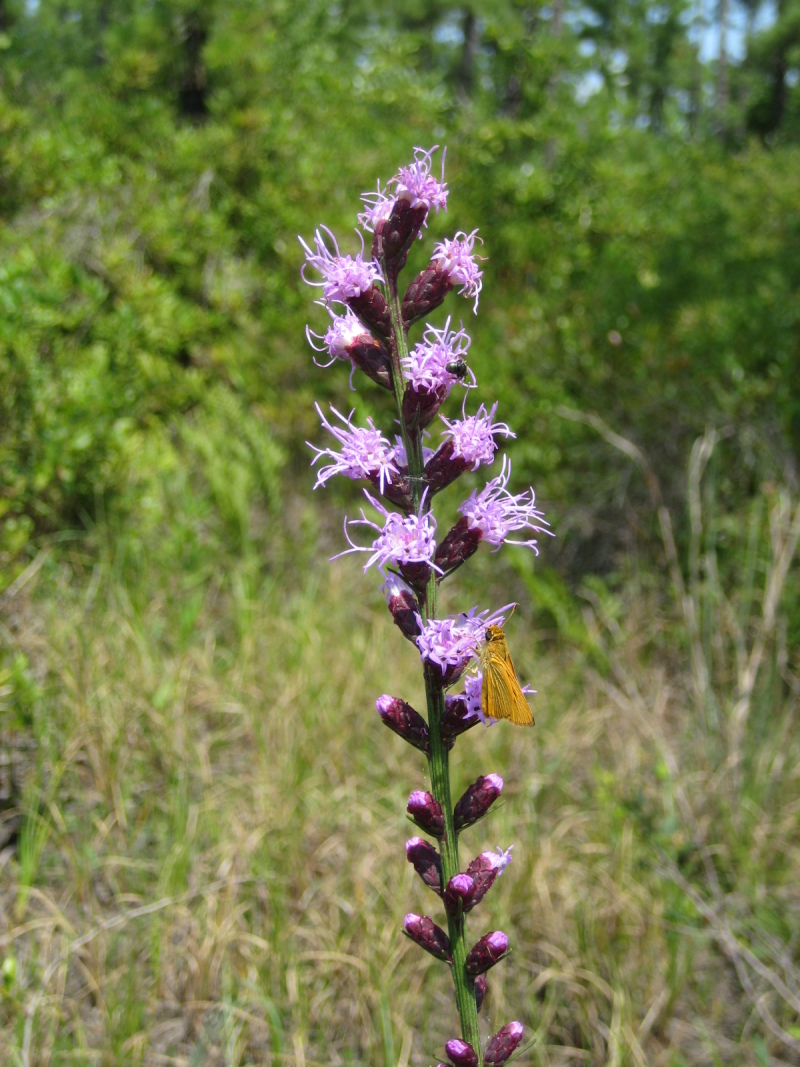
Liatris spicata var. resinosa (photo by R. Thornhill).

**Figure 176.**
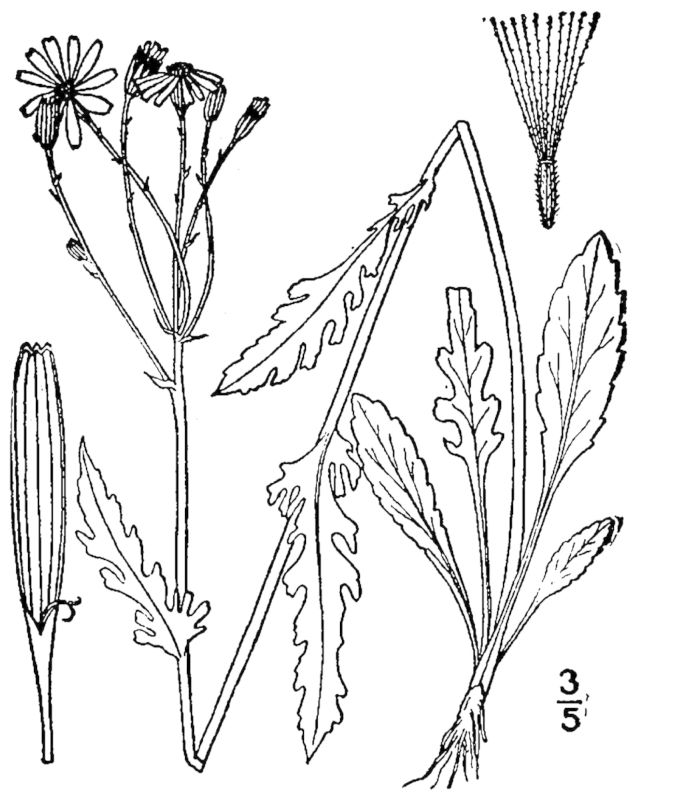
*Packera paupercula* (from Britton and Brown 1913).

**Figure 177a.**
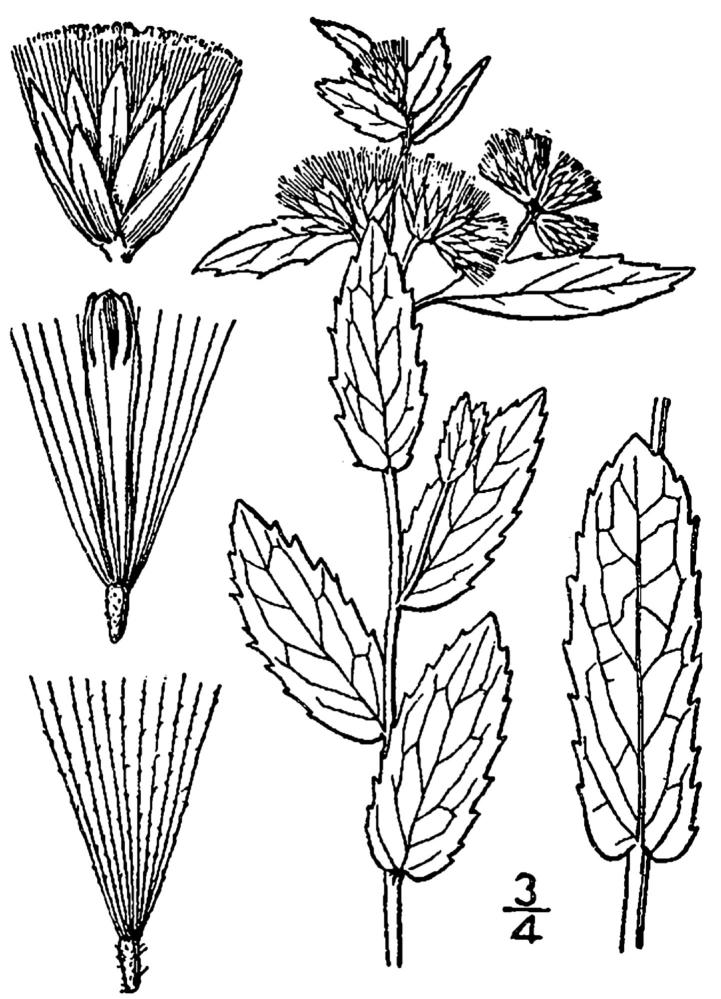
From Britton and Brown (1913).

**Figure 177b.**
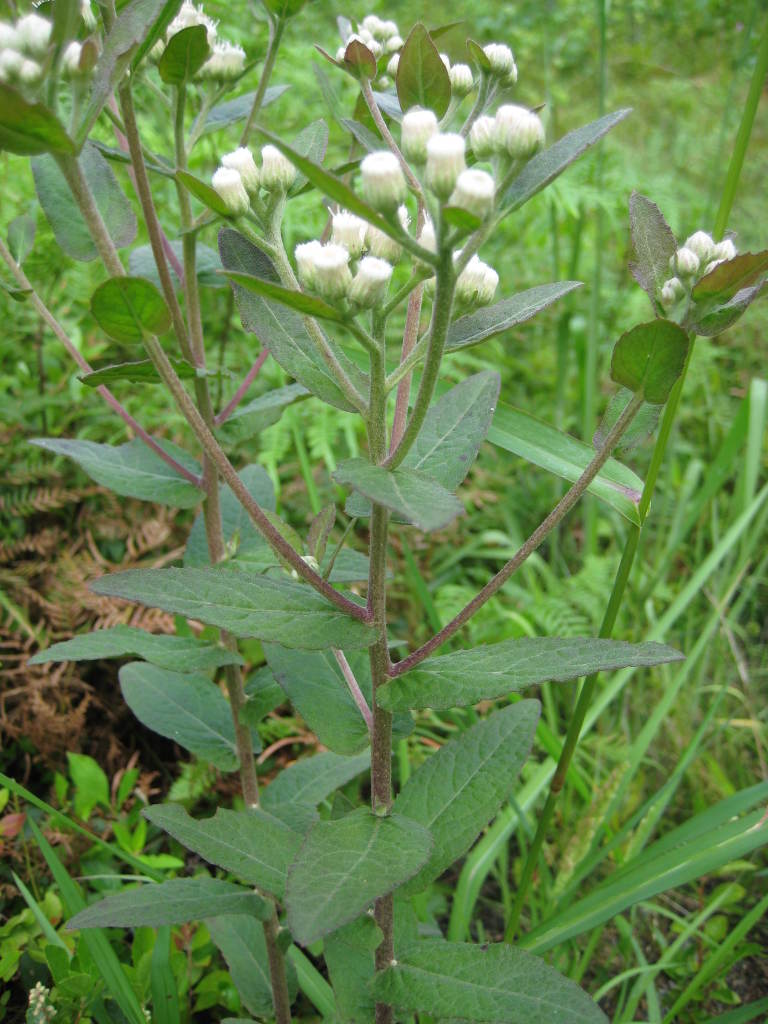
Photo by R. Thornhill.

**Figure 178a.**
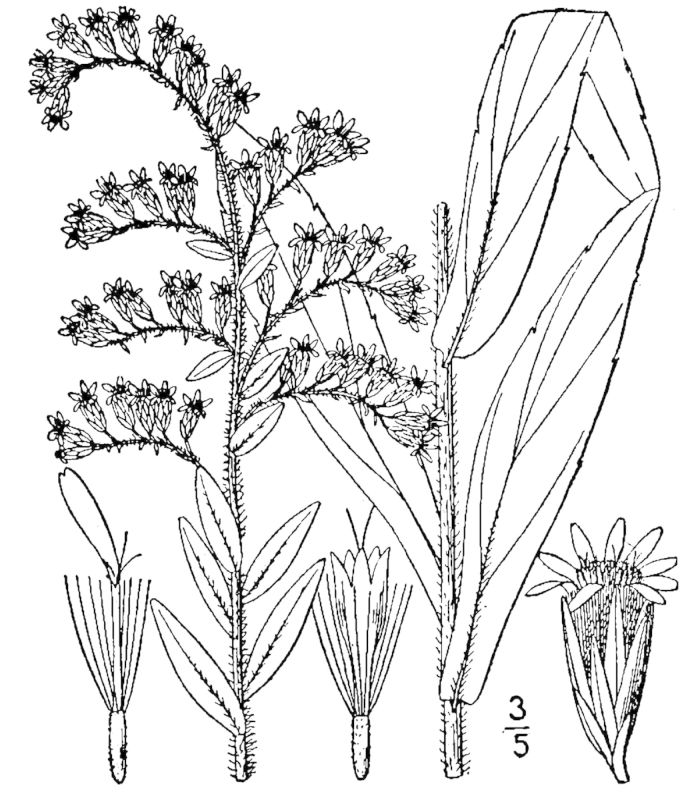
*Solidago fistulosa* (from Britton and Brown 1913).

**Figure 178b.**
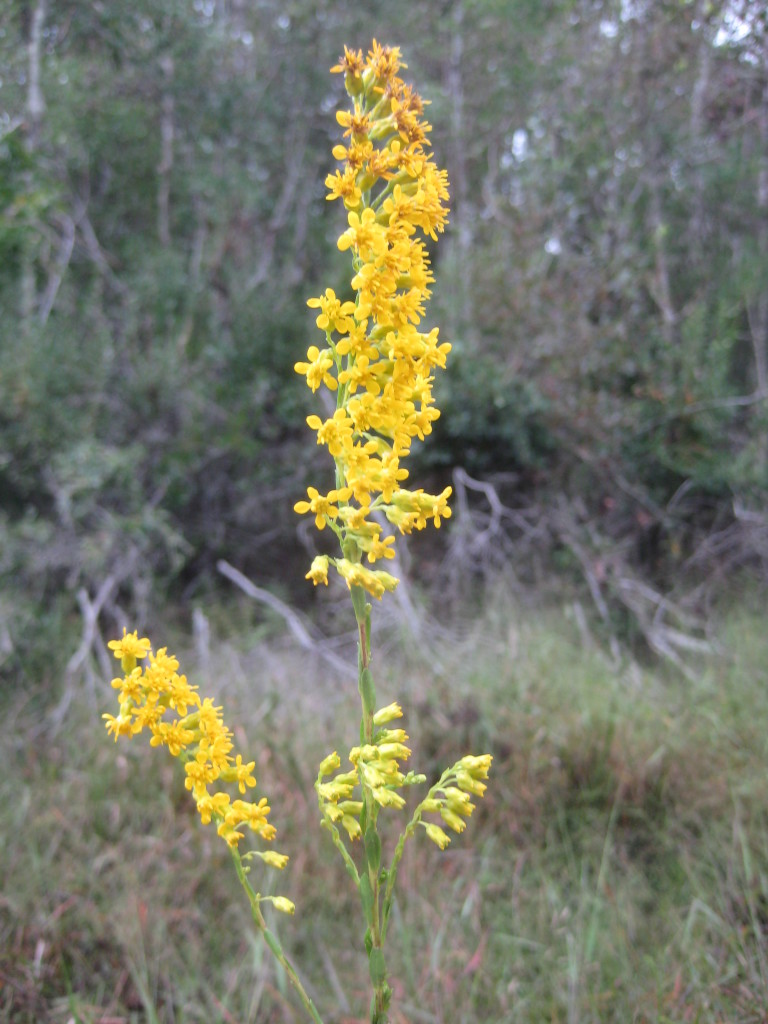
*Solidago gracillima* (photo by R. Thornhill).

**Figure 178c.**
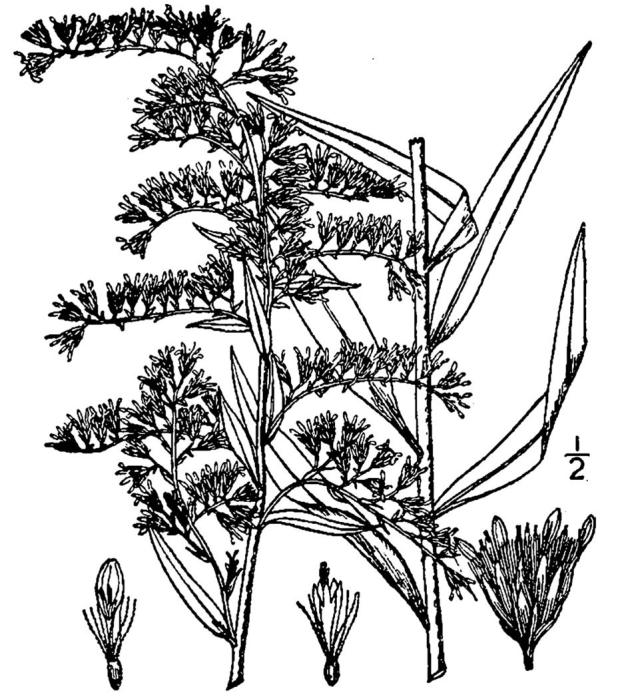
*Solidago odora* (from Britton and Brown 1913).

**Figure 178d.**
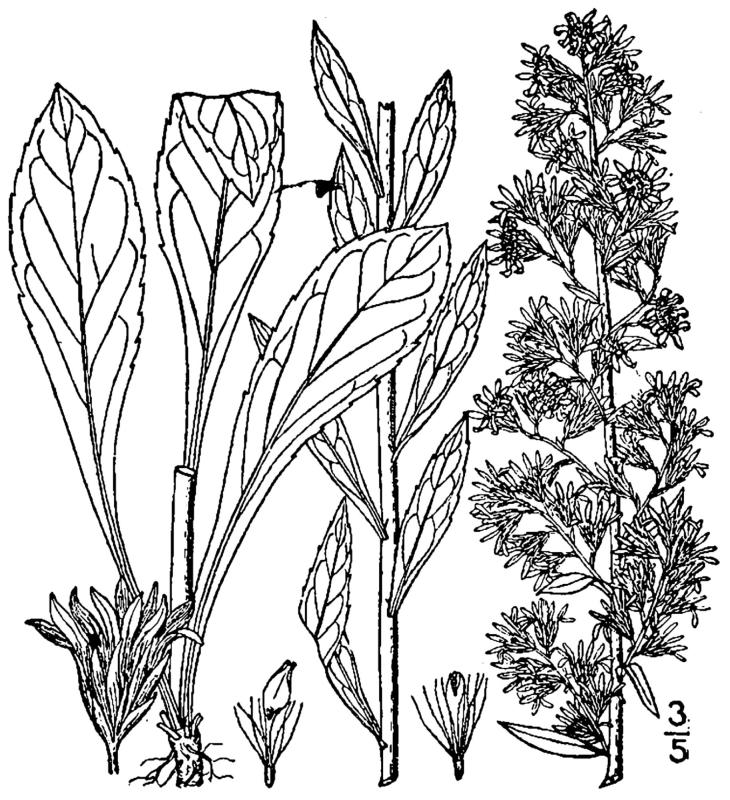
*Solidago puberula* (from Britton and Brown 1913).

**Figure 178e.**
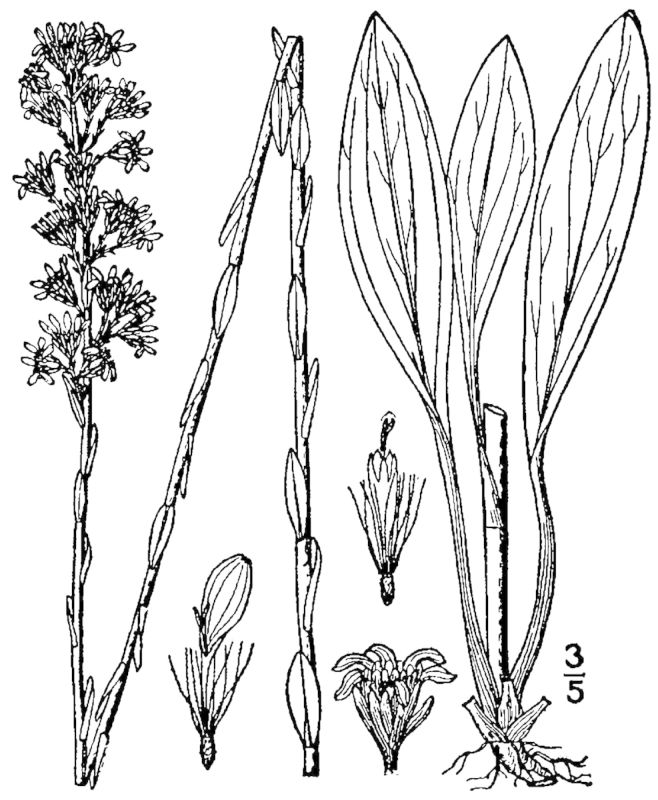
*Solidago stricta* (from Britton and Brown 1913).

**Figure 179a.**
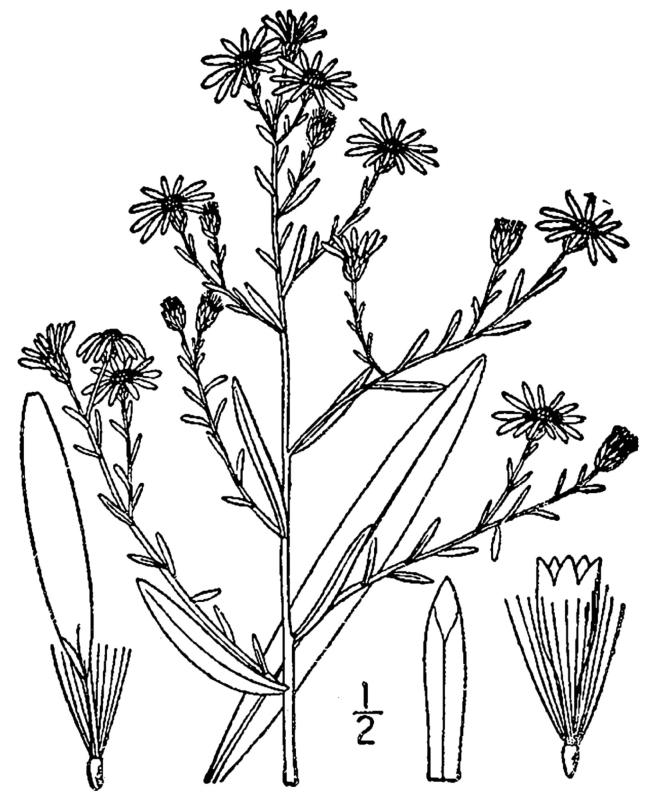
*Symphyotrichum dumosum* (from Britton and Brown 1913).

**Figure 179b.**
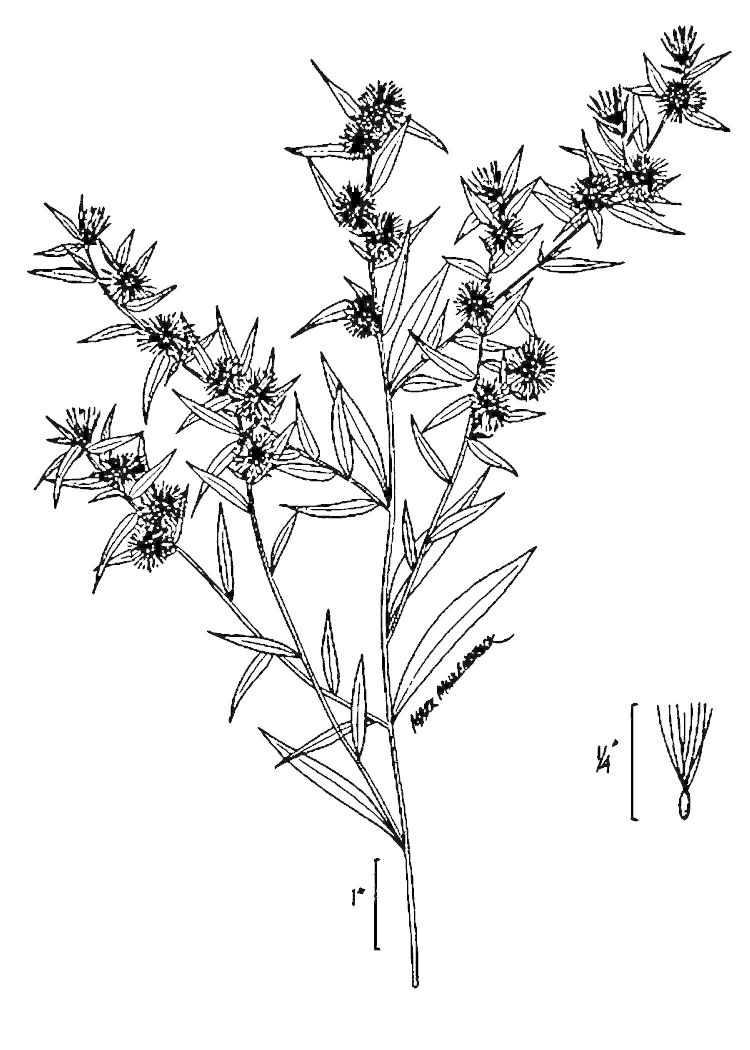
*Symphyotrichum lateriflorum* (from USDA-NRCS 2012).

**Figure 179c.**
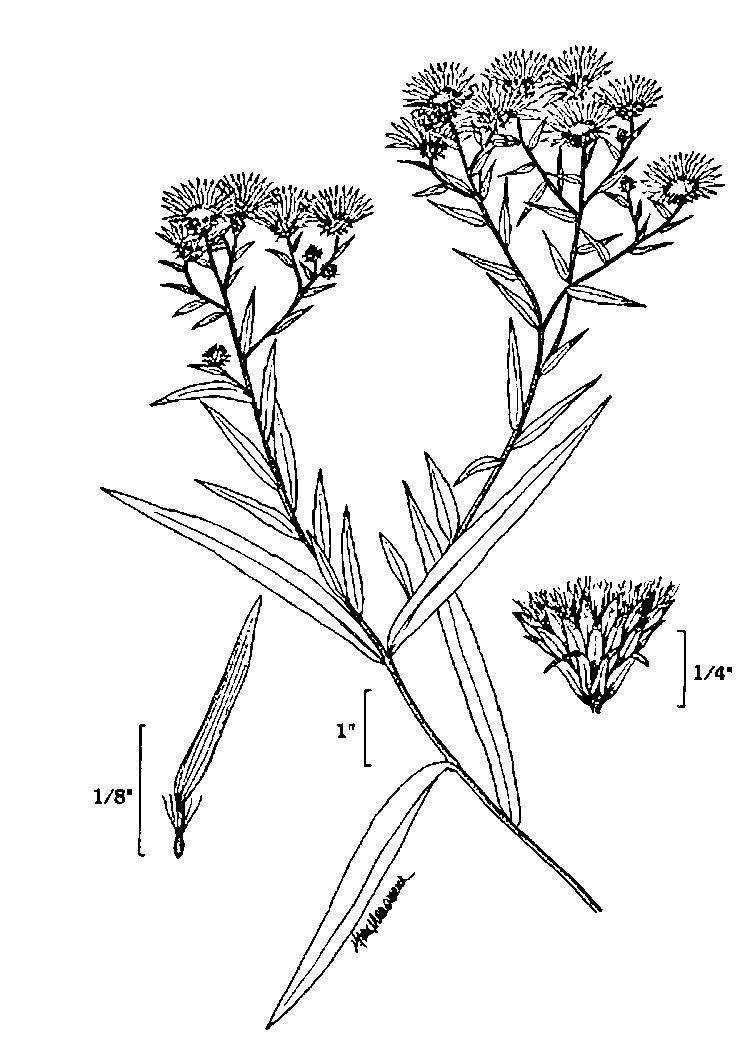
*Symphyotrichum novi-belgii* (from USDA-NRCS 2012).

**Figure 179d.**
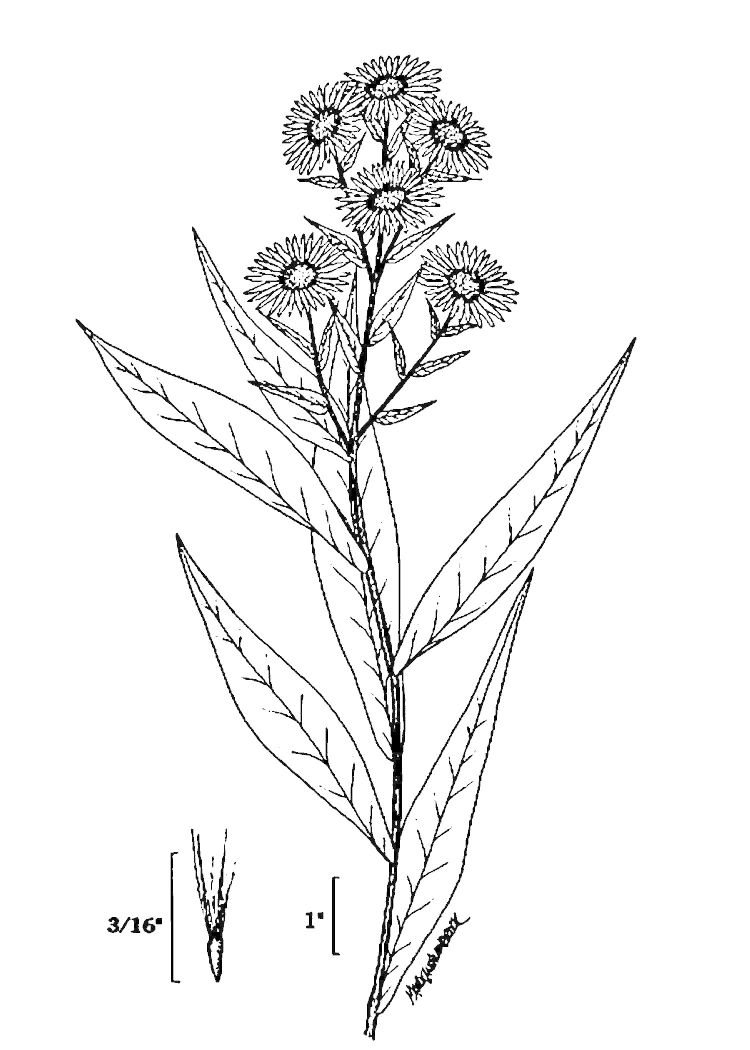
*Symphyotrichum pilosum* (from USDA-NRCS 2012).

**Figure 180a.**
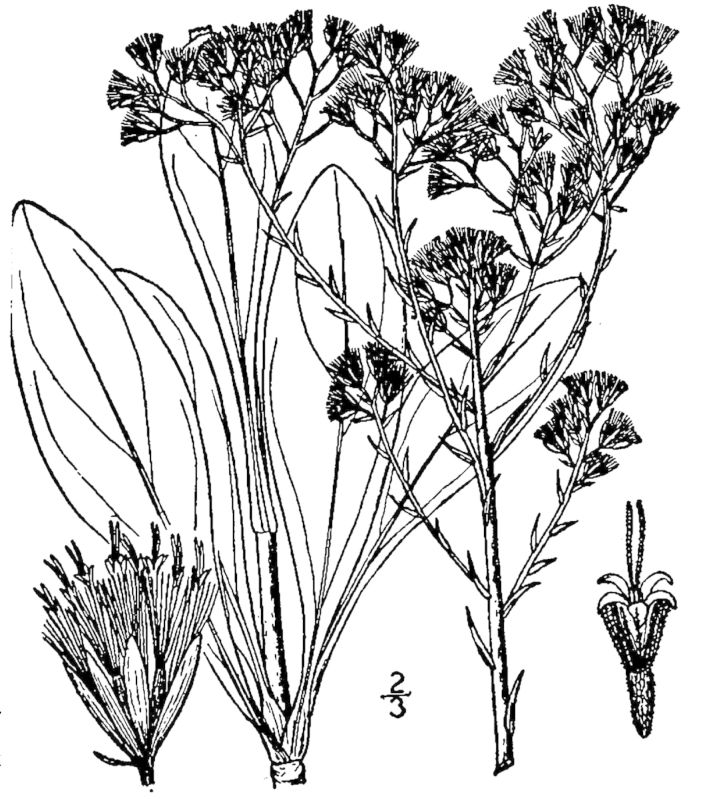
*Trilisa odoratissima* (from Britton and Brown 1913).

**Figure 180b.**
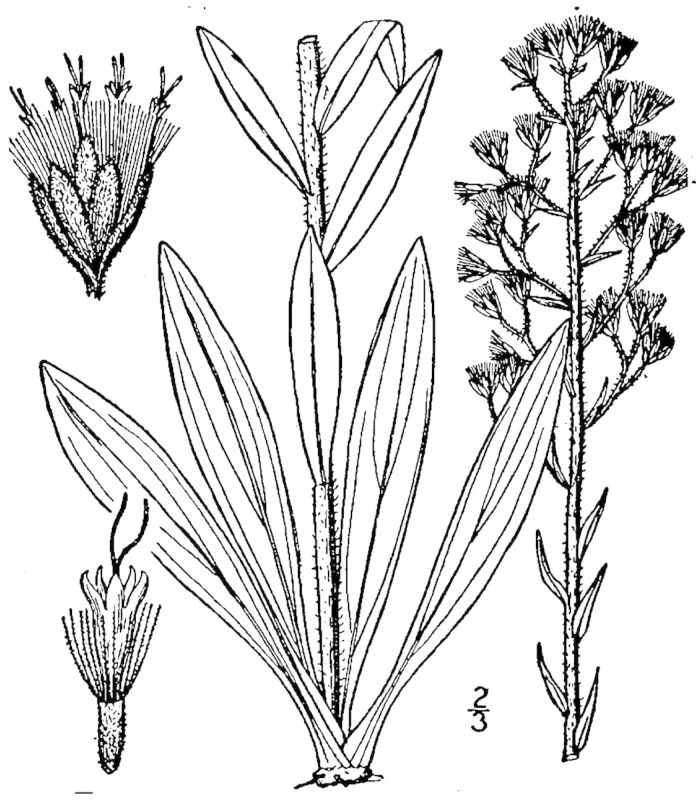
*Trilisa paniculata* (from Britton and Brown 1913).

**Figure 181.**
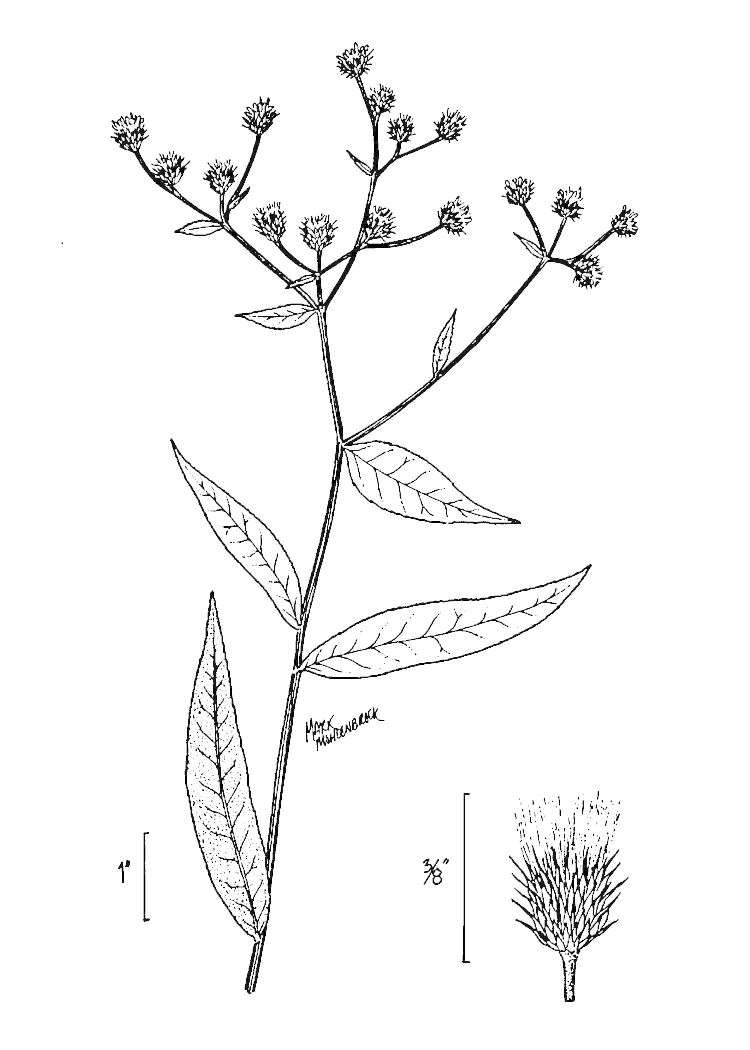
*Vernonia noveboracensis* (from USDA-NRCS 2012).

**Figure 182.**
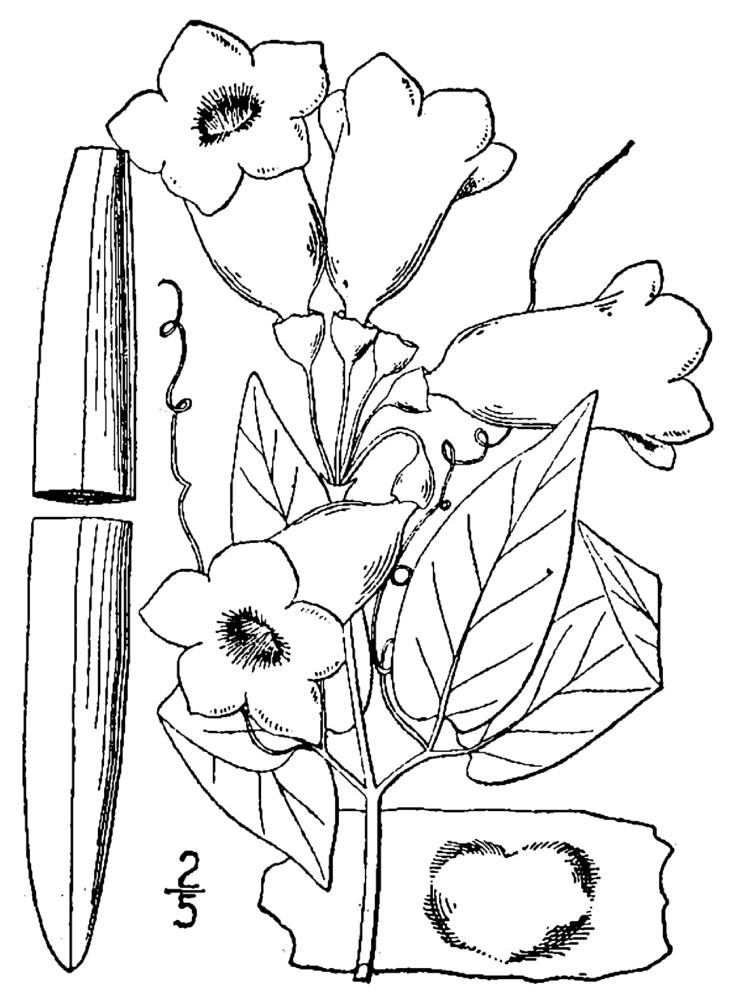
*Bignonia capreolata* (from Britton and Brown 1913).

**Figure 183.**
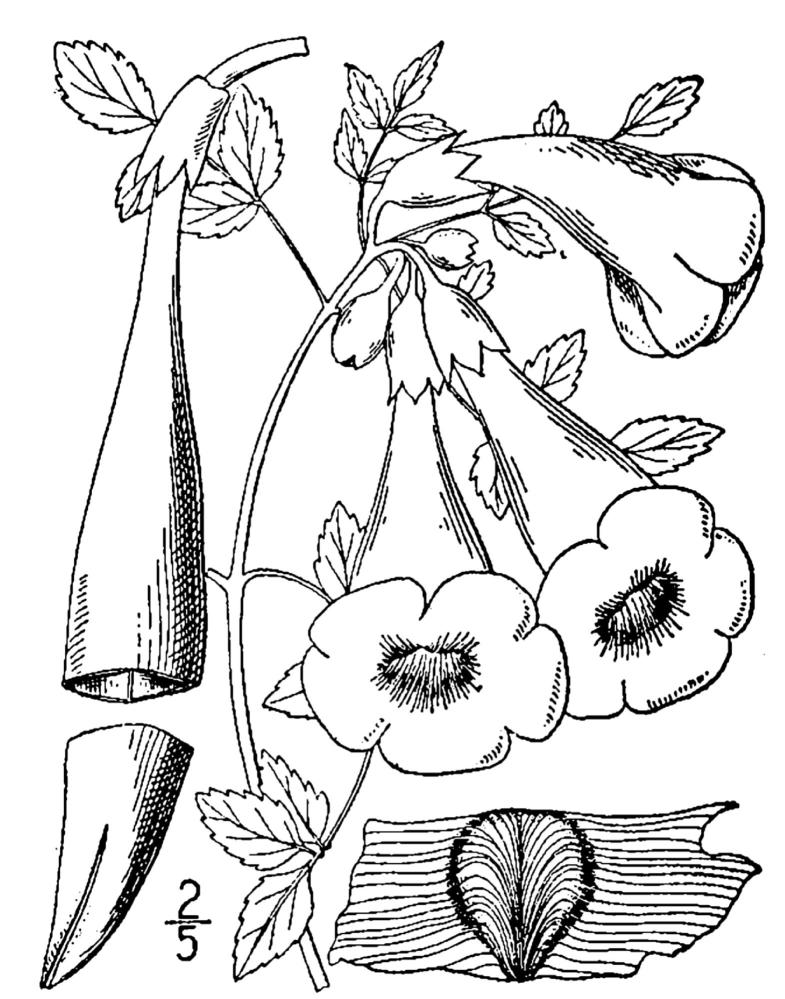
*Campsis radicans* (from Britton and Brown 1913).

**Figure 184a.**
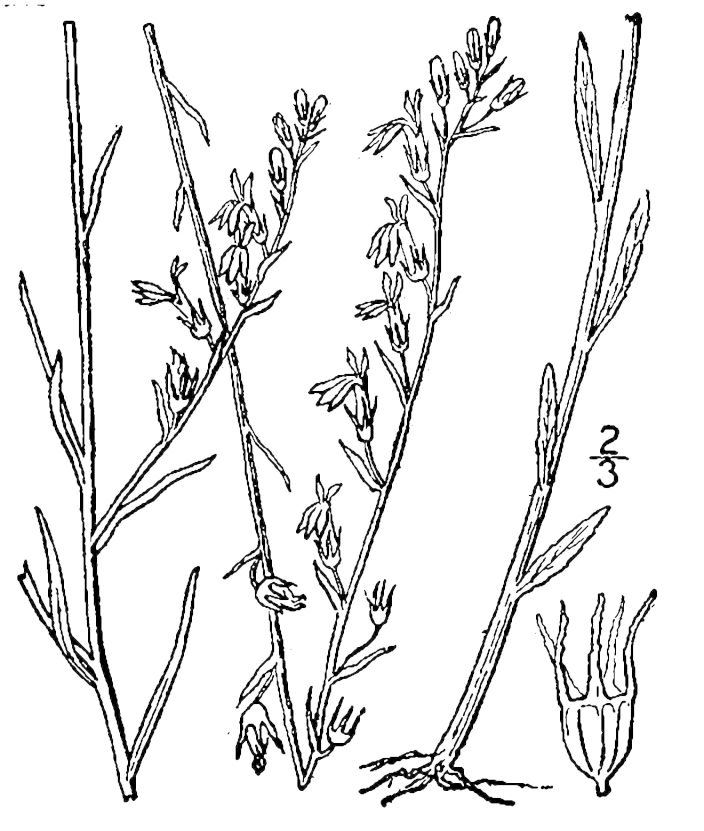
*Lobelia canbyi* (from Britton and Brown 1913).

**Figure 184b.**
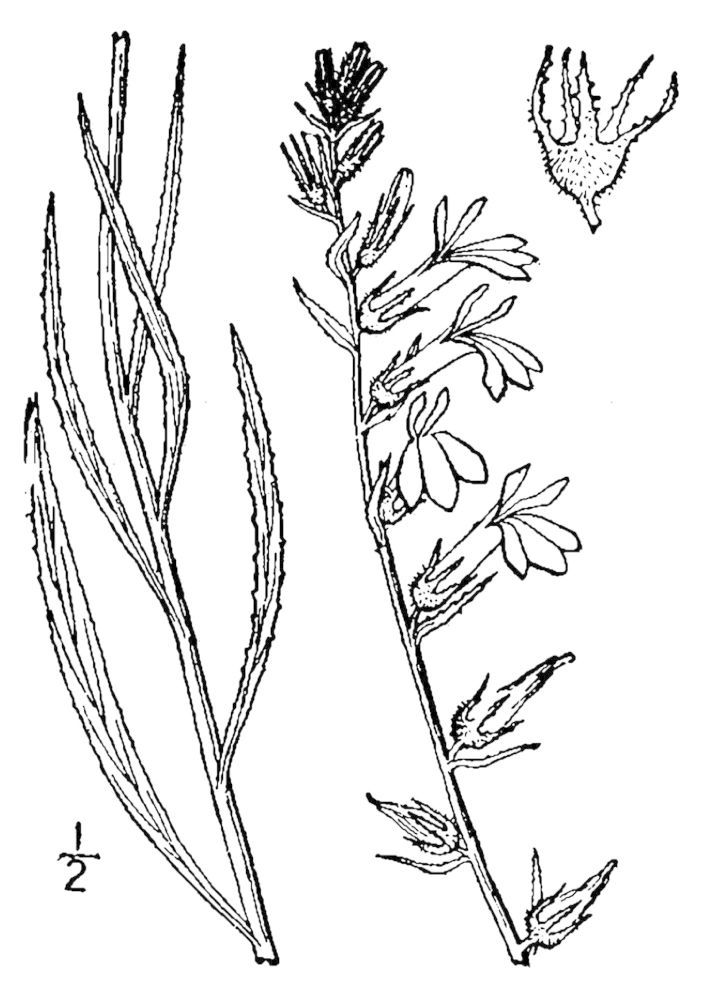
*Lobelia glandulosa* (from Britton and Brown 1913).

**Figure 184c.**
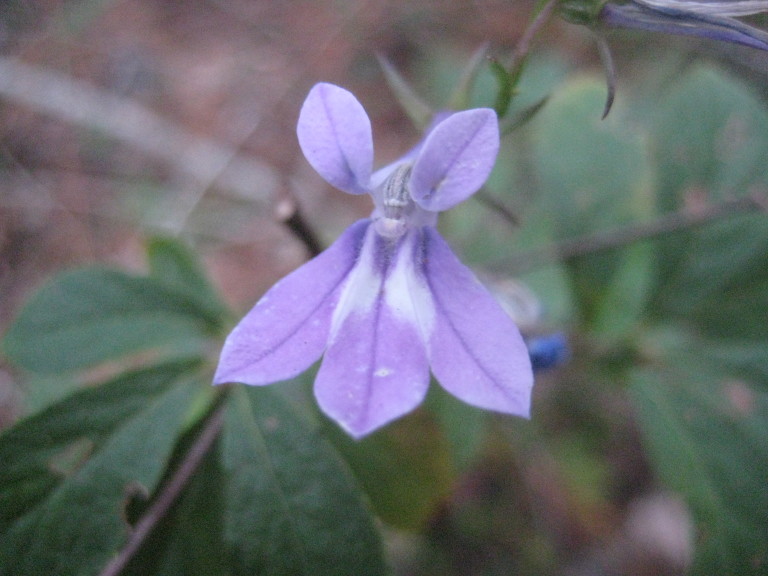
*Lobelia glandulosa* (photo by R. Thornhill).

**Figure 184d.**
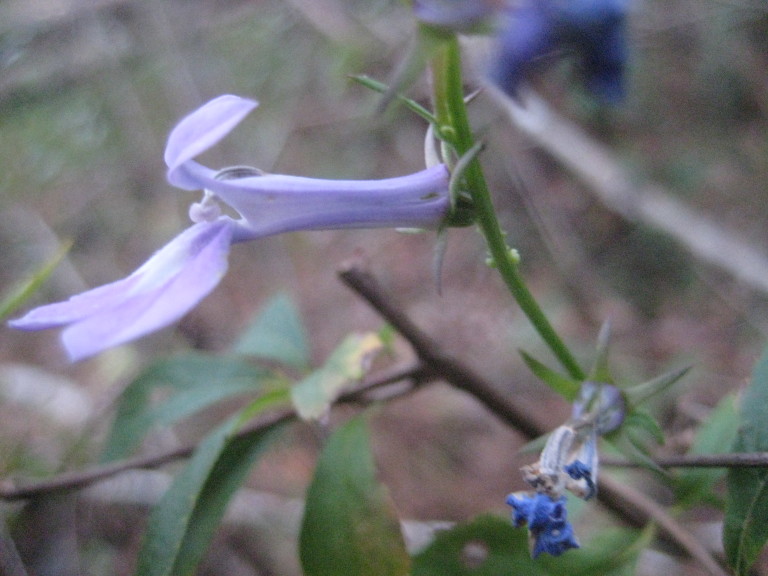
*Lobelia glandulosa*: note the "fenestrate" corolla (i.e., the narrow, slit-like opening at base of corolla tube; photo by R. Thornhill).

**Figure 184e.**
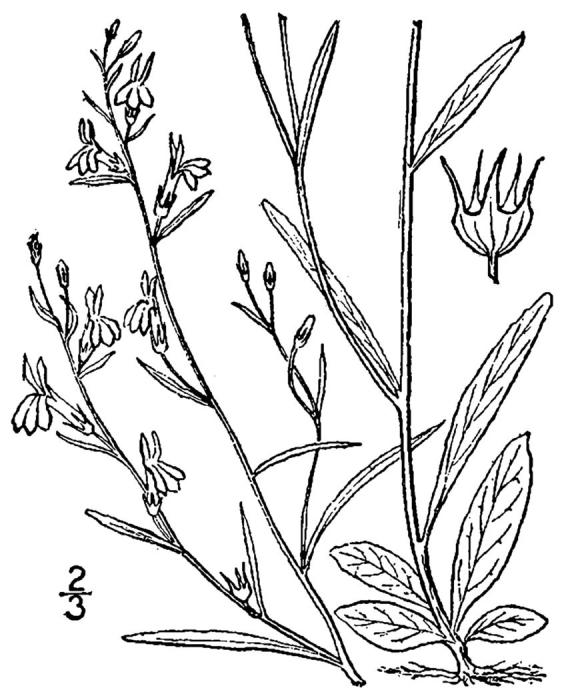
*Lobelia nuttallii* (from Britton and Brown 1913).

**Figure 184f.**
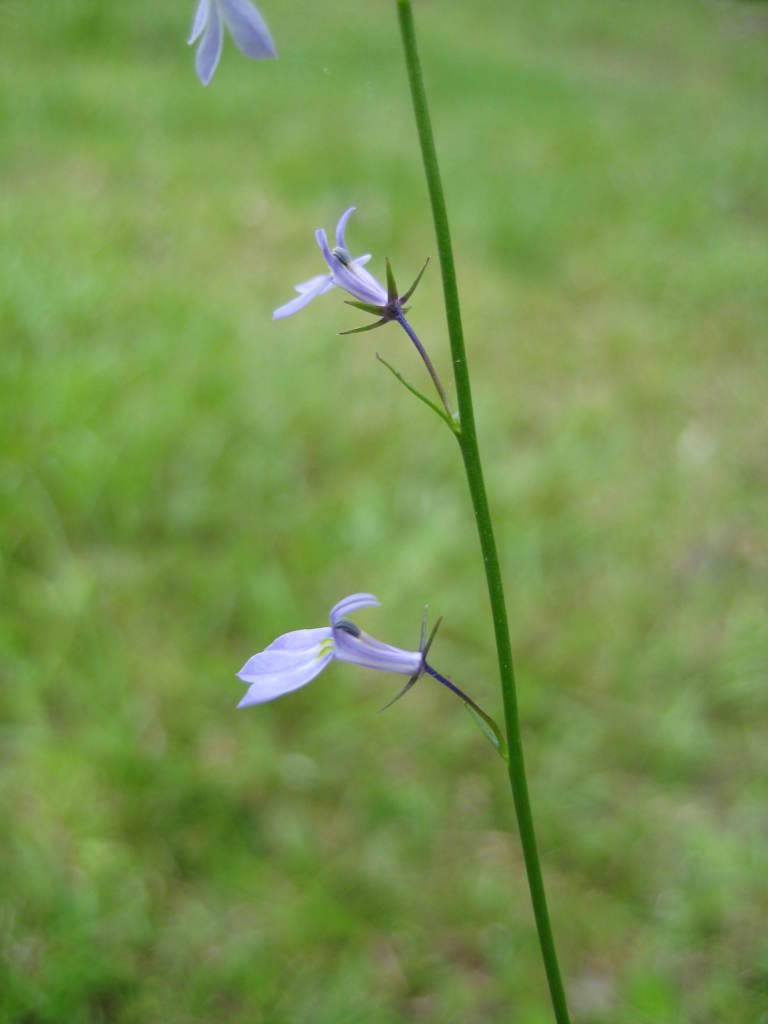
*Lobelia nuttallii* (photo by R. Thornhill).

**Figure 185a.**
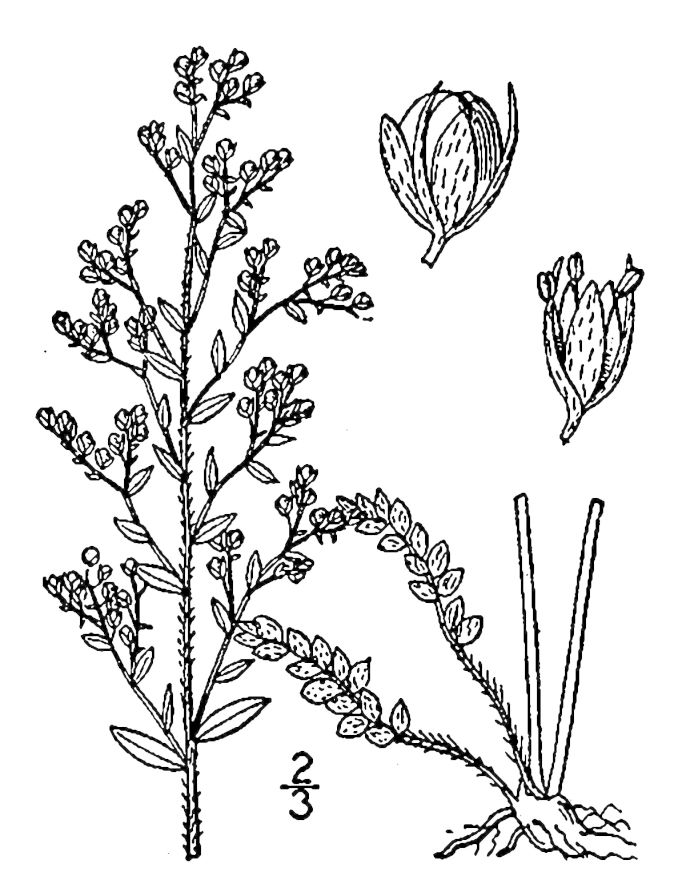
*Lechea minor* (from Britton and Brown 1913).

**Figure 185b.**
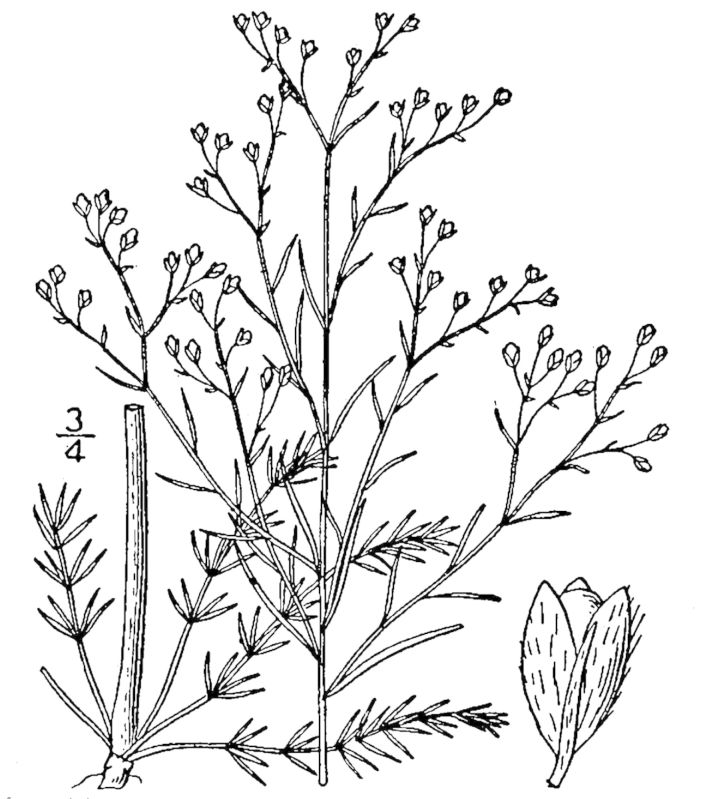
*Lechea pulchella* (from Britton and Brown 1913).

**Figure 186a.**
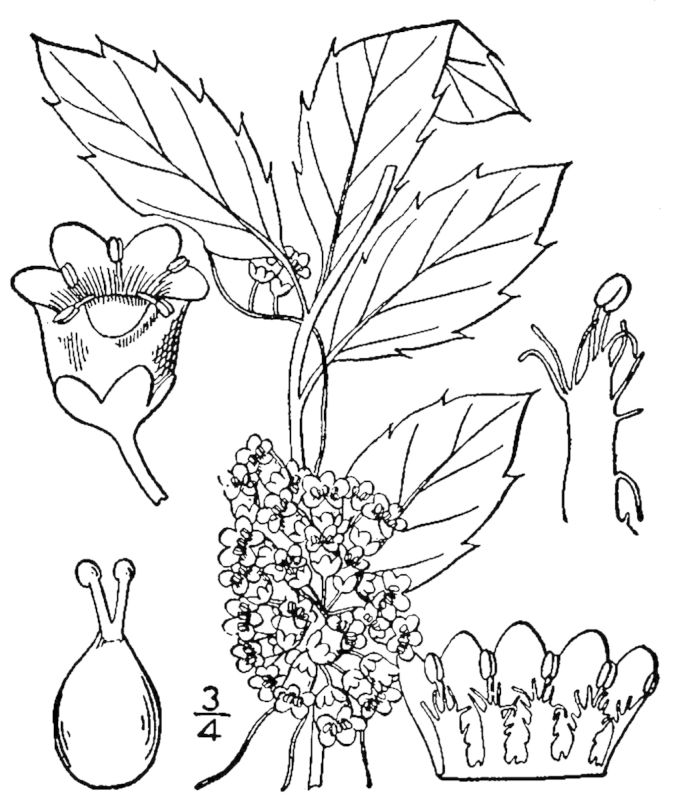
*Cuscuta gronovii* (from Britton and Brown 1913).

**Figure 186b.**
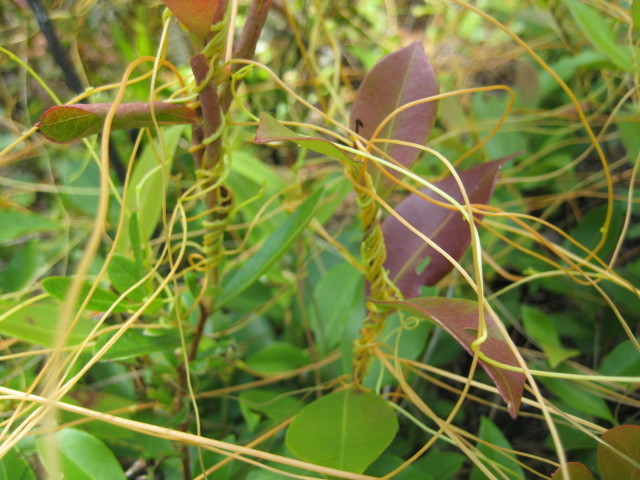
*Cuscuta* species: note the distinctive orange stems (photo by R. Thornhill).

**Figure 187a.**
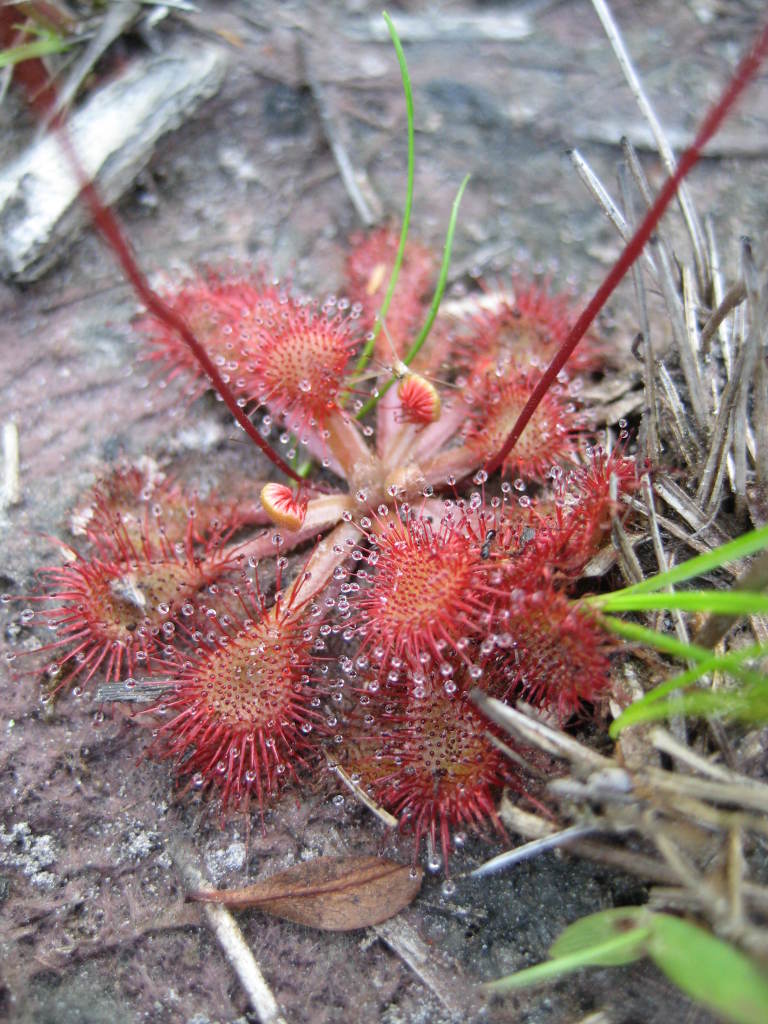
*Drosera capillaris* (photo by R. Thornhill).

**Figure 187b.**
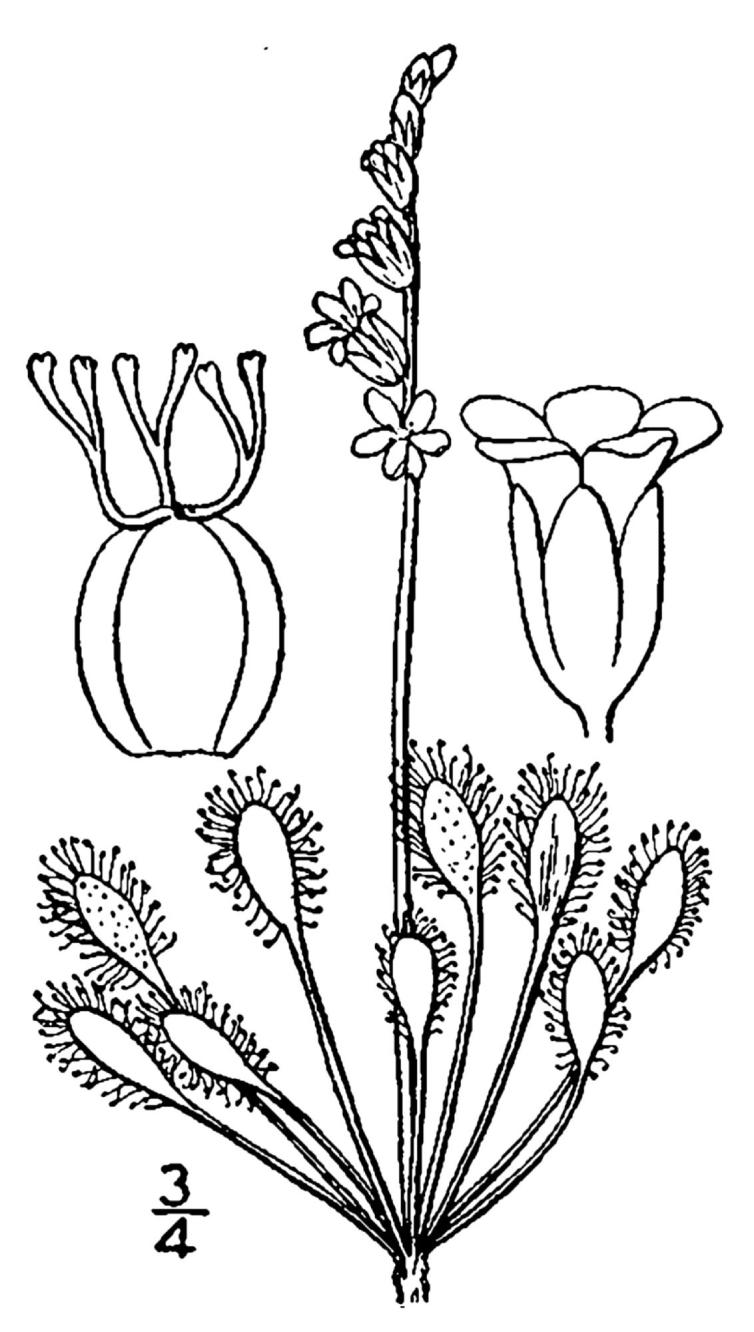
*Drosera intermedia* (from Britton and Brown 1913).

**Figure 187c.**
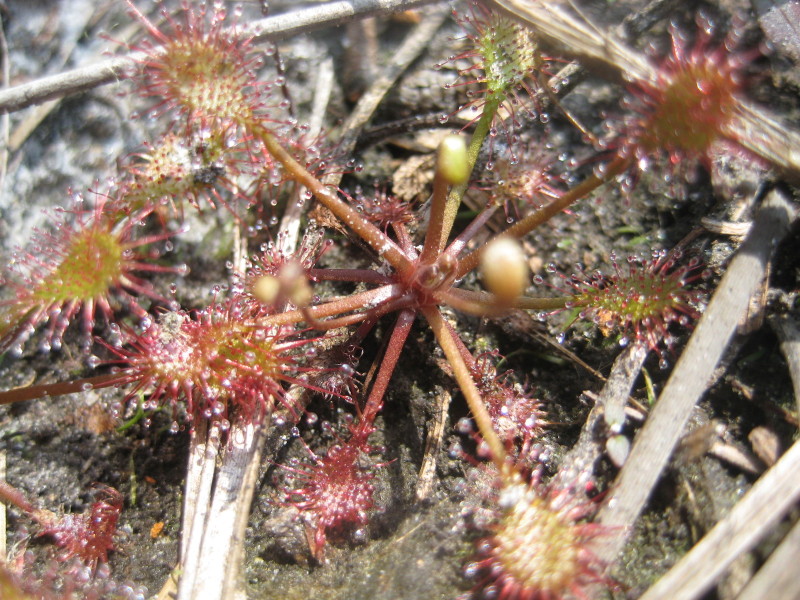
*Drosera intermedia* (photo by R. Thornhill).

**Figure 188a.**
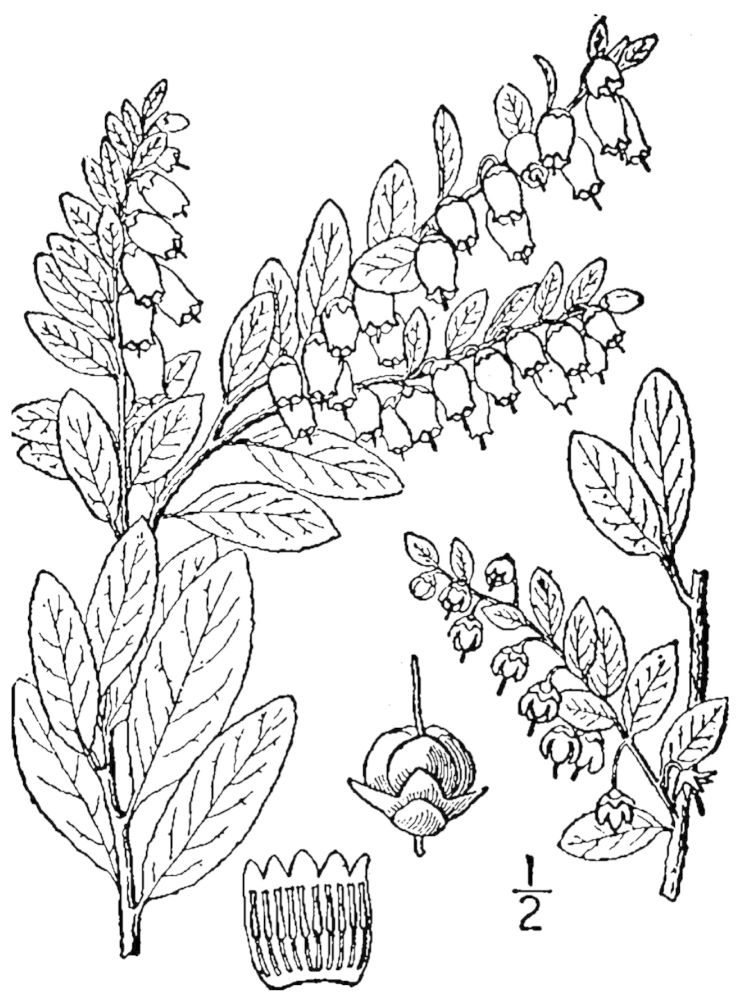
From Britton and Brown (1913).

**Figure 188b.**
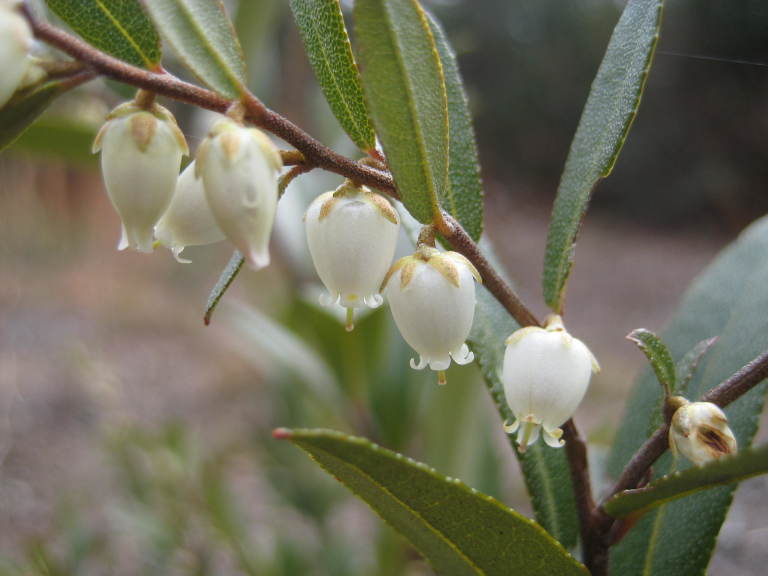
Photo by R. Thornhill.

**Figure 189.**
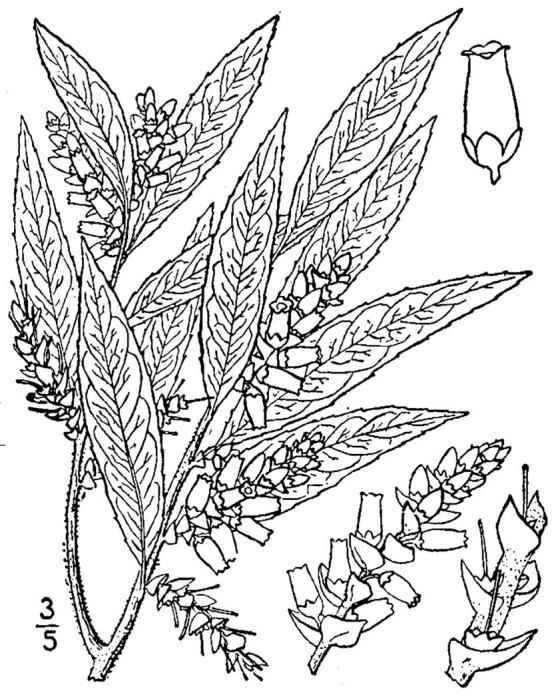
*Leucothoe axillaris* (from Britton and Brown 1913).

**Figure 190a.**
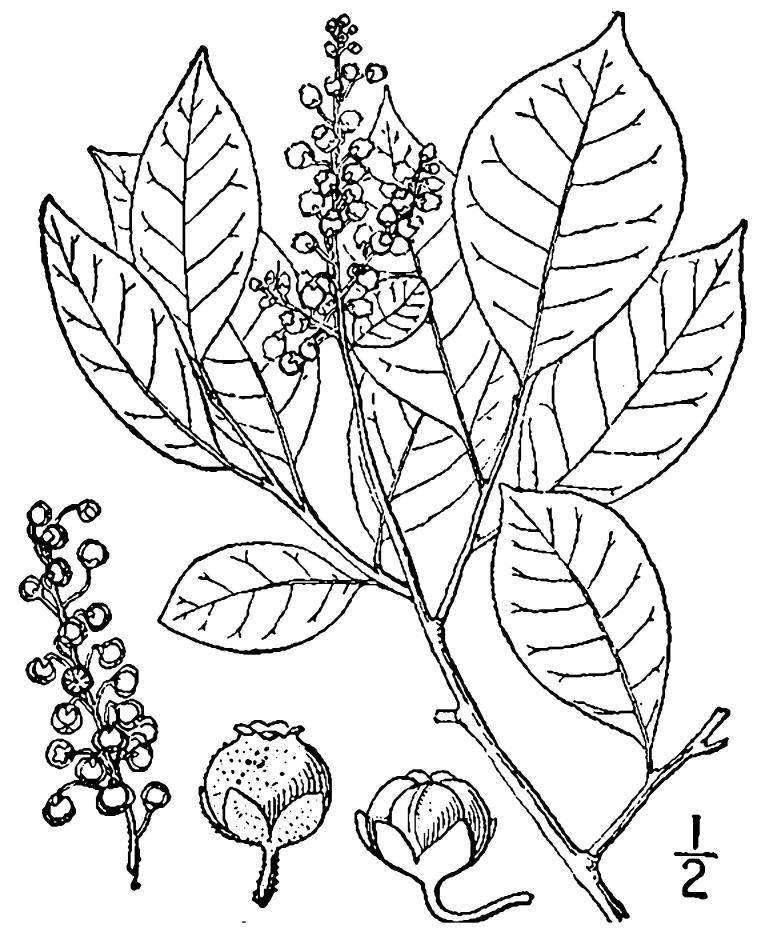
*Lyonia ligustrina* (from Britton and Brown 1913).

**Figure 190b.**
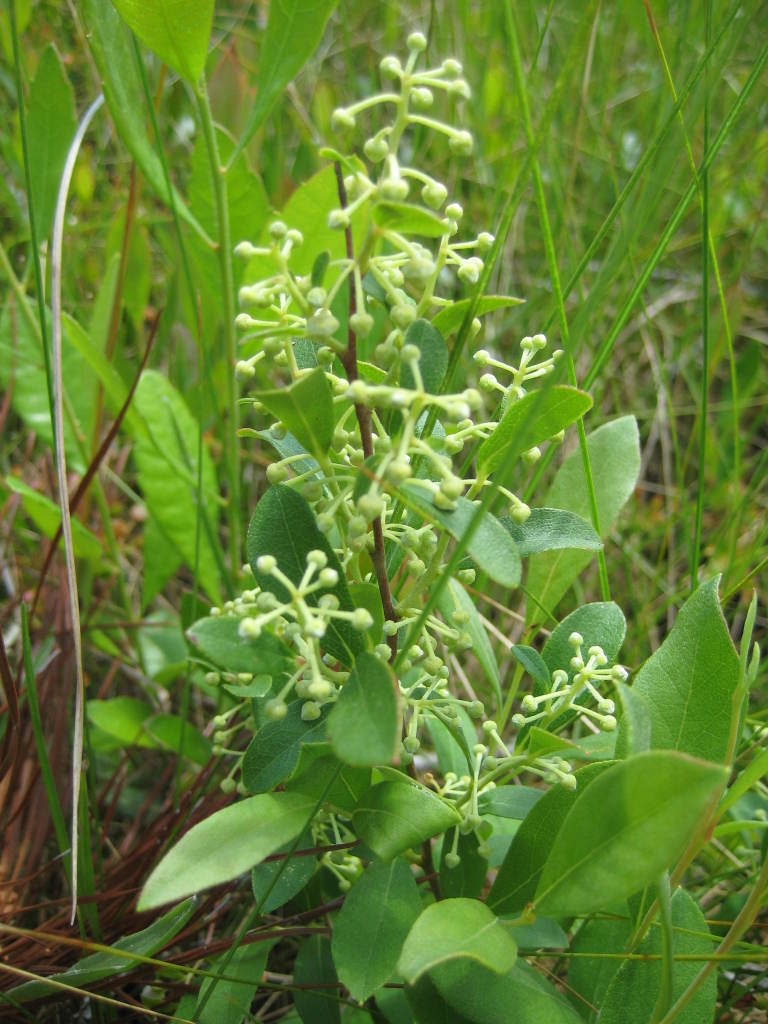
*Lyonia ligustrina* (photo by R. Thornhill).

**Figure 190c.**
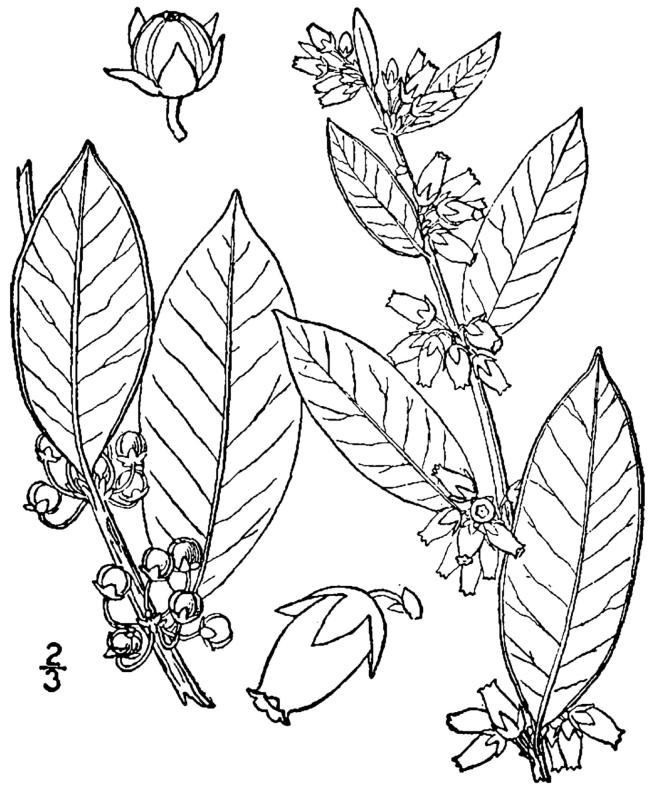
*Lyonia lucida* (from Britton and Brown 1913).

**Figure 190d.**
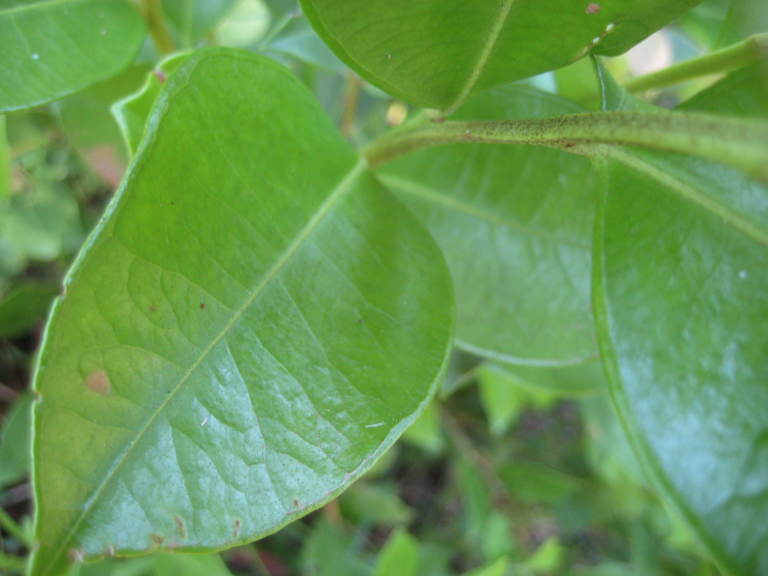
*Lyonia lucida*: note the glossy leaf surface and the presence of a perimarginal vein just inside the leaf margin (photo by R. Thornhill).

**Figure 190e.**
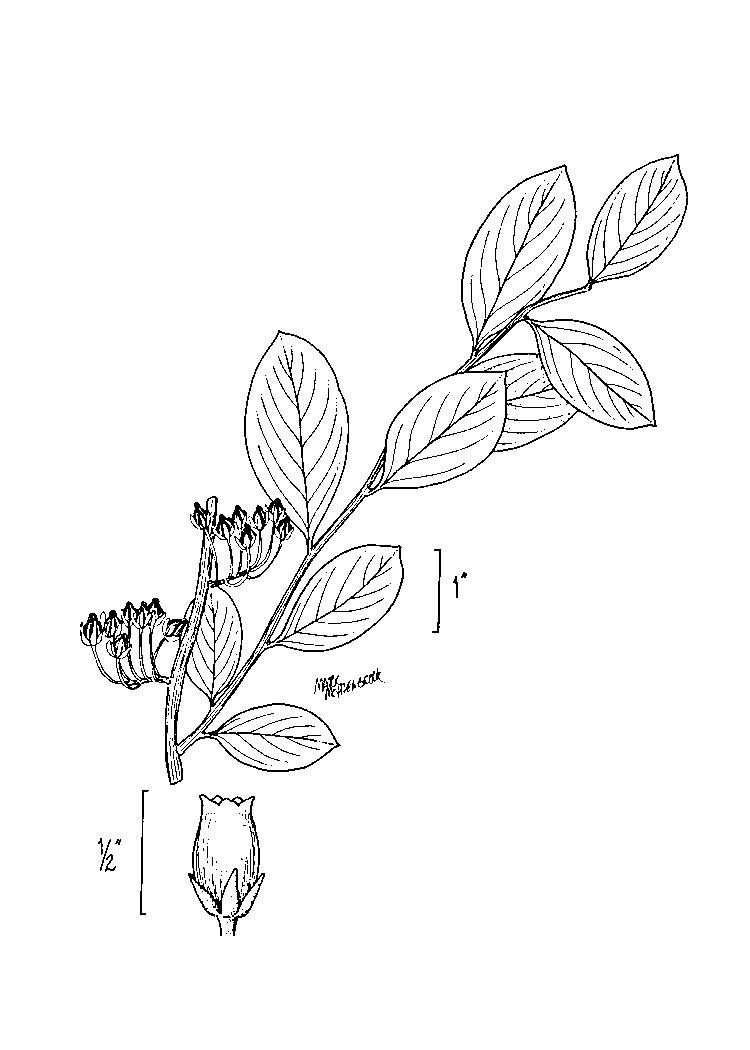
*Lyonia mariana* (from USDA-NRCS 2012).

**Figure 190f.**
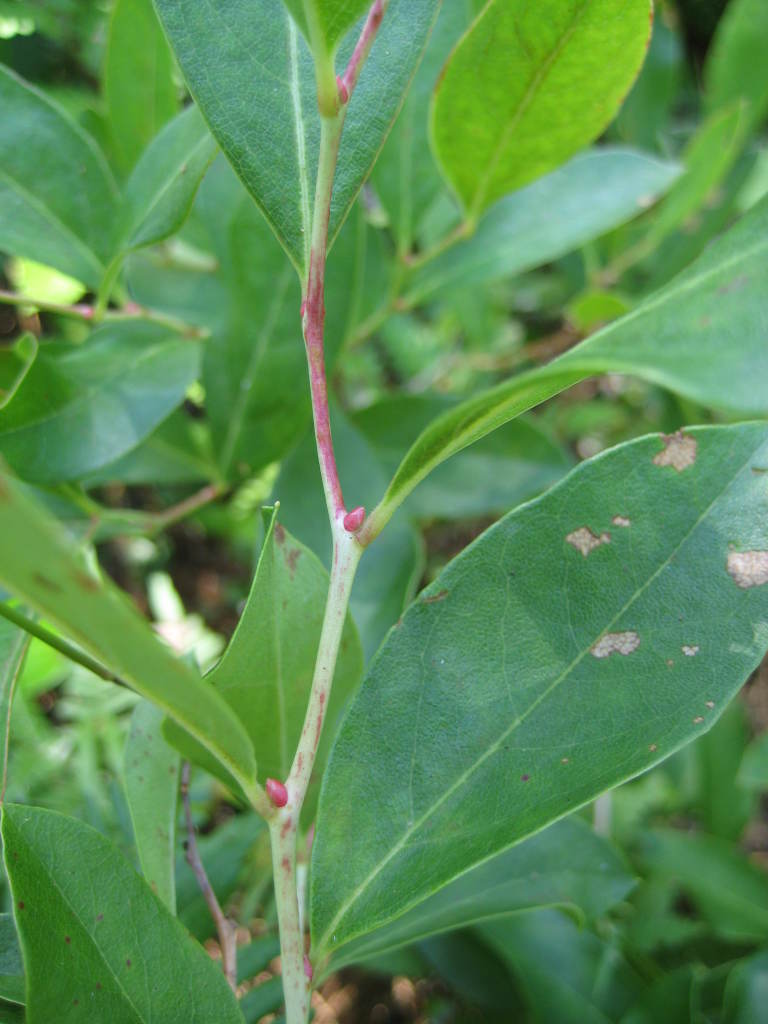
*Lyonia mariana*: note the bright pink, globose axillary buds (photo by R. Thornhill).

**Figure 191a.**
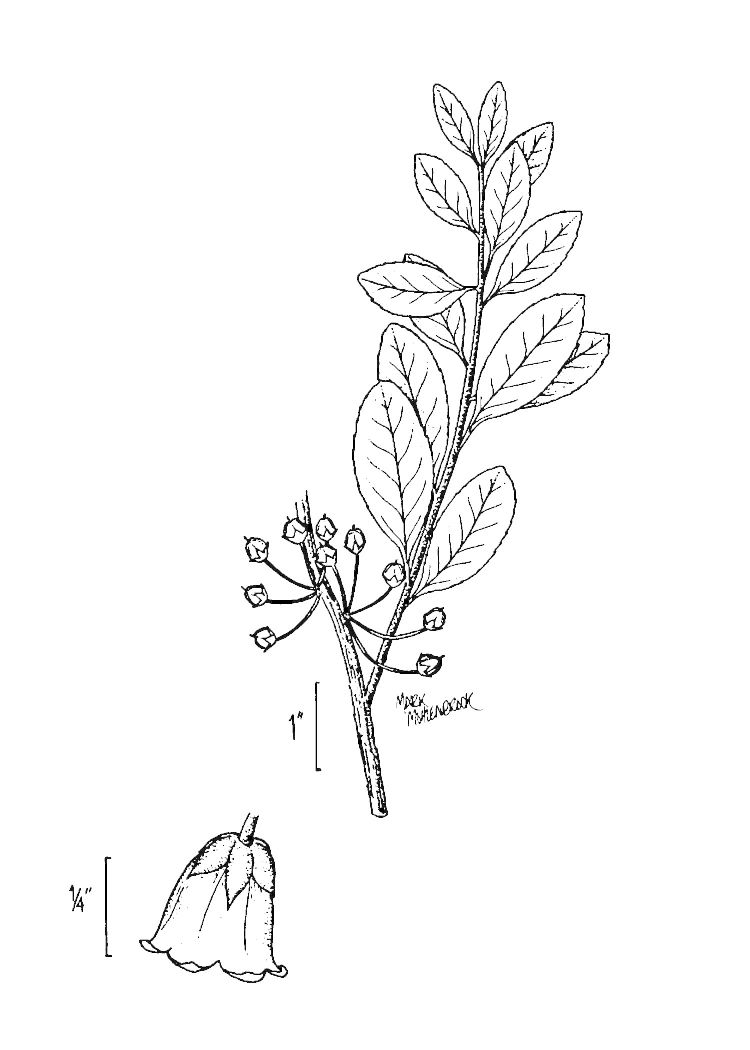
From USDA-NRCS (2012).

**Figure 191b.**
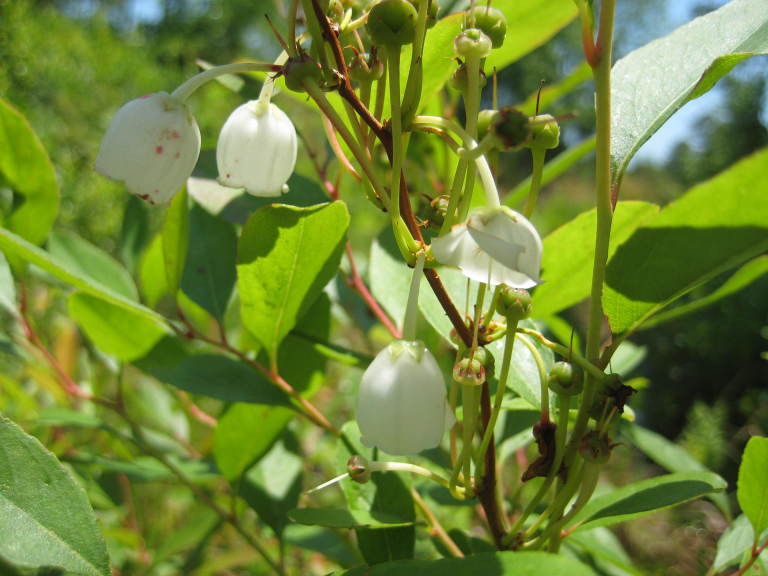
Photo by R. Thornhill.

**Figure 192.**
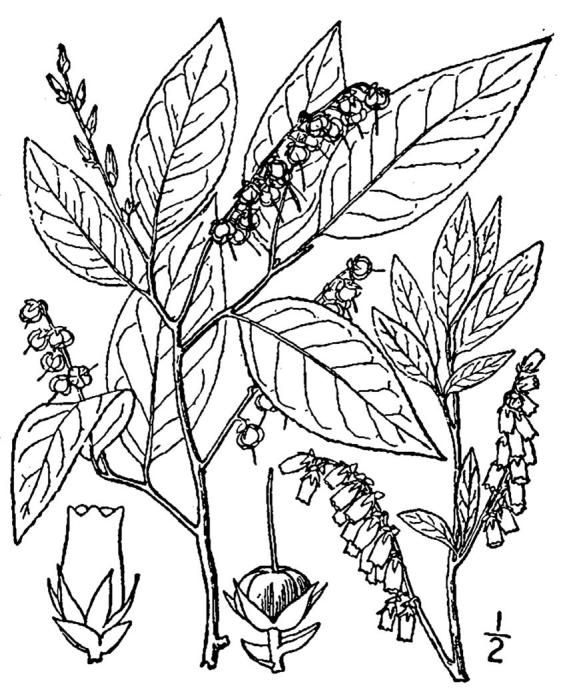
*Eubotrys racemosa* (from Britton and Brown 1913).

**Figure 193a.**
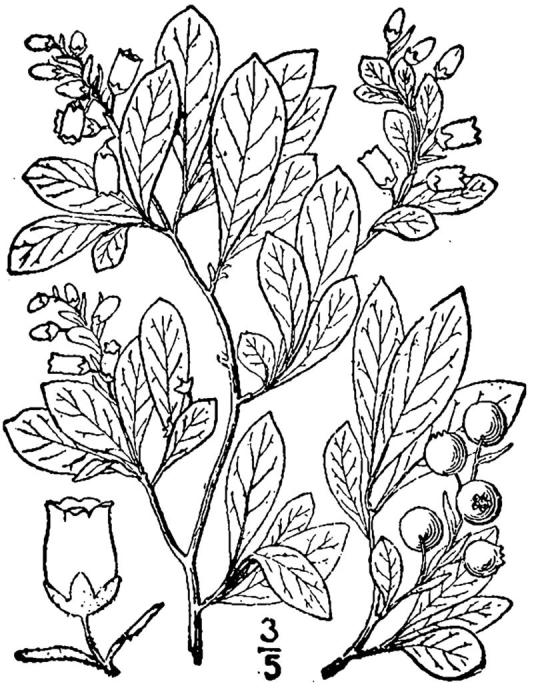
*Gaylussacia dumosa* (from Britton and Brown 1913).

**Figure 193b.**
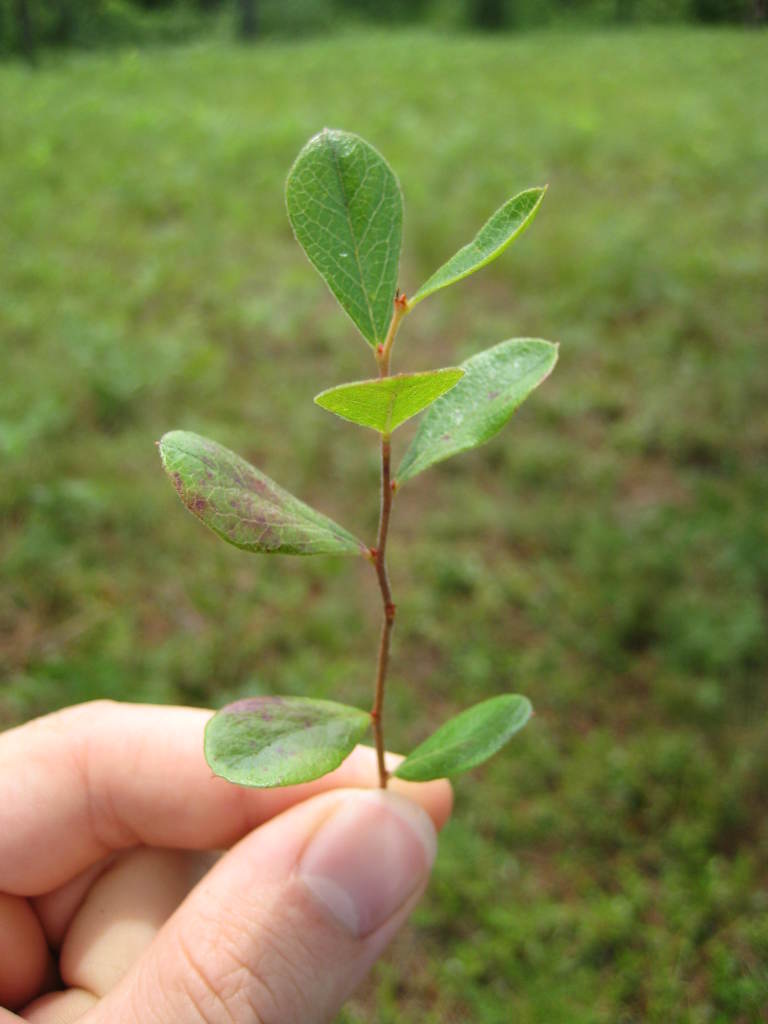
*Gaylussacia dumosa* (photo by R. Thornhill).

**Figure 193c.**
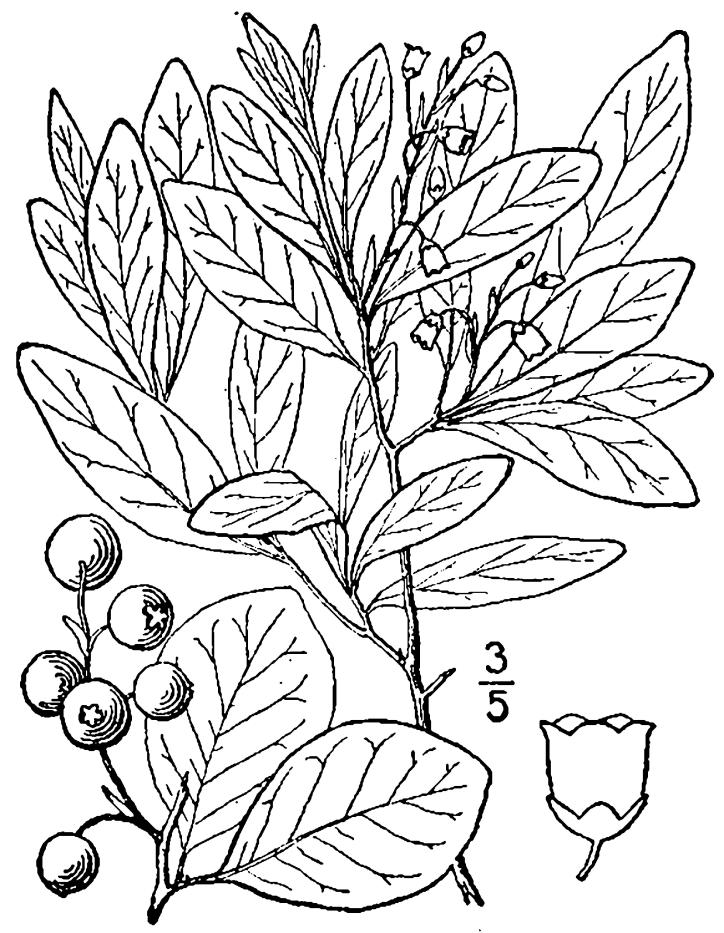
*Gaylussacia frondosa* (from Britton and Brown 1913).

**Figure 193d.**
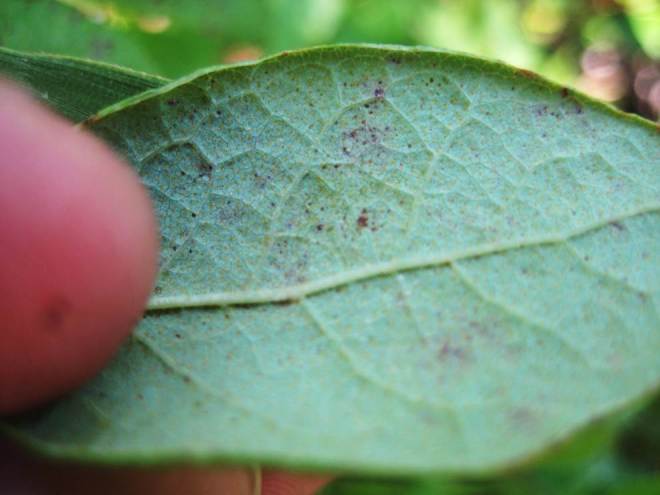
*Gaylussacia frondosa*: close-up of abaxial leaf surface showing overall blue-green color and small golden glands (photo by R. Thornhill).

**Figure 194a.**
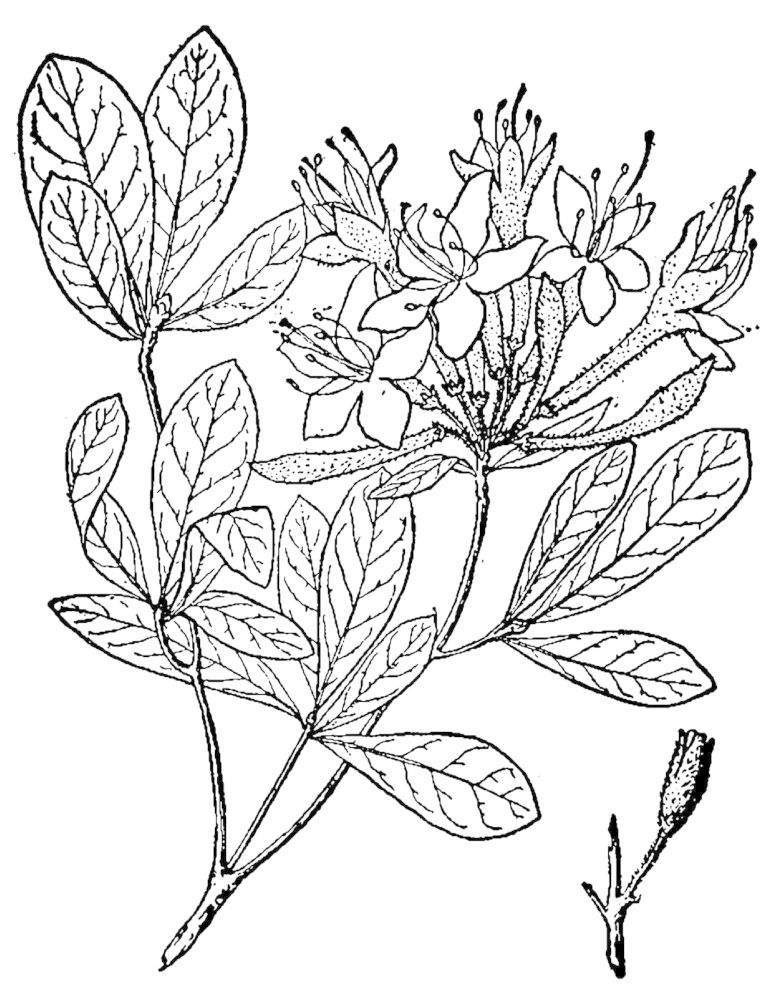
From Britton and Brown (1913).

**Figure 194b.**
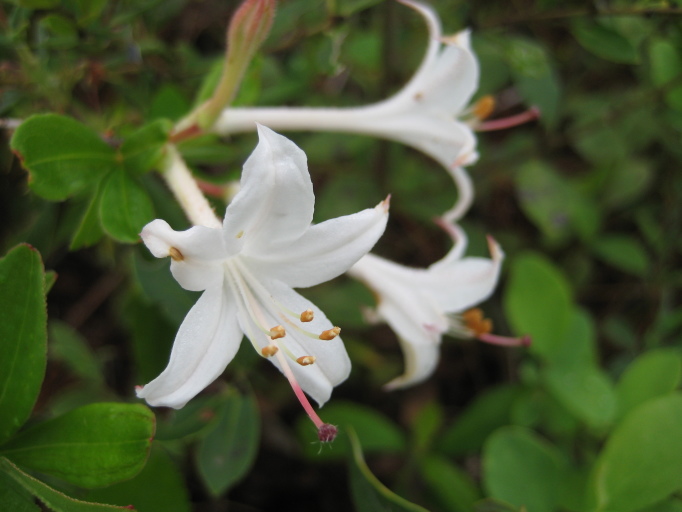
Photo by R. Thornhill.

**Figure 195a.**
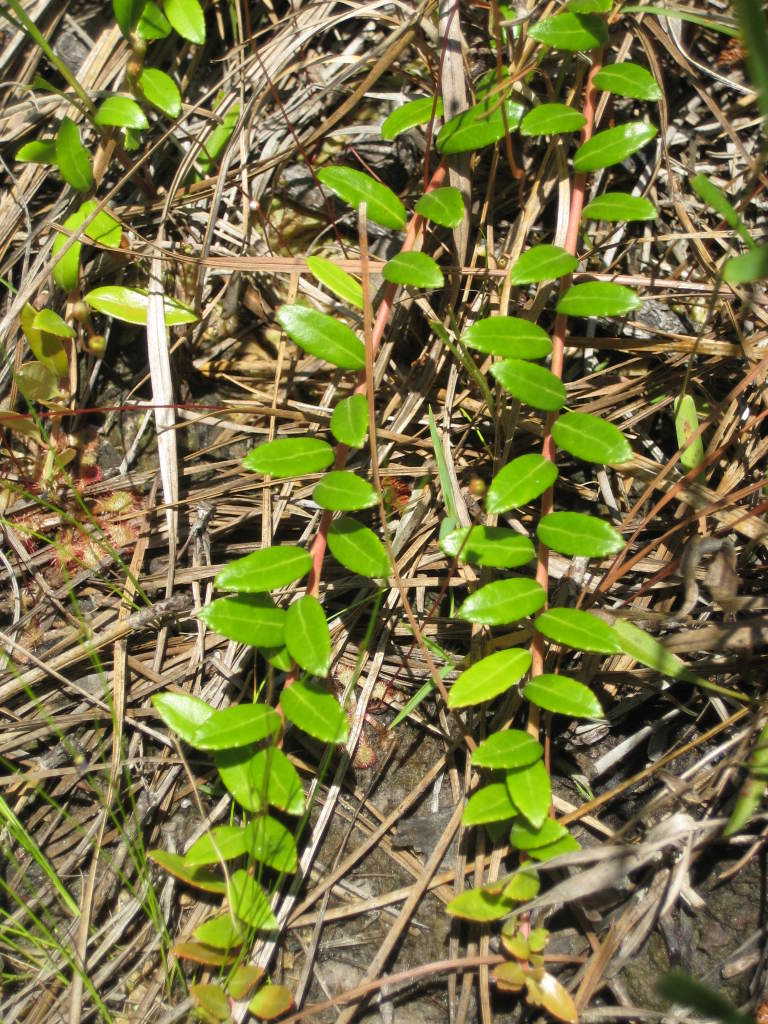
*Vaccinium crassifolium* (photo by R. Thornhill).

**Figure 195b.**
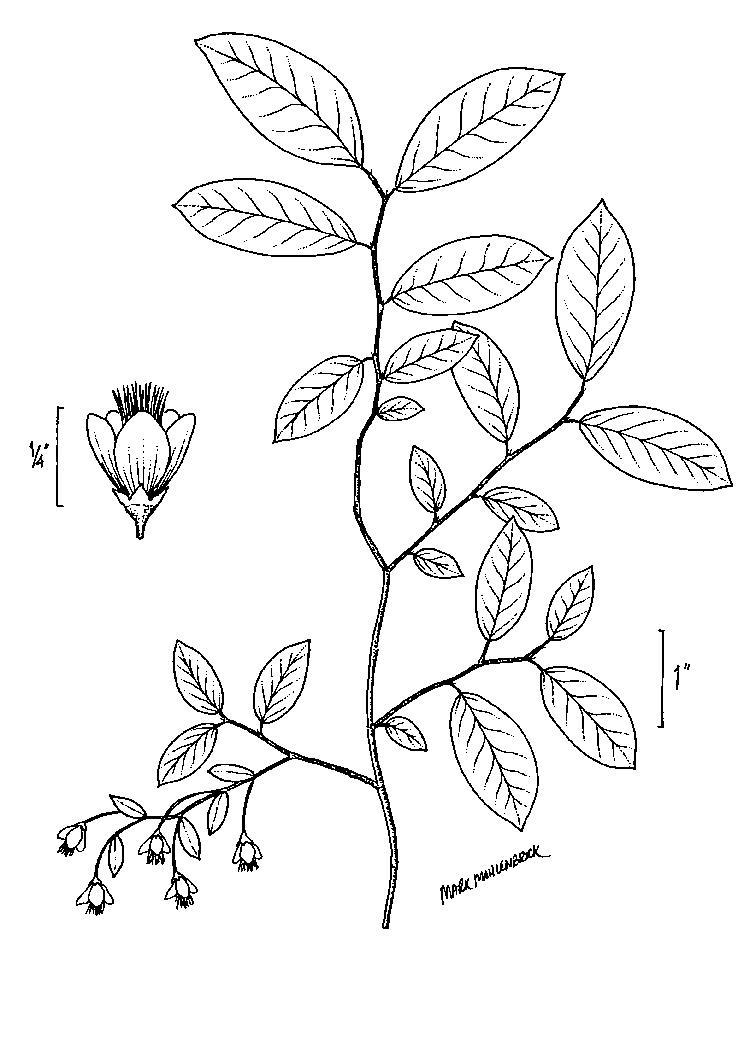
*Vaccinium stamineum* (from USDA-NRCS 2012).

**Figure 195c.**
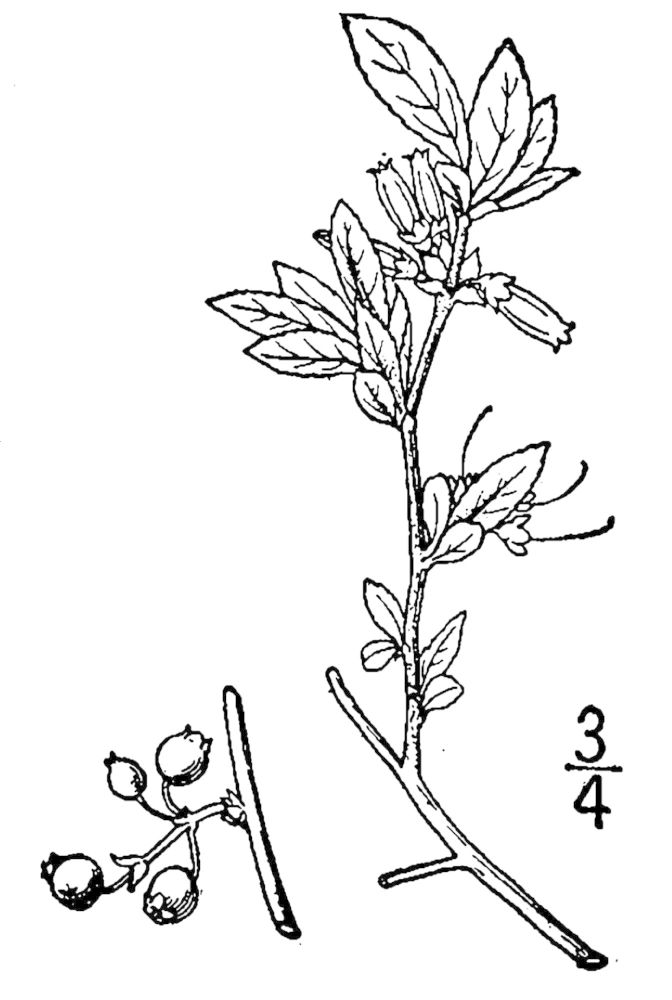
*Vaccinium tenellum* (from Britton and Brown 1913).

**Figure 195d.**
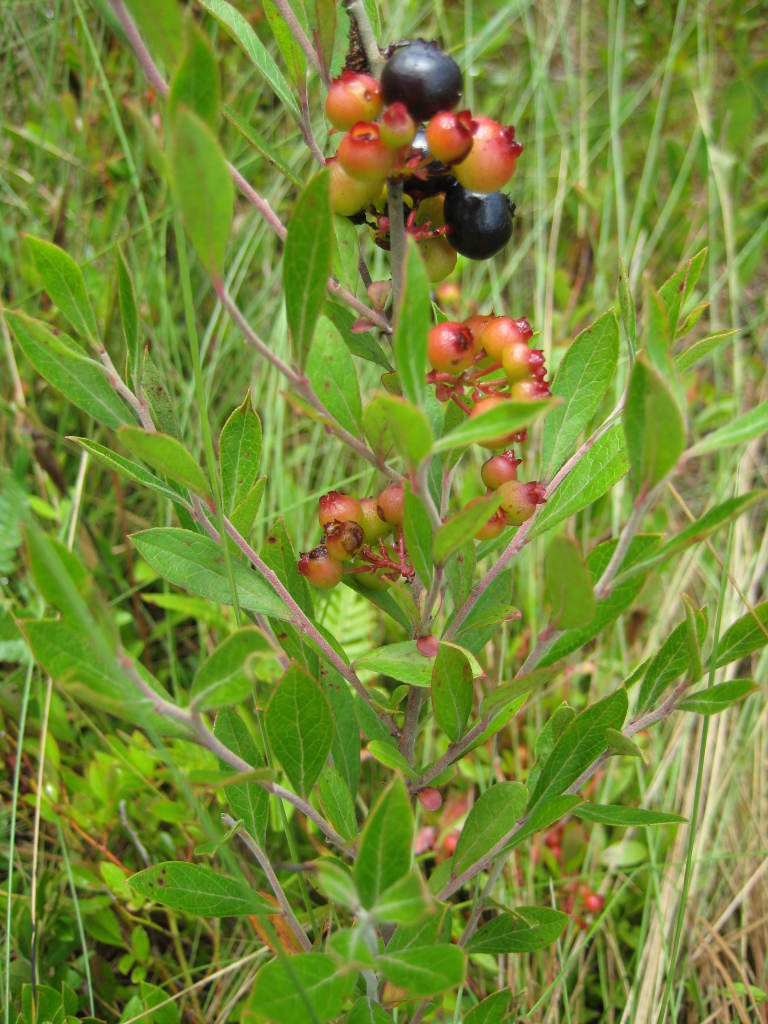
*Vaccinium tenellum* (photo by R. Thornhill).

**Figure 196a.**
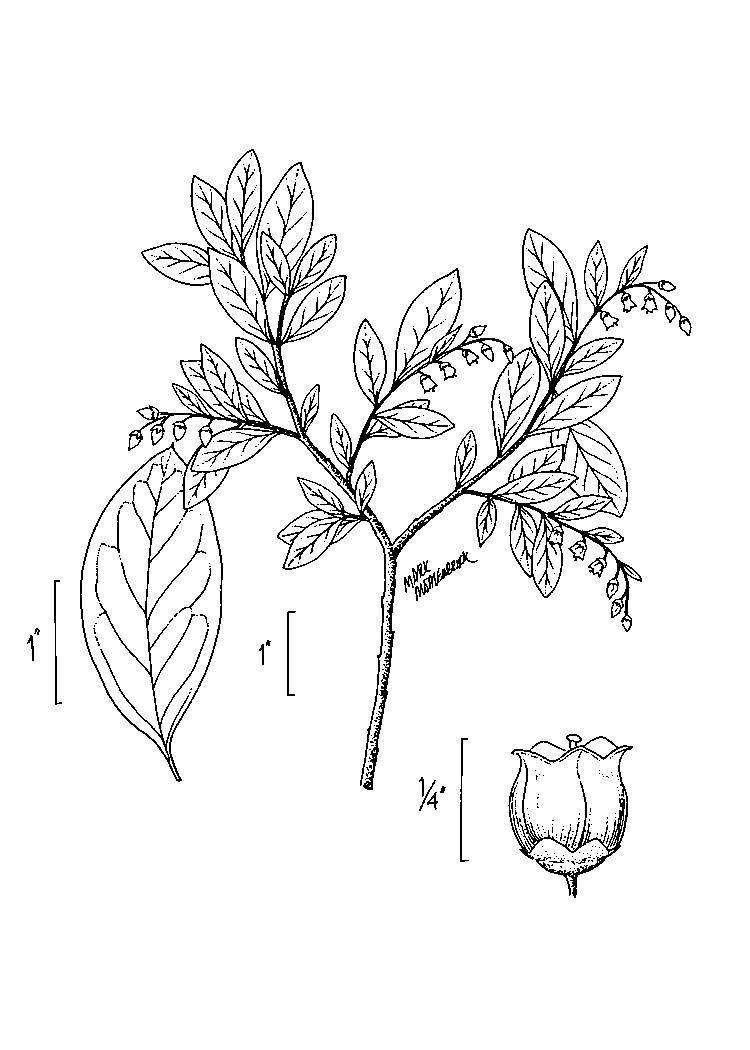
*Vaccinium arboreum* (from USDA-NRCS 2012).

**Figure 196b.**
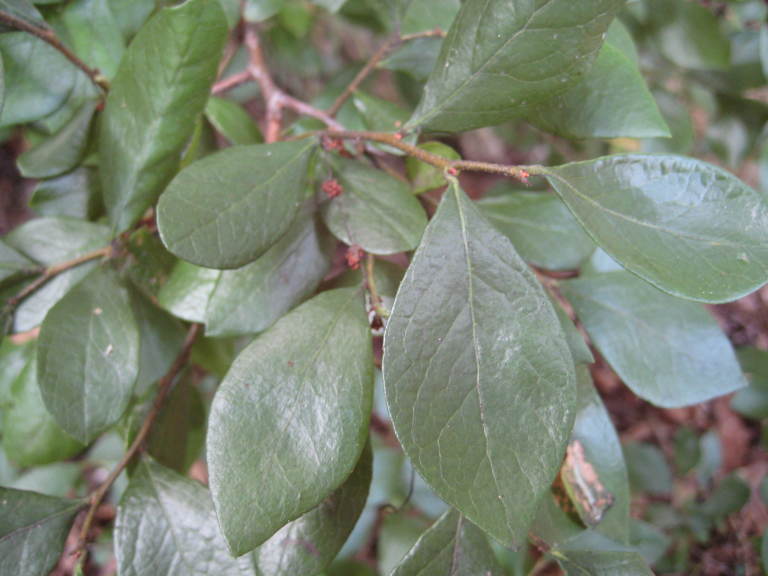
*Vaccinium arboreum* (photo by R. Thornhill).

**Figure 196c.**
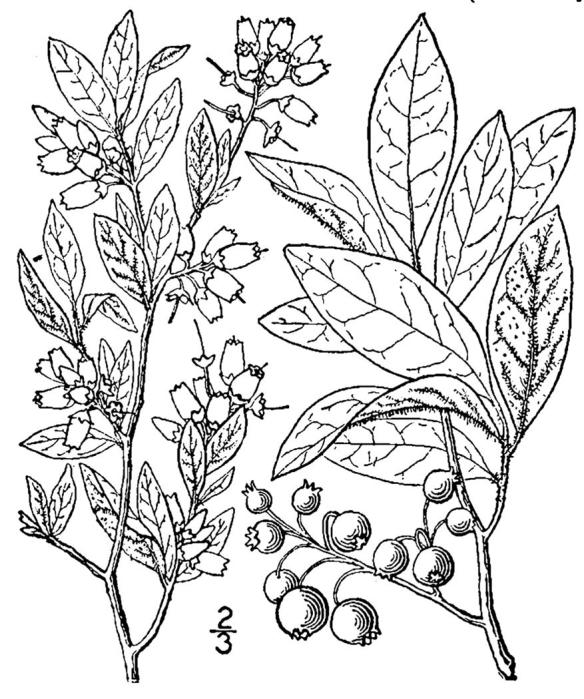
*Vaccinium fuscatum* (from Britton and Brown 1913).

**Figure 196d.**
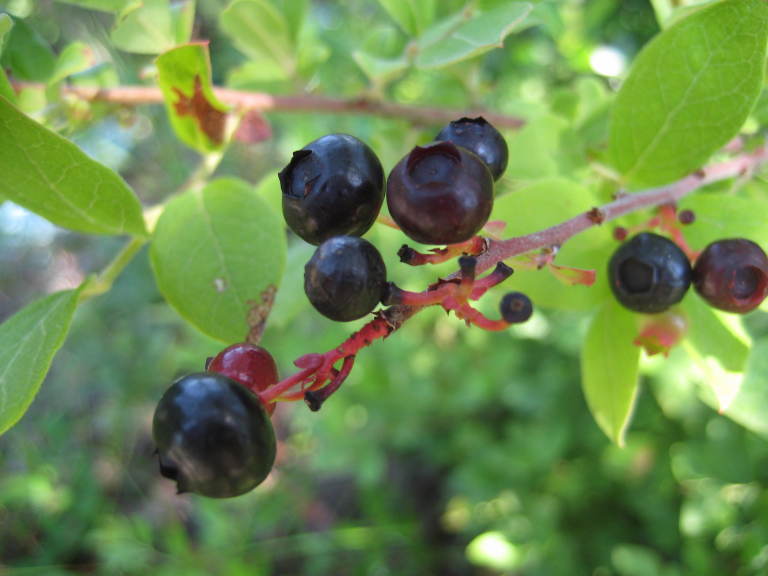
*Vaccinium fuscatum* (photo by R. Thornhill).

**Figure 197a.**
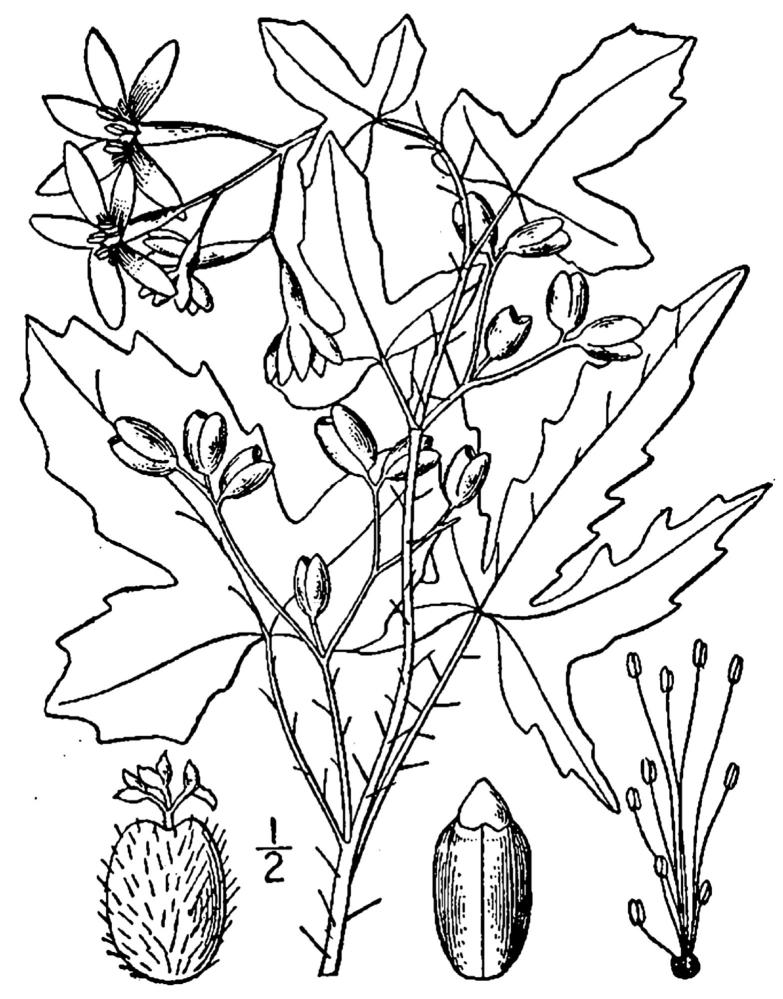
From Britton and Brown (1913).

**Figure 197b.**
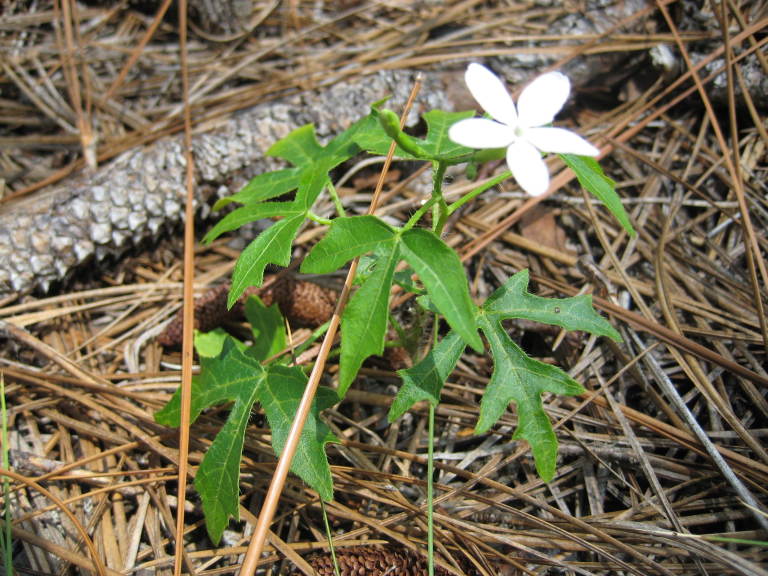
Photo by R. Thornhill.

**Figure 198.**
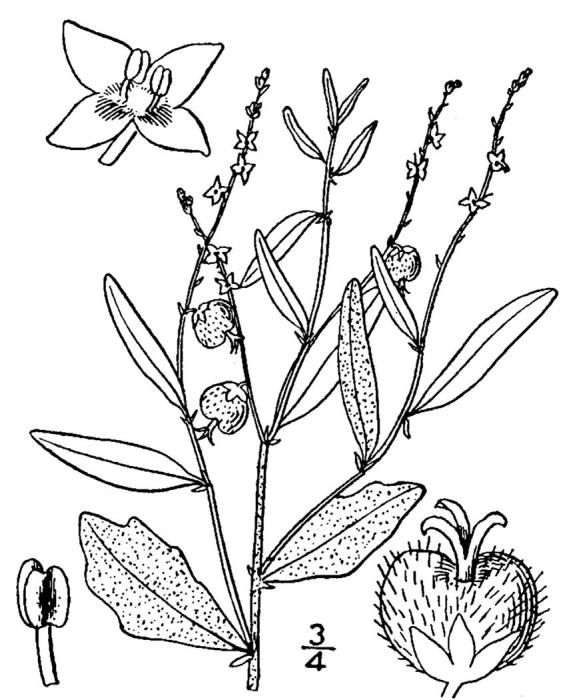
*Tragia urens* (from Britton and Brown 1913).

**Figure 199.**
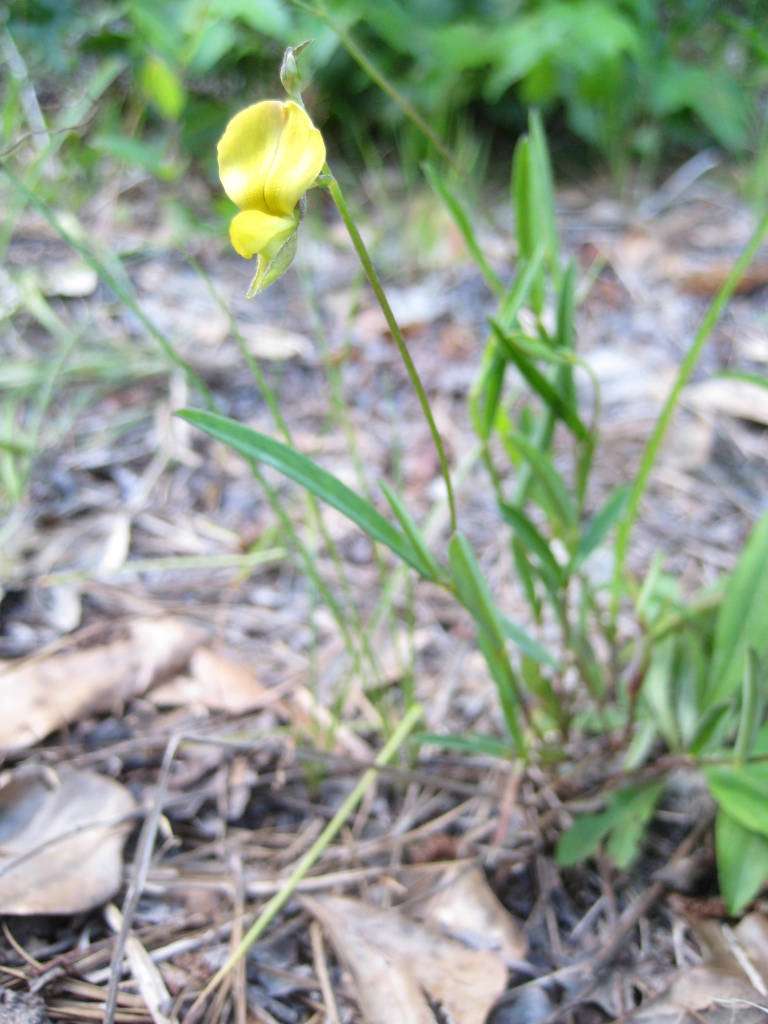
*Crotalaria purshii*

**Figure 200.**
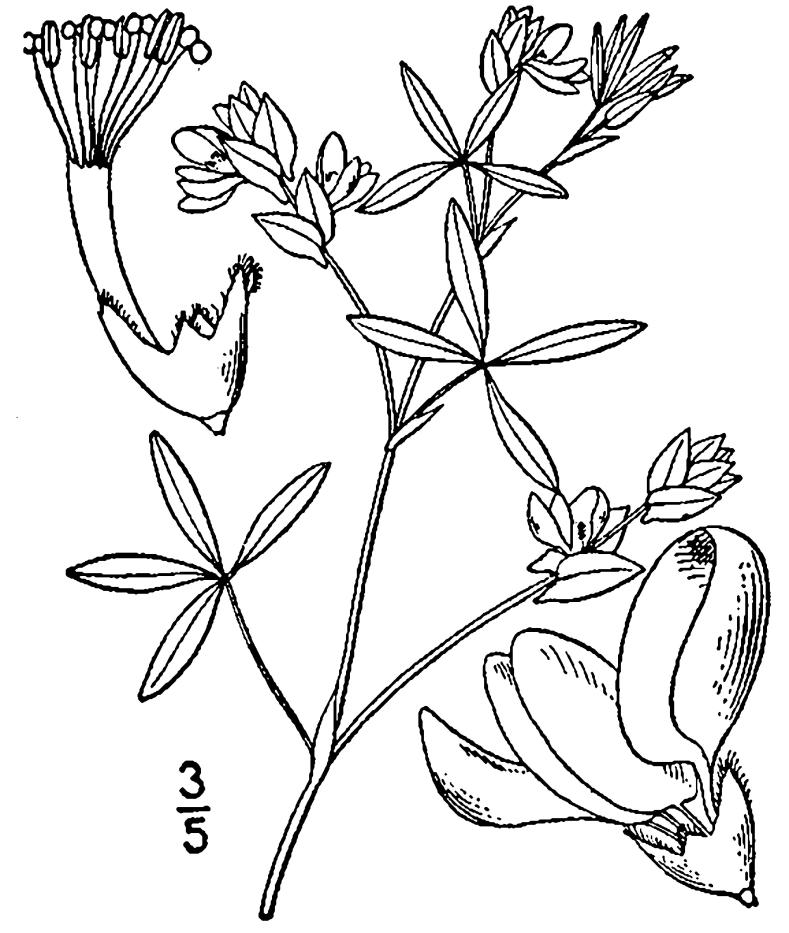
*Zornia bracteata* (from Britton and Brown 1913).

**Figure 201.**
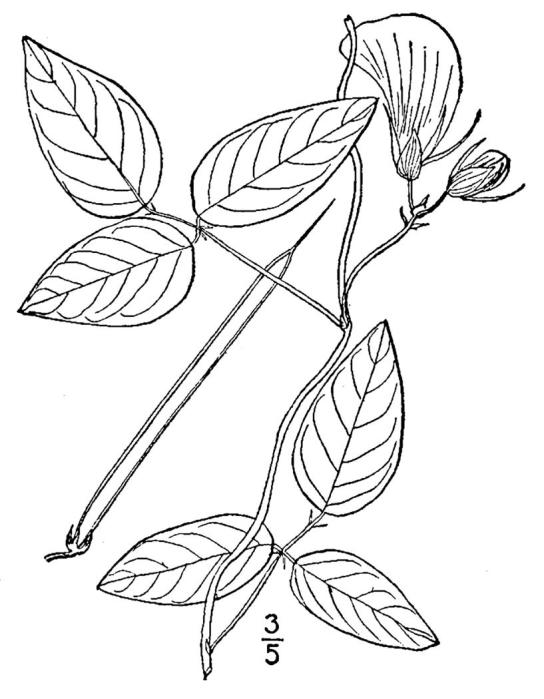
*Centrosema virginianum* (from Britton and Brown 1913).

**Figure 202a.**
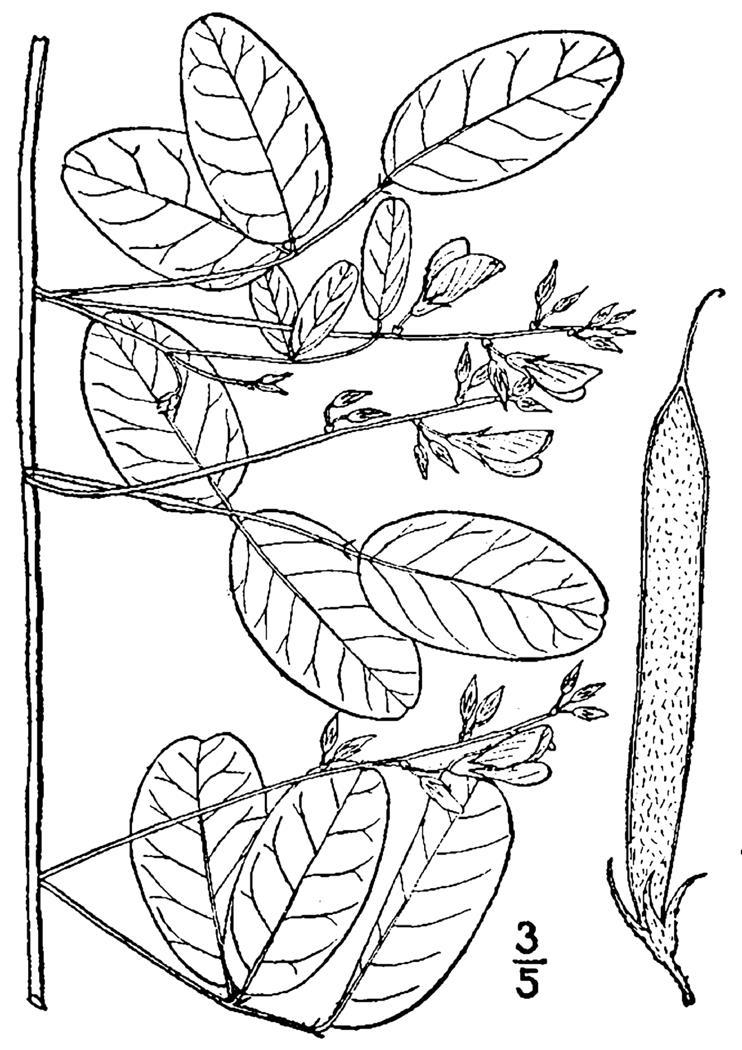
From Britton and Brown (1913).

**Figure 202b.**
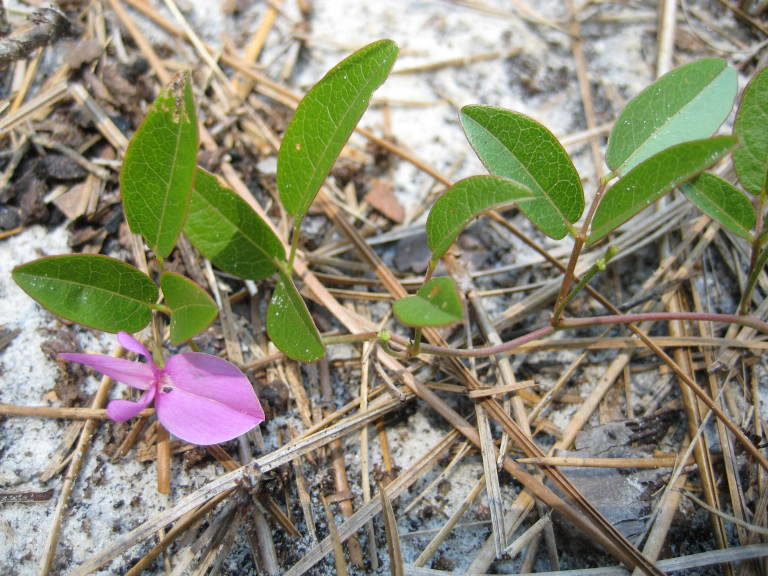
Photo by R. Thornhill.

**Figure 203.**
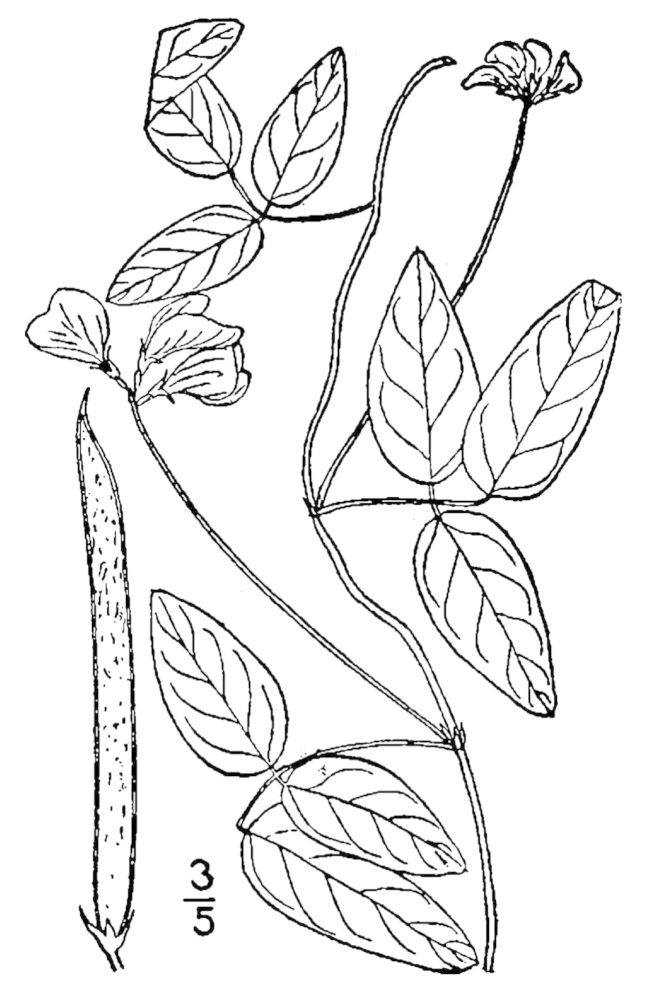
*Strophostyles umbellata* (from Britton and Brown 1913).

**Figure 204.**
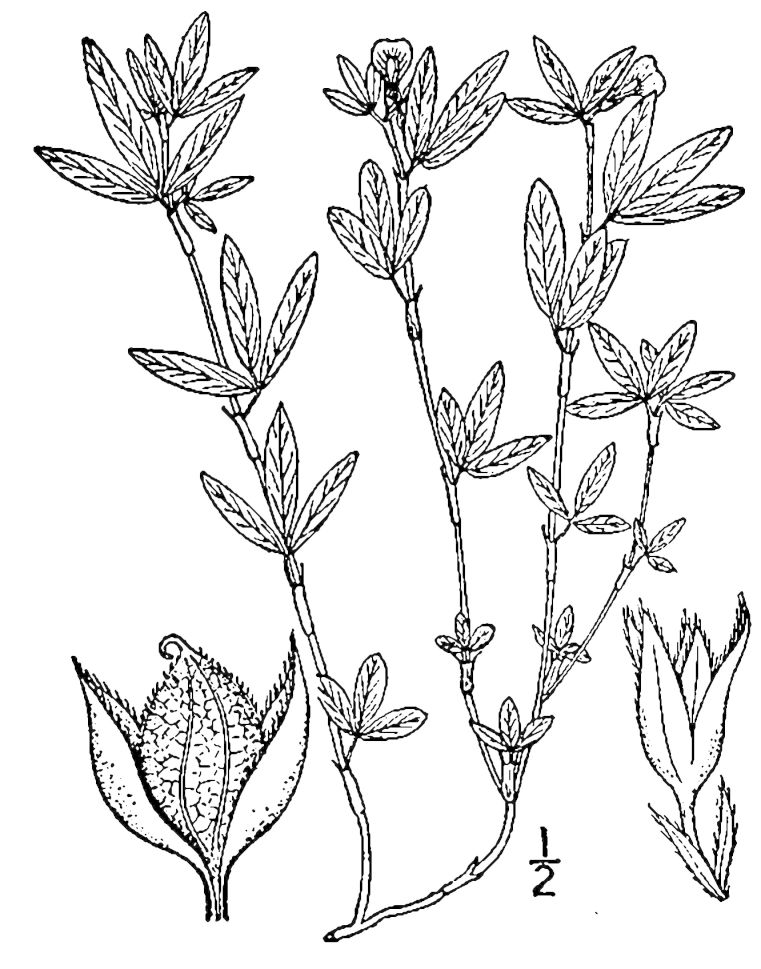
*Stylosanthes biflora* (from Britton and Brown 1913).

**Figure 205.**
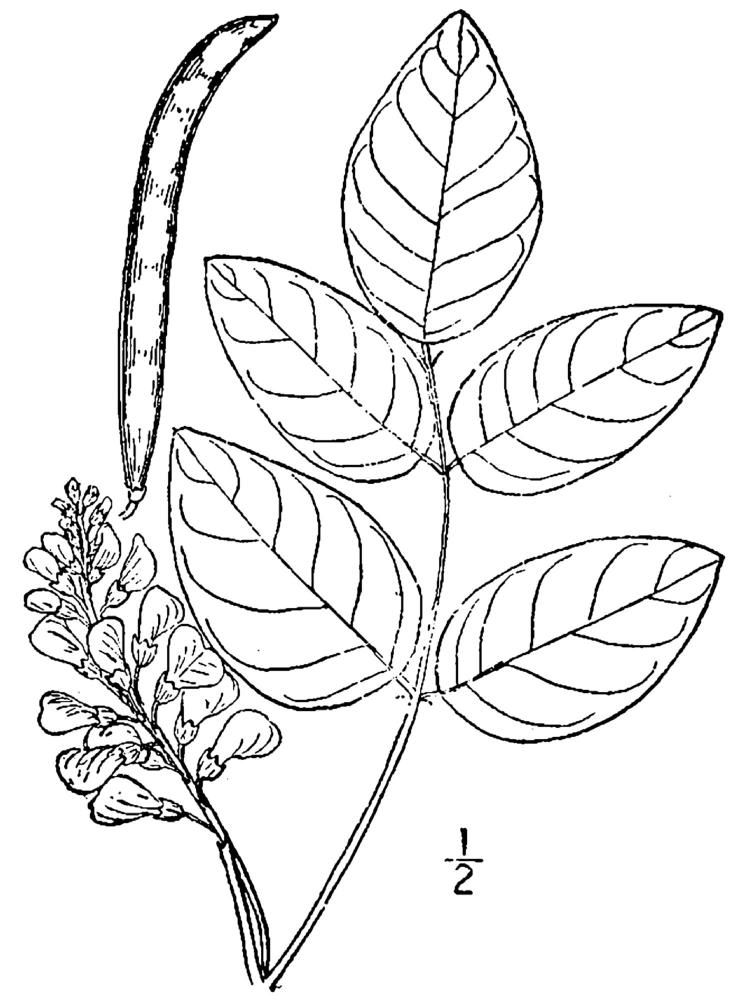
*Apios americana* (from USDA-NRCS 2012).

**Figure 206a.**
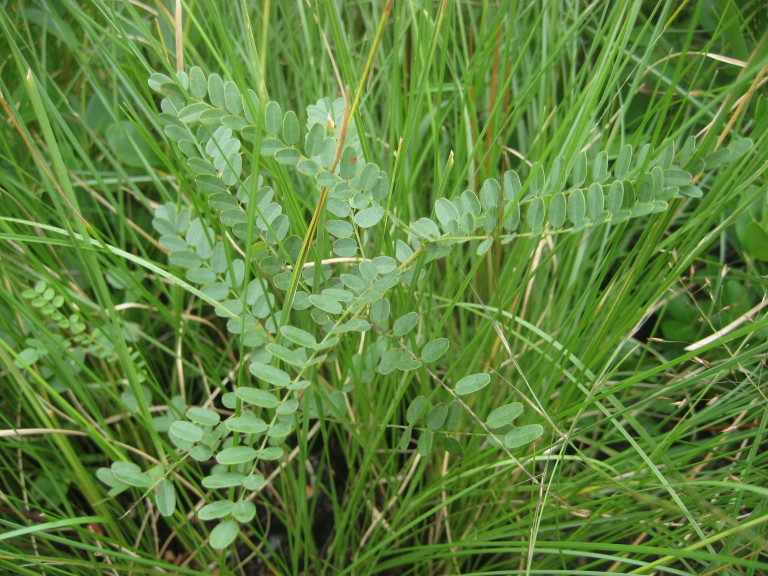
*Amorpha georgiana* (photo by R. Thornhill).

**Figure 206b.**
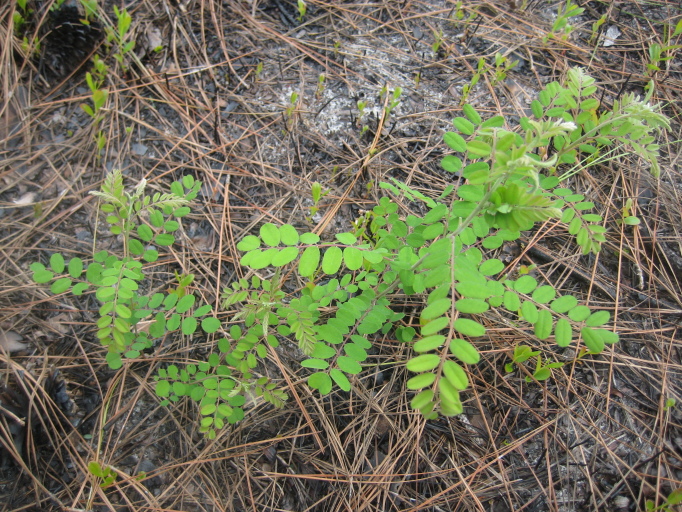
*Amorpha herbacea* (photo by R. Thornhill).

**Figure 206c.**
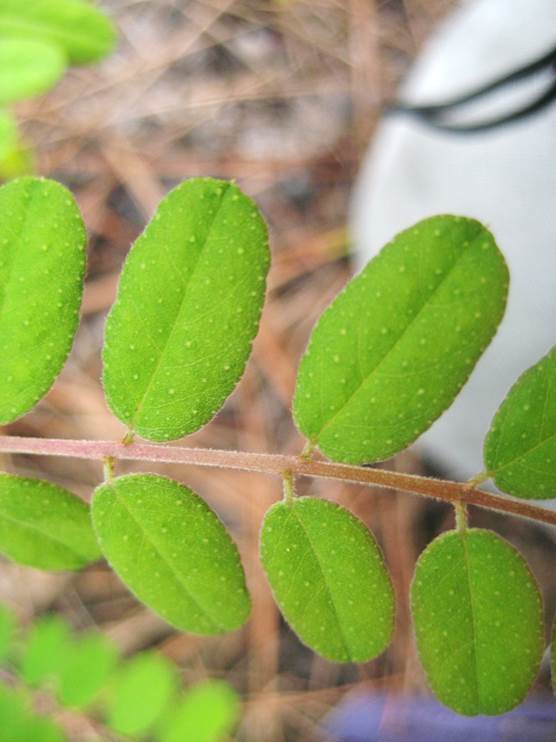
*Amorpha herbacea*: close-up of leaflets showing translucent glandular punctae (photo by R. Thornhill).

**Figure 206d.**
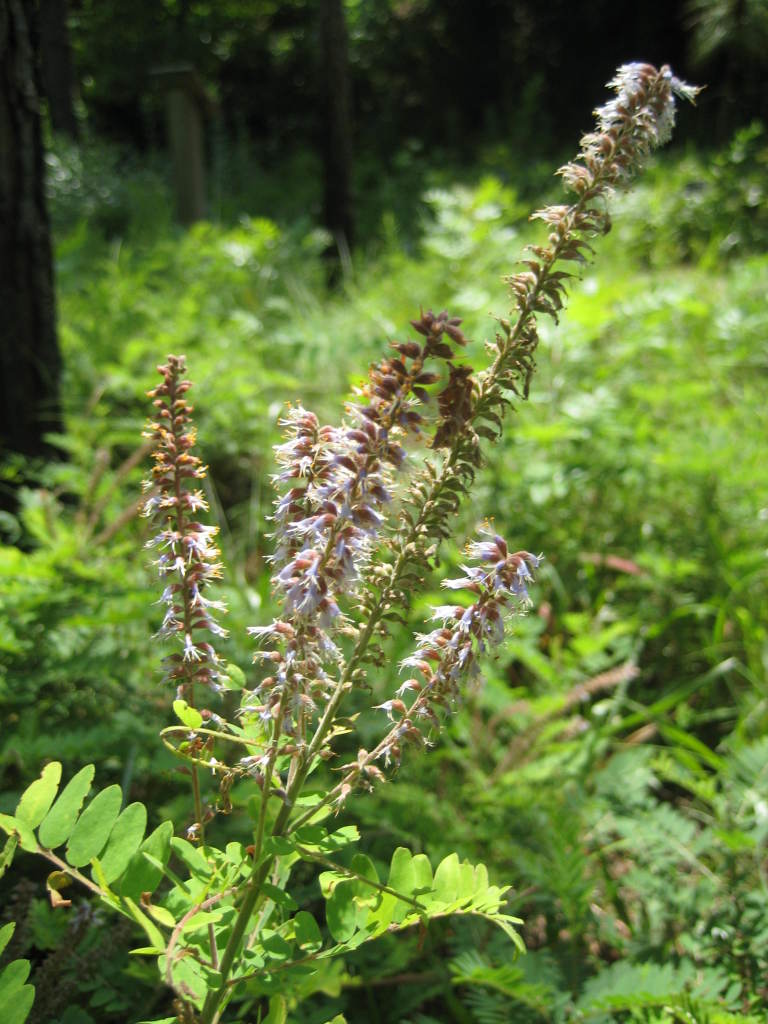
*Amorpha herbacea*: inflorescence (photo by R. Thornhill).

**Figure 207a.**
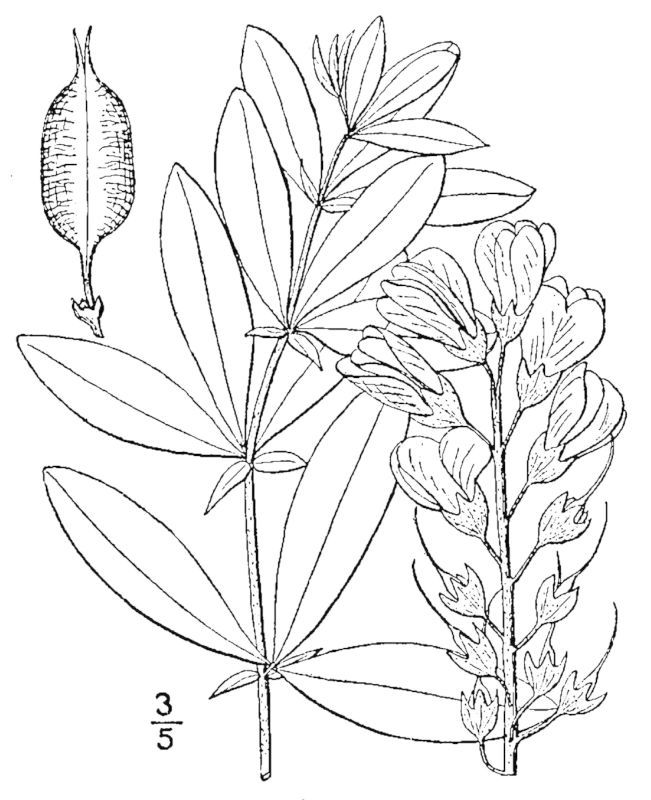
*Baptisia cinerea* (from Britton and Brown 1913).

**Figure 207b.**
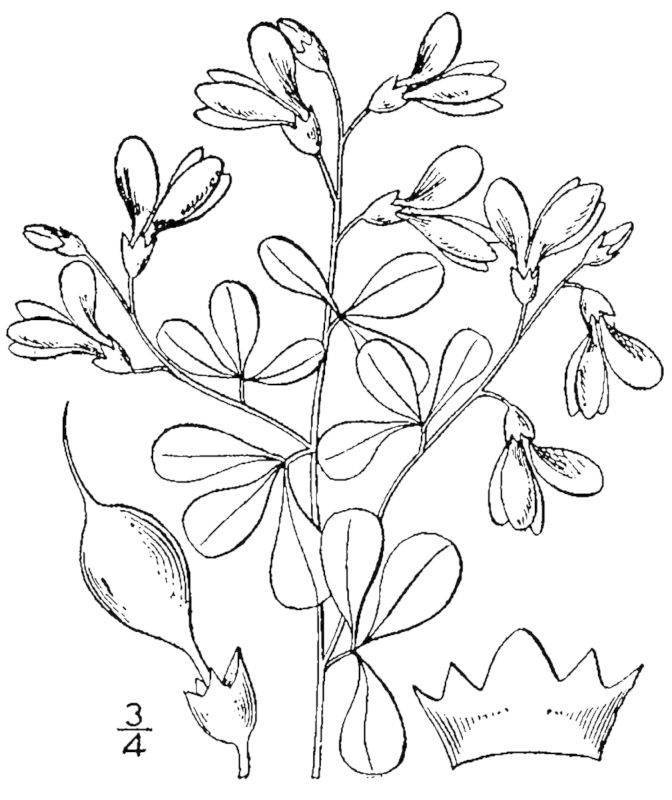
*Baptisia tinctoria* (from Britton and Brown 1913).

**Figure 208a.**
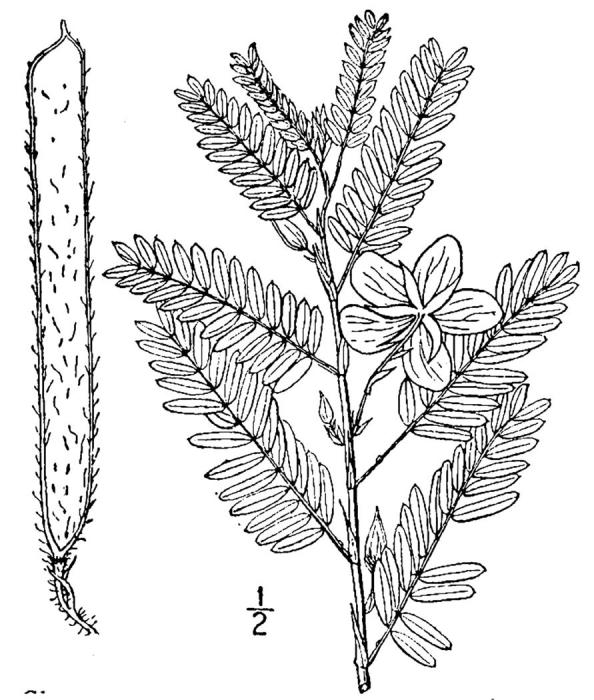
*Cassia fasciculata* (from Britton and Brown 1913).

**Figure 208b.**
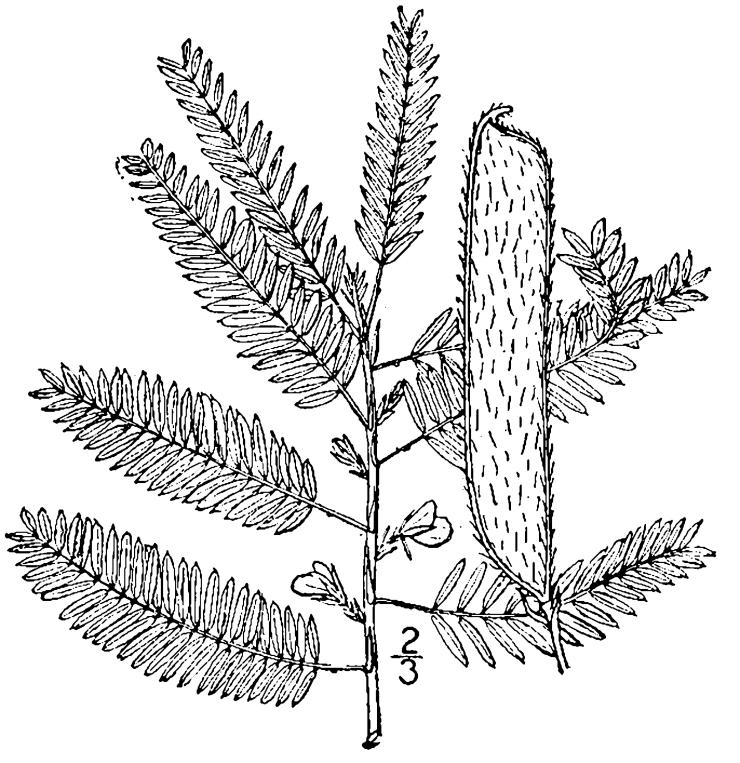
*Cassia nictitans* (from Britton and Brown 1913).

**Figure 209a.**
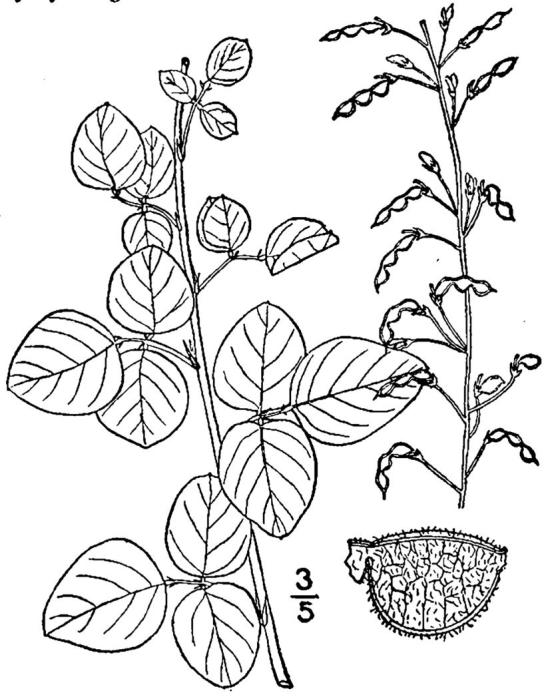
*Desmodium lineatum* (from Britton and Brown 1913).

**Figure 209b.**
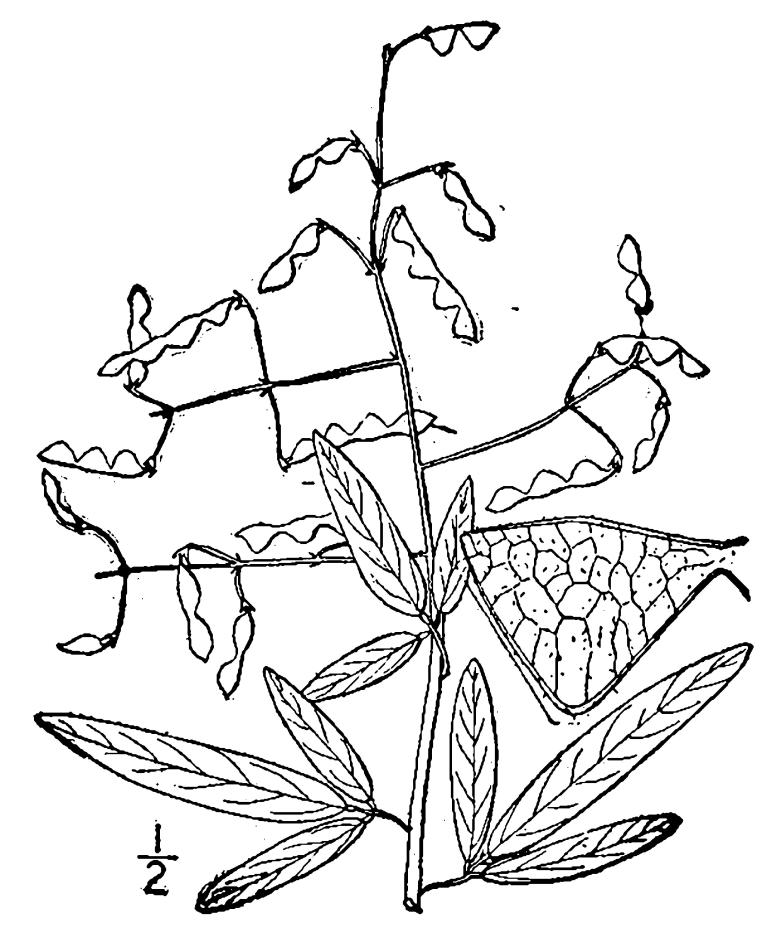
*Desmodium paniculatum* (from Britton and Brown 1913).

**Figure 210a.**
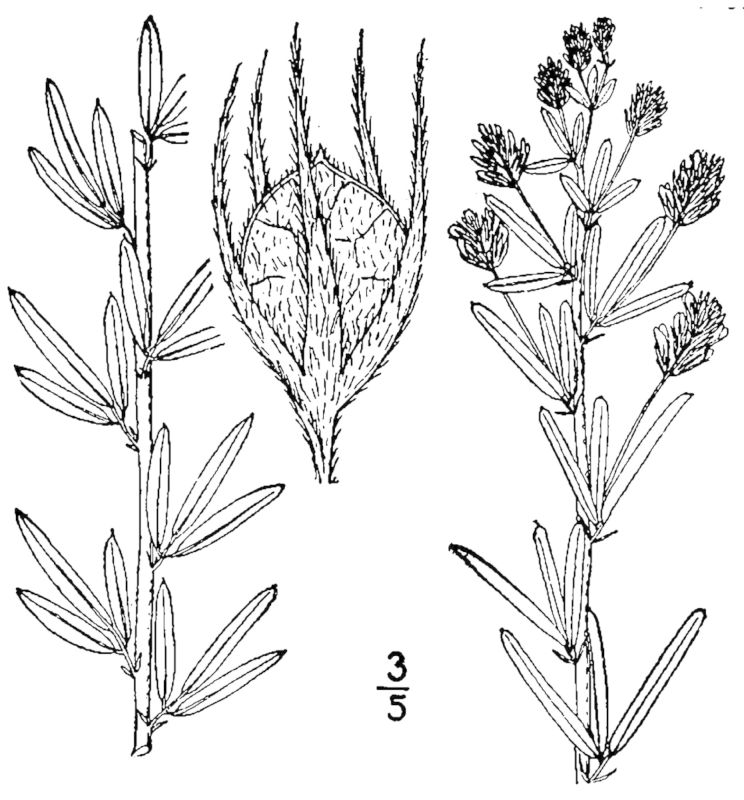
*Lespedeza angustifolia* (from Britton and Brown 1913).

**Figure 210b.**
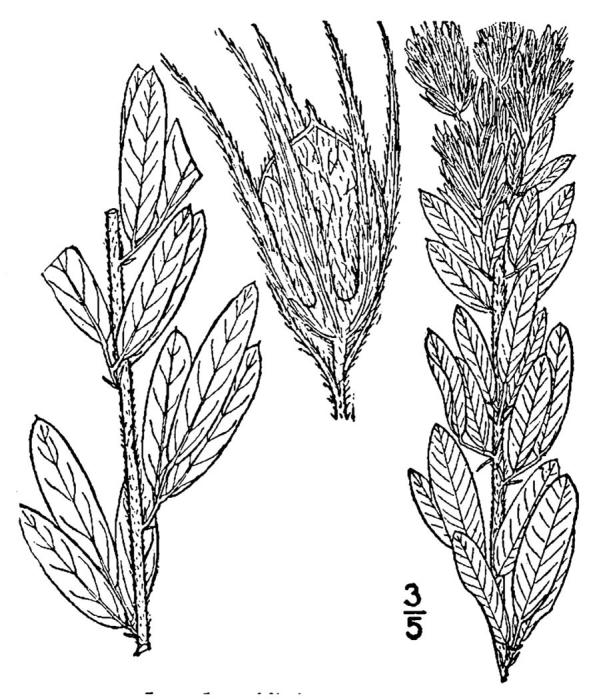
*Lespedeza capitata* (from Britton and Brown 1913).

**Figure 210c.**
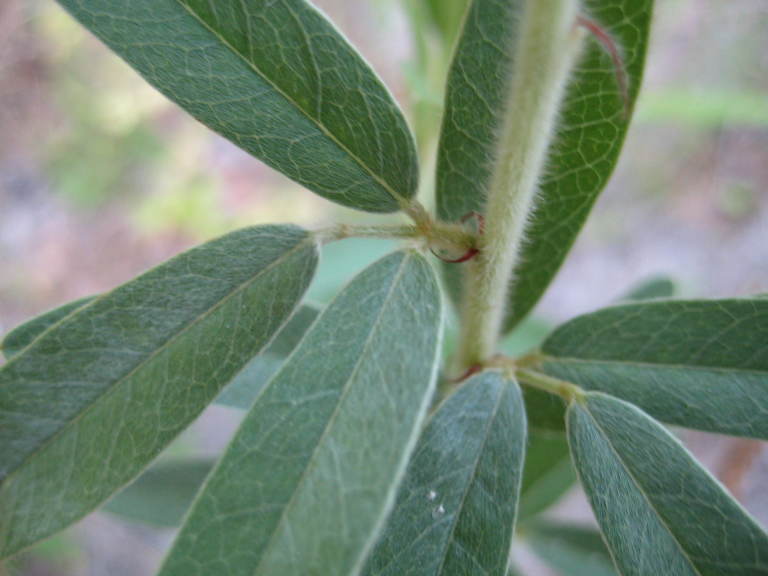
*Lespedeza capitata*: close-up of leaves and stem (photo by R. Thornhill).

**Figure 210d.**
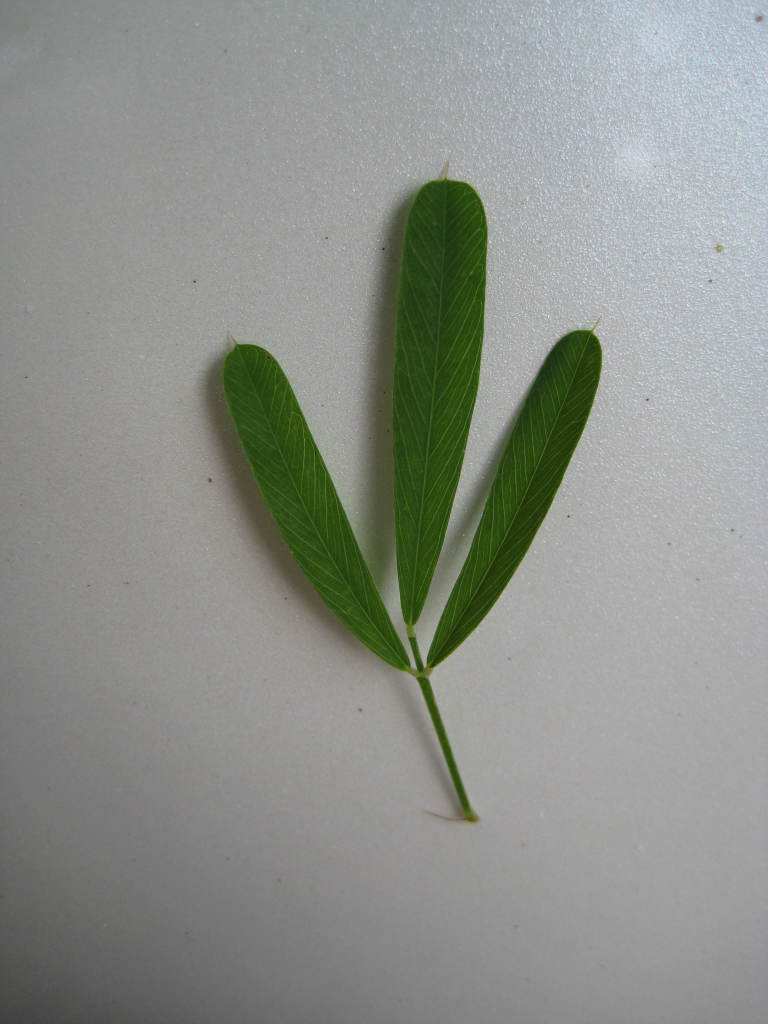
*Lespedeza cuneata*: close-up of leaf (note distinctive oblanceolate shape of leaflets; photo by R. Thornhill).

**Figure 210e.**
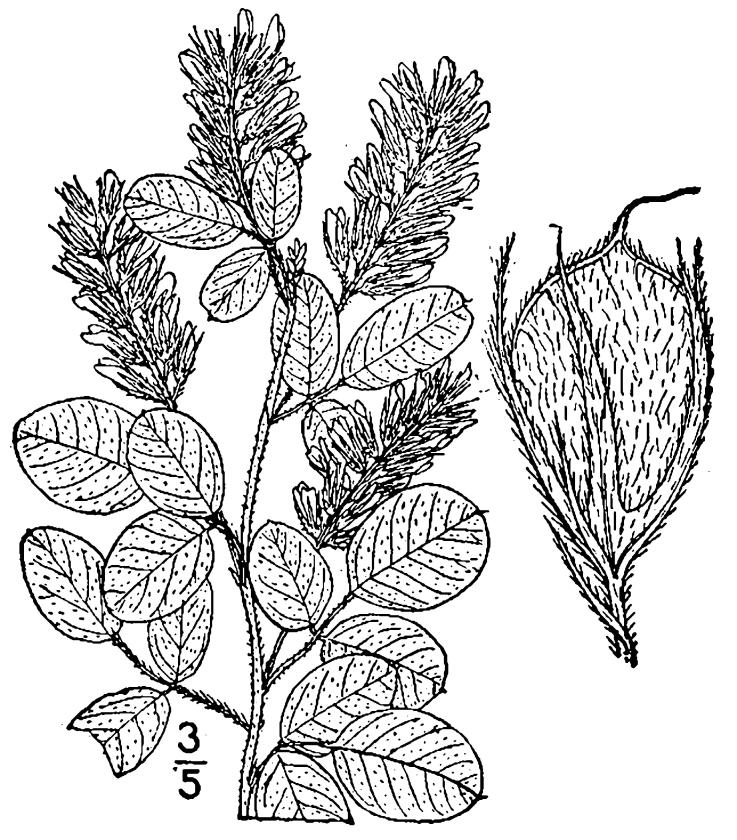
*Lespedeza hirta* (from Britton and Brown 1913).

**Figure 210f.**
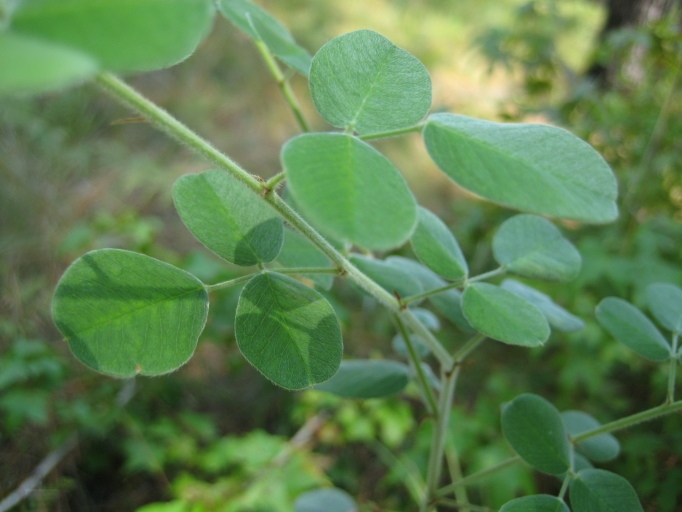
Lespedeza hirta var. curtissii: close-up of leaves and stem (photo by R. Thornhill).

**Figure 211a.**
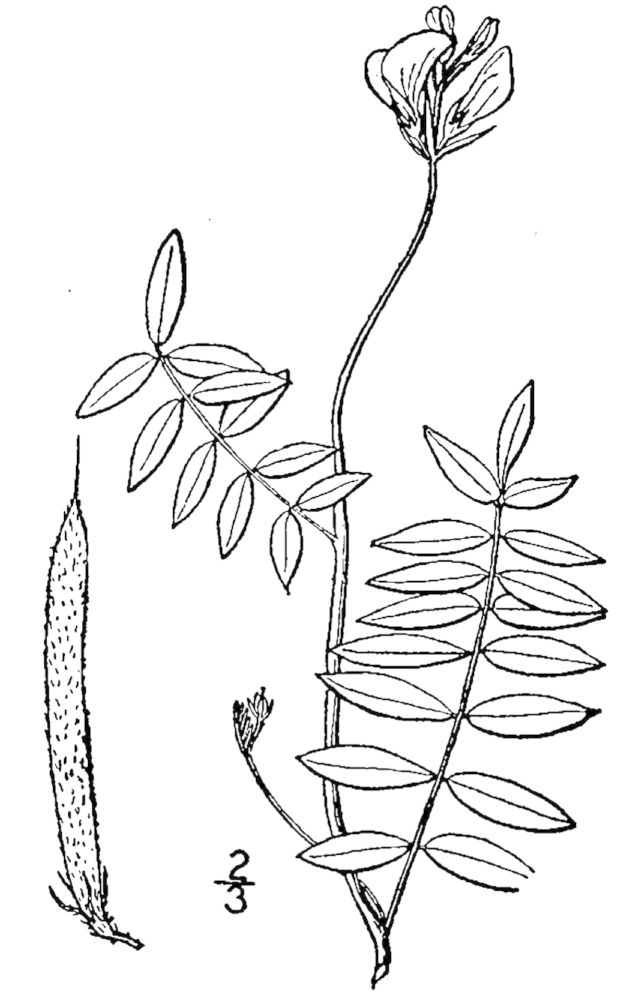
*Tephrosia hispidula* (from Britton and Brown 1913).

**Figure 211b.**
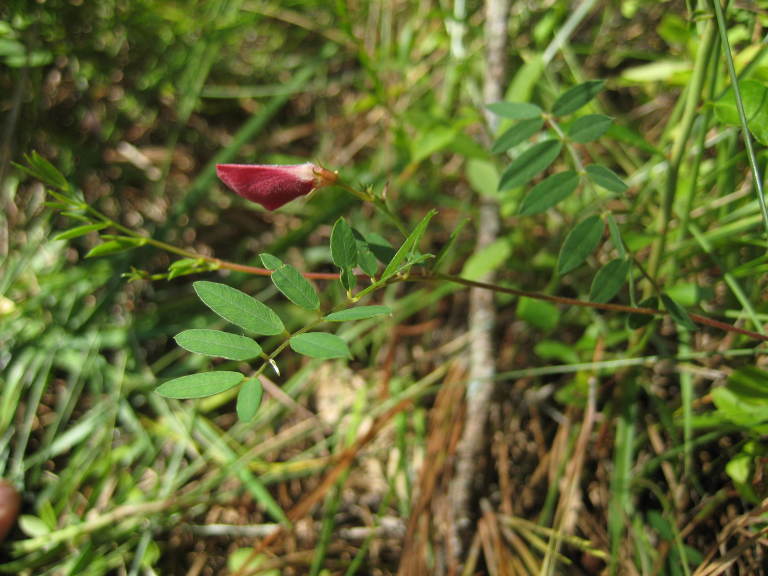


**Figure 211c.**
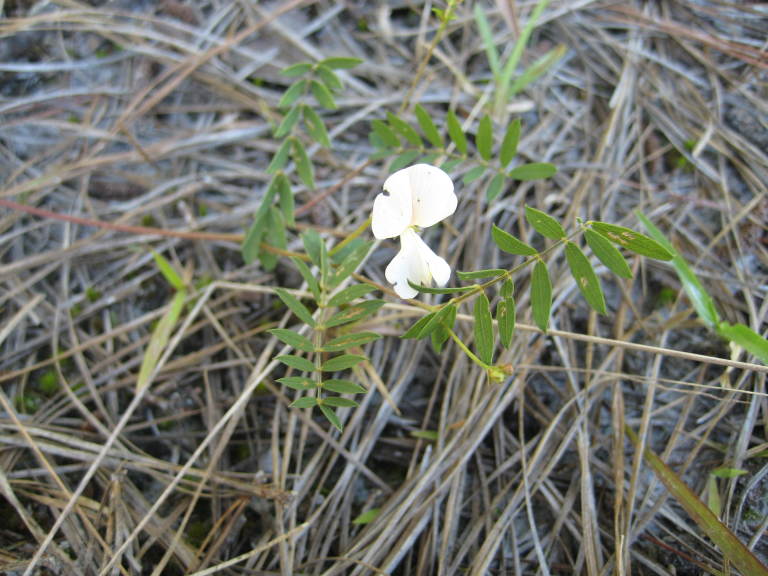
*Tephrosia hispidula*: mature flowers often turn white (photo by R. Thornhill).

**Figure 211d.**
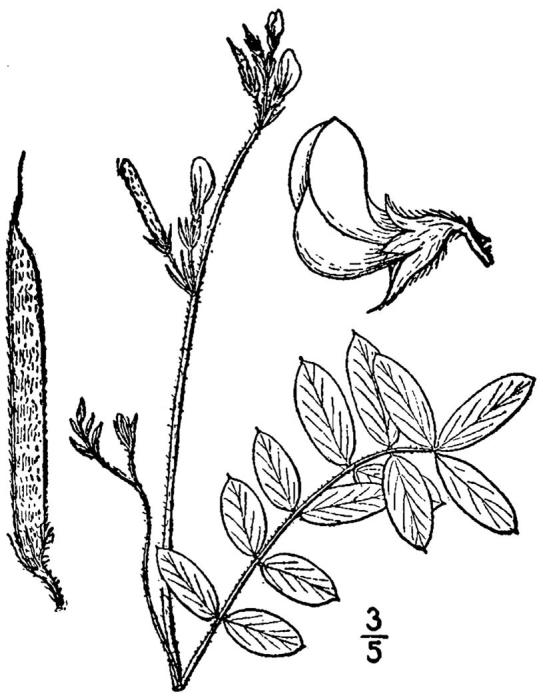
*Tephrosia spicata* (from Britton and Brown 1913).

**Figure 212a.**
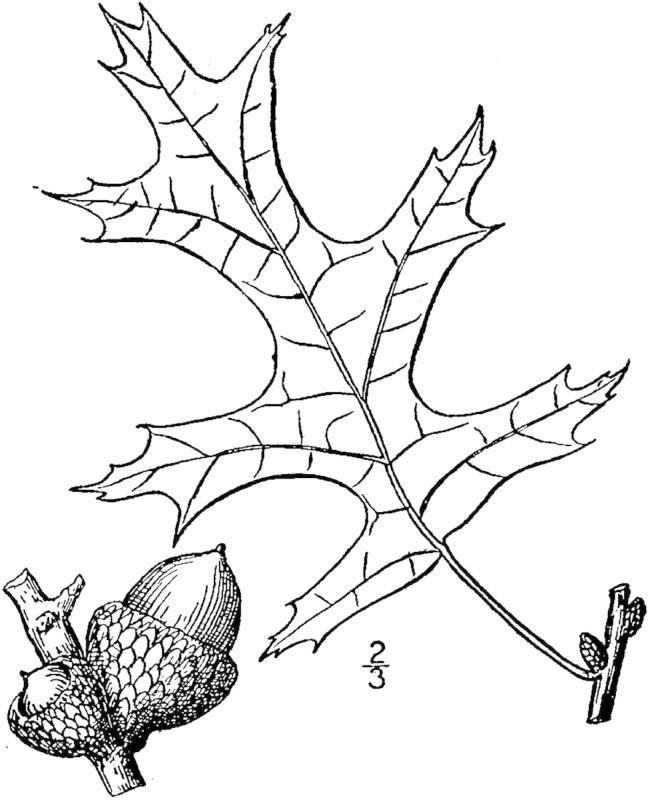
*Quercus coccinea* (from Britton and Brown 1913).

**Figure 212b.**
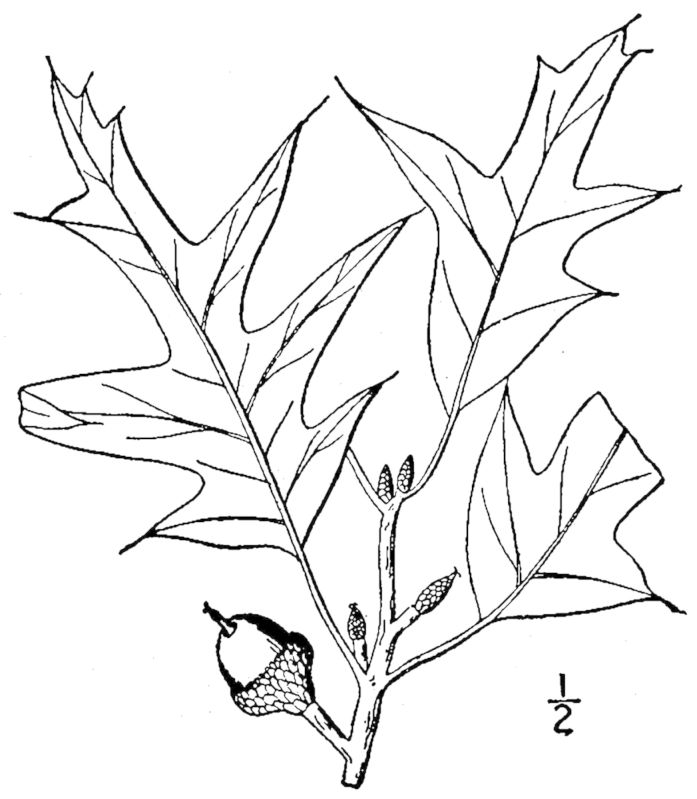
*Quercus falcata* (from Britton and Brown 1913).

**Figure 212c.**
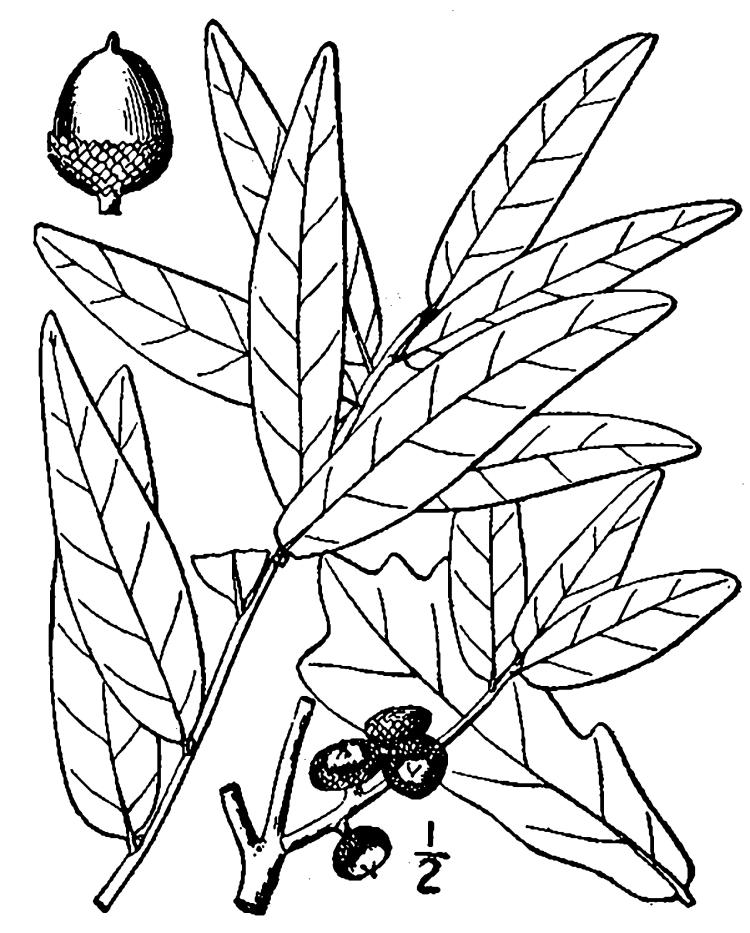
*Quercus laurifolia* (from Britton and Brown 1913).

**Figure 212d.**
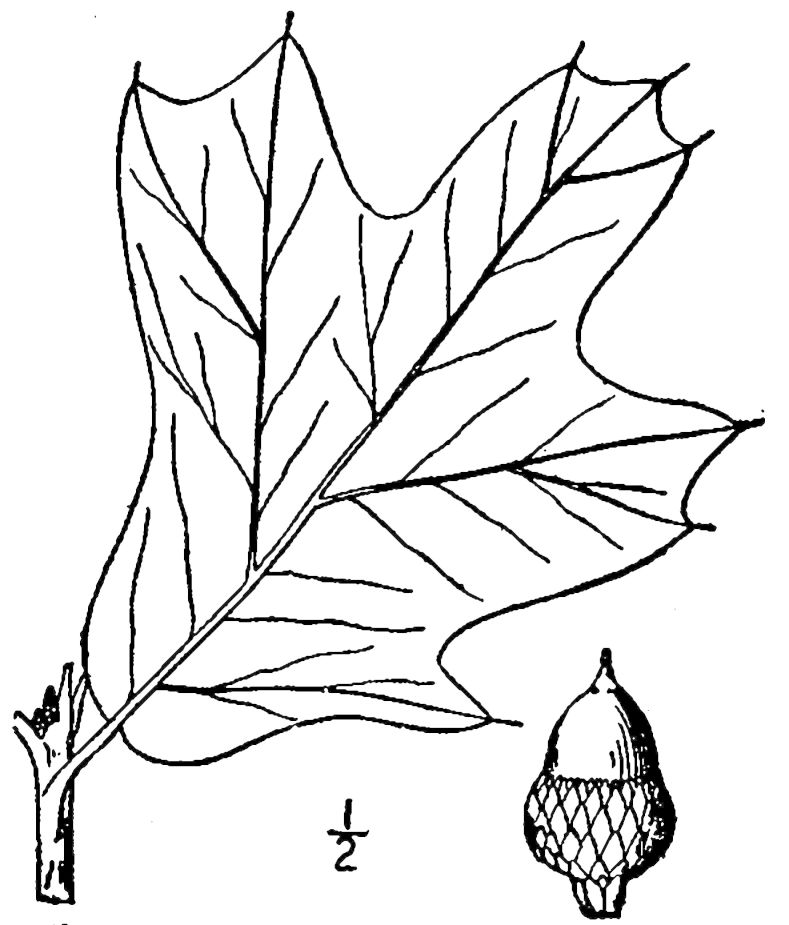
*Quercus marilandica* (from Britton and Brown 1913).

**Figure 213a.**
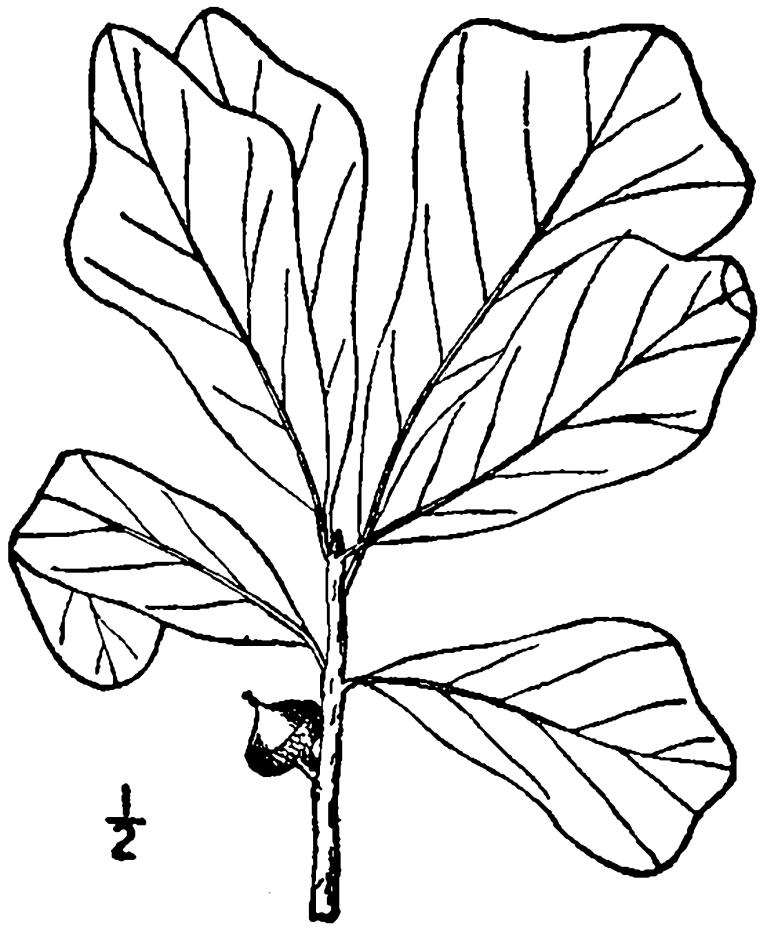
*Quercus nigra* (from Britton and Brown 1913).

**Figure 213b.**
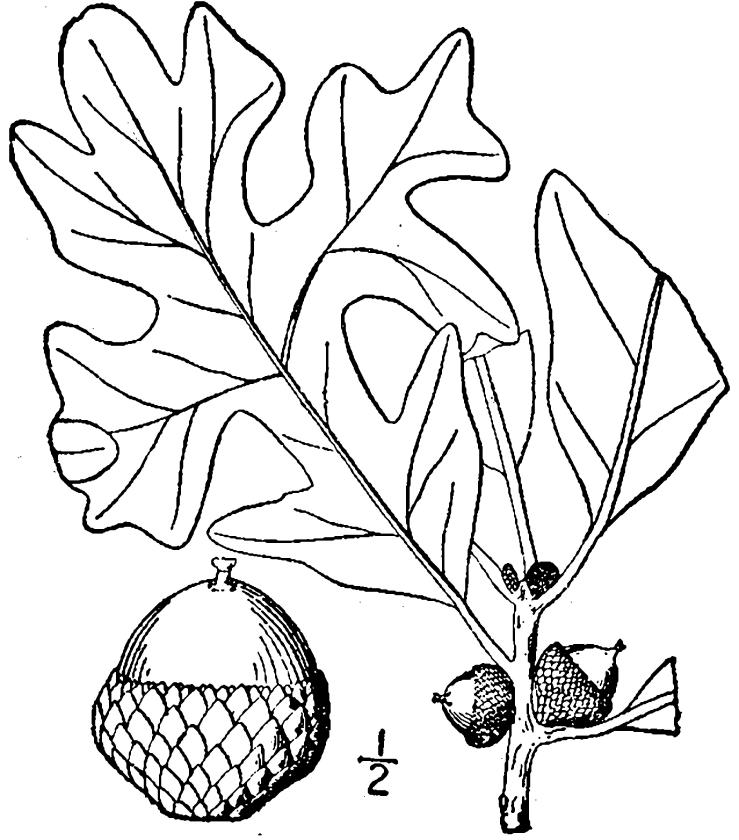
*Quercus stellata* (from Britton and Brown 1913).

**Figure 213c.**
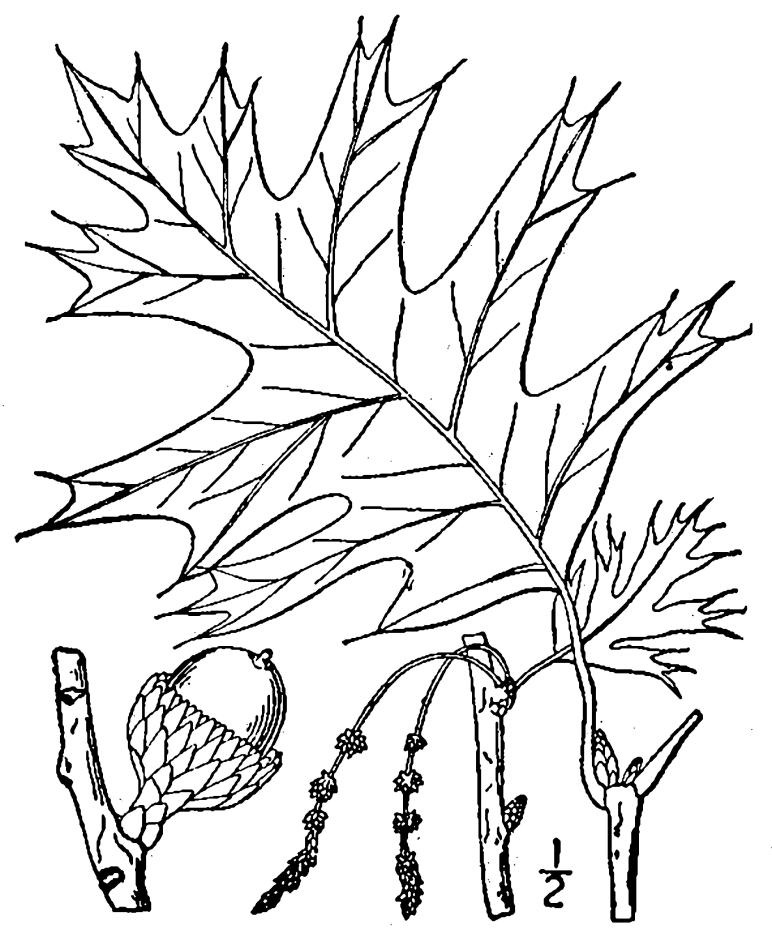
*Quercus velutina* (from Britton and Brown 1913).

**Figure 214a.**
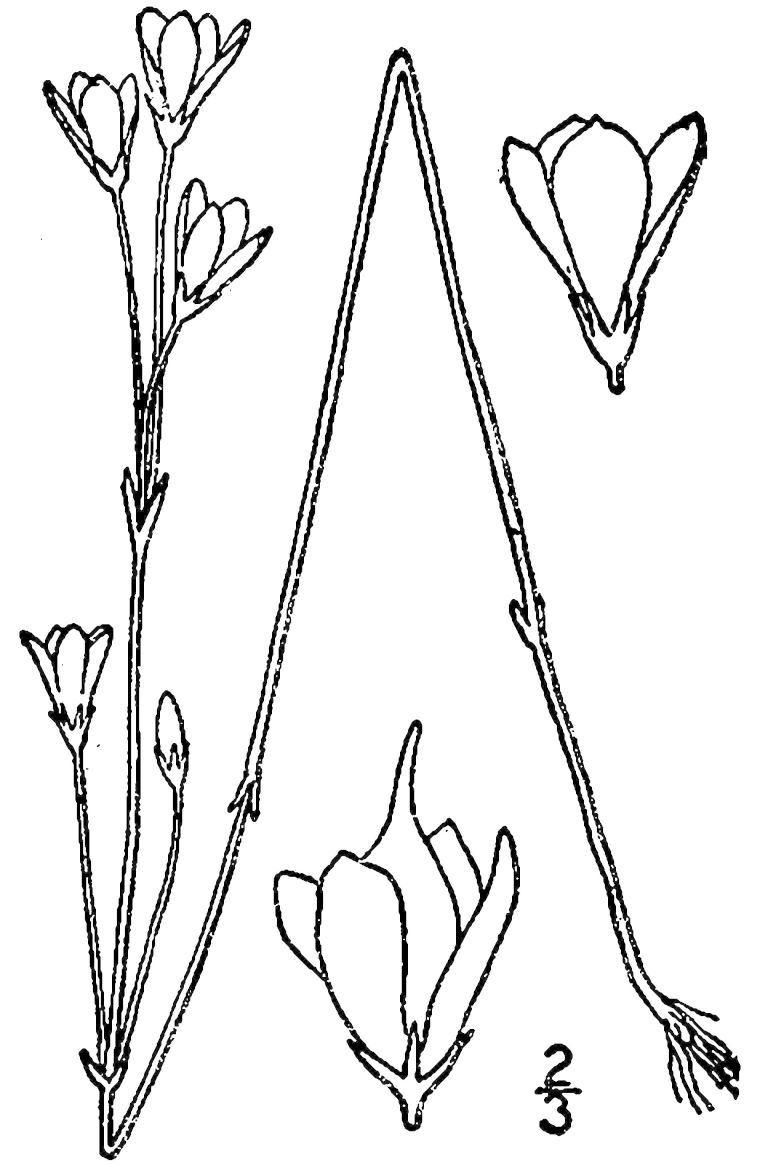
*Bartonia verna* (from Britton and Brown 1913).

**Figure 214b.**
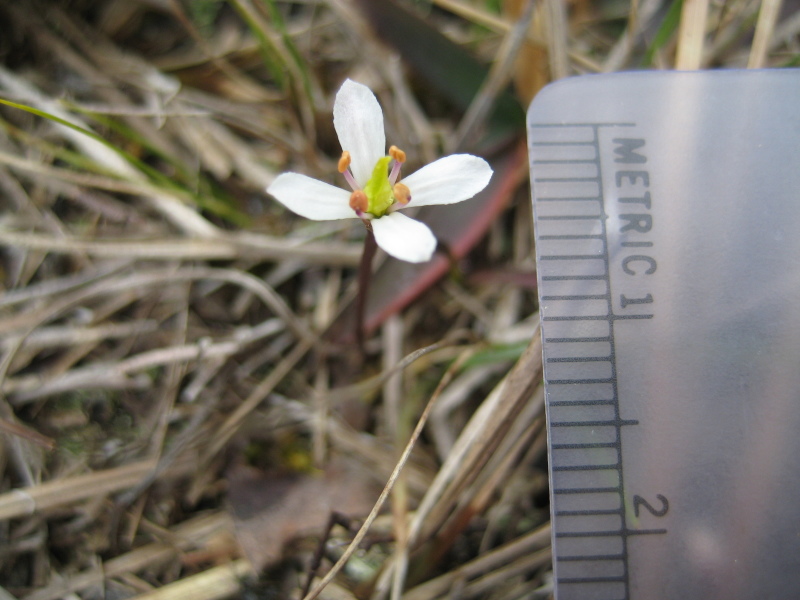
*Bartonia virginica* (photo by R. Thornhill).

**Figure 214c.**
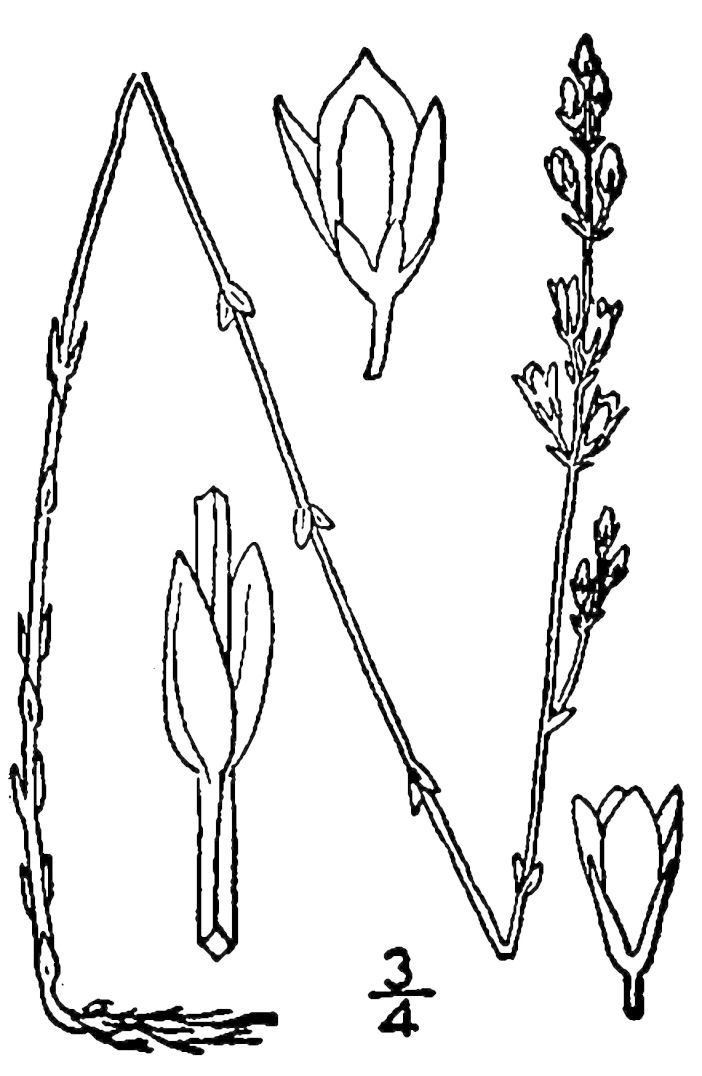
*Bartonia virginica* (from Britton and Brown 1913).

**Figure 215a.**
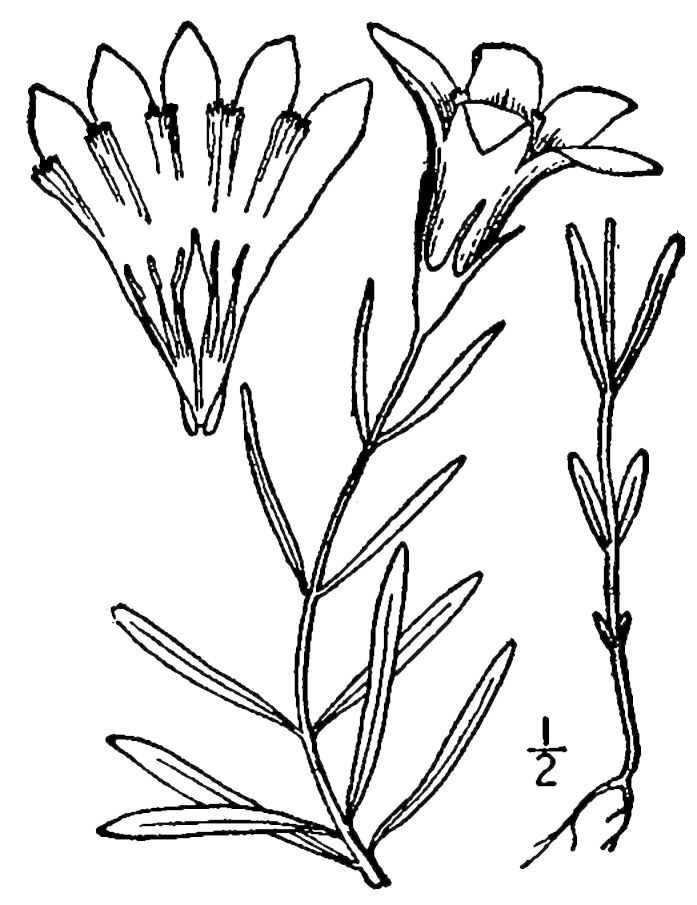
*Gentiana autumnalis* (from Britton and Brown 1913).

**Figure 215b.**
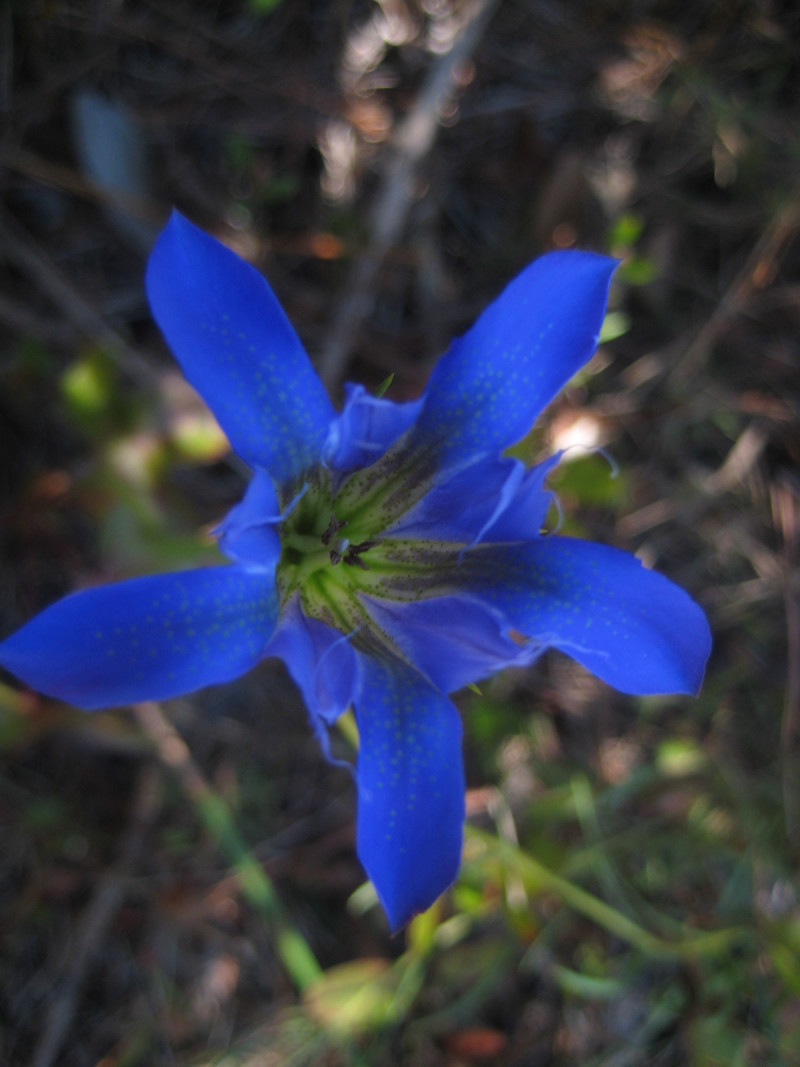
*Gentiana autumnalis* (photo by R. Thornhill).

**Figure 215c.**
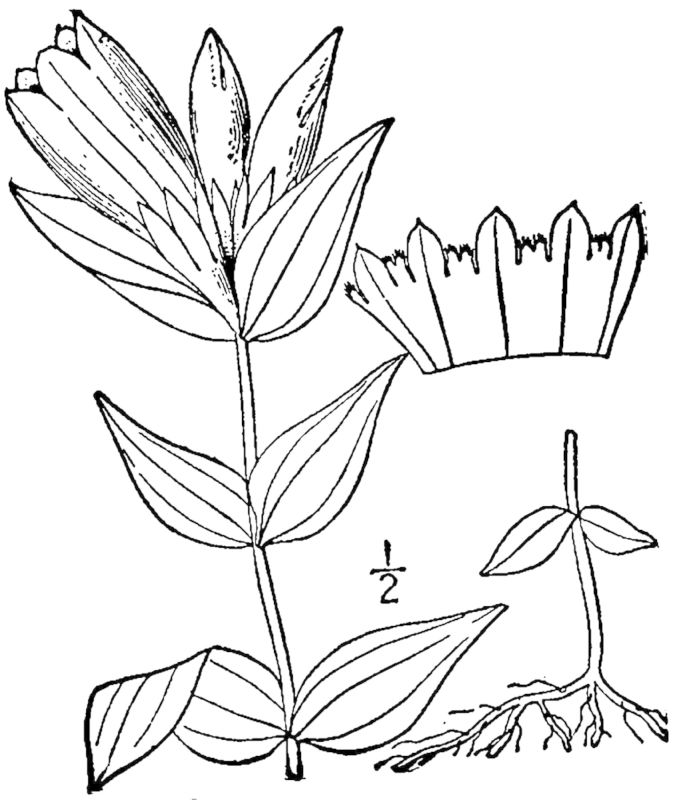
*Gentiana catesbaei* (from Britton and Brown 1913).

**Figure 215d.**
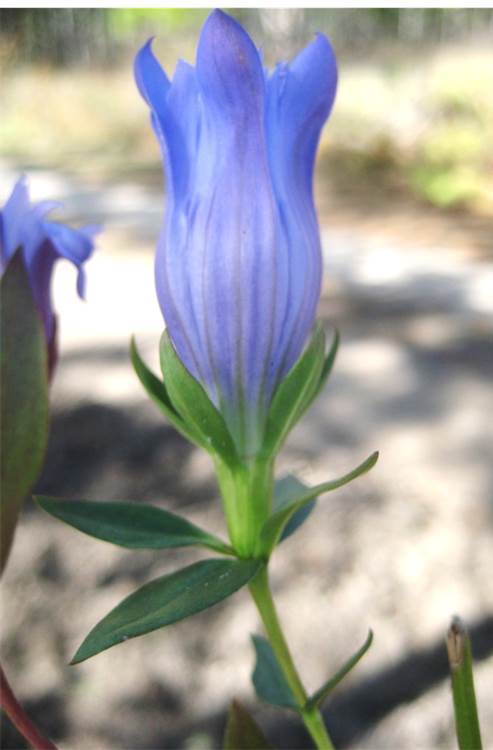
*Gentiana catesbaei* (photo by R. Thornhill).

**Figure 215e.**
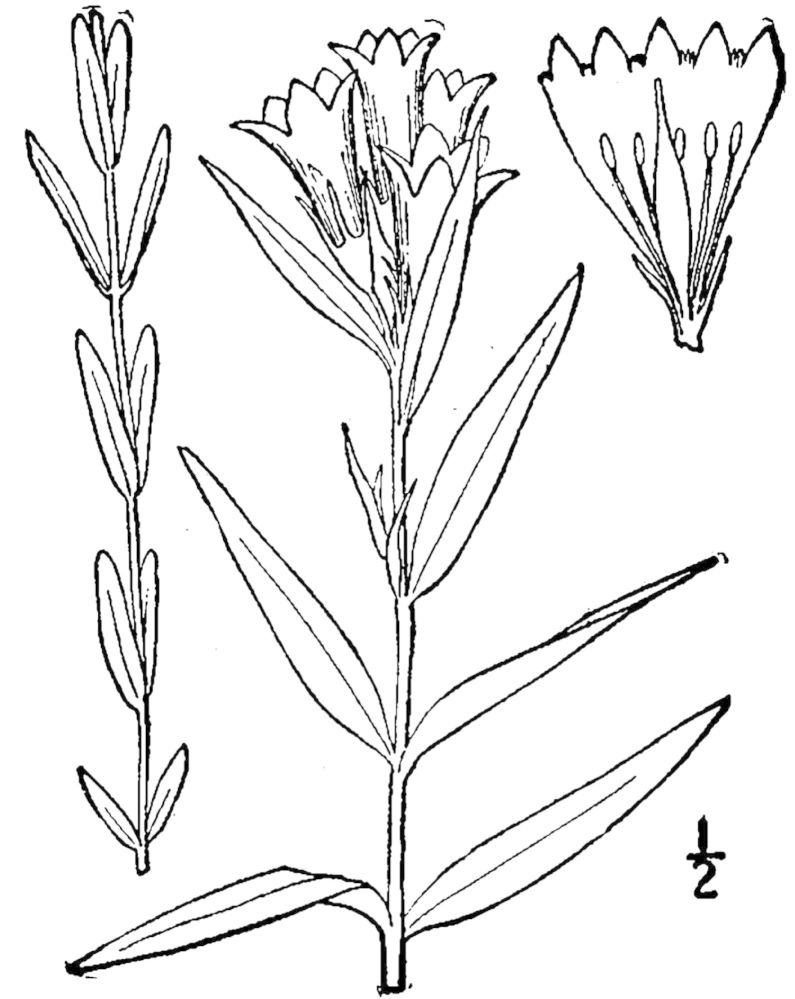
*Gentiana saponaria* (from Britton and Brown 1913).

**Figure 216a.**
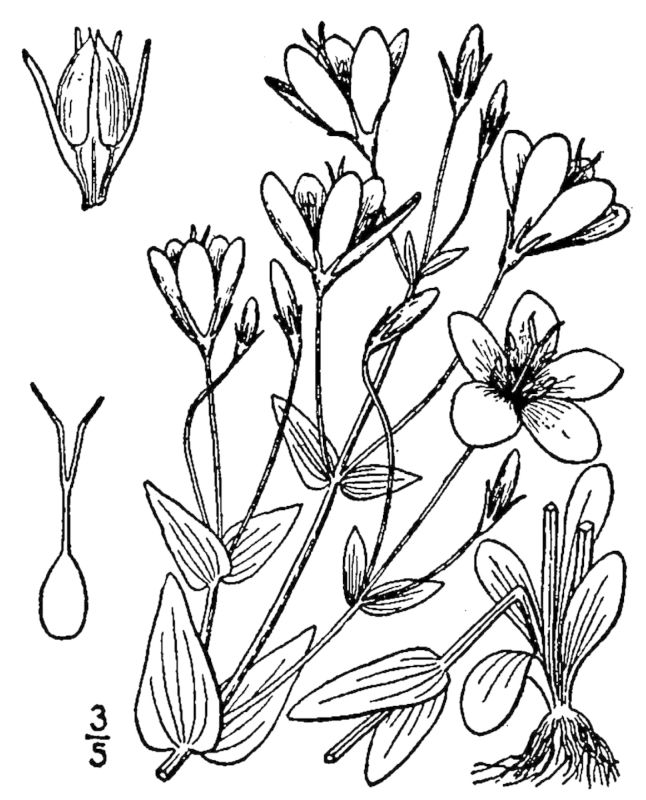
*Sabatia angularis* (from Britton and Brown 1913).

**Figure 216b.**
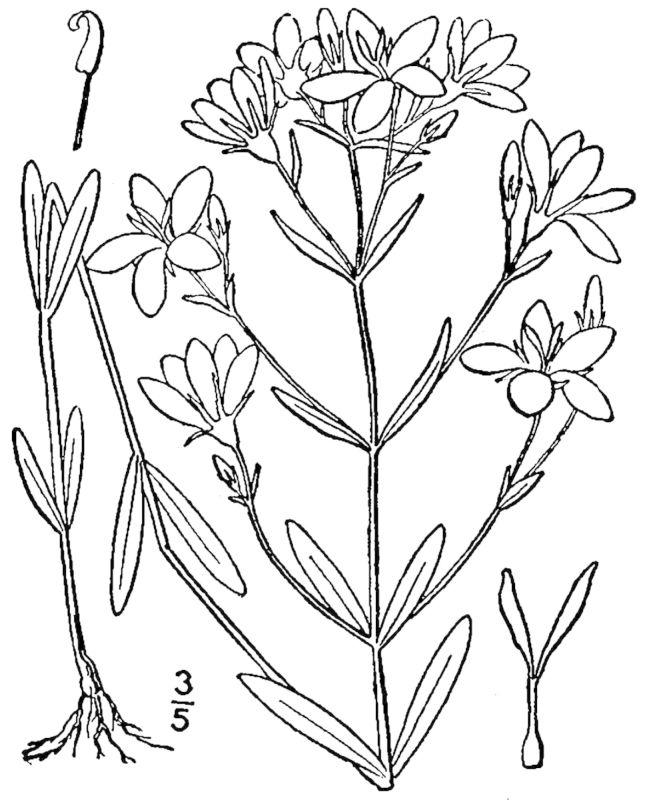
*Sabatia brachiata* (from Britton and Brown 1913).

**Figure 216c.**
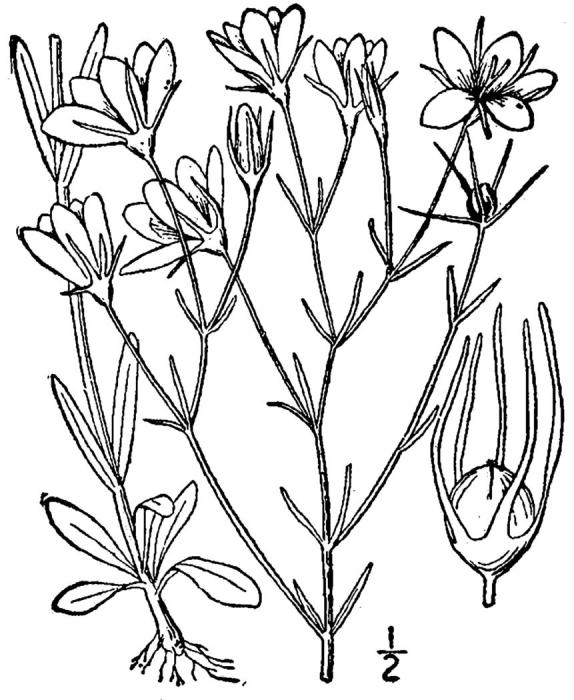
*Sabatia campanulata* (from Britton and Brown 1913).

**Figure 216d.**
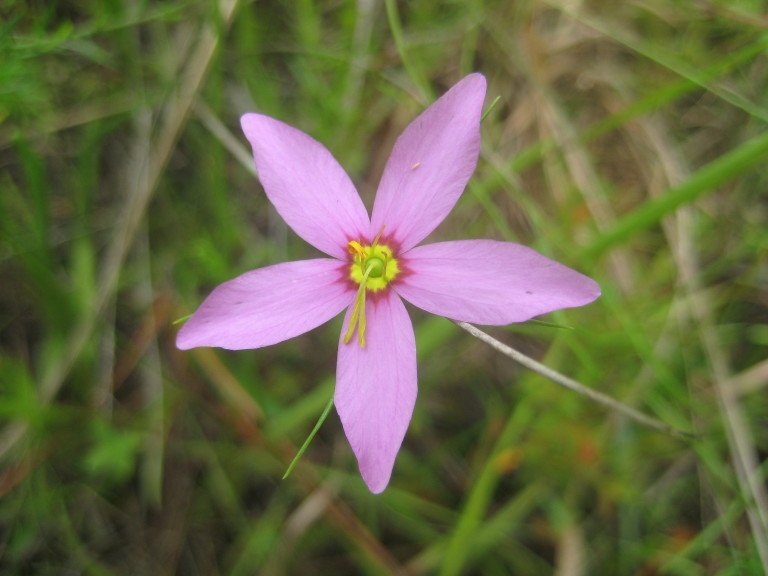
*Sabatia campanulata* (photo by R. Thornhill).

**Figure 217a.**
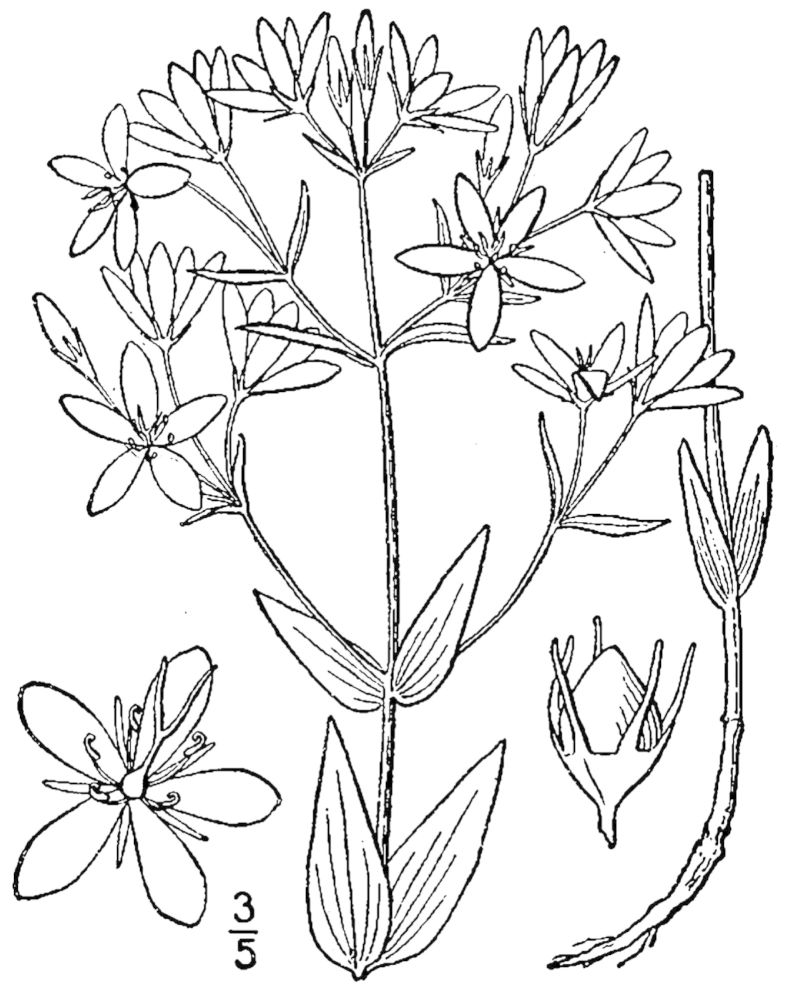
*Sabatia difformis* (from Britton and Brown 1913).

**Figure 217b.**
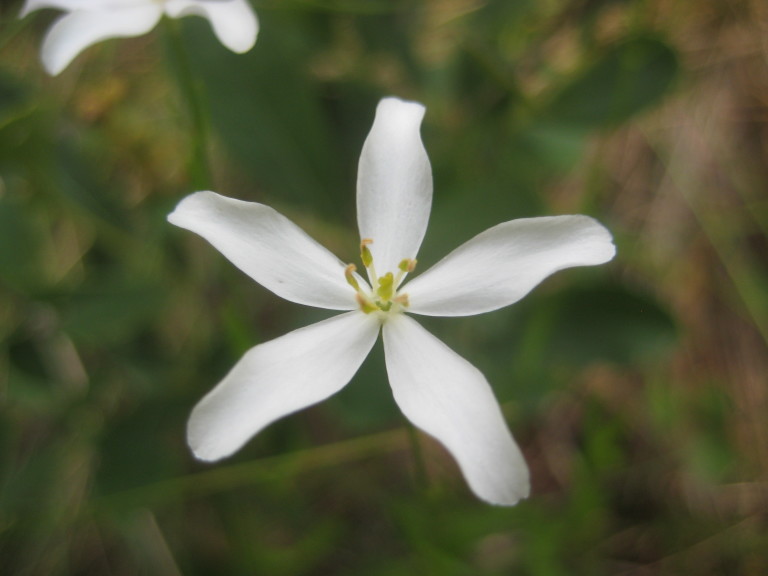
*Sabatia difformis* (photo by R. Thornhill).

**Figure 217c.**
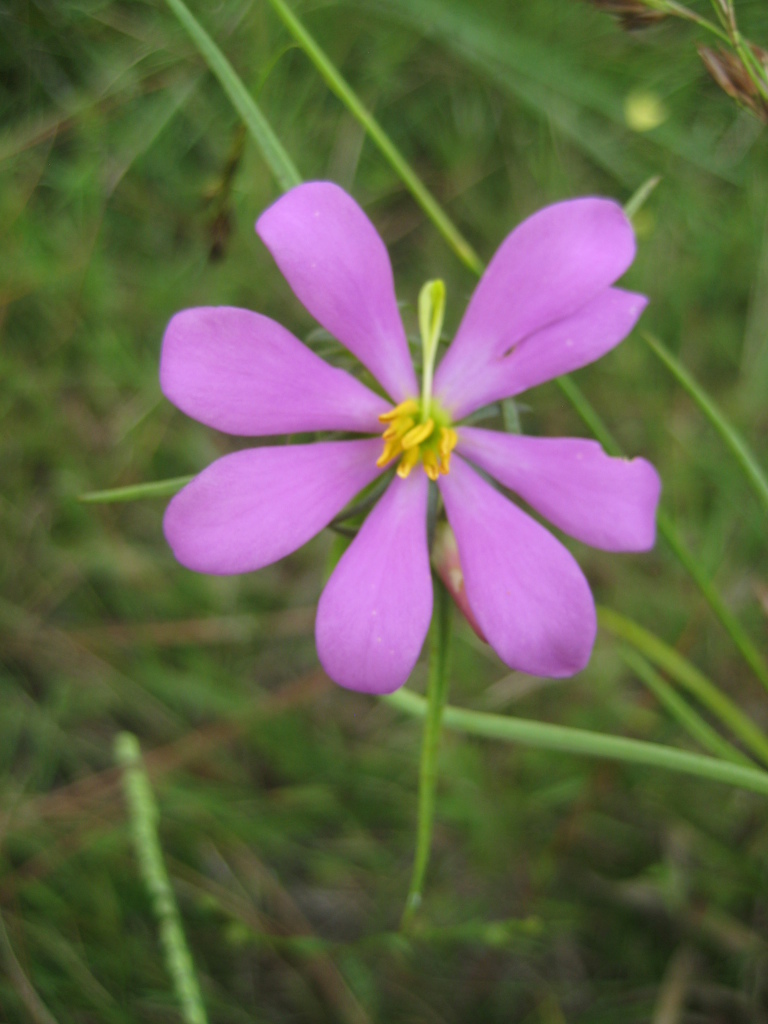
*Sabatia gentianoides* (photo by R. Thornhill).

**Figure 218.**
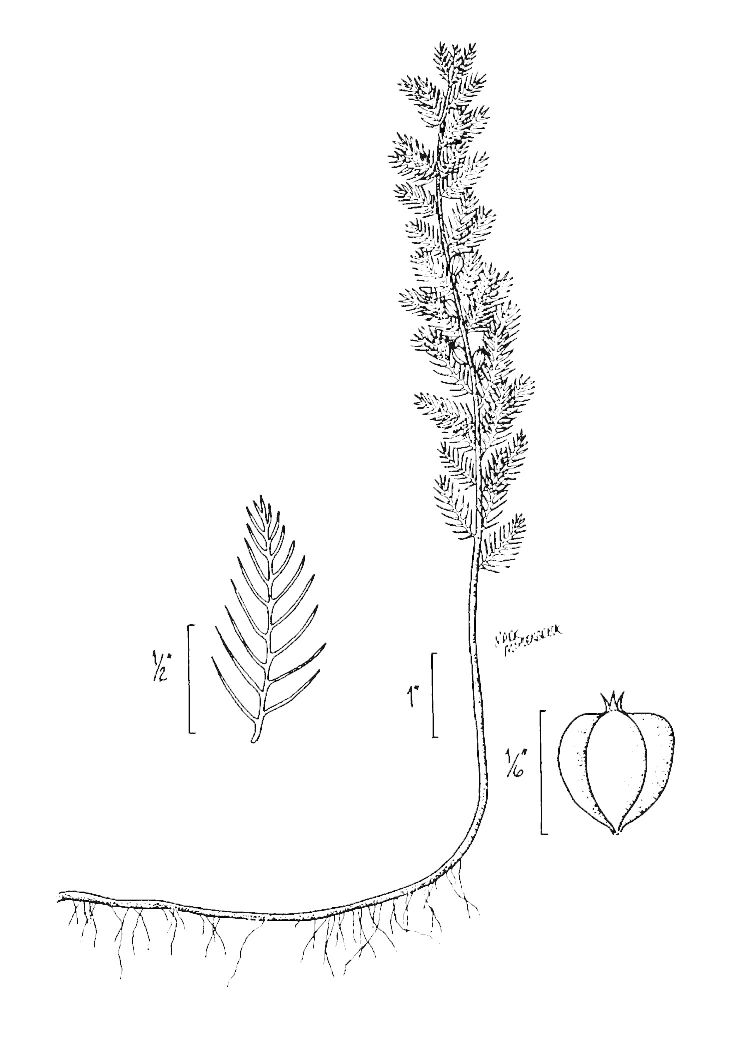
*Proserpinaca pectinata* (from USDA-NRCS 2012).

**Figure 219a.**
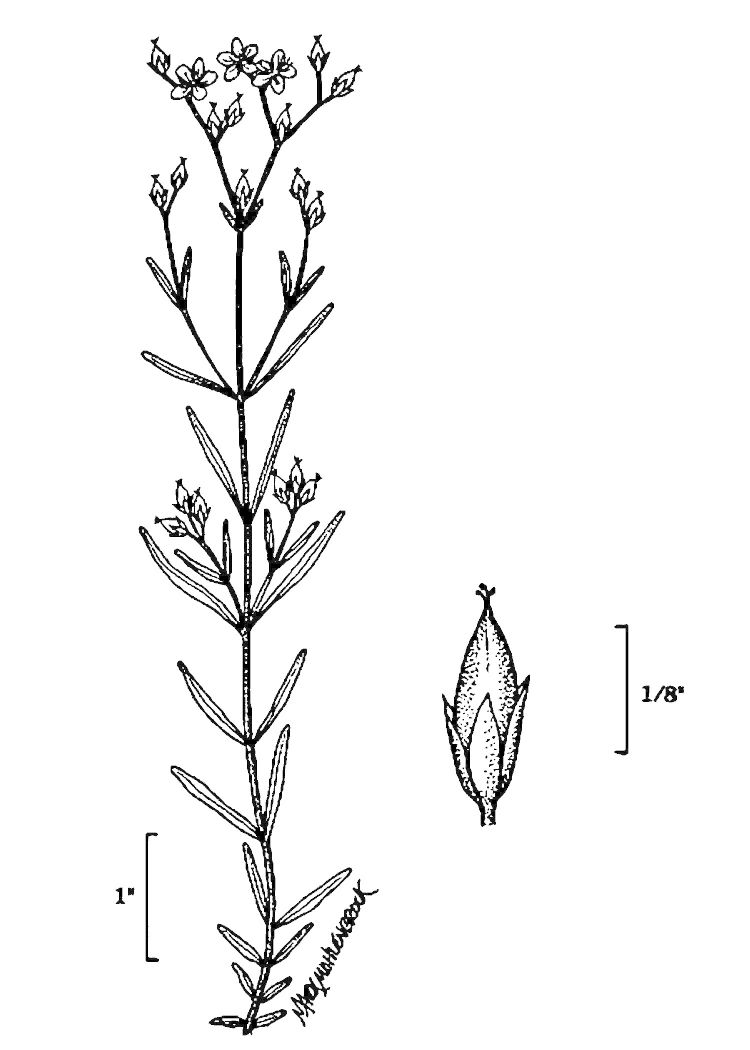
*Hypericum canadense* (from USDA-NRCS 2012).

**Figure 219b.**
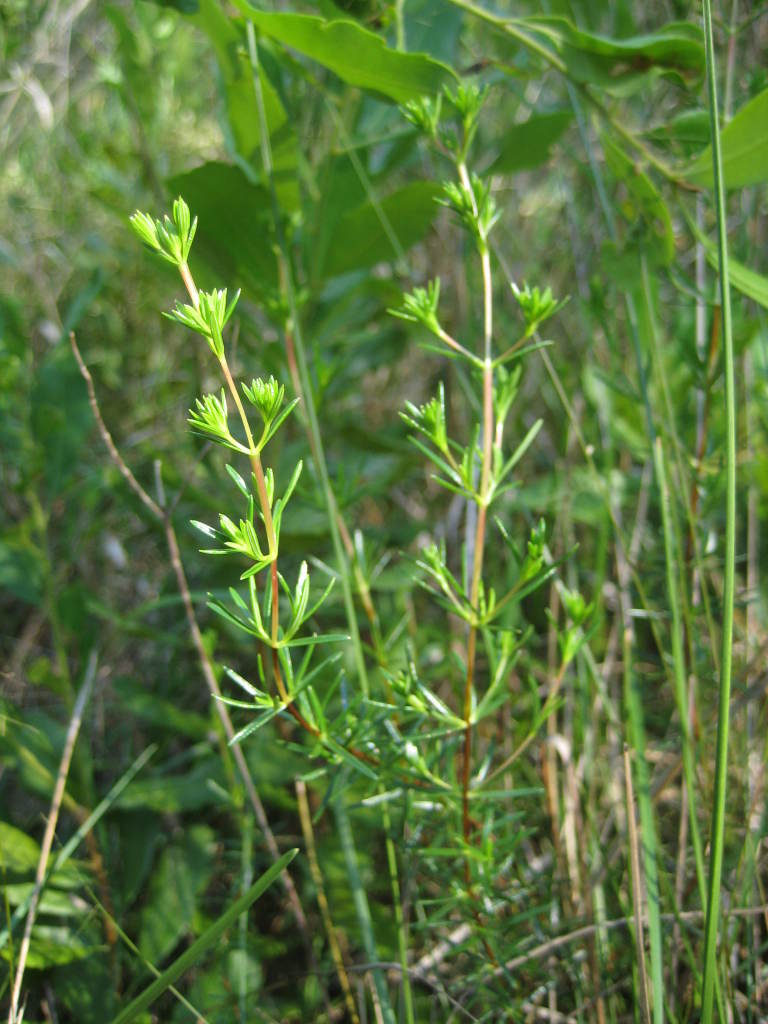
*Hypericum brachyphyllum* (photo by R. Thornhill).

**Figure 219c.**
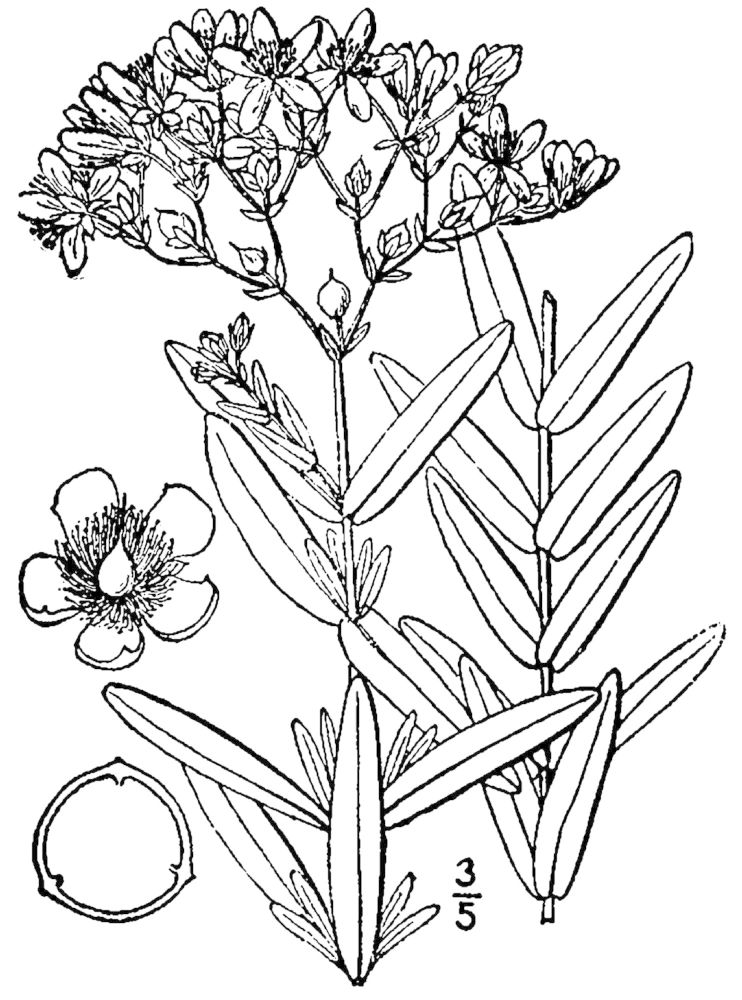
*Hypericum cistifolium* (from Britton and Brown 1913).

**Figure 219d.**
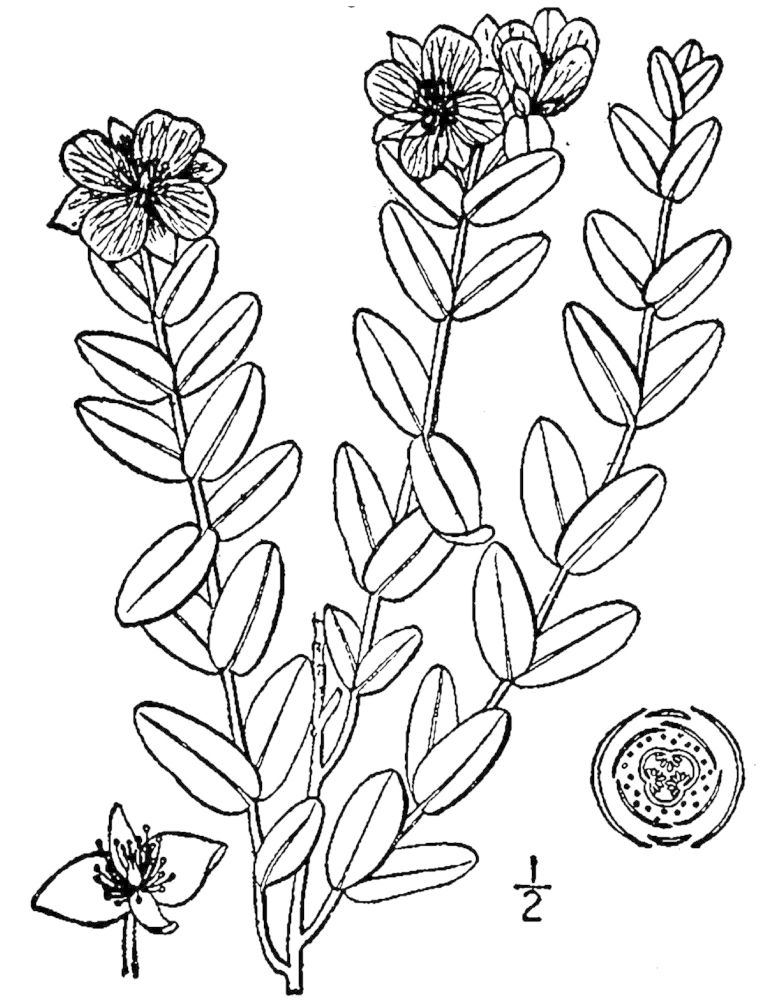
*Hypericum crux-andreae* (from Britton and Brown 1913).

**Figure 219e.**
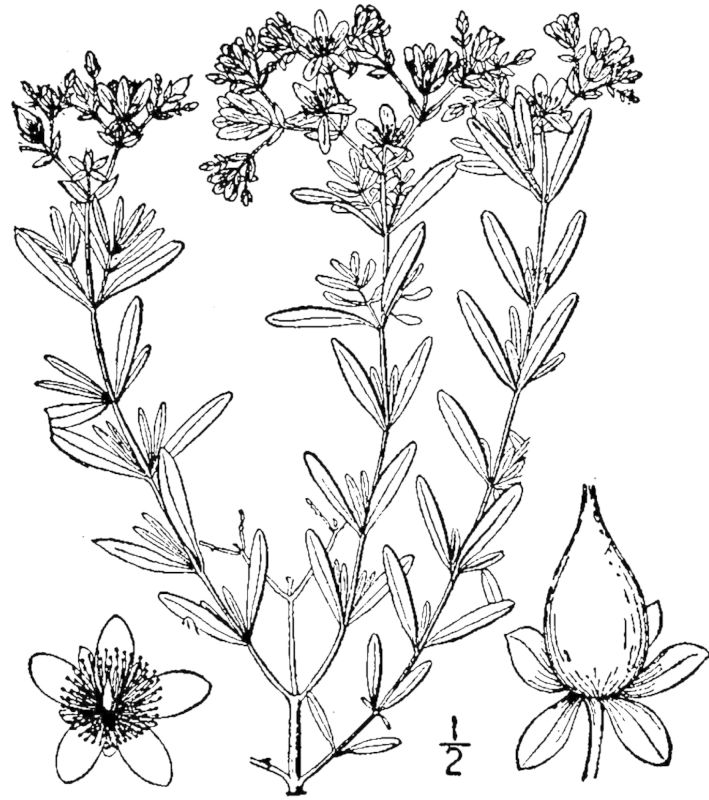
*Hypericum densiflorum* (from Britton and Brown 1913).

**Figure 219f.**
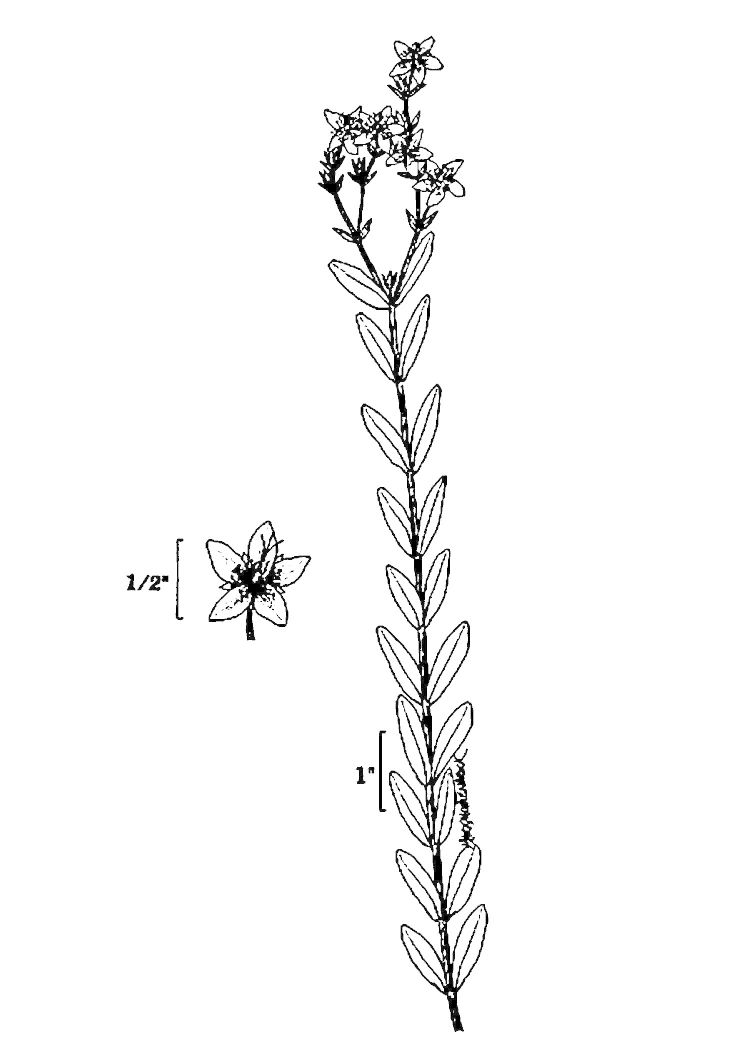
*Hypericum denticulatum* (from USDA-NRCS 2012).

**Figure 220a.**
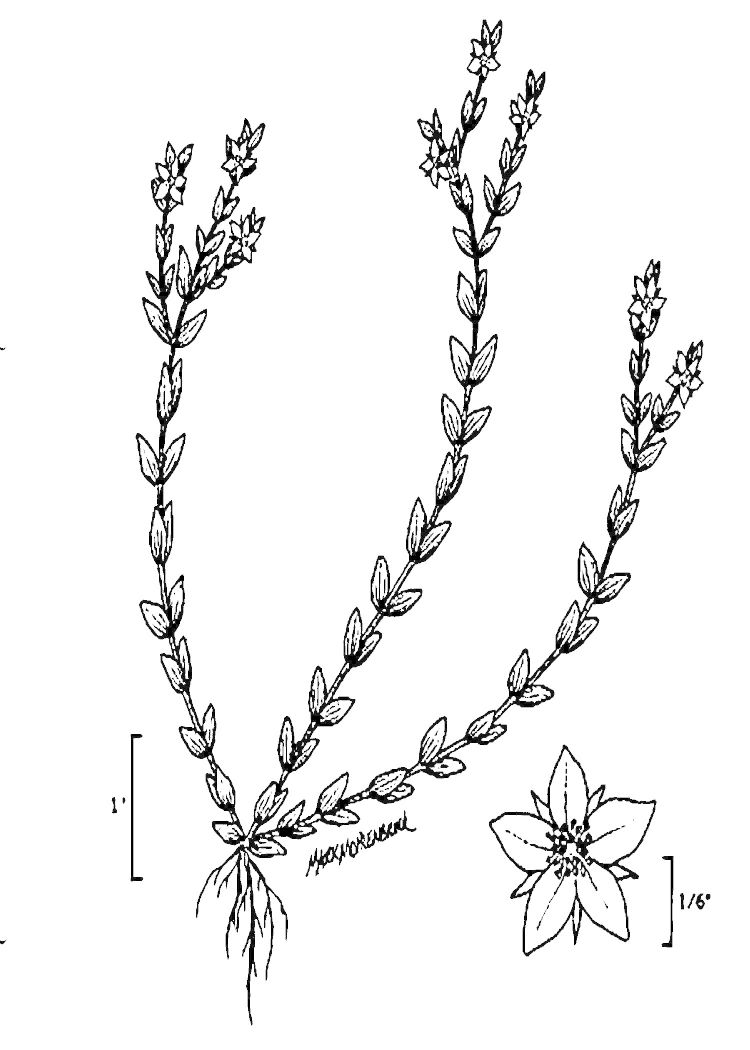
*Hypericum galioides* (from USDA-NRCS 2012).

**Figure 220b.**
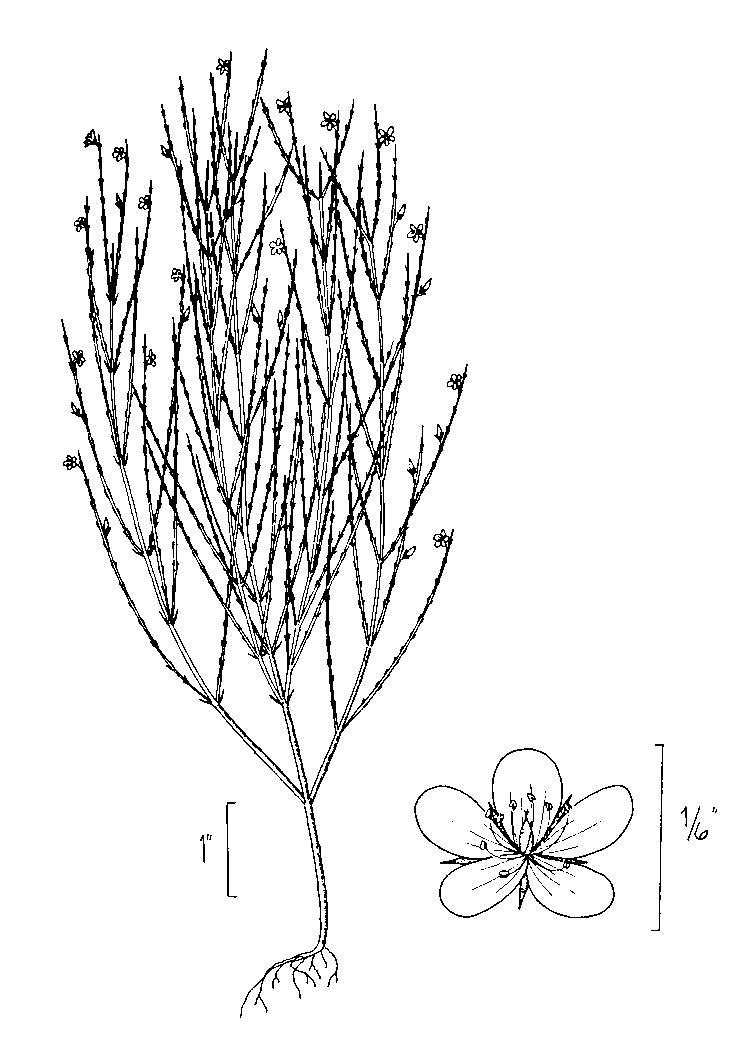
*Hypericum gentianoides* (from USDA-NRCS 2012).

**Figure 220c.**
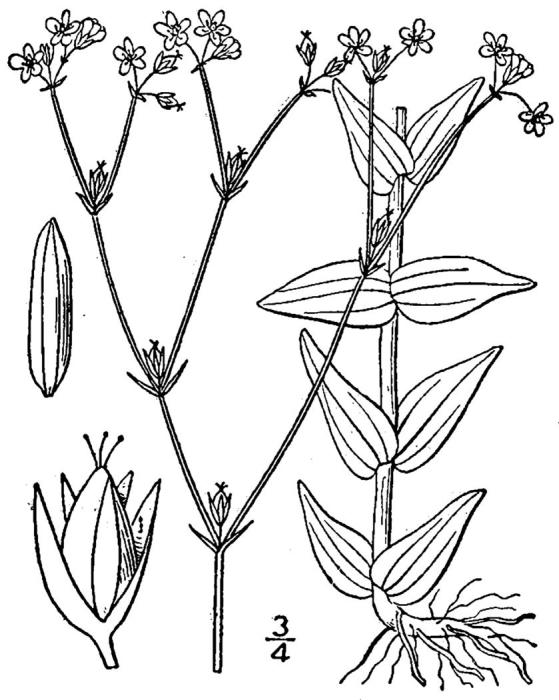
*Hypericum gymnanthum* (from Britton and Brown 1913).

**Figure 220d.**
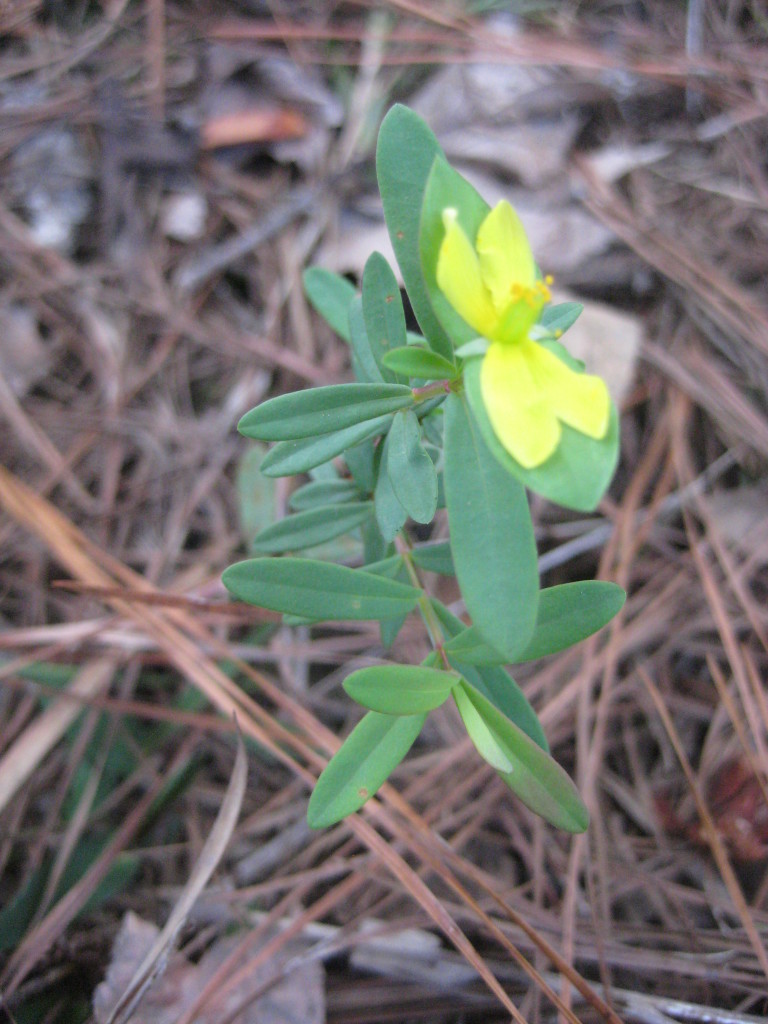
*Hypericum hypericoides* (photo by R. Thornhill).

**Figure 221a.**
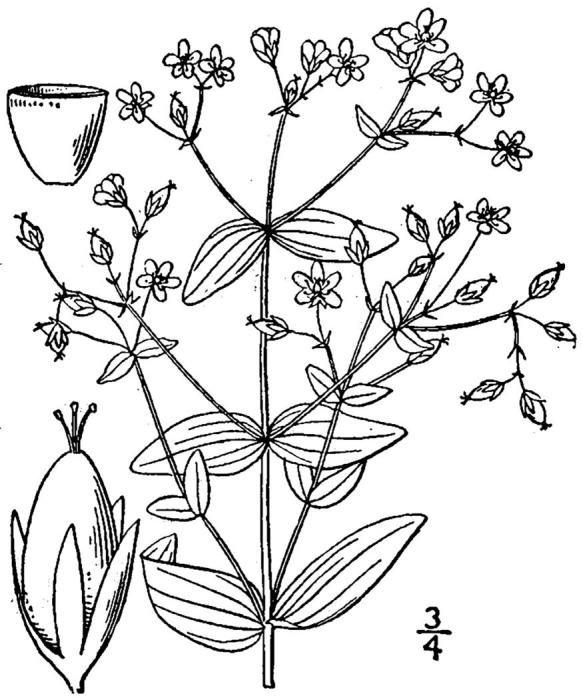
*Hypericum mutilum* (from Britton and Brown 1913).

**Figure 221b.**
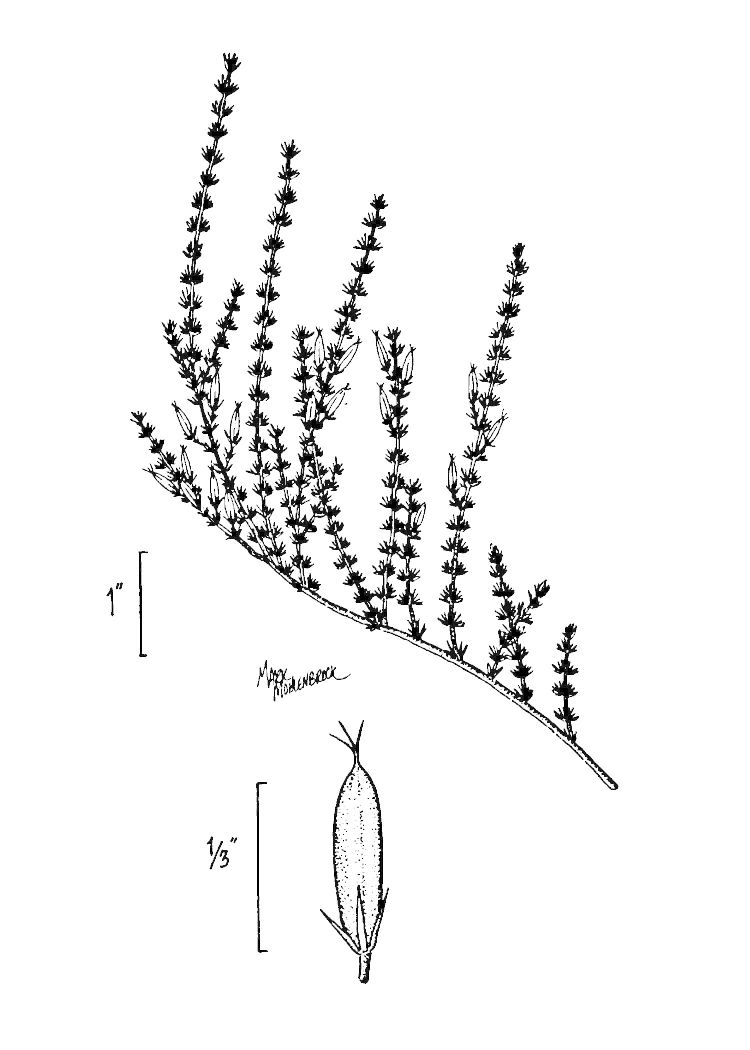
*Hypericum tenuifolium* (from USDA-NRCS 2012).

**Figure 221c.**
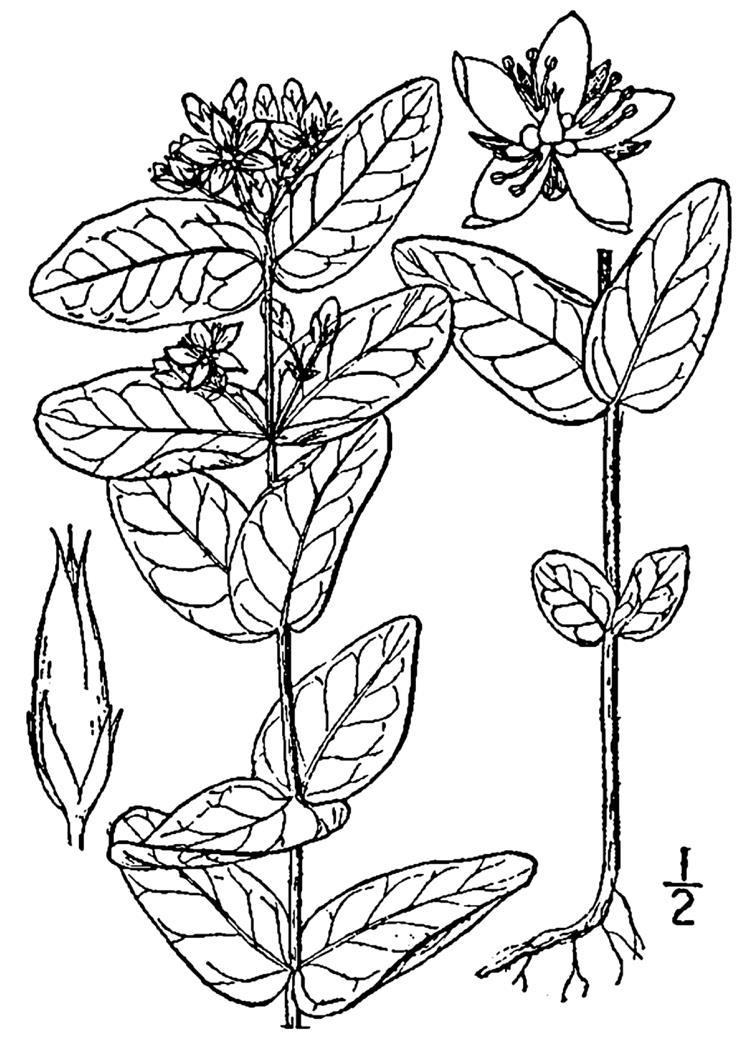
*Hypericum virginicum* (from Britton and Brown 1913).

**Figure 222a.**
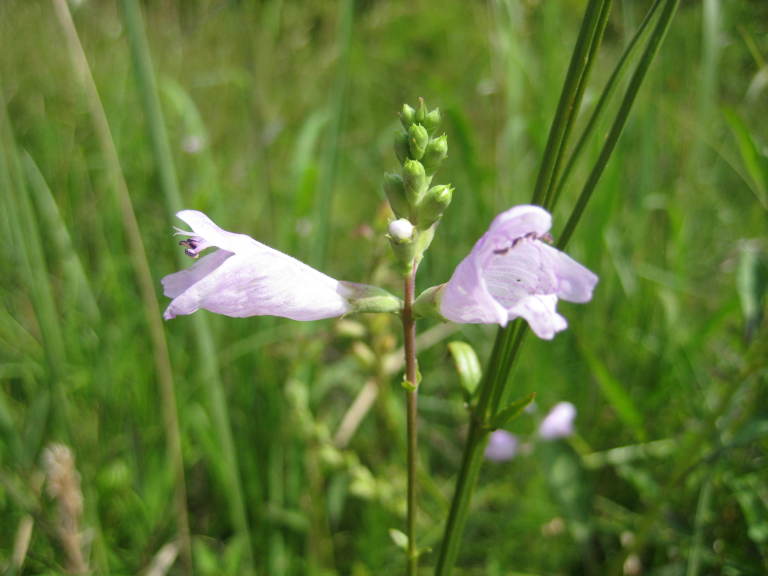
Inflorescence (photo by R. Thornhill).

**Figure 222b.**
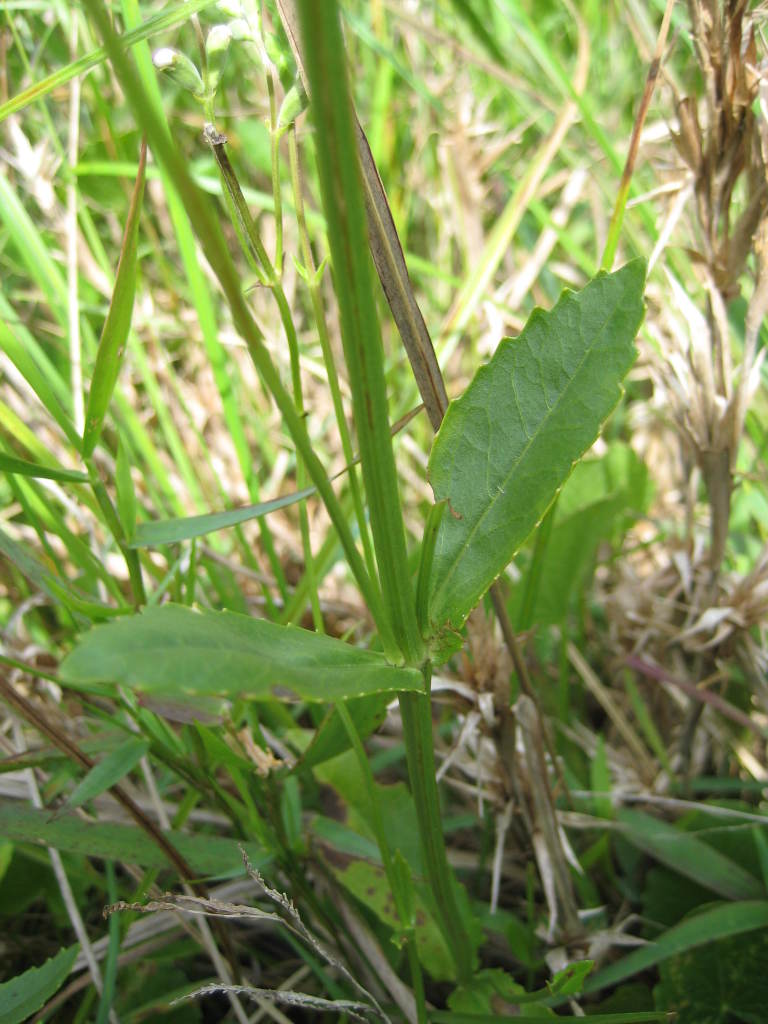
Cauline leaves (photo by R. Thornhill).

**Figure 223a.**
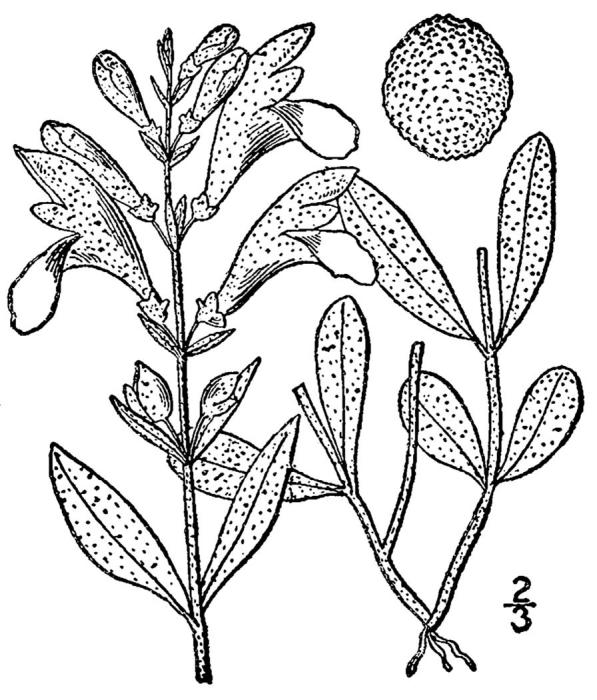
*Scutellaria integrifolia* (from Britton and Brown 1913).

**Figure 223b.**
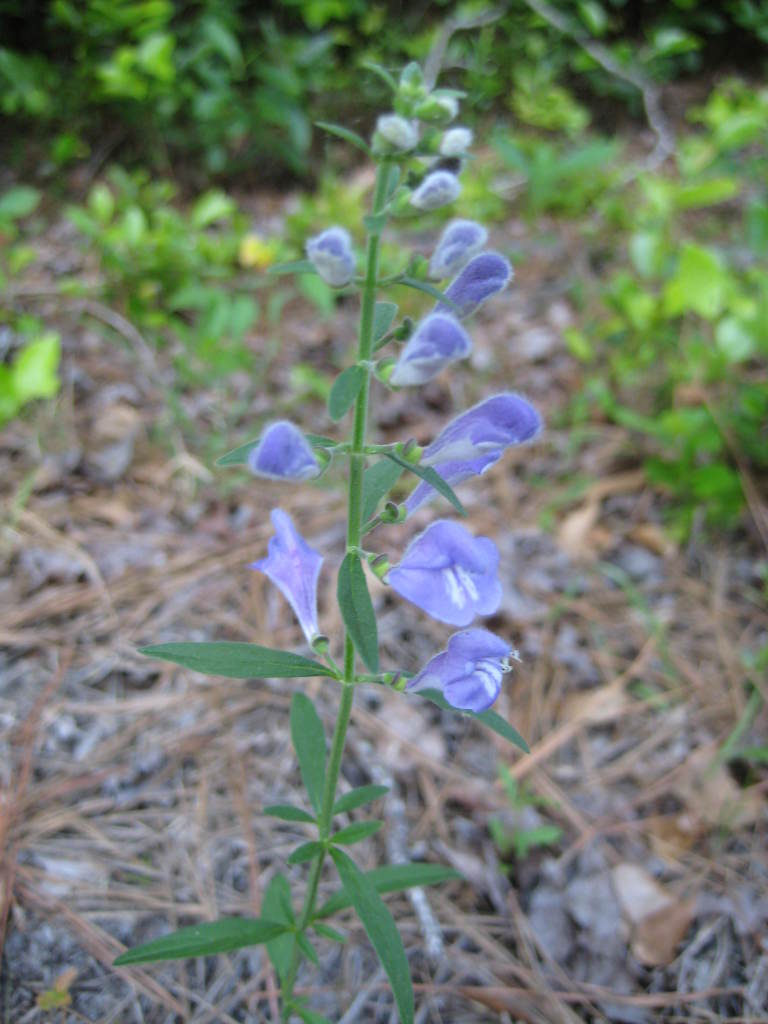
Photo by R. Thornhill.

**Figure 224a.**
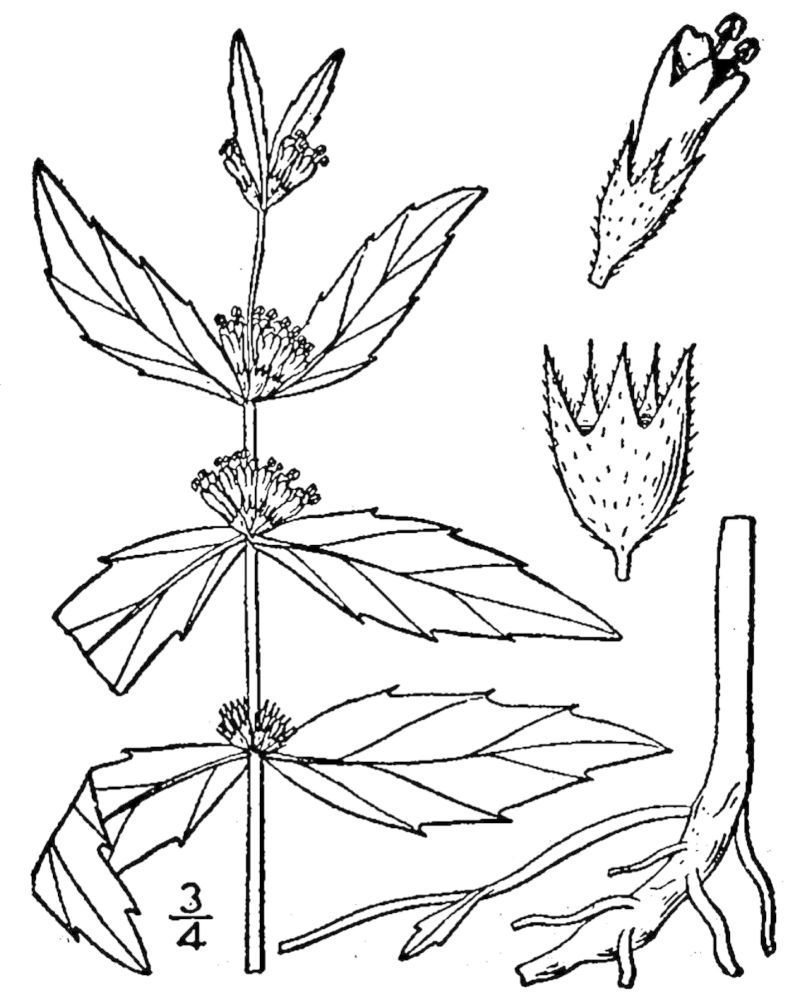
*Lycopus amplectens* (from Britton and Brown 1913).

**Figure 224b.**
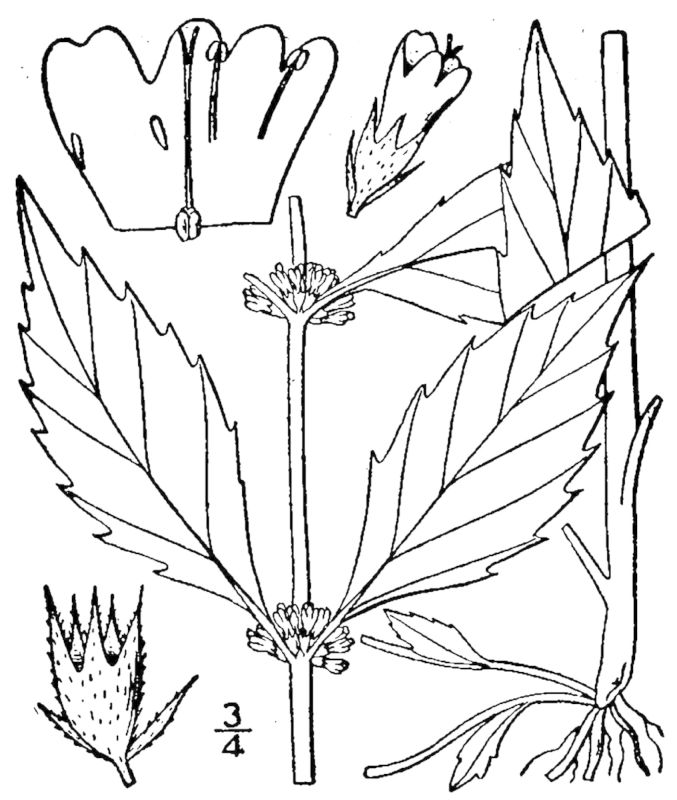
*Lycopus rubellus* (from Britton and Brown 1913).

**Figure 225a.**
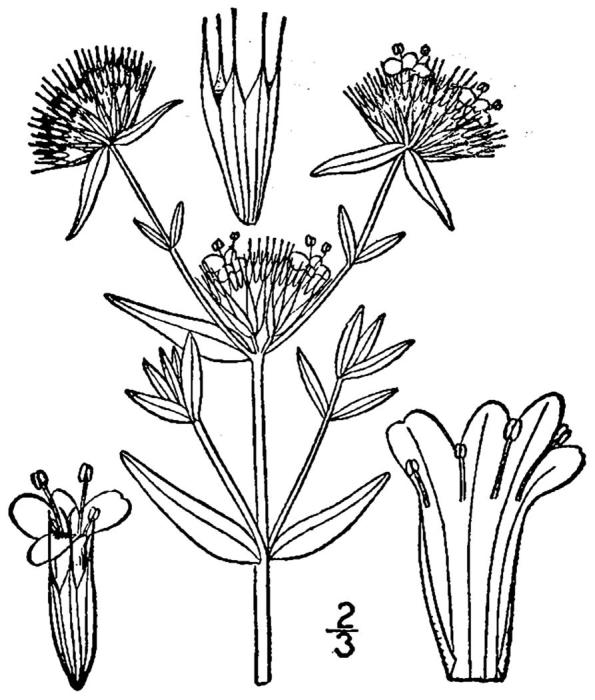
*Pycnanthemum flexuosum* (from Britton and Brown 1913).

**Figure 225b.**
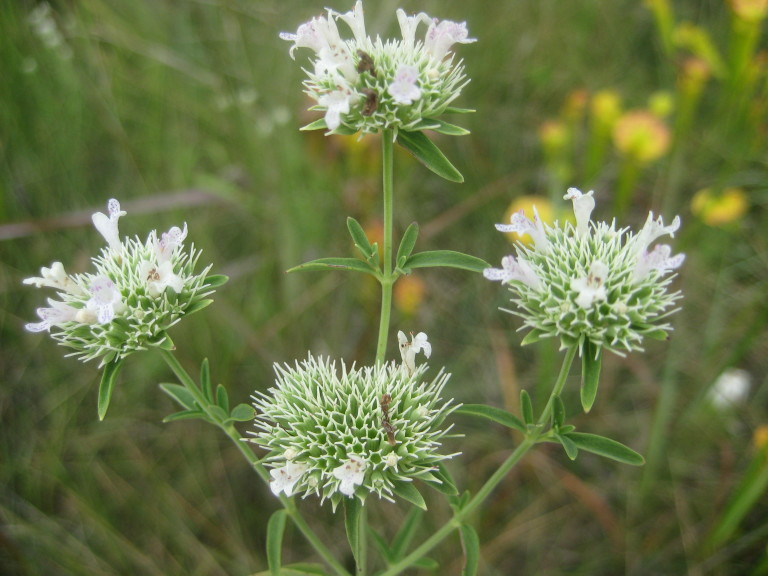
*Pycnanthemum flexuosum* (photo by R. Thornhill).

**Figure 225c.**
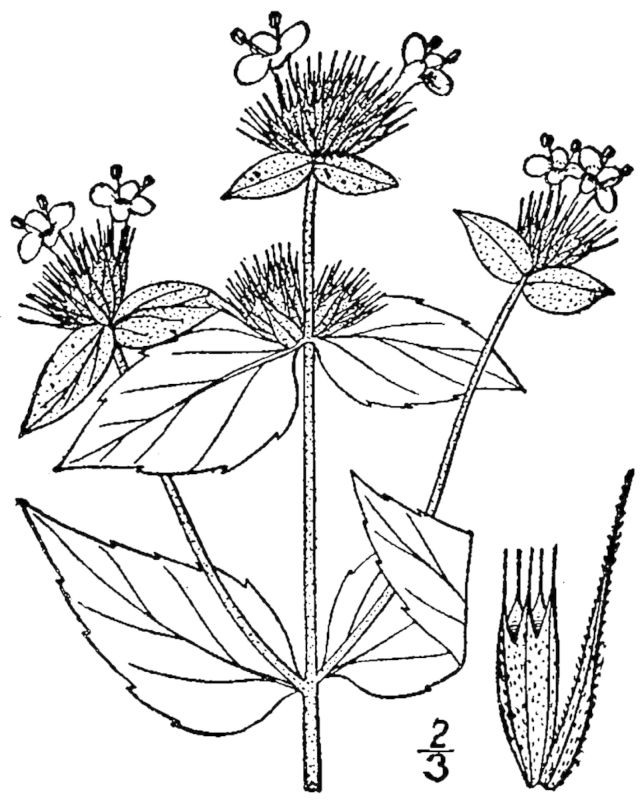
*Pycnanthemum setosum* (from Britton and Brown 1913).

**Figure 225d.**
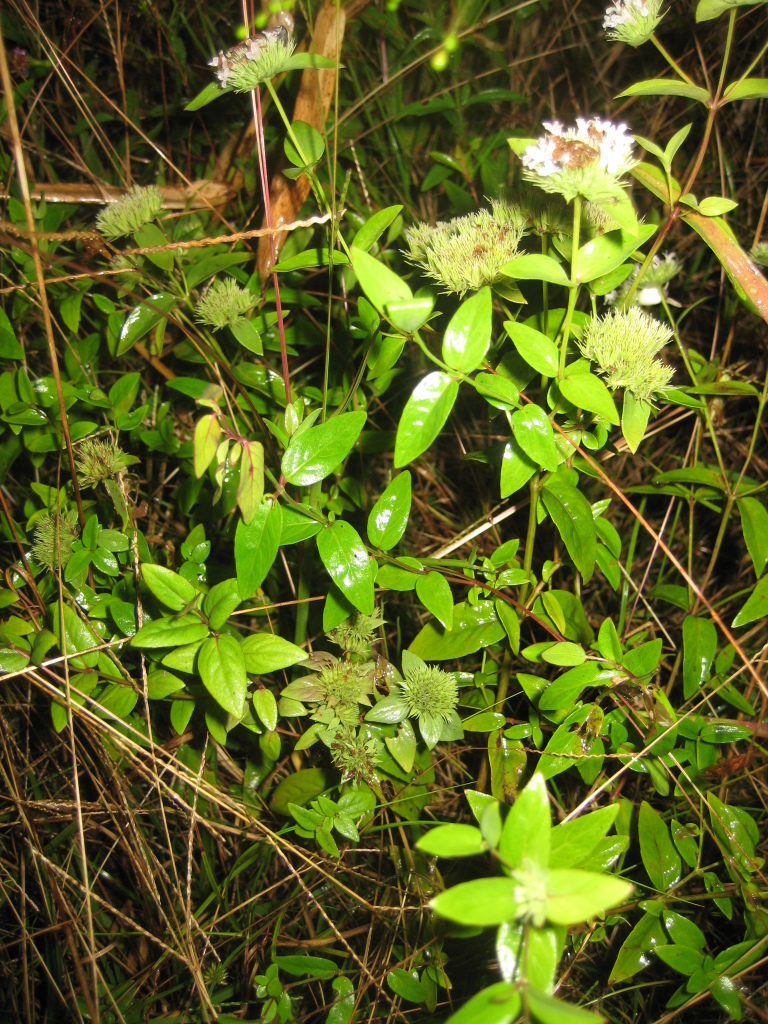
*Pycnanthemum setosum* (photo by R. Thornhill).

**Figure 226a.**
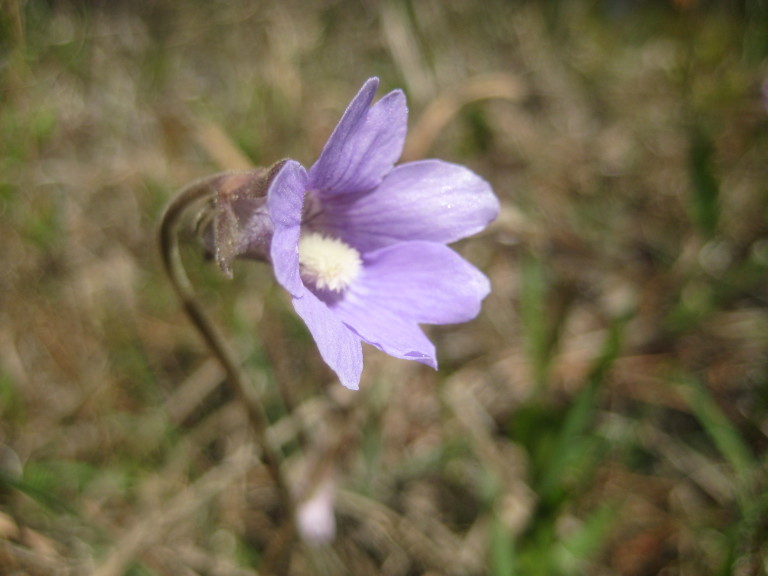
*Pinguicula cerulea* (photo by R. Thornhill).

**Figure 226b.**
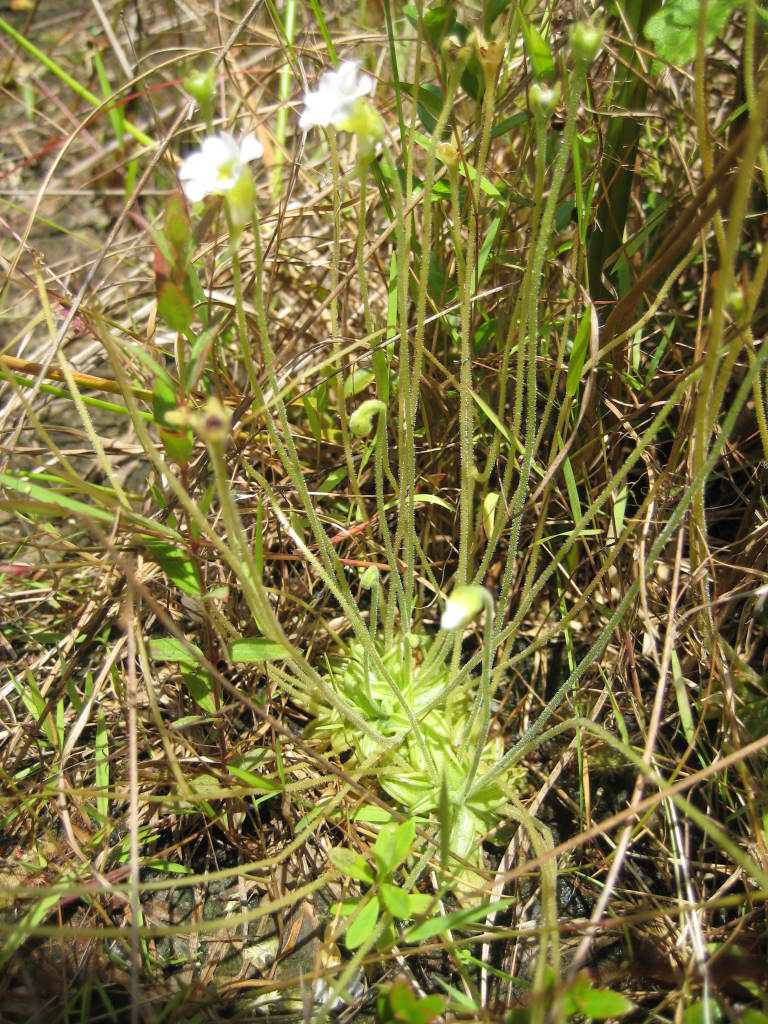
*Pinguicula pumila* (photo by R. Thornhill).

**Figure 227a.**
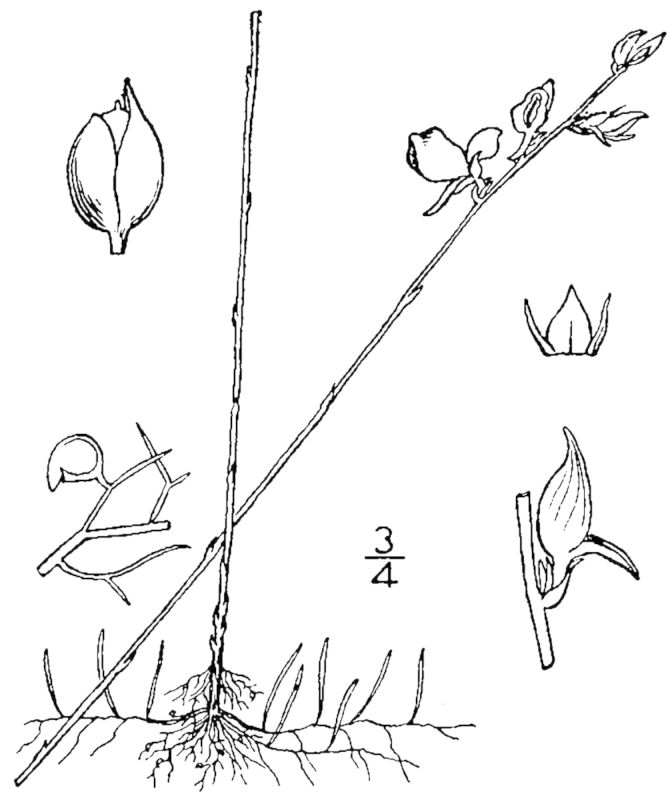
*Utricularia juncea* (from Britton and Brown 1913).

**Figure 227b.**
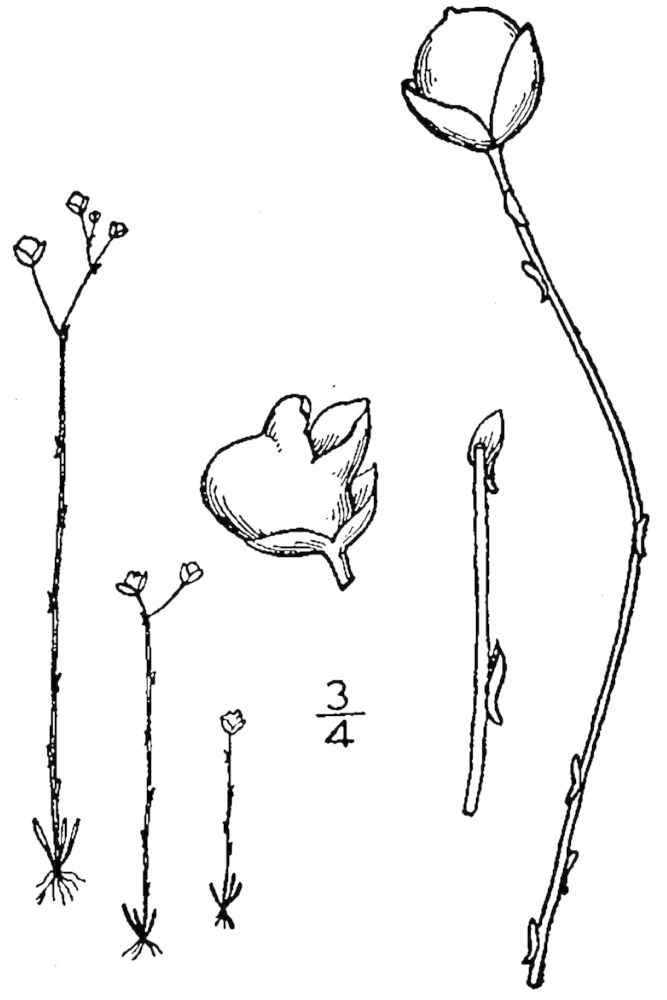
*Utricularia subulata* (from Britton and Brown 1913).

**Figure 228a.**
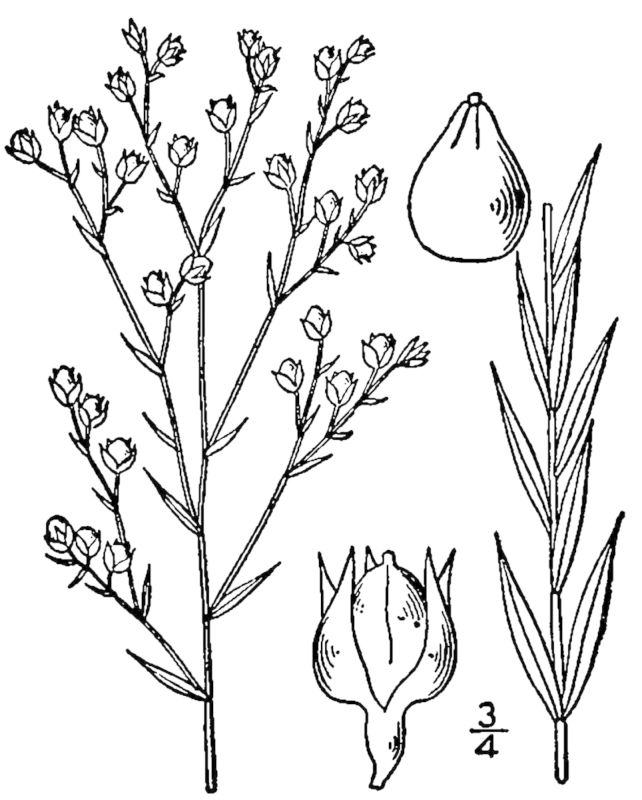
Linum floridanum var. floridanum (from Britton and Brown 1913).

**Figure 228b.**
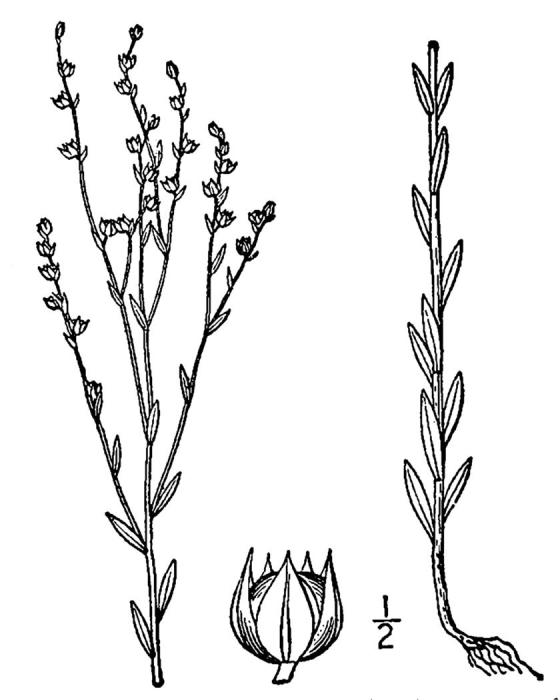
*Linum medium* (from Britton and Brown 1913).

**Figure 228c.**
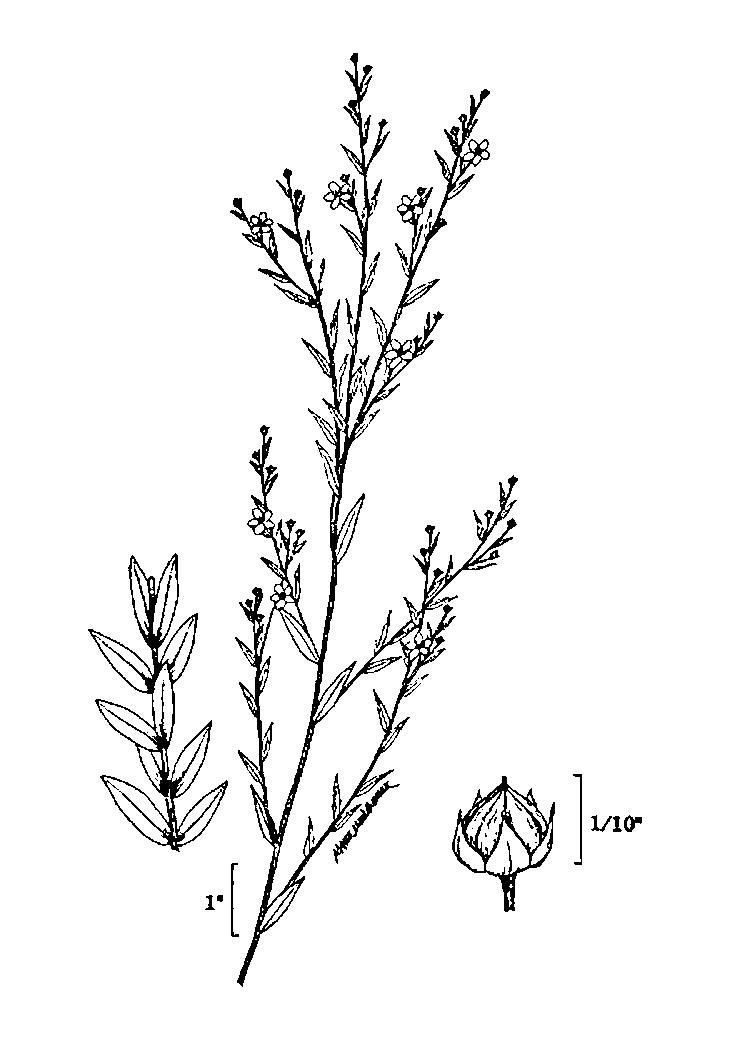
*Linum striatum* (from USDA-NRCS 2012).

**Figure 229a.**
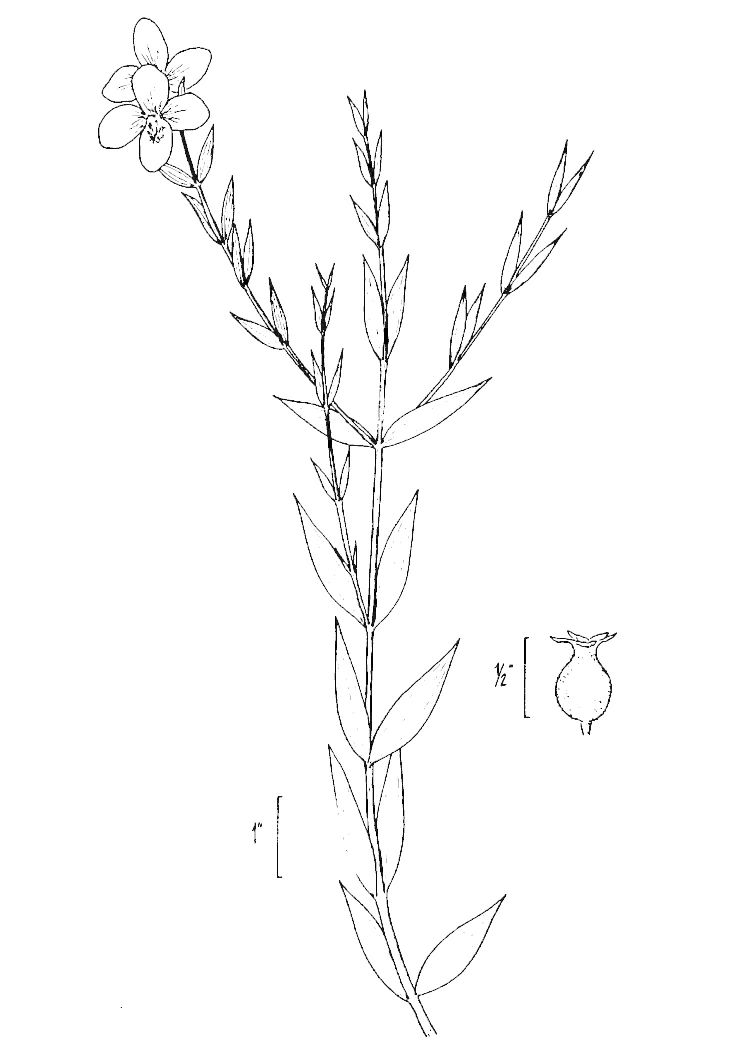
*Rhexia alifanus* (from USDA-NRCS 2012).

**Figure 229b.**
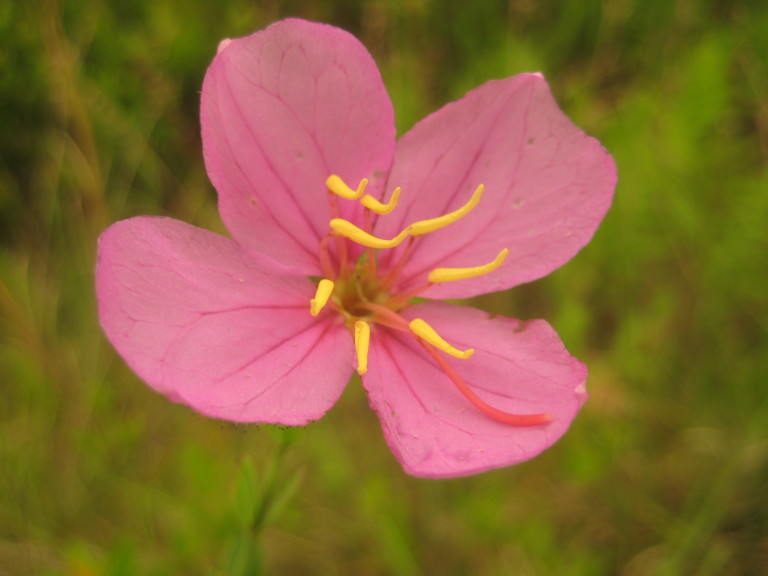
*Rhexia alifanus*: flower close-up (photo by R. Thornhill).

**Figure 229c.**
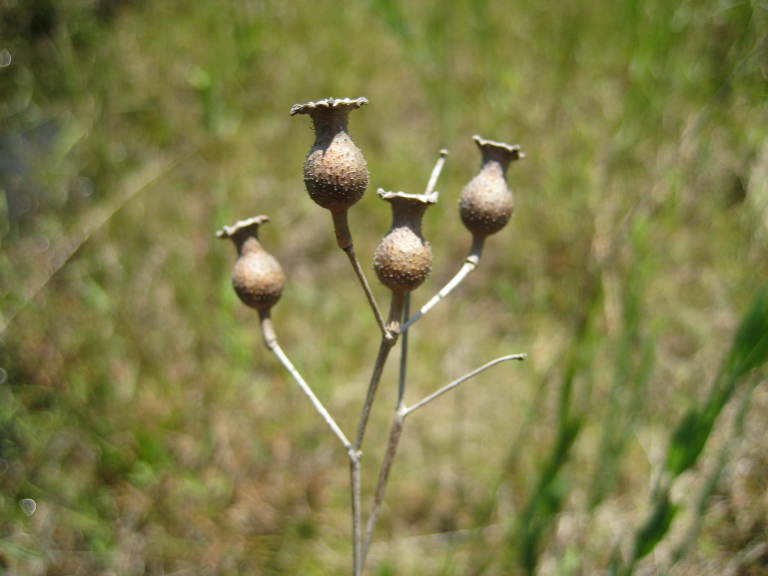
*Rhexia alifanus*: fruits (photo by R. Thornhill).

**Figure 229d.**
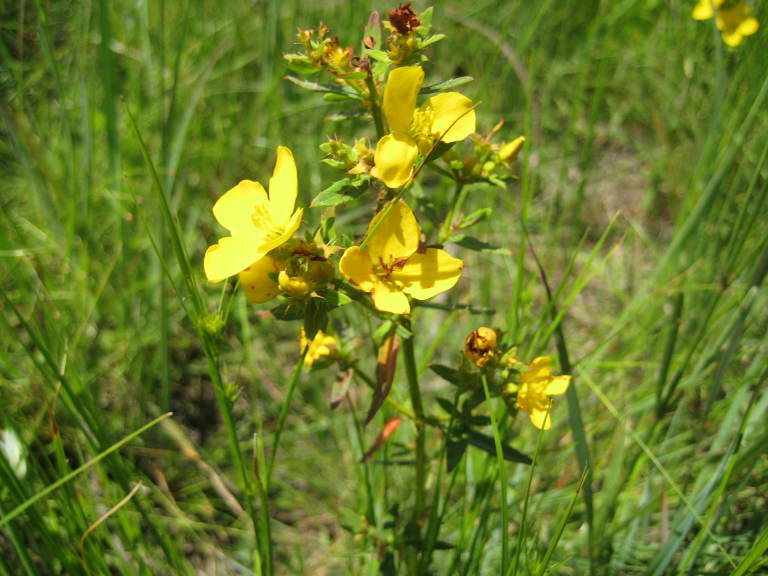
*Rhexia lutea* (photo by R. Thornhill).

**Figure 229e.**
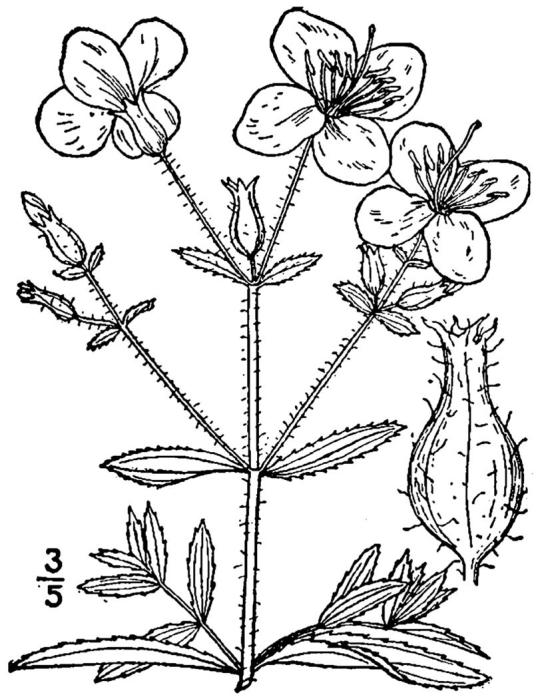
*Rhexia mariana* (from Britton and Brown 1913).

**Figure 229f.**
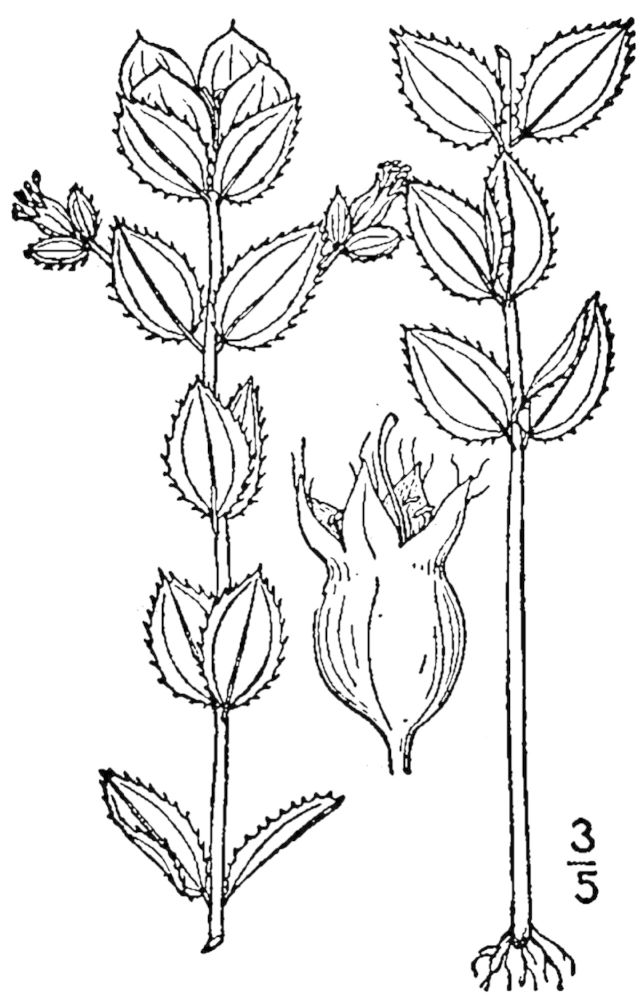
*Rhexia petiolata* (from Britton and Brown 1913).

**Figure 230a.**
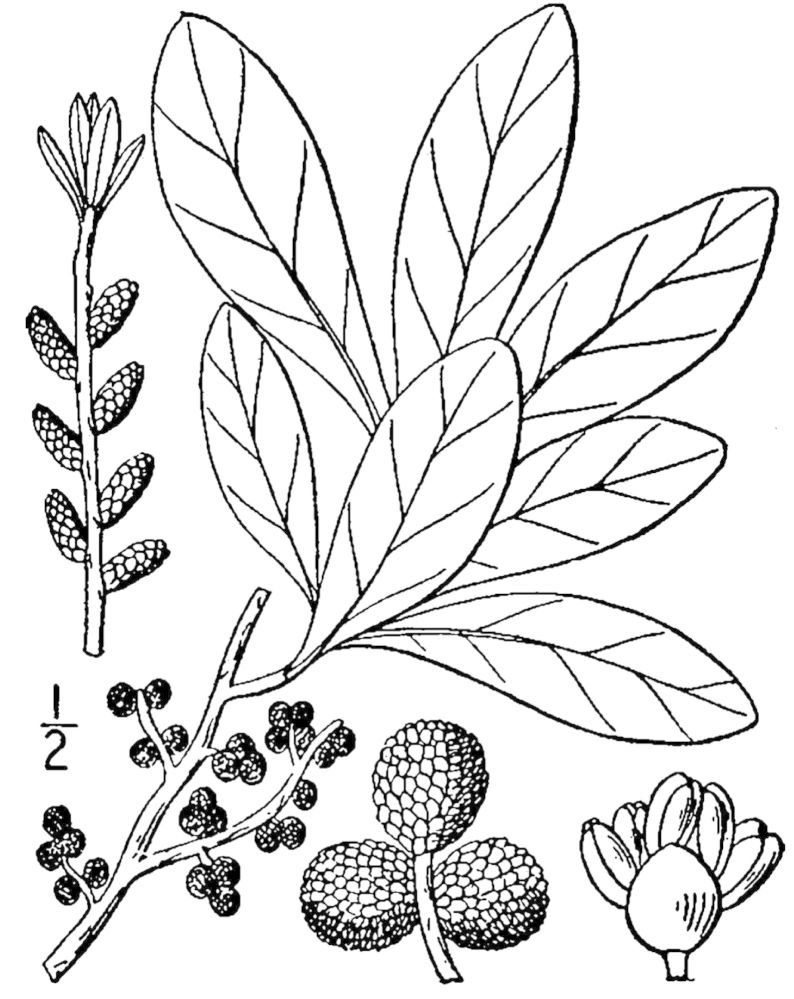
*Morella caroliniensis* (from Britton and Brown 1913).

**Figure 230b.**
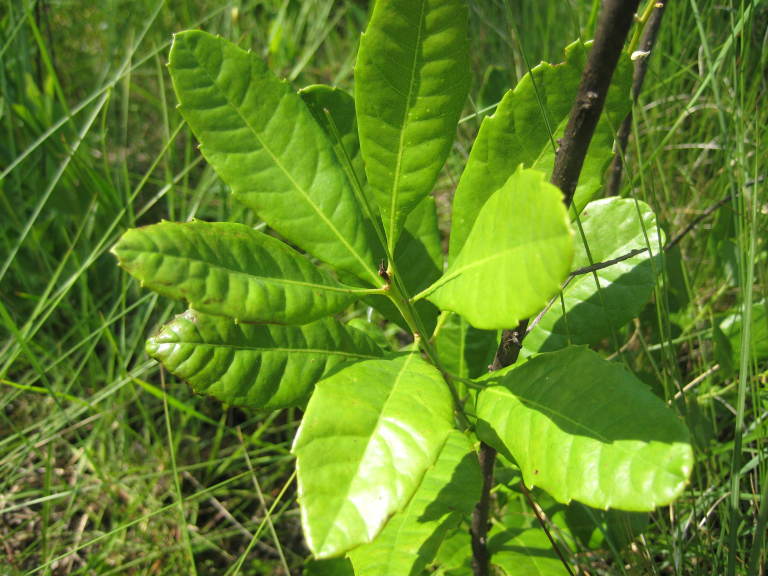
*Morella caroliniensis* (photo by R. Thornhill).

**Figure 230c.**
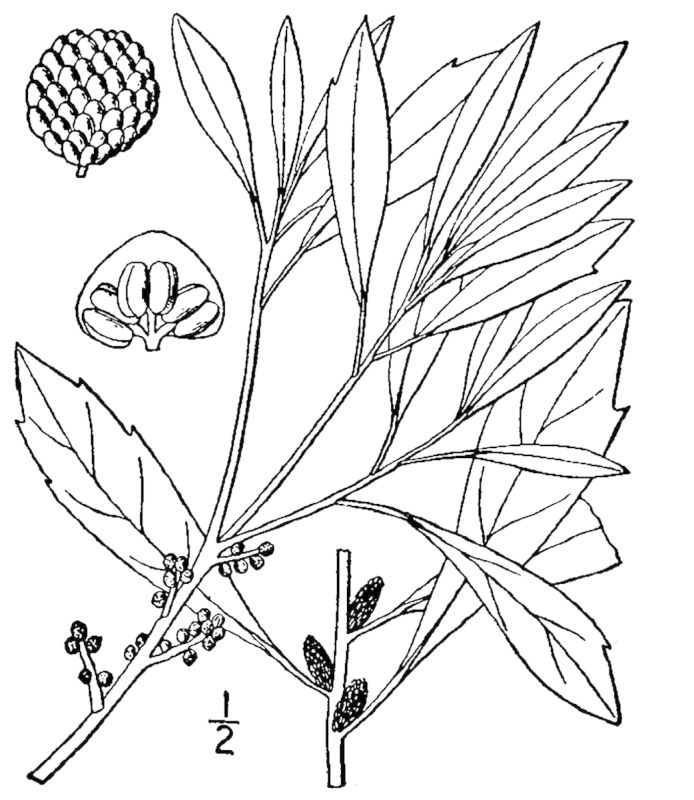
*Morella cerifera* (from Britton and Brown 1913).

**Figure 230d.**
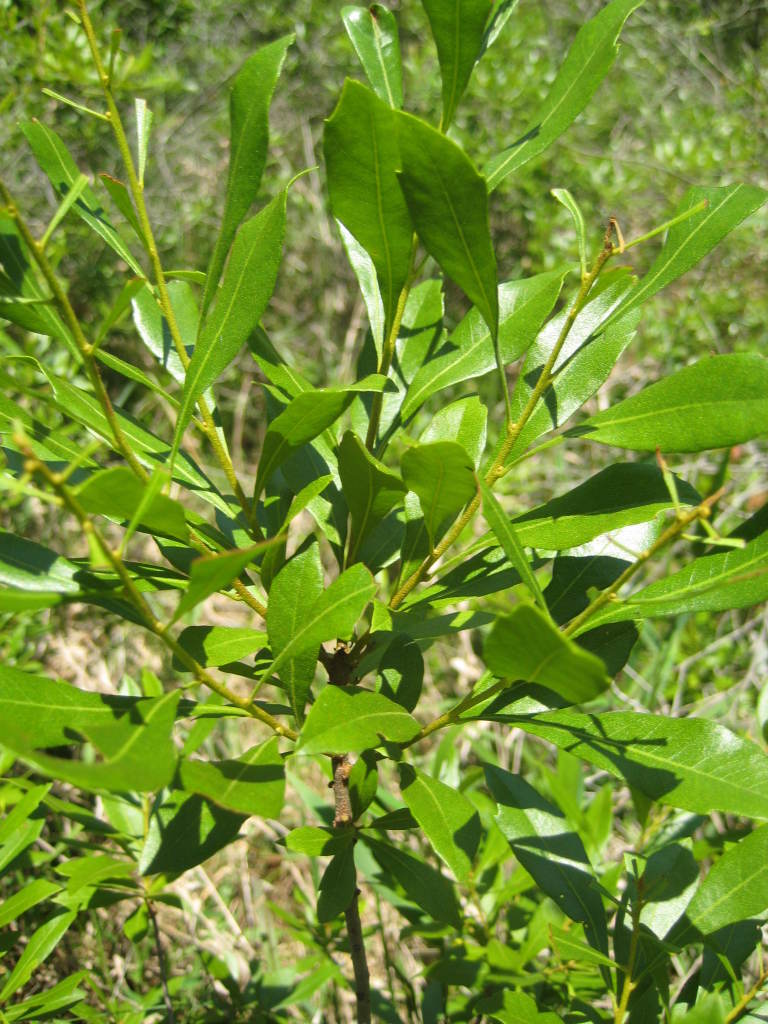
*Morella cerifera* (photo by R. Thornhill).

**Figure 230e.**
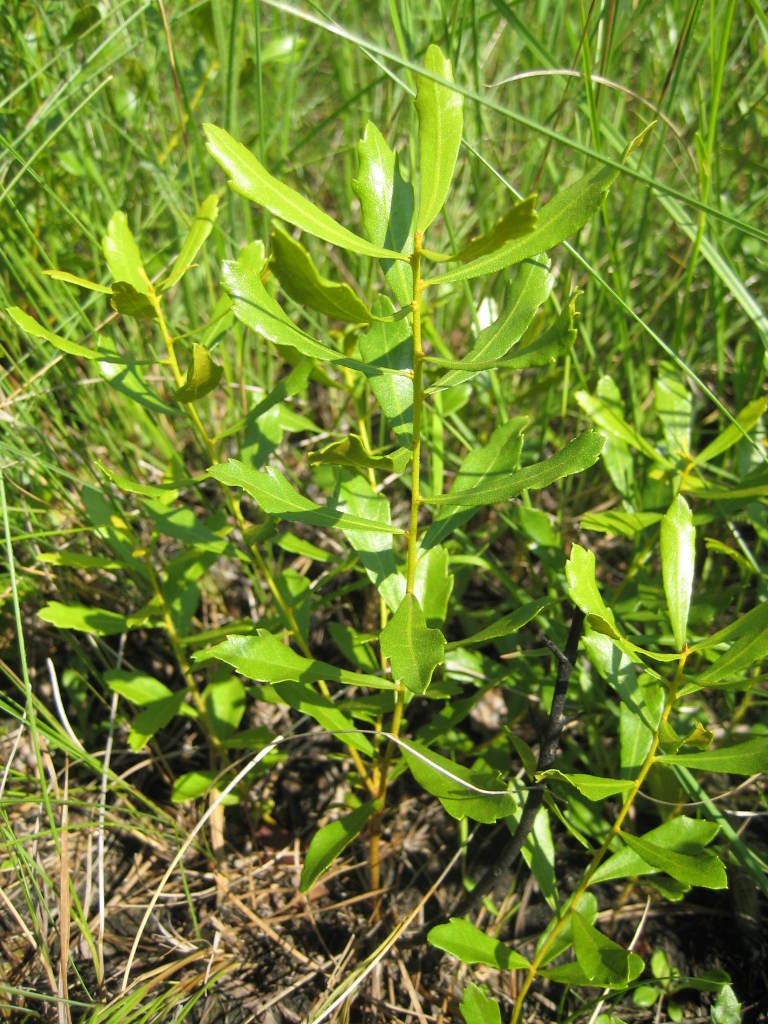
*Morella pumila* (photo by R. Thornhill).

**Figure 231a.**
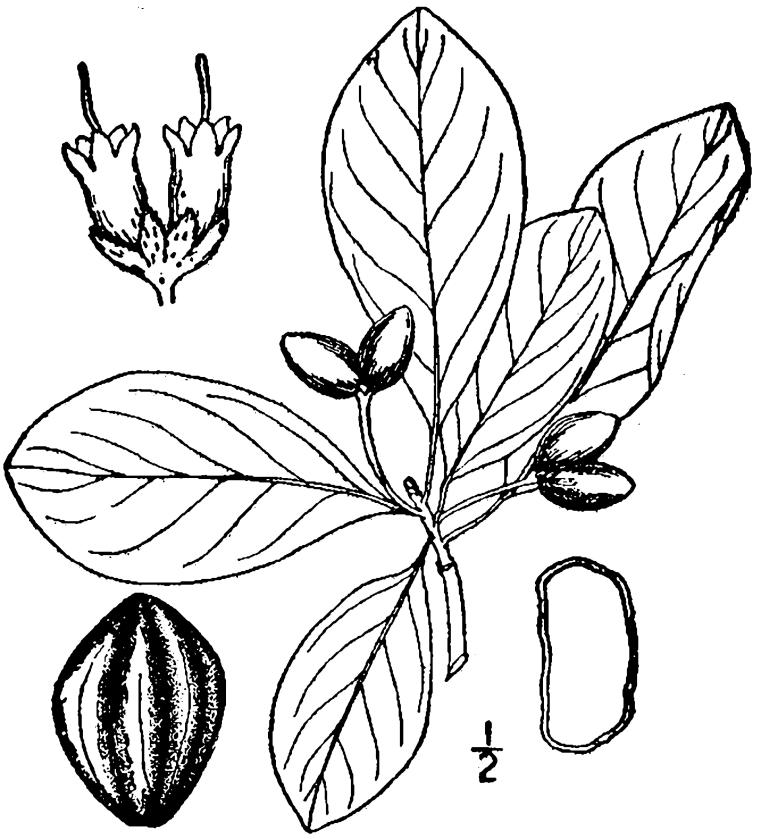
*Nyssa biflora* (from Britton and Brown 1913).

**Figure 231b.**
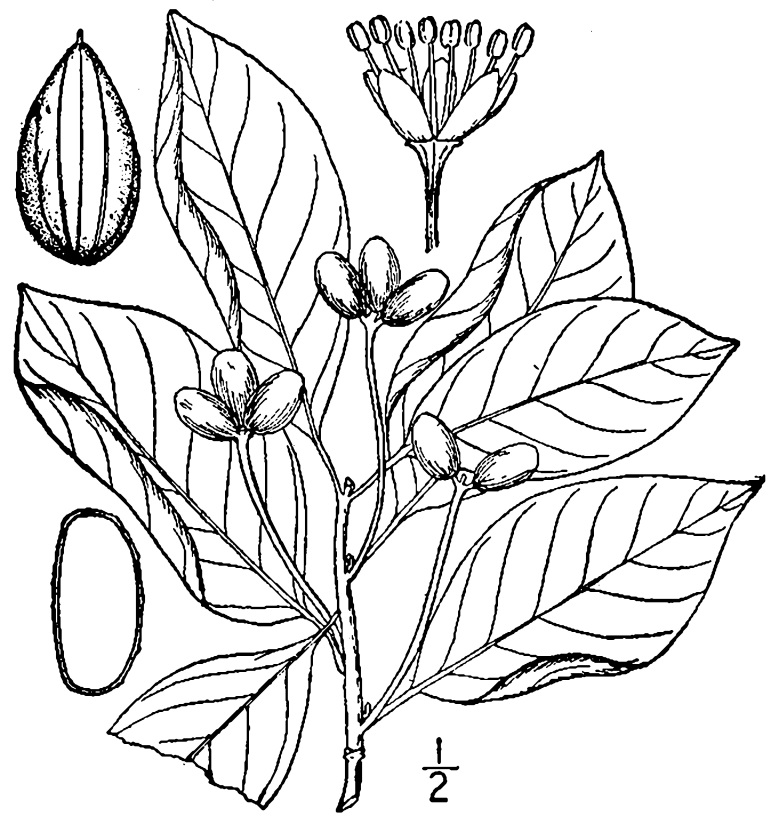
*Nyssa sylvatica* (from Britton and Brown 1913).

**Figure 232a.**
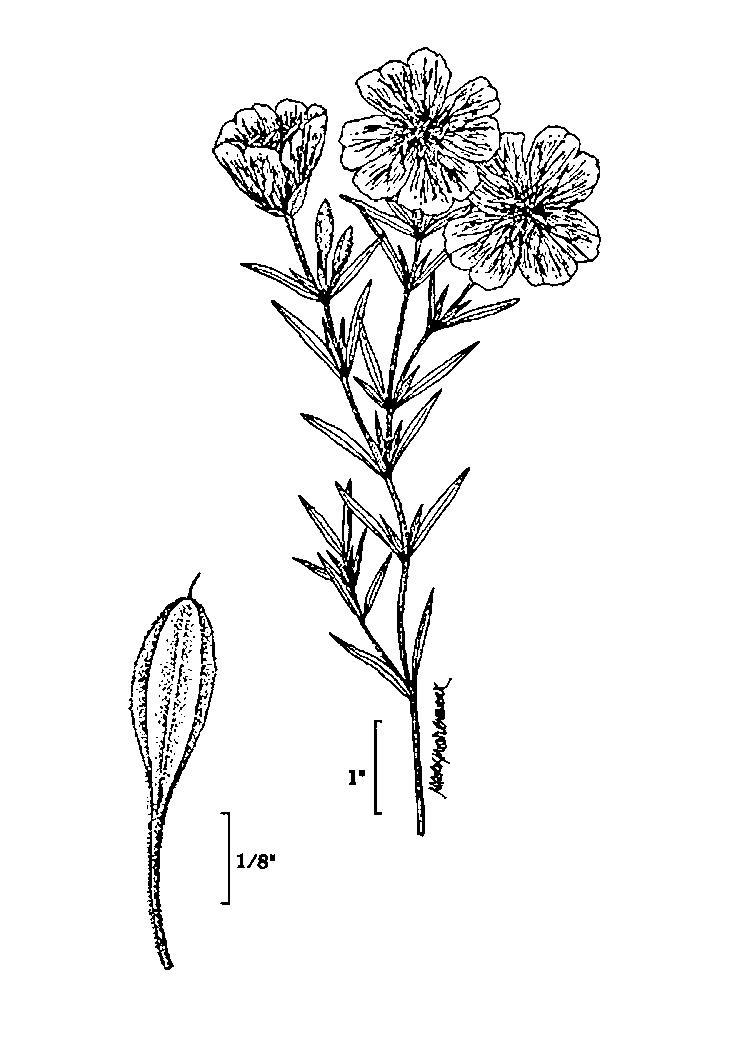
From USDA-NRCS (2012).

**Figure 232b.**
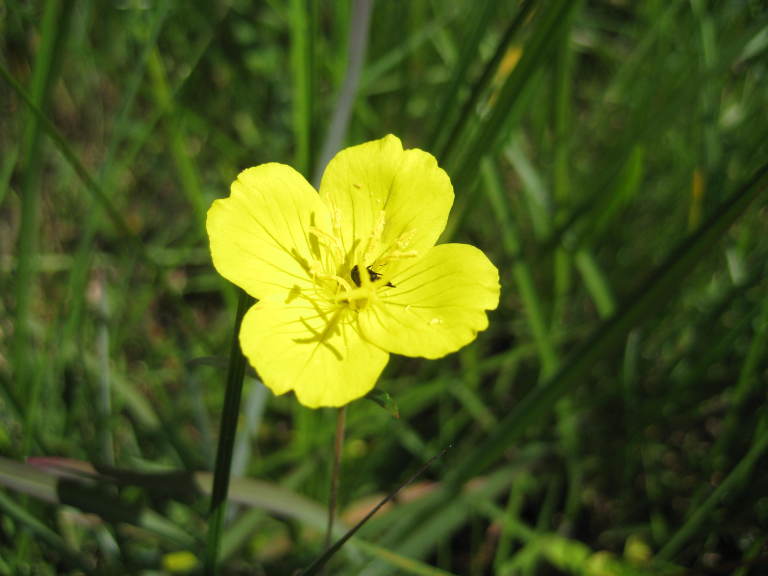
Photo by R. Thornhill.

**Figure 233a.**
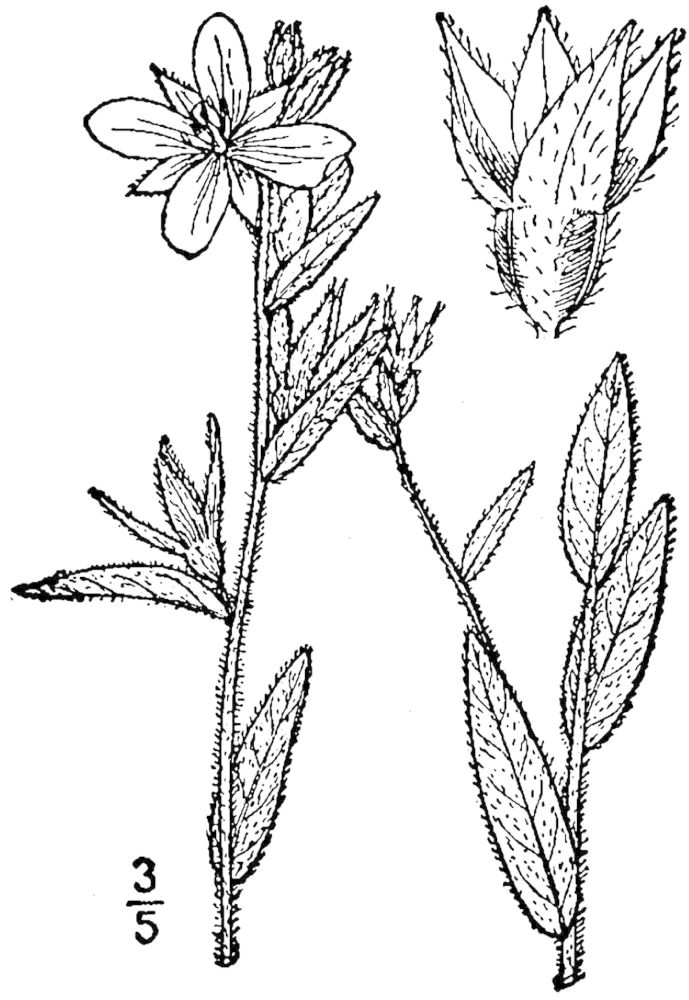
*Ludwigia hirtella* (from Britton and Brown 1913).

**Figure 233b.**
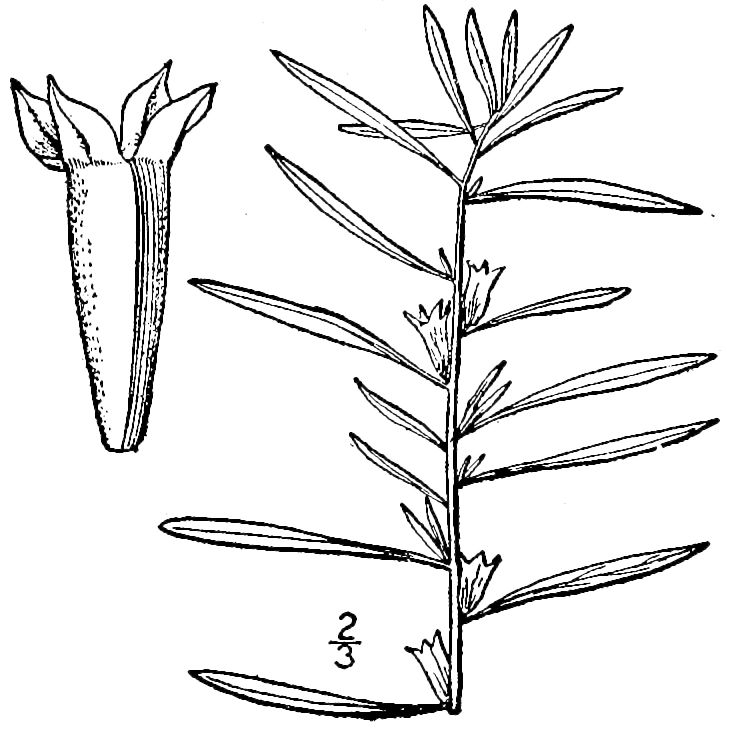
*Ludwigia linearis* (from Britton and Brown 1913).

**Figure 233c.**
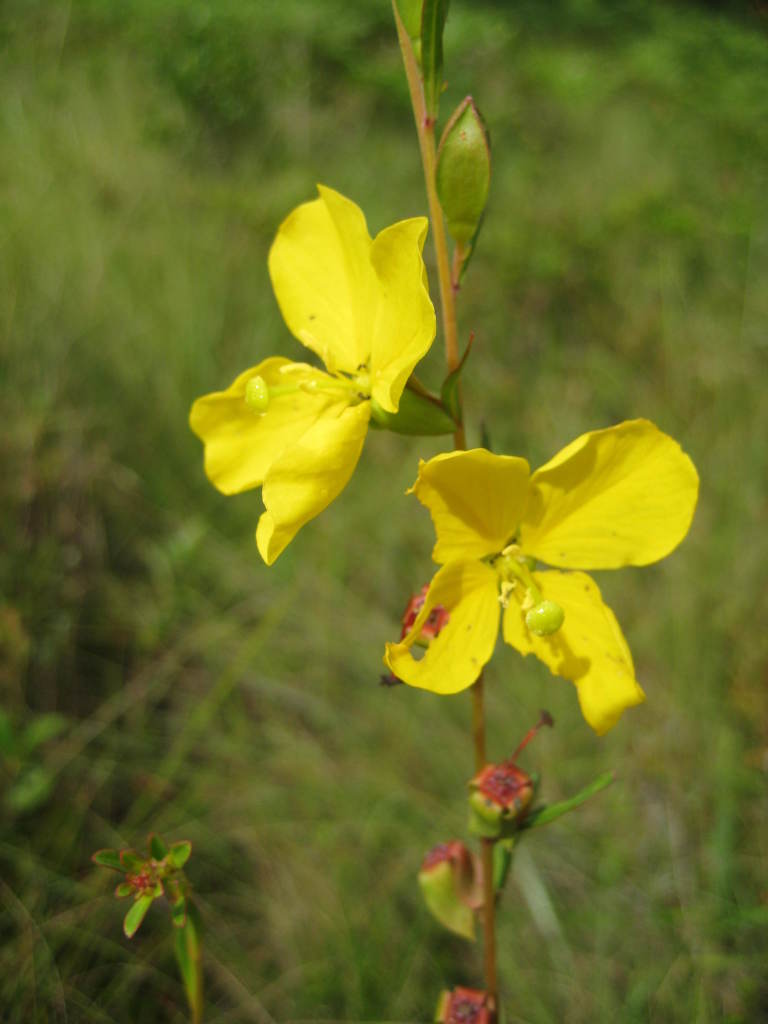
*Ludwigia virgata* (photo by R. Thornhill).

**Figure 234a.**
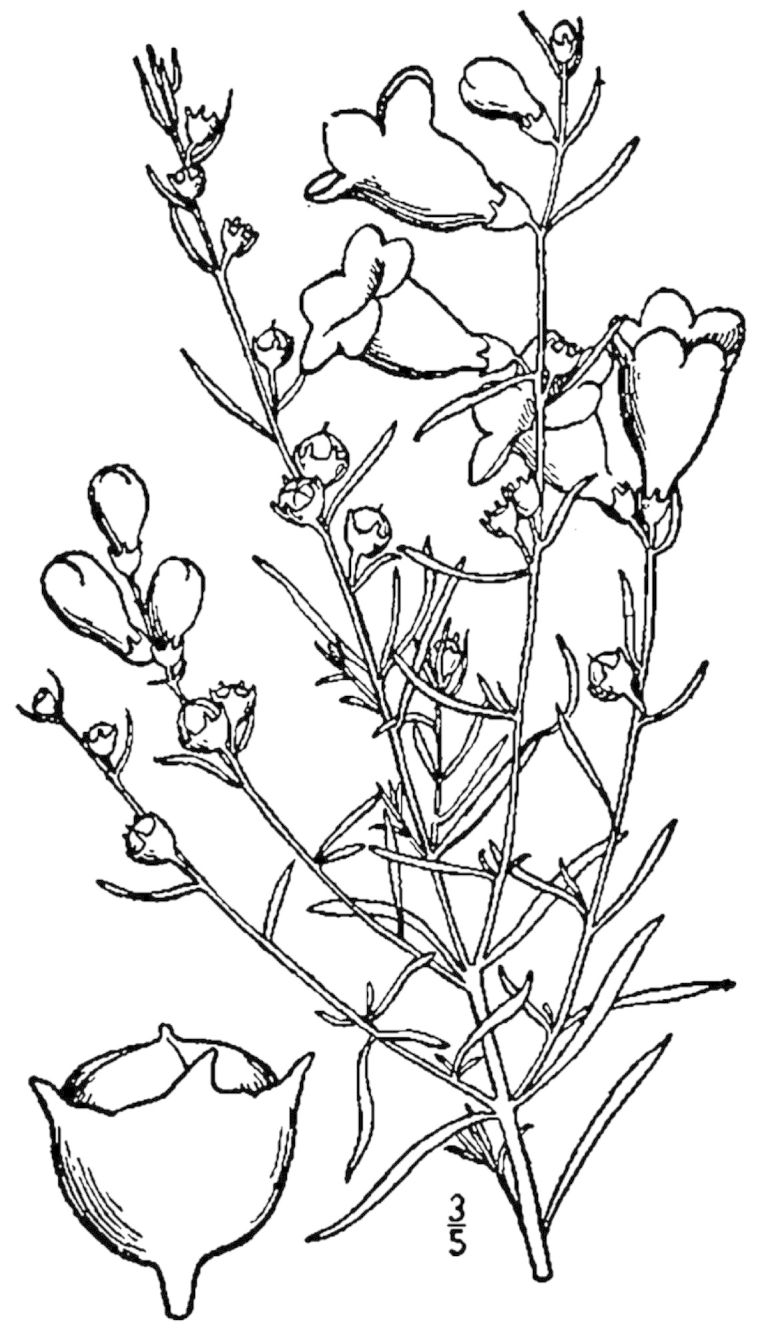
*Agalinis fasciculata* (from Britton and Brown 1913).

**Figure 234b.**
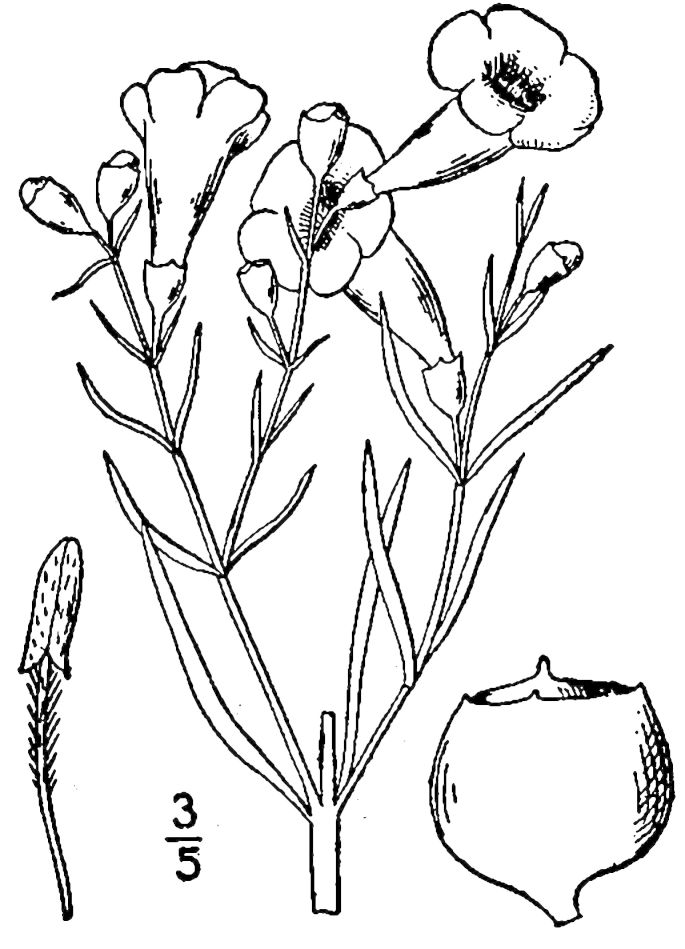
*Agalinis linifolia* (from Britton and Brown 1913).

**Figure 234c.**
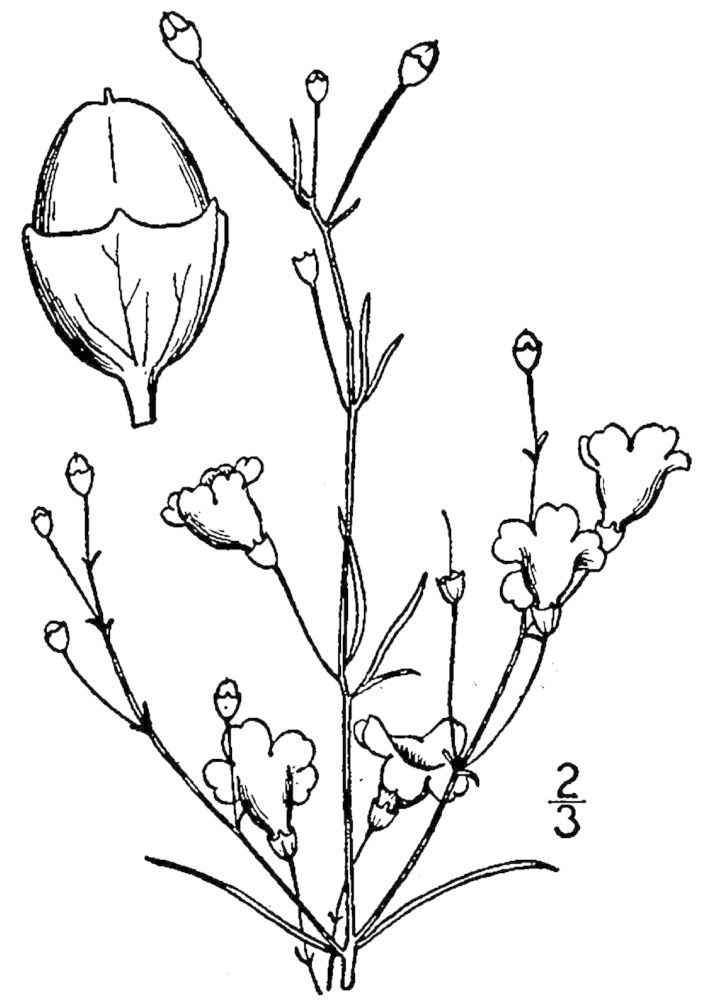
*Agalinis obtusifolia* (from Britton and Brown 1913).

**Figure 234d.**
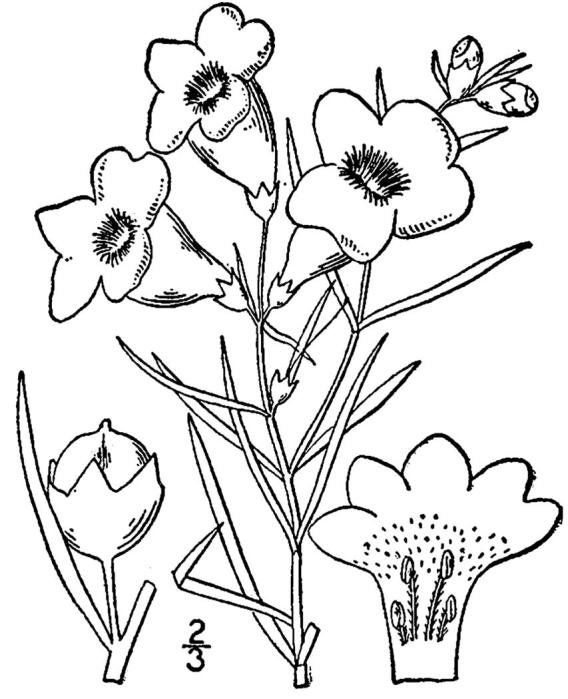
*Agalinis purpurea* (from Britton and Brown 1913).

**Figure 234e.**
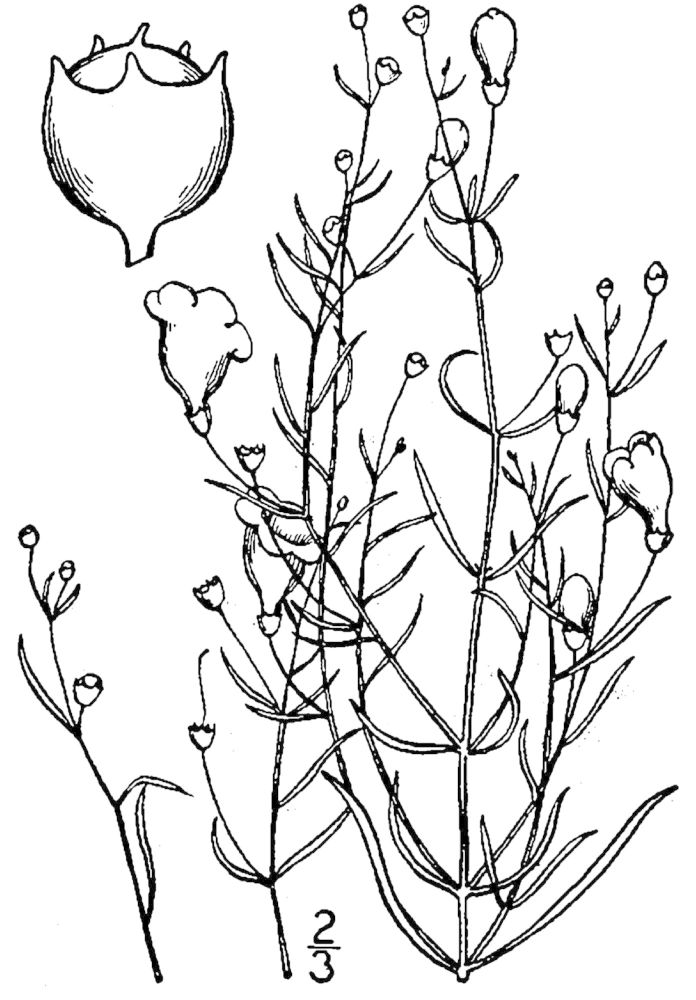
*Agalinis setacea* (from Britton and Brown 1913).

**Figure 235a.**
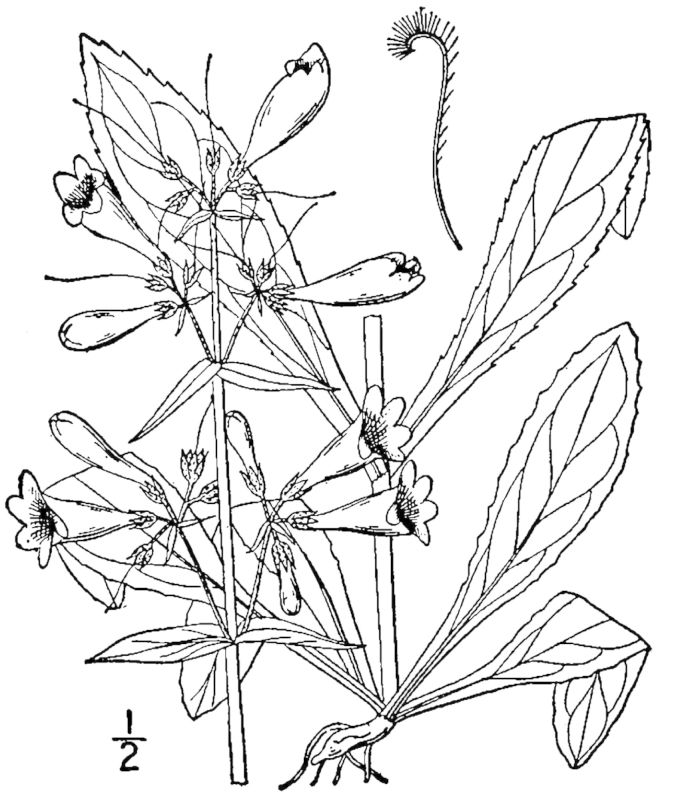
From Britton and Brown (1913).

**Figure 235b.**
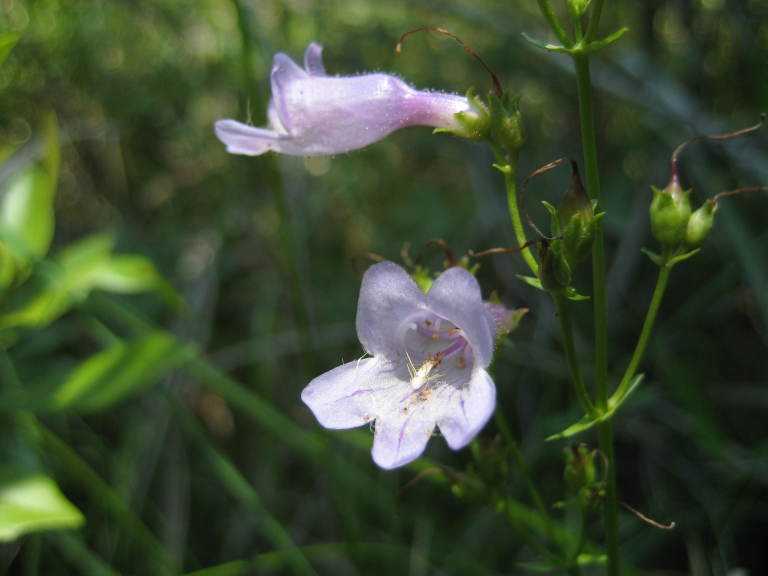
Photo by R. Thornhill.

**Figure 236a.**
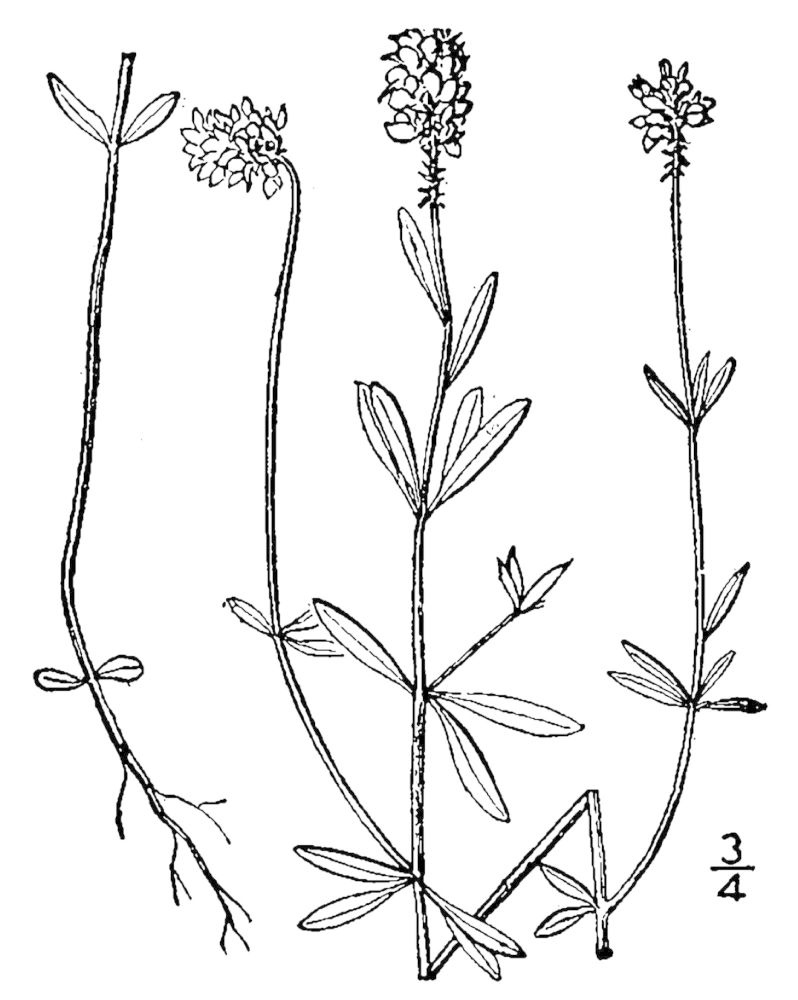
*Polygala brevifolia* (from Britton and Brown 1913).

**Figure 236b.**
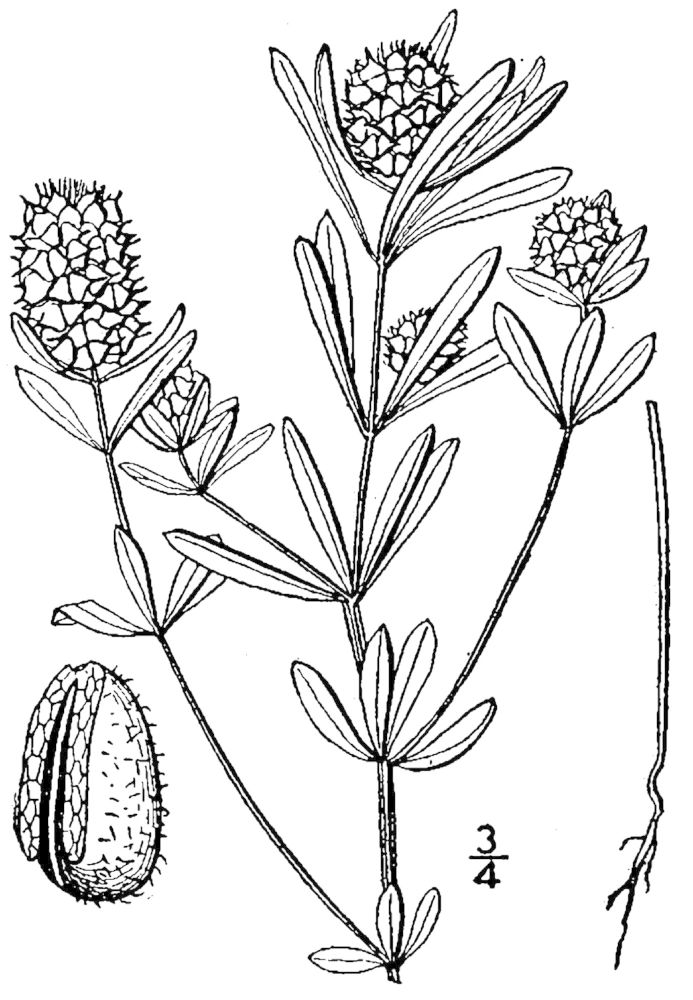
*Polygala cruciata* (from Britton and Brown 1913).

**Figure 236c.**
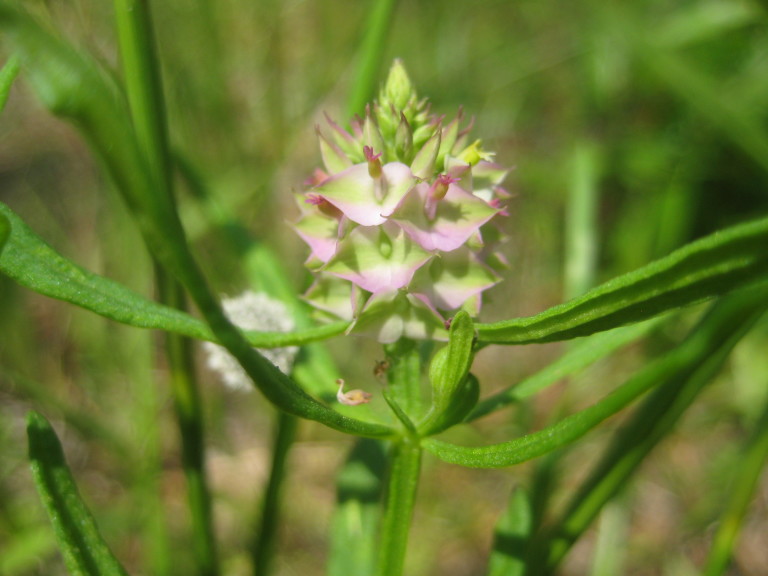
*Polygala cruciata* (photo by R. Thornhill).

**Figure 236d.**
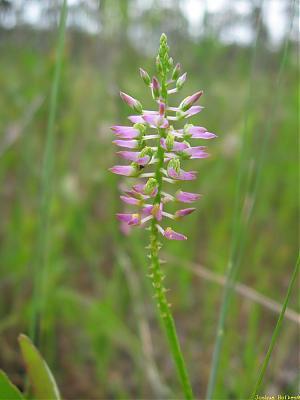
*Polygala hookeri* (photo by R. Thornhill).

**Figure 236e.**
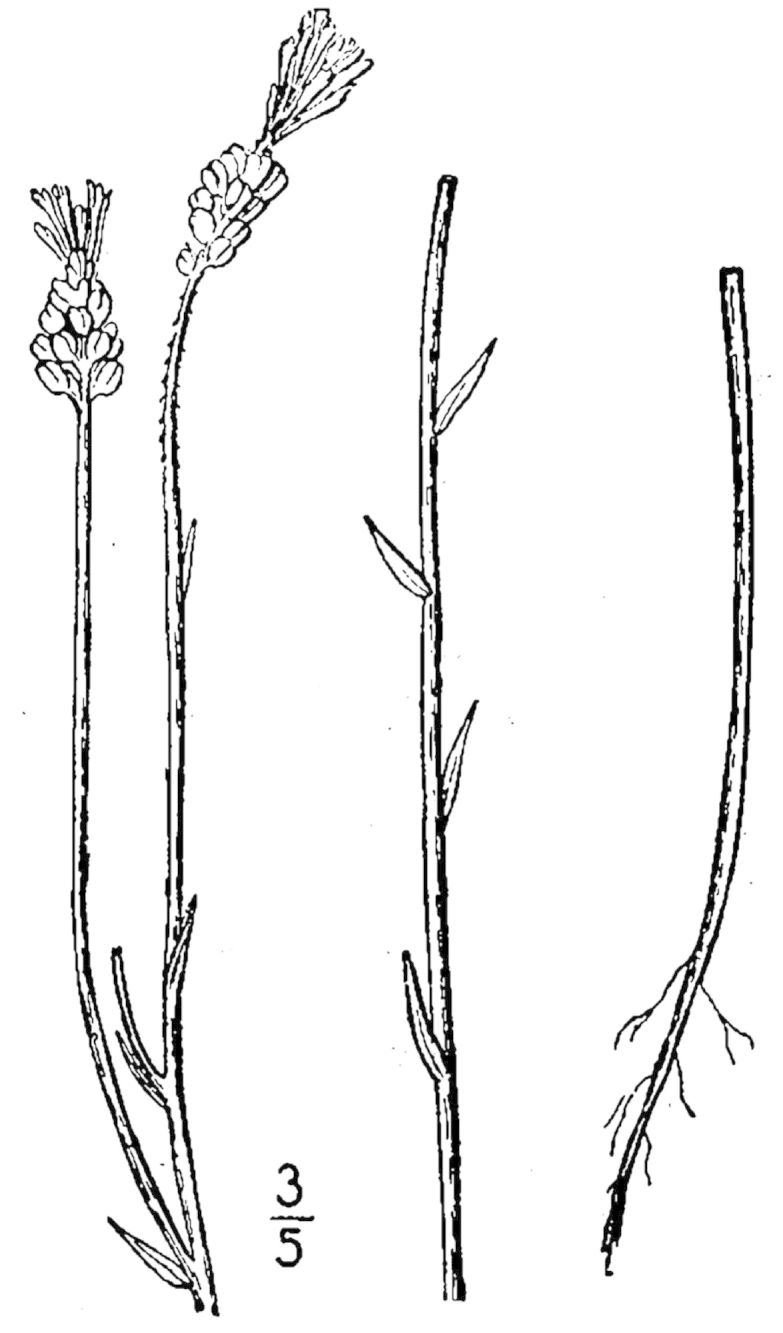
*Polygala incarnata* (from Britton and Brown 1913).

**Figure 236f.**
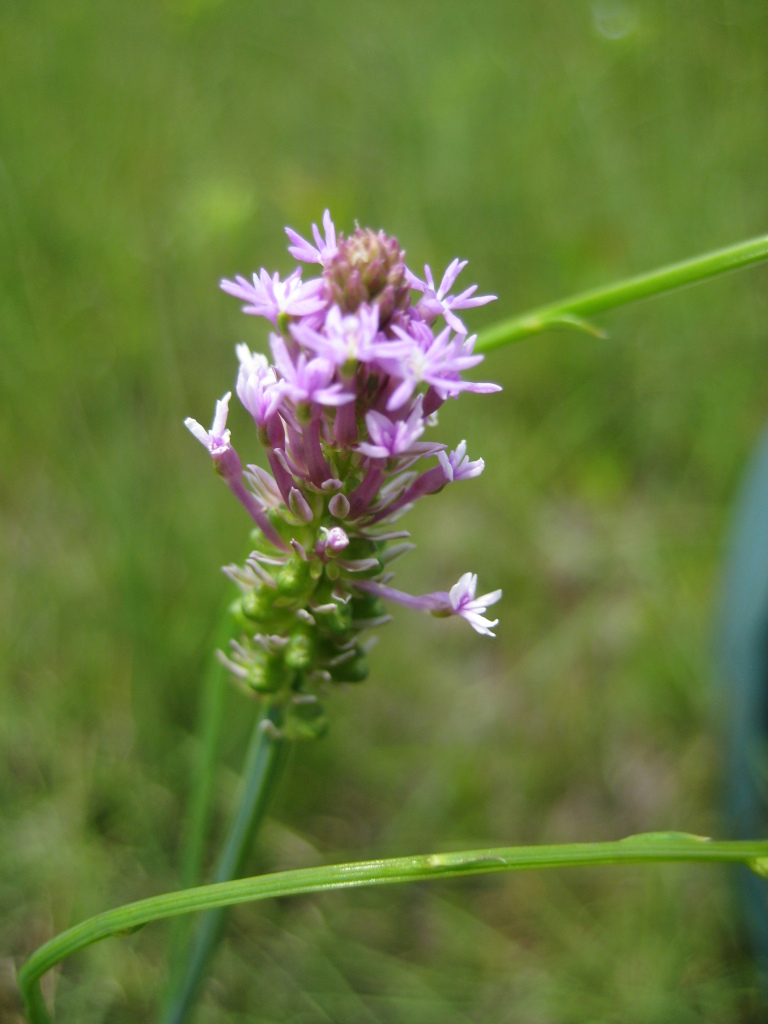
*Polygala incarnata* (photo by R. Thornhill).

**Figure 237a.**
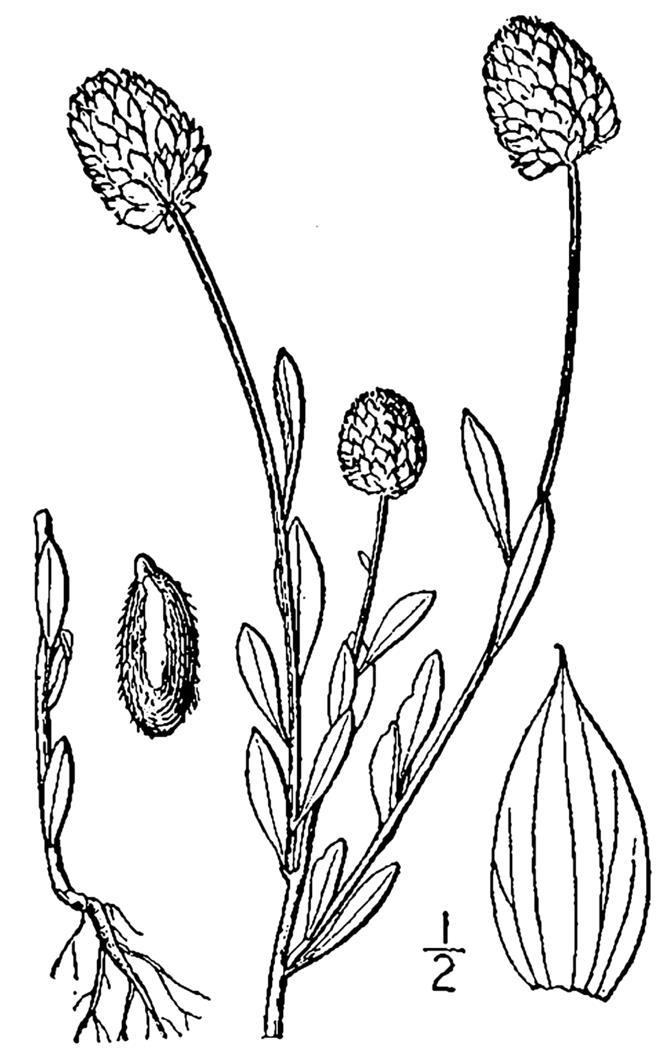
From Britton and Brown (1913).

**Figure 237b.**
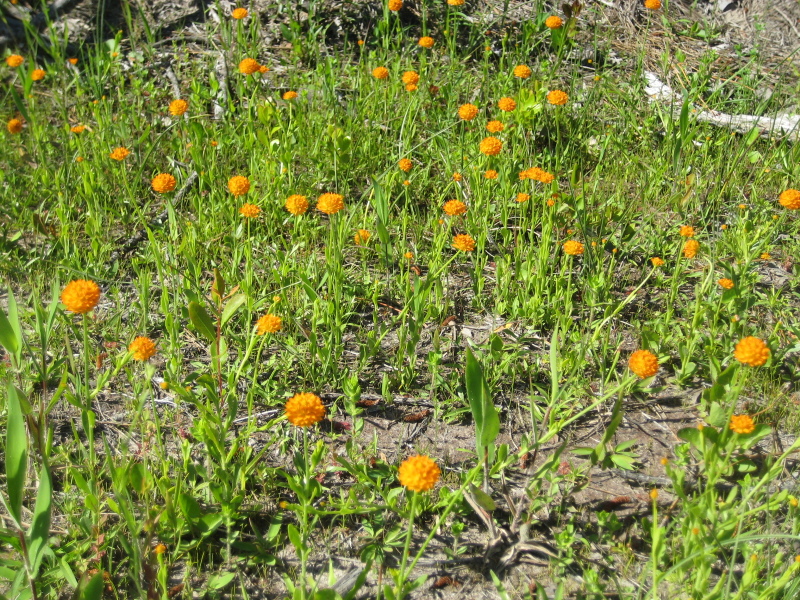
Photo by R. Thornhill.

**Figure 237c.**
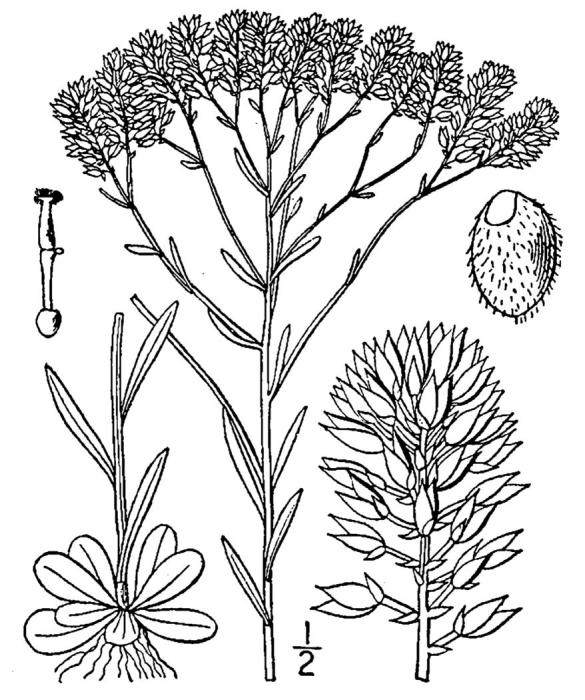
*Polygala ramosa* (from Britton and Brown 1913).

**Figure 237d.**
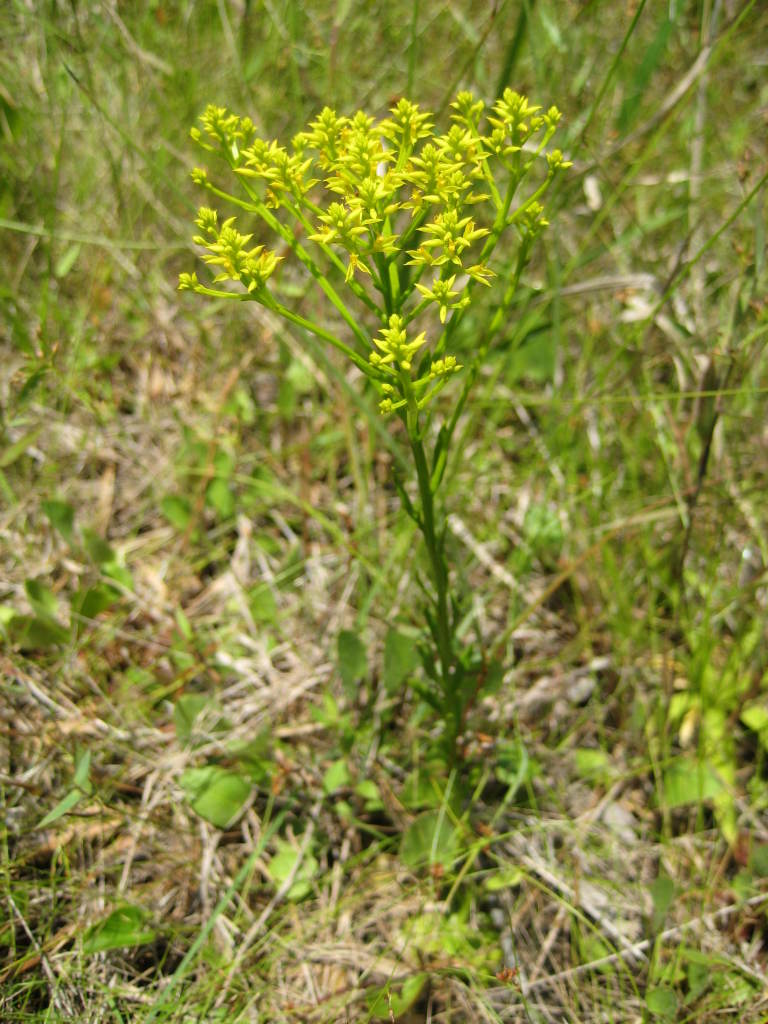
*Polygala ramosa* (photo by R. Thornhill).

**Figure 237e.**
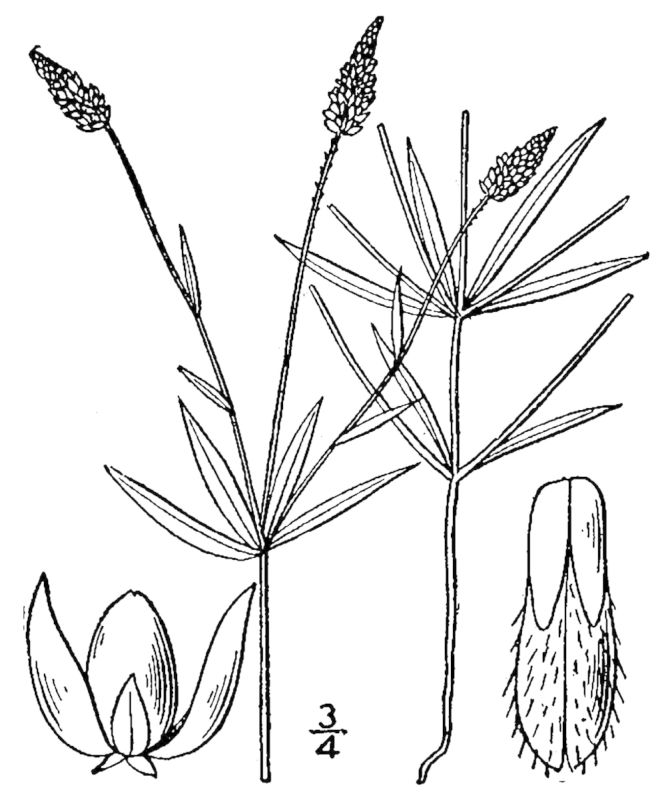
*Polygala verticillata* (from Britton and Brown 1913).

**Figure 238.**
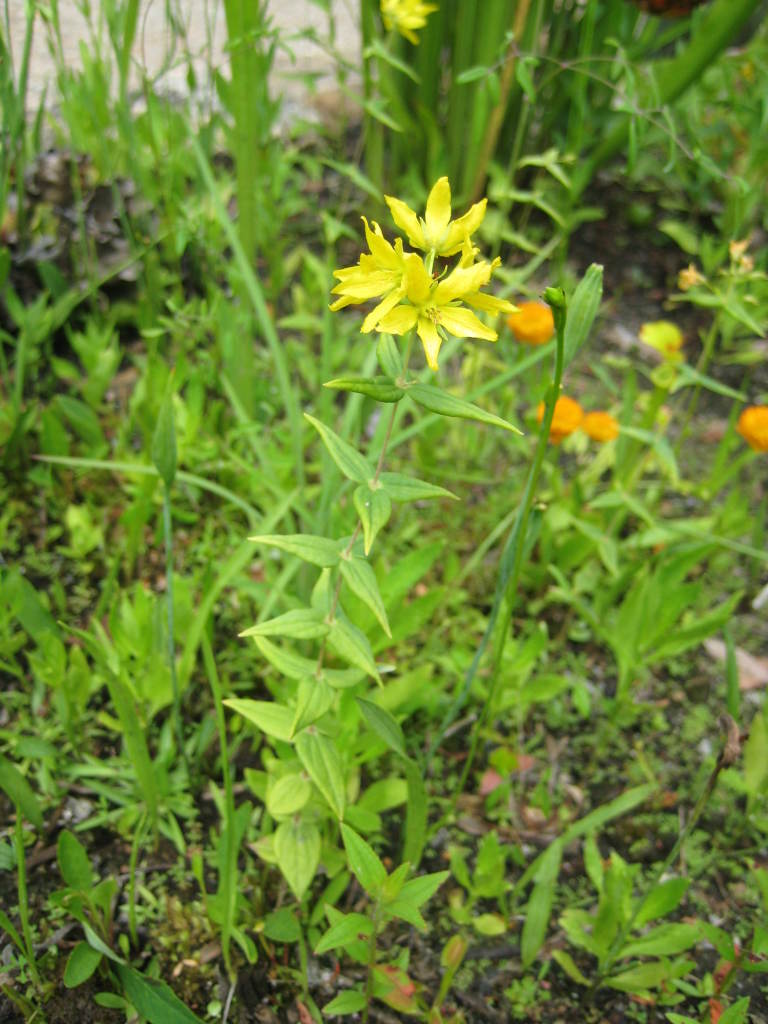
*Lysimachia asperulifolia* (photo by R. Thornhill).

**Figure 239.**
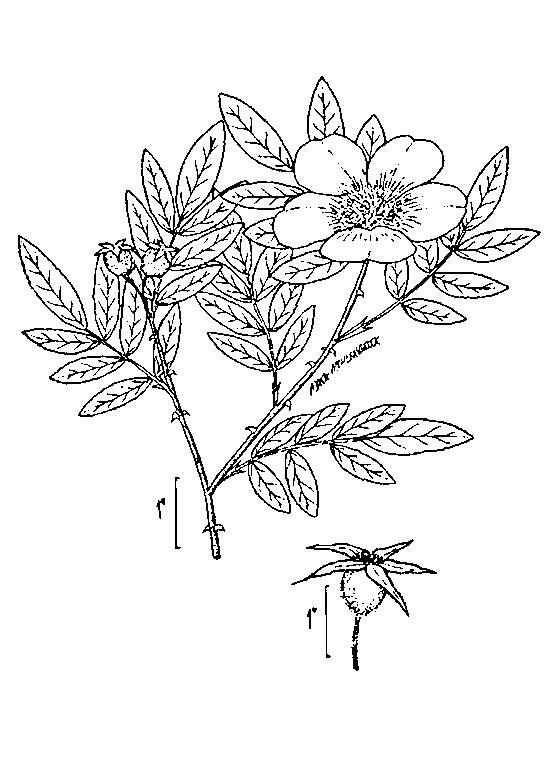
*Rosa palustris* (from USDA-NRCS 2012).

**Figure 240a.**
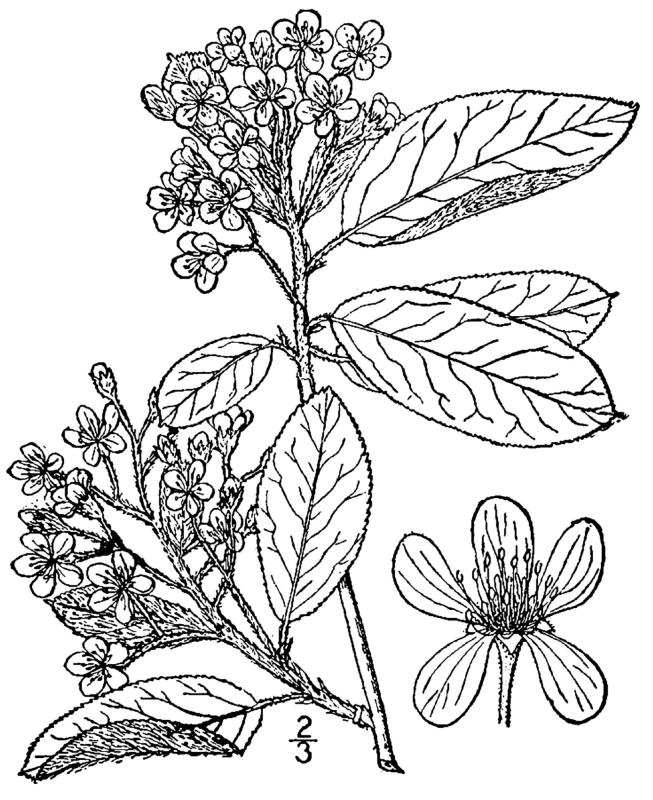
From USDA-NRCS (2012).

**Figure 240b.**
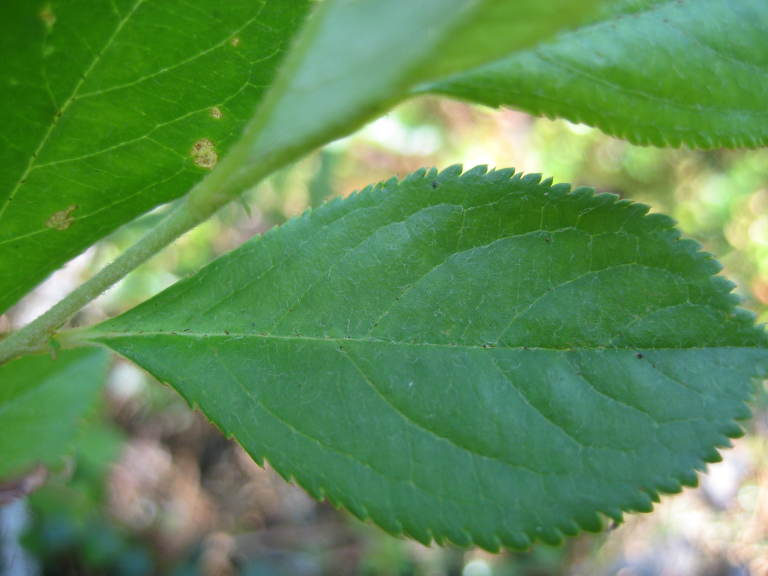
Note the distinctive, small, dark trichomes on the midvein of the adaxial leaf surface (photo by R. Thornhill).

**Figure 241a.**
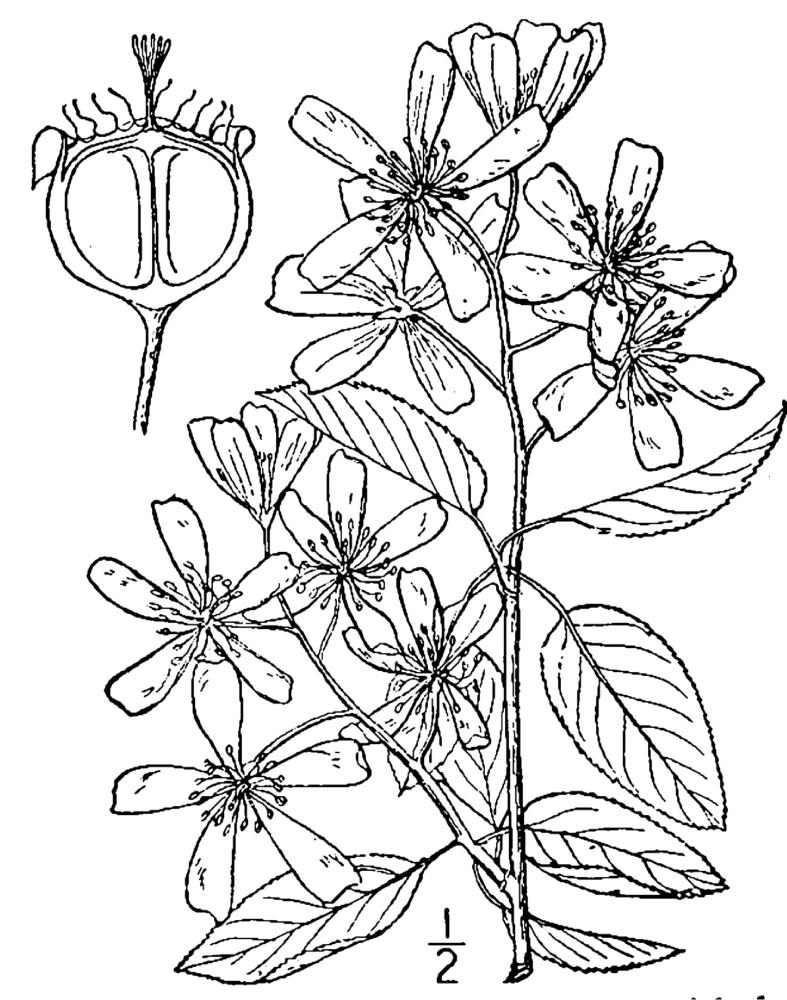
*Amelanchier canadensis* (from Britton and Brown 1913).

**Figure 241b.**
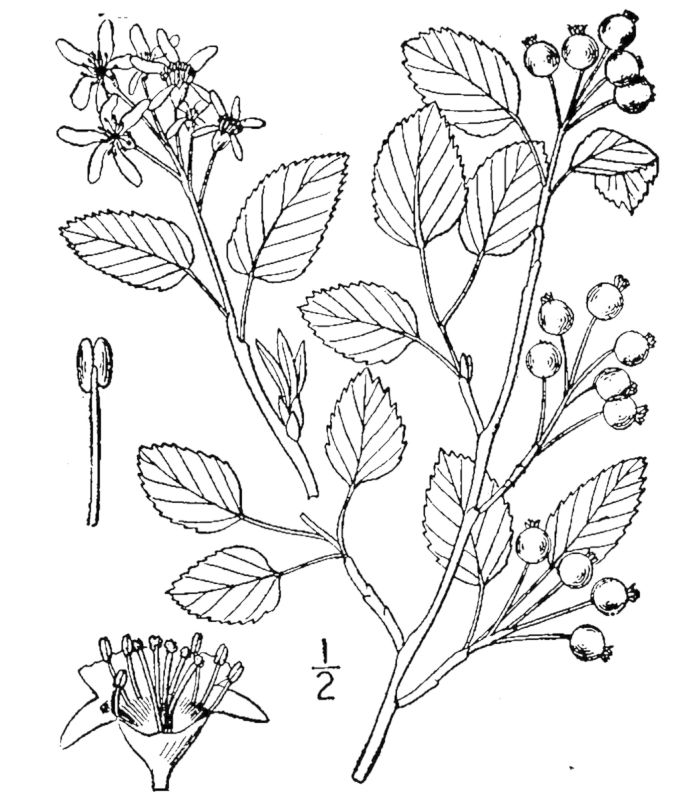
*Amelanchier spicata* (from Britton and Brown 1913).

**Figure 242a.**
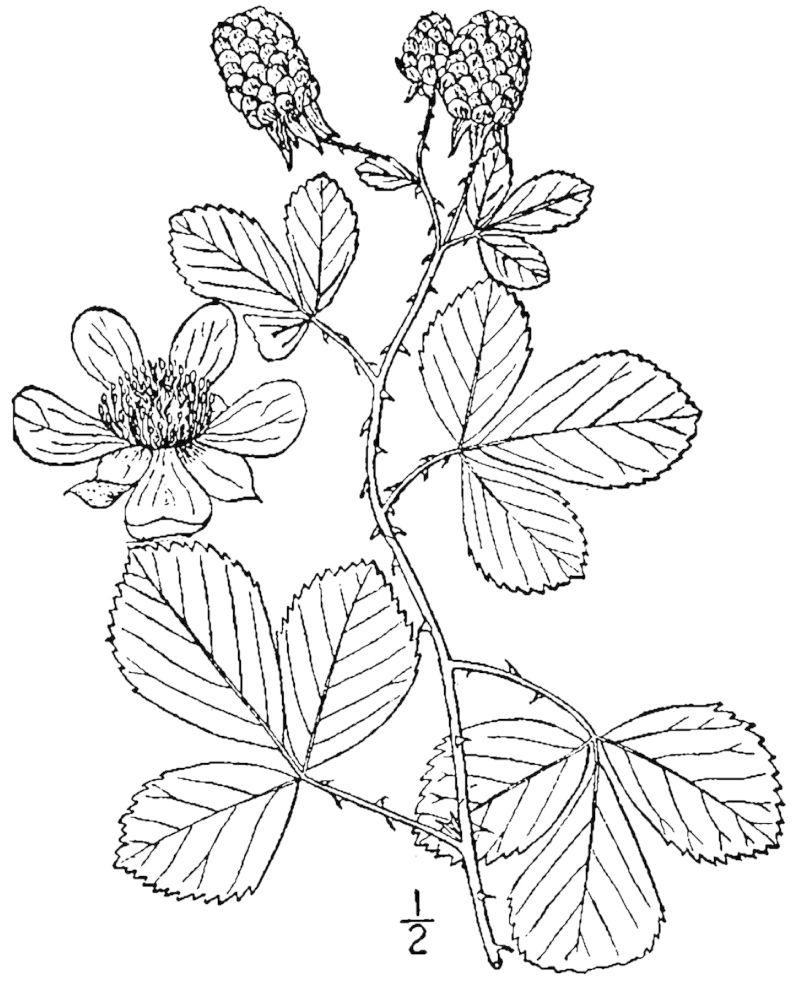
*Rubus cuneifolius* (from Britton and Brown 1913).

**Figure 242b.**
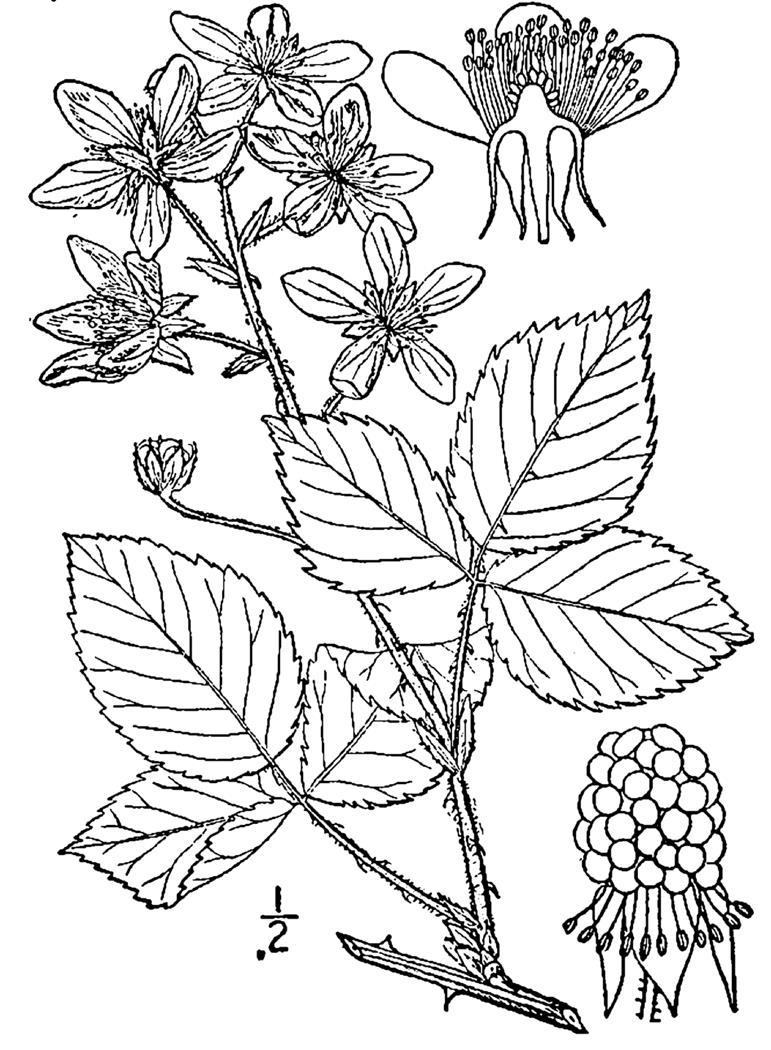
*Rubus pensilvanicus* (from Britton and Brown 1913).

**Figure 243.**
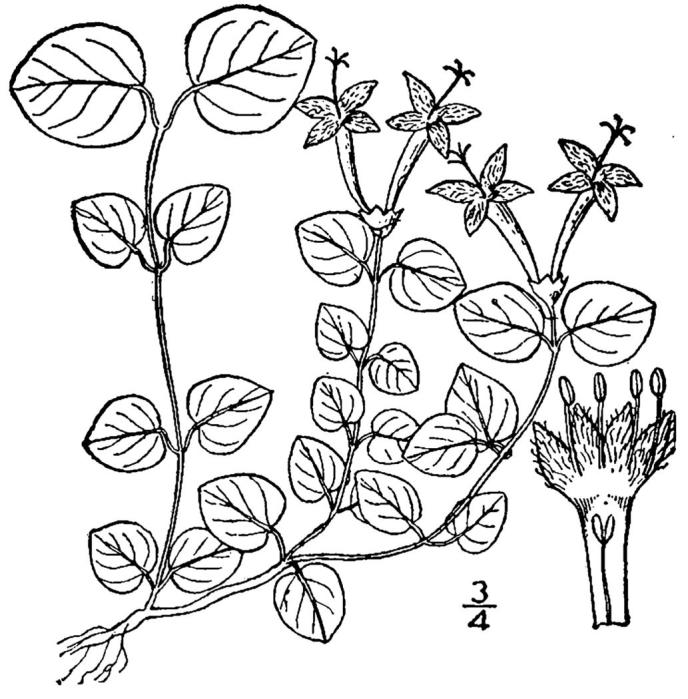
*Mitchella repens* (from Britton and Brown 1913).

**Figure 244.**
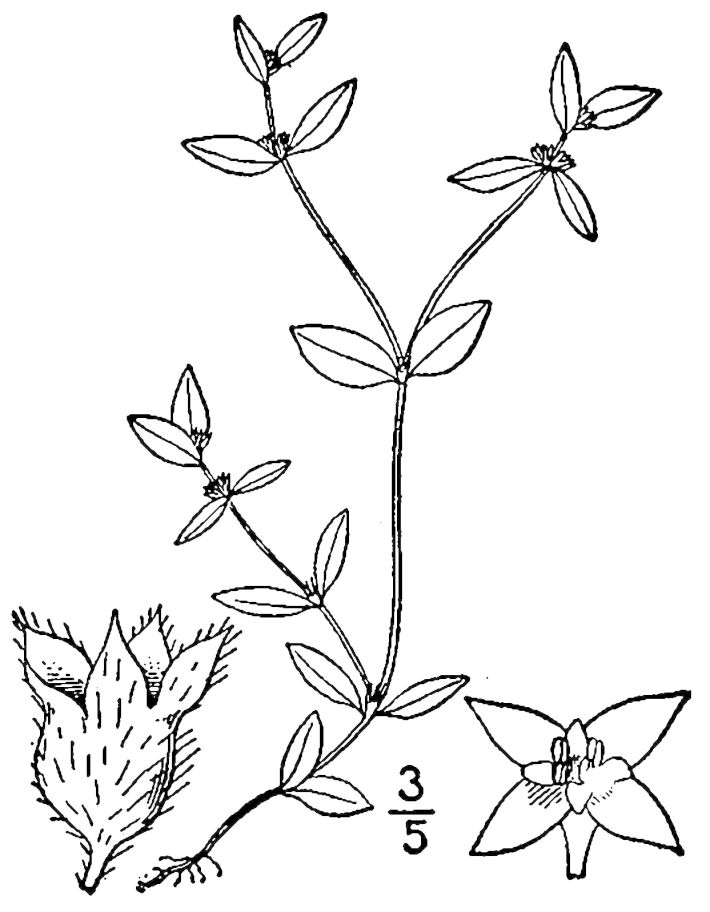
*Oldenlandia uniflora* (from Britton and Brown 1913).

**Figure 245a.**
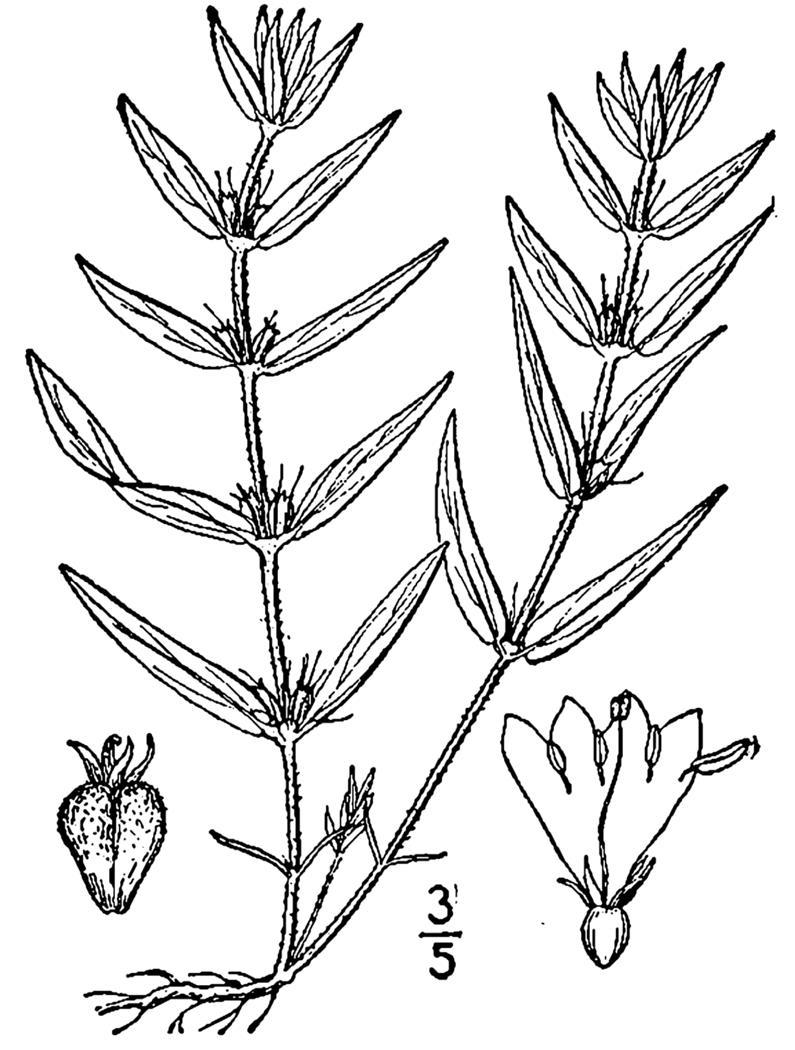
*Diodia teres* (from Britton and Brown 1913).

**Figure 245b.**
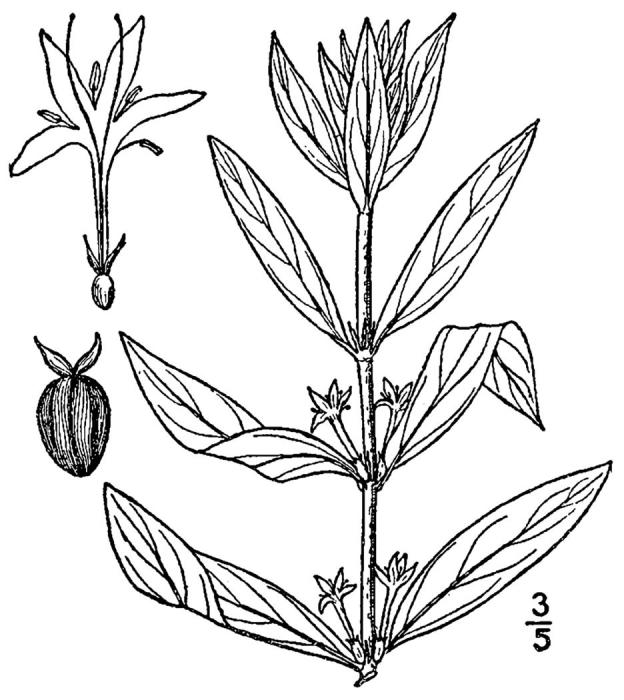
*Diospyros virginiana* (from Britton and Brown 1913).

**Figure 246a.**
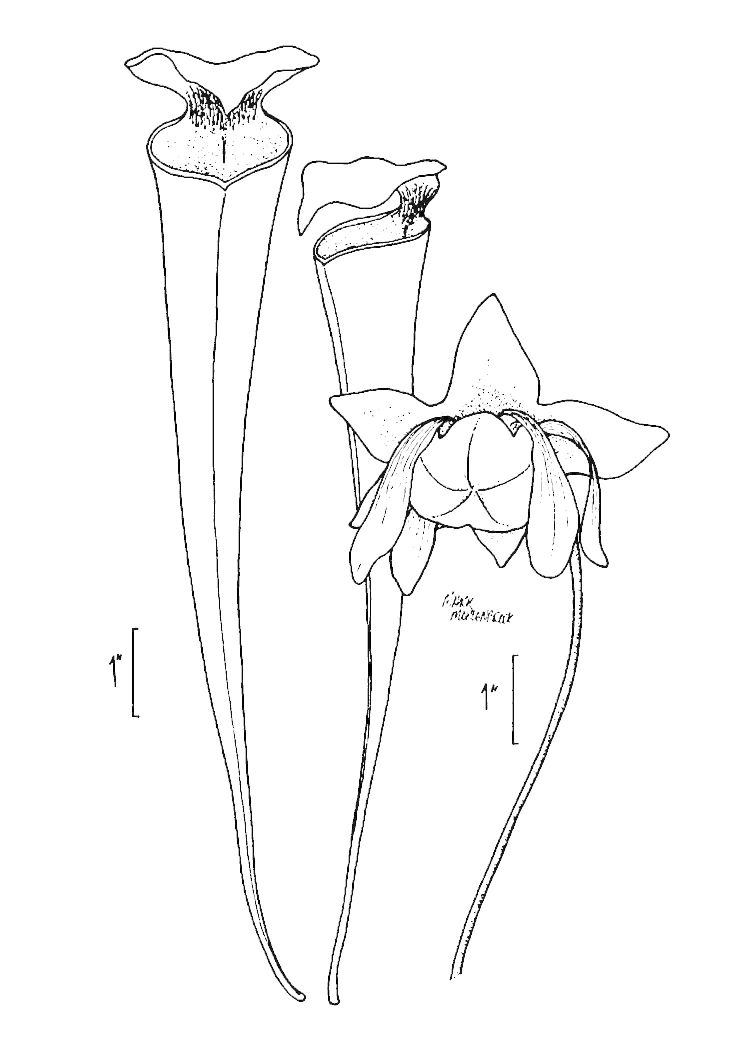
*Sarracenia flava* (from USDA-NRCS 2012).

**Figure 246b.**
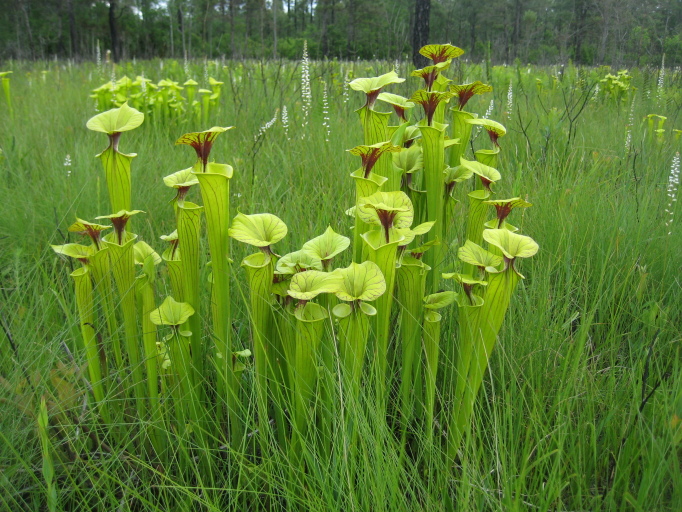
*Sarracenia flava* (photo by R. Thornhill).

**Figure 246c.**
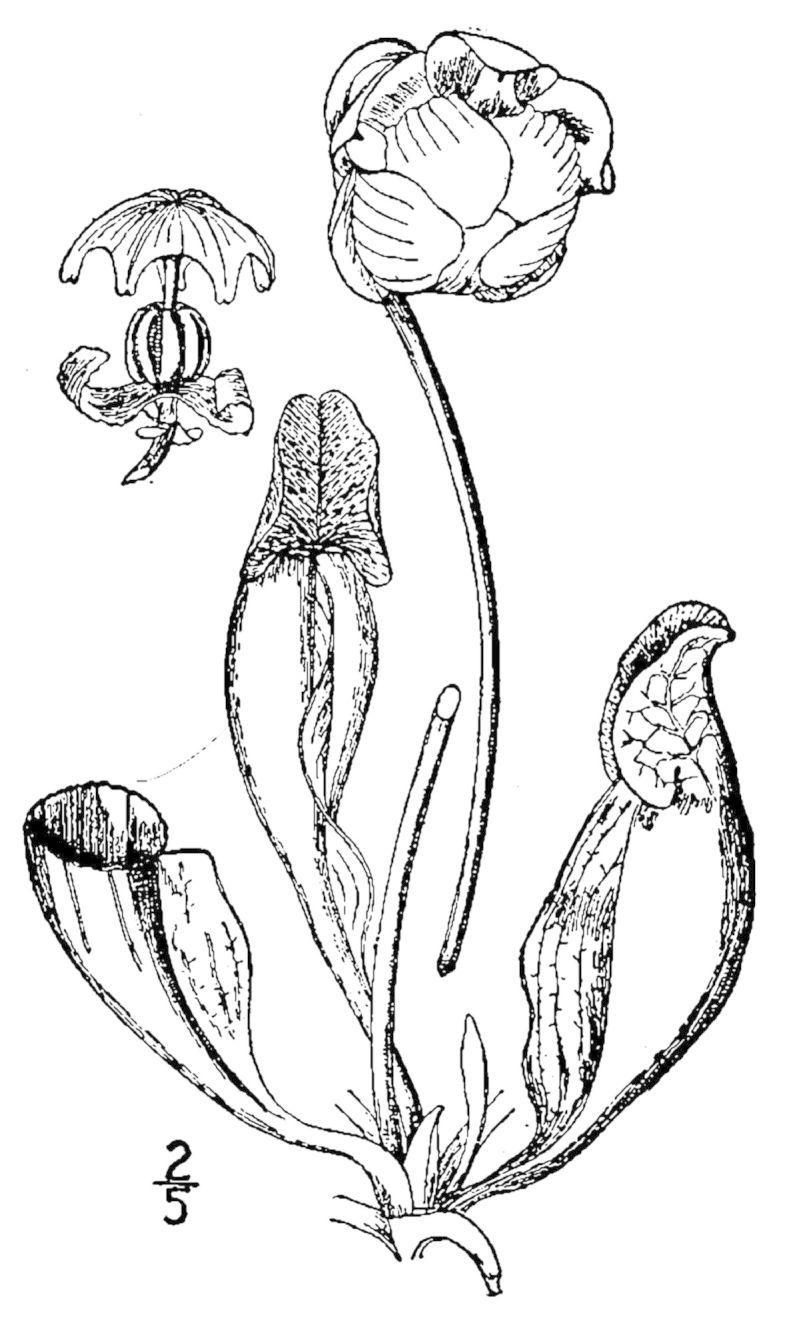
*Sarracenia purpurea* (from Britton and Brown 1913).

**Figure 246d.**
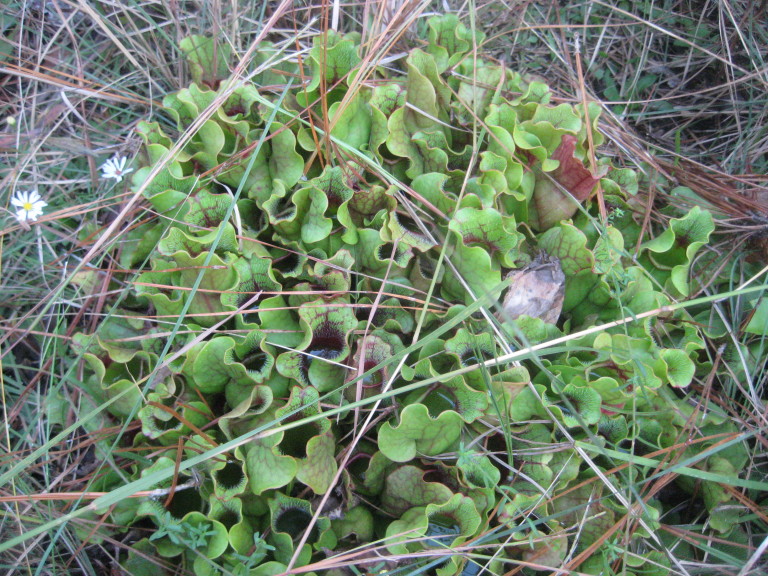
Sarracenia purpurea var. venosa (photo by R. Thornhill).

**Figure 246e.**
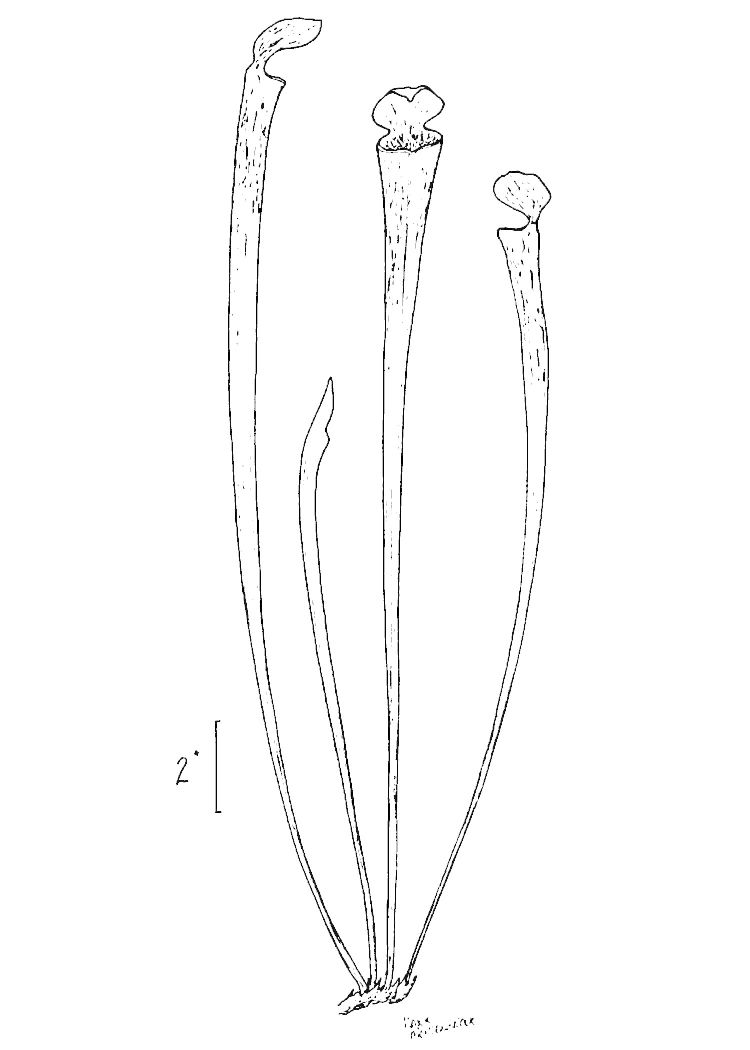
*Sarracenia rubra* (from USDA-NRCS 2012).

**Figure 246f.**
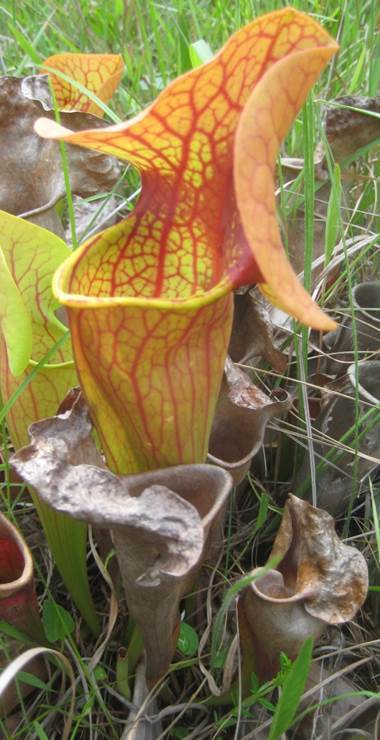
Sarracenia × catesbaei (= *Sarraceniaflava* × *Sarraceniapurpurea*) (photo by R. Thornhill).

**Figure 247a.**
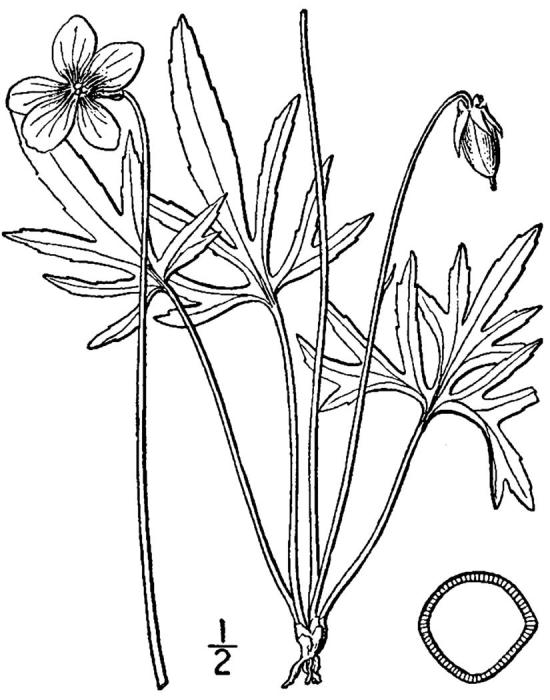
*Viola brittoniana* (from Britton and Brown 1913).

**Figure 247b.**
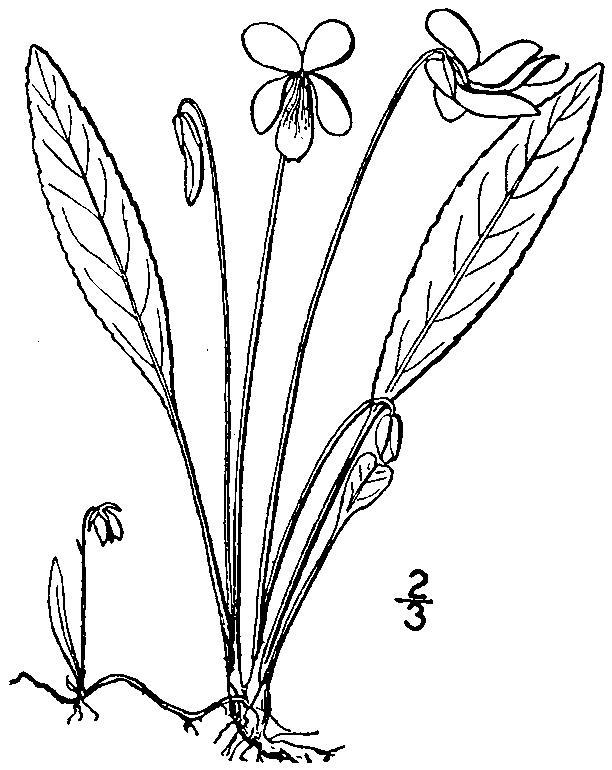
*Viola lanceolata* (from Britton and Brown 1913).

**Figure 247c.**
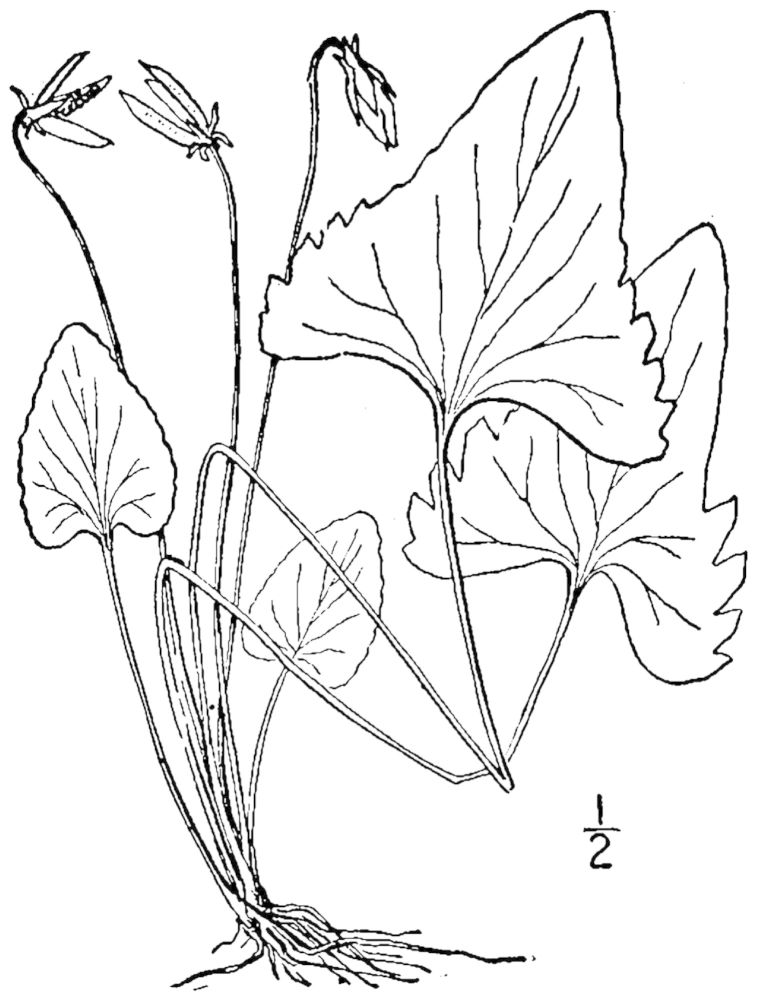
Viola sagittata var. sagittata (from Britton and Brown 1913).

**Figure 247d.**
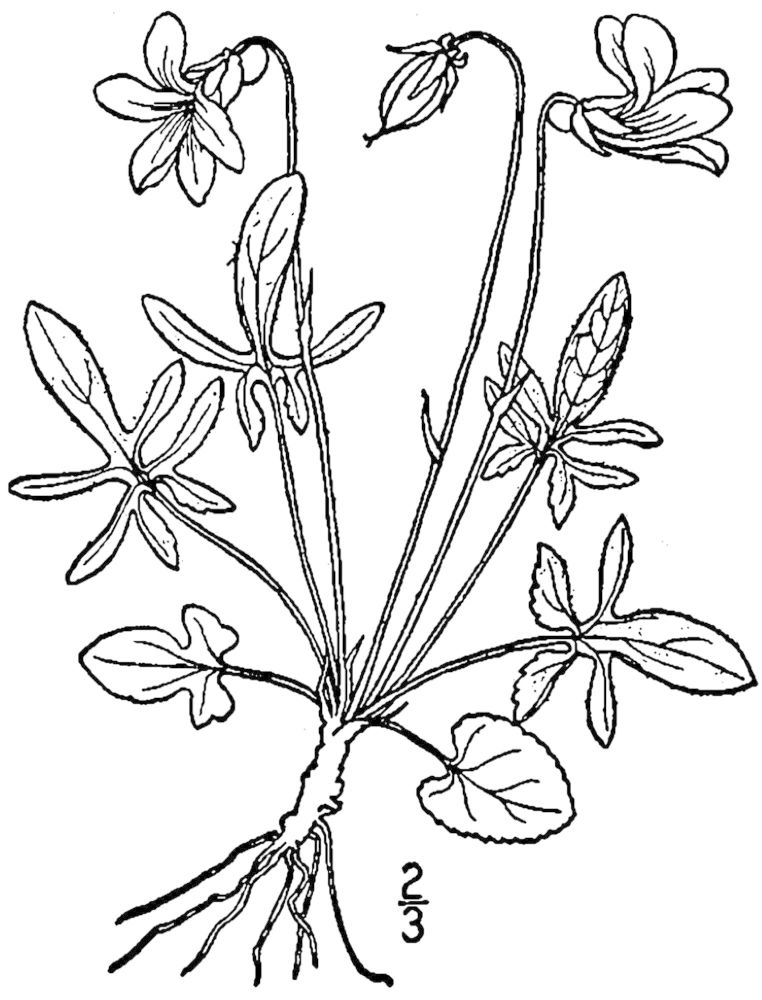
*Viola septemloba* (from Britton and Brown 1913).

**Figure 247e.**
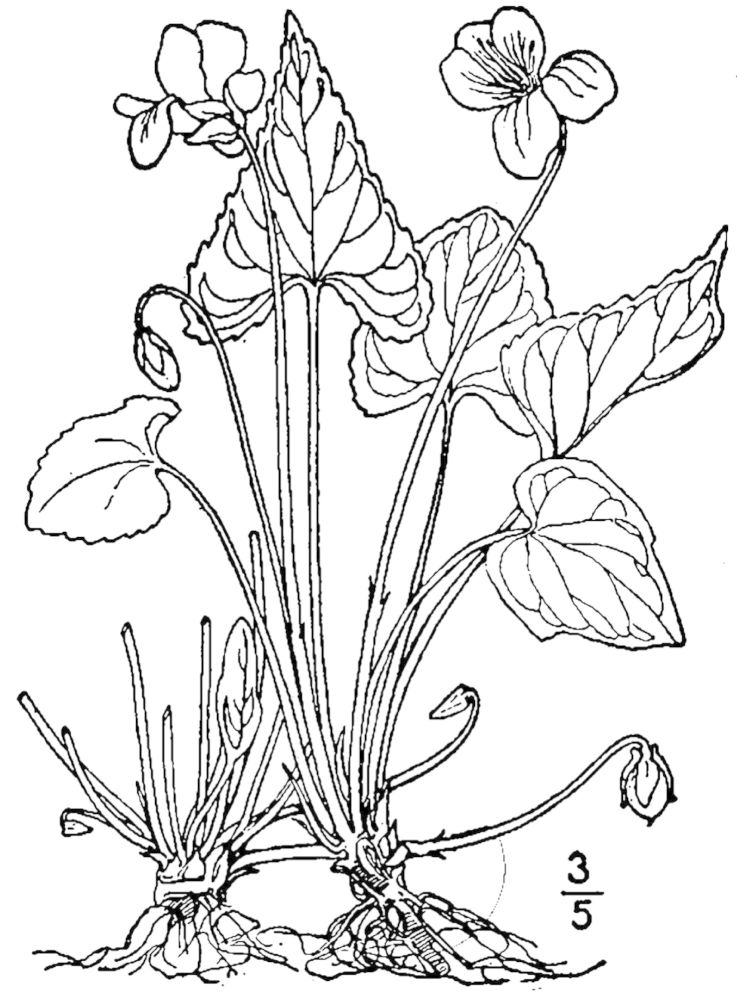
Viola sororia var. missouriensis (from Britton and Brown 1913).

**Table 1.** Plant community types in Shaken Creek Preserve included in this work. Community types and their associated ranks follow Schafale (2012). Community types are presented in order of increasing soil moisture – i.e., from driest community type (Pine/Scrub Oak Sandhill (Mesic Transition Subtype)) to wettest (Very Wet Loamy Pine Savanna). **S1** = Critically Imperiled, 1–5 occurrences in state; **S2** = Imperiled, 6–20 occurrences in state; **S3** = Vulnerable, 21–100 occurrences in state; **G1** = Critically Imperiled, 1–5 occurrences in world; **G2** = Imperiled, 6–20 occurrences in world; **G3** = Vulnerable, 21–100 occurrences in world

Plant Community Type (sensu Schafale 2012)	State Rank	Global Rank
Pine/Scrub Oak Sandhill (Mesic Transition subtype)	S2S3	G2G3
Mesic Pine Savanna (Coastal Plain subtype)	S2	G2G3
Wet Pine Flatwoods (Typic subtype)	S3	G3
Sandy Pine Savanna (Typic subtype)	S3	G3
Sandy Pine Savanna (Rush Featherling subtype)	S1	G1
Wet Loamy Pine Savanna	S1	G1
Very Wet Loamy Pine Savanna	S1	G1

**Table 2.** List of rare taxa (i.e., "Significantly Rare" or rarer sensu Gadd and Finnegan 2012) collected or reported from savannas, flatwoods, or sandhills in Shaken Creek Preserve or the vicinity (i.e., within a 2-mile radius of Shaken Creek Preserve, including Sandy Run Savannas State Natural Area). Status and rank designations follow Gadd and Finnegan (2012). Parentheses around a taxon indicate that the taxon is not known from Shaken Creek Preserve but is known from the vicinity. Taxa for which voucher specimens have been collected (by the senior author or others) are indicated with a "yes" in the second column. The taxonomy followed in this work and that of Gadd and Finnegan (2012) differ in the following instances: 1) Gadd and Finnegan (2012) do not report infraspecific taxa within *Arnoglossum ovatum* (Walter) H. Rob.; therefore, the status and ranks listed in the table below for Arnoglossum ovatum var. lanceolatum (Nutt.) D.B. Ward apply to the species, not just the variety (though var. *Arnoglossumovatumlanceolatum* is currently the only infraspecific taxon within *Arnoglossum ovatum* reported for NC (Weakley (2012))); 2) the recently named *Coreopsis palustris* Sorrie is listed by Gadd and Finnegan (2012) as *Coreopsis helianthoides* Beadle; 3) *Packera paupercula* (Michx.) Á. Löve & D. Löve is listed by Gadd and Finnegan (2012) as *Packera crawfordii* (Britton) A.M. Mahoney & R.R. Kowal (see the note in the Identification Key to *Packera* for a brief discussion regarding the taxonomy of this species and its treatment in this work). Finally, the infraspecific global rank given by Gadd and Finnegan (2012) and reported here for *Rhynchospora pinetorum* Britton & Small is probably a reflection of the recognition of that taxon by many authors as Rhynchospora globularis (Chapman) Small var. pinetorum (Small) Gale. State Status: **E** = Endangered; **T** = Threatened; **SC** = Special Concern: **-V** = Vulnerable, **-H** = Historical; **SR** = Significantly Rare: **-L** = Limited to North Carolina and adjacent states (endemic/near endemic), **-T** = Throughout, **-P** = Periphery of Range, **-O** = Other. Federal Status: **E** = Endangered; **FSC** = Federal Species of Concern. State Rank: **SH** = historical (known only from historical populations); **S1** = Critically imperiled, 1–5 populations in state; **S2** = Imperiled, 6–20 populations in state; **S3** = Vulnerable, 21–100 populations in state. Global Rank: **G1** = Critically imperiled, 1–5 populations in world; **G2** = Imperiled, 6–20 populations in world; **G3** = Vulnerable, 21–100 populations in world; **G4** = Apparently secure, 101–1000 populations in world; **G5** = Secure, 1001^+^ populations in world; **T**# = Global rank of a subspecies or variety; **Q** = Questionable taxonomy; **?** = Uncertain.

Taxon	Vouchered?	State Status	Federal Status	State Rank	Global Rank
(***Agalinis virgata*** Raf.)		SR-P		S2	G3G4Q
***Agrostis altissima*** (Walter) Tuck.		SR-T		S2	G4
***Allium* species 1**	Yes	SR-L	FSC	S1S2	G1G2
***Amorpha georgiana*** Wilbur	Yes	E	FSC	S2	G3
(***Andropogon mohrii*** (Hack.) Hack. ex Vasey)	Yes	T		S2	G4?
***Aristida simpliciflora*** Chapm.	Yes	E		S1S2	G3G4
***Arnoglossum ovatum*** (Walter) H. Rob. var. ***Aristidaovatumlanceolatum*** (Nutt.) D.B. Ward	Yes	SR-P		S2	G4G5
***Asclepias pedicellata*** Walter	Yes	SC-V		S3	G4
(***Baccharis glomeruliflora*** Pers.)	Yes	SC-H		S1	G4
***Carex lutea*** LeBlond	Yes	E	E	S2	G2
***Cirsium lecontei*** Torr. & A. Gray	Yes	SC-V		S2	G2G3
***Cladium mariscoides*** (Muhl.) Torr.		SR-O		S3	G5
(***Coreopsis palustris*** Sorrie)	Yes	SR-P		S1S2	G3G4Q
***Coreopsis* species 1**	Yes	SR-L		S1	G1?
***Dichanthelium caerulescens*** (Hack. ex Hitchc.) Correll	Yes	E		S1S2	G2G3
***Dionaea muscipula*** J. Ellis	Yes	SC-V	FSC	S3	G3
(***Eryngium aquaticum*** L. var. ***Eryngiumaquaticumravenelii*** (A. Gray) Mathias & Constance)	Yes	SR-P		S1	G4T2T4Q
(***Helenium pinnatifidum*** (Schwein. ex Nutt.) Rydb.)		SR-P		S2	G4
***Hypericum brachyphyllum*** (Spach) Steud.	Yes	SC-V		S1S2	G5
***Hypoxis sessilis*** L.	Yes	SR-P		SH	G4
***Isolepis carinata*** Hook. & Arn. ex Torr.	Yes	SR-P		S1	G5
(***Linum floridanum*** (Planch.) Trel. var. ***Linumfloridanumchrysocarpum***) C.M. Rogers	Yes	T		S1S2	G5?T3?
***Lysimachia asperulifolia*** Poir.	Yes	E	E	S3	G3
***Muhlenbergia torreyana*** (Schult.) Hitchc.	Yes	SC-V		S2	G3
(***Packera paupercula*** (Michx.) Á. Löve & D. Löve)	Yes	SR-T		S1	G2G3
***Panicum dichotomiflorum*** Michx. var. ***Panicumdichotomiflorumpuritanorum*** Svenson	Yes	SR-P		S1	G5T4
***Parnassia caroliniana*** Michx.	Yes	T	FSC	S2	G3
***Pinguicula pumila*** Michx.	Yes	E		S2	G4
(***Plantago sparsiflora*** Michx.)	Yes	T	FSC	S1S2	G3
***Platanthera integra*** (Nutt.) A. Gray ex L.C. Beck	Yes	SC-V		S2	G3G4
(***Platanthera nivea*** (Nutt.) Luer)		T		S1	G5
***Polygala hookeri*** Torr. & A. Gray	Yes	SC-V		S2S3	G3
***Pycnanthemum setosum*** Nutt.	Yes	SR-T		S2	G4
***Rhynchospora decurrens*** Chapm.	Yes	T	FSC	S1S2	G3G4
(***Rhynchospora divergens*** Chapm. ex M.A. Curtis)	Yes	SR-P		S2	G4
***Rhynchospora galeana*** Naczi, W.M. Knapp & G. Moor	Yes	SR-P		S2S3	G3G4
***Rhynchospora microcarpa*** Baldwin ex A. Gray	Yes	SR-P		S2	G5
***Rhynchospora pinetorum*** Britton & Small	Yes	SR-T		S2	G5?T3?
***Rhynchospora thornei*** Kral	Yes	SC-V	FSC	S2	G3
(***Scirpus lineatus*** Michx.)	Yes	T		S2	G4
***Scleria baldwinii*** (Torr.) Steud.	Yes	T		S2	G4
(***Scleria* species 1**)	Yes	SR-L	FSC	S1	G1
(***Scleria verticillata*** Muhl. ex Willd.)	Yes	SR-P		S2	G5
***Spiranthes eatonii*** Ames ex P.M. Br.	Yes	E		S2	G2G4
***Spiranthes laciniata*** (Small) Ames	Yes	SC-V		S2	G4G5
(***Spiranthes longilabris*** Lindl.)		E		S1	G3
***Thalictrum cooleyi*** H.E. Ahles	Yes	E	E	S2	G2
(***Trillium pusillum*** Michx. var. ***Trilliumpusillumpusillum***)		E	FSC	S2	G3T2
(***Xyris floridana*** (Kral) E.L. Bridges & Orzell)		T		S1	G5
(***Xyris scabrifolia*** R.M. Harper)	Yes	SC-V	FSC	S2	G3

**Table 3.** List of North Carolina Watch List taxa collected or reported from savannas, flatwoods, or sandhills in Shaken Creek Preserve or the vicinity (i.e., within a 2-mile radius of Shaken Creek Preserve, including Sandy Run Savannas State Natural Area). Status and rank designations follow Gadd and Finnegan (2012) with the exception of *Paspalum praecox*, which lacks varietal recognition in Gadd and Finnegan (2012) but which is here treated as comprising two varieties, for which the status and ranks are simply the same as those given by Gadd and Finnegan (2012) for the species. Parentheses around a taxon indicate that the taxon is not known from Shaken Creek Preserve but is known from the vicinity. Taxa for which voucher specimens have been collected (by the senior author or others) are indicated with a "Y" in the second column. State Status: **W** = Watch List: **W1** = rare but relatively secure, **W2** = rare but taxonomically questionable, **W5B** = exploited plants, **W7** = rare and poorly known. State Rank: **S2** = Imperiled, 6–20 populations in state; **S3** = Vulnerable, 21–100 populations in state; **S4** = Apparently secure, 101–1000 populations in state; **S5** = Secure, 1001^+^ populations in state. Global Rank: **G2** = Imperiled, 6–20 populations in world; **G3** = Vulnerable, 21–100 populations in world; **G4** = Apparently secure, 101–1000 populations in world; **G5** = Secure, 1001^+^ populations in world; **T**# = Global rank of a subspecies or variety; **Q** = Questionable taxonomy; **?** = Uncertain.

Taxon	Vouchered	State Status	State Rank	Global Rank
***Agalinis aphylla*** (Nutt.) Raf.	Yes	W1	S3	G3G4
***Agalinis linifolia*** (Nutt.) Britton	Yes	W1	S3	G4?
***Agalinis obtusifolia*** Raf.	Yes	W1	S2S3	G4G5Q
***Aletris farinosa*** L.	Yes	W5B	S5	G5
***Amphicarpum amphicarpon*** (Pursh) Nash	Yes	W1	S3	G4
***Andropogon perangustatus*** Nash	Yes	W1	S2S3	G4
***Andropogon virginicus*** L. var. ***Andropogonvirginicusdecipiens*** C.S. Campb.	Yes	W7	S1S2	G5T4
***Anthenantia rufa*** (Elliott) Schult.	Yes	W1	S2	G5
***Asclepias longifolia*** Michx.	Yes	W1	S2S3	G4G5
***Bartonia verna*** Raf. ex Barton	Yes	W1	S2	G5?
***Calamovilfa brevipilis*** (Torr.) Hack. ex Scribn. & Southw.	Yes	W1	S3	G4
(***Carex chapmanii*** Steud.)		W1	S3	G3
(***Carex physorhyncha*** Liebm. ex Steud.)		W1	S2S3	G5T5
***Chamaelirium luteum*** (L.) A. Gray	Yes	W5B	S5	G5
***Cleistesiopsis divaricata*** (L.) Pansarin & F. Barros	Yes	W1	S3	G4
***Cleistesiopsis oricamporum*** P.M. Br.		W7	S2	G3?
***Coelorachis rugosa*** (Nutt.) Nash	Yes	W1	S3	G5
***Dichanthelium dichotomum*** (L.) Gould var. ***Dichantheliumdichotomumroanokense*** (Ashe) LeBlond	Yes	W1	S2	G5T4?
***Dichanthelium ovale*** (Elliott) Gould & C.A. Clark var. ***Dichantheliumovaleovale***	Yes	W1	S2S3	G5T5
***Eleocharis equisetoides*** (Elliott) Torr.	Yes	W1	S3	G4
***Eryngium yuccifolium*** Michx. var. ***Eryngiumyuccifoliumsynchaetum*** A. Gray ex J.M. Coult. & Rose	Yes	W2	S2	G5T5
***Eupatorium recurvans*** Small	Yes	W7	S1?	G3G4Q
***Ludwigia maritima*** R.M. Harper	Yes	W7	S2S3	G5
***Lycopus amplectens*** Raf.	Yes	W1	S3	G5
***Lysimachia loomisii*** Torr.	Yes	W1	S3	G3
***Oenothera fruticosa*** L. var. ***Oenotherafruticosaunguiculata*** Fernald	Yes	W7	S2S3	G5T2T3
***Paspalum praecox*** Walter var. ***Paspalumpraecoxcurtisianum*** (Steud.) Vasey	Yes	W1	S2S3	G4
***Paspalum praecox*** Walter var. ***Paspalumpraecoxpraecox***	Yes	W1	S2S3	G4
***Rhynchospora nitens*** (Vahl) A. Gray	Yes	W1	S3	G4?
(***Rhynchospora oligantha*** A. Gray)	Yes	W1	S3	G4
***Rhynchospora pallida*** M.A. Curtis	Yes	W1	S3	G3
(***Rhynchospora scirpoides*** (Torr.) Griseb.)	Yes	W1	S3	G4
***Rhynchospora wrightiana*** Boeck.	Yes	W1	S3	G5
***Sarracenia flava*** L.	Yes	W5B	S3S4	G5?
***Sarracenia rubra*** Walter ssp. ***Sarraceniarubrarubra***	Yes	W5B	S3	G4T3T4
***Scleria georgiana*** Core	Yes	W1	S3	G4
***Solidago gracillima*** Torr. & A. Gray	Yes	W1	S3	G4?
***Solidago pulchra*** Small	Yes	W1	S3	G3
***Sporobolus pinetorum*** Weakley & P.M. Peterson	Yes	W1	S3	G3
(***Syngonanthus flavidulus*** (Michx.) Ruhland)	Yes	W1	S3	G5
***Viola brittoniana*** Pollard	Yes	W7	S2?	G4G5
***Xyris brevifolia*** Michx.	Yes	W1	S3	G4G5
(***Xyris flabelliformis*** Chapm.)		W1	S1	G4
(***Xyris iridifolia*** Chapm.)	Yes	W7	S2	G4G5T4T5
***Xyris* species 1**	Yes	W2	S2	G2

**Table 4.** Descriptions for estimating abundance of taxa collected by the senior author in Shaken Creek Preserve (adapted from Palmer et al. 1995).

Density	Description
Abundant	Dominant or co-dominant in one or more common communities
Frequent	Numerous in one or more common communities but not dominant in any common community
Occasional	Widely scattered in several communities, including one or more common communities
Infrequent	Few individuals or colonies but found in several locations or communities
Rare	Few individuals or colonies limited to one or very few locations or communities

**Table 5.** Summary of vascular plant taxa collected or reported from savannas, flatwoods, or sandhills in Shaken Creek Preserve or the vicinity (i.e., in Sandy Run and/or within a 2-mile radius of Shaken Creek Preserve). The first number in each three-number series indicates the number of taxa collected or reported from Shaken Creek Preserve. The second number in each series indicates the number of additional taxa collected or reported from the vicinity of, but not in, Shaken Creek Preserve. The third number in each series, which appears in parentheses, is the sum of the preceding two numbers and indicates the total number of taxa collected or reported from either Shaken Creek Preserve or the vicinity. (The lone exotic taxon is Pinus elliottii Engelm. var. elliottii, which was planted by a timber company in a flatwoods in Shaken Creek Preserve prior to the site’s purchase by The Nature Conservancy.)

Group	Families	Genera	Species and Subspecies/Varieties		
			Native	Exotic	Total
Pteridophytes	5, 0 (5)	7, 0 (7)	9, 0 (9)	0, 0 (0)	9, 0 (9)
Gymnosperms	2, 0 (2)	4, 0 (4)	6, 0 (6)	1, 0 (1)	7, 0 (7)
Basal angiosperms & magnoliids	2, 0 (2)	3, 1 (4)	3, 1 (4)	0, 0 (0)	3, 1 (4)
Monocotyledons	20, 2 (22)	67, 4 (71)	200, 42 (242)	0, 0 (0)	200, 42 (242)
Eudicotyledons	54, 5 (59)	123, 22 (145)	231, 59 (290)	0, 0 (0)	231, 59 (290)
**Total**	**83, 7 (90)**	**204, 27 (231)**	**449, 102 (551)**	**1, 0 (1)**	**450, 102 (552)**
